# Identification and quantification of defective virus genomes in high throughput sequencing data using DVG-profiler, a novel post-sequence alignment processing algorithm

**DOI:** 10.1371/journal.pone.0216944

**Published:** 2019-05-17

**Authors:** Trent J. Bosma, Konstantinos Karagiannis, Luis Santana-Quintero, Natalia Ilyushina, Tatiana Zagorodnyaya, Svetlana Petrovskaya, Majid Laassri, Raymond P. Donnelly, Steven Rubin, Vahan Simonyan, Christian J. Sauder

**Affiliations:** 1 Division of Viral Products, Center for Biologics Evaluation and Research, Food and Drug Administration, Silver Spring, Maryland, United States of America; 2 Department of Biochemistry and Molecular Medicine, George Washington University Medical Center, Washington, DC, United States of America; 3 Office of Biostatistics and Epidemiology, Center for Biologics Evaluation and Research, Food and Drug Administration, Silver Spring, Maryland, United States of America; 4 Office of Hematology and Oncology Products, Center for Drug Evaluation and Research, Food and Drug Administration, Silver Spring, Maryland, United States of America; 5 Division of Biotechnology Review and Research II, Center for Drug Evaluation and Research, Food and Drug Administration, Silver Spring, Maryland, United States of America; University of Helsinki, FINLAND

## Abstract

Most viruses are known to spontaneously generate defective viral genomes (DVG) due to errors during replication. These DVGs are subgenomic and contain deletions that render them unable to complete a full replication cycle in the absence of a co-infecting, non-defective helper virus. DVGs, especially of the copyback type, frequently observed with paramyxoviruses, have been recognized to be important triggers of the antiviral innate immune response. DVGs have therefore gained interest for their potential to alter the attenuation and immunogenicity of vaccines. To investigate this potential, accurate identification and quantification of DVGs is essential. Conventional methods, such as RT-PCR, are labor intensive and will only detect primer sequence-specific species. High throughput sequencing (HTS) is much better suited for this undertaking. Here, we present an HTS-based algorithm called DVG-profiler to identify and quantify all DVG sequences in an HTS data set generated from a virus preparation. DVG-profiler identifies DVG breakpoints relative to a reference genome and reports the directionality of each segment from within the same read. The specificity and sensitivity of the algorithm was assessed using both *in silico* data sets as well as HTS data obtained from parainfluenza virus 5, Sendai virus and mumps virus preparations. HTS data from the latter were also compared with conventional RT-PCR data and with data obtained using an alternative algorithm. The data presented here demonstrate the high specificity, sensitivity, and robustness of DVG-profiler. This algorithm was implemented within an open source cloud-based computing environment for analyzing HTS data. DVG-profiler might prove valuable not only in basic virus research but also in monitoring live attenuated vaccines for DVG content and to assure vaccine lot to lot consistency.

## Introduction

Defective interfering particles (DIPs) were first described more than 70 years ago in influenza virus stocks [1, 2] and have since been shown to be a biproduct of viral replication for most viruses, particularly RNA viruses [3]. DIPs are missing substantial parts of their parental genome but still contain the genetic elements that are required for genome replication. Therefore, DIPs only replicate in the presence of complete full-length parent viruses. These act as helper viruses providing the proteins required for replication of the DIPs truncated genomes. The term DIP has evolved since its coining [4] to encompass many forms of defective viruses, including those that do not interfere with virus replication. It is therefore more accurate to refer to these entities as Defective Viral Genomes (DVGs). DVGs propagate well *in vitro* and are packaged into particles like standard viruses. In addition to interfering with viral replication, particles containing DVGs have been shown to affect viral virulence and evolution and more recently have been reported to be present *in vivo* in several human viral infections. They are also thought to play a pivotal role in natural virus-host interactions, such as persistent infections [3, 5–8]. Research on DVGs has gained renewed interest in recent years due to the recognition that certain types of DVGs can act as potent inducers of the innate immune response. Thus, DVGs are being considered for use as natural adjuvants and as antivirals [6, 8, 9]. With respect to the manufacture of live attenuated virus vaccines, measuring and controlling the content of DVGs in vaccine stocks may be of importance to ensure lot to lot consistency and quality.

**Fig 1:**
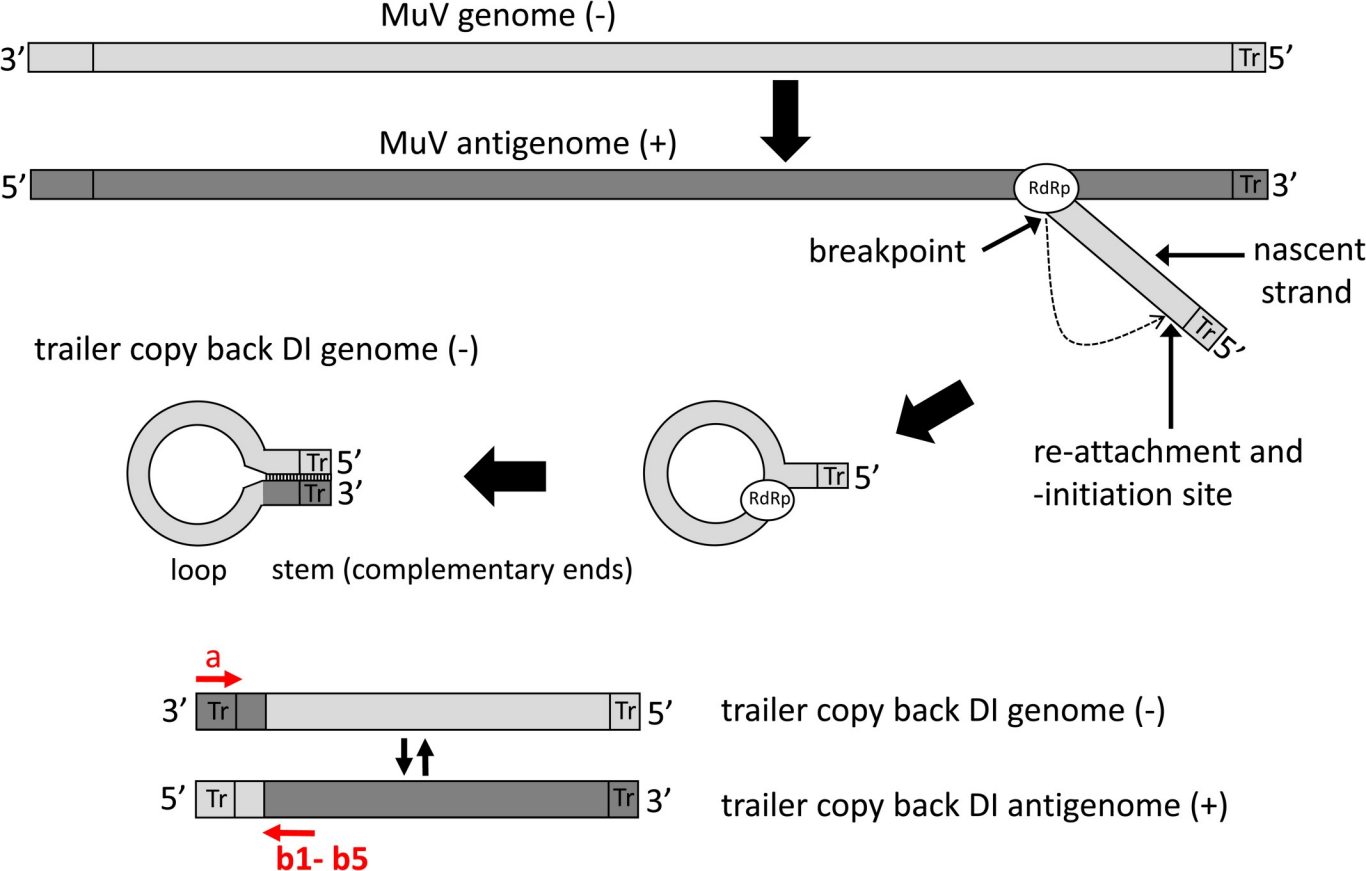
Generation of trailer copy back DVGs during paramyxovirus replication. Trailer copyback DVGs have been proposed to arise during synthesis of the viral genome through detachment of the RNA dependent RNA polymerase (RdRp) from the antigenomic template and switching back to the nascent strand. The positions of detachment from the original template, the breakpoint, and the point of reattachment to the nascent strand, the reinitiation site, are illustrated. Due to the template switching and copying back on the nascent strand the 3’ genomic promoter region has been replaced by a sequence complementary to the 5’ genomic promoter. Copyback DVGs display stem and loop regions of variable length. Mumps virus (MuV) specific DVGs were amplified by RT-PCR using primer a for the reverse transcription reaction and primers a and b1 –b5 for the PCR. The orientation of the primers only allows amplification of DVG- derived sequences, but not from wild-type genomes.

For RNA viruses, DVGs are believed to be generated following premature detachment of the RNA dependent RNA polymerase (RdRp) from its template during replication (the break point) and either reattach (the re-initiation site) to the same template at a random site or reattach to another RNA template or the nascent strand at a random site [3, 9, 10]. The template and position where reattachment occurs determines which type of DVG is created. A deletion DVG results if the re-initiation site is on the same template at a position closer to the 5’end, thereby skipping parts of the genome and resulting in deletions of up to 90% of the genome. An insertion DVG results if the re-initiation site occurs at a position that is 3’ to the break point. A copyback DVG results from reattachment of the RdRp to the nascent strand, usually at a position close to the 5’end, thus copying back a strand that is complementary to its own 5’end (Fig 1). Therefore, a copyback DVG is characterized by a stem region formed by the complementary ends and a single stranded loop region. Both stem and loop can vary considerably in length (between fewer than 100 nt to several-hundred nt). A snapback DVG is similar to a copyback DVG but is almost totally complementary in nature. It is likely the product of the RdRp detaching from the template and overtaking another replication complex on the same template, followed by synthesis across the replication fork [10]. As a consequence, like copyback DVGs, snapback DVGs possess terminal complementarity, but the length of the complementary region in the latter is much longer and the loop is reduced to only a few nucleotides. Both copyback and snapback DVGs have been described for several families of negative strand RNA viruses, including segmented [2, 9, 11–13] and non-segmented viruses [14–16]. Copyback DVGs appear to be the predominant species of DVGs found in several members of the paramyxoviruses, such as Sendai virus, measles virus, and parainfluenza virus, and most of our current knowledge about copyback DVGs stems from research of these viruses [17–31]. In theory, copyback DVGs should be generated both during replication of the genome, when replication starts at the antigenomic 3’end containing the trailer (tr) region, and during formation of the antisense genome, which starts at the genomic 3’end containing the leader region. However, there is a strong bias towards generation of trailer region copyback DVGs, particularly for paramyxoviruses, possibly due to the duplication of the 5’ proximal promoter for replication which is more potent than the 3’ end promoter.

Initially, detection of copyback DVGs required use of RT-PCR with DVG-specific primers [18]. With this approach, both primers are in the same sense with respect to the standard genome, but in opposite orientation with respect to the copyback DVG and thus will not amplify DNA products from the standard genome. However, to detect the DVG, one of the primers must bind to the very 3’ terminus of the DVG, while the other primer must bind somewhere in the loop region of the DVG (Fig 1). Since the positions of the breakpoint and reinitiation site are not known, and thus the location of the loop is unknown, a series of primers located at different intervals from the 5’end of the DVG genome must be used. Thus, the ability to identify copyback DVGs can be a challenging task. In theory, amplification of copyback DVGs should be feasible using the terminal primer only, as has been demonstrated for measles virus [29]. However, the thoroughness of this approach is questionable and has not been demonstrated. Additionally, identification of the amplified DVGs requires gel purification or subcloning of PCR amplicons and Sanger sequencing, which is time consuming. Moreover, if one were interested in knowing the relative proportions of each DVG species in a virus sample, quantitative real time RT-PCR methods would need to be developed and optimized for each individual DVG identified, which is not practicable. More recently, high throughput sequencing (HTS) metagenomics has been explored as a means of more efficiently identifying and quantifying DVGs in a virus sample. Identification of these DVGs fully depends on the proper and efficient detection of reads that span the junction site within the DVGs. Due to the nature of copyback DVGs, the regions flanking the junction site will align to different regions of the reference genome. Due to this challenge, standard bioinformatics alignment algorithms will fail to detect these genomes and therefore specific algorithms are required. Killip et al., (2013) reported development of such a tool to identify numerous DVGs in samples of parainfluenza virus 5 (PIV5) that were enriched for DVGs [31]. However, this tool has not been made openly available yet. The open source alignment tool TopHat2 [32] has been used to detect copyback DVGs in Sendai virus samples [22]. However, this tool has not been optimized for detection of copyback DVGs and its specificity and sensitivity to detect these DVGs has not been assessed.

Given the renewed interest in DVGs, open source bioinformatics tools for the reliable and efficient detection and quantitation of DVGs are needed. Here we present the development and validation of such an algorithm we have termed DVG-profiler. The tool was successfully used to reliably detect multiple species of DVGs including copyback, deletional, and insertional DVGs in multiple mumps virus stock preparations.

## Materials and methods

### Development of DVG-profiler

To detect the DVGs that might be present in HTS data generated from virus samples, a new algorithm is proposed. The algorithm was implemented as part of the High-performance Integrated Virtual Environment (HIVE) platform at CBER, FDA. HIVE is a distributed computing environment used for biological research, including analysis of HTS data [33,34]. The source code was written in C++ and compiled with g++ 4.8 20150623 (Red Hat 4.8.5–28) on CentOS 7.5.1804. The source code is available through GitHub repository https://github.com/kkaragiannis/DVG-profiler/.

#### Definitions

The input of the algorithm is not the sequencing reads but a set of alignments. Sequence alignment is a method to assess similarity of sequences.

To understand the algorithm, we need to define some basic definitions.

**Definition 1**. Let ∑ = {*A*,*C*,*G*,*T*,−} be an alphabet where ‘−‘ denotes a space, and let *S* = *s*_1_*s*_2_…*s*_*m*_ and *Q* = *q*_1_*q*_2_…*q*_*n*_ be two sequences over ∑, where ‘S’ is the subject and ‘Q’ is the query. An alignment of sequences S and Q is a two-row matrix A with entries in ∑ such that:

1. The first row contains the letters of S in order; the second row contains the letters of Q in order,
2. Each column contains at least one letter of alphabet ∑

From a biological point of view, an alignment of two sequences (pairwise alignment) is a hypothesis about how the sequences evolved from their most recent common ancestor (sequence homology). An alignment can have three types of mutation events for each pair (*s*_*i*_,*q*_*i*_) ∈ {∑ *iff s*_*i*_ ≠− *and q*_*i*_ ≠−}

- Substitution–when a single nucleotide is replaced by another. (*s*_*i*_ ≠ *q*_*i*_)
- Insertion–a nucleotide is inserted at a position where (*s*_*i*_ ≠ −)
- Deletion–a nucleotide is deleted at a position where (*q*_*i*_ ≠ −)

**Definition 2.** For 1 ≤ *i*,*j* ≤ *l* and *s*_*i*_,*q*_*i*_ ∈ ∑ a column $\left(\begin{array}{c} s_{i}\\ q_{i} \end{array}\right)$ of an alignment A of length ‘l’ is called a match if (*s*_*i*_ = *q*_*i*_) and mismatch (or substitution) if (*s*_*i*_ ≠ *q*_*i*_). A column $\left(\begin{array}{c} -\\ q_{i} \end{array}\right)$ is called an insertion and a column $\left(\begin{array}{c} s_{i}\\ - \end{array}\right)$ is called a deletion. The column $\left(\begin{array}{c} -\\ - \end{array}\right)$ can't exist. For example, in the alignment A:

$$A=\left(\begin{array}{ccccccccc} C & - & A & C & G & - & A & T & T\\ C & T & A & G & G & T & A & - & T \end{array}\right)$$

The sequences have a match in the 5 columns: {1,3,5,7,9}. There is a mismatch in column {4}, 2 insertions in columns: {2, 6} and 1 deletion in column {8}.

**Definition 3.** Consider an alphabet ∑ = {a_1_,…a_k_} and a function m: ΣxΣ→Z that assigns a score m(a_i_,a_j_) to each pair of letters. Then for 1 ≤ i,j ≤ k a matrix is called a scoring matrix for an alphabet ∑. Each pair of symbols is assigned a specific score that is used to determine the overall score of the alignment A.

**Definition 4.** An alignment score is the sum of scores of each pair of symbols in the alignment.

$$S(a)=\sum_{i=1}^{k}\left(m(s_{i},q_{i})\right)$$

A partial alignment score can be calculated as well if we specified the start and the end of the alignment positions ‘x’ and ‘y’.

$$S(a^{x,y})=\sum_{i=x}^{y}\left(m(s_{i},q_{i})\right)$$

Where 1 ≤ x ≤ y ≤ k, where the length of the alignment is ‘k’.

**Definition 5.** Let two alignments *a*_*i*_ and *a*_*j*_ of the same sequence Q contain the subrange [*q*_*l*_,*q*_*m*_].

$$a_{i}=\left(\begin{array}{c} s_{k}\ldots s_{l}\ldots s_{m}\\ q_{k}\ldots q_{l}\ldots q_{m} \end{array}\right)$$

$$a_{j}=\left(\begin{array}{c} s_{l}\ldots s_{m}\ldots s_{n}\\ q_{l}\ldots q_{m}\ldots q_{n} \end{array}\right)$$

Where *k* ≤ *l* ≤ *m* ≤ *n*. Let *t* be a position so that *l* ≤ *t* ≤ *m*. Then we define 2 composite scores of the subrange [*q*_*l*_,*q*_*m*_].

$$OS(p_{ij},t)=S(a_{i}^{l,t})+S(a_{j}^{t+1,m})$$

$$\bar{OS}(p_{ij},t)=S(a_{i}^{t+1,m})+S(a_{j}^{l,t})$$

**Definition 6.** *t* = argmax_*x*≤*t*≤*y*_ *OS*(*p*_*ij*_,*t*), Finding *t* that maximizes *OS*(*p*_*ij*_,*t*) is simply achieved by examining all positions of range [*QS*_*j*_,*QE*_*i*_].

**Definition 7**. A perfect score of alignments is when we find a match in all the positions of the alignment, this is when:

$$S(a)=\sum_{i=1}^{k}\left(m(s_{i},q_{i})\right)=k\cdot m_{score}$$

Where *m*_*score*_ is the value assigned to a match in the alignment (see definition 3).

**Fig 2:**
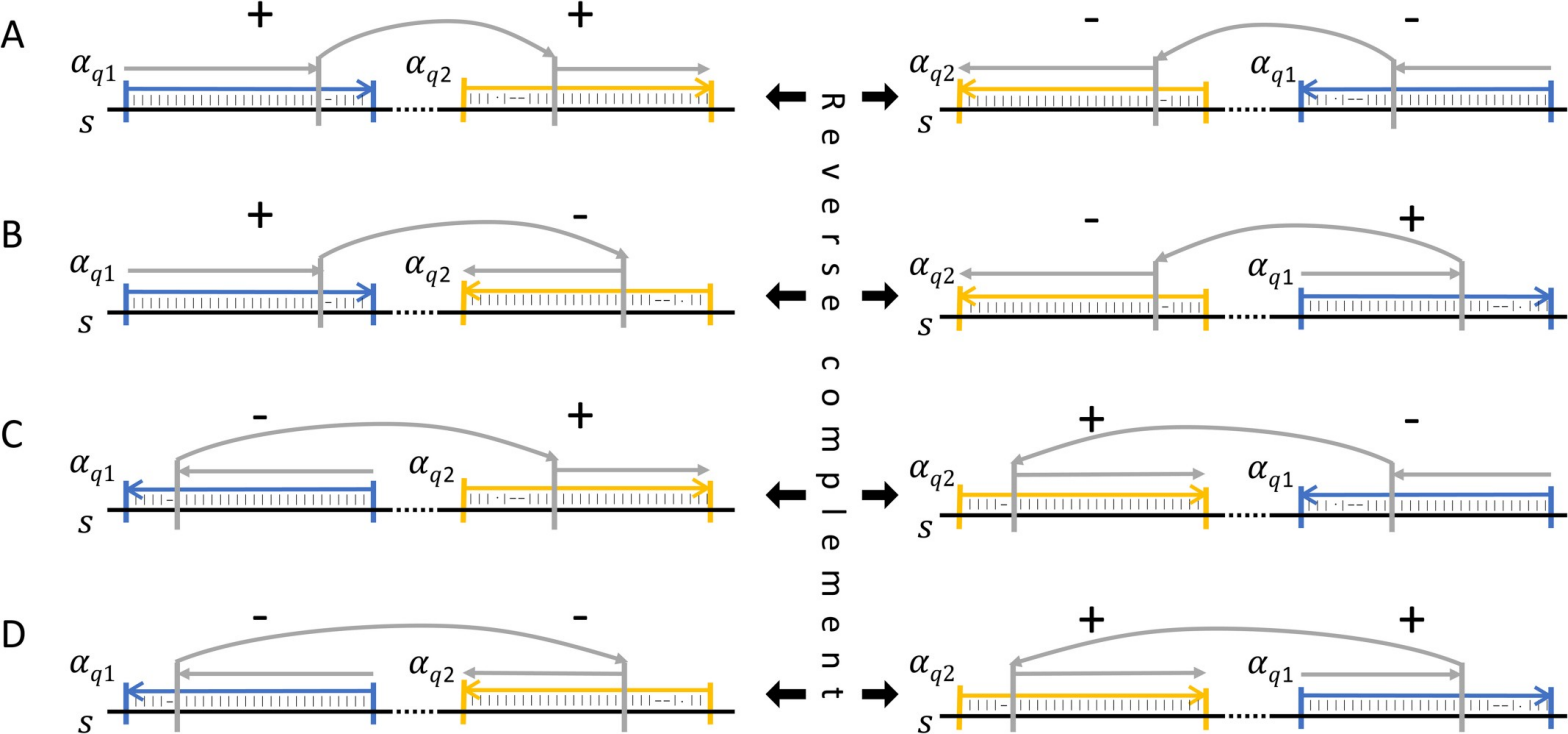
Different orientations of alignment pairs. Each pair consists of two alignments of the same read. The first alignment is marked as the one that involves the left-most part of the read and the second alignment as the one that involves the right-most part of the read. The first alignment is highlighted in blue and the second is highlighted in yellow. If the first alignment has mapped the read to a position in the subject preceding the second alignment, then the pair, as well as the read that covers the junction, is of forward orientation. In the opposite case, the pair, as well as the read that covers the junction, is of reverse complement orientation. When both the first and the second alignment are of forward orientation (A), the pair corresponds to a deletion when the pair itself is of forward orientation. The pair describes a deletion again when both alignment and the pair itself is of reverse orientation (A). Similarly, different combinations of each alignment orientation and their symmetric reverse complement of the pair are described for 3’copy-back (B), 5’ copy-back (C) and insertion of duplicated sequence (D). The grey vertical lines denote the location of the decided breakpoint on the read in case of multiple solutions of the maximum score path.

**Definition 8**. To describe a pair *p*_*ij*_ of alignments, we need to consider the position in the subject to which each alignment corresponds. Let the corresponding position of *QS*_*i*_ in the subject be *SS*_*i*_ and the *SE*_*i*_ the equivalent for *QE*_*i*_. If *QS*_*i*_ ≤ *QS*_*j*_ and *SS*_*i*_ ≤ *SS*_*j*_ then the pair *p*_*ij*_ has forward orientation, but if *QS*_*i*_ ≤ *QS*_*j*_ and *SS*_*i*_ > *SS*_*j*_ then the pair *p*_*ij*_ has reverse orientation. Fig 2 shows the association of forward pairs and their reverse complement.

**Definition 9.** Let a viral non-DVG sequence of length *k* be ***s*** = *b*_1_*b*_2_…*b*_*k*_ over the alphabet *S* = {*A*,*C*,*G*,*T*}. There are four different types of DVGs derived from sequence ***s*.**

The first type of DVG is the result of a deletion:

$$s_{del}=b_{1}b_{2}\ldots b_{i-1}b_{i}b_{j}b_{j+1}\ldots b_{k}\;where\;i<j-1$$

The second DVG is the result of an insertion:

$$s_{ins}=b_{1}b_{2}\ldots b_{i-1}b_{i}b_{j}b_{j+1}\ldots b_{k}\;where\;i>j-1$$

The third type of DVG known as copyback (cb) is described as:

$$s_{cb}=b_{1}b_{2}\ldots b_{j-1}b_{j}{\ldots b_{i-1}b}_{i}b_{j}b_{j-1}\ldots b_{2}b_{1}\;where\;j+x\leq i\leq k$$

Where *x* is greater than 5 nt and corresponds to the hairpin loop

The last type of DVG known as snapback (sb) is:

$$s_{sb}=b_{1}b_{2}\ldots b_{j-1}b_{j}{\ldots b_{i-1}b}_{i}b_{j}b_{j-1}\ldots b_{2}b_{1}\;where\;j\leq i\leq j+x\leq k$$

Where *x* is less than 5 nt.

DVG sequence ***s*** describes a single strand genomic sequence because the event takes place during replication. The definition applies to both sense and antisense genomes by considering ***s*** = *b*_1_*b*_2_…*b*_*k*_ to always describe the sequence in the direction of replication.

#### Description of the DVG-profiler algorithm

**Fig 3:**
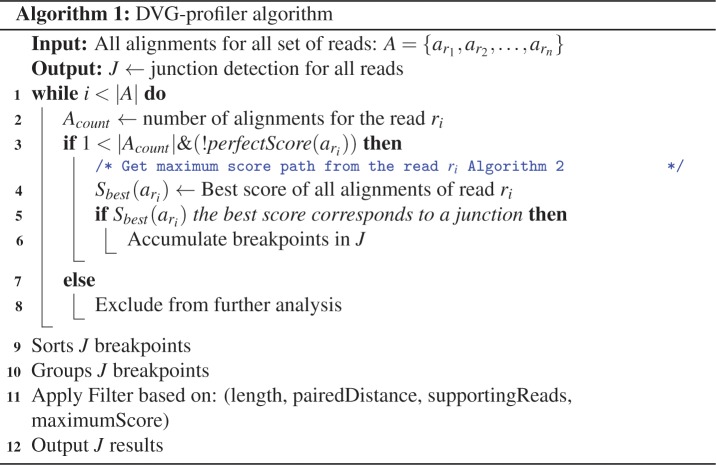
Algorithm 1 pseudocode of DVG-profiler. The input of the algorithm is a set of alignments sorted by reads, so alignments from the same read appear consecutively. Each subset of alignments derived from the same read is examined for all the combinations to find the best pair. Pairs reporting breakpoints are sorted, grouped and filtered before being presented. See materials and methods for further information.

**Fig 4:**
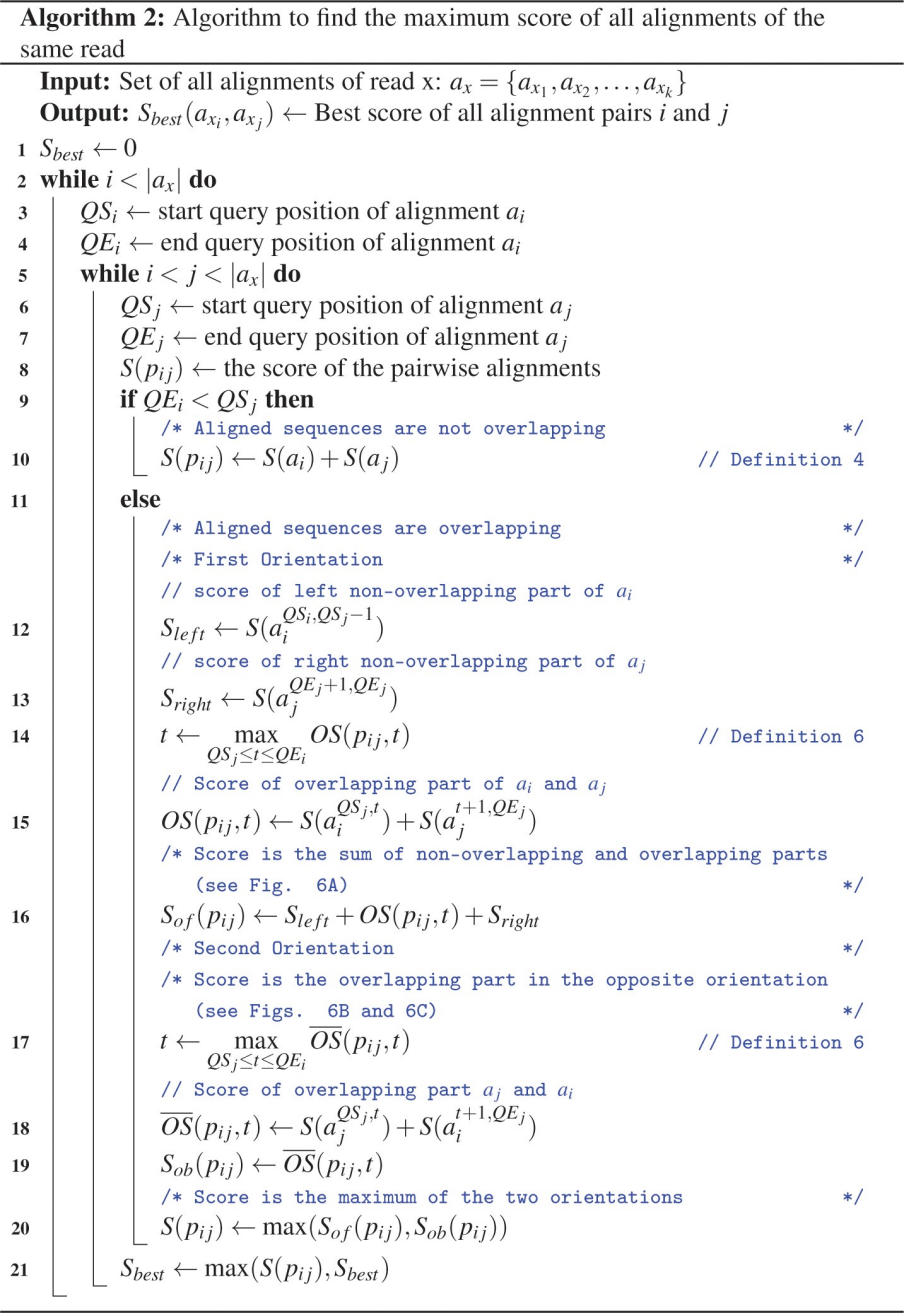
Algorithm 2 pseudocode to calculate the maximum score path. Depicted is the pseudocode of algorithm 2, looping through all the possible combinations of pairs of ‘i’ and ‘j’ alignments, calculating the score of each combination and retaining the best score overall. To calculate the score, each pair is inspected for an overlapping region (if it exists) and calculates the score for different orientations. The best pair of alignments is selected based on the highest score.

**Fig 5:**
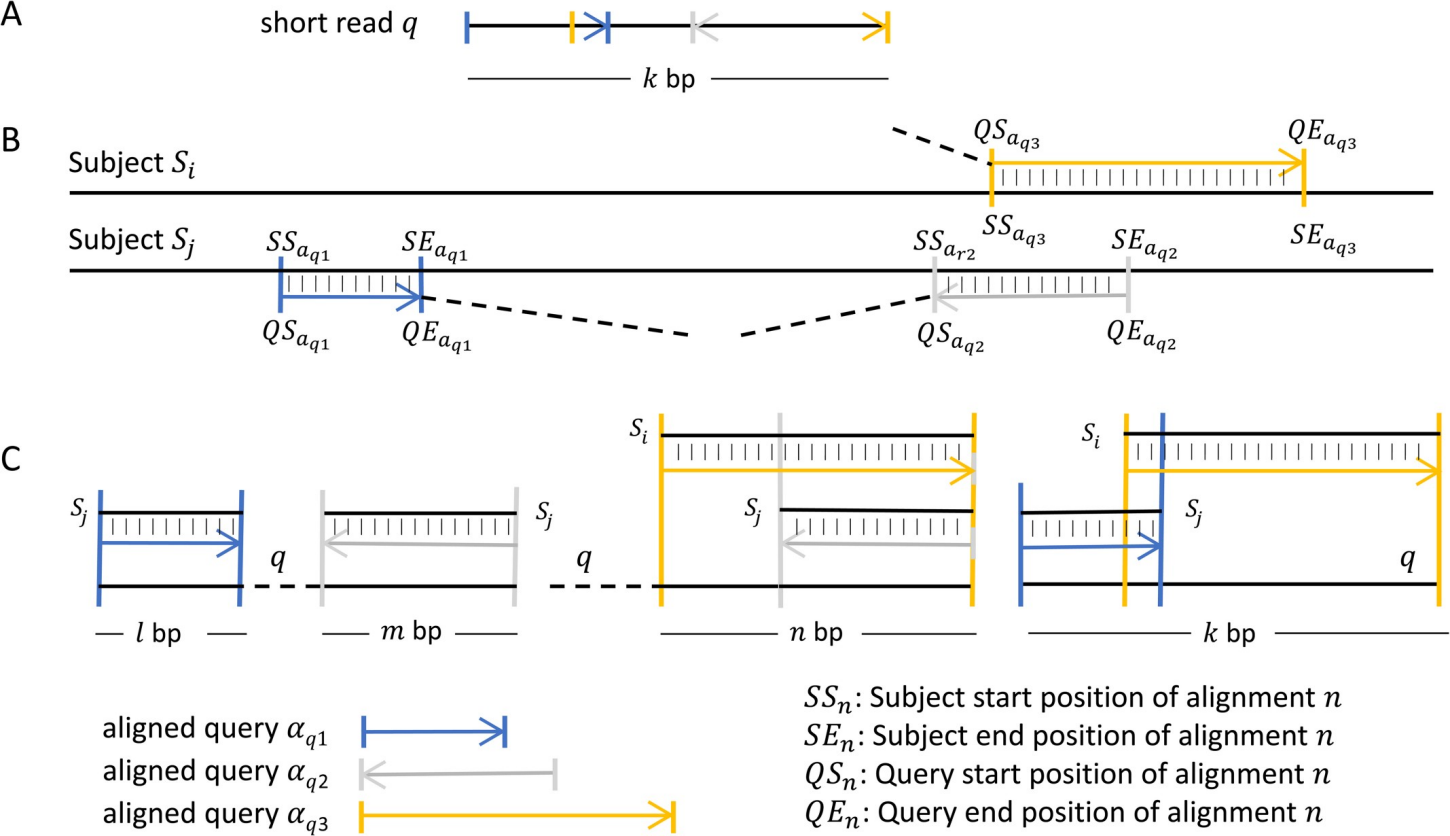
Multiple alignments of the same read. Schematic representation of multiple alignments of one read (A) against more than one reference (B). When examining a pair of such alignments the aligned parts of the read may cover a subset of the read or the total read and, in either case they may or may not overlap (C).

Fig 3 depicts the pseudocode of the general DVG algorithm and in Fig 4, the pseudocode to calculate the maximum score from all alignments of the same read is presented. DVG-profiler algorithm assumes that the alignments are sorted per read, so all the alignments of each read are presented sequentially to the next step (Fig 5A). The algorithm is not limited by the number of references selected during the alignment step, hence a read can be aligned against more than one subject (Fig 5B). The algorithm counts the number of alignments of each read and if it has more than one alignment and the alignments do not have a perfect score (see definition 7 and line 3 of Fig 3), then we further inspect the reads in pairs, finding the best pair of alignments that give us the best score that corresponds to a junction (line 4 of Fig 3). Otherwise, reads that have only one hit or have an alignment with a perfect score, are excluded from the analysis. It is expected that certain DVGs will accumulate mutations and will start to diverge from the original DVG. These mutations may occur close to the junction positions and can impact how these reads align against the reference, potentially resulting in shorter alignments and therefore in junctions detected in few positions away from the original DVG. To prevent this from happening, DVG-profiler uses a peak detection algorithm to group multiple positions reported within a user specified window (lines 9–11 of Fig 3). The results include both the raw junction positions and the ones after the peak detection algorithm, so the user can further inspect the results. Additionally, DVG-profiler allows different filtration mechanisms that are based on:

- number of reads supporting each junction.
- minimum length of the aligned read
- maximum distance from the aligned pairs that the aligned subsequences are distant from each other.
- minimum score of the overlapping region of the maximum score path.

All the filtration mechanisms are optional and require a user specified value. Furthermore, DVG-profiler filters detected junctions based on the number of supporting pairs of each orientation (Definition 8) individually or as the summation of the two. DVG-profiler can filter out detected junctions that have less than *x* supporting forward pairs, or reverse complement pairs or both forward and reverse. Additionally, post computational filters can be applied that will filter junctions for which the coverage is biased towards one direction.

#### Maximum score path

In Fig 4, the algorithm 2 shows the pseudocode of the calculation of the maximum score path. Input of the algorithm is all the alignments of a single read. The algorithm will inspect all the combinations of alignments to determine the best score for a specific pair. For example, if we get 2 alignments on one read, we only calculate the score path one time because we get only 1 pair. If we get 5 alignments, the algorithm will inspect all the possible pair combinations $\left(\begin{array}{c} n\\ 2 \end{array}\right)=\frac{n!}{2(n-2)!};$ for n = 5, then $\left(\begin{array}{c} 5\\ 2 \end{array}\right)=10$, so we get 10 different combinations and keep the best score overall.

**Fig 6:**
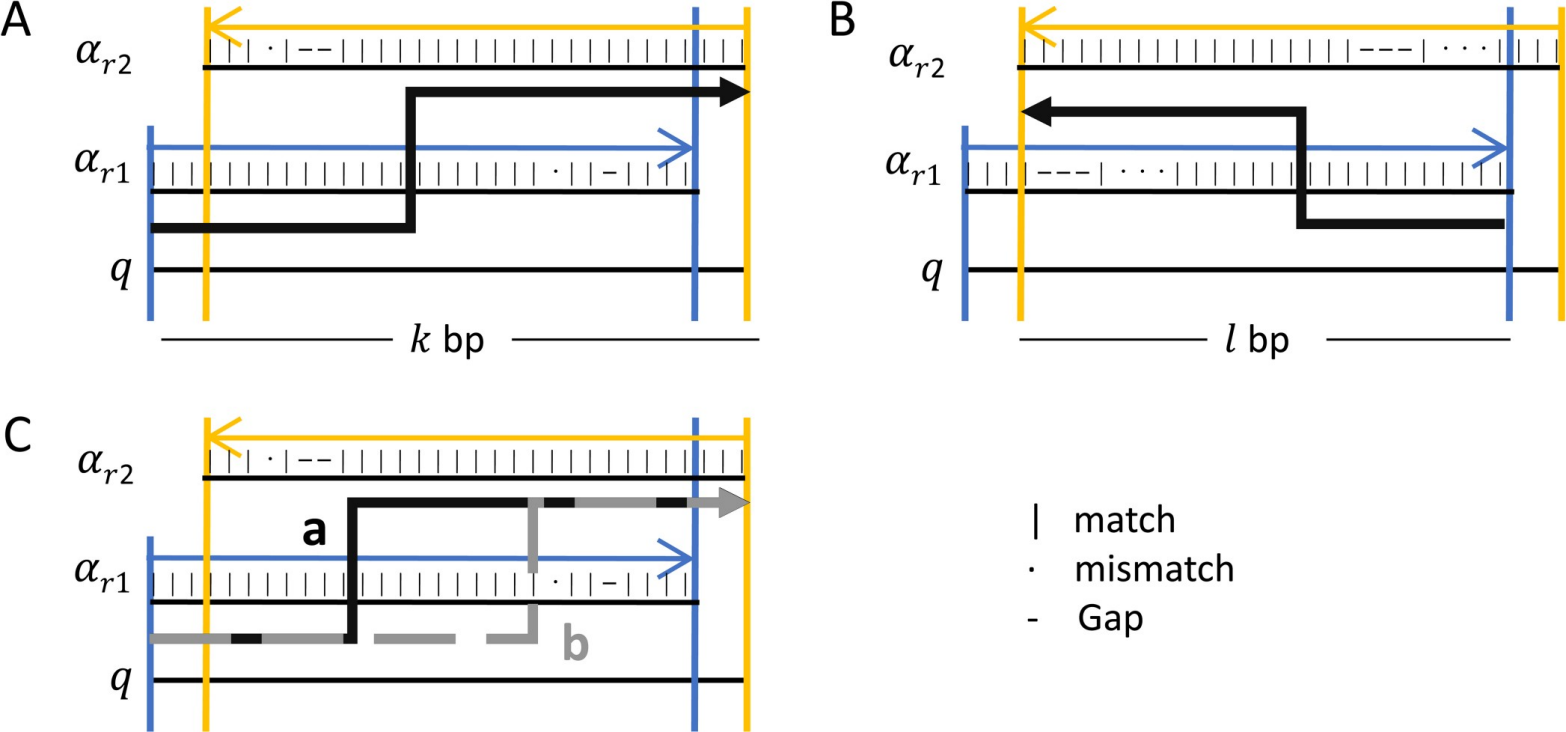
Overlapping subsequences of read. Schematic representation of a pair of alignments of the same read when the aligned read subsequences are overlapping. The pair is examined for all possible orientations to find the higher possible score (A and B). Once the best orientation is identified, the maximum score path is calculated detecting the corresponding breakpoint on the read (C). Different combinations of the orientation of each alignment corresponds to a different type of DVG.

For a single combination of alignments or pair, we first check if they overlap or not. In case of not overlapping (line 10 of Algorithm 2, Fig 4), then the score is just the sum of both alignments (see Definition 4). If they do overlap (lines 12–19, Fig 4), then we calculate the scoring function in two parts, each one corresponds to different orientations to get the score of both orientations (see Definition 5). In the first orientation (Fig 6A), the score is the sum of the non-overlapping part and the score of the overlap between alignments. For the second orientation (Fig 6B and 6C), the score of the overlap is calculated in the opposite orientation with $\bar{OS}(p_{ij},t)$, and when the selection of t is performed randomly, different junction positions might be reported from different reads despite the fact that all junctions have the same origin, to avoid this problem, DVG-profiler is choosing ‘t’ stably, choosing always the greater value of t in the range [x, y]. Once a final score is calculated for a specific pair of alignments, the value is compared to the rest of combinations and the algorithm will keep the best value with information regarding the alignments involve in the generation of the maximum score.

### *In silico* datasets

To assess the sensitivity of DVG-profiler, two DVG template sequences were generated using a mumps virus genome with RefSeq accession number NC_002200.1 as reference [35]. DVG S1 is a 5’ copyback DVG with breakpoint and reinitiation sites at positions 13257 and 14191, and a total size of 3322 bases. DVG S2 is a deletion DVG with a total size of 10320 bases. The deletion encompasses 5065 bases between positions 522 and 5587. *In silico* random reads were then produced from the two DVG template sequences, each one representing a different sample. Five samples were generated for each DVG (ISDP CB1 to ISDP CB5 and ISDP D1 to ISDP D5, respectively) using the same template sequences but with 5 different read lengths as described in S1 Table. The sequencing reads created include in the title line of the FASTA format the information of the template sequence, the position, and the directionality used to generate each short read.

To evaluate the performance of DVG-profiler against DI-tector, another recently published open-source bioinformatics DVG detection algorithm [36], 8 template sequences were generated using the same genome as described in S2 Table. To assess the sensitivity, five samples were composed of randomly generated reads from the reference sequence spiked with reads from dvg3 (S2 Table) in five different concentrations as described in S3 Table. One sample (“SPD”) that contains sequencing reads generated from all 8 DVG templates (S2 Table) and the reference sequence was also created to measure the specificity of the tools. Performance of the tools was measured by recall *R* = *TP*/(*TP* + *FN* (false negative)) and precision as *Pr* = *TP*/(*TP* + *FP*) where TP (true positives) are considered the reported DVGs with reported breakpoints that accurately match the template sequences. Also the divergence between the true and the predicted prevalence of the DVG was measured using the Jensen-Shannon divergence [37] *D*_*JS*_(*P*||*Q*) = 1/2*D*_*KL*_(*P*||*M*) + 1/2*D*_*KL*_(*Q*||*M*) where *M* = (*P* +*Q*)/2 and *D*_*KL*_(*P*||*M*) is the Kullback–Leibler divergence [38] between P and M. The distribution of the prevalence was calculated using the a priori knowledge of the reference’s depth of coverage. Comparison of DVG-profiler against DI-tector was performed on CentOS 7.5.1804 installed on an Intel 2 Quad core 2.26 GHz with 24GB of RAM system.

### Cells

Vero cells (ATCC CCL-81), BHK-BSR-T7/5 cells [39] and A549 cells (ATCC CCL-185) were grown in Dulbecco’s modified Eagle’s medium (DMEM, Quality Biological) supplemented with 2 mM L-glutamine and 9% fetal calf serum (Quality Biological). BHK-BSR-T7/5 cells were cultivated in the presence of 1mg/ml geneticin (ThermoFisher Scientific) at every other passage.

### Plasmids and DNA fragments

Plasmid p88moddelNhe is a cDNA clone of the wild type mumps virus clinical isolate 88–1961, constructed as previously described [40]. Plasmid p88+JL(M/F/SH/HN) is identical to p88moddelNhe in which the matrix (M), fusion (F), small hydrophobic (SH), and hemagglutinin-neuraminidase (HN) genes were replaced with those derived from the Jeryl Lynn mumps virus vaccine strain, as described elsewhere [40]. Plasmid p88-1961-JLHNStart is identical to plasmid p88moddelNhe except for nucleotide change C6541A in the HN gene start signal which was created by site-directed mutagenesis. Plasmids expressing the N, P and L genes from MuV strain 88–1961 (p-N, p-P, p-L) have been described previously [40, 41].

Plasmids Topo1-10 and Topo2-4 were used for generating MuV RNA standards for qRT-PCR and are derived from pCR2.1-TOPO (ThermoFisher Scientific) plasmids. They contain MuV sequences encompassing nucleotides 44–1112 and 14767–15384, respectively. These were obtained as PCR fragments following amplification of plasmid p88moddelNhe using primer pairs mu0f/1112r (for Topo1-10) and 14790f/A (for Topo2-4) (S4 Table). The orientation of the inserts with respect to the T7 RNA polymerase promoter is antisense, allowing generation of RNA transcripts in genomic sense orientation. Supplemental information on *in vitro* transcription of linearized plasmids Topo-1-10 and Topo2-4 as well as on qRT-PCR is provided in S1 File.

A 970 bp DNA fragment of the Urabe mumps virus vaccine strain encoding a copyback DVG (DVG Urabe930) was synthesized by Eurofins (Louisville, KY). The breakpoint is at nucleotide position 14,687 and the reinitiation site is at nucleotide position 15,153, resulting in a copyback DVG of 930 bases and with stem and loop sizes of 232 nt and 466 nt, respectively. The nucleotide sequence of DVG Urabe930 is provided in S1 File.

### Rescue of recombinant viruses from cDNA

Rescue of virus from plasmid p88-1961-JLHNStart was performed with BHK-BSR-T7/5 cells using helper plasmids p-N, p-P and p-L as described previously [40]. Rescue of virus was done in triplicate and supernatants were collected from BHK-BSR-T7/5 cells 13 days post transfection. For simplicity, rescued viruses from p88-1961-JLHNStart were named virus #1, #2 and #3. Following rescue in BHK-BSR-T7/5 cells, cell supernatants containing viruses #1, #2 and #3 were transferred to Vero cell monolayers in 75 cm^2^ flasks. Three to four days later, cell culture supernatants were harvested, clarified by low speed centrifugation and aliquoted for frozen storage as virus stocks. Virus was similarly rescued from plasmid p88+JL(M/F/SH/HN), but only the first rescue was used here. Virus titers were determined by plaque assay as described previously [42].

### Viral RNA extraction

Prior to viral RNA extraction, 161 μl of virus containing cell culture supernatants were treated for 2 hours at 37 °C with 11,400 gel units of micrococcal nuclease (New England Biolabs), followed by stopping the reaction with 15 μl of 0.5 M EGTA (Ethylene glycol-bis (β-aminoethyl ether)-N,N,N',N'-tetraacetic acid; BioWORLD). Viral RNA was extracted using the QIAamp MinElute Virus spin kit (Qiagen) and eluted in 25 μl of DEPC treated water.

### RT-PCR

Copyback DVGs were detected by DVG- specific RT-PCR according to Calain et al., 1992. [18] The method is based on using two primers in the same orientation, with one primer (a) binding to the very 3’end of the genome, thus being selective for amplification of trailer copyback DVGs (Fig 1). Viral RNA (3–5 μl) was reverse transcribed using the Superscript II reverse transcriptase kit (Invitrogen) and primer a (S4 Table). Two to five μl of cDNA was amplified by PCR using primers a and primers b1-b5 (S4 Table) employing expand high-fidelity polymerase (Sigma Aldrich) in a total volume of 50 μl (94 °C for 2 min; 40 cycles of 94 °C for 30s, 55 °C for 30 s, 72 °C for 1–2 minutes). To detect DVGs with a deletion between positions 14589 and 15046, RT was carried out using primer a and PCR was conducted using primers a and b6 (S4 Table). Controls included reactions in the absence of the RT enzyme as well as PCR reactions in the absence of cDNA. PCR reactions were analyzed in 1.5–2% ethidium-bromide stained agarose gels. PCR fragments were purified from gels using the QIAquick gel extraction kit (Qiagen) and were either directly sequenced or subcloned into plasmid pCR2.1-TOPO using the TOPO TA cloning kit (ThermoFisher Scientific). Subcloned fragments were sequenced with primers M13f and M13r (ThermoFisher Scientific). Capillary Sanger sequencing was done by Macrogen USA Corp.

### High throughput sequencing (HTS)

#### HiSeq sequencing

RNA extracted from viruses #1, #2 and #3 was processed following the protocol for the Illumina TruSeq Stranded mRNA Preparation Kit (Illumina), but without the polyA enrichment step. Briefly, approximately 100 ng of RNA was chemically fragmented and reverse-transcribed into cDNAs. Double strand cDNAs were adenylated at the 3’ends and individually indexed, followed by limited-cycle (15) amplification, and purification using Agencourt AMPure magentic beads (Beckman Coulter). After analyzing the cDNA libraries for size and quality using a BioAnalyser (Agilent Technologies), paired-end sequencing (100 x 2 cycles) of twelve multiplexed RNA samples per lane was carried out on an Illumina HiSeq2500 sequencer. HiSeq sequencing data generated for viruses #1, #2 and #3 and for the repeat HiSeq run for virus #2, are available under https://www.ncbi.nlm.nih.gov/sra/PRJNA525871 (files are named Virus_1_18C, Virus_2_19C, Virus_3_20C and Virus_2_HiSeq_rpt_11S)

#### MiSeq sequencing

DNA library preparation was done using the NEBNext Ultra RNA Library Prep kit for Illumina (New England Biolabs). Briefly, 100 ng of RNA extracted from virus r88+JL(M/F/SH/HN) was chemically fragmented using fragmentation buffer. For spiking experiments, 50 ng of RNA was mixed with 1.79x 10^4^ or 1.79x10^6^, respectively, molecules of *in vitro* transcribed DVG Urabe930 RNA in the presence of fragmentation buffer (see S1 File for information on generation and quantitation of *in vivo* transcribed DVG Urabe930 RNA). Fragmented RNA was subsequently reverse transcribed, and the DNA second strand was synthesized. The resulting DNA fragments were ligated to Illumina paired end (PE) adaptors, then amplified using 12 cycles of PCR with multiplex indexed primers and purified by magnetic beads (Agencourt AMPure PCR purification system, BeckmanCoulter). After analyzing the DNA libraries for size and quality (BioAnalyzer, Agilent Technologies), deep sequencing was performed using MiSeq (Illumina) producing 250 nucleotide paired-end reads. MiSeq sequencing data generated for virus r88+JL(M/F/SH/HN) as well as for virus r88+JL(M/F/SH/HN), spiked with 1.79 x 10^4^ or 1.79 x 10^6^, respectively, molecules of *in vitro* transcribed DVG Urabe930 RNA, are available under https://www.ncbi.nlm.nih.gov/sra/PRJNA525871 (files are named r88+JL(MFSHHN)_JLQ, Low_spike_r88+JL(MFSHHN)_JL4, and High_spike_r88+JL(MFSHHN)_JL6.

### Calculation of average coverage for full-length viral genomes

To estimate the average coverage for full-length viral genomes, the total number of reads at 22 positions in the genomes between position 500 and 11,000, in intervals of 500 nucleotides, were added and divided by 22, resulting in the average coverage.

## Results

### Identification of DVGs in viruses #1, #2 and #3

**Fig 7:**
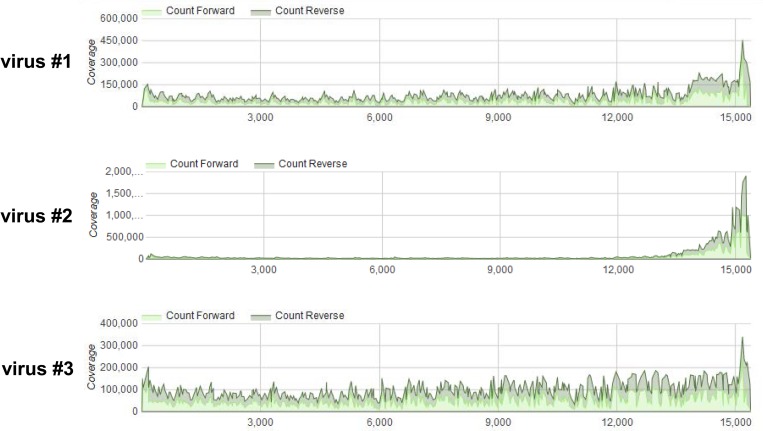
Differences in depth of coverage obtained by HTS from three recombinant MuV rescued from the same cDNA. Crude virus-containing cell culture supernatants were treated with Micrococcus nuclease followed by viral RNA extraction and high throughput sequencing using a HiSeq instrument. Data were analyzed using CBERs specialized high-performance integrated virtual environment (HIVE) platform. Note the steep increase in depth of coverage at the 5’ end of virus #2 compared to viruses #1 and #3.

**Fig 8:**
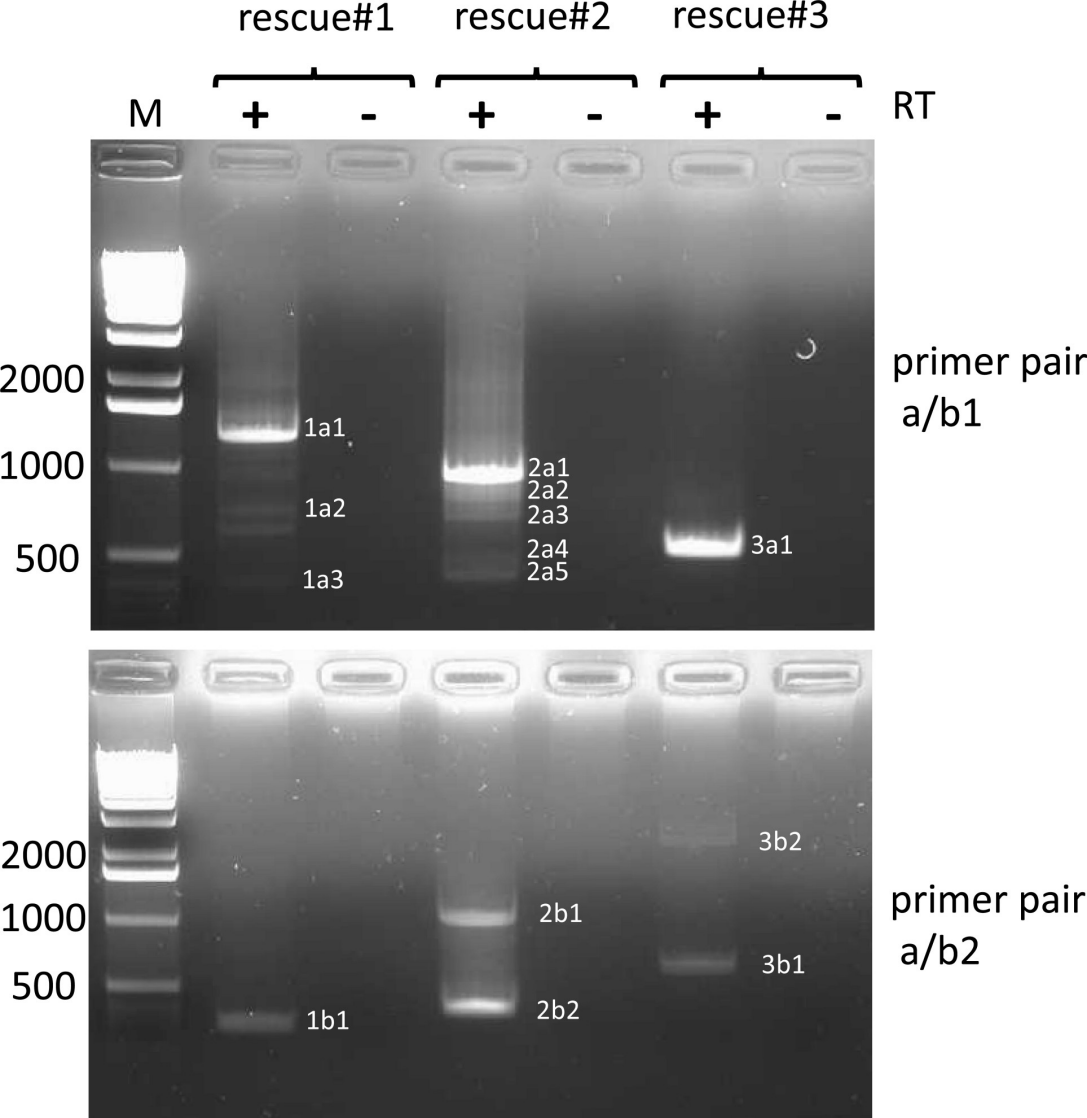
Identification of trailer copyback DVGs in viruses #1, #2 and #3 by RT-PCR. RNA extracted from viruses #1, #2 and #3 was reverse transcribed using primer a and cDNA was amplified with primer pairs a/b1 (upper panel) or a/b2 (lower panel) as outlined in material and methods. Controls (-) were treated the same way except for omission of the RT enzyme. PCR products were analyzed on 1.5% ethidium bromide stained agarose gels. Fragments that were excised from the gels are indicated with numbers. Numbers on the left indicate length of the size markers in nucleotides. (1 kb DNA ladder, Invitrogen / Thermo Fisher).

Viral RNA was prepared from viruses #1, #2, and #3 and subjected to HTS on a HiSeq instrument. Analysis of the depth of coverage over the entire length of the genome (Fig 7) revealed a disproportionately high degree of coverage at the 5’end of the genome of virus #2, reminiscent of a pattern indicative of the presence of high amounts of trailer copyback DVGs [31]. This 5’ peak was not observed for viruses 1 and 3. However, employing DVG-specific RT-PCR using two sets of primers (a/b1 and a/b2, (Fig 1, S4 Table) revealed the presence of DVGs in all three virus stocks (Fig 8). Multiple bands were visible in most samples. The amplicons were RT- dependent as evidenced by the absence of PCR products when the RT enzyme was omitted from the reactions. All amplicons were sequenced and were determined to represent trailer copyback DVGs. As summarized in Table 1, in total (using both primer sets), five, eight and two different DVGs were identified in viruses #1, #2 and #3, respectively.

**Table 1:** 5’ copyback DVG genomes identified in viruses #1, #2 and #3 employing DVG-specific RT-PCR.

Virus rescue #	Predicted size of DVG (nt)	Break-point position	Reinitia-tion position	Loop size (nt)	Stem size (nt)	Detected by RT-PCR using primer pair ^b^	Size of PCR fragment in bp ^c^
1	2262	13811- 13812 ^a^	14697- 14696 ^a^	884- 886	688- 689	a/b5	776
	2226	13580	14964	1384	421	a/b1	1635 (1a1) ^d^
	1941	13865	14964	1099	421	a/b1	1350 (1a1) ^d^
	1261	14575	14934	359	451	a/b1	670 (1a2)
	997	14770	15004	234	381	a/b1	404 (1a3)
	792	14874	15104	230	281	a/b2	327 (1b1)
2	2598	13308	14864	1556	521	a/b4	860 (2d1)
	2526	13566- 13568 ^a^	14678- 14676 ^a^	1108–1112	707	a/b4	788 (2d2) ^d^
	2490	13617	14663	1046	722	a/b4	751 (2d2) ^d^
	2310	13316	15144	1828	241	a/b4	572 (2d3) ^d^
	2300	13339	15131	1791	254	a/b4	563 (2d3) ^d^
	2184	13347	15239	1892	146	a/b4	446 (2d4) ^d^
	1905	13480	15064	1584	321	a/b4	488 (2d4) ^d^
	1584 1125^e^	13907–13909 ^a^	15279–15277 ^a^	1368–1372 909–913	108–106 108–106	a/b1 a/b2 a/b3 a/b3	993 (2a1) 1231(2b1) 1331 (2c1) 870 (2c2)^e^
	1429	14456	14885	429	500	a/b1	838 (2a2) ^d^
	1381 922^f^	14223–14224 ^a^	15166–15165 ^a^	941–943 482–484	219–220 219–220	a/b1 a/b3	790 (2a2) ^d^ 668 (2c3)^f^
	1320	14342	15108	766	277	a/b1	728 (2a3) ^d^
	1278	14596	14896	300	489	a/b1	685 (2a3) ^d^
	1092	14761	14917	156	468	a/b1	500 (2a4)
	1014	14730–14733 ^a^	15026–15023 ^a^	290–296	362–359	a/b1	423 (2a5)
	870	14868	15032	164	353	a/b2	407 (2b2)
	870	14856- 14863 ^a^	15043- 15036 ^a^	173- 187	349- 342	a/b3	615 (2c4)
	731	14861–14862 ^a^	15178- 15177 ^a^	317- 315	207- 208	a/b3	477 (2c5) ^d^
	678	14947- 14950 ^a^	15145- 15142 ^a^	198- 192	240- 243	a/b4	426 (2c5) ^d^
	708	14864	15198	333	187	a/b4	454 (2c5) ^d^
	564	14934	15272	338	113	a/b3	310 (2c6)
3	1134	14706	14930	224	455	a/b1 a/b2	543 (3a1) 675 (3b1)
	2878	12661	15231	2570	154	a/b2	2418 (3b2)^g^

^a^ The exact nucleotide position of the breakpoint and the reinitiation site could not be determined due to inverse complementarity of a sequence of two to eight nucleotides at the DVG junctions.

^b^ Sequences of primers used are listed in S4 Table.

^c^ Numbers in parentheses indicate ethidium bromide stained PCR bands in agarose gels as depicted in Figs 8 and 9; an agarose gel depicting the RT-PCR result for primer pair a/b5 using RNA prepared from virus #1 is shown in S1 Fig.

^d^ more than one DVG was detected in the DNA extracted from agarose gel bands labeled 1a1, 2a2 and 2a3, 2d2, 2c5, 2d4.

^e^ and ^f^ These copyback DVGs displayed a deletion of 457 nt between positions 14585 and 15043 resulting in sizes of 1125 b or 992 b, respectively, and of PCR fragments of 870 bp or 668 bp length, respectively.

^g^ This 5’ copyback DVG was only identified by RT-PCR and not by DVG- profiler.

### DVG-profiler, a post sequence alignment processing algorithm

As shown in Table 1, the choice of the primer pairs for DVG-specific RT-PCR determines the number and nature of DVGs being amplified from a given sample. Thus, it is likely that additional DVGs were present in the three viruses but were missed due to the specificity of the primers chosen. Furthermore, the presence of deletion and insertion type DVGs possibly present in the virus preparations cannot be detected using this approach. Given that DVG-specific RT-PCR is not quantitative, the relative abundance of DVGs in these virus preparations cannot be determined. To address these deficiencies, we sought to develop a post sequence alignment processing algorithm. The development of the algorithm, named DVG-profiler, is described in the materials and methods section and the pseudocode is depicted in Figs 3 and 4. The algorithm was implemented as part of the HIVE platform at CBER, FDA and the source code is available through GitHub repository https://github.com/kkaragiannis/DVG-profiler/.

### Validation and verification of DVG-profiler

#### Sensitivity assessment using *in silico* reads

To assess the sensitivity of DVG-profiler we used samples ISDP CB1-5 with 50, 100, 150, 200, and 250 nt read length respectively, and samples ISDP D1-5 with the same read lengths as samples ISDP CB1-5 (S1 Table). Each sample was aligned against the mumps virus reference with RefSeq accession number NC_002200.1 using the Hexagon aligner [43]. Each alignment was repeated 4 times changing the parameter that controls length of the shortest alignment to be included in the results from 10 to 15, 20 and 25 bp. The alignments were then used as an input for DVG-profiler to detect the junctions in both sample groups. The pairs of alignments that supported each junction were examined and each read mapped to the position from which it was initially generated was counted as a true positive (TP). No additional filters were applied, allowing detection of junctions supported by a single read. DVG-profiler was able to detect all reads covering the junction given that the reads were generated enough base pairs away from the breakpoint, so the alignments would not be filtered for being shorter than the threshold (S2 Fig). If we consider sensitivity as *Sn* = *TP*/(*TP* + *FN*) where TP are all the reads that cover the junction and where FN are false-negative reads, then DVG-profiler’s sensitivity was found to improve with longer read lengths and with shorter alignments allowed by the alignment process (S3 Fig).

#### Comparison against other state of the art tools

While this manuscript was in preparation, Beauclair et al., (2018)[36] reported development of an open-source bioinformatics DVG detection algorithm named DI-tector. DI-tector aligns the reads against a reference genome and examines non-perfectly aligned reads. Each read is divided into two subreads multiple times to account for all potential breakpoints within the read. These *in-silico* generated reads are then aligned against the same reference sequence and the alignments of the subreads are paired back together. We compared the sensitivity of DI-tector and DVG-profiler to detect a DVG with one copyback junction species spiked in a sample, generated from the reference sequence, in different concentrations (samples SED1 to SED5, S5 Table). Both tools were able to detect the DVG at all concentrations, but DI-tector produced false positive DVGs in all samples where DVG-profiler did not. DI-tector produced one false positive for samples SED 1 and 2, with the spiked DVG at concentrations 0.05% and 0.54%, respectively. The same tool produced two false positives for samples SED 3, 4 and 5, with the spiked DVG at concentrations 5.15%, 35.2%, and 84.45%, respectively. This resulted in higher precision reported for DVG-profiler compared to DI-tector. Furthermore, DVG-profiler more accurately determined the abundance of the DVGs for all samples (S5 Table).

In addition to sensitivity, the tools were tested for their specificity using a sample spiked with multiple DVGs. Reads were generated using the reference sequence and 8 more DVGs as described in S2 Table. Both tools successfully detected all DVGs resulting in 100% recall but DI-tector produced 23 false positives, decreasing its precision to 25.81% compared to DVG-profiler that achieved 100% precision. The false positive DVGs produced by DI-tector also affected the accuracy of the predicted abundance distribution which exhibits higher divergence from the original one (S5 Table). The tools were also compared for their speed performance and DVG-profiler was found to be faster by almost two orders of magnitude in all sample sizes (S5 Table). However, it should be pointed out that DI-tector includes the alignment process which was expectedly the slowest step of the algorithm, whereas DVG-profiler is a post -alignment algorithm and as such does not include this step. Given that the alignment process using the HIVE platform is very fast (minutes instead of hours), this could account for the difference in time between the two tools.

#### Identification of copyback DVGs in viruses #1, #2 and #3 using DVG-profiler

**Fig 9:**
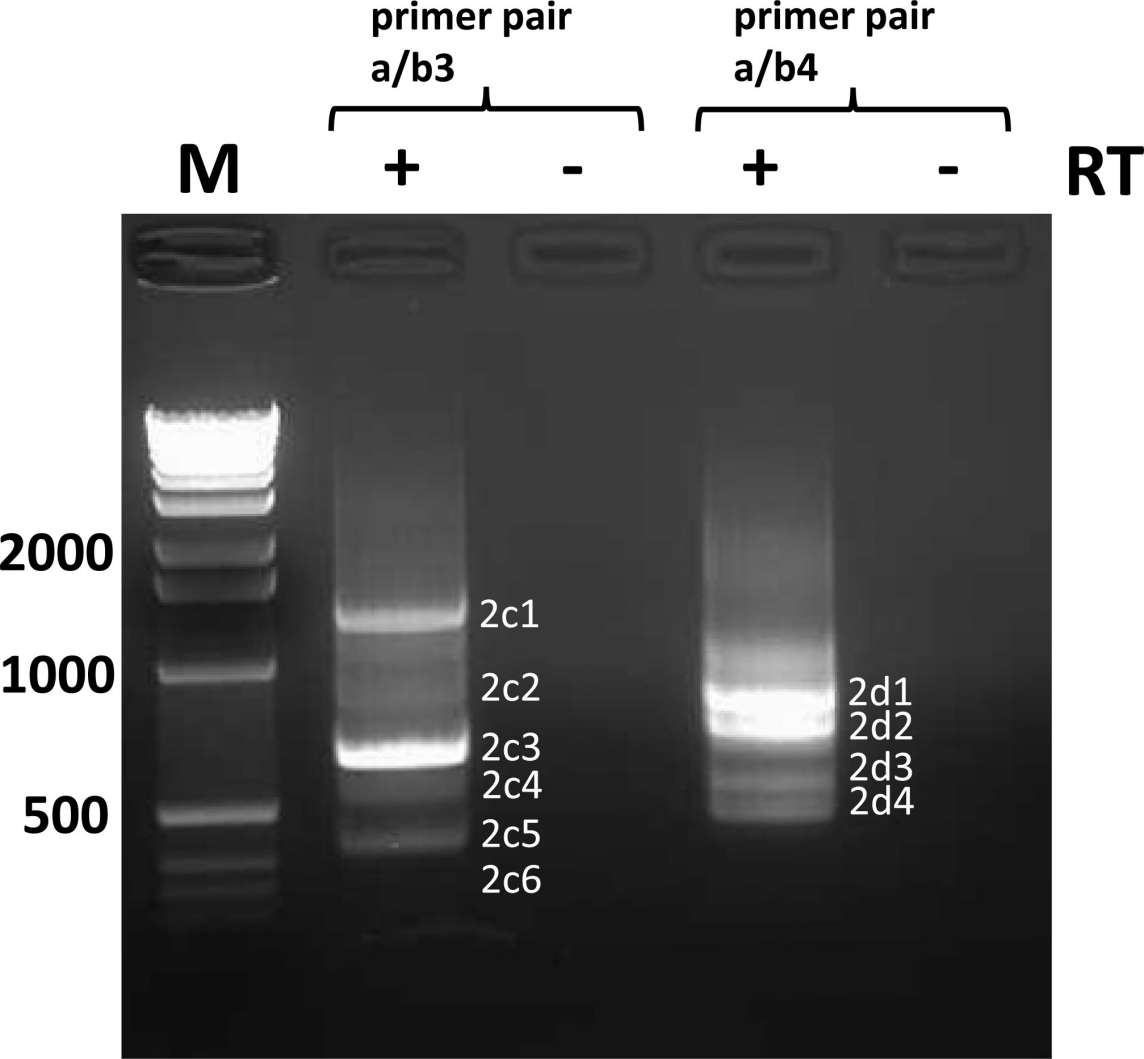
Identification of trailer copyback DVGs in virus #2 by RT-PCR using primer pairs a/b3 and a/b4. RNA extracted from virus #2 was reverse transcribed using primer a and cDNA was amplified with primer pairs a/b3 and a/b4. See materials and methods and Fig 8 for further information.

Having developed a powerful and sensitive tool to detect DVGs using *in silico* data sets, we next evaluated the specificity and sensitivity of DVG-profiler using our HTS HiSeq data obtained from viruses #1, #2 and #3. The raw data from these analyses are provided in S6–S8 Tables (viruses #1, #2, #3, respectively) and summaries of the results are provided in Tables 2 (virus #2) and 3 (viruses #1 and #3). 13 out of the 15 DVGs that were initially identified in viruses #1, #2 and #3 by DVG-specific RT-PCR using primer pairs a/b1 and a/b2 were also identified by DVG-profiler. The two DVGs not detected were 12661/15231 and 14596/14896, identified by RT-PCR using primer pair a/b2 in virus #3, and primer pair a/b1 in virus #2. However, numerous copyback DVGs not detected by RT-PCR were detected at a high abundance using DVG-profiler (Tables 2 and 3). To confirm that these species, detected by DVG-profiler, exist, and are not an artefact of DVG-profiler, RT-PCR was performed using additional primer pairs (a/b3; a/b4 for virus #2; a/b5 for virus #1) designed to detect several of the species detected by DVG-profiler. As shown in Fig 9 and S1 Fig and summarized in Tables 1 and 2, use of these additional primer pairs confirmed the presence of several of the DVGs detected by DVG-profiler that were not detected by the initial RT-PCR attempts using different primer pairs. Intriguingly, even those DVGs that were detected by DVG-profiler at very low levels (as few as four sequencing reads), were detectable by RT-PCR when the appropriate primers were used, demonstrating the remarkable sensitivity and breadth of detection of DVG-profiler.

**Table 2:** 5’ copyback DVGs identified in virus #2 using DVG-profiler.

DVG specific reads (no. of reads in repeat HiSeq run) ^a^	Breakpoint / Reinitiation position ^c^	Predicted size of DVG (nt)	Detected by RT-PCR^d^ (primer pair used)	Ratio DVG / full- length genome ^e^
111136 (195390)	14869 /15030	870	+ (a/b2)	4.63 (6.5)
23962 (41613)	14223 / 15165	1381	+ (a/b1)	1 (1.38)
19627 (25859)	13308 / 14863	2598	+ (a/b4)	0.82 (0.86)
15867 (32700)	14947 / 15144	678	+ (a/b4)	0.66 (1.09)
10349 (16149)	13616 /14663	2490	+ (a/b4)	0.43 (0.54)
6866 (18251)	13908 / 15277	1584	+ (a/b1,b2,b3)	0.29 (0.61)
5433 (7180)	14456 / 14885	1429	+ (a/b1)	0.23 (0.24)
5282 (9665)	14342 / 15107	1320	+ (a/b1)	0.22 (0.32)
3577 (6738)	14730 / 15025	1014	+ (a/b1)	0.15 (0.22)
3398 (6016)	14238 / 15025	1512	**-**	0.14 (0.2)
3317 (3498)	13479 / 15064	1905	+ (a/b4)	0.14 (0.12)
3065 (4903)	13629 / 13775	3366	**-**	0.13 (0.16)
3035 (3491)	14666 / 14777	1334	**-**	0.13 (0.12)
3004 (4683)	12591 / 14836	3343	**-**	0.12 (0.16)
2902 (4174)	13562 / 14651	2557	**-**	0.12 (0.14)
2248 (2898)	13332 / 14154	3286	**-**	0.09 (0.1)
2218 (8503)	13055 / 13442	4273	**-**	0.09 (0.28)
2081 (2982)	13316 / 15143	2310	+ (a/b4)	0.09 (0.1)
2060 (3942)	14869 / 15036 ^f^	870	+ (a/b3)	0.09 (0.13)
2013 (4464)	14350 / 14770	1650	**-**	0.08 (0.15)
1921 (3717)	14331 / 14428	2013	**-**	0.08 (0.12)
1880 (3068)	13751 / 14324	2695	**-**	0.08 (0.1)
1691 (2907)	13549 / 14514	2712	**-**	0.07 (0.1)
1690 (2219)	13326 / 13837	3608	**-**	0.07 (0.07)
1457 (2888)	13431 / 13720	3620	**-**	0.06 (0.1)
1451 (2286)	13141 /14880	2750	**-**	0.06 (0.08)
1373 (2221)	14360 / 14873	1537		0.06 (0.07)
1286 (1463)	11435 / 14306	5029	**-**	0.05 (0.05)
1271 (3042)	14761 /14917	1092	+ (a/b1)	0.05 (0.1)
1175 (2171)	13462 / 13684	3624	**-**	0.05 (0.07)
1112 (1363)	11435 / 15162	4173	**-**	0.05 (0.05)
4 DVG (662–1111)^b^				
661 (1171)	13347 / 15239	2184	+ (a/b4)	0.03 (0.04)
9 DVG (416–660)^b^				
415 (423)	13562 / 14677	2526	+ (a/b4)	0.02 (0.01)
2 DVG (353–414)^b^				
352 (781)	14859 / 15177	731	+ (a/b3)	0.01 (0.03)
122 DVG (47–351)^b^				
47 (134)	14864 / 15197	708	+ (a/b4)	0.002 (0.004)
119 DVG (19–47)^b^				
19 (23)	13340 / 15131	2300	+ (a/b4)	0.0008 (0.0008)
470 DVG (4–19)^b^				
4 (36)	14933 / 15271	564	+ (a/b3)	0.0002 (0.0012)
889 DVG (2–4)^b^				
0 (6)	14596 / 14896	1278	+ (a/b1)	0 (0.0002)

^a^ Listed are all 5’ copyback DVGs identified with 1000 or more reads. In addition, all copyback DVGs with less than 1000 reads but identified by RT-PCR were listed as well.

^b^ Indicates number of 5’ copyback DVGs within the indicated range of reads (in parentheses) that were identified by DVG-profiler, but not by RT-PCR, and not individually listed here (See S7 Table for comprehensive list of DVGs).

^c^ In addition to the proposed breakpoint and re-initiation positions for each DVG (called left and right position), DVG- profiler also provides a range (called group) of possible breakpoint and re-initiation positions for each DVG identified. For simplicity, the left and right group start -and end-positions are not included in this table, but in S7 Table.

^d^ PCR fragments were either directly sequenced or subcloned followed by sequencing

^e^ The ratio was calculated based on average estimated coverages of 24000 and 30062 reads, respectively, for full-length genomes in the first run and the repeat run of virus #2 (numbers in parentheses).

^f^ DVG-profiler identified a DVG with the following left and right group start and end positions: 14865–72 / 15034–15040. It is not 100% identical to the DVG 14856–14863 / 15036–15043 found by RT-PCR, but closely related.

**Table 3:** 5’ copyback DVGs identified in viruses #1 and #3 using DVG- profiler.

Virus #	DVG- specific reads ^a^	Breakpoint / Reinitiation position	Predicted size of DVG (nt)	Detected by RT-PCR ^d^ (primer pair used)	Ratio DVG / full-length genome ^e^
1	15339	13811 / 14696	2262	+ (a/b5)	0.25
	914	14011 / 14784	1974	-	0.015
	472	14873 / 15104	792	+ (a/b2)	0.008
	331	12101 / 14607	4062	-	0.005
	321	10692 / 14873	5203	-	0.005
	313	13722 / 13903	3144	-	0.005
	227	5074 / 15083	10611	-	0.004
	180	14561 / 14756	1452	-	0.003
	147	13419 / 14189	3162	-	0.002
	131	13865 / 14964	1941	+ (a/b1)	0.002
	95	14905 / 14943	921	-	0.002
	52 DVG (10–95) ^b^				
	10	14770 / 15003	997	+ (a/b1)	0.0002
	4 DVG (9–10) ^b^				
	9	14578 / 14932 ^c^	1261	+ (a/b1)	0.0001
	15 DVG (7–9) ^b^				
	7	13579 / 14964	2226	+ (a/b1)	0.0001
	231 DVG (2–9) ^b^				
3	346	5074 / 15079	10615	-	0.004
	307	10694 / 14876	5198	-	0.003
	206	14706 / 14930	1134	+ (a/b1,b2)	0.002
	131	12903 / 14080	3785	-	0.002
	99	7607 / 9517	13644	-	0.001
	75	14905 / 14943	922	-	0.001
	61	12577 / 13325	4872	-	0.001
	253 DVG (2–60) ^b^				

^a^ Listed are all 5’ copyback DVGs identified with 95 or more reads for virus #1 and 61 or more reads for virus #3. In addition, all copyback DVGs with less than 95 reads but identified by RT-PCR in these viruses were listed as well.

^b^ Indicates number of 5’ copyback DVGs within the indicated range of reads (in parentheses) that were identified by DVG-profiler, but not by RT-PCR, and not individually listed here (See S6 and S8 Tables for comprehensive list of DVGs for viruses # 1 and #3, respectively).

^c^ DVG-profiler identified a DVG with the following left and right group start and end positions: 14576–14580 / 14932. It is therefore not 100% identical to the DVG 14575 / 14934 found by RT-PCR, but closely related.

^d^ PCR fragments were either directly sequenced or subcloned followed by sequencing

^e^ The ratios were calculated based on average estimated coverages of 62550 and 86151 reads for full-length genomes in viruses #1 and #3, respectively

As shown in Table 2, DVG-profiler did not detect reads for a DVG with breakpoint and reinitiation sites at genome positions 14596 and 14896, respectively. This DVG was detected in virus #2 by RT-PCR using the primer pair a/b1 (Fig 8 and Table 1). In an attempt to detect this DVG using DVG-profiler, a new RNA sample was prepared from virus #2 and a repeat HTS HiSeq run was conducted using this sample. Reads were again analyzed with DVG-profiler and raw data from this analysis are provided in S9 Table (Repeat HiSeq run for virus #2). The overall depth of coverage for the full-length genome and therefore also the number of reads for individual copyback DVGs in this repeat HiSeq run was slightly higher compared to the first run (Table 2). Indeed, DVG 14596/14896 mentioned above could now be detected in six reads from the repeat HiSeq run. Likewise, DVG 14933/15271 that was detected in only four reads in the first HiSeq run was detected in 36 reads from the repeat HiSeq run. Of note, there was a very good correlation between the two runs with respect to the relative numbers of reads per copyback DVG (r = 0.92, S4 Fig). Moreover, 98.9% of the copyback DVGs with more than 50 reads identified in the first HiSeq run also were found in the repeat HiSeq run and vice versa (S10 Table).

#### Identification of deletion and insertion type DVGs in viruses #1 and #2

DVG-profiler was designed to not only detect copyback DVGs but also genomes with deletions and insertions. Deletions and insertions are recognized in the downloaded DVG-profiler table by equal strandedness of both the forward and the reverse run (recognized by either +, + (= Deletion) or -,— (= Insertion) (Fig 2). As documented in S6–S8 Tables and summarized in Tables 4 and 5, numerous deletion and insertion type DVGs were identified. The most abundant of these was DVG 14589/15046 (virus #2) which displayed a deletion of 457 nucleotides. To verify the existence of this DVG, conventional (non-DVG specific) RT-PCR was carried out using forward primer b6 and reverse primer a (S4 Table) enabling differentiation between full-length amplicons (1010 bp) and amplicons with the deletion (553 bp). Indeed, besides a strong prominent band at 1010 bp, a fainter band of the expected size of 553 bp was visible when analyzed by electrophoresis in an ethidium bromide stained agarose gel. Subcloning and subsequent sequencing of this band confirmed the presence of the 457 nt deletion.

**Table 4:** Insertion- type DVGs identified in viruses #1, #2 and #3 using DVG-profiler.

Rescue	Reads found with insertion (no. of reads in repeat HiSeq run)^a^	Breakpoint/Reinitiation position	Size of insertion (nt)	Ratio DVG / full-length genome ^b^
1	4295 (N/A)	13811 / 14707	896	0.069 (N/A)
	2612 (N/A)	13819 / 14712	893	0.042 (N/A)
	1671 (N/A)	13833 / 14707	874	0.027 (N/A)
	955 (N/A)	13720 / 13898	178	0.015 (N/A)
	315 (N/A)	12108 / 14613	2505	0.005 (N/A)
	197 (N/A)	14019 / 14787	768	0.003 (N/A)
	107 (N/A)	13826 / 14700	874	0.002 (N/A)
	105 (N/A)	14009 / 14769	760	0.002 (N/A)
	100 (N/A)	14043 / 14606	563	0.0016 (N/A)
2	14252 (15767)	14869 / 15023	155	0.59 (0.52)
	2871 (3317)	14464 / 14891	420	0.12 (0.11)
	2537 (2234)	14456 / 14877	434	0.1 (0.07)
	2121 (2651)	14456 / 14882	426	0.09 (0.09)
	1521 (1981)	13610 / 14660	1050	0.06 (0.07)
	1497 (605)	14847 / 15025	179	0.06 (0.02)
	1219 (91)	14877 / 15046	169	0.05 (0.003)
	511 (85)	14865 / 15050	185	0.021 (0.0028)
	403 (231)	14726 / 15024	298	0.016 (0.007)
	351 (280)	14482 / 14896	414	0.014 (0.009)
	349 (57)	14943 / 15156	213	0.014 (0.002)
	322 (42)	14915 / 15043	128	0.013 (0.001)
	283 (75)	14873 / 15035	162	0.011 (0.002)
	253 (239)	14865 / 15014	149	0.01 (0.008)
	215 (23)	14920 / 15044	124	0.009 (0.0007)
	211 (102)	14873 / 15189	316	0.009 (0.003)
	191 (126)	13430 / 13705	275	0.008 (0.004)
	177 (26)	13458 / 13668	210	0.007 (0.0009)
	173 (85)	14931 / 15078	147	0.007 (0.003)
	158 (43)	13661 / 13798	137	0.006 (0.001)
	154 (0)	13917 / 15288	1371	0.006 (0)
	153 (46)	14462 / 14895	433	0.006 (0.001)
	149 (139)	13559 / 14638	1079	0.006 (0.004)
	132 (137)	13356 / 15252	1896	0.005 (0.004)
	114 (31)	14899 / 15040	141	0.004 (0.001)
	108 (61)	13324 / 13808	484	0.004 (0.002)
	108 (36)	14291 / 15177	886	0.004 (0.001)
	102 (15)	14880 / 15040	160	0.004 (0.0005)
	100 (80)	14347 / 15110	763	0.004 (0.003)
3	230 (N/A)	14716 / 14941	225	0.003 (N/A)
	119 (N/A)	12911 / 14085	1174	0.001 (N/A)
	103 (N/A)	1135 / 1323	188	0.001 (N/A)

^a^ Listed are all insertion- type DVGs identified with 100 or more reads.

^b^ The ratios were calculated based on average estimated coverages of 62550, 24000, 30062 and 86151 reads for full-length genomes in viruses #1, #2, virus #2 (repeat run), virus #3, respectively

N/A not applicable

**Table 5:** Deletion- type DVGs identified in viruses #1 and #2 using DVG-profiler.

Rescue	Reads found with deletion (no. of reads in repeat HiSeq run)^a^	Breakpoint/Reinitiation position	Size of deletion (nt)	Ratio DVG / full-length genome ^c^
**1**	281 (N/A)	13814 / 14707	893	0.014 (N/A)
	179 (N/A)	12274 / 14422	2148	0.003 (N/A)
	119 (N/A)	5163 / 10845	5682	0.002 (N/A)
**2**	62570 (70986)	14589 / 15046^b^	457	2.6 (2.36)
	2015 (1276)	14960 / 15166	207	0.08 (0.04)
	2009 (949)	13916 / 15284	1368	0.08 (0.03)
	1079 (1069)	5785 / 15175	9390	0.04 (0.03)
	665 (1159)	6249 / 14475	8226	0.028 (0.04)
	490 (753)	4175 / 13739	9564	0.02 (0.025)
	304 (242)	14949 / 15155	206	0.012 (0.008)
	296 (288)	14365 / 14881	516	0.012 (0.01)
	244 (246)	2422 / 13755	11333	0.01 (0.008)
	219 (241)	14365 / 14693	328	0.01 (0.008)
	200 (398)	812 / 13431	12619	0.008 (0.013)
	181 (78)	14457 / 14885	428	0.008 (0.003)
	169 (127)	11443 / 15171	3728	0.007 (0.004)
	169 (121)	3196 / 12185	8989	0.007 (0.004)
	168 (209)	2127 / 10975	8848	0.006 (0.007)
	140 (128)	5029 / 12136	7107	0.005 (0.004)
	111 (27)	14890/ 15029	139	0.005 (0.001)
	110 (37)	13315 / 14875	1560	0.004 (0.001)
	106 (146)	1896 / 14927	13031	0.004 (0.005)
	106 (89)	2294 / 14177	11883	0.004 (0.003)

^a^ Listed are all deletion- type DVGs identified with 100 or more reads.

^b^ This deletion was detected in copyback DVGs 13907/15279 and 14223/15166 using primer pair a/b3 (see Table 1). In addition, the existence of the deletion was confirmed by conventional RT-PCR using primers b6 and a that are flanking the deletion.

^c^ The ratios were calculated based on average estimated coverages of 62550, 24000 and 30062 reads for full-length genomes in viruses #1, #2, and virus #2 (repeat run), respectively

N/A not applicable

Among the 14 deletion/insertion type DVGs with more than 1000 reads identified in viruses #1 and #2, only four were deletion type DVGs. In all other cases, insertions in form of duplications of MuV sequences ranging in size between 169 nt and 1050 nt were found (Table 4). For instance, in case of insertion-DVG 14456/14877 (virus #2), a break point at position 14877 is followed by five unmatched nucleotides (cagtt) and reinitiation at position 14456 where the genome sequence resumes in the same orientation presumably up to the genomic 5’end. Of note, we did not observe real snapback DVGs in the virus samples analyzed. Since DVG-profiler does not distinguish between copyback and snapback DVGs, it is possible that some few snapback DVGs escaped our notice, given that there were over 2000 DVGs to be examined. In addition to copyback, insertion and deletion DVGs, we also identified hybrid DVGs that were hybrids of these types. For example, insertion DVG 14847/15025 possessed a breakpoint at position 15025 which is followed by 16 nucleotides matching to genomic positions 15052 to 15030 that are followed by a second breakpoint at position 14868 where the sequence resumes in the same orientation as the sequence before the first breakpoint. Thus, this “mosaic” DVG consists of a duplication and a short stretch of genome resembling a copyback DVG (14868/15030). Two additional “mosaic DVGs were identified in which the 457 nt deletion 14589/15046 mentioned above was detected in the loop regions of copyback DVGs 13908/15277 and 14223/15165 (amplicons 2c2 and 2c3, Fig 9; Table 1). Of note, these two “mosaic” DVGs were shown to be present in virus #2 together with their parental copyback DVGs that lacked the deletion (Table 1).

### Sensitivity of HTS and DVG-profiler to detect DVGs

To further test the sensitivity of DVG-profiler to detect DVGs, we generated an *in vitro* transcribed RNA that encodes a DVG. This DVG was previously identified by DVG-specific RT-PCR from a recombinant MuV coding for the Urabe vaccine strain. The DVG has an overall size of 930 bases and displays a breakpoint / re-initiation junction at nucleotide positions 14687/15153. The RNA transcripts were spiked into RNA prepared from a recombinant mumps virus (r88+JL(M/F/SH/HN). RNA from this virus had been tested previously by HTS (S11 Table) as well as by DVG-specific RT-PCR (using primer pair a/b1) to verify the absence of the 930 nt DVG to be used for spiking. The number of DVG-specific *in vitro* transcribed RNA molecules and full-length virus RNA (r88+JL(M/F/SH/HN) molecules per microliter was determined by qRT-PCR using suitable RNA standards for quantification. *In vitro* transcribed DVG RNA (1.79x10^4^ (low spike) or 1.79x10^6^ molecules (high spike)) was mixed with 7.68 x 10^6^ molecules of full-length recombinant viral RNA and subjected to HTS on a MiSeq instrument. The data were analyzed using DVG-profiler and raw data are provided in S12 and S13 Tables (representing low spike and high spike samples, respectively). 4712 and 55 reads, respectively, specific to the *in vitro* transcribed spiked DVG (14687/15153) were identified in samples spiked with the high or low number of DVG molecules, respectively (corresponding rows in S12 and S13 Tables are each highlighted in green). Based on the average estimated depth of coverage for the full-length genomes (22198 and 25137 reads for the high spike and low spike samples, respectively), DVG / genome ratios of 0.212 and 0.0022, respectively were calculated, which is in very good agreement with the DVG / genome ratios that were predicted based on the calculated amount of input RNA molecules (ratios of 0.233 and 0.0023 for the high spike and low spike samples, respectively).

### Quantitative assessment of DVG- genome ratios

Having shown that the HTS combined with DVG-profiler is suitable to estimate the ratio of DVGs to full-length genomes, we determined the approximate DVG / genome ratios of individual DVGs found in viruses #1, #2 and #3. The number of reads per individual DVGs relative to the average number of full-length reads was determined and data are summarized in Tables 2–5. As shown (Table 2), copyback DVG 14869 / 15030 was present in virus #2 at a DVG / genome ratio of 4.6 to 6.5 and deletion type DVG 14589 / 15046 at a DVG / genome ratio of 2.4 to 2.6 (Table 5). In contrast, the most abundant copyback DVGs in viruses #1 and #3 only displayed DVG / genome ratios of 0.25 and 0.004, respectively (Table 3). These data confirm our initial conclusion (Fig 7) that virus #2 possesses a significantly greater abundance of copyback DVGs as compared to viruses #1 and #3. Since copyback DVGs are potent inducers of innate immune responses [6, 19, 20–22, 26, 29–31] and given that DVGs were detected at much higher levels in virus #2 than in viruses #1 and #3, we postulated that virus #2 would trigger a more robust innate immune response *in vitro*. This was tested with human A549 respiratory epithelial cells that were infected with viruses #1, #2, and #3 and incubated for up to 48 hours. Cell culture supernatants and total RNA prepared from infected cells was analyzed for expression of innate immune response genes *IFNB1*, *IFNL1* and *IFNL2/3* by ELISA [44] and qRT-PCR [45] (S1 File). It was found that virus #2 induced a more potent immune response during the first 24 hours post infection (S5 Fig). Furthermore, virus #1, which exhibited a higher DVG / genome ratio compared to virus #3, also induced a stronger immune response than did virus #3, but at lower levels as compared to virus #2 (S5 Fig). These findings are consistent with the presence of different amounts of copyback DVGs in viruses #1, #2 and #3 and further corroborate the quantitation based on DVG-profiler data.

### Validation of DVG-profiler using data sets obtained from parainfluenza virus 5 and Sendai virus using HTS

To further test the suitability of DVG-profiler to detect copyback DVGs in paramyxovirus samples, we analyzed datasets for two other paramyxoviruses.

In 2013, Killip *et al*. reported the generation of parainfluenza virus 5 (PIV5) preparations with high DVG content. The authors analyzed these preparations by HTS for the presence of DVGs using their own bioinformatics algorithm [31]. The FASTQ-formatted dataset for virus “vM12” was kindly provided to us by the authors and was reanalyzed here using DVG-profiler. As shown in S14 Table, the results obtained are in very good agreement with those published by Killip *et al*. [31]. Accordingly, as highlighted in yellow in S14 Table, DVG-profiler correctly identified the three most abundant copyback DVGs at a similar prevalence to that reported, further validating the functionality of our algorithm.

As mentioned, Beauclair *et al*., [36] recently reported development of another open-source bioinformatics tool, named DI-tector, for identifying DVGs in HTS data. To demonstrate the ability of their tool to identify DVGs, the authors used publicly available HTS data from RNA extracted from Huh-7 cells infected with Sendai virus, another paramyxovirus (www.ncbi.nlm.nih.gov/sra, accession numbers SRX2600182 and SRX2600183). Either total RNA or RNA depleted of ribosomal RNA was employed. We obtained the same datasets and analyzed them using our DVG-profiler. We initially aligned the datasets to the same Sendai virus reference used by Beauclair *et al*. [36] (NC_001552.1). The most prevalent copyback DVG we identified was a well-known DVG (14932/15291) that is prevalent in the Cantell strain of Sendai virus [22]. As shown in S15 and S16 Tables, we identified 522 and 15825 reads, respectively, in the datasets for total RNA and rRNA-depleted RNA. Since the Sendai reference strain used by Beauclair *et al*. [36] did not match 100% to the Cantell strain, we repeated our analysis using the Cantell strain of Sendai virus as a reference (AB855654). As documented in S17 and S18 Tables, we found 18044 and 680 reads for DVG 14932/15291 in the rRNA depleted RNA and total RNA datasets, respectively, resulting in estimated DVG/standard genome ratios of 0.93 and 0.45, respectively (based on an average depth of coverage for standard virus genomes of 19412 and 1503 reads, respectively). In contrast to our finding, Beauclair *et al*.[36] report only 58 reads for DVG 14932/15291 in the rRNA depleted RNA and no reads for this DVG in the total RNA sample. The most abundant DVG identified by DI-tector was not the Cantell strain-specific DVG, but a 3’ copyback DVG (1933/338) with 17 and 110 reads, respectively, in the total RNA dataset and in the rRNA-depleted RNA dataset. Notably, this copyback DVG was not identified by DVG-profiler in any of the two datasets.

The analysis of the Sendai virus datasets suggested that DVG-profiler is a more sensitive tool for detection of DVGs as compared to DI-tector. To further investigate this, we analyzed our HTS dataset generated for virus #2 (first HiSeq run) using the DI-tector package. The results of this analysis are documented in S19 Table. We first compared the reads of all 5’ copyback DVGs shown in Table 2 with those found with DI-tector. As shown in S20 Table, all 31 5’ copyback DVGs with more than 1000 reads identified with DVG-profiler also were found with DI-tector, albeit with a considerably lower number of reads. However, among the six 5’ copyback DVGs that were detected by RT-PCR and DVG-profiler with less than 1000 reads, only three were also found with DI-tector, further supporting the higher sensitivity of DVG-profiler. A comprehensive comparison of all copyback DVGs (S21 Table), insertion type DVGs (S22 Table) and deletion type DVGs (S23 Table) identified with DI-tector with those found with DVG-profiler further corroborated this notion. As summarized in Table 6, the percentage of DVGs that were detected by both DVG-profiler and DI-tector decreased with decreasing number of DVG-profiler reads per DVG. It should be mentioned that there was one 5’copyback DVG (8928/14744; 2 reads), three 3’ copyback DVGs (86/92; 4 reads; 15203/11551, 8 reads; 14785/14199; 2 reads), one deletion type DVG (5209/14878; 5 reads) and two insertion type DVGs (15098/15059; 2 reads; 14452/14390; 2 reads) that were found using DI-tector, but not using DVG-profiler. However, these numbers are extremely low compared to the more than 4000 DVGs that were found by DVG-profiler and not by DI-tector (Table 6). Whether these few DVGs were false positive hits or real DVGs missed by DVG-profiler has not been further investigated.

**Table 6:** Comparison of the sensitivity of DVG-profiler and DI-tector to detect copyback, deletion and insertion DVGs in virus #2.

Reads per DVG identified using DVG-profiler	Number of DVGs identified with DVG-profiler vs DI-tector (percentage of same DVGs found by DI-tector compared to DVG-profiler)		
	Copyback DVGs	Insertion DVGs	Deletion DVGs
**1000–120000**	31 / 31 (100)	7 / 6 (85.7)	4 / 4 (100)
**100–999**	59 / 11 (81.4)	23 / 14 (60.9)	16 / 14 (87.5)
**50–99**	84 / 20 (23.8)	26 / 5 (19.23)	22 / 8 (36.4)
**10–49**	395 / 16 (4.05)	144 / 15 (10.42)	139 / 11 (7.9)
**2–9**	1793 / 8 (0.44)	869 / 1 (0.12)	947 / 4 (0.42)

## Discussion

In this report, we propose a novel bioinformatics algorithm that allows selective detection of all defective viral genomes present within an HTS data set obtained from a virus sample. To establish the specificity of the DVG-profiler algorithm that we developed, we took advantage of HTS data derived from three recombinant MuVs rescued from the same cDNA. One of these viruses displayed a sharp spike in the number of reads at the 5’ terminus, suggestive of the presence of a high concentration of copyback DVGs. The presence of copyback DVGs in these samples was confirmed by DVG specific RT-PCR, which initially -based on two primer sets- identified 15 unique copyback DVGs. Of these, 13 were also identified by DVG-profiler. The inability of DVG-profiler to detect the other two DVG species is likely a reflection of the relative abundance of these species being below the threshold of detection by the HTS technology. Supporting this assumption is the fact that one of these DVG species (14596/14896) was subsequently detected–albeit in only six sequencing reads- when HTS was repeated using a new RNA sample prepared from the same virus. In this repeat run, the overall depth of coverage was significantly higher compared to the first run, increasing the likelihood of detecting this DVG. Based on this and other data presented here, the sensitivity of HTS to detect very few DVG molecules in a background of total RNA extracted from cell culture supernatant appears to be inferior to that of RT-PCR. However, we also demonstrate here the ability of HTS to identify hundreds of DVG species that could not be detected in our RT-PCR runs, but this is because of RT-PCR primer specificity. Indeed, using two additional DVG specific primer sets, we confirmed the presence of 12 of these DVG species in virus #2. Nevertheless, it would be impractical, if not virtually impossible, to design a panel of PCR primers capable of detecting all possible copyback, insertional, and deletional DVGs. Further, some DVG amplicons can be quite large and fail to amplify sufficiently under the chosen PCR conditions to be visualized on the agarose gel.

RdRps of RNA viruses exhibit higher error rates than DNA polymerases. In addition, DVGs may be subject to extensive RNA editing induced by the action of the ADAR (adenosine deaminase acting on RNA) enzyme resulting in clusters of A to G or U to C transitions [28, 46]. Thus, our alignment tool had to accommodate the presence of mismatches close to the junction sites. The number of allowed mismatches can be chosen as a parameter for the initial alignment required before using DVG-profiler and in our analysis, we chose a 15% mismatch tolerance. While this setting provides a high degree of sensitivity, it is at the cost of specificity, leading to some false -positive hits due to the presence of homologous sequence strings within the viral genome (reference sequence) or to homology of cellular gene sequences to the reference sequence. Therefore, to verify that DVGs identified represent *bona fide* DVGs and not artefacts, it is recommended to download the alignments and reads for a given DVG and align the reads against the reference sequences. More stringent alignment and DVG-profiler settings can also be used to check specificity, but this comes at the cost of sensitivity. DVG-profiler is an algorithm that succeeds an alignment process, consequently the performance of the alignment step constitutes a limiting factor. Hence, proper parameterization of the aligner is required before any application of the DVG-profiler.

While we cannot be certain if artefacts occur and are called by DVG-profiler as *bona fide* DVGs, the ratio of those false DVGs to genome would be infinitesimally small, and, if encountered in a viral sample, their biological significance would be questionable. To reduce or even eliminate the possibility of such false positives from entering the data, the initial screening should employ loose parameters such as 17 mer minimum match alignment and 15% mismatch tolerance to cover all DVGs and then in a subsequent run, the parameters can be tightened, for example 15 mer minimum match alignment with 5% mismatch.

In addition to identifying all DVGs present in a given virus sample, a quantitative assessment of findings is crucial to determine the significance of DVGs identified. Optimal quantitation of a given DVG currently relies on qRT-PCR. The latter requires primers that are specifically designed for a given DVG, primers that only recognize the full-length viral genomes, generation of RNA standards derived from the DVG, as well as standards to determine the amount of full-length viral genomes. This approach is clearly impracticable. Here we show that using HTS and DVG-profiler, the number of reads can be used to approximate the relative abundance of DVGs. We also show here that DVGs present at DVG/genome ratios as low as 0.002 can still be detected by HTS. However, the fact that even 18,000 molecules of copyback DVG only yielded 55 hits suggests that DVGs that are detected in only very few reads are present in the viral sample in numbers above several hundred to 1000 molecules. Those numbers can easily be detected by RT-PCR, but appear to be the limit of detection for HTS, when present in a large background of full-length viral genomes and cellular RNA. Thus, RT-PCR appears to be more sensitive in detecting low copy numbers of DVGs as compared to HTS. However, as mentioned earlier, DVGs that exist below the threshold of detection by HTS are unlikely to be of biological significance.

It should be mentioned that the number of reads per DVG might be biased in situations where certain areas of the viral sequence are amplified disproportionally compared to others. To mitigate this bias and potential PCR or sequencing errors and PCR sampling bias, attempts are currently under way to make libraries using adapters that contain unique molecular identifiers (UMIs) as used in methods for detecting low frequency mutations. This should allow for more accurate estimation of both the average genome numbers and the relative numbers of DVGs.

The suitability of DVG-profiler to accurately detect DVGs in virus samples was further assessed using previously published HTS datasets derived from purified preparations of PIV5 that were highly enriched with DVGs [31]. The fact that our results were in very good agreement with the published results further validates DVG-profiler. The recently published DI-tector algorithm by Beauclair *et al*. [36] for detecting DVGs in HTS data differs from DVG-profiler in its bioinformatics approach. DVG-profiler is an algorithm designed to detect all possible junctions, given a set of sequences, considering higher mutation rates often exhibited by the same viruses that produce the DVGs. DVG-profiler applies a peak detection filter that groups junctions reported around the same positions that are potentially products of further mutated DVGs. Compared to DI-tector it reports the number of reads that cover the junction for each direction which enables users to filter potential sequencing or alignment artefacts. Moreover, DVG-profiler allows the detection of junctions between more than one sequence. Although this feature is beyond the scope of identifying DVGs, it enables further analyses, including detection of insertions from other sequences and nested DVGs. To directly compare the two tools, we used an *in silico* data set as well as datasets derived from Sendai virus and MuV infected cells. Using an *in silico* data set we could show that DVG-profiler exhibited higher precision compared to DI-tector. Furthermore, using publicly available HTS datasets established from Sendai virus infected cells, DVG-profiler appeared to be more sensitive in that a well-known DVG present in the Cantell strain of Sendai virus was detected by DVG-profiler in abundance in both total RNA, and ribosomal RNA (rRNA) depleted RNA prepared from infected cells. In contrast, DI-tector only detected the same DVG in the rRNA depleted sample but not in the total RNA sample. Finally, when subjecting our HTS data to analysis using DI-tector, we found that the latter was much less sensitive in its ability to detect DVGs with low abundance compared to DVG-profiler. At this point we do not know whether this is intrinsic to the different algorithms to detect DVGs or due to differences in the alignment algorithm. Since the DI-tector package available to the public does not allow one to separate the alignment algorithm from the DVG detecting algorithm, we were not able to address this question. It should be mentioned that DVG-profiler also appeared to exhibit much better speed performance compared to DI-tector. This was observed when comparing the *in silico* dataset and when analyzing the HTS data using DI-tector. Analysis of the latter took several hours to complete compared to only several minutes using DVG-profiler. However, again, whether these obvious differences in speed performance are due to the alignment algorithm or DVG algorithm, or both, remains to be shown.

In summary, our data presented here shows that DVG-profiler is a fast, highly sensitive and specific tool to detect DVGs in viral RNA preparations subjected to HTS. Given the importance of DVGs in innate immunity and its potential impact on vaccine efficacy, this tool might prove valuable not only in basic virus research but also in monitoring live attenuated vaccines for DVG content and to assure lot to lot consistency.

## Supporting information

S1 Fig Identification by RT-PCR of trailer copyback DVG 13811/14697 in virus #1 using primer pair a/b5. RNA extracted from virus #1 was reverse transcribed using primer a and cDNA was amplified with primer pair a/b5. The PCR product was analyzed on a 0.8% ethidium bromide stained agarose gel. A fragment of the expected size of 776 bp was seen in the PCR reaction using cDNA prepared with the RT enzyme (+) but not in the PCR reaction using material that was not subjected to reverse transcription (-). See materials and methods and Fig 8 for further information.
(TIF)

S2 Fig Positional histogram of sensitivity assessment. The alignments supporting each detected junction were examined and the true and false positives were computed for each position used to generate reads that cover the junctions. Analyses were performed on samples ISDP CB1-5 (5’ copy-back DVG) and ISDP D1-5 (DVG with deletion). Each sample was analyzed 5 times with 4 different values of minimum read length allowed by the alignment process, namely 10, 15, 20 and 25 nucleotides. The results for each threshold TP10, TP15, TP20 and TP25 are available for all samples.
(TIF)

S3 Fig Impact of read length in DVG-profilers sensitivity. Sensitivity values after analysis of DVG S1 and S2 (5’cb and deletion DVG respectively) *in silico* datasets containing reads of different length (50, 100, 150, 200 and 250bp) and aligned with different thresholds for minimum read length (10, 15, 20 and 25).
(TIF)

S4 Fig Test of reproducibility across two independent RNA preparations. To test the reproducibility of HTS and DVG-profiler results, a second sample of RNA was prepared from virus #2 and subjected to a second HTS run on a HiSeq instrument (“2^nd^ HiSeq run”). Data were analyzed using DVG-profiler. Numbers of reads per copyback DVG (panel a) or deletion / insertion DVG (panel b) identified in the 1^st^ HiSeq run were correlated to the number of reads for the same copyback DVGs or deletion /insertion DVGs recorded in the 2^nd^ HiSeq run (all DVGs with ≥ 50 reads in the 1^st^ and 2^nd^ HiSeq run were correlated). Correlation analysis was carried out using the SigmaPlot 11.0 software package (Systat software, Inc., Chicago, Il). Correlation coefficients (r) are indicated in the graphs.
(TIF)

S5 Fig Kinetics of type I and type III interferon expression in A549 cells following infection with viruses #1, #2 and #3. A549 cells were infected with viruses #1, #2 or #3 at an m.o.i. of 0.43 and cell culture supernatants were collected at 0, 8, 16, 24 and 48 hours post infection to measure the secreted levels of type I (IFN-β) and type III (IFN-λ1, 2, 3) interferons by ELISA (upper panels). Expression levels of the type I and type III interferon genes (*IFNB1*, *IFNL1* and *IFNL2/3*) were determined by qRT-PCR using total RNA extracts prepared from A549 cell cultures at the indicated time points. Expression levels of the interferon genes were normalized against expression levels of the housekeeping gene, GAPDH, and are plotted as fold- increase compared to uninfected cells (lower panels). Each time point was measured in triplicate and each point represents the mean ± SD of triplicate determinations.
(TIF)

S1 Table *In silico* random reads generated from 5’ copyback DVG dvg S1 and deletion DVG dvg S2. (DOCX)

S2 Table *In silico* generation of eight template sequences. (DOCX)

S3 Table *In silico* spiking of reads generated from the reference genome with different concentrations of reads generated from dvg3 or with reads generated from a mixture of eight different DVGs (dvg1 –dvg8). (DOCX)

S4 Table Sequences of primers used for RT-PCR and qRT-PCR. (DOCX)

S5 Table Comparison of the sensitivity and specificity of DVG-profiler and DI-tector algorithms using data sets generated *in silico*. (DOCX)

S6 Table DVG-profiler raw data generated from HTS data obtained for virus #1 (HiSeq). (PDF)

S7 Table DVG-profiler raw data generated from HTS data obtained for virus #2 (HiSeq). (PDF)

S8 Table DVG-profiler raw data generated from HTS data obtained for virus #3 (HiSeq). (PDF)

S9 Table DVG-profiler raw data generated from HTS data obtained for virus #2, repeat HiSeq run. (PDF)

S10 Table Repeatability of results. (DOCX)

S11 Table DVG-profiler raw data generated from HTS data obtained for virus r88+JL(M/F/SH/HN). (PDF)

S12 Table DVG-profiler raw data generated from HTS data: RNA prepared from virus r88+JL(M/F/SH/HN), mixed with 1.79 x 10^4^ molecules (low spike) of *in vitro* transcribed RNAs from DVG 14687/15153 (930 nt). (PDF)

S13 Table DVG-profiler raw data generated from HTS data: RNA prepared from virus r88+JL(M/F/SH/HN), mixed with 1.79 x 10^6^ molecules (high spike) of *in vitro* transcribed RNAs from DVG 14687/15153 (930 nt). (PDF)

S14 Table DVG-profiler raw data generated from HTS data obtained for PIV5 virus “vM12” [31] (Provided by Rick Randall, University of St. Andrews, St. Andrews, UK). (PDF)

S15 Table DVG-profiler raw data generated from publicly available HTS data (www.ncbi.nlm.nih.gov/sra, accession number SRX2600183): Total RNA extracted from Sendai virus infected Huh-7 cells using as reference a Sendai virus strain with accession number NC_001552.1. (PDF)

S16 Table DVG-profiler raw data generated from publicly available HTS data (www.ncbi.nlm.nih.gov/sra, accession number SRX2600182): RNA extracted from Sendai virus infected Huh-7 cells and depleted of ribosomal RNA, using as reference Sendai virus strain with accession number NC_001552.1. (PDF)

S17 Table DVG-profiler raw data generated from publicly available HTS data (www.ncbi.nlm.nih.gov/sra, accession number SRX2600182): RNA extracted from Sendai virus infected Huh-7 cells and depleted of ribosomal RNA, using as reference Sendai virus strain Cantell with accession number AB855654.1. (PDF)

S18 Table DVG-profiler raw data generated from publicly available HTS data (www.ncbi.nlm.nih.gov/sra, accession number SRX2600183): Total RNA extracted from Sendai virus infected Huh-7 cells, using as reference Sendai virus strain Cantell with accession number AB855654.1. (PDF)

S19 Table DI-tector raw data generated from HTS data obtained from virus #2 (first HiSeq run). (DOCX)

S20 Table Comparison of the number of reads of all 5’copyback DVGs shown in Table 2 (all DVG-profiler data with over 1000 reads and RT-PCR positive hits; virus #2, HiSeq first run) with the number of reads recorded for these copyback DVGs when using DI-tector. (DOCX)

S21 Table Comparison of the number of reads for all copyback DVGs with less than 1000 reads identified in virus #2 (HiSeq first run) using DVG-profiler, with the number of reads for these DVGs when using DI-tector. Highlighted in yellow: all DVGs with no reads identified using DI-tector.
(PDF)

S22 Table Comparison of the number of reads for all insertion-type DVGs with more than 100 reads identified in virus #2 (HiSeq first run) using DVG-profiler, with the number of reads for these DVGs when using DI-tector. (DOCX)

S23 Table Comparison of the number of reads for all deletion-type DVGs with more than 100 reads identified in virus #2 (HiSeq first run) using DVG-profiler, with the number of reads for these DVGs when using DI-tector. (DOCX)

S1 File Supplemental materials and methods. (DOCX)
