# Supplementary figures and images for: Identification and quantification of defective virus genomes in high throughput sequencing data using DVG-profiler, a novel post-sequence alignment processing algorithm
Source: PLoS One. 2019 May 17;14(5):e0216944. doi: 10.1371/journal.pone.0216944 (PMC6524942; doi:10.1371/journal.pone.0216944)

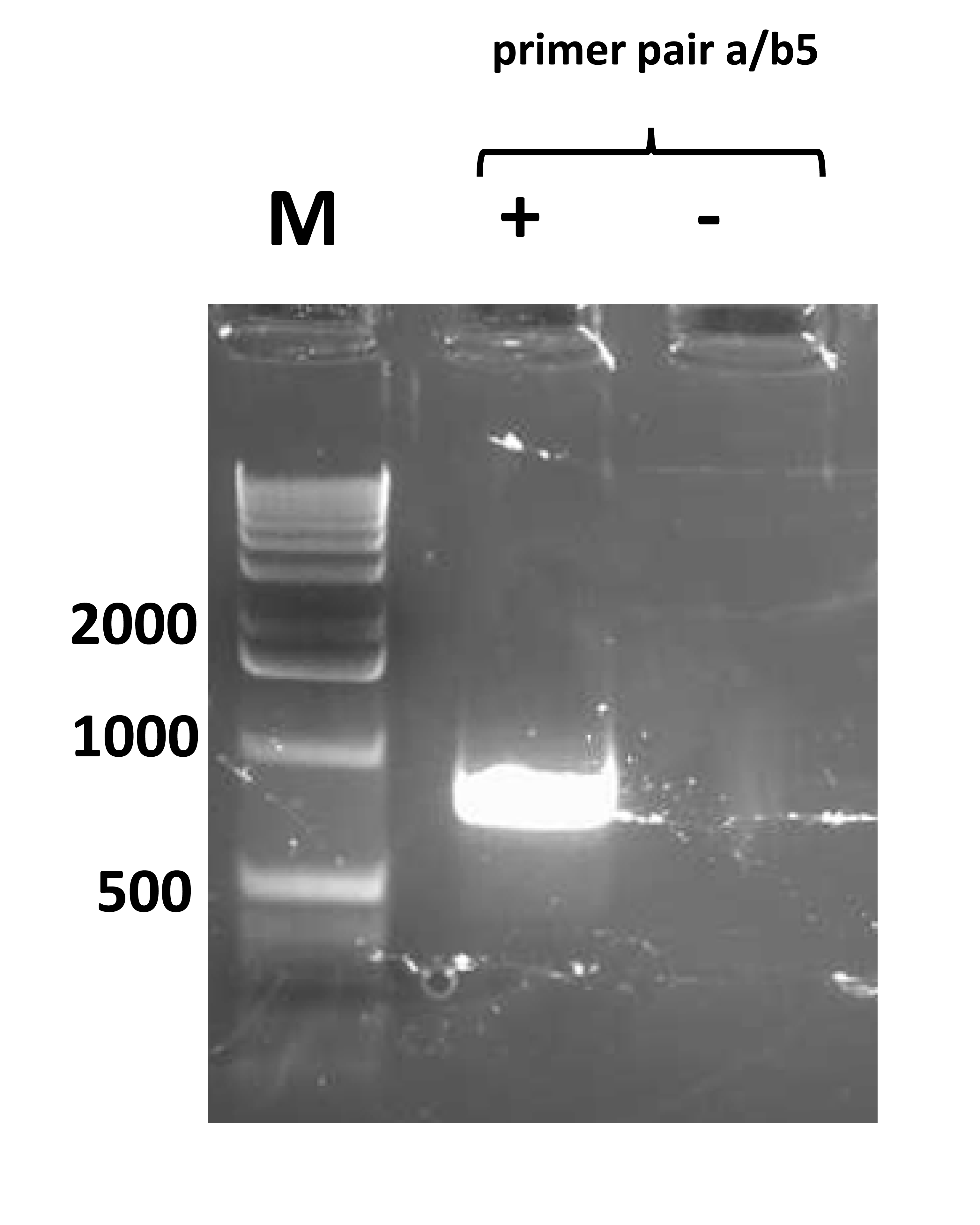

Supplement: S1 Fig — RNA extracted from virus #1 was reverse transcribed using primer a and cDNA was amplified with primer pair a/b5. The PCR product was analyzed on a 0.8% ethidium bromide stained agarose gel. A fragment of the expected size of 776 bp was seen in the PCR reaction using cDNA prepared with the RT enzyme (+) but not in the PCR reaction using material that was not subjected to reverse transcription (-). See materials and methods and Fig 8 for further information. (TIF) [file pone.0216944.s001.tif]

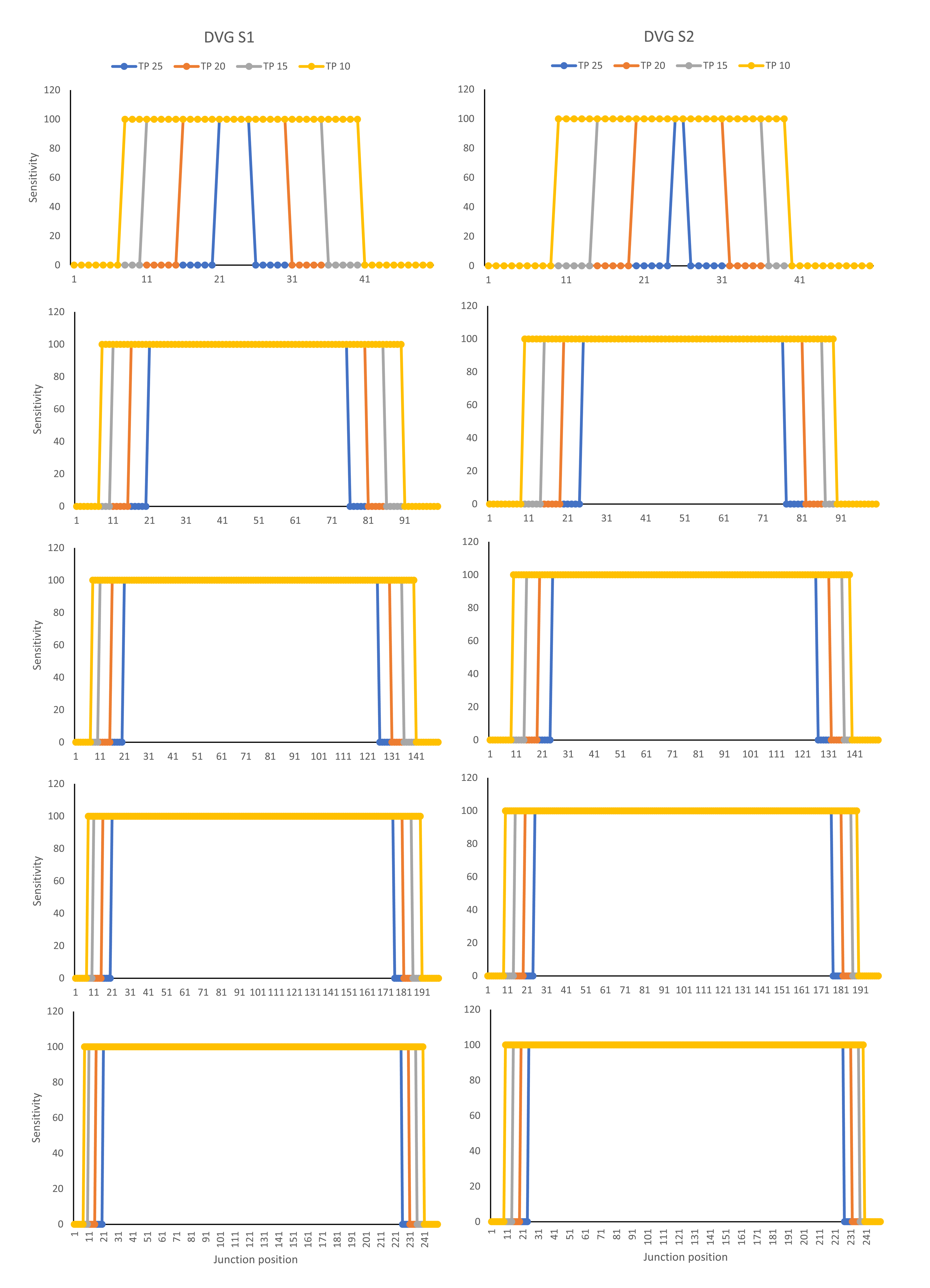

Supplement: S2 Fig — The alignments supporting each detected junction were examined and the true and false positives were computed for each position used to generate reads that cover the junctions. Analyses were performed on samples ISDP CB1-5 (5’ copy-back DVG) and ISDP D1-5 (DVG with deletion). Each sample was analyzed 5 times with 4 different values of minimum read length allowed by the alignment process, namely 10, 15, 20 and 25 nucleotides. The results for each threshold TP10, TP15, TP20 and TP25 are available for all samples. (TIF) [file pone.0216944.s002.tif]

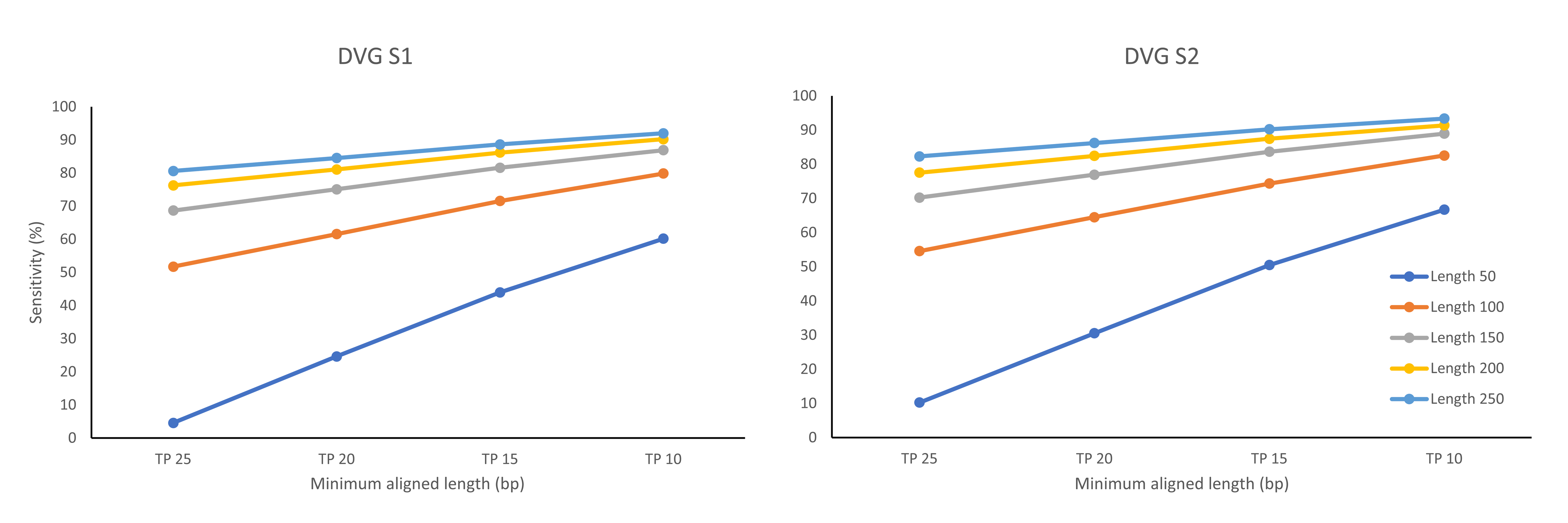

Supplement: S3 Fig — Sensitivity values after analysis of DVG S1 and S2 (5’cb and deletion DVG respectively) in silico datasets containing reads of different length (50, 100, 150, 200 and 250bp) and aligned with different thresholds for minimum read length (10, 15, 20 and 25). (TIF) [file pone.0216944.s003.tif]

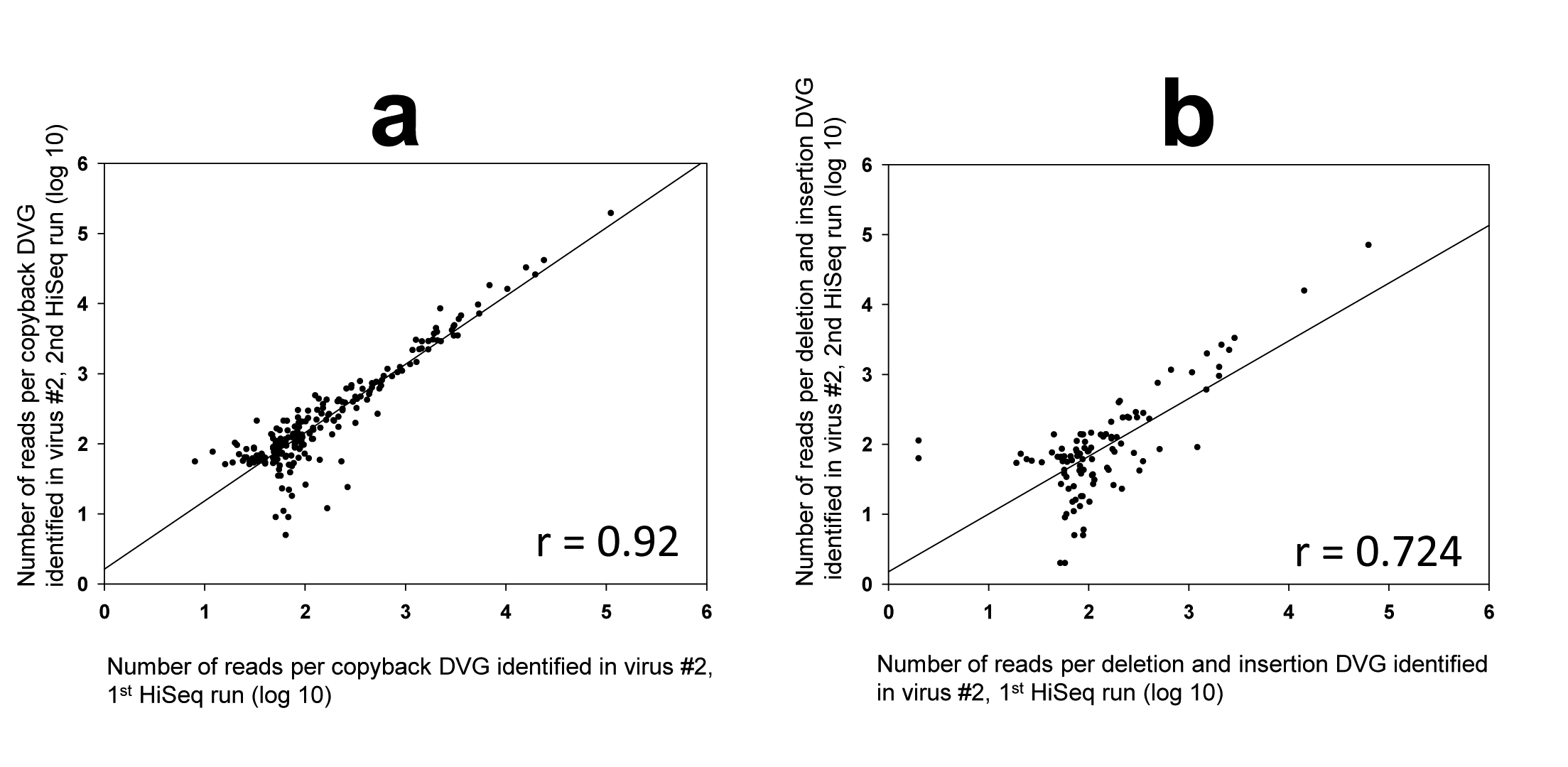

Supplement: S4 Fig — To test the reproducibility of HTS and DVG-profiler results, a second sample of RNA was prepared from virus #2 and subjected to a second HTS run on a HiSeq instrument (“2nd HiSeq run”). Data were analyzed using DVG-profiler. Numbers of reads per copyback DVG (panel a) or deletion / insertion DVG (panel b) identified in the 1st HiSeq run were correlated to the number of reads for the same copyback DVGs or deletion /insertion DVGs recorded in the 2nd HiSeq run (all DVGs with ≥ 50 reads in the 1st and 2nd HiSeq run were correlated). Correlation analysis was carried out using the SigmaPlot 11.0 software package (Systat software, Inc., Chicago, Il). Correlation coefficients (r) are indicated in the graphs. (TIF) [file pone.0216944.s004.tif]

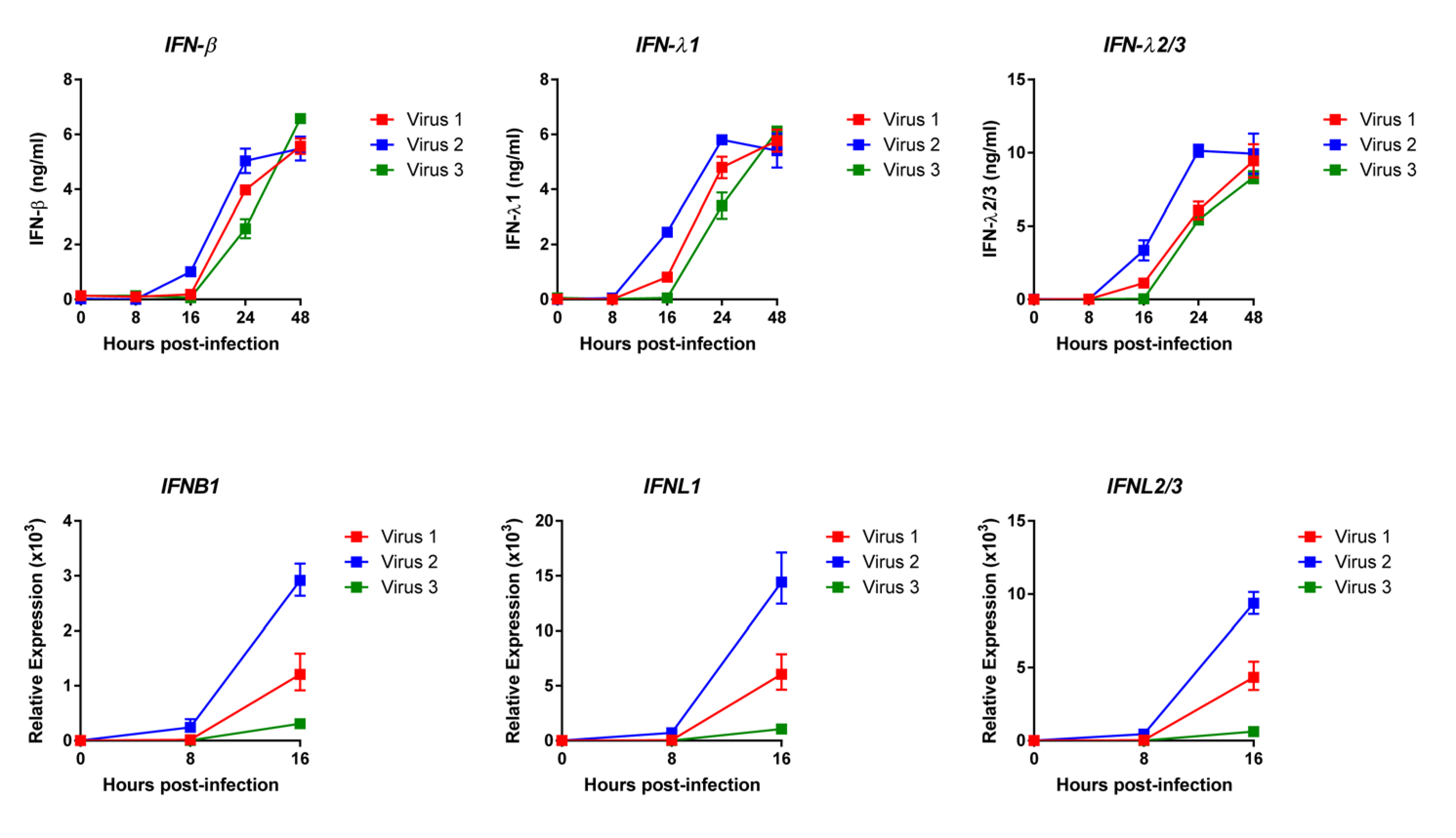

Supplement: S5 Fig — A549 cells were infected with viruses #1, #2 or #3 at an m.o.i. of 0.43 and cell culture supernatants were collected at 0, 8, 16, 24 and 48 hours post infection to measure the secreted levels of type I (IFN-β) and type III (IFN-λ1, 2, 3) interferons by ELISA (upper panels). Expression levels of the type I and type III interferon genes (IFNB1, IFNL1 and IFNL2/3) were determined by qRT-PCR using total RNA extracts prepared from A549 cell cultures at the indicated time points. Expression levels of the interferon genes were normalized against expression levels of the housekeeping gene, GAPDH, and are plotted as fold- increase compared to uninfected cells (lower panels). Each time point was measured in triplicate and each point represents the mean ± SD of triplicate determinations. (TIF) [file pone.0216944.s005.tif]
