# Supplementary material for: Identification and quantification of defective virus genomes in high throughput sequencing data using DVG-profiler, a novel post-sequence alignment processing algorithm
Source: PLoS One. 2019 May 17;14(5):e0216944. doi: 10.1371/journal.pone.0216944 (PMC6524942; doi:10.1371/journal.pone.0216944)
Supplement: S1 Table — (DOCX) [file pone.0216944.s006.docx]

**S1 Table. *In silico* random reads generated from 5’ copyback DVG dvg S1 and deletion DVG dvg S2.**

| **Sample name** | **Reads length** | **Read count** | **Genomes** |
| --- | --- | --- | --- |
| ISDP CB1 | 50 | 1,000,000 | dvg S1 |
| ISDP CB2 | 100 | 1,000,000 | dvg S1 |
| ISDP CB3 | 150 | 1,000,000 | dvg S1 |
| ISDP CB4 | 200 | 1,000,000 | dvg S1 |
| ISDP CB5 | 250 | 1,000,000 | dvg S1 |
| ISDP D1 | 50 | 1,000,000 | dvg S2 |
| ISDP D2 | 100 | 1,000,000 | dvg S2 |
| ISDP D3 | 150 | 1,000,000 | dvg S2 |
| ISDP D4 | 200 | 1,000,000 | dvg S2 |
| ISDP D5 | 250 | 1,000,000 | dvg S2 |
