# Supplementary material for: Identification and quantification of defective virus genomes in high throughput sequencing data using DVG-profiler, a novel post-sequence alignment processing algorithm
Source: PLoS One. 2019 May 17;14(5):e0216944. doi: 10.1371/journal.pone.0216944 (PMC6524942; doi:10.1371/journal.pone.0216944)
Supplement: S2 Table — (DOCX) [file pone.0216944.s007.docx]

**S2 Table. *In silico* generation of eight template sequences.**

| **Name** | **Genome** | **Breakpoint** | **Reinitiation point** | **Delta** | **Length (b)** |
| --- | --- | --- | --- | --- | --- |
| ref | Reference | n/a | n/a | n/a | 15384 |
| dvg1 | 5’ cb | 13363 | 14924 | 561 | 2483 |
| dvg2 | 5’ cb | 13257 | 14191 | 934 | 3322 |
| dvg3 | 3’ cb | 520 | 2312 | 1792 | 2832 |
| dvg4 | 3’ cb | 5585 | 5686 | 101 | 11271 |
| dvg5 | Deletion | 522 | 5587 | 5065 | 10320 |
| dvg6 | Deletion | 521 | 542 | 21 | 15364 |
| dvg7 | Insertion | 2311 | 2292 | 19 | 15404 |
| dvg8 | Insertion | 5686 | 5586 | 100 | 15485 |
