# Supplementary material for: Identification and quantification of defective virus genomes in high throughput sequencing data using DVG-profiler, a novel post-sequence alignment processing algorithm
Source: PLoS One. 2019 May 17;14(5):e0216944. doi: 10.1371/journal.pone.0216944 (PMC6524942; doi:10.1371/journal.pone.0216944)
Supplement: S3 Table — (DOCX) [file pone.0216944.s008.docx]

**S3 Table. *In silico* spiking of reads generated from the reference genome with different concentrations of reads generated from dvg3 or with reads generated from a mixture of eight different DVGs (dvg1 – dvg8).**

| **Dataset** | **Composition** | **Read count** | **Read length** | **Av. Depth of Coverage** | **Prevalence (%)** |
| --- | --- | --- | --- | --- | --- |
| **SED1** | ref | 1,000,000 | 100 | 6500.26 | 99.95 |
|  | dvg3 | 100 | 100 | 3.53 | 0.05 |
| **SED2** | ref | 1,000,000 | 100 | 6500.26 | 99.46 |
|  | dvg3 | 1,000 | 100 | 35.31 | 0.54 |
| **SED3** | ref | 1,000,000 | 100 | 6500.26 | 94.85 |
|  | dvg3 | 10,000 | 100 | 353.11 | 5.15 |
| **SED4** | ref | 1,000,000 | 100 | 6500.26 | 64.80 |
|  | dvg3 | 100,000 | 100 | 3531.07 | 35.20 |
| **SED5** | ref | 1,000,000 | 100 | 6500.26 | 15.55 |
|  | dvg3 | 1,000,000 | 100 | 35310.73 | 84.45 |
| **SPD** | ref | 167463 | 100 | 1088.55 | 11.11 |
|  | dvg1 | 27029 | 100 | 1088.55 | 11.11 |
|  | dvg2 | 36162 | 100 | 1088.55 | 11.11 |
|  | dvg3 | 30828 | 100 | 1088.55 | 11.11 |
|  | dvg4 | 122691 | 100 | 1088.55 | 11.11 |
|  | dvg5 | 112339 | 100 | 1088.55 | 11.11 |
|  | dvg6 | 167245 | 100 | 1088.55 | 11.11 |
|  | dvg7 | 167681 | 100 | 1088.55 | 11.11 |
|  | dvg8 | 168563 | 100 | 1088.55 | 11.11 |
