# Supplementary material for: Identification and quantification of defective virus genomes in high throughput sequencing data using DVG-profiler, a novel post-sequence alignment processing algorithm
Source: PLoS One. 2019 May 17;14(5):e0216944. doi: 10.1371/journal.pone.0216944 (PMC6524942; doi:10.1371/journal.pone.0216944)
Supplement: S4 Table — (DOCX) [file pone.0216944.s009.docx]

**S4 Table. Sequences of primers used for RT-PCR and qRT-PCR.**

| Primer name | Nucleotide position in MuV genome | Polarity | Sequence (5’→ 3’) |
| --- | --- | --- | --- |
| a | 15,363 - 15,384 | reverse | ACCAAGGGGAGAAAGTAAAATC |
| b1 | 14,775 - 14,793 | reverse | AGGGTGTCATGATCTGATG |
| b2 | 14,906 - 14,925 | reverse | GGTAAGGTGAGACGAGTTTG |
| b3 | 15,107 - 15,130 | reverse | GCAATAGATTACACACCCAATGGC |
| b4 | 13,623 - 13,647 | reverse | GTAAGGGATGCTGATTCGGATGAAC |
| b5 | 13,877 - 13,899 | reverse | ATAGAGGCTCCACTTCCCTCTGC |
| b6 | 14,375 - 14,394 | forward | CGATCTTCATACTCCGACCC |
| mu0f | 44 - 66 | forward | gtaagaaacagtaagcccggaag |
| 1112r | 1,090 - 1,112 | reverse | ccattatttggggagcctccaac |
| 14790f | 14,767 – 14,790 | forward | gggccaatcatcagatcatgacac |
| 612F | 612 - 630 | forward | catgtgatgagattgagca |
| 667R | 647 – 667 | reverse | agcctggattagtacactgta |
| 15315F | 15,315- 15,337 | forward | cataaccacatcagtattaaatc |
