# Supplementary material for: Identification and quantification of defective virus genomes in high throughput sequencing data using DVG-profiler, a novel post-sequence alignment processing algorithm
Source: PLoS One. 2019 May 17;14(5):e0216944. doi: 10.1371/journal.pone.0216944 (PMC6524942; doi:10.1371/journal.pone.0216944)
Supplement: S5 Table — (DOCX) [file pone.0216944.s010.docx]

**S5 Table.** **Comparison of the sensitivity and specificity of DVG-profiler and DI-tector using data sets generated *in silico*.**

| **Dataset** | **DVGs** | **TP** | **FP** | **Recall (%)** | **Precision (%)** | **Divergence** | **Time** |
| --- | --- | --- | --- | --- | --- | --- | --- |
| **DVG-Profiler** | | | | | | | |
| **SED1** | 1 | 1 | 0 | 100 | 100 | 4.24E-04 | 12 s |
| **SED2** | 1 | 1 | 0 | 100 | 100 | 1.10E-05 | 37 s |
| **SED3** | 1 | 1 | 0 | 100 | 100 | 2.94E-02 | 58 s |
| **SED4** | 1 | 1 | 0 | 100 | 100 | 5.61E-02 | 73 s |
| **SED5** | 1 | 1 | 0 | 100 | 100 | 3.99E-02 | 95 s |
| **SPD** | 8 | 8 | 0 | 100 | 100 | 7.87E-02 | 95 s |
| **DI-tector** | | | | | | | |
| **SED1** | 1 | 1 | 1 | 100 | 50.00 | 4.70E-03 | 17 min |
| **SED2** | 1 | 1 | 1 | 100 | 50.00 | 4.50E-02 | 20 min |
| **SED3** | 1 | 1 | 2 | 100 | 33.33 | 0.42 | 28 min |
| **SED4** | 1 | 1 | 2 | 100 | 33.33 | 3.37 | 36 min |
| **SED5** | 1 | 1 | 2 | 100 | 33.33 | 8.01 | 52 min |
| **SPD** | 8 | 8 | 23 | 100 | 25.81 | 7.40 | ~ 2 h |
