# Supplementary material for: Identification and quantification of defective virus genomes in high throughput sequencing data using DVG-profiler, a novel post-sequence alignment processing algorithm
Source: PLoS One. 2019 May 17;14(5):e0216944. doi: 10.1371/journal.pone.0216944 (PMC6524942; doi:10.1371/journal.pone.0216944)
Supplement: S6 Table — (PDF) [file pone.0216944.s011.pdf]

Position (left)	Group start (left)	Group end (left)	Strandness (left)	Position (right)	Group start (right)	Group end (right)	Strandness (right)	Forward hits	Reverse hits	Fw and Rev
13811	13807	13815	-	14696	14692	14699	+	10303	5036	15339
13811	13807	13815	-	14707	14703	14709	-	3809	486	4295
13819	13816	13824	-	14712	14709	14716	-	518	2094	2612
13833	13830	13837	-	14707	14705	14711	-	971	700	1671
13722	13720	13727	-	13899	13898	13903	-	769	186	955
14011	14009	14014	-	14784	14781	14786	+	475	439	914
14873	14873	14876	-	15104	15101	15107	+	273	199	472
12101	12101	12105	-	14607	14605	14607	+	152	179	331
10694	10692	10697	-	14876	14873	14876	+	24	297	321
12112	12108	12113	-	14616	14613	14618	-	65	250	315
13722	13720	13727	-	13903	13900	13905	+	169	144	313
13814	13814	13815	+	14707	14707	14708	+	178	103	281
5078	5074	5079	-	15083	-	-	+	11	216	227
14020	14019	14025	-	14790	14787	14794	-	113	84	197
14561	14561	14565	-	14756	14756	14758	+	65	115	180
12274	-	-	+	14422	-	-	+	89	90	179
14689	14687	14692	+	14715	14712	14717	-	156	7	163
97	94	101	+	82	81	83	-	91	58	149
13419	13415	13419	-	14189	-	-	+	89	58	147
13866	13863	13867	-	14964	14964	14967	+	51	80	131
82	78	85	+	97	-	-	-	82	48	130
5165	5163	5166	+	10846	10845	10847	+	40	79	119
13827	13826	13828	-	14701	14700	14702	-	106	1	107
14011	14009	14014	-	14769	-	-	-	73	32	105
14045	14043	14049	-	14606	14605	14606	-	54	46	100
14905	14904	14908	-	14943	14940	14944	+	35	60	95
10581	10581	10584	-	13720	-	-	+	47	42	89
14134	14129	14134	-	14200	14195	14200	-	8	73	81
14045	14043	14049	-	14612	14612	14614	+	43	37	80
7607	7604	7607	-	9517	9517	9518	+	6	73	79
14054	14051	14058	-	14621	14618	14624	-	31	48	79
14020	14019	14025	-	14202	14202	14203	-	0	72	72
9379	9378	9379	+	14317	-	-	+	31	39	70
11916	11916	11917	-	14437	14436	14437	+	26	44	70
1407	1405	1407	-	1450	-	-	+	40	24	64
4787	4784	4787	+	12112	-	-	+	28	30	58
714	712	718	+	742	738	742	-	30	27	57
14578	14576	14580	-	14823	14821	14823	+	33	24	57
2493	-	-	+	2600	-	-	+	32	24	56
2941	2938	2941	-	2962	-	-	+	54	1	55
624	624	629	+	14814	-	-	+	21	32	53
700	698	700	+	756	-	-	-	33	20	53
13780	13776	13780	-	14355	14355	14357	+	22	29	51
3215	3212	3218	-	3340	3340	3342	-	34	16	50
12591	12589	12594	-	14427	-	-	+	31	19	50
12634	12631	12636	-	14859	-	-	+	29	19	48
9038	-	-	+	12902	-	-	+	29	17	46
14051	14048	14055	+	14621	14618	14622	+	20	26	46
2023	2022	2026	+	2055	2055	2056	-	17	28	45
9887	-	-	-	13910	-	-	+	21	21	42
12536	12531	12539	-	12684	-	-	-	0	41	41
13407	13406	13409	-	14189	14187	14189	+	25	15	40
9039	9037	9041	-	11254	11252	11255	+	20	19	39
4733	4728	4733	+	15117	-	-	+	12	26	38
13655	13655	13659	-	14761	-	-	+	6	32	38
13827	13826	13828	-	14719	-	-	-	22	14	36
9211	9207	9211	+	13387	13385	13387	+	24	11	35
748	748	749	+	708	707	708	-	16	16	32
2962	2960	2962	-	2941	-	-	+	31	1	32
63	59	66	+	88	85	88	-	29	2	31
149	148	154	+	13547	-	-	+	16	15	31
10642	10641	10643	-	15063	15062	15063	+	10	21	31
12101	12101	12105	-	14605	14605	14608	-	12	19	31
13041	13041	13042	-	15076	15076	15077	+	14	17	31
123	118	127	+	150	-	-	-	14	16	30
1142	1139	1143	-	1327	1327	1328	-	11	19	30
2807	2805	2809	+	15245	-	-	+	14	16	30
4908	4908	4911	+	9450	9450	9451	+	22	8	30
8394	-	-	+	13393	-	-	+	14	15	29
12331	12330	12331	-	14574	14574	14575	+	22	7	29
1764	1764	1766	-	1787	1785	1787	+	16	12	28
116	111	117	+	197	195	202	+	20	7	27
2117	2116	2120	-	2173	2172	2173	-	0	27	27
11199	11198	11200	-	14740	14740	14741	+	12	15	27
13668	13664	13672	-	14766	14766	14768	-	17	10	27
14428	14424	14428	-	14464	14464	14467	+	25	1	26
14435	14435	14438	-	14457	-	-	+	0	26	26
4979	4975	4982	-	5013	5010	5015	-	5	20	25
10071	-	-	-	15250	-	-	+	14	11	25
12431	12431	12432	-	14168	14167	14168	+	13	12	25
123	121	125	-	150	150	153	+	24	0	24
756	-	-	+	700	-	-	-	14	10	24
12045	12045	12046	-	12840	-	-	-	18	6	24
14377	-	-	-	14599	-	-	+	17	7	24
13940	13936	13943	-	15077	15077	15078	+	16	7	23
2055	2055	2056	+	2023	2022	2023	-	13	9	22
13866	13863	13867	-	14808	14808	14811	+	11	11	22

893	893	898	+	924 -	-		-	6	15	21
13446	13445	13446	-	14191 -	-		-	11	10	21
14011	14009	14014	-	14200 -	-		-	19	2	21
9435	9431	9435	+	14259 -	-		+	8	12	20
149	148	154	+	123 -	-		-	9	10	19
11373	11373	11377	+	11602 -	-		-	19	0	19
13683 -	-		-	14537 -	-		+	9	10	19
13882	13879	13883	+	13923	13922	13924	-	19	0	19
57	56	57	+	94	94	95	-	0	18	18
4398 -	-		+	11046 -	-		+	9	9	18
97	94	101	+	57 -	-		-	17	0	17
838	835	841	+	9136 -	-		+	15	2	17
2895	2893	2896	+	12945 -	-		+	7	10	17
5078	5074	5082	+	15083	15079	15087	-	15	2	17
7505	7502	7505	-	7594 -	-		-	0	17	17
10410	10407	10410	-	13041	13041	13043	+	0	17	17
11431	11430	11433	+	11525	11525	11529	+	14	3	17
12094 -	-		-	12567 -	-		+	9	8	17
13833	13831	13835	+	14707	14707	14709	+	13	4	17
13887	13887	13891	+	13918	13915	13918	-	15	2	17
14039	14038	14041	-	14604 -	-		-	7	10	17
1137	1135	1137	-	1327	1326	1327	-	7	9	16
1771	1771	1772	+	1779	1779	1780	-	4	12	16
2915	2915	2916	-	14818	14817	14818	+	6	10	16
12118	12117	12121	-	14622	14619	14624	-	6	10	16
14296 -	-		-	14355 -	-		+	4	12	16
6556	6554	6558	-	6633	6632	6635	-	13	2	15
10036 -	-		-	13350 -	-		-	1	14	15
10498	10496	10498	-	10517	10515	10517	+	9	6	15
12277	12274	12281	-	12869 -	-		-	8	7	15
14428	14426	14428	+	14464 -	-		-	1	14	15
14435 -	-		+	14457 -	-		-	15	0	15
14451	14448	14451	-	14549	14549	14551	+	7	8	15
1442	1439	1446	+	1460	1457	1460	-	6	8	14
1620	1618	1621	+	1703 -	-		+	0	14	14
3309	3307	3313	+	3332	3330	3335	+	8	6	14
4868	4868	4869	-	9714	9713	9714	+	2	12	14
10277	10274	10278	-	10473 -	-		-	3	11	14
157	155	162	+	173	173	174	-	2	11	13
924	922	928	+	893 -	-		-	6	7	13
2740	2737	2744	+	14775 -	-		+	7	6	13
3247	3244	3250	-	3273	3273	3274	+	11	2	13
3690	3688	3690	-	3934	3933	3934	-	5	8	13
5856	5853	5858	-	6048	6048	6050	-	0	13	13
6228	6224	6231	-	6458	6458	6459	-	8	5	13
8177	8173	8177	+	8422 -	-		-	13	0	13
9401	9399	9401	-	13940	13940	13941	+	6	7	13
9533	9529	9534	+	11075 -	-		+	10	3	13
9541	9540	9544	+	9753	9752	9753	+	4	9	13
11782 -	-		-	14343 -	-		+	3	10	13
13431 -	-		-	14176 -	-		+	7	6	13
13801	13800	13805	+	14696	14695	14696	+	9	4	13
14159	14157	14166	-	14194 -	-		-	0	13	13
150	148	154	-	123 -	-		+	12	0	12
700 -	-		-	756 -	-		+	6	6	12
1625	1625	1629	+	1708 -	-		+	12	0	12
1641	1641	1644	+	1696 -	-		-	12	0	12
1661	1658	1665	+	1681 -	-		+	6	6	12
4891	4889	4892	+	4927 -	-		+	6	6	12
11926	11923	11926	-	14450	14447	14450	-	4	8	12
13202	13199	13202	-	13937	13937	13940	+	3	9	12
14064	14062	14064	-	14199	14197	14199	-	0	12	12
465	465	471	+	491	488	495	+	4	7	11
1142	1139	1143	-	1337	1337	1341	-	5	6	11
1407	1407	1412	+	1450	1446	1450	-	9	2	11
1615	1611	1616	+	1706	1705	1706	+	1	10	11
1632	1632	1636	+	1705 -	-		-	0	11	11
1911	1911	1917	+	1973 -	-		-	3	8	11
2194	2190	2194	+	2430	2426	2430	+	7	4	11
3436	3435	3440	-	3483	3483	3484	+	9	2	11
3537	3534	3538	-	3728	3727	3731	-	5	6	11
4000	4000	4004	+	4064	4062	4066	+	5	6	11
4687	4683	4691	-	5066	5065	5071	-	7	4	11
6212	6211	6212	+	13436	13436	13437	+	7	4	11
9631 -	-		-	12936 -	-		+	9	2	11
11469 -	-		-	13972 -	-		+	6	5	11
14031	14027	14035	+	14758	14758	14763	-	6	5	11
14879	14878	14883	-	15113	15112	15115	-	4	7	11
123	118	127	+	197	197	201	+	6	4	10
484	480	485	+	501	497	502	+	4	6	10
989	987	990	+	14837 -	-		+	6	4	10
2028	2028	2031	+	12507	12507	12510	+	3	7	10
2202	2202	2203	+	2258	2257	2258	-	8	2	10
3229	3226	3232	+	3217	3217	3220	+	1	9	10
3229	3226	3232	+	4486	4486	4487	+	2	8	10
6307	6306	6311	+	6342	6340	6343	+	5	5	10
8402	8402	8404	-	13092 -	-		+	4	6	10
11565 -	-		-	14338 -	-		+	6	4	10

12067 -	-		-	14981 -	-		+	8	2	10
12529	12527	12530	-	13615	13615	13616	+	6	4	10
12625	12625	12628	-	13760	13759	13760	-	2	8	10
12660	12657	12661	-	14881	14880	14881	+	7	3	10
13772	13768	13772	+	13969	13969	13970	-	10	0	10
13819	13819	13823	+	14714	14712	14714	+	7	3	10
13872	13871	13876	-	14510 -	-		+	7	3	10
14024	14020	14025	+	14764	14764	14767	-	5	5	10
14139	14138	14142	-	14628 -	-		-	2	8	10
14464 -	-		+	14428 -	-		-	10	0	10
14770	14770	14771	-	15003 -	-		+	4	6	10
123	118	127	+	192	189	192	+	8	1	9
318	315	321	-	1878	1873	1878	-	1	8	9
1259	1255	1260	-	1412	1412	1415	-	9	0	9
1642	1639	1645	-	1672 -	-		-	0	9	9
2400	2396	2403	-	2535	2535	2538	-	5	4	9
2867	2866	2870	-	3021	3021	3024	-	5	4	9
3680	3677	3680	+	9302 -	-		+	5	4	9
4664	4659	4667	-	5068	5066	5071	-	5	4	9
4742	4738	4745	-	5420	5418	5422	-	3	6	9
5920	5917	5920	+	14950 -	-		+	0	9	9
7825	7825	7830	-	11157	11157	11158	-	0	9	9
7940	7937	7945	-	8047 -	-		-	0	9	9
8452	8451	8454	+	12070	12069	12070	+	6	3	9
8958	8958	8959	-	14808	14807	14808	+	4	5	9
10573	10570	10573	-	13944 -	-		+	2	7	9
10604	10604	10607	+	13786	13782	13786	+	6	3	9
11400	11398	11402	+	14189 -	-		+	6	3	9
12101	12101	12105	-	15079	15078	15079	+	6	3	9
12460	12457	12464	-	12726	12725	12730	-	7	2	9
12611	12607	12611	-	12644 -	-		-	0	9	9
13091	13089	13091	-	14437	14435	14437	-	4	5	9
13412	13412	13413	-	13781 -	-		+	4	5	9
13838	13837	13840	+	14707	14707	14708	+	7	2	9
13940	13936	13943	-	14966 -	-		-	5	4	9
14578	14576	14580	-	14932 -	-		+	3	6	9
108	105	110	+	195	192	196	+	5	3	8
157	155	160	-	173	173	174	+	8	0	8
2018	2014	2023	-	2056	2055	2056	+	6	2	8
3373	3372	3373	-	3418 -	-		+	3	5	8
4693	4693	4696	+	9763 -	-		+	1	7	8
5207	5207	5209	-	5651	5651	5652	-	3	5	8
6240	6236	6245	-	6388	6388	6391	-	4	4	8
6251	6246	6253	-	6458	6455	6460	-	6	2	8
6302	6299	6304	+	6350	6349	6350	+	5	3	8
6329	6327	6330	+	6352	6351	6356	+	3	5	8
6627 -	-		+	12320 -	-		+	5	3	8
7144	7140	7148	+	13372 -	-		+	2	6	8
9905	9902	9909	-	11549 -	-		-	3	5	8
10498	10496	10500	+	10515	10513	10517	-	1	7	8
10598 -	-		+	13776 -	-		+	2	6	8
12502	12502	12507	-	12706 -	-		-	0	8	8
12673	12672	12674	-	13023	13022	13024	-	3	5	8
12687 -	-		-	14266 -	-		+	4	4	8
13307	13307	13313	-	15180	15180	15181	-	4	4	8
13595 -	-		-	14750 -	-		+	2	6	8
13940	13936	13943	-	14042 -	-		-	7	1	8
123	118	127	+	895	894	897	+	7	0	7
312	310	314	-	567	567	571	-	2	5	7
392	391	396	+	439	437	439	+	4	3	7
404	403	409	+	528	528	531	+	2	5	7
417	414	419	+	504	501	504	+	1	6	7
475	474	477	+	491 -	-		+	4	3	7
491	490	495	+	516	515	516	+	3	4	7
1142	1139	1143	-	1873 -	-		-	4	3	7
1230	1226	1234	-	5977	5975	5977	+	5	2	7
1466	1462	1471	-	4408	4404	4408	+	1	6	7
1650	1648	1651	+	1680	1680	1681	+	7	0	7
1910	1910	1911	-	15363 -	-		-	1	6	7
1991	1987	1993	+	2577	2576	2577	+	5	2	7
2014	2010	2018	+	2111	2109	2111	+	2	5	7
2030	2028	2034	-	12510	12507	12513	-	6	1	7
2070	2070	2071	-	2203	2203	2204	-	0	7	7
2514	2513	2514	+	11910	11910	11911	+	6	1	7
2518	2517	2518	-	2547	2546	2547	-	2	5	7
2536	2534	2539	-	2730	2730	2732	-	5	2	7
3483	3480	3483	-	3436 -	-		+	7	0	7
3700	3700	3704	-	7334	7334	7338	+	2	5	7
4907	4906	4911	-	5264	5263	5264	-	3	4	7
5241	5241	5244	+	13587 -	-		+	5	2	7
6228	6224	6231	-	6465	6464	6467	-	3	4	7
7476	7474	7476	+	10566	10564	10566	+	4	3	7
7966 -	-		-	8065 -	-		-	0	7	7
8016 -	-		-	14581 -	-		+	3	4	7
8025	8021	8027	+	14600	14596	14600	+	6	1	7
9664	9660	9664	-	13053	13053	13054	+	4	3	7
9905 -	-		+	11549 -	-		+	7	0	7
10120	10116	10120	+	14044	14040	14044	+	6	1	7

10193 -	-		+	10421 -	-		+	4	3	7
10410	10407	10410	-	14818 -	-		+	0	7	7
11018	11018	11020	-	14969	14967	14969	+	6	1	7
11426 -	-		+	11520 -	-		+	1	6	7
11616	11613	11616	-	13675 -	-		+	3	4	7
12300	12298	12300	-	12473 -	-		-	4	3	7
12474	12474	12476	-	12684 -	-		-	6	1	7
12536	12531	12539	-	13628 -	-		-	5	2	7
12635	12632	12635	+	12953	12953	12956	-	7	0	7
12647	12647	12649	-	14870	14870	14873	-	7	0	7
12911	12908	12914	-	14480 -	-		+	3	4	7
13579	13579	13580	-	14964 -	-		+	2	5	7
14008	14008	14012	+	14317	14317	14318	-	7	0	7
14011	14009	14014	-	14750	14750	14753	-	4	3	7
14045	14043	14049	-	14137	14137	14140	+	3	4	7
14457 -	-		+	14435 -	-		-	0	7	7
14579	14579	14581	+	14635 -	-		-	4	3	7
299	296	302	+	437	434	437	+	3	3	6
312	310	314	-	1874	1873	1878	-	1	5	6
355	355	358	-	6685 -	-		+	1	5	6
387	387	389	+	435	433	439	+	4	2	6
581	578	585	+	594	594	597	+	1	5	6
708 -	-		-	748 -	-		+	6	0	6
924	922	928	+	10754 -	-		+	5	1	6
977	977	981	-	1167	1163	1167	-	3	3	6
1194	1191	1196	+	1246	1244	1247	+	2	4	6
1552	1550	1553	-	1579 -	-		-	0	6	6
1620	1618	1621	+	1710 -	-		+	6	0	6
1792	1792	1797	+	14817	14817	14818	+	2	4	6
1950	1946	1954	+	14247 -	-		+	5	1	6
1991	1987	1993	+	2317	2317	2318	+	3	3	6
1991	1987	1993	+	12803 -	-		+	4	2	6
2014	2010	2018	+	12493	12489	12493	+	2	4	6
2018	2014	2023	-	2583 -	-		-	3	3	6
2071	2067	2071	+	14881 -	-		+	3	3	6
2118	2115	2120	+	2149	2149	2150	+	2	4	6
2268	2268	2270	-	2245 -	-		+	6	0	6
2466	2464	2466	+	2443 -	-		-	1	5	6
2664	2661	2664	+	2719	2715	2720	-	5	1	6
2795 -	-		+	14651 -	-		+	2	4	6
2827	2827	2830	+	13837 -	-		+	4	2	6
2878	2874	2881	-	3018	3016	3018	-	2	4	6
2941	2938	2941	-	3149	3148	3151	-	3	3	6
2962	2960	2962	-	3010	3010	3012	+	6	0	6
3320	3319	3320	-	14932 -	-		+	3	3	6
3373	3373	3379	+	3419 -	-		+	2	4	6
4484	4482	4486	+	4475	4475	4476	+	4	2	6
4687	4683	4691	-	4907	4907	4908	-	3	3	6
4687	4683	4691	-	5448 -	-		-	3	3	6
4853	4852	4855	+	5000	4999	5001	+	2	4	6
4929	4926	4933	+	7655	7655	7656	+	3	3	6
5178	5176	5182	+	5265	5265	5266	+	4	2	6
5235 -	-		+	14895 -	-		+	3	3	6
5257	5256	5257	-	5285	5285	5286	+	4	2	6
5723	5720	5728	+	5787	5783	5791	+	3	3	6
5789	5787	5793	-	12189 -	-		-	3	3	6
6234 -	-		-	6510 -	-		-	3	3	6
6293	6289	6297	+	6364	6364	6365	+	4	2	6
6302	6299	6304	+	6314	6314	6317	+	4	2	6
6323	6320	6324	+	6342	6339	6343	+	2	4	6
7281	7277	7285	+	7309	7308	7313	+	5	1	6
7919	7919	7921	-	8171	8171	8172	-	3	3	6
7979	7979	7980	+	8193 -	-		+	3	3	6
8154	8151	8154	-	12921 -	-		+	5	1	6
8860 -	-		+	14489 -	-		+	4	2	6
9230 -	-		-	13872	13872	13874	+	1	5	6
9417	9417	9418	-	14992	14991	14992	+	4	2	6
9672 -	-		-	13237 -	-		+	5	1	6
9948	9945	9949	-	14112	14112	14113	-	3	3	6
9969	9969	9970	-	9995	9994	9995	+	2	4	6
10424 -	-		-	10542 -	-		-	3	3	6
10576	10574	10577	+	13960 -	-		+	2	4	6
10699	10698	10702	-	14870	14867	14870	+	1	5	6
11429 -	-		-	13741 -	-		+	3	3	6
11431	11430	11433	+	11552 -	-		+	6	0	6
11804	11801	11804	-	15005 -	-		+	1	5	6
12640	12640	12644	-	13365 -	-		+	3	3	6
12640	12640	12644	-	13366 -	-		-	6	0	6
12739 -	-		-	14370 -	-		+	0	6	6
12938	12938	12940	+	13329 -	-		-	6	0	6
13079	13079	13080	-	14425	14425	14426	+	3	3	6
13218	13214	13218	-	13942	13938	13942	-	3	3	6
13297	13297	13298	+	15007	15007	15008	+	3	3	6
13328 -	-		-	14988 -	-		-	3	3	6
13668	13664	13672	-	13798 -	-		-	0	6	6
13722	13720	13727	-	13872 -	-		-	6	0	6
13739	13739	13740	+	13939	13939	13940	+	3	3	6
13819	13816	13824	-	14702	14701	14703	-	1	5	6

13882	13879	13883	+	14980	14978	14981	+	4	2	6
13893	-	-	-	14169	-	-	-	3	3	6
13952	13950	13952	+	15088	-	-	+	1	5	6
14159	14157	14166	-	14817	14817	14818	-	0	6	6
14442	-	-	-	14513	-	-	-	0	6	6
15211	15211	15213	+	15235	-	-	+	6	0	6
108	105	110	+	607	-	-	+	3	2	5
116	111	117	+	192	191	192	+	4	1	5
132	127	132	-	143	-	-	+	0	5	5
227	223	230	+	260	260	262	-	1	4	5
291	288	293	+	313	310	316	+	2	3	5
368	366	370	+	6670	-	-	-	5	0	5
368	366	370	+	10556	-	-	+	0	5	5
569	567	573	-	1025	1022	1029	-	3	2	5
655	653	659	-	11857	-	-	+	5	0	5
689	685	692	+	12869	12868	12869	+	4	1	5
766	765	766	-	13777	13777	13778	+	3	2	5
779	776	779	+	13769	-	-	+	0	5	5
844	843	849	+	13690	-	-	+	2	3	5
1011	1007	1011	+	1023	1023	1025	+	2	3	5
1031	1028	1031	+	1053	1051	1053	+	3	2	5
1194	1191	1196	+	1222	1222	1226	+	2	3	5
1194	1191	1196	+	1275	1275	1277	+	2	3	5
1407	1407	1412	+	1440	1437	1440	+	0	5	5
1448	1448	1451	+	6702	6702	6705	+	5	0	5
1460	1457	1463	+	1442	-	-	-	4	1	5
1477	1474	1477	+	1477	1474	1477	+	5	0	5
1555	1552	1555	+	1600	-	-	+	2	3	5
1563	1562	1564	+	1809	-	-	+	0	5	5
1785	1785	1789	+	1766	1765	1766	-	4	1	5
1910	1910	1911	-	1973	-	-	+	3	2	5
1950	1946	1954	+	2002	2001	2002	+	5	0	5
1985	1982	1986	+	2154	2153	2154	+	3	2	5
2664	2661	2664	+	13578	-	-	+	2	3	5
2696	2696	2700	+	4320	4320	4324	+	0	5	5
2904	2901	2904	+	14828	14828	14831	-	2	3	5
2962	2959	2962	+	3011	3010	3011	-	3	2	5
2970	2968	2974	-	3063	-	-	-	5	0	5
3055	3054	3055	+	3077	-	-	-	0	5	5
3281	3277	3284	+	3821	3821	3822	+	3	2	5
3519	3515	3522	-	3749	3748	3749	-	3	2	5
3656	3654	3656	+	13525	-	-	+	3	2	5
3701	-	-	+	7337	-	-	-	3	2	5
4587	4583	4592	-	5640	-	-	-	2	3	5
4591	4591	4592	+	11887	-	-	+	4	1	5
4710	4706	4710	-	4800	-	-	-	5	0	5
4894	4893	4896	-	4919	4919	4920	+	4	1	5
5178	5176	5182	+	5277	-	-	+	4	1	5
5294	5289	5294	+	5251	5247	5252	-	3	2	5
5856	5853	5858	-	5873	5870	5873	+	2	3	5
5862	5860	5867	+	5864	5863	5864	-	4	1	5
6120	6119	6120	+	10774	-	-	+	2	3	5
6296	6293	6297	-	6464	6461	6464	-	0	5	5
6307	6306	6311	+	6351	-	-	+	3	2	5
6351	6350	6354	-	6508	6508	6509	-	3	2	5
6402	6398	6402	-	6452	6450	6454	+	2	3	5
6742	6740	6744	-	6950	-	-	-	3	2	5
6818	6818	6821	+	12386	-	-	+	1	4	5
6936	6934	6936	+	15368	15368	15369	-	5	0	5
7295	7295	7298	-	11199	11199	11202	+	1	4	5
7570	7567	7570	+	7852	-	-	+	5	0	5
7677	-	-	+	9361	-	-	-	5	0	5
7926	7926	7930	-	7986	-	-	-	1	4	5
8040	8036	8042	-	14167	14165	14167	+	3	2	5
8614	8614	8615	-	13270	13269	13270	+	4	1	5
9039	9037	9041	-	11229	-	-	-	2	3	5
9103	9101	9103	-	9130	-	-	+	4	1	5
9286	9283	9289	-	13269	13269	13272	+	1	4	5
9899	9895	9899	-	13915	13911	13915	-	2	3	5
9948	9945	9949	-	14118	-	-	-	0	5	5
10096	10096	10099	+	15292	-	-	+	1	4	5
10223	10223	10227	+	10399	10399	10403	+	3	2	5
10517	10515	10518	+	10496	10495	10498	-	2	3	5
10704	10704	10706	-	12445	-	-	+	5	0	5
10803	10803	10804	+	14247	14247	14248	+	1	4	5
10899	-	-	-	13660	-	-	+	0	5	5
11067	-	-	-	13780	-	-	+	3	2	5
11308	-	-	+	11599	-	-	-	5	0	5
11400	11396	11401	-	14189	-	-	-	2	3	5
11598	11597	11600	-	13483	13483	13484	+	2	3	5
11822	11821	11826	-	14447	14446	14447	+	3	2	5
12045	12045	12046	-	12855	-	-	-	4	1	5
12118	12117	12121	-	14630	14627	14630	-	4	1	5
12201	-	-	-	12980	-	-	-	0	5	5
12496	-	-	-	12700	-	-	-	5	0	5
12536	12531	12539	-	13511	-	-	-	2	3	5
12536	12531	12539	-	13514	-	-	+	1	4	5
12591	12589	12594	-	12625	-	-	-	0	5	5

12640	12640	12644	-	14867	14867	14869	-	2	3	5
12647	12647	12649	-	13371 -	-		-	0	5	5
12790 -	-		-	13791 -	-		+	1	4	5
12911	12908	12914	-	13042 -	-		-	3	2	5
12945	12945	12949	-	13642 -	-		-	5	0	5
13024 -	-		-	14463 -	-		+	1	4	5
13801	13800	13805	+	14379 -	-		+	0	5	5
13930	13927	13933	-	14036	14036	14037	-	5	0	5
13930	13927	13933	-	14343 -	-		+	3	2	5
14008	14008	14012	+	14160	14160	14162	+	2	3	5
14045	14043	14049	-	14368 -	-		+	1	4	5
14316	14313	14320	-	14291 -	-		+	5	0	5
14588	14585	14590	+	14634 -	-		-	3	2	5
14596	14596	14600	+	14619	14619	14623	-	5	0	5
14933	14932	14933	-	15078	15077	15078	-	1	4	5
15143	15139	15143	+	15293 -	-		+	2	3	5
15189	15187	15190	+	15204 -	-		+	5	0	5
88	87	92	+	63 -	-		-	0	4	4
108	105	110	+	132 -	-		+	2	2	4
108	105	110	+	1229 -	-		+	2	2	4
117	113	119	-	264	262	264	-	1	3	4
123	121	125	-	1681	1681	1682	-	2	2	4
123	118	127	+	434	434	437	+	3	1	4
123	118	127	+	515 -	-		+	2	2	4
123	118	127	+	1523 -	-		+	2	2	4
131	128	134	+	143	141	143	-	3	1	4
131	128	134	+	191 -	-		+	2	2	4
131	128	134	+	243 -	-		+	2	2	4
132	127	132	-	367	363	368	-	2	2	4
150	148	154	-	307 -	-		-	2	2	4
175	172	179	+	13297 -	-		+	2	2	4
223	220	224	-	641 -	-		-	2	2	4
291	288	293	+	325 -	-		+	2	2	4
299	296	302	+	311	311	314	+	2	2	4
312	310	314	-	553	553	555	-	2	2	4
312	309	315	+	435 -	-		+	2	2	4
363	363	365	+	490	488	490	+	1	3	4
372	370	374	-	571	567	571	-	4	0	4
381	380	382	+	449	449	450	-	2	2	4
381	380	382	+	465	465	466	+	2	2	4
404	403	409	+	522	519	522	+	1	3	4
426	423	429	-	3198	3198	3199	-	2	2	4
426	423	430	+	501	501	502	+	2	2	4
457	454	462	+	522 -	-		+	2	2	4
465	465	471	+	501	498	501	+	2	2	4
481	478	484	-	967 -	-		-	2	2	4
488	487	491	-	1681 -	-		-	2	2	4
521	521	522	+	576 -	-		+	2	2	4
580	576	582	-	1563 -	-		-	2	2	4
581	578	585	+	629 -	-		+	2	2	4
620	618	624	-	1312 -	-		-	2	2	4
743	743	745	-	713 -	-		+	4	0	4
924	922	928	+	981	978	981	+	2	2	4
963	963	968	+	1460 -	-		+	2	2	4
1011	1007	1011	+	1021 -	-		-	0	4	4
1011	1007	1011	+	1079 -	-		+	1	3	4
1137	1135	1137	-	1332	1332	1335	-	2	2	4
1224	1223	1227	+	1286	1286	1288	+	3	1	4
1244	1243	1247	-	1436	1432	1437	-	2	2	4
1294	1291	1294	-	1468 -	-		-	0	4	4
1395	1393	1397	-	1565 -	-		-	2	2	4
1395	1395	1398	+	1461	1459	1461	-	2	2	4
1440	1440	1444	-	1653 -	-		-	2	2	4
1442	1439	1446	+	1454	1454	1455	+	2	2	4
1442	1439	1446	+	6701 -	-		+	4	0	4
1446	1446	1448	-	1521 -	-		-	4	0	4
1460	1457	1463	+	1478	1475	1480	+	1	3	4
1568	1568	1572	+	1580	1580	1583	+	2	2	4
1580	1577	1581	+	1681 -	-		+	2	2	4
1589	1585	1593	-	1815 -	-		-	2	2	4
1613	1611	1615	-	14346 -	-		-	2	2	4
1650	1648	1651	+	1722	1722	1723	+	2	2	4
1661	1658	1665	+	1707 -	-		+	0	4	4
1792	1792	1797	+	1808	1808	1810	+	1	3	4
1795	1795	1799	-	3348 -	-		-	2	2	4
1823 -	-		+	2331 -	-		+	0	4	4
1924	1920	1927	-	2218	2217	2220	-	2	2	4
1969	1969	1973	-	2082 -	-		-	1	3	4
1979	1975	1980	+	2586 -	-		+	2	2	4
1990	1988	1994	-	2153	2153	2154	-	2	2	4
1991	1987	1993	+	2236 -	-		+	2	2	4
2006	2005	2008	+	12482	12481	12482	+	3	1	4
2010	2010	2011	-	2216 -	-		-	2	2	4
2085	2081	2085	+	2112	2112	2113	-	2	2	4
2085	2081	2085	+	2220	2220	2221	+	2	2	4
2118	2115	2120	+	2515 -	-		+	2	2	4
2212	2209	2215	+	2188	2188	2191	-	3	1	4
2245	2243	2245	-	2268 -	-		+	4	0	4

2395 -	-		+	13333 -	-		+	3	1	4
2434	2430	2435	-	2583 -	-		-	2	2	4
2482 -	-		+	12076 -	-		+	2	2	4
2491	2490	2495	-	2531	2527	2531	-	2	2	4
2540 -	-		+	14024 -	-		+	2	2	4
2551	2549	2554	+	11539	11537	11540	+	2	2	4
2639	2635	2643	+	2660	2660	2661	+	2	2	4
2652	2652	2656	-	2723	2723	2724	-	2	2	4
2746	2745	2746	+	14962 -	-		+	2	2	4
2807	2805	2809	+	2832 -	-		-	2	2	4
2857	2854	2857	+	2931	2931	2932	+	2	2	4
2931	2927	2935	-	3149	3148	3150	-	2	2	4
2931	2927	2935	-	3207	3204	3207	-	2	2	4
3065 -	-		+	3098 -	-		-	1	3	4
3273	3269	3275	-	3247	3247	3251	+	4	0	4
3327 -	-		-	3628 -	-		-	2	2	4
3341	3340	3342	-	4388	4387	4388	-	1	3	4
3390	3389	3393	+	3409	3407	3409	+	2	2	4
3437	3437	3441	+	3452	3449	3452	+	1	3	4
3537	3534	3538	-	3723 -	-		-	2	2	4
3557	3556	3557	+	3600	3597	3600	+	1	3	4
3604	3604	3608	+	3677	3673	3677	+	2	2	4
3628	3628	3631	+	3682	3678	3682	+	1	3	4
3645	3642	3646	-	4146	4145	4146	-	3	1	4
3673	3672	3675	+	3688	3688	3690	+	2	2	4
3754	3754	3757	+	14416 -	-		+	3	1	4
3773	3770	3774	+	13395 -	-		+	4	0	4
3775	3775	3780	-	4015 -	-		-	2	2	4
3775	3775	3780	-	4039	4039	4040	-	2	2	4
3790	3790	3793	-	14615 -	-		+	4	0	4
3838	3836	3838	+	13081 -	-		+	3	1	4
3864	3861	3864	-	4146	4144	4146	-	3	1	4
3935	3932	3937	-	4542	4542	4543	-	2	2	4
4132	4132	4135	-	4406 -	-		-	2	2	4
4484	4482	4486	+	12972 -	-		+	3	1	4
4513	4513	4514	+	5899	5899	5900	+	4	0	4
4561	4557	4565	-	5516	5516	5517	-	2	2	4
4585	4580	4588	+	4637	4633	4637	+	3	1	4
4601	4599	4601	-	4710 -	-		-	2	2	4
4654	4650	4658	-	4806 -	-		-	2	2	4
4687	4683	4691	-	5176	5176	5177	-	2	2	4
4705	4701	4705	-	5488 -	-		-	2	2	4
4744	4742	4747	+	5435	5433	5435	+	4	0	4
4755	4755	4756	+	7569	7569	7570	+	2	2	4
4765	4765	4768	+	5002	5002	5003	+	2	2	4
4767	4766	4770	-	4926	4926	4927	-	2	2	4
4805	4801	4806	-	5058	5058	5059	-	2	2	4
5030	5026	5034	-	5370 -	-		-	2	2	4
5055	5053	5055	-	6114 -	-		-	0	4	4
5069	5066	5069	+	15093	15093	15096	-	2	2	4
5277	5273	5280	-	5538	5535	5539	-	2	2	4
5359	5358	5363	-	5586 -	-		-	2	2	4
5399	5399	5400	-	5604	5604	5605	-	2	2	4
5414	5413	5417	+	5446	5444	5446	+	1	3	4
5449	5449	5452	-	5605	5602	5605	-	1	3	4
5473	5472	5476	-	5651 -	-		-	2	2	4
5525	5523	5525	-	7594 -	-		+	0	4	4
5615	5611	5619	-	5855	5853	5855	-	2	2	4
5615	5611	5619	-	6041 -	-		-	2	2	4
5667 -	-		+	8756 -	-		+	1	3	4
5726	5724	5726	-	6041	6039	6041	-	2	2	4
6204	6201	6204	-	6215	6212	6215	-	2	2	4
6228	6224	6231	-	6389 -	-		-	2	2	4
6229	6228	6229	+	10369 -	-		+	1	3	4
6240	6236	6245	-	6400	6396	6400	-	2	2	4
6240	6236	6245	-	6470 -	-		-	2	2	4
6240	6236	6245	-	6509 -	-		-	2	2	4
6323	6320	6324	+	6363	6362	6367	+	1	3	4
6328	6324	6328	-	6497 -	-		-	2	2	4
6473 -	-		-	6519 -	-		+	3	1	4
6580	6576	6580	-	6738	6738	6739	-	2	2	4
6606	6603	6608	-	6917 -	-		-	2	2	4
6644	6642	6646	+	7608 -	-		+	2	2	4
6651	6648	6654	-	6681 -	-		-	0	4	4
6818	6818	6821	+	6888	6888	6889	+	2	2	4
7065	7061	7069	-	7205 -	-		-	2	2	4
7065	7061	7066	+	7084	7082	7084	+	2	2	4
7619	7619	7623	+	7636 -	-		+	2	2	4
7637	7636	7637	-	8341 -	-		-	2	2	4
7813 -	-		-	11334 -	-		+	0	4	4
7861	7861	7863	-	7969 -	-		-	2	2	4
8188	8184	8189	-	8397	8397	8398	-	2	2	4
8263	8263	8264	+	8304	8304	8305	-	3	1	4
8588 -	-		+	8989 -	-		+	2	2	4
8761 -	-		+	12892 -	-		+	2	2	4
8873 -	-		+	13230 -	-		+	2	2	4
8946	8945	8946	-	14358	14358	14359	+	1	3	4
9800	9798	9801	+	11138	11138	11139	+	2	2	4

9874	-	-	+	13923	-	-	-	2	2	4
9921	-	-	-	11085	-	-	-	4	0	4
9942	9942	9945	+	14109	14108	14109	+	1	3	4
9948	9945	9949	-	10016	-	-	+	2	2	4
9976	9976	9978	-	9988	-	-	+	3	1	4
10082	-	-	+	13587	-	-	+	1	3	4
10097	-	-	-	10211	-	-	-	4	0	4
10374	10374	10377	-	13243	13240	13243	+	3	1	4
10543	10540	10543	-	14619	-	-	+	2	2	4
10549	-	-	+	10564	-	-	+	2	2	4
10576	10574	10577	+	13716	-	-	+	1	3	4
10660	10657	10660	+	11429	-	-	+	3	1	4
10678	-	-	-	10939	-	-	-	2	2	4
10694	10692	10697	-	13735	-	-	-	2	2	4
10704	10704	10706	+	11640	-	-	+	2	2	4
10773	10771	10773	+	12664	12664	12666	-	3	1	4
10870	-	-	-	12603	-	-	+	2	2	4
11075	11075	11079	+	13786	-	-	+	0	4	4
11120	11120	11122	+	11161	-	-	+	2	2	4
11174	11173	11174	-	13996	-	-	+	3	1	4
11336	11336	11339	+	11851	11848	11851	-	2	2	4
11361	-	-	+	13692	-	-	-	4	0	4
11395	11392	11395	+	11729	-	-	-	4	0	4
11405	11404	11409	-	14196	14194	14198	-	1	3	4
11576	11573	11576	-	11667	-	-	-	4	0	4
11634	-	-	-	11815	-	-	-	2	2	4
11926	11923	11926	-	14307	-	-	+	2	2	4
12006	12006	12008	-	12967	12965	12967	+	3	1	4
12123	12122	12123	-	12342	12342	12344	-	0	4	4
12146	12142	12149	-	14621	-	-	-	0	4	4
12209	12209	12210	+	12182	12179	12182	-	3	1	4
12371	-	-	+	12851	-	-	+	2	2	4
12447	12446	12450	+	14262	14262	14263	+	2	2	4
12488	-	-	-	12570	-	-	-	2	2	4
12514	12514	12517	-	12640	-	-	-	0	4	4
12529	12527	12530	-	12677	-	-	-	4	0	4
12536	12531	12539	-	13622	13621	13622	-	3	1	4
12542	-	-	-	12603	-	-	+	3	1	4
12566	12566	12570	-	14242	14240	14242	-	2	2	4
12609	-	-	+	14439	-	-	+	2	2	4
12716	12715	12721	-	13229	-	-	+	2	2	4
12881	-	-	-	12948	-	-	-	0	4	4
12911	12908	12914	-	13094	-	-	+	0	4	4
13307	13307	13313	-	13879	-	-	-	4	0	4
13453	13453	13457	-	14198	14198	14199	-	3	1	4
13501	-	-	+	13531	-	-	-	2	2	4
13651	-	-	+	14029	-	-	+	0	4	4
13722	13720	13727	-	13758	-	-	+	4	0	4
13722	13720	13727	-	13860	-	-	-	4	0	4
13722	13720	13727	-	14214	-	-	+	4	0	4
13819	13816	13824	-	14685	14682	14685	+	2	2	4
13819	13816	13824	-	14707	-	-	-	2	2	4
13838	13837	13840	+	13878	-	-	+	2	2	4
13940	13936	13943	-	14095	14091	14095	-	4	0	4
14039	14038	14041	-	14371	-	-	-	2	2	4
14082	14078	14082	+	14107	14104	14107	+	3	1	4
14091	14089	14091	-	14255	-	-	-	4	0	4
14291	14287	14292	-	14316	-	-	+	3	1	4
14303	14299	14304	+	14384	14384	14385	+	1	3	4
14346	14346	14349	-	14404	14404	14405	+	2	2	4
14504	14504	14507	-	14551	-	-	+	4	0	4
15078	-	-	-	15157	-	-	-	0	4	4
15241	15241	15245	-	15275	15271	15275	+	2	2	4
15251	-	-	+	15269	-	-	+	0	4	4
36	-	-	+	6362	-	-	+	3	0	3
75	74	80	-	291	291	292	-	2	1	3
88	87	92	+	820	-	-	+	3	0	3
97	94	101	+	1195	1195	1199	+	2	1	3
108	105	110	+	201	-	-	+	3	0	3
108	105	110	+	435	431	435	+	2	1	3
116	111	117	+	132	132	133	+	3	0	3
116	111	117	+	185	182	185	+	2	1	3
116	111	117	+	1270	1270	1275	+	3	0	3
123	118	127	+	131	131	134	+	3	0	3
123	118	127	+	153	-	-	+	2	1	3
123	118	127	+	177	177	179	+	3	0	3
123	118	127	+	186	186	187	+	2	1	3
123	118	127	+	203	-	-	+	3	0	3
123	118	127	+	1149	1149	1151	+	2	1	3
132	127	132	-	267	266	267	-	1	2	3
141	137	143	-	307	306	307	-	2	1	3
142	138	144	+	200	200	202	+	2	1	3
149	148	154	+	1012	-	-	+	3	0	3
173	169	177	-	157	-	-	+	3	0	3
227	223	230	+	270	-	-	+	0	3	3
231	228	232	-	246	243	246	+	1	2	3
231	228	232	-	257	257	260	+	2	1	3
263	259	268	-	368	366	368	-	2	1	3

263	259	268	-	569	567	569	-	2	1	3
291	288	293	+	301	-	-	+	1	2	3
307	304	308	-	582	582	584	-	1	2	3
318	315	321	-	1868	1867	1868	-	1	2	3
365	362	368	-	573	569	573	-	2	1	3
392	391	396	+	415	415	419	+	3	0	3
392	391	396	+	423	423	427	+	1	2	3
404	403	409	+	502	-	-	+	1	2	3
408	408	410	-	626	625	626	-	0	3	3
449	-	-	-	616	614	616	-	0	3	3
457	454	462	+	473	473	474	+	2	1	3
457	454	462	+	501	-	-	+	1	2	3
475	474	477	+	502	501	502	+	2	1	3
481	478	484	-	929	-	-	-	3	0	3
491	490	495	+	502	502	504	+	0	3	3
541	538	543	+	620	-	-	+	3	0	3
548	548	552	+	621	617	621	+	2	1	3
572	568	573	+	585	585	588	+	0	3	3
634	634	635	-	665	-	-	+	3	0	3
642	642	646	+	854	851	855	+	2	1	3
665	661	665	+	634	634	636	-	1	2	3
684	681	690	-	894	894	897	-	1	2	3
705	705	707	+	749	749	751	-	2	1	3
750	-	-	-	774	-	-	+	0	3	3
770	767	771	+	802	802	805	+	1	2	3
779	776	779	+	803	-	-	+	1	2	3
798	795	802	-	6362	-	-	-	0	3	3
893	893	898	+	968	-	-	+	3	0	3
928	927	933	-	1149	-	-	-	3	0	3
928	927	933	-	1194	-	-	-	3	0	3
961	957	963	-	1015	1011	1015	-	1	2	3
970	968	970	-	1137	-	-	-	1	2	3
1018	1018	1019	+	12454	-	-	+	2	1	3
1062	1062	1064	-	13674	-	-	+	3	0	3
1075	1073	1075	+	1084	1084	1085	-	1	2	3
1088	1088	1091	+	1222	-	-	+	0	3	3
1116	1114	1120	+	1148	1144	1148	+	2	1	3
1194	1191	1196	+	1287	-	-	+	1	2	3
1200	1199	1204	+	1243	1243	1246	+	2	1	3
1200	1199	1204	+	1284	-	-	+	2	1	3
1206	1206	1210	+	1286	-	-	+	3	0	3
1224	1223	1225	-	1437	-	-	-	3	0	3
1230	1226	1234	-	1550	1550	1553	-	1	2	3
1230	1226	1234	-	1562	-	-	-	2	1	3
1388	1386	1388	+	1458	-	-	+	0	3	3
1407	1407	1412	+	1428	-	-	+	2	1	3
1424	1421	1425	+	1439	1439	1440	+	1	2	3
1436	1434	1436	+	1481	-	-	+	1	2	3
1460	1457	1463	+	1397	-	-	-	1	2	3
1460	1457	1463	+	1460	-	-	+	3	0	3
1466	1462	1471	-	1600	-	-	-	1	2	3
1538	1537	1539	+	1595	1595	1599	+	1	2	3
1615	1611	1616	+	14107	-	-	-	0	3	3
1814	1814	1816	+	9583	-	-	+	0	3	3
1847	1843	1847	+	12275	-	-	+	2	1	3
1894	1892	1894	+	14896	14895	14896	+	2	1	3
1911	1911	1917	+	2000	-	-	+	1	2	3
1950	1946	1954	+	1990	-	-	+	3	0	3
1985	1982	1986	-	2180	2178	2180	-	2	1	3
1985	1982	1986	+	2013	-	-	+	3	0	3
1985	1982	1986	+	2115	-	-	+	0	3	3
1985	1982	1986	+	2584	-	-	+	2	1	3
1990	1988	1994	-	2148	2148	2149	-	1	2	3
1990	1988	1994	-	2309	-	-	-	1	2	3
2023	2022	2026	+	12503	12503	12505	+	2	1	3
2054	2054	2059	-	2023	-	-	+	3	0	3
2095	2090	2099	-	2177	-	-	+	3	0	3
2095	2090	2099	-	2971	-	-	-	3	0	3
2106	2106	2108	-	2170	-	-	+	0	3	3
2246	2245	2246	+	14709	-	-	+	1	2	3
2339	2339	2343	-	2639	-	-	-	1	2	3
2340	2339	2343	+	2401	-	-	+	1	2	3
2400	2396	2403	-	2576	-	-	-	3	0	3
2443	2439	2445	+	2466	-	-	-	1	2	3
2451	-	-	+	13460	-	-	+	1	2	3
2466	-	-	-	2443	-	-	+	3	0	3
2498	2498	2499	+	2598	2596	2598	+	3	0	3
2751	2751	2757	-	2990	-	-	-	0	3	3
2846	2844	2850	+	2867	-	-	+	0	3	3
2931	2927	2935	-	3188	-	-	-	3	0	3
3060	-	-	+	3072	-	-	-	3	0	3
3111	3111	3112	+	3145	-	-	+	3	0	3
3216	-	-	+	12593	-	-	+	2	1	3
3268	3264	3275	+	11739	-	-	+	2	1	3
3309	3307	3313	+	3767	3767	3770	+	3	0	3
3325	3320	3325	+	3341	3340	3341	+	0	3	3
3334	3329	3338	-	3764	3764	3765	-	1	2	3
3477	3477	3481	+	15068	15068	15069	+	1	2	3

3489	3485	3493	+	9953 -	-		+	3	0	3
3489	3485	3493	+	14254 -	-		+	1	2	3
3519	3515	3522	-	3727 -	-		-	1	2	3
3577	3572	3580	-	4295	4294	4295	-	1	2	3
3628	3626	3632	-	4190 -	-		-	3	0	3
3650	3650	3653	-	3825	3821	3825	-	1	2	3
3678	3674	3682	-	3712 -	-		-	1	2	3
3720	3716	3722	+	3856 -	-		+	2	1	3
3720	3716	3722	+	7321	7317	7322	-	1	2	3
3767	3765	3768	+	13389 -	-		+	0	3	3
3773	3770	3774	+	11828	11828	11829	+	2	1	3
3773	3770	3774	+	15210 -	-		-	0	3	3
3838	3836	3838	+	3864 -	-		+	3	0	3
3838	3836	3838	+	4074 -	-		-	3	0	3
3885	3885	3886	+	10210 -	-		+	3	0	3
4000	4000	4004	+	4055	4055	4058	+	2	1	3
4020	4017	4023	+	4036	4035	4036	+	2	1	3
4035	4035	4036	+	14565 -	-		+	1	2	3
4055	4051	4059	-	4287	4287	4291	-	1	2	3
4086	4085	4087	-	4277	4275	4277	-	1	2	3
4420 -	-		+	4422 -	-		-	3	0	3
4464	4464	4466	+	5933 -	-		+	0	3	3
4585	4580	4588	+	5651 -	-		+	0	3	3
4585	4580	4588	+	14391 -	-		+	3	0	3
4635	4631	4635	+	13995 -	-		+	1	2	3
4664	4659	4667	-	5092	5092	5093	-	1	2	3
4665	4665	4670	+	4677 -	-		+	0	3	3
4682	4678	4682	-	4933 -	-		-	0	3	3
4687	4683	4691	-	5060 -	-		-	3	0	3
4751	4746	4753	-	5325 -	-		-	0	3	3
4891	4889	4892	+	4909 -	-		+	3	0	3
4979	4975	4982	-	5316 -	-		-	3	0	3
4979	4976	4979	+	10817 -	-		+	1	2	3
4999	4997	5001	-	5178	5177	5178	-	2	1	3
5039 -	-		-	5207 -	-		-	3	0	3
5046 -	-		-	5205 -	-		-	3	0	3
5072 -	-		-	5354 -	-		-	3	0	3
5096	5092	5096	+	13334 -	-		+	2	1	3
5102	5102	5105	-	11815 -	-		+	2	1	3
5102	5102	5105	-	12056 -	-		-	0	3	3
5128 -	-		+	5155	5153	5155	+	2	1	3
5174	5174	5180	-	5586	5586	5587	-	1	2	3
5174	5174	5180	-	10847 -	-		+	1	2	3
5262	5260	5266	+	12863 -	-		+	1	2	3
5862	5860	5867	+	5887	5887	5888	+	0	3	3
5876 -	-		-	13752 -	-		-	3	0	3
5883	5883	5887	-	13759 -	-		-	0	3	3
5920	5917	5920	+	13389 -	-		+	0	3	3
6090 -	-		+	12078 -	-		+	3	0	3
6213	6209	6216	-	11117	11113	11117	+	0	3	3
6240	6236	6245	-	6458	6457	6458	-	2	1	3
6285	6283	6285	+	6363	6363	6367	+	2	1	3
6293	6289	6297	+	6355 -	-		+	3	0	3
6293	6289	6297	+	7882	7882	7884	+	2	1	3
6302	6299	6304	+	6360	6357	6360	+	2	1	3
6315	6313	6318	+	6344	6340	6344	+	1	2	3
6316	6316	6317	-	6483	6482	6483	-	1	2	3
6323	6320	6324	+	6351 -	-		+	1	2	3
6340	6337	6344	+	6584	6584	6586	+	1	2	3
6396	6396	6398	+	6457 -	-		+	0	3	3
6519	6517	6524	-	6473 -	-		+	3	0	3
6533	6533	6534	+	6524 -	-		+	0	3	3
6586	6584	6588	-	6945	6945	6948	-	2	1	3
6621	6617	6623	-	6790 -	-		-	3	0	3
6695	6695	6699	-	6719	6717	6719	+	2	1	3
6750	6749	6750	-	7805 -	-		-	0	3	3
6800	6800	6801	-	6887 -	-		-	0	3	3
7000	6996	7000	-	14486 -	-		+	2	1	3
7016 -	-		-	7385 -	-		-	0	3	3
7075	7071	7075	-	7132 -	-		-	0	3	3
7101	7100	7102	-	7183	7181	7183	-	1	2	3
7177	7173	7181	+	13837 -	-		+	1	2	3
7203	7200	7207	+	7519	7518	7519	+	1	2	3
7207	7205	7210	-	7555 -	-		-	3	0	3
7245	7245	7247	-	7507 -	-		-	0	3	3
7385	7381	7386	-	7548	7548	7549	-	2	1	3
7416 -	-		-	8403 -	-		-	0	3	3
7447	7447	7450	+	7501 -	-		+	1	2	3
7505	7502	7505	-	7808 -	-		+	0	3	3
7587	7583	7591	+	7649	7648	7649	+	2	1	3
7649 -	-		+	7849 -	-		-	3	0	3
7766	7765	7772	+	7811 -	-		+	0	3	3
7926	7926	7930	-	8178 -	-		-	1	2	3
7940	7937	7945	-	7991 -	-		-	0	3	3
8036	8035	8036	+	8070 -	-		+	3	0	3
8040	8036	8042	-	14168 -	-		-	2	1	3
8059 -	-		-	13734 -	-		+	1	2	3
8075	8075	8080	+	13754	13754	13755	+	2	1	3

8083	8083	8087	-	8245	-	-	-	0	3	3
8140	-	-	-	8236	-	-	-	3	0	3
8147	8147	8149	-	8243	-	-	-	0	3	3
8210	8209	8214	-	8414	8411	8414	-	2	1	3
8371	-	-	+	11563	-	-	+	1	2	3
8472	8472	8473	+	14436	-	-	+	3	0	3
8547	8546	8547	+	13125	13124	13125	+	1	2	3
8624	8624	8627	+	8952	-	-	-	3	0	3
8748	8748	8751	-	8989	-	-	-	3	0	3
8774	8772	8774	-	14417	14417	14419	+	1	2	3
9388	9388	9391	-	12106	-	-	-	3	0	3
9446	-	-	-	9916	-	-	-	0	3	3
9541	9540	9544	+	9723	-	-	-	0	3	3
9622	-	-	-	12874	-	-	+	3	0	3
10061	10060	10065	-	10086	-	-	-	0	3	3
10511	-	-	-	10502	-	-	+	0	3	3
10543	10540	10543	-	12868	-	-	+	2	1	3
10554	10552	10558	-	11911	-	-	+	3	0	3
10581	10581	10584	-	13706	-	-	-	3	0	3
10647	-	-	-	10933	-	-	-	0	3	3
10793	10789	10794	+	12296	12296	12297	+	2	1	3
11061	-	-	-	11005	-	-	+	3	0	3
11394	11390	11394	-	14186	-	-	+	0	3	3
11400	11398	11402	+	11483	-	-	-	3	0	3
11405	11405	11406	+	12510	-	-	+	3	0	3
11421	11417	11422	+	11509	-	-	+	3	0	3
11421	11417	11422	+	13883	-	-	+	3	0	3
11616	11613	11616	-	14246	14246	14247	+	1	2	3
11688	-	-	-	14035	-	-	+	1	2	3
11741	-	-	+	11804	-	-	+	0	3	3
11758	-	-	+	11774	-	-	+	3	0	3
11834	11834	11835	-	12282	-	-	-	3	0	3
11834	11834	11835	-	12310	12310	12311	+	1	2	3
11842	11841	11842	-	14461	-	-	-	1	2	3
12088	12087	12088	-	14589	-	-	-	3	0	3
12101	12101	12105	-	14586	-	-	-	2	1	3
12112	12108	12112	+	14616	14616	14618	+	1	2	3
12187	-	-	+	12229	-	-	+	0	3	3
12218	12215	12219	-	14194	-	-	-	3	0	3
12268	12266	12269	+	14422	14420	14423	+	2	1	3
12277	12274	12281	-	14440	14440	14444	-	2	1	3
12336	-	-	-	13944	-	-	+	2	1	3
12431	12431	12432	-	14152	-	-	-	1	2	3
12519	12519	12520	-	12645	-	-	-	0	3	3
12566	12566	12570	-	13332	-	-	+	1	2	3
12603	12600	12603	-	12542	-	-	+	3	0	3
12618	-	-	-	12687	-	-	-	0	3	3
12618	12614	12618	+	12995	-	-	-	3	0	3
12673	12672	12674	-	14477	-	-	+	2	1	3
12759	12759	12760	-	13972	-	-	+	0	3	3
12945	12945	12949	-	13644	-	-	+	1	2	3
13291	13291	13294	-	13509	13508	13509	-	0	3	3
13462	13462	13463	-	14985	14984	14985	+	3	0	3
13495	-	-	-	14136	-	-	+	2	1	3
13532	-	-	+	13679	-	-	+	0	3	3
13630	13627	13630	-	13798	-	-	-	0	3	3
13722	13720	13727	-	13888	-	-	-	2	1	3
13730	13730	13733	-	14081	14080	14081	+	2	1	3
13759	13755	13763	-	14544	-	-	+	0	3	3
13801	13799	13805	-	14704	14700	14704	+	2	1	3
13851	13848	13851	-	14715	-	-	-	3	0	3
13855	13855	13856	+	14979	-	-	-	3	0	3
13861	13858	13861	-	13995	-	-	-	3	0	3
13866	13863	13867	-	14000	-	-	-	0	3	3
13866	13863	13867	-	14019	-	-	-	0	3	3
14001	13999	14001	-	14016	14016	14018	+	1	2	3
14029	14027	14030	-	14797	14797	14799	-	2	1	3
14082	14078	14082	+	14293	-	-	+	0	3	3
14083	-	-	-	14341	-	-	-	3	0	3
14139	14138	14142	-	14801	-	-	+	1	2	3
14159	14157	14166	-	14651	-	-	+	0	3	3
14159	14157	14166	-	15005	-	-	+	2	1	3
14291	14287	14292	-	14360	14359	14360	+	3	0	3
14346	14346	14349	-	14620	-	-	-	0	3	3
14363	14363	14365	+	14580	-	-	+	0	3	3
14365	14361	14365	-	14582	14580	14582	-	1	2	3
14540	-	-	-	14640	-	-	-	3	0	3
14556	-	-	-	14641	-	-	-	3	0	3
14791	14790	14791	-	14921	14918	14921	-	1	2	3
14879	14878	14883	-	15122	15118	15122	-	1	2	3
14977	14977	14981	+	15265	-	-	+	0	3	3
15158	15157	15159	+	15179	15176	15179	+	3	0	3
15189	15187	15190	+	15235	15231	15236	+	2	1	3
15223	15223	15226	+	15284	15283	15284	+	1	2	3
15223	15223	15226	+	15353	-	-	+	0	3	3
59	-	-	-	515	-	-	+	0	2	2
75	74	80	-	372	-	-	-	0	2	2
88	87	92	+	87	87	88	-	1	1	2

88	87	92	+	181	-	-		+	1	1	2
88	87	92	+	426	-	-		+	2	0	2
88	87	92	+	735	-	-		+	2	0	2
103	102	103	+	1539	-	-		+	2	0	2
108	105	110	+	183	-	-		+	1	1	2
108	105	110	+	325	-	-		+	1	1	2
108	105	110	+	455		451	455	+	2	0	2
108	105	110	+	594	-	-		+	1	1	2
108	105	110	+	1581	-	-		+	1	1	2
108	105	110	+	1651	-	-		+	1	1	2
116	111	117	+	527	-	-		+	2	0	2
116	111	117	+	802	-	-		+	1	1	2
116	111	117	+	891		891	894	+	1	1	2
116	111	117	+	1097	-	-		+	2	0	2
117	113	119	-	377		373	377	-	2	0	2
123	121	125	-	1555	-	-		-	1	1	2
123	118	127	+	397	-	-		+	1	1	2
123	118	127	+	883	-	-		+	2	0	2
123	118	127	+	936	-	-		+	1	1	2
123	118	127	+	967		967	971	+	1	1	2
123	118	127	+	1194	-	-		+	1	1	2
123	118	127	+	1213	-	-		+	1	1	2
123	118	127	+	1343		1343	1345	+	2	0	2
123	118	127	+	1469		1469	1471	+	1	1	2
123	118	127	+	1600	-	-		+	2	0	2
123	118	127	+	1804	-	-		+	1	1	2
123	118	127	+	2447	-	-		-	1	1	2
131	128	134	+	154	-	-		+	1	1	2
131	128	134	+	198	-	-		+	2	0	2
131	128	134	+	1258	-	-		+	1	1	2
131	128	134	+	1824	-	-		+	1	1	2
132	127	132	-	301	-	-		-	0	2	2
141	137	143	-	14792	-	-		+	2	0	2
149	148	154	+	180	-	-		+	1	1	2
149	148	154	+	196		192	196	+	1	1	2
149	148	154	+	258		258	261	+	1	1	2
149	148	154	+	325		325	326	+	1	1	2
149	148	154	+	893	-	-		+	2	0	2
149	148	154	+	1723	-	-		+	0	2	2
150	148	154	-	528		528	529	-	1	1	2
157	155	160	-	1086	-	-		-	1	1	2
162	162	165	-	307	-	-		-	1	1	2
173	169	177	-	290	-	-		-	1	1	2
173	169	177	-	330	-	-		-	1	1	2
173	169	177	-	449	-	-		-	1	1	2
175	172	179	+	157	-	-		-	1	1	2
175	172	179	+	176	-	-		+	2	0	2
175	172	179	+	514	-	-		+	0	2	2
175	172	179	+	521		521	522	+	1	1	2
175	172	179	+	528	-	-		+	0	2	2
175	172	179	+	1521	-	-		+	2	0	2
181	181	184	-	367		364	367	-	1	1	2
192	187	196	-	349	-	-		-	2	0	2
192	187	196	-	5909	-	-		-	1	1	2
192	191	192	+	313	-	-		+	0	2	2
199	194	201	+	448	-	-		+	0	2	2
199	194	201	+	3392	-	-		-	0	2	2
221	217	221	+	937		934	937	+	1	1	2
223	220	224	-	408	-	-		-	1	1	2
223	220	224	-	521	-	-		-	2	0	2
223	220	224	-	677		677	678	-	1	1	2
227	223	230	+	312	-	-		+	2	0	2
237 -	-		-	583	-	-		-	1	1	2
258	258	260	+	230		228	230	-	1	1	2
263	259	268	-	320		318	320	-	1	1	2
263	259	268	-	417	-	-		-	2	0	2
263	259	268	-	522		522	523	-	0	2	2
263	259	268	-	829	-	-		-	2	0	2
263	259	268	-	14488	-	-		+	1	1	2
265	262	267	+	302		299	302	+	2	0	2
265	262	267	+	544	-	-		+	1	1	2
278	276	278	+	310	-	-		+	1	1	2
278	276	278	+	667	-	-		-	2	0	2
284	283	284	+	903	-	-		-	1	1	2
291	289	293	-	427	-	-		-	0	2	2
291	289	293	-	1879	-	-		-	2	0	2
291	288	293	+	415	-	-		+	2	0	2
291	288	293	+	487	-	-		+	0	2	2
291	288	293	+	629	-	-		+	2	0	2
291	288	293	+	1734		1734	1738	+	1	1	2
299	296	302	+	325		325	328	+	2	0	2
299	296	302	+	431	-	-		+	2	0	2
301	298	301	-	601	-	-		-	1	1	2
301	298	301	-	620	-	-		-	0	2	2
307	304	308	-	1806	-	-		-	2	0	2
312	310	314	-	585		585	587	-	0	2	2
312	309	315	+	372	-	-		+	1	1	2
318	315	321	-	601	-	-		-	0	2	2

327	327	328	+	409	-	-	+	2	0	2
329	325	332	-	519	-	-	-	1	1	2
336	336	338	+	13951	13949	13951	+	1	1	2
357	355	361	+	536	-	-	+	0	2	2
365	362	368	-	511	-	-	-	1	1	2
365	362	368	-	533	533	534	-	1	1	2
365	362	368	-	539	-	-	-	1	1	2
365	362	368	-	586	-	-	-	1	1	2
368	366	370	+	1808	-	-	+	0	2	2
372	370	374	-	522	-	-	-	1	1	2
372	370	374	-	616	-	-	-	1	1	2
372	370	374	-	684	-	-	+	0	2	2
372	370	374	-	1243	-	-	-	0	2	2
372	370	374	-	5175	-	-	-	1	1	2
373	372	375	+	488	-	-	+	0	2	2
380	380	381	-	532	532	533	-	1	1	2
387	387	389	+	417	-	-	+	0	2	2
398	397	399	+	434	434	435	+	1	1	2
398	397	399	+	492	-	-	+	0	2	2
404	403	409	+	417	415	417	+	0	2	2
404	403	409	+	516	-	-	+	1	1	2
404	403	409	+	1755	-	-	+	0	2	2
411	410	411	+	487	487	488	+	0	2	2
417	414	419	+	491	-	-	+	1	1	2
417	414	419	+	1595	-	-	+	2	0	2
426	423	429	-	653	650	653	-	1	1	2
426	423	430	+	512	-	-	+	1	1	2
426	423	430	+	1619	-	-	+	1	1	2
426	423	430	+	10118	-	-	-	2	0	2
441	437	441	-	550	546	550	-	1	1	2
457	454	462	+	483	480	483	+	1	1	2
457	454	462	+	490	490	491	+	0	2	2
457	454	462	+	509	-	-	+	2	0	2
459	456	459	-	1396	-	-	-	2	0	2
465	465	471	+	1112	-	-	+	1	1	2
465	465	471	+	1176	-	-	+	1	1	2
475	474	477	+	522	-	-	+	1	1	2
481	478	484	-	830	-	-	-	2	0	2
481	478	484	-	882	882	884	-	1	1	2
481	478	484	-	2973	-	-	+	1	1	2
484	480	485	+	929	-	-	+	1	1	2
491	490	495	+	490	-	-	+	2	0	2
501	499	504	+	551	-	-	+	1	1	2
501	499	504	+	589	-	-	+	0	2	2
501	499	504	+	594	594	596	+	1	1	2
501	499	504	+	1673	-	-	+	1	1	2
501	499	504	+	1816	-	-	+	1	1	2
516	513	520	-	6310	6310	6311	-	1	1	2
521	521	522	+	840	-	-	+	1	1	2
522	522	523	-	685	-	-	-	0	2	2
541	540	546	-	566	-	-	+	1	1	2
541	540	546	-	1463	-	-	-	1	1	2
541	538	543	+	597	-	-	+	0	2	2
551	551	554	-	599	-	-	-	1	1	2
569	567	573	-	659	657	659	-	1	1	2
569	567	573	-	800	-	-	-	1	1	2
569	567	573	-	895	-	-	-	0	2	2
580	576	582	-	978	974	978	-	2	0	2
581	578	585	+	975	-	-	+	2	0	2
581	578	585	+	988	-	-	+	1	1	2
581	578	585	+	1327	-	-	+	1	1	2
581	578	585	+	1509	-	-	+	1	1	2
581	578	585	+	1791	-	-	+	1	1	2
588	586	589	-	1193	-	-	-	2	0	2
594	592	597	-	884	-	-	-	0	2	2
594	592	597	-	1194	-	-	-	2	0	2
594	592	597	-	1860	-	-	-	2	0	2
611	611	616	-	696	-	-	-	0	2	2
620	618	624	-	696	-	-	-	1	1	2
620	618	624	-	897	-	-	-	1	1	2
620	618	624	-	902	902	904	-	1	1	2
620	618	624	-	987	985	987	-	1	1	2
620	618	624	-	1794	-	-	-	1	1	2
624	624	629	+	672	-	-	+	0	2	2
624	624	629	+	980	-	-	+	2	0	2
634	631	638	+	665	-	-	-	1	1	2
634	631	638	+	935	935	936	-	1	1	2
634	631	638	+	945	-	-	+	0	2	2
647	647	651	-	953	953	954	-	1	1	2
654	654	658	+	855	-	-	+	1	1	2
655	653	659	-	798	-	-	-	1	1	2
655	653	659	-	860	860	861	-	1	1	2
655	653	659	-	893	-	-	-	1	1	2
661	661	665	-	634	-	-	+	2	0	2
671	668	671	+	911	911	912	+	2	0	2
672	-	-	-	14677	-	-	-	1	1	2
679	675	683	+	912	-	-	+	1	1	2
679	675	683	+	1406	-	-	+	2	0	2

679	675	683	+	1595 -	-		+	0	2	2
684	681	690	-	827 -	-		-	0	2	2
689	685	692	+	768	768	769	+	1	1	2
692 -	-		-	3272 -	-		-	0	2	2
700	698	700	+	813 -	-		+	1	1	2
725	725	726	-	758	758	759	+	0	2	2
738 -	-		-	799 -	-		-	1	1	2
761	760	761	-	780 -	-		+	1	1	2
761	760	761	-	1048	1045	1048	-	0	2	2
798	795	802	-	1224	1224	1225	-	1	1	2
802	800	806	+	839 -	-		+	0	2	2
827	825	827	+	1433 -	-		+	1	1	2
833	830	835	-	1171 -	-		+	0	2	2
833	830	835	-	1194	1194	1198	-	1	1	2
838 -	-		-	9136 -	-		-	0	2	2
844	843	849	+	968 -	-		+	1	1	2
862	858	864	-	1072 -	-		-	0	2	2
862	858	864	-	1136	1136	1137	-	1	1	2
888	887	888	+	1073	1073	1074	+	2	0	2
892	889	895	-	1075 -	-		-	2	0	2
892	889	895	-	1083 -	-		-	2	0	2
893	893	898	+	912	910	912	+	1	1	2
898 -	-		-	1045 -	-		-	1	1	2
915	913	915	-	7915 -	-		-	2	0	2
915	913	915	-	10539 -	-		+	1	1	2
916	912	918	+	954	952	954	+	1	1	2
916	912	918	+	1120 -	-		-	2	0	2
928	927	933	-	959 -	-		+	1	1	2
928	927	933	-	1090	1088	1090	-	1	1	2
928	927	933	-	1108 -	-		-	1	1	2
928	927	933	-	1432 -	-		-	2	0	2
936	935	936	-	3411 -	-		+	2	0	2
940	940	943	+	961 -	-		+	0	2	2
961	957	963	-	1179 -	-		-	1	1	2
971	971	972	+	1021 -	-		+	1	1	2
977	977	981	-	1107 -	-		-	1	1	2
980	980	981	+	1179	1179	1180	+	0	2	2
989	987	990	+	2778 -	-		+	1	1	2
1002	998	1005	+	1024	1020	1024	+	2	0	2
1002	998	1005	+	1149 -	-		+	1	1	2
1011	1007	1011	+	1222 -	-		+	0	2	2
1018	1018	1019	+	1073 -	-		+	1	1	2
1031	1029	1031	-	1067 -	-		+	2	0	2
1031	1028	1031	+	1045 -	-		+	0	2	2
1043	1043	1044	-	1137 -	-		-	1	1	2
1043	1043	1044	-	1227 -	-		-	1	1	2
1043	1043	1046	+	1328 -	-		+	1	1	2
1043	1043	1046	+	6627 -	-		+	1	1	2
1051	1048	1051	-	1094 -	-		-	1	1	2
1051	1048	1051	-	1200 -	-		-	1	1	2
1051	1048	1051	-	1224	1224	1225	-	1	1	2
1051	1048	1051	-	1311 -	-		-	1	1	2
1068	1068	1070	-	1092 -	-		+	1	1	2
1080	1080	1082	+	1341 -	-		+	2	0	2
1088	1085	1092	-	1327 -	-		-	0	2	2
1088	1085	1092	-	1339	1337	1339	-	0	2	2
1101	1101	1104	-	10451 -	-		-	2	0	2
1107	1105	1112	+	1235	1232	1235	+	1	1	2
1115	1112	1117	-	1454 -	-		-	0	2	2
1126	1126	1129	+	4055 -	-		+	0	2	2
1137	1135	1137	-	1434 -	-		-	1	1	2
1150	1148	1152	-	1203 -	-		-	1	1	2
1150	1148	1152	-	1322 -	-		-	1	1	2
1150	1148	1152	-	1328 -	-		-	1	1	2
1170	1170	1174	+	1193	1189	1193	+	1	1	2
1179	1177	1180	+	1293 -	-		-	1	1	2
1179	1177	1180	+	7491 -	-		+	1	1	2
1194	1191	1196	+	1255	1255	1256	+	1	1	2
1194	1191	1196	+	1547 -	-		+	1	1	2
1194	1191	1196	+	1678	1678	1682	+	1	1	2
1197	1193	1201	-	1716 -	-		-	2	0	2
1197	1193	1201	-	5737	5737	5738	-	1	1	2
1200	1199	1204	+	1207 -	-		+	1	1	2
1206	1206	1210	+	1228 -	-		+	2	0	2
1212	1209	1213	-	1570 -	-		-	2	0	2
1212	1209	1213	-	9146 -	-		-	1	1	2
1212 -	-		+	1247 -	-		+	0	2	2
1224	1223	1225	-	1393 -	-		-	1	1	2
1224	1223	1227	+	1244	1244	1245	+	1	1	2
1224	1223	1227	+	1638 -	-		+	0	2	2
1238 -	-		-	1551 -	-		-	1	1	2
1242	1240	1242	+	1245	1242	1245	+	1	1	2
1244	1243	1247	-	1681 -	-		-	0	2	2
1259	1255	1260	-	1363 -	-		+	0	2	2
1259	1255	1260	-	1431 -	-		+	0	2	2
1297 -	-		+	1328 -	-		+	1	1	2
1310 -	-		-	4571 -	-		+	0	2	2
1323	1323	1326	-	2534 -	-		-	1	1	2

1328	1326	1329	+	1341	-	-	+	2	0	2
1328	1326	1329	+	1363	-	-	+	2	0	2
1330	1328	1331	-	1440	-	-	-	1	1	2
1330	1328	1331	-	1599	1599	1600	-	0	2	2
1366	1362	1369	+	1492	-	-	+	2	0	2
1376	-	-	+	1453	-	-	+	1	1	2
1376	-	-	+	1463	-	-	+	2	0	2
1376	-	-	+	3167	-	-	+	0	2	2
1383	1380	1387	-	1473	-	-	+	0	2	2
1383	1380	1387	-	14996	-	-	+	1	1	2
1395	1393	1397	-	1790	-	-	-	1	1	2
1395	1393	1397	-	1824	1824	1825	-	1	1	2
1395	1395	1398	+	1467	-	-	+	0	2	2
1407	1407	1412	+	1445	-	-	+	1	1	2
1407	1407	1412	+	1654	-	-	+	2	0	2
1414	1414	1415	+	1706	-	-	+	1	1	2
1431	1427	1432	+	1444	1444	1445	+	0	2	2
1436	1434	1436	+	1476	-	-	+	0	2	2
1440	1440	1444	-	1571	-	-	-	1	1	2
1440	1440	1444	-	1883	-	-	-	1	1	2
1442	1439	1446	+	1482	1479	1482	+	2	0	2
1446	1446	1448	-	2238	-	-	-	1	1	2
1460	1457	1463	+	4410	-	-	-	0	2	2
1466	1462	1471	-	1542	-	-	-	1	1	2
1466	1462	1471	-	1754	-	-	-	2	0	2
1491	-	-	-	1565	-	-	-	2	0	2
1497	1494	1500	+	1594	-	-	+	2	0	2
1497	1494	1500	+	1809	-	-	+	1	1	2
1501	1498	1501	-	1855	-	-	-	0	2	2
1512	1511	1515	-	1791	-	-	-	2	0	2
1531	-	-	+	1614	-	-	+	2	0	2
1552	1550	1553	-	3434	3434	3435	-	1	1	2
1555	1552	1555	+	1578	-	-	+	2	0	2
1563	1563	1567	-	1790	-	-	-	1	1	2
1563	1563	1567	-	1873	-	-	-	1	1	2
1563	1563	1567	-	2312	-	-	-	1	1	2
1563	1562	1564	+	1575	-	-	+	1	1	2
1568	1568	1572	+	1606	-	-	+	1	1	2
1568	1568	1572	+	1798	-	-	+	0	2	2
1574	1570	1578	-	1689	-	-	-	1	1	2
1574	1570	1578	-	1866	-	-	-	1	1	2
1574	1570	1578	-	1878	-	-	-	1	1	2
1580	1577	1581	+	1764	-	-	+	1	1	2
1580	1577	1581	+	1870	-	-	+	0	2	2
1580	1577	1581	+	2795	-	-	+	1	1	2
1589	1585	1593	-	1808	-	-	+	0	2	2
1592	1588	1592	+	1713	-	-	+	0	2	2
1599	1595	1602	-	1792	1792	1796	-	2	0	2
1599	1599	1600	+	1685	1685	1686	+	1	1	2
1609	-	-	+	1708	-	-	+	1	1	2
1636	1636	1637	-	1792	1792	1793	-	1	1	2
1641	1641	1644	+	1683	1680	1683	+	1	1	2
1642	1639	1645	-	1714	-	-	-	1	1	2
1655	1654	1659	-	1826	-	-	-	2	0	2
1661	1658	1665	+	8939	-	-	+	0	2	2
1668	-	-	+	1710	-	-	+	2	0	2
1673	1673	1674	+	1691	1691	1692	+	1	1	2
1678	1677	1681	-	3431	-	-	-	2	0	2
1698	-	-	-	10485	-	-	+	2	0	2
1700	1700	1704	+	1712	-	-	+	1	1	2
1717	1717	1721	-	3145	-	-	+	0	2	2
1717	1717	1721	-	4522	-	-	-	0	2	2
1724	1724	1726	+	1807	-	-	+	0	2	2
1739	1736	1739	-	6309	-	-	-	1	1	2
1739	1736	1739	-	9040	-	-	+	2	0	2
1740	1736	1742	+	1755	-	-	+	0	2	2
1740	1736	1742	+	1793	-	-	+	1	1	2
1740	1736	1742	+	1815	-	-	+	0	2	2
1740	1736	1742	+	3243	-	-	+	1	1	2
1740	1736	1742	+	14393	-	-	+	2	0	2
1746	1746	1747	+	14707	-	-	+	0	2	2
1751	-	-	+	14712	-	-	+	2	0	2
1772	-	-	-	1779	-	-	+	0	2	2
1785	1785	1787	-	1766	-	-	+	2	0	2
1785	1785	1787	-	1999	-	-	-	2	0	2
1785	1785	1787	-	12312	-	-	+	2	0	2
1795	1795	1799	-	6354	-	-	-	1	1	2
1810	1806	1810	-	1855	-	-	-	1	1	2
1810	1806	1810	-	8184	-	-	-	1	1	2
1826	1826	1827	-	6348	-	-	-	1	1	2
1881	1877	1882	+	3169	-	-	+	1	1	2
1881	1877	1882	+	4849	-	-	+	1	1	2
1901	1901	1903	-	2130	-	-	-	1	1	2
1905	-	-	+	1901	-	-	+	2	0	2
1911	1911	1917	+	1983	1981	1983	+	1	1	2
1924	1920	1927	-	2062	-	-	-	1	1	2
1928	1924	1928	+	5971	-	-	+	2	0	2
1935	1933	1935	+	1948	-	-	+	2	0	2

1941		1941	1944	-	2131	-	-		-	1	1	2
1942		1937	1945	+	1963	-	-		+	2	0	2
1942		1937	1945	+	2001	-	-		+	2	0	2
1942		1937	1945	+	2013	-	-		+	2	0	2
1942		1937	1945	+	2628	-	-		+	2	0	2
1942		1937	1945	+	2899	-	-		+	2	0	2
1950		1946	1954	+	2013		2013	2014	+	1	1	2
1950		1946	1954	+	2222	-	-		+	1	1	2
1950		1946	1954	+	4303	-	-		+	1	1	2
1961		1960	1964	-	2134	-	-		-	1	1	2
1961		1960	1964	-	2215	-	-		-	1	1	2
1961		1960	1964	-	2271	-	-		-	0	2	2
1961		1960	1964	-	2340	-	-		-	0	2	2
1967		1963	1968	+	2001	-	-		+	1	1	2
1967		1963	1968	+	2622	-	-		+	2	0	2
1967		1963	1968	+	2768	-	-		+	2	0	2
1969		1969	1973	-	1911	-	-		+	2	0	2
1985		1982	1986	+	2548	-	-		+	1	1	2
1985		1982	1986	+	2721	-	-		+	1	1	2
1985		1982	1986	+	2743	-	-		+	1	1	2
1985		1982	1986	+	3829	-	-		+	1	1	2
1985		1982	1986	+	6538	-	-		+	1	1	2
1985		1982	1986	+	12824	-	-		+	0	2	2
1990		1988	1994	-	2269	-	-		-	0	2	2
1991		1987	1993	+	2118	-	-		+	2	0	2
1991		1987	1993	+	2582		2582	2583	+	1	1	2
1991		1987	1993	+	2740	-	-		+	2	0	2
1991		1987	1993	+	3333		3333	3334	+	1	1	2
2001	-	-		+	3761	-	-		+	1	1	2
2014		2010	2018	+	2367	-	-		+	1	1	2
2014		2010	2018	+	14366	-	-		+	1	1	2
2018		2014	2023	-	2310		2310	2311	-	1	1	2
2018		2014	2023	-	12493	-	-		-	2	0	2
2023		2022	2026	+	3340	-	-		-	1	1	2
2025		2025	2026	-	12505		12505	12506	-	1	1	2
2030		2028	2034	-	2273	-	-		-	0	2	2
2042		2038	2042	-	2095	-	-		-	1	1	2
2042		2038	2042	-	3325	-	-		-	2	0	2
2047		2047	2049	-	2243	-	-		-	1	1	2
2050		2046	2050	+	12529		12525	12529	+	1	1	2
2054		2054	2059	-	2155	-	-		-	2	0	2
2054		2054	2059	-	2432	-	-		-	1	1	2
2063		2063	2065	-	2159	-	-		-	0	2	2
2071		2067	2071	+	13494	-	-		+	2	0	2
2082		2079	2085	-	2326	-	-		-	0	2	2
2095		2090	2099	-	2305	-	-		-	1	1	2
2095		2090	2099	-	2559	-	-		-	1	1	2
2101		2098	2101	+	6011		6011	6012	+	1	1	2
2111		2111	2112	+	2085	-	-		-	0	2	2
2111		2111	2112	+	2143	-	-		+	0	2	2
2118		2115	2120	+	2138	-	-		+	0	2	2
2118		2115	2120	+	2163		2163	2165	+	0	2	2
2118		2115	2120	+	2394	-	-		+	0	2	2
2132		2130	2134	-	2170	-	-		-	1	1	2
2132		2130	2134	-	2324	-	-		-	0	2	2
2137		2137	2141	-	2187	-	-		-	1	1	2
2137		2137	2141	-	2275	-	-		-	2	0	2
2170		2170	2174	-	2205	-	-		-	0	2	2
2170		2170	2174	-	2314		2312	2314	-	2	0	2
2177		2173	2177	+	12245	-	-		+	1	1	2
2181		2181	2184	-	2321	-	-		-	0	2	2
2181		2181	2184	-	6304	-	-		-	0	2	2
2182	-	-		+	2315	-	-		+	1	1	2
2194		2190	2194	+	2209	-	-		-	2	0	2
2194		2190	2194	+	7221	-	-		+	2	0	2
2245		2243	2245	-	2426	-	-		-	1	1	2
2246		2245	2246	+	2268	-	-		-	0	2	2
2252	-	-		-	14654	-	-		-	0	2	2
2283		2278	2284	+	2316	-	-		+	2	0	2
2283		2278	2284	+	2426	-	-		+	1	1	2
2283		2278	2284	+	2435	-	-		+	0	2	2
2283		2278	2284	+	3230	-	-		+	0	2	2
2317	-	-		+	2424	-	-		+	1	1	2
2317	-	-		+	2432	-	-		+	1	1	2
2340		2339	2343	+	2427		2427	2428	+	1	1	2
2360		2360	2364	+	2392	-	-		+	0	2	2
2382		2382	2385	+	2397		2397	2398	+	0	2	2
2388		2386	2388	-	11425	-	-		-	0	2	2
2400		2396	2403	-	2554	-	-		-	0	2	2
2400		2396	2403	-	2745	-	-		+	0	2	2
2400		2396	2403	-	3080	-	-		-	2	0	2
2419		2415	2419	-	2643	-	-		-	1	1	2
2426		2426	2427	+	2444	-	-		+	1	1	2
2434		2430	2435	-	2532	-	-		-	1	1	2
2434		2430	2435	-	2619	-	-		-	0	2	2
2439		2439	2443	-	2576	-	-		-	1	1	2
2466		2464	2466	+	3032	-	-		+	1	1	2
2491		2490	2495	-	2619	-	-		-	1	1	2

2498	2498	2499	+	2517 -	-		-	1	1	2
2536	2534	2539	-	2732 -	-		+	0	2	2
2536	2534	2539	-	2773 -	-		-	0	2	2
2536	2534	2539	-	3046 -	-		-	1	1	2
2543 -	-		-	2671 -	-		-	2	0	2
2594	2590	2594	-	2744 -	-		-	1	1	2
2639	2635	2643	+	12150 -	-		+	2	0	2
2654 -	-		+	2882 -	-		+	2	0	2
2704 -	-		+	4992 -	-		-	2	0	2
2714 -	-		+	2905 -	-		+	2	0	2
2718 -	-		-	2784 -	-		-	2	0	2
2723	2723	2725	+	2746 -	-		+	0	2	2
2723	2723	2725	+	2957 -	-		+	0	2	2
2724	2720	2724	-	2926 -	-		-	1	1	2
2724	2720	2724	-	10502 -	-		+	0	2	2
2732	2731	2733	-	3206	3206	3207	-	1	1	2
2740	2737	2744	+	2767 -	-		+	1	1	2
2744 -	-		-	6291 -	-		-	1	1	2
2746	2745	2746	+	2808 -	-		+	1	1	2
2763	2759	2763	-	14972	14968	14972	-	1	1	2
2768	2765	2768	+	2839 -	-		+	1	1	2
2768	2765	2768	+	10226 -	-		+	2	0	2
2787	2787	2790	-	6842 -	-		-	2	0	2
2827	2827	2830	+	2849 -	-		+	1	1	2
2832 -	-		+	2809 -	-		-	1	1	2
2846	2844	2850	+	3008	3008	3009	+	1	1	2
2846	2844	2850	+	13618 -	-		-	2	0	2
2854	2854	2856	-	3014 -	-		-	0	2	2
2867	2866	2870	-	3621 -	-		-	1	1	2
2868	2865	2868	+	3023	3021	3023	+	1	1	2
2868	2865	2868	+	3631	3629	3631	+	1	1	2
2873	2870	2876	+	2929 -	-		+	1	1	2
2873	2870	2876	+	2935 -	-		+	1	1	2
2873	2870	2876	+	2994 -	-		+	1	1	2
2885	2881	2885	+	12956 -	-		-	0	2	2
2895	2893	2896	+	2900 -	-		+	2	0	2
2926	2925	2926	+	3018 -	-		+	0	2	2
2931	2927	2935	-	3092	3092	3093	-	1	1	2
2931	2927	2935	-	3198 -	-		-	2	0	2
2931	2928	2933	+	2999 -	-		+	0	2	2
2931	2928	2933	+	13632 -	-		-	2	0	2
2940	2940	2944	+	2963 -	-		-	1	1	2
2962	2959	2962	+	2941 -	-		-	0	2	2
2962	2959	2962	+	14530 -	-		+	1	1	2
2970	2968	2974	-	3003 -	-		+	0	2	2
2970	2968	2974	-	3113 -	-		-	2	0	2
2970	2968	2974	-	3210 -	-		-	1	1	2
2992 -	-		+	3976 -	-		+	0	2	2
3004 -	-		+	3053 -	-		+	0	2	2
3011	3011	3012	+	3044 -	-		+	2	0	2
3061	3061	3062	-	5249	5249	5250	-	1	1	2
3072 -	-		+	3060 -	-		-	0	2	2
3087 -	-		-	3578 -	-		-	1	1	2
3098	3098	3100	+	3065 -	-		-	1	1	2
3115	3111	3115	-	3558 -	-		-	1	1	2
3166 -	-		+	10624 -	-		+	0	2	2
3182 -	-		-	3462 -	-		-	2	0	2
3190	3186	3190	-	3342 -	-		-	1	1	2
3190	3186	3190	-	3390 -	-		-	1	1	2
3207	3203	3208	-	3435 -	-		-	1	1	2
3215	3212	3218	-	3290 -	-		-	0	2	2
3215	3212	3218	-	8116 -	-		+	0	2	2
3216 -	-		+	4061 -	-		+	2	0	2
3220 -	-		-	3229 -	-		-	2	0	2
3220 -	-		-	3343 -	-		-	0	2	2
3222 -	-		+	8110 -	-		-	0	2	2
3247	3244	3250	-	3393 -	-		-	0	2	2
3247	3244	3250	-	3435 -	-		-	1	1	2
3247	3244	3250	-	3508 -	-		-	1	1	2
3247	3244	3250	-	3726	3724	3726	-	1	1	2
3247	3244	3250	-	3850 -	-		-	0	2	2
3259	3256	3261	-	3460 -	-		-	1	1	2
3259	3256	3261	-	3478	3475	3478	-	2	0	2
3259	3256	3261	-	3550 -	-		-	1	1	2
3259	3256	3261	-	3559 -	-		-	2	0	2
3259	3256	3261	-	3766 -	-		-	0	2	2
3259	3256	3261	-	5941 -	-		-	2	0	2
3259	3258	3262	+	5565	5565	5566	+	1	1	2
3268	3264	3275	+	6047 -	-		+	1	1	2
3273	3269	3275	-	3373 -	-		-	2	0	2
3273	3269	3275	-	3384	3380	3384	-	1	1	2
3273	3269	3275	-	3494 -	-		-	2	0	2
3279	3279	3284	-	3490	3487	3490	-	2	0	2
3281	3277	3284	+	6076 -	-		+	1	1	2
3287	3286	3287	-	3486 -	-		-	2	0	2
3289	3287	3292	+	3323 -	-		+	1	1	2
3302 -	-		+	5755 -	-		+	2	0	2
3309	3307	3313	+	3341 -	-		+	2	0	2

3309	3307	3313	+	3487	-	-	+	1	1	2
3309	3307	3313	+	5725	-	-	+	1	1	2
3315	3314	3319	+	3335	-	-	+	1	1	2
3315	3314	3319	+	3428	-	-	+	1	1	2
3315	3314	3319	+	5003	-	-	+	0	2	2
3325	3320	3325	+	5854	-	-	+	1	1	2
3325	3320	3325	+	7872	-	-	+	1	1	2
3334	3329	3338	-	3555	-	-	-	2	0	2
3334	3329	3338	-	3798	-	-	-	1	1	2
3334	3329	3338	-	4290	-	-	-	0	2	2
3334	3329	3338	-	4387	-	-	-	0	2	2
3334	3329	3338	-	4464	4464	4465	-	1	1	2
3346	3346	3348	-	3495	-	-	-	1	1	2
3373	3372	3373	-	3586	-	-	-	0	2	2
3373	3373	3379	+	3431	3429	3431	+	1	1	2
3373	3373	3379	+	3437	-	-	+	1	1	2
3373	3373	3379	+	5621	-	-	+	1	1	2
3382	3382	3385	+	3400	-	-	+	1	1	2
3390	3389	3393	+	3557	-	-	+	2	0	2
3390	3389	3393	+	4008	-	-	+	1	1	2
3390	3389	3393	+	4768	-	-	+	2	0	2
3400	3398	3400	+	14080	-	-	+	1	1	2
3402	3398	3403	-	5861	5861	5862	-	1	1	2
3419	3418	3423	+	3439	-	-	+	2	0	2
3419	3418	3423	+	5701	-	-	+	2	0	2
3428	3426	3432	+	4327	-	-	+	1	1	2
3436	3435	3440	-	4397	-	-	-	1	1	2
3437	3437	3441	+	3469	-	-	+	2	0	2
3437	3437	3441	+	3475	-	-	+	2	0	2
3437	3437	3441	+	4081	-	-	+	1	1	2
3437	3437	3441	+	6264	-	-	+	1	1	2
3445	3444	3446	-	3786	-	-	-	2	0	2
3449	-	-	+	11996	-	-	-	0	2	2
3454	3454	3456	+	11194	-	-	+	1	1	2
3469	3465	3469	+	11965	-	-	+	1	1	2
3477	3477	3481	+	3807	-	-	+	2	0	2
3483	-	-	+	9951	-	-	+	0	2	2
3489	3485	3493	+	9755	-	-	-	0	2	2
3493	3492	3496	-	3727	-	-	-	0	2	2
3493	3492	3496	-	3976	3976	3977	-	1	1	2
3510	3507	3510	-	3820	-	-	-	0	2	2
3521	3519	3522	+	5640	-	-	+	1	1	2
3533	3530	3537	+	3729	3727	3729	+	1	1	2
3533	3530	3537	+	3814	-	-	+	1	1	2
3533	3530	3537	+	4318	-	-	+	2	0	2
3537	3534	3538	-	4295	-	-	-	2	0	2
3546	-	-	+	3607	-	-	+	2	0	2
3547	3545	3549	-	3790	-	-	-	1	1	2
3561	3557	3562	-	3820	-	-	-	0	2	2
3568	3565	3568	+	3642	-	-	+	2	0	2
3571	-	-	-	3748	-	-	-	1	1	2
3577	3572	3580	-	4228	-	-	-	1	1	2
3578	3574	3580	+	3696	-	-	+	2	0	2
3578	3574	3580	+	4000	-	-	+	1	1	2
3590	3590	3591	+	12146	-	-	+	2	0	2
3591	3589	3593	-	3750	3747	3750	-	1	1	2
3591	3589	3593	-	4042	-	-	-	1	1	2
3591	3589	3593	-	4295	4292	4295	-	2	0	2
3600	-	-	-	3983	-	-	-	2	0	2
3622	3619	3622	-	3757	-	-	-	1	1	2
3622	3619	3622	-	4679	-	-	-	2	0	2
3628	3626	3632	-	3724	-	-	-	1	1	2
3628	3626	3632	-	3801	-	-	-	1	1	2
3628	3626	3632	-	4069	-	-	-	1	1	2
3628	3626	3632	-	4389	-	-	-	1	1	2
3634	-	-	-	3761	-	-	-	1	1	2
3635	3635	3639	+	3689	-	-	+	1	1	2
3639	3638	3640	-	4026	4026	4027	-	1	1	2
3639	3638	3640	-	5626	5626	5627	-	1	1	2
3644	3644	3647	+	4106	4104	4106	+	1	1	2
3645	3642	3646	-	3820	-	-	-	2	0	2
3645	3642	3646	-	3885	-	-	-	2	0	2
3645	3642	3646	-	4138	-	-	-	0	2	2
3656	3654	3656	+	3682	3680	3682	+	1	1	2
3662	-	-	-	3846	-	-	-	1	1	2
3667	-	-	+	3679	-	-	+	1	1	2
3678	3674	3682	-	3936	-	-	-	1	1	2
3678	3674	3682	-	4013	4013	4014	-	1	1	2
3688	3687	3688	+	3934	-	-	+	0	2	2
3690	3688	3690	-	3886	3884	3886	-	1	1	2
3694	3692	3694	+	3823	-	-	-	0	2	2
3720	3716	3722	+	4307	-	-	+	0	2	2
3727	-	-	-	4295	-	-	-	1	1	2
3766	3764	3770	-	4021	-	-	-	0	2	2
3767	3765	3768	+	3801	-	-	+	0	2	2
3782	-	-	+	4031	-	-	-	2	0	2
3784	3784	3785	-	3897	-	-	-	1	1	2
3784	3784	3785	-	4034	-	-	-	2	0	2

3794	3794	3795	+	3857	-	-	+	2	0	2
3794	3794	3795	+	4329	-	-	+	1	1	2
3801	-	-	+	3821	-	-	+	1	1	2
3803	3802	3803	-	4665	-	-	-	2	0	2
3810	3809	3810	-	3934	-	-	-	2	0	2
3853	3851	3854	+	14293	-	-	-	0	2	2
3863	3862	3864	+	12869	12869	12870	+	1	1	2
3885	3885	3886	+	3972	-	-	+	1	1	2
3912	3910	3913	-	4289	4289	4290	-	1	1	2
3912	3910	3913	-	4384	-	-	-	2	0	2
3935	3932	3937	-	4101	-	-	-	0	2	2
3948	-	-	+	7960	-	-	+	2	0	2
3976	3973	3980	+	4013	-	-	+	2	0	2
3976	3973	3980	+	4027	4024	4027	+	0	2	2
3987	-	-	+	4125	-	-	+	2	0	2
3989	3988	3989	-	4268	-	-	-	0	2	2
4000	4000	4004	+	4038	-	-	+	1	1	2
4000	4000	4004	+	11732	-	-	+	2	0	2
4015	4011	4015	+	4039	-	-	+	1	1	2
4015	4011	4015	+	4290	-	-	+	2	0	2
4020	4017	4023	+	4041	4041	4042	+	1	1	2
4020	4017	4023	+	4056	-	-	+	1	1	2
4020	4017	4023	+	8529	-	-	+	0	2	2
4020	4017	4023	+	9503	-	-	-	0	2	2
4020	4017	4023	+	9508	-	-	-	2	0	2
4036	4034	4039	-	4269	4267	4269	-	1	1	2
4036	4034	4039	-	5138	-	-	-	0	2	2
4036	4034	4039	-	5145	-	-	-	1	1	2
4043	4042	4047	-	4289	-	-	-	0	2	2
4055	4051	4059	-	4278	-	-	-	1	1	2
4057	4056	4058	+	12324	-	-	+	1	1	2
4064	4061	4064	-	4278	-	-	-	0	2	2
4064	4061	4064	-	4342	-	-	-	1	1	2
4071	4069	4071	-	4277	-	-	-	0	2	2
4071	4069	4071	-	12620	-	-	+	0	2	2
4122	4118	4125	+	4215	4215	4216	+	1	1	2
4132	4132	4135	-	4385	-	-	-	1	1	2
4145	4143	4146	-	4298	4298	4299	-	1	1	2
4145	4143	4146	-	4387	4387	4389	-	2	0	2
4145	4143	4146	-	4457	-	-	-	0	2	2
4145	4143	4146	-	4567	4565	4567	-	1	1	2
4145	-	-	+	13247	-	-	+	1	1	2
4150	4149	4150	-	4305	-	-	-	2	0	2
4158	-	-	-	4179	-	-	+	1	1	2
4166	-	-	-	4237	-	-	-	2	0	2
4181	4181	4184	+	14575	-	-	-	0	2	2
4261	4260	4261	+	4356	-	-	+	1	1	2
4266	4263	4269	+	4299	-	-	+	0	2	2
4266	4263	4269	+	4329	-	-	+	0	2	2
4266	4263	4269	+	4860	-	-	-	0	2	2
4267	4265	4267	-	4760	-	-	-	0	2	2
4271	-	-	+	4304	-	-	+	1	1	2
4278	4278	4279	+	4569	-	-	-	2	0	2
4290	4288	4293	+	4319	4319	4320	+	2	0	2
4302	4300	4302	-	4718	-	-	-	1	1	2
4302	4300	4302	-	6053	-	-	-	2	0	2
4334	-	-	+	14489	-	-	+	1	1	2
4361	-	-	-	6297	-	-	-	1	1	2
4381	4381	4385	+	4423	-	-	+	2	0	2
4438	-	-	-	4855	-	-	-	0	2	2
4464	4464	4466	+	4495	-	-	+	2	0	2
4472	4469	4475	-	4730	-	-	-	0	2	2
4484	4482	4486	+	4578	-	-	+	1	1	2
4488	4488	4489	-	9051	9051	9052	-	1	1	2
4494	4494	4495	+	4729	-	-	+	2	0	2
4494	4494	4495	+	5730	-	-	+	1	1	2
4517	-	-	-	4590	-	-	-	0	2	2
4530	-	-	+	5066	-	-	-	2	0	2
4534	4534	4538	-	5933	-	-	-	1	1	2
4535	-	-	+	4588	-	-	+	1	1	2
4541	4539	4545	+	4956	-	-	+	2	0	2
4541	4539	4545	+	5104	-	-	+	2	0	2
4541	4539	4545	+	5999	-	-	+	2	0	2
4543	-	-	-	5853	5853	5854	-	0	2	2
4552	4552	4556	+	5464	-	-	+	1	1	2
4561	4557	4565	-	5585	5585	5587	-	1	1	2
4567	4567	4568	-	5700	-	-	-	2	0	2
4568	-	-	+	4599	-	-	+	2	0	2
4576	4576	4578	+	5499	-	-	+	2	0	2
4585	4580	4588	+	4969	-	-	-	2	0	2
4585	4580	4588	+	5662	-	-	+	1	1	2
4585	4580	4588	+	5688	-	-	+	1	1	2
4587	4583	4592	-	4751	-	-	-	1	1	2
4587	4583	4592	-	4972	4972	4973	-	1	1	2
4587	4583	4592	-	5529	-	-	-	2	0	2
4587	4583	4592	-	10372	-	-	-	0	2	2
4591	4591	4592	+	9849	-	-	+	2	0	2
4617	4614	4620	+	15149	-	-	-	1	1	2

4633	4629	4638	-	5625	-	-	-	2	0	2
4635	4631	4635	+	11372	-	-	+	1	1	2
4648	-	-	+	4696	-	-	+	2	0	2
4653	4650	4656	+	4662	-	-	+	0	2	2
4653	4650	4656	+	4776	-	-	+	1	1	2
4664	4659	4667	-	5305	-	-	-	2	0	2
4664	4659	4667	-	5565	-	-	-	2	0	2
4679	4679	4681	+	10068	10068	10069	-	1	1	2
4682	4678	4682	-	5091	-	-	-	0	2	2
4687	4683	4691	-	5073	-	-	-	2	0	2
4687	4683	4691	-	5109	-	-	-	1	1	2
4693	4692	4697	-	4929	-	-	-	2	0	2
4693	4692	4697	-	5071	-	-	-	1	1	2
4693	4692	4697	-	5092	-	-	-	0	2	2
4693	4692	4697	-	5653	5653	5654	-	1	1	2
4693	4693	4696	+	5453	-	-	+	2	0	2
4705	4701	4705	-	4838	-	-	-	0	2	2
4710	4706	4710	-	4909	4909	4910	-	1	1	2
4733	4728	4733	+	4795	-	-	+	2	0	2
4733	4728	4733	+	4840	4838	4840	+	1	1	2
4742	4738	4745	-	5433	5429	5433	-	0	2	2
4744	4742	4747	+	4757	-	-	+	1	1	2
4744	4742	4747	+	15113	15111	15113	-	1	1	2
4750	-	-	+	4799	-	-	+	1	1	2
4751	4746	4753	-	4947	-	-	-	1	1	2
4755	4755	4756	+	4976	-	-	+	2	0	2
4765	4765	4768	+	4836	-	-	+	2	0	2
4767	4766	4770	-	5400	5400	5401	-	1	1	2
4774	4772	4774	+	6352	-	-	+	0	2	2
4778	4775	4782	-	4962	-	-	-	1	1	2
4778	4775	4782	-	5354	-	-	-	1	1	2
4778	4775	4782	-	5834	-	-	-	1	1	2
4780	-	-	+	4793	-	-	+	1	1	2
4805	4801	4806	-	5180	-	-	-	2	0	2
4805	4801	4806	-	5205	5205	5208	-	1	1	2
4805	4801	4806	-	5326	5324	5326	-	0	2	2
4830	4829	4830	+	14046	-	-	+	0	2	2
4837	4837	4839	+	4956	-	-	+	0	2	2
4837	4837	4839	+	5264	-	-	+	2	0	2
4843	4843	4846	+	5086	-	-	+	2	0	2
4847	4844	4847	-	5249	-	-	-	1	1	2
4853	4852	4855	+	4908	-	-	+	1	1	2
4862	4859	4862	+	4997	-	-	+	1	1	2
4877	4877	4879	+	4859	-	-	-	1	1	2
4891	4889	4892	+	4957	-	-	+	1	1	2
4891	4889	4892	+	5155	-	-	+	1	1	2
4894	4893	4896	-	9434	-	-	-	0	2	2
4907	4906	4911	-	5047	-	-	-	1	1	2
4907	4906	4911	-	5071	-	-	-	0	2	2
4925	4923	4927	-	5108	-	-	-	2	0	2
4940	-	-	-	12865	-	-	-	0	2	2
4957	-	-	-	5250	-	-	-	1	1	2
4966	4962	4966	-	5474	5474	5475	-	1	1	2
4973	-	-	-	5205	-	-	-	1	1	2
4979	4976	4979	+	4997	-	-	+	0	2	2
4979	4976	4979	+	5013	-	-	+	0	2	2
4999	4997	5001	-	5127	5127	5128	-	1	1	2
5030	5026	5034	-	5179	-	-	-	1	1	2
5030	5026	5034	-	5265	-	-	-	1	1	2
5030	5026	5034	-	5405	-	-	-	2	0	2
5062	5060	5062	+	5086	-	-	+	2	0	2
5066	5062	5069	-	5537	5537	5538	-	1	1	2
5066	5062	5069	-	15100	15096	15100	+	2	0	2
5096	5094	5096	-	15065	15063	15065	+	0	2	2
5102	5102	5105	-	5321	-	-	-	0	2	2
5113	5109	5114	-	5308	5306	5308	-	1	1	2
5113	5109	5114	-	5355	-	-	-	0	2	2
5121	-	-	-	5295	-	-	-	1	1	2
5122	-	-	+	13456	-	-	+	0	2	2
5128	-	-	-	5295	-	-	-	1	1	2
5136	5136	5138	-	5278	-	-	-	1	1	2
5136	5136	5138	-	5537	-	-	-	2	0	2
5153	5150	5153	-	5450	-	-	-	2	0	2
5153	5150	5153	-	10860	10857	10860	+	1	1	2
5178	5176	5182	+	5253	-	-	+	1	1	2
5221	5218	5225	-	5389	-	-	-	1	1	2
5221	5218	5225	-	5422	5422	5423	-	1	1	2
5221	5218	5225	-	5428	-	-	-	1	1	2
5221	5218	5225	-	5639	-	-	-	0	2	2
5222	5222	5225	+	5273	-	-	+	2	0	2
5251	5250	5252	-	5289	5289	5290	+	2	0	2
5251	5250	5252	-	5421	-	-	-	1	1	2
5251	5250	5252	-	5587	-	-	-	2	0	2
5262	5260	5266	+	5275	-	-	+	1	1	2
5262	5260	5266	+	5280	-	-	-	2	0	2
5268	-	-	+	12756	-	-	+	2	0	2
5277	5273	5280	-	5421	-	-	-	1	1	2
5277	5273	5280	-	15273	-	-	-	1	1	2

5290	5290	5293	-	5449	-	-	-	1	1	2
5316	5313	5317	+	5408	-	-	+	0	2	2
5332	-	-	+	5632	-	-	-	2	0	2
5359	5358	5363	-	5892	5888	5892	-	0	2	2
5401	5401	5402	+	5938	-	-	+	0	2	2
5408	5406	5408	+	5485	-	-	+	0	2	2
5414	5413	5417	+	5452	-	-	+	2	0	2
5414	5413	5417	+	5461	-	-	+	0	2	2
5449	5449	5452	-	5600	-	-	-	2	0	2
5456	5456	5457	+	5485	-	-	+	0	2	2
5473	5472	5476	-	5788	5788	5789	-	1	1	2
5526	5526	5529	+	15145	-	-	+	2	0	2
5539	-	-	-	5661	-	-	-	1	1	2
5539	-	-	+	6008	-	-	+	2	0	2
5585	-	-	+	5657	-	-	+	1	1	2
5604	-	-	+	5641	-	-	+	2	0	2
5647	5647	5648	+	5678	5678	5679	+	1	1	2
5650	5650	5654	-	5820	-	-	-	0	2	2
5650	5650	5654	-	6039	-	-	-	1	1	2
5656	5656	5660	-	6039	-	-	-	1	1	2
5656	5655	5656	+	5674	-	-	+	1	1	2
5656	5655	5656	+	5703	-	-	+	2	0	2
5668	-	-	-	5974	-	-	-	1	1	2
5668	-	-	-	6034	-	-	-	1	1	2
5685	5682	5685	-	5885	-	-	-	1	1	2
5695	-	-	+	6025	-	-	+	2	0	2
5707	-	-	+	13381	-	-	+	1	1	2
5854	5854	5855	+	5888	-	-	+	0	2	2
5870	-	-	+	5856	-	-	-	2	0	2
5885	5882	5885	+	12640	-	-	+	1	1	2
5917	5914	5919	-	6026	-	-	-	2	0	2
5940	5940	5941	+	5986	-	-	+	2	0	2
5940	5940	5941	+	12366	-	-	+	0	2	2
5945	5942	5945	-	6081	-	-	-	1	1	2
5965	-	-	+	5999	-	-	+	2	0	2
6009	-	-	-	9239	-	-	-	2	0	2
6023	6023	6027	+	8921	-	-	+	2	0	2
6033	6033	6034	-	6233	-	-	-	0	2	2
6045	6045	6047	-	6199	-	-	-	1	1	2
6059	-	-	+	6078	6076	6078	+	0	2	2
6140	-	-	+	12063	-	-	+	2	0	2
6157	-	-	+	9838	-	-	-	2	0	2
6192	6190	6192	-	6211	-	-	+	0	2	2
6203	6199	6203	+	10997	10994	10997	+	1	1	2
6213	6209	6216	-	13211	-	-	+	2	0	2
6228	6224	6231	-	6315	-	-	-	1	1	2
6228	6224	6231	-	6482	6482	6483	-	1	1	2
6228	6224	6231	-	6501	6501	6502	-	1	1	2
6240	6236	6245	-	6303	-	-	-	1	1	2
6240	6236	6245	-	6447	6447	6450	-	2	0	2
6240	6236	6245	-	6463	6463	6464	-	2	0	2
6240	6236	6245	-	6482	-	-	-	1	1	2
6240	6236	6245	-	6520	-	-	-	1	1	2
6251	6246	6253	-	6389	-	-	-	1	1	2
6251	6246	6253	-	6406	6406	6407	-	1	1	2
6251	6246	6253	-	6430	-	-	-	1	1	2
6251	6246	6253	-	6438	-	-	-	1	1	2
6251	6246	6253	-	6479	-	-	-	1	1	2
6251	6246	6253	-	6494	6491	6494	-	2	0	2
6269	-	-	-	6490	-	-	-	1	1	2
6278	6274	6280	+	6368	-	-	+	2	0	2
6278	6274	6280	+	6793	-	-	+	2	0	2
6293	6289	6297	+	6314	-	-	+	1	1	2
6293	6289	6297	+	6324	-	-	+	1	1	2
6293	6289	6297	+	6329	-	-	+	1	1	2
6293	6289	6297	+	7837	-	-	+	1	1	2
6302	6299	6304	+	6320	-	-	+	2	0	2
6302	6299	6304	+	6389	6389	6390	+	1	1	2
6302	6299	6304	+	6406	6406	6407	+	1	1	2
6302	6299	6304	+	6453	-	-	-	2	0	2
6302	6299	6304	+	7424	-	-	+	1	1	2
6307	6306	6311	+	6356	-	-	+	1	1	2
6307	6306	6311	+	6363	6363	6364	+	2	0	2
6307	6306	6311	+	7428	-	-	+	2	0	2
6307	6306	6311	+	7967	-	-	+	1	1	2
6309	-	-	-	6459	-	-	-	1	1	2
6315	6313	6318	+	6330	-	-	+	1	1	2
6315	6313	6318	+	6351	-	-	+	1	1	2
6315	6313	6318	+	6971	-	-	+	1	1	2
6316	6316	6317	-	6458	-	-	-	1	1	2
6328	6324	6328	-	6948	-	-	-	2	0	2
6329	6327	6330	+	6358	-	-	+	1	1	2
6329	6327	6330	+	6377	6373	6377	+	1	1	2
6329	6327	6330	+	6578	-	-	+	2	0	2
6340	-	-	-	6509	-	-	-	1	1	2
6340	6337	6344	+	6340	-	-	+	0	2	2
6340	6337	6344	+	6352	-	-	+	1	1	2
6340	6337	6344	+	7385	-	-	+	0	2	2

6345	-	-	-	6501	-	-	-	1	1	2
6345	-	-	-	14552	-	-	-	0	2	2
6349	6349	6351	+	6361	-	-	+	0	2	2
6349	6349	6351	+	6374	-	-	+	1	1	2
6351	6350	6354	-	6461	6459	6461	-	1	1	2
6351	6350	6354	-	7178	7178	7180	-	1	1	2
6357	-	-	-	6493	-	-	-	1	1	2
6363	-	-	-	6586	-	-	-	1	1	2
6373	6371	6373	-	8803	-	-	+	1	1	2
6373	-	-	+	6407	-	-	+	2	0	2
6435	-	-	-	9388	-	-	+	1	1	2
6450	6448	6450	-	6402	-	-	+	1	1	2
6458	6458	6459	+	7251	-	-	+	2	0	2
6502	-	-	-	6675	-	-	-	1	1	2
6509	6508	6510	+	6576	6576	6577	+	1	1	2
6509	6508	6510	+	12965	-	-	+	2	0	2
6556	6554	6558	-	6885	-	-	-	0	2	2
6559	6559	6562	+	13590	-	-	+	0	2	2
6563	6561	6564	-	6831	-	-	-	1	1	2
6563	6561	6564	-	7836	-	-	-	2	0	2
6586	6584	6588	-	6826	6826	6827	-	2	0	2
6586	6584	6588	-	6864	-	-	-	1	1	2
6586	6584	6588	-	8403	8401	8403	-	1	1	2
6598	6598	6600	-	7385	7385	7386	+	1	1	2
6607	-	-	+	6641	-	-	+	1	1	2
6621	6617	6623	-	7024	-	-	-	1	1	2
6641	6641	6645	-	6768	-	-	-	1	1	2
6644	6642	6646	+	7867	-	-	+	2	0	2
6644	6642	6646	+	14184	-	-	+	1	1	2
6651	-	-	+	12406	-	-	+	2	0	2
6659	6659	6661	-	6751	-	-	-	1	1	2
6659	6659	6661	-	7715	-	-	-	0	2	2
6660	6658	6660	+	6673	-	-	+	2	0	2
6660	6658	6660	+	6712	-	-	+	1	1	2
6674	6674	6678	+	7817	-	-	+	1	1	2
6674	6674	6678	+	9983	-	-	+	1	1	2
6677	6675	6677	-	6906	-	-	-	0	2	2
6699	6699	6700	+	6762	6762	6763	+	1	1	2
6699	6699	6700	+	6802	-	-	+	2	0	2
6711	6710	6712	+	6745	-	-	+	2	0	2
6711	6710	6712	+	6760	-	-	+	2	0	2
6745	-	-	+	6763	-	-	+	1	1	2
6755	6751	6755	+	14602	-	-	+	1	1	2
6763	6763	6765	+	9517	-	-	+	1	1	2
6778	-	-	-	7171	-	-	-	0	2	2
6854	-	-	+	7739	-	-	+	0	2	2
6866	6864	6866	-	7234	-	-	-	1	1	2
6874	6874	6878	-	7581	-	-	-	2	0	2
6903	-	-	-	7162	-	-	-	1	1	2
6908	6907	6908	-	7117	-	-	-	1	1	2
6908	6907	6908	-	7715	-	-	-	2	0	2
6948	-	-	+	7797	-	-	+	1	1	2
6949	6949	6951	-	7053	-	-	-	1	1	2
6953	-	-	+	7068	-	-	+	1	1	2
6959	6956	6959	-	7420	-	-	-	2	0	2
6967	-	-	-	8152	-	-	-	1	1	2
6969	6965	6969	+	6974	6974	6975	+	0	2	2
6986	-	-	+	7238	-	-	+	0	2	2
7000	6996	7000	-	14107	-	-	+	0	2	2
7031	7031	7033	-	8251	8249	8251	-	1	1	2
7065	7061	7069	-	7210	7210	7211	-	1	1	2
7065	7061	7069	-	7250	-	-	-	1	1	2
7065	7061	7069	-	7277	-	-	-	1	1	2
7065	7061	7069	-	7412	-	-	-	2	0	2
7082	7082	7087	-	7236	7234	7236	-	1	1	2
7082	7082	7087	-	7260	7260	7264	-	1	1	2
7082	7082	7087	-	8252	8250	8252	-	1	1	2
7101	7100	7102	-	8408	-	-	-	1	1	2
7133	7129	7133	-	7282	-	-	-	1	1	2
7133	7129	7133	-	9283	-	-	+	2	0	2
7144	7140	7148	+	8728	-	-	+	1	1	2
7155	-	-	+	12609	-	-	+	0	2	2
7171	-	-	-	8309	-	-	-	0	2	2
7189	-	-	-	7922	-	-	+	2	0	2
7193	7190	7193	+	7239	7235	7239	+	0	2	2
7203	7200	7207	+	7524	-	-	+	2	0	2
7203	7200	7207	+	7648	-	-	+	1	1	2
7207	7205	7210	-	7404	-	-	-	1	1	2
7207	7205	7210	-	7463	-	-	-	1	1	2
7223	7223	7224	-	7371	-	-	-	1	1	2
7223	7223	7225	+	7251	-	-	+	1	1	2
7238	-	-	+	7355	-	-	+	2	0	2
7253	7252	7253	+	7581	-	-	+	0	2	2
7293	-	-	+	7307	-	-	+	2	0	2
7385	7385	7386	+	7417	-	-	+	2	0	2
7396	7395	7397	-	7603	-	-	-	1	1	2
7402	7401	7402	-	14746	-	-	+	1	1	2
7423	-	-	-	7476	-	-	+	2	0	2

7423 -	-		-	7662 -	-		-	2	0	2
7438 -	-		+	7754 -	-		-	2	0	2
7458 -	-		+	7913 -	-		+	2	0	2
7475 -	-		-	8137 -	-		-	0	2	2
7485 -	-		+	8785 -	-		-	2	0	2
7490	7487	7490	-	7631	7631	7632	-	1	1	2
7490	7487	7490	-	8180 -	-		-	1	1	2
7500 -	-		-	7589 -	-		-	2	0	2
7549	7549	7550	-	8319 -	-		-	1	1	2
7556	7553	7559	+	7569 -	-		+	2	0	2
7556	7553	7559	+	7728 -	-		-	2	0	2
7579 -	-		-	7784 -	-		-	2	0	2
7611	7611	7613	+	9512	9512	9513	-	1	1	2
7615 -	-		-	7869 -	-		-	0	2	2
7615 -	-		-	8171 -	-		-	1	1	2
7619	7619	7623	+	9500	9500	9501	-	2	0	2
7630	7630	7634	-	7636 -	-		-	2	0	2
7630	7630	7634	-	13196 -	-		-	1	1	2
7632 -	-		+	7656 -	-		+	1	1	2
7654 -	-		-	9386 -	-		-	0	2	2
7670 -	-		+	7960 -	-		-	2	0	2
7685	7681	7686	-	7910	7910	7911	-	0	2	2
7697	7696	7698	-	7911 -	-		-	0	2	2
7697	7696	7698	-	7930	7928	7930	-	1	1	2
7699	7698	7699	+	7906 -	-		-	2	0	2
7718 -	-		-	7929 -	-		-	0	2	2
7726	7726	7728	+	8572 -	-		+	0	2	2
7735	7735	7737	+	7867 -	-		+	1	1	2
7738	7738	7739	-	7866 -	-		-	1	1	2
7771 -	-		-	8000 -	-		-	2	0	2
7781	7779	7785	+	7817	7813	7817	+	1	1	2
7825	7825	7830	-	7915 -	-		-	1	1	2
7842	7838	7847	+	7873	7870	7873	+	2	0	2
7871	7868	7875	-	8280	8277	8280	-	0	2	2
7906	7906	7910	-	7980 -	-		-	1	1	2
7906	7906	7910	-	8180	8177	8180	-	2	0	2
7911 -	-		+	7931 -	-		+	0	2	2
7922	7919	7923	+	7943	7943	7944	+	2	0	2
7952 -	-		-	8134 -	-		-	1	1	2
7966 -	-		-	7995 -	-		-	0	2	2
7966 -	-		-	8112 -	-		-	1	1	2
7973	7972	7973	-	8170 -	-		-	0	2	2
7980 -	-		-	8175 -	-		-	0	2	2
7997	7997	7998	-	8185	8185	8186	-	1	1	2
8011	8009	8011	+	8026 -	-		+	1	1	2
8026	8024	8026	-	8182 -	-		-	1	1	2
8070	8067	8070	+	8135 -	-		+	2	0	2
8072	8072	8075	-	15219 -	-		-	0	2	2
8075	8075	8080	+	13746 -	-		+	1	1	2
8075	8075	8080	+	13760 -	-		+	1	1	2
8075	8075	8080	+	13840 -	-		+	0	2	2
8083	8083	8087	-	9121	9121	9122	-	1	1	2
8083	8083	8087	-	12083 -	-		-	2	0	2
8107	8107	8111	-	8407 -	-		-	2	0	2
8136 -	-		+	8226 -	-		+	1	1	2
8154	8151	8154	-	8398 -	-		-	1	1	2
8154	8152	8154	+	12921 -	-		-	0	2	2
8176	8174	8177	-	8423 -	-		+	0	2	2
8176	8174	8177	-	9957 -	-		-	0	2	2
8177	8173	8177	+	8193	8193	8194	+	1	1	2
8177	8173	8177	+	8200 -	-		+	0	2	2
8195 -	-		-	8412 -	-		-	1	1	2
8341	8339	8341	-	8830 -	-		+	0	2	2
8409 -	-		+	8407	8405	8407	+	2	0	2
8433 -	-		-	9346 -	-		-	1	1	2
8472	8472	8473	+	8483 -	-		+	2	0	2
8480	8477	8483	-	8710 -	-		-	1	1	2
8508	8503	8510	+	8803 -	-		+	1	1	2
8508	8503	8510	+	11505 -	-		+	1	1	2
8521 -	-		+	11198 -	-		+	1	1	2
8695	8695	8697	-	8935 -	-		-	2	0	2
8724	8722	8724	+	8739	8739	8740	+	1	1	2
8772	8772	8777	+	8835 -	-		+	0	2	2
8796 -	-		-	8997 -	-		+	0	2	2
8801	8799	8801	-	9151 -	-		-	2	0	2
8808	8804	8808	+	14076 -	-		+	0	2	2
8831	8828	8831	+	11355	11351	11355	-	0	2	2
8834 -	-		-	11349 -	-		+	0	2	2
8913 -	-		-	10459 -	-		-	0	2	2
8936	8936	8940	+	8950 -	-		+	1	1	2
8936	8936	8940	+	9096 -	-		+	1	1	2
8953 -	-		-	14813 -	-		+	2	0	2
9029	9029	9030	+	10607	10607	10608	+	1	1	2
9051 -	-		+	9082 -	-		+	1	1	2
9052 -	-		-	9324 -	-		-	2	0	2
9061	9061	9062	-	9271	9271	9272	-	1	1	2
9162	9162	9164	-	9468 -	-		-	1	1	2
9197 -	-		+	9197 -	-		+	0	2	2

9206	-	-	-	9507	-	-	-	1	1	2
9218	-	-	+	9461	-	-	-	2	0	2
9286	9283	9289	-	9369	-	-	-	1	1	2
9300	9300	9303	-	9555	9555	9556	-	1	1	2
9346	-	-	-	11145	-	-	+	1	1	2
9346	9345	9347	+	9375	9375	9376	+	1	1	2
9369	-	-	+	9395	-	-	+	1	1	2
9371	9367	9375	-	12103	-	-	+	1	1	2
9393	9393	9394	+	11300	11300	11301	+	1	1	2
9429	9429	9433	-	12430	-	-	+	1	1	2
9471	-	-	-	14537	-	-	+	1	1	2
9489	9485	9489	-	9803	-	-	-	1	1	2
9507	-	-	-	9754	-	-	-	1	1	2
9514	9514	9517	+	9529	-	-	+	1	1	2
9589	-	-	+	14043	-	-	-	0	2	2
9591	-	-	-	14477	-	-	+	1	1	2
9608	-	-	+	15145	-	-	+	2	0	2
9664	9660	9664	-	13043	-	-	-	1	1	2
9800	9798	9801	+	14290	-	-	+	0	2	2
9806	-	-	-	13793	-	-	+	2	0	2
9880	-	-	+	9894	-	-	+	1	1	2
9895	-	-	+	13911	-	-	+	0	2	2
9905	9902	9909	-	11081	-	-	+	1	1	2
9911	-	-	-	10058	-	-	-	0	2	2
9929	-	-	-	11125	-	-	-	0	2	2
9935	-	-	+	9954	-	-	-	1	1	2
9941	9941	9942	-	14108	14108	14109	-	1	1	2
9997	-	-	-	10217	-	-	-	0	2	2
10007	-	-	-	12848	-	-	-	1	1	2
10050	10048	10050	-	10211	-	-	-	2	0	2
10061	10060	10065	-	10224	-	-	-	1	1	2
10088	10087	10088	+	15285	-	-	+	1	1	2
10096	10096	10099	+	10383	10383	10384	+	1	1	2
10147	-	-	+	10204	-	-	+	0	2	2
10153	-	-	+	15145	-	-	+	1	1	2
10258	-	-	+	10290	-	-	+	1	1	2
10272	-	-	-	15277	-	-	+	0	2	2
10302	-	-	-	10496	-	-	-	1	1	2
10311	-	-	-	12639	-	-	+	2	0	2
10324	10320	10326	+	10493	10493	10494	+	1	1	2
10334	-	-	-	10470	-	-	-	0	2	2
10346	10346	10349	-	10544	-	-	-	2	0	2
10374	10374	10377	-	13243	-	-	-	2	0	2
10456	10455	10458	-	13550	-	-	-	2	0	2
10529	10529	10532	-	13229	-	-	+	2	0	2
10542	-	-	+	10564	-	-	+	1	1	2
10543	10540	10543	-	10837	-	-	-	2	0	2
10554	10552	10558	-	10664	-	-	-	1	1	2
10570	-	-	+	13759	-	-	+	2	0	2
10583	10583	10586	+	11165	-	-	+	0	2	2
10583	10583	10586	+	13952	-	-	+	0	2	2
10640	-	-	+	10739	-	-	+	1	1	2
10654	10653	10654	-	13743	-	-	+	1	1	2
10704	10704	10706	-	14864	-	-	+	1	1	2
10719	10719	10721	+	15347	-	-	-	2	0	2
10727	10727	10728	-	10944	10944	10945	-	1	1	2
10746	-	-	-	12689	-	-	+	2	0	2
10869	-	-	+	11360	-	-	+	0	2	2
10954	10954	10957	-	13954	-	-	+	0	2	2
10961	10959	10961	+	12212	12209	12212	+	0	2	2
10977	10974	10978	-	12726	-	-	-	2	0	2
10987	-	-	-	11221	-	-	-	2	0	2
11024	-	-	-	11225	-	-	+	1	1	2
11082	11082	11083	+	14703	14703	14704	-	1	1	2
11106	-	-	-	14401	-	-	+	0	2	2
11120	11120	11122	+	13498	13498	13499	+	1	1	2
11134	11134	11138	+	14788	-	-	+	2	0	2
11161	11158	11165	-	11325	-	-	-	1	1	2
11161	11158	11165	-	11338	11338	11339	-	1	1	2
11161	11158	11165	-	14035	-	-	+	1	1	2
11161	11158	11165	-	14914	14914	14915	+	1	1	2
11188	11186	11188	-	11281	-	-	-	0	2	2
11188	11186	11188	-	11492	-	-	-	1	1	2
11227	-	-	-	13799	-	-	-	0	2	2
11234	11234	11239	-	14776	-	-	+	2	0	2
11248	11248	11250	-	13831	-	-	-	1	1	2
11266	-	-	-	11339	-	-	-	1	1	2
11269	-	-	+	11626	-	-	-	2	0	2
11300	11296	11301	+	11606	11606	11607	-	2	0	2
11356	-	-	-	14601	-	-	+	0	2	2
11373	11373	11377	+	15072	-	-	+	2	0	2
11384	11384	11387	+	11624	-	-	-	2	0	2
11384	11384	11387	+	11649	11647	11649	-	2	0	2
11395	11392	11395	+	11642	-	-	-	2	0	2
11429	-	-	-	13739	-	-	-	2	0	2
11442	-	-	+	11433	11433	11434	-	1	1	2
11482	11482	11484	-	13962	-	-	-	1	1	2
11498	-	-	+	11519	-	-	+	1	1	2

11525	11525	11529	-	11547	11543	11547	+	1	1	2
11551 -	-		-	12150 -	-		+	1	1	2
11598	11597	11600	-	11925 -	-		-	2	0	2
11605 -	-		-	12150 -	-		+	1	1	2
11605 -	-		+	11732 -	-		+	0	2	2
11642	11641	11642	-	13228 -	-		-	1	1	2
11681	11681	11684	-	13040 -	-		+	0	2	2
11687 -	-		+	11863 -	-		+	1	1	2
11715 -	-		+	14051 -	-		+	0	2	2
11719 -	-		-	14046 -	-		-	0	2	2
11753 -	-		-	12147 -	-		+	0	2	2
11804	11801	11804	-	11989	11987	11989	-	1	1	2
11822	11821	11826	-	14034 -	-		-	2	0	2
11836	11835	11836	+	11866 -	-		+	1	1	2
11848	11848	11850	-	11934 -	-		-	0	2	2
11874 -	-		+	12801 -	-		+	0	2	2
11878	11878	11880	-	11983 -	-		-	1	1	2
11900	11898	11900	-	13336 -	-		-	0	2	2
11911 -	-		-	14519 -	-		+	0	2	2
11932	11929	11932	+	11953 -	-		-	0	2	2
11949	11949	11950	+	11986	11986	11987	-	1	1	2
11987	11986	11987	+	12275 -	-		-	2	0	2
11994 -	-		-	12021 -	-		-	0	2	2
12002 -	-		+	12667 -	-		+	1	1	2
12045	12045	12046	-	12980 -	-		-	1	1	2
12068 -	-		+	12142 -	-		+	2	0	2
12093 -	-		+	12195 -	-		+	1	1	2
12094 -	-		-	12570 -	-		-	1	1	2
12101	12101	12105	-	12338 -	-		-	0	2	2
12112	12108	12113	-	13042 -	-		-	1	1	2
12124	12120	12127	+	15096 -	-		+	0	2	2
12145	12145	12149	+	12267 -	-		+	1	1	2
12146	12142	12149	-	14365 -	-		+	1	1	2
12159 -	-		-	12227 -	-		+	1	1	2
12168 -	-		-	12218 -	-		+	1	1	2
12176 -	-		-	12257 -	-		-	1	1	2
12234	12230	12234	-	12367 -	-		+	2	0	2
12247	12246	12251	-	14369 -	-		+	0	2	2
12258 -	-		+	14435 -	-		-	2	0	2
12267 -	-		-	12776 -	-		-	2	0	2
12277	12274	12281	-	12508	12506	12508	-	2	0	2
12282	12282	12284	+	12322 -	-		-	2	0	2
12289	12289	12291	+	12879 -	-		+	2	0	2
12310 -	-		-	14247 -	-		+	1	1	2
12324 -	-		+	14573 -	-		+	2	0	2
12331	12330	12331	-	14566 -	-		-	1	1	2
12367	12364	12367	-	13552 -	-		-	0	2	2
12390	12386	12390	+	14194	14192	14194	-	1	1	2
12431	12431	12432	-	14161 -	-		-	1	1	2
12438	12438	12440	-	12462 -	-		+	2	0	2
12439	12435	12439	+	14179 -	-		+	2	0	2
12447	12446	12450	+	13286 -	-		+	2	0	2
12454 -	-		-	12703 -	-		-	1	1	2
12460	12457	12464	-	12438 -	-		+	2	0	2
12474	12474	12476	-	12696 -	-		+	0	2	2
12474	12474	12476	-	13679 -	-		+	0	2	2
12501 -	-		+	12792 -	-		+	1	1	2
12502	12502	12507	-	13453	13451	13453	+	1	1	2
12530	12530	12531	+	12607	12607	12608	+	1	1	2
12549 -	-		+	14556 -	-		+	0	2	2
12591	12589	12594	-	14089	14089	14090	+	1	1	2
12603	12600	12603	-	14094 -	-		-	2	0	2
12603	12600	12603	-	14490 -	-		+	2	0	2
12611	12607	12611	-	14242 -	-		-	2	0	2
12625	12625	12629	+	13760 -	-		+	1	1	2
12660	12657	12661	-	13007 -	-		-	0	2	2
12697	12696	12697	-	12949 -	-		-	0	2	2
12704 -	-		-	12854 -	-		-	1	1	2
12716	12715	12721	-	13580 -	-		-	0	2	2
12797	12796	12797	-	13804 -	-		-	1	1	2
12813 -	-		-	12931 -	-		-	0	2	2
12928 -	-		-	13968 -	-		+	0	2	2
12956	12955	12956	+	13248 -	-		+	1	1	2
12956	12955	12956	+	14043 -	-		-	0	2	2
12979 -	-		+	13095 -	-		+	2	0	2
13057	13057	13058	+	13940 -	-		+	2	0	2
13057	13057	13058	+	15090 -	-		+	0	2	2
13104	13101	13104	-	14814 -	-		+	1	1	2
13117	13117	13118	-	13644	13644	13645	+	1	1	2
13126 -	-		-	13796 -	-		-	2	0	2
13148 -	-		+	14834 -	-		+	1	1	2
13178	13177	13178	-	14369 -	-		+	0	2	2
13234 -	-		-	13471 -	-		-	1	1	2
13240 -	-		-	13414 -	-		-	1	1	2
13291	13291	13294	-	13908 -	-		+	0	2	2
13300	13296	13300	-	15177 -	-		+	0	2	2
13307	13307	13313	-	15185	15185	15186	-	1	1	2
13328	13328	13331	+	14436 -	-		+	0	2	2

13354	-	-	-	13725	-	-	+	2	0	2
13361	13357	13364	-	13722	-	-	+	0	2	2
13361	13357	13364	-	14408	-	-	-	2	0	2
13384	-	-	-	13474	-	-	-	0	2	2
13395	13395	13396	-	13792	-	-	+	2	0	2
13395	13395	13396	-	14305	-	-	-	2	0	2
13395	13395	13396	-	14525	-	-	+	1	1	2
13400	-	-	+	14187	-	-	+	2	0	2
13412	13412	13413	-	13772	-	-	-	1	1	2
13426	-	-	-	14487	-	-	+	2	0	2
13476	-	-	+	13819	-	-	+	0	2	2
13503	13501	13503	-	14169	-	-	-	1	1	2
13508	13508	13509	+	13523	-	-	-	0	2	2
13526	-	-	-	13781	-	-	-	0	2	2
13538	-	-	+	13685	-	-	+	0	2	2
13550	-	-	-	14994	-	-	+	0	2	2
13603	13603	13605	+	14761	14759	14761	+	0	2	2
13616	-	-	-	14846	-	-	-	1	1	2
13655	13655	13659	-	13793	-	-	-	2	0	2
13668	13664	13672	-	13757	-	-	-	1	1	2
13709	13709	13710	-	14148	14148	14149	-	1	1	2
13722	13720	13727	-	14297	-	-	-	1	1	2
13730	13730	13733	-	13933	-	-	-	2	0	2
13730	13730	13733	-	14551	-	-	-	0	2	2
13730	-	-	+	13910	-	-	+	1	1	2
13759	13755	13763	-	13729	-	-	+	2	0	2
13772	13768	13772	+	13871	-	-	+	1	1	2
13773	-	-	-	13916	-	-	-	2	0	2
13779	13779	13782	+	13728	-	-	-	0	2	2
13780	13776	13780	-	14409	-	-	+	2	0	2
13801	13799	13805	-	13927	-	-	-	1	1	2
13801	13800	13805	+	14384	-	-	+	2	0	2
13811	13807	13815	-	14701	-	-	+	0	2	2
13811	13807	13815	-	14702	14698	14702	-	2	0	2
13819	13819	13823	+	13840	-	-	+	0	2	2
13819	13819	13823	+	14702	-	-	+	0	2	2
13827	13826	13827	+	14701	-	-	+	0	2	2
13833	13830	13837	-	14145	-	-	-	0	2	2
13833	13830	13837	-	14731	-	-	-	1	1	2
13851	13848	13851	-	14676	-	-	+	0	2	2
13855	13855	13856	+	14964	-	-	+	0	2	2
13872	13871	13876	-	14567	-	-	-	0	2	2
13872	13871	13876	-	14641	-	-	-	2	0	2
13875	13875	13878	+	13902	-	-	-	2	0	2
13875	13875	13878	+	14335	-	-	+	1	1	2
13875	13875	13878	+	14563	-	-	+	2	0	2
13879	13879	13882	-	14815	14815	14816	-	1	1	2
13882	13879	13883	+	14622	-	-	+	2	0	2
13882	13879	13883	+	14897	-	-	-	1	1	2
13899	-	-	-	14017	-	-	-	0	2	2
13929	13929	13931	+	13958	-	-	+	1	1	2
13930	13927	13933	-	14031	-	-	-	2	0	2
13940	13936	13943	-	14058	-	-	-	1	1	2
13947	-	-	-	14569	-	-	-	0	2	2
13966	13962	13966	-	14337	14333	14337	+	1	1	2
13968	-	-	+	15098	-	-	+	0	2	2
13977	13977	13981	+	13960	-	-	-	1	1	2
14011	14009	14014	-	14067	-	-	-	1	1	2
14020	14019	14025	-	14769	-	-	+	2	0	2
14024	14020	14025	+	14794	14790	14794	+	2	0	2
14031	14027	14035	+	14776	-	-	-	0	2	2
14039	14038	14041	-	14586	-	-	-	2	0	2
14039	14039	14040	+	14290	-	-	-	2	0	2
14054	14051	14058	-	14332	-	-	+	1	1	2
14068	14067	14072	+	14096	14096	14097	+	1	1	2
14068	14067	14072	+	14385	-	-	+	1	1	2
14082	14078	14082	+	14115	-	-	+	0	2	2
14091	14089	14091	-	14347	-	-	-	0	2	2
14106	14106	14109	-	15271	-	-	+	0	2	2
14139	14138	14142	-	14168	-	-	-	0	2	2
14159	14157	14166	-	14581	14581	14582	+	1	1	2
14184	14180	14184	+	14210	-	-	-	2	0	2
14210	-	-	+	14182	-	-	-	0	2	2
14215	-	-	+	14705	-	-	+	0	2	2
14227	-	-	+	14319	-	-	+	1	1	2
14251	-	-	-	14256	-	-	+	1	1	2
14252	14250	14252	+	14266	-	-	+	1	1	2
14291	14287	14292	-	14322	-	-	+	1	1	2
14314	-	-	+	14337	-	-	+	1	1	2
14328	-	-	+	14354	-	-	+	1	1	2
14341	14341	14342	-	15107	15107	15108	+	1	1	2
14346	14346	14349	-	15328	-	-	-	1	1	2
14397	14394	14397	+	14506	14506	14507	+	1	1	2
14397	14394	14397	+	14695	-	-	+	1	1	2
14409	-	-	-	14563	-	-	-	2	0	2
14414	14414	14416	-	14568	-	-	-	0	2	2
14422	14421	14424	+	14493	-	-	+	1	1	2
14422	14421	14424	+	14586	14586	14587	+	1	1	2

14435	14435	14438	-	14507	-	-	-	2	0	2
14456	14456	14458	-	14590	-	-	-	0	2	2
14456	14456	14458	-	14640	-	-	-	1	1	2
14503	14503	14504	+	14551	14551	14552	-	0	2	2
14513	14509	14513	-	14526	-	-	+	0	2	2
14519	14519	14522	-	14687	-	-	-	0	2	2
14545	-	-	+	14775	-	-	-	1	1	2
14561	14561	14565	-	15105	-	-	+	1	1	2
14578	14576	14580	-	14804	-	-	-	1	1	2
14605	14605	14608	+	14614	-	-	-	2	0	2
14674	-	-	-	14769	-	-	-	1	1	2
14731	-	-	-	14988	-	-	-	1	1	2
14744	-	-	-	14918	-	-	-	0	2	2
14747	14743	14747	+	14805	-	-	-	2	0	2
14758	14758	14762	+	14772	-	-	+	2	0	2
14758	14758	14762	+	14915	-	-	+	0	2	2
14781	14779	14781	-	15009	-	-	-	1	1	2
14781	14779	14781	-	15014	15014	15015	-	2	0	2
14804	14804	14808	+	15193	-	-	+	1	1	2
14812	14809	14812	-	14841	-	-	-	0	2	2
14836	-	-	-	15161	-	-	-	0	2	2
14905	14904	14908	-	14952	-	-	+	0	2	2
14921	-	-	+	14948	14948	14949	+	0	2	2
14933	14931	14933	+	14950	-	-	-	2	0	2
14951	14951	14952	-	15102	15102	15103	-	1	1	2
14951	14951	14952	-	15157	-	-	-	0	2	2
14970	14970	14973	-	15108	-	-	-	0	2	2
15007	15007	15010	-	15161	-	-	-	2	0	2
15007	15007	15010	-	15186	-	-	-	1	1	2
15008	-	-	+	15039	-	-	+	1	1	2
15058	15056	15059	+	15106	15103	15106	+	1	1	2
15121	-	-	+	15180	-	-	+	0	2	2
15143	15139	15143	+	15234	-	-	+	2	0	2
15165	15161	15170	+	15177	15177	15178	+	1	1	2
15165	15161	15170	+	15190	15188	15190	+	1	1	2
15165	15161	15170	+	15222	-	-	+	2	0	2
15172	-	-	+	15187	-	-	+	2	0	2
15177	15175	15177	+	15293	-	-	+	2	0	2
15189	15187	15190	+	15199	15199	15200	+	1	1	2
15189	15187	15190	+	15297	-	-	+	1	1	2
15192	-	-	-	15255	-	-	-	1	1	2
15204	15204	15205	-	15288	15288	15289	-	1	1	2
15204	15203	15205	+	15235	-	-	+	2	0	2
15217	-	-	+	15293	-	-	+	0	2	2
15220	-	-	-	15331	-	-	-	1	1	2
15275	-	-	-	15384	-	-	-	1	1	2
