# Supplementary material for: Identification and quantification of defective virus genomes in high throughput sequencing data using DVG-profiler, a novel post-sequence alignment processing algorithm
Source: PLoS One. 2019 May 17;14(5):e0216944. doi: 10.1371/journal.pone.0216944 (PMC6524942; doi:10.1371/journal.pone.0216944)
Supplement: S7 Table — (PDF) [file pone.0216944.s012.pdf]

Position (left)	Group start (left)	Group end (left)	Strandness (left)	Position (right)	Group start (right)	Group end (right)	Strandness (right)	Forward hits	Reverse hits	Fw and Rev
14869	14865	14872	-	15030	15026	15033	+	45190	65946	111136
14589	14584	14593	+	15046	15041	15050	+	33784	28786	62570
14947	14943	14953	-	15144	15140	15147	+	9692	6175	15867
14869	14865	14872	-	15023	15021	15029	-	9656	4596	14252
13308	13303	13312	-	14863	14862	14866	+	8745	10882	19627
14223	14219	14227	-	15165	15163	15168	+	8538	15424	23962
13616	13613	13619	-	14663	14662	14666	+	4844	5505	10349
13908	13906	13912	-	15277	15276	15280	+	2897	3969	6866
14456	14452	14461	-	14885	14882	14888	+	2716	2717	5433
14342	14339	14346	-	15107	15106	15110	+	2711	2571	5282
14464	14462	14468	-	14891	14887	14893	-	1752	1119	2871
14730	14726	14733	-	15025	15022	15028	+	1723	1854	3577
14456	14452	14461	-	14877	14874	14880	-	1671	866	2537
12591	12589	12594	-	14836	14834	14839	+	1648	1356	3004
13479	13475	13483	-	15064	15063	15067	+	1626	1691	3317
14238	14235	14243	-	15025	15022	15027	+	1357	2041	3398
14456	14452	14461	-	14882	14882	14886	-	1232	889	2121
14666	14661	14671	-	14777	14773	14780	+	1210	1825	3035
14960	14955	14964	+	15166	15162	15170	+	1193	822	2015
13629	13624	13635	-	13775	13774	13779	+	1189	1876	3065
13562	13559	13567	-	14651	14650	14654	+	1186	1716	2902
14331	14328	14337	-	14428	14425	14432	+	1139	782	1921
13332	13328	13336	-	14154	14154	14157	+	1074	1174	2248
13141	13138	13145	-	14880	14877	14882	+	1032	419	1451
13316	13313	13322	-	15143	15143	15145	+	1018	1063	2081
13055	13053	13058	-	13442	13439	13447	+	1012	1206	2218
14847	14842	14851	-	15025	15022	15030	-	929	568	1497
13751	13748	13755	-	14324	14323	14326	+	871	1009	1880
14869	14865	14872	-	15036	15034	15040	+	862	1198	2060
14350	14347	14353	-	14770	14767	14773	+	832	1181	2013
14360	14355	14365	-	14873	14872	14874	+	830	543	1373
13916	13915	13920	+	15284	15283	15288	+	828	1181	2009
13431	13430	13436	-	13720	13717	13723	+	815	642	1457
14761	14759	14764	-	14917	14916	14920	+	796	475	1271
13326	13324	13327	-	13837	13837	13841	+	728	962	1690
11435	11431	11439	-	14306	14305	14308	+	675	611	1286
13462	13458	13467	-	13684	13683	13686	+	671	504	1175
13610	13607	13611	-	14660	-	-	-	664	857	1521
11435	11431	11439	-	15162	15162	15164	+	589	523	1112
13479	13475	13483	-	14952	14950	14954	+	500	423	923
6250	6249	6251	+	14476	14475	14478	+	497	168	665
13549	13548	13553	-	14514	14514	14516	+	484	1207	1691
15025	15021	15028	+	15048	15048	15052	-	479	49	528
5785	5784	5789	+	15175	-	-	+	448	631	1079
12911	12906	12914	-	15044	15041	15046	+	360	472	832
13347	13343	13351	-	15239	15239	15241	+	359	302	661
12903	12900	12905	-	14740	14736	14741	+	330	277	607
14916	14915	14918	-	15043	15043	15048	-	318	4	322
14108	14106	14112	-	14207	14207	14211	+	312	199	511
15116	15112	15119	+	15143	15140	15146	-	311	6	317
11823	11822	11824	-	13780	13779	13781	+	302	581	883
13498	13496	13504	-	14739	14739	14741	+	292	442	734
14877	14873	14879	-	15046	15043	15048	-	283	936	1219
14730	14726	14733	-	15025	15024	15030	-	273	130	403
13498	13496	13504	-	14633	14633	14634	+	251	299	550
11228	11226	11231	-	14777	14774	14779	+	250	213	463
14485	14482	14492	-	14899	14896	14900	-	225	126	351
14869	14865	14872	-	15052	15050	15052	-	214	297	511
13549	13548	13553	-	14559	14559	14562	+	212	360	572
4176	4175	4179	+	13740	13739	13743	+	201	289	490
12921	12917	12924	-	14968	14968	14971	+	193	389	582
14869	14865	14872	-	15015	15014	15019	-	186	67	253
13562	13559	13567	-	14677	14676	14677	+	181	234	415
14370	14365	14373	+	14885	14881	14888	+	181	115	296
12101	12100	12106	-	12332	12329	12333	+	180	193	373
13431	13430	13436	-	13706	13705	13708	-	177	14	191
14951	14949	14954	+	15157	15155	15160	+	173	131	304
13462	13458	13467	-	13668	13668	13671	-	172	5	177
13863	13859	13866	-	14987	14987	14989	+	171	270	441
14164	14161	14166	-	14739	14737	14741	+	161	303	464
14859	14857	14862	-	15177	15177	15179	+	158	194	352
14443	14442	14447	-	15066	15066	15067	+	154	175	329
14704	14700	14708	-	14799	14799	14802	+	154	171	325
14947	14943	14953	-	15157	15156	15159	-	152	197	349
14370	14365	14373	+	14693	14693	14694	+	150	69	219
14725	14723	14725	-	15066	15066	15067	+	149	139	288
13967	13963	13968	-	15008	15008	15010	+	142	93	235
14485	14482	14492	-	14678	14677	14680	+	134	182	316
11857	11856	11858	-	15222	15221	15222	+	130	85	215
12481	12476	12482	-	14148	14148	14149	+	125	111	236
14456	14452	14461	-	15030	15029	15032	+	122	93	215
2426	2422	2429	+	13755	13755	13758	+	120	124	244
13265	13262	13269	-	13627	13625	13629	+	119	131	250

14462	14457	14466	+	14889	14885	14891	+	118	63	181
14786	14781	14789	-	15167	15167	15170	+	117	141	258
13359	13356	13363	-	15255	15252	15255	-	116	16	132
13587	13584	13591	-	14608	14608	14610	+	111	246	357
13326	13324	13327	-	13808	-	-	-	108	0	108
13252	13251	13256	-	15039	15037	15041	+	106	329	435
15092	15088	15096	+	15161	15160	15165	-	105	4	109
13555	13554	13558	-	14665	14665	14666	+	104	132	236
11446	11443	11450	+	15175	15171	15177	+	103	66	169
12121	12119	12123	-	14406	-	-	+	101	110	211
12797	12797	12800	-	14948	14944	14949	+	100	199	299
13916	13914	13916	-	15284	15284	15287	-	98	0	98
14905	14899	14908	-	15044	15040	15047	-	95	19	114
14666	14661	14671	-	14926	14923	14929	+	93	197	290
13921	13917	13925	-	15292	15288	15296	-	92	62	154
13751	13748	13755	-	14551	14551	14552	+	91	103	194
3198	3196	3199	+	12186	12185	12186	+	89	80	169
14947	14943	14953	-	15138	15136	15138	+	88	63	151
14307	14302	14308	-	14932	14932	14935	+	86	131	217
5033	5029	5034	+	12137	12136	12137	+	85	55	140
12291	12289	12293	-	14981	-	-	+	85	66	151
14464	14462	14468	-	14897	14895	14899	-	84	69	153
12553	12549	12557	-	14196	14196	14199	+	79	91	170
14518	14512	14521	-	14967	14966	14969	+	79	53	132
14905	14899	14908	-	14943	14943	14947	+	78	187	265
10847	10843	10847	-	13370	13370	13373	+	76	88	164
2131	2127	2136	+	10975	-	-	+	74	94	168
13770	13766	13775	-	14533	14532	14533	+	74	88	162
14928	14923	14933	+	15161	15157	15161	-	73	93	166
14767	14765	14771	-	14917	-	-	+	71	36	107
814	812	814	+	13432	13431	13432	+	69	131	200
14869	14865	14872	-	15043	15043	15046	+	69	61	130
13885	13880	13891	-	14370	14370	14373	+	68	105	173
13431	13430	13436	-	13697	13697	13699	-	67	9	76
13094	13091	13095	-	13469	13468	13471	+	65	61	126
14877	14873	14879	-	15191	15189	15194	-	65	146	211
13317	13315	13320	+	14879	14875	14883	+	64	46	110
13639	13637	13642	-	14454	14454	14455	+	64	78	142
14957	14956	14960	-	15157	15155	15159	-	62	27	89
82	78	83	+	97	96	98	-	61	34	95
1944	1941	1949	+	14652	14651	14652	+	61	14	75
13779	13776	13782	-	15033	15032	15033	+	61	59	120
14435	14432	14438	+	14457	-	-	-	61	0	61
14580	14579	14582	+	15051	15050	15052	-	61	169	230
14589	14584	14593	+	15042	15040	15042	-	61	40	101
15015	15010	15018	+	15038	15038	15042	-	60	14	74
1621	1619	1625	+	14102	-	-	-	59	40	99
13297	13293	13302	-	14334	14334	14335	+	59	26	85
14154	14150	14156	-	14377	14375	14381	+	59	87	146
12334	12332	12335	-	14577	14576	14577	+	58	22	80
15072	15068	15076	-	15151	15151	15155	-	58	5	63
1900	1896	1901	+	14927	-	-	+	57	49	106
11708	11706	11709	-	14207	14207	14208	+	57	22	79
12334	12332	12335	-	14793	14793	14796	+	57	60	117
13623	13620	13623	-	14671	-	-	-	57	0	57
12717	12713	12721	-	13372	13372	13375	+	56	81	137
13659	13655	13660	-	13793	13793	13794	-	56	2	58
97	94	97	+	82	-	-	-	55	27	82
14300	14297	14301	-	14435	-	-	-	55	27	82
2182	2179	2183	+	12375	12372	12375	+	54	38	92
13326	13324	13327	-	13772	13771	13772	-	54	28	82
14350	14347	14353	-	15110	15110	15112	-	54	46	100
14847	14842	14851	-	15039	15037	15040	-	54	35	89
5078	5074	5082	+	15083	15079	15087	-	53	14	67
2627	2625	2627	+	14993	14993	14995	+	52	39	91
9873	9871	9873	-	13952	13952	13953	+	52	68	120
11994	11992	11995	-	14467	14467	14468	+	52	69	121
12911	12906	12914	-	14036	-	-	+	52	32	84
14739	14734	14743	-	15041	15037	15044	-	52	19	71
14001	13997	14005	-	14693	14692	14693	+	51	38	89
14895	14890	14900	+	15030	15029	15032	+	51	60	111
2298	2294	2301	+	14177	14177	14178	+	50	56	106
13044	13040	13049	-	13609	-	-	+	50	61	111
14079	14077	14083	-	14866	14865	14866	+	50	29	79
14248	14245	14252	-	14289	-	-	+	50	44	94
14423	14419	14423	-	15021	-	-	+	50	32	82
14877	14873	14879	-	15039	15035	15041	-	50	233	283
13104	13102	13107	-	14941	-	-	+	49	43	92
14300	14297	14301	-	14446	14445	14447	+	49	58	107
12113	12109	12116	-	14450	-	-	+	48	40	88
13499	13495	13503	+	15080	-	-	+	48	40	88
14847	14842	14851	-	15044	15042	15045	+	48	26	74
14360	14355	14365	-	14880	14877	14881	-	47	34	81
7592	-	-	-	14561	-	-	+	46	47	93

8959	8955	8959	-	14807	14807	14811	+	45	32	77
13272	13270	13276	-	14359 -	-		+	45	56	101
14456	14452	14461	-	14791 -	-		-	45	31	76
13562	13559	13567	-	14638	14638	14639	-	44	105	149
2368	2366	2369	-	14409	14409	14411	+	43	50	93
11847	11844	11851	-	14698 -	-		+	43	36	79
14120	14114	14126	-	14464	14464	14467	+	43	36	79
14294	14291	14295	-	15180	15177	15180	-	43	65	108
14882	14880	14887	-	15042	15040	15046	-	43	59	102
8969	8969	8971	+	14797	14796	14797	-	42	6	48
11631	11628	11634	+	14357 -	-		+	42	56	98
13246	13241	13247	-	14247	14247	14249	+	42	34	76
13544	13538	13545	-	14509	14505	14512	-	42	163	205
13664	13661	13668	-	14187 -	-		-	42	0	42
14869	14865	14872	-	15078	15077	15082	-	42	42	84
1932	1927	1935	+	14681	14677	14681	+	41	11	52
3227	3227	3231	+	14501 -	-		+	41	47	88
4591	4588	4592	+	13645	13645	13646	+	41	25	66
10258	10256	10262	-	14175	14172	14175	+	41	19	60
12641	12639	12641	-	13605	13604	13605	+	41	19	60
13317	13315	13320	+	14872	14871	14873	+	41	19	60
8082	8082	8083	+	14811	14811	14812	+	40	13	53
12712	12708	12712	-	14403 -	-		+	40	53	93
13316	13313	13322	-	14875	14871	14879	-	40	34	74
13347	13343	13351	-	14273 -	-		+	40	45	85
13490	13486	13492	+	15071 -	-		+	40	32	72
14377	14377	14380	-	15012	15012	15013	+	40	26	66
14870	14866	14870	+	14895	14892	14895	-	40	6	46
14964	14962	14968	-	15154	15151	15155	-	40	44	84
4167	4167	4168	-	14590	14589	14590	+	39	17	56
14578	14576	14583	-	15051	15049	15051	+	39	20	59
10697	10694	10699	-	15008 -	-		+	38	31	69
14485	14482	14492	-	15249	15249	15250	+	38	46	84
13111	13109	13111	-	14370 -	-		+	37	28	65
13416	13413	13420	-	13665	13663	13669	+	37	18	55
14239	14238	14243	+	15153	15149	15153	-	37	32	69
13751	13748	13755	-	14330	14327	14333	+	36	29	65
14324	14320	14324	-	14837 -	-		+	36	49	85
14586	14586	14590	-	14647	14646	14647	+	36	17	53
12341	12341	12345	-	13947 -	-		+	35	24	59
13664	13661	13668	-	13798	13798	13800	-	35	123	158
8953	8950	8953	-	14813	14813	14814	+	34	0	34
12101	12100	12106	-	14401	14401	14403	+	34	59	93
12321	12318	12326	-	14584	14581	14585	+	34	15	49
14265	14263	14271	-	14408	14407	14412	+	34	40	74
14578	14576	14583	-	15153 -	-		+	34	52	86
2190	2187	2194	+	13081 -	-		+	33	16	49
13265	13262	13269	+	15052	15049	15053	+	33	20	53
13297	13293	13302	-	14255 -	-		+	33	19	52
13605	13602	13606	+	14675	14674	14678	-	33	18	51
13947	13941	13951	-	14134	14134	14137	+	33	15	48
14518	14512	14521	-	14929	14927	14929	+	33	13	46
8931 -	-		+	13497 -	-		+	32	54	86
12075	12070	12076	-	14560	14560	14564	+	32	41	73
13308	13303	13312	-	14360	14357	14360	+	32	22	54
13498	13496	13504	-	15080	15079	15082	-	32	10	42
13562	13559	13567	-	14648 -	-		-	32	51	83
14238	14235	14243	-	15015	15014	15015	-	32	14	46
14739	14734	14743	-	15036	15032	15036	-	32	13	45
1959	1953	1960	+	11517 -	-		+	31	14	45
2173	2173	2177	+	15240	15240	15241	-	31	54	85
2515	2514	2516	+	13987	13986	13987	+	31	23	54
13674	13669	13678	-	14672 -	-		-	31	16	47
14331	14328	14337	-	14593	14593	14594	+	31	18	49
14428	14428	14432	-	14464 -	-		+	31	0	31
11380	11380	11381	-	14896	14896	14897	+	30	34	64
14775	14775	14778	+	14931	14927	14932	+	30	41	71
14921	14920	14926	-	15048	15044	15052	-	30	185	215
15037	15036	15039	+	15049	15049	15052	-	30	17	47
991	988	991	+	14131 -	-		+	29	28	57
3166	3165	3169	+	13810	13810	13812	+	29	53	82
12605	12599	12607	-	13952	13952	13953	+	29	54	83
13664	13661	13668	-	14179	14178	14180	+	29	58	87
13709	13704	13713	-	14437	14437	14438	+	29	16	45
14252	14252	14257	+	15046	15042	15050	+	29	20	49
14331	14328	14337	-	14422	14418	14424	-	29	28	57
14877	14873	14879	-	15036	15030	15040	+	29	35	64
15037	15036	15039	+	15072	15068	15072	-	29	5	34
15047	15043	15048	+	15114	15113	15117	-	29	8	37
15185	15181	15190	+	15205	15201	15205	-	29	0	29
1959	1953	1960	+	13014	13013	13014	+	28	19	47
12583	12583	12586	-	14154	14151	14154	+	28	19	47
13316	13313	13322	-	14843	14843	14845	+	28	43	71
13317	13315	13320	+	14843	14843	14845	-	28	2	30

14207	14206	14211	+	15180	15176	15180	-	28	112	140
14423	14419	14423	-	15094	15093	15094	+	28	27	55
14565	14560	14568	-	15006	15005	15008	+	28	33	61
14877	14872	14882	+	14890	14890	14894	-	28	10	38
14905	14899	14908	-	14996	14995	15000	-	28	12	40
14957	14956	14960	-	15166	15162	15166	-	28	30	58
63	59	63	+	88	88	92	-	27	2	29
2941 -	-		-	2962 -	-		+	27	4	31
4181	4181	4183	+	13739 -	-		-	27	24	51
12956	12952	12958	-	14369	14367	14369	+	27	70	97
13416	13413	13420	-	14529 -	-		+	27	26	53
13639	13637	13642	-	13797	13794	13797	-	27	60	87
14248	14245	14252	-	15180	15178	15180	-	27	33	60
14854	14852	14856	-	15045	15042	15046	+	27	53	80
14864	14863	14864	-	15197 -	-		+	27	20	47
14869	14865	14872	-	15039	15039	15043	-	27	17	44
14877	14872	14882	+	15039	15039	15043	+	27	34	61
14928	14927	14928	-	15161	15161	15162	+	27	0	27
588	588	592	+	14791	14791	14793	+	26	13	39
12481	12476	12482	-	14136	14136	14138	+	26	31	57
13578	13574	13583	-	14604	14601	14608	-	26	29	55
13674	13669	13678	-	14421 -	-		+	26	56	82
13741	13737	13744	-	14730	14729	14732	+	26	22	48
14877	14873	14879	-	15094	15093	15096	+	26	25	51
10278	10276	10282	-	13627 -	-		+	25	14	39
12161	12159	12162	-	14564	14563	14565	+	25	61	86
13385	13380	13388	-	14384	14384	14385	+	25	30	55
13533	13528	13536	-	14457	14454	14457	+	25	32	57
14019	14017	14021	-	14758 -	-		+	25	15	40
14607	14603	14610	-	14967	14966	14967	+	25	32	57
13187	13183	13190	-	14498	14498	14502	+	24	11	35
14174	14170	14180	-	14752	14752	14755	+	24	39	63
14780	14780	14783	+	14929 -	-		+	24	0	24
700	698	703	+	756 -	-		-	23	13	36
7581 -	-		+	13545 -	-		+	23	11	34
11276	11273	11277	-	15023	15023	15026	+	23	34	57
12113	12109	12116	-	14591 -	-		+	23	33	56
12433	12431	12436	-	14077 -	-		+	23	31	54
12978	12973	12978	+	14053 -	-		+	23	16	39
13326	13324	13327	-	14880	14878	14880	-	23	1	24
13479	13475	13483	-	14933	14932	14933	+	23	15	38
13623	13620	13623	-	14680 -	-		-	23	11	34
13936	13932	13940	-	14949 -	-		+	23	25	48
14693	14688	14697	-	14852	14851	14854	+	23	17	40
14739	14735	14743	+	15041	15037	15041	+	23	12	35
14847	14842	14851	-	15020 -	-		-	23	9	32
14905	14899	14908	-	15050 -	-		-	23	9	32
14933	14931	14936	-	15078	15078	15081	-	23	150	173
15062	15059	15068	+	15078	15076	15081	-	23	6	29
15217	15214	15220	+	15222 -	-		+	23	8	31
12577	12573	12581	-	14136 -	-		+	22	29	51
12896	12895	12898	-	13079 -	-		+	22	25	47
13394	13389	13397	-	14177 -	-		+	22	16	38
13659	13655	13660	-	14465 -	-		+	22	36	58
13741	13737	13744	-	14239	14238	14240	+	22	7	29
13876	13872	13879	-	14379	14379	14381	+	22	37	59
14079	14077	14083	-	14613	14613	14614	+	22	28	50
14324	14320	14324	-	14989	14987	14989	+	22	19	41
14377	14377	14380	-	14687	14687	14689	+	22	6	28
15143	15140	15148	+	15116	15112	15119	-	22	164	186
2298	2294	2301	+	14730	14730	14731	+	21	39	60
4602	4597	4605	+	14104	14103	14105	+	21	5	26
12240	12235	12244	-	14660	14659	14663	+	21	15	36
13365	13365	13368	-	14536	14533	14536	+	21	15	36
13728	13726	13732	+	13779 -	-		-	21	5	26
14120	14114	14126	-	14216	14216	14219	-	21	22	43
14651	14650	14654	+	14679	14675	14679	-	21	3	24
14847	14842	14851	-	15013	15013	15014	-	21	15	36
15102	15098	15105	+	15145 -	-		-	21	0	21
15111	15106	15111	+	15152	15148	15152	-	21	4	25
15212	15210	15212	+	15236 -	-		-	21	1	22
123	119	124	+	150	150	154	-	20	12	32
4602	4597	4605	+	14370 -	-		+	20	10	30
8409	8405	8410	+	14247	14247	14248	+	20	8	28
13147	13146	13151	-	14624 -	-		+	20	7	27
13972	13972	13973	-	14385 -	-		+	20	18	38
14014	14009	14017	+	14704 -	-		+	20	3	23
14589	14584	14593	+	15025 -	-		-	20	8	28
14847	14842	14851	-	14978	14975	14978	-	20	28	48
14869	14865	14872	-	15057 -	-		-	20	17	37
12481	12476	12482	-	14303 -	-		+	19	19	38
12520	12517	12522	-	15185	15185	15186	+	19	39	58
12717	12713	12721	-	13349 -	-		-	19	13	32
13147	13146	13151	-	13944	13941	13944	+	19	17	36

13566	13562	13569	+	15066	15066	15067	+	19	49	68
13610	13607	13611	-	14677	14677	14679	+	19	22	41
13810	13807	13811	-	14859 -	-		+	19	7	26
13908	13906	13912	-	15116 -	-		+	19	38	57
14370	14365	14373	+	14779	14779	14780	+	19	11	30
14559	14556	14559	-	15005 -	-		+	19	14	33
2962	2961	2962	-	2941 -	-		+	18	0	18
11164	11163	11164	-	15087 -	-		+	18	7	25
12240	12235	12244	-	14581	14580	14581	+	18	16	34
13055	13053	13058	-	13425	13424	13428	-	18	12	30
13720	13717	13722	-	13808	13808	13811	+	18	19	37
14019	14017	14021	-	14416	14416	14420	+	18	20	38
14344	14340	14348	+	14845	14845	14847	+	18	1	19
14435	14435	14438	-	14507 -	-		-	18	0	18
14565	14560	14568	-	15057	15057	15061	+	18	36	54
14877	14872	14882	+	15030	15029	15030	+	18	44	62
15143	15140	15148	+	15156 -	-		+	18	6	24
123	121	123	-	150	150	153	+	17	2	19
655	654	659	-	11857	11856	11857	+	17	2	19
714	712	716	+	742	741	742	-	17	12	29
7502	7497	7506	-	14361	14360	14366	+	17	31	48
12520	12517	12522	-	13948	13948	13951	+	17	20	37
12577	12573	12581	-	15186	15186	15189	+	17	35	52
12903	12900	12905	-	13743 -	-		+	17	16	33
13451	13448	13454	-	14714 -	-		+	17	3	20
13629	13624	13635	-	13792	13790	13796	-	17	28	45
13639	13637	13642	-	14676	14676	14679	-	17	8	25
13863	13859	13866	-	14992	14992	14996	-	17	7	24
13947	13941	13951	-	14378 -	-		+	17	17	34
14019	14017	14021	-	14285	14284	14289	+	17	16	33
14233	14231	14234	+	15158	15157	15160	-	17	13	30
14456	14452	14461	-	14836	14836	14837	+	17	19	36
14859	14857	14862	-	15033	15031	15033	+	17	31	48
764	761	767	+	14539	14536	14539	+	16	26	42
10406	10403	10410	-	13044	13041	13045	+	16	35	51
11446	11443	11450	+	14316	14316	14319	+	16	14	30
12113	12109	12116	-	14165 -	-		+	16	13	29
12199	12195	12201	-	14586 -	-		+	16	52	68
12577	12573	12581	-	14576 -	-		+	16	21	37
12732	12728	12734	-	15030 -	-		+	16	0	16
13408	13404	13411	-	14524 -	-		+	16	7	23
13533	13528	13536	-	14688	14688	14690	+	16	24	40
14456	14452	14461	-	14891	14888	14892	-	16	3	19
14805	14801	14808	+	14883	14879	14886	-	16	2	18
14847	14842	14851	-	15008	15004	15008	-	16	12	28
1801	1801	1805	+	13741	13741	13742	+	15	15	30
1972	1967	1976	+	13356	13355	13356	+	15	11	26
2875	2874	2875	+	14864 -	-		+	15	20	35
10842 -	-		-	13349 -	-		-	15	10	25
11830	11828	11834	-	13796	13796	13797	-	15	27	42
13332	13328	13336	-	13837	13834	13839	+	15	22	37
13462	13458	13467	-	14731 -	-		+	15	3	18
13488	13485	13490	-	14882 -	-		-	15	5	20
13578	13574	13583	-	14672	14671	14672	-	15	0	15
13751	13748	13755	-	14436 -	-		+	15	0	15
13893	13893	13895	-	14171	14170	14171	+	15	13	28
13916	13914	13916	-	15103 -	-		+	15	19	34
14504	14501	14508	-	14551 -	-		+	15	5	20
14612	14611	14617	-	15005 -	-		+	15	17	32
14683	14681	14686	-	14788	14785	14791	-	15	15	30
14730	14726	14733	-	15015	15014	15015	-	15	7	22
14761	14759	14764	-	15025 -	-		+	15	11	26
14859	14857	14862	-	15079	15079	15083	-	15	19	34
14864	14863	14864	-	15195	15194	15195	-	15	9	24
15076	15072	15079	+	15061	15061	15064	-	15	5	20
638 -	-		+	14180 -	-		+	14	13	27
1141	1138	1145	+	14676	14676	14680	+	14	19	33
1924	1922	1924	+	12988 -	-		+	14	10	24
2016	2012	2018	+	13347 -	-		+	14	6	20
2488	2486	2489	+	13768	13766	13768	+	14	9	23
4188	4188	4192	-	14567 -	-		+	14	8	22
10543	10541	10544	-	14634 -	-		+	14	17	31
11920	11916	11926	-	15159 -	-		+	14	12	26
12121	12119	12123	-	14436	14436	14438	+	14	15	29
13055	13053	13058	-	13338 -	-		-	14	9	23
13055	13053	13058	-	13397	13397	13401	-	14	6	20
13953	13952	13956	-	14284 -	-		+	14	8	22
14238	14235	14243	-	15170 -	-		-	14	33	47
14462	14457	14466	+	14428	14427	14429	-	14	0	14
14847	14844	14850	+	15044	15042	15045	-	14	41	55
14882	14880	14887	-	15055	15053	15056	-	14	16	30
14947	14943	14953	-	15133	15133	15134	+	14	20	34
15102	15098	15105	+	15150	15150	15151	-	14	0	14
97	94	97	+	57	56	57	-	13	1	14

1407	1407	1409	-	1450 -	-		+	13	22	35
2788	2785	2791	+	14254 -	-		+	13	5	18
3902 -	-		+	13550 -	-		+	13	8	21
5785	5784	5789	+	15179	15179	15180	-	13	0	13
7832	7830	7832	+	14504	14504	14505	+	13	10	23
12167	12166	12172	-	14707 -	-		+	13	6	19
12187	12183	12190	-	15120 -	-		+	13	13	26
12307	12303	12310	-	14268	14268	14272	+	13	17	30
12732	12728	12734	-	14540 -	-		+	13	18	31
13265	13262	13269	-	13565	13565	13567	+	13	20	33
13278	13277	13282	-	14035 -	-		+	13	29	42
13408	13404	13411	-	14027	14027	14028	+	13	6	19
13408	13404	13411	-	15217 -	-		+	13	0	13
13451	13448	13454	-	13736	13734	13738	-	13	1	14
13544	13538	13545	-	14647	14644	14647	-	13	0	13
13706 -	-		+	13730 -	-		-	13	1	14
13779	13776	13782	-	15204 -	-		+	13	12	25
14207	14207	14208	-	15174 -	-		+	13	0	13
14456	14452	14461	-	14897 -	-		-	13	30	43
14540	14535	14540	-	14640 -	-		-	13	1	14
14859	14857	14862	-	14932	14930	14932	+	13	9	22
14869	14865	14872	-	15045	15045	15047	-	13	16	29
14947	14943	14953	-	15092	15090	15096	-	13	6	19
14988	14987	14991	-	15156	15156	15157	-	13	7	20
1037	1033	1041	+	14351	14350	14352	+	12	7	19
2037	2034	2039	+	13095	13095	13098	+	12	11	23
2173	2173	2177	+	12826 -	-		+	12	3	15
2493	2491	2493	+	2600 -	-		+	12	11	23
2539	2539	2543	+	14293 -	-		+	12	4	16
2962	2961	2962	-	3010	3009	3010	+	12	0	12
12026	12023	12029	-	14369 -	-		+	12	17	29
12611	12609	12614	-	13431	13430	13431	+	12	8	20
12717	12713	12721	-	14704	14704	14705	+	12	18	30
13117 -	-		-	14665 -	-		+	12	11	23
13394	13389	13397	-	14764	14764	14765	+	12	9	21
13473	13469	13474	-	15040	15040	15042	-	12	18	30
13510	13506	13513	+	14747	14746	14749	+	12	15	27
13555	13554	13558	-	14570	14570	14571	-	12	22	34
13738	13733	13743	+	14324 -	-		+	12	17	29
14019	14017	14021	-	14332	14332	14333	+	12	19	31
14120	14114	14126	-	14229	14225	14231	-	12	11	23
14202	14201	14204	-	15128 -	-		+	12	12	24
14578	14576	14583	-	14891	14891	14892	+	12	7	19
14612	14611	14617	-	15061 -	-		-	12	4	16
14748	14744	14749	-	15187 -	-		+	12	7	19
14947	14943	14953	-	15138	15135	15139	-	12	15	27
15085	15080	15086	+	15152 -	-		-	12	0	12
15177	15173	15177	+	15201	15201	15205	-	12	0	12
15284	15282	15290	-	15384	15383	15384	-	12	10	22
193	192	194	+	15006	15005	15008	+	11	0	11
1621	1619	1625	+	1710	1708	1710	+	11	0	11
1951 -	-		+	14761 -	-		+	11	0	11
2526	2522	2528	+	13524	13524	13526	+	11	16	27
2915	2915	2916	-	14818	14817	14818	+	11	44	55
3448	3448	3449	+	14213	14212	14213	+	11	9	20
3731	3727	3731	+	13619	13618	13620	+	11	6	17
4003	4002	4007	+	14738 -	-		+	11	5	16
5069	5065	5070	+	15097	15092	15097	-	11	14	25
5773	5772	5773	-	15182	15182	15183	+	11	1	12
10406	10403	10410	-	14699	14697	14699	+	11	7	18
10485	10482	10486	-	14968 -	-		+	11	24	35
11551	11550	11554	-	14340 -	-		+	11	8	19
11950	11947	11950	-	14307 -	-		+	11	0	11
13055	13053	13058	-	14885 -	-		+	11	0	11
13227	13224	13230	-	13461	13458	13461	-	11	11	22
13347	13343	13351	-	14159 -	-		-	11	6	17
13372	13372	13375	-	13431	13430	13431	+	11	1	12
13431	13430	13436	-	14763	14763	14764	+	11	12	23
13462	13458	13467	-	13660 -	-		-	11	0	11
13462	13458	13467	-	14164 -	-		+	11	10	21
13479	13475	13483	-	14315	14314	14315	+	11	9	20
13720	13717	13722	-	14266	14265	14270	+	11	7	18
13731	13728	13734	-	15095 -	-		+	11	3	14
13770	13766	13775	-	14800	14800	14802	+	11	42	53
13848	13843	13850	-	15012 -	-		+	11	0	11
13870	13867	13871	-	14865	14864	14865	+	11	18	29
13878	13874	13882	+	15067 -	-		+	11	6	17
13947	13941	13951	-	14143	14143	14147	-	11	2	13
14248	14245	14252	-	15042 -	-		-	11	0	11
14350	14347	14353	-	14930	14930	14933	+	11	3	14
14443	14442	14447	-	15037 -	-		-	11	1	12
14462	14457	14466	+	14902	14900	14902	+	11	10	21
14666	14661	14671	-	14944	14943	14945	-	11	11	22
14676	14672	14680	-	15065	15065	15066	+	11	28	39

14725	14723	14725	-	15024	15024	15027	+	11	15	26
14780	14780	14783	+	14936	14936	14938	+	11	15	26
14869	14865	14872	-	14979	14978	14980	-	11	9	20
14877	14872	14882	+	15189	15185	15189	+	11	7	18
14974	14969	14978	-	15146	15143	15147	-	11	41	52
14978	14978	14982	+	15048 -	-		-	11	14	25
15037	15036	15039	+	15079	15079	15083	-	11	2	13
15042	15040	15042	+	15074 -	-		-	11	0	11
714	712	716	+	14673 -	-		+	10	9	19
748	748	749	+	708	707	708	-	10	9	19
1054	1050	1054	+	14631	14630	14631	+	10	2	12
2023	2022	2024	+	2055	2055	2056	-	10	12	22
4476	4476	4478	+	14874	14874	14875	+	10	4	14
8934	8932	8934	-	14491 -	-		+	10	10	20
9706	9706	9707	-	14517	14517	14518	+	10	5	15
10300 -	-		-	14955 -	-		+	10	7	17
10436	10434	10436	-	14777 -	-		+	10	14	24
11435	11431	11439	-	14301 -	-		-	10	11	21
11920	11916	11926	-	14297 -	-		+	10	12	22
12321	12318	12326	-	13681	13681	13682	+	10	3	13
12352	12350	12352	-	13701 -	-		+	10	11	21
12788	12788	12792	-	13973 -	-		+	10	16	26
12956	12952	12958	-	15077	15077	15078	+	10	49	59
13055	13053	13058	-	14566 -	-		+	10	3	13
13089	13087	13090	-	13708	13707	13708	+	10	8	18
13187	13183	13190	-	14991 -	-		+	10	8	18
13347	13343	13351	-	14531	14531	14532	+	10	13	23
13431	13430	13436	-	13714	13711	13715	-	10	2	12
13479	13475	13483	-	14537	14537	14539	+	10	14	24
13516	13511	13517	-	14715 -	-		+	10	21	31
13562	13559	13567	-	13963	13959	13963	+	10	11	21
13674	13669	13678	-	14575 -	-		+	10	15	25
13700	13697	13700	-	14148 -	-		+	10	6	16
13761	13757	13765	+	14337	14335	14337	+	10	19	29
13853	13851	13857	-	14255	14251	14255	+	10	14	24
13936	13932	13940	-	14042	14041	14042	-	10	8	18
14025	14023	14029	-	14355	14351	14355	+	10	9	19
14079	14077	14083	-	14605 -	-		-	10	8	18
14174	14170	14180	-	14815 -	-		+	10	6	16
14331	14328	14337	-	14454	14449	14454	-	10	12	22
14403	14399	14407	-	14787 -	-		-	10	0	10
14479	14474	14479	-	14923 -	-		+	10	5	15
14501	14496	14505	+	14689	14686	14689	+	10	6	16
14607	14603	14610	-	14844 -	-		+	10	0	10
14622	14619	14626	-	15043	15043	15044	+	10	30	40
14730	14726	14733	-	14866 -	-		-	10	1	11
14847	14842	14851	-	14955	14955	14956	+	10	13	23
14854	14852	14856	-	15025	15023	15027	-	10	7	17
14854	14852	14856	-	15117 -	-		-	10	8	18
15241	15241	15245	-	15275	15271	15275	+	10	7	17
266	263	270	-	14331	14331	14333	+	9	17	26
278	276	283	-	15107	15107	15108	+	9	2	11
424 -	-		+	14279 -	-		+	9	4	13
2055	2052	2056	+	2023	2022	2023	-	9	5	14
2397	2397	2401	+	11823	11823	11824	+	9	8	17
2553	2551	2554	+	14355	14355	14357	+	9	6	15
2565	2565	2566	+	14343	14343	14344	+	9	9	18
4300	4298	4300	+	13030	13028	13030	+	9	7	16
4434	4431	4438	-	14868	14868	14871	+	9	21	30
11602	11599	11605	-	13948	13948	13949	+	9	11	20
12321	12318	12326	-	14464 -	-		+	9	2	11
12433	12431	12436	-	15092 -	-		+	9	24	33
12495	12495	12496	-	13785	13785	13786	+	9	14	23
12605	12599	12607	-	13875	13872	13875	+	9	5	14
12605	12599	12607	-	14916	14915	14916	+	9	5	14
12641	12639	12641	-	14144 -	-		+	9	8	17
13308	13303	13312	-	14815	14814	14815	-	9	68	77
13308	13303	13312	-	14877	14875	14877	-	9	11	20
13308	13303	13312	-	15040 -	-		-	9	37	46
13462	13458	13467	-	13834	13834	13835	+	9	9	18
13988	13985	13989	-	14290	14287	14290	+	9	9	18
14019	14017	14021	-	14668 -	-		+	9	0	9
14035	14034	14036	-	14700 -	-		+	9	7	16
14085 -	-		-	14614 -	-		-	9	10	19
14207	14207	14208	-	15180 -	-		+	9	0	9
14260	14260	14261	-	14865 -	-		+	9	4	13
14364	14360	14364	+	14880 -	-		+	9	33	42
14375	14374	14379	+	14900 -	-		+	9	0	9
14704	14700	14708	-	14804	14804	14808	+	9	8	17
14739	14734	14743	-	15025 -	-		+	9	8	17
14739	14735	14743	+	15034	15034	15035	+	9	9	18
14761	14759	14764	-	14924	14922	14924	+	9	4	13
14810	14809	14811	+	14878	14877	14878	-	9	2	11
14847	14842	14851	-	15057	15055	15057	-	9	11	20

14869	14865	14872	-	15033	15032	15034	-	9	3	12
14877	14873	14879	-	15029	15025	15029	+	9	15	24
14877	14873	14879	-	15059	15059	15063	-	9	7	16
14905	14899	14908	-	14987	14983	14990	-	9	1	10
14910	14909	14913	-	15046	15043	15046	-	9	2	11
15047	15043	15048	+	15057	15053	15057	-	9	4	13
15076	15072	15079	+	15104	15103	15105	-	9	0	9
15085	15080	15086	+	15114	15114	15115	-	9	1	10
15143	15140	15148	+	15164	15164	15168	-	9	7	16
290	288	294	+	14672	14672	14674	+	8	5	13
893	893	897	+	924 -	-		-	8	9	17
1259 -	-		-	1415	1412	1418	-	8	0	8
2100	2097	2103	+	14034	14033	14034	+	8	3	11
2119	2117	2120	+	12674	12673	12676	+	8	4	12
2344	2341	2344	+	14686	14685	14686	+	8	2	10
3258	3255	3258	+	12979 -	-		+	8	7	15
3317	3313	3317	+	13295 -	-		+	8	2	10
3628	3628	3632	+	14710	14710	14711	+	8	8	16
4181	4181	4183	+	14575	14575	14576	-	8	184	192
4186	4185	4186	+	14570 -	-		-	8	0	8
7365 -	-		-	14283 -	-		+	8	13	21
10498	10496	10498	-	10515	10515	10517	+	8	3	11
10697	10694	10699	-	14876	14873	14876	+	8	56	64
10754	10752	10758	-	14993	14992	14993	+	8	3	11
11097	11092	11099	-	12640 -	-		+	8	12	20
11369	11369	11371	-	15253	15251	15253	+	8	2	10
11468	11464	11472	-	15077	15077	15080	+	8	11	19
11551	11550	11554	-	13311 -	-		+	8	0	8
11665	11664	11669	-	14676 -	-		+	8	5	13
11945 -	-		-	13364 -	-		+	8	6	14
12101	12100	12106	-	13635	13634	13635	+	8	10	18
12187	12183	12190	-	13620	13619	13620	+	8	6	14
12257	12255	12258	-	14846 -	-		+	8	8	16
12418	12418	12423	-	14487 -	-		+	8	2	10
12788	12788	12792	-	14401 -	-		+	8	2	10
13013	13009	13017	-	13229 -	-		+	8	6	14
13147	13146	13151	-	14631 -	-		+	8	3	11
13168	13162	13169	-	13881	13879	13881	+	8	5	13
13246	13241	13247	-	13526 -	-		+	8	14	22
13252	13251	13256	-	15025 -	-		-	8	8	16
13326	13324	13327	-	14437 -	-		+	8	14	22
13347	13343	13351	-	14688	14687	14688	+	8	6	14
13349	13348	13351	+	15243 -	-		+	8	5	13
13416	13413	13420	-	14013	14013	14015	+	8	4	12
13473	13469	13474	-	13684 -	-		+	8	6	14
13518	13516	13522	+	14753	14751	14754	+	8	5	13
13629	13624	13635	-	14688	14688	14692	-	8	5	13
13659	13655	13660	-	14663	14662	14663	+	8	7	15
13674	13669	13678	-	14193	14191	14193	-	8	47	55
13715 -	-		-	14678 -	-		+	8	7	15
13791	13789	13794	-	14566 -	-		+	8	5	13
13810	13807	13811	-	14578	14575	14578	+	8	5	13
13863	13859	13866	-	14332 -	-		+	8	4	12
13868	13864	13871	+	13890	13889	13891	-	8	1	9
13947	13941	13951	-	14634 -	-		+	8	7	15
13978	13977	13982	-	13985 -	-		+	8	0	8
14120	14114	14126	-	14221 -	-		-	8	9	17
14138	14133	14141	-	14428	14427	14428	+	8	5	13
14159	14157	14160	-	14388 -	-		-	8	0	8
14212	14211	14215	-	14581	14581	14584	+	8	5	13
14239	14238	14243	+	15171	15167	15173	+	8	7	15
14360	14355	14365	-	14447 -	-		+	8	5	13
14369	14366	14375	-	15007	15007	15008	+	8	8	16
14428	14428	14432	-	14499 -	-		+	8	4	12
14435	14435	14438	-	14690	14688	14690	+	8	7	15
14518	14512	14521	-	14914 -	-		+	8	5	13
14646	14645	14649	+	14683	14680	14683	-	8	1	9
14660	14655	14661	+	14668 -	-		-	8	0	8
14666	14661	14671	-	14818	14814	14818	-	8	5	13
14794	14792	14797	+	14818	14815	14819	-	8	0	8
14847	14842	14851	-	15099	15097	15102	+	8	6	14
14859	14857	14862	-	15183 -	-		-	8	1	9
14864	14860	14865	+	14898	14894	14898	-	8	1	9
14869	14865	14872	-	15091	15088	15094	-	8	1	9
14877	14872	14882	+	15200 -	-		+	8	0	8
14905	14899	14908	-	15028	15025	15028	-	8	0	8
15177	15173	15177	+	15181 -	-		-	8	0	8
15241	15240	15246	+	15275	15275	15276	-	8	0	8
150	149	150	-	123	123	124	+	7	1	8
150	146	150	+	123	123	127	-	7	3	10
714	712	716	+	14635 -	-		+	7	8	15
756	756	759	+	700 -	-		-	7	6	13
764	761	767	+	15033 -	-		+	7	15	22
969 -	-		+	13365 -	-		+	7	1	8

1141	1138	1145	+	14370	14368	14370	+	7	6	13
1972	1967	1976	+	14357 -	-		+	7	14	21
2016	2012	2018	+	14707	14706	14707	+	7	6	13
2055	2052	2056	+	14331 -	-		+	7	1	8
2455	2454	2459	+	14358 -	-		+	7	5	12
2473	2471	2477	+	14580	14580	14581	+	7	11	18
2560	2558	2561	+	12802 -	-		+	7	1	8
2583	2579	2583	+	12334 -	-		+	7	10	17
3317	3313	3317	+	14886 -	-		+	7	4	11
4031	4027	4031	+	12407	12406	12407	+	7	5	12
8066	8065	8066	-	14795	14794	14795	-	7	6	13
8744	8742	8746	+	13516	13514	13516	+	7	7	14
8990	8990	8993	-	14345 -	-		+	7	10	17
9165 -	-		+	13593 -	-		+	7	5	12
10823	10819	10827	-	13646 -	-		+	7	5	12
11195	11191	11198	-	14522 -	-		+	7	3	10
11435	11431	11439	-	14296 -	-		-	7	7	14
11576	11575	11578	-	14145	14145	14147	+	7	9	16
11602	11599	11605	-	14412 -	-		+	7	7	14
12094	12090	12094	-	13978 -	-		+	7	9	16
12187	12183	12190	-	14266	14265	14266	+	7	1	8
12321	12318	12326	-	13774 -	-		+	7	11	18
12407	12402	12411	-	14487 -	-		+	7	5	12
12433	12431	12436	-	13497 -	-		+	7	6	13
12445	12441	12450	-	14508 -	-		+	7	16	23
12611	12609	12614	-	14845	14845	14849	-	7	5	12
12851	12850	12854	-	14049 -	-		+	7	26	33
13265	13262	13269	-	13555 -	-		-	7	0	7
13297	13293	13302	-	14464 -	-		+	7	8	15
13308	13303	13312	-	14841	14837	14843	-	7	11	18
13316	13313	13322	-	13484 -	-		+	7	9	16
13330	13326	13336	+	15251 -	-		-	7	0	7
13462	13458	13467	-	14668 -	-		+	7	6	13
13555	13554	13558	-	14660 -	-		-	7	17	24
13581	13579	13585	+	14680	14679	14680	-	7	1	8
13644	13643	13648	-	14755 -	-		-	7	2	9
13709	13704	13713	-	14592 -	-		+	7	1	8
13759	13757	13760	-	14344 -	-		-	7	0	7
13776	13771	13780	+	13969	13969	13970	-	7	0	7
13776	13771	13780	+	14346	14343	14346	+	7	1	8
13876	13872	13879	-	14995	14995	14997	-	7	15	22
13908	13906	13912	-	15293	15291	15293	-	7	5	12
14065	14062	14069	-	14809 -	-		+	7	10	17
14108	14106	14112	-	14206	14203	14208	-	7	4	11
14130	14128	14130	-	14196 -	-		-	7	0	7
14193	14190	14196	-	15010 -	-		+	7	5	12
14207	14206	14211	+	15165 -	-		+	7	7	14
14223	14219	14227	-	15185 -	-		-	7	14	21
14252	14252	14257	+	15031	15031	15034	+	7	12	19
14284	14282	14289	-	14631	14631	14634	+	7	6	13
14370	14365	14373	+	14771	14770	14771	+	7	13	20
14403	14399	14407	-	15064	15064	15066	+	7	11	18
14456	14452	14461	-	14867	14864	14867	-	7	7	14
14485	14482	14492	-	14672	14671	14672	-	7	6	13
14666	14661	14671	-	14770	14770	14772	+	7	7	14
14676	14672	14679	+	14780	14780	14783	+	7	13	20
14704	14700	14708	-	15079	15075	15082	-	7	1	8
14775	14772	14780	-	14932	14929	14937	-	7	24	31
14786	14781	14789	-	15056	15056	15057	+	7	7	14
14869	14865	14872	-	15066	15063	15069	-	7	6	13
14905	14903	14907	+	14943 -	-		-	7	3	10
14957	14956	14960	-	15149	15146	15150	-	7	3	10
14974	14969	14978	-	15159	15159	15163	-	7	4	11
14999 -	-		+	15051 -	-		-	7	1	8
15025	15021	15028	+	15140	15138	15140	-	7	0	7
15032	15029	15034	+	15156 -	-		-	7	1	8
15062	15059	15068	+	15088	15088	15089	-	7	0	7
15085	15080	15086	+	15064	15061	15068	-	7	9	16
15085	15080	15086	+	15103	15103	15107	-	7	0	7
15160	15156	15163	+	15212	15210	15212	-	7	0	7
15192	15192	15195	+	15210 -	-		-	7	0	7
15346 -	-		+	15363 -	-		-	7	0	7
267	265	267	+	15120	15120	15121	-	6	1	7
448 -	-		+	14165	14164	14165	+	6	10	16
672	670	672	+	14154	14152	14154	+	6	5	11
714	712	716	+	15217 -	-		+	6	5	11
764	761	767	+	13872 -	-		+	6	1	7
802	801	804	+	14836 -	-		+	6	10	16
1852	1848	1856	+	13708 -	-		+	6	8	14
1959	1953	1960	+	14537	14536	14537	+	6	4	10
2023	2022	2024	+	13742 -	-		+	6	0	6
2455	2454	2459	+	12965	12965	12966	+	6	2	8
2526	2522	2528	+	13302 -	-		+	6	2	8
2680	2677	2684	+	12467 -	-		+	6	8	14

2718	2718	2722	-	2784	2781	2785	-	6	2	8
2891	2890	2895	+	14962	14961	14962	+	6	16	22
3023	-	-	+	13427	-	-	+	6	9	15
3106	-	-	+	13030	-	-	+	6	1	7
3154	3153	3156	+	14818	-	-	+	6	10	16
3214	3211	3218	+	14830	-	-	+	6	6	12
3485	-	-	+	13629	-	-	+	6	0	6
4805	4802	4806	+	14693	14689	14693	+	6	4	10
5078	-	-	-	15083	-	-	+	6	83	89
5408	5408	5411	+	13252	-	-	+	6	37	43
5476	5476	5477	-	15063	15062	15063	+	6	2	8
6257	6256	6261	+	14465	-	-	-	6	0	6
6936	-	-	+	15368	-	-	-	6	0	6
8733	-	-	-	13061	-	-	+	6	5	11
9355	9355	9359	-	14610	14606	14610	+	6	6	12
9562	9562	9564	-	14688	-	-	+	6	2	8
9618	9617	9619	-	14269	-	-	+	6	5	11
10543	10541	10544	-	14693	14692	14693	+	6	5	11
10739	10738	10742	-	14438	14438	14439	+	6	7	13
10885	10885	10886	-	14226	-	-	+	6	6	12
10894	10892	10898	-	15237	15237	15239	+	6	10	16
11117	11114	11117	-	13946	-	-	+	6	0	6
11170	11169	11174	-	14409	-	-	+	6	2	8
11183	11182	11186	-	13863	13863	13865	+	6	4	10
11522	11519	11523	-	14147	14146	14147	+	6	2	8
11665	11664	11669	-	13979	-	-	+	6	3	9
11836	11835	11839	-	14187	14186	14187	+	6	3	9
12101	12100	12106	-	14828	14828	14829	+	6	7	13
12433	12431	12436	-	13750	13750	13751	+	6	5	11
12433	12431	12436	-	14923	-	-	+	6	4	10
12577	12573	12581	-	13098	-	-	+	6	5	11
12591	12589	12594	-	15263	15263	15266	+	6	2	8
12739	12737	12743	-	15216	15216	15218	+	6	8	14
12833	12830	12836	-	13814	-	-	+	6	17	23
12903	12900	12905	-	13742	-	-	-	6	0	6
12940	12936	12945	-	13443	13442	13443	+	6	5	11
12973	12970	12979	-	14019	14019	14020	+	6	9	15
13071	13067	13076	+	13445	13445	13446	+	6	0	6
13147	13146	13151	-	14782	-	-	+	6	3	9
13199	13196	13203	-	13646	-	-	+	6	3	9
13347	13343	13351	-	14705	-	-	+	6	0	6
13451	13448	13454	-	13743	13740	13743	-	6	5	11
13490	13486	13492	+	15102	-	-	+	6	0	6
13518	13516	13522	+	14874	14873	14874	+	6	1	7
13523	13521	13525	-	14378	14377	14378	+	6	2	8
13569	13568	13570	-	14566	14566	14567	-	6	9	15
13569	13568	13570	-	14676	14675	14677	+	6	4	10
13674	13669	13678	-	14095	14095	14096	+	6	8	14
13674	13669	13678	-	15008	15007	15009	-	6	4	10
13700	13697	13700	-	14858	-	-	+	6	2	8
13700	13697	13700	-	15127	-	-	+	6	0	6
13728	13726	13732	+	13752	-	-	-	6	0	6
13770	13766	13775	-	14139	-	-	+	6	1	7
13799	13796	13804	-	14401	-	-	+	6	6	12
13876	13872	13879	-	15267	-	-	+	6	8	14
13921	13917	13925	-	14667	14666	14667	+	6	4	10
13936	13932	13940	-	14036	-	-	-	6	0	6
13988	13985	13989	-	15089	-	-	+	6	7	13
13992	13988	13992	+	15134	-	-	+	6	15	21
13994	13994	13995	-	14459	-	-	+	6	0	6
14012	14008	14012	-	14994	-	-	+	6	10	16
14164	14161	14166	-	14752	14751	14752	-	6	8	14
14254	14253	14257	-	14896	-	-	+	6	9	15
14265	14263	14271	-	14757	-	-	+	6	3	9
14284	14282	14289	-	14806	-	-	+	6	3	9
14294	14291	14295	-	14316	14314	14316	+	6	5	11
14296	14292	14299	+	14325	14322	14325	-	6	6	12
14316	14314	14318	-	14291	-	-	+	6	1	7
14350	14347	14353	-	15121	-	-	-	6	4	10
14360	14355	14365	-	14808	14807	14808	-	6	3	9
14360	14355	14365	-	15032	-	-	-	6	0	6
14423	14419	14423	-	14526	14524	14526	+	6	3	9
14452	14448	14455	+	14474	-	-	-	6	5	11
14456	14452	14461	-	14731	14728	14732	+	6	8	14
14456	14452	14461	-	15016	-	-	+	6	0	6
14586	14586	14590	-	14710	14709	14710	-	6	0	6
14605	14601	14609	+	15092	-	-	+	6	8	14
14739	14734	14743	-	14805	-	-	+	6	0	6
14739	14734	14743	-	15046	-	-	-	6	0	6
14739	14735	14743	+	15029	-	-	+	6	4	10
14818	14813	14818	+	14794	14792	14796	-	6	4	10
14818	14813	14818	+	14864	14864	14866	-	6	2	8
14854	14852	14856	-	15040	15037	15040	+	6	11	17
14864	14863	14864	-	15015	15015	15017	-	6	2	8

14864	14863	14864	-	15033	15029	15036	+	6	5	11
14928	14927	14928	-	15015 -	-		-	6	0	6
14974	14969	14978	-	15108 -	-		-	6	12	18
15004	15000	15009	+	15045	15045	15048	-	6	3	9
15062	15059	15068	+	15083	15083	15086	-	6	2	8
15085	15080	15086	+	15129 -	-		-	6	0	6
15102	15098	15105	+	15156	15156	15161	-	6	0	6
15111	15106	15111	+	15129	15129	15133	-	6	0	6
15124	15120	15126	+	15149	15149	15151	-	6	1	7
15185	15181	15190	+	15169	15169	15173	-	6	12	18
15192	15191	15192	-	15255 -	-		-	6	6	12
123	119	124	+	14644 -	-		+	5	4	9
242	242	243	+	12230	12230	12231	+	5	2	7
278	276	283	-	14436 -	-		-	5	0	5
544 -	-		+	14476 -	-		+	5	0	5
603	602	603	+	14571	14570	14571	+	5	1	6
700 -	-		-	756 -	-		+	5	3	8
802	801	804	+	14036 -	-		+	5	3	8
814	812	814	+	14252 -	-		+	5	0	5
838 -	-		+	9136 -	-		+	5	0	5
893	893	897	+	12913	12913	12914	+	5	2	7
893	893	897	+	14619 -	-		+	5	2	7
923	920	927	+	14993 -	-		+	5	4	9
1605 -	-		-	14118 -	-		+	5	12	17
1764	1763	1764	-	1787 -	-		+	5	3	8
1793	1792	1796	+	14508 -	-		+	5	0	5
2435	2431	2436	+	14077 -	-		+	5	1	6
2435	2431	2436	+	14836	14836	14837	+	5	6	11
2673 -	-		+	14049 -	-		+	5	5	10
2771	2768	2772	+	14187 -	-		+	5	3	8
2788	2785	2791	+	13741 -	-		+	5	0	5
3272	3271	3273	+	15092	15091	15092	+	5	1	6
3341	3340	3343	+	10939	10938	10939	+	5	3	8
3354 -	-		+	14297 -	-		+	5	2	7
4591	4588	4592	+	13837 -	-		+	5	4	9
4749	4748	4751	+	9165	9165	9166	+	5	7	12
4879	4877	4879	+	13742 -	-		+	5	3	8
5524	5524	5525	+	14680 -	-		+	5	0	5
7437	7434	7437	+	12478 -	-		+	5	4	9
8171	8167	8171	-	12404 -	-		+	5	6	11
8485	8481	8489	-	13380 -	-		+	5	5	10
9508 -	-		-	14631 -	-		+	5	1	6
10097	10096	10098	-	10211 -	-		-	5	0	5
10097	10096	10098	-	14708 -	-		+	5	4	9
10196 -	-		-	14783 -	-		+	5	17	22
10935 -	-		-	12743 -	-		+	5	2	7
10993	10991	10996	-	14166	14165	14166	+	5	4	9
11170	11169	11174	-	13773 -	-		+	5	0	5
11305	11305	11307	+	13957	13957	13959	+	5	0	5
11319 -	-		-	13175	13173	13175	+	5	4	9
11551	11550	11554	-	14557 -	-		+	5	8	13
11920	11916	11926	-	13741	13740	13741	+	5	4	9
11955	11955	11957	-	12726 -	-		-	5	0	5
11979	11975	11979	-	14056 -	-		+	5	9	14
11994	11992	11995	-	13581 -	-		+	5	5	10
12018	12016	12021	-	14294 -	-		+	5	0	5
12167	12166	12172	-	14376	14376	14378	+	5	6	11
12233	12230	12234	-	13346 -	-		+	5	4	9
12334	12332	12335	-	14259 -	-		+	5	6	11
12407	12402	12411	-	14314	14314	14315	+	5	7	12
12449	12448	12449	+	14100 -	-		+	5	0	5
12465	12461	12467	-	13703 -	-		+	5	3	8
12536	12535	12542	-	14835 -	-		+	5	12	17
12568	12565	12569	-	14482 -	-		+	5	2	7
12616	12615	12620	-	14778 -	-		+	5	4	9
12616	12615	12620	-	14896 -	-		+	5	4	9
12676	12672	12678	-	13022 -	-		-	5	1	6
12732	12728	12734	-	14882 -	-		+	5	8	13
12812	12812	12816	+	12820 -	-		-	5	0	5
12911	12906	12914	-	13372	13371	13374	+	5	7	12
12973	12970	12979	-	14814	14814	14815	+	5	5	10
12983 -	-		+	14039 -	-		+	5	0	5
13000	12997	13002	-	15130 -	-		+	5	6	11
13055	13053	13058	-	13457 -	-		-	5	0	5
13055	13053	13058	-	14877 -	-		-	5	0	5
13141	13138	13145	-	14890	14886	14890	-	5	17	22
13168	13162	13169	-	14959 -	-		+	5	2	7
13316	13313	13322	-	13744 -	-		-	5	1	6
13408	13404	13411	-	14305 -	-		+	5	5	10
13408	13404	13411	-	14401	14400	14401	+	5	2	7
13425	13422	13425	+	13459	13459	13462	-	5	1	6
13431	13430	13436	-	13723 -	-		-	5	0	5
13431	13430	13436	-	14991 -	-		+	5	0	5
13456 -	-		-	14571 -	-		+	5	0	5

13462	13458	13467	-	14576	14575	14576	+	5	5	10
13473	13469	13474	-	14034	14034	14035	+	5	2	7
13478	13478	13479	+	13692	-	-	+	5	0	5
13498	13496	13504	-	13780	13778	13780	+	5	2	7
13561	13558	13561	+	14674	14674	14677	-	5	1	6
13581	13579	13585	+	14692	-	-	+	5	0	5
13616	13613	13619	-	14660	14656	14660	-	5	7	12
13629	13624	13635	-	14673	14672	14676	-	5	36	41
13639	13637	13642	-	14436	-	-	-	5	5	10
13674	13669	13678	-	14187	-	-	+	5	3	8
13684	13680	13687	-	14469	-	-	-	5	0	5
13695	13693	13695	-	14502	14500	14502	+	5	6	11
13720	13717	13722	-	14969	-	-	+	5	6	11
13791	13789	13794	-	14154	-	-	-	5	2	7
13826	13825	13831	-	14369	-	-	+	5	5	10
13826	13825	13831	-	15091	-	-	+	5	6	11
13848	13843	13850	-	14538	-	-	-	5	4	9
13851	13849	13856	+	14687	-	-	+	5	3	8
13885	13880	13891	-	14500	14500	14502	+	5	3	8
13978	13977	13982	-	14247	-	-	+	5	3	8
14090	14089	14094	-	14880	14880	14881	-	5	5	10
14187	14183	14189	-	15089	15087	15089	+	5	3	8
14202	14201	14204	-	14692	14692	14693	-	5	6	11
14265	14263	14271	-	14881	14880	14882	+	5	3	8
14324	14320	14326	+	14941	-	-	+	5	4	9
14342	14339	14346	-	14509	-	-	+	5	3	8
14360	14355	14365	-	14770	14766	14774	+	5	9	14
14369	14366	14375	-	14715	-	-	+	5	2	7
14435	14432	14438	+	15060	-	-	-	5	13	18
14495	14494	14499	-	14831	-	-	+	5	0	5
14518	14512	14521	-	15063	-	-	+	5	4	9
14526	14524	14530	-	14751	14751	14752	+	5	2	7
14527	14526	14527	+	14534	-	-	+	5	6	11
14565	14560	14568	-	15237	-	-	+	5	4	9
14570	14569	14573	-	14812	14811	14812	+	5	3	8
14586	14586	14590	-	14775	-	-	-	5	0	5
14589	14584	14593	+	14868	-	-	+	5	2	7
14589	14584	14593	+	15054	-	-	-	5	1	6
14607	14603	14610	-	15043	-	-	+	5	11	16
14633	14632	14636	-	15043	-	-	+	5	13	18
14676	14672	14680	-	14944	14940	14944	-	5	30	35
14676	14672	14679	+	14653	14651	14653	-	5	12	17
14676	14672	14679	+	14940	14940	14944	+	5	12	17
14725	14723	14725	-	15042	-	-	-	5	3	8
14730	14726	14733	-	15034	-	-	-	5	2	7
14739	14734	14743	-	15018	-	-	+	5	4	9
14739	14735	14743	+	15048	15046	15048	+	5	13	18
14761	14759	14764	-	15062	15061	15062	+	5	4	9
14761	14759	14764	-	15206	-	-	-	5	10	15
14769	14765	14770	+	14925	-	-	+	5	11	16
14826	14825	14827	+	14856	14856	14857	-	5	0	5
14854	14852	14856	-	15173	15170	15177	-	5	7	12
14864	14860	14865	+	14818	-	-	-	5	0	5
14869	14865	14872	-	15117	-	-	-	5	0	5
14869	14865	14872	-	15156	-	-	-	5	1	6
14882	14880	14887	-	15002	-	-	-	5	1	6
14885	14885	14888	+	14895	-	-	-	5	0	5
14894	14890	14897	-	15060	-	-	-	5	0	5
14895	14890	14900	+	15036	15036	15040	+	5	9	14
14941	14939	14942	-	15086	15085	15086	-	5	6	11
14947	14943	14953	-	15063	-	-	-	5	1	6
14981	14979	14983	-	15055	-	-	-	5	6	11
15032	15029	15034	+	15065	15064	15065	-	5	2	7
15062	15059	15068	+	15094	15093	15094	-	5	0	5
15062	15059	15068	+	15149	-	-	-	5	0	5
15124	15120	15126	+	15141	15137	15141	-	5	1	6
15177	15173	15177	+	15189	15189	15190	+	5	1	6
15205	15200	15208	+	15185	15185	15189	-	5	33	38
242	242	243	+	13587	-	-	+	4	1	5
495	495	499	+	14781	14781	14782	+	4	8	12
544	-	-	+	15142	-	-	+	4	8	12
646	646	649	-	14882	14881	14882	+	4	0	4
655	654	659	-	14868	14868	14871	+	4	1	5
756	756	759	-	700	-	-	+	4	0	4
802	801	804	+	14763	14763	14764	+	4	13	17
826	825	826	+	13552	13551	13552	+	4	5	9
935	932	939	+	14350	14350	14353	+	4	0	4
962	-	-	+	13775	-	-	+	4	1	5
1054	1050	1054	+	12723	-	-	+	4	5	9
1054	1050	1054	+	14083	-	-	+	4	4	8
1089	1088	1092	+	1139	1136	1142	+	4	4	8
1099	1095	1101	+	14730	-	-	+	4	1	5
1184	1184	1185	+	14732	-	-	+	4	1	5
1227	1227	1230	-	2225	-	-	-	4	4	8

1641	1638	1641	+	1696 -	-		-	4	0	4
1869 -	-		-	14439 -	-		-	4	0	4
1886	1883	1891	+	12830	12830	12831	+	4	2	6
1900	1896	1901	+	12599 -	-		+	4	1	5
1900	1896	1901	+	14097	14095	14097	+	4	3	7
1910	1908	1911	-	15363	15362	15363	-	4	64	68
2016	2012	2018	+	12493	12491	12493	+	4	1	5
2075	2072	2076	+	14298	14294	14298	+	4	10	14
2119	2117	2120	+	14887 -	-		+	4	0	4
2131	2127	2136	+	4160 -	-		+	4	8	12
2190	2187	2194	+	2430	2426	2430	+	4	2	6
2249	2248	2251	+	13852 -	-		+	4	0	4
2252	2252	2256	-	14654 -	-		-	4	4	8
2344	2341	2344	+	12230	12229	12230	+	4	1	5
2473	2471	2477	+	13435	13435	13436	+	4	6	10
2479 -	-		+	14725 -	-		+	4	0	4
2652	2651	2652	+	13872	13871	13872	+	4	1	5
2680	2677	2684	+	14580	14580	14581	+	4	4	8
2788	2785	2791	+	11028	11027	11028	+	4	2	6
2861	2858	2862	+	14207 -	-		+	4	1	5
2904	2900	2907	+	14828	14825	14831	-	4	13	17
3184	3183	3188	+	13667 -	-		+	4	11	15
3436 -	-		-	3483 -	-		+	4	1	5
3480 -	-		+	13764 -	-		+	4	4	8
3577	3577	3581	+	12380 -	-		+	4	2	6
3628	3628	3632	+	10240 -	-		+	4	7	11
3661	3659	3661	+	14400 -	-		+	4	3	7
3860 -	-		-	4033 -	-		-	4	4	8
3860	3859	3864	+	13229	13229	13230	+	4	3	7
3860	3859	3864	+	13346 -	-		+	4	2	6
4402 -	-		+	11164 -	-		+	4	2	6
4457	4455	4460	+	13199 -	-		+	4	1	5
4602	4597	4605	+	15290	15290	15291	+	4	8	12
4610	4610	4611	+	10982 -	-		+	4	2	6
4874 -	-		+	14300 -	-		+	4	0	4
5629 -	-		+	9901 -	-		+	4	0	4
5941 -	-		+	12439	12439	12442	+	4	2	6
6556	6554	6556	-	6633 -	-		-	4	0	4
7239	7238	7243	-	11548 -	-		+	4	0	4
7423	7423	7427	-	12488	12488	12489	+	4	1	5
8177	8177	8179	+	8422 -	-		-	4	0	4
8188 -	-		+	14292	14292	14293	+	4	1	5
8355 -	-		-	15071 -	-		+	4	1	5
8953	8950	8953	-	14798 -	-		+	4	3	7
8998	8996	8999	-	14163	14163	14165	+	4	8	12
9078	9078	9080	-	14137 -	-		+	4	4	8
9107	9107	9110	-	14732	14729	14732	+	4	1	5
9273	9270	9273	-	12377 -	-		+	4	0	4
9859	9858	9860	-	14634	14633	14634	+	4	1	5
9905 -	-		+	11549 -	-		+	4	0	4
10031	10031	10032	-	14139 -	-		+	4	2	6
10089	10089	10092	-	13769 -	-		+	4	1	5
10097	10096	10098	-	14480 -	-		+	4	0	4
10163	10159	10163	-	10299	10297	10299	-	4	4	8
10370	10366	10373	-	14993 -	-		+	4	1	5
10512	10509	10514	-	15031 -	-		+	4	5	9
10543	10541	10544	-	13773 -	-		+	4	2	6
10657 -	-		-	12468 -	-		+	4	0	4
10739	10738	10742	-	13553 -	-		+	4	2	6
10855	10855	10858	-	11856 -	-		+	4	3	7
11127	11127	11128	-	14758 -	-		+	4	6	10
11145	11141	11146	-	14248 -	-		+	4	3	7
11247	11244	11247	+	14786 -	-		+	4	3	7
11440 -	-		+	14349 -	-		+	4	0	4
11451	11450	11456	-	14331	14331	14335	-	4	2	6
11510	11506	11514	-	13385 -	-		+	4	0	4
11774	11769	11774	-	13901 -	-		+	4	8	12
11842 -	-		+	13831 -	-		+	4	0	4
11866	11863	11867	-	13794 -	-		-	4	9	13
11873	11871	11874	-	12956 -	-		+	4	5	9
11895	11895	11898	-	13791 -	-		-	4	6	10
11914	11911	11914	-	13930	13929	13930	+	4	3	7
11920	11916	11926	-	13266	13265	13266	+	4	2	6
11979	11975	11979	-	13521 -	-		+	4	6	10
12018	12016	12021	-	14306 -	-		+	4	1	5
12053	12052	12057	-	14379	14377	14379	+	4	2	6
12139	12136	12141	-	14352	14350	14352	+	4	3	7
12161	12159	12162	-	13835 -	-		+	4	3	7
12176	12176	12180	-	13385 -	-		+	4	0	4
12199	12195	12201	-	14463	14463	14465	+	4	8	12
12252	12252	12253	-	14046	14046	14047	+	4	4	8
12257	12255	12258	-	13276 -	-		+	4	5	9
12269	12269	12270	-	15128 -	-		+	4	5	9
12291	12289	12293	-	13679 -	-		+	4	1	5

12364	12360	12365	-	14108	-	-	+	4	2	6
12547	-	-	-	14238	-	-	+	4	12	16
12577	12573	12581	-	13163	13163	13165	+	4	12	16
12641	12639	12641	-	14908	14907	14908	+	4	1	5
12652	12652	12656	-	14307	-	-	+	4	1	5
12692	12692	12694	+	14608	-	-	+	4	1	5
12732	12728	12734	-	15004	-	-	+	4	12	16
12759	12757	12761	-	14619	-	-	+	4	2	6
12775	12771	12780	-	13014	-	-	+	4	1	5
12775	12771	12780	-	13284	13284	13285	+	4	6	10
12775	12771	12780	-	14056	14055	14056	+	4	2	6
12884	12881	12888	-	13559	-	-	+	4	2	6
12973	12970	12979	-	14298	-	-	+	4	4	8
12983	12980	12988	-	13262	-	-	+	4	2	6
13008	13007	13008	-	13063	-	-	+	4	2	6
13025	13023	13029	-	14024	-	-	+	4	8	12
13102	13099	13105	+	13717	13714	13717	+	4	2	6
13141	13138	13145	-	14318	-	-	+	4	0	4
13141	13138	13145	-	14870	-	-	+	4	3	7
13252	13251	13256	-	14211	-	-	+	4	2	6
13265	13262	13269	-	14163	-	-	+	4	1	5
13265	13262	13269	-	14599	-	-	+	4	6	10
13308	13303	13312	-	14344	-	-	-	4	4	8
13332	13328	13336	-	14140	-	-	-	4	2	6
13332	13328	13336	-	14801	-	-	+	4	4	8
13332	13328	13336	-	15185	15185	15188	+	4	5	9
13340	13337	13341	-	15131	15131	15132	+	4	15	19
13347	13343	13351	-	14838	-	-	+	4	0	4
13347	13343	13351	-	15243	-	-	-	4	0	4
13365	13365	13368	-	13911	13911	13912	+	4	0	4
13385	13380	13388	-	14165	14163	14165	-	4	0	4
13408	13404	13411	-	14344	-	-	+	4	8	12
13416	13413	13420	-	14836	-	-	+	4	1	5
13446	13443	13446	-	13738	13737	13738	-	4	2	6
13451	13448	13454	-	14779	-	-	+	4	3	7
13462	13458	13467	-	13772	-	-	-	4	1	5
13462	13458	13467	-	14355	-	-	+	4	1	5
13473	13469	13474	-	14532	-	-	+	4	4	8
13488	13485	13490	-	15071	-	-	-	4	4	8
13498	13496	13504	-	13768	-	-	+	4	0	4
13508	13507	13510	-	13836	13836	13839	+	4	2	6
13523	13521	13525	-	14575	14575	14576	+	4	5	9
13544	13538	13545	-	14007	14007	14008	+	4	5	9
13551	13548	13555	+	14663	-	-	-	4	7	11
13578	13574	13583	-	14569	14569	14574	-	4	3	7
13581	13579	13585	+	14676	14676	14679	+	4	11	15
13587	13584	13591	-	14265	14264	14265	+	4	2	6
13629	13624	13635	-	14144	-	-	+	4	1	5
13629	13624	13635	-	14212	14212	14213	+	4	4	8
13629	13628	13631	+	14741	-	-	+	4	0	4
13639	13637	13642	-	14463	14463	14464	-	4	0	4
13674	13669	13678	-	14647	14645	14647	-	4	3	7
13674	13669	13678	-	15003	15003	15004	-	4	0	4
13709	13704	13713	-	14532	-	-	+	4	3	7
13728	13726	13732	+	13758	-	-	-	4	2	6
13741	13737	13744	-	13863	-	-	-	4	0	4
13741	13737	13744	-	14836	-	-	+	4	1	5
13761	13757	13765	+	14361	14358	14361	+	4	0	4
13776	13771	13780	+	14351	14351	14355	+	4	13	17
13779	13776	13782	-	15025	15025	15027	-	4	4	8
13791	13789	13794	-	15020	-	-	+	4	5	9
13799	13796	13804	-	15050	15049	15050	-	4	2	6
13810	13807	13811	-	15203	15201	15204	+	4	2	6
13841	13839	13843	+	13859	-	-	-	4	0	4
13870	13867	13871	-	14994	14990	14994	-	4	20	24
13947	13941	13951	-	14667	-	-	+	4	2	6
13967	13963	13968	-	14488	-	-	+	4	3	7
13978	13977	13982	-	14751	14750	14751	+	4	1	5
13988	13985	13989	-	15128	15128	15129	-	4	3	7
13994	13994	13995	-	14615	-	-	+	4	0	4
14008	-	-	+	14318	-	-	-	4	0	4
14014	14009	14017	+	14310	-	-	-	4	0	4
14035	14034	14036	-	14437	-	-	-	4	0	4
14065	14062	14069	-	14752	-	-	+	4	5	9
14120	14114	14126	-	14211	14211	14212	-	4	5	9
14154	14150	14156	-	14197	14197	14198	-	4	9	13
14200	14200	14201	+	14227	-	-	-	4	0	4
14229	14228	14233	-	15164	15164	15168	-	4	4	8
14248	14245	14252	-	14712	-	-	+	4	0	4
14248	14245	14252	-	15031	-	-	-	4	0	4
14254	14253	14257	-	14266	14265	14266	+	4	15	19
14254	14253	14257	-	15175	-	-	-	4	4	8
14276	14276	14277	+	14344	-	-	-	4	0	4
14284	14282	14289	-	14895	-	-	+	4	4	8

14316	14314	14318	-	14933 -	-		-	4	0	4
14331	14328	14337	-	14464	14463	14466	-	4	2	6
14331	14328	14337	-	14836	14836	14837	+	4	8	12
14331	14328	14337	-	14841	14837	14841	-	4	5	9
14342	14339	14346	-	14467	14465	14467	-	4	25	29
14350	14347	14353	-	14732	14731	14732	+	4	3	7
14350	14347	14353	-	14889 -	-		+	4	1	5
14358	14353	14358	+	14369	14368	14369	-	4	0	4
14358	14353	14358	+	15091 -	-		-	4	9	13
14360	14355	14365	-	14432	14432	14436	+	4	7	11
14360	14355	14365	-	14708	14708	14711	+	4	4	8
14360	14355	14365	-	14777	14777	14780	-	4	14	18
14360	14355	14365	-	14892	14892	14893	-	4	1	5
14360	14355	14365	-	15015	15012	15015	+	4	1	5
14370	14365	14373	+	14759 -	-		-	4	0	4
14370	14365	14373	+	14876	14876	14877	+	4	0	4
14370	14365	14373	+	15081	15081	15084	-	4	7	11
14387	14384	14392	+	14846	14844	14846	-	4	1	5
14391	14390	14393	-	14844	14841	14844	+	4	0	4
14403	14399	14407	-	14864 -	-		+	4	2	6
14423	14419	14423	-	14941 -	-		+	4	6	10
14428	14424	14429	+	15052 -	-		-	4	3	7
14462	14457	14466	+	14493 -	-		-	4	0	4
14470	14469	14473	-	14895	14895	14899	-	4	4	8
14470	14469	14475	+	14895 -	-		+	4	4	8
14479	14474	14479	-	14978	14978	14980	+	4	9	13
14485	14482	14492	-	14638	14637	14638	+	4	2	6
14485	14482	14492	-	14660	14660	14664	-	4	0	4
14487	14484	14491	+	14500 -	-		-	4	0	4
14501	14496	14505	+	14551 -	-		-	4	2	6
14504	14501	14508	-	14852 -	-		+	4	0	4
14511	14510	14511	-	14916	14916	14917	-	4	5	9
14547	14545	14550	-	14752 -	-		+	4	5	9
14551	14551	14554	+	14504	14501	14504	-	4	1	5
14559	14556	14559	-	14617 -	-		+	4	0	4
14565	14560	14568	-	14637 -	-		+	4	2	6
14578	14576	14583	-	14885 -	-		-	4	0	4
14612	14611	14617	-	15043	15043	15046	+	4	7	11
14622	14619	14626	-	15033 -	-		-	4	0	4
14660	14655	14661	+	14926 -	-		+	4	3	7
14666	14661	14671	-	14913 -	-		-	4	12	16
14676	14672	14680	-	15092 -	-		+	4	15	19
14706	14705	14706	+	14882 -	-		-	4	1	5
14730	14726	14733	-	14782 -	-		-	4	0	4
14818	14813	14818	+	14801	14798	14803	-	4	9	13
14818	14813	14818	+	15030 -	-		+	4	7	11
14854	14852	14856	-	15057 -	-		-	4	2	6
14854	14852	14856	-	15180	15180	15184	+	4	0	4
14869	14865	14872	-	15099 -	-		-	4	0	4
14869	14865	14872	-	15200	15196	15200	-	4	6	10
14882	14880	14887	-	15102	15102	15105	-	4	1	5
14894	14890	14897	-	15018	15018	15019	-	4	0	4
14895	14890	14900	+	14877	14875	14877	-	4	13	17
14905	14899	14908	-	15033	15033	15035	-	4	1	5
14922	14919	14922	+	14941	14939	14941	+	4	3	7
14967	14966	14968	+	15042 -	-		-	4	0	4
14973	14969	14975	+	15179	15176	15179	+	4	5	9
14973	14969	14975	+	15193	15190	15193	+	4	0	4
14974	14969	14978	-	15094 -	-		-	4	0	4
15004	15000	15009	+	15040	15037	15040	+	4	0	4
15030	15028	15032	-	15030 -	-		+	4	3	7
15042	15040	15042	+	15064	15064	15065	-	4	0	4
15072	15068	15076	-	15174	15171	15174	-	4	1	5
15076	15072	15079	+	15092	15090	15092	-	4	0	4
15085	15080	15086	+	15058	15054	15059	-	4	3	7
15124	15120	15126	+	15161 -	-		-	4	0	4
15138	15135	15138	+	15160	15159	15160	-	4	2	6
15192	15192	15195	+	15219 -	-		-	4	2	6
15275	15273	15275	+	15241	15241	15243	-	4	15	19
1 -	-		-	14984 -	-		+	3	1	4
157	154	158	+	173	173	174	-	3	8	11
228	228	230	+	14850 -	-		+	3	1	4
365	363	367	+	15143 -	-		+	3	2	5
534	534	536	+	14677	14676	14677	+	3	1	4
684	682	686	+	13320 -	-		+	3	1	4
700	698	703	+	14808 -	-		+	3	0	3
707	707	708	-	748	748	749	+	3	1	4
884	880	884	+	14384	14383	14384	+	3	1	4
957	957	959	+	14409 -	-		+	3	2	5
998 -	-		+	13700 -	-		+	3	1	4
1422	1421	1422	+	13835 -	-		+	3	0	3
1460	1460	1463	+	1442 -	-		-	3	1	4
1637	1633	1637	-	1667 -	-		-	3	0	3
1869	1869	1870	+	12231 -	-		+	3	0	3

1869	1869	1870	+	12238 -	-		-	3	0	3
1886	1883	1891	+	13986	13983	13986	+	3	2	5
1900	1896	1901	+	3316 -	-		-	3	2	5
1932	1927	1935	+	13126	13123	13126	+	3	1	4
1932	1927	1935	+	13451	13451	13454	+	3	1	4
1944	1941	1949	+	12598 -	-		+	3	1	4
1982	1982	1983	+	11612 -	-		+	3	2	5
2006	2006	2010	+	12482 -	-		+	3	0	3
2037	2034	2039	+	13774	13770	13774	+	3	2	5
2037	2034	2039	+	14895 -	-		+	3	0	3
2075	2072	2076	+	13322 -	-		+	3	2	5
2075	2072	2076	+	14000	14000	14002	+	3	4	7
2168	2165	2168	+	13142	13139	13142	+	3	5	8
2219 -	-		-	2434 -	-		-	3	3	6
2244	2243	2244	-	14348 -	-		+	3	1	4
2244	2244	2246	+	13824	13824	13826	+	3	6	9
2298	2294	2301	+	11714 -	-		+	3	10	13
2325	2322	2325	+	13593 -	-		+	3	0	3
2364	2362	2364	+	13518	13516	13518	+	3	1	4
2386	2386	2389	+	12742 -	-		+	3	3	6
2386	2386	2389	+	13199 -	-		+	3	2	5
2408	2405	2409	+	13747 -	-		+	3	0	3
2426	2422	2429	+	12727 -	-		+	3	2	5
2435	2431	2436	+	13835 -	-		+	3	1	4
2443 -	-		-	2466 -	-		+	3	1	4
2444	2443	2444	+	14198 -	-		+	3	0	3
2466 -	-		-	2443 -	-		+	3	0	3
2493	2491	2493	+	12966	12966	12967	+	3	3	6
2546 -	-		+	12872 -	-		+	3	2	5
2641	2639	2645	+	12270	12270	12271	+	3	3	6
2771	2768	2772	+	13423 -	-		+	3	3	6
2801	2799	2802	+	9953	9951	9953	+	3	1	4
2837	2836	2839	+	13871	13871	13872	+	3	3	6
2891	2890	2895	+	13851 -	-		+	3	0	3
2897	2896	2897	+	14968 -	-		+	3	0	3
3003 -	-		+	3032 -	-		+	3	0	3
3118	3115	3118	+	14787	14784	14787	+	3	1	4
3184	3183	3188	+	14300 -	-		+	3	3	6
3227	3227	3231	+	13613 -	-		+	3	1	4
3273	3273	3275	-	3247 -	-		+	3	0	3
3273	3273	3275	-	3373	3373	3377	-	3	0	3
3317	3313	3317	+	14731 -	-		+	3	2	5
3341	3340	3343	+	8675 -	-		+	3	3	6
3386	3386	3387	+	13346 -	-		+	3	5	8
3419	3418	3421	+	8850 -	-		+	3	0	3
3429 -	-		+	12909 -	-		+	3	2	5
3468	3468	3469	+	13819 -	-		+	3	3	6
3500	3498	3501	+	14503	14503	14504	+	3	5	8
3541 -	-		+	11659 -	-		+	3	1	4
3673	3669	3673	+	3946 -	-		-	3	0	3
3731	3727	3731	+	14837	14836	14837	+	3	5	8
3821	3821	3824	+	13985 -	-		+	3	2	5
3830 -	-		+	12957	12955	12957	+	3	0	3
3841 -	-		+	13261 -	-		+	3	2	5
4003	4002	4007	+	14180 -	-		+	3	0	3
4003	4002	4007	+	14550 -	-		+	3	0	3
4146	4145	4146	+	13404 -	-		+	3	5	8
4157 -	-		+	13385 -	-		+	3	2	5
4186	4185	4186	+	13734	13733	13734	-	3	2	5
4258	4256	4258	+	14266 -	-		+	3	1	4
4330	4330	4333	+	14068 -	-		+	3	2	5
4471	4469	4471	+	12613 -	-		+	3	0	3
4894 -	-		-	4919 -	-		+	3	0	3
4921	4921	4922	+	14438	14437	14438	+	3	1	4
5045 -	-		+	13043 -	-		+	3	1	4
5408 -	-		-	15039 -	-		+	3	33	36
5729	5729	5730	+	15186	15186	15187	-	3	3	6
5827	5827	5828	+	14773 -	-		+	3	5	8
6037	6033	6037	-	14814 -	-		+	3	4	7
6257	6256	6261	+	14485 -	-		+	3	0	3
6441 -	-		+	13161	13161	13162	+	3	2	5
6449	6448	6449	-	12578	12578	12579	+	3	1	4
6753	6751	6757	+	15141 -	-		+	3	4	7
7492	7492	7495	-	12069 -	-		+	3	0	3
7597 -	-		-	7846 -	-		-	3	3	6
7607 -	-		-	9517 -	-		+	3	9	12
8140 -	-		-	8236 -	-		-	3	0	3
8411 -	-		-	15031 -	-		+	3	2	5
8465	8463	8465	-	13679 -	-		-	3	0	3
8531 -	-		-	13094 -	-		+	3	2	5
8656 -	-		+	14272 -	-		+	3	7	10
9065	9061	9065	-	15012	15008	15012	+	3	1	4
9078	9078	9080	-	13451	13451	13452	+	3	3	6
9147 -	-		-	14783 -	-		+	3	0	3

9154	9151	9157	-	15021	15021	15024	+	3	5	8
9217	9213	9220	+	12709	-	-	+	3	0	3
9273	9270	9273	-	14986	-	-	+	3	4	7
9355	9353	9355	+	12979	-	-	+	3	3	6
9414	9412	9414	-	14581	-	-	+	3	1	4
9562	9562	9564	-	14371	-	-	+	3	4	7
9618	9617	9619	-	15122	-	-	+	3	6	9
9831	-	-	-	12064	-	-	+	3	0	3
9836	9836	9838	-	12269	-	-	+	3	3	6
9878	9877	9878	-	14777	-	-	+	3	0	3
9905	9902	9905	-	11549	-	-	-	3	2	5
9932	9929	9932	-	14409	-	-	+	3	0	3
9984	9984	9988	-	15025	-	-	+	3	2	5
10011	10008	10013	-	14020	14019	14020	+	3	2	5
10019	10017	10019	-	14594	14594	14595	+	3	0	3
10036	-	-	+	13350	-	-	+	3	3	6
10055	10054	10055	-	11858	-	-	+	3	0	3
10076	10073	10078	-	14369	-	-	+	3	2	5
10131	10129	10133	-	13416	-	-	+	3	1	4
10131	10129	10133	-	14578	-	-	+	3	4	7
10210	-	-	-	15069	-	-	+	3	1	4
10258	10256	10262	-	14518	14517	14518	+	3	1	4
10651	10648	10651	+	15251	-	-	+	3	0	3
10724	10723	10725	+	14848	-	-	-	3	0	3
10725	10723	10726	-	14847	14846	14848	+	3	6	9
10955	10952	10956	-	14282	-	-	+	3	4	7
11028	11027	11032	-	14120	-	-	+	3	2	5
11139	11138	11139	-	13300	-	-	+	3	2	5
11156	11152	11156	-	13148	-	-	+	3	0	3
11258	11258	11261	-	14261	-	-	+	3	3	6
11276	11273	11277	-	12187	-	-	+	3	9	12
11281	-	-	-	13968	-	-	+	3	1	4
11361	11357	11361	+	13692	13692	13696	-	3	1	4
11395	11392	11395	+	11729	-	-	-	3	0	3
11429	-	-	-	13808	-	-	+	3	0	3
11435	11431	11439	-	14425	14422	14425	-	3	8	11
11446	11443	11447	-	14757	14756	14757	+	3	2	5
11446	11443	11450	+	15167	-	-	+	3	0	3
11451	11450	11456	-	12382	-	-	+	3	0	3
11453	11453	11456	+	14315	14311	14315	+	3	2	5
11510	11506	11514	-	13913	-	-	+	3	1	4
11534	-	-	-	12144	-	-	+	3	2	5
11576	11575	11578	-	11667	11666	11667	-	3	0	3
11610	11607	11610	-	13962	-	-	-	3	0	3
11617	11614	11622	-	14281	-	-	+	3	3	6
11641	11637	11644	-	13692	-	-	+	3	7	10
11648	11647	11652	-	14615	-	-	+	3	5	8
11696	11693	11699	-	13523	-	-	+	3	4	7
11696	11693	11699	-	14456	-	-	+	3	0	3
11708	11706	11709	-	14360	-	-	+	3	5	8
11713	11712	11715	-	13578	-	-	+	3	0	3
11733	11730	11736	-	13780	-	-	+	3	5	8
11733	11730	11736	-	13794	-	-	-	3	0	3
11830	11828	11834	-	14202	-	-	+	3	3	6
11836	11835	11839	-	13054	-	-	+	3	0	3
11895	11895	11898	-	12052	-	-	-	3	0	3
11895	11895	11898	-	14695	-	-	+	3	7	10
11914	11911	11914	-	12305	-	-	+	3	0	3
11914	11911	11914	-	12503	-	-	-	3	0	3
11950	11947	11950	-	12717	12717	12720	+	3	6	9
11964	11964	11967	-	15024	-	-	+	3	0	3
12026	12023	12029	-	12534	-	-	+	3	0	3
12032	12032	12035	-	13967	13967	13970	+	3	4	7
12040	12037	12044	-	13779	-	-	+	3	3	6
12061	12059	12063	-	13346	-	-	+	3	1	4
12061	12059	12063	-	14964	-	-	+	3	2	5
12069	12065	12069	-	14057	14056	14057	+	3	5	8
12085	12085	12086	-	14730	-	-	+	3	2	5
12088	12088	12090	+	15165	-	-	-	3	0	3
12097	-	-	+	14598	-	-	+	3	0	3
12147	12142	12151	-	13793	13793	13794	-	3	3	6
12147	12142	12151	-	15251	-	-	+	3	0	3
12208	12208	12209	+	14618	-	-	+	3	0	3
12227	12225	12228	-	13753	13753	13756	+	3	4	7
12240	12235	12244	-	13267	-	-	+	3	0	3
12240	12235	12244	-	13770	-	-	+	3	6	9
12246	12246	12250	-	14689	-	-	+	3	3	6
12269	12269	12270	-	13989	-	-	+	3	0	3
12282	12279	12282	-	12980	-	-	-	3	2	5
12291	12289	12293	-	14992	-	-	-	3	3	6
12321	12318	12326	-	13781	-	-	+	3	0	3
12407	12402	12411	-	15044	-	-	+	3	3	6
12433	12431	12436	-	13841	-	-	+	3	1	4
12445	12441	12450	-	13810	-	-	+	3	3	6

12465	12461	12467	-	14570	-	-	+	3	9	12
12465	12461	12467	-	14913	14913	14914	+	3	5	8
12495	12495	12496	-	13772	-	-	-	3	1	4
12501	12501	12502	-	14524	14524	14525	+	3	1	4
12508	12506	12509	-	14103	-	-	+	3	3	6
12520	12517	12522	-	13959	-	-	-	3	0	3
12536	12535	12542	-	13439	-	-	+	3	1	4
12536	12535	12542	-	15196	-	-	-	3	5	8
12568	12565	12569	-	14211	14209	14211	-	3	6	9
12591	12589	12594	-	14823	-	-	-	3	0	3
12605	12599	12607	-	13972	-	-	+	3	1	4
12605	12599	12607	-	14711	-	-	+	3	2	5
12625	12623	12625	+	13760	-	-	+	3	0	3
12635	12634	12635	+	12953	-	-	-	3	0	3
12641	12639	12641	-	12843	-	-	-	3	0	3
12641	12639	12641	-	12908	12908	12909	+	3	4	7
12652	12652	12656	-	13850	-	-	+	3	4	7
12683	12683	12687	-	13605	-	-	+	3	0	3
12692	12692	12694	+	14861	14861	14862	+	3	2	5
12717	12713	12721	-	13365	13364	13365	+	3	2	5
12717	12713	12721	-	13812	-	-	+	3	0	3
12726	12726	12730	+	13832	13828	13832	+	3	3	6
12739	12737	12743	-	13696	-	-	+	3	10	13
12759	12757	12761	-	13972	13972	13974	+	3	5	8
12775	12771	12780	-	14178	-	-	+	3	1	4
12788	12788	12792	-	13345	-	-	+	3	0	3
12864	12864	12869	-	13478	13478	13479	+	3	3	6
12903	12900	12905	-	13523	-	-	+	3	3	6
12911	12906	12914	-	13422	-	-	+	3	3	6
12911	12906	12914	-	13697	-	-	+	3	0	3
12931	12930	12935	+	15152	15149	15152	+	3	2	5
12947	12944	12949	+	15129	-	-	+	3	0	3
12965	12965	12968	-	14757	-	-	+	3	6	9
12973	12970	12979	-	14559	14558	14559	+	3	9	12
12983	12980	12988	-	14032	-	-	-	3	3	6
12990	12990	12994	-	14785	-	-	+	3	2	5
13008	13007	13008	-	13058	13058	13059	+	3	0	3
13013	13009	13017	-	13148	-	-	+	3	6	9
13013	13009	13017	-	14525	-	-	+	3	3	6
13055	13053	13058	-	13126	-	-	-	3	3	6
13060	13059	13064	-	13454	-	-	-	3	1	4
13082	13079	13082	-	14429	-	-	+	3	0	3
13111	13109	13111	-	13603	13603	13604	+	3	1	4
13136	13136	13137	-	14639	-	-	+	3	6	9
13141	13138	13145	-	13662	-	-	+	3	5	8
13147	13146	13151	-	13553	-	-	+	3	0	3
13147	13146	13151	-	13942	-	-	-	3	0	3
13157	13155	13161	-	14634	-	-	-	3	6	9
13158	13157	13158	+	14632	-	-	+	3	0	3
13187	13183	13190	-	14721	-	-	+	3	1	4
13199	13196	13203	-	14679	-	-	+	3	5	8
13209	13205	13209	-	14746	14746	14748	+	3	8	11
13227	13224	13230	-	14401	-	-	+	3	0	3
13246	13241	13247	-	13809	-	-	+	3	4	7
13252	13251	13256	-	13901	-	-	+	3	0	3
13272	13270	13276	-	13995	-	-	+	3	0	3
13279	-	-	+	14035	-	-	+	3	0	3
13297	13293	13302	-	13432	-	-	+	3	4	7
13297	13293	13302	-	13827	-	-	+	3	3	6
13308	13303	13312	-	14284	-	-	+	3	9	12
13316	13313	13322	-	14343	-	-	-	3	0	3
13316	13313	13322	-	14981	-	-	+	3	1	4
13330	13326	13336	+	14880	14878	14882	+	3	5	8
13332	13328	13336	-	13479	-	-	+	3	1	4
13332	13328	13336	-	14152	-	-	-	3	2	5
13332	13328	13336	-	14412	-	-	+	3	3	6
13340	13337	13341	-	14826	-	-	+	3	0	3
13347	13343	13351	-	14171	14168	14171	+	3	1	4
13353	13352	13353	-	14969	-	-	+	3	3	6
13385	13380	13388	-	14877	-	-	-	3	3	6
13385	13380	13388	-	15250	-	-	-	3	0	3
13385	13380	13388	-	15278	-	-	+	3	1	4
13394	13389	13397	-	15204	-	-	+	3	0	3
13408	13404	13411	-	13942	-	-	+	3	1	4
13431	13430	13436	-	14632	14631	14632	+	3	1	4
13446	13443	13447	+	14191	-	-	+	3	0	3
13451	13448	13454	-	14611	-	-	+	3	8	11
13451	13448	13454	-	14705	-	-	+	3	4	7
13471	13468	13475	+	14950	-	-	+	3	5	8
13479	13475	13483	-	13942	13942	13943	+	3	3	6
13479	13475	13483	-	15146	-	-	-	3	3	6
13498	13496	13504	-	13553	-	-	+	3	4	7
13516	13511	13517	-	14456	-	-	+	3	1	4
13526	13525	13527	+	15082	15082	15083	-	3	3	6

13538	-	-	+	13685	-	-	+	3	7	10
13549	13548	13553	-	14518	14514	14518	-	3	0	3
13551	13548	13555	+	13601	13601	13602	+	3	3	6
13578	13574	13583	-	14613	14613	14614	+	3	9	12
13587	13584	13591	-	13888	-	-	+	3	1	4
13593	13593	13597	-	14391	-	-	+	3	0	3
13644	13643	13648	-	13799	13795	13800	-	3	5	8
13644	13643	13648	-	14843	14842	14843	+	3	4	7
13651	13649	13653	-	14467	14467	14470	-	3	2	5
13651	13649	13653	-	14849	-	-	-	3	0	3
13659	13655	13660	-	13801	-	-	-	3	0	3
13659	13655	13660	-	14438	-	-	+	3	0	3
13674	13669	13678	-	14766	-	-	+	3	3	6
13684	13680	13687	-	13937	13935	13937	+	3	2	5
13684	13680	13687	-	14717	-	-	+	3	1	4
13699	13695	13699	+	13737	-	-	-	3	0	3
13720	13717	13722	-	14715	-	-	+	3	4	7
13751	13748	13755	-	14276	-	-	-	3	2	5
13751	13751	13753	+	14743	-	-	+	3	0	3
13770	13766	13775	-	14348	14345	14348	-	3	0	3
13776	13771	13780	+	14303	-	-	-	3	1	4
13779	13776	13782	-	14609	14608	14609	+	3	1	4
13800	13798	13803	+	13821	-	-	-	3	0	3
13814	13814	13816	+	13861	-	-	-	3	0	3
13826	13825	13831	-	14841	14841	14842	+	3	3	6
13837	-	-	-	14155	-	-	+	3	1	4
13841	13839	13843	+	13850	-	-	-	3	0	3
13842	13839	13842	-	14529	14526	14529	-	3	0	3
13876	13872	13879	-	14349	-	-	-	3	3	6
13885	13880	13891	-	14070	-	-	-	3	0	3
13898	13898	13899	+	15277	15277	15278	+	3	42	45
13916	13914	13916	-	14261	-	-	+	3	0	3
13927	13927	13929	-	14031	-	-	-	3	0	3
13936	13932	13940	-	14514	-	-	+	3	0	3
13967	13963	13968	-	14290	-	-	+	3	0	3
13967	13963	13968	-	14557	14557	14558	+	3	0	3
13972	13972	13973	-	14208	-	-	-	3	0	3
13978	13977	13982	-	14697	-	-	+	3	2	5
14001	13997	14005	-	14016	14016	14018	+	3	18	21
14001	13997	14005	-	14148	14147	14148	+	3	2	5
14038	14034	14039	+	14117	-	-	-	3	0	3
14040	14039	14043	-	14559	-	-	+	3	1	4
14046	14044	14048	-	14665	-	-	+	3	1	4
14046	14044	14048	-	14868	-	-	+	3	0	3
14096	14096	14101	-	14628	14624	14629	-	3	4	7
14108	14106	14112	-	15080	15079	15080	+	3	1	4
14127	14127	14130	+	14156	-	-	-	3	0	3
14138	14133	14141	-	14951	-	-	+	3	10	13
14152	14150	14156	+	14678	-	-	-	3	0	3
14164	14161	14166	-	14574	14571	14574	+	3	2	5
14202	14201	14204	-	14897	-	-	+	3	0	3
14223	14219	14227	-	14516	14516	14518	-	3	3	6
14238	14235	14243	-	14706	14706	14708	+	3	4	7
14238	14235	14243	-	14844	-	-	+	3	2	5
14238	14235	14243	-	15035	-	-	-	3	0	3
14265	-	-	+	14279	-	-	-	3	0	3
14270	14270	14272	+	14283	-	-	-	3	1	4
14284	14282	14289	-	14883	14880	14883	-	3	2	5
14296	14292	14299	+	14315	-	-	-	3	0	3
14303	14302	14305	+	14336	-	-	-	3	0	3
14307	14302	14308	-	14639	-	-	+	3	0	3
14316	14314	14318	-	14868	14868	14870	+	3	11	14
14331	14328	14337	-	14383	14381	14383	-	3	3	6
14331	14328	14337	-	14409	-	-	-	3	3	6
14331	14328	14337	-	14434	14434	14435	+	3	4	7
14331	14328	14337	-	14438	-	-	-	3	3	6
14331	14328	14337	-	14475	-	-	-	3	3	6
14336	14336	14339	+	14598	-	-	-	3	0	3
14360	14355	14365	-	14580	14577	14580	-	3	1	4
14360	14355	14365	-	14711	-	-	-	3	0	3
14369	14366	14375	-	14566	-	-	-	3	0	3
14369	14366	14375	-	15053	-	-	+	3	1	4
14370	14365	14373	+	14985	-	-	+	3	0	3
14375	14374	14379	+	14360	14358	14360	-	3	0	3
14375	14374	14379	+	15078	15078	15081	-	3	1	4
14398	14395	14398	-	15037	-	-	-	3	0	3
14403	14399	14407	-	15042	-	-	-	3	0	3
14423	14419	14423	-	14933	-	-	-	3	1	4
14435	14432	14438	+	14885	-	-	+	3	4	7
14442	14440	14445	+	15050	15050	15051	-	3	4	7
14456	14452	14461	-	14543	-	-	-	3	2	5
14456	14452	14461	-	15043	15040	15043	+	3	2	5
14462	14457	14466	+	14480	-	-	-	3	0	3
14464	14462	14468	-	14848	-	-	-	3	2	5

14464	14462	14468	-	14916	-	-	-	3	1	4
14479	14474	14479	-	14891	14891	14892	-	3	0	3
14485	14482	14492	-	14507	-	-	+	3	0	3
14485	14482	14492	-	14911	14911	14914	+	3	3	6
14504	14501	14508	-	14807	-	-	-	3	0	3
14504	14501	14508	-	14893	-	-	-	3	0	3
14510	14507	14512	+	14544	14541	14544	-	3	0	3
14515	-	-	+	14739	-	-	-	3	0	3
14518	14512	14521	-	14669	-	-	-	3	0	3
14518	14512	14521	-	14901	14899	14901	-	3	15	18
14532	14532	14533	-	15199	-	-	+	3	3	6
14540	14535	14540	-	14873	-	-	+	3	0	3
14545	14540	14548	+	15137	15134	15137	+	3	3	6
14547	14545	14550	-	14864	-	-	+	3	4	7
14553	-	-	-	15010	-	-	+	3	2	5
14559	14556	14559	-	15067	15065	15067	+	3	1	4
14561	14556	14564	+	15022	-	-	+	3	15	18
14565	14560	14568	-	14835	-	-	-	3	0	3
14565	14560	14568	-	15048	-	-	+	3	0	3
14565	14560	14568	-	15086	-	-	+	3	14	17
14570	14569	14573	-	14637	14633	14637	+	3	2	5
14586	14586	14590	-	15221	-	-	+	3	3	6
14612	14611	14617	-	14894	14894	14898	-	3	3	6
14612	14611	14615	+	15061	15059	15061	+	3	1	4
14622	14619	14626	-	14864	14864	14866	+	3	3	6
14622	14619	14626	-	15068	-	-	-	3	0	3
14632	14628	14632	+	14667	-	-	-	3	0	3
14638	14638	14641	-	15021	-	-	+	3	6	9
14638	14634	14640	+	14662	14661	14662	-	3	0	3
14651	14650	14654	+	14634	14633	14634	-	3	13	16
14660	14655	14661	+	14776	14776	14780	+	3	1	4
14666	14661	14671	-	14680	-	-	+	3	0	3
14666	14661	14671	-	14787	14783	14787	-	3	0	3
14676	14672	14680	-	14768	14768	14769	+	3	4	7
14676	14672	14680	-	15043	15042	15043	-	3	3	6
14693	14688	14697	-	14895	-	-	-	3	1	4
14693	14688	14697	-	15152	-	-	+	3	6	9
14704	14700	14708	-	14807	-	-	-	3	0	3
14725	14723	14725	-	15015	-	-	-	3	3	6
14725	14723	14725	-	15071	-	-	-	3	1	4
14748	14744	14749	-	15293	15292	15293	+	3	7	10
14761	14759	14764	-	14866	14866	14867	-	3	3	6
14767	14765	14771	-	14924	-	-	+	3	2	5
14767	14765	14771	-	14988	14987	14988	-	3	2	5
14786	14781	14789	-	15198	-	-	-	3	0	3
14789	14784	14789	+	15178	15178	15182	+	3	4	7
14800	14798	14800	-	15009	-	-	+	3	2	5
14806	14801	14809	-	15181	-	-	-	3	1	4
14832	14832	14836	+	14858	14858	14860	-	3	2	5
14838	14836	14841	-	14956	-	-	+	3	0	3
14847	14842	14851	-	14967	14965	14967	-	3	5	8
14847	14842	14851	-	15032	15032	15034	-	3	3	6
14847	14842	14851	-	15217	-	-	+	3	3	6
14857	14854	14859	+	15040	15040	15043	-	3	2	5
14859	14857	14862	-	14989	-	-	-	3	1	4
14864	14863	14864	-	15027	-	-	-	3	0	3
14870	14866	14870	+	15183	-	-	+	3	0	3
14877	14873	14879	-	15043	-	-	+	3	2	5
14877	14873	14879	-	15068	15066	15068	-	3	3	6
14877	14872	14882	+	14807	-	-	-	3	0	3
14885	14885	14888	+	15133	-	-	+	3	3	6
14894	14890	14897	-	14981	-	-	-	3	0	3
14894	14890	14897	-	15008	15005	15008	-	3	2	5
14894	14890	14897	-	15035	15035	15036	-	3	0	3
14910	14909	14913	-	15051	15051	15053	-	3	0	3
14916	14915	14918	-	15059	15059	15060	-	3	0	3
14947	14943	14953	-	15083	15083	15086	-	3	2	5
14947	14943	14953	-	15098	15098	15099	-	3	1	4
14947	14943	14953	-	15125	15124	15125	-	3	2	5
14957	14956	14960	-	15092	-	-	-	3	0	3
14964	14962	14968	-	15034	-	-	-	3	0	3
14964	14962	14968	-	15058	-	-	-	3	0	3
14967	14966	14968	+	15156	-	-	+	3	0	3
14967	14966	14968	+	15176	-	-	+	3	0	3
14973	14969	14975	+	15147	-	-	+	3	3	6
15004	15000	15009	+	15056	15055	15056	+	3	5	8
15015	15010	15018	+	15173	15169	15173	-	3	0	3
15025	15021	15028	+	15043	-	-	-	3	0	3
15030	15028	15032	-	15121	-	-	-	3	0	3
15032	15029	15034	+	15070	15070	15071	-	3	0	3
15053	15053	15057	+	15084	15084	15088	-	3	1	4
15070	15069	15071	+	15103	-	-	-	3	0	3
15076	15072	15079	+	15086	-	-	-	3	0	3
15078	15077	15083	-	15108	-	-	+	3	0	3

15092	15088	15096	+	15066 -	-		-	3	3	6
15092	15088	15096	+	15140 -	-		-	3	0	3
15102	15098	15105	+	15116 -	-		-	3	0	3
15102	15098	15105	+	15138 -	-		-	3	0	3
15102	15098	15105	+	15278 -	-		-	3	0	3
15116	15112	15119	+	15181 -	-		-	3	0	3
15169	15165	15171	+	15185 -	-		-	3	3	6
15185	15181	15190	+	15204	15204	15208	+	3	0	3
15185	15181	15190	+	15213	15213	15217	+	3	0	3
15185	15181	15190	+	15282	15278	15282	+	3	1	4
15205	15200	15208	+	15216 -	-		+	3	0	3
15217	15214	15217	-	15298 -	-		-	3	0	3
15228	15225	15229	+	15245 -	-		-	3	0	3
15340 -	-		+	15369 -	-		-	3	0	3
74	72	74	+	14066	14064	14066	+	2	4	6
77	76	77	-	331 -	-		-	2	0	2
77	76	77	-	14069 -	-		-	2	2	4
112 -	-		-	480 -	-		-	2	0	2
130	129	131	+	143	142	143	-	2	1	3
157	157	160	-	173 -	-		+	2	0	2
231	228	232	-	243	243	246	+	2	1	3
278	276	283	-	426 -	-		-	2	0	2
278	276	283	-	14514 -	-		-	2	0	2
290	288	294	+	14453 -	-		+	2	0	2
302	300	307	+	13042 -	-		-	2	2	4
302	300	307	+	13046	13044	13046	+	2	1	3
302	300	307	+	14923 -	-		+	2	0	2
307 -	-		-	583 -	-		-	2	0	2
370	369	373	+	6670 -	-		-	2	0	2
373	372	374	-	568	568	569	-	2	2	4
488	484	489	+	12644 -	-		+	2	2	4
495	495	499	+	14047 -	-		+	2	0	2
534	534	536	+	14339	14339	14341	+	2	5	7
655	655	658	+	13436 -	-		+	2	0	2
684	682	686	+	14138	14136	14138	+	2	2	4
689	688	693	+	10313 -	-		+	2	0	2
700	698	703	+	10934 -	-		+	2	2	4
700	698	703	+	13127 -	-		+	2	0	2
722	721	723	+	12945 -	-		+	2	1	3
770	769	775	+	14241 -	-		+	2	4	6
770	769	775	+	15033	15032	15033	+	2	9	11
802	801	804	+	12454 -	-		+	2	1	3
849	845	852	+	9147 -	-		+	2	0	2
854	854	855	+	14246	14246	14247	+	2	2	4
871 -	-		+	13742 -	-		+	2	2	4
923	920	927	+	893	890	893	-	2	3	5
923	920	927	+	13308 -	-		+	2	0	2
923	920	927	+	14591 -	-		+	2	0	2
935	932	939	+	13674 -	-		+	2	1	3
957	957	959	+	3052 -	-		+	2	0	2
957	957	959	+	12999 -	-		+	2	0	2
961	959	961	-	928 -	-		+	2	0	2
979	979	980	+	14144 -	-		+	2	0	2
991	988	991	+	14294 -	-		+	2	0	2
991	988	991	+	14609 -	-		+	2	0	2
1037	1033	1041	+	14939	14939	14940	+	2	3	5
1037	1033	1041	+	15069 -	-		+	2	1	3
1054	1050	1054	+	13404 -	-		+	2	2	4
1066 -	-		+	12826 -	-		+	2	0	2
1068 -	-		-	1092 -	-		+	2	0	2
1082	1078	1082	+	13028 -	-		+	2	3	5
1085	1082	1085	-	11404 -	-		-	2	0	2
1099	1095	1101	+	12897 -	-		+	2	0	2
1241	1239	1241	+	14405 -	-		+	2	7	9
1242	1239	1244	-	1393	1393	1394	-	2	2	4
1364	1360	1364	-	15011 -	-		+	2	2	4
1407	1407	1408	+	1450	1449	1450	-	2	1	3
1442	1440	1446	+	1460 -	-		-	2	2	4
1451	1449	1451	+	6705 -	-		+	2	0	2
1458	1457	1462	-	12825 -	-		-	2	2	4
1559 -	-		+	1595 -	-		+	2	2	4
1664 -	-		-	1724 -	-		-	2	0	2
1705 -	-		+	1632 -	-		-	2	0	2
1733 -	-		-	1828 -	-		-	2	0	2
1769	1769	1772	+	1779	1779	1780	-	2	2	4
1769	1769	1772	+	14123 -	-		+	2	2	4
1769	1769	1772	+	14504	14501	14504	+	2	2	4
1785	1785	1786	+	1766	1765	1766	-	2	1	3
1809	1809	1811	+	14255 -	-		+	2	0	2
1863	1860	1866	+	12927 -	-		+	2	2	4
1863	1860	1866	+	13797	13797	13799	+	2	4	6
1886	1883	1891	+	12635	12635	12636	+	2	2	4
1900	1896	1901	+	1925 -	-		+	2	0	2
1900	1896	1901	+	14921 -	-		+	2	2	4

1908	1904	1911	+	15235	-	-	+	2	2	4
1917	1915	1917	+	12147	-	-	+	2	1	3
1924	1922	1924	+	1962	-	-	-	2	0	2
1932	1927	1935	+	10985	10985	10986	+	2	2	4
1932	1927	1935	+	13298	-	-	+	2	0	2
1938	1937	1939	+	10866	-	-	+	2	3	5
1938	1937	1939	+	14761	14759	14761	+	2	2	4
1944	1941	1949	+	13617	-	-	+	2	0	2
1959	1953	1960	+	11522	-	-	-	2	0	2
1959	1953	1960	+	13715	-	-	+	2	3	5
1972	1967	1976	+	1911	-	-	-	2	0	2
2006	2006	2010	+	2136	-	-	+	2	0	2
2016	2012	2018	+	12090	-	-	+	2	2	4
2016	2012	2018	+	12171	-	-	-	2	0	2
2016	2012	2018	+	14034	-	-	+	2	0	2
2016	2012	2018	+	14760	-	-	+	2	2	4
2023	2022	2024	+	13027	-	-	+	2	0	2
2043	2043	2047	+	14607	-	-	+	2	0	2
2051	2051	2052	-	12530	12530	12531	-	2	0	2
2070	-	-	+	12474	-	-	+	2	3	5
2070	-	-	+	13344	-	-	+	2	0	2
2070	-	-	+	13405	-	-	+	2	0	2
2093	2093	2094	+	13487	-	-	+	2	3	5
2106	-	-	-	7398	-	-	+	2	0	2
2109	2106	2111	+	12346	-	-	+	2	1	3
2119	2117	2120	+	14065	14063	14065	+	2	1	3
2131	2127	2136	+	13356	-	-	+	2	3	5
2153	2153	2155	+	13871	13871	13873	+	2	12	14
2173	2173	2177	+	14193	-	-	-	2	1	3
2190	2187	2194	+	12601	-	-	+	2	1	3
2190	2187	2194	+	13099	-	-	-	2	0	2
2198	2196	2203	+	2258	2257	2258	-	2	1	3
2249	2248	2251	+	11207	-	-	+	2	0	2
2249	2248	2251	+	12743	-	-	+	2	1	3
2249	2248	2251	+	13811	-	-	+	2	0	2
2252	2252	2256	-	15022	15022	15023	+	2	2	4
2268	-	-	-	2245	-	-	+	2	0	2
2276	2271	2280	+	12387	-	-	+	2	1	3
2276	2271	2280	+	12808	-	-	+	2	0	2
2317	-	-	+	12985	-	-	+	2	0	2
2397	2397	2400	-	2651	-	-	-	2	0	2
2408	2405	2409	+	13776	-	-	-	2	0	2
2408	2405	2409	+	14705	-	-	+	2	0	2
2414	2413	2418	+	12111	-	-	+	2	0	2
2414	2413	2418	+	13940	-	-	+	2	6	8
2414	2413	2418	+	14384	-	-	+	2	3	5
2426	2422	2429	+	11911	11910	11911	+	2	1	3
2426	2422	2429	+	13738	-	-	-	2	4	6
2435	2431	2436	+	13515	13515	13518	+	2	5	7
2449	-	-	+	11828	-	-	+	2	1	3
2473	2471	2477	+	11658	11657	11658	+	2	6	8
2493	2491	2493	+	10723	-	-	+	2	2	4
2495	2493	2495	-	2600	-	-	-	2	0	2
2500	2500	2501	+	13372	13372	13373	+	2	1	3
2515	2514	2516	+	10795	-	-	+	2	1	3
2515	2514	2516	+	12060	-	-	+	2	0	2
2545	2542	2545	-	10668	-	-	+	2	0	2
2574	2570	2574	+	3102	-	-	+	2	0	2
2574	2570	2574	+	15018	-	-	+	2	2	4
2588	2588	2592	+	13796	13796	13798	+	2	3	5
2620	2620	2622	+	12220	-	-	+	2	1	3
2620	2620	2622	+	12707	-	-	+	2	3	5
2620	2620	2622	+	13012	-	-	+	2	0	2
2641	2639	2645	+	14231	-	-	+	2	2	4
2752	2748	2752	-	3216	-	-	-	2	0	2
2779	2778	2780	+	9602	-	-	+	2	2	4
2808	2807	2809	+	13356	-	-	+	2	1	3
2814	2813	2814	+	9858	9857	9858	+	2	3	5
2827	2827	2832	+	10885	-	-	+	2	0	2
2827	2827	2832	+	12421	-	-	+	2	2	4
2827	2827	2832	+	14691	14689	14691	+	2	2	4
2853	2853	2856	+	11937	-	-	+	2	0	2
2927	2924	2931	+	14806	14802	14806	-	2	0	2
2975	-	-	-	3048	-	-	-	2	0	2
2975	2975	2979	+	10215	-	-	+	2	0	2
2975	2975	2979	+	12695	-	-	+	2	1	3
3060	-	-	+	3072	-	-	-	2	0	2
3065	-	-	+	3098	-	-	-	2	4	6
3142	3140	3143	+	14009	-	-	+	2	4	6
3146	-	-	-	13846	-	-	+	2	0	2
3149	3149	3150	+	14759	14759	14760	+	2	3	5
3154	3153	3156	+	11471	-	-	+	2	0	2
3166	3165	3169	+	12202	-	-	+	2	3	5
3184	3183	3188	+	13553	-	-	+	2	2	4

3198	3196	3199	+	11827 -	-		+	2	1	3
3204 -	-		-	3572 -	-		-	2	0	2
3227	3227	3231	+	12966 -	-		+	2	0	2
3227	3227	3231	+	15105 -	-		+	2	0	2
3243	3240	3243	+	11578 -	-		+	2	0	2
3258	3255	3258	+	12455	12454	12455	+	2	1	3
3272	3271	3273	+	12363 -	-		+	2	1	3
3273	3273	3275	-	5654 -	-		+	2	0	2
3279	3275	3283	+	11711	11711	11712	+	2	2	4
3279	3275	3283	+	14414	14414	14415	+	2	2	4
3288 -	-		+	8043 -	-		+	2	0	2
3320 -	-		-	6768 -	-		-	2	2	4
3341	3340	3343	+	11446 -	-		+	2	0	2
3373	3370	3377	+	11809 -	-		+	2	5	7
3373	3370	3377	+	12605 -	-		-	2	0	2
3380 -	-		+	15081 -	-		+	2	2	4
3392	3391	3393	+	14901 -	-		+	2	1	3
3416	3414	3417	-	3443	3440	3443	+	2	1	3
3419	3418	3421	+	3380 -	-		-	2	0	2
3419	3418	3421	+	11687 -	-		+	2	1	3
3419	3418	3421	+	14503	14501	14503	+	2	2	4
3483	3479	3483	-	3436 -	-		+	2	0	2
3485 -	-		+	13769 -	-		+	2	0	2
3508	3506	3511	+	12014	12014	12016	+	2	3	5
3517 -	-		+	11173 -	-		+	2	5	7
3566	3565	3570	+	13800 -	-		+	2	0	2
3577	3577	3581	+	12078 -	-		+	2	2	4
3650	3650	3652	+	14360 -	-		+	2	2	4
3661	3659	3661	+	13861 -	-		+	2	4	6
3673	3669	3673	+	13444 -	-		+	2	0	2
3690	3690	3692	+	3771 -	-		+	2	0	2
3712	3710	3712	+	13169 -	-		+	2	1	3
3720	3720	3721	-	13629 -	-		+	2	0	2
3773	3771	3773	+	13455 -	-		+	2	0	2
3821	3821	3824	+	14490 -	-		+	2	1	3
3886	3885	3886	+	10228	10227	10228	+	2	3	5
3896 -	-		+	12056 -	-		+	2	1	3
3966 -	-		-	12853 -	-		+	2	0	2
3972	3972	3976	+	13720 -	-		+	2	0	2
4053	4052	4053	+	13149	13148	13149	+	2	1	3
4056 -	-		-	5908 -	-		-	2	0	2
4061 -	-		+	12965 -	-		+	2	0	2
4105	4103	4107	+	10659 -	-		+	2	0	2
4105	4103	4107	+	13735 -	-		+	2	0	2
4188	4188	4192	-	13760 -	-		-	2	0	2
4244 -	-		+	11468 -	-		+	2	1	3
4300	4298	4300	+	14409 -	-		+	2	2	4
4420	4418	4420	+	4422 -	-		-	2	0	2
4438	4435	4442	+	13567 -	-		+	2	0	2
4457	4455	4460	+	4763 -	-		-	2	0	2
4471	4469	4471	+	14883 -	-		-	2	0	2
4488	4484	4488	+	12306 -	-		+	2	2	4
4517 -	-		-	4590 -	-		-	2	2	4
4544	4544	4547	+	13431 -	-		+	2	6	8
4602	4597	4605	+	14371 -	-		-	2	0	2
4610	4610	4611	+	11439 -	-		+	2	0	2
4663	4663	4665	-	12228 -	-		+	2	1	3
4687	4685	4688	+	12125 -	-		+	2	0	2
4707 -	-		+	13227 -	-		+	2	0	2
4710	4708	4711	-	4800 -	-		-	2	0	2
4737	4737	4739	+	14565 -	-		+	2	0	2
4749	4748	4751	+	14408 -	-		+	2	1	3
4887	4887	4889	+	13029 -	-		+	2	1	3
5010	5010	5011	-	13896	13896	13897	+	2	2	4
5099	5095	5099	+	13415 -	-		+	2	1	3
5137 -	-		+	5136 -	-		+	2	0	2
5207	5207	5209	+	13404 -	-		+	2	0	2
5252	5251	5253	+	14103 -	-		+	2	0	2
5291	5290	5295	-	5345	5344	5345	-	2	2	4
5324	5324	5327	+	13952	13952	13953	+	2	2	4
5408	5408	5411	+	5444 -	-		+	2	0	2
5434 -	-		-	14523 -	-		+	2	0	2
5773	5773	5775	+	10461 -	-		+	2	1	3
5780	5778	5780	+	15185	15185	15187	-	2	36	38
5819 -	-		+	13793 -	-		+	2	2	4
5846 -	-		-	14753 -	-		+	2	0	2
6037	6033	6037	-	14609 -	-		+	2	3	5
6117 -	-		-	6303 -	-		-	2	2	4
6142 -	-		+	13210 -	-		+	2	0	2
6191 -	-		+	14033 -	-		+	2	0	2
6269 -	-		+	11987 -	-		+	2	0	2
6272	6272	6276	-	6582 -	-		-	2	0	2
6272	6272	6276	-	14532 -	-		+	2	2	4
6291	6289	6292	+	7920 -	-		+	2	0	2

6302	6302	6303	+	6363 -	-		+	2	0	2
6303 -	-		-	13911 -	-		+	2	0	2
6386 -	-		-	14476 -	-		+	2	0	2
6532	6529	6533	+	11824 -	-		+	2	0	2
6579	6577	6579	+	14571 -	-		+	2	3	5
6584	6584	6588	-	12269 -	-		+	2	0	2
6781 -	-		-	13447 -	-		-	2	0	2
7049	7049	7052	+	7636 -	-		+	2	2	4
7109 -	-		+	12557 -	-		+	2	2	4
7144	7144	7146	+	13841 -	-		+	2	0	2
7170 -	-		+	7598 -	-		-	2	0	2
7239	7238	7243	-	13711 -	-		-	2	0	2
7354	7352	7355	-	13523	13521	13523	-	2	0	2
7479	7479	7483	-	8174 -	-		-	2	0	2
7502	7497	7506	-	13296 -	-		+	2	0	2
7517	7515	7517	+	14348 -	-		-	2	0	2
7520	7520	7524	-	15142 -	-		+	2	2	4
7538	7534	7538	-	14330	14330	14331	+	2	1	3
7578	7578	7581	-	13841 -	-		-	2	0	2
7846	7842	7846	-	8222 -	-		-	2	2	4
8068	8065	8069	+	14799	14797	14799	+	2	2	4
8082 -	-		-	8234 -	-		-	2	0	2
8089 -	-		+	15260 -	-		+	2	0	2
8131	8131	8135	-	13621 -	-		+	2	0	2
8131	8131	8135	-	14357 -	-		+	2	0	2
8149	8149	8151	+	12601 -	-		+	2	1	3
8177 -	-		-	12229 -	-		-	2	2	4
8190 -	-		-	12997 -	-		+	2	0	2
8319	8318	8320	-	11492	11492	11493	+	2	2	4
8332	8332	8333	-	15094	15092	15094	+	2	0	2
8400 -	-		+	13664 -	-		+	2	0	2
8465	8463	8465	-	14577	14577	14578	+	2	2	4
8471 -	-		+	8603 -	-		+	2	0	2
8485	8481	8489	-	14722 -	-		+	2	0	2
8596 -	-		-	12801 -	-		+	2	2	4
8699	8699	8700	-	14377	14377	14378	+	2	2	4
8727 -	-		+	13107 -	-		+	2	2	4
8802	8798	8803	-	15198	15198	15199	+	2	2	4
8862 -	-		+	15006 -	-		+	2	0	2
8875	8875	8876	-	14148 -	-		+	2	8	10
8875	8875	8876	-	15023 -	-		+	2	2	4
8968	8968	8969	-	13934	13934	13935	+	2	2	4
9052	9051	9052	-	14158 -	-		+	2	0	2
9065	9061	9065	-	14378 -	-		+	2	2	4
9086	9086	9090	+	13374 -	-		+	2	1	3
9100	9098	9103	-	9130 -	-		+	2	0	2
9128 -	-		+	9488 -	-		+	2	2	4
9198 -	-		-	12518 -	-		+	2	6	8
9261	9258	9261	+	9412	9412	9414	+	2	1	3
9273	9270	9273	-	12600 -	-		+	2	0	2
9289 -	-		+	9502 -	-		-	2	0	2
9350	9346	9351	-	13427 -	-		+	2	3	5
9350	9346	9351	-	13840 -	-		+	2	0	2
9424 -	-		+	9785 -	-		-	2	0	2
9464 -	-		-	14435 -	-		+	2	2	4
9529	9529	9533	-	10140 -	-		+	2	2	4
9529	9529	9533	-	15294 -	-		+	2	2	4
9536 -	-		-	14688 -	-		+	2	0	2
9574	9570	9575	-	14186 -	-		+	2	0	2
9618	9617	9619	-	14376	14376	14377	+	2	2	4
9671	9671	9673	+	9706	9706	9707	-	2	0	2
9704	9704	9707	+	9673 -	-		-	2	0	2
9706	9706	9707	-	14168 -	-		+	2	0	2
9724 -	-		-	11271 -	-		+	2	0	2
9796 -	-		+	12994 -	-		+	2	0	2
9831 -	-		-	14986 -	-		+	2	3	5
9850	9848	9850	+	9875	9871	9875	+	2	2	4
9984	9984	9988	-	14338 -	-		+	2	0	2
9993 -	-		+	10057 -	-		-	2	0	2
10011	10008	10013	-	13299	13297	13299	+	2	2	4
10011	10008	10013	-	14677 -	-		+	2	0	2
10024 -	-		-	14715 -	-		+	2	0	2
10050	10046	10053	-	10211	10211	10212	-	2	0	2
10065	10062	10065	-	13126 -	-		+	2	1	3
10076	10073	10078	-	14625 -	-		+	2	0	2
10097	10096	10098	-	14408 -	-		-	2	2	4
10103	10102	10103	+	14720 -	-		+	2	0	2
10180	10180	10181	-	14223 -	-		+	2	2	4
10210 -	-		-	14751 -	-		+	2	0	2
10258	10256	10262	-	14637 -	-		+	2	4	6
10278	10276	10282	-	13633 -	-		-	2	0	2
10318	10316	10319	-	14060	14059	14060	+	2	3	5
10318	10316	10319	-	14411 -	-		+	2	0	2
10348 -	-		-	13640 -	-		-	2	0	2

10370	10366	10373	-	14677 -	-		+	2	0	2
10383	10379	10388	-	14524 -	-		+	2	1	3
10425	10421	10425	-	14617	14615	14617	+	2	1	3
10436	10434	10436	-	14923 -	-		+	2	0	2
10472	10471	10473	-	14662 -	-		+	2	2	4
10496	10493	10498	+	10517	10515	10517	-	2	2	4
10543	10541	10544	-	10860 -	-		-	2	0	2
10549 -	-		-	10857 -	-		-	2	0	2
10568	10568	10571	-	14582 -	-		+	2	0	2
10587 -	-		-	10712 -	-		-	2	0	2
10605 -	-		-	13966 -	-		+	2	0	2
10651	10648	10651	+	10712 -	-		+	2	0	2
10662 -	-		+	10672 -	-		-	2	0	2
10671	10668	10671	-	14353	14350	14353	+	2	3	5
10710 -	-		-	14821 -	-		-	2	0	2
10724	10723	10725	+	14252 -	-		+	2	0	2
10725	10723	10726	-	14199 -	-		+	2	2	4
10792	10792	10793	+	14712 -	-		+	2	0	2
10797	10797	10798	-	12301	12298	12301	-	2	0	2
10803	10803	10806	-	13045 -	-		-	2	0	2
10847	10843	10847	-	13362 -	-		-	2	0	2
10855	10855	10858	-	12327 -	-		+	2	2	4
10894	10892	10898	-	12125 -	-		+	2	2	4
10993	10991	10996	-	14410 -	-		+	2	0	2
10993	10991	10996	-	14565	14565	14566	+	2	2	4
11028	11027	11032	-	14601 -	-		+	2	0	2
11097	11092	11099	-	12645 -	-		+	2	0	2
11117	11114	11117	-	13693 -	-		+	2	2	4
11122 -	-		+	13498 -	-		+	2	0	2
11156	11152	11156	-	13571 -	-		+	2	0	2
11156	11152	11156	-	14471 -	-		+	2	3	5
11170	11169	11174	-	14158 -	-		+	2	0	2
11218	11213	11218	-	12219 -	-		+	2	0	2
11218	11213	11218	-	13857 -	-		+	2	2	4
11228	11226	11231	-	13772	13772	13773	+	2	2	4
11247	11244	11247	+	13231 -	-		+	2	0	2
11249	11247	11250	-	14169 -	-		+	2	2	4
11293	11292	11293	+	13945	13944	13945	+	2	4	6
11331 -	-		-	14783 -	-		+	2	5	7
11393	11390	11396	-	13811 -	-		+	2	0	2
11451	11450	11456	-	12944 -	-		+	2	0	2
11458	11458	11462	-	14289 -	-		+	2	0	2
11468	11464	11472	-	12718 -	-		+	2	0	2
11476	11475	11479	-	15033 -	-		+	2	4	6
11494	11492	11498	-	13427 -	-		+	2	1	3
11494	11492	11498	-	14477 -	-		+	2	1	3
11510	11506	11514	-	12742 -	-		+	2	0	2
11522	11519	11523	-	14259 -	-		+	2	2	4
11522	11519	11523	-	14275	14273	14275	+	2	2	4
11542	11541	11542	-	14096 -	-		+	2	4	6
11576	11575	11578	-	14344 -	-		+	2	0	2
11586	11582	11587	-	13753 -	-		+	2	2	4
11586	11582	11587	-	14470	14470	14471	-	2	2	4
11586	11582	11587	-	15175 -	-		+	2	1	3
11602	11599	11605	-	13003 -	-		+	2	2	4
11602	11599	11605	-	14090 -	-		+	2	2	4
11617	11614	11622	-	14401	14400	14401	+	2	1	3
11631	11628	11634	+	14880 -	-		+	2	0	2
11641	11637	11644	-	13580	13579	13580	+	2	4	6
11658 -	-		-	11840 -	-		-	2	2	4
11713	11712	11715	-	14217 -	-		-	2	1	3
11733	11730	11736	-	14772	14771	14772	+	2	6	8
11758	11754	11761	-	14359	14356	14359	+	2	1	3
11758	11754	11761	-	14391 -	-		+	2	0	2
11771 -	-		+	11792 -	-		+	2	0	2
11774	11769	11774	-	14565	14563	14565	+	2	1	3
11830	11828	11834	-	13801 -	-		-	2	5	7
11857	11856	11858	-	14540 -	-		+	2	1	3
11866	11863	11867	-	12282 -	-		+	2	0	2
11906	11906	11907	-	13564 -	-		+	2	2	4
11914	11911	11914	-	14759	14759	14760	+	2	2	4
11914	11911	11914	-	15045 -	-		+	2	5	7
11915 -	-		+	14709 -	-		+	2	0	2
11920	11916	11926	-	13965	13965	13966	+	2	2	4
11939	11939	11941	-	15185 -	-		-	2	0	2
11950	11947	11950	-	14524 -	-		+	2	3	5
11964	11962	11965	+	12733	12733	12734	+	2	0	2
11979	11975	11979	-	14779 -	-		+	2	1	3
11979	11975	11979	-	15161	15161	15162	+	2	4	6
12040	12037	12044	-	14386 -	-		-	2	0	2
12053	12052	12057	-	13683 -	-		+	2	4	6
12053	12052	12057	-	13952 -	-		+	2	2	4
12075	12070	12076	-	12137 -	-		+	2	1	3
12075	12070	12076	-	13862 -	-		+	2	2	4

12094		12090	12094	-	12454	-	-		-	2	0	2
12101		12100	12106	-	12329		12325	12332	-	2	7	9
12101		12100	12106	-	12334	-	-		-	2	0	2
12101		12100	12106	-	13674	-	-		+	2	1	3
12113		12109	12116	-	13943	-	-		+	2	4	6
12113		12109	12116	-	14573	-	-		-	2	0	2
12113		12109	12116	-	15201	-	-		+	2	0	2
12147		12142	12151	-	13550	-	-		+	2	0	2
12147		12142	12151	-	13944	-	-		+	2	2	4
12147		12142	12151	-	14548	-	-		+	2	2	4
12147		12142	12151	-	14660	-	-		-	2	0	2
12161		12159	12162	-	13873	-	-		-	2	0	2
12161		12159	12162	-	13930	-	-		+	2	0	2
12167		12166	12172	-	13685	-	-		+	2	0	2
12187		12183	12190	-	14138		14138	14139	+	2	2	4
12187		12183	12190	-	14837	-	-		+	2	0	2
12199		12195	12201	-	13084	-	-		+	2	0	2
12208		12208	12209	+	14596		14596	14597	+	2	1	3
12220	-	-		-	14336	-	-		+	2	0	2
12233		12230	12234	-	14556	-	-		-	2	0	2
12246		12246	12250	-	13333	-	-		+	2	0	2
12274		12274	12276	-	12410		12410	12411	-	2	2	4
12291		12289	12293	-	12997	-	-		-	2	0	2
12307		12303	12310	-	14199	-	-		+	2	1	3
12307		12303	12310	-	14292	-	-		+	2	0	2
12314		12313	12314	+	13741		13740	13741	+	2	1	3
12321		12318	12326	-	13372	-	-		+	2	0	2
12322		12317	12324	+	13967	-	-		-	2	2	4
12322		12317	12324	+	14291	-	-		+	2	0	2
12358		12357	12359	-	14842	-	-		+	2	0	2
12364		12360	12365	-	12461	-	-		-	2	0	2
12383		12380	12386	-	12977	-	-		+	2	1	3
12394	-	-		+	12685	-	-		-	2	0	2
12407		12402	12411	-	13768	-	-		+	2	0	2
12426		12425	12426	-	13738	-	-		-	2	0	2
12465		12461	12467	-	13941	-	-		+	2	4	6
12481		12476	12482	-	14668		14668	14669	+	2	6	8
12501		12501	12502	-	12634	-	-		+	2	1	3
12508		12506	12509	-	14619	-	-		+	2	9	11
12514		12513	12515	-	14786		14786	14787	+	2	2	4
12531		12531	12535	+	12565	-	-		-	2	0	2
12536		12535	12542	-	14571	-	-		+	2	2	4
12553		12549	12557	-	14578	-	-		+	2	1	3
12560		12560	12561	-	13416	-	-		+	2	2	4
12560		12560	12561	-	15006	-	-		+	2	2	4
12591		12589	12594	-	13699	-	-		-	2	0	2
12591		12589	12594	-	14634	-	-		+	2	1	3
12596		12596	12598	-	13141	-	-		-	2	0	2
12605		12599	12607	-	12965	-	-		+	2	3	5
12605		12599	12607	-	13955		13955	13956	-	2	1	3
12611		12609	12614	-	13318	-	-		+	2	0	2
12616		12615	12620	-	14290	-	-		+	2	0	2
12618	-	-		+	12995	-	-		-	2	0	2
12633	-	-		-	14494	-	-		+	2	0	2
12640	-	-		+	12708	-	-		-	2	0	2
12641		12639	12641	-	13604	-	-		-	2	0	2
12662		12659	12666	-	13158	-	-		+	2	0	2
12662		12659	12666	-	13847	-	-		-	2	2	4
12662		12658	12662	+	15088	-	-		+	2	0	2
12667		12666	12668	+	13858		13858	13859	+	2	1	3
12676		12672	12678	-	14841	-	-		+	2	7	9
12683		12683	12687	-	14764	-	-		+	2	0	2
12685		12684	12685	+	14598	-	-		+	2	0	2
12685		12684	12685	+	14851	-	-		+	2	1	3
12708	-	-		+	12640	-	-		-	2	0	2
12713	-	-		+	14224	-	-		+	2	2	4
12717		12713	12721	-	14401		14401	14402	+	2	0	2
12739		12737	12743	-	12993	-	-		-	2	0	2
12739		12737	12743	-	14551		14549	14551	-	2	4	6
12745		12744	12749	-	14554	-	-		-	2	0	2
12745		12744	12749	-	14885		14883	14885	+	2	2	4
12754		12753	12755	-	13707	-	-		-	2	3	5
12754		12753	12755	-	13838		13838	13839	+	2	7	9
12775		12771	12780	-	14150	-	-		+	2	2	4
12775		12771	12780	-	14410	-	-		+	2	0	2
12797		12797	12800	-	14267	-	-		+	2	0	2
12797		12797	12800	-	14672	-	-		+	2	6	8
12802		12801	12807	-	14991		14991	14992	+	2	7	9
12813		12810	12815	-	14226		14225	14226	+	2	1	3
12828		12825	12828	-	13659	-	-		+	2	0	2
12840		12840	12841	+	12923		12923	12924	-	2	2	4
12851		12850	12854	-	13685		13685	13686	+	2	1	3
12864		12864	12869	-	13957	-	-		+	2	2	4
12877		12874	12877	-	14189	-	-		-	2	0	2

12884	12881	12888	-	13266 -	-		+	2	2	4
12896	12895	12898	-	14107 -	-		-	2	1	3
12903	12900	12905	-	14177 -	-		+	2	2	4
12911	12906	12914	-	14117 -	-		-	2	0	2
12911	12906	12914	-	14684 -	-		+	2	4	6
12915	12914	12915	+	13205 -	-		-	2	0	2
12921	12917	12924	-	13844 -	-		+	2	2	4
12921	12917	12924	-	14059 -	-		+	2	2	4
12921	12917	12924	-	14508 -	-		+	2	5	7
12931	12930	12935	+	15067 -	-		+	2	1	3
12940	12936	12945	-	13697 -	-		+	2	3	5
12947	12944	12949	+	14107 -	-		+	2	3	5
12949	12948	12949	-	13773 -	-		+	2	0	2
12949	12948	12949	-	14634 -	-		+	2	5	7
12956	12952	12958	-	14308	14308	14309	+	2	4	6
12956	12952	12958	-	14467	14467	14469	+	2	10	12
12961 -	-		+	14699 -	-		+	2	0	2
12965	12965	12968	-	13860	13860	13861	+	2	3	5
12965	12965	12968	-	14307 -	-		+	2	2	4
12965	12965	12968	-	15005 -	-		+	2	3	5
12970	12967	12970	+	14492 -	-		+	2	0	2
12978	12973	12978	+	14988 -	-		+	2	0	2
12983	12980	12988	-	14014 -	-		-	2	0	2
12983	12980	12988	-	14569	14569	14572	-	2	2	4
13000	12997	13002	-	13546 -	-		+	2	0	2
13000	12997	13002	-	13869 -	-		-	2	0	2
13013	13009	13017	-	14265	14265	14266	+	2	4	6
13013	13009	13017	-	14331 -	-		+	2	2	4
13013	13009	13017	-	15144 -	-		+	2	0	2
13019 -	-		-	13157 -	-		-	2	12	14
13025	13023	13029	-	13695 -	-		+	2	0	2
13025	13023	13029	-	14435 -	-		+	2	7	9
13036	13035	13038	-	13278 -	-		+	2	0	2
13036	13035	13038	-	13576 -	-		-	2	0	2
13043	13041	13043	+	13442 -	-		+	2	4	6
13044	13040	13049	-	14156	14156	14157	+	2	2	4
13044	13040	13049	-	14559	14559	14560	+	2	4	6
13050	13050	13054	+	15089	15087	15089	+	2	2	4
13055	13053	13058	-	13199 -	-		+	2	1	3
13055	13053	13058	-	13444 -	-		-	2	1	3
13055	13053	13058	-	14633	14633	14634	+	2	2	4
13055	13053	13058	-	15030 -	-		+	2	0	2
13060	13059	13064	-	13150 -	-		-	2	0	2
13071	13067	13071	-	14836 -	-		+	2	0	2
13071	13067	13076	+	13483 -	-		+	2	0	2
13082	13079	13082	-	14307 -	-		+	2	1	3
13089	13087	13090	-	13609 -	-		+	2	4	6
13089	13087	13090	-	14409 -	-		+	2	0	2
13104	13102	13107	-	14206 -	-		+	2	0	2
13104	13102	13107	-	14706	14706	14707	+	2	2	4
13126	13123	13129	-	13299 -	-		+	2	1	3
13126	13123	13129	-	14139 -	-		+	2	1	3
13136	13136	13137	-	13967 -	-		+	2	0	2
13141	13138	13145	-	13653 -	-		-	2	0	2
13147	13146	13151	-	14339 -	-		+	2	1	3
13152	13148	13152	+	13577 -	-		-	2	0	2
13157	13155	13161	-	14458 -	-		+	2	0	2
13168	13162	13169	-	13548 -	-		+	2	2	4
13168	13162	13169	-	13735 -	-		+	2	0	2
13168	13162	13169	-	13989	13987	13989	+	2	3	5
13199	13196	13203	-	13323	13320	13323	-	2	2	4
13199	13196	13203	-	14680 -	-		-	2	0	2
13199	13196	13203	-	14951	14951	14952	-	2	2	4
13199	13196	13203	-	14997 -	-		-	2	0	2
13201 -	-		+	13415 -	-		-	2	0	2
13209	13205	13209	-	13996 -	-		+	2	6	8
13219 -	-		+	14438 -	-		+	2	0	2
13227	13224	13230	-	14317 -	-		+	2	3	5
13227	13225	13230	+	13245	13245	13247	+	2	1	3
13252	13251	13256	-	14289 -	-		+	2	0	2
13258	13257	13260	-	13455 -	-		-	2	0	2
13265	13262	13269	-	13575 -	-		-	2	0	2
13265	13262	13269	-	13797 -	-		+	2	0	2
13265	13262	13269	-	14184 -	-		+	2	0	2
13265	13262	13269	-	14621 -	-		+	2	0	2
13272	13270	13276	-	13576 -	-		-	2	0	2
13272	13270	13276	-	14383 -	-		+	2	2	4
13288	13288	13291	-	13404 -	-		-	2	1	3
13297	13293	13302	-	14105 -	-		+	2	0	2
13297	13293	13302	-	14253 -	-		-	2	0	2
13297	13293	13302	-	14258	14256	14259	-	2	8	10
13297	13293	13302	-	14355	14354	14355	-	2	1	3
13304	13302	13308	+	14264	14264	14265	+	2	1	3
13304	13302	13308	+	14344 -	-		+	2	1	3

13308	13303	13312	-	14456	14456	14458	+	2	11	13
13308	13303	13312	-	14531	-	-	+	2	2	4
13316	13313	13322	-	13742	-	-	+	2	7	9
13316	13313	13322	-	15022	-	-	-	2	1	3
13332	13328	13336	-	13750	-	-	-	2	0	2
13332	13328	13336	-	14446	-	-	-	2	0	2
13332	13328	13336	-	14597	-	-	+	2	0	2
13332	13328	13336	-	15100	-	-	+	2	0	2
13347	13343	13351	-	13675	-	-	+	2	0	2
13347	13343	13351	-	13901	-	-	+	2	1	3
13347	13343	13351	-	13981	-	-	+	2	1	3
13347	13343	13351	-	14280	-	-	-	2	0	2
13359	13356	13363	-	14012	-	-	+	2	0	2
13365	13365	13368	-	14015	-	-	+	2	1	3
13365	13365	13368	-	14314	-	-	+	2	0	2
13378	13377	13381	+	13462	-	-	-	2	0	2
13379	13377	13379	-	14732	-	-	+	2	0	2
13379	13377	13379	-	14968	-	-	+	2	0	2
13385	13380	13388	-	14187	-	-	+	2	0	2
13394	13389	13397	-	14412	-	-	+	2	3	5
13408	13404	13411	-	14017	14014	14017	-	2	0	2
13408	13404	13411	-	14150	-	-	-	2	0	2
13408	13404	13411	-	14471	-	-	-	2	0	2
13408	13404	13411	-	15174	-	-	+	2	0	2
13424	13424	13426	-	14026	-	-	-	2	2	4
13424	13424	13426	-	14887	-	-	+	2	0	2
13431	13430	13436	-	13638	-	-	-	2	1	3
13431	13430	13436	-	13758	-	-	-	2	0	2
13431	13430	13436	-	13763	-	-	-	2	0	2
13446	13443	13446	-	14191	-	-	-	2	0	2
13446	13443	13447	+	13770	-	-	+	2	0	2
13459	13457	13462	+	13498	13494	13498	-	2	1	3
13462	13458	13467	-	13586	-	-	-	2	0	2
13462	13458	13467	-	13688	-	-	-	2	0	2
13462	13458	13467	-	13708	-	-	-	2	0	2
13462	13458	13467	-	13812	-	-	-	2	2	4
13462	13458	13467	-	14264	14264	14265	+	2	2	4
13462	13458	13467	-	15137	-	-	+	2	0	2
13471	13468	13475	+	13486	-	-	-	2	0	2
13479	13475	13483	-	14683	-	-	-	2	0	2
13479	13475	13483	-	14917	-	-	-	2	2	4
13488	13485	13490	-	13881	-	-	+	2	0	2
13488	13485	13490	-	14044	14044	14045	+	2	4	6
13490	13486	13492	+	14146	-	-	+	2	0	2
13498	13496	13504	-	13846	-	-	+	2	1	3
13498	13496	13504	-	14261	-	-	+	2	0	2
13498	13496	13504	-	14930	-	-	-	2	0	2
13499	13495	13503	+	15085	15085	15089	+	2	5	7
13508	13507	13510	-	14747	14746	14748	-	2	13	15
13510	13506	13513	+	14971	-	-	+	2	1	3
13516	13511	13517	-	14144	-	-	+	2	0	2
13526	13525	13527	+	14726	-	-	+	2	0	2
13532	13531	13533	+	13501	-	-	-	2	0	2
13532	13531	13533	+	14464	-	-	+	2	3	5
13533	13528	13536	-	14622	14619	14622	+	2	2	4
13544	13538	13545	-	13666	-	-	-	2	0	2
13544	13538	13545	-	14268	-	-	+	2	2	4
13551	13548	13555	+	14663	-	-	+	2	0	2
13555	13554	13558	-	14524	14521	14524	-	2	5	7
13561	13558	13561	+	14519	-	-	+	2	0	2
13562	13559	13567	-	13568	-	-	+	2	2	4
13562	13559	13567	-	13862	-	-	+	2	2	4
13566	13562	13569	+	14898	-	-	+	2	0	2
13575	13572	13578	+	13606	13606	13607	-	2	2	4
13575	13572	13578	+	14570	14569	14570	+	2	3	5
13578	13574	13583	-	13720	-	-	-	2	2	4
13578	13574	13583	-	14077	-	-	+	2	0	2
13578	13574	13583	-	14533	-	-	-	2	0	2
13587	13584	13591	-	14909	-	-	+	2	4	6
13593	13593	13597	-	13837	-	-	+	2	2	4
13599	-	-	-	14634	-	-	-	2	0	2
13605	13602	13606	-	13855	-	-	-	2	0	2
13605	13602	13606	-	13973	-	-	+	2	2	4
13605	13602	13606	-	14563	-	-	+	2	0	2
13610	13607	13611	-	14669	14667	14669	+	2	0	2
13616	13613	13619	-	13730	13730	13731	+	2	2	4
13616	13613	13619	-	14325	-	-	+	2	3	5
13616	13613	13619	-	14528	14528	14529	+	2	3	5
13616	13613	13619	-	14668	14668	14669	+	2	4	6
13616	13613	13619	-	14676	-	-	-	2	0	2
13616	13613	13619	-	14682	-	-	-	2	0	2
13616	13613	13619	-	14696	-	-	-	2	0	2
13623	13620	13623	-	14833	-	-	+	2	0	2
13623	13622	13624	+	13643	-	-	-	2	1	3

13623	13622	13624	+	13775 -	-		+	2	0	2
13629	13624	13635	-	13782	13782	13784	-	2	2	4
13629	13624	13635	-	13816	13814	13816	-	2	2	4
13639	13637	13642	-	14616 -	-		-	2	3	5
13644	13643	13648	-	14022 -	-		-	2	0	2
13644	13643	13648	-	14227 -	-		+	2	6	8
13644	13643	13648	-	14538	14537	14538	+	2	6	8
13651	13649	13653	-	13788 -	-		-	2	2	4
13651	13648	13653	+	14877 -	-		-	2	2	4
13659	13655	13660	-	14599 -	-		+	2	0	2
13664	13661	13668	-	13775	13775	13778	-	2	3	5
13664	13661	13668	-	14169	14167	14169	-	2	1	3
13664	13661	13668	-	14637	14637	14638	-	2	1	3
13674	13669	13678	-	14222 -	-		-	2	0	2
13674	13669	13678	-	14433	14433	14435	-	2	2	4
13681	13677	13683	+	13776 -	-		-	2	0	2
13681	13677	13683	+	14201 -	-		+	2	0	2
13684	13680	13687	-	14200 -	-		-	2	0	2
13684	13680	13687	-	14641 -	-		+	2	0	2
13700	13697	13700	-	13856 -	-		+	2	0	2
13700	13697	13700	-	13870 -	-		-	2	0	2
13700	13697	13700	-	14056 -	-		+	2	2	4
13700	13697	13700	-	14143 -	-		-	2	2	4
13709	13704	13713	-	13877 -	-		+	2	0	2
13709	13704	13713	-	14423 -	-		+	2	5	7
13709	13704	13713	-	14435 -	-		-	2	2	4
13709	13704	13713	-	14769	14767	14769	+	2	1	3
13715 -	-		-	14625 -	-		+	2	0	2
13720	13717	13722	-	14189 -	-		+	2	1	3
13726 -	-		-	13758 -	-		+	2	0	2
13731	13728	13734	-	13933 -	-		-	2	0	2
13731	13728	13734	-	14563 -	-		+	2	0	2
13738	13733	13743	+	13773 -	-		-	2	0	2
13741	13737	13744	-	14439 -	-		+	2	0	2
13741	13737	13744	-	14706 -	-		-	2	0	2
13741	13737	13744	-	14729 -	-		-	2	1	3
13751	13748	13755	-	13729 -	-		+	2	0	2
13759	13757	13760	-	14335 -	-		-	2	0	2
13761	13757	13765	+	13726 -	-		-	2	0	2
13761	13757	13765	+	14331	14328	14331	+	2	0	2
13764	13763	13764	-	14515 -	-		-	2	2	4
13770	13766	13775	-	14350 -	-		+	2	2	4
13770	13766	13775	-	14445 -	-		-	2	0	2
13770	13766	13775	-	14841 -	-		-	2	0	2
13776	13771	13780	+	13829 -	-		-	2	1	3
13776	13771	13780	+	14333 -	-		+	2	1	3
13776	13771	13780	+	14848 -	-		+	2	0	2
13779	13776	13782	-	14264	14264	14265	+	2	2	4
13779	13776	13782	-	15044 -	-		-	2	0	2
13784	13784	13785	-	13787 -	-		+	2	0	2
13784	13784	13785	-	14814 -	-		-	2	0	2
13791	13789	13794	-	15219 -	-		-	2	0	2
13800	13798	13803	+	13884 -	-		-	2	0	2
13800	13798	13803	+	14265 -	-		+	2	0	2
13810	13807	13811	-	13983 -	-		-	2	1	3
13814	13814	13816	+	14414 -	-		+	2	0	2
13826	13825	13831	-	14038 -	-		+	2	0	2
13848	13843	13850	-	13901 -	-		+	2	2	4
13851	13849	13856	+	13866 -	-		-	2	0	2
13853	13851	13857	-	14533 -	-		-	2	0	2
13861 -	-		+	13879 -	-		-	2	0	2
13863	13859	13866	-	13876	13876	13879	+	2	4	6
13868	13864	13871	+	14262 -	-		+	2	0	2
13870	13867	13871	-	14019 -	-		+	2	2	4
13870	13867	13871	-	14239 -	-		+	2	0	2
13870	13867	13871	-	15237 -	-		+	2	1	3
13876	13872	13879	-	14017	14014	14019	+	2	4	6
13876	13872	13879	-	14675	14672	14675	-	2	3	5
13878	13874	13882	+	14987 -	-		+	2	1	3
13885	13880	13891	-	13906 -	-		+	2	1	3
13885	13880	13891	-	14128 -	-		+	2	0	2
13891	13888	13891	+	13867	13864	13869	-	2	2	4
13891	13888	13891	+	14862 -	-		+	2	2	4
13900	13898	13903	-	13986 -	-		+	2	0	2
13908	13906	13912	-	13885 -	-		+	2	2	4
13908	13906	13912	-	14717 -	-		+	2	1	3
13908	13906	13912	-	15202	15202	15204	+	2	3	5
13916	13915	13920	+	13890	13890	13891	-	2	0	2
13921	13917	13925	-	14023 -	-		-	2	2	4
13936	13932	13940	-	14045 -	-		+	2	0	2
13941 -	-		+	14966 -	-		+	2	0	2
13947	13941	13951	-	14740	14739	14740	+	2	1	3
13953	13952	13956	-	14804	14801	14805	+	2	4	6
13972	13972	13973	-	14995	14993	14995	-	2	0	2

13988	13985	13989	-	14477	14477	14478	+	2	3	5
13988	13985	13989	-	14563	-	-	-	2	2	4
13988	13985	13989	-	14707	-	-	+	2	0	2
13988	13985	13989	-	15251	-	-	+	2	2	4
14001	13997	14005	-	14704	-	-	-	2	0	2
14001	13997	14005	-	15060	-	-	+	2	0	2
14002	-	-	+	14130	-	-	-	2	0	2
14019	14017	14021	-	14919	-	-	-	2	0	2
14020	14019	14020	+	14160	-	-	+	2	0	2
14035	14034	14036	-	14628	-	-	+	2	1	3
14035	14034	14036	-	14655	-	-	-	2	0	2
14038	14034	14039	+	14290	-	-	-	2	0	2
14046	14044	14048	-	14421	-	-	+	2	1	3
14046	14044	14048	-	15061	15061	15062	+	2	4	6
14056	14054	14061	-	14648	-	-	-	2	0	2
14056	14054	14061	-	14662	-	-	+	2	1	3
14065	14062	14069	-	14531	14531	14533	+	2	4	6
14079	14077	14083	-	14341	-	-	-	2	0	2
14097	14096	14099	+	14882	-	-	+	2	0	2
14108	14106	14112	-	14200	14200	14201	-	2	1	3
14108	14106	14112	-	14707	14707	14709	+	2	2	4
14120	14114	14126	-	14234	-	-	-	2	1	3
14120	14114	14126	-	14706	14705	14706	+	2	1	3
14120	14114	14126	-	14837	-	-	+	2	0	2
14138	14133	14141	-	14759	14759	14760	+	2	2	4
14138	14133	14141	-	14917	-	-	-	2	0	2
14143	14141	14143	+	14197	14197	14198	-	2	0	2
14154	14150	14156	-	14191	-	-	-	2	0	2
14154	14150	14156	-	14339	-	-	-	2	0	2
14154	14150	14156	-	14768	14765	14768	-	2	3	5
14159	14157	14160	-	14848	-	-	+	2	6	8
14164	14161	14166	-	14450	-	-	+	2	1	3
14164	14161	14166	-	14859	-	-	-	2	2	4
14172	14171	14177	+	14658	-	-	-	2	0	2
14174	14170	14180	-	14775	14775	14776	+	2	3	5
14174	14170	14180	-	14782	-	-	-	2	0	2
14174	14170	14180	-	14803	14799	14804	-	2	1	3
14174	14170	14180	-	15253	15253	15256	+	2	3	5
14187	14183	14189	-	14673	-	-	+	2	2	4
14187	14183	14189	-	14677	-	-	-	2	2	4
14187	14183	14189	-	14851	-	-	+	2	2	4
14194	14192	14194	+	14652	-	-	-	2	0	2
14200	14200	14201	+	14602	-	-	+	2	0	2
14207	14207	14208	-	15020	-	-	+	2	0	2
14207	14206	14211	+	14359	14357	14359	+	2	2	4
14212	14211	14215	-	15057	-	-	+	2	0	2
14212	14211	14215	-	15169	15165	15169	+	2	19	21
14223	14219	14227	-	15207	15205	15207	-	2	0	2
14238	14235	14243	-	14236	-	-	+	2	1	3
14238	14235	14243	-	14385	-	-	+	2	3	5
14238	14235	14243	-	14586	-	-	-	2	0	2
14238	14235	14243	-	14706	-	-	-	2	0	2
14238	14235	14243	-	15023	15023	15024	-	2	0	2
14238	14235	14243	-	15153	-	-	+	2	7	9
14238	14235	14243	-	15272	-	-	+	2	0	2
14239	14238	14243	+	15177	-	-	+	2	0	2
14248	14245	14252	-	14430	14427	14430	+	2	1	3
14248	14245	14252	-	14433	14433	14434	-	2	2	4
14248	14245	14252	-	14968	-	-	+	2	3	5
14260	14260	14261	-	15183	-	-	-	2	2	4
14265	14263	14271	-	14926	14925	14926	+	2	3	5
14284	14282	14289	-	14459	-	-	+	2	0	2
14284	14282	14289	-	14494	-	-	+	2	2	4
14284	14282	14289	-	14777	-	-	+	2	0	2
14288	14286	14288	+	14330	14330	14332	-	2	1	3
14294	14291	14295	-	14435	-	-	-	2	0	2
14294	14291	14295	-	14873	-	-	+	2	0	2
14300	14297	14301	-	14935	14932	14935	-	2	5	7
14300	14297	14301	-	14951	-	-	+	2	13	15
14307	14302	14308	-	14330	14330	14331	+	2	2	4
14307	14302	14308	-	14472	-	-	-	2	0	2
14307	14302	14308	-	14914	-	-	+	2	3	5
14307	14302	14308	-	15268	-	-	+	2	8	10
14316	14314	14318	-	14438	14436	14438	-	2	0	2
14316	14314	14318	-	14749	14747	14749	+	2	0	2
14324	14320	14324	-	15018	-	-	+	2	0	2
14350	14347	14353	-	14758	-	-	-	2	0	2
14350	14347	14353	-	14763	-	-	-	2	0	2
14360	14355	14365	-	14572	-	-	-	2	4	6
14360	14355	14365	-	14576	-	-	+	2	0	2
14364	14360	14364	+	14375	-	-	-	2	0	2
14369	14366	14375	-	14439	-	-	+	2	0	2
14369	14366	14375	-	14886	14886	14887	-	2	2	4
14369	14366	14375	-	15031	15031	15033	+	2	2	4

14370	14365	14373	+	15211	15209	15211	+	2	2	4
14375	14374	14379	+	14890	14889	14890	+	2	3	5
14377	14377	14380	-	14802 -	-		-	2	0	2
14384	14383	14385	-	15005 -	-		+	2	3	5
14391	14390	14393	-	14512	14509	14512	-	2	0	2
14398	14395	14398	-	15120 -	-		-	2	0	2
14403	14399	14407	-	15187 -	-		+	2	0	2
14403	14399	14407	-	15206 -	-		-	2	0	2
14409	14407	14413	+	14448 -	-		-	2	0	2
14423	14419	14423	-	14967 -	-		+	2	0	2
14435	14435	14438	-	14891 -	-		+	2	4	6
14452	14448	14455	+	14455	14453	14455	+	2	0	2
14452	14448	14455	+	14535 -	-		-	2	0	2
14456	14452	14461	-	14572 -	-		-	2	0	2
14456	14452	14461	-	14664 -	-		-	2	0	2
14456	14452	14461	-	15027 -	-		-	2	0	2
14462	14457	14466	+	14897	14897	14898	+	2	2	4
14464	14462	14468	-	14849 -	-		+	2	2	4
14464	14462	14468	-	15087 -	-		+	2	1	3
14470	14469	14475	+	14452 -	-		-	2	2	4
14470	14469	14475	+	14462	14462	14463	-	2	1	3
14470	14469	14475	+	14688 -	-		+	2	0	2
14470	14469	14475	+	14750	14749	14750	+	2	1	3
14479	14474	14479	-	14983 -	-		+	2	0	2
14485	14482	14492	-	14540	14540	14542	+	2	1	3
14485	14482	14492	-	14633 -	-		-	2	2	4
14485	14482	14492	-	14875 -	-		-	2	0	2
14495	14494	14499	-	14680	14677	14680	+	2	5	7
14504	14501	14508	-	14914 -	-		-	2	0	2
14510	14507	14512	+	14484 -	-		-	2	0	2
14510	14507	14512	+	14651 -	-		-	2	0	2
14518	14512	14521	-	14890 -	-		-	2	2	4
14518	14512	14521	-	14895 -	-		+	2	0	2
14526	14524	14530	-	14721 -	-		+	2	2	4
14526	14524	14530	-	14781	14777	14781	+	2	1	3
14526	14524	14530	-	14890 -	-		-	2	1	3
14532	14531	14533	+	14641 -	-		-	2	0	2
14540	14535	14540	-	15116 -	-		+	2	0	2
14545	14540	14548	+	14497	14496	14498	-	2	2	4
14559	14556	14559	-	15037 -	-		-	2	2	4
14559	14556	14559	-	15042 -	-		-	2	1	3
14559	14556	14559	-	15125	15125	15127	+	2	2	4
14561	14556	14564	+	14622	14622	14623	+	2	0	2
14561	14556	14564	+	15384 -	-		-	2	4	6
14565	14560	14568	-	14861 -	-		-	2	0	2
14565	14560	14568	-	15194 -	-		-	2	0	2
14570	14569	14573	-	14662 -	-		+	2	2	4
14570	14569	14573	-	15063	15061	15063	+	2	2	4
14578	14576	14583	-	15251	15249	15251	-	2	5	7
14589	14584	14593	+	14634 -	-		-	2	0	2
14589	14584	14593	+	14849 -	-		-	2	0	2
14589	14584	14593	+	15384	15383	15384	-	2	4	6
14595	14592	14596	-	14654 -	-		+	2	1	3
14595	14592	14596	-	15066 -	-		-	2	0	2
14605	14601	14609	+	14565 -	-		+	2	0	2
14605	14601	14609	+	15120 -	-		+	2	0	2
14607	14603	14610	-	15122 -	-		-	2	0	2
14612	14611	14615	+	14690 -	-		+	2	0	2
14617	14617	14620	+	15013 -	-		+	2	0	2
14622	14619	14626	-	14633 -	-		+	2	0	2
14622	14619	14626	-	14998 -	-		-	2	0	2
14622	14619	14626	-	15070 -	-		+	2	4	6
14632	14628	14632	+	14650 -	-		-	2	0	2
14632	14628	14632	+	15023 -	-		-	2	0	2
14638	14638	14641	-	15043 -	-		+	2	3	5
14638	14634	14640	+	14685	14685	14687	-	2	1	3
14646	14646	14647	-	15231 -	-		-	2	0	2
14651	14650	14654	+	15158 -	-		+	2	0	2
14653	14649	14656	-	14676	14675	14676	+	2	24	26
14653	14649	14656	-	15070 -	-		+	2	0	2
14660 -	-		-	15384 -	-		-	2	2	4
14660	14655	14661	+	15164	15163	15164	+	2	3	5
14676	14672	14680	-	14789 -	-		-	2	0	2
14676	14672	14679	+	14692 -	-		-	2	0	2
14681	14680	14684	+	14726 -	-		-	2	0	2
14693	14688	14697	-	14787 -	-		+	2	0	2
14693	14688	14697	-	14824 -	-		-	2	1	3
14700	14698	14700	+	15129 -	-		-	2	0	2
14704	14700	14708	-	14787 -	-		-	2	0	2
14704	14700	14708	-	14864	14864	14866	-	2	1	3
14711	14711	14712	+	14812 -	-		+	2	0	2
14716	14716	14719	-	15033	15031	15033	+	2	1	3
14725	14723	14725	-	15037 -	-		-	2	1	3
14725	14723	14725	-	15063	15061	15063	-	2	3	5

14730	14726	14733	-	15066	-	-	+	2	1	3
14739	14734	14743	-	15055	-	-	+	2	1	3
14739	14735	14743	+	14852	-	-	-	2	0	2
14739	14735	14743	+	15064	-	-	+	2	0	2
14761	14759	14764	-	14838	14836	14838	+	2	0	2
14761	14759	14764	-	14884	-	-	-	2	0	2
14761	14759	14764	-	14913	14911	14913	-	2	3	5
14761	14759	14764	-	14928	14928	14929	-	2	0	2
14767	14765	14771	-	14911	14907	14911	-	2	0	2
14769	14765	14770	+	14818	14818	14819	-	2	2	4
14786	14781	14789	-	15041	-	-	-	2	0	2
14789	14784	14789	+	15068	-	-	+	2	0	2
14794	14792	14797	+	14866	-	-	+	2	1	3
14805	14801	14808	+	14874	14872	14874	-	2	0	2
14806	14801	14809	-	14937	-	-	-	2	0	2
14832	14832	14836	+	15204	15204	15206	-	2	1	3
14838	14836	14841	-	14929	14929	14930	+	2	6	8
14838	14836	14841	-	15057	-	-	+	2	2	4
14842	14838	14842	+	14858	-	-	-	2	2	4
14842	14838	14842	+	15197	-	-	-	2	0	2
14847	14842	14851	-	15035	-	-	+	2	0	2
14847	14842	14851	-	15093	15093	15094	-	2	1	3
14847	14842	14851	-	15154	-	-	-	2	0	2
14847	14842	14851	-	15163	15163	15164	-	2	7	9
14847	14844	14850	+	15061	-	-	-	2	0	2
14857	14854	14859	+	14838	14834	14838	-	2	8	10
14857	14854	14859	+	15032	-	-	-	2	0	2
14859	14857	14862	-	15027	15025	15028	-	2	2	4
14864	14863	14864	-	15174	-	-	+	2	3	5
14864	14860	14865	+	14961	-	-	+	2	0	2
14870	14866	14870	+	14879	-	-	-	2	0	2
14870	14866	14870	+	15176	-	-	+	2	0	2
14877	14873	14879	-	15015	15015	15018	+	2	4	6
14877	14872	14882	+	14900	14898	14900	-	2	1	3
14877	14872	14882	+	14978	-	-	+	2	0	2
14877	14872	14882	+	15015	15015	15019	-	2	6	8
14877	14872	14882	+	15194	-	-	+	2	2	4
14882	14880	14887	-	15018	-	-	+	2	1	3
14882	14880	14887	-	15204	-	-	+	2	0	2
14889	-	-	-	15047	-	-	-	2	2	4
14894	14890	14897	-	14992	14991	14992	-	2	2	4
14894	14890	14897	-	15043	-	-	+	2	2	4
14894	14890	14897	-	15047	15044	15047	-	2	2	4
14895	14890	14900	+	14870	-	-	-	2	9	11
14905	14899	14908	-	14952	14952	14954	+	2	14	16
14905	14899	14908	-	14970	-	-	-	2	0	2
14905	14899	14908	-	15103	-	-	+	2	2	4
14905	14899	14908	-	15117	-	-	+	2	0	2
14905	14899	14908	-	15151	15151	15152	+	2	2	4
14905	14903	14907	+	14976	14976	14977	+	2	2	4
14905	14903	14907	+	15032	-	-	+	2	2	4
14910	14909	14913	-	15001	-	-	-	2	1	3
14910	14909	14913	-	15033	-	-	-	2	0	2
14910	14909	14913	-	15083	15080	15083	+	2	1	3
14921	14920	14926	-	15211	-	-	-	2	2	4
14933	14931	14936	-	15157	15157	15159	+	2	13	15
14933	14931	14936	-	15271	-	-	+	2	2	4
14935	14934	14939	+	15128	-	-	+	2	0	2
14943	14942	14946	+	15163	15160	15163	-	2	1	3
14947	14943	14953	-	15019	-	-	-	2	0	2
14947	14943	14953	-	15104	15101	15108	-	2	7	9
14947	14943	14953	-	15116	15115	15117	-	2	10	12
14957	14956	14960	-	15115	-	-	-	2	0	2
14957	14956	14960	-	15210	-	-	-	2	0	2
14960	14955	14964	+	15140	-	-	-	2	0	2
14960	14955	14964	+	15159	-	-	+	2	0	2
14964	14962	14968	-	15068	15065	15068	-	2	0	2
14974	14969	14978	-	15044	-	-	-	2	2	4
14974	14969	14978	-	15202	-	-	-	2	2	4
14978	14978	14982	+	14955	-	-	-	2	0	2
14978	14978	14982	+	15180	-	-	+	2	0	2
14986	14984	14988	+	15026	-	-	+	2	0	2
15002	14997	15003	-	15052	15052	15053	-	2	7	9
15002	14997	15003	-	15072	15072	15076	+	2	5	7
15014	15010	15016	-	15066	-	-	-	2	0	2
15014	15010	15016	-	15165	15161	15166	-	2	3	5
15015	15010	15018	+	15023	-	-	+	2	0	2
15025	15020	15027	-	15206	-	-	-	2	2	4
15030	15028	15032	-	15079	15077	15079	+	2	4	6
15042	15040	15042	+	15100	-	-	-	2	0	2
15053	15051	15055	-	15108	-	-	+	2	5	7
15053	15051	15055	-	15129	15129	15133	-	2	1	3
15053	15051	15055	-	15148	15148	15149	-	2	4	6
15053	15053	15057	+	15108	-	-	-	2	2	4

15070	15069	15071	+	15093	15091	15093	-	2	0	2
15072	15068	15076	-	15125	15122	15129	-	2	16	18
15076	15072	15079	+	15048	-	-	-	2	0	2
15078	15077	15083	-	15228	-	-	-	2	0	2
15085	15084	15087	-	15107	-	-	+	2	5	7
15092	15088	15096	+	15072	15072	15074	-	2	2	4
15092	15088	15096	+	15293	-	-	-	2	0	2
15102	15098	15105	+	15121	-	-	+	2	2	4
15102	15098	15105	+	15125	15125	15126	-	2	0	2
15102	15098	15105	+	15145	-	-	+	2	4	6
15104	15099	15107	-	15141	15139	15142	-	2	3	5
15111	15106	15111	+	15139	-	-	-	2	0	2
15116	15112	15119	+	15085	15084	15085	-	2	5	7
15116	15112	15119	+	15098	-	-	-	2	7	9
15143	15140	15148	+	15111	-	-	-	2	1	3
15152	15149	15153	+	15168	-	-	-	2	0	2
15160	15156	15163	+	15148	15148	15149	-	2	2	4
15169	15165	15171	+	15205	15201	15205	+	2	0	2
15177	15173	15177	+	15186	-	-	-	2	0	2
15177	15173	15177	+	15204	15201	15204	+	2	1	3
15183	15182	15183	-	15246	-	-	-	2	2	4
15185	15181	15190	+	15239	15236	15239	-	2	0	2
15205	15200	15208	+	15171	15171	15173	-	2	12	14
15205	15200	15208	+	15240	-	-	-	2	0	2
15206	15204	15208	-	15270	-	-	-	2	0	2
15212	15210	15212	+	15191	15191	15195	-	2	3	5
15217	15214	15220	+	15229	-	-	+	2	0	2
15217	15214	15220	+	15234	15230	15234	-	2	0	2
15241	15240	15246	+	15228	-	-	-	2	2	4
15260	15258	15260	+	15258	15258	15260	-	2	3	5
15262	-	-	-	15342	-	-	-	2	2	4
15271	15271	15272	-	15384	-	-	-	2	0	2
15279	15275	15279	-	15382	15379	15383	-	2	1	3
15284	15282	15290	-	15314	-	-	+	2	0	2
15314	-	-	-	15283	-	-	+	2	0	2
1	-	-	-	15242	-	-	+	1	1	2
57	56	57	+	94	94	95	-	1	20	21
113	112	116	+	1435	1435	1436	+	1	1	2
130	129	131	+	14289	-	-	+	1	1	2
130	129	131	+	14572	-	-	+	1	3	4
150	146	150	+	13793	-	-	+	1	3	4
228	228	230	+	260	-	-	-	1	2	3
231	228	232	-	260	257	260	+	1	1	2
235	235	238	+	250	250	251	-	1	1	2
260	257	261	+	228	228	231	-	1	2	3
266	263	270	-	14607	14607	14608	+	1	1	2
273	272	273	-	14433	-	-	-	1	2	3
278	276	283	-	346	-	-	-	1	1	2
323	321	323	+	14213	-	-	+	1	1	2
368	366	368	-	410	-	-	+	1	1	2
368	366	368	-	573	-	-	-	1	1	2
370	369	373	+	415	-	-	+	1	1	2
373	372	374	-	584	584	585	-	1	1	2
415	-	-	+	13291	-	-	+	1	1	2
448	-	-	-	641	-	-	-	1	1	2
482	482	483	-	653	653	654	-	1	1	2
488	484	489	+	1459	-	-	+	1	6	7
506	505	506	+	12566	12566	12567	+	1	1	2
534	534	536	+	8898	-	-	+	1	1	2
545	-	-	-	1108	-	-	-	1	1	2
563	563	564	+	1810	-	-	+	1	1	2
569	-	-	-	944	-	-	-	1	1	2
569	-	-	+	4844	-	-	+	1	1	2
655	655	658	+	917	-	-	+	1	1	2
684	-	-	-	827	-	-	-	1	1	2
684	682	686	+	705	705	706	+	1	1	2
689	688	693	+	10822	-	-	+	1	1	2
707	707	708	+	14649	-	-	-	1	1	2
722	721	723	+	13461	13459	13461	+	1	1	2
736	734	736	+	15010	-	-	+	1	2	3
737	734	737	-	806	-	-	-	1	1	2
748	748	749	+	14332	-	-	+	1	1	2
770	769	775	+	13069	-	-	+	1	2	3
770	769	775	+	13404	-	-	+	1	3	4
770	769	775	+	14065	-	-	+	1	5	6
797	793	797	-	13466	-	-	+	1	1	2
831	-	-	+	955	-	-	+	1	1	2
884	880	884	+	1395	-	-	+	1	1	2
884	880	884	+	14987	-	-	+	1	1	2
935	932	939	+	11237	11234	11237	+	1	1	2
955	-	-	-	1108	-	-	-	1	1	2
974	970	974	-	1121	1117	1121	-	1	1	2
979	979	980	+	12319	-	-	+	1	1	2
991	988	991	+	14896	-	-	+	1	2	3

1021	1020	1025	+	13543	13543	13544	+	1	1	2
1054	1051	1057	-	1242 -	-		-	1	1	2
1073 -	-		+	14225 -	-		+	1	1	2
1082	1078	1082	+	14710	14710	14711	+	1	6	7
1120	1120	1121	+	14266	14266	14267	+	1	2	3
1131 -	-		+	13587 -	-		+	1	1	2
1141	1138	1145	+	13380	13380	13381	+	1	1	2
1142	1139	1142	-	1327	1327	1328	-	1	4	5
1142	1139	1142	-	1601	1599	1601	-	1	1	2
1149	1149	1152	-	1203 -	-		-	1	1	2
1149	1149	1152	-	1339 -	-		-	1	1	2
1149	1149	1152	-	1795 -	-		-	1	1	2
1187 -	-		-	1261 -	-		-	1	1	2
1242	1239	1244	-	1436 -	-		-	1	1	2
1285	1285	1286	-	1363	1363	1364	-	1	1	2
1318	1315	1318	-	1440	1437	1440	-	1	1	2
1396	1396	1399	-	1461	1459	1462	+	1	2	3
1467	1467	1471	+	1546	1542	1546	+	1	1	2
1485 -	-		+	13918 -	-		+	1	1	2
1498	1498	1499	+	13989	13989	13990	+	1	1	2
1615	1611	1616	+	14107	14107	14108	-	1	10	11
1665	1665	1668	+	1707	1707	1710	+	1	7	8
1677 -	-		-	15163 -	-		+	1	1	2
1788 -	-		-	13748 -	-		+	1	1	2
1793	1792	1796	+	14550 -	-		+	1	1	2
1801	1801	1805	+	11374 -	-		+	1	1	2
1801	1801	1805	+	13909 -	-		+	1	2	3
1803 -	-		-	13741 -	-		+	1	1	2
1809	1809	1811	+	14300	14300	14302	+	1	5	6
1835 -	-		+	14915 -	-		+	1	3	4
1840 -	-		+	1879 -	-		+	1	1	2
1852	1848	1856	+	14332 -	-		+	1	2	3
1863	1860	1866	+	14071 -	-		+	1	1	2
1886	1883	1891	+	13646 -	-		+	1	1	2
1900	1896	1901	+	14666	14666	14667	+	1	3	4
1908	1904	1911	+	1973 -	-		-	1	2	3
1908	1904	1911	+	12042	12042	12043	+	1	1	2
1908	1904	1911	+	13818 -	-		+	1	1	2
1917	1915	1917	+	1981	1981	1983	+	1	2	3
1917	1915	1917	+	13849 -	-		+	1	1	2
1932	1927	1935	+	1949 -	-		+	1	1	2
1944	1941	1949	+	11248 -	-		+	1	1	2
1944	1941	1949	+	14045 -	-		+	1	3	4
1944	1941	1949	+	14638 -	-		-	1	1	2
1959	1953	1960	+	13773 -	-		+	1	1	2
1959	1953	1960	+	14650	14650	14651	+	1	2	3
1988	1988	1990	+	2118	2118	2119	+	1	1	2
2018	2014	2018	-	2309 -	-		-	1	1	2
2023 -	-		-	2055 -	-		+	1	1	2
2023	2022	2024	+	13159	13159	13160	+	1	1	2
2023	2022	2024	+	13736 -	-		+	1	2	3
2028	2028	2031	+	14751 -	-		+	1	1	2
2055	2052	2056	+	8947	8947	8948	+	1	1	2
2065	2065	2066	+	15165 -	-		-	1	2	3
2075	2072	2076	+	15156 -	-		+	1	1	2
2085	2081	2085	+	2112 -	-		-	1	1	2
2086	2085	2086	-	2183	2183	2185	-	1	2	3
2119	2117	2120	+	11551 -	-		+	1	1	2
2119	2117	2120	+	13950 -	-		+	1	1	2
2131	2127	2136	+	12165 -	-		+	1	1	2
2190	2187	2194	+	2212	2209	2212	-	1	1	2
2198	2196	2203	+	14384 -	-		+	1	3	4
2213	2213	2215	+	12521 -	-		+	1	1	2
2258	2257	2258	+	12067 -	-		+	1	1	2
2276	2271	2280	+	14965 -	-		+	1	1	2
2284	2282	2284	-	13431 -	-		+	1	1	2
2298	2294	2301	+	12432	12432	12433	+	1	1	2
2325	2322	2325	+	13281 -	-		+	1	1	2
2344	2341	2344	+	12069 -	-		+	1	1	2
2344	2341	2344	+	13167 -	-		+	1	1	2
2351 -	-		-	2795 -	-		-	1	1	2
2357 -	-		+	12911 -	-		+	1	3	4
2370	2367	2373	+	12896	12896	12897	+	1	1	2
2370	2367	2373	+	13422	13422	13423	+	1	2	3
2386	2386	2389	+	14249 -	-		+	1	1	2
2455	2454	2459	+	13976 -	-		+	1	1	2
2457	2457	2461	-	2534 -	-		-	1	1	2
2473	2471	2477	+	13123 -	-		+	1	1	2
2473	2471	2477	+	14412 -	-		+	1	1	2
2488	2486	2489	+	13229 -	-		+	1	1	2
2488	2486	2489	+	13457 -	-		+	1	1	2
2500	2500	2501	+	13907 -	-		+	1	1	2
2506	2505	2507	+	9718	9718	9719	+	1	5	6
2532 -	-		+	13080 -	-		+	1	2	3

2583	2579	2583	+	14259	-	-	+	1	1	2
2588	2588	2592	+	15005	15001	15005	-	1	1	2
2620	2620	2622	+	15064	15064	15066	+	1	3	4
2641	2639	2645	+	11557	-	-	+	1	1	2
2647	-	-	-	13224	-	-	+	1	1	2
2680	2677	2684	+	13898	-	-	+	1	1	2
2771	2768	2772	+	9976	9976	9977	-	1	1	2
2788	2785	2791	+	10195	-	-	+	1	1	2
2827	2827	2832	+	2809	-	-	-	1	1	2
2827	2827	2832	+	13448	-	-	+	1	1	2
2828	2827	2828	-	12709	-	-	+	1	1	2
2845	2843	2845	+	14539	-	-	+	1	2	3
2861	2858	2862	+	12352	-	-	+	1	1	2
2861	2858	2862	+	13674	-	-	+	1	2	3
2866	-	-	+	14782	-	-	+	1	1	2
2879	2879	2880	-	14950	14950	14951	-	1	13	14
2915	2914	2915	+	14416	14416	14417	+	1	1	2
2927	2924	2931	+	2980	-	-	-	1	1	2
2927	2924	2931	+	13779	-	-	+	1	1	2
2927	2924	2931	+	14750	-	-	+	1	1	2
2941	2938	2941	+	2962	-	-	-	1	2	3
2941	2938	2941	+	3124	-	-	+	1	1	2
2960	2960	2961	+	3011	3010	3011	-	1	2	3
2975	2975	2979	+	14808	-	-	+	1	1	2
3098	-	-	+	3065	-	-	-	1	2	3
3105	-	-	-	3134	-	-	+	1	1	2
3142	3140	3143	+	14273	14270	14273	+	1	1	2
3166	3165	3169	+	14104	-	-	+	1	2	3
3175	3171	3175	+	9753	-	-	+	1	1	2
3198	3196	3199	+	13246	13245	13246	+	1	2	3
3227	3227	3231	+	14490	-	-	-	1	2	3
3279	3275	3283	+	12421	12418	12421	+	1	1	2
3294	3294	3296	+	13320	13320	13322	+	1	2	3
3317	3313	3317	+	14740	-	-	+	1	1	2
3322	3322	3326	+	9713	-	-	+	1	3	4
3322	3322	3326	+	13741	-	-	+	1	1	2
3341	3340	3343	+	6005	6002	6005	+	1	1	2
3362	-	-	+	13721	-	-	+	1	1	2
3373	3370	3377	+	10649	-	-	+	1	1	2
3401	3401	3405	+	3422	3418	3422	+	1	1	2
3423	3423	3424	-	3434	3434	3435	+	1	1	2
3475	3473	3475	-	14663	-	-	+	1	1	2
3508	3506	3511	+	12220	12220	12221	+	1	1	2
3546	3546	3551	+	14646	14642	14646	+	1	1	2
3566	3565	3570	+	10454	-	-	+	1	2	3
3576	-	-	-	4032	-	-	-	1	1	2
3661	3659	3661	+	11701	11700	11701	+	1	2	3
3678	-	-	-	3712	-	-	-	1	1	2
3704	-	-	-	7334	-	-	+	1	1	2
3712	3710	3712	+	14206	14204	14206	+	1	1	2
3720	3720	3721	-	7317	-	-	+	1	1	2
3789	-	-	+	12165	-	-	+	1	1	2
3835	3835	3836	+	13688	-	-	+	1	1	2
3855	-	-	+	10827	-	-	+	1	1	2
3911	-	-	+	12257	-	-	+	1	1	2
3911	-	-	+	14501	-	-	+	1	1	2
3954	-	-	+	12984	-	-	+	1	1	2
3972	3972	3976	+	13920	13920	13921	+	1	1	2
3987	3986	3987	+	11001	-	-	+	1	1	2
4017	4013	4017	-	4098	-	-	-	1	1	2
4017	4013	4017	-	12417	-	-	+	1	1	2
4105	4103	4107	+	14080	-	-	+	1	1	2
4143	4143	4145	-	4296	4296	4297	-	1	1	2
4146	4145	4146	+	10037	-	-	+	1	2	3
4212	4212	4215	+	13302	-	-	+	1	2	3
4212	4212	4215	+	14230	-	-	+	1	1	2
4217	4217	4221	-	14390	-	-	+	1	1	2
4288	-	-	+	8458	-	-	+	1	1	2
4346	4345	4346	+	12349	12349	12350	+	1	3	4
4346	4345	4346	+	14000	-	-	+	1	4	5
4374	-	-	+	14705	-	-	+	1	1	2
4438	4435	4442	+	13059	-	-	+	1	1	2
4457	4455	4460	+	15053	-	-	+	1	2	3
4488	4484	4488	+	4476	-	-	+	1	4	5
4488	4484	4488	+	11919	-	-	+	1	2	3
4488	4484	4488	+	12810	-	-	+	1	3	4
4498	4498	4499	+	13288	13288	13289	+	1	1	2
4591	4588	4592	+	12544	-	-	+	1	1	2
4591	4588	4592	+	14318	-	-	+	1	1	2
4610	4610	4611	+	11437	-	-	-	1	1	2
4629	4629	4631	+	10079	-	-	+	1	1	2
4648	4646	4648	+	14819	-	-	+	1	5	6
4654	-	-	+	8572	-	-	+	1	1	2
4663	4663	4665	-	5084	-	-	-	1	1	2

4772 -	-		+	12703 -	-		+	1	1	2
4774 -	-		-	5086 -	-		-	1	1	2
4805	4802	4806	+	10484	10484	10485	+	1	1	2
4815	4815	4818	-	14410	14407	14410	+	1	1	2
4879	4877	4879	+	4859 -	-		-	1	1	2
4879	4877	4879	+	13507 -	-		+	1	1	2
4921	4921	4922	+	13093 -	-		+	1	3	4
4935 -	-		+	13659 -	-		+	1	3	4
4979	4979	4983	-	5013	5013	5017	-	1	10	11
5018 -	-		+	13981 -	-		+	1	1	2
5110 -	-		+	5134 -	-		+	1	1	2
5142 -	-		-	5322 -	-		-	1	1	2
5193	5193	5195	+	10977 -	-		+	1	1	2
5193	5193	5195	+	13942 -	-		+	1	1	2
5242 -	-		-	14109 -	-		+	1	1	2
5295 -	-		+	12903 -	-		+	1	5	6
5358	5354	5361	+	13404 -	-		+	1	5	6
5358	5354	5361	+	15384 -	-		-	1	1	2
5560	5560	5561	+	11428 -	-		+	1	1	2
5606	5606	5607	+	14264	14264	14265	+	1	2	3
5724 -	-		+	15190 -	-		-	1	4	5
5785	5784	5789	+	15191	15190	15191	-	1	3	4
5874	5873	5874	+	14292 -	-		+	1	1	2
5914 -	-		-	14289 -	-		+	1	1	2
6225 -	-		+	13309 -	-		+	1	1	2
6257	6256	6261	+	14731 -	-		+	1	1	2
6275 -	-		+	8186 -	-		+	1	1	2
6291	6289	6292	+	6328 -	-		+	1	1	2
6302	6302	6303	+	6350 -	-		+	1	1	2
6307	6306	6307	+	13326 -	-		+	1	1	2
6356 -	-		+	10694 -	-		+	1	1	2
6403	6403	6406	+	12061	12061	12064	+	1	3	4
6542	6542	6544	+	12291	12291	12292	+	1	1	2
6561	6561	6563	-	6751	6749	6751	-	1	1	2
6635 -	-		-	13058 -	-		+	1	1	2
6711 -	-		+	11463 -	-		+	1	1	2
6774 -	-		-	15043 -	-		+	1	1	2
6775 -	-		+	13783 -	-		+	1	1	2
6788	6788	6790	+	14288	14288	14290	+	1	5	6
6794 -	-		-	15029 -	-		+	1	5	6
6821	6821	6824	-	13830	13827	13830	-	1	1	2
6863 -	-		-	13303 -	-		+	1	1	2
6941 -	-		+	6959 -	-		-	1	1	2
7138 -	-		+	14978 -	-		-	1	5	6
7239	7238	7243	-	7386	7386	7387	-	1	1	2
7239	7238	7243	-	9378 -	-		+	1	1	2
7365 -	-		-	14289 -	-		+	1	1	2
7400 -	-		+	7411 -	-		+	1	1	2
7417 -	-		+	14329 -	-		+	1	1	2
7479	7479	7483	-	11206 -	-		+	1	1	2
7517	7515	7517	+	13649 -	-		+	1	1	2
7636 -	-		+	7882 -	-		+	1	1	2
7728 -	-		-	7872 -	-		-	1	1	2
7817	7817	7822	+	14528	14525	14528	-	1	1	2
7817	7817	7822	+	15070 -	-		+	1	1	2
7844	7844	7845	+	14602	14599	14602	+	1	1	2
7872 -	-		+	11652 -	-		+	1	1	2
8111 -	-		+	8124 -	-		+	1	1	2
8233	8232	8234	-	15312	15312	15313	-	1	9	10
8545 -	-		+	8571 -	-		-	1	1	2
8572 -	-		-	11373 -	-		+	1	1	2
8616	8615	8616	-	15133	15133	15134	+	1	2	3
8645 -	-		-	15152 -	-		+	1	1	2
8786	8782	8786	+	15174	15170	15174	+	1	1	2
8787 -	-		-	12779 -	-		+	1	1	2
8832	8831	8835	-	14490 -	-		+	1	1	2
8854	8852	8854	-	14621	14619	14621	+	1	1	2
8968	8968	8969	-	12165 -	-		+	1	2	3
8969	8969	8971	+	14666	14666	14669	+	1	2	3
8998	8996	8999	-	13628 -	-		+	1	2	3
9017	9016	9017	-	14774 -	-		+	1	1	2
9038 -	-		-	14580 -	-		-	1	1	2
9052	9051	9052	-	12141 -	-		+	1	1	2
9052	9051	9052	-	14019 -	-		+	1	1	2
9060	9060	9061	+	9984	9984	9985	+	1	1	2
9086	9086	9090	+	14149 -	-		+	1	2	3
9100	9098	9103	-	11847 -	-		+	1	6	7
9280	9280	9284	-	14775 -	-		+	1	4	5
9400	9397	9400	-	14103 -	-		+	1	2	3
9414	9412	9414	-	13544	13544	13545	+	1	1	2
9439 -	-		-	14214 -	-		+	1	4	5
9446	9444	9446	-	14976 -	-		+	1	1	2
9574	9570	9575	-	14576	14576	14577	+	1	2	3
9590	9589	9593	-	13811 -	-		+	1	1	2

9600	9600	9601	-	13872 -	-		+	1	1	2
9600	9600	9601	-	14600 -	-		+	1	1	2
9606	9606	9608	-	13345 -	-		+	1	1	2
9623	9623	9625	-	15278 -	-		+	1	2	3
9666 -	-		-	13218 -	-		+	1	1	2
9674	9671	9674	-	12569 -	-		+	1	1	2
9900 -	-		-	10003 -	-		-	1	1	2
9916	9916	9920	-	14873 -	-		+	1	1	2
9916	9916	9920	-	14961 -	-		+	1	1	2
9942 -	-		+	14109	14108	14109	+	1	9	10
9945	9945	9948	-	14113	14112	14114	-	1	4	5
9984	9984	9988	-	12261 -	-		+	1	2	3
10036	10035	10038	-	13350 -	-		-	1	5	6
10106	10106	10108	-	12982	12982	12984	+	1	2	3
10114	10114	10115	+	13123 -	-		+	1	1	2
10126 -	-		-	14547 -	-		+	1	1	2
10151	10150	10151	-	15062	15062	15063	+	1	1	2
10156	10156	10157	-	14294 -	-		+	1	1	2
10230	10228	10230	+	15095	15093	15095	+	1	1	2
10311 -	-		-	15250 -	-		+	1	2	3
10343	10341	10343	-	14078	14078	14080	+	1	3	4
10370	10366	10373	-	14934 -	-		+	1	1	2
10370	10366	10373	-	15348	15348	15349	-	1	1	2
10383	10379	10388	-	14498 -	-		-	1	1	2
10472	10471	10473	-	14164	14164	14166	+	1	3	4
10504	10504	10507	-	14636 -	-		+	1	3	4
10543	10541	10544	-	14334 -	-		+	1	2	3
10581	10578	10583	-	13432	13432	13433	+	1	1	2
10581	10578	10583	-	13810 -	-		+	1	2	3
10645	10644	10645	-	14154	14154	14155	+	1	2	3
10684	10682	10685	-	11998	11998	11999	-	1	1	2
10684	10682	10685	-	14756	14756	14757	+	1	1	2
10692	10692	10694	+	14879	14876	14879	-	1	2	3
10697	10694	10699	-	11887 -	-		+	1	1	2
10697	10694	10699	-	12635 -	-		+	1	1	2
10706 -	-		+	14864 -	-		-	1	1	2
10725	10723	10726	-	15288 -	-		+	1	1	2
10823	10819	10827	-	13518 -	-		+	1	1	2
10831	10830	10835	-	13728	13728	13729	+	1	1	2
10847	10843	10847	-	14717 -	-		+	1	4	5
10862	10859	10862	-	13163	13163	13165	+	1	2	3
10885	10885	10886	-	13871	13871	13872	+	1	2	3
10900 -	-		-	13827 -	-		+	1	2	3
10935 -	-		-	14501 -	-		+	1	2	3
10969	10965	10969	-	13926 -	-		+	1	2	3
10993	10991	10996	-	14169	14169	14170	-	1	1	2
11020	11018	11020	-	14547 -	-		+	1	1	2
11028	11027	11032	-	13334	13331	13334	+	1	1	2
11070 -	-		-	12344 -	-		+	1	3	4
11097	11092	11099	-	15141	15141	15142	+	1	7	8
11127	11127	11128	-	13413 -	-		+	1	1	2
11127	11127	11128	-	14962 -	-		+	1	1	2
11149 -	-		+	13536 -	-		+	1	1	2
11164	11163	11164	-	14241	14241	14242	+	1	1	2
11189 -	-		-	12590 -	-		+	1	1	2
11195	11191	11198	-	13125 -	-		+	1	1	2
11218	11213	11218	-	13739	13739	13742	+	1	2	3
11239	11239	11241	-	13988 -	-		-	1	1	2
11239	11239	11241	-	14785	14785	14786	-	1	2	3
11258	11258	11261	-	13037 -	-		+	1	1	2
11276	11273	11277	-	13984 -	-		+	1	1	2
11290	11288	11294	-	12759 -	-		+	1	1	2
11355	11355	11357	-	13780 -	-		+	1	1	2
11400 -	-		-	14189 -	-		-	1	1	2
11419	11418	11422	-	13880	13880	13881	-	1	1	2
11446	11443	11447	-	15175	15172	15175	-	1	6	7
11451	11450	11456	-	12866 -	-		+	1	1	2
11463	11460	11463	+	14316	14316	14317	+	1	1	2
11468	11464	11472	-	15178 -	-		-	1	1	2
11494	11492	11498	-	12543 -	-		+	1	1	2
11494	11492	11498	-	14145 -	-		+	1	1	2
11510	11506	11514	-	13400 -	-		+	1	1	2
11510	11506	11514	-	14164	14164	14165	+	1	1	2
11510	11506	11514	-	14343 -	-		+	1	3	4
11556	11556	11557	-	12066 -	-		+	1	1	2
11665	11664	11669	-	12846	12846	12847	+	1	1	2
11665	11664	11669	-	14049 -	-		+	1	2	3
11678 -	-		-	14531 -	-		+	1	1	2
11687	11686	11687	-	12872 -	-		+	1	1	2
11687	11686	11687	-	14294	14294	14295	+	1	1	2
11701 -	-		-	13740 -	-		+	1	1	2
11713	11712	11715	-	14707 -	-		+	1	1	2
11758	11754	11761	-	14620 -	-		+	1	1	2
11774	11769	11774	-	13320 -	-		+	1	1	2

11782	11781	11786	-	14343 -	-		+	1	1	2
11793	11788	11794	-	13247	13247	13248	+	1	2	3
11793	11788	11794	-	14740	14738	14742	+	1	11	12
11836	11835	11839	-	12696 -	-		+	1	1	2
11847	11844	11851	-	13795	13792	13795	-	1	1	2
11847	11844	11851	-	14711 -	-		-	1	4	5
11866	11863	11867	-	14187 -	-		+	1	2	3
11866	11863	11867	-	14488 -	-		+	1	1	2
11866	11863	11867	-	15017 -	-		+	1	1	2
11890	11890	11891	-	13791 -	-		+	1	1	2
11920	11916	11926	-	14565 -	-		+	1	2	3
11931	11928	11931	-	14225 -	-		+	1	1	2
11932	11929	11934	+	11953 -	-		-	1	3	4
11950	11947	11950	-	13942 -	-		+	1	1	2
11964	11964	11967	-	14106 -	-		+	1	1	2
11978	11978	11979	+	14732 -	-		+	1	1	2
12002	11999	12002	-	13345 -	-		+	1	1	2
12002	11999	12002	-	14475 -	-		-	1	4	5
12018	12016	12021	-	13224	13224	13225	+	1	2	3
12026	12023	12029	-	13208 -	-		+	1	1	2
12040	12037	12044	-	14760 -	-		+	1	1	2
12053	12052	12057	-	14184 -	-		+	1	1	2
12061	12059	12063	-	14543 -	-		+	1	1	2
12069	12065	12069	-	12579 -	-		+	1	1	2
12075	12070	12076	-	15044 -	-		+	1	2	3
12094	12090	12094	-	12445 -	-		+	1	5	6
12113	12109	12116	-	14435 -	-		-	1	2	3
12139	12136	12141	-	12256 -	-		-	1	1	2
12139	12136	12141	-	13785 -	-		+	1	1	2
12161	12159	12162	-	14180 -	-		+	1	1	2
12161	12159	12162	-	14318 -	-		+	1	1	2
12161	12159	12162	-	14827	14827	14828	+	1	1	2
12167	12166	12172	-	14360 -	-		+	1	1	2
12168	12168	12169	+	12217	12217	12218	-	1	1	2
12187	12183	12190	-	14148	14146	14148	-	1	1	2
12187	12183	12190	-	14518 -	-		+	1	3	4
12187	12183	12190	-	15113 -	-		-	1	1	2
12204	12203	12208	-	12317	12313	12317	-	1	1	2
12212 -	-		-	14525	14525	14527	+	1	3	4
12233	12230	12234	-	12725 -	-		+	1	1	2
12274	12274	12276	-	13901 -	-		+	1	1	2
12282	12279	12282	-	12986 -	-		+	1	4	5
12291	12289	12293	-	13384 -	-		+	1	3	4
12291	12289	12293	-	13587 -	-		+	1	1	2
12299	12299	12303	+	13397	13393	13397	+	1	1	2
12307	12303	12310	-	13147 -	-		+	1	1	2
12321	12318	12326	-	14591 -	-		+	1	1	2
12321	12318	12326	-	14634 -	-		+	1	1	2
12337 -	-		+	14598 -	-		+	1	1	2
12349	12345	12349	+	12308 -	-		-	1	1	2
12349	12345	12349	+	15271	15270	15271	+	1	2	3
12364	12360	12365	-	13148 -	-		+	1	3	4
12364	12360	12365	-	14294 -	-		+	1	1	2
12383	12380	12386	-	14525	14524	14525	+	1	2	3
12389	12388	12390	-	14025 -	-		+	1	1	2
12407	12402	12411	-	13645	13645	13646	+	1	2	3
12407	12402	12411	-	14651 -	-		+	1	3	4
12408	12408	12409	+	12677	12677	12678	+	1	1	2
12418	12418	12423	-	14613 -	-		+	1	1	2
12433	12431	12436	-	14146	14146	14147	+	1	1	2
12465	12461	12467	-	13028	13028	13029	+	1	1	2
12465	12461	12467	-	14464 -	-		+	1	1	2
12495	12495	12496	-	14518 -	-		+	1	1	2
12503	12499	12503	+	14320 -	-		+	1	1	2
12514	12513	12515	-	12653 -	-		-	1	1	2
12520	12517	12522	-	15207	15204	15207	-	1	1	2
12525	12524	12525	-	14565 -	-		+	1	4	5
12531	12527	12531	-	13292	13290	13292	+	1	1	2
12536	12535	12542	-	12603 -	-		+	1	1	2
12553	12549	12557	-	13545 -	-		+	1	1	2
12553	12549	12557	-	14253 -	-		+	1	2	3
12560	12560	12561	-	14605 -	-		-	1	1	2
12577	12573	12581	-	14212 -	-		-	1	2	3
12596	12596	12598	-	13373 -	-		+	1	1	2
12605	12599	12607	-	12836	12834	12836	+	1	1	2
12605	12599	12607	-	12976 -	-		+	1	1	2
12605	12599	12607	-	13919 -	-		-	1	1	2
12605	12599	12607	-	14567 -	-		+	1	2	3
12641	12639	12641	-	14835 -	-		+	1	2	3
12652	12652	12656	-	13298 -	-		+	1	1	2
12652	12652	12656	-	14391 -	-		+	1	4	5
12662	12659	12666	-	14339	14339	14342	+	1	5	6
12662	12658	12662	+	13592 -	-		-	1	1	2
12676	12672	12678	-	13593 -	-		+	1	6	7

12676	12672	12678	-	14193	-	-	+	1	1	2
12696	12692	12699	-	13598	-	-	-	1	1	2
12696	12692	12699	-	15265	-	-	+	1	7	8
12712	12708	12712	-	13897	-	-	+	1	3	4
12732	12728	12734	-	14892	-	-	-	1	1	2
12739	12737	12743	-	13470	13470	13472	+	1	1	2
12739	12737	12743	-	13949	13949	13951	+	1	2	3
12745	12744	12749	-	13344	13344	13345	+	1	1	2
12765	12765	12766	-	12802	-	-	+	1	1	2
12775	12771	12780	-	15087	-	-	+	1	8	9
12802	12801	12807	-	13832	13830	13832	+	1	1	2
12802	12801	12807	-	14411	14411	14412	-	1	1	2
12812	12812	12816	+	14935	-	-	+	1	1	2
12828	12825	12828	-	13027	-	-	+	1	1	2
12828	12825	12828	-	13810	-	-	+	1	1	2
12828	12825	12828	-	14382	-	-	+	1	2	3
12834	12834	12835	+	12929	12929	12930	-	1	1	2
12838	12837	12841	-	12883	-	-	-	1	2	3
12838	12837	12841	-	14067	-	-	+	1	5	6
12843	12843	12846	-	14463	-	-	+	1	1	2
12903	12900	12905	-	14476	-	-	+	1	5	6
12911	12906	12914	-	14023	-	-	-	1	4	5
12911	12906	12914	-	14740	-	-	+	1	1	2
12931	12930	12935	+	14720	-	-	-	1	2	3
12931	12930	12935	+	14980	-	-	+	1	2	3
12940	12936	12945	-	13793	-	-	-	1	1	2
12940	12936	12945	-	14177	-	-	+	1	4	5
12940	12936	12945	-	14360	-	-	+	1	1	2
12949	12948	12949	-	13123	-	-	+	1	1	2
12956	12952	12958	-	14981	-	-	+	1	1	2
12965	12965	12968	-	13851	-	-	-	1	1	2
12970	12967	12970	+	14063	-	-	-	1	3	4
12973	12970	12979	-	14323	14322	14323	+	1	3	4
12978	12973	12978	+	12999	-	-	-	1	1	2
12978	12973	12978	+	15095	15092	15095	+	1	1	2
12990	12990	12994	-	14892	-	-	+	1	1	2
13000	12997	13002	-	13587	-	-	-	1	1	2
13013	13009	13017	-	13741	13741	13742	-	1	1	2
13013	13009	13017	-	14530	14530	14531	+	1	2	3
13021	13019	13021	+	13136	-	-	+	1	1	2
13025	13023	13029	-	14222	14222	14226	+	1	2	3
13025	13023	13029	-	14369	-	-	+	1	4	5
13031	13030	13032	-	14264	14264	14265	+	1	2	3
13031	13031	13032	+	13113	13113	13115	+	1	3	4
13036	13035	13038	-	13413	-	-	+	1	3	4
13044	13040	13049	-	13795	-	-	+	1	7	8
13044	13040	13049	-	15074	-	-	+	1	1	2
13055	13053	13058	-	13246	-	-	+	1	1	2
13060	13059	13064	-	13835	-	-	-	1	4	5
13060	13059	13064	-	14297	-	-	+	1	1	2
13071	13067	13076	+	13143	13143	13144	+	1	1	2
13089	13087	13090	-	14345	-	-	+	1	1	2
13094	13091	13095	-	13416	-	-	+	1	1	2
13094	13091	13095	-	13873	13870	13873	+	1	1	2
13094	13091	13095	-	14266	-	-	+	1	2	3
13111	13109	13111	-	14371	-	-	-	1	1	2
13126	13123	13129	-	14293	14293	14294	+	1	1	2
13126	13123	13129	-	14591	-	-	+	1	1	2
13141	13138	13145	-	13318	13315	13318	-	1	3	4
13147	13146	13151	-	14289	-	-	+	1	4	5
13177	13174	13177	-	14535	-	-	-	1	1	2
13182	13180	13182	-	13892	13890	13892	-	1	7	8
13184	-	-	+	13965	-	-	+	1	1	2
13187	13183	13190	-	15021	-	-	+	1	2	3
13187	13183	13190	-	15062	-	-	+	1	2	3
13199	13196	13203	-	15158	-	-	+	1	1	2
13209	13205	13209	-	13830	13828	13830	+	1	1	2
13209	13205	13209	-	14716	14716	14717	+	1	1	2
13212	13212	13213	+	13227	-	-	+	1	1	2
13212	13212	13213	+	13855	-	-	-	1	1	2
13218	13218	13221	-	14707	-	-	+	1	1	2
13227	13224	13230	-	13285	-	-	+	1	1	2
13227	13224	13230	-	14377	-	-	+	1	2	3
13227	13224	13230	-	14676	14676	14677	+	1	6	7
13246	13241	13247	-	14261	14260	14261	-	1	3	4
13246	13241	13247	-	14763	-	-	+	1	1	2
13246	13241	13247	-	15010	-	-	+	1	2	3
13252	13251	13256	-	14630	-	-	+	1	2	3
13265	13262	13269	-	13570	-	-	+	1	3	4
13272	13270	13276	-	13912	-	-	-	1	1	2
13278	13277	13282	-	13635	13632	13635	-	1	1	2
13297	13293	13302	-	13593	13591	13593	+	1	1	2
13297	13293	13302	-	14980	14980	14982	+	1	1	2
13308	13303	13312	-	13847	-	-	+	1	1	2

13308	13303	13312	-	14032 -	-		-	1	1	2
13308	13303	13312	-	14271	14271	14272	-	1	1	2
13308	13303	13312	-	14711 -	-		-	1	1	2
13308	13303	13312	-	14867 -	-		-	1	6	7
13308	13303	13312	-	14870	14868	14870	+	1	3	4
13308	13303	13312	-	14895	14892	14898	-	1	3	4
13316	13313	13322	-	15029	15029	15030	+	1	3	4
13317	13315	13320	+	15167	15167	15168	+	1	1	2
13326	13324	13327	-	13780 -	-		-	1	1	2
13330	13326	13336	+	14894	14893	14894	+	1	2	3
13330	13326	13336	+	15093 -	-		+	1	4	5
13332	13328	13336	-	13411 -	-		+	1	1	2
13332	13328	13336	-	13496 -	-		-	1	1	2
13332	13328	13336	-	14373 -	-		-	1	1	2
13332	13328	13336	-	14487 -	-		+	1	1	2
13359	13356	13363	-	13447 -	-		-	1	1	2
13365	13365	13368	-	13604 -	-		-	1	2	3
13365	13365	13368	-	14008 -	-		+	1	1	2
13394	13389	13397	-	14169	14169	14172	-	1	1	2
13399	13399	13401	-	14391	14391	14392	-	1	2	3
13408	13404	13411	-	14314 -	-		+	1	1	2
13416	13413	13420	-	13833 -	-		+	1	1	2
13416	13413	13420	-	14095 -	-		+	1	1	2
13446	13443	13446	-	13709 -	-		+	1	1	2
13451	13448	13454	-	14647 -	-		-	1	1	2
13462	13458	13467	-	13679 -	-		+	1	1	2
13471	13468	13475	+	15064	15064	15066	+	1	10	11
13473	13469	13474	-	14519 -	-		+	1	1	2
13479	13475	13483	-	13689	13689	13690	-	1	1	2
13479	13475	13483	-	14182 -	-		-	1	1	2
13479	13475	13483	-	14638 -	-		+	1	1	2
13479	13475	13483	-	15099 -	-		+	1	1	2
13488	13485	13490	-	13700 -	-		-	1	1	2
13488	13485	13490	-	14612	14612	14613	+	1	2	3
13505 -	-		+	15054 -	-		-	1	2	3
13510	13506	13513	+	15093	15091	15093	+	1	3	4
13516	13511	13517	-	14300 -	-		+	1	1	2
13516	13511	13517	-	14426	14426	14429	+	1	2	3
13516	13511	13517	-	14749 -	-		-	1	2	3
13516	13511	13517	-	15267 -	-		+	1	1	2
13523	13521	13525	-	13646	13646	13647	+	1	1	2
13523	13521	13525	-	13965	13965	13966	+	1	1	2
13544	13538	13545	-	14498 -	-		-	1	3	4
13544	13538	13545	-	14514 -	-		-	1	5	6
13544	13538	13545	-	14878	14878	14880	+	1	12	13
13549	13548	13553	-	14488 -	-		-	1	3	4
13549	13548	13553	-	14637 -	-		-	1	7	8
13549	13548	13553	-	14663 -	-		+	1	1	2
13555	13554	13558	-	13973 -	-		+	1	1	2
13555	13554	13558	-	14560 -	-		+	1	1	2
13562	13559	13567	-	14561 -	-		-	1	22	23
13562	13559	13567	-	14574	14573	14575	-	1	4	5
13562	13559	13567	-	14677 -	-		-	1	1	2
13566	13562	13569	+	14005 -	-		+	1	1	2
13569	13568	13570	-	14583 -	-		-	1	1	2
13569	13568	13570	-	14836 -	-		+	1	1	2
13578	13574	13583	-	13596 -	-		+	1	1	2
13578	13574	13583	-	13694	13694	13695	+	1	1	2
13578	13574	13583	-	14046 -	-		+	1	1	2
13578	13574	13583	-	15061 -	-		+	1	1	2
13581	13579	13585	+	14868 -	-		+	1	1	2
13581	13579	13585	+	15061 -	-		-	1	1	2
13587	13584	13591	-	14219 -	-		+	1	1	2
13593	13593	13597	-	14193 -	-		+	1	1	2
13599	13596	13599	+	14680 -	-		-	1	1	2
13605	13602	13606	-	14200	14199	14200	-	1	2	3
13605	13602	13606	-	14410	14410	14411	+	1	4	5
13605	13602	13606	-	14619	14619	14620	-	1	1	2
13616	13613	13619	-	13701 -	-		+	1	1	2
13616	13613	13619	-	13862 -	-		+	1	3	4
13616	13613	13619	-	14840	14840	14841	+	1	2	3
13623	13620	13623	-	14106 -	-		+	1	2	3
13629	13624	13635	-	13829 -	-		-	1	2	3
13629	13624	13635	-	14537	14535	14537	+	1	1	2
13629	13624	13635	-	14867 -	-		+	1	1	2
13639	13637	13642	-	14444 -	-		+	1	1	2
13639	13637	13642	-	14635 -	-		+	1	1	2
13644	13643	13648	-	14214 -	-		+	1	9	10
13644	13643	13648	-	14530	14530	14532	+	1	2	3
13651	13649	13653	-	14230 -	-		-	1	1	2
13651	13649	13653	-	14381 -	-		+	1	1	2
13651	13649	13653	-	14622 -	-		-	1	1	2
13651	13648	13653	+	14029	14028	14029	+	1	10	11
13656	13655	13660	+	13700	13696	13700	-	1	7	8

13659	13655	13660	-	13986	13986	13987	+	1	1	2
13659	13655	13660	-	14445 -	-		-	1	5	6
13659	13655	13660	-	14660 -	-		-	1	2	3
13668 -	-		+	14187 -	-		+	1	1	2
13674	13669	13678	-	13758 -	-		-	1	1	2
13674	13669	13678	-	13790 -	-		-	1	1	2
13674	13669	13678	-	13839 -	-		-	1	1	2
13674	13669	13678	-	13937 -	-		+	1	2	3
13688	13687	13688	+	15019	15018	15019	+	1	3	4
13695	13693	13695	-	14784	14782	14784	+	1	1	2
13700	13697	13700	-	14261 -	-		+	1	1	2
13720	13717	13722	-	13911 -	-		+	1	1	2
13731	13728	13734	-	14089 -	-		+	1	3	4
13731	13728	13734	-	14939 -	-		+	1	1	2
13738	13733	13743	+	13748	13748	13751	-	1	3	4
13738	13733	13743	+	13799	13795	13799	-	1	1	2
13738	13733	13743	+	14535 -	-		+	1	3	4
13741	13737	13744	-	14377 -	-		+	1	1	2
13751	13748	13755	-	14053 -	-		+	1	1	2
13751	13748	13755	-	14336 -	-		-	1	1	2
13751	13748	13755	-	14350 -	-		+	1	5	6
13751	13748	13755	-	14377 -	-		-	1	1	2
13751	13748	13755	-	14743 -	-		-	1	1	2
13759	13757	13760	-	14544	14544	14546	+	1	9	10
13770	13766	13775	-	14456 -	-		-	1	2	3
13770	13766	13775	-	14482 -	-		+	1	1	2
13770	13766	13775	-	14848 -	-		+	1	1	2
13779	13776	13782	-	14527 -	-		+	1	1	2
13779	13776	13782	-	14595	14591	14595	-	1	1	2
13791	13789	13794	-	14105	14102	14105	+	1	2	3
13799	13796	13804	-	14085 -	-		-	1	1	2
13830	13829	13833	+	13808 -	-		-	1	1	2
13842	13839	13842	-	14119 -	-		-	1	1	2
13842	13839	13842	-	14550 -	-		+	1	1	2
13848	13843	13850	-	14046	14044	14046	-	1	1	2
13848	13843	13850	-	15278	15278	15279	+	1	1	2
13851	13849	13856	+	14868 -	-		+	1	3	4
13853	13851	13857	-	15045 -	-		+	1	2	3
13863	13859	13866	-	14110 -	-		-	1	1	2
13863	13859	13866	-	14341 -	-		+	1	1	2
13863	13859	13866	-	14662 -	-		+	1	2	3
13878	13874	13882	+	14350 -	-		-	1	1	2
13885	13880	13891	-	13915 -	-		+	1	1	2
13885	13885	13886	+	14987	14987	14988	+	1	1	2
13891	13888	13891	+	13915 -	-		-	1	2	3
13893	13893	13895	-	14169	14167	14169	-	1	1	2
13893	13893	13895	-	15110 -	-		-	1	1	2
13908	13906	13912	-	14693 -	-		+	1	5	6
13908	13906	13912	-	15232 -	-		-	1	1	2
13916	13914	13916	-	14065 -	-		+	1	1	2
13921	13917	13925	-	14133	14133	14134	-	1	1	2
13921	13917	13925	-	14660 -	-		-	1	1	2
13927	13925	13927	+	13942 -	-		+	1	1	2
13936	13932	13940	-	14422	14422	14423	+	1	1	2
13936	13932	13940	-	14578 -	-		+	1	6	7
13947	13941	13951	-	14619 -	-		+	1	3	4
13988	13985	13989	-	14137	14134	14137	+	1	1	2
13992	13988	13992	+	14405 -	-		+	1	1	2
14001	13997	14005	-	14084	14081	14084	-	1	1	2
14001	13997	14005	-	14694 -	-		-	1	1	2
14012	14008	14012	-	14208 -	-		+	1	1	2
14012	14008	14012	-	14613 -	-		+	1	3	4
14012	14008	14012	-	14806 -	-		+	1	4	5
14025	14023	14029	-	14356 -	-		-	1	1	2
14025	14023	14029	-	14651 -	-		+	1	1	2
14025	14023	14029	-	15003	15000	15003	-	1	1	2
14029	14029	14033	+	14033 -	-		+	1	1	2
14035	14034	14036	-	14773 -	-		+	1	1	2
14038	14034	14039	+	14336 -	-		+	1	1	2
14038	14034	14039	+	14360	14356	14360	+	1	1	2
14040	14039	14043	-	14402 -	-		+	1	1	2
14040	14039	14043	-	14642 -	-		-	1	1	2
14056	14054	14061	-	14597	14597	14598	-	1	1	2
14073	14071	14073	-	14292	14290	14295	+	1	4	5
14079	14077	14083	-	14318 -	-		+	1	1	2
14092	14089	14095	+	14888	14887	14891	+	1	4	5
14096	14096	14101	-	14622 -	-		-	1	2	3
14108	14106	14112	-	14215	14215	14217	+	1	3	4
14108	14106	14112	-	14640	14638	14640	-	1	2	3
14108	14106	14112	-	14647 -	-		+	1	1	2
14120	14114	14126	-	14206 -	-		-	1	1	2
14120	14114	14126	-	14866 -	-		+	1	1	2
14138	14133	14141	-	14476	14476	14480	-	1	3	4
14138	14133	14141	-	14628 -	-		-	1	1	2

14164	14161	14166	-	14180	-	-	-				1	7	8
14164	14161	14166	-	14730	14730	14731	+				1	1	2
14164	14161	14166	-	14776	-	-	-				1	1	2
14174	14170	14180	-	14501	14500	14501	+				1	2	3
14174	14170	14180	-	14566	-	-	+				1	1	2
14174	14170	14180	-	14747	14747	14748	+				1	1	2
14174	14170	14180	-	14907	-	-	+				1	1	2
14187	14183	14189	-	14572	-	-	-				1	1	2
14187	14183	14189	-	14975	-	-	+				1	2	3
14187	14183	14189	-	15063	-	-	+				1	4	5
14200	14200	14201	+	15057	15057	15058	-				1	1	2
14202	14201	14204	-	15266	-	-	+				1	3	4
14207	14207	14208	-	15029	-	-	-				1	1	2
14215	-	-	+	14705	-	-	+				1	5	6
14223	14219	14227	-	15170	-	-	+				1	1	2
14228	14228	14229	+	14769	-	-	+				1	1	2
14229	14228	14233	-	15107	-	-	-				1	10	11
14229	14228	14233	-	15165	-	-	+				1	4	5
14238	14235	14243	-	14718	-	-	+				1	1	2
14238	14235	14243	-	14730	14730	14731	+				1	3	4
14245	14245	14246	+	15147	-	-	-				1	2	3
14248	14245	14252	-	15147	-	-	+				1	3	4
14254	14253	14257	-	15145	-	-	+				1	1	2
14260	14260	14261	-	15203	-	-	-				1	1	2
14265	14263	14271	-	14795	14795	14797	+				1	2	3
14284	14282	14289	-	14546	14546	14547	+				1	1	2
14294	14291	14295	-	15088	15088	15090	+				1	2	3
14296	14292	14299	+	14355	-	-	-				1	1	2
14296	14292	14299	+	14932	-	-	+				1	1	2
14300	14297	14301	-	14474	14474	14475	-				1	1	2
14300	14297	14301	-	14837	-	-	+				1	3	4
14303	14302	14305	+	14326	-	-	-				1	1	2
14324	14320	14324	-	14966	14966	14967	+				1	3	4
14331	14328	14337	-	14812	-	-	-				1	1	2
14331	14328	14337	-	15122	-	-	+				1	1	2
14342	14339	14346	-	14462	14462	14463	-				1	1	2
14342	14339	14346	-	14813	14813	14815	+				1	2	3
14342	14339	14346	-	14858	14858	14860	+				1	1	2
14344	14340	14348	+	14375	-	-	-				1	2	3
14344	14340	14348	+	14467	14467	14468	+				1	3	4
14350	14347	14353	-	14864	-	-	-				1	1	2
14350	14347	14353	-	14966	14966	14967	+				1	1	2
14358	14353	14358	+	14370	14369	14370	+				1	2	3
14358	14353	14358	+	15128	-	-	+				1	1	2
14360	14355	14365	-	14777	-	-	+				1	1	2
14360	14355	14365	-	14815	-	-	-				1	2	3
14364	14360	14364	+	14580	14579	14580	+				1	4	5
14369	14366	14375	-	14574	14574	14575	+				1	1	2
14369	14366	14375	-	14868	-	-	-				1	1	2
14369	14366	14375	-	14894	-	-	-				1	2	3
14369	14366	14375	-	15081	-	-	+				1	1	2
14369	14366	14375	-	15205	-	-	+				1	1	2
14403	14399	14407	-	14933	-	-	-				1	1	2
14409	14408	14411	-	14490	-	-	-				1	1	2
14409	14408	14411	-	15186	15186	15187	-				1	1	2
14409	14407	14413	+	14450	-	-	+				1	1	2
14416	14414	14416	-	14537	-	-	+				1	3	4
14416	14414	14416	-	14876	14873	14876	-				1	1	2
14428	14428	14432	-	14631	-	-	+				1	3	4
14428	14424	14429	+	14464	14464	14465	-				1	27	28
14428	14424	14429	+	14472	14470	14472	+				1	1	2
14435	14435	14438	-	15036	15035	15036	+				1	2	3
14435	14432	14438	+	14621	-	-	+				1	1	2
14443	14442	14447	-	14461	14461	14462	+				1	1	2
14451	14449	14451	-	14511	14511	14513	-				1	2	3
14456	14452	14461	-	14784	-	-	-				1	1	2
14456	14452	14461	-	14807	-	-	+				1	1	2
14456	14452	14461	-	14872	-	-	-				1	1	2
14456	14452	14461	-	15184	-	-	+				1	1	2
14462	14457	14466	+	14488	-	-	+				1	1	2
14464	14462	14468	-	14842	14842	14843	-				1	1	2
14464	14462	14468	-	15202	-	-	+				1	5	6
14479	14474	14479	-	15030	-	-	+				1	3	4
14479	14478	14482	+	14457	-	-	-				1	1	2
14479	14478	14482	+	14463	14463	14464	-				1	3	4
14479	14478	14482	+	14885	-	-	+				1	1	2
14487	14484	14491	+	14510	14507	14510	-				1	1	2
14495	14494	14499	-	14555	14555	14556	+				1	1	2
14501	14496	14505	+	14668	14664	14668	-				1	1	2
14501	14496	14505	+	14744	-	-	+				1	1	2
14504	14501	14508	-	14529	14525	14532	+				1	2	3
14504	14501	14508	-	15103	-	-	+				1	1	2
14518	14512	14521	-	14526	-	-	+				1	6	7
14518	14512	14521	-	14841	14838	14841	+				1	1	2

14518	14512	14521	-	15010		15010	15011	+	1	2	3
14532	14531	14533	+	15121	-	-		+	1	1	2
14540	14535	14540	-	14693	-	-		+	1	1	2
14547	14545	14550	-	14649	-	-		-	1	1	2
14547	14545	14550	-	14674		14670	14674	-	1	1	2
14565	14560	14568	-	14991		14991	14993	+	1	3	4
14565	14560	14568	-	15017	-	-		+	1	1	2
14570	14569	14573	-	14609		14606	14609	+	1	1	2
14570	14569	14573	-	14668		14668	14669	+	1	1	2
14572	14569	14573	+	15055		15054	15055	-	1	3	4
14578	14576	14583	-	14662	-	-		+	1	1	2
14578	14576	14583	-	14828	-	-		+	1	1	2
14580	14579	14582	+	14635	-	-		-	1	3	4
14586	14586	14590	-	14660	-	-		-	1	1	2
14607	14603	14610	-	14718		14714	14718	-	1	2	3
14612	14611	14617	-	14781		14781	14782	-	1	1	2
14612	14611	14615	+	15010		15010	15011	-	1	1	2
14622	14619	14626	-	14968	-	-		-	1	1	2
14622	14619	14626	-	15016	-	-		-	1	1	2
14622	14619	14626	-	15026	-	-		-	1	1	2
14622	14619	14626	-	15039	-	-		-	1	1	2
14633	14632	14636	-	15025	-	-		-	1	1	2
14633	14632	14636	-	15044	-	-		-	1	2	3
14638	14638	14641	-	15153	-	-		+	1	10	11
14646	14645	14649	+	15052		15052	15053	+	1	1	2
14646	14645	14649	+	15164	-	-		+	1	1	2
14651	14650	14654	+	15135	-	-		-	1	1	2
14666	14661	14671	-	14737	-	-		+	1	1	2
14666	14661	14671	-	14917		14917	14918	+	1	2	3
14666	14661	14671	-	14960	-	-		-	1	1	2
14676	14672	14680	-	14780		14780	14783	-	1	2	3
14676	14672	14680	-	15057	-	-		+	1	2	3
14676	14672	14680	-	15188		15188	15189	-	1	4	5
14681	14680	14684	+	14803		14802	14803	+	1	2	3
14683	14681	14686	-	14710		14710	14712	+	1	2	3
14691	14689	14692	+	14703	-	-		+	1	1	2
14693	14688	14697	-	14799	-	-		+	1	1	2
14700	14698	14700	+	15137	-	-		-	1	1	2
14704	14700	14708	-	14802	-	-		-	1	1	2
14716	14716	14719	-	14738	-	-		+	1	1	2
14730	14726	14733	-	14804		14804	14807	-	1	2	3
14730	14726	14733	-	15036		15032	15036	+	1	5	6
14730	14726	14733	-	15079	-	-		-	1	1	2
14739	14734	14743	-	14789	-	-		-	1	4	5
14749	14745	14751	+	15042		15042	15043	+	1	1	2
14761	14759	14764	-	15015	-	-		-	1	2	3
14767	14765	14771	-	14838	-	-		-	1	1	2
14767	14765	14771	-	14901	-	-		-	1	1	2
14767	14765	14771	-	14906		14904	14906	+	1	1	2
14767	14765	14771	-	14925	-	-		-	1	1	2
14767	14765	14771	-	15028		15028	15029	-	1	3	4
14767	14765	14771	-	15203	-	-		-	1	1	2
14769	14765	14770	+	15028	-	-		+	1	2	3
14769	14765	14770	+	15381		15377	15381	-	1	1	2
14786	14781	14789	-	14807		14804	14808	+	1	2	3
14786	14781	14789	-	14913		14913	14915	+	1	1	2
14789	14784	14789	+	14985		14985	14986	+	1	1	2
14791	14790	14793	-	14922		14920	14922	-	1	2	3
14791	14790	14793	-	15206	-	-		-	1	3	4
14812	14812	14817	-	14900		14900	14901	+	1	1	2
14842	14838	14842	+	15030		15030	15032	+	1	4	5
14847	14842	14851	-	15152		15152	15153	+	1	4	5
14854	14852	14856	-	15039	-	-		-	1	1	2
14859	14857	14862	-	15020		15016	15020	-	1	2	3
14859	14857	14862	-	15056		15054	15056	-	1	1	2
14864	14860	14865	+	14836	-	-		-	1	2	3
14869	14865	14872	-	15071		15071	15073	-	1	7	8
14870	14866	14870	+	15080	-	-		+	1	1	2
14877	14873	14879	-	14972		14969	14972	-	1	1	2
14877	14873	14879	-	15019		15019	15022	-	1	1	2
14877	14873	14879	-	15024		15020	15024	+	1	4	5
14877	14872	14882	+	15052		15051	15052	+	1	2	3
14877	14872	14882	+	15205	-	-		+	1	1	2
14894	14890	14897	-	15203		15203	15206	-	1	1	2
14895	14890	14900	+	14864		14860	14864	-	1	2	3
14895	14890	14900	+	14885		14882	14885	-	1	5	6
14895	14890	14900	+	15019	-	-		+	1	1	2
14895	14890	14900	+	15051		15051	15052	+	1	16	17
14905	14899	14908	-	15051		15051	15054	+	1	1	2
14910	14909	14913	-	15028		15028	15029	-	1	1	2
14916	14915	14918	-	14966		14966	14969	+	1	2	3
14916	14915	14918	-	15266	-	-		+	1	1	2
14921	14920	14926	-	15019	-	-		-	1	1	2
14921	14920	14926	-	15140	-	-		-	1	1	2

14922	14919	14922	+	14949	14948	14949	+	1	2	3
14928	14923	14933	+	14941	-	-	-	1	1	2
14928	14923	14933	+	15030	15026	15030	+	1	4	5
14928	14923	14933	+	15224	-	-	+	1	1	2
14941	14939	14942	-	14956	14956	14958	+	1	4	5
14941	14939	14942	-	14995	-	-	+	1	1	2
14941	14939	14942	-	15100	15099	15102	-	1	8	9
14943	14942	14946	+	14905	-	-	-	1	1	2
14943	14942	14946	+	15057	15053	15057	+	1	1	2
14947	14943	14953	-	15011	-	-	-	1	1	2
14951	14949	14954	+	14988	-	-	+	1	1	2
14960	14955	14964	+	15154	15153	15156	+	1	3	4
14964	14962	14968	-	15160	15158	15163	-	1	46	47
14973	14969	14975	+	15169	-	-	+	1	1	2
14974	14969	14978	-	15166	-	-	-	1	1	2
14981	14979	14983	-	15114	15114	15115	-	1	3	4
14986	14984	14988	+	15206	-	-	+	1	1	2
14988	14987	14991	-	15073	-	-	-	1	1	2
15015	15010	15018	+	15044	-	-	+	1	1	2
15015	15010	15018	+	15161	-	-	-	1	1	2
15019	-	-	-	15138	-	-	-	1	2	3
15019	-	-	-	15161	-	-	-	1	1	2
15025	15020	15027	-	15038	15038	15041	+	1	2	3
15025	15020	15027	-	15048	-	-	+	1	9	10
15025	15020	15027	-	15143	-	-	-	1	1	2
15025	15021	15028	+	15060	15057	15060	+	1	1	2
15032	15029	15034	+	15081	15079	15081	-	1	1	2
15037	15034	15040	-	15081	15079	15082	+	1	2	3
15059	15059	15066	-	15078	15078	15079	+	1	1	2
15059	15059	15066	-	15216	15212	15216	-	1	1	2
15062	15059	15068	+	15371	-	-	-	1	3	4
15076	15072	15079	+	15067	15067	15070	-	1	1	2
15076	15072	15079	+	15180	-	-	+	1	1	2
15085	15080	15086	+	15079	15076	15079	-	1	1	2
15092	15088	15096	+	15050	15047	15050	-	1	1	2
15092	15088	15096	+	15055	15054	15055	-	1	2	3
15102	15098	15105	+	15073	-	-	-	1	4	5
15104	15099	15107	-	15135	15135	15136	-	1	1	2
15104	15099	15107	-	15179	-	-	-	1	1	2
15113	-	-	-	15194	-	-	-	1	1	2
15133	15129	15133	+	15103	15103	15104	-	1	1	2
15133	15129	15133	+	15149	15146	15149	-	1	1	2
15138	15135	15138	+	15216	-	-	+	1	1	2
15144	15144	15146	-	15273	15270	15273	-	1	2	3
15150	-	-	-	15211	-	-	-	1	1	2
15152	15149	15153	+	15129	15129	15130	-	1	1	2
15152	15149	15153	+	15182	15180	15182	-	1	1	2
15185	15181	15190	+	15230	-	-	+	1	1	2
15198	-	-	+	15219	-	-	-	1	1	2
15205	15200	15208	+	15283	-	-	+	1	1	2
15206	15204	15208	-	15257	-	-	-	1	1	2
15217	15214	15220	+	15195	-	-	-	1	4	5
15217	15214	15220	+	15285	15282	15285	+	1	1	2
15236	-	-	-	15338	-	-	-	1	1	2
15236	15232	15238	+	15212	15212	15215	-	1	13	14
15384	-	-	+	15382	-	-	-	1	1	2
1	-	-	-	13	-	-	+	0	2	2
88	-	-	+	63	-	-	-	0	4	4
186	186	190	+	14999	14999	15001	+	0	9	9
228	228	230	+	13166	-	-	+	0	2	2
290	288	294	+	12591	-	-	+	0	2	2
298	-	-	-	620	-	-	-	0	2	2
355	-	-	-	6685	-	-	+	0	2	2
434	-	-	-	1551	-	-	-	0	3	3
448	-	-	+	15077	-	-	+	0	3	3
467	-	-	-	1831	-	-	-	0	2	2
479	479	480	+	15078	-	-	+	0	2	2
563	563	564	+	894	-	-	+	0	2	2
581	581	585	-	958	-	-	-	0	3	3
606	604	606	-	867	-	-	-	0	2	2
608	-	-	+	13683	-	-	+	0	2	2
619	619	620	+	13848	-	-	+	0	2	2
633	629	634	+	13641	-	-	+	0	2	2
714	712	716	+	14428	-	-	-	0	2	2
791	-	-	+	1100	-	-	+	0	2	2
961	959	961	-	1015	-	-	-	0	3	3
969	-	-	+	14294	-	-	+	0	2	2
1010	1007	1010	+	1021	1021	1024	-	0	4	4
1106	1106	1107	+	14966	-	-	+	0	3	3
1212	1212	1216	+	1286	-	-	+	0	2	2
1259	-	-	+	1412	-	-	+	0	2	2
1310	-	-	-	4571	-	-	+	0	2	2
1388	-	-	+	1458	-	-	+	0	2	2
1394	-	-	+	1496	-	-	+	0	2	2

1458		1457	1462	-	15172	-	-		-	0	2	2
1564		1564	1565	-	13773	-	-		+	0	2	2
1615		1611	1616	+	1706		1705	1706	+	0	14	14
1621		1619	1625	+	1703	-	-		+	0	5	5
1632		1632	1635	+	1705	-	-		-	0	4	4
1642		1642	1643	-	1672		1672	1673	-	0	15	15
1686	-	-		-	3232	-	-		-	0	3	3
1781	-	-		-	2005	-	-		-	0	3	3
1793		1792	1796	+	13094	-	-		+	0	2	2
1863		1860	1866	+	12228	-	-		+	0	3	3
1863		1860	1866	+	12233	-	-		-	0	3	3
1877	-	-		+	12228	-	-		+	0	3	3
1886		1883	1891	+	15249	-	-		-	0	2	2
1908		1904	1911	+	1901	-	-		+	0	2	2
1908		1904	1911	+	13742	-	-		+	0	2	2
1908		1904	1911	+	14760	-	-		+	0	2	2
1932		1927	1935	+	14566	-	-		+	0	2	2
1944		1941	1949	+	14759	-	-		+	0	4	4
1972		1967	1976	+	13941	-	-		+	0	2	2
1982		1982	1983	+	12537	-	-		+	0	2	2
1985	-	-		-	2132	-	-		-	0	2	2
2028		2028	2031	+	12507	-	-		+	0	6	6
2035	-	-		-	12514	-	-		-	0	2	2
2043		2043	2047	+	11163	-	-		+	0	2	2
2070		2069	2071	-	2203		2203	2204	-	0	4	4
2100		2097	2103	+	14215	-	-		+	0	3	3
2106	-	-		-	2170	-	-		+	0	2	2
2114	-	-		+	14066	-	-		-	0	2	2
2117		2114	2117	-	2173	-	-		-	0	16	16
2131		2127	2136	+	14522	-	-		-	0	2	2
2142		2138	2142	+	12920	-	-		-	0	2	2
2153		2153	2155	+	12864	-	-		+	0	2	2
2182		2179	2183	+	14705	-	-		+	0	2	2
2205		2204	2205	+	14379	-	-		+	0	4	4
2228	-	-		-	3223	-	-		+	0	2	2
2244		2244	2246	+	13818	-	-		-	0	2	2
2249		2248	2251	+	10553	-	-		+	0	2	2
2249		2248	2251	+	13151	-	-		+	0	7	7
2258		2257	2258	+	13544	-	-		+	0	4	4
2284		2282	2284	-	14116	-	-		-	0	2	2
2298		2294	2301	+	11879		11877	11879	+	0	2	2
2397		2397	2401	+	13543	-	-		+	0	6	6
2414		2413	2418	+	14860	-	-		-	0	4	4
2426		2422	2429	+	14706	-	-		+	0	2	2
2444		2443	2444	+	2466	-	-		-	0	2	2
2495		2493	2495	-	2531	-	-		-	0	2	2
2560		2558	2561	+	13940	-	-		+	0	2	2
2574		2570	2574	+	13410	-	-		+	0	2	2
2687	-	-		-	11018	-	-		+	0	2	2
2757	-	-		-	2799	-	-		-	0	3	3
2808		2807	2809	+	2832	-	-		-	0	2	2
2845		2843	2845	+	14777	-	-		+	0	2	2
2866	-	-		+	10606	-	-		+	0	2	2
2915		2914	2915	+	12275	-	-		+	0	2	2
2968	-	-		-	3003	-	-		+	0	5	5
2992	-	-		+	12988	-	-		+	0	2	2
2992	-	-		+	15010	-	-		+	0	3	3
2998	-	-		-	3220	-	-		+	0	2	2
3055	-	-		+	3077	-	-		-	0	4	4
3115	-	-		-	15198	-	-		-	0	2	2
3137		3136	3137	+	14633	-	-		+	0	2	2
3151		3150	3151	-	13841	-	-		+	0	2	2
3154		3153	3156	+	12063	-	-		+	0	2	2
3154		3153	3156	+	13583	-	-		+	0	2	2
3154		3153	3156	+	14128	-	-		+	0	2	2
3166		3165	3169	+	14679	-	-		+	0	2	2
3175		3171	3175	+	14513	-	-		-	0	2	2
3215	-	-		-	3340	-	-		-	0	3	3
3227		3227	3231	+	4486	-	-		+	0	2	2
3263		3262	3267	-	14667		14666	14667	-	0	3	3
3279		3275	3283	+	11109	-	-		+	0	3	3
3301	-	-		-	15213	-	-		+	0	2	2
3313		3313	3315	-	3462	-	-		-	0	2	2
3392		3391	3393	+	14185	-	-		+	0	3	3
3401		3401	3405	+	14759	-	-		+	0	4	4
3410	-	-		+	12464	-	-		+	0	2	2
3419		3418	3421	+	10098	-	-		+	0	2	2
3441	-	-		+	14222	-	-		-	0	2	2
3448		3448	3449	+	13769	-	-		+	0	2	2
3455		3454	3459	+	14993		14990	14993	+	0	2	2
3468		3468	3469	+	15289	-	-		+	0	2	2
3483		3479	3483	-	15069		15067	15069	-	0	2	2
3490	-	-		+	12221	-	-		-	0	2	2
3500		3498	3501	+	14256	-	-		+	0	3	3

3517 -	-		+	13741 -	-		+	0	3	3
3577	3577	3581	+	12702 -	-		+	0	2	2
3577	3577	3581	+	14401 -	-		+	0	3	3
3690	3690	3692	+	12474 -	-		+	0	3	3
3754	3751	3754	+	14026	14023	14026	+	0	2	2
3773	3771	3773	+	15210 -	-		-	0	9	9
3835	3835	3836	+	15112	15111	15112	-	0	24	24
3846 -	-		-	15103 -	-		+	0	7	7
3895 -	-		-	12948 -	-		+	0	2	2
3921 -	-		-	10139 -	-		-	0	2	2
3942 -	-		+	12688 -	-		+	0	2	2
3943 -	-		-	12370 -	-		+	0	2	2
3972	3972	3976	+	13548 -	-		+	0	3	3
3982	3978	3982	+	15116 -	-		+	0	5	5
4031	4027	4031	+	13954 -	-		+	0	3	3
4039 -	-		+	11203 -	-		+	0	2	2
4076 -	-		-	14411 -	-		+	0	2	2
4106 -	-		-	14778 -	-		+	0	3	3
4168	4168	4171	+	14525 -	-		+	0	2	2
4181 -	-		-	15310 -	-		-	0	2	2
4238 -	-		-	13740 -	-		+	0	2	2
4258	4258	4261	-	15144 -	-		+	0	3	3
4330	4330	4333	+	13262 -	-		+	0	2	2
4335 -	-		+	10587 -	-		+	0	3	3
4434	4431	4438	-	14456 -	-		+	0	3	3
4438	4435	4442	+	15036 -	-		+	0	12	12
4476	4476	4478	+	12284 -	-		+	0	3	3
4481 -	-		+	14045 -	-		+	0	3	3
4629	4629	4631	+	13413 -	-		+	0	3	3
4662	4659	4665	+	14358 -	-		+	0	2	2
4678 -	-		+	12778 -	-		+	0	2	2
4678 -	-		+	14242 -	-		+	0	10	10
4687	4685	4688	+	14586 -	-		+	0	2	2
4694 -	-		+	13587 -	-		+	0	2	2
4713 -	-		+	13869 -	-		+	0	2	2
4764	4764	4768	+	11137 -	-		+	0	2	2
4834 -	-		+	13972 -	-		+	0	2	2
4864 -	-		+	13187 -	-		+	0	2	2
4944	4944	4947	+	13838 -	-		+	0	2	2
4944	4944	4947	+	14791 -	-		+	0	2	2
5089	5086	5089	-	15037	15034	15037	-	0	6	6
5099	5095	5099	+	14179 -	-		+	0	3	3
5184 -	-		+	5276 -	-		+	0	2	2
5316 -	-		+	11766 -	-		+	0	4	4
5393 -	-		+	10572 -	-		+	0	2	2
5445 -	-		+	13497 -	-		+	0	2	2
5525 -	-		-	7594 -	-		+	0	2	2
5660 -	-		-	13501 -	-		+	0	3	3
5762 -	-		+	15202 -	-		-	0	2	2
5773	5773	5775	+	15182 -	-		-	0	6	6
5778 -	-		-	15177 -	-		+	0	21	21
5785	5784	5789	+	14639 -	-		+	0	2	2
5786	5785	5786	-	15179 -	-		+	0	3	3
5798 -	-		-	15197 -	-		-	0	2	2
5827	5827	5828	+	12903 -	-		+	0	2	2
5856 -	-		-	6048 -	-		-	0	7	7
5913 -	-		+	14253 -	-		+	0	4	4
5942 -	-		-	9221 -	-		+	0	2	2
6294	6293	6294	-	6554 -	-		+	0	3	3
6532	6529	6533	+	13632 -	-		+	0	2	2
6556	6554	6556	-	7724 -	-		-	0	3	3
6578 -	-		-	8094 -	-		-	0	2	2
6723 -	-		-	9246 -	-		-	0	2	2
6753	6751	6757	+	13861 -	-		+	0	3	3
6985 -	-		+	6976	6976	6977	+	0	2	2
7092 -	-		-	13792 -	-		-	0	2	2
7098 -	-		-	7134 -	-		-	0	2	2
7126 -	-		-	14864 -	-		-	0	2	2
7133	7130	7133	+	13613 -	-		+	0	2	2
7301 -	-		+	7317 -	-		+	0	4	4
7314 -	-		-	11341 -	-		+	0	2	2
7345 -	-		-	9690 -	-		+	0	3	3
7502	7497	7506	-	7594 -	-		-	0	5	5
7527 -	-		-	14332 -	-		+	0	2	2
7564 -	-		+	7846 -	-		+	0	2	2
7591 -	-		+	13249 -	-		+	0	2	2
7617 -	-		-	8088 -	-		-	0	2	2
7625 -	-		+	14404 -	-		+	0	2	2
7887 -	-		+	14943 -	-		+	0	2	2
7917 -	-		-	8111 -	-		-	0	2	2
7966 -	-		-	8065 -	-		-	0	2	2
8037 -	-		-	13751 -	-		+	0	2	2
8154 -	-		+	12921 -	-		-	0	2	2
8309	8309	8313	-	13837 -	-		+	0	2	2

8349		8349	8350	-	15076	-	-		+	0	2	2
8472	-	-		-	8999	-	-		+	0	2	2
8521		8521	8524	-	13429	-	-		+	0	2	2
8682	-	-		-	13662	-	-		+	0	2	2
8770		8768	8770	-	15153	-	-		+	0	7	7
8953		8950	8953	-	12591	-	-		+	0	2	2
9038	-	-		-	14561	-	-		+	0	2	2
9161	-	-		-	13148	-	-		+	0	2	2
9180	-	-		-	14677	-	-		+	0	2	2
9263	-	-		-	14198	-	-		+	0	2	2
9394	-	-		-	9618	-	-		-	0	3	3
9400		9397	9400	-	14281	-	-		+	0	2	2
9426		9426	9429	-	13415	-	-		+	0	2	2
9446		9444	9446	-	9916	-	-		-	0	3	3
9574	-	-		+	14677	-	-		-	0	2	2
9649	-	-		-	13530	-	-		+	0	2	2
9674		9671	9674	-	13418	-	-		+	0	2	2
9799		9799	9801	-	14580	-	-		+	0	2	2
9859		9858	9860	-	13057	-	-		+	0	2	2
9932		9929	9932	-	13103	-	-		+	0	4	4
9950		9950	9954	-	14117	-	-		-	0	2	2
9967		9964	9971	-	15234	-	-		+	0	2	2
9973	-	-		-	14222	-	-		-	0	2	2
10019		10017	10019	-	12364	-	-		+	0	2	2
10019		10017	10019	-	13953	-	-		+	0	2	2
10036		10035	10038	-	12202	-	-		+	0	2	2
10050		10046	10053	-	14281	-	-		+	0	3	3
10089		10089	10092	-	14090	-	-		+	0	2	2
10193	-	-		+	10421	-	-		+	0	2	2
10230		10228	10230	+	13684		13684	13685	+	0	2	2
10258		10256	10262	-	14169	-	-		-	0	2	2
10268	-	-		-	13925	-	-		+	0	3	3
10278		10276	10282	-	10473	-	-		-	0	2	2
10286	-	-		-	13637	-	-		-	0	2	2
10334		10332	10334	-	10470	-	-		-	0	3	3
10411		10408	10411	+	11705	-	-		-	0	2	2
10420		10416	10420	-	13741	-	-		+	0	2	2
10425		10421	10425	-	14189	-	-		+	0	3	3
10454	-	-		+	14800	-	-		+	0	2	2
10462	-	-		-	14482	-	-		+	0	2	2
10485		10482	10486	-	13910	-	-		+	0	3	3
10512		10509	10514	-	10502	-	-		+	0	3	3
10512		10509	10514	-	14537	-	-		+	0	3	3
10517	-	-		+	10496	-	-		-	0	2	2
10532	-	-		+	12868	-	-		+	0	2	2
10532	-	-		+	14192	-	-		+	0	2	2
10533		10529	10533	-	12147	-	-		+	0	2	2
10533		10529	10533	-	13000	-	-		+	0	3	3
10587	-	-		-	13444	-	-		+	0	2	2
10620		10616	10623	-	11879	-	-		+	0	2	2
10631	-	-		-	11857	-	-		+	0	3	3
10666	-	-		-	13658	-	-		+	0	2	2
10697		10694	10699	-	14870	-	-		+	0	3	3
10744	-	-		+	14440	-	-		+	0	2	2
10748		10744	10748	-	13750	-	-		+	0	3	3
10773		10773	10775	-	14080	-	-		+	0	2	2
10803		10803	10806	-	13978	-	-		+	0	2	2
10831		10830	10835	-	13248		13244	13248	-	0	2	2
10855		10855	10858	-	12629	-	-		+	0	3	3
10920		10917	10920	-	13351	-	-		+	0	2	2
10920		10917	10920	-	14499	-	-		+	0	2	2
10969		10965	10969	-	14784	-	-		+	0	2	2
10993		10991	10996	-	11241	-	-		-	0	2	2
11007		11006	11008	-	14549	-	-		+	0	2	2
11047	-	-		-	14064	-	-		+	0	3	3
11089		11088	11089	-	14694	-	-		+	0	5	5
11097		11092	11099	-	12634	-	-		-	0	4	4
11117		11114	11117	-	12660	-	-		-	0	2	2
11122		11121	11123	-	13941	-	-		+	0	10	10
11183		11182	11186	-	12081	-	-		+	0	2	2
11258		11258	11261	-	11284	-	-		-	0	2	2
11270		11266	11270	-	15015	-	-		-	0	2	2
11344	-	-		-	14840	-	-		+	0	2	2
11374		11374	11375	-	13593	-	-		+	0	2	2
11393		11390	11396	-	13048	-	-		-	0	2	2
11393		11390	11396	-	15008	-	-		+	0	3	3
11435		11431	11439	-	14331	-	-		+	0	2	2
11446		11443	11450	+	14441	-	-		+	0	25	25
11458		11458	11462	-	13372	-	-		+	0	2	2
11468		11464	11472	-	11969	-	-		-	0	2	2
11517	-	-		-	12658	-	-		+	0	3	3
11526	-	-		+	14224	-	-		+	0	2	2
11551		11550	11554	-	13230	-	-		+	0	2	2
11556		11556	11557	-	13309	-	-		+	0	7	7

11641	11637	11644	-	14531	-	-	+	0	2	2
11733	11730	11736	-	14253	-	-	+	0	3	3
11742	11738	11742	-	14089	-	-	+	0	2	2
11774	11769	11774	-	12880	-	-	+	0	2	2
11774	11769	11774	-	13559	-	-	+	0	3	3
11782	11781	11786	-	14019	-	-	+	0	2	2
11782	11781	11786	-	14265	-	-	+	0	2	2
11782	11781	11786	-	14840	-	-	+	0	3	3
11782	11781	11786	-	15031	-	-	+	0	2	2
11793	11788	11794	-	12632	-	-	+	0	2	2
11802	-	-	+	13795	-	-	-	0	2	2
11808	-	-	+	13796	-	-	-	0	2	2
11816	11816	11818	-	13499	-	-	+	0	2	2
11836	11835	11839	-	13043	-	-	-	0	3	3
11836	11835	11839	-	13804	-	-	-	0	2	2
11837	-	-	+	13826	-	-	+	0	4	4
11873	11871	11874	-	15228	-	-	-	0	2	2
11890	11890	11891	-	13930	-	-	+	0	2	2
11890	11890	11891	-	14295	-	-	+	0	2	2
11895	11895	11898	-	13736	-	-	+	0	4	4
11906	11906	11907	-	13678	-	-	+	0	2	2
11920	11916	11926	-	14264	-	-	+	0	2	2
11950	11947	11950	-	13362	-	-	+	0	5	5
11955	11955	11957	-	14302	-	-	+	0	16	16
11964	11964	11967	-	12622	-	-	+	0	3	3
11964	11964	11967	-	12733	-	-	-	0	5	5
11964	11964	11967	-	15121	-	-	+	0	5	5
12002	11999	12002	-	14068	-	-	-	0	2	2
12010	12010	12011	-	14067	-	-	-	0	2	2
12010	12010	12011	-	14833	14833	14834	+	0	2	2
12018	12016	12021	-	14190	14188	14190	+	0	2	2
12085	12085	12086	-	15084	-	-	-	0	3	3
12088	12088	12090	+	14589	-	-	+	0	6	6
12094	12090	12094	-	12446	-	-	-	0	2	2
12113	12109	12116	-	12340	-	-	-	0	3	3
12113	12109	12116	-	13404	-	-	+	0	2	2
12121	12119	12123	-	12342	-	-	-	0	3	3
12134	12132	12134	-	12912	-	-	+	0	2	2
12147	12142	12151	-	12291	12289	12291	-	0	5	5
12147	12142	12151	-	14664	-	-	+	0	4	4
12199	12195	12201	-	13229	-	-	+	0	3	3
12199	12195	12201	-	14247	-	-	+	0	4	4
12199	12195	12201	-	14488	-	-	+	0	5	5
12199	12195	12201	-	14729	-	-	+	0	3	3
12202	-	-	+	14612	-	-	+	0	3	3
12204	12203	12208	-	14815	-	-	+	0	3	3
12233	12230	12234	-	14368	-	-	+	0	3	3
12240	12235	12244	-	13255	-	-	-	0	2	2
12246	12246	12250	-	13837	-	-	+	0	3	3
12257	12255	12258	-	13646	-	-	+	0	3	3
12257	12255	12258	-	14761	-	-	+	0	2	2
12269	12269	12270	-	13178	-	-	+	0	2	2
12274	12274	12276	-	14307	-	-	+	0	2	2
12307	12303	12310	-	14286	-	-	+	0	2	2
12321	12318	12326	-	14577	-	-	+	0	5	5
12322	12317	12324	+	14285	-	-	+	0	2	2
12328	-	-	-	13779	-	-	+	0	3	3
12341	12341	12345	-	13122	-	-	+	0	2	2
12341	12341	12345	-	13751	-	-	+	0	2	2
12356	12356	12360	+	13957	-	-	+	0	2	2
12358	12357	12359	-	14475	-	-	+	0	3	3
12383	12380	12386	-	13766	-	-	+	0	10	10
12383	12380	12386	-	13829	-	-	+	0	3	3
12399	12396	12399	-	14752	-	-	+	0	5	5
12407	12402	12411	-	13065	-	-	+	0	3	3
12407	12402	12411	-	14265	-	-	+	0	2	2
12407	12402	12411	-	14591	-	-	+	0	2	2
12418	12418	12423	-	14439	-	-	+	0	3	3
12438	12437	12439	-	14294	-	-	+	0	2	2
12442	12442	12444	+	14093	-	-	+	0	3	3
12481	12476	12482	-	12666	-	-	-	0	3	3
12481	12476	12482	-	13720	-	-	+	0	2	2
12503	12499	12503	+	14165	-	-	+	0	2	2
12514	12513	12515	-	12640	-	-	-	0	3	3
12514	12513	12515	-	13701	-	-	+	0	5	5
12531	12527	12531	-	14635	-	-	-	0	2	2
12531	12527	12531	-	14650	-	-	+	0	6	6
12536	12535	12542	-	12684	-	-	-	0	17	17
12536	12535	12542	-	12776	-	-	+	0	2	2
12536	12535	12542	-	12822	-	-	-	0	3	3
12536	12535	12542	-	12897	-	-	+	0	2	2
12553	12549	12557	-	13792	-	-	+	0	3	3
12553	12549	12557	-	14124	-	-	-	0	2	2
12553	12549	12557	-	14182	-	-	-	0	3	3

12553	12549	12557	-	14841	-	-	+	0	2	2
12591	12589	12594	-	12625	-	-	-	0	3	3
12591	12589	12594	-	13319	-	-	-	0	3	3
12605	12599	12607	-	14337	-	-	+	0	2	2
12611	12609	12614	-	12644	-	-	-	0	2	2
12616	12615	12620	-	14854	-	-	-	0	2	2
12625	12625	12629	-	13760	-	-	-	0	2	2
12662	12658	12662	+	13008	-	-	+	0	2	2
12676	12672	12678	-	13346	-	-	+	0	2	2
12676	12672	12678	-	14587	-	-	+	0	5	5
12676	12672	12678	-	14842	-	-	-	0	2	2
12683	12683	12687	-	13893	-	-	+	0	3	3
12683	12683	12687	-	13979	-	-	+	0	3	3
12689	12688	12690	-	14342	-	-	+	0	2	2
12689	12688	12690	-	14762	-	-	+	0	4	4
12689	12688	12690	-	14857	-	-	-	0	2	2
12696	12692	12699	-	13357	-	-	+	0	2	2
12696	12692	12699	-	13739	-	-	-	0	2	2
12712	12708	12712	-	14653	-	-	+	0	2	2
12717	12713	12721	-	14511	-	-	-	0	3	3
12739	12737	12743	-	13988	-	-	+	0	3	3
12739	12737	12743	-	15024	-	-	+	0	12	12
12775	12771	12780	-	13459	-	-	+	0	2	2
12775	12771	12780	-	14622	-	-	+	0	3	3
12775	12771	12780	-	14778	-	-	+	0	3	3
12788	12788	12792	-	13534	13532	13534	+	0	2	2
12802	12801	12807	-	14416	-	-	-	0	3	3
12813	12810	12815	-	12931	-	-	-	0	5	5
12820	-	-	+	12812	-	-	-	0	4	4
12822	-	-	-	13107	-	-	+	0	2	2
12833	12830	12836	-	13393	-	-	-	0	4	4
12838	12837	12841	-	14068	-	-	-	0	2	2
12846	-	-	+	13793	-	-	-	0	2	2
12884	12881	12888	-	14105	-	-	+	0	2	2
12884	12881	12888	-	14193	-	-	-	0	4	4
12884	12881	12888	-	14810	14809	14810	+	0	3	3
12894	12894	12898	+	12871	-	-	-	0	4	4
12896	12895	12898	-	13683	-	-	+	0	3	3
12896	12895	12898	-	14283	-	-	+	0	2	2
12896	12895	12898	-	14331	-	-	+	0	9	9
12896	12895	12898	-	15157	-	-	+	0	3	3
12903	12900	12905	-	13388	-	-	-	0	3	3
12903	12900	12905	-	14464	-	-	+	0	3	3
12911	12906	12914	-	14000	-	-	+	0	3	3
12911	12906	12914	-	14858	-	-	-	0	2	2
12921	12917	12924	-	13099	-	-	+	0	3	3
12921	12917	12924	-	14869	14868	14869	+	0	3	3
12921	12917	12924	-	15019	-	-	+	0	6	6
12921	12917	12924	-	15062	-	-	+	0	10	10
12926	12925	12928	-	15022	-	-	+	0	2	2
12931	-	-	-	13315	-	-	-	0	2	2
12931	12930	12935	+	15168	-	-	+	0	2	2
12940	12936	12945	-	13119	-	-	+	0	2	2
12940	12936	12945	-	13799	-	-	+	0	3	3
12940	12936	12945	-	13878	-	-	+	0	3	3
12940	12936	12945	-	14170	-	-	+	0	2	2
12940	12936	12945	-	14262	-	-	+	0	2	2
12947	12944	12949	+	15000	-	-	-	0	3	3
12954	-	-	+	15041	-	-	+	0	2	2
12956	12952	12958	-	13498	-	-	+	0	2	2
12956	12952	12958	-	14424	-	-	+	0	2	2
12956	12952	12958	-	14588	-	-	+	0	2	2
12965	12965	12968	-	13735	13735	13736	+	0	3	3
12973	12970	12979	-	14016	14014	14016	-	0	3	3
12973	12970	12979	-	14106	-	-	+	0	3	3
12973	12970	12979	-	14412	-	-	+	0	4	4
12973	12970	12979	-	14750	-	-	+	0	3	3
12978	12973	12978	+	14033	-	-	+	0	3	3
12983	12980	12988	-	14488	-	-	+	0	2	2
13013	13009	13017	-	13224	-	-	+	0	3	3
13013	13009	13017	-	13743	-	-	+	0	3	3
13031	13030	13032	-	15152	-	-	+	0	2	2
13044	13040	13049	-	14731	-	-	+	0	2	2
13050	13050	13054	+	13637	-	-	+	0	3	3
13055	13053	13058	-	14315	-	-	+	0	2	2
13060	13059	13064	-	14883	-	-	+	0	7	7
13066	-	-	+	13670	-	-	+	0	3	3
13089	13087	13090	-	14842	-	-	+	0	6	6
13094	13091	13095	-	13215	-	-	-	0	2	2
13094	13091	13095	-	13431	-	-	+	0	2	2
13104	13102	13107	-	13492	-	-	-	0	2	2
13104	13102	13107	-	13765	-	-	+	0	2	2
13104	13102	13107	-	14933	-	-	-	0	3	3
13126	13123	13129	-	14490	-	-	+	0	2	2

13141	13138	13145	-	14870	14867	14870	-	0	2	2
13147	13146	13151	-	14314 -	-		+	0	4	4
13147	13146	13151	-	14334 -	-		-	0	2	2
13147	13146	13151	-	14891 -	-		-	0	2	2
13157	13155	13161	-	13904 -	-		+	0	3	3
13168	13162	13169	-	13511 -	-		+	0	2	2
13182	13180	13182	-	14490 -	-		-	0	2	2
13187	13183	13190	-	13872 -	-		+	0	2	2
13193 -	-		-	15004 -	-		-	0	2	2
13209	13205	13209	-	13672 -	-		+	0	2	2
13227	13224	13230	-	15078 -	-		+	0	4	4
13237 -	-		-	14107 -	-		+	0	2	2
13246	13241	13247	-	14470	14467	14470	+	0	4	4
13257	13254	13257	+	14513 -	-		+	0	3	3
13258	13257	13260	-	13899 -	-		+	0	9	9
13265	13262	13269	-	15050 -	-		-	0	6	6
13265	13262	13269	+	13907 -	-		+	0	3	3
13272	13270	13276	-	14110 -	-		+	0	2	2
13278	13277	13282	-	13993 -	-		+	0	4	4
13278	13277	13282	-	14034 -	-		-	0	3	3
13278	13277	13282	-	14042 -	-		+	0	2	2
13278	13277	13282	-	14409 -	-		+	0	6	6
13288	13288	13291	-	13694 -	-		+	0	2	2
13288	13288	13291	-	14095	14095	14097	+	0	3	3
13297	13293	13302	-	13509 -	-		-	0	5	5
13308	13303	13312	-	14466 -	-		+	0	2	2
13308	13303	13312	-	15215 -	-		+	0	3	3
13316	13313	13322	-	14538 -	-		+	0	3	3
13317	13315	13320	+	14887 -	-		+	0	2	2
13317	13315	13320	+	14925 -	-		+	0	2	2
13317	13315	13320	+	15083 -	-		+	0	3	3
13322 -	-		+	14842 -	-		-	0	15	15
13326	13324	13327	-	13458 -	-		+	0	2	2
13326	13324	13327	-	13750 -	-		-	0	2	2
13326	13324	13327	-	14423 -	-		+	0	5	5
13330	13326	13336	+	14397 -	-		+	0	3	3
13330	13326	13336	+	14988 -	-		+	0	8	8
13330	13326	13336	+	15085 -	-		+	0	10	10
13330	13326	13336	+	15162 -	-		+	0	2	2
13332	13328	13336	-	13704 -	-		+	0	4	4
13332	13328	13336	-	13812 -	-		-	0	87	87
13332	13328	13336	-	14882 -	-		-	0	14	14
13340 -	-		+	15001 -	-		+	0	4	4
13347	13343	13351	-	13602 -	-		+	0	3	3
13349	13348	13351	+	15007 -	-		+	0	2	2
13353	13352	13353	-	14835 -	-		+	0	8	8
13365	13365	13368	-	13699 -	-		-	0	2	2
13372	13372	13375	-	14020 -	-		-	0	2	2
13372	13372	13375	-	14756 -	-		+	0	3	3
13385	13380	13388	-	13908 -	-		+	0	3	3
13385	13380	13388	-	14076 -	-		+	0	2	2
13394	13389	13397	-	14512 -	-		+	0	3	3
13394	13389	13397	-	14515 -	-		-	0	2	2
13394	13389	13397	-	14660 -	-		-	0	2	2
13394	13394	13397	+	14194 -	-		-	0	4	4
13394	13394	13397	+	14797 -	-		+	0	3	3
13416	13413	13420	-	15213 -	-		+	0	7	7
13431	13430	13436	-	14145 -	-		+	0	3	3
13438 -	-		-	13726 -	-		-	0	7	7
13451	13448	13454	-	14160 -	-		+	0	5	5
13459	13457	13462	+	14181 -	-		-	0	3	3
13462	13458	13467	-	13592 -	-		-	0	2	2
13462	13458	13467	-	14163 -	-		-	0	2	2
13466	13465	13466	+	15064 -	-		+	0	6	6
13471	13468	13475	+	13689	13685	13689	+	0	10	10
13471	13468	13475	+	14645 -	-		-	0	3	3
13479	13475	13483	-	13705 -	-		-	0	4	4
13479	13475	13483	-	15047 -	-		-	0	2	2
13488	13485	13490	-	14685 -	-		+	0	2	2
13490	13486	13492	+	15097 -	-		+	0	5	5
13498	13496	13504	-	15089 -	-		+	0	2	2
13499	13495	13503	+	13999 -	-		-	0	2	2
13499	13495	13503	+	14722 -	-		-	0	2	2
13516	13511	13517	-	14222 -	-		+	0	3	3
13523	13521	13525	-	14532 -	-		+	0	2	2
13532	13531	13533	+	13574 -	-		-	0	2	2
13532	13531	13533	+	13679 -	-		+	0	11	11
13544	13538	13545	-	13908 -	-		+	0	2	2
13544	13538	13545	-	14452 -	-		-	0	3	3
13549	13548	13553	-	13655 -	-		-	0	2	2
13549	13548	13553	-	14581 -	-		+	0	3	3
13551	13548	13555	+	14518	14514	14518	+	0	2	2
13555	13554	13558	-	14653 -	-		+	0	2	2
13562	13559	13567	-	14465 -	-		+	0	2	2

13562	13559	13567	-	14564	-	-	+	0	5	5
13566	13562	13569	+	13604	-	-	-	0	2	2
13575	13572	13578	+	14575	-	-	+	0	2	2
13575	13572	13578	+	14636	-	-	-	0	2	2
13575	13572	13578	+	14672	-	-	+	0	17	17
13578	13574	13583	-	14186	-	-	+	0	2	2
13578	13574	13583	-	14582	14578	14586	-	0	5	5
13578	13574	13583	-	14677	-	-	-	0	17	17
13581	13579	13585	+	14684	-	-	+	0	4	4
13587	13584	13591	-	14537	-	-	-	0	2	2
13587	13584	13591	-	14669	-	-	+	0	3	3
13605	13602	13606	-	14575	-	-	+	0	2	2
13616	13613	13619	-	14613	-	-	+	0	2	2
13616	13613	13619	-	14751	-	-	+	0	2	2
13616	13613	13619	-	14834	-	-	+	0	2	2
13623	13622	13624	+	14736	-	-	+	0	4	4
13629	13624	13635	-	14678	-	-	-	0	6	6
13629	13624	13635	-	14747	-	-	+	0	3	3
13639	13637	13642	-	14021	-	-	-	0	3	3
13639	13637	13642	-	14220	-	-	-	0	4	4
13644	13643	13648	-	14466	-	-	-	0	4	4
13651	13648	13653	+	14849	-	-	+	0	2	2
13656	13655	13660	+	13675	13672	13675	+	0	2	2
13656	13655	13660	+	14445	-	-	+	0	2	2
13659	13655	13660	-	13788	13788	13789	-	0	2	2
13659	13655	13660	-	14854	-	-	-	0	5	5
13663	-	-	+	13701	-	-	-	0	2	2
13664	13661	13668	-	13805	-	-	-	0	3	3
13664	13661	13668	-	14260	-	-	+	0	3	3
13668	-	-	+	13700	-	-	-	0	4	4
13674	13669	13678	-	13877	-	-	-	0	3	3
13674	13669	13678	-	14123	-	-	+	0	2	2
13674	13669	13678	-	14227	-	-	-	0	2	2
13681	13677	13683	+	14182	-	-	+	0	2	2
13720	13717	13722	-	14267	-	-	-	0	2	2
13738	13733	13743	+	14729	-	-	+	0	2	2
13751	13748	13755	-	14484	-	-	-	0	3	3
13751	13748	13755	-	14614	-	-	+	0	2	2
13751	13751	13753	+	14357	-	-	+	0	2	2
13759	13757	13760	-	13870	-	-	-	0	3	3
13759	13757	13760	-	14432	-	-	+	0	11	11
13770	13766	13775	-	13805	13802	13805	-	0	3	3
13770	13766	13775	-	14139	-	-	-	0	2	2
13770	13766	13775	-	14502	-	-	+	0	3	3
13770	13766	13775	-	14542	14542	14543	-	0	2	2
13776	13771	13780	+	14358	-	-	+	0	2	2
13779	13776	13782	-	15210	-	-	-	0	2	2
13800	13798	13803	+	15048	-	-	+	0	3	3
13841	13839	13843	+	13878	-	-	-	0	2	2
13848	13843	13850	-	14226	-	-	+	0	3	3
13851	13849	13856	+	15020	-	-	+	0	2	2
13853	13851	13857	-	14253	-	-	-	0	2	2
13853	13851	13857	-	15007	-	-	+	0	18	18
13863	13859	13866	-	14019	-	-	-	0	3	3
13863	13859	13866	-	15016	-	-	-	0	4	4
13863	13859	13866	-	15203	-	-	+	0	3	3
13868	13864	13871	+	13911	-	-	-	0	2	2
13868	13864	13871	+	14379	-	-	+	0	2	2
13870	13867	13871	-	14031	-	-	-	0	2	2
13870	13867	13871	-	14675	-	-	+	0	2	2
13876	13872	13879	-	14233	-	-	+	0	2	2
13876	13872	13879	-	14640	-	-	-	0	2	2
13878	13874	13882	+	14370	-	-	+	0	5	5
13885	13880	13891	-	14371	-	-	-	0	4	4
13885	13885	13886	+	13906	-	-	-	0	3	3
13900	13898	13903	-	14185	-	-	-	0	3	3
13908	13906	13912	-	15113	-	-	-	0	2	2
13921	13917	13925	-	13999	-	-	-	0	2	2
13936	13932	13940	-	14909	-	-	+	0	2	2
13947	13941	13951	-	14044	-	-	+	0	2	2
13954	-	-	+	13981	-	-	+	0	2	2
13964	13964	13966	+	13999	-	-	-	0	2	2
13972	13972	13973	-	14516	-	-	+	0	2	2
13978	13977	13982	-	14365	-	-	+	0	2	2
13978	13977	13982	-	14390	-	-	-	0	2	2
13988	13985	13989	-	14741	-	-	+	0	4	4
14001	13997	14005	-	14713	-	-	+	0	2	2
14012	14008	14012	-	14076	-	-	-	0	2	2
14014	14009	14017	+	14291	-	-	+	0	2	2
14025	14023	14029	-	14836	-	-	+	0	2	2
14035	14034	14036	-	14808	-	-	+	0	2	2
14040	14039	14043	-	14150	14148	14150	-	0	2	2
14046	14044	14048	-	14155	-	-	-	0	2	2
14046	14044	14048	-	14199	-	-	-	0	3	3

14065	14062	14069	-	14199	-	-	-	0	2	2
14065	14061	14065	+	14112	-	-	+	0	2	2
14079	14077	14083	-	14586	-	-	-	0	2	2
14079	14077	14083	-	14875	-	-	-	0	2	2
14090	14089	14094	-	14619	-	-	-	0	2	2
14096	14096	14101	-	14987	-	-	+	0	2	2
14120	14114	14126	-	14511	-	-	+	0	2	2
14130	14128	14130	-	14669	-	-	-	0	3	3
14138	14133	14141	-	14200	-	-	-	0	11	11
14138	14133	14141	-	14608	-	-	+	0	4	4
14152	14150	14156	+	14739	-	-	+	0	4	4
14159	14157	14160	-	14194	-	-	-	0	21	21
14164	14161	14166	-	14391	-	-	-	0	12	12
14166	14166	14169	+	14130	-	-	-	0	2	2
14166	14166	14169	+	14752	-	-	+	0	2	2
14166	14166	14169	+	14931	-	-	-	0	2	2
14182	-	-	-	14807	-	-	-	0	2	2
14183	14182	14183	+	14196	-	-	+	0	2	2
14202	14201	14204	-	14217	-	-	+	0	3	3
14207	14207	14208	-	15153	-	-	-	0	3	3
14212	14211	14215	-	15176	-	-	+	0	5	5
14217	14216	14218	-	14249	-	-	+	0	4	4
14217	14216	14218	-	14472	-	-	-	0	2	2
14221	14221	14224	+	14710	-	-	+	0	2	2
14223	14219	14227	-	14287	-	-	+	0	2	2
14223	14219	14227	-	14537	-	-	-	0	2	2
14223	14219	14227	-	14589	-	-	-	0	3	3
14223	14219	14227	-	15033	-	-	+	0	2	2
14223	14219	14227	-	15074	-	-	-	0	3	3
14223	14219	14227	-	15151	-	-	-	0	6	6
14223	14219	14227	-	15173	15171	15173	-	0	3	3
14228	14228	14229	+	15163	-	-	-	0	2	2
14229	14228	14233	-	14247	-	-	+	0	2	2
14229	14228	14233	-	14273	-	-	+	0	2	2
14238	14235	14243	-	14769	-	-	+	0	6	6
14238	14235	14243	-	14800	-	-	+	0	2	2
14238	14235	14243	-	14852	-	-	+	0	4	4
14239	14238	14243	+	14428	-	-	+	0	2	2
14245	14245	14246	+	14325	-	-	+	0	2	2
14252	14252	14257	+	15009	-	-	-	0	3	3
14254	14253	14257	-	14405	-	-	-	0	6	6
14254	14253	14257	-	14668	-	-	+	0	2	2
14254	14253	14257	-	14710	-	-	+	0	5	5
14254	14253	14257	-	14847	-	-	+	0	5	5
14254	14253	14257	-	14968	-	-	+	0	2	2
14254	14253	14257	-	15034	-	-	-	0	3	3
14254	14253	14257	-	15046	-	-	-	0	3	3
14265	14263	14271	-	14255	-	-	+	0	2	2
14265	14263	14271	-	14463	-	-	-	0	3	3
14270	14270	14272	+	14348	-	-	-	0	4	4
14284	14282	14289	-	14524	-	-	+	0	2	2
14284	14282	14289	-	15144	-	-	-	0	3	3
14294	14291	14295	-	14928	-	-	-	0	3	3
14300	14297	14301	-	14782	-	-	-	0	2	2
14303	14302	14305	+	14961	-	-	+	0	2	2
14316	14314	14318	-	14465	-	-	+	0	2	2
14324	14320	14324	-	14937	14937	14941	-	0	23	23
14324	14320	14326	+	15347	-	-	+	0	2	2
14330	14329	14331	+	15122	-	-	-	0	2	2
14331	14328	14337	-	14443	-	-	-	0	2	2
14331	14328	14337	-	14507	14507	14508	-	0	4	4
14331	14328	14337	-	14590	-	-	-	0	2	2
14336	14336	14339	+	14837	-	-	+	0	5	5
14342	14339	14346	-	14567	-	-	-	0	2	2
14342	14339	14346	-	14845	-	-	-	0	8	8
14344	14340	14348	+	14614	-	-	+	0	3	3
14344	14340	14348	+	14651	-	-	+	0	2	2
14350	14347	14353	-	14471	14468	14471	-	0	3	3
14350	14347	14353	-	14711	14711	14712	-	0	2	2
14350	14347	14353	-	14851	-	-	-	0	3	3
14350	14349	14352	+	14864	-	-	+	0	4	4
14350	14349	14352	+	15112	-	-	+	0	2	2
14360	14355	14365	-	14503	-	-	-	0	9	9
14360	14355	14365	-	14571	-	-	+	0	3	3
14360	14355	14365	-	14623	-	-	-	0	2	2
14360	14355	14365	-	14696	-	-	-	0	2	2
14360	14355	14365	-	14857	-	-	-	0	2	2
14360	14355	14365	-	15030	-	-	+	0	3	3
14360	14355	14365	-	15091	-	-	+	0	8	8
14364	14360	14364	+	14456	-	-	+	0	2	2
14370	14365	14373	+	14894	14894	14897	+	0	5	5
14370	14365	14373	+	14980	-	-	+	0	2	2
14375	14374	14379	+	14786	-	-	+	0	2	2
14375	14374	14379	+	14802	-	-	+	0	3	3

14377	14377	14380	-	14846 -	-		+	0	2	2
14381 -	-		+	14782 -	-		+	0	2	2
14384	14383	14385	-	14448 -	-		-	0	2	2
14384	14383	14385	-	14800 -	-		+	0	2	2
14398	14395	14398	-	14787 -	-		+	0	3	3
14403	14399	14407	-	14457 -	-		-	0	3	3
14403	14399	14407	-	15051 -	-		-	0	2	2
14403	14399	14407	-	15080 -	-		-	0	2	2
14403	14399	14407	-	15124 -	-		-	0	2	2
14404	14403	14404	+	15080 -	-		+	0	3	3
14409	14407	14413	+	14563 -	-		+	0	2	2
14416	14414	14416	-	14568	14566	14568	-	0	4	4
14416	14414	14416	-	14967 -	-		+	0	4	4
14422	14419	14422	+	14422 -	-		+	0	3	3
14435	14435	14438	-	14457 -	-		+	0	28	28
14435	14435	14438	-	14586 -	-		+	0	2	2
14435	14432	14438	+	14900 -	-		+	0	2	2
14435	14432	14438	+	15066 -	-		+	0	2	2
14435	14432	14438	+	15073 -	-		+	0	2	2
14442	14440	14445	+	14397 -	-		-	0	2	2
14442	14440	14445	+	14893 -	-		+	0	3	3
14443	14442	14447	-	14513 -	-		-	0	24	24
14452	14448	14455	+	14500 -	-		+	0	2	2
14462	14457	14466	+	14435 -	-		-	0	12	12
14464	14462	14468	-	14902 -	-		-	0	4	4
14470	14469	14475	+	14467 -	-		-	0	2	2
14470	14469	14475	+	14841 -	-		-	0	2	2
14485	14482	14492	-	14705	14705	14707	+	0	3	3
14501	14496	14505	+	14531 -	-		-	0	4	4
14504	14501	14508	-	14827 -	-		+	0	6	6
14511	14510	14511	-	14535	14533	14535	+	0	2	2
14518	14512	14521	-	14565 -	-		+	0	2	2
14518	14512	14521	-	14860 -	-		-	0	3	3
14518	14512	14521	-	14913 -	-		-	0	3	3
14526	14524	14530	-	14675	14673	14676	-	0	13	13
14540	14535	14540	-	14890 -	-		-	0	2	2
14540	14535	14540	-	15031 -	-		-	0	2	2
14561	14556	14564	+	14617 -	-		+	0	2	2
14565	14560	14568	-	14613 -	-		+	0	8	8
14565	14560	14568	-	14746	14746	14750	+	0	3	3
14570	14569	14573	-	14769 -	-		-	0	3	3
14570	14569	14573	-	15046 -	-		+	0	8	8
14570	14569	14573	-	15092 -	-		-	0	2	2
14578	14576	14583	-	15096 -	-		-	0	2	2
14578	14576	14583	-	15122 -	-		+	0	5	5
14589	14584	14593	+	14853	14853	14857	+	0	13	13
14595	14592	14596	-	14716 -	-		-	0	2	2
14595	14592	14596	-	14781 -	-		-	0	9	9
14596	14594	14596	+	15051	15049	15051	+	0	2	2
14596	14594	14596	+	15115 -	-		+	0	2	2
14605	14601	14609	+	15145 -	-		+	0	2	2
14607	14603	14610	-	14781 -	-		-	0	4	4
14612	14611	14617	-	14840 -	-		+	0	14	14
14612	14611	14615	+	15008 -	-		+	0	2	2
14622	14619	14626	-	14674 -	-		+	0	3	3
14622	14619	14626	-	14731	14730	14731	+	0	3	3
14622	14619	14626	-	14786 -	-		-	0	2	2
14626	14623	14626	+	15033 -	-		+	0	3	3
14633	14632	14636	-	15037 -	-		+	0	2	2
14633	14632	14636	-	15039 -	-		-	0	3	3
14633	14632	14636	-	15062	15062	15063	+	0	2	2
14646	14646	14647	-	14681 -	-		+	0	3	3
14646	14645	14649	+	14635 -	-		-	0	3	3
14651	14650	14654	+	14770 -	-		+	0	6	6
14653	14649	14656	-	14668 -	-		+	0	2	2
14653	14649	14656	-	15239 -	-		-	0	2	2
14653	14649	14656	-	15278 -	-		+	0	2	2
14666	14661	14671	-	14920 -	-		-	0	2	2
14666	14661	14671	-	15042 -	-		-	0	2	2
14676	14672	14680	-	14777	14777	14778	+	0	4	4
14676	14672	14680	-	14967 -	-		+	0	3	3
14676	14672	14679	+	14901 -	-		-	0	2	2
14681	14680	14684	+	14791	14789	14791	+	0	7	7
14683	14681	14686	-	14809 -	-		-	0	2	2
14691	14689	14692	+	14792 -	-		+	0	2	2
14693	14688	14697	-	14715	14712	14715	+	0	6	6
14693	14688	14697	-	14846 -	-		-	0	2	2
14699 -	-		-	14785 -	-		+	0	2	2
14700	14698	14700	+	14698 -	-		+	0	2	2
14700	14698	14700	+	15121 -	-		+	0	3	3
14704	14700	14708	-	14835 -	-		-	0	2	2
14704	14700	14708	-	15043 -	-		+	0	2	2
14706	14705	14706	+	14807 -	-		+	0	9	9
14706	14705	14706	+	15023 -	-		+	0	4	4

14711	14711	14714	-	14812	-	-	-	0	3	3
14721	14718	14721	+	15023	-	-	+	0	7	7
14727	14727	14731	+	15023	-	-	+	0	2	2
14730	14726	14733	-	14846	-	-	-	0	2	2
14730	14726	14733	-	14868	-	-	+	0	2	2
14730	14726	14733	-	15004	-	-	-	0	2	2
14739	14735	14743	+	15002	-	-	-	0	2	2
14748	14744	14749	-	14918	-	-	-	0	6	6
14761	14759	14764	-	14938	14938	14939	-	0	2	2
14769	14765	14770	+	14938	-	-	+	0	2	2
14775	14772	14780	-	15045	-	-	-	0	3	3
14775	14772	14780	-	15194	-	-	-	0	2	2
14775	14775	14778	+	15165	-	-	-	0	2	2
14786	14781	14789	-	15153	-	-	-	0	2	2
14786	14781	14789	-	15182	-	-	-	0	3	3
14791	14790	14793	-	14816	-	-	+	0	2	2
14791	14790	14793	-	15255	-	-	-	0	3	3
14799	-	-	+	14832	-	-	+	0	2	2
14800	14798	14800	-	14936	-	-	-	0	2	2
14805	14801	14808	+	14818	-	-	-	0	2	2
14805	14801	14808	+	15180	-	-	+	0	3	3
14805	14801	14808	+	15214	-	-	+	0	2	2
14806	14801	14809	-	15043	-	-	+	0	7	7
14806	14801	14809	-	15153	-	-	+	0	2	2
14812	14812	14817	-	14841	-	-	-	0	4	4
14824	14823	14828	-	14858	-	-	+	0	4	4
14847	14842	14851	-	15117	15117	15120	-	0	3	3
14852	-	-	+	15070	-	-	+	0	5	5
14859	14857	14862	-	14981	-	-	-	0	2	2
14859	14857	14862	-	15039	-	-	+	0	2	2
14859	14857	14862	-	15156	-	-	-	0	2	2
14869	14865	14872	-	15058	-	-	+	0	4	4
14869	14865	14872	-	15094	-	-	+	0	2	2
14869	14865	14872	-	15188	15187	15190	-	0	4	4
14870	14866	14870	+	15100	-	-	+	0	3	3
14870	14866	14870	+	15196	-	-	+	0	5	5
14877	14873	14879	-	15213	-	-	+	0	3	3
14877	14872	14882	+	14907	-	-	+	0	2	2
14877	14872	14882	+	15118	-	-	+	0	2	2
14882	14880	14887	-	14987	-	-	-	0	2	2
14882	14880	14887	-	15009	-	-	-	0	5	5
14882	14880	14887	-	15011	-	-	+	0	3	3
14885	14885	14888	+	15029	15029	15030	+	0	2	2
14894	14890	14897	-	15009	-	-	+	0	2	2
14894	14890	14897	-	15023	-	-	-	0	3	3
14894	14890	14897	-	15103	-	-	-	0	2	2
14895	14890	14900	+	15027	15025	15027	-	0	5	5
14895	14890	14900	+	15197	-	-	+	0	2	2
14905	14899	14908	-	14927	14925	14927	+	0	2	2
14910	14909	14913	-	14935	-	-	+	0	3	3
14910	14909	14913	-	14945	-	-	+	0	2	2
14910	14909	14913	-	14991	-	-	-	0	3	3
14921	14920	14926	-	14957	14957	14958	+	0	2	2
14922	14919	14922	+	15029	-	-	+	0	2	2
14933	14931	14936	-	14950	14950	14952	+	0	36	36
14933	14931	14936	-	14994	-	-	-	0	2	2
14933	14931	14936	-	15019	-	-	-	0	4	4
14933	14931	14936	-	15224	-	-	-	0	2	2
14935	14934	14939	+	15143	-	-	+	0	2	2
14947	14943	14953	-	15131	15130	15131	-	0	4	4
14947	14943	14953	-	15164	-	-	-	0	2	2
14947	14943	14953	-	15213	15210	15213	-	0	3	3
14951	14949	14954	+	15143	-	-	+	0	2	2
14957	14956	14960	-	15132	15132	15133	+	0	64	64
14960	14955	14964	+	15172	-	-	+	0	3	3
14973	14969	14975	+	15161	-	-	+	0	2	2
14974	14969	14978	-	15003	-	-	+	0	2	2
14974	14969	14978	-	15007	-	-	-	0	2	2
14974	14969	14978	-	15037	-	-	-	0	3	3
14974	14969	14978	-	15152	-	-	-	0	2	2
14981	14979	14983	-	15097	-	-	-	0	3	3
14981	14979	14983	-	15166	-	-	-	0	2	2
14993	14993	14995	-	15057	-	-	+	0	2	2
14993	14993	14995	-	15187	-	-	-	0	2	2
15002	14997	15003	-	15051	-	-	+	0	2	2
15004	15000	15009	+	14988	-	-	+	0	2	2
15004	15000	15009	+	15022	-	-	+	0	5	5
15004	15000	15009	+	15049	-	-	+	0	2	2
15008	-	-	-	15058	-	-	-	0	2	2
15014	15010	15016	-	15154	-	-	-	0	3	3
15025	15020	15027	-	15080	-	-	+	0	4	4
15025	15020	15027	-	15099	-	-	+	0	2	2
15025	15021	15028	+	15030	-	-	+	0	3	3
15037	15034	15040	-	15072	-	-	+	0	18	18

15037	15034	15040	-	15153	-	-	-	0	2	2
15042	15041	15046	-	15074	15074	15075	+	0	68	68
15042	15041	15046	-	15184	-	-	-	0	2	2
15053	15051	15055	-	15196	-	-	-	0	3	3
15059	15059	15066	-	15071	-	-	+	0	3	3
15059	15059	15066	-	15165	-	-	+	0	2	2
15072	15068	15076	-	15179	15179	15182	-	0	6	6
15072	15068	15076	-	15190	-	-	-	0	2	2
15078	15077	15083	-	15157	15156	15157	-	0	69	69
15085	15084	15087	-	15143	-	-	-	0	2	2
15090	15090	15095	-	15129	-	-	-	0	5	5
15102	15098	15105	+	15085	15085	15086	-	0	9	9
15104	15099	15107	-	15133	-	-	+	0	2	2
15104	15099	15107	-	15278	-	-	+	0	2	2
15111	15106	15111	+	15085	15083	15085	-	0	3	3
15111	15106	15111	+	15091	-	-	-	0	3	3
15116	15112	15119	+	15044	-	-	-	0	6	6
15133	15129	15133	+	15158	-	-	-	0	2	2
15138	15135	15138	+	15105	-	-	-	0	3	3
15138	15135	15138	+	15122	-	-	-	0	2	2
15143	15140	15148	+	15094	15090	15094	-	0	2	2
15143	15140	15148	+	15102	-	-	-	0	5	5
15143	15140	15148	+	15122	-	-	-	0	2	2
15152	15149	15153	+	15099	15097	15099	-	0	3	3
15152	15149	15153	+	15124	-	-	-	0	2	2
15152	15149	15153	+	15142	-	-	-	0	2	2
15205	15200	15208	+	15177	-	-	-	0	4	4
15205	15200	15208	+	15194	-	-	-	0	2	2
15217	15214	15220	+	15216	-	-	+	0	3	3
15217	15214	15220	+	15331	-	-	+	0	2	2
15223	15222	15223	+	15353	-	-	+	0	2	2
15228	15225	15229	+	15229	15225	15229	+	0	3	3
15241	15240	15246	+	15208	-	-	-	0	2	2
15251	15248	15251	+	15269	-	-	+	0	20	20
15266	15266	15267	+	15252	15252	15255	-	0	6	6
