# Supplementary material for: Identification and quantification of defective virus genomes in high throughput sequencing data using DVG-profiler, a novel post-sequence alignment processing algorithm
Source: PLoS One. 2019 May 17;14(5):e0216944. doi: 10.1371/journal.pone.0216944 (PMC6524942; doi:10.1371/journal.pone.0216944)
Supplement: S8 Table — (PDF) [file pone.0216944.s013.pdf]

Position (left)	Group start (left)	Group end (left)	Strandness (left)	Position (right)	Group start (right)	Group end (right)	Strandness (right)	Forward hits	Reverse hits	Fw and rev
5078	5074	5082	-	15083	15079	15083	+	21	325	346
10694	-	-	-	14876	-	-	+	15	292	307
14720	14716	14720	-	14943	14941	14946	-	70	160	230
97	94	102	+	82	81	84	-	138	81	219
82	79	85	+	97	97	98	-	134	80	214
14706	14705	14710	-	14930	-	-	+	101	105	206
12903	12901	12907	-	14080	-	-	+	51	80	131
12914	12911	12915	-	14088	14085	14088	-	20	99	119
1139	1135	1143	-	1327	1323	1329	-	45	58	103
7607	-	-	-	9517	-	-	+	11	88	99
700	699	701	+	756	755	756	-	49	43	92
2941	2938	2945	-	2962	2961	2963	+	73	9	82
11389	11388	11389	-	13939	-	-	-	46	34	80
714	710	716	+	742	741	742	-	44	31	75
14905	14904	14908	-	14943	14940	14944	+	27	48	75
14134	14129	14134	-	14200	14196	14200	-	8	66	74
12536	12534	12538	-	12684	12684	12685	-	0	72	72
2493	2491	2496	+	2600	-	-	+	35	36	71
12924	12923	12925	+	14083	-	-	+	0	70	70
1407	1407	1410	-	1450	1447	1450	+	35	31	66
4979	4974	4984	-	5013	5012	5017	-	11	55	66
12577	12573	12579	-	13325	13325	13326	+	27	34	61
14713	14713	14714	-	14942	14938	14942	-	23	37	60
2962	2960	2963	-	2941	2941	2942	+	59	0	59
12930	12930	12934	-	14089	-	-	-	0	57	57
748	748	750	+	708	706	708	-	41	14	55
123	118	126	+	150	149	154	-	19	29	48
3537	3534	3538	-	3728	3727	3731	-	22	25	47
12930	12929	12935	+	14089	14088	14092	+	46	1	47
3215	3212	3217	-	3340	-	-	-	26	19	45
14022	14021	14023	-	14202	14201	14203	-	0	44	44
116	111	117	+	200	196	201	+	37	6	43
12577	12573	12579	-	14622	-	-	+	18	25	43
2023	2022	2026	+	2055	2055	2056	-	18	23	41
10036	-	-	-	13350	-	-	-	5	32	37
11920	11920	11922	-	14008	14008	14011	+	14	23	37
13953	13952	13957	-	14513	14513	14515	-	25	11	36
14713	14713	14714	-	14936	-	-	-	1	35	36
63	62	63	+	88	88	89	-	33	1	34
1764	1761	1766	-	1787	1785	1787	+	21	13	34
14706	14705	14710	-	14913	14912	14917	-	22	12	34
2117	2115	2120	-	2173	2171	2173	-	0	33	33
372	366	376	-	539	538	543	-	16	16	32
11389	11387	11392	+	13939	-	-	+	17	15	32
12766	12765	12767	-	14601	14600	14601	+	18	14	32
10496	10491	10498	-	10517	10515	10520	+	24	7	31
12425	12425	12429	-	13139	13137	13141	-	13	18	31
150	148	153	+	123	123	125	-	17	12	29
756	-	-	+	700	-	-	-	13	16	29
3436	3432	3439	-	3483	3483	3484	+	19	10	29
5295	5291	5298	+	12531	12527	12531	+	13	15	28
12069	12065	12074	-	13871	13870	13871	+	15	13	28
12261	12257	12261	-	15208	15204	15208	-	12	16	28
12606	12603	12607	-	13332	-	-	-	28	0	28
12924	12924	12925	-	14083	-	-	-	28	0	28
14435	14435	14436	-	14457	-	-	+	0	28	28
97	94	102	+	57	56	57	-	26	1	27
839	835	840	+	12988	-	-	+	14	13	27
6303	6301	6307	+	6363	6362	6365	+	15	12	27
11389	11388	11389	-	13933	-	-	+	17	10	27
13953	13952	13957	-	14518	-	-	+	17	10	27
14713	14710	14716	+	14942	-	-	+	17	10	27
1642	1640	1642	-	1672	-	-	-	0	26	26
11539	11539	11541	-	13723	13721	13723	+	13	13	26
13961	13960	13965	-	14528	14526	14528	-	4	22	26
1615	1611	1616	+	1706	1705	1706	+	0	25	25
11306	11306	11310	-	13686	-	-	+	16	9	25
2055	2052	2056	+	2023	2022	2024	-	14	10	24
6303	6301	6307	+	6350	6346	6352	+	14	10	24
7505	7504	7507	-	7594	7593	7594	-	0	23	23
12601	12597	12601	-	13416	13414	13417	-	18	5	23
12868	-	-	-	13203	-	-	+	11	12	23
13772	13771	13776	+	13969	13967	13970	-	23	0	23
14428	-	-	-	14464	-	-	+	23	0	23
372	366	376	-	571	567	576	-	7	15	22
839	835	840	+	9136	-	-	+	21	1	22
1911	1908	1915	+	1973	-	-	-	8	14	22
6329	6328	6332	+	6352	6349	6356	+	8	14	22
12612	12610	12613	-	13338	13336	13338	-	0	22	22
1620	1619	1622	+	1703	-	-	+	0	21	21
2962	2960	2963	-	3010	3008	3011	+	20	1	21
3393	3389	3393	+	13131	-	-	+	10	11	21
12635	12632	12635	+	12953	-	-	-	21	0	21

57	-	-	+	94	-	-	-	0	20	20
123	118	126	-	150	150	153	+	19	1	20
893	893	897	+	924	-	-	-	8	12	20
5177	5172	5183	+	5277	5273	5278	+	9	11	20
10277	10273	10281	-	10473	-	-	-	2	18	20
12993	12989	12994	-	14602	14601	14602	+	9	11	20
14159	14155	14161	-	14194	14191	14194	-	3	17	20
14699	14699	14701	-	14913	-	-	-	7	13	20
12582	12582	12586	-	13405	-	-	+	7	12	19
14713	14710	14716	+	14936	14933	14939	+	10	9	19
123	118	126	+	192	191	195	+	12	6	18
700	700	702	-	756	-	-	+	11	7	18
3247	3242	3250	-	3273	3270	3273	+	15	3	18
3273	3269	3275	-	3247	3247	3249	+	17	1	18
5856	5856	5858	-	6048	6048	6050	-	0	18	18
8177	8175	8177	+	8422	8422	8423	-	18	0	18
12653	12651	12654	-	14078	14078	14080	+	11	7	18
14504	14504	14506	-	14551	14549	14551	+	12	6	18
116	111	117	+	192	191	194	+	12	5	17
4889	4887	4891	+	4927	4924	4928	+	8	9	17
10125	10125	10126	-	13282	13281	13282	+	10	7	17
12425	12425	12429	-	13125	-	-	+	5	12	17
13953	13952	13957	-	14524	-	-	-	17	0	17
1632	1630	1637	+	1705	1705	1707	-	2	14	16
2023	2022	2026	-	2055	2054	2056	+	14	2	16
3690	3685	3692	-	3934	3933	3938	-	8	8	16
11373	-	-	+	11602	-	-	-	16	0	16
11671	-	-	-	13080	-	-	+	8	8	16
157	154	162	+	173	171	174	-	6	9	15
1408	1407	1411	+	1450	1449	1450	-	11	4	15
2070	2070	2072	-	2203	-	-	-	0	15	15
4001	4000	4002	+	4062	4062	4066	+	9	6	15
4667	4663	4671	-	5071	5068	5071	-	9	6	15
5525	5523	5525	-	7594	-	-	+	2	13	15
6292	6289	6294	+	6365	6362	6367	+	11	4	15
12589	12589	12593	-	12625	-	-	-	0	15	15
12896	12892	12898	-	13457	13456	13457	+	7	8	15
123	118	126	+	197	197	201	+	9	5	14
150	148	154	-	123	-	-	+	14	0	14
390	387	395	+	435	433	439	+	8	6	14
655	653	659	-	11857	11853	11857	+	14	0	14
684	680	688	-	830	829	833	-	6	8	14
707	707	708	-	748	748	749	+	8	6	14
1226	1222	1232	-	1433	1431	1437	-	8	6	14
1625	-	-	+	1708	-	-	+	14	0	14
1641	1641	1644	+	1696	-	-	-	14	0	14
1785	1783	1788	+	1766	1763	1766	-	9	5	14
7697	7695	7700	-	7912	7909	7913	-	7	7	14
9905	9904	9909	-	11549	-	-	-	4	10	14
14933	14932	14935	-	15078	-	-	-	0	14	14
290	288	293	+	310	309	313	+	5	8	13
1442	1439	1446	+	1460	-	-	-	5	8	13
1620	1619	1622	+	1710	-	-	+	13	0	13
1665	1665	1668	+	1710	1707	1710	+	7	6	13
2194	2189	2194	+	2430	2426	2430	+	11	2	13
6556	6555	6558	-	6633	-	-	-	10	3	13
9269	9265	9270	-	9440	9437	9443	-	7	6	13
9905	9905	9910	+	11549	-	-	+	12	1	13
802	798	805	+	911	911	914	+	7	5	12
1408	1407	1411	+	1440	-	-	+	6	6	12
1466	1462	1469	-	4408	4405	4408	+	9	3	12
2968	2968	2970	-	3003	-	-	+	0	12	12
3537	3534	3538	-	3723	-	-	-	6	6	12
3967	3967	3971	+	12151	-	-	+	7	5	12
4587	4583	4588	-	5640	5640	5642	-	5	7	12
4685	4682	4689	-	5068	5065	5071	-	7	5	12
6309	6308	6313	+	6351	6351	6352	+	6	6	12
6315	6314	6318	+	6351	6349	6353	+	8	4	12
7940	7937	7944	-	8047	-	-	-	1	11	12
8526	-	-	+	12660	-	-	+	10	2	12
11680	11677	11682	-	14709	14709	14712	+	5	7	12
12313	12313	12316	-	14418	14417	14418	+	7	5	12
12589	12589	12593	-	13417	-	-	-	10	2	12
13961	13960	13965	-	14784	14782	14784	+	7	5	12
74	70	79	-	264	261	265	-	7	4	11
116	111	117	+	132	129	132	+	10	1	11
465	463	471	+	491	488	492	+	4	7	11
618	615	621	-	894	891	897	-	6	5	11
1199	1197	1204	+	1243	1242	1245	+	5	6	11
2245	2244	2245	-	2268	-	-	+	9	2	11
2718	2715	2722	-	2784	2781	2785	-	9	2	11
3313	3309	3318	+	3323	3323	3326	+	6	5	11
5206	5202	5207	+	5265	5264	5266	+	6	5	11
6251	6249	6254	-	6458	6458	6461	-	6	5	11

12069	12065	12074	-	13866 -	-		-	9	2	11
12073	12073	12076	+	13876	13876	13879	+	3	8	11
12076	12076	12079	-	13879 -	-		-	0	11	11
12244 -	-		-	15194 -	-		+	7	4	11
12279	12278	12279	-	14876	14876	14877	+	6	5	11
13246	13242	13246	-	13930 -	-		+	5	6	11
13688	13686	13692	-	14854 -	-		+	5	6	11
13726	13723	13729	-	13758	13755	13758	+	9	2	11
14542	14540	14542	-	15033 -	-		+	7	4	11
116	111	117	+	185	181	187	+	6	4	10
223	221	228	+	265	262	267	+	6	4	10
365	361	365	-	566	566	569	-	6	4	10
2006	2006	2010	+	12482 -	-		+	9	1	10
2960	2959	2963	+	3011	3010	3011	-	5	5	10
3229	3225	3233	+	3217	3217	3220	+	6	4	10
4739	4736	4745	-	5418	5418	5421	-	3	7	10
4868	4868	4870	-	9714	9713	9714	+	4	6	10
6033 -	-		-	6233 -	-		-	0	10	10
6228	6223	6232	-	6458 -	-		-	9	1	10
6303	6301	6307	+	6340	6336	6344	+	5	5	10
6309	6308	6313	+	6363	6360	6367	+	7	3	10
6320	6319	6325	+	6351	6351	6352	+	4	6	10
6349	6346	6353	+	6361	6358	6361	+	1	9	10
7130	7126	7130	-	7221 -	-		-	6	4	10
7570 -	-		+	7852 -	-		+	10	0	10
10894	10893	10894	-	14650 -	-		+	3	7	10
11312 -	-		-	13701 -	-		-	8	2	10
13199	13196	13199	-	14024 -	-		+	3	7	10
14706	14705	14710	-	14933	14933	14937	-	7	3	10
15203	15202	15203	+	15235 -	-		+	7	3	10
312	307	316	-	567	565	571	-	7	2	9
368	363	373	+	490	490	491	+	5	4	9
368	363	373	+	1816	1815	1817	+	1	8	9
372	366	376	-	547	544	547	-	4	5	9
390	387	395	+	426	422	429	+	6	3	9
426	423	431	+	502	498	506	+	5	4	9
491	488	494	+	502	502	504	+	2	7	9
1112	1107	1120	+	1148	1148	1151	+	3	6	9
1193	1188	1196	+	1287	1286	1289	+	4	5	9
1796	1793	1797	+	1808	1808	1810	+	3	6	9
1911	1908	1911	-	1973	1973	1974	+	8	1	9
2028	2027	2031	+	12507	12507	12510	+	2	7	9
2662	2658	2662	+	2719 -	-		-	3	6	9
4484	4482	4485	+	4476	4475	4476	+	3	6	9
4685	4682	4689	-	5060	5060	5064	-	4	5	9
5177	5172	5183	+	5200	5200	5208	+	7	2	9
5177	5172	5183	+	5266	5262	5269	+	2	7	9
6309	6308	6313	+	6330	6326	6330	+	4	5	9
6473 -	-		-	6519 -	-		+	7	2	9
9795	9795	9796	-	14441 -	-		+	2	7	9
9948	9945	9949	-	14113	14112	14114	-	1	8	9
10193 -	-		+	10421 -	-		+	5	4	9
10496	10496	10498	+	10517	10515	10517	-	3	6	9
10699	10699	10702	-	14870	14867	14870	+	1	8	9
11055	11055	11058	-	13319 -	-		+	6	3	9
11680	11676	11682	+	13095 -	-		+	7	2	9
12658	12656	12659	+	13008 -	-		+	4	5	9
12766	12765	12767	-	14582 -	-		-	6	3	9
12896	12892	12898	-	13042 -	-		-	6	3	9
12914	12911	12914	+	14088	14085	14088	+	5	4	9
14166	14166	14170	-	14180 -	-		-	1	8	9
14359	14356	14364	-	14503 -	-		-	0	9	9
107	103	110	+	194	192	194	+	8	0	8
173	171	176	+	157	156	157	-	3	5	8
267	263	267	-	585	583	585	-	3	5	8
290	288	293	+	437	434	437	+	4	4	8
312	307	316	-	1873	1870	1874	-	3	5	8
329	328	333	-	1683	1680	1683	-	4	4	8
404	399	407	+	516	512	519	+	5	3	8
723	720	727	+	738	736	738	+	4	4	8
776	773	779	+	803	799	803	+	4	4	8
1148	1147	1152	-	1328	1327	1328	-	4	4	8
1193	1188	1196	+	1227	1227	1231	+	4	4	8
1193	1188	1196	+	1277	1275	1278	+	4	4	8
1448	1448	1451	+	6702	6702	6705	+	8	0	8
1771	1768	1772	+	1779	1779	1780	-	2	6	8
1911	1908	1915	+	14197 -	-		+	4	4	8
3055	3053	3055	+	3077 -	-		-	0	8	8
3483	3480	3486	-	3436 -	-		+	8	0	8
3690	3685	3692	-	3885	3884	3885	-	6	2	8
3701	3698	3705	-	7337	7335	7338	+	4	4	8
3765	3760	3769	+	3802	3799	3806	+	5	3	8
4145	4143	4148	-	4266	4262	4268	-	4	4	8
4145	4143	4148	-	4389	4388	4389	-	3	5	8

4586	4582	4589	+	5675	5672	5675	+	4	4	8
4893	4889	4894	-	4919	4919	4923	+	5	3	8
6398	6396	6398	+	6457	6455	6459	+	3	5	8
6584	6580	6587	-	6827	6826	6827	-	4	4	8
6674	6671	6675	-	6825	6825	6827	-	4	4	8
6674	6671	6675	-	6854	6854	6858	-	4	4	8
7246	7242	7248	-	7379	7376	7379	-	4	4	8
7991	7987	7995	-	8065	8061	8065	-	4	4	8
8423	8421	8423	+	12065	-	-	+	6	2	8
9103	9099	9103	-	9130	-	-	+	8	0	8
9601	9600	9602	-	12665	12665	12666	+	4	4	8
10082	10078	10082	+	12026	12025	12026	+	5	3	8
11186	-	-	-	11281	-	-	-	0	8	8
11688	11686	11690	+	14723	14722	14723	+	7	1	8
11925	-	-	-	14021	-	-	-	0	8	8
12451	12451	12454	-	13129	-	-	+	3	5	8
12582	12582	12586	-	13401	13398	13401	-	4	4	8
12612	12610	12613	-	12644	-	-	-	0	8	8
12672	12667	12673	-	13022	13022	13023	-	4	4	8
12873	12872	12873	+	13210	-	-	+	3	5	8
14718	14718	14720	+	14940	14940	14943	+	5	3	8
131	128	133	+	153	153	157	+	6	1	7
290	288	293	+	301	301	304	+	4	3	7
300	297	303	+	436	436	440	+	4	3	7
355	352	355	-	6685	-	-	+	0	7	7
372	366	376	-	584	580	587	-	5	2	7
465	463	471	+	498	497	501	+	4	3	7
522	518	526	-	897	896	897	-	5	2	7
684	682	687	+	832	831	833	+	6	1	7
924	921	928	+	893	-	-	-	2	5	7
972	968	972	+	992	991	992	+	4	3	7
1010	1006	1012	+	1021	-	-	-	0	7	7
1112	1107	1120	+	1143	1140	1144	+	5	2	7
1181	1178	1184	+	1287	-	-	+	4	3	7
1210	1206	1212	+	1228	1225	1228	+	3	4	7
1359	1359	1365	-	1485	1485	1490	-	4	3	7
1396	1393	1398	-	1461	1459	1462	+	3	4	7
1562	1559	1564	+	1575	1575	1577	+	3	4	7
2055	2052	2055	-	2023	-	-	+	7	0	7
2085	2083	2089	-	2112	2110	2112	+	3	4	7
2115	2111	2118	+	2156	2154	2156	+	6	1	7
2400	2396	2402	-	2536	2534	2537	-	3	4	7
2867	2863	2871	-	3022	3018	3023	-	4	3	7
3229	3225	3233	+	4486	4486	4488	+	2	5	7
3313	3309	3318	+	3331	3331	3335	+	5	2	7
3313	3309	3318	+	3340	3340	3342	+	7	0	7
4567	4561	4571	-	5700	-	-	-	2	5	7
4587	4583	4588	-	5652	5650	5655	-	4	3	7
4710	4705	4710	-	4800	-	-	-	7	0	7
4889	4887	4891	+	14343	14343	14345	+	4	3	7
4998	4995	5002	-	5192	5192	5193	-	3	4	7
6292	6289	6294	+	6554	-	-	-	7	0	7
6298	6295	6300	+	6352	-	-	+	4	3	7
6303	6301	6307	+	6317	6317	6320	+	4	3	7
6309	6308	6313	+	6346	6343	6346	+	3	4	7
6329	6328	6332	+	6363	6363	6364	+	3	4	7
6341	6336	6344	+	6364	6363	6368	+	1	6	7
7635	7631	7639	-	7910	7910	7912	-	4	3	7
7926	7923	7928	-	7986	-	-	-	3	4	7
8087	8087	8088	-	8245	8245	8246	-	0	7	7
8147	8147	8151	-	8243	-	-	-	0	7	7
8154	8153	8154	-	12921	-	-	+	7	0	7
9394	9393	9397	-	9618	-	-	-	0	7	7
9539	9539	9541	+	9752	9752	9753	+	1	6	7
10515	10515	10517	+	10498	10496	10498	-	6	1	7
12113	12109	12114	-	13058	-	-	+	5	2	7
12618	12614	12618	+	12995	-	-	-	7	0	7
12766	12765	12767	-	12802	12802	12803	+	3	4	7
12813	-	-	-	12931	-	-	-	0	7	7
12896	12892	12898	-	14077	-	-	-	3	4	7
13961	13957	13966	+	14528	14524	14528	+	6	1	7
14184	-	-	-	14731	-	-	+	4	3	7
14482	14482	14487	-	14639	-	-	-	4	3	7
88	87	92	+	63	59	63	-	1	5	6
116	111	117	+	1587	1587	1588	+	3	3	6
123	118	126	-	1566	1566	1570	-	4	2	6
136	135	139	+	194	192	197	+	4	2	6
245	242	248	-	386	-	-	-	5	1	6
273	273	278	-	560	-	-	-	6	0	6
312	307	316	-	1878	1878	1881	-	1	5	6
368	363	373	+	465	465	468	+	4	2	6
458	454	458	+	484	480	484	+	2	4	6
481	477	485	-	617	617	620	-	3	3	6
491	488	494	+	516	516	519	+	2	4	6

510	508	511	+	525	-	-	+	3	3	6
618	615	621	-	899	898	899	-	2	4	6
928	925	929	-	959	-	-	+	6	0	6
1029	1026	1031	-	1067	-	-	+	4	2	6
1029	1026	1031	-	1272	1272	1275	-	1	5	6
1139	1135	1143	-	1338	1337	1341	-	3	3	6
1193	1188	1196	+	1242	1242	1243	+	5	1	6
1264	1263	1264	-	1417	1414	1417	-	0	6	6
1466	1462	1469	-	1600	1600	1601	-	3	3	6
1660	1660	1662	+	1683	1681	1683	+	2	4	6
2115	2111	2118	+	2149	2149	2150	+	1	5	6
2181	2177	2183	-	2321	-	-	-	0	6	6
2202	2202	2203	+	2258	2257	2258	-	5	1	6
2309	2306	2313	-	2536	2535	2536	-	2	4	6
2518	2518	2521	-	2547	-	-	-	0	6	6
2745	2743	2748	+	2768	2764	2768	+	1	5	6
2882	2881	2882	+	2909	-	-	-	1	5	6
3247	3242	3250	-	3474	3471	3475	-	3	3	6
3346	3341	3350	-	3495	-	-	-	3	3	6
3373	-	-	-	3418	-	-	+	5	1	6
3519	3517	3522	-	3748	3748	3750	-	3	3	6
3557	3556	3560	+	3592	-	-	+	3	3	6
3760	3760	3761	-	4032	4028	4032	-	2	4	6
3766	3762	3769	-	4033	4032	4035	-	2	4	6
3935	3932	3938	-	4103	4100	4103	-	2	4	6
4027	4025	4030	-	4179	-	-	-	3	3	6
4667	4663	4671	-	5092	5089	5092	-	2	4	6
4691	4691	4695	-	5067	5067	5070	-	5	1	6
5257	5257	5260	-	5285	-	-	+	6	0	6
5262	5258	5264	+	5275	5275	5279	+	1	5	6
5685	5684	5685	-	5833	5833	5834	-	3	3	6
5695	5695	5696	-	5911	-	-	-	3	3	6
6303	6301	6307	+	6357	6355	6358	+	4	2	6
6650	6650	6651	+	6758	-	-	+	3	3	6
7170	7169	7174	+	7598	-	-	-	6	0	6
7645	7643	7650	-	7917	7914	7917	-	3	3	6
7697	7695	7700	-	7930	7928	7930	-	1	5	6
7699	7698	7703	+	7906	-	-	-	6	0	6
7730	7726	7732	-	7869	7868	7871	-	2	4	6
7742	-	-	-	7833	-	-	-	3	3	6
7830	-	-	-	8026	-	-	-	0	6	6
7946	7945	7947	-	8228	8228	8229	-	3	3	6
7966	7962	7966	-	8065	-	-	-	0	6	6
8113	8109	8113	+	11257	-	-	+	0	6	6
8147	8147	8151	-	13415	-	-	-	4	2	6
8272	-	-	-	8296	-	-	+	0	6	6
8467	8463	8467	-	14748	-	-	+	3	3	6
8511	8509	8515	+	11523	-	-	+	4	2	6
8727	-	-	+	9099	-	-	+	2	4	6
9341	9341	9342	-	14488	14488	14489	+	3	3	6
9394	9393	9397	-	9606	-	-	-	3	3	6
9539	9539	9541	+	13316	-	-	+	2	4	6
9753	9750	9753	-	13930	-	-	+	2	4	6
11075	11072	11079	+	13332	-	-	+	0	6	6
11250	-	-	-	11332	-	-	-	0	6	6
11361	11361	11364	+	13692	-	-	-	6	0	6
11425	11421	11427	+	11547	11547	11549	+	0	6	6
11430	11430	11432	+	11552	-	-	+	6	0	6
11554	11552	11554	-	13478	13478	13480	+	2	4	6
11920	11920	11922	-	14018	-	-	-	6	0	6
12101	12101	12103	-	13043	-	-	-	5	1	6
12509	-	-	-	14575	-	-	+	3	3	6
12529	12526	12529	-	12677	-	-	-	6	0	6
12625	12625	12629	-	13760	-	-	-	0	6	6
12886	12882	12890	-	14612	14609	14612	-	2	4	6
13013	13012	13014	-	14615	14615	14616	-	3	3	6
13640	-	-	-	13703	-	-	-	2	4	6
13664	13662	13668	-	13798	13798	13800	-	0	6	6
13780	-	-	-	14565	-	-	+	4	2	6
81	80	82	-	264	261	265	-	3	2	5
123	118	126	+	178	175	182	+	4	1	5
123	118	126	+	895	894	897	+	4	1	5
131	128	133	+	1224	1223	1228	+	3	2	5
223	221	228	+	260	260	261	-	4	1	5
308	305	314	+	320	319	320	+	2	3	5
312	307	316	-	588	584	588	-	1	4	5
320	318	323	-	584	584	588	-	3	2	5
359	355	361	+	1806	1806	1807	+	2	3	5
359	355	361	+	1829	1825	1829	+	1	4	5
368	363	373	+	6670	-	-	-	5	0	5
404	399	407	+	424	424	427	+	1	4	5
410	408	411	+	514	514	515	+	3	2	5
501	501	505	+	596	594	596	+	4	1	5
580	575	585	+	607	605	607	+	2	3	5

627	623	629	-	800	797	800	-	3	2	5
655	653	659	-	861	859	861	-	3	2	5
665	663	669	-	634	-	-	+	5	0	5
714	710	716	+	14294	-	-	+	2	3	5
745	742	745	-	779	779	782	+	5	0	5
840	839	844	-	1176	1176	1179	-	2	3	5
916	912	920	+	1120	-	-	-	5	0	5
1051	1048	1054	-	1325	1322	1328	-	1	4	5
1083	1079	1083	+	1341	1338	1341	+	2	3	5
1086	1082	1090	-	1287	1286	1287	-	2	3	5
1089	1085	1092	+	1340	-	-	+	4	1	5
1148	1147	1152	-	1875	1873	1878	-	2	3	5
1193	1188	1196	+	1211	1211	1212	+	4	1	5
1199	1197	1204	+	1228	1228	1231	+	2	3	5
1244	1243	1248	-	1436	1436	1438	-	2	3	5
1373	1371	1373	-	1564	-	-	-	2	3	5
1421	1417	1423	+	1494	1494	1497	-	3	2	5
1442	1439	1446	+	1439	-	-	+	0	5	5
1466	1462	1469	-	1677	1673	1678	-	3	2	5
1595	1591	1596	-	1883	1879	1883	-	4	1	5
1641	1641	1644	+	1684	1680	1684	+	1	4	5
1991	1988	1993	+	12112	-	-	+	2	3	5
2015	2012	2018	+	2038	2037	2038	+	2	3	5
2015	2012	2018	+	12493	-	-	+	1	4	5
2018	2014	2020	-	2216	2214	2217	-	2	3	5
2018	2014	2020	-	2307	2307	2311	-	2	3	5
2039	2035	2042	-	2095	-	-	-	0	5	5
2085	2084	2087	+	2112	2110	2112	-	0	5	5
2120	2119	2121	+	2155	2153	2157	+	3	2	5
2219	2217	2221	-	2434	-	-	-	1	4	5
2316	2312	2316	+	2371	2368	2371	+	3	2	5
2434	2430	2436	-	2640	2638	2640	-	2	3	5
2499	2498	2499	+	2598	2597	2598	+	5	0	5
2616	-	-	+	10576	-	-	+	3	2	5
2843	2841	2845	-	3043	3043	3047	-	3	2	5
2877	2872	2881	-	3021	3019	3023	-	2	3	5
2877	2872	2881	-	3105	3105	3106	-	3	2	5
2997	2992	3001	-	3221	3220	3221	+	0	5	5
3258	3255	3262	-	3436	3436	3439	-	1	4	5
3393	3389	3393	+	3401	3400	3401	+	3	2	5
3436	3432	3439	-	3630	3627	3631	-	2	3	5
3572	3570	3574	-	4294	4294	4297	-	1	4	5
3592	3588	3596	-	4295	4295	4298	-	0	5	5
3615	3614	3617	-	4290	4287	4290	-	5	0	5
3701	3701	3704	+	7337	-	-	-	5	0	5
3935	3932	3938	-	4145	4144	4145	-	1	4	5
4012	4007	4013	+	4056	4054	4061	+	3	2	5
4012	4007	4013	+	4065	4065	4069	+	4	1	5
4065	4061	4072	-	4296	4296	4300	-	0	5	5
4269	4265	4271	+	4338	4336	4338	+	3	2	5
4715	4713	4719	+	5033	5031	5033	+	3	2	5
4743	4738	4747	+	5420	5420	5422	+	3	2	5
4743	4738	4747	+	5429	5428	5431	+	2	3	5
4855	4852	4859	-	4999	4998	4999	-	2	3	5
4909	4905	4910	-	5278	-	-	-	3	2	5
5039	5039	5041	-	5207	-	-	-	5	0	5
5046	5046	5049	-	5205	-	-	-	5	0	5
5109	5104	5112	-	5321	-	-	-	0	5	5
5252	5251	5252	-	5289	-	-	+	3	2	5
5674	5671	5675	-	5820	-	-	-	2	3	5
5860	5858	5863	+	5887	-	-	+	3	2	5
6228	6223	6232	-	6388	6388	6392	-	2	3	5
6236	6234	6239	-	6458	-	-	-	2	3	5
6293	6290	6294	-	6461	6459	6464	-	1	4	5
6303	6301	6307	+	6329	6328	6329	+	4	1	5
6304	6304	6306	-	6458	6458	6459	-	2	3	5
6351	6349	6353	-	6508	6508	6511	-	2	3	5
6509	6508	6510	+	13212	-	-	+	4	1	5
6658	-	-	+	6719	-	-	+	1	4	5
6800	6800	6802	-	6887	-	-	-	0	5	5
7075	7071	7075	-	7132	-	-	-	0	5	5
7240	7236	7240	-	7387	7386	7387	-	2	3	5
7282	7279	7285	+	7314	7313	7314	+	3	2	5
7496	7495	7501	-	14603	-	-	+	4	1	5
7699	7698	7703	+	7912	-	-	+	4	1	5
7706	7706	7708	+	7718	-	-	+	2	3	5
8104	8100	8108	-	8252	8249	8252	-	3	2	5
8901	-	-	+	10042	-	-	+	3	2	5
9039	-	-	+	11049	-	-	+	3	2	5
9103	9099	9103	-	13864	-	-	+	3	2	5
9359	9358	9359	+	14477	14476	14477	+	3	2	5
9533	9530	9535	-	13258	-	-	+	1	4	5
10258	10258	10263	-	13871	13870	13871	+	3	2	5
10313	10313	10317	+	10389	10387	10389	+	0	5	5

10529	10529	10532	-	10609 -	-		-	2	3	5
11019	11019	11020	-	14566	14565	14566	+	3	2	5
11319	11319	11322	-	14470 -	-		+	2	3	5
11466	11462	11466	+	13560 -	-		+	1	4	5
11802	11802	11803	-	13645 -	-		+	0	5	5
11956	11952	11958	-	12102	12102	12104	-	2	3	5
12069	12065	12074	-	13876 -	-		-	5	0	5
12123	12122	12125	-	12342	12342	12344	-	0	5	5
12326 -	-		+	14429 -	-		+	2	3	5
12458 -	-		-	13142 -	-		-	2	3	5
12582	12582	12586	-	12620 -	-		-	5	0	5
12606	12603	12607	-	12542 -	-		+	5	0	5
12718	12718	12720	+	12765	12762	12765	+	2	3	5
12758	12754	12760	-	12930 -	-		-	0	5	5
12873	12873	12876	-	13210 -	-		-	1	4	5
13066	13066	13069	+	13097	13093	13097	+	2	3	5
13091	13087	13093	-	14288	14288	14292	+	2	3	5
13267	13267	13268	-	13487	13486	13487	+	3	2	5
13385 -	-		-	14300 -	-		+	2	3	5
13385 -	-		-	14301 -	-		-	5	0	5
13651	13649	13651	+	14029 -	-		+	0	5	5
13953	13952	13957	-	14498 -	-		-	3	2	5
14008	14004	14008	-	14076	14072	14076	-	2	3	5
14064 -	-		-	14199 -	-		-	0	5	5
14706	14705	14710	-	14920 -	-		-	3	2	5
14725	14724	14730	-	14946	14946	14947	-	0	5	5
14744 -	-		-	14918 -	-		-	1	4	5
74	70	79	-	484 -	-		-	2	2	4
107	103	110	+	124 -	-		+	4	0	4
107	103	110	+	425	425	426	+	2	2	4
116	111	117	+	153	152	153	+	3	1	4
116	111	117	+	218	216	218	+	2	2	4
123	118	126	-	301	298	301	-	2	2	4
123	118	126	+	142	142	143	+	3	1	4
123	118	126	+	373	372	374	+	3	1	4
123	118	126	+	883	883	885	+	4	0	4
123	118	126	+	1467	1467	1471	+	3	1	4
130	127	134	-	143 -	-		+	0	4	4
130	127	134	-	362	362	365	-	2	2	4
131	128	133	+	143	141	143	-	4	0	4
131	128	133	+	191 -	-		+	3	1	4
131	128	133	+	1094 -	-		+	2	2	4
157	154	162	+	13385	13385	13386	+	1	3	4
175	171	179	-	157 -	-		+	4	0	4
186	184	187	+	192 -	-		+	2	2	4
229	224	231	-	246 -	-		+	2	2	4
245	242	248	-	584 -	-		-	2	2	4
262	260	262	-	464	464	465	-	2	2	4
267	263	267	-	410 -	-		-	1	3	4
267	263	267	-	760	760	762	-	4	0	4
267	263	267	-	4058 -	-		-	2	2	4
290	288	293	+	336 -	-		+	2	2	4
312	307	316	-	606	606	607	-	2	2	4
316	315	321	+	329 -	-		+	2	2	4
329	328	333	-	541 -	-		-	2	2	4
355	352	355	-	439 -	-		-	2	2	4
368	363	373	+	544	541	545	+	1	3	4
368	363	373	+	1803 -	-		+	1	3	4
372	366	376	-	521	521	522	-	2	2	4
372	366	376	-	659	659	660	-	2	2	4
404	399	407	+	502	502	506	+	1	3	4
426	423	431	+	459	455	463	+	3	1	4
466	462	471	-	629	629	631	-	1	3	4
481	477	485	-	798 -	-		-	2	2	4
511	506	515	-	668 -	-		-	2	2	4
522	518	526	-	655	653	655	-	2	2	4
611	611	614	-	696 -	-		-	0	4	4
627	623	629	-	893	893	898	-	2	2	4
692	688	695	+	833	832	835	+	3	1	4
750	747	750	-	774 -	-		+	0	4	4
802	798	805	+	832 -	-		+	2	2	4
832	827	835	-	1174	1174	1176	-	2	2	4
883	881	886	+	979	979	980	+	1	3	4
898	894	899	-	1003 -	-		-	2	2	4
898	894	899	-	1073 -	-		-	2	2	4
958	958	960	+	974	972	974	+	2	2	4
970	968	973	-	1150 -	-		-	2	2	4
970	968	973	-	1387	1386	1387	-	1	3	4
997	995	997	-	1227 -	-		-	2	2	4
1029	1027	1031	+	1053	1051	1053	+	2	2	4
1029	1027	1031	+	1089	1088	1089	+	1	3	4
1051	1048	1054	-	1316	1315	1318	-	1	3	4
1096	1096	1100	-	1178 -	-		-	2	2	4
1109	1107	1110	-	1211 -	-		-	2	2	4

1112	1107	1120	+	1136	1136	1138	+	2	2	4
1148	1147	1152	-	1316	1316	1317	-	2	2	4
1148	1147	1152	-	1346	1345	1347	-	2	2	4
1181	1178	1184	+	15123	-	-	+	2	2	4
1199	1197	1204	+	1217	1217	1218	+	0	4	4
1210	1206	1212	+	1255	1255	1256	+	3	1	4
1226	1222	1232	-	1526	1526	1528	-	3	1	4
1383	1379	1383	-	1428	-	-	-	2	2	4
1388	1385	1391	-	1550	-	-	-	2	2	4
1388	1385	1391	-	1598	1598	1599	-	1	3	4
1434	1431	1436	+	1467	1467	1468	+	3	1	4
1437	1434	1437	-	1839	1839	1840	-	2	2	4
1460	1457	1463	+	1397	1397	1398	-	2	2	4
1498	1497	1498	+	1515	-	-	+	2	2	4
1562	1559	1564	+	1708	-	-	+	2	2	4
1580	1575	1584	+	1595	1591	1600	+	1	3	4
1580	1575	1584	+	1808	1805	1808	+	2	2	4
1615	1611	1615	-	1753	-	-	-	2	2	4
1678	1676	1680	-	1808	1808	1809	-	2	2	4
1683	1679	1685	+	6510	-	-	+	2	2	4
1735	1732	1738	-	1873	1873	1874	-	4	0	4
1897	1897	1901	+	11503	-	-	+	1	3	4
1924	1922	1928	+	1942	-	-	+	2	2	4
1924	1922	1928	+	1962	1961	1962	-	3	1	4
1948	1948	1951	+	8699	-	-	+	3	1	4
1954	1953	1957	+	3521	3521	3522	+	2	2	4
1964	1961	1968	+	2227	2227	2229	+	2	2	4
1973	1969	1974	-	1911	-	-	+	4	0	4
1976	1972	1979	+	1995	1991	1996	+	3	1	4
1985	1981	1986	+	2115	2113	2115	+	1	3	4
1985	1981	1986	+	2584	2583	2584	+	2	2	4
1991	1988	1995	-	2127	2127	2130	-	2	2	4
1991	1988	1995	-	2139	-	-	-	2	2	4
1991	1988	1995	-	2149	2149	2154	-	1	3	4
1991	1988	1995	-	2228	2226	2228	-	1	3	4
2028	2028	2030	-	12510	12507	12510	-	3	1	4
2039	2035	2042	-	2269	2269	2274	-	2	2	4
2039	2035	2042	-	12518	12514	12518	-	0	4	4
2109	2106	2112	-	2170	-	-	+	0	4	4
2170	2165	2170	-	2205	-	-	-	0	4	4
2170	2165	2170	-	6458	-	-	-	2	2	4
2219	2217	2221	-	10372	-	-	-	2	2	4
2252	2252	2256	-	14654	-	-	-	1	3	4
2434	2430	2436	-	2622	-	-	-	3	1	4
2466	2466	2468	-	2443	-	-	+	4	0	4
2512	2511	2512	-	2825	-	-	-	2	2	4
2537	2534	2537	-	2771	2771	2774	-	2	2	4
2583	2581	2586	-	2723	-	-	-	2	2	4
2696	-	-	+	4320	-	-	+	0	4	4
2730	2727	2733	-	2956	-	-	-	2	2	4
2769	2765	2769	-	2868	-	-	-	4	0	4
2775	2772	2775	-	2874	-	-	-	0	4	4
2867	2863	2871	-	3045	3043	3045	-	1	3	4
2927	2924	2930	-	3150	3148	3150	-	1	3	4
2951	2947	2951	-	3150	3150	3151	-	1	3	4
2975	2972	2976	+	3017	3017	3020	+	2	2	4
3055	3053	3055	+	15066	-	-	+	1	3	4
3155	3152	3156	-	4267	-	-	-	2	2	4
3220	-	-	-	3229	-	-	-	4	0	4
3247	3242	3250	-	3481	3481	3482	-	2	2	4
3273	3269	3275	-	3494	-	-	-	3	1	4
3313	3309	3318	+	3365	-	-	+	2	2	4
3368	3368	3370	+	3462	-	-	+	2	2	4
3373	3373	3375	+	3419	-	-	+	2	2	4
3406	3402	3410	-	11716	-	-	-	0	4	4
3446	3444	3449	-	4069	-	-	-	2	2	4
3577	3575	3579	-	3786	-	-	-	2	2	4
3617	3617	3619	+	3684	-	-	+	2	2	4
3629	3628	3631	-	3727	3724	3727	-	2	2	4
3629	3628	3631	-	3785	-	-	-	2	2	4
3640	3639	3644	+	3688	-	-	+	2	2	4
3640	3639	3644	+	13497	-	-	+	2	2	4
3661	3658	3663	+	3678	3677	3678	+	1	3	4
3672	-	-	-	3821	-	-	-	2	2	4
3672	3668	3674	+	3690	3688	3690	+	2	2	4
3678	3675	3678	-	3712	-	-	-	0	4	4
3701	3701	3704	+	3749	-	-	+	2	2	4
3711	-	-	+	4026	4026	4028	+	2	2	4
3721	3721	3724	-	4434	4431	4434	-	2	2	4
3750	3748	3754	+	3763	-	-	+	2	2	4
3795	3793	3796	-	4025	4021	4025	-	2	2	4
3801	3799	3805	-	3961	3961	3962	-	2	2	4
3859	3857	3862	-	4103	4100	4103	-	3	1	4
3859	3857	3862	-	4146	4146	4147	-	1	3	4

3921	3917	3922	-	3994 -	-		-	2	2	4
3935	3932	3938	-	4029	4027	4029	-	2	2	4
3935	3932	3938	-	6670 -	-		+	0	4	4
4020	4018	4024	+	4042	4038	4043	+	1	3	4
4102	4100	4106	-	4266	4265	4270	-	2	2	4
4102	4100	4106	-	4294	4291	4294	-	2	2	4
4268	4264	4268	-	4539 -	-		-	2	2	4
4278	4278	4281	+	4319	4317	4319	+	0	4	4
4299	4296	4302	-	4465	4463	4465	-	3	1	4
4336	4335	4336	-	4568	4564	4568	-	2	2	4
4434	4430	4436	-	4587	4587	4591	-	2	2	4
4567	4561	4571	-	5253	5250	5253	-	2	2	4
4567	4561	4571	-	5603 -	-		-	2	2	4
4567	4561	4571	-	5833	5832	5833	-	2	2	4
4613	4609	4614	-	5060	5060	5061	-	2	2	4
4658	4654	4660	-	5091	5091	5092	-	2	2	4
4667	4663	4671	-	4708 -	-		-	0	4	4
4667	4663	4671	-	5603	5601	5603	-	2	2	4
4715	4712	4715	-	5031	5027	5033	-	3	1	4
4739	4736	4745	-	4908	4908	4909	-	2	2	4
4749	4749	4751	+	4785	4782	4785	+	2	2	4
4753	4750	4757	-	5001	4998	5002	-	2	2	4
4772	4772	4773	+	5364 -	-		+	2	2	4
4779	4776	4783	-	5025	5025	5028	-	2	2	4
4813	4811	4817	-	5028	5026	5032	-	4	0	4
4853	4851	4855	+	4999	4999	5001	+	1	3	4
4855	4852	4859	-	4974 -	-		-	2	2	4
4909	4905	4910	-	5071	5067	5072	-	2	2	4
4909	4905	4910	-	5138	5138	5139	-	2	2	4
4909	4905	4910	-	5203	5203	5208	-	2	2	4
4909	4905	4910	-	5265	5265	5268	-	3	1	4
4909	4905	4913	+	4926	4925	4930	+	3	1	4
4928	4925	4932	+	4957	4957	4959	+	2	2	4
4928	4925	4932	+	4971 -	-		+	2	2	4
4956	4955	4958	-	5177 -	-		-	2	2	4
4963	4963	4966	-	5110 -	-		-	2	2	4
4998	4995	5002	-	5132	5132	5133	-	2	2	4
5093	5093	5097	-	5326	5326	5330	-	1	3	4
5177	5173	5179	-	5275	5273	5277	-	2	2	4
5273	5272	5276	-	5499 -	-		-	2	2	4
5280	5277	5280	-	5262 -	-		+	4	0	4
5654	5651	5655	-	5819 -	-		-	2	2	4
5703	5700	5704	-	5815	5811	5815	-	2	2	4
6228	6223	6232	-	6397 -	-		-	2	2	4
6278	6275	6279	+	6351	6351	6352	+	2	2	4
6298	6295	6300	+	6329	6325	6329	+	2	2	4
6298	6295	6300	+	6360	6360	6364	+	3	1	4
6303	6301	6307	+	6906 -	-		+	2	2	4
6315	6314	6318	+	6335	6332	6335	+	2	2	4
6325	6324	6328	-	6559	6559	6560	+	0	4	4
6356	6356	6359	+	6372	6369	6372	+	3	1	4
6520	6519	6524	-	6473 -	-		+	4	0	4
6520	6519	6524	-	6718	6716	6718	-	2	2	4
6520	6519	6524	-	13229 -	-		-	0	4	4
6576	6573	6579	-	6707 -	-		-	2	2	4
6576	6573	6579	-	12322 -	-		+	3	1	4
6584	6580	6587	-	6837	6837	6838	-	2	2	4
6617	6617	6618	+	6644	6644	6645	+	2	2	4
6640	6639	6645	-	6785 -	-		-	2	2	4
6674	6671	6675	-	6860 -	-		-	2	2	4
6695	6693	6697	-	6719	6717	6719	+	3	1	4
6700	6699	6704	+	6808	6805	6808	+	2	2	4
6732 -	-		+	6934 -	-		+	2	2	4
6878	6874	6881	+	7345 -	-		+	2	2	4
6912	6908	6913	-	8209 -	-		+	2	2	4
7084	7081	7087	-	7198	7198	7200	-	2	2	4
7209	7205	7209	-	7378 -	-		-	2	2	4
7392	7392	7397	-	8400	8399	8400	-	3	1	4
7469 -	-		+	7452 -	-		-	0	4	4
7496	7495	7501	-	7589	7588	7589	-	3	1	4
7622	7619	7623	+	9501 -	-		-	4	0	4
7655	7654	7658	-	7972	7972	7973	-	3	1	4
7797	7795	7797	-	9224 -	-		-	3	1	4
7926	7923	7928	-	8163 -	-		-	0	4	4
7940	7937	7944	-	7991	7991	7992	-	2	2	4
7976	7976	7979	-	8350 -	-		-	1	3	4
8140	8136	8140	-	8236 -	-		-	4	0	4
8199 -	-		+	8217 -	-		+	2	2	4
8263	8263	8264	+	8304	8304	8305	-	2	2	4
8264	8263	8264	-	8304	8304	8305	+	4	0	4
9220	9218	9220	+	9459	9459	9461	-	4	0	4
9302 -	-		-	9487 -	-		-	2	2	4
9350 -	-		-	9645 -	-		-	2	2	4
9383	9379	9384	-	9492	9492	9493	-	2	2	4

9649	-	-	+	9945	-	-	-	4	0	4
9942	9941	9946	+	14112	14109	14112	+	0	4	4
9994	9994	9995	-	10235	10235	10236	-	2	2	4
10043	10041	10043	-	13683	13681	13683	+	2	2	4
10097	-	-	-	10211	-	-	-	4	0	4
10125	10125	10126	-	13281	-	-	-	4	0	4
10284	-	-	-	13878	-	-	-	4	0	4
10326	-	-	+	10494	-	-	+	2	2	4
11122	11120	11122	+	13498	-	-	+	4	0	4
11218	11214	11222	-	14613	-	-	+	2	2	4
11356	-	-	-	14253	-	-	+	2	2	4
11395	11395	11398	+	11729	-	-	-	4	0	4
11430	11430	11432	+	11525	-	-	+	3	1	4
11508	11506	11510	-	11652	11652	11654	-	0	4	4
11575	11575	11577	-	13732	13732	13734	-	1	3	4
11726	11722	11727	+	12930	-	-	+	2	2	4
12023	12023	12024	+	12120	-	-	+	2	2	4
12069	12065	12074	-	13856	13852	13856	-	3	1	4
12138	-	-	-	12290	-	-	-	3	1	4
12149	12147	12149	-	12277	-	-	-	0	4	4
12300	12299	12300	+	12376	-	-	+	1	3	4
12301	12297	12303	-	12395	12391	12395	-	1	3	4
12326	-	-	-	14429	-	-	-	1	3	4
12399	12396	12399	+	13813	-	-	+	2	2	4
12514	12514	12518	-	12640	-	-	-	0	4	4
12625	12623	12629	+	13760	-	-	+	4	0	4
12672	12667	12673	-	14085	14085	14086	-	1	3	4
12696	-	-	+	12869	-	-	+	2	2	4
12758	12754	12760	-	13061	-	-	+	3	1	4
12930	12929	12935	+	14094	14094	14095	+	0	4	4
12947	12944	12948	+	13277	-	-	-	4	0	4
12993	12989	12994	-	14582	-	-	-	2	2	4
13039	13038	13039	-	13199	-	-	-	2	2	4
13199	13196	13199	-	14014	-	-	-	1	3	4
13293	13293	13298	-	13509	-	-	-	0	4	4
13329	-	-	-	13457	-	-	-	2	2	4
13345	13345	13346	+	13392	13392	13393	+	2	2	4
13680	13677	13680	-	14845	-	-	-	2	2	4
13910	-	-	-	14884	-	-	-	4	0	4
14442	-	-	-	14513	-	-	-	0	4	4
14706	14705	14710	-	14834	14834	14836	-	3	1	4
14720	14716	14720	-	14938	-	-	-	0	4	4
14734	-	-	-	14971	-	-	-	2	2	4
15069	15069	15072	-	15204	-	-	-	2	2	4
74	70	79	-	291	291	294	-	2	1	3
107	103	110	+	118	-	-	+	3	0	3
107	103	110	+	132	132	136	+	2	1	3
107	103	110	+	153	152	153	+	2	1	3
107	103	110	+	611	607	611	+	2	1	3
107	103	110	+	1825	1823	1825	+	1	2	3
116	111	117	+	424	424	428	+	2	1	3
116	111	117	+	894	-	-	+	2	1	3
123	118	126	+	425	425	428	+	3	0	3
123	118	126	+	1341	1339	1341	+	2	1	3
130	127	134	-	297	294	298	-	1	2	3
130	127	134	-	572	568	572	-	2	1	3
156	156	159	-	173	-	-	+	3	0	3
175	171	179	-	306	-	-	-	1	2	3
200	197	201	+	309	309	310	+	1	2	3
242	242	247	+	266	-	-	+	2	1	3
245	242	248	-	442	442	443	-	2	1	3
290	288	293	+	344	-	-	+	1	2	3
291	289	293	-	542	542	545	-	1	2	3
300	297	303	+	321	321	323	+	1	2	3
304	299	304	-	1868	1868	1869	-	2	1	3
308	305	314	+	328	328	329	+	2	1	3
308	305	314	+	339	336	339	+	1	2	3
308	305	314	+	432	432	435	+	2	1	3
308	305	314	+	625	-	-	+	3	0	3
312	307	316	-	601	601	603	-	1	2	3
312	307	316	-	831	831	832	-	1	2	3
312	307	316	-	1868	-	-	-	1	2	3
365	361	365	-	572	-	-	-	2	1	3
368	363	373	+	531	529	531	+	1	2	3
372	366	376	-	490	-	-	-	2	1	3
372	366	376	-	1110	1110	1111	-	2	1	3
397	396	397	+	414	414	416	+	2	1	3
404	399	407	+	522	-	-	+	2	1	3
404	399	407	+	529	528	529	+	1	2	3
410	408	411	+	451	451	453	+	1	2	3
411	408	416	-	1673	1673	1677	-	0	3	3
416	413	422	+	490	490	491	+	1	2	3
416	413	422	+	502	501	502	+	1	2	3
421	419	423	-	625	625	626	-	0	3	3

449	448	450	+	481	-	-		+	1	2	3
457	455	459	-	617		615	617	-	1	2	3
458	454	458	+	491		487	491	+	1	2	3
466	462	471	-	759		759	761	-	3	0	3
481	477	485	-	736		733	736	-	2	1	3
481	477	485	-	890		890	894	-	2	1	3
481	477	485	-	929		929	932	-	2	1	3
481	477	485	-	967	-	-		-	3	0	3
491	488	494	+	508		508	510	+	1	2	3
501	501	505	+	589	-	-		+	3	0	3
569	567	574	-	1227		1226	1227	-	2	1	3
569	566	570	+	585		585	588	+	1	2	3
580	575	585	+	588		584	588	+	2	1	3
580	575	585	+	618		618	620	+	2	1	3
585	581	590	-	974		974	976	-	3	0	3
585	581	590	-	1141		1137	1141	-	1	2	3
589	588	593	+	589	-	-		+	0	3	3
618	615	621	-	883		883	887	-	1	2	3
618	615	621	-	985		985	989	-	1	2	3
627	623	629	-	969	-	-		-	3	0	3
634	634	638	-	665	-	-		+	3	0	3
654	652	658	+	834	-	-		+	1	2	3
684	682	687	+	804		800	806	+	2	1	3
692	688	695	+	763	-	-		+	3	0	3
760	756	762	-	700	-	-		+	3	0	3
776	773	779	+	791		790	791	+	2	1	3
799	795	803	-	1198		1198	1200	-	1	2	3
799	795	803	-	1229		1227	1229	-	1	2	3
802	798	805	+	839		839	841	+	1	2	3
817	813	820	+	834		834	835	+	1	2	3
832	827	835	-	1051		1047	1053	-	2	1	3
832	827	835	-	1192		1192	1193	-	1	2	3
832	827	835	-	11482	-	-		+	0	3	3
839	835	840	+	856		855	856	+	2	1	3
840	839	844	-	1027		1027	1028	-	2	1	3
860	857	864	-	1050		1048	1050	-	1	2	3
883	881	886	+	929		929	930	+	2	1	3
916	912	920	+	951		951	955	+	1	2	3
924	921	928	+	943		939	943	+	2	1	3
924	921	928	+	969		969	972	+	1	2	3
924	921	928	+	1223		1223	1224	+	1	2	3
1010	1006	1012	+	1074		1074	1075	+	2	1	3
1020	1018	1025	+	1010		1009	1010	-	3	0	3
1020	1018	1025	+	1074		1073	1074	+	1	2	3
1046	1043	1047	-	1227	-	-		-	2	1	3
1046	1043	1047	-	1312	-	-		-	3	0	3
1046	1042	1050	+	1042		1042	1046	+	1	2	3
1051	1048	1054	-	1224		1224	1227	-	2	1	3
1051	1048	1054	-	1273		1271	1273	-	1	2	3
1068	1068	1072	-	1092		1091	1092	+	3	0	3
1089	1085	1092	+	1139	-	-		+	1	2	3
1089	1085	1092	+	1146		1144	1148	+	1	2	3
1109	1107	1110	-	1328	-	-		-	1	2	3
1112	1107	1120	+	1259		1259	1261	+	2	1	3
1130	1129	1134	-	1327		1326	1327	-	2	1	3
1139	1135	1143	-	1348	-	-		-	2	1	3
1139	1135	1143	-	1601	-	-		-	3	0	3
1181	1178	1184	+	1212		1212	1213	+	1	2	3
1193	1188	1196	+	1247		1246	1247	+	2	1	3
1210	1206	1212	+	1815		1815	1816	+	0	3	3
1224	1224	1228	+	1243		1243	1247	+	0	3	3
1226	1222	1232	-	5977	-	-		+	0	3	3
1400	1397	1401	+	1428		1428	1429	+	1	2	3
1460	1457	1463	+	1442	-	-		-	2	1	3
1491	1489	1495	-	1565	-	-		-	2	1	3
1493	1493	1494	+	1493	-	-		+	3	0	3
1545	1543	1549	+	1600		1600	1601	+	2	1	3
1562	1559	1564	+	1598	-	-		+	2	1	3
1580	1575	1584	+	1606		1603	1606	+	3	0	3
1592	1587	1596	+	1713	-	-		+	0	3	3
1607	1606	1607	+	1709	-	-		+	0	3	3
1625	1621	1629	-	1793		1793	1794	-	3	0	3
1637	1636	1637	-	1667	-	-		-	3	0	3
1653	1653	1654	+	1685		1685	1687	+	1	2	3
1683	1679	1685	+	1719		1716	1720	+	1	2	3
1802	1798	1804	-	6458		6454	6458	-	3	0	3
1840	1836	1840	-	1997	-	-		-	3	0	3
1905	1905	1906	+	1901		1900	1901	+	3	0	3
1911	1908	1911	-	15363		15362	15363	-	1	2	3
1923	1922	1925	-	1961		1961	1962	+	2	1	3
1976	1972	1979	+	1911	-	-		-	3	0	3
1991	1988	1993	+	14729	-	-		+	3	0	3
2018	2014	2020	-	2185		2185	2189	-	1	2	3
2023	2022	2026	+	12505	-	-		+	2	1	3

2039	2038	2039	+	11855	-	-	+	0	3	3
2049	2046	2050	+	12529	12525	12529	+	1	2	3
2050	2046	2050	-	12529	12526	12529	-	0	3	3
2065	2065	2067	-	2198	-	-	-	3	0	3
2100	2099	2100	+	2217	-	-	+	2	1	3
2109	2106	2112	-	2295	-	-	-	2	1	3
2115	2111	2118	+	2085	-	-	-	1	2	3
2115	2111	2118	+	2162	2162	2165	+	1	2	3
2170	2165	2170	-	2835	-	-	-	2	1	3
2209	2209	2212	+	2191	2188	2191	-	1	2	3
2268	-	-	-	2245	-	-	+	3	0	3
2273	2271	2273	+	2383	-	-	+	1	2	3
2395	2393	2395	-	2534	2534	2535	-	2	1	3
2436	2433	2440	+	2511	2511	2512	+	2	1	3
2457	2457	2458	+	12975	-	-	+	3	0	3
2713	-	-	+	2913	-	-	+	0	3	3
2730	2727	2733	-	2919	-	-	-	3	0	3
2757	2755	2757	-	2799	-	-	-	1	2	3
2835	2834	2839	-	3206	-	-	-	2	1	3
2843	2838	2843	+	13241	-	-	+	3	0	3
2874	2872	2876	+	2927	-	-	+	3	0	3
2877	2872	2881	-	3062	-	-	-	2	1	3
2911	2908	2915	-	3187	3187	3188	-	2	1	3
2975	2974	2976	-	3048	-	-	-	3	0	3
2975	2972	2976	+	2991	2989	2991	+	1	2	3
2997	2992	3001	-	3209	3207	3209	-	1	2	3
2997	2992	3001	-	3360	-	-	-	3	0	3
3038	3038	3042	-	3205	-	-	-	3	0	3
3060	-	-	+	3072	-	-	-	3	0	3
3065	3062	3068	+	3098	-	-	-	1	2	3
3091	-	-	+	3709	-	-	+	2	1	3
3229	3225	3233	+	3335	3331	3335	+	1	2	3
3247	3242	3250	-	6459	-	-	-	3	0	3
3258	3255	3262	-	3482	-	-	-	0	3	3
3258	3255	3262	-	3753	-	-	-	0	3	3
3267	3265	3269	+	11191	-	-	+	1	2	3
3273	3269	3275	-	3377	-	-	-	2	1	3
3273	3269	3275	-	4466	4463	4466	-	2	1	3
3274	3273	3277	+	4856	-	-	+	3	0	3
3295	-	-	+	3359	-	-	+	0	3	3
3302	3302	3304	+	3325	3323	3325	+	2	1	3
3313	3309	3318	+	3940	-	-	+	2	1	3
3329	3325	3329	-	3360	-	-	+	2	1	3
3346	3341	3350	-	4385	4385	4388	-	3	0	3
3378	3377	3382	+	3422	3420	3422	-	2	1	3
3546	3546	3547	+	4310	4310	4311	+	1	2	3
3557	3556	3560	+	3669	3669	3673	+	1	2	3
3576	3575	3579	+	3643	-	-	+	0	3	3
3577	3575	3579	-	3748	-	-	-	1	2	3
3577	3575	3579	-	4310	-	-	-	2	1	3
3624	3624	3627	+	3647	-	-	+	1	2	3
3629	3628	3631	-	4266	4266	4267	-	2	1	3
3640	3639	3644	+	4105	4103	4107	+	2	1	3
3643	3640	3646	-	3990	3986	3990	-	1	2	3
3643	3640	3646	-	4268	4265	4268	-	2	1	3
3661	3658	3663	+	3688	-	-	+	3	0	3
3690	3686	3692	+	3934	3931	3935	+	3	0	3
3721	3721	3724	-	7317	7313	7317	+	1	2	3
3736	3736	3737	-	3827	3827	3828	-	1	2	3
3749	3748	3750	-	4034	4033	4034	-	2	1	3
3766	3762	3769	-	4021	4020	4021	-	2	1	3
3786	3784	3790	-	4027	4023	4031	-	1	2	3
3787	3787	3791	+	4054	4054	4056	+	3	0	3
3886	3885	3886	-	4103	4100	4103	-	1	2	3
3929	-	-	-	4125	-	-	-	1	2	3
3935	3932	3938	-	4266	4266	4269	-	2	1	3
3979	3975	3979	+	4127	4123	4127	+	2	1	3
4012	4007	4013	+	4039	4036	4039	+	1	2	3
4020	4018	4024	+	4048	-	-	+	2	1	3
4037	4034	4041	-	4414	-	-	-	2	1	3
4059	4058	4059	-	4299	4298	4299	-	1	2	3
4065	4061	4072	-	4277	4277	4278	-	1	2	3
4075	4075	4077	-	4272	-	-	-	3	0	3
4166	4164	4169	-	4237	-	-	-	2	1	3
4227	4223	4228	-	4403	-	-	-	1	2	3
4257	4257	4258	-	4423	4423	4424	-	0	3	3
4290	4290	4293	+	4323	4320	4324	+	2	1	3
4389	4389	4393	-	4514	-	-	-	1	2	3
4517	4517	4521	-	4590	-	-	-	1	2	3
4522	4521	4522	+	4565	4565	4567	+	2	1	3
4537	4536	4540	-	5853	-	-	-	0	3	3
4537	4536	4540	-	5912	-	-	-	0	3	3
4567	4561	4571	-	5821	5820	5821	-	1	2	3
4598	4597	4601	-	5353	5350	5353	-	1	2	3

4606	4606	4609	+	6083	6082	6083	+	2	1	3
4624	4621	4624	+	5589	-	-	+	2	1	3
4632	4629	4634	-	4847	4846	4847	-	2	1	3
4658	4654	4660	-	5068	-	-	-	1	2	3
4684	4683	4687	+	5066	5066	5067	+	1	2	3
4685	4682	4689	-	5073	5073	5076	-	3	0	3
4685	4682	4689	-	5087	5084	5091	-	1	2	3
4697	4696	4697	-	4929	-	-	-	3	0	3
4700	-	-	+	5046	-	-	-	3	0	3
4715	4712	4715	-	5430	5430	5433	-	1	2	3
4715	4712	4715	-	5451	5449	5451	-	0	3	3
4729	4727	4737	+	4756	4756	4760	+	0	3	3
4743	4738	4747	+	5435	5433	5435	+	2	1	3
4813	4811	4817	-	5109	5109	5113	-	1	2	3
4813	4811	4817	-	5326	5322	5326	-	1	2	3
4853	4851	4855	+	4924	4924	4927	+	1	2	3
4853	4851	4855	+	4957	-	-	+	2	1	3
4855	4852	4859	-	4877	-	-	+	3	0	3
4855	4852	4859	-	5176	5176	5180	-	0	3	3
4859	4858	4859	+	4877	4877	4878	-	0	3	3
4920	4918	4922	+	4982	4978	4982	+	0	3	3
4928	4925	4932	+	4966	-	-	+	2	1	3
4928	4925	4932	+	4989	4986	4989	+	1	2	3
4935	4934	4938	+	4977	4973	4977	+	1	2	3
4940	4940	4943	-	12865	-	-	-	0	3	3
4979	4974	4984	-	5216	5216	5217	-	1	2	3
4998	4995	5002	-	5152	5152	5154	-	2	1	3
4998	4995	5002	-	5177	-	-	-	2	1	3
5029	5027	5033	-	5606	-	-	-	3	0	3
5054	-	-	-	6113	-	-	-	0	3	3
5121	5118	5125	-	5489	5489	5493	-	3	0	3
5177	5172	5183	+	5654	5654	5655	+	1	2	3
5208	5205	5209	-	5652	5652	5655	-	0	3	3
5294	5293	5297	-	5537	-	-	-	3	0	3
5321	5320	5324	-	5518	5518	5522	-	1	2	3
5334	5332	5338	+	5747	5747	5749	-	3	0	3
5359	5356	5362	-	5538	-	-	-	1	2	3
5434	5430	5439	-	5695	5694	5695	-	0	3	3
5502	5502	5504	-	10937	-	-	+	2	1	3
5917	-	-	-	6026	-	-	-	3	0	3
6228	6223	6232	-	6353	6352	6353	-	2	1	3
6228	6223	6232	-	6510	6508	6510	-	0	3	3
6242	6240	6247	-	6491	6491	6494	-	2	1	3
6242	6240	6247	-	6519	6518	6519	-	1	2	3
6252	6250	6255	+	6368	-	-	+	3	0	3
6267	6264	6267	+	6366	-	-	+	3	0	3
6285	6282	6287	+	6357	6356	6357	+	2	1	3
6292	6289	6294	+	6350	-	-	+	2	1	3
6292	6289	6294	+	6773	-	-	-	3	0	3
6309	6308	6313	+	6341	-	-	+	2	1	3
6315	6314	6318	+	6344	-	-	+	0	3	3
6320	6315	6320	-	6494	6492	6494	-	2	1	3
6320	6319	6325	+	6346	-	-	+	3	0	3
6356	6356	6359	+	6523	6523	6526	+	0	3	3
6370	6368	6374	-	8803	-	-	+	3	0	3
6413	-	-	+	6456	6455	6456	+	1	2	3
6450	-	-	-	6402	-	-	+	3	0	3
6533	6533	6536	+	6524	-	-	+	0	3	3
6576	6573	6579	-	6809	-	-	-	3	0	3
6584	6580	6587	-	6710	6709	6710	-	1	2	3
6584	6580	6587	-	6821	-	-	-	1	2	3
6640	6639	6645	-	6873	6873	6876	-	2	1	3
6640	6639	6645	-	7842	7842	7845	-	2	1	3
6651	6648	6651	-	6681	-	-	-	0	3	3
6674	6671	6675	-	6887	-	-	-	2	1	3
6710	6708	6712	-	6947	6947	6950	-	2	1	3
6722	6722	6725	+	6743	6743	6744	+	2	1	3
6742	6738	6742	+	6762	6760	6762	+	2	1	3
6754	6754	6755	+	6769	6769	6770	+	2	1	3
6941	6941	6944	+	6959	-	-	-	1	2	3
7069	7064	7069	-	7620	7620	7622	-	1	2	3
7098	7098	7101	-	7134	-	-	-	1	2	3
7181	7181	7183	+	7587	-	-	-	2	1	3
7195	7190	7198	+	7224	-	-	+	2	1	3
7200	7200	7203	-	7378	-	-	-	1	2	3
7209	7205	7209	-	7448	-	-	-	2	1	3
7423	-	-	-	7662	-	-	-	3	0	3
7556	7556	7560	-	7638	-	-	-	1	2	3
7564	7560	7564	+	7846	-	-	+	0	3	3
7635	7631	7639	-	7787	7786	7787	-	1	2	3
7649	7647	7649	+	7849	-	-	-	3	0	3
7677	7673	7678	+	9361	9360	9361	-	2	1	3
7697	7695	7700	-	7919	-	-	-	0	3	3
7773	7771	7775	+	7814	7810	7814	+	2	1	3

7825	-	-	-	11157	-	-	-	0	3	3
7920	7918	7920	-	8175	8172	8175	-	0	3	3
7966	7962	7966	-	7995	-	-	-	0	3	3
7976	7976	7981	+	8130	8130	8131	+	2	1	3
7988	7986	7992	+	8026	8026	8027	+	2	1	3
7991	7987	7995	-	8195	8191	8195	-	0	3	3
8064	8064	8066	+	8041	8041	8043	-	1	2	3
8131	8127	8134	-	8283	8283	8284	-	2	1	3
8562	8561	8562	+	8711	8710	8711	+	1	2	3
8614	8614	8616	+	8990	-	-	+	0	3	3
8749	8745	8749	-	8900	-	-	-	0	3	3
8831	8827	8831	-	11351	-	-	+	1	2	3
8944	8942	8947	-	14601	-	-	+	1	2	3
9053	-	-	-	13167	-	-	+	3	0	3
9130	9130	9135	-	9103	-	-	+	3	0	3
9524	9522	9524	-	12382	-	-	-	3	0	3
9673	9672	9673	-	11694	11694	11695	+	1	2	3
9802	9802	9804	+	14446	14446	14448	+	1	2	3
9857	9857	9860	-	14300	-	-	+	1	2	3
9994	-	-	+	9970	-	-	-	2	1	3
10136	10133	10138	-	13293	13291	13293	-	2	1	3
10277	10273	10281	-	10485	-	-	-	1	2	3
10284	10282	10284	+	13878	-	-	+	1	2	3
10521	10521	10522	-	10606	-	-	-	1	2	3
10539	10539	10542	-	12417	-	-	-	2	1	3
10542	10542	10544	+	10564	-	-	+	0	3	3
11115	11115	11116	+	11184	-	-	+	3	0	3
11218	11214	11222	-	12075	-	-	-	3	0	3
11238	11236	11238	-	11284	-	-	-	1	2	3
11245	-	-	-	11327	-	-	-	3	0	3
11409	11408	11409	+	13959	-	-	+	2	1	3
11416	-	-	-	13451	-	-	+	2	1	3
11425	11421	11427	+	11520	-	-	+	0	3	3
11539	11539	11541	-	13706	13706	13708	-	2	1	3
11616	11616	11617	-	13470	-	-	+	2	1	3
11633	11632	11633	-	14820	-	-	+	3	0	3
11680	11676	11682	+	13087	13087	13088	+	3	0	3
11688	11686	11690	+	13100	-	-	+	2	1	3
11690	-	-	-	13100	-	-	-	2	1	3
11700	11696	11703	-	14721	-	-	-	1	2	3
11840	11840	11841	-	11915	11915	11916	-	1	2	3
11965	11965	11968	+	14426	-	-	+	2	1	3
12073	12073	12076	+	13144	-	-	+	0	3	3
12095	12093	12095	+	13852	13852	13853	-	1	2	3
12113	12109	12114	-	12340	-	-	-	0	3	3
12127	-	-	+	12194	-	-	+	3	0	3
12149	12147	12149	-	13582	-	-	+	1	2	3
12344	12341	12345	+	12312	12308	12312	-	0	3	3
12346	12346	12350	-	12546	-	-	-	3	0	3
12542	-	-	-	12603	-	-	+	2	1	3
12582	12582	12586	-	13334	13334	13336	-	2	1	3
12582	12582	12586	-	13406	-	-	-	2	1	3
12589	12589	12593	-	13260	-	-	-	2	1	3
12589	12589	12593	-	13262	-	-	+	0	3	3
12606	12603	12607	-	14633	-	-	-	2	1	3
12618	12616	12619	-	12687	12683	12687	-	0	3	3
12625	12625	12629	-	13663	-	-	+	1	2	3
12780	12778	12780	+	14610	14608	14610	+	2	1	3
12784	12782	12784	-	13076	13073	13076	-	0	3	3
12837	12837	12840	-	14610	14610	14612	-	2	1	3
12880	12879	12881	-	12948	-	-	-	0	3	3
12880	12879	12881	-	13220	-	-	-	0	3	3
12924	12924	12925	-	14098	14097	14098	-	1	2	3
12947	12944	12948	+	14111	-	-	+	0	3	3
13056	13052	13056	-	13263	-	-	-	1	2	3
13146	13146	13150	-	13330	13326	13330	-	3	0	3
13211	13208	13211	-	14036	-	-	-	3	0	3
13390	-	-	-	14385	-	-	+	2	1	3
13407	-	-	-	14903	-	-	+	2	1	3
13476	13476	13480	-	13591	13590	13591	-	1	2	3
13737	13737	13740	+	14534	14534	14537	+	1	2	3
13794	13793	13798	+	13953	13952	13953	+	1	2	3
13891	13891	13894	-	13915	13912	13915	+	1	2	3
13953	13952	13957	-	14505	14505	14506	-	3	0	3
14213	14213	14214	-	14261	-	-	-	2	1	3
14213	14213	14214	-	14773	-	-	+	1	2	3
14221	-	-	-	14786	-	-	-	2	1	3
14346	14346	14348	-	14467	-	-	-	0	3	3
14435	14435	14436	-	14507	-	-	-	3	0	3
14542	14540	14542	-	14644	14640	14644	-	1	2	3
14565	14564	14566	-	14841	14841	14842	+	1	2	3
14580	-	-	-	14859	-	-	-	0	3	3
14586	14582	14586	+	15043	-	-	+	0	3	3
14688	-	-	-	14912	-	-	-	1	2	3

14832	14832	14834	-	15120	15120	15122	-	2	1	3
15245	15241	15245	-	15271	15271	15275	+	1	2	3
15251	-	-	+	15269	-	-	+	0	3	3
58	-	-	-	2997	-	-	-	0	2	2
74	70	79	-	224	-	-	-	1	1	2
74	70	79	-	1179	-	-	-	2	0	2
74	70	79	-	1839	-	-	-	1	1	2
74	70	79	-	1873	-	-	-	1	1	2
74	70	79	-	14240	14240	14241	+	1	1	2
88	87	92	+	1577	-	-	+	1	1	2
88	87	92	+	1673	-	-	+	2	0	2
88	87	92	+	1790	-	-	+	1	1	2
97	94	102	+	213	-	-	-	1	1	2
97	94	102	+	426	-	-	+	2	0	2
97	94	102	+	1590	-	-	+	1	1	2
97	94	102	+	3354	-	-	+	2	0	2
107	103	110	+	143	-	-	+	2	0	2
107	103	110	+	180	180	183	+	2	0	2
107	103	110	+	217	-	-	+	1	1	2
107	103	110	+	449	-	-	+	2	0	2
107	103	110	+	523	-	-	+	1	1	2
107	103	110	+	4044	-	-	+	1	1	2
109	105	114	-	360	-	-	-	0	2	2
109	105	114	-	379	379	380	-	1	1	2
109	105	114	-	1431	-	-	-	2	0	2
109	105	114	-	1673	-	-	-	2	0	2
116	115	117	-	1570	-	-	-	1	1	2
116	111	117	+	159	156	159	-	1	1	2
116	111	117	+	1024	-	-	+	1	1	2
116	111	117	+	1477	-	-	+	1	1	2
123	118	126	-	337	337	340	-	1	1	2
123	118	126	-	456	-	-	+	1	1	2
123	118	126	-	462	-	-	-	1	1	2
123	118	126	+	137	133	137	+	1	1	2
123	118	126	+	153	-	-	+	2	0	2
123	118	126	+	203	-	-	+	2	0	2
123	118	126	+	291	291	292	+	1	1	2
123	118	126	+	367	-	-	+	1	1	2
123	118	126	+	416	414	416	+	2	0	2
123	118	126	+	890	-	-	+	1	1	2
123	118	126	+	972	-	-	+	1	1	2
123	118	126	+	1144	-	-	+	2	0	2
123	118	126	+	1686	-	-	+	2	0	2
123	118	126	+	1802	-	-	+	1	1	2
123	118	126	+	1807	-	-	+	1	1	2
130	127	134	-	279	-	-	-	1	1	2
130	127	134	-	367	-	-	-	1	1	2
130	127	134	-	448	448	450	-	1	1	2
130	127	134	-	915	915	916	-	1	1	2
130	127	134	-	1388	-	-	-	1	1	2
131	128	133	+	160	-	-	+	1	1	2
131	128	133	+	176	176	177	+	1	1	2
131	128	133	+	301	-	-	+	1	1	2
131	128	133	+	366	366	367	+	1	1	2
131	128	133	+	389	-	-	+	2	0	2
131	128	133	+	576	-	-	+	1	1	2
131	128	133	+	672	-	-	+	1	1	2
131	128	133	+	884	-	-	+	2	0	2
131	128	133	+	1291	-	-	+	1	1	2
131	128	133	+	1312	-	-	+	1	1	2
136	135	139	+	177	177	178	+	2	0	2
136	135	139	+	1830	-	-	+	1	1	2
138	136	138	-	265	-	-	-	1	1	2
138	136	138	-	580	-	-	-	0	2	2
142	141	144	+	178	-	-	+	1	1	2
143	141	144	-	296	-	-	-	1	1	2
150	148	154	-	265	-	-	-	1	1	2
150	148	154	-	301	-	-	-	1	1	2
150	148	154	-	1199	-	-	-	1	1	2
150	148	154	-	1439	-	-	-	1	1	2
150	148	154	-	1873	-	-	-	1	1	2
150	148	154	-	13439	-	-	+	1	1	2
150	148	153	+	186	182	186	+	1	1	2
150	148	153	+	192	192	193	+	0	2	2
150	148	153	+	321	-	-	+	0	2	2
150	148	153	+	488	-	-	+	1	1	2
150	148	153	+	1524	-	-	+	2	0	2
157	154	162	+	324	-	-	+	2	0	2
165	165	170	+	529	529	530	+	1	1	2
173	171	176	+	195	192	195	+	0	2	2
175	171	179	-	361	361	362	-	1	1	2
175	171	179	-	370	370	373	-	2	0	2
178	177	182	+	192	-	-	+	0	2	2
178	177	182	+	201	-	-	+	0	2	2

178	177	182	+	335 -	-		+	1	1	2
178	177	182	+	551	551	552	+	1	1	2
186	183	189	-	306 -	-		-	1	1	2
186	183	189	-	452 -	-		-	0	2	2
186	183	189	-	587 -	-		-	1	1	2
186	184	187	+	516 -	-		+	1	1	2
191	189	194	+	373	373	374	+	0	2	2
191	189	194	+	527 -	-		+	2	0	2
191	189	194	+	999 -	-		+	1	1	2
191	189	194	+	1498 -	-		+	1	1	2
196	191	201	-	265 -	-		-	1	1	2
196	191	201	-	565	565	568	-	1	1	2
196	191	201	-	916 -	-		-	0	2	2
196	191	201	-	1877	1877	1878	-	1	1	2
223	221	228	+	270 -	-		+	0	2	2
229	224	231	-	260	257	260	+	2	0	2
229	224	231	-	384 -	-		-	1	1	2
235	235	239	-	896 -	-		-	1	1	2
237	235	238	+	250	250	251	-	1	1	2
245	242	248	-	407 -	-		-	1	1	2
245	242	248	-	450 -	-		-	0	2	2
245	242	248	-	617 -	-		-	2	0	2
245	242	248	-	681 -	-		-	1	1	2
245	242	248	-	897 -	-		-	1	1	2
245	242	248	-	1079 -	-		-	1	1	2
245	242	248	-	1778 -	-		-	1	1	2
251	251	255	+	251 -	-		+	0	2	2
255	251	255	-	1883 -	-		-	1	1	2
262	260	262	-	1092 -	-		-	1	1	2
265	262	270	+	370 -	-		+	0	2	2
265	262	270	+	410	410	412	+	1	1	2
265	262	270	+	426	426	428	+	1	1	2
267	263	267	-	442 -	-		-	1	1	2
267	263	267	-	450 -	-		-	1	1	2
267	263	267	-	567	567	570	-	2	0	2
267	263	267	-	686 -	-		+	0	2	2
267	263	267	-	871 -	-		-	1	1	2
267	263	267	-	1379 -	-		-	1	1	2
267	263	267	-	15122 -	-		+	1	1	2
278	274	278	+	542 -	-		+	2	0	2
280 -	-		-	426 -	-		-	2	0	2
290	288	293	+	316 -	-		+	1	1	2
290	288	293	+	328	325	328	+	1	1	2
290	288	293	+	425	425	426	+	0	2	2
290	288	293	+	431	429	431	+	2	0	2
290	288	293	+	444 -	-		+	1	1	2
290	288	293	+	1080 -	-		+	1	1	2
290	288	293	+	1224 -	-		+	0	2	2
290	288	293	+	1794 -	-		+	1	1	2
290	288	293	+	1839	1839	1840	+	1	1	2
291	289	293	-	502 -	-		-	2	0	2
291	289	293	-	603 -	-		-	1	1	2
295	294	296	+	589 -	-		+	1	1	2
296	295	298	-	620 -	-		-	0	2	2
300	297	303	+	309 -	-		+	2	0	2
300	297	303	+	329 -	-		+	0	2	2
300	297	303	+	425 -	-		+	2	0	2
300	297	303	+	629	629	630	+	1	1	2
304	299	304	-	436 -	-		-	1	1	2
304	299	304	-	582 -	-		-	1	1	2
308	305	314	+	396 -	-		+	1	1	2
308	305	314	+	528	528	530	+	0	2	2
308	305	314	+	606	602	606	+	2	0	2
312	307	316	-	513 -	-		-	1	1	2
312	307	316	-	556	552	556	-	2	0	2
312	307	316	-	575 -	-		-	1	1	2
312	307	316	-	733 -	-		-	0	2	2
312	307	316	-	1007 -	-		-	1	1	2
312	307	316	-	1089 -	-		-	1	1	2
312	307	316	-	1388 -	-		-	0	2	2
312	307	316	-	1887 -	-		+	1	1	2
316	315	321	+	367 -	-		+	1	1	2
316	315	321	+	424 -	-		+	1	1	2
320	318	323	-	498 -	-		-	1	1	2
320	318	323	-	1048 -	-		-	1	1	2
320	318	323	-	1148	1148	1150	-	0	2	2
320	318	323	-	1873 -	-		-	0	2	2
329	328	333	-	465 -	-		-	2	0	2
329	328	333	-	504 -	-		-	1	1	2
329	328	333	-	531	531	532	-	1	1	2
329	328	333	-	576 -	-		-	0	2	2
332	327	332	+	373 -	-		+	1	1	2
332	327	332	+	832	832	833	+	1	1	2
338	336	339	-	407 -	-		-	1	1	2

338	336	339	-	479	479	480	-	1	1	2
338	336	339	-	1793	-	-	-	1	1	2
338	335	338	+	544	-	-	+	1	1	2
345	344	345	+	516	-	-	+	0	2	2
359	355	361	+	458	-	-	+	2	0	2
359	355	361	+	6685	-	-	-	2	0	2
365	361	365	-	526	-	-	-	1	1	2
365	361	365	-	544	-	-	-	1	1	2
365	361	365	-	586	-	-	-	1	1	2
368	363	373	+	413	409	413	+	2	0	2
368	363	373	+	539	-	-	+	0	2	2
368	363	373	+	593	-	-	+	0	2	2
368	363	373	+	1808	-	-	+	1	1	2
372	366	376	-	533	-	-	-	1	1	2
372	366	376	-	552	-	-	-	2	0	2
372	366	376	-	558	-	-	-	2	0	2
372	366	376	-	625	-	-	-	1	1	2
372	366	376	-	653	-	-	-	1	1	2
372	366	376	-	998	-	-	-	1	1	2
372	366	376	-	1885	-	-	-	0	2	2
375	374	378	+	490	490	491	+	1	1	2
375	374	378	+	1803	-	-	+	1	1	2
375	374	378	+	1809	-	-	+	0	2	2
378	377	378	-	560	-	-	-	0	2	2
378	377	378	-	1797	-	-	-	1	1	2
385	380	385	+	428	424	428	+	2	0	2
385	380	385	+	579	-	-	+	1	1	2
388	-	-	-	521	-	-	-	1	1	2
390	387	395	+	404	-	-	+	1	1	2
390	387	395	+	415	-	-	+	2	0	2
390	387	395	+	1194	-	-	+	1	1	2
390	387	395	+	6475	-	-	+	1	1	2
404	399	407	+	1582	-	-	+	1	1	2
405	404	406	-	519	519	520	-	1	1	2
410	408	411	+	466	-	-	+	1	1	2
411	408	416	-	567	-	-	-	1	1	2
411	408	416	-	760	760	762	-	0	2	2
411	408	416	-	825	825	827	-	0	2	2
411	408	416	-	970	-	-	-	0	2	2
411	408	416	-	1030	-	-	-	0	2	2
416	413	422	+	512	-	-	+	2	0	2
416	413	422	+	655	-	-	+	2	0	2
416	413	422	+	1636	1636	1637	+	1	1	2
421	419	423	-	682	-	-	-	1	1	2
426	423	431	+	490	490	491	+	2	0	2
426	423	431	+	1611	-	-	+	2	0	2
439	436	440	+	501	-	-	+	1	1	2
442	440	442	-	969	-	-	-	1	1	2
444	444	445	+	459	459	460	+	1	1	2
449	446	453	-	679	679	680	-	0	2	2
449	446	453	-	899	-	-	-	1	1	2
457	455	459	-	599	-	-	-	1	1	2
457	455	459	-	625	622	625	-	0	2	2
458	454	458	+	512	510	512	+	1	1	2
465	463	471	+	659	-	-	+	1	1	2
466	462	471	-	648	648	651	-	1	1	2
466	462	471	-	884	-	-	-	1	1	2
474	474	477	+	491	-	-	+	0	2	2
474	474	477	+	509	-	-	+	1	1	2
481	477	485	-	584	-	-	-	1	1	2
481	477	485	-	688	688	690	-	1	1	2
481	477	485	-	761	-	-	-	1	1	2
481	477	485	-	923	923	924	-	1	1	2
481	477	485	-	943	-	-	-	0	2	2
484	480	484	+	530	-	-	+	0	2	2
484	480	484	+	602	-	-	+	1	1	2
490	490	492	-	580	-	-	-	1	1	2
490	490	492	-	662	660	662	-	1	1	2
490	490	492	-	683	-	-	-	2	0	2
491	488	494	+	494	-	-	+	0	2	2
491	488	494	+	526	-	-	+	2	0	2
491	488	494	+	594	-	-	+	0	2	2
491	488	494	+	607	-	-	+	1	1	2
491	488	494	+	1803	-	-	+	1	1	2
501	501	505	+	558	-	-	+	1	1	2
502	498	503	-	634	-	-	-	1	1	2
502	498	503	-	889	-	-	-	1	1	2
502	498	503	-	1694	-	-	-	1	1	2
502	498	503	-	1729	-	-	-	1	1	2
510	508	511	+	555	555	559	+	1	1	2
510	508	511	+	580	-	-	+	1	1	2
510	508	511	+	594	-	-	+	1	1	2
511	506	515	-	830	-	-	-	1	1	2
511	506	515	-	895	895	897	-	1	1	2

511	506	515	-	1035	1035	1036	-	1	1	2
518	516	521	+	533	-	-	+	0	2	2
522	518	526	-	618	-	-	-	1	1	2
522	518	526	-	815	-	-	-	1	1	2
522	518	526	-	1092	-	-	-	2	0	2
522	518	526	-	1188	-	-	-	2	0	2
530	528	535	-	672	672	673	-	1	1	2
530	528	535	-	725	725	726	-	1	1	2
530	528	535	-	2761	-	-	+	2	0	2
530	528	537	+	1685	1683	1685	+	2	0	2
541	539	545	-	737	-	-	-	1	1	2
541	539	545	-	1048	-	-	-	1	1	2
545	541	548	+	617	-	-	+	0	2	2
545	541	548	+	13773	-	-	+	1	1	2
556	553	560	-	692	692	693	-	0	2	2
556	553	560	-	1134	-	-	-	1	1	2
558	-	-	+	583	581	583	+	0	2	2
569	567	574	-	1027	-	-	-	1	1	2
569	566	570	+	896	-	-	+	1	1	2
577	576	577	-	1019	-	-	-	0	2	2
577	576	577	-	1584	-	-	-	1	1	2
580	575	585	+	1650	-	-	+	1	1	2
580	575	585	+	1793	1793	1794	+	1	1	2
585	581	590	-	797	-	-	-	2	0	2
585	581	590	-	882	882	884	-	1	1	2
585	581	590	-	896	-	-	-	1	1	2
585	581	590	-	917	-	-	-	0	2	2
585	581	590	-	1386	1386	1387	-	1	1	2
585	581	590	-	1676	1676	1677	-	1	1	2
589	588	593	+	896	896	897	+	1	1	2
596	592	602	-	973	970	973	-	1	1	2
596	592	602	-	1226	1226	1227	-	2	0	2
596	592	602	-	1722	1722	1723	-	1	1	2
596	592	602	-	3171	-	-	+	1	1	2
596	592	602	-	15149	-	-	-	1	1	2
607	605	610	+	608	605	608	+	2	0	2
615	615	618	+	912	909	912	+	1	1	2
618	615	621	-	944	-	-	-	1	1	2
618	615	621	-	955	-	-	-	1	1	2
618	615	621	-	999	-	-	-	1	1	2
627	623	629	-	775	775	776	-	1	1	2
627	623	629	-	829	-	-	-	1	1	2
627	623	629	-	835	-	-	-	1	1	2
627	623	629	-	944	-	-	-	1	1	2
633	633	635	+	8905	8905	8906	-	1	1	2
634	634	638	-	673	-	-	-	1	1	2
634	634	638	-	11876	-	-	+	1	1	2
649	-	-	-	929	-	-	+	0	2	2
654	652	658	+	844	-	-	+	0	2	2
655	653	659	-	772	770	772	-	1	1	2
655	653	659	-	802	-	-	-	1	1	2
665	663	669	-	894	-	-	-	1	1	2
673	673	678	-	896	894	896	-	0	2	2
673	673	678	-	11844	-	-	+	0	2	2
684	680	688	-	896	-	-	-	2	0	2
684	680	688	-	944	-	-	-	1	1	2
684	682	687	+	904	-	-	+	2	0	2
684	682	687	+	1144	1144	1145	+	1	1	2
684	682	687	+	3643	3643	3644	+	1	1	2
691	691	692	-	826	-	-	-	1	1	2
692	688	695	+	818	-	-	+	1	1	2
692	688	695	+	838	-	-	+	1	1	2
692	688	695	+	926	924	926	+	1	1	2
692	688	695	+	1576	1576	1580	+	2	0	2
723	720	727	+	802	-	-	+	1	1	2
727	727	730	-	10068	-	-	+	0	2	2
735	732	739	-	1029	1026	1029	-	1	1	2
740	-	-	+	1099	-	-	+	1	1	2
745	742	745	-	924	-	-	-	1	1	2
750	747	750	-	708	708	709	+	1	1	2
760	756	762	-	1028	-	-	-	1	1	2
770	770	772	+	802	802	803	+	2	0	2
789	785	791	+	1213	-	-	+	0	2	2
799	795	803	-	1599	-	-	-	0	2	2
802	798	805	+	827	825	827	+	1	1	2
817	813	820	+	856	-	-	+	1	1	2
818	814	820	-	921	917	921	-	1	1	2
818	814	820	-	1011	1008	1011	-	1	1	2
827	823	832	+	1023	1023	1024	+	1	1	2
832	827	835	-	997	-	-	-	1	1	2
832	827	835	-	1087	-	-	-	0	2	2
832	827	835	-	1180	-	-	-	0	2	2
840	839	844	-	995	992	995	-	1	1	2
840	839	844	-	1873	-	-	-	1	1	2

849	849	853	-	1069	-	-	-	1	1	2
849	849	853	-	2999	-	-	-	0	2	2
860	857	864	-	1005	1001	1005	-	1	1	2
860	857	864	-	1108	-	-	-	1	1	2
860	857	864	-	1144	-	-	-	2	0	2
868	867	868	+	1231	-	-	+	1	1	2
883	881	886	+	1112	-	-	+	1	1	2
884	882	886	-	1678	-	-	-	0	2	2
892	891	892	-	1106	-	-	-	2	0	2
893	893	897	+	981	981	982	+	1	1	2
898	894	899	-	1048	1048	1049	-	1	1	2
898	894	899	-	1108	-	-	-	0	2	2
898	894	899	-	1138	-	-	-	0	2	2
898	894	899	-	1386	1386	1387	-	1	1	2
898	894	899	-	1677	-	-	-	1	1	2
912	-	-	-	1233	-	-	-	1	1	2
916	912	920	+	924	-	-	+	1	1	2
916	912	920	+	2940	-	-	+	0	2	2
917	916	918	-	1029	-	-	-	1	1	2
923	923	924	-	1193	-	-	-	1	1	2
923	923	924	-	1199	-	-	-	2	0	2
924	921	928	+	957	-	-	+	1	1	2
924	921	928	+	981	-	-	+	2	0	2
924	921	928	+	991	989	991	+	0	2	2
924	921	928	+	12565	-	-	-	2	0	2
928	925	929	-	1108	-	-	-	1	1	2
928	925	929	-	1178	-	-	-	2	0	2
928	925	929	-	1196	-	-	-	1	1	2
952	948	952	-	1104	-	-	-	0	2	2
958	958	960	+	1005	-	-	+	1	1	2
959	955	961	-	3052	-	-	-	0	2	2
964	-	-	-	1103	-	-	-	0	2	2
970	968	973	-	1092	-	-	-	1	1	2
970	968	973	-	1111	-	-	-	1	1	2
970	968	973	-	1348	1348	1351	-	1	1	2
972	968	972	+	1247	-	-	+	0	2	2
977	975	980	-	1090	-	-	-	1	1	2
977	975	980	-	1107	1107	1108	-	1	1	2
977	977	979	+	988	-	-	+	1	1	2
982	981	982	+	1205	-	-	+	1	1	2
989	987	991	-	12966	-	-	-	2	0	2
999	999	1004	+	1073	1073	1074	+	0	2	2
999	999	1004	+	1089	-	-	+	1	1	2
1003	1002	1003	-	1224	-	-	-	1	1	2
1003	1002	1003	-	1475	-	-	-	0	2	2
1020	1018	1025	+	1822	-	-	+	0	2	2
1029	1026	1031	-	1199	-	-	-	1	1	2
1029	1026	1031	-	1348	-	-	-	1	1	2
1029	1027	1031	+	1140	1140	1141	+	1	1	2
1035	1035	1038	+	1045	-	-	+	2	0	2
1035	1035	1038	+	1350	1348	1350	+	0	2	2
1046	1042	1050	+	1352	-	-	+	1	1	2
1051	1048	1054	-	1176	-	-	-	1	1	2
1051	1048	1054	-	1200	-	-	-	1	1	2
1051	1048	1054	-	1210	-	-	-	2	0	2
1051	1048	1054	-	1434	1431	1434	-	2	0	2
1073	1070	1076	+	1349	-	-	-	0	2	2
1073	1070	1076	+	1372	-	-	+	1	1	2
1076	1076	1079	-	1271	-	-	-	2	0	2
1086	1082	1090	-	1175	-	-	-	1	1	2
1086	1082	1090	-	1337	1337	1339	-	0	2	2
1089	1085	1092	+	1327	1327	1328	+	2	0	2
1099	1099	1100	+	1130	1130	1131	+	1	1	2
1109	1107	1110	-	1193	1193	1194	-	1	1	2
1109	1107	1110	-	1223	1223	1224	-	2	0	2
1109	1107	1110	-	1395	-	-	-	1	1	2
1112	1107	1120	+	1245	-	-	+	1	1	2
1112	1107	1120	+	13363	-	-	+	2	0	2
1130	1129	1134	-	1476	-	-	-	1	1	2
1130	1129	1134	-	14516	-	-	+	2	0	2
1133	1130	1138	+	1152	-	-	+	1	1	2
1139	1135	1143	-	1296	1296	1297	-	1	1	2
1139	1135	1143	-	1868	-	-	-	1	1	2
1148	1147	1152	-	1203	-	-	-	1	1	2
1148	1147	1152	-	1795	-	-	-	1	1	2
1148	1147	1152	-	1897	-	-	-	1	1	2
1162	-	-	-	1277	-	-	-	1	1	2
1170	1170	1173	+	1199	-	-	+	2	0	2
1170	1170	1173	+	1230	1226	1230	+	0	2	2
1181	1178	1184	+	1274	1274	1275	+	1	1	2
1181	1178	1184	+	1298	1298	1299	-	1	1	2
1181	1178	1184	+	1349	-	-	+	1	1	2
1186	1186	1187	+	1286	1286	1287	+	1	1	2
1187	1187	1188	-	1336	-	-	-	1	1	2

1192	1191	1194	-	1272 -	-		-	1	1	2
1192	1191	1194	-	1407 -	-		-	2	0	2
1193	1188	1196	+	1346 -	-		+	1	1	2
1193	1188	1196	+	1495 -	-		+	0	2	2
1199	1197	1204	+	1222 -	-		+	0	2	2
1199	1197	1204	+	1236 -	-		+	0	2	2
1201	1200	1202	-	1316 -	-		-	1	1	2
1201	1200	1202	-	1544 -	-		-	1	1	2
1210	1206	1212	+	1278	1275	1278	+	1	1	2
1213	1209	1218	-	1687 -	-		-	1	1	2
1213	1209	1218	-	6361	6361	6362	-	1	1	2
1221 -	-		-	1434	1432	1434	-	1	1	2
1226	1222	1232	-	1453 -	-		-	1	1	2
1226	1222	1232	-	1492 -	-		-	1	1	2
1234	1234	1238	-	1434 -	-		-	0	2	2
1234	1234	1238	-	1600 -	-		-	0	2	2
1244	1243	1248	-	1379	1379	1380	-	1	1	2
1254	1254	1256	-	1469 -	-		-	2	0	2
1259	1258	1260	-	1415 -	-		-	2	0	2
1275	1273	1280	-	1682	1678	1682	-	0	2	2
1284	1284	1286	+	1327 -	-		+	2	0	2
1285	1285	1288	-	1582	1579	1582	-	1	1	2
1293	1293	1297	-	1432 -	-		-	1	1	2
1311	1309	1312	-	1415 -	-		-	1	1	2
1311	1309	1312	-	1643 -	-		-	1	1	2
1318	1314	1318	-	1505 -	-		-	2	0	2
1318	1314	1318	-	1599 -	-		-	2	0	2
1323 -	-		-	1550 -	-		-	2	0	2
1327	1325	1327	+	1364 -	-		+	2	0	2
1328	1326	1330	-	1434 -	-		-	1	1	2
1328	1326	1330	-	1517 -	-		-	1	1	2
1328	1326	1330	-	1600 -	-		-	0	2	2
1335	1335	1340	-	1549 -	-		-	1	1	2
1335	1335	1340	-	1563	1561	1563	-	0	2	2
1348	1348	1350	-	1599 -	-		-	1	1	2
1348	1348	1350	-	1718 -	-		-	2	0	2
1348	1348	1350	-	8909 -	-		+	0	2	2
1357 -	-		+	1382 -	-		+	2	0	2
1359	1359	1365	-	1563 -	-		-	0	2	2
1377	1376	1377	+	1463	1463	1464	+	1	1	2
1388	1385	1391	-	1458 -	-		-	2	0	2
1388	1385	1391	-	1802 -	-		-	1	1	2
1390	1386	1390	+	1549 -	-		+	1	1	2
1395	1394	1395	+	1562 -	-		+	1	1	2
1396	1393	1398	-	1499 -	-		-	1	1	2
1396	1393	1398	-	1602	1598	1602	-	1	1	2
1400	1397	1401	+	1538 -	-		-	2	0	2
1400	1397	1401	+	1594 -	-		+	1	1	2
1407	1407	1410	-	1739 -	-		-	0	2	2
1408	1407	1411	+	1428 -	-		+	1	1	2
1408	1407	1411	+	1528 -	-		+	2	0	2
1421	1417	1423	+	1690 -	-		+	2	0	2
1432	1432	1433	-	1545 -	-		-	1	1	2
1434	1431	1436	+	1444	1440	1444	+	1	1	2
1434	1431	1436	+	1476 -	-		+	0	2	2
1434	1431	1436	+	1485	1481	1485	+	1	1	2
1437	1434	1437	-	1505 -	-		-	1	1	2
1437	1434	1437	-	1513 -	-		-	1	1	2
1437	1434	1437	-	1624 -	-		-	1	1	2
1444	1442	1448	-	6233	6229	6233	-	1	1	2
1460	1457	1463	+	4410 -	-		-	2	0	2
1466	1462	1469	-	1718 -	-		-	0	2	2
1466	1466	1469	+	1508 -	-		+	1	1	2
1476	1474	1479	-	1551 -	-		-	1	1	2
1476	1474	1479	-	1878 -	-		-	1	1	2
1476	1474	1479	-	6305 -	-		-	1	1	2
1488	1485	1491	+	1490	1487	1490	+	2	0	2
1488	1485	1491	+	1532 -	-		+	0	2	2
1488	1485	1491	+	1594	1594	1595	+	1	1	2
1488	1485	1491	+	1623 -	-		+	1	1	2
1498	1497	1498	+	1611 -	-		+	1	1	2
1513	1509	1515	-	1585 -	-		-	1	1	2
1513	1509	1515	-	1607 -	-		-	1	1	2
1522	1522	1523	-	1677 -	-		-	1	1	2
1550	1549	1550	-	1795	1793	1795	-	1	1	2
1553	1552	1554	+	1581	1577	1581	+	0	2	2
1553	1552	1554	+	1603	1603	1604	+	1	1	2
1555	1552	1558	-	1579 -	-		-	0	2	2
1555	1552	1558	-	1686 -	-		-	2	0	2
1555	1552	1558	-	8120 -	-		-	0	2	2
1562	1559	1564	+	1581 -	-		+	2	0	2
1563 -	-		-	1777 -	-		-	1	1	2
1568	1565	1571	-	1777 -	-		-	1	1	2
1568	1565	1571	-	8127 -	-		-	2	0	2

1568	1568	1572	+	1600	-	-	+	0	2	2
1580	1575	1584	+	1794	-	-	+	0	2	2
1580	1575	1584	+	1802	-	-	+	1	1	2
1592	1587	1596	+	1810	-	-	+	1	1	2
1595	1591	1596	-	1875	-	-	-	2	0	2
1595	1591	1596	-	1894	1891	1894	-	1	1	2
1600	1599	1603	-	1753	1753	1754	-	1	1	2
1600	1599	1603	-	1795	1793	1795	-	2	0	2
1602	-	-	+	1728	-	-	+	1	1	2
1615	1611	1615	-	3311	-	-	-	1	1	2
1625	1621	1629	-	1708	-	-	-	0	2	2
1625	1621	1629	-	4762	4762	4763	-	1	1	2
1632	1630	1637	+	1845	1842	1845	+	1	1	2
1641	1641	1644	+	1811	-	-	+	1	1	2
1658	1655	1658	-	1785	-	-	-	2	0	2
1665	1665	1668	+	1681	-	-	+	2	0	2
1673	1671	1673	+	1704	-	-	+	2	0	2
1678	1676	1680	-	1793	1793	1794	-	1	1	2
1678	1676	1680	-	1823	-	-	-	1	1	2
1678	1676	1680	-	1883	1881	1883	-	1	1	2
1678	1676	1680	-	1896	-	-	-	1	1	2
1683	1679	1685	+	1793	1793	1794	+	1	1	2
1712	-	-	+	1741	1737	1741	+	1	1	2
1726	1723	1730	+	1754	-	-	+	1	1	2
1735	1732	1738	-	1818	-	-	-	1	1	2
1740	-	-	-	1901	-	-	-	2	0	2
1746	1746	1747	-	1874	-	-	-	1	1	2
1750	1749	1754	+	1793	-	-	+	1	1	2
1758	-	-	-	1944	-	-	-	1	1	2
1765	1764	1765	+	1840	-	-	+	1	1	2
1771	1768	1772	+	1803	-	-	+	1	1	2
1771	1768	1772	+	1852	-	-	+	0	2	2
1771	1768	1772	+	14741	-	-	+	0	2	2
1778	1778	1779	+	1772	1772	1773	-	0	2	2
1786	-	-	-	1999	-	-	-	2	0	2
1791	1791	1794	-	6368	-	-	-	1	1	2
1802	1798	1804	-	6348	6348	6349	-	1	1	2
1808	1807	1808	-	6293	-	-	-	1	1	2
1808	1807	1808	-	6356	6356	6357	-	1	1	2
1816	-	-	+	1840	-	-	+	0	2	2
1840	1836	1840	-	8525	-	-	-	1	1	2
1840	1836	1840	-	15293	-	-	+	1	1	2
1917	-	-	+	1983	-	-	+	2	0	2
1933	1930	1934	+	1948	-	-	+	2	0	2
1933	1930	1934	+	2166	-	-	+	1	1	2
1942	1939	1946	+	2001	2001	2002	+	2	0	2
1942	1939	1946	+	2178	-	-	+	2	0	2
1942	1939	1946	+	2588	-	-	+	1	1	2
1942	1939	1946	+	2743	-	-	+	2	0	2
1942	1939	1946	+	3098	-	-	+	2	0	2
1942	1939	1946	+	4321	-	-	+	2	0	2
1942	1939	1946	+	5841	5841	5842	+	1	1	2
1943	1940	1945	-	2144	-	-	-	1	1	2
1943	1940	1945	-	2730	-	-	-	1	1	2
1948	1948	1951	+	2178	-	-	+	2	0	2
1948	1948	1951	+	3024	-	-	+	1	1	2
1948	1948	1951	+	4160	-	-	+	1	1	2
1954	1953	1957	+	2013	2013	2014	+	2	0	2
1954	1953	1957	+	2841	-	-	+	2	0	2
1964	1961	1968	+	2011	-	-	+	2	0	2
1964	1961	1968	+	4173	-	-	+	1	1	2
1964	1961	1968	+	4768	-	-	+	1	1	2
1964	1961	1968	+	5678	-	-	+	2	0	2
1973	1969	1974	-	6272	6272	6273	-	1	1	2
1982	1982	1985	-	2283	-	-	-	0	2	2
1985	1981	1986	+	2091	-	-	+	1	1	2
1985	1981	1986	+	2139	-	-	+	1	1	2
1985	1981	1986	+	2314	2314	2318	+	2	0	2
1985	1981	1986	+	2926	2926	2927	+	1	1	2
1985	1981	1986	+	3181	3181	3182	+	1	1	2
1991	1988	1995	-	2797	-	-	-	0	2	2
1991	1988	1995	-	2840	-	-	-	1	1	2
1991	1988	1993	+	2118	-	-	+	2	0	2
1991	1988	1993	+	2128	-	-	+	1	1	2
1991	1988	1993	+	2284	-	-	+	1	1	2
1991	1988	1993	+	2347	-	-	+	1	1	2
1991	1988	1993	+	2469	-	-	+	1	1	2
1991	1988	1993	+	3184	-	-	+	1	1	2
2000	1997	2001	+	3163	3159	3163	+	1	1	2
2000	1997	2001	+	13460	13460	13461	+	1	1	2
2001	2000	2005	-	2576	-	-	-	0	2	2
2001	2000	2005	-	6116	-	-	-	2	0	2
2001	2000	2005	-	12476	-	-	-	2	0	2
2006	2006	2010	+	12489	12487	12489	+	0	2	2

2010	2007	2011	-	2106	2103	2106	-	1	1	2
2015	2012	2018	+	2109	2109	2110	+	0	2	2
2015	2012	2018	+	2229 -	-		+	1	1	2
2018	2014	2020	-	2141	2141	2142	-	1	1	2
2018	2014	2020	-	2269	2269	2270	-	0	2	2
2018	2014	2020	-	2296 -	-		-	1	1	2
2018	2014	2020	-	3152 -	-		-	1	1	2
2023	2022	2026	-	2105	2105	2106	+	0	2	2
2023	2022	2026	-	12505 -	-		-	1	1	2
2039	2035	2042	-	2125	2125	2126	-	1	1	2
2039	2035	2042	-	2219 -	-		-	1	1	2
2039	2035	2042	-	2315 -	-		-	1	1	2
2055	2052	2055	-	12530 -	-		-	1	1	2
2055	2052	2056	+	12502 -	-		-	1	1	2
2066	2065	2069	+	2171 -	-		+	0	2	2
2085	2083	2089	-	2219 -	-		-	1	1	2
2085	2084	2087	+	2126 -	-		+	0	2	2
2099	2099	2103	-	2177 -	-		+	2	0	2
2106 -	-		+	4056 -	-		+	1	1	2
2109	2106	2112	-	2168 -	-		-	1	1	2
2109	2106	2112	-	2269 -	-		-	1	1	2
2109	2106	2112	-	2305	2305	2308	-	1	1	2
2109	2106	2112	-	2324 -	-		-	1	1	2
2109	2106	2112	-	2385	2385	2386	-	1	1	2
2109	2106	2112	-	2766 -	-		-	1	1	2
2115	2111	2118	+	2132 -	-		+	1	1	2
2115	2111	2118	+	2143 -	-		+	1	1	2
2117	2115	2120	-	2745 -	-		-	1	1	2
2120	2119	2121	+	2144	2141	2144	+	0	2	2
2131	2130	2132	+	2131 -	-		+	0	2	2
2131	2130	2132	+	2153 -	-		+	1	1	2
2132	2127	2132	-	2354 -	-		-	0	2	2
2137	2134	2140	-	2544	2544	2547	-	1	1	2
2144 -	-		+	2932 -	-		+	1	1	2
2154	2152	2158	-	2737 -	-		-	0	2	2
2165	2165	2169	+	2339 -	-		+	2	0	2
2176	2174	2178	+	2240 -	-		+	1	1	2
2184	2183	2188	+	2212 -	-		-	0	2	2
2209	2209	2212	+	2299 -	-		+	1	1	2
2218	2218	2220	+	2433 -	-		+	1	1	2
2218	2218	2220	+	2478 -	-		+	1	1	2
2219	2217	2221	-	2416 -	-		-	1	1	2
2219	2217	2221	-	2794 -	-		-	2	0	2
2230	2230	2231	-	11402	11402	11403	-	1	1	2
2235	2235	2238	+	3122 -	-		+	1	1	2
2245	2244	2245	-	2855 -	-		-	1	1	2
2245	2243	2245	+	2268 -	-		-	0	2	2
2268	2265	2268	+	2194	2194	2195	-	1	1	2
2268	2265	2268	+	2245 -	-		-	1	1	2
2278 -	-		+	2332 -	-		+	1	1	2
2278 -	-		+	13053 -	-		+	1	1	2
2283	2282	2284	-	2766 -	-		-	1	1	2
2283	2283	2286	+	2363 -	-		+	1	1	2
2283	2283	2286	+	2383 -	-		+	0	2	2
2296	2293	2299	+	2439 -	-		+	0	2	2
2303	2300	2303	-	2554 -	-		-	0	2	2
2303	2300	2303	-	9673 -	-		+	0	2	2
2303 -	-		+	2387	2384	2387	+	1	1	2
2309	2306	2313	-	2635	2635	2638	-	1	1	2
2309	2306	2313	-	3510 -	-		-	0	2	2
2310	2307	2310	+	2387	2385	2387	+	1	1	2
2325	2323	2325	-	2536 -	-		-	0	2	2
2329 -	-		+	13436 -	-		+	2	0	2
2339	2339	2343	+	2389	2389	2390	+	1	1	2
2364	2360	2364	+	2395 -	-		+	1	1	2
2364	2360	2364	+	2754	2752	2754	+	1	1	2
2381	2377	2385	-	2428 -	-		-	1	1	2
2381	2377	2385	-	3470 -	-		-	0	2	2
2381	2377	2385	-	10378 -	-		-	1	1	2
2400	2396	2402	-	2638	2638	2639	-	1	1	2
2400	2396	2402	-	2745 -	-		+	0	2	2
2415 -	-		+	2563 -	-		+	1	1	2
2416	2412	2419	-	2560 -	-		-	1	1	2
2416	2412	2419	-	2993	2993	2994	-	1	1	2
2427	2424	2427	-	2584 -	-		-	1	1	2
2427	2424	2427	-	2639 -	-		-	2	0	2
2434	2430	2436	-	2505 -	-		-	1	1	2
2434	2430	2436	-	2556 -	-		-	1	1	2
2434	2430	2436	-	2566 -	-		-	1	1	2
2434	2430	2436	-	2605	2605	2606	-	1	1	2
2434	2430	2436	-	2661 -	-		-	1	1	2
2434	2430	2436	-	2741 -	-		-	1	1	2
2439	2437	2443	-	2736 -	-		-	2	0	2
2445	2442	2449	+	2486 -	-		+	0	2	2

2468	2468	2469	+	13628	13628	13629	+	1	1	2
2472	2472	2474	-	2591	-	-	-	1	1	2
2472	2472	2474	-	2714	-	-	-	1	1	2
2473	2473	2477	+	2495	2495	2496	+	0	2	2
2490	2487	2491	-	2601	-	-	-	1	1	2
2490	2487	2491	-	3021	-	-	-	1	1	2
2523	-	-	-	2639	-	-	-	1	1	2
2528	-	-	-	2774	-	-	-	1	1	2
2535	2535	2537	+	2796	-	-	+	1	1	2
2535	2535	2537	+	14504	-	-	+	2	0	2
2551	2551	2555	-	2923	2923	2924	-	1	1	2
2551	2551	2555	-	4846	-	-	+	1	1	2
2564	2561	2564	+	2728	-	-	+	1	1	2
2578	2576	2578	-	2715	2713	2715	-	1	1	2
2583	2581	2586	-	2746	-	-	-	1	1	2
2591	2591	2592	-	2743	-	-	-	1	1	2
2594	2594	2598	+	2687	-	-	+	1	1	2
2607	2606	2607	+	2627	2627	2628	+	0	2	2
2620	2618	2620	-	2844	-	-	-	2	0	2
2628	2626	2629	+	2790	2787	2790	+	2	0	2
2640	2637	2642	-	2754	-	-	-	1	1	2
2640	2637	2642	-	2801	-	-	-	0	2	2
2640	2637	2642	-	2834	2834	2838	-	0	2	2
2640	2637	2642	-	2875	2871	2875	-	0	2	2
2640	2637	2643	+	2856	-	-	+	0	2	2
2651	-	-	+	4135	-	-	+	1	1	2
2653	2651	2653	-	2840	-	-	-	0	2	2
2667	2664	2667	-	3111	-	-	-	0	2	2
2690	2690	2694	-	2853	2853	2854	-	1	1	2
2718	2715	2722	-	2767	2765	2767	-	1	1	2
2718	2715	2722	-	2862	2862	2865	-	1	1	2
2718	2715	2722	-	3205	-	-	-	1	1	2
2730	2727	2733	-	5976	5974	5976	-	0	2	2
2731	-	-	+	2752	-	-	+	0	2	2
2736	2735	2736	+	14699	-	-	+	0	2	2
2740	2737	2745	-	2892	2890	2892	-	1	1	2
2745	2743	2748	+	2779	2779	2780	+	2	0	2
2752	2749	2753	-	3121	-	-	-	1	1	2
2757	2755	2757	-	2931	-	-	-	1	1	2
2757	2753	2760	+	2774	2772	2774	+	2	0	2
2757	2753	2760	+	2799	-	-	+	2	0	2
2769	2766	2769	+	2860	2856	2860	+	2	0	2
2769	2766	2769	+	5136	-	-	+	1	1	2
2775	2772	2775	-	8416	8414	8416	-	1	1	2
2786	2785	2786	-	2881	-	-	-	1	1	2
2808	2808	2809	+	2832	2832	2833	-	0	2	2
2825	2824	2825	-	2879	2879	2880	-	1	1	2
2835	2834	2839	-	2931	-	-	-	1	1	2
2835	2834	2839	-	2967	-	-	-	1	1	2
2835	2834	2839	-	3016	3016	3018	-	1	1	2
2835	2834	2839	-	3042	3042	3043	-	1	1	2
2835	2834	2839	-	3149	-	-	-	0	2	2
2843	2841	2845	-	3108	-	-	-	0	2	2
2843	2838	2843	+	6864	-	-	+	1	1	2
2849	2849	2854	+	2868	-	-	+	1	1	2
2849	2849	2854	+	3040	-	-	+	1	1	2
2854	2853	2856	-	3149	3149	3150	-	2	0	2
2867	2863	2871	-	3220	-	-	-	0	2	2
2877	2872	2881	-	3016	3016	3017	-	1	1	2
2882	2881	2882	+	2900	-	-	+	1	1	2
2892	2892	2893	+	2940	-	-	+	1	1	2
2898	2896	2898	-	3044	-	-	-	1	1	2
2910	2909	2911	+	3439	-	-	+	1	1	2
2927	2924	2930	-	3016	-	-	-	1	1	2
2927	2924	2930	-	3103	-	-	-	1	1	2
2927	2924	2930	-	3117	-	-	-	1	1	2
2931	2931	2935	+	3018	3018	3021	+	1	1	2
2931	2931	2935	+	3077	-	-	+	0	2	2
2932	2932	2935	-	3102	3102	3103	-	1	1	2
2932	2932	2935	-	8188	-	-	+	1	1	2
2941	2938	2945	-	3113	-	-	-	1	1	2
2941	2938	2945	-	3119	-	-	-	1	1	2
2941	2938	2945	-	3155	-	-	-	1	1	2
2951	2947	2951	-	2982	-	-	-	2	0	2
2956	-	-	-	2987	-	-	-	0	2	2
2960	2959	2963	+	2940	2940	2941	-	1	1	2
2968	2968	2970	-	3138	-	-	-	2	0	2
2968	-	-	+	3025	-	-	+	1	1	2
2975	2972	2976	+	3267	3267	3268	+	1	1	2
2975	2972	2976	+	3335	-	-	+	1	1	2
3010	-	-	-	3295	-	-	+	0	2	2
3010	3010	3011	+	2960	2960	2961	-	1	1	2
3016	-	-	-	3193	-	-	-	1	1	2
3021	3018	3023	-	3115	-	-	-	2	0	2

3021	3018	3023	-	3443	-	-	-	2	0	2
3062	-	-	-	3194	-	-	-	1	1	2
3065	3062	3068	+	3085	-	-	+	1	1	2
3077	-	-	+	3119	3119	3120	+	1	1	2
3078	3074	3078	-	3202	-	-	-	1	1	2
3098	-	-	+	3065	-	-	-	1	1	2
3113	3113	3118	-	4633	4633	4634	-	1	1	2
3125	3125	3129	-	6464	6464	6465	-	1	1	2
3150	3149	3150	-	6310	6308	6310	-	1	1	2
3155	3152	3156	-	3327	-	-	-	1	1	2
3155	3152	3156	-	3342	-	-	-	1	1	2
3185	-	-	-	6192	-	-	-	2	0	2
3199	3199	3201	+	3246	3244	3246	+	1	1	2
3207	3203	3210	-	3390	-	-	-	1	1	2
3207	3203	3210	-	11295	-	-	-	1	1	2
3215	3212	3217	-	5146	-	-	-	1	1	2
3221	3221	3222	+	3260	-	-	+	1	1	2
3227	3227	3229	-	5805	-	-	-	1	1	2
3234	3233	3238	-	3370	-	-	-	1	1	2
3234	3233	3238	-	3437	-	-	-	2	0	2
3234	3233	3238	-	4390	4390	4391	-	1	1	2
3247	3242	3250	-	3312	-	-	-	1	1	2
3247	3242	3250	-	3330	-	-	-	1	1	2
3247	3242	3250	-	3390	-	-	-	1	1	2
3247	3242	3250	-	3717	3717	3718	-	1	1	2
3247	3242	3250	-	3765	-	-	-	1	1	2
3247	3242	3250	-	4604	-	-	+	1	1	2
3258	3255	3262	-	3389	-	-	-	1	1	2
3258	3255	3262	-	3493	-	-	-	1	1	2
3258	3255	3262	-	3765	-	-	-	0	2	2
3258	3255	3262	-	3846	-	-	-	1	1	2
3258	3255	3262	-	3859	3859	3861	-	1	1	2
3258	3255	3262	-	4290	-	-	-	1	1	2
3258	3255	3262	-	4633	-	-	-	1	1	2
3259	3257	3263	+	4340	-	-	+	1	1	2
3259	3257	3263	+	4398	-	-	+	2	0	2
3267	3265	3269	+	5297	5297	5298	+	1	1	2
3267	3265	3269	+	5638	-	-	+	2	0	2
3273	3269	3275	-	3438	-	-	-	1	1	2
3273	3269	3275	-	4399	-	-	-	1	1	2
3273	3269	3275	-	6034	-	-	-	1	1	2
3274	3273	3277	+	3558	-	-	+	1	1	2
3274	3273	3277	+	6049	6046	6049	+	1	1	2
3280	3279	3281	+	3316	-	-	+	2	0	2
3290	3287	3292	-	3486	3486	3487	-	1	1	2
3313	3309	3313	-	3469	3466	3469	-	1	1	2
3313	3309	3318	+	3428	-	-	+	1	1	2
3313	3309	3318	+	4125	-	-	+	1	1	2
3313	3309	3318	+	5657	5657	5658	+	1	1	2
3322	3319	3325	+	3429	3429	3430	+	1	1	2
3322	3319	3325	+	3589	-	-	-	2	0	2
3322	3319	3325	+	5450	-	-	+	1	1	2
3329	3325	3329	-	3767	3764	3767	-	2	0	2
3329	3325	3329	-	4744	-	-	-	2	0	2
3335	3331	3337	-	3452	-	-	-	1	1	2
3335	3331	3337	-	3486	3486	3487	-	1	1	2
3335	3331	3337	-	3588	-	-	-	2	0	2
3335	3331	3337	-	3617	-	-	-	1	1	2
3335	3331	3337	-	4361	4357	4361	-	1	1	2
3335	3331	3337	-	4392	-	-	-	1	1	2
3335	3331	3337	-	4463	4463	4465	-	1	1	2
3337	3334	3340	+	3324	-	-	+	1	1	2
3340	3338	3340	-	3507	-	-	-	1	1	2
3340	3338	3340	-	3555	3555	3559	-	2	0	2
3342	-	-	+	3603	-	-	+	1	1	2
3346	3341	3350	-	3521	-	-	-	1	1	2
3346	3341	3350	-	12627	-	-	+	2	0	2
3355	-	-	+	4017	-	-	+	1	1	2
3373	3373	3375	+	6806	-	-	+	1	1	2
3378	3377	3382	+	3392	-	-	+	2	0	2
3378	3377	3382	+	3441	-	-	+	1	1	2
3378	3377	3382	+	13144	13142	13144	-	1	1	2
3389	3386	3393	-	3704	-	-	-	2	0	2
3393	3389	3393	+	3407	-	-	+	0	2	2
3393	3389	3393	+	3429	-	-	+	2	0	2
3393	3389	3393	+	3557	-	-	+	2	0	2
3406	3402	3406	+	3677	-	-	+	0	2	2
3414	3414	3416	-	3443	3441	3443	+	0	2	2
3421	3418	3421	-	3576	-	-	-	1	1	2
3422	3421	3422	+	3378	-	-	-	1	1	2
3429	3426	3429	-	3727	-	-	-	2	0	2
3429	3426	3429	-	3846	-	-	-	0	2	2
3429	3426	3430	+	3449	3445	3449	+	2	0	2
3429	3426	3430	+	3492	-	-	+	1	1	2

3429	3426	3430	+	3592	3592	3593	+	1	1	2
3429	3426	3430	+	4240	-	-	+	0	2	2
3429	3426	3430	+	5693	-	-	+	1	1	2
3434	3433	3438	+	3752	3752	3753	+	1	1	2
3434	3433	3438	+	4070	-	-	+	1	1	2
3436	3432	3439	-	3640	-	-	-	1	1	2
3436	3432	3439	-	3645	-	-	-	1	1	2
3436	3432	3439	-	3820	-	-	-	1	1	2
3436	3432	3439	-	4286	-	-	-	0	2	2
3441	3439	3441	+	3460	3460	3461	+	1	1	2
3441	3439	3441	+	4327	-	-	+	1	1	2
3446	3444	3449	-	3627	-	-	-	1	1	2
3446	3444	3449	-	3638	-	-	-	1	1	2
3446	3444	3449	-	3785	3785	3786	-	1	1	2
3446	3444	3449	-	4037	-	-	-	1	1	2
3455	3452	3459	-	4290	-	-	-	2	0	2
3455	3451	3455	+	3496	-	-	+	0	2	2
3468	3467	3468	-	3767	-	-	-	2	0	2
3474	-	-	-	4020	-	-	-	1	1	2
3486	-	-	+	3804	-	-	+	1	1	2
3490	3488	3492	-	3762	-	-	-	0	2	2
3490	3488	3492	-	4434	-	-	-	2	0	2
3505	-	-	+	3798	-	-	-	0	2	2
3510	3506	3512	-	3749	3749	3750	-	1	1	2
3510	3506	3512	-	3765	-	-	-	0	2	2
3536	3536	3538	+	3521	-	-	-	2	0	2
3536	3536	3538	+	3766	-	-	+	1	1	2
3536	3536	3538	+	4319	4317	4319	+	1	1	2
3541	3541	3543	+	3612	-	-	-	0	2	2
3541	3541	3543	+	4308	-	-	+	1	1	2
3557	3557	3562	-	3726	-	-	-	2	0	2
3557	3557	3562	-	4372	-	-	-	1	1	2
3557	3556	3560	+	3697	3697	3698	+	1	1	2
3557	3556	3560	+	3773	-	-	+	1	1	2
3557	3556	3560	+	3789	-	-	+	1	1	2
3562	3562	3566	+	3592	-	-	+	1	1	2
3562	3562	3566	+	4100	-	-	+	1	1	2
3562	3562	3566	+	4309	4305	4309	+	1	1	2
3576	3575	3579	+	3627	-	-	+	1	1	2
3576	3575	3579	+	3688	-	-	+	1	1	2
3576	3575	3579	+	3709	-	-	+	1	1	2
3577	3575	3579	-	3729	-	-	-	0	2	2
3577	3575	3579	-	4270	-	-	-	1	1	2
3577	3575	3579	-	4773	-	-	-	1	1	2
3581	3581	3582	+	3658	-	-	+	0	2	2
3582	3582	3584	-	3936	3936	3937	-	0	2	2
3591	3587	3594	+	4303	-	-	+	2	0	2
3592	3588	3596	-	3719	-	-	-	0	2	2
3592	3588	3596	-	3750	3747	3750	-	1	1	2
3592	3588	3596	-	3804	3804	3805	-	1	1	2
3592	3588	3596	-	3821	-	-	-	0	2	2
3592	3588	3596	-	3855	-	-	-	1	1	2
3592	3588	3596	-	4290	-	-	-	2	0	2
3596	3596	3600	+	3627	-	-	+	1	1	2
3600	3599	3605	-	3707	-	-	-	2	0	2
3600	3599	3605	-	3749	-	-	-	1	1	2
3600	3599	3605	-	3857	3855	3857	-	1	1	2
3600	3599	3605	-	4058	-	-	-	1	1	2
3615	3614	3617	-	13628	-	-	+	0	2	2
3617	3617	3619	+	3679	3677	3679	+	1	1	2
3629	3628	3631	-	3771	3771	3772	-	1	1	2
3629	3628	3631	-	3821	-	-	-	1	1	2
3629	3628	3631	-	3854	3854	3855	-	1	1	2
3629	3628	3631	-	4190	-	-	-	2	0	2
3636	-	-	-	3977	-	-	-	1	1	2
3640	3639	3644	+	3674	3674	3678	+	1	1	2
3640	3639	3644	+	3821	3821	3822	+	1	1	2
3643	3640	3646	-	3820	3820	3821	-	2	0	2
3643	3640	3646	-	3884	-	-	-	1	1	2
3643	3640	3646	-	4008	-	-	-	1	1	2
3643	3640	3646	-	7435	-	-	-	1	1	2
3646	3646	3647	+	3678	-	-	+	2	0	2
3653	3650	3656	-	5678	-	-	-	2	0	2
3661	3658	3663	+	3673	3673	3674	+	0	2	2
3690	3685	3692	-	4389	4387	4389	-	1	1	2
3690	3685	3692	-	5607	-	-	+	1	1	2
3690	3686	3692	+	3885	3885	3886	+	1	1	2
3695	3695	3696	-	14233	14233	14234	+	1	1	2
3701	3698	3705	-	4295	4292	4295	-	0	2	2
3721	3721	3724	-	3935	-	-	-	1	1	2
3722	3718	3723	+	3827	-	-	+	1	1	2
3744	-	-	-	4016	-	-	-	1	1	2
3749	3748	3750	-	4044	4044	4045	-	0	2	2
3750	3748	3754	+	3833	-	-	+	2	0	2

3750	3748	3754	+	4042 -	-		+	0	2	2
3750	3748	3754	+	4054 -	-		+	2	0	2
3760	3760	3761	-	4017 -	-		-	1	1	2
3765	3760	3769	+	3833 -	-		+	0	2	2
3765	3760	3769	+	4033 -	-		+	0	2	2
3765	3760	3769	+	4041 -	-		+	0	2	2
3766	3762	3769	-	3934	3934	3935	-	1	1	2
3766	3762	3769	-	4075	4075	4076	-	1	1	2
3766	3762	3769	-	4343 -	-		-	1	1	2
3766	3762	3769	-	4358 -	-		-	1	1	2
3771	3771	3773	-	4032 -	-		-	1	1	2
3773	3772	3774	+	3791 -	-		+	0	2	2
3773	3772	3774	+	15210 -	-		-	0	2	2
3779	3776	3779	-	4023 -	-		-	1	1	2
3779	3776	3779	-	4037	4037	4038	-	1	1	2
3782	3778	3785	+	4194 -	-		+	1	1	2
3786	3784	3790	-	3933	3933	3934	-	1	1	2
3786	3784	3790	-	4433 -	-		-	1	1	2
3801	3799	3805	-	3933 -	-		-	1	1	2
3801	3799	3805	-	3987	3987	3988	-	1	1	2
3805	3805	3808	+	4022 -	-		+	1	1	2
3812	3812	3814	+	4112 -	-		+	2	0	2
3819	3819	3822	-	4021 -	-		-	1	1	2
3838	3838	3842	+	3936 -	-		+	1	1	2
3838	3838	3842	+	4637 -	-		+	1	1	2
3838	3838	3842	+	15235 -	-		+	0	2	2
3844	3844	3845	+	15040 -	-		+	0	2	2
3855	3851	3860	+	3934 -	-		+	1	1	2
3855	3851	3860	+	3974 -	-		+	2	0	2
3855	3851	3860	+	5414 -	-		+	1	1	2
3859	3857	3862	-	3986 -	-		-	1	1	2
3859	3857	3862	-	4008	4006	4008	-	1	1	2
3886	3885	3886	-	4032 -	-		-	1	1	2
3896	3896	3899	-	4134	4130	4134	-	2	0	2
3896	3896	3899	-	4145	4142	4145	-	1	1	2
3901 -	-		-	3971 -	-		-	1	1	2
3901 -	-		-	4104 -	-		-	2	0	2
3911	3911	3912	-	4289	4289	4290	-	1	1	2
3937	3934	3937	+	4036 -	-		+	1	1	2
3962 -	-		+	7486 -	-		+	1	1	2
3963	3963	3967	-	4104 -	-		-	1	1	2
3963	3963	3967	-	4273	4269	4273	-	1	1	2
3987	3986	3988	+	4021 -	-		+	2	0	2
4009	4009	4012	-	4666 -	-		-	1	1	2
4020	4018	4024	+	4033 -	-		+	2	0	2
4021 -	-		-	4211 -	-		-	2	0	2
4021 -	-		-	4305 -	-		-	1	1	2
4026	4026	4028	+	4060	4058	4060	+	2	0	2
4027	4025	4030	-	4304 -	-		+	0	2	2
4032	4032	4033	+	4054	4052	4054	+	1	1	2
4037	4034	4041	-	4276	4276	4277	-	1	1	2
4043	4043	4046	+	4072	4069	4072	+	1	1	2
4059	4058	4059	-	4282 -	-		-	1	1	2
4065	4061	4072	-	4289	4289	4290	-	1	1	2
4065	4061	4072	-	4432 -	-		-	1	1	2
4065	4061	4072	-	4591 -	-		-	1	1	2
4075	4075	4077	-	4300	4298	4300	-	1	1	2
4102	4100	4106	-	4247 -	-		-	1	1	2
4102	4100	4106	-	4260 -	-		-	1	1	2
4102	4100	4106	-	4309	4309	4310	-	1	1	2
4102	4100	4106	-	4432 -	-		-	1	1	2
4102	4100	4106	-	4846	4846	4847	-	1	1	2
4115	4114	4115	-	4381	4381	4382	-	1	1	2
4120	4116	4120	+	4149	4147	4149	+	1	1	2
4120	4116	4120	+	4217	4213	4217	+	1	1	2
4123	4123	4126	-	4463 -	-		-	1	1	2
4145	4143	4148	-	4295	4295	4296	-	1	1	2
4145	4143	4148	-	4300 -	-		-	1	1	2
4179	4178	4179	+	4201 -	-		+	1	1	2
4185	4185	4186	-	4541 -	-		-	1	1	2
4198	4198	4199	-	4816 -	-		-	1	1	2
4214	4212	4216	-	4336 -	-		-	1	1	2
4214	4212	4216	-	4361 -	-		-	1	1	2
4247	4245	4247	+	4366 -	-		+	2	0	2
4268	4264	4268	-	4432	4432	4433	-	1	1	2
4269	4265	4271	+	4304	4302	4304	+	1	1	2
4269	4265	4271	+	4405	4405	4406	+	2	0	2
4274	4274	4278	-	4458 -	-		-	1	1	2
4278	4278	4281	+	9218 -	-		+	1	1	2
4286	4286	4289	-	4604 -	-		-	0	2	2
4290	4290	4293	+	4305	4302	4305	+	2	0	2
4302	4302	4303	+	4303 -	-		+	0	2	2
4302	4302	4303	+	12696 -	-		+	2	0	2
4304	4304	4309	-	4461 -	-		-	1	1	2

4304	4304	4309	-	4472	4468	4472	-	0	2	2
4311	4311	4316	-	4461	4461	4462	-	1	1	2
4311	-	-	+	4352	-	-	+	2	0	2
4356	-	-	-	4569	-	-	-	1	1	2
4361	4361	4362	+	8114	-	-	+	1	1	2
4364	4361	4364	-	4472	-	-	-	1	1	2
4374	4371	4374	-	4488	4485	4488	-	1	1	2
4380	4380	4384	-	4626	4622	4626	-	1	1	2
4404	4402	4404	-	4541	-	-	-	1	1	2
4404	4402	4406	+	4555	-	-	+	1	1	2
4404	4402	4406	+	4600	-	-	+	1	1	2
4412	4412	4414	-	4543	4543	4544	-	1	1	2
4421	4420	4421	+	10663	-	-	-	2	0	2
4434	4430	4436	-	4617	-	-	-	1	1	2
4434	4430	4436	-	10110	-	-	-	1	1	2
4446	4446	4450	-	4593	4593	4594	-	0	2	2
4455	4455	4456	+	4416	-	-	-	1	1	2
4455	4455	4456	+	4471	-	-	+	0	2	2
4484	4482	4485	+	4536	-	-	+	0	2	2
4484	4482	4485	+	5913	-	-	+	0	2	2
4502	-	-	+	5900	-	-	+	1	1	2
4513	4509	4513	+	4840	4838	4840	+	1	1	2
4513	4509	4513	+	5456	-	-	+	1	1	2
4529	4529	4533	+	5688	-	-	+	1	1	2
4529	4529	4533	+	5877	-	-	-	1	1	2
4544	4541	4548	+	5857	5854	5857	+	1	1	2
4554	4553	4554	-	15111	-	-	-	2	0	2
4555	4552	4557	+	4576	-	-	+	1	1	2
4560	4558	4560	-	4590	-	-	-	0	2	2
4564	4564	4568	+	5833	-	-	+	1	1	2
4567	4561	4571	-	4777	4777	4778	-	1	1	2
4567	4561	4571	-	4924	-	-	-	0	2	2
4567	4561	4571	-	5515	5515	5517	-	0	2	2
4577	4573	4578	-	4705	4705	4706	-	1	1	2
4577	4573	4578	-	10021	10017	10021	+	0	2	2
4586	4582	4589	+	4969	-	-	-	2	0	2
4586	4582	4589	+	5615	-	-	+	0	2	2
4586	4582	4589	+	13437	-	-	-	1	1	2
4587	4583	4588	-	4726	-	-	-	1	1	2
4596	4592	4599	+	4654	-	-	+	1	1	2
4596	4592	4599	+	4696	4692	4696	-	2	0	2
4596	4592	4599	+	4706	4706	4707	+	1	1	2
4598	4597	4601	-	4783	-	-	-	1	1	2
4598	4597	4601	-	5001	-	-	-	0	2	2
4598	4597	4601	-	5072	-	-	-	0	2	2
4598	4597	4601	-	5676	-	-	-	1	1	2
4598	4597	4601	-	5700	-	-	-	0	2	2
4606	4606	4609	+	5657	-	-	+	0	2	2
4617	4615	4619	+	4642	-	-	+	2	0	2
4619	4619	4620	-	5586	-	-	-	1	1	2
4624	4621	4624	+	5481	-	-	+	1	1	2
4632	4629	4634	-	4811	-	-	-	1	1	2
4632	4629	4634	-	5124	-	-	-	2	0	2
4633	4633	4635	+	5585	-	-	+	1	1	2
4633	4633	4635	+	5666	-	-	+	0	2	2
4637	4637	4638	-	5216	-	-	-	2	0	2
4637	4637	4638	-	5469	-	-	-	2	0	2
4637	4637	4638	-	5587	-	-	-	0	2	2
4641	4639	4642	+	5032	-	-	+	0	2	2
4648	4648	4652	+	4685	-	-	+	2	0	2
4648	4648	4652	+	4693	-	-	+	2	0	2
4658	4654	4660	-	4751	-	-	-	1	1	2
4658	4654	4660	-	4805	-	-	-	1	1	2
4658	4654	4660	-	5062	-	-	-	1	1	2
4658	4654	4660	-	5499	-	-	-	1	1	2
4667	4663	4671	-	5099	-	-	-	2	0	2
4667	4663	4671	-	5288	-	-	-	1	1	2
4667	4663	4671	-	5509	5509	5510	-	1	1	2
4684	4683	4687	+	4768	4764	4768	+	1	1	2
4685	4682	4689	-	4933	-	-	-	0	2	2
4685	4682	4689	-	5657	-	-	-	2	0	2
4704	4703	4704	-	5026	-	-	-	2	0	2
4715	4712	4715	-	4990	-	-	-	2	0	2
4715	4713	4719	+	8585	8581	8585	-	1	1	2
4729	4727	4737	+	4998	-	-	+	1	1	2
4729	4727	4737	+	5469	-	-	+	1	1	2
4739	4736	4745	-	5026	-	-	-	1	1	2
4739	4736	4745	-	10365	-	-	-	1	1	2
4743	4738	4747	+	4909	-	-	+	2	0	2
4743	4738	4747	+	5011	5011	5013	+	1	1	2
4743	4738	4747	+	5026	5026	5028	+	1	1	2
4743	4738	4747	+	5297	-	-	+	1	1	2
4753	4750	4757	-	12043	-	-	-	1	1	2
4754	4753	4756	+	5122	-	-	+	0	2	2

4754	4753	4756	+	5386 -	-		+	0	2	2
4767	4763	4767	+	5029 -	-		+	1	1	2
4767	4763	4767	+	5141	5139	5141	+	1	1	2
4768	4765	4768	-	4929 -	-		-	1	1	2
4768	4765	4768	-	5139 -	-		-	1	1	2
4768	4765	4768	-	5352	5352	5353	-	1	1	2
4773	4773	4774	-	4905 -	-		-	1	1	2
4773	4773	4774	-	5356 -	-		-	1	1	2
4779	4776	4783	-	4922 -	-		-	1	1	2
4779	4776	4783	-	4989 -	-		-	1	1	2
4779	4776	4783	-	5001	5001	5002	-	0	2	2
4779	4776	4783	-	5761 -	-		-	1	1	2
4795	4795	4797	+	4816 -	-		+	1	1	2
4804	4804	4805	+	4839 -	-		+	1	1	2
4804	4804	4805	+	5326 -	-		+	1	1	2
4804	4804	4805	+	5390 -	-		+	1	1	2
4805	4804	4807	-	5122 -	-		-	2	0	2
4805	4804	4807	-	5322	5322	5323	-	1	1	2
4805	4804	4807	-	5329	5326	5329	-	1	1	2
4813	4811	4817	-	5057 -	-		-	1	1	2
4813	4811	4817	-	5156 -	-		-	1	1	2
4813	4811	4817	-	5264	5264	5265	-	1	1	2
4813	4811	4817	-	14895 -	-		+	0	2	2
4834 -	-		-	5184 -	-		-	2	0	2
4845	4842	4847	+	4909	4907	4909	+	1	1	2
4845	4842	4847	+	5308	5306	5308	+	1	1	2
4846	4846	4850	-	5068	5066	5068	-	0	2	2
4855	4852	4859	-	5007 -	-		-	1	1	2
4855	4852	4859	-	5028	5028	5032	-	0	2	2
4855	4852	4859	-	5307 -	-		-	0	2	2
4869	4865	4873	+	4909 -	-		+	1	1	2
4873	4873	4877	-	5323	5320	5323	-	1	1	2
4886	4885	4886	-	4897 -	-		+	0	2	2
4889	4887	4891	+	4946 -	-		+	0	2	2
4893	4889	4894	-	5176	5176	5177	-	1	1	2
4899 -	-		+	5569 -	-		+	0	2	2
4909	4905	4910	-	5128 -	-		-	1	1	2
4909	4905	4910	-	5181	5181	5182	-	0	2	2
4909	4905	4913	+	5028 -	-		+	2	0	2
4920	4918	4920	-	4894	4892	4894	+	1	1	2
4920	4918	4920	-	5147 -	-		-	1	1	2
4925	4923	4927	-	5067 -	-		-	1	1	2
4925	4923	4927	-	5177 -	-		-	1	1	2
4928	4925	4932	+	7763 -	-		-	2	0	2
4940	4940	4943	-	13105 -	-		+	1	1	2
4940	4939	4940	+	12865 -	-		+	2	0	2
4945 -	-		+	5011 -	-		+	1	1	2
4956	4955	4958	-	5085 -	-		-	1	1	2
4956	4955	4958	-	5226	5226	5227	-	1	1	2
4965	4965	4968	+	4991 -	-		+	1	1	2
4965	4965	4968	+	5013 -	-		+	1	1	2
4979	4974	4984	-	5111 -	-		-	1	1	2
4988	4986	4988	-	5138	5138	5139	-	1	1	2
4988	4986	4988	-	5146 -	-		-	0	2	2
4997	4997	5001	+	5085	5083	5085	+	1	1	2
4998	4995	5002	-	5140 -	-		-	1	1	2
4998	4995	5002	-	5182 -	-		-	1	1	2
4998	4995	5002	-	5305	5305	5306	-	1	1	2
5028 -	-		+	5111 -	-		+	1	1	2
5029	5027	5033	-	5165	5165	5166	-	1	1	2
5029	5027	5033	-	5180	5180	5181	-	1	1	2
5029	5027	5033	-	5193 -	-		-	1	1	2
5029	5027	5033	-	5459 -	-		-	1	1	2
5039 -	-		+	7439 -	-		+	2	0	2
5046	5046	5049	-	5217 -	-		-	1	1	2
5048	5048	5050	+	5070 -	-		+	1	1	2
5069	5066	5072	-	5296 -	-		-	1	1	2
5069	5066	5072	-	5322 -	-		-	1	1	2
5069	5066	5072	-	5432 -	-		-	0	2	2
5069	5066	5072	-	15096	15093	15096	+	1	1	2
5071	5067	5074	+	5183 -	-		+	2	0	2
5071	5067	5074	+	9002 -	-		+	0	2	2
5071	5067	5074	+	15093	15090	15093	-	1	1	2
5080	5078	5080	+	15083	15081	15083	-	2	0	2
5084	5083	5087	-	5324	5324	5325	-	1	1	2
5085	5082	5086	+	5111	5108	5111	+	1	1	2
5085	5082	5086	+	15079	15076	15079	-	0	2	2
5092	5091	5096	+	5153	5153	5154	+	1	1	2
5092	5091	5096	+	15065 -	-		-	1	1	2
5093	5093	5097	-	15063	15063	15064	+	0	2	2
5109	5104	5112	-	5249 -	-		-	1	1	2
5109	5104	5112	-	5309	5307	5309	-	1	1	2
5109	5104	5112	-	5326 -	-		-	1	1	2
5109	5104	5112	-	5694 -	-		-	1	1	2

5121	5118	5125	-	5307	-	-	-	1	1	2
5121	5121	5123	+	5337	-	-	+	1	1	2
5128	5128	5132	+	5295	-	-	+	1	1	2
5133	5133	5135	-	5602	-	-	-	0	2	2
5140	5139	5144	-	5305	5305	5306	-	2	0	2
5140	5139	5144	-	5326	-	-	-	1	1	2
5140	5139	5144	-	5603	5603	5604	-	1	1	2
5153	5152	5155	-	5308	5306	5308	-	1	1	2
5153	5152	5155	-	5324	5324	5325	-	1	1	2
5153	5152	5155	-	5515	-	-	-	0	2	2
5154	5151	5156	+	5265	-	-	+	1	1	2
5159	-	-	+	5661	-	-	-	1	1	2
5177	5172	5183	+	5308	-	-	+	1	1	2
5177	5172	5183	+	5459	-	-	+	1	1	2
5200	-	-	+	5262	-	-	+	0	2	2
5206	5202	5207	+	5278	-	-	+	1	1	2
5206	5202	5207	+	5655	-	-	+	1	1	2
5208	5205	5209	-	5409	5406	5409	-	1	1	2
5208	5205	5209	-	5435	-	-	-	1	1	2
5221	5221	5222	-	5389	-	-	-	1	1	2
5237	5237	5240	-	5615	5615	5616	-	1	1	2
5242	5242	5243	-	5371	5371	5372	-	1	1	2
5247	5246	5247	-	5294	-	-	+	1	1	2
5254	5250	5256	+	5279	-	-	+	1	1	2
5254	5250	5256	+	5286	5286	5287	-	1	1	2
5254	5250	5256	+	5296	5296	5297	+	0	2	2
5254	5250	5256	+	10995	10995	10996	+	1	1	2
5254	5250	5256	+	12854	-	-	+	1	1	2
5266	5262	5267	-	5521	-	-	-	2	0	2
5266	5262	5267	-	5603	-	-	-	1	1	2
5266	5262	5267	-	5885	-	-	-	0	2	2
5266	5262	5267	-	6046	6046	6047	-	1	1	2
5268	5268	5269	+	5280	5280	5281	+	1	1	2
5273	5272	5276	-	5617	-	-	-	2	0	2
5289	5285	5289	-	5252	-	-	+	2	0	2
5294	5293	5297	-	5476	-	-	-	1	1	2
5295	5291	5298	+	5342	5342	5343	+	1	1	2
5295	5291	5298	+	5621	-	-	+	1	1	2
5326	5322	5328	+	5340	-	-	+	0	2	2
5326	5322	5328	+	5365	-	-	+	1	1	2
5326	5322	5328	+	5444	-	-	+	1	1	2
5342	5342	5343	-	5524	5522	5524	-	1	1	2
5342	5342	5343	-	5616	-	-	-	1	1	2
5347	-	-	+	5359	-	-	+	0	2	2
5349	5349	5353	-	5593	-	-	-	2	0	2
5359	5356	5362	-	5547	-	-	-	1	1	2
5363	5361	5363	+	5384	-	-	+	1	1	2
5411	5407	5411	+	5444	-	-	+	1	1	2
5422	5421	5426	-	5539	-	-	-	1	1	2
5422	5420	5425	+	5446	5444	5446	+	2	0	2
5422	5420	5425	+	12392	12389	12392	+	0	2	2
5434	5430	5439	-	5963	5963	5964	-	1	1	2
5452	5451	5452	-	5673	5673	5674	-	1	1	2
5459	5458	5460	-	5604	-	-	-	1	1	2
5459	5458	5460	-	5648	-	-	-	2	0	2
5476	5474	5481	-	5638	-	-	-	1	1	2
5476	5474	5481	-	7712	-	-	+	0	2	2
5489	5488	5489	+	5676	-	-	-	2	0	2
5502	5502	5504	-	5813	-	-	-	1	1	2
5537	5537	5538	+	6050	-	-	+	0	2	2
5588	5584	5592	+	5788	-	-	+	0	2	2
5604	-	-	+	5653	-	-	+	1	1	2
5619	5617	5620	-	5933	-	-	-	1	1	2
5627	-	-	-	5833	-	-	-	1	1	2
5640	5636	5642	-	5833	-	-	-	2	0	2
5649	5645	5650	-	6004	6001	6004	-	0	2	2
5654	5651	5655	-	5874	5874	5875	-	1	1	2
5654	5651	5655	-	5892	-	-	-	1	1	2
5654	5651	5655	-	6017	-	-	-	0	2	2
5674	5671	5675	-	5813	5809	5813	-	1	1	2
5674	5671	5675	-	5833	-	-	-	1	1	2
5679	5679	5682	+	5694	-	-	+	1	1	2
5685	5684	5685	-	5811	-	-	-	1	1	2
5687	-	-	+	5815	-	-	+	0	2	2
5695	5695	5696	-	5834	5834	5835	-	1	1	2
5703	5700	5704	-	5786	-	-	-	1	1	2
5703	5700	5704	-	5873	-	-	-	1	1	2
5713	-	-	-	5999	-	-	-	1	1	2
5722	5719	5722	+	5783	5783	5784	+	1	1	2
5727	5727	5728	+	5790	5790	5791	+	1	1	2
5733	5733	5734	-	8754	8754	8755	+	1	1	2
5738	5738	5739	-	6040	-	-	-	0	2	2
5743	-	-	-	5774	-	-	+	1	1	2
5756	5755	5759	-	5882	-	-	-	2	0	2

5761	5761	5763	-	6005	6003	6005	-	1	1	2
5761	5761	5763	-	6019 -	-		-	1	1	2
5761 -	-		+	5784 -	-		+	1	1	2
5776 -	-		+	5787 -	-		+	2	0	2
5791	5788	5792	-	5999	5999	6000	-	0	2	2
5804	5804	5805	-	6000 -	-		-	1	1	2
5804	5804	5805	-	6038 -	-		-	1	1	2
5815	5812	5818	+	5834 -	-		+	0	2	2
5815	5812	5818	+	5987 -	-		+	1	1	2
5836	5833	5836	-	6059 -	-		-	0	2	2
5836	5833	5836	-	6129 -	-		-	1	1	2
5853	5853	5854	+	5880	5876	5880	+	2	0	2
5860	5858	5863	+	5892	5892	5893	+	0	2	2
5892	5892	5894	+	5946 -	-		+	2	0	2
5911 -	-		-	6200 -	-		-	1	1	2
5983 -	-		+	6017 -	-		+	1	1	2
6008	6008	6011	-	6128 -	-		-	1	1	2
6008	6008	6011	-	6182 -	-		-	1	1	2
6028 -	-		-	6162 -	-		-	2	0	2
6041	6039	6041	-	6194	6194	6195	-	1	1	2
6041	6039	6041	-	12116 -	-		+	1	1	2
6082 -	-		-	6194 -	-		-	1	1	2
6112 -	-		-	8542 -	-		-	0	2	2
6187 -	-		+	6324 -	-		-	2	0	2
6198 -	-		-	11120 -	-		+	2	0	2
6211 -	-		-	11117 -	-		+	0	2	2
6221	6221	6222	-	6386 -	-		-	2	0	2
6221	6221	6222	-	6456 -	-		-	1	1	2
6228	6223	6232	-	6320 -	-		-	1	1	2
6228	6223	6232	-	6404 -	-		-	1	1	2
6228	6223	6232	-	6425	6425	6428	-	2	0	2
6228	6223	6232	-	6464	6464	6465	-	2	0	2
6228	6223	6232	-	6482	6479	6482	-	1	1	2
6228	6223	6232	-	6494	6490	6494	-	2	0	2
6228	6223	6232	-	14346 -	-		-	2	0	2
6236	6234	6239	-	6264 -	-		+	1	1	2
6236	6234	6239	-	6389 -	-		-	1	1	2
6236	6234	6239	-	6396	6396	6397	-	1	1	2
6236	6234	6239	-	6501 -	-		-	2	0	2
6242	6240	6247	-	6445	6445	6446	-	2	0	2
6242	6240	6247	-	6479	6479	6480	-	1	1	2
6242	6240	6247	-	6948 -	-		-	1	1	2
6251	6249	6254	-	6383 -	-		-	1	1	2
6251	6249	6254	-	6389 -	-		-	1	1	2
6251	6249	6254	-	6416 -	-		-	1	1	2
6251	6249	6254	-	6465 -	-		-	0	2	2
6251	6249	6254	-	6885 -	-		-	1	1	2
6267	6264	6267	+	13940 -	-		+	1	1	2
6269	6265	6271	-	6396	6396	6397	-	1	1	2
6269	6265	6271	-	6490 -	-		-	1	1	2
6285	6282	6287	+	6351	6351	6352	+	2	0	2
6285	6282	6287	+	6363	6363	6364	+	2	0	2
6285	6282	6287	+	6397 -	-		+	1	1	2
6292	6289	6294	+	6357	6357	6359	+	2	0	2
6292	6289	6294	+	6373	6373	6374	+	1	1	2
6298	6295	6300	+	6340	6340	6341	+	1	1	2
6298	6295	6300	+	6370	6368	6370	+	2	0	2
6298	6295	6300	+	6389 -	-		+	1	1	2
6298	6295	6300	+	6453 -	-		-	2	0	2
6303	6301	6307	+	6334 -	-		+	1	1	2
6303	6301	6307	+	6371	6368	6371	+	1	1	2
6309	6308	6313	+	6319	6319	6320	+	1	1	2
6309	6308	6313	+	7930	7930	7931	+	1	1	2
6309	6308	6313	+	7945 -	-		+	1	1	2
6309	6308	6313	+	8360 -	-		+	1	1	2
6315	6314	6318	+	6363 -	-		+	1	1	2
6320	6315	6320	-	6407	6407	6408	-	1	1	2
6320	6319	6325	+	6324	6321	6324	+	1	1	2
6320	6319	6325	+	6332	6332	6333	+	1	1	2
6325	6324	6328	-	6427 -	-		-	1	1	2
6333	6330	6333	-	6440 -	-		-	1	1	2
6334	6334	6335	+	6351	6351	6352	+	1	1	2
6338	6338	6342	-	6456 -	-		-	1	1	2
6341	6336	6344	+	6483 -	-		+	1	1	2
6349	6346	6353	+	6373	6373	6374	+	1	1	2
6356	6356	6359	+	6384 -	-		+	1	1	2
6361	6361	6363	+	6364 -	-		+	0	2	2
6370	6368	6374	-	6491 -	-		-	1	1	2
6370	6368	6374	-	6555 -	-		-	1	1	2
6398	6398	6402	-	6450	6450	6451	+	2	0	2
6398	6398	6402	-	6959 -	-		-	1	1	2
6458	6456	6462	+	6491 -	-		+	0	2	2
6458	6456	6462	+	6512	6510	6512	+	1	1	2
6458	6456	6462	+	6587 -	-		+	1	1	2

6480	6480	6481	-	6682	6682	6683	-	0	2	2
6491	6487	6491	-	6672	-	-	-	1	1	2
6509	6508	6510	+	7052	-	-	+	1	1	2
6509	6508	6510	+	8352	-	-	+	1	1	2
6520	6519	6524	-	6673	6673	6674	-	1	1	2
6524	6521	6524	+	7229	7226	7229	+	1	1	2
6556	6555	6558	-	6723	-	-	-	1	1	2
6562	6561	6562	-	7056	-	-	-	0	2	2
6576	6573	6579	-	7103	-	-	-	2	0	2
6584	6580	6587	-	6716	6716	6717	-	1	1	2
6584	6580	6587	-	6854	-	-	-	1	1	2
6584	6580	6587	-	6947	6947	6948	-	1	1	2
6588	-	-	+	7999	-	-	+	2	0	2
6594	6590	6595	-	6812	-	-	+	0	2	2
6594	6590	6595	-	6826	6826	6827	-	2	0	2
6599	6599	6603	-	6976	-	-	-	1	1	2
6608	6607	6608	+	7109	-	-	+	1	1	2
6608	6607	6608	+	8231	-	-	+	1	1	2
6609	6607	6609	-	6778	6778	6779	-	1	1	2
6614	6614	6617	-	6758	-	-	-	1	1	2
6614	6614	6617	-	6787	-	-	-	1	1	2
6614	6614	6617	-	6792	6792	6794	-	1	1	2
6640	6639	6645	-	6750	6748	6750	-	1	1	2
6642	6642	6644	+	6731	-	-	-	1	1	2
6651	6648	6651	-	6951	-	-	-	1	1	2
6658	6657	6659	-	6770	-	-	-	1	1	2
6658	6657	6659	-	6854	-	-	-	1	1	2
6665	-	-	+	6866	-	-	+	1	1	2
6673	6671	6676	+	6719	-	-	+	1	1	2
6694	-	-	+	6959	-	-	+	1	1	2
6700	6699	6704	+	7114	-	-	+	1	1	2
6719	6719	6724	-	7504	-	-	-	1	1	2
6738	6737	6742	-	6959	6959	6960	-	1	1	2
6738	6737	6742	-	8328	8328	8329	-	1	1	2
6756	6756	6759	-	7117	-	-	-	0	2	2
6756	6756	6759	-	8037	-	-	-	1	1	2
6795	6794	6795	-	7001	-	-	-	0	2	2
6820	6818	6820	-	7243	-	-	-	1	1	2
6826	6823	6829	+	6841	-	-	+	1	1	2
6840	6840	6841	+	7409	7409	7410	+	1	1	2
6854	6854	6856	-	7076	-	-	-	1	1	2
6854	6854	6856	-	13176	-	-	+	0	2	2
6854	6850	6858	+	7340	-	-	-	0	2	2
6854	6850	6858	+	7664	-	-	+	0	2	2
6878	6875	6882	-	7351	-	-	-	0	2	2
6890	6889	6890	-	7067	-	-	-	1	1	2
6912	6908	6913	-	7165	-	-	-	1	1	2
6917	-	-	-	6896	-	-	+	2	0	2
6917	-	-	+	7779	-	-	+	1	1	2
6936	6935	6936	+	15368	-	-	-	2	0	2
6946	6946	6949	-	7393	-	-	-	1	1	2
6946	6946	6952	+	6970	-	-	+	0	2	2
6958	6958	6959	+	7069	-	-	+	0	2	2
6971	6971	6972	+	7099	7099	7100	+	1	1	2
6987	6983	6987	-	8151	-	-	-	2	0	2
7019	7015	7021	+	7036	7034	7036	+	2	0	2
7019	7015	7021	+	7052	7050	7052	+	1	1	2
7020	7016	7020	-	7643	-	-	-	1	1	2
7034	7033	7037	-	8183	-	-	-	2	0	2
7041	7041	7045	+	7544	-	-	+	1	1	2
7069	7064	7069	-	8407	-	-	-	1	1	2
7084	7081	7087	-	7145	7143	7145	-	1	1	2
7084	7081	7087	-	7264	-	-	-	0	2	2
7084	7081	7087	-	7343	7343	7345	-	0	2	2
7084	7081	7087	-	7392	-	-	-	0	2	2
7089	7089	7092	-	7367	-	-	-	2	0	2
7098	7098	7101	-	7160	-	-	-	1	1	2
7098	7098	7101	-	7184	-	-	-	0	2	2
7103	7100	7103	+	8096	-	-	+	1	1	2
7107	7107	7111	-	7277	-	-	-	1	1	2
7108	-	-	+	7581	-	-	+	1	1	2
7116	7116	7117	-	7301	7301	7302	-	1	1	2
7120	7117	7120	+	7147	-	-	+	1	1	2
7130	7126	7132	+	7221	-	-	+	1	1	2
7156	7154	7157	-	7521	-	-	-	1	1	2
7158	7155	7158	+	7258	7258	7259	-	1	1	2
7165	7165	7166	-	7601	-	-	-	1	1	2
7170	7169	7174	+	7502	7502	7503	+	1	1	2
7195	7190	7198	+	7850	-	-	+	1	1	2
7200	7200	7203	-	7447	7447	7448	-	1	1	2
7209	7205	7209	-	7304	-	-	-	1	1	2
7218	7214	7219	-	7387	-	-	-	1	1	2
7218	7214	7219	-	7409	-	-	-	1	1	2
7224	7224	7229	-	7381	-	-	-	1	1	2

7224	7224	7229	-	7386	-	-	-	1	1	2
7229	7225	7229	+	7239	-	-	+	1	1	2
7234	7231	7234	-	7385	7383	7385	-	1	1	2
7240	7236	7240	-	7395	-	-	-	1	1	2
7244	7244	7246	+	7448	-	-	-	2	0	2
7246	7242	7248	-	7386	-	-	-	1	1	2
7246	7242	7248	-	7402	-	-	-	1	1	2
7246	7242	7248	-	7698	7698	7699	-	1	1	2
7265	7265	7266	-	7352	7350	7352	-	1	1	2
7277	-	-	-	12332	-	-	+	1	1	2
7282	7279	7285	+	7293	-	-	+	1	1	2
7285	-	-	-	8036	-	-	-	2	0	2
7293	7292	7293	-	7435	-	-	-	1	1	2
7293	7289	7295	+	7308	7304	7308	+	2	0	2
7313	-	-	+	7385	-	-	+	1	1	2
7327	-	-	-	9597	-	-	+	2	0	2
7345	7345	7347	-	7609	-	-	-	1	1	2
7352	-	-	-	7387	-	-	+	1	1	2
7392	7392	7397	-	7472	-	-	-	1	1	2
7392	7392	7397	-	7640	7637	7640	-	1	1	2
7406	7402	7406	-	7759	7759	7760	-	1	1	2
7406	7402	7406	-	7850	-	-	-	1	1	2
7415	7411	7417	-	7536	7536	7537	-	1	1	2
7415	7411	7417	-	7552	7550	7552	-	1	1	2
7442	7438	7446	+	7502	-	-	+	1	1	2
7442	7438	7446	+	7579	-	-	+	2	0	2
7442	7438	7446	+	8200	-	-	+	1	1	2
7447	7446	7449	-	7565	7565	7566	-	1	1	2
7458	7454	7459	+	8328	8328	8329	+	1	1	2
7461	7461	7465	-	7593	-	-	-	1	1	2
7461	7461	7465	-	7617	7615	7617	-	1	1	2
7475	7475	7476	-	8177	-	-	-	2	0	2
7481	-	-	-	8401	8399	8401	-	0	2	2
7489	7486	7489	-	7566	7563	7566	-	1	1	2
7495	7492	7495	+	12520	-	-	+	1	1	2
7515	7515	7519	+	7696	7692	7696	+	1	1	2
7538	7534	7538	-	7518	-	-	+	2	0	2
7538	7534	7538	-	7669	-	-	-	0	2	2
7544	7544	7548	-	7589	-	-	-	0	2	2
7551	7551	7554	+	7604	-	-	+	1	1	2
7556	7556	7560	-	7919	-	-	-	1	1	2
7566	7562	7568	-	7823	-	-	-	1	1	2
7566	7562	7568	-	7843	-	-	-	1	1	2
7578	7576	7581	-	7930	-	-	-	0	2	2
7588	7588	7591	+	7619	7619	7620	+	1	1	2
7588	7588	7591	+	12367	-	-	+	1	1	2
7604	7604	7606	+	8372	-	-	+	1	1	2
7604	7604	7606	+	9377	9377	9378	-	1	1	2
7611	-	-	+	9513	-	-	-	2	0	2
7618	7614	7620	-	7786	7786	7787	-	1	1	2
7618	7614	7620	-	7923	-	-	-	1	1	2
7622	7619	7623	+	7649	7645	7649	+	0	2	2
7624	7623	7624	-	9395	-	-	-	1	1	2
7630	7629	7630	-	7636	-	-	-	2	0	2
7635	7631	7639	-	7770	-	-	-	1	1	2
7635	7631	7639	-	7928	7928	7930	-	1	1	2
7635	7631	7639	-	8011	8011	8013	-	1	1	2
7636	7636	7637	+	7630	-	-	+	2	0	2
7645	7643	7650	-	7975	-	-	-	2	0	2
7645	7643	7650	-	8026	8026	8027	-	1	1	2
7655	7654	7658	-	7787	-	-	-	1	1	2
7655	7654	7658	-	7979	7977	7979	-	2	0	2
7655	7654	7658	-	8036	-	-	-	1	1	2
7666	-	-	-	7834	-	-	-	2	0	2
7683	7679	7684	-	7845	-	-	-	1	1	2
7683	7679	7684	-	7905	7905	7906	-	1	1	2
7683	7679	7684	-	7917	7917	7918	-	1	1	2
7697	7695	7700	-	7900	-	-	-	1	1	2
7697	7695	7700	-	7943	7943	7944	-	1	1	2
7723	-	-	-	7830	-	-	-	1	1	2
7729	7729	7732	+	7871	7871	7872	+	1	1	2
7737	7737	7738	-	7848	7848	7849	-	1	1	2
7762	7760	7762	+	7810	-	-	+	1	1	2
7778	7777	7781	-	8023	-	-	-	0	2	2
7780	7779	7784	+	7813	-	-	+	1	1	2
7780	7779	7784	+	7961	-	-	+	1	1	2
7787	7786	7788	-	10833	10833	10834	+	1	1	2
7801	7797	7804	+	13237	-	-	+	0	2	2
7816	7813	7816	+	7849	-	-	+	0	2	2
7830	-	-	-	8165	-	-	-	1	1	2
7836	-	-	+	7920	-	-	+	2	0	2
7844	7840	7844	+	7865	-	-	+	1	1	2
7844	7840	7844	+	8167	-	-	+	1	1	2
7844	7840	7844	+	13449	-	-	+	0	2	2

7863	7860	7863	+	8339 -	-		+	1	1	2
7871	7869	7874	-	8347 -	-		-	2	0	2
7923	7921	7923	+	7945	7945	7946	+	0	2	2
7931	7930	7935	-	8102 -	-		-	0	2	2
7931	7930	7935	-	8184 -	-		-	1	1	2
7931	7930	7935	-	8277 -	-		-	1	1	2
7940	7937	7944	-	8070 -	-		-	1	1	2
7946	7945	7947	-	8109	8109	8110	-	1	1	2
7966	7962	7966	+	9934 -	-		+	1	1	2
7976	7976	7979	-	8070 -	-		-	2	0	2
7976	7976	7981	+	8199	8199	8200	+	1	1	2
7982	7982	7983	-	8187	8187	8188	-	1	1	2
8010	8007	8010	+	8205 -	-		+	1	1	2
8015 -	-		+	8115 -	-		+	1	1	2
8016 -	-		-	8184 -	-		-	1	1	2
8022	8022	8026	-	8130	8130	8131	-	2	0	2
8035	8035	8036	-	8113	8113	8114	-	1	1	2
8035	8035	8036	-	8129	8129	8130	-	1	1	2
8036	8036	8037	+	8057 -	-		+	1	1	2
8036	8036	8037	+	8072 -	-		+	1	1	2
8042 -	-		+	8100 -	-		+	1	1	2
8070	8069	8074	+	8098	8095	8098	+	2	0	2
8073	8070	8073	-	10942 -	-		-	2	0	2
8082	8078	8082	+	13780 -	-		+	1	1	2
8113	8109	8113	+	8159 -	-		+	0	2	2
8154	8154	8157	+	12921 -	-		-	0	2	2
8165	8161	8170	-	8411 -	-		-	1	1	2
8165	8161	8170	-	13089 -	-		-	1	1	2
8184	8184	8188	+	8222 -	-		+	1	1	2
8189	8186	8192	-	8412	8408	8412	-	1	1	2
8189	8186	8192	-	9770 -	-		+	1	1	2
8198	8198	8199	-	8410 -	-		-	1	1	2
8206 -	-		+	8224 -	-		+	0	2	2
8212	8210	8212	-	8397 -	-		-	1	1	2
8221	8217	8224	-	8360 -	-		-	1	1	2
8221	8217	8224	-	8407	8405	8407	-	1	1	2
8239	8239	8240	-	8341 -	-		-	2	0	2
8276	8274	8279	+	8290	8290	8291	+	2	0	2
8276	8274	8279	+	8308 -	-		+	1	1	2
8461 -	-		-	11489 -	-		+	2	0	2
8464	8463	8464	+	14797 -	-		+	2	0	2
8467	8463	8467	-	8598 -	-		-	2	0	2
8472	8472	8474	-	8788 -	-		-	1	1	2
8472	8472	8474	-	8999 -	-		+	0	2	2
8474	8472	8475	+	9384 -	-		+	1	1	2
8474	8472	8475	+	11765 -	-		+	1	1	2
8481	8479	8484	-	8586 -	-		-	0	2	2
8481	8479	8484	-	8604 -	-		-	1	1	2
8493	8493	8499	+	10038	10038	10039	+	1	1	2
8493	8493	8499	+	14314 -	-		+	2	0	2
8498	8494	8500	-	9016 -	-		-	1	1	2
8498	8494	8500	-	9244 -	-		+	1	1	2
8517	8517	8518	+	14283	14283	14284	+	1	1	2
8524 -	-		-	12793 -	-		-	0	2	2
8544	8541	8544	-	8719 -	-		-	1	1	2
8545 -	-		+	8571 -	-		-	1	1	2
8555 -	-		-	8695 -	-		-	1	1	2
8625	8622	8625	-	8788 -	-		-	1	1	2
8637	8637	8641	+	8974	8972	8974	+	1	1	2
8637	8637	8641	+	15328 -	-		+	1	1	2
8659	8656	8659	+	8939 -	-		+	1	1	2
8659	8656	8659	+	9386 -	-		+	1	1	2
8763 -	-		+	14259 -	-		+	1	1	2
8778 -	-		+	8838	8836	8838	+	1	1	2
8788	8784	8788	+	8835 -	-		+	0	2	2
8788	8784	8788	+	9169 -	-		+	2	0	2
8792 -	-		-	8992 -	-		-	1	1	2
8804	8801	8804	+	8989 -	-		-	2	0	2
8820	8819	8823	-	9223 -	-		-	1	1	2
8831	8827	8831	-	9307	9307	9308	-	1	1	2
8935 -	-		-	9100 -	-		-	1	1	2
8944	8942	8947	-	9101	9098	9101	-	1	1	2
8949	8948	8952	-	9098	9098	9099	-	1	1	2
8949	8948	8952	-	9161 -	-		-	1	1	2
8957	8957	8959	-	13447 -	-		-	2	0	2
8973 -	-		-	9221 -	-		-	2	0	2
8993	8993	8997	+	9225 -	-		-	2	0	2
9038	9038	9039	-	9223	9223	9224	-	1	1	2
9073	9071	9073	+	9132 -	-		+	1	1	2
9086	9086	9090	-	9260 -	-		-	1	1	2
9103	9099	9103	-	12731	12731	12732	-	1	1	2
9108	9108	9114	+	9489	9489	9490	+	1	1	2
9108	9108	9114	+	13869 -	-		+	0	2	2
9108	9108	9114	+	13874	13874	13875	+	1	1	2

9121	-	-	-	9207	-	-	-	1	1	2
9130	9130	9135	-	9374	9374	9375	-	1	1	2
9139	-	-	-	9810	-	-	-	1	1	2
9157	9157	9160	-	13413	-	-	+	1	1	2
9157	9157	9160	-	13964	-	-	-	1	1	2
9165	-	-	+	10088	-	-	+	0	2	2
9197	9197	9199	+	9197	-	-	+	0	2	2
9226	-	-	-	9336	-	-	-	1	1	2
9250	-	-	-	9406	-	-	-	0	2	2
9255	9255	9256	-	9493	9493	9494	-	1	1	2
9261	9260	9261	-	9441	-	-	-	1	1	2
9261	9260	9261	-	13782	-	-	-	2	0	2
9261	-	-	+	9439	-	-	+	1	1	2
9266	-	-	+	9440	-	-	+	1	1	2
9269	9265	9270	-	9458	-	-	-	1	1	2
9269	9265	9270	-	9858	-	-	-	0	2	2
9301	9300	9301	+	9422	-	-	+	0	2	2
9329	9326	9329	+	9407	-	-	+	1	1	2
9329	9326	9329	+	9414	-	-	+	2	0	2
9335	9335	9336	+	9398	9396	9398	+	0	2	2
9335	9335	9336	+	9405	-	-	+	2	0	2
9383	9379	9384	-	10115	-	-	-	0	2	2
9394	9393	9397	-	9664	9662	9664	-	1	1	2
9394	9393	9397	-	9757	9757	9758	-	1	1	2
9394	9393	9397	-	13010	-	-	+	2	0	2
9406	9402	9406	+	9734	-	-	-	2	0	2
9426	-	-	-	9650	-	-	-	1	1	2
9463	-	-	+	9489	-	-	+	1	1	2
9475	-	-	-	9556	-	-	-	1	1	2
9475	9475	9476	+	9492	-	-	+	1	1	2
9475	9475	9476	+	9875	-	-	+	0	2	2
9488	9488	9489	-	9645	-	-	-	1	1	2
9488	9488	9489	-	9752	-	-	-	1	1	2
9491	9488	9491	+	9535	-	-	+	0	2	2
9491	9488	9491	+	9938	-	-	+	1	1	2
9530	9526	9534	+	9556	9552	9556	+	1	1	2
9533	9530	9535	-	9756	-	-	-	2	0	2
9539	9539	9541	+	9747	9747	9748	+	1	1	2
9544	-	-	+	9723	-	-	-	0	2	2
9551	9551	9553	+	9718	9718	9719	+	1	1	2
9556	9555	9557	+	9625	9625	9626	+	1	1	2
9575	9574	9575	+	9626	9626	9627	+	1	1	2
9651	-	-	-	9753	-	-	-	1	1	2
9663	9663	9664	+	9707	9705	9707	+	2	0	2
9715	-	-	-	14345	-	-	-	1	1	2
9722	9721	9722	-	9939	-	-	-	1	1	2
9722	9721	9722	-	11694	-	-	+	0	2	2
9752	9748	9752	+	10552	-	-	+	0	2	2
9752	9748	9752	+	12810	12807	12810	+	1	1	2
9761	9760	9761	+	10068	-	-	-	2	0	2
9794	9794	9798	+	9857	-	-	+	1	1	2
9795	9795	9796	-	9975	-	-	-	1	1	2
9866	9863	9870	-	9920	-	-	-	0	2	2
9866	9863	9870	-	10183	-	-	-	1	1	2
9873	-	-	-	10008	-	-	-	1	1	2
9897	9896	9897	-	12598	-	-	+	2	0	2
9905	9904	9909	-	10337	-	-	-	1	1	2
9928	9926	9928	-	11283	-	-	+	1	1	2
9928	9926	9928	-	13827	-	-	+	2	0	2
9931	9931	9934	+	9945	-	-	+	1	1	2
9931	9931	9934	+	10118	-	-	+	1	1	2
9968	9968	9970	-	9994	-	-	+	2	0	2
9968	9968	9970	-	11998	-	-	+	0	2	2
10025	10024	10025	-	10273	-	-	-	1	1	2
10061	10061	10062	-	10086	-	-	-	0	2	2
10097	10097	10101	+	10351	-	-	+	2	0	2
10124	-	-	+	10145	-	-	+	1	1	2
10129	-	-	+	10327	-	-	-	2	0	2
10143	10143	10146	+	12916	12916	12917	-	1	1	2
10153	-	-	+	10210	10208	10210	+	2	0	2
10184	-	-	-	10491	-	-	-	1	1	2
10203	10203	10206	-	10370	-	-	-	1	1	2
10220	10220	10221	+	10375	-	-	+	0	2	2
10258	10258	10263	-	10311	-	-	-	1	1	2
10258	10258	10263	-	10389	-	-	-	1	1	2
10272	10272	10277	+	13874	-	-	+	0	2	2
10290	-	-	+	10327	-	-	+	0	2	2
10327	-	-	-	13879	-	-	-	2	0	2
10334	10332	10334	-	10470	-	-	-	0	2	2
10340	10340	10342	-	10479	10477	10479	-	0	2	2
10353	-	-	+	10429	-	-	+	2	0	2
10360	-	-	-	13149	-	-	+	2	0	2
10408	-	-	+	13043	-	-	-	0	2	2
10508	-	-	+	10632	-	-	+	2	0	2

10521	10521	10522	-	12405	-	-	+	0	2	2
10529	10529	10532	-	10786	-	-	-	1	1	2
10539	10539	10542	-	10823	10819	10823	-	1	1	2
10539	10539	10542	-	10940	10940	10941	-	1	1	2
10542	10542	10544	+	10570	-	-	+	0	2	2
10548	10546	10550	-	10861	-	-	-	0	2	2
10548	10546	10550	-	10886	10886	10887	-	1	1	2
10548	10546	10550	-	11291	-	-	-	2	0	2
10549	10549	10552	+	10652	10649	10652	+	2	0	2
10575	10572	10576	+	10819	-	-	+	2	0	2
10579	10579	10582	-	15090	15087	15090	+	1	1	2
10588	10586	10588	-	10741	-	-	-	1	1	2
10602	10602	10603	-	10690	10690	10691	-	1	1	2
10645	10645	10649	+	10725	-	-	+	1	1	2
10645	10645	10649	+	10926	-	-	-	2	0	2
10648	-	-	-	10855	-	-	-	1	1	2
10679	10676	10679	+	10764	-	-	+	0	2	2
10706	-	-	-	14864	-	-	+	1	1	2
10775	-	-	-	12664	-	-	+	2	0	2
10793	-	-	+	12296	-	-	+	2	0	2
10844	-	-	+	11272	-	-	+	0	2	2
10847	-	-	-	10910	-	-	-	1	1	2
10847	-	-	-	12403	-	-	+	2	0	2
10858	-	-	+	10997	-	-	+	2	0	2
10889	-	-	+	10978	-	-	+	0	2	2
10912	-	-	+	14657	-	-	+	0	2	2
10939	10935	10940	-	11019	-	-	-	1	1	2
10939	10935	10940	-	11066	11066	11067	-	1	1	2
10965	10965	10966	-	11080	11080	11081	-	1	1	2
10978	10978	10982	-	11106	-	-	-	0	2	2
10987	10984	10991	-	12979	-	-	-	0	2	2
11005	-	-	-	11061	-	-	+	1	1	2
11032	11030	11032	+	11207	-	-	+	2	0	2
11032	11030	11032	+	14578	-	-	+	1	1	2
11036	11035	11040	-	11271	-	-	-	0	2	2
11038	11038	11040	+	14571	-	-	+	1	1	2
11045	-	-	-	13951	-	-	+	1	1	2
11070	11066	11072	-	11560	-	-	-	1	1	2
11079	11076	11079	-	11215	-	-	-	1	1	2
11079	11076	11079	-	11255	-	-	-	0	2	2
11099	11098	11099	-	11271	-	-	-	0	2	2
11127	-	-	-	11283	-	-	-	0	2	2
11181	11181	11182	-	12903	12903	12904	+	1	1	2
11194	11194	11196	-	12054	-	-	-	1	1	2
11194	11194	11196	-	12067	-	-	+	0	2	2
11194	11194	11196	-	12077	12077	12078	-	1	1	2
11203	11200	11203	+	12078	12075	12078	+	1	1	2
11226	11225	11230	-	11506	-	-	-	2	0	2
11226	11225	11230	-	12072	-	-	-	1	1	2
11226	11225	11230	-	12083	12081	12083	-	1	1	2
11238	11236	11238	-	13430	-	-	+	1	1	2
11255	-	-	-	12925	-	-	-	1	1	2
11306	11306	11310	-	13699	13695	13699	-	1	1	2
11330	11328	11330	-	11446	-	-	-	1	1	2
11330	11328	11330	-	11475	-	-	-	1	1	2
11345	-	-	+	13926	-	-	+	1	1	2
11361	11361	11364	+	14262	-	-	+	0	2	2
11368	11364	11368	-	14262	-	-	-	0	2	2
11379	-	-	-	14266	-	-	+	1	1	2
11389	11388	11389	-	13964	-	-	-	1	1	2
11400	11399	11401	+	13950	-	-	+	2	0	2
11409	11408	11409	+	11476	-	-	-	2	0	2
11425	11421	11427	+	13883	-	-	+	2	0	2
11450	-	-	+	14441	-	-	+	0	2	2
11458	11456	11458	-	11784	-	-	-	1	1	2
11459	-	-	+	11854	-	-	-	2	0	2
11495	-	-	+	11577	-	-	+	0	2	2
11500	-	-	+	11576	-	-	+	0	2	2
11534	11534	11535	-	11708	-	-	-	1	1	2
11592	-	-	-	11771	-	-	-	1	1	2
11616	11616	11617	-	13209	-	-	-	1	1	2
11616	11616	11617	-	13224	-	-	+	1	1	2
11622	11622	11623	-	11647	11647	11648	-	0	2	2
11655	11652	11655	-	14573	-	-	+	1	1	2
11668	11667	11668	+	14582	-	-	+	0	2	2
11680	11677	11682	-	12650	-	-	+	1	1	2
11734	11733	11734	+	11844	-	-	+	2	0	2
11742	-	-	+	15167	-	-	+	1	1	2
11770	11770	11771	-	15277	15277	15278	+	1	1	2
11793	-	-	-	11927	-	-	-	0	2	2
11802	11802	11803	-	13088	-	-	-	0	2	2
11827	-	-	+	11847	-	-	+	1	1	2
11846	11844	11846	+	11866	11866	11867	+	1	1	2
11950	-	-	-	12060	-	-	-	1	1	2

11965	11965	11968	+	11980	-	-	+	1	1	2
11965	11965	11968	+	12069	-	-	+	1	1	2
11984	11982	11984	+	12278	-	-	-	2	0	2
12003	12003	12006	+	12143	-	-	+	1	1	2
12003	12003	12006	+	12356	-	-	-	2	0	2
12031	-	-	-	12367	-	-	-	2	0	2
12036	12036	12037	-	12269	12269	12270	-	1	1	2
12036	12036	12037	-	12336	12336	12337	-	0	2	2
12068	-	-	+	12142	-	-	+	2	0	2
12069	12065	12074	-	12208	-	-	-	0	2	2
12095	12093	12095	+	12195	-	-	+	1	1	2
12104	12100	12104	+	12334	-	-	+	0	2	2
12117	-	-	+	12236	-	-	+	2	0	2
12122	12122	12125	+	12228	12224	12228	+	1	1	2
12143	-	-	-	12306	-	-	-	1	1	2
12183	-	-	+	12349	-	-	+	2	0	2
12208	12206	12208	-	13103	-	-	-	2	0	2
12282	-	-	+	12307	-	-	+	1	1	2
12300	12299	12300	+	14999	-	-	+	0	2	2
12308	12308	12309	-	12345	-	-	+	1	1	2
12312	12308	12312	+	12344	-	-	-	1	1	2
12399	12396	12399	+	12668	-	-	-	2	0	2
12413	-	-	-	13663	-	-	+	1	1	2
12446	-	-	+	13129	-	-	+	0	2	2
12466	-	-	+	13150	-	-	+	1	1	2
12495	12491	12496	-	15104	-	-	+	0	2	2
12505	12503	12506	+	12621	12621	12622	-	1	1	2
12509	-	-	-	14426	-	-	+	0	2	2
12529	12526	12529	-	12641	12638	12641	-	0	2	2
12536	12534	12538	-	14080	-	-	-	0	2	2
12536	12534	12538	-	14914	-	-	+	0	2	2
12577	12573	12579	-	12807	12807	12808	-	1	1	2
12577	12573	12579	-	13158	-	-	-	1	1	2
12618	12616	12619	-	13723	-	-	+	2	0	2
12625	12625	12629	-	12783	-	-	-	0	2	2
12625	12623	12629	+	12848	-	-	-	2	0	2
12635	12632	12635	+	12910	-	-	-	2	0	2
12640	12640	12644	+	12708	-	-	-	2	0	2
12640	12640	12644	+	12945	12945	12946	-	2	0	2
12658	12656	12659	+	12644	-	-	-	0	2	2
12658	12656	12659	+	12911	-	-	+	1	1	2
12660	12658	12660	-	15222	-	-	+	1	1	2
12670	12669	12675	+	12703	-	-	+	0	2	2
12670	12669	12675	+	15311	15311	15312	+	1	1	2
12696	12696	12700	-	12875	-	-	-	1	1	2
12696	12696	12700	-	14019	-	-	+	1	1	2
12704	12704	12708	-	12842	-	-	-	1	1	2
12717	12717	12718	-	14613	14613	14614	+	1	1	2
12758	12754	12760	-	12984	-	-	-	2	0	2
12792	12790	12792	-	12983	-	-	-	1	1	2
12792	12790	12792	-	13264	-	-	-	1	1	2
12837	12837	12840	-	12883	-	-	-	0	2	2
12846	12845	12849	-	14372	-	-	-	2	0	2
12851	12851	12852	-	14374	-	-	-	0	2	2
12873	12873	12876	-	12943	-	-	-	2	0	2
12924	12923	12925	+	13494	-	-	+	1	1	2
12924	12923	12925	+	14140	-	-	+	2	0	2
12940	12938	12941	+	13176	-	-	-	2	0	2
12947	12944	12948	+	13296	-	-	-	2	0	2
12948	12948	12951	-	13400	-	-	+	2	0	2
12973	-	-	-	13188	-	-	-	1	1	2
12975	12974	12975	+	12993	-	-	+	0	2	2
12993	12989	12994	-	13162	13162	13163	-	1	1	2
12993	12989	12994	-	14573	-	-	-	1	1	2
13016	13014	13016	+	14572	-	-	-	2	0	2
13029	-	-	-	14859	-	-	+	1	1	2
13039	13038	13039	-	13119	13119	13120	-	1	1	2
13056	13052	13056	-	13273	-	-	+	1	1	2
13091	13087	13093	-	13215	-	-	-	1	1	2
13145	13145	13148	+	13161	-	-	+	0	2	2
13145	13145	13148	+	13169	-	-	+	0	2	2
13146	13146	13150	-	13294	-	-	-	1	1	2
13162	13158	13164	-	13450	-	-	-	1	1	2
13213	-	-	+	14041	-	-	+	0	2	2
13218	13214	13218	-	13353	-	-	-	0	2	2
13218	13214	13218	-	14039	-	-	-	0	2	2
13218	13218	13221	+	14046	-	-	+	2	0	2
13251	-	-	-	13435	-	-	-	1	1	2
13286	13284	13288	-	14377	-	-	+	1	1	2
13293	13293	13298	-	13523	13523	13524	+	1	1	2
13352	13351	13352	+	13369	13365	13369	+	0	2	2
13352	13351	13352	+	13381	-	-	+	0	2	2
13396	-	-	+	14393	-	-	+	0	2	2
13397	13397	13401	-	14392	14389	14392	-	0	2	2

13427 -	-		-	13587 -	-		+	0	2	2
13461 -	-		-	14836 -	-		+	1	1	2
13492 -	-		-	13572 -	-		-	1	1	2
13502 -	-		-	13978 -	-		+	1	1	2
13516	13516	13519	+	13522 -	-		+	1	1	2
13516	13516	13519	+	14044 -	-		+	1	1	2
13516	13516	13519	+	14050 -	-		-	1	1	2
13528	13528	13531	-	13989 -	-		-	0	2	2
13561 -	-		-	14088 -	-		+	0	2	2
13574	13574	13575	-	13764	13764	13765	-	1	1	2
13589 -	-		+	13605 -	-		+	0	2	2
13600	13598	13600	-	14272 -	-		-	1	1	2
13680	13677	13680	-	13844 -	-		-	1	1	2
13688	13686	13692	-	13775 -	-		-	1	1	2
13697 -	-		-	14861 -	-		-	0	2	2
13720	13718	13720	-	13851 -	-		-	1	1	2
13720	13720	13721	+	13785	13785	13786	+	1	1	2
13737	13737	13741	-	15101 -	-		-	2	0	2
13768 -	-		-	13805 -	-		-	0	2	2
13831	13831	13832	-	14222 -	-		-	2	0	2
13837 -	-		+	13933 -	-		+	1	1	2
13839 -	-		-	14313 -	-		-	1	1	2
13862	13861	13863	-	14509 -	-		-	1	1	2
13862	13861	13863	-	14780 -	-		-	1	1	2
13961	13960	13965	-	14018 -	-		-	1	1	2
13961	13960	13965	-	14888 -	-		-	1	1	2
13969 -	-		+	14533 -	-		+	0	2	2
13979	13979	13980	-	14495	14495	14496	-	1	1	2
14006	14002	14009	+	14317	14317	14318	-	2	0	2
14006	14002	14009	+	14321 -	-		+	0	2	2
14015 -	-		-	14129 -	-		-	2	0	2
14134	14129	14134	-	14284	14284	14285	+	1	1	2
14150 -	-		+	14164 -	-		+	0	2	2
14166	14166	14170	-	15297 -	-		-	1	1	2
14173	14173	14174	-	14198	14198	14199	-	1	1	2
14184 -	-		-	14706 -	-		-	1	1	2
14184 -	-		-	14719 -	-		-	1	1	2
14200 -	-		-	14386 -	-		-	2	0	2
14228 -	-		-	14250 -	-		+	1	1	2
14294	14291	14294	-	14316	14313	14316	+	1	1	2
14316	14313	14318	-	14291 -	-		+	2	0	2
14316	14313	14318	-	14438	14436	14438	-	1	1	2
14316	14313	14318	-	14578 -	-		-	2	0	2
14324 -	-		-	14638 -	-		-	2	0	2
14339	14336	14339	-	14383 -	-		-	0	2	2
14339	14336	14339	-	14507 -	-		-	0	2	2
14359	14356	14364	-	14631	14631	14632	-	1	1	2
14398	14397	14398	+	14519 -	-		+	1	1	2
14420 -	-		-	14577 -	-		-	1	1	2
14449	14448	14449	-	14510 -	-		-	1	1	2
14457	14457	14458	+	14435 -	-		-	0	2	2
14464 -	-		+	14428 -	-		-	2	0	2
14482	14482	14487	-	15149	15149	15150	-	1	1	2
14490 -	-		-	15122 -	-		-	1	1	2
14497	14495	14497	+	14538	14536	14538	-	1	1	2
14515 -	-		+	14739 -	-		-	2	0	2
14520 -	-		+	14534 -	-		+	1	1	2
14565	14564	14566	-	14858 -	-		-	1	1	2
14574 -	-		+	14879 -	-		+	1	1	2
14586	14582	14586	+	14969 -	-		+	2	0	2
14596	14595	14597	+	14792 -	-		-	2	0	2
14603	14601	14603	+	15041	15039	15041	+	1	1	2
14699	14699	14701	-	14933	14933	14934	+	1	1	2
14751 -	-		-	14818 -	-		-	1	1	2
14756 -	-		-	14922 -	-		-	2	0	2
14770 -	-		+	15028 -	-		+	0	2	2
14800	14799	14801	+	14868 -	-		+	1	1	2
14800	14799	14801	+	15030 -	-		+	0	2	2
14807	14807	14809	+	14874 -	-		-	1	1	2
14880 -	-		-	15059 -	-		-	1	1	2
14912 -	-		+	14945 -	-		-	2	0	2
14938	14938	14941	-	15086	15083	15086	-	0	2	2
14950	14950	14953	+	14990 -	-		+	1	1	2
14950	14950	14953	+	15176 -	-		+	1	1	2
15007	15006	15011	+	15025	15025	15026	+	1	1	2
15007	15006	15011	+	15043	15043	15044	+	1	1	2
15020 -	-		+	15300 -	-		+	1	1	2
15165 -	-		+	15201 -	-		+	1	1	2
15177 -	-		+	15189 -	-		+	2	0	2
15234 -	-		+	15279 -	-		+	1	1	2
