# Supplementary material for: Identification and quantification of defective virus genomes in high throughput sequencing data using DVG-profiler, a novel post-sequence alignment processing algorithm
Source: PLoS One. 2019 May 17;14(5):e0216944. doi: 10.1371/journal.pone.0216944 (PMC6524942; doi:10.1371/journal.pone.0216944)
Supplement: S9 Table — (PDF) [file pone.0216944.s014.pdf]

Position (left)	Group start (left)	Group end (left)	Strandness (left)	Position (right)	Group start (right)	Group end (right)	Strandness (right)	Forward hits	Reverse hits	Fw and Rev
14869	14865	14872	-	15030	15026	15033	+	85859	109531	195390
14589	14584	14594	+	15046	15042	15052	+	41709	29277	70986
14947	14942	14953	-	15144	15140	15147	+	18641	14059	32700
13308	13303	13313	-	14863	14862	14866	+	12912	12947	25859
14869	14865	14872	-	15023	15020	15029	-	10746	5021	15767
14223	14219	14230	-	15165	15163	15170	+	10203	31410	41613
13908	13906	13912	-	15277	15276	15282	+	6375	11876	18251
13616	13612	13619	-	14663	14662	14666	+	6355	9794	16149
14342	14339	14346	-	15107	15104	15109	+	5717	3948	9665
13055	13051	13058	-	13442	13439	13446	+	3819	4684	8503
14456	14452	14461	-	14885	14882	14889	+	3580	3600	7180
14732	14727	14737	-	15025	15022	15028	+	3278	3460	6738
12591	12589	12594	-	14836	14834	14839	+	3036	1647	4683
14331	14328	14337	-	14428	14425	14433	+	2199	1518	3717
14761	14759	14764	-	14917	14916	14920	+	2038	1004	3042
14464	14463	14470	-	14891	14887	14895	-	2027	1290	3317
14869	14865	14872	-	15036	15034	15040	+	2000	1942	3942
13141	13139	13145	-	14880	14877	14883	+	1730	556	2286
13629	13625	13631	-	13775	13774	13778	+	1730	3173	4903
13479	13475	13484	-	15064	15063	15067	+	1711	1787	3498
13562	13556	13567	-	14651	14648	14655	+	1680	2494	4174
13316	13315	13320	-	15143	15141	15145	+	1664	1318	2982
14350	14348	14353	-	14770	14767	14773	+	1642	2822	4464
14238	14234	14240	-	15025	15024	15028	+	1626	4390	6016
13330	13324	13334	-	14156	14152	14157	+	1606	1292	2898
14666	14661	14670	-	14777	14772	14781	+	1552	1939	3491
13431	13428	13435	-	13720	13717	13723	+	1525	1363	2888
14456	14452	14461	-	14882	14880	14886	-	1473	1178	2651
14360	14355	14365	-	14873	14870	14875	+	1441	780	2221
14456	14452	14461	-	14877	14875	14877	-	1299	935	2234
13751	13746	13755	-	14324	14323	14326	+	1246	1822	3068
13462	13457	13464	-	13684	13682	13686	+	1192	979	2171
13549	13547	13553	-	14514	14510	14516	+	1007	1900	2907
6250	6249	6253	+	14476	14475	14479	+	976	183	1159
11435	11429	11438	-	15162	15162	15165	+	938	425	1363
13330	13324	13334	-	13837	13834	13840	+	924	1295	2219
11435	11429	11438	-	14306	14305	14310	+	877	586	1463
14960	14956	14964	+	15166	15162	15169	+	816	460	1276
277	272	280	-	15108	15106	15110	+	812	969	1781
13347	13344	13353	-	15239	15235	15241	+	647	524	1171
12903	12901	12905	-	14740	14739	14741	+	572	355	927
13479	13475	13484	-	14950	14946	14954	+	545	551	1096
13610	13605	13611	-	14660	14657	14660	-	532	1449	1981
3510	3506	3514	-	15098	15094	15101	-	468	173	641
5785	5783	5789	+	15175	15173	15178	+	467	602	1069
14108	14106	14112	-	14207	14207	14212	+	466	297	763
11823	11821	11826	-	13780	13777	13782	+	442	797	1239
13916	13916	13920	+	15284	15284	15288	+	434	515	949
14847	14843	14850	-	15025	15022	15029	-	408	197	605
12911	12906	12916	-	15044	15042	15048	+	380	666	1046
11228	11226	11234	-	14777	14776	14779	+	368	266	634
14725	14725	14726	-	15066	15065	15068	+	359	272	631
14859	14856	14862	-	15177	15177	15180	+	286	495	781
12101	12098	12104	-	12332	12332	12333	+	282	324	606
13967	13964	13968	-	15008	15007	15009	+	267	127	394
15025	15021	15029	+	15048	15048	15052	-	262	5	267
4176	4175	4179	+	13740	13740	13743	+	250	503	753
13498	13495	13504	-	14633	14633	14635	+	248	363	611
14489	14484	14493	-	14678	14675	14681	+	247	221	468
13549	13547	13553	-	14559	14559	14561	+	245	430	675
13498	13495	13504	-	14739	14739	14741	+	243	677	920
12921	12919	12925	-	14968	14966	14971	+	236	570	806
14947	14942	14953	-	15138	15136	15139	+	236	131	367
14666	14661	14670	-	14926	14923	14929	+	233	451	684
10847	10844	10850	-	13370	13370	13373	+	230	196	426
13094	13088	13097	-	13469	13467	13471	+	219	272	491
14786	14782	14790	-	15167	15163	15170	+	213	399	612
14443	14442	14447	-	15066	15065	15070	+	205	230	435
12291	12287	12293	-	14981	14981	14982	+	204	120	324
14767	14765	14770	-	14917	14917	14919	+	202	94	296
15116	15114	15119	+	15143	15139	15145	-	198	0	198
13863	13861	13866	-	14987	14987	14990	+	196	364	560
14370	14366	14373	+	14885	14885	14887	+	195	93	288
14307	14302	14308	-	14932	14932	14935	+	193	234	427
12121	12119	12122	-	14406	-	-	+	192	211	403
14164	14161	14168	-	14739	14735	14742	+	190	542	732
14703	14701	14708	-	14800	14797	14804	+	188	135	323
13265	13261	13268	-	13627	13626	13629	+	187	198	385
11857	11857	11861	-	15222	15218	15222	+	182	61	243
14869	14865	14872	-	15015	15013	15018	-	177	62	239
13562	13556	13567	-	14677	14676	14679	+	169	254	423
14518	14510	14522	-	14967	14966	14969	+	169	135	304
12717	12714	12721	-	13372	13372	13375	+	168	271	439
14370	14366	14373	+	14693	14693	14694	+	168	73	241
14732	14727	14737	-	15025	15025	15029	-	165	66	231
14951	14948	14955	+	15157	15157	15161	+	162	80	242
12481	12478	12484	-	14148	14148	14150	+	158	157	315
12797	12795	12797	-	14948	14948	14950	+	153	245	398
13587	13586	13589	-	14608	14606	14611	+	152	319	471
14377	14372	14382	-	15012	15009	15013	+	148	64	212

11708	11706	11711	-	14207	14207	14209	+	147	29	176
291	290	292	-	14452 -	-		-	146	35	181
14489	14484	14493	-	14899	14899	14900	-	143	137	280
291	290	292	-	15094	15091	15095	+	141	52	193
814	812	818	+	13432	13431	13436	+	139	259	398
13252	13247	13256	-	15039	15039	15041	+	135	377	512
5078	5074	5082	+	15083	15079	15088	-	127	28	155
13359	13355	13363	-	15255	15252	15255	-	126	11	137
13555	13554	13555	-	14665	14665	14667	+	125	175	300
12553	12549	12558	-	14196	14196	14200	+	122	136	258
13431	13428	13435	-	13706	13706	13708	-	115	11	126
14154	14150	14156	-	14377	14375	14381	+	112	157	269
14869	14865	14872	-	15043	15041	15046	+	112	109	221
2426	2424	2429	+	13755	13755	13758	+	107	139	246
13298	13296	13302	-	14255	14255	14257	+	107	58	165
2366	2363	2370	-	14411	14409	14411	+	104	103	207
4167	4164	4168	-	14590	14589	14590	+	104	52	156
13751	13746	13755	-	14551	14551	14555	+	101	111	212
13272	13269	13276	-	14359	14356	14361	+	98	110	208
14300	14298	14301	-	14446	14445	14447	+	98	135	233
14456	14452	14461	-	15030	15029	15033	+	97	77	174
13347	13344	13353	-	14273 -	-		+	95	142	237
13885	13880	13885	-	14370	14370	14373	+	95	171	266
12334	12331	12338	-	14577	14576	14578	+	93	22	115
13246	13240	13246	-	14247	14247	14249	+	92	46	138
13770	13764	13773	-	14533	14532	14534	+	89	129	218
14423	14418	14424	-	15021	15021	15025	+	87	57	144
14559	14555	14563	-	15005	15005	15008	+	87	126	213
13416	13413	13420	-	14529 -	-		+	85	24	109
13947	13941	13951	-	14134 -	-		+	84	35	119
13544	13539	13545	-	14509	14505	14510	-	83	332	415
3227	3226	3232	+	14501 -	-		+	82	56	138
5033	5032	5033	+	12137	12136	12137	+	82	46	128
14864	14863	14864	-	15197 -	-		+	78	56	134
13431	13428	13435	-	13697	13697	13699	-	77	2	79
14518	14510	14522	-	14929	14927	14930	+	76	61	137
2131	2126	2135	+	10975	10973	10978	+	75	134	209
13416	13413	13420	-	13665	13663	13669	+	75	23	98
12334	12331	12338	-	14795	14793	14795	+	74	43	117
14578	14576	14582	-	15153	15153	15154	+	74	124	198
3198	3196	3199	+	12186	12185	12186	+	73	48	121
14456	14452	14461	-	14791 -	-		-	73	38	111
1900	1896	1904	+	14927	14923	14927	+	70	76	146
12577	12573	12581	-	14136 -	-		+	70	32	102
12712	12709	12712	-	14403 -	-		+	70	65	135
11994	11992	11997	-	14467	14467	14468	+	68	90	158
13104	13100	13107	-	14941 -	-		+	68	46	114
11446	11442	11450	+	15175	15171	15176	+	67	60	127
13298	13296	13302	-	14334 -	-		+	67	57	124
14248	14244	14252	-	14289	14289	14293	+	67	57	124
14877	14873	14878	-	15094	15093	15096	+	67	34	101
13779	13776	13783	-	15033	15032	15034	+	66	51	117
1944	1943	1945	+	14652	14651	14654	+	65	19	84
12321	12318	12325	-	14584	14581	14584	+	65	11	76
13639	13637	13642	-	14454 -	-		+	64	105	169
14001	13996	14005	-	14693	14692	14693	+	64	50	114
14079	14079	14083	-	14866	14864	14866	+	63	41	104
14324	14319	14326	-	14837	14837	14838	+	63	63	126
10258	10256	10261	-	14175	14172	14175	+	62	36	98
13668 -	-		-	14187 -	-		-	62	1	63
15092	15087	15096	+	15161	15160	15165	-	60	2	62
2627	2627	2631	+	14993 -	-		+	58	30	88
12481	12478	12484	-	14136	14136	14138	+	56	56	112
12604	12600	12607	-	13952	13952	13953	+	56	56	112
13908	13906	13912	-	15116	15113	15116	+	56	36	92
9873	9870	9873	-	13952	13952	13954	+	55	113	168
14607	14602	14610	-	14967	14965	14968	+	55	63	118
1932	1927	1934	+	14681	14677	14681	+	54	11	65
12611	12608	12614	-	13431	13430	13431	+	54	49	103
11847	11843	11851	-	14698 -	-		+	53	42	95
12911	12906	12916	-	14036 -	-		+	52	55	107
14586	14584	14590	-	14647	14646	14648	+	52	18	70
12641	12636	12645	-	13605	13605	13607	+	51	48	99
14877	14873	14878	-	15032	15029	15036	+	51	50	101
2179	2177	2183	+	12372	12372	12375	+	50	58	108
14423	14418	14424	-	15094	15093	15094	+	50	29	79
2298	2297	2301	+	14178	14177	14178	+	49	40	89
4188	4186	4189	-	14567	14566	14567	+	49	22	71
13936	13932	13940	-	14949	14949	14953	+	49	37	86
8082	8082	8083	+	14811	14811	14812	+	48	18	66
12577	12573	12581	-	15186	15186	15188	+	48	50	98
13992	13988	13993	+	15134	15133	15134	+	48	25	73
14079	14079	14083	-	14614	14613	14614	+	48	28	76
14120	14115	14126	-	14464	14463	14464	+	48	40	88
14462	14458	14465	+	14889	14885	14891	+	48	30	78
14869	14865	14872	-	15052	15050	15054	-	48	37	85
15015	15011	15019	+	15038	15038	15041	-	48	0	48
7592	7592	7594	-	14561 -	-		+	47	55	102
8969	8965	8971	+	14797	14796	14797	-	47	16	63
10406	10403	10410	-	13044	13041	13045	+	47	44	91
15143	15140	15146	+	15156 -	-		+	47	14	61

2515	2512	2515	+	13987	13986	13987	+	46	40	86
8931	8931	8935	+	13497	-	-	+	46	91	137
13330	13324	13334	-	13808	13808	13812	-	46	15	61
14267	14263	14270	-	14408	14407	14411	+	46	78	124
14350	14348	14353	-	15110	15110	15112	-	46	34	80
13308	13303	13313	-	14360	14357	14360	+	45	53	98
14854	14851	14855	-	15045	15044	15046	+	45	60	105
10543	10541	10544	-	14634	-	-	+	44	41	85
12113	12109	12116	-	14450	14447	14450	+	43	41	84
12518	12512	12523	-	15185	15185	15186	+	43	66	109
13623	13620	13623	-	14671	-	-	-	43	0	43
13673	13669	13675	-	14421	14421	14424	+	43	64	107
13751	13746	13755	-	14329	14327	14333	+	43	51	94
11380	-	-	-	14896	-	-	+	42	23	65
14916	14912	14918	-	15043	15043	15047	-	42	0	42
7365	-	-	-	14283	-	-	+	41	55	96
8952	8948	8955	-	14814	14813	14814	+	41	0	41
12577	12573	12581	-	14576	-	-	+	41	31	72
13044	13040	13046	-	13609	-	-	+	41	99	140
15037	15033	15040	+	15072	15072	15073	-	41	3	44
11631	-	-	+	14357	-	-	+	40	39	79
12101	12098	12104	-	14401	14400	14401	+	40	89	129
13111	13108	13111	-	14370	-	-	+	40	27	67
13533	13529	13537	-	14457	14454	14457	+	40	55	95
13663	13657	13666	-	14179	14178	14180	+	40	134	174
13914	13913	13917	-	15103	-	-	+	40	28	68
4181	4180	4184	+	13739	13738	13739	-	39	17	56
12970	12967	12973	-	14750	14750	14753	+	39	81	120
13876	13872	13879	-	14379	14379	14381	+	39	65	104
14331	14328	14337	-	14593	14593	14596	+	39	20	59
14489	14484	14493	-	15249	15249	15250	+	39	109	148
10694	10690	10697	-	15008	-	-	+	38	12	50
12341	12340	12346	-	13947	13946	13948	+	38	36	74
13779	13776	13783	-	15204	-	-	+	38	26	64
15047	15043	15050	+	15114	15113	15117	-	38	20	58
12956	12952	12960	-	14369	-	-	+	37	60	97
13394	13392	13395	-	14177	14176	14179	+	37	24	61
13408	13404	13411	-	14027	14027	14028	+	37	17	54
1959	1953	1963	+	11517	-	-	+	36	12	48
12075	12075	12078	-	14560	-	-	+	36	32	68
14174	14174	14180	-	14752	14752	14755	+	36	79	115
14761	14759	14764	-	15025	15025	15028	+	36	48	84
991	988	993	+	14131	-	-	+	35	32	67
1959	1953	1963	+	13014	13013	13014	+	35	12	47
7581	7581	7582	+	13545	13545	13547	+	35	20	55
13330	13324	13334	-	13772	-	-	-	35	14	49
13720	13718	13723	-	13809	13808	13815	+	35	34	69
14607	14602	14610	-	14844	-	-	+	35	0	35
3493	3493	3497	-	15080	15080	15085	-	34	23	57
4591	4588	4592	+	13645	13645	13646	+	34	33	67
13700	13696	13701	-	14148	-	-	+	34	17	51
12732	12727	12735	-	14540	-	-	+	33	21	54
13055	13051	13058	-	14885	-	-	+	33	0	33
13562	13556	13567	-	14648	14646	14650	-	33	106	139
14019	14015	14022	-	14332	14332	14333	+	33	55	88
13741	13738	13745	-	14730	14729	14730	+	32	21	53
14019	14015	14022	-	14284	14284	14287	+	32	36	68
14377	14372	14382	-	14687	14687	14689	+	32	19	51
2526	2521	2527	+	13524	-	-	+	31	27	58
12583	12583	12586	-	14154	14151	14154	+	31	7	38
13562	13556	13567	-	14638	14638	14639	-	31	108	139
14694	14690	14698	-	14852	14851	14853	+	31	32	63
14847	14843	14850	-	15039	15037	15039	-	31	12	43
14859	14856	14862	-	15033	15031	15035	+	31	59	90
277	272	280	-	14333	14331	14333	+	30	27	57
1621	1619	1625	+	14102	-	-	-	30	42	72
13663	13657	13666	-	14465	14465	14467	+	29	43	72
14284	14280	14285	-	14895	-	-	+	29	27	56
14947	14942	14953	-	15133	15133	15134	+	29	16	45
1924	1922	1925	+	12988	-	-	+	28	13	41
2173	2171	2176	+	15240	15240	15242	-	28	274	302
4434	4431	4434	-	14871	14868	14871	+	28	34	62
12903	12901	12905	-	13743	-	-	+	28	33	61
13893	13893	13895	-	14171	-	-	+	28	28	56
14589	14584	14594	+	15025	-	-	-	28	7	35
14859	14856	14862	-	14932	14930	14933	+	28	20	48
15217	15215	15222	+	15222	-	-	+	28	15	43
10278	10276	10282	-	13627	-	-	+	27	21	48
12240	12236	12241	-	14580	14580	14582	+	27	29	56
13385	13381	13388	-	14384	14384	14385	+	27	50	77
13479	13475	13484	-	14932	14932	14933	+	27	39	66
14238	14234	14240	-	15015	-	-	-	27	9	36
14877	14873	14878	-	15046	15044	15047	-	27	64	91
655	654	659	-	11857	11856	11857	+	26	4	30
2190	2186	2194	+	13081	-	-	+	26	40	66
12896	12896	12898	-	13079	-	-	+	26	18	44
13462	13457	13464	-	14668	-	-	+	26	1	27
2788	2784	2791	+	14254	-	-	+	25	9	34
4599	4596	4603	+	14104	14103	14104	+	25	17	42
12113	12109	12116	-	14591	-	-	+	25	24	49
12257	12252	12259	-	14846	14846	14847	+	25	18	43

12433	12429	12439	-	14077	-	-	+	25	54	79
12717	12714	12721	-	14704	-	-	+	25	22	47
13498	13495	13504	-	15080	-	-	-	25	3	28
13720	13718	13723	-	14266	14265	14266	+	25	9	34
14369	14367	14371	-	15008	15007	15008	+	25	13	38
14725	14725	14726	-	15024	-	-	+	25	39	64
14748	14743	14752	-	15187	-	-	+	25	12	37
14877	14875	14882	+	15039	15039	15041	+	25	31	56
1038	1035	1041	+	14352	14351	14352	+	24	30	54
5214	5212	5215	-	14975	14973	14976	-	24	103	127
13293	13291	13294	-	14464	-	-	+	24	8	32
13365	13364	13367	-	14536	-	-	+	24	19	43
13408	13404	13411	-	14524	14523	14524	+	24	12	36
13456	-	-	-	14571	-	-	+	24	8	32
13579	13575	13584	-	14605	14604	14609	-	24	40	64
13579	13575	13584	-	14672	-	-	-	24	1	25
14324	14319	14326	-	14987	14987	14989	+	24	25	49
14902	14899	14908	-	15044	15040	15047	-	24	7	31
15063	15058	15066	+	15081	15076	15083	-	24	1	25
588	588	589	+	14791	-	-	+	23	20	43
12307	12303	12310	-	14272	14268	14272	+	23	14	37
13416	13413	13420	-	14013	14012	14016	+	23	15	38
13462	13457	13464	-	13668	13667	13671	-	23	3	26
13709	13703	13713	-	14437	14437	14438	+	23	12	35
14331	14328	14337	-	14418	14415	14422	-	23	15	38
14350	14348	14353	-	14930	-	-	+	23	26	49
15072	15068	15075	-	15151	15150	15154	-	23	0	23
15086	15082	15086	+	15152	15152	15155	-	23	1	24
2016	2013	2019	+	14706	14706	14707	+	22	14	36
2298	2297	2301	+	14730	-	-	+	22	24	46
10300	10296	10301	-	14955	14955	14956	+	22	12	34
12161	12159	12162	-	14564	14564	14565	+	22	41	63
12240	12236	12241	-	14660	-	-	+	22	20	42
12518	12512	12523	-	13948	-	-	+	22	27	49
13187	13182	13188	-	14498	14498	14501	+	22	7	29
13610	13605	13611	-	14677	14677	14681	+	22	25	47
13810	13807	13815	-	14859	-	-	+	22	18	40
14464	14463	14470	-	14899	14896	14899	-	22	24	46
14870	14866	14873	+	14895	14893	14895	-	22	4	26
14877	14873	14878	-	15191	-	-	-	22	80	102
5069	5064	5072	+	15093	15090	15098	-	21	16	37
10543	10541	10544	-	14693	14692	14693	+	21	11	32
12433	12429	12439	-	15092	-	-	+	21	46	67
12481	12478	12484	-	14303	-	-	+	21	36	57
13316	13315	13320	-	14843	14843	14845	+	21	18	39
13741	13738	13745	-	14239	14238	14240	+	21	8	29
14019	14015	14022	-	14416	14416	14420	+	21	28	49
14947	14942	14953	-	15157	15157	15158	-	21	36	57
15025	15021	15029	+	15138	15134	15140	-	21	2	23
3272	3270	3275	+	15092	15091	15092	+	20	9	29
3317	3313	3317	+	13295	-	-	+	20	3	23
11164	11160	11164	-	15087	-	-	+	20	14	34
11665	11664	11669	-	14676	14676	14680	+	20	13	33
12547	12544	12547	-	14238	-	-	+	20	13	33
13298	13296	13302	-	13432	13432	13434	+	20	15	35
13451	13448	13454	-	14611	-	-	+	20	8	28
13451	13448	13454	-	14714	-	-	+	20	9	29
13720	13718	13723	-	14189	-	-	+	20	6	26
13870	13867	13871	-	14865	-	-	+	20	19	39
14035	14032	14038	-	14700	-	-	+	20	12	32
14761	14759	14764	-	14924	14922	14924	+	20	16	36
14761	14759	14764	-	15062	15061	15062	+	20	9	29
15032	15030	15032	+	15156	-	-	-	20	0	20
20	17	20	-	14315	14312	14315	+	19	14	33
8958	8958	8959	-	14808	14807	14808	+	19	34	53
9706	-	-	-	14517	-	-	+	19	3	22
10885	10882	10887	-	14226	-	-	+	19	2	21
12199	12194	12203	-	14586	14586	14588	+	19	104	123
12732	12727	12735	-	15030	-	-	+	19	0	19
13317	13315	13320	+	14872	14871	14872	+	19	15	34
13431	13428	13435	-	14991	-	-	+	19	1	20
13473	13468	13474	-	15040	15037	15042	-	19	27	46
13728	13728	13731	-	15095	-	-	+	19	7	26
13848	13843	13849	-	15012	-	-	+	19	0	19
13876	13872	13879	-	14995	-	-	-	19	15	34
13972	13971	13974	-	14385	14385	14386	+	19	21	40
14202	14198	14205	-	15128	15128	15130	+	19	38	57
14580	14578	14583	+	15051	15051	15052	-	19	37	56
14678	14675	14679	-	15092	-	-	+	19	24	43
266	262	270	-	14331	14331	14333	+	18	19	37
2539	2539	2543	+	14293	-	-	+	18	15	33
3860	3860	3864	+	13346	-	-	+	18	7	25
9945	9942	9948	-	14112	14108	14116	-	18	28	46
12094	12090	12095	-	12445	-	-	+	18	10	28
12121	12119	12122	-	14436	14436	14438	+	18	26	44
12978	12976	12980	+	14053	-	-	+	18	15	33
13265	13264	13266	+	15052	15051	15053	+	18	9	27
13317	13315	13320	+	14877	14877	14879	+	18	19	37
13947	13941	13951	-	14634	-	-	+	18	8	26
13953	13952	13956	-	14284	14281	14287	+	18	20	38
193	192	193	+	15006	15005	15006	+	17	0	17

1140	1138	1143	+	14676	14676	14677	+	17	23	40
1908	1905	1911	-	15364	15360	15364	-	17	4	21
4031	4030	4035	+	12407	12406	12407	+	17	6	23
12187	12184	12190	-	13620	13619	13622	+	17	8	25
12291	12287	12293	-	13384	-	-	+	17	14	31
13246	13240	13246	-	13526	13526	13527	+	17	27	44
13728	13728	13731	-	14089	14087	14089	+	17	7	24
13751	13746	13755	-	14436	-	-	+	17	0	17
13982	13978	13982	-	13985	-	-	+	17	0	17
14120	14115	14126	-	14216	14215	14219	-	17	18	35
14207	14207	14211	+	15180	15176	15180	-	17	42	59
14360	14355	14365	-	14879	14875	14880	-	17	25	42
14456	14452	14461	-	14836	14836	14838	+	17	25	42
14456	14452	14461	-	14891	-	-	-	17	0	17
14501	14498	14507	-	14852	14850	14852	+	17	6	23
14589	14584	14594	+	15042	15039	15042	-	17	9	26
14596	14595	14599	+	14792	-	-	-	17	0	17
14638	14634	14642	+	14662	-	-	-	17	0	17
14810	14810	14811	+	14878	14877	14878	-	17	1	18
14877	14875	14882	+	15029	15029	15032	+	17	47	64
14933	14932	14936	-	15078	15078	15081	-	17	68	85
1802	1801	1806	+	13742	13741	13742	+	16	27	43
2680	2679	2680	+	12467	-	-	+	16	22	38
7832	-	-	+	14504	-	-	+	16	7	23
8990	8987	8990	-	14345	-	-	+	16	15	31
11274	11271	11277	-	15023	15023	15026	+	16	19	35
12026	12024	12030	-	14369	-	-	+	16	23	39
12433	12429	12439	-	13497	-	-	+	16	24	40
12449	12445	12449	+	14100	-	-	+	16	1	17
12496	12494	12496	-	13785	13785	13786	+	16	28	44
12604	12600	12607	-	14916	14915	14916	+	16	3	19
13278	13277	13282	-	14035	-	-	+	16	24	40
13408	13404	13411	-	15217	-	-	+	16	0	16
13562	13556	13567	-	14571	14570	14575	-	16	9	25
13988	13984	13992	-	15129	15126	15129	-	16	22	38
14019	14015	14022	-	14758	-	-	+	16	28	44
14025	14023	14028	-	14355	14352	14356	+	16	32	48
14138	14134	14142	-	14951	-	-	+	16	23	39
14578	14576	14582	-	14891	-	-	+	16	31	47
14877	14873	14878	-	15039	15035	15039	-	16	59	75
15109	15108	15112	+	15152	15147	15152	-	16	3	19
15189	15184	15192	+	15204	15201	15204	+	16	5	21
603	598	604	+	14571	14570	14571	+	15	4	19
1972	1967	1976	+	13356	13355	13356	+	15	7	22
4300	4297	4303	+	13030	13028	13030	+	15	8	23
4476	4476	4481	+	14874	14874	14875	+	15	7	22
10955	10952	10955	-	14282	-	-	+	15	15	30
11195	11190	11195	-	14522	-	-	+	15	5	20
11945	11944	11947	-	13364	13362	13365	+	15	29	44
12321	12318	12325	-	13774	-	-	+	15	11	26
12407	12403	12410	-	14487	-	-	+	15	8	23
12433	12429	12439	-	13750	13750	13751	+	15	11	26
12467	12462	12470	-	14570	-	-	+	15	14	29
13036	-	-	-	13413	-	-	+	15	11	26
13149	13146	13152	-	13944	-	-	+	15	17	32
13462	13457	13464	-	14164	-	-	+	15	4	19
13462	13457	13464	-	14576	-	-	+	15	1	16
13479	13475	13484	-	14315	14314	14315	+	15	13	28
13544	13539	13545	-	14647	14644	14647	-	15	0	15
13566	13565	13568	+	15066	15066	15067	+	15	44	59
13673	13669	13675	-	14672	-	-	-	15	8	23
13855	13850	13859	-	14255	14251	14255	+	15	20	35
13919	13918	13921	-	14667	14666	14667	+	15	13	28
13988	13984	13992	-	14290	14287	14290	+	15	16	31
14294	14291	14295	-	15180	15177	15180	-	15	21	36
14360	14355	14365	-	14447	-	-	+	15	7	22
14479	14475	14482	-	14983	14980	14983	+	15	5	20
14518	14510	14522	-	15063	-	-	+	15	6	21
14847	14843	14850	-	15044	15044	15045	+	15	3	18
1959	1953	1963	+	14537	14536	14537	+	14	6	20
2037	2034	2038	+	13095	-	-	+	14	23	37
2397	2396	2401	+	11823	11823	11824	+	14	10	24
2493	2487	2493	+	2600	-	-	+	14	2	16
2861	2861	2862	+	14207	-	-	+	14	16	30
2879	2879	2883	-	14950	14950	14951	-	14	14	28
3166	3165	3166	+	13810	-	-	+	14	59	73
3902	-	-	+	13550	-	-	+	14	15	29
4599	4596	4603	+	14370	14368	14371	+	14	7	21
6936	6936	6939	+	15368	-	-	-	14	0	14
12233	12229	12233	-	13346	-	-	+	14	7	21
12911	12906	12916	-	13372	13372	13374	+	14	17	31
13149	13146	13152	-	14631	-	-	+	14	6	20
13317	13315	13320	+	14843	14843	14845	-	14	4	18
13431	13428	13435	-	14763	14763	14764	+	14	11	25
13663	13657	13666	-	14662	14662	14663	+	14	19	33
13715	13714	13717	-	14678	14678	14680	+	14	8	22
13770	13764	13773	-	14800	14800	14801	+	14	46	60
14012	14008	14013	-	14994	-	-	+	14	6	20
14159	14157	14160	-	14388	-	-	-	14	0	14
14622	14619	14626	-	15043	15043	15044	+	14	40	54
14892	14889	14895	+	15030	15030	15032	+	14	13	27

14902	14899	14908	-	14996	14995	14996	-	14	1	15
14964	14962	14968	-	15153	15152	15154	-	14	24	38
655	654	659	-	14868	14868	14871	+	13	3	16
764	760	765	+	14539	-	-	+	13	22	35
1852	1850	1856	+	13708	-	-	+	13	20	33
3184	3180	3188	+	13667	-	-	+	13	8	21
4181	4180	4184	+	14575	14573	14576	-	13	204	217
4749	4748	4753	+	9165	-	-	+	13	13	26
8875	8875	8876	-	14148	-	-	+	13	16	29
9618	9614	9619	-	14269	-	-	+	13	7	20
10406	10403	10410	-	14699	14697	14699	+	13	8	21
11274	11271	11277	-	12187	-	-	+	13	8	21
11465	11463	11469	-	15080	15077	15080	+	13	4	17
11542	11542	11543	-	14096	-	-	+	13	3	16
12094	12090	12095	-	13978	-	-	+	13	17	30
12568	12565	12568	-	14482	-	-	+	13	6	19
12577	12573	12581	-	13098	-	-	+	13	5	18
12732	12727	12735	-	14882	-	-	+	13	23	36
13149	13146	13152	-	14624	-	-	+	13	5	18
13265	13261	13268	-	13565	13565	13568	+	13	33	46
13330	13324	13334	-	15185	15185	15188	+	13	14	27
13347	13344	13353	-	14159	-	-	-	13	10	23
13555	13554	13555	-	14660	-	-	-	13	30	43
13678	13676	13680	-	14191	-	-	-	13	9	22
13810	13807	13815	-	14578	14575	14578	+	13	10	23
13936	13932	13940	-	14910	14909	14910	+	13	5	18
14202	14198	14205	-	14693	14692	14695	-	13	4	17
14360	14355	14365	-	14770	14766	14773	+	13	24	37
14360	14355	14365	-	14993	-	-	+	13	2	15
14435	14434	14439	-	14507	14505	14507	-	13	0	13
14847	14843	14850	-	14975	-	-	-	13	0	13
15102	15098	15105	+	15145	-	-	-	13	0	13
15189	15184	15192	+	15205	15201	15205	-	13	0	13
3215	3215	3218	-	3340	3340	3342	-	12	13	25
3860	3860	3864	+	13229	-	-	+	12	0	12
4186	4185	4190	+	14570	-	-	-	12	0	12
5773	-	-	-	15182	-	-	+	12	0	12
8409	-	-	+	14247	-	-	+	12	4	16
8998	8996	8999	-	14163	14163	14165	+	12	23	35
10485	10481	10486	-	14968	-	-	+	12	25	37
10694	10690	10697	-	14876	14873	14876	+	12	125	137
11641	11638	11644	-	13692	-	-	+	12	15	27
12101	12098	12104	-	13634	13634	13635	+	12	11	23
12676	12671	12678	-	13593	-	-	+	12	12	24
13000	12995	13002	-	15130	-	-	+	12	6	18
13007	13005	13008	-	13058	13058	13059	+	12	2	14
13013	13009	13017	-	13229	-	-	+	12	9	21
13055	13051	13058	-	14634	14633	14634	+	12	7	19
13117	13117	13121	-	14665	-	-	+	12	7	19
13308	13303	13313	-	14284	-	-	+	12	20	32
13308	13303	13313	-	14815	14814	14815	-	12	56	68
13479	13475	13484	-	14537	14537	14539	+	12	25	37
13587	13586	13589	-	13888	-	-	+	12	11	23
13898	13898	13900	-	13986	-	-	+	12	1	13
14238	14234	14240	-	15170	-	-	-	12	28	40
14239	14238	14242	+	15153	-	-	-	12	10	22
14307	14302	14308	-	15268	-	-	+	12	11	23
14403	14399	14406	-	15064	15064	15066	+	12	15	27
14435	14434	14439	-	14690	14688	14690	+	12	10	22
14540	14536	14541	-	14640	-	-	-	12	0	12
14732	14727	14737	-	15032	-	-	-	12	0	12
14847	14843	14850	-	15099	15097	15099	+	12	7	19
14854	14851	14855	-	15117	15115	15117	-	12	32	44
14978	14974	14978	+	15048	-	-	-	12	6	18
15086	15082	15086	+	15103	15103	15107	-	12	1	13
15086	15082	15086	+	15114	15114	15117	-	12	0	12
242	240	246	+	12230	-	-	+	11	14	25
544	544	549	+	15142	-	-	+	11	11	22
715	712	716	+	15217	-	-	+	11	12	23
1052	1049	1056	+	12723	-	-	+	11	7	18
1448	1446	1452	+	6702	6701	6706	+	11	0	11
1869	1869	1871	+	12231	-	-	+	11	0	11
1951	1948	1951	+	14761	14759	14761	+	11	9	20
2435	2431	2436	+	14837	14836	14837	+	11	6	17
2546	2546	2548	+	12872	-	-	+	11	6	17
2768	2764	2771	+	13423	-	-	+	11	10	21
3486	3482	3489	-	15073	15069	15075	-	11	102	113
9078	-	-	-	14137	-	-	+	11	4	15
9355	9354	9359	-	14610	14606	14610	+	11	4	15
12167	12167	12171	-	14707	-	-	+	11	10	21
12187	12184	12190	-	14266	14265	14266	+	11	10	21
12187	12184	12190	-	15120	-	-	+	11	18	29
12445	12441	12448	-	14508	-	-	+	11	22	33
12676	12671	12678	-	14841	-	-	+	11	8	19
12741	12736	12744	-	13696	-	-	+	11	21	32
12928	12926	12931	-	14824	-	-	+	11	34	45
13055	13051	13058	-	13379	13379	13381	+	11	7	18
13055	13051	13058	-	14566	-	-	+	11	6	17
13111	13108	13111	-	13603	13603	13604	+	11	12	23
13127	13124	13129	-	14294	14293	14294	+	11	6	17
13339	13335	13341	-	15131	15131	15132	+	11	12	23

13347	13344	13353	-	14688	14687	14688	+	11	11	22
13516	13516	13517	-	14715	14715	14717	+	11	38	49
13645	13644	13649	-	14214	-	-	+	11	15	26
14360	14355	14365	-	15015	15012	15015	+	11	7	18
14437	14432	14442	+	14457	-	-	-	11	0	11
14456	14452	14461	-	14731	14731	14732	+	11	16	27
14479	14475	14482	-	14923	-	-	+	11	15	26
14540	14536	14541	-	15116	-	-	+	11	4	15
14565	14564	14568	-	14637	-	-	+	11	4	15
14578	14576	14582	-	15051	-	-	+	11	12	23
14638	14634	14642	+	14651	14649	14651	-	11	0	11
14847	14843	14850	-	15008	15004	15008	-	11	7	18
14869	14865	14872	-	15039	15037	15042	-	11	2	13
14869	14865	14872	-	15047	15045	15047	-	11	7	18
14877	14875	14882	+	14890	14890	14892	-	11	2	13
15037	15033	15040	+	15049	15048	15052	-	11	2	13
303	301	303	-	15079	15079	15081	+	10	12	22
448	447	448	+	14164	14164	14165	+	10	10	20
672	670	676	+	14154	14152	14155	+	10	4	14
715	712	716	+	14635	-	-	+	10	11	21
2075	2071	2076	+	14002	14000	14002	+	10	7	17
2499	2499	2502	+	2598	-	-	+	10	0	10
2875	2874	2878	+	14864	-	-	+	10	14	24
2891	2890	2891	+	14962	14961	14962	+	10	12	22
5408	5405	5409	+	13252	-	-	+	10	66	76
8140	8140	8144	-	8236	-	-	-	10	0	10
9107	9106	9110	-	14732	14729	14732	+	10	4	14
9562	9562	9564	-	14688	-	-	+	10	20	30
11117	11114	11117	-	13946	-	-	+	10	0	10
11920	11915	11924	-	15159	-	-	+	10	7	17
12577	12573	12581	-	13165	13163	13166	+	10	10	20
12936	12934	12936	-	13723	-	-	+	10	0	10
12970	12967	12973	-	15005	-	-	+	10	12	22
13149	13146	13152	-	13680	13680	13681	+	10	3	13
13246	13240	13246	-	14470	-	-	+	10	10	20
13252	13247	13256	-	15025	-	-	-	10	6	16
13695	13692	13695	-	14502	14500	14502	+	10	4	14
13728	13726	13732	+	13779	-	-	-	10	0	10
13947	13941	13951	-	14143	14143	14147	-	10	0	10
13947	13941	13951	-	14378	14374	14378	+	10	19	29
13988	13984	13992	-	15089	-	-	+	10	5	15
14001	13996	14005	-	14148	-	-	+	10	6	16
14307	14302	14308	-	14331	14331	14333	+	10	0	10
14360	14355	14365	-	14571	-	-	+	10	7	17
14364	14362	14364	+	14880	-	-	+	10	26	36
14452	14451	14455	+	14474	14473	14474	-	10	2	12
14678	14675	14679	-	15065	15065	15066	+	10	50	60
14741	14738	14742	-	14805	14804	14805	+	10	7	17
14847	14845	14852	+	15044	15044	15045	-	10	33	43
14864	14863	14864	-	15033	15028	15035	+	10	6	16
14902	14899	14908	-	14984	14984	14985	-	10	2	12
14933	14932	14936	-	15271	-	-	+	10	26	36
15170	15167	15174	+	15349	15347	15349	-	10	0	10
15212	15211	15212	+	15236	-	-	-	10	3	13
15284	15283	15287	-	15384	-	-	-	10	4	14
544	544	549	+	14476	-	-	+	9	5	14
2075	2071	2076	+	14298	14297	14298	+	9	7	16
2493	2487	2493	+	13768	-	-	+	9	0	9
2800	2799	2802	+	9953	9951	9953	+	9	1	10
2827	2826	2831	+	12709	-	-	+	9	2	11
2915	2912	2917	-	14818	14817	14821	+	9	75	84
8485	-	-	-	13380	-	-	+	9	1	10
9280	9280	9281	-	14775	-	-	+	9	3	12
9508	9508	9510	-	14631	-	-	+	9	4	13
9687	9687	9689	-	14398	14395	14398	+	9	4	13
10995	10993	10996	-	14166	14165	14166	+	9	6	15
11293	11290	11293	+	13945	13942	13945	+	9	3	12
11551	11548	11554	-	14340	-	-	+	9	7	16
12107	12105	12107	-	14372	-	-	+	9	0	9
12240	12236	12241	-	13770	-	-	+	9	1	10
12321	12318	12325	-	13681	13681	13682	+	9	5	14
12334	12331	12338	-	14259	-	-	+	9	3	12
12536	12535	12540	-	14835	-	-	+	9	10	19
12619	12615	12620	-	14778	14777	14778	+	9	7	16
12641	12636	12645	-	14144	-	-	+	9	8	17
12732	12727	12735	-	15004	-	-	+	9	10	19
12775	12772	12778	-	13461	-	-	+	9	0	9
12924	12921	12928	+	14828	14826	14829	-	9	11	20
12985	12982	12988	-	14032	-	-	-	9	13	22
13044	13040	13046	-	14156	14156	14157	+	9	4	13
13164	13161	13168	-	13548	-	-	+	9	5	14
13164	13161	13168	-	14959	-	-	+	9	3	12
13187	13182	13188	-	14991	-	-	+	9	11	20
13207	13203	13210	-	14746	14746	14748	+	9	28	37
13330	13324	13334	-	14437	-	-	+	9	17	26
13365	13364	13367	-	14015	-	-	+	9	5	14
13431	13428	13435	-	13723	-	-	-	9	0	9
13569	13568	13572	-	14566	14566	14567	-	9	11	20
13572	13572	13575	+	14450	-	-	+	9	1	10
13720	13718	13723	-	14715	-	-	+	9	6	15
13720	13718	13723	-	14969	-	-	+	9	7	16

13830	13826	13834	-	15091	-	-	+	9	7	16
13947	13941	13951	-	14667	14667	14668	+	9	4	13
13967	13964	13968	-	14488	14488	14489	+	9	4	13
14194	-	-	+	15329	15328	15329	-	9	3	12
14202	14198	14205	-	14897	14896	14897	+	9	8	17
14252	14249	14257	+	15042	15042	15046	+	9	15	24
14300	14298	14301	-	14951	-	-	+	9	16	25
14324	14319	14326	-	14966	14966	14967	+	9	9	18
14342	14339	14346	-	14509	-	-	+	9	6	15
14423	14418	14424	-	14526	14524	14526	+	9	0	9
14495	14494	14495	-	14680	14677	14680	+	9	11	20
14565	14564	14568	-	15057	-	-	+	9	26	35
14605	14600	14609	+	15092	15088	15095	+	9	10	19
14676	14672	14677	+	14940	14940	14944	+	9	19	28
14739	14738	14742	+	15041	-	-	+	9	16	25
14741	14738	14742	-	15041	15039	15044	-	9	2	11
14869	14865	14872	-	15057	15057	15061	-	9	6	15
14894	14890	14897	-	14981	-	-	-	9	0	9
15063	15058	15066	+	15149	-	-	-	9	0	9
74	72	78	+	14070	14064	14070	+	8	9	17
287	284	291	+	14672	14668	14673	+	8	11	19
424	424	428	+	14279	-	-	+	8	8	16
495	495	499	+	14781	14781	14782	+	8	9	17
638	634	638	+	14180	-	-	+	8	9	17
1140	1138	1143	+	14368	14368	14370	+	8	21	29
1621	1619	1625	+	1708	1708	1710	+	8	0	8
1891	1887	1894	+	13536	-	-	+	8	2	10
2006	2005	2006	+	12482	-	-	+	8	0	8
2043	2043	2047	+	14607	-	-	+	8	4	12
2118	2115	2121	+	12674	12673	12674	+	8	4	12
2343	2341	2345	+	14686	14685	14686	+	8	6	14
2435	2431	2436	+	13835	-	-	+	8	7	15
2583	2582	2583	+	12334	-	-	+	8	9	17
2867	2866	2870	+	12881	-	-	+	8	11	19
3258	3256	3259	+	12979	-	-	+	8	3	11
4599	4596	4603	+	15290	15290	15291	+	8	12	20
6257	-	-	+	14465	-	-	-	8	1	9
7502	7498	7506	-	14361	14360	14365	+	8	28	36
8320	8320	8321	+	15106	15105	15106	-	8	3	11
9061	9060	9065	-	14508	-	-	+	8	1	9
10167	10164	10168	-	14308	14307	14308	+	8	4	12
10823	10819	10827	-	13646	-	-	+	8	2	10
10935	10933	10935	-	12743	-	-	+	8	3	11
11369	11368	11371	-	15253	15251	15253	+	8	1	9
11576	11576	11578	-	14147	14145	14147	+	8	5	13
11920	11915	11924	-	14297	-	-	+	8	14	22
11994	11992	11997	-	13581	-	-	+	8	15	23
12053	12052	12054	-	14379	14377	14379	+	8	8	16
12352	12352	12356	-	13701	-	-	+	8	8	16
12884	12881	12886	-	13559	-	-	+	8	1	9
13141	13139	13145	-	14318	-	-	+	8	0	8
13141	13139	13145	-	14890	14886	14890	-	8	17	25
13207	13203	13210	-	13996	-	-	+	8	10	18
13308	13303	13313	-	14344	-	-	-	8	0	8
13308	13303	13313	-	14841	14837	14841	-	8	8	16
13330	13324	13334	-	14880	-	-	-	8	0	8
13394	13392	13395	-	14764	-	-	+	8	9	17
13399	13399	13401	-	14391	14391	14393	-	8	0	8
13416	13413	13420	-	14094	14094	14095	+	8	4	12
13462	13457	13464	-	14731	-	-	+	8	5	13
13507	13507	13512	-	13836	13836	13839	+	8	9	17
13517	13516	13518	+	14874	14873	14874	+	8	3	11
13533	13529	13537	-	14688	14688	14690	+	8	44	52
13562	13556	13567	-	14561	-	-	-	8	12	20
13678	13676	13680	-	15008	15007	15008	-	8	2	10
13700	13696	13701	-	14858	14858	14859	+	8	4	12
13936	13932	13940	-	14042	-	-	-	8	2	10
14079	14079	14083	-	14605	-	-	-	8	4	12
14238	14234	14240	-	14706	14706	14708	+	8	22	30
14254	14253	14258	-	14896	-	-	+	8	14	22
14331	14328	14337	-	14454	14450	14454	-	8	7	15
14437	14432	14442	+	14885	14885	14886	+	8	18	26
14501	14498	14507	-	15103	-	-	+	8	2	10
14501	14498	14505	+	14689	14689	14691	+	8	3	11
14571	14570	14572	-	14662	14660	14662	+	8	4	12
14601	-	-	-	14777	-	-	-	8	0	8
14612	14611	14615	-	15005	-	-	+	8	38	46
14694	14690	14698	-	15152	-	-	+	8	10	18
14732	14727	14737	-	15015	-	-	-	8	2	10
14847	14843	14850	-	14955	14955	14958	+	8	6	14
14869	14865	14872	-	15078	-	-	-	8	10	18
14905	14904	14907	+	14943	-	-	-	8	1	9
14968	14966	14971	+	15176	15176	15178	+	8	2	10
15170	15167	15174	+	15185	15181	15189	-	8	0	8
764	760	765	+	15033	-	-	+	7	10	17
1184	1180	1184	+	14732	14729	14732	+	7	10	17
1959	1953	1963	+	13715	-	-	+	7	7	14
1972	1967	1976	+	14357	-	-	+	7	10	17
2252	2252	2256	-	14654	-	-	-	7	4	11
2473	2470	2473	+	14581	14580	14581	+	7	5	12
2515	2512	2515	+	10795	-	-	+	7	1	8

2526	2521	2527	+	13302	-	-	+	7	4	11
2560	2558	2561	+	12802	-	-	+	7	0	7
3317	3313	3317	+	14886	-	-	+	7	6	13
3480	3476	3480	+	13764	-	-	+	7	11	18
7607	7603	7607	-	9517	-	-	+	7	70	77
8934	8932	8934	-	14491	-	-	+	7	11	18
10715	10712	10715	-	13737	-	-	+	7	8	15
10847	10844	10850	-	14717	-	-	+	7	1	8
11170	11170	11173	-	14409	-	-	+	7	5	12
11319	11317	11320	-	13175	-	-	+	7	17	24
11331	11331	11336	-	14783	-	-	+	7	3	10
11551	11548	11554	-	13311	-	-	+	7	0	7
11602	11597	11605	-	13948	-	-	+	7	7	14
11602	11597	11605	-	14412	-	-	+	7	6	13
11641	11638	11644	-	13580	13579	13580	+	7	13	20
12307	12303	12310	-	14982	-	-	+	7	1	8
12604	12600	12607	-	13972	-	-	+	7	6	13
12611	12608	12614	-	13318	13318	13319	+	7	1	8
12635	12632	12635	-	14774	14774	14775	+	7	4	11
12956	12952	12960	-	15077	-	-	+	7	65	72
12965	12964	12966	-	14757	14756	14757	+	7	18	25
12970	12967	12973	-	14815	14814	14815	+	7	6	13
12976	12975	12979	-	14019	14019	14020	+	7	21	28
13013	13009	13017	-	13148	13148	13149	+	7	10	17
13025	13023	13029	-	14024	-	-	+	7	7	14
13104	13100	13107	-	14933	-	-	-	7	1	8
13316	13315	13320	-	13484	-	-	+	7	7	14
13316	13315	13320	-	14879	14875	14879	-	7	9	16
13372	13372	13375	-	13431	13429	13431	+	7	5	12
13408	13404	13411	-	14344	-	-	+	7	5	12
13408	13404	13411	-	14401	14400	14401	+	7	1	8
13431	13428	13435	-	13715	13711	13718	-	7	0	7
13431	13428	13435	-	14632	14631	14632	+	7	4	11
13462	13457	13464	-	13834	-	-	+	7	3	10
13473	13468	13474	-	13684	-	-	+	7	5	12
13479	13475	13484	-	13942	13942	13944	+	7	9	16
13488	13485	13491	-	14882	-	-	-	7	2	9
13562	13556	13567	-	13963	13960	13963	+	7	28	35
13579	13575	13584	-	14569	14569	14573	-	7	1	8
13663	13657	13666	-	13793	13792	13793	-	7	2	9
13663	13657	13666	-	13798	-	-	-	7	36	43
13673	13669	13675	-	14187	-	-	+	7	8	15
13738	13738	13742	+	14324	-	-	+	7	6	13
13908	13906	13912	-	15202	-	-	+	7	9	16
13919	13918	13921	-	14660	-	-	-	7	6	13
13972	13971	13974	-	14487	-	-	+	7	5	12
14056	14054	14059	-	14679	-	-	+	7	4	11
14065	14062	14068	-	14809	-	-	+	7	2	9
14091	14089	14096	-	14255	-	-	-	7	0	7
14138	14134	14142	-	14759	14759	14760	+	7	11	18
14202	14198	14205	-	15266	-	-	+	7	16	23
14207	14207	14208	-	15174	-	-	+	7	0	7
14254	14253	14258	-	14266	14265	14266	+	7	35	42
14300	14298	14301	-	14435	-	-	-	7	6	13
14342	14339	14346	-	14978	-	-	-	7	1	8
14443	14442	14447	-	15037	-	-	-	7	7	14
14456	14452	14461	-	15043	-	-	+	7	5	12
14501	14498	14505	+	14551	-	-	-	7	0	7
14518	14510	14522	-	14914	-	-	+	7	5	12
14526	14524	14530	-	14751	-	-	+	7	4	11
14565	14564	14568	-	15237	-	-	+	7	5	12
14586	14584	14590	-	15221	-	-	+	7	5	12
14638	14638	14641	-	15153	15153	15154	+	7	17	24
14767	14765	14770	-	14924	-	-	+	7	4	11
14775	14775	14778	+	14932	14931	14932	+	7	18	25
14780	14780	14782	+	14929	-	-	+	7	0	7
14805	14800	14806	+	14883	14882	14886	-	7	1	8
14854	14851	14855	-	15025	15022	15027	-	7	4	11
14859	14856	14862	-	14989	14989	14990	-	7	3	10
14869	14865	14872	-	14978	14978	14979	-	7	4	11
14870	14866	14873	+	15200	15196	15200	+	7	3	10
14877	14875	14882	+	14807	-	-	-	7	0	7
14882	14879	14886	-	15042	15041	15042	-	7	8	15
15072	15068	15075	-	15125	15122	15129	-	7	29	36
15143	15140	15146	+	15116	15113	15119	-	7	129	136
15177	15176	15180	+	15189	-	-	+	7	0	7
124	121	127	+	14644	-	-	+	6	0	6
814	812	818	+	14252	-	-	+	6	6	12
849	848	849	+	9147	9146	9147	+	6	0	6
943	943	944	+	11928	-	-	+	6	1	7
957	954	960	+	14634	-	-	+	6	0	6
1082	1078	1082	+	13028	-	-	+	6	2	8
1099	1097	1103	+	14730	-	-	+	6	11	17
1852	1850	1856	+	14301	-	-	+	6	6	12
1900	1896	1904	+	3316	3316	3319	-	6	3	9
2016	2013	2019	+	13347	-	-	+	6	17	23
2155	2152	2155	+	13873	13871	13873	+	6	6	12
2173	2171	2176	+	12826	-	-	+	6	1	7
2244	-	-	-	14348	-	-	+	6	7	13
2426	2424	2429	+	14706	-	-	+	6	7	13
2435	2431	2436	+	14077	-	-	+	6	3	9

2565	2565	2566	+	14343	14343	14344	+	6	9	15
2788	2784	2791	+	11028	11027	11028	+	6	1	7
3258	3256	3259	+	12455	-	-	+	6	0	6
3341	3339	3345	+	8675	-	-	+	6	4	10
3570	-	-	+	13800	-	-	+	6	0	6
3581	3577	3581	+	12078	-	-	+	6	2	8
3731	3730	3731	+	13620	13619	13620	+	6	7	13
3771	3771	3774	+	13455	-	-	+	6	1	7
7352	7349	7355	-	13523	13521	13523	-	6	2	8
8171	8170	8172	-	12404	-	-	+	6	2	8
8733	-	-	-	13061	-	-	+	6	4	10
9273	9270	9277	-	14986	-	-	+	6	8	14
9606	9604	9607	-	13345	-	-	+	6	2	8
9618	9614	9619	-	15122	-	-	+	6	4	10
10370	10367	10374	-	14993	-	-	+	6	3	9
10420	10416	10421	-	10941	-	-	+	6	0	6
10462	10459	10462	-	14482	-	-	+	6	1	7
11028	11024	11032	-	14120	-	-	+	6	4	10
11097	11096	11100	-	12640	-	-	+	6	20	26
11249	11248	11252	-	13722	13721	13722	+	6	4	10
11435	11429	11438	-	14301	-	-	-	6	1	7
11523	11519	11526	-	14147	14146	14147	+	6	3	9
11950	11948	11950	-	14307	-	-	+	6	0	6
12053	12052	12054	-	13683	-	-	+	6	3	9
12062	12058	12064	-	14543	-	-	+	6	7	13
12101	12098	12104	-	14828	14828	14829	+	6	6	12
12102	12102	12106	+	15154	-	-	-	6	0	6
12113	12109	12116	-	13943	-	-	+	6	7	13
12291	12287	12293	-	14707	-	-	+	6	3	9
12321	12318	12325	-	14464	-	-	+	6	4	10
12476	12471	12477	-	13721	13720	13721	+	6	3	9
12476	12471	12477	-	14668	14668	14671	+	6	8	14
12591	12589	12594	-	15263	-	-	+	6	8	14
12641	12636	12645	-	14908	14907	14908	+	6	4	10
12652	12649	12653	-	13850	-	-	+	6	3	9
12696	12692	12697	-	15265	15265	15267	+	6	27	33
12741	12736	12744	-	15216	15216	15218	+	6	10	16
12788	12787	12792	-	13973	-	-	+	6	37	43
12833	12833	12835	-	13814	-	-	+	6	26	32
12941	12938	12945	-	13697	-	-	+	6	3	9
13025	13023	13029	-	14369	-	-	+	6	4	10
13127	13124	13129	-	14736	-	-	+	6	3	9
13141	13139	13145	-	14870	-	-	+	6	4	10
13265	13261	13268	-	14163	14163	14166	+	6	7	13
13347	13344	13353	-	14835	14835	14838	+	6	7	13
13451	13448	13454	-	13738	13736	13738	-	6	1	7
13451	13448	13454	-	14705	-	-	+	6	1	7
13569	13568	13572	-	14446	-	-	-	6	0	6
13605	13605	13609	+	14675	14674	14675	-	6	3	9
13616	13612	13619	-	14656	14654	14660	-	6	32	38
13623	13620	13623	-	14680	-	-	-	6	2	8
13709	13703	13713	-	14532	-	-	+	6	3	9
13791	13789	13797	-	14077	-	-	+	6	1	7
13791	13789	13797	-	14566	-	-	+	6	3	9
13863	13861	13866	-	14332	-	-	+	6	10	16
13863	13861	13866	-	14662	-	-	+	6	1	7
13876	13872	13879	-	15267	-	-	+	6	25	31
13994	13994	13995	-	14459	-	-	+	6	0	6
14003	14003	14007	+	15146	-	-	+	6	0	6
14046	14044	14049	-	14953	-	-	+	6	5	11
14065	14062	14068	-	14752	-	-	+	6	4	10
14091	14089	14096	-	14881	14880	14881	-	6	1	7
14138	14134	14142	-	14837	-	-	+	6	2	8
14174	14174	14180	-	14815	-	-	+	6	4	10
14260	-	-	-	14865	-	-	+	6	14	20
14331	14328	14337	-	14409	14408	14409	-	6	6	12
14331	14328	14337	-	14836	-	-	+	6	1	7
14369	14367	14371	-	14715	-	-	+	6	3	9
14403	14399	14406	-	14787	-	-	-	6	0	6
14415	14410	14416	-	14968	-	-	+	6	0	6
14423	14418	14424	-	14941	14941	14942	+	6	5	11
14527	-	-	+	14534	-	-	+	6	1	7
14622	14619	14626	-	14730	14730	14733	+	6	6	12
14732	14727	14737	-	14921	14921	14924	+	6	4	10
14732	14727	14737	-	15161	-	-	+	6	6	12
14769	14769	14770	+	14925	-	-	+	6	8	14
14772	14771	14773	-	15197	15197	15198	+	6	0	6
14786	14782	14790	-	15056	15056	15057	+	6	12	18
14800	14797	14800	-	15009	-	-	+	6	6	12
14838	14834	14843	+	14858	-	-	-	6	0	6
14859	14856	14862	-	15079	-	-	-	6	2	8
14877	14873	14878	-	15156	-	-	+	6	4	10
14970	14969	14974	-	15159	15158	15160	-	6	1	7
15004	15000	15010	+	15056	15055	15059	+	6	11	17
15068	-	-	+	15118	-	-	-	6	0	6
15073	15073	15076	+	15103	15103	15104	-	6	0	6
15092	15087	15096	+	15140	-	-	-	6	0	6
15102	15098	15105	+	15116	15115	15116	-	6	0	6
15124	15121	15128	+	15149	15149	15152	-	6	0	6
120	-	-	-	1550	-	-	-	5	0	5
297	-	-	-	15087	-	-	+	5	6	11

556	555	556	+	14698	14697	14698	+	5	3	8
881	880	884	+	14383	14383	14384	+	5	6	11
943	943	944	+	14634 -	-		+	5	2	7
1038	1035	1041	+	14940	14939	14940	+	5	6	11
1038	1035	1041	+	15069 -	-		+	5	2	7
1066	1066	1068	+	12826 -	-		+	5	3	8
1605	1605	1609	-	14118 -	-		+	5	14	19
2037	2034	2038	+	10521 -	-		+	5	1	6
2037	2034	2038	+	13774	13770	13774	+	5	3	8
2037	2034	2038	+	14895 -	-		+	5	1	6
2042	2040	2045	-	12521	12518	12521	-	5	2	7
2052	2049	2055	+	14331 -	-		+	5	4	9
2070	2069	2070	+	13405 -	-		+	5	2	7
2168	2166	2168	+	13142 -	-		+	5	0	5
2298	2297	2301	+	11714 -	-		+	5	6	11
2414	2412	2416	+	13940 -	-		+	5	4	9
2455	2452	2459	+	12965 -	-		+	5	2	7
2493	2487	2493	+	13457 -	-		+	5	4	9
2588	2588	2590	+	12230 -	-		+	5	1	6
2588	2588	2590	+	13690 -	-		+	5	2	7
2673 -	-		+	14049 -	-		+	5	5	10
2780	2778	2780	+	14126 -	-		+	5	2	7
2861	2861	2862	+	13674 -	-		+	5	2	7
2904	2900	2907	+	14828	14825	14831	-	5	13	18
3023	3021	3023	+	13427 -	-		+	5	13	18
3243	3239	3243	+	11937 -	-		+	5	2	7
3373	3370	3379	+	11809 -	-		+	5	2	7
3386	3384	3389	+	13346 -	-		+	5	6	11
3485	3485	3487	+	13629	13629	13630	+	5	1	6
3751	3749	3751	+	14023 -	-		+	5	3	8
3835	3832	3836	+	15112	15111	15113	-	5	35	40
3841	3841	3842	+	13261 -	-		+	5	2	7
3942	3942	3946	+	12688 -	-		+	5	2	7
4003	4001	4004	+	14738 -	-		+	5	8	13
4157	4156	4157	+	13385 -	-		+	5	1	6
4300	4297	4303	+	14409 -	-		+	5	6	11
4476	4476	4481	+	12283	12283	12284	+	5	5	10
4879	4876	4880	+	13742 -	-		+	5	10	15
5295	5295	5298	+	12903 -	-		+	5	4	9
5778	5778	5780	-	15185 -	-		+	5	0	5
5780	5778	5780	+	15177 -	-		-	5	0	5
5827	5827	5828	+	14773 -	-		+	5	2	7
5914 -	-		-	14289 -	-		+	5	2	7
7083	7082	7087	-	11543 -	-		+	5	7	12
8218	8216	8218	-	14565 -	-		+	5	1	6
8258	8258	8261	-	15339 -	-		-	5	1	6
8270 -	-		+	12805 -	-		-	5	0	5
8313	8313	8314	-	14428 -	-		+	5	3	8
8596 -	-		-	12801 -	-		+	5	1	6
9016	9012	9018	-	14067 -	-		+	5	4	9
9198	9195	9198	-	12518 -	-		+	5	1	6
9414 -	-		-	14581 -	-		+	5	0	5
9672	9671	9675	-	14934	14931	14934	+	5	1	6
9984	9981	9987	-	15025 -	-		+	5	0	5
10258	10256	10261	-	14518	14517	14518	+	5	2	7
10300	10296	10301	-	15074 -	-		+	5	4	9
10543	10541	10544	-	14833	14832	14833	+	5	4	9
10571	10568	10573	-	14582	14580	14582	+	5	2	7
10581	10580	10583	-	13810 -	-		+	5	3	8
10657	10653	10657	-	12468 -	-		+	5	2	7
10720	10720	10724	-	14846	14846	14848	+	5	2	7
10742	10737	10742	-	14439 -	-		+	5	0	5
10869	10866	10870	-	15154	15153	15154	+	5	2	7
10995	10993	10996	-	12568 -	-		+	5	4	9
10995	10993	10996	-	14565	14565	14566	+	5	5	10
11391	11388	11394	-	15010	15008	15010	+	5	1	6
11435	11429	11438	-	14592 -	-		+	5	1	6
11586	11583	11586	-	15175 -	-		+	5	3	8
11665	11664	11669	-	12847	12846	12847	+	5	3	8
12090	12088	12090	+	15165 -	-		-	5	0	5
12113	12109	12116	-	14165 -	-		+	5	11	16
12136	12133	12139	-	14352 -	-		+	5	0	5
12307	12303	12310	-	13347 -	-		+	5	2	7
12307	12303	12310	-	14199 -	-		+	5	3	8
12407	12403	12410	-	15044 -	-		+	5	5	10
12433	12429	12439	-	14923 -	-		+	5	5	10
12508	12508	12510	-	14619 -	-		+	5	6	11
12518	12512	12523	-	13701 -	-		+	5	7	12
12560	12560	12563	-	15006 -	-		+	5	5	10
12598	12596	12599	-	13373 -	-		+	5	0	5
12604	12600	12607	-	12965 -	-		+	5	1	6
12604	12600	12607	-	13497	13494	13497	+	5	2	7
12604	12600	12607	-	13875	13872	13875	+	5	3	8
12676	12671	12678	-	14587 -	-		+	5	8	13
12717	12714	12721	-	13364	13364	13365	+	5	13	18
12741	12736	12744	-	13472 -	-		+	5	0	5
12775	12772	12778	-	13284 -	-		+	5	0	5
12797	12795	12797	-	14672 -	-		+	5	9	14
12851	12848	12855	-	14049 -	-		+	5	25	30
12956	12952	12960	-	14467	14467	14469	+	5	9	14
12990	12990	12993	-	14892 -	-		+	5	5	10

13013	13009	13017	-	13989	-	-	+	5	1	6
13025	13023	13029	-	13394	13394	13397	+	5	7	12
13136	13132	13137	-	13499	13499	13502	+	5	11	16
13141	13139	13145	-	13662	-	-	+	5	0	5
13149	13146	13152	-	14339	-	-	+	5	3	8
13199	13199	13201	-	14679	-	-	+	5	3	8
13246	13240	13246	-	13809	-	-	+	5	5	10
13252	13247	13256	-	13901	-	-	+	5	0	5
13330	13324	13334	-	14423	14422	14423	+	5	11	16
13330	13324	13334	-	14801	-	-	+	5	0	5
13347	13344	13353	-	13901	13901	13902	+	5	4	9
13385	13381	13388	-	14877	-	-	-	5	0	5
13451	13448	13454	-	14160	-	-	+	5	0	5
13488	13485	13491	-	15071	-	-	-	5	0	5
13498	13495	13504	-	13778	13778	13780	+	5	7	12
13498	13495	13504	-	13846	-	-	+	5	7	12
13498	13495	13504	-	14261	-	-	+	5	6	11
13523	13522	13527	-	14575	14575	14576	+	5	7	12
13544	13539	13545	-	14268	-	-	+	5	4	9
13544	13539	13545	-	14878	14878	14880	+	5	16	21
13579	13575	13584	-	14613	14613	14614	+	5	15	20
13587	13586	13589	-	14264	14264	14265	+	5	6	11
13639	13637	13642	-	13797	13794	13797	-	5	13	18
13673	13669	13675	-	14095	-	-	+	5	16	21
13687	13687	13688	+	15018	15018	15019	+	5	1	6
13709	13703	13713	-	14592	-	-	+	5	1	6
13779	13776	13783	-	14662	-	-	+	5	1	6
13936	13932	13940	-	14578	-	-	+	5	0	5
13953	13952	13956	-	14801	-	-	+	5	2	7
13972	13971	13974	-	14517	-	-	+	5	0	5
13994	13994	13995	-	14615	-	-	+	5	1	6
14108	14106	14112	-	15079	15079	15080	+	5	5	10
14193	14188	14196	-	14860	14859	14860	-	5	1	6
14193	14188	14196	-	15010	-	-	+	5	8	13
14248	14244	14252	-	14427	14427	14431	+	5	6	11
14248	14244	14252	-	14669	-	-	+	5	0	5
14284	14280	14285	-	14806	-	-	+	5	8	13
14307	14302	14308	-	14914	-	-	+	5	3	8
14331	14328	14337	-	14841	14837	14841	-	5	3	8
14331	14328	14337	-	14970	-	-	+	5	2	7
14350	14348	14353	-	14731	14731	14732	+	5	5	10
14350	14348	14353	-	14848	-	-	+	5	2	7
14364	14362	14364	+	14779	14779	14780	+	5	5	10
14375	14374	14381	+	14900	-	-	+	5	0	5
14385	14383	14388	-	14800	14800	14801	+	5	5	10
14464	14463	14470	-	14625	14622	14625	+	5	1	6
14501	14498	14505	+	14531	14530	14532	-	5	3	8
14518	14510	14522	-	14901	14899	14901	-	5	12	17
14526	14524	14530	-	14781	14781	14782	+	5	4	9
14565	14564	14568	-	14847	14844	14847	+	5	2	7
14617	14617	14620	+	15013	-	-	+	5	0	5
14622	14619	14626	-	14864	-	-	+	5	9	14
14622	14619	14626	-	15070	-	-	+	5	9	14
14633	14632	14635	-	14894	-	-	-	5	0	5
14651	14648	14651	+	14679	-	-	-	5	0	5
14653	14649	14655	-	15278	15278	15280	+	5	7	12
14666	14661	14670	-	14920	14917	14920	+	5	15	20
14725	14725	14726	-	15015	-	-	-	5	1	6
14761	14759	14764	-	15206	-	-	-	5	0	5
14854	14851	14855	-	15057	15056	15057	-	5	1	6
14864	14863	14864	-	15017	15015	15017	-	5	3	8
14864	14863	14864	-	15195	15194	15195	-	5	2	7
14882	14879	14886	-	15053	15050	15056	-	5	2	7
14923	14919	14926	-	15048	15047	15052	-	5	18	23
14923	14919	14926	-	15204	-	-	+	5	6	11
14964	14962	14968	-	15068	-	-	-	5	0	5
15015	15011	15019	+	15161	-	-	-	5	0	5
15042	15041	15042	+	15074	-	-	-	5	0	5
15063	15058	15066	+	15088	-	-	-	5	1	6
15063	15058	15066	+	15103	15099	15103	-	5	0	5
15072	15068	15075	-	15174	-	-	-	5	0	5
15072	15068	15075	-	15262	15260	15262	+	5	0	5
158	156	158	+	9207	-	-	-	4	0	4
202	201	202	+	13979	13978	13979	+	4	1	5
228	225	229	+	14850	-	-	+	4	5	9
370	-	-	+	6670	-	-	-	4	0	4
521	521	523	+	12258	-	-	+	4	2	6
684	682	689	+	13320	-	-	+	4	5	9
825	822	826	+	13551	13551	13552	+	4	2	6
838	835	838	+	9136	9133	9136	+	4	0	4
855	854	855	+	14247	14246	14247	+	4	1	5
871	870	871	+	13742	13739	13742	+	4	1	5
881	880	884	+	14902	-	-	+	4	2	6
921	918	921	-	7382	-	-	+	4	0	4
930	929	931	-	8479	8478	8479	-	4	0	4
962	-	-	+	13775	-	-	+	4	0	4
969	968	969	+	14930	-	-	+	4	3	7
1574	1571	1575	+	2321	-	-	+	4	0	4
1785	1785	1786	-	1999	-	-	-	4	0	4
1932	1927	1934	+	1949	-	-	+	4	0	4
1932	1927	1934	+	13126	-	-	+	4	0	4

1944	1943	1945	+	13847	-	-	+	4	1	5
1944	1943	1945	+	14045	-	-	+	4	3	7
2075	2071	2076	+	13471	-	-	+	4	1	5
2249	2248	2252	+	13852	-	-	+	4	3	7
2252	2252	2256	-	15023	15022	15023	+	4	2	6
2272	2272	2274	+	12394	-	-	+	4	2	6
2317	2315	2317	+	12985	-	-	+	4	4	8
2368	2368	2370	+	12897	-	-	+	4	0	4
2435	2431	2436	+	13518	-	-	+	4	0	4
2455	2452	2459	+	14358	-	-	+	4	7	11
2473	2470	2473	+	11657	11657	11658	+	4	2	6
2499	2499	2502	+	13372	-	-	+	4	0	4
2553	2553	2554	+	14355	-	-	+	4	4	8
2620	2620	2622	+	15066	15064	15066	+	4	3	7
2768	2764	2771	+	14187	-	-	+	4	0	4
2780	2778	2780	+	9602	-	-	+	4	3	7
2808	-	-	+	13356	-	-	+	4	2	6
3115	3115	3119	+	14787	14784	14787	+	4	5	9
3184	3180	3188	+	14300	-	-	+	4	1	5
3214	3213	3218	+	14830	-	-	+	4	3	7
3227	3226	3232	+	15105	-	-	+	4	3	7
3249	3248	3249	+	9837	-	-	+	4	0	4
3283	3280	3283	+	12421	12418	12421	+	4	3	7
3288	3287	3292	+	8043	8042	8043	+	4	2	6
3366	3362	3366	+	14333	-	-	+	4	0	4
3565	3565	3567	+	10454	-	-	+	4	0	4
3581	3577	3581	+	14401	-	-	+	4	2	6
3628	3628	3629	+	10240	10240	10241	+	4	3	7
3912	3911	3912	-	4290	-	-	-	4	0	4
4374	-	-	+	14705	-	-	+	4	1	5
4438	4434	4442	+	15036	15032	15036	+	4	23	27
4455	4455	4457	+	15053	-	-	+	4	12	16
4499	4495	4499	+	13289	13288	13289	+	4	2	6
4663	4663	4667	-	12228	-	-	+	4	0	4
4940	4937	4940	-	12865	-	-	-	4	0	4
4944	4939	4947	+	14791	-	-	+	4	4	8
5252	5251	5252	+	14103	-	-	+	4	3	7
5354	5354	5358	+	14475	-	-	+	4	0	4
5435	-	-	+	15199	-	-	-	4	0	4
5785	5783	5789	+	15179	15179	15180	-	4	0	4
6037	6033	6037	-	14814	-	-	+	4	1	5
6556	6552	6556	-	6633	-	-	-	4	0	4
6682	6682	6684	-	14439	-	-	+	4	0	4
6808	6808	6810	+	13815	-	-	+	4	0	4
8065	8061	8066	-	14794	14794	14795	-	4	6	10
8770	8770	8774	-	15153	-	-	+	4	12	16
8952	8948	8955	-	13433	13431	13433	+	4	3	7
9129	-	-	-	13354	-	-	+	4	4	8
9967	9964	9967	-	15234	-	-	+	4	5	9
10031	10030	10032	-	14139	-	-	+	4	1	5
10210	10207	10214	-	15069	-	-	+	4	1	5
10389	10388	10392	-	13222	-	-	+	4	0	4
10485	10481	10486	-	12358	-	-	+	4	0	4
10499	10496	10500	+	10514	10514	10517	-	4	2	6
10548	10548	10550	-	14137	-	-	+	4	2	6
10564	10562	10564	-	12922	-	-	+	4	1	5
10694	10692	10698	+	14876	14876	14879	-	4	1	5
10776	10773	10778	-	12659	12659	12661	+	4	0	4
10797	10793	10799	-	12298	-	-	-	4	0	4
10860	10858	10862	-	13165	13163	13165	+	4	1	5
10885	10882	10887	-	13871	13871	13872	+	4	4	8
11006	11004	11008	-	12268	-	-	+	4	1	5
11028	11024	11032	-	13602	-	-	+	4	0	4
11127	11126	11128	-	14758	-	-	+	4	3	7
11139	-	-	-	13300	-	-	+	4	0	4
11145	11141	11145	-	14248	-	-	+	4	0	4
11446	11442	11450	+	14441	14440	14441	+	4	24	28
11452	11448	11456	-	14331	14329	14331	-	4	0	4
11493	11490	11494	-	14145	-	-	+	4	1	5
11511	11510	11513	-	15251	15250	15251	+	4	3	7
11523	11519	11526	-	11791	-	-	+	4	0	4
11665	11664	11669	-	13979	-	-	+	4	6	10
11693	11690	11696	-	14456	-	-	+	4	3	7
11708	11706	11711	-	14360	-	-	+	4	2	6
11864	11863	11868	-	12958	-	-	-	4	0	4
11864	11863	11868	-	15017	15017	15021	+	4	3	7
11873	11870	11877	-	12956	-	-	+	4	0	4
11914	11911	11914	-	13930	13929	13930	+	4	5	9
11975	11974	11979	-	13521	-	-	+	4	3	7
12018	12016	12023	-	14294	-	-	+	4	3	7
12018	12016	12023	-	14306	-	-	+	4	6	10
12046	12043	12048	-	14388	14386	14388	-	4	4	8
12087	12085	12087	-	14730	-	-	+	4	1	5
12136	12133	12139	-	13785	-	-	+	4	5	9
12161	12159	12162	-	14315	-	-	+	4	0	4
12176	12174	12178	-	13385	-	-	+	4	4	8
12187	12184	12190	-	13380	-	-	+	4	2	6
12199	12194	12203	-	13433	-	-	+	4	10	14
12199	12194	12203	-	14463	14463	14465	+	4	6	10
12208	12208	12211	+	14618	-	-	+	4	0	4
12240	12236	12241	-	13267	-	-	+	4	13	17

12257	12252	12259	-	14046	14046	14047	+	4	10	14
12269	12266	12272	-	15128 -	-		+	4	2	6
12275	12274	12275	-	14804 -	-		+	4	2	6
12291	12287	12293	-	13679 -	-		+	4	4	8
12467	12462	12470	-	13703 -	-		+	4	7	11
12536	12535	12540	-	14571 -	-		+	4	4	8
12560	12560	12563	-	14204 -	-		-	4	0	4
12560	12560	12563	-	14608 -	-		+	4	2	6
12577	12573	12581	-	13491	13490	13491	+	4	3	7
12604	12600	12607	-	13955	13955	13956	-	4	1	5
12604	12600	12607	-	14567 -	-		+	4	0	4
12604	12600	12607	-	14662 -	-		+	4	0	4
12652	12649	12653	-	14307 -	-		+	4	5	9
12676	12671	12678	-	13346 -	-		+	4	7	11
12685 -	-		+	14851 -	-		+	4	2	6
12717	12714	12721	-	14840	14840	14842	+	4	6	10
12732	12727	12735	-	14566 -	-		+	4	2	6
12734	12733	12734	+	14892 -	-		+	4	0	4
12775	12772	12778	-	13014 -	-		+	4	5	9
12864	12861	12869	-	13479	13478	13479	+	4	2	6
12874	12870	12879	-	13430	13427	13430	+	4	2	6
12896	12896	12898	-	14248	14247	14248	+	4	2	6
12930	12930	12931	+	15149 -	-		+	4	0	4
12948	12947	12950	-	13123	13123	13124	+	4	1	5
12970	12967	12973	-	14298	14298	14301	+	4	9	13
12976	12975	12979	-	14323	14322	14323	+	4	9	13
12983 -	-		+	14039 -	-		+	4	0	4
12985	12982	12988	-	13262 -	-		+	4	3	7
13000	12995	13002	-	14287 -	-		+	4	4	8
13007	13005	13008	-	13063 -	-		+	4	0	4
13043	13039	13043	+	13442 -	-		+	4	2	6
13055	13051	13058	-	13338 -	-		-	4	0	4
13070	13069	13071	-	13319 -	-		+	4	4	8
13087 -	-		-	13609 -	-		+	4	2	6
13094	13088	13097	-	13416 -	-		+	4	6	10
13117	13117	13121	-	14660 -	-		-	4	1	5
13149	13146	13152	-	14782 -	-		+	4	5	9
13164	13161	13168	-	13881 -	-		+	4	3	7
13252	13247	13256	-	14246	14246	14248	+	4	5	9
13265	13261	13268	-	13797	13794	13797	+	4	8	12
13272	13269	13276	-	13625 -	-		+	4	0	4
13293	13291	13294	-	13593 -	-		+	4	0	4
13298	13296	13302	-	14741 -	-		+	4	7	11
13316	13315	13320	-	13742	13742	13746	+	4	9	13
13316	13315	13320	-	14871	14871	14872	-	4	2	6
13316	13315	13320	-	14981	14978	14981	+	4	3	7
13317	13315	13320	+	14475	14475	14478	+	4	0	4
13330	13324	13334	-	13656	13656	13659	+	4	10	14
13339	13335	13341	-	13479 -	-		+	4	2	6
13349	13346	13353	+	15243 -	-		+	4	2	6
13385	13381	13388	-	14076 -	-		+	4	0	4
13408	13404	13411	-	13942 -	-		+	4	8	12
13416	13413	13420	-	14836 -	-		+	4	3	7
13440	13438	13440	-	14294 -	-		+	4	2	6
13446	13442	13446	-	13709	13708	13709	+	4	5	9
13446	13442	13446	-	13737 -	-		-	4	0	4
13451	13448	13454	-	13743	13741	13743	-	4	4	8
13532	13532	13533	+	15076 -	-		-	4	0	4
13533	13529	13537	-	14537 -	-		-	4	0	4
13544	13539	13545	-	14514 -	-		-	4	13	17
13549	13547	13553	-	14430	14429	14430	+	4	5	9
13555	13554	13555	-	14560	14559	14560	+	4	1	5
13562	13556	13567	-	14148 -	-		+	4	0	4
13587	13586	13589	-	14836 -	-		+	4	4	8
13587	13586	13589	-	14909 -	-		+	4	5	9
13610	13605	13611	-	13973 -	-		+	4	4	8
13623	13620	13623	-	14106	14106	14109	+	4	4	8
13629	13625	13631	-	14144 -	-		+	4	9	13
13629	13625	13631	-	14212	14212	14213	+	4	9	13
13634	13632	13635	-	13792	13790	13792	-	4	3	7
13645	13644	13649	-	14538	14537	14538	+	4	10	14
13651	13650	13653	-	14381 -	-		+	4	6	10
13673	13669	13675	-	14766 -	-		+	4	3	7
13695	13692	13695	-	14784	14782	14784	+	4	4	8
13700	13696	13701	-	14261 -	-		+	4	3	7
13700	13696	13701	-	15127 -	-		+	4	1	5
13720	13718	13723	-	13911 -	-		+	4	1	5
13741	13738	13745	-	14836 -	-		+	4	0	4
13761	13761	13765	+	14337 -	-		+	4	1	5
13770	13764	13773	-	14482 -	-		+	4	6	10
13770	13764	13773	-	14848	14848	14852	+	4	5	9
13774	13772	13777	+	13969	13967	13969	-	4	0	4
13779	13776	13783	-	14264	14264	14265	+	4	4	8
13791	13789	13797	-	15020 -	-		+	4	4	8
13870	13867	13871	-	14019 -	-		+	4	0	4
13870	13867	13871	-	14265 -	-		-	4	0	4
13898	13897	13899	+	15277	15277	15278	+	4	134	138
13908	13906	13912	-	14939	14939	14940	+	4	5	9
13928	13927	13929	-	14202 -	-		+	4	0	4
13936	13932	13940	-	14036 -	-		-	4	0	4
13936	13932	13940	-	14662 -	-		+	4	3	7

13936	13932	13940	-	14884 -	-		-	4	0	4
13988	13984	13992	-	15168	15166	15168	+	4	2	6
14001	13996	14005	-	14713	14713	14714	+	4	7	11
14019	14015	14022	-	14668 -	-		+	4	4	8
14035	14032	14038	-	14628 -	-		+	4	4	8
14046	14044	14049	-	14665	14665	14666	+	4	9	13
14079	14079	14083	-	14318 -	-		+	4	2	6
14079	14079	14083	-	14341 -	-		-	4	0	4
14108	14106	14112	-	14725 -	-		+	4	0	4
14120	14115	14126	-	14221	14221	14225	-	4	5	9
14120	14115	14126	-	14866 -	-		+	4	0	4
14202	14198	14205	-	14768	14768	14769	+	4	0	4
14207	14207	14211	+	15165 -	-		+	4	10	14
14243	14241	14243	-	14769 -	-		+	4	8	12
14248	14244	14252	-	14849 -	-		+	4	0	4
14248	14244	14252	-	14968 -	-		+	4	16	20
14267	14263	14270	-	14762 -	-		-	4	0	4
14284	14280	14285	-	14631	14631	14633	+	4	3	7
14294	14291	14295	-	15090	15088	15090	+	4	4	8
14316	14311	14318	-	14868	14868	14870	+	4	5	9
14320	14320	14324	+	14941	14937	14941	+	4	2	6
14342	14339	14346	-	14467 -	-		-	4	19	23
14342	14339	14346	-	15165 -	-		+	4	3	7
14346	14344	14350	+	14845	14845	14847	+	4	0	4
14360	14355	14365	-	14708 -	-		+	4	0	4
14360	14355	14365	-	14808	14807	14808	-	4	7	11
14370	14366	14373	+	14779	14779	14780	+	4	1	5
14403	14399	14406	-	15317	15316	15317	-	4	0	4
14422	14420	14425	+	14464 -	-		+	4	2	6
14422	14420	14425	+	15052 -	-		-	4	2	6
14428	14427	14429	-	14499 -	-		+	4	2	6
14435	14434	14439	-	14891 -	-		+	4	0	4
14435	14434	14439	-	15282	15280	15282	-	4	0	4
14489	14484	14493	-	14540	14540	14541	+	4	5	9
14489	14484	14493	-	14705 -	-		+	4	4	8
14489	14484	14493	-	14914 -	-		+	4	2	6
14501	14498	14507	-	14824 -	-		-	4	1	5
14501	14498	14505	+	14540 -	-		-	4	0	4
14532 -	-		-	14886 -	-		-	4	0	4
14532 -	-		-	15199 -	-		+	4	1	5
14561	14557	14566	+	15022 -	-		+	4	8	12
14571	14570	14572	-	14812 -	-		+	4	2	6
14573	14571	14573	+	14980 -	-		-	4	4	8
14589	14584	14594	+	14853	14853	14857	+	4	6	10
14595	14592	14597	-	14896 -	-		+	4	2	6
14605	14600	14609	+	15120 -	-		+	4	0	4
14622	14619	14626	-	15033 -	-		-	4	0	4
14651	14648	14651	+	15158 -	-		+	4	0	4
14656	14652	14661	+	15164	15163	15164	+	4	10	14
14666	14661	14670	-	14781	14778	14781	-	4	0	4
14666	14661	14670	-	14818 -	-		-	4	1	5
14666	14661	14670	-	14913 -	-		-	4	8	12
14688	14685	14689	-	15043	15042	15043	+	4	1	5
14694	14690	14698	-	14824 -	-		-	4	3	7
14732	14727	14737	-	14854	14854	14857	+	4	3	7
14732	14727	14737	-	15031	15031	15036	+	4	7	11
14739	14738	14742	+	15035	15034	15037	+	4	7	11
14761	14759	14764	-	15015 -	-		-	4	3	7
14777	14774	14780	-	15121 -	-		+	4	9	13
14805	14800	14806	+	14818 -	-		-	4	0	4
14842	14841	14842	-	15153 -	-		+	4	0	4
14847	14843	14850	-	15020 -	-		-	4	2	6
14869	14865	14872	-	15066	15066	15067	-	4	0	4
14869	14865	14872	-	15117	15115	15117	-	4	3	7
14869	14865	14872	-	15156 -	-		-	4	2	6
14870	14866	14873	+	15183 -	-		+	4	0	4
14870	14866	14873	+	15189	15189	15190	+	4	2	6
14877	14873	14878	-	15038	15038	15039	+	4	6	10
14894	14890	14897	-	14973	14970	14974	-	4	2	6
14894	14890	14897	-	14991	14991	14992	-	4	0	4
14898	14898	14900	+	14882 -	-		-	4	0	4
14902	14899	14908	-	15028	15024	15028	-	4	0	4
14902	14899	14908	-	15085 -	-		-	4	1	5
14916	14912	14918	-	15236	15234	15236	+	4	3	7
14916	14912	14918	-	15266 -	-		+	4	7	11
14928	14928	14933	+	15161	15157	15161	-	4	8	12
14947	14942	14953	-	15063 -	-		-	4	0	4
14947	14942	14953	-	15130 -	-		-	4	4	8
14957	14956	14960	-	15157	15157	15158	-	4	2	6
14964	14962	14968	-	15041 -	-		-	4	0	4
15014	15010	15016	-	15066 -	-		-	4	1	5
15037	15033	15040	+	15079	15079	15082	-	4	1	5
15047	15043	15050	+	15057 -	-		-	4	0	4
15053	15053	15057	+	15084	15084	15086	-	4	0	4
15053	15053	15057	+	15108 -	-		-	4	4	8
15081	15077	15081	+	15064	15061	15067	-	4	9	13
15102	15098	15105	+	15150	15150	15151	-	4	1	5
15124	15121	15128	+	15141	15137	15141	-	4	0	4
15177	15176	15180	+	15201 -	-		-	4	0	4
15354	15351	15354	-	15360 -	-		-	4	0	4
277	272	280	-	560 -	-		-	3	0	3

286	283	287	-	15099	15098	15099	+	3	2	5
321 -	-		+	13599 -	-		+	3	0	3
336 -	-		-	498 -	-		-	3	0	3
442 -	-		-	14345 -	-		+	3	0	3
534	530	534	+	14339	14337	14339	+	3	1	4
534	530	534	+	14981 -	-		+	3	0	3
699	696	703	+	14559 -	-		+	3	0	3
715	712	716	+	14673 -	-		+	3	5	8
736	733	736	+	15010 -	-		+	3	1	4
767 -	-		-	1076 -	-		-	3	0	3
770	769	775	+	14419	14419	14421	+	3	1	4
802	802	803	+	14836 -	-		+	3	4	7
896	893	896	+	12913	12912	12913	+	3	0	3
923	920	927	+	14993 -	-		+	3	7	10
935	935	937	+	14994	14993	14994	+	3	2	5
969	968	969	+	13365 -	-		+	3	4	7
989 -	-		-	12966 -	-		-	3	0	3
1004 -	-		+	13742 -	-		+	3	0	3
1052	1049	1056	+	14631	14630	14631	+	3	2	5
1140	1138	1143	+	13380	13380	13381	+	3	3	6
1140	1138	1143	+	14634	14634	14635	+	3	2	5
1193	1193	1195	+	13871	13871	13873	+	3	4	7
1399	1396	1403	-	4473	4473	4477	+	3	0	3
1448	1444	1449	-	2238 -	-		-	3	6	9
1455	1455	1458	+	1458 -	-		+	3	0	3
1485 -	-		+	15195 -	-		+	3	0	3
1780 -	-		-	13722 -	-		-	3	0	3
1785	1785	1786	-	13759 -	-		+	3	3	6
1852	1850	1856	+	14001	13998	14002	+	3	2	5
1863	1860	1866	+	13797	13797	13799	+	3	9	12
1863	1860	1866	+	14071 -	-		+	3	1	4
1900	1896	1904	+	13061 -	-		+	3	1	4
1909	1906	1913	+	15235 -	-		+	3	2	5
1932	1927	1934	+	13451	13451	13454	+	3	1	4
1938	1937	1939	+	14761	14759	14761	+	3	3	6
1944	1943	1945	+	15061	15060	15061	+	3	1	4
1959	1953	1963	+	11759 -	-		+	3	0	3
1959	1953	1963	+	13996 -	-		+	3	0	3
1984	1982	1988	+	11613	11612	11613	+	3	1	4
2024	2024	2028	-	14531 -	-		+	3	0	3
2024	2024	2028	+	13742 -	-		+	3	1	4
2052	2049	2055	+	11961 -	-		+	3	0	3
2085	2081	2085	+	14950 -	-		+	3	1	4
2118	2115	2121	+	14010 -	-		+	3	2	5
2118	2115	2121	+	14065	14063	14065	+	3	1	4
2131	2126	2135	+	4160 -	-		+	3	8	11
2233	2232	2233	+	13633 -	-		+	3	0	3
2249	2248	2252	+	13151	13150	13151	+	3	8	11
2300	2299	2300	-	9673 -	-		+	3	0	3
2357 -	-		+	10055 -	-		+	3	2	5
2397	2396	2401	+	10450 -	-		+	3	3	6
2397	2396	2401	+	14255 -	-		+	3	0	3
2473	2470	2473	+	11972 -	-		+	3	1	4
2473	2470	2473	+	13436	13435	13436	+	3	4	7
2526	2521	2527	+	12242 -	-		+	3	4	7
2532 -	-		+	13080 -	-		+	3	7	10
2638	2638	2641	+	14146 -	-		+	3	2	5
2800	2799	2802	+	14044	14044	14045	+	3	1	4
2813	2813	2814	+	9857	9857	9858	+	3	3	6
2827	2826	2831	+	12421 -	-		+	3	1	4
2897	2897	2898	+	14968 -	-		+	3	0	3
3045 -	-		+	12303	12303	12304	+	3	3	6
3106	3102	3110	+	13030 -	-		+	3	2	5
3163 -	-		-	13677 -	-		+	3	0	3
3272	3270	3275	+	12363 -	-		+	3	0	3
3272	3270	3275	+	14678 -	-		+	3	3	6
3277	3276	3277	+	12061 -	-		+	3	0	3
3322	3322	3323	+	9713 -	-		+	3	1	4
3373	3370	3379	+	3419 -	-		+	3	1	4
3400 -	-		-	11895 -	-		-	3	0	3
3419	3418	3419	+	13613	13612	13613	+	3	5	8
3436 -	-		+	14057 -	-		+	3	4	7
3448	3446	3452	+	14212	14212	14213	+	3	8	11
3498	3498	3501	+	14256 -	-		+	3	6	9
3508	3505	3512	+	12014	12014	12016	+	3	7	10
3508	3505	3512	+	12220	12220	12221	+	3	5	8
3611	3607	3615	-	9351 -	-		+	3	0	3
3660	3660	3661	+	14400 -	-		+	3	2	5
3678	3674	3680	+	8304 -	-		+	3	1	4
3731	3730	3731	+	14837	14836	14837	+	3	5	8
3771	3771	3774	+	15210	15208	15211	-	3	6	9
4105	4102	4107	+	14080 -	-		+	3	2	5
4135	4135	4136	+	14108	14107	14108	+	3	2	5
4212	4211	4215	+	13302 -	-		+	3	0	3
4318	4318	4319	-	11463 -	-		+	3	0	3
4346	4345	4348	+	12350	12349	12350	+	3	2	5
4346	4345	4348	+	14072 -	-		+	3	0	3
4843	4842	4843	+	13453	13452	13453	+	3	4	7
4864	4864	4868	+	13187 -	-		+	3	0	3
5039 -	-		-	5207 -	-		-	3	0	3
5046 -	-		-	5205 -	-		-	3	0	3

5078	5076	5079	-	15083	15082	15084	+	3	84	87
5295	5295	5299	-	14464	-	-	+	3	0	3
5476	5473	5477	-	15063	15062	15063	+	3	2	5
5484	-	-	-	15145	15145	15146	+	3	3	6
5847	-	-	+	14547	-	-	+	3	0	3
6119	6118	6120	+	8286	-	-	-	3	0	3
6220	6216	6221	-	11774	11774	11775	+	3	3	6
6257	-	-	+	14485	-	-	+	3	0	3
6530	6530	6533	+	14697	-	-	+	3	1	4
6571	6571	6572	-	15144	-	-	+	3	0	3
6631	-	-	+	14281	-	-	+	3	0	3
6890	6890	6891	+	15068	15068	15069	+	3	5	8
7417	-	-	+	14329	-	-	+	3	1	4
7534	7530	7537	-	14332	14328	14336	+	3	3	6
7570	-	-	+	7852	-	-	+	3	0	3
7595	7592	7595	+	13858	13856	13858	+	3	2	5
7830	7827	7830	-	10456	-	-	+	3	0	3
8103	8103	8107	-	13666	-	-	+	3	0	3
8113	8112	8113	-	13524	13523	13524	+	3	1	4
8233	8231	8234	-	15312	15312	15313	-	3	6	9
8349	8348	8351	-	15074	15074	15075	+	3	0	3
8411	8411	8415	-	15031	-	-	+	3	0	3
8423	-	-	-	14222	-	-	+	3	3	6
8490	-	-	-	13393	-	-	+	3	0	3
8614	8614	8615	-	15134	-	-	+	3	0	3
8635	-	-	-	11869	-	-	+	3	0	3
8656	-	-	+	14272	-	-	+	3	4	7
8799	8799	8800	-	14554	14553	14554	+	3	1	4
8952	8948	8955	-	14798	-	-	+	3	1	4
9089	9085	9089	-	12429	12427	12429	+	3	3	6
9107	9106	9110	-	14428	-	-	+	3	0	3
9155	-	-	-	13784	-	-	+	3	0	3
9499	9496	9499	-	15321	-	-	-	3	2	5
9873	9870	9873	-	13955	-	-	-	3	0	3
9920	9920	9921	-	14873	-	-	+	3	3	6
10031	10030	10032	-	12147	-	-	+	3	0	3
10058	10058	10060	-	11072	-	-	+	3	0	3
10196	10196	10197	-	14783	-	-	+	3	12	15
10210	10207	10214	-	14751	-	-	+	3	4	7
10300	10296	10301	-	14409	-	-	+	3	1	4
10311	10309	10311	-	15250	-	-	+	3	1	4
10324	10323	10324	-	14602	14602	14603	+	3	0	3
10471	10471	10473	-	14166	14164	14166	+	3	1	4
10581	10580	10583	-	13432	13432	13433	+	3	1	4
10588	10587	10588	-	14090	-	-	+	3	0	3
10624	10621	10624	-	13770	13767	13770	+	3	3	6
10668	10665	10670	-	14353	-	-	+	3	0	3
10720	10720	10724	-	14254	-	-	+	3	3	6
10726	10725	10733	-	14253	-	-	+	3	0	3
10726	10725	10733	-	14838	14835	14840	+	3	1	4
10742	10737	10742	-	13553	-	-	+	3	9	12
10755	10754	10755	-	14992	14992	14993	+	3	4	7
10776	10773	10778	-	13986	-	-	+	3	0	3
10776	10773	10778	-	14807	-	-	+	3	0	3
10823	10819	10827	-	14369	-	-	+	3	1	4
10842	-	-	-	14564	-	-	+	3	2	5
10847	10844	10850	-	12275	12274	12275	+	3	2	5
10875	10875	10876	-	11621	-	-	+	3	0	3
10892	10892	10894	-	15239	15237	15239	+	3	3	6
10995	10993	10996	-	14410	-	-	+	3	0	3
11020	11019	11021	-	12999	12999	13000	+	3	3	6
11028	11024	11032	-	14126	-	-	-	3	0	3
11028	11024	11032	-	14351	-	-	+	3	0	3
11156	11152	11158	-	14471	-	-	+	3	6	9
11218	11214	11218	-	13857	-	-	+	3	2	5
11249	11248	11252	-	14169	-	-	+	3	0	3
11305	-	-	+	13957	-	-	+	3	0	3
11313	11309	11313	-	14350	-	-	+	3	1	4
11435	11429	11438	-	14422	14422	14426	-	3	17	20
11493	11490	11494	-	12940	-	-	+	3	0	3
11523	11519	11526	-	14259	-	-	+	3	2	5
11534	11530	11536	-	12144	-	-	+	3	0	3
11534	11530	11536	-	13872	-	-	+	3	0	3
11556	11556	11559	-	13396	-	-	+	3	1	4
11617	11613	11617	-	14281	-	-	+	3	3	6
11699	11699	11702	-	13010	13009	13010	+	3	2	5
11830	11828	11834	-	13443	-	-	+	3	2	5
11920	11915	11924	-	13740	13740	13741	+	3	5	8
11931	11927	11931	-	14225	-	-	+	3	3	6
11965	11963	11965	-	14870	-	-	+	3	3	6
11975	11974	11979	-	14415	-	-	+	3	0	3
12026	12024	12030	-	12388	-	-	+	3	0	3
12062	12058	12064	-	12177	-	-	+	3	0	3
12062	12058	12064	-	14187	14187	14188	+	3	3	6
12094	12090	12095	-	13765	-	-	+	3	4	7
12101	12098	12104	-	13674	-	-	+	3	4	7
12113	12109	12116	-	14124	-	-	+	3	0	3
12146	12143	12147	-	14266	-	-	+	3	0	3
12146	12143	12147	-	14443	-	-	-	3	0	3
12146	12143	12147	-	15251	15250	15251	+	3	2	5
12161	12159	12162	-	13835	-	-	+	3	4	7

12161	12159	12162	-	14828	-	-	+	3	0	3
12199	12194	12203	-	13084	-	-	+	3	0	3
12208	12208	12211	+	14596	-	-	+	3	0	3
12257	12252	12259	-	13276	-	-	+	3	6	9
12321	12318	12325	-	14706	-	-	+	3	1	4
12341	12340	12346	-	13404	-	-	+	3	0	3
12341	12340	12346	-	14438	-	-	+	3	0	3
12390	12389	12390	-	13404	-	-	+	3	0	3
12407	12403	12410	-	14265	14265	14266	+	3	3	6
12445	12441	12448	-	13810	-	-	+	3	0	3
12467	12462	12470	-	14176	-	-	+	3	0	3
12467	12462	12470	-	14913	14913	14914	+	3	14	17
12518	12512	12523	-	14239	-	-	+	3	7	10
12536	12535	12540	-	14662	-	-	+	3	3	6
12553	12549	12558	-	12957	-	-	+	3	0	3
12553	12549	12558	-	14607	-	-	+	3	0	3
12560	12560	12563	-	13139	-	-	+	3	3	6
12577	12573	12581	-	12642	-	-	+	3	2	5
12591	12589	12594	-	13319	-	-	-	3	0	3
12591	12589	12594	-	13837	-	-	+	3	2	5
12598	12596	12599	-	13082	-	-	+	3	0	3
12598	12596	12599	-	14266	-	-	+	3	0	3
12604	12600	12607	-	14387	-	-	+	3	0	3
12611	12608	12614	-	14845	14845	14849	-	3	4	7
12635	12632	12635	-	14494	14493	14494	+	3	2	5
12717	12714	12721	-	13080	-	-	-	3	0	3
12741	12736	12744	-	13988	-	-	+	3	7	10
12754	12753	12759	-	14465	-	-	+	3	0	3
12761	12760	12765	-	14619	-	-	+	3	6	9
12775	12772	12778	-	14056	-	-	+	3	0	3
12780	12779	12780	-	14150	-	-	+	3	1	4
12838	12836	12841	-	14283	-	-	+	3	1	4
12851	12848	12855	-	13685	13685	13686	+	3	1	4
12864	12861	12869	-	13957	-	-	+	3	2	5
12903	12901	12905	-	13523	-	-	+	3	14	17
12903	12901	12905	-	13742	-	-	-	3	5	8
12903	12901	12905	-	14177	-	-	+	3	2	5
12911	12906	12916	-	14858	14856	14858	-	3	0	3
12924	12921	12928	+	15141	-	-	+	3	0	3
12965	12964	12966	-	13310	-	-	+	3	2	5
13013	13009	13017	-	14331	-	-	+	3	3	6
13013	13009	13017	-	14525	14524	14525	+	3	2	5
13025	13023	13029	-	14134	-	-	+	3	0	3
13025	13023	13029	-	14435	-	-	+	3	3	6
13044	13040	13046	-	15074	-	-	+	3	2	5
13055	13051	13058	-	13861	-	-	+	3	0	3
13070	13069	13071	-	14836	-	-	+	3	2	5
13094	13088	13097	-	13707	13707	13708	+	3	10	13
13094	13088	13097	-	14266	-	-	+	3	0	3
13104	13100	13107	-	13847	-	-	+	3	0	3
13111	13108	13111	-	13328	-	-	+	3	0	3
13127	13124	13129	-	13299	-	-	+	3	4	7
13136	13132	13137	-	14639	-	-	+	3	3	6
13141	13139	13145	-	14458	-	-	+	3	1	4
13149	13146	13152	-	13481	-	-	+	3	0	3
13149	13146	13152	-	14045	-	-	+	3	0	3
13164	13161	13168	-	13662	-	-	+	3	0	3
13164	13161	13168	-	14390	14388	14390	+	3	0	3
13207	13203	13210	-	13672	-	-	+	3	3	6
13224	13220	13228	-	14317	-	-	+	3	8	11
13265	13261	13268	-	14329	14329	14330	+	3	3	6
13272	13269	13276	-	14110	-	-	+	3	2	5
13272	13269	13276	-	14383	-	-	+	3	3	6
13278	13277	13282	-	14410	14409	14410	+	3	2	5
13308	13303	13313	-	14877	-	-	-	3	1	4
13308	13303	13313	-	15035	-	-	+	3	0	3
13316	13315	13320	-	14014	-	-	+	3	3	6
13322	13321	13322	-	14876	14875	14876	-	3	4	7
13330	13324	13334	-	13411	13409	13411	+	3	3	6
13330	13324	13334	-	14532	14531	14532	+	3	1	4
13347	13344	13353	-	13981	-	-	+	3	1	4
13347	13344	13353	-	14531	14531	14532	+	3	9	12
13347	13344	13353	-	14969	-	-	+	3	2	5
13359	13355	13363	-	15014	15012	15014	+	3	1	4
13365	13364	13367	-	13910	13910	13911	+	3	4	7
13377	13376	13379	-	14379	-	-	-	3	0	3
13398	13394	13402	+	14399	-	-	+	3	0	3
13408	13404	13411	-	14601	-	-	+	3	1	4
13440	13438	13440	-	14421	-	-	+	3	0	3
13446	13442	13446	-	13442	-	-	+	3	0	3
13446	13442	13446	-	14537	14535	14537	-	3	0	3
13462	13457	13464	-	13698	-	-	+	3	0	3
13462	13457	13464	-	13772	-	-	-	3	1	4
13462	13457	13464	-	14264	14264	14265	+	3	3	6
13462	13457	13464	-	14355	-	-	+	3	6	9
13462	13457	13464	-	14552	-	-	-	3	0	3
13479	13475	13484	-	14880	-	-	-	3	0	3
13498	13495	13504	-	14651	-	-	+	3	1	4
13516	13516	13517	-	14300	-	-	+	3	5	8
13549	13547	13553	-	14578	14578	14581	+	3	2	5
13579	13575	13584	-	13849	-	-	-	3	0	3

13579	13575	13584	-	14880 -	-		+	3	1	4
13594	13593	13595	-	13839 -	-		+	3	5	8
13616	13612	13619	-	13731	13730	13731	+	3	0	3
13634	13632	13635	-	14215 -	-		-	3	0	3
13639	13637	13642	-	14436 -	-		-	3	4	7
13639	13637	13642	-	14463 -	-		-	3	0	3
13639	13637	13642	-	14676	14676	14679	-	3	2	5
13673	13669	13675	-	14184	14183	14184	-	3	1	4
13685	13682	13688	-	14564 -	-		+	3	1	4
13685	13682	13688	-	14717 -	-		+	3	0	3
13695	13692	13695	-	14081	14081	14082	+	3	3	6
13709	13703	13713	-	14248 -	-		+	3	4	7
13709	13703	13713	-	14410 -	-		+	3	3	6
13709	13703	13713	-	15135 -	-		-	3	0	3
13720	13718	13723	-	14797	14797	14798	+	3	3	6
13728	13726	13732	+	13758 -	-		-	3	0	3
13741	13738	13745	-	14759	14759	14762	+	3	3	6
13757	13756	13761	-	14344 -	-		-	3	0	3
13770	13764	13773	-	14444	14444	14445	-	3	6	9
13770	13764	13773	-	14502 -	-		+	3	2	5
13779	13776	13783	-	14331 -	-		+	3	0	3
13799	13799	13804	-	14915 -	-		+	3	1	4
13810	13807	13815	-	14080 -	-		+	3	3	6
13810	13807	13815	-	14652	14651	14652	+	3	2	5
13848	13843	13849	-	13901 -	-		+	3	2	5
13851	13850	13855	+	14687 -	-		+	3	5	8
13855	13850	13859	-	14608 -	-		+	3	0	3
13876	13872	13879	-	14017	14014	14019	+	3	3	6
13878	13875	13882	+	13923	13923	13924	-	3	1	4
13885	13880	13885	-	14371 -	-		-	3	8	11
13908	13906	13912	-	13885 -	-		+	3	0	3
13908	13906	13912	-	14717	14717	14720	+	3	6	9
13914	13913	13917	-	14261	14260	14261	+	3	1	4
13936	13932	13940	-	14423	14422	14423	+	3	3	6
13947	13941	13951	-	14095 -	-		-	3	0	3
13953	13952	13956	-	14010 -	-		+	3	0	3
13953	13952	13956	-	14714 -	-		+	3	1	4
13967	13964	13968	-	14558 -	-		+	3	0	3
14001	13996	14005	-	15060 -	-		+	3	0	3
14003	14003	14007	+	14909	14907	14909	+	3	0	3
14012	14009	14016	+	14704 -	-		+	3	0	3
14025	14023	14028	-	14651 -	-		+	3	4	7
14035	14032	14038	-	14692 -	-		-	3	1	4
14040	14040	14042	-	14559 -	-		+	3	4	7
14065	14062	14068	-	14528 -	-		-	3	0	3
14100	14100	14103	-	14915	14914	14915	+	3	2	5
14108	14106	14112	-	14215	14214	14215	+	3	0	3
14108	14106	14112	-	14640	14638	14640	-	3	1	4
14108	14106	14112	-	14647 -	-		+	3	0	3
14120	14115	14126	-	14211 -	-		-	3	1	4
14120	14115	14126	-	14229 -	-		-	3	3	6
14138	14134	14142	-	14619	14619	14620	-	3	1	4
14145	14145	14148	-	14350 -	-		+	3	0	3
14159	14157	14160	-	14900 -	-		+	3	0	3
14174	14174	14180	-	14177 -	-		+	3	3	6
14174	14174	14180	-	14803 -	-		-	3	0	3
14180	14177	14183	+	14157 -	-		-	3	3	6
14187	14182	14187	-	14677 -	-		-	3	2	5
14187	14182	14187	-	14975 -	-		+	3	2	5
14193	14188	14196	-	14795 -	-		+	3	0	3
14193	14188	14196	-	15063 -	-		+	3	11	14
14212	14210	14216	-	14581 -	-		+	3	0	3
14219	14215	14222	+	15150 -	-		+	3	3	6
14223	14219	14230	-	14287 -	-		+	3	4	7
14223	14219	14230	-	15185 -	-		-	3	5	8
14248	14244	14252	-	15180	15178	15180	-	3	7	10
14267	14263	14270	-	14880	14880	14881	+	3	5	8
14284	14280	14285	-	14459	14458	14459	+	3	1	4
14284	14280	14285	-	14523	14523	14524	+	3	4	7
14286	14283	14286	+	14340 -	-		+	3	2	5
14289	14287	14289	-	14494 -	-		+	3	8	11
14300	14298	14301	-	14987 -	-		+	3	1	4
14316	14311	14318	-	14465 -	-		+	3	2	5
14331	14328	14337	-	14474	14470	14474	-	3	0	3
14342	14339	14346	-	14432	14428	14432	+	3	0	3
14342	14339	14346	-	14860	14858	14860	+	3	2	5
14350	14348	14353	-	14470	14470	14471	-	3	3	6
14358	14354	14358	+	15091	15091	15095	-	3	4	7
14360	14355	14365	-	14436	14432	14436	+	3	1	4
14360	14355	14365	-	15032 -	-		-	3	0	3
14370	14366	14373	+	15084	15081	15086	-	3	6	9
14403	14399	14406	-	14943 -	-		+	3	1	4
14417	14413	14417	+	14445 -	-		-	3	0	3
14447	14447	14450	+	14540	14540	14544	-	3	0	3
14456	14452	14461	-	14707	14706	14707	-	3	1	4
14464	14463	14470	-	15101 -	-		+	3	0	3
14501	14498	14507	-	14831	14827	14831	+	3	1	4
14507	14507	14510	+	14548	14548	14549	-	3	0	3
14518	14510	14522	-	14895	14894	14895	+	3	2	5
14540	14536	14541	-	14873 -	-		+	3	2	5
14547	14547	14550	-	14864 -	-		+	3	2	5

14559	14555	14563	-	14617	14612	14617	+	3	2	5
14565	14564	14568	-	14993	14991	14993	+	3	3	6
14565	14564	14568	-	15086	-	-	+	3	8	11
14623	14622	14624	+	15191	15190	15191	+	3	2	5
14647	-	-	-	15231	-	-	-	3	0	3
14656	14652	14661	+	14676	14672	14676	-	3	2	5
14656	14652	14661	+	14776	-	-	+	3	0	3
14673	14671	14674	-	14940	-	-	-	3	0	3
14676	14672	14677	+	14653	-	-	-	3	10	13
14685	14681	14687	+	14969	14969	14970	+	3	10	13
14694	14690	14698	-	14797	-	-	+	3	1	4
14716	14713	14719	-	15033	-	-	+	3	0	3
14721	14717	14721	+	15023	-	-	+	3	3	6
14725	14725	14726	-	15037	15037	15041	-	3	4	7
14725	14725	14726	-	15071	-	-	-	3	1	4
14732	14727	14737	-	15018	-	-	+	3	3	6
14732	14727	14737	-	15043	15039	15043	-	3	0	3
14741	14738	14742	-	15036	-	-	-	3	1	4
14745	-	-	+	14856	-	-	-	3	0	3
14748	14743	14752	-	15293	-	-	+	3	7	10
14767	14765	14770	-	14863	-	-	+	3	0	3
14777	14774	14780	-	14932	14930	14932	-	3	3	6
14800	14797	14800	-	15122	-	-	+	3	0	3
14818	14814	14822	+	14806	14803	14806	-	3	0	3
14818	14814	14822	+	15030	-	-	+	3	0	3
14837	14834	14838	-	14930	14929	14930	+	3	2	5
14838	14834	14843	+	14879	14879	14881	-	3	0	3
14847	14843	14850	-	14967	14965	14967	-	3	3	6
14847	14843	14850	-	15014	15013	15014	-	3	4	7
14847	14843	14850	-	15055	15055	15057	-	3	3	6
14847	14843	14850	-	15217	-	-	+	3	4	7
14858	14855	14862	+	14827	-	-	-	3	0	3
14859	14856	14862	-	15027	15025	15027	-	3	2	5
14869	14865	14872	-	15052	15051	15052	+	3	1	4
14869	14865	14872	-	15104	-	-	-	3	0	3
14869	14865	14872	-	15181	-	-	-	3	0	3
14877	14873	14878	-	15027	15025	15027	-	3	2	5
14877	14873	14878	-	15043	15043	15044	+	3	2	5
14882	14879	14886	-	15002	-	-	-	3	0	3
14887	14884	14887	+	14895	-	-	-	3	0	3
14892	14889	14895	+	14870	-	-	-	3	9	12
14892	14889	14895	+	14877	14875	14877	-	3	10	13
14894	14890	14897	-	15008	15008	15011	-	3	2	5
14902	14899	14908	-	14943	14943	14947	+	3	21	24
14902	14899	14908	-	14970	14970	14972	-	3	0	3
14922	14921	14926	+	14952	14949	14952	+	3	8	11
14928	14927	14928	-	15161	-	-	+	3	0	3
14941	14938	14941	-	15086	15085	15088	-	3	3	6
14947	14942	14953	-	15022	15022	15024	-	3	2	5
14947	14942	14953	-	15092	15092	15096	-	3	7	10
14970	14969	14974	-	15044	-	-	-	3	0	3
14982	14978	14986	-	15085	15083	15085	-	3	1	4
15004	15000	15010	+	15022	15022	15026	+	3	3	6
15004	15000	15010	+	15045	-	-	-	3	0	3
15004	15000	15010	+	15154	-	-	-	3	0	3
15032	15030	15032	+	15110	-	-	-	3	0	3
15032	15030	15032	+	15132	-	-	-	3	0	3
15042	15041	15042	+	15119	-	-	-	3	0	3
15042	15041	15042	+	15153	-	-	-	3	0	3
15047	15043	15050	+	15129	-	-	-	3	0	3
15063	15058	15066	+	15094	15094	15097	-	3	0	3
15072	15068	15075	-	15213	-	-	+	3	0	3
15081	15077	15081	+	15139	15136	15139	-	3	0	3
15086	15082	15086	+	15062	15062	15063	-	3	1	4
15102	15098	15105	+	15133	-	-	-	3	0	3
15157	15154	15157	+	15183	15182	15183	+	3	2	5
15162	15162	15165	+	15238	15235	15238	+	3	0	3
15189	15184	15192	+	15210	-	-	-	3	2	5
15251	15251	15252	+	15269	15268	15271	+	3	33	36
15277	15277	15278	-	15384	-	-	-	3	1	4
1	-	-	-	103	-	-	-	2	0	2
23	23	24	+	14842	-	-	+	2	0	2
77	74	77	-	12982	12980	12982	+	2	2	4
77	74	77	-	14069	-	-	-	2	2	4
277	272	280	-	14342	-	-	+	2	0	2
277	272	280	-	14433	14433	14436	-	2	3	5
344	-	-	-	5477	-	-	+	2	0	2
355	350	358	-	1327	-	-	-	2	2	4
379	377	383	+	14955	14953	14955	+	2	2	4
448	447	448	+	15077	-	-	+	2	5	7
458	458	462	+	14326	-	-	-	2	0	2
458	458	462	+	15124	-	-	-	2	2	4
465	464	465	+	11223	-	-	+	2	0	2
488	488	490	+	1459	-	-	+	2	2	4
495	495	499	+	12624	12624	12625	+	2	1	3
544	544	549	+	13682	13682	13683	+	2	2	4
596	596	597	+	14613	-	-	+	2	0	2
596	596	597	+	15149	-	-	+	2	0	2
603	598	604	+	3544	-	-	-	2	0	2
603	598	604	+	12760	-	-	+	2	0	2
620	618	622	+	13847	13847	13851	+	2	3	5

635 -	-		-	11876 -	-		+	2	1	3
638	634	638	+	10792 -	-		+	2	0	2
672	670	676	+	15042 -	-		-	2	0	2
699	696	703	+	13127 -	-		+	2	2	4
705	704	707	+	14649 -	-		-	2	0	2
715	712	716	+	14425 -	-		+	2	0	2
770	769	775	+	14241 -	-		+	2	3	5
796	794	797	-	15125 -	-		+	2	0	2
802	802	803	+	12454 -	-		+	2	0	2
802	802	803	+	14763 -	-		+	2	12	14
833	829	833	-	1201 -	-		-	2	0	2
855	854	855	+	13791 -	-		+	2	4	6
896	893	896	+	14693 -	-		+	2	0	2
903	902	903	+	14133 -	-		+	2	0	2
923	920	927	+	13308 -	-		+	2	2	4
923	920	927	+	14288 -	-		+	2	0	2
957	954	960	+	14409 -	-		+	2	1	3
969	968	969	+	14294 -	-		+	2	0	2
979	978	980	+	12319 -	-		+	2	3	5
979	978	980	+	12884 -	-		+	2	0	2
991	988	993	+	14837 -	-		+	2	3	5
998	998	1001	+	13700 -	-		+	2	2	4
1029	1029	1032	-	14523 -	-		+	2	0	2
1099	1097	1103	+	13167 -	-		+	2	1	3
1121	1121	1122	-	14205	14205	14206	+	2	2	4
1121	1120	1121	+	14267	14266	14267	+	2	1	3
1129 -	-		-	14724 -	-		+	2	2	4
1129 -	-		+	14215 -	-		+	2	1	3
1140	1138	1143	+	12192 -	-		+	2	0	2
1140	1138	1143	+	14982 -	-		+	2	2	4
1145	1142	1149	-	1328	1325	1328	-	2	3	5
1201	1201	1202	+	12306	12306	12307	+	2	2	4
1201	1201	1202	+	14035 -	-		+	2	0	2
1241	1240	1242	+	14752 -	-		+	2	0	2
1249	1248	1252	+	11005 -	-		+	2	2	4
1253	1249	1253	-	14066 -	-		+	2	0	2
1399	1396	1403	-	1603 -	-		-	2	0	2
1426	1422	1428	+	15144 -	-		+	2	0	2
1478	1478	1479	+	15188	15188	15189	+	2	2	4
1495	1491	1497	-	1565 -	-		-	2	0	2
1505	1501	1505	+	1676 -	-		+	2	0	2
1598	1594	1600	-	1846 -	-		-	2	0	2
1616	1615	1617	+	14529 -	-		+	2	3	5
1665	1665	1666	+	14842 -	-		+	2	0	2
1702	1702	1704	+	14868 -	-		+	2	1	3
1722	1718	1722	+	1754 -	-		+	2	0	2
1792	1791	1795	+	14868	14867	14871	+	2	3	5
1809	1808	1811	+	14283 -	-		+	2	3	5
1816 -	-		+	14350 -	-		+	2	2	4
1835	1830	1836	+	14915 -	-		+	2	2	4
1852	1850	1856	+	8813 -	-		+	2	0	2
1863	1860	1866	+	12233	12233	12234	-	2	2	4
1869	1869	1873	-	13187 -	-		+	2	0	2
1869	1869	1873	-	14439 -	-		-	2	1	3
1869	1869	1871	+	13457 -	-		+	2	2	4
1877 -	-		+	12228 -	-		+	2	2	4
1885	1882	1885	+	13242 -	-		+	2	0	2
1885	1882	1885	+	13507 -	-		+	2	1	3
1891	1887	1894	+	14565 -	-		+	2	1	3
1900	1896	1904	+	12599 -	-		+	2	3	5
1900	1896	1904	+	14921 -	-		+	2	0	2
1909	1906	1913	+	13744 -	-		+	2	0	2
1917	1917	1919	+	12147 -	-		+	2	2	4
1938	1937	1939	+	10866 -	-		+	2	1	3
1969 -	-		-	15143 -	-		+	2	3	5
2014	2011	2014	-	2307 -	-		-	2	0	2
2016	2013	2019	+	12090 -	-		+	2	1	3
2024	2024	2028	+	12161 -	-		-	2	0	2
2030	2030	2031	+	9704 -	-		+	2	0	2
2030	2030	2031	+	14751 -	-		+	2	0	2
2047 -	-		-	12525 -	-		-	2	0	2
2052	2049	2055	+	12534 -	-		+	2	0	2
2054	2052	2059	-	12534	12530	12534	-	2	1	3
2070	2069	2070	+	13525 -	-		+	2	0	2
2085	2081	2085	+	12335 -	-		+	2	0	2
2093	2092	2093	+	13487 -	-		+	2	1	3
2099 -	-		-	12410 -	-		-	2	0	2
2131	2126	2135	+	12165 -	-		+	2	2	4
2131	2126	2135	+	13356 -	-		+	2	3	5
2141	2137	2141	-	14868 -	-		+	2	1	3
2146 -	-		-	14863 -	-		+	2	0	2
2155	2152	2155	+	11689 -	-		+	2	0	2
2155	2152	2155	+	12144 -	-		+	2	0	2
2155	2152	2155	+	12864	12864	12865	+	2	3	5
2155	2152	2155	+	13362 -	-		+	2	0	2
2155	2152	2155	+	14567 -	-		+	2	1	3
2168	2164	2168	-	12383 -	-		+	2	0	2
2179	2177	2183	+	2197 -	-		+	2	2	4
2179	2177	2183	+	14694 -	-		+	2	0	2
2190	2186	2194	+	12601 -	-		+	2	0	2
2213	2213	2219	+	12327 -	-		+	2	1	3

2213		2213	2219	+	12521	-	-		+	2	0	2
2213		2213	2219	+	13936	-	-		+	2	0	2
2238	-	-		+	13825	-	-		+	2	0	2
2244	-	-		+	13824	-	-		+	2	4	6
2249		2248	2252	+	11207	-	-		+	2	2	4
2258		2257	2258	+	13250	-	-		+	2	0	2
2293		2293	2294	+	11821	-	-		+	2	0	2
2293		2293	2294	+	14401	-	-		+	2	2	4
2303		2302	2303	+	13229	-	-		+	2	1	3
2317		2315	2317	+	10400	-	-		-	2	0	2
2325		2322	2325	+	13281	-	-		+	2	0	2
2368		2368	2370	+	13209	-	-		-	2	0	2
2386		2385	2390	+	12742	-	-		+	2	2	4
2386		2385	2390	+	13199	-	-		+	2	0	2
2414		2412	2416	+	12276	-	-		+	2	0	2
2426		2424	2429	+	11911		11910	11911	+	2	1	3
2449	-	-		+	11828	-	-		+	2	2	4
2455		2452	2459	+	13303	-	-		+	2	0	2
2455		2452	2459	+	14485	-	-		+	2	2	4
2473		2470	2473	+	14412	-	-		+	2	0	2
2479	-	-		+	14725	-	-		+	2	0	2
2493		2487	2493	+	12742	-	-		+	2	0	2
2493		2487	2493	+	13229	-	-		+	2	3	5
2493		2487	2493	+	13994		13994	13995	-	2	2	4
2499		2499	2502	+	13597	-	-		+	2	0	2
2515		2512	2515	+	11274	-	-		+	2	0	2
2515		2512	2515	+	12444		12442	12444	+	2	1	3
2539		2539	2543	+	14357	-	-		+	2	2	4
2557	-	-		-	13732	-	-		+	2	0	2
2571		2571	2573	+	2584	-	-		+	2	2	4
2583		2582	2583	+	13258	-	-		+	2	2	4
2588		2588	2590	+	11468	-	-		+	2	1	3
2597	-	-		+	12535	-	-		+	2	0	2
2647		2645	2647	+	11388	-	-		+	2	0	2
2673	-	-		-	4611	-	-		-	2	0	2
2687		2683	2692	-	4314		4314	4315	-	2	0	2
2696		2692	2696	+	10945	-	-		+	2	0	2
2718	-	-		-	2784	-	-		-	2	0	2
2734		2733	2736	+	13329	-	-		+	2	0	2
2734		2733	2736	+	15246	-	-		+	2	0	2
2753	-	-		+	13451	-	-		+	2	3	5
2775	-	-		+	3086	-	-		+	2	2	4
2780		2778	2780	+	9390	-	-		+	2	2	4
2780		2778	2780	+	12128		12126	12128	+	2	1	3
2800		2799	2802	+	10816	-	-		+	2	1	3
2800		2799	2802	+	12274	-	-		+	2	0	2
2867		2866	2870	+	13861	-	-		-	2	0	2
2915		2912	2917	-	14984	-	-		-	2	1	3
2931		2926	2931	+	13632	-	-		-	2	0	2
2932		2929	2932	-	14805		14802	14805	+	2	1	3
2970	-	-		-	3063	-	-		-	2	1	3
2975		2975	2976	-	3048	-	-		-	2	0	2
2975	-	-		+	12695	-	-		+	2	0	2
3023		3021	3023	+	13461	-	-		+	2	2	4
3034		3034	3037	+	14836	-	-		+	2	0	2
3040	-	-		-	14265	-	-		+	2	0	2
3098	-	-		-	14301	-	-		-	2	0	2
3106		3102	3110	+	13004		13003	13004	+	2	1	3
3133	-	-		-	14804	-	-		-	2	0	2
3154		3153	3154	+	15039	-	-		+	2	0	2
3157		3152	3157	-	13827	-	-		+	2	0	2
3166		3165	3166	+	12202		12202	12205	+	2	2	4
3184		3180	3188	+	11814	-	-		+	2	0	2
3184		3180	3188	+	13678	-	-		-	2	0	2
3227		3226	3232	+	4486		4486	4487	+	2	6	8
3227		3226	3232	+	12924	-	-		+	2	0	2
3272		3270	3275	+	11072	-	-		+	2	2	4
3274		3271	3275	-	3494	-	-		-	2	0	2
3274		3271	3275	-	4386	-	-		-	2	0	2
3288		3287	3292	+	14104	-	-		+	2	0	2
3297		3294	3297	+	13323	-	-		+	2	0	2
3297		3294	3297	+	15090		15088	15090	-	2	0	2
3360	-	-		+	14423	-	-		+	2	2	4
3373		3370	3379	+	13327	-	-		+	2	0	2
3381	-	-		+	13217	-	-		+	2	1	3
3391		3391	3393	+	14185		14185	14187	+	2	3	5
3401		3398	3403	+	13568	-	-		+	2	0	2
3448		3446	3452	+	13769	-	-		+	2	5	7
3474		3473	3474	-	14663	-	-		+	2	0	2
3498		3498	3501	+	14503		14503	14504	+	2	10	12
3501		3499	3502	-	15088		15088	15090	-	2	7	9
3508		3505	3512	+	12000	-	-		+	2	0	2
3550		3546	3550	+	14642		14642	14646	+	2	3	5
3598		3598	3601	-	3674	-	-		-	2	0	2
3628		3628	3629	+	14710		14710	14711	+	2	9	11
3667		3667	3672	+	13442		13442	13444	+	2	5	7
3689		3689	3690	-	4147	-	-		+	2	0	2
3697		3697	3701	+	12228	-	-		+	2	0	2
3742	-	-		+	11848	-	-		+	2	0	2
3795		3793	3799	+	11535	-	-		+	2	2	4
3821		3820	3821	+	13985	-	-		+	2	4	6

3821		3820	3821	+	14490	-	-		+	2	5	7
3931		3928	3935	+	12883	-	-		+	2	3	5
3957		3957	3959	+	13683	-	-		+	2	2	4
3976		3972	3976	+	13720	-	-		+	2	1	3
4003		4001	4004	+	14180	-	-		+	2	3	5
4003		4001	4004	+	14550	-	-		+	2	0	2
4075		4072	4075	+	14317	-	-		+	2	0	2
4157		4156	4157	+	12641	-	-		+	2	2	4
4188		4186	4189	-	4270	-	-		-	2	0	2
4198		4198	4200	-	12008	-	-		+	2	0	2
4212		4211	4215	+	12939	-	-		+	2	0	2
4221		4220	4225	-	13740	-	-		+	2	1	3
4258	-	-		+	14266	-	-		+	2	3	5
4266		4265	4267	+	11910		11910	11911	+	2	2	4
4300		4297	4303	+	11233	-	-		+	2	2	4
4333		4333	4336	+	13262		13262	13264	+	2	5	7
4355	-	-		+	12018	-	-		+	2	2	4
4438		4434	4442	+	13059	-	-		+	2	2	4
4465	-	-		+	13495	-	-		+	2	0	2
4471		4470	4471	+	12613	-	-		+	2	2	4
4484		4484	4488	+	4476		4474	4476	+	2	1	3
4484		4484	4488	+	12810	-	-		+	2	0	2
4545		4545	4550	+	13137	-	-		+	2	0	2
4571		4571	4573	-	10021		10021	10022	+	2	0	2
4591		4588	4592	+	12544	-	-		+	2	0	2
4610		4608	4610	+	10982	-	-		+	2	0	2
4640	-	-		-	14182	-	-		+	2	0	2
4663		4663	4667	-	8659	-	-		+	2	1	3
4710		4710	4711	-	4800	-	-		-	2	0	2
4805		4802	4805	+	13793	-	-		+	2	0	2
4834		4833	4835	+	13972		13972	13973	+	2	6	8
4862	-	-		-	12838	-	-		+	2	0	2
4921		4921	4925	+	12577	-	-		+	2	0	2
4921		4921	4925	+	14438		14437	14438	+	2	1	3
4937		4936	4937	+	15178	-	-		+	2	1	3
4940		4937	4940	-	13368	-	-		-	2	0	2
4944		4939	4947	+	12531	-	-		+	2	2	4
5048		5045	5048	+	12230	-	-		+	2	2	4
5055	-	-		-	5170	-	-		-	2	0	2
5057		5053	5057	+	13217	-	-		+	2	0	2
5179		5177	5179	+	14591	-	-		+	2	0	2
5223		5220	5223	-	9295		9292	9295	+	2	2	4
5288	-	-		-	15010	-	-		+	2	0	2
5324		5324	5327	+	13952		13952	13954	+	2	5	7
5324		5324	5327	+	13997	-	-		+	2	0	2
5375		5373	5375	-	14198		14198	14199	+	2	2	4
5392		5392	5396	-	13258	-	-		+	2	0	2
5450		5449	5454	+	12452	-	-		+	2	0	2
5461	-	-		+	13315	-	-		+	2	0	2
5489		5489	5493	-	14667	-	-		+	2	0	2
5878		5874	5881	+	12790	-	-		+	2	1	3
5916	-	-		+	7111	-	-		-	2	0	2
5941	-	-		+	12439	-	-		+	2	1	3
5966		5963	5966	+	12339	-	-		-	2	0	2
6037		6033	6037	-	14609	-	-		+	2	0	2
6104	-	-		+	14426	-	-		+	2	0	2
6119		6118	6120	+	10728	-	-		+	2	1	3
6142		6140	6142	+	13210		13208	13210	+	2	1	3
6228		6226	6233	-	12823		12821	12823	+	2	2	4
6269	-	-		+	11987	-	-		+	2	0	2
6274		6274	6278	+	11674	-	-		+	2	0	2
6302		6302	6306	+	6350	-	-		+	2	0	2
6332		6329	6334	+	14210	-	-		+	2	0	2
6494		6492	6494	-	14255	-	-		+	2	0	2
6556		6552	6556	-	7010	-	-		-	2	0	2
6598		6598	6599	-	10617		10616	10617	+	2	1	3
6704	-	-		-	14189	-	-		+	2	0	2
6809		6805	6809	-	15164	-	-		+	2	0	2
6883	-	-		+	11557	-	-		+	2	0	2
6936		6936	6939	+	10911	-	-		+	2	0	2
6998		6996	6998	+	7735	-	-		+	2	0	2
7083		7082	7087	-	13850		13848	13850	+	2	2	4
7099	-	-		-	7394	-	-		-	2	0	2
7145		7144	7145	-	14874	-	-		+	2	0	2
7212		7212	7215	+	7236	-	-		+	2	2	4
7225	-	-		-	7385	-	-		-	2	2	4
7352		7349	7355	-	14253	-	-		+	2	1	3
7414		7413	7415	-	13449		13448	13450	+	2	2	4
7467		7464	7467	-	13345	-	-		+	2	0	2
7502		7498	7506	-	14403	-	-		+	2	0	2
7569		7569	7572	-	15367		15367	15368	-	2	0	2
7578		7574	7581	-	13841	-	-		-	2	1	3
7606		7603	7606	+	14568		14565	14568	+	2	2	4
7622	-	-		+	9501	-	-		-	2	0	2
7626	-	-		-	14659	-	-		+	2	1	3
7809	-	-		+	11141	-	-		+	2	0	2
7836		7836	7841	-	7866		7863	7866	+	2	0	2
8287	-	-		+	14831	-	-		-	2	0	2
8332		8332	8336	-	14721	-	-		+	2	0	2
8355		8354	8355	-	15071		15071	15072	+	2	6	8
8445	-	-		-	14512	-	-		+	2	1	3

8463	-	-	-	14578	-	-	+	2	0	2
8572	8572	8574	-	11373	-	-	+	2	0	2
8614	8614	8615	-	12957	-	-	+	2	2	4
8834	8834	8836	-	11349	-	-	+	2	0	2
8834	8834	8836	-	14765	14763	14765	+	2	2	4
8952	8948	8955	-	12591	-	-	+	2	0	2
9100	9097	9100	-	12150	12149	12150	+	2	1	3
9209	9206	9213	-	14331	-	-	+	2	0	2
9263	-	-	-	11803	-	-	+	2	0	2
9554	9550	9554	+	14699	14699	14700	-	2	2	4
9562	9562	9564	-	14371	-	-	+	2	5	7
9600	-	-	-	14600	-	-	+	2	0	2
9666	9662	9666	-	13218	-	-	+	2	0	2
9744	-	-	-	12445	-	-	+	2	0	2
9816	9815	9817	-	14138	-	-	+	2	3	5
9846	9842	9846	-	15265	-	-	+	2	2	4
9860	9857	9862	-	14633	14633	14634	+	2	3	5
9873	9870	9873	-	14622	-	-	+	2	2	4
9878	9877	9879	-	13416	-	-	+	2	0	2
9878	9877	9879	-	14357	-	-	+	2	1	3
9905	-	-	+	11549	-	-	+	2	4	6
9942	9938	9946	+	14109	14108	14112	+	2	5	7
9945	9942	9948	-	15249	15249	15250	+	2	2	4
9991	9989	9992	-	11280	-	-	+	2	0	2
10008	10006	10008	-	11451	-	-	+	2	0	2
10019	10017	10019	-	13953	-	-	+	2	3	5
10024	10023	10026	-	13646	13646	13647	+	2	1	3
10024	10023	10026	-	15104	-	-	+	2	1	3
10032	10029	10036	+	14030	14029	14030	+	2	3	5
10038	10035	10041	-	12509	-	-	+	2	2	4
10038	10035	10041	-	13332	-	-	+	2	3	5
10038	10035	10041	-	13986	-	-	+	2	2	4
10052	10048	10054	-	14135	-	-	-	2	0	2
10052	10048	10054	-	14147	-	-	+	2	0	2
10065	10061	10068	-	13126	-	-	+	2	2	4
10080	10078	10080	-	14625	-	-	+	2	0	2
10097	10094	10098	-	10211	-	-	-	2	0	2
10097	10094	10098	-	14480	-	-	+	2	0	2
10115	10114	10115	+	12509	-	-	+	2	0	2
10131	10129	10131	-	14578	-	-	+	2	6	8
10156	10156	10159	-	14294	-	-	+	2	1	3
10228	10228	10232	-	10871	-	-	-	2	0	2
10228	10228	10232	-	11835	-	-	+	2	0	2
10230	10227	10230	+	13685	13682	13685	+	2	11	13
10258	10256	10261	-	14169	-	-	-	2	1	3
10258	10256	10261	-	14637	-	-	+	2	2	4
10278	10276	10282	-	13346	-	-	+	2	0	2
10300	10296	10301	-	10995	-	-	+	2	0	2
10300	10296	10301	-	13222	-	-	+	2	0	2
10311	10309	10311	-	14500	-	-	+	2	3	5
10337	10337	10341	-	14080	-	-	+	2	0	2
10360	10360	10364	-	14680	14678	14680	+	2	2	4
10370	10367	10374	-	14677	-	-	+	2	2	4
10406	10403	10410	-	13522	-	-	+	2	0	2
10425	10424	10425	-	14189	-	-	+	2	2	4
10436	10434	10436	-	14777	-	-	+	2	24	26
10462	10459	10462	-	13408	-	-	+	2	3	5
10479	10477	10479	-	14268	14266	14268	+	2	2	4
10485	10481	10486	-	14207	-	-	+	2	0	2
10485	10481	10486	-	14856	-	-	+	2	0	2
10504	-	-	-	14636	-	-	+	2	4	6
10512	10508	10514	-	14525	-	-	+	2	0	2
10512	10508	10514	-	14537	-	-	+	2	4	6
10543	10541	10544	-	13773	-	-	+	2	3	5
10571	10568	10573	-	12802	-	-	+	2	0	2
10571	10568	10573	-	14615	-	-	+	2	0	2
10571	10568	10573	-	14876	-	-	+	2	1	3
10601	-	-	+	14768	-	-	-	2	0	2
10644	10644	10648	-	14731	14729	14731	+	2	1	3
10677	10674	10680	-	14968	-	-	+	2	0	2
10706	10702	10710	+	14864	14861	14864	-	2	8	10
10724	10724	10725	+	13773	-	-	+	2	0	2
10726	10725	10733	-	14179	-	-	+	2	0	2
10726	10725	10733	-	14199	-	-	+	2	1	3
10726	10725	10733	-	14847	14843	14847	+	2	3	5
10755	10754	10755	-	12604	-	-	+	2	3	5
10797	10793	10799	-	12146	-	-	+	2	0	2
10817	10815	10817	-	13588	-	-	+	2	0	2
10829	-	-	-	14193	-	-	+	2	0	2
10860	10858	10862	-	14454	-	-	+	2	0	2
10875	10875	10876	-	12760	-	-	+	2	0	2
10885	10882	10887	-	14215	-	-	-	2	0	2
10911	10908	10911	-	11081	-	-	+	2	1	3
10918	10918	10920	-	12986	-	-	+	2	0	2
10942	-	-	-	13460	-	-	+	2	0	2
10969	10966	10970	-	13926	13926	13929	+	2	5	7
10983	10982	10987	-	13978	-	-	+	2	0	2
11006	11004	11008	-	13621	-	-	+	2	0	2
11020	11019	11021	-	14547	-	-	+	2	1	3
11028	11024	11032	-	14601	-	-	+	2	0	2
11047	-	-	-	14064	-	-	+	2	1	3

11097	11096	11100	-	15141	15141	15142	+	2	7	9
11103	11103	11106	-	12940 -	-		+	2	0	2
11164	11160	11164	-	14689 -	-		+	2	1	3
11170	11170	11173	-	13773 -	-		+	2	0	2
11188	11186	11189	-	13203 -	-		+	2	0	2
11195	11190	11195	-	12924	12923	12924	+	2	1	3
11195	11190	11195	-	15246 -	-		+	2	0	2
11213 -	-		+	12696 -	-		-	2	0	2
11228	11226	11234	-	14247 -	-		+	2	1	3
11240	11236	11241	-	13728 -	-		+	2	0	2
11240	11236	11241	-	14785	14785	14786	-	2	2	4
11257	11257	11262	-	14124 -	-		+	2	0	2
11287 -	-		-	14142 -	-		+	2	0	2
11313	11310	11313	+	13872 -	-		+	2	0	2
11313	11310	11313	+	14133 -	-		+	2	2	4
11319	11317	11320	-	13096 -	-		+	2	1	3
11330	11327	11330	+	14739 -	-		+	2	2	4
11391	11388	11394	-	13013 -	-		+	2	0	2
11418	11416	11422	-	12810 -	-		+	2	1	3
11418	11416	11422	-	14529 -	-		+	2	2	4
11435	11429	11438	-	13808 -	-		+	2	1	3
11435	11429	11438	-	14532 -	-		+	2	1	3
11446	11442	11450	+	14318 -	-		+	2	0	2
11465	11463	11469	-	12688 -	-		+	2	0	2
11465	11463	11469	-	12718 -	-		+	2	0	2
11484	11483	11487	-	11493 -	-		-	2	0	2
11484	11483	11487	-	11864 -	-		+	2	0	2
11493	11490	11494	-	12166 -	-		+	2	0	2
11493	11490	11494	-	15042 -	-		+	2	0	2
11511	11510	11513	-	14343 -	-		+	2	5	7
11534	11530	11536	-	14693 -	-		+	2	0	2
11551	11548	11554	-	11799 -	-		-	2	0	2
11551	11548	11554	-	13215 -	-		-	2	0	2
11556	11556	11559	-	12066 -	-		+	2	2	4
11576	11576	11578	-	14975 -	-		+	2	0	2
11602	11597	11605	-	14076 -	-		+	2	0	2
11602	11597	11605	-	15181 -	-		+	2	0	2
11607 -	-		-	14255 -	-		+	2	1	3
11617	11613	11617	-	14760 -	-		+	2	1	3
11617	11613	11617	-	14986 -	-		+	2	1	3
11641	11638	11644	-	13941	13937	13941	+	2	3	5
11648	11648	11649	-	14615 -	-		+	2	4	6
11678	11678	11680	-	13740	13736	13740	+	2	3	5
11693	11690	11696	-	13523 -	-		+	2	3	5
11708	11706	11711	-	13701 -	-		+	2	0	2
11708	11706	11711	-	14324 -	-		+	2	0	2
11733	11731	11734	-	13780 -	-		+	2	11	13
11733	11731	11734	-	14772	14771	14772	+	2	12	14
11758	11754	11759	-	13053 -	-		+	2	2	4
11774	11771	11774	-	13320 -	-		+	2	1	3
11774	11771	11774	-	14461 -	-		+	2	0	2
11783	11780	11787	-	14840 -	-		+	2	0	2
11808	11804	11808	-	13357 -	-		+	2	2	4
11823	11821	11826	-	13640 -	-		+	2	0	2
11830	11828	11834	-	13796	13794	13797	-	2	5	7
11836	11836	11840	-	13223 -	-		+	2	0	2
11847	11843	11851	-	14711 -	-		-	2	0	2
11864	11863	11868	-	12282 -	-		+	2	0	2
11873	11870	11877	-	14709 -	-		+	2	1	3
11890 -	-		-	12146 -	-		+	2	0	2
11891 -	-		+	13721 -	-		+	2	3	5
11920	11915	11924	-	12139 -	-		-	2	0	2
11920	11915	11924	-	15053 -	-		-	2	0	2
11926	11925	11926	-	13966	13965	13966	+	2	1	3
11931	11927	11931	-	12421 -	-		+	2	2	4
11965	11963	11965	-	13218 -	-		+	2	0	2
11975	11974	11979	-	14056 -	-		+	2	3	5
11987	11987	11989	-	13267 -	-		-	2	0	2
12002	12000	12003	-	14008 -	-		+	2	0	2
12018	12016	12023	-	13224	13224	13225	+	2	2	4
12018	12016	12023	-	14750	14750	14751	+	2	2	4
12026	12024	12030	-	13208 -	-		+	2	0	2
12026	12024	12030	-	14525 -	-		+	2	1	3
12032	12032	12034	-	13967 -	-		+	2	4	6
12037	12037	12041	-	15143 -	-		+	2	0	2
12046	12043	12048	-	13978 -	-		-	2	0	2
12062	12058	12064	-	12843 -	-		-	2	0	2
12070	12068	12072	-	12903 -	-		+	2	0	2
12070	12068	12072	-	14134 -	-		+	2	0	2
12075	12075	12078	-	12308 -	-		-	2	0	2
12081	12077	12085	+	15042 -	-		+	2	0	2
12087	12085	12087	-	14633 -	-		+	2	1	3
12113	12109	12116	-	14435 -	-		-	2	3	5
12126	12126	12129	-	14606	14605	14606	-	2	1	3
12127	12125	12127	+	12194 -	-		+	2	0	2
12136	12133	12139	-	14568 -	-		+	2	0	2
12146	12143	12147	-	13451	13451	13452	+	2	1	3
12146	12143	12147	-	13709 -	-		+	2	0	2
12146	12143	12147	-	13768 -	-		+	2	0	2
12146	12143	12147	-	14836	14836	14837	+	2	2	4
12161	12159	12162	-	13772 -	-		-	2	0	2

12187	12184	12190	-	12798	12797	12798	+	2	1	3
12208	12208	12211	+	14476	-	-	+	2	0	2
12218	12215	12220	-	13372	13372	13373	+	2	2	4
12218	12215	12220	-	14194	-	-	-	2	0	2
12225	12225	12228	-	12476	-	-	-	2	2	4
12233	12229	12233	-	14090	-	-	+	2	1	3
12233	12229	12233	-	14933	14933	14934	+	2	3	5
12240	12236	12241	-	14510	-	-	+	2	0	2
12240	12236	12241	-	14575	-	-	+	2	2	4
12246	12245	12246	-	13333	-	-	+	2	0	2
12257	12252	12259	-	14836	-	-	-	2	0	2
12257	12252	12259	-	14935	-	-	+	2	0	2
12282	12282	12283	-	12986	-	-	+	2	3	5
12299	12299	12301	-	14991	-	-	-	2	0	2
12307	12303	12310	-	13759	-	-	-	2	0	2
12307	12303	12310	-	14288	14286	14288	+	2	2	4
12307	12303	12310	-	14381	-	-	+	2	0	2
12307	12303	12310	-	14497	-	-	+	2	1	3
12308	12304	12308	+	13401	-	-	+	2	0	2
12308	12304	12308	+	13768	-	-	+	2	0	2
12314	12313	12317	+	13739	13738	13739	-	2	2	4
12321	12318	12325	-	14591	-	-	+	2	0	2
12327	12326	12329	-	13775	-	-	+	2	0	2
12327	12326	12329	-	14471	-	-	+	2	1	3
12334	12331	12338	-	14741	-	-	+	2	0	2
12341	12340	12346	-	13751	-	-	+	2	2	4
12364	12362	12367	-	14108	-	-	+	2	2	4
12376	12372	12376	-	14566	-	-	+	2	0	2
12407	12403	12410	-	13646	-	-	+	2	0	2
12418	12413	12420	-	14439	-	-	+	2	1	3
12418	12413	12420	-	14487	-	-	+	2	4	6
12418	12413	12420	-	14492	-	-	-	2	0	2
12418	12413	12420	-	14599	-	-	-	2	2	4
12433	12429	12439	-	14147	14146	14147	+	2	1	3
12449	12445	12449	+	14660	-	-	+	2	0	2
12476	12471	12477	-	13719	-	-	-	2	0	2
12481	12478	12484	-	13493	-	-	+	2	2	4
12486	12486	12487	-	13346	-	-	+	2	1	3
12496	12494	12496	-	13061	-	-	+	2	0	2
12496	12494	12496	-	14518	-	-	+	2	3	5
12501	12500	12501	-	14524	-	-	+	2	0	2
12518	12512	12523	-	13011	13011	13012	+	2	2	4
12529	12524	12531	-	13068	-	-	+	2	2	4
12536	12535	12540	-	13380	-	-	+	2	0	2
12553	12549	12558	-	12827	-	-	+	2	0	2
12553	12549	12558	-	14253	-	-	+	2	0	2
12553	12549	12558	-	14578	-	-	+	2	2	4
12560	12560	12563	-	13416	-	-	+	2	7	9
12577	12573	12581	-	14222	14222	14223	-	2	2	4
12577	12573	12581	-	14298	-	-	+	2	2	4
12591	12589	12594	-	14634	-	-	+	2	0	2
12604	12600	12607	-	14269	-	-	+	2	0	2
12611	12608	12614	-	13077	-	-	+	2	0	2
12618	12615	12618	+	14345	-	-	+	2	0	2
12634	-	-	+	12646	-	-	-	2	0	2
12641	12636	12645	-	13604	-	-	-	2	3	5
12641	12636	12645	-	14518	-	-	+	2	0	2
12647	-	-	-	15278	-	-	+	2	0	2
12652	12649	12653	-	13573	-	-	+	2	2	4
12661	12657	12666	-	13855	-	-	-	2	0	2
12661	12657	12666	-	14339	14339	14342	+	2	2	4
12683	12682	12688	-	13605	-	-	+	2	7	9
12683	12682	12688	-	13893	-	-	+	2	2	4
12683	12682	12688	-	13979	-	-	+	2	3	5
12696	12692	12697	-	13741	-	-	+	2	7	9
12717	12714	12721	-	14170	-	-	+	2	2	4
12732	12727	12735	-	12971	12971	12972	+	2	2	4
12741	12736	12744	-	13345	-	-	+	2	0	2
12741	12736	12744	-	13533	-	-	+	2	2	4
12741	12736	12744	-	13951	13949	13951	+	2	1	3
12741	12736	12744	-	14008	-	-	+	2	0	2
12754	12753	12759	-	13838	-	-	+	2	9	11
12775	12772	12778	-	13699	-	-	+	2	3	5
12802	12799	12802	-	14410	-	-	+	2	0	2
12802	12799	12802	-	14547	-	-	+	2	2	4
12802	12799	12802	-	14991	14991	14992	+	2	9	11
12838	12836	12841	-	14994	-	-	+	2	0	2
12851	12848	12855	-	13553	-	-	+	2	1	3
12864	12861	12869	-	14179	14179	14180	+	2	3	5
12874	12870	12879	-	12943	-	-	-	2	0	2
12884	12881	12886	-	13266	-	-	+	2	2	4
12884	12881	12886	-	14810	-	-	+	2	0	2
12891	12891	12892	-	14103	14102	14103	+	2	4	6
12921	12919	12925	-	14179	-	-	+	2	3	5
12921	12919	12925	-	14508	-	-	+	2	8	10
12928	12926	12931	-	13874	-	-	-	2	0	2
12935	12935	12938	+	15054	-	-	+	2	0	2
12941	12938	12945	-	13442	13442	13443	+	2	29	31
12941	12938	12945	-	13596	13596	13599	+	2	3	5
12956	12952	12960	-	14981	-	-	+	2	3	5
12956	12952	12960	-	15250	15250	15252	+	2	8	10

12978	12976	12980	+	14761	-	-	+	2	1	3
12990	12990	12993	-	14785	-	-	+	2	2	4
13000	12995	13002	-	14024	14024	14025	+	2	6	8
13013	13009	13017	-	13543	-	-	+	2	2	4
13025	13023	13029	-	13695	-	-	+	2	1	3
13025	13023	13029	-	14535	-	-	-	2	0	2
13044	13040	13046	-	14423	-	-	+	2	2	4
13044	13040	13046	-	14566	-	-	-	2	0	2
13055	13051	13058	-	13199	-	-	+	2	0	2
13055	13051	13058	-	14877	14875	14877	-	2	1	3
13060	13059	13064	-	13454	-	-	-	2	0	2
13070	13069	13071	-	14676	-	-	+	2	0	2
13082	13079	13082	-	13199	-	-	+	2	0	2
13094	13088	13097	-	13372	-	-	+	2	2	4
13094	13088	13097	-	13978	-	-	+	2	6	8
13094	13088	13097	-	14841	-	-	+	2	0	2
13104	13100	13107	-	13957	-	-	+	2	1	3
13111	13108	13111	-	14775	-	-	+	2	0	2
13127	13124	13129	-	14591	-	-	+	2	0	2
13141	13139	13145	-	13318	-	-	-	2	3	5
13141	13139	13145	-	14269	-	-	+	2	1	3
13149	13146	13152	-	13553	13553	13554	+	2	1	3
13149	13146	13152	-	14347	-	-	+	2	2	4
13164	13161	13168	-	14517	-	-	+	2	0	2
13164	13161	13168	-	14605	-	-	+	2	0	2
13174	13169	13178	-	14247	14247	14251	+	2	3	5
13174	13169	13178	-	14289	-	-	+	2	2	4
13174	13169	13178	-	15007	-	-	+	2	0	2
13199	13199	13201	-	13646	-	-	+	2	2	4
13207	13203	13210	-	13983	-	-	-	2	0	2
13224	13220	13228	-	14714	-	-	-	2	0	2
13224	13220	13228	-	15078	15078	15079	+	2	2	4
13224	13220	13228	-	15176	15174	15176	+	2	1	3
13237	13234	13237	+	15075	-	-	+	2	2	4
13246	13240	13246	-	13518	-	-	-	2	0	2
13252	13247	13256	-	14289	-	-	+	2	2	4
13258	13258	13260	-	14481	-	-	-	2	2	4
13265	13261	13268	-	13404	-	-	-	2	2	4
13265	13261	13268	-	14599	-	-	+	2	0	2
13265	13261	13268	-	14620	-	-	+	2	0	2
13272	13269	13276	-	14715	-	-	+	2	1	3
13278	13277	13282	-	13408	-	-	+	2	2	4
13285	-	-	+	14461	-	-	+	2	2	4
13288	13287	13289	-	13404	-	-	-	2	0	2
13293	-	-	+	13814	-	-	+	2	0	2
13298	13296	13302	-	14256	14256	14259	-	2	4	6
13308	13303	13313	-	13557	-	-	+	2	0	2
13308	13303	13313	-	14868	14867	14870	+	2	3	5
13308	13303	13313	-	14892	14892	14895	-	2	1	3
13308	13303	13313	-	15040	-	-	-	2	17	19
13308	13303	13313	-	15215	15215	15216	+	2	3	5
13316	13315	13320	-	13744	-	-	-	2	0	2
13316	13315	13320	-	14538	-	-	+	2	2	4
13316	13315	13320	-	14552	14551	14552	+	2	1	3
13330	13324	13334	-	14401	14400	14401	+	2	1	3
13339	13335	13341	-	14487	-	-	+	2	0	2
13339	13335	13341	-	14705	-	-	+	2	3	5
13365	13364	13367	-	14515	-	-	-	2	0	2
13377	13376	13379	-	14732	-	-	+	2	0	2
13377	13376	13379	-	14968	-	-	+	2	1	3
13385	13381	13388	-	13695	13695	13696	+	2	3	5
13385	13381	13388	-	15250	-	-	-	2	0	2
13394	13392	13395	-	13507	-	-	+	2	2	4
13394	13392	13395	-	13700	-	-	-	2	0	2
13408	13404	13411	-	13739	-	-	-	2	1	3
13410	13408	13410	+	13432	13429	13432	+	2	0	2
13416	13413	13420	-	14524	14522	14524	+	2	1	3
13431	13428	13435	-	13758	-	-	-	2	0	2
13440	13438	13440	-	13727	13726	13727	-	2	3	5
13451	13448	13454	-	14095	-	-	+	2	0	2
13451	13448	13454	-	14307	-	-	+	2	0	2
13456	-	-	-	14529	-	-	+	2	0	2
13462	13457	13464	-	13660	-	-	-	2	0	2
13462	13457	13464	-	13692	-	-	-	2	0	2
13462	13457	13464	-	13772	13772	13775	+	2	3	5
13462	13457	13464	-	13812	-	-	-	2	0	2
13473	13468	13474	-	14034	-	-	+	2	6	8
13479	13475	13484	-	14638	-	-	+	2	0	2
13483	13483	13484	+	13597	-	-	-	2	0	2
13488	13485	13491	-	13881	13881	13882	+	2	3	5
13498	13495	13504	-	14370	14368	14370	+	2	1	3
13498	13495	13504	-	14651	-	-	-	2	0	2
13498	13495	13504	-	14723	-	-	+	2	2	4
13498	13495	13504	-	14923	-	-	+	2	0	2
13507	13507	13512	-	14426	-	-	+	2	1	3
13507	13504	13508	+	14971	-	-	+	2	1	3
13517	13516	13518	+	14030	-	-	-	2	2	4
13523	13522	13527	-	14377	14377	14378	+	2	11	13
13549	13547	13553	-	14488	-	-	-	2	4	6
13555	13554	13555	-	13891	-	-	+	2	1	3
13562	13556	13567	-	14678	14678	14679	-	2	1	3

13569	13568	13572	-	14789	-	-	-	2	0	2
13579	13575	13584	-	14188	-	-	+	2	0	2
13579	13578	13582	+	14456	-	-	+	2	0	2
13587	13586	13589	-	14282	-	-	+	2	0	2
13594	13593	13595	-	14193	-	-	+	2	2	4
13594	13593	13595	-	14391	-	-	+	2	0	2
13594	13593	13595	-	14960	-	-	+	2	2	4
13604	13599	13604	-	14201	14199	14201	-	2	1	3
13604	13599	13604	-	14272	-	-	-	2	0	2
13604	13599	13604	-	14294	-	-	+	2	4	6
13604	13599	13604	-	14410	14410	14411	+	2	6	8
13604	13599	13604	-	14676	-	-	-	2	0	2
13610	13605	13611	-	14668	14668	14669	+	2	0	2
13610	13605	13611	-	14757	-	-	+	2	4	6
13616	13612	13619	-	14325	-	-	+	2	2	4
13616	13612	13619	-	14669	14668	14669	+	2	3	5
13616	13612	13619	-	14920	14917	14920	-	2	1	3
13623	13620	13623	-	14731	14730	14731	+	2	1	3
13629	13625	13631	-	14438	14435	14439	+	2	3	5
13629	13625	13631	-	14537	14535	14537	+	2	2	4
13629	13625	13631	-	14679	-	-	-	2	2	4
13629	13625	13631	-	14688	-	-	-	2	0	2
13634	13632	13635	-	13775	13775	13777	+	2	6	8
13645	13644	13649	-	13799	13798	13799	-	2	1	3
13645	13644	13649	-	14874	-	-	+	2	3	5
13656	13654	13656	-	14599	-	-	+	2	0	2
13663	13657	13666	-	14436	14436	14437	+	2	2	4
13673	13669	13675	-	13937	-	-	+	2	4	6
13678	13676	13680	-	14435	-	-	-	2	1	3
13690	13690	13691	-	14496	-	-	+	2	0	2
13700	13696	13701	-	13856	13853	13856	+	2	0	2
13700	13696	13701	-	14056	-	-	+	2	2	4
13700	13696	13701	-	14562	-	-	+	2	0	2
13709	13703	13713	-	13863	-	-	-	2	0	2
13709	13703	13713	-	14435	-	-	-	2	0	2
13709	13703	13713	-	14660	-	-	-	2	1	3
13720	13718	13723	-	14369	-	-	+	2	1	3
13720	13718	13723	-	14522	14522	14523	-	2	2	4
13728	13728	13731	-	14939	-	-	+	2	1	3
13733	13733	13736	-	14563	-	-	+	2	0	2
13733	13733	13736	-	14808	-	-	+	2	0	2
13738	13738	13742	+	14534	14534	14535	+	2	0	2
13738	13738	13742	+	14563	14563	14564	-	2	0	2
13757	13756	13761	-	14337	14335	14337	-	2	0	2
13779	13776	13783	-	14291	-	-	+	2	0	2
13780	-	-	+	14315	-	-	+	2	0	2
13786	13786	13787	-	14523	-	-	+	2	0	2
13791	13789	13797	-	14154	-	-	-	2	0	2
13791	13789	13797	-	15219	-	-	-	2	2	4
13799	13799	13804	-	14401	-	-	+	2	4	6
13810	13807	13815	-	14146	-	-	+	2	0	2
13829	13829	13833	+	15143	-	-	+	2	0	2
13830	13826	13834	-	14369	-	-	+	2	3	5
13848	13843	13849	-	14538	-	-	-	2	2	4
13855	13850	13859	-	14255	-	-	-	2	0	2
13862	13861	13862	+	14260	-	-	+	2	0	2
13863	13861	13866	-	14341	-	-	+	2	0	2
13863	13861	13866	-	14996	14992	14996	-	2	1	3
13870	13867	13871	-	14239	-	-	+	2	0	2
13870	13867	13871	-	14339	-	-	-	2	2	4
13870	13867	13871	-	15249	-	-	-	2	0	2
13876	13872	13879	-	14202	-	-	+	2	0	2
13876	13872	13879	-	14675	14672	14675	-	2	2	4
13878	13875	13882	+	15067	-	-	+	2	1	3
13891	13887	13891	+	13984	-	-	+	2	0	2
13893	13893	13895	-	15110	-	-	-	2	0	2
13898	13897	13899	+	14312	-	-	+	2	2	4
13908	13906	13912	-	15165	-	-	+	2	0	2
13910	13907	13910	+	14048	-	-	+	2	0	2
13914	13913	13917	-	13891	-	-	+	2	0	2
13928	13927	13929	-	14031	-	-	-	2	0	2
13928	13927	13929	-	14037	-	-	-	2	0	2
13936	13932	13940	-	14941	-	-	-	2	2	4
13947	13941	13951	-	14324	-	-	+	2	2	4
13947	13941	13951	-	14730	-	-	+	2	2	4
13947	13941	13951	-	14740	-	-	+	2	0	2
13947	13941	13951	-	14818	-	-	+	2	1	3
13953	13952	13956	-	14315	-	-	+	2	0	2
13967	13964	13968	-	14289	14289	14290	+	2	3	5
13982	13978	13982	-	14247	-	-	+	2	0	2
13982	13978	13982	-	14391	-	-	-	2	0	2
13988	13984	13992	-	14239	-	-	+	2	0	2
13988	13984	13992	-	14707	-	-	+	2	2	4
14001	13996	14005	-	14589	-	-	-	2	0	2
14012	14008	14013	-	14206	-	-	+	2	1	3
14012	14008	14013	-	15156	15155	15156	-	2	6	8
14012	14009	14016	+	14146	-	-	+	2	0	2
14012	14009	14016	+	14192	-	-	+	2	2	4
14012	14009	14016	+	14317	-	-	-	2	0	2
14012	14009	14016	+	14761	-	-	+	2	0	2
14019	14015	14022	-	14061	-	-	-	2	0	2

14019	14015	14022	-	14660 -	-		-	2	1	3
14025	14023	14028	-	14205	14203	14206	-	2	5	7
14028	14025	14032	+	15003 -	-		+	2	0	2
14046	14044	14049	-	14868 -	-		+	2	0	2
14046	14044	14049	-	15061	15061	15062	+	2	3	5
14051	14050	14052	-	14642 -	-		-	2	0	2
14071 -	-		-	14292 -	-		+	2	1	3
14079	14079	14083	-	14691 -	-		-	2	0	2
14085	14085	14087	-	14877	14877	14879	-	2	1	3
14091	14089	14096	-	14136 -	-		+	2	0	2
14092	14089	14096	+	14172 -	-		+	2	0	2
14100	14098	14102	+	14262 -	-		+	2	0	2
14100	14098	14102	+	14627 -	-		+	2	0	2
14108	14106	14112	-	14505 -	-		-	2	0	2
14108	14106	14112	-	15216 -	-		+	2	1	3
14120	14115	14126	-	14234 -	-		-	2	3	5
14122 -	-		+	14168 -	-		-	2	0	2
14130	14129	14130	-	14196 -	-		-	2	3	5
14137	14133	14138	+	14171 -	-		-	2	0	2
14138	14134	14142	-	14223	14223	14224	-	2	2	4
14138	14134	14142	-	14427	14427	14428	+	2	2	4
14138	14134	14142	-	14443 -	-		-	2	2	4
14138	14134	14142	-	14944 -	-		+	2	1	3
14145	14145	14148	-	14625 -	-		-	2	0	2
14152	14151	14156	+	14174 -	-		-	2	0	2
14152	14151	14156	+	14375 -	-		-	2	0	2
14154	14150	14156	-	14197	14197	14198	-	2	7	9
14159	14157	14160	-	14848 -	-		+	2	2	4
14164	14161	14168	-	14752	14751	14752	-	2	4	6
14171	14169	14171	+	14138 -	-		-	2	0	2
14174	14174	14180	-	14382 -	-		+	2	2	4
14174	14174	14180	-	14775	14775	14776	+	2	2	4
14174	14174	14180	-	15253	15253	15256	+	2	3	5
14180	14177	14183	+	14210 -	-		-	2	0	2
14187	14182	14187	-	14643	14643	14644	+	2	0	2
14187	14182	14187	-	14820	14818	14820	+	2	1	3
14187	14182	14187	-	15089 -	-		+	2	0	2
14199 -	-		+	15322 -	-		-	2	0	2
14212	14210	14216	-	14493 -	-		+	2	1	3
14219	14215	14222	+	14705 -	-		+	2	3	5
14233	14231	14233	-	14773 -	-		+	2	9	11
14238	14234	14240	-	14730	14730	14731	+	2	5	7
14243	14241	14243	-	14385 -	-		+	2	0	2
14245 -	-		+	15147 -	-		-	2	2	4
14248	14244	14252	-	14712 -	-		+	2	0	2
14248	14244	14252	-	14842 -	-		+	2	6	8
14248	14244	14252	-	15042 -	-		-	2	0	2
14267	14263	14270	-	14757 -	-		+	2	11	13
14267	14263	14270	-	14925	14925	14926	+	2	2	4
14293	14293	14296	+	14325	14322	14325	-	2	4	6
14294	14291	14295	-	14873 -	-		+	2	2	4
14300	14298	14301	-	14402 -	-		-	2	2	4
14316	14311	14318	-	14831	14829	14831	+	2	1	3
14316	14311	14318	-	15076	15076	15077	+	2	2	4
14330	14330	14334	+	14343 -	-		+	2	0	2
14330	14330	14334	+	14841 -	-		+	2	1	3
14330	14330	14334	+	15122	15118	15122	-	2	7	9
14331	14328	14337	-	14435	14435	14436	+	2	0	2
14331	14328	14337	-	14438 -	-		-	2	1	3
14342	14339	14346	-	14462 -	-		-	2	0	2
14342	14339	14346	-	14553 -	-		+	2	0	2
14342	14339	14346	-	14881 -	-		+	2	0	2
14360	14355	14365	-	14675 -	-		+	2	0	2
14360	14355	14365	-	14777	14777	14778	-	2	5	7
14360	14355	14365	-	14777 -	-		+	2	2	4
14360	14355	14365	-	14859 -	-		+	2	0	2
14360	14355	14365	-	15037	15037	15038	-	2	4	6
14369	14367	14371	-	14438	14438	14439	+	2	0	2
14369	14367	14371	-	14867 -	-		+	2	0	2
14370	14366	14373	+	14771 -	-		+	2	1	3
14370	14366	14373	+	14877 -	-		+	2	0	2
14370	14366	14373	+	14980 -	-		-	2	0	2
14375	14374	14381	+	14889 -	-		+	2	0	2
14377	14372	14382	-	14785	14785	14786	-	2	0	2
14377	14372	14382	-	14889	14887	14889	-	2	1	3
14385	14383	14388	-	15074 -	-		-	2	0	2
14391	14390	14393	-	14986 -	-		+	2	0	2
14396	14395	14397	-	15037 -	-		-	2	0	2
14403	14399	14406	-	14948 -	-		+	2	2	4
14408	14407	14409	-	14563 -	-		-	2	0	2
14408 -	-		+	14452	14448	14452	-	2	0	2
14422	14420	14425	+	15266	15266	15268	+	2	1	3
14435	14434	14439	-	14808 -	-		+	2	1	3
14437	14432	14442	+	15051	15050	15051	-	2	1	3
14443	14442	14447	-	15042 -	-		-	2	0	2
14451	14449	14451	-	14602 -	-		+	2	3	5
14451	14449	14451	-	15384 -	-		-	2	0	2
14452	14451	14455	+	14535 -	-		-	2	0	2
14456	14452	14461	-	14897 -	-		-	2	9	11
14456	14452	14461	-	15177 -	-		+	2	0	2
14462	14458	14465	+	14897	14895	14897	+	2	4	6

14462	14458	14465	+	14902	14900	14902	+	2	6	8
14464	14463	14470	-	15057	15055	15057	+	2	2	4
14464	14463	14470	-	15202	-	-	+	2	4	6
14472	14472	14473	-	14892	-	-	-	2	2	4
14479	14475	14482	-	14542	-	-	+	2	0	2
14479	14475	14482	-	14891	14891	14892	-	2	0	2
14489	14484	14493	-	14630	-	-	+	2	2	4
14489	14484	14493	-	14646	-	-	-	2	0	2
14489	14484	14493	-	14664	-	-	-	2	0	2
14489	14484	14493	-	14836	14835	14836	+	2	1	3
14518	14510	14522	-	14923	-	-	+	2	1	3
14526	14524	14530	-	14675	-	-	-	2	5	7
14532	14531	14536	+	14641	-	-	-	2	0	2
14532	14531	14536	+	15161	-	-	+	2	0	2
14547	14547	14550	-	14752	-	-	+	2	3	5
14553	-	-	-	15010	-	-	+	2	2	4
14559	14555	14563	-	14641	-	-	-	2	0	2
14559	14555	14563	-	15065	15061	15065	+	2	1	3
14565	14564	14568	-	14662	-	-	+	2	0	2
14565	14564	14568	-	14764	-	-	+	2	0	2
14565	14564	14568	-	14784	-	-	-	2	0	2
14573	14571	14573	+	15011	-	-	+	2	0	2
14586	14584	14590	-	14779	-	-	+	2	5	7
14589	14584	14594	+	15037	-	-	-	2	1	3
14595	14592	14597	-	15115	-	-	-	2	0	2
14607	14602	14610	-	14825	-	-	-	2	2	4
14612	14611	14615	-	14897	14894	14897	-	2	0	2
14612	14611	14615	-	15061	-	-	-	2	0	2
14632	14631	14632	+	15039	-	-	+	2	0	2
14633	14632	14635	-	15037	15037	15038	+	2	8	10
14633	14632	14635	-	15043	-	-	+	2	4	6
14638	14638	14641	-	14998	-	-	-	2	0	2
14638	14638	14641	-	15043	-	-	+	2	8	10
14638	14634	14642	+	14741	-	-	+	2	0	2
14656	14652	14661	+	14668	-	-	-	2	0	2
14673	14671	14674	-	14777	-	-	+	2	2	4
14673	14671	14674	-	15057	-	-	+	2	6	8
14676	14672	14677	+	14914	-	-	-	2	2	4
14703	14701	14708	-	14792	-	-	-	2	0	2
14705	14705	14708	+	14882	-	-	-	2	3	5
14727	14724	14728	+	14872	-	-	-	2	0	2
14732	14727	14737	-	15055	-	-	+	2	3	5
14732	14727	14737	-	15322	-	-	-	2	0	2
14739	14738	14742	+	15048	-	-	+	2	3	5
14745	-	-	+	15042	-	-	+	2	0	2
14745	-	-	+	15283	-	-	+	2	2	4
14748	14743	14752	-	14926	-	-	+	2	3	5
14772	14771	14773	-	15194	-	-	-	2	1	3
14786	14782	14790	-	15198	15197	15198	-	2	1	3
14789	14785	14794	+	14818	14815	14818	-	2	0	2
14805	14800	14806	+	14876	-	-	-	2	0	2
14806	14802	14807	-	15153	-	-	+	2	4	6
14806	14802	14807	-	15181	-	-	-	2	4	6
14811	14810	14815	-	14871	-	-	+	2	0	2
14811	14810	14815	-	14916	-	-	+	2	0	2
14838	14834	14843	+	15024	-	-	-	2	0	2
14847	14843	14850	-	15061	-	-	+	2	0	2
14854	14851	14855	-	15173	-	-	-	2	3	5
14864	14863	14864	-	15174	-	-	+	2	1	3
14869	14865	14872	-	15004	15004	15008	-	2	2	4
14869	14865	14872	-	15091	-	-	-	2	0	2
14877	14873	14878	-	15015	15015	15019	-	2	1	3
14877	14873	14878	-	15208	-	-	-	2	0	2
14877	14875	14882	+	15044	15044	15046	+	2	2	4
14877	14875	14882	+	15072	15072	15074	+	2	5	7
14877	14875	14882	+	15185	-	-	+	2	0	2
14882	14879	14886	-	15032	15030	15032	+	2	0	2
14902	14899	14908	-	15079	-	-	+	2	0	2
14902	14899	14908	-	15099	-	-	-	2	2	4
14902	14899	14908	-	15120	15117	15120	+	2	0	2
14910	14909	14911	+	14990	-	-	+	2	0	2
14916	14912	14918	-	15032	-	-	-	2	0	2
14916	14912	14918	-	15085	-	-	-	2	0	2
14916	14912	14918	-	15144	-	-	+	2	0	2
14922	14921	14926	+	14943	14940	14943	+	2	5	7
14922	14921	14926	+	14957	14956	14957	+	2	3	5
14928	14927	14928	-	15211	-	-	-	2	0	2
14928	14928	14933	+	14951	-	-	+	2	2	4
14933	14932	14936	-	15089	-	-	-	2	0	2
14947	14942	14953	-	15135	15135	15138	-	2	1	3
14951	14948	14955	+	14977	-	-	+	2	2	4
14951	14948	14955	+	14990	-	-	+	2	0	2
14951	14948	14955	+	15023	15023	15027	+	2	2	4
14957	14956	14960	-	15092	-	-	-	2	1	3
14957	14956	14960	-	15143	-	-	+	2	4	6
14960	14956	14964	+	15015	15015	15016	+	2	2	4
14964	14962	14968	-	15030	-	-	+	2	0	2
14964	14962	14968	-	15034	-	-	-	2	0	2
14964	14962	14968	-	15058	-	-	-	2	0	2
14970	14969	14974	-	15118	-	-	+	2	2	4
14970	14969	14974	-	15169	-	-	-	2	2	4

14978	14974	14978	+	15010 -	-		+	2	0	2
14991	14991	14994	-	15384 -	-		-	2	0	2
15002	14998	15003	-	15052	15051	15053	-	2	6	8
15004	15000	15010	+	15041 -	-		+	2	1	3
15004	15000	15010	+	15101 -	-		-	2	0	2
15020	15019	15021	-	15165 -	-		+	2	0	2
15025	15023	15027	-	15206 -	-		-	2	2	4
15037	15033	15040	+	15064	15064	15065	-	2	0	2
15037	15033	15040	+	15096 -	-		-	2	0	2
15047	15043	15050	+	15075 -	-		-	2	0	2
15056	15053	15060	-	15185 -	-		-	2	0	2
15063	15058	15066	+	15083 -	-		+	2	2	4
15063	15058	15066	+	15121 -	-		+	2	2	4
15068 -	-		+	15086 -	-		-	2	0	2
15073	15073	15076	+	15086 -	-		-	2	0	2
15078	15077	15085	-	15253 -	-		+	2	3	5
15081	15077	15081	+	15165	15162	15165	-	2	0	2
15086	15082	15086	+	15119 -	-		-	2	0	2
15086	15082	15086	+	15129 -	-		-	2	0	2
15090	15087	15093	-	15129 -	-		-	2	15	17
15102	15098	15105	+	15158 -	-		-	2	0	2
15124	15121	15128	+	15168 -	-		-	2	0	2
15142	15142	15145	-	15237 -	-		+	2	2	4
15148	15147	15152	+	15111 -	-		-	2	1	3
15177	15176	15180	+	15186 -	-		-	2	0	2
15180 -	-		-	15310 -	-		-	2	0	2
15182	15181	15183	+	15195	15195	15196	+	2	0	2
15182	15181	15183	+	15203 -	-		+	2	0	2
15189	15184	15192	+	15169	15169	15170	-	2	2	4
15189	15184	15192	+	15235 -	-		+	2	0	2
15189	15184	15192	+	15284 -	-		+	2	0	2
15189	15184	15192	+	15289	15287	15289	+	2	1	3
15192	15189	15192	-	15255 -	-		-	2	4	6
15212	15211	15212	+	15227 -	-		+	2	0	2
15217	15215	15222	+	15216 -	-		-	2	0	2
15241	15241	15245	-	15275 -	-		+	2	0	2
15267	15266	15272	-	15320 -	-		-	2	2	4
15290	15290	15295	-	15348	15348	15349	+	2	0	2
1 -	-		-	14984 -	-		+	1	2	3
1 -	-		-	15079	15079	15081	+	1	4	5
23	23	24	+	14312 -	-		-	1	7	8
226	222	226	-	829 -	-		-	1	1	2
287	284	291	+	14447	14447	14449	+	1	24	25
300	300	302	+	13044	13044	13047	+	1	8	9
355	350	358	-	6685	6682	6688	+	1	6	7
388	386	391	+	14349 -	-		+	1	1	2
480	480	482	+	13495 -	-		+	1	1	2
480	480	482	+	15078 -	-		+	1	2	3
511	509	511	+	522	521	525	+	1	4	5
563 -	-		+	14189 -	-		-	1	2	3
588	588	589	+	11874	11874	11875	+	1	1	2
608 -	-		+	13683 -	-		+	1	3	4
620	618	622	+	13885 -	-		+	1	2	3
642	642	646	-	795 -	-		-	1	1	2
642	642	646	-	14882 -	-		+	1	1	2
667	667	668	+	13123	13123	13124	+	1	4	5
699	696	703	+	13939 -	-		+	1	1	2
705	704	707	+	10976 -	-		+	1	1	2
705	704	707	+	12872 -	-		+	1	2	3
715	712	716	+	14666 -	-		+	1	1	2
722	722	725	+	12945 -	-		+	1	2	3
764	760	765	+	911 -	-		+	1	1	2
764	760	765	+	13872	13872	13876	+	1	8	9
770	769	775	+	13069 -	-		+	1	5	6
770	769	775	+	13404 -	-		+	1	2	3
797	797	798	+	911 -	-		+	1	1	2
805 -	-		-	13458 -	-		+	1	1	2
814	812	818	+	14199	14199	14200	+	1	1	2
838 -	-		-	9136 -	-		-	1	4	5
849	848	849	+	13258 -	-		+	1	1	2
862	861	862	+	898 -	-		+	1	1	2
921	918	921	-	1093 -	-		-	1	1	2
929 -	-		+	9869 -	-		+	1	1	2
935	935	937	+	11239	11237	11239	+	1	1	2
957	954	960	+	12339 -	-		+	1	1	2
1031	1031	1032	+	14609 -	-		+	1	1	2
1038	1035	1041	+	12040 -	-		+	1	1	2
1038	1035	1041	+	14381 -	-		+	1	1	2
1052	1049	1056	+	12069	12069	12071	+	1	2	3
1066	1066	1068	+	12802 -	-		+	1	1	2
1066	1066	1068	+	14025 -	-		+	1	1	2
1082	1078	1082	+	14710	14710	14711	+	1	1	2
1106	1106	1109	+	14966	14966	14970	+	1	2	3
1115 -	-		+	14226 -	-		+	1	1	2
1115 -	-		+	14488 -	-		+	1	1	2
1139	1137	1139	-	1328 -	-		-	1	1	2
1140	1138	1143	+	14454 -	-		+	1	1	2
1140	1138	1143	+	14508 -	-		+	1	1	2
1145	1142	1149	-	13946	13945	13946	+	1	4	5
1170	1168	1170	+	14411 -	-		+	1	1	2
1223	1222	1228	+	12147 -	-		+	1	1	2

1228	1226	1230	-	1434 -	-		-	1	1	2
1228	1226	1230	-	1549 -	-		-	1	1	2
1228	1226	1230	-	5977	5977	5979	+	1	2	3
1241	1240	1242	+	14405 -	-		+	1	2	3
1363	1363	1364	-	1489	1489	1490	-	1	2	3
1372	1370	1372	+	13965 -	-		+	1	1	2
1434	1431	1438	+	1464 -	-		+	1	1	2
1448	1444	1449	-	13854	13854	13855	-	1	1	2
1496	1496	1497	+	14368	14368	14369	-	1	1	2
1537	1537	1541	+	14222 -	-		+	1	2	3
1611 -	-		+	1705 -	-		+	1	1	2
1616	1615	1617	+	14107	14107	14108	-	1	31	32
1653	1653	1656	-	14823 -	-		-	1	2	3
1679 -	-		-	1873 -	-		-	1	1	2
1681 -	-		+	1808 -	-		+	1	1	2
1693	1693	1694	+	13663 -	-		+	1	1	2
1741	1740	1741	-	15304	15304	15305	-	1	1	2
1769	1769	1773	+	1811	1809	1811	+	1	1	2
1809	1808	1811	+	14118	14117	14118	+	1	2	3
1809	1808	1811	+	14302	14300	14302	+	1	1	2
1835	1830	1836	+	8909 -	-		+	1	1	2
1835	1830	1836	+	10051 -	-		+	1	2	3
1835	1830	1836	+	12060	12060	12061	+	1	1	2
1835	1830	1836	+	12487 -	-		+	1	1	2
1840	1838	1840	-	11727	11725	11727	-	1	1	2
1840 -	-		+	12604 -	-		+	1	1	2
1858 -	-		+	11356 -	-		+	1	1	2
1885	1882	1885	+	12636	12635	12636	+	1	2	3
1926	1926	1930	-	1957 -	-		+	1	1	2
1959	1953	1963	+	11521	11521	11522	-	1	1	2
1979 -	-		+	13941 -	-		+	1	2	3
1984	1982	1988	+	2115	2115	2118	+	1	2	3
1984	1982	1988	+	13516 -	-		+	1	4	5
1996	1996	1997	-	6996	6996	6997	+	1	1	2
2000	2000	2002	+	3077 -	-		+	1	1	2
2014	2011	2014	-	12493	12490	12493	-	1	1	2
2016	2013	2019	+	14623 -	-		+	1	1	2
2037	2035	2038	-	2308	2308	2309	-	1	1	2
2037	2034	2038	+	13719	13719	13720	+	1	1	2
2042	2040	2045	-	2095 -	-		-	1	1	2
2043	2043	2047	+	11281 -	-		+	1	2	3
2075	2071	2076	+	15156 -	-		+	1	1	2
2083 -	-		-	14000 -	-		+	1	1	2
2099	2095	2101	+	14033	14033	14034	+	1	6	7
2123	2123	2124	-	10975 -	-		+	1	2	3
2125 -	-		+	14524	14524	14525	-	1	6	7
2155	2152	2155	+	12911 -	-		+	1	1	2
2168	2164	2168	-	14848 -	-		+	1	1	2
2168	2166	2168	+	14448 -	-		+	1	3	4
2179	2177	2183	+	2953	2953	2957	+	1	1	2
2179	2177	2183	+	14632 -	-		+	1	1	2
2190	2186	2194	+	2430	2426	2430	+	1	1	2
2222 -	-		+	13587 -	-		+	1	1	2
2249	2248	2252	+	12743 -	-		+	1	5	6
2272	2272	2274	+	12808 -	-		+	1	1	2
2325	2322	2325	+	13610 -	-		+	1	1	2
2325	2322	2325	+	13704 -	-		+	1	1	2
2343	2341	2345	+	12229	12229	12230	+	1	1	2
2343	2341	2345	+	12905	12902	12905	+	1	1	2
2343	2341	2345	+	13639 -	-		+	1	1	2
2362	2361	2364	+	14177 -	-		+	1	1	2
2362	2361	2364	+	14561 -	-		+	1	1	2
2397	2396	2401	+	10862	10862	10863	+	1	1	2
2397	2396	2401	+	12846	12844	12846	+	1	1	2
2397	2396	2401	+	13543 -	-		+	1	2	3
2401	2397	2404	-	3110 -	-		-	1	1	2
2414	2412	2416	+	12759 -	-		+	1	1	2
2436	2434	2437	-	2622 -	-		-	1	1	2
2444 -	-		+	14198 -	-		+	1	3	4
2448	2448	2451	-	14389	14386	14389	+	1	1	2
2455	2452	2459	+	13282 -	-		+	1	1	2
2473	2470	2473	+	13123 -	-		+	1	3	4
2508	2506	2508	+	9718	9718	9719	+	1	1	2
2553	2553	2554	+	14199 -	-		+	1	4	5
2560	2558	2561	+	13940 -	-		+	1	3	4
2583	2582	2583	+	14259 -	-		+	1	3	4
2583	2582	2583	+	14751 -	-		+	1	1	2
2588	2588	2590	+	13010 -	-		+	1	1	2
2588	2588	2590	+	13798	13796	13798	+	1	1	2
2608	2607	2608	+	13013 -	-		+	1	2	3
2620	2620	2622	+	12754 -	-		+	1	1	2
2638	2638	2641	+	12270	12270	12271	+	1	1	2
2638	2638	2641	+	12450	12450	12451	+	1	4	5
2734	2733	2736	+	14401	14401	14402	+	1	1	2
2768	2764	2771	+	9219 -	-		+	1	1	2
2845	2845	2847	+	14539 -	-		+	1	2	3
2855	2855	2857	+	14878 -	-		-	1	1	2
2879	2879	2883	-	15143 -	-		+	1	1	2
2897	2897	2898	+	13721	13721	13722	+	1	1	2
2915	2913	2915	+	12275 -	-		+	1	3	4
2932	2929	2932	-	9255 -	-		+	1	1	2

2938	2938	2942	-	3022 -	-		-	1	1	2
2992 -	-		+	15010 -	-		+	1	1	2
3149	3149	3150	+	14759	14759	14760	+	1	4	5
3154	3153	3154	+	14128 -	-		+	1	1	2
3154	3153	3154	+	14818	14818	14819	+	1	12	13
3157	3152	3157	-	3220 -	-		+	1	1	2
3192 -	-		-	6308 -	-		-	1	1	2
3220	3219	3222	-	3556	3556	3557	-	1	1	2
3220	3219	3222	+	11445	11445	11446	+	1	1	2
3227	3226	3232	+	3217	3217	3220	+	1	2	3
3227	3226	3232	+	10823 -	-		+	1	2	3
3227	3226	3232	+	12240 -	-		+	1	1	2
3258	3256	3259	+	13126 -	-		+	1	1	2
3263	3259	3263	-	14779	14779	14780	+	1	3	4
3263 -	-		+	11518 -	-		+	1	1	2
3272	3270	3275	+	11711	11711	11712	+	1	1	2
3281	3280	3285	-	14612 -	-		+	1	1	2
3307 -	-		-	14730 -	-		+	1	1	2
3341	3339	3345	+	10938	10938	10939	+	1	1	2
3341	3339	3345	+	12272	12268	12272	+	1	1	2
3341	3339	3345	+	12329 -	-		+	1	1	2
3341	3339	3345	+	12868 -	-		+	1	2	3
3341	3339	3345	+	14308 -	-		+	1	1	2
3366	3362	3366	+	13721 -	-		+	1	1	2
3366	3362	3366	+	15053	15053	15054	+	1	1	2
3373	3370	3379	+	14650	14650	14652	+	1	1	2
3401	3398	3403	+	14759	14759	14761	+	1	7	8
3448	3446	3452	+	13010 -	-		+	1	1	2
3467 -	-		+	15055 -	-		+	1	1	2
3480	3476	3480	+	10206 -	-		+	1	1	2
3517	3515	3518	+	11173 -	-		+	1	10	11
3538	3537	3539	-	3728 -	-		-	1	1	2
3538	3537	3539	-	15129	15129	15130	-	1	3	4
3541	3541	3544	+	11659 -	-		+	1	1	2
3565	3565	3567	+	13297	13297	13299	+	1	3	4
3628	3628	3629	+	10726	10726	10727	+	1	1	2
3640	3640	3643	+	3660 -	-		+	1	1	2
3689	3689	3690	-	3884	3884	3885	-	1	1	2
3690	3686	3693	+	3933 -	-		+	1	1	2
3690	3686	3693	+	12474 -	-		+	1	1	2
3721	3719	3724	-	7317	7315	7317	+	1	3	4
3760	3759	3760	-	15216 -	-		-	1	1	2
3786 -	-		+	13404 -	-		+	1	1	2
3867	3865	3867	-	4026 -	-		-	1	1	2
3882	3882	3884	-	14448	14446	14448	+	1	1	2
3987	3986	3990	+	14241 -	-		+	1	1	2
3993 -	-		+	14193 -	-		-	1	1	2
4015	4015	4017	+	13346	13346	13347	+	1	1	2
4027 -	-		-	15357	15357	15358	-	1	1	2
4075	4072	4075	+	14009	14009	14010	+	1	1	2
4167	4164	4168	-	15046 -	-		+	1	2	3
4198	4198	4200	-	13740 -	-		+	1	1	2
4218	4218	4220	+	13596 -	-		+	1	1	2
4279	4279	4282	+	12223 -	-		+	1	1	2
4309	4306	4309	+	10819 -	-		+	1	2	3
4318 -	-		+	12838 -	-		+	1	1	2
4393	4393	4394	+	12115	12115	12116	+	1	1	2
4421	4420	4421	+	10663 -	-		-	1	1	2
4434	4431	4434	-	14730 -	-		+	1	1	2
4438	4434	4442	+	13450	13450	13452	-	1	2	3
4438	4434	4442	+	13567	13567	13568	+	1	1	2
4438	4434	4442	+	14676	14676	14678	+	1	1	2
4445	4445	4446	+	14447	14447	14448	+	1	1	2
4484	4484	4488	+	11919 -	-		+	1	2	3
4545	4545	4550	+	7295	7295	7296	+	1	1	2
4591	4588	4592	+	13837 -	-		+	1	3	4
4598	4598	4602	-	4986	4986	4987	-	1	1	2
4654 -	-		+	8572 -	-		+	1	1	2
4662	4658	4666	+	5091 -	-		+	1	1	2
4678	4678	4681	+	12780	12778	12780	+	1	1	2
4678	4678	4681	+	14242 -	-		+	1	11	12
4710	4710	4711	-	4810 -	-		-	1	1	2
4713	4713	4715	+	13869	13869	13871	+	1	2	3
4726 -	-		-	7811 -	-		-	1	1	2
4728	4724	4728	+	14634 -	-		+	1	1	2
4749	4748	4753	+	14408 -	-		+	1	1	2
4764	4764	4766	+	14454 -	-		+	1	6	7
4805	4802	4805	+	14693 -	-		+	1	1	2
4834	4833	4835	+	13870 -	-		+	1	1	2
4856	4853	4859	+	13424 -	-		+	1	2	3
4868 -	-		-	9714 -	-		+	1	1	2
4887	4884	4890	+	13029 -	-		+	1	2	3
4887	4884	4890	+	13295	13295	13296	+	1	1	2
4921	4921	4925	+	13093 -	-		+	1	1	2
4926 -	-		-	14610 -	-		-	1	1	2
4944	4939	4947	+	7752	7752	7753	-	1	2	3
4998	4998	5002	-	5192 -	-		-	1	1	2
5096	5092	5097	-	15063	15063	15065	+	1	12	13
5207	5203	5207	+	5221 -	-		+	1	1	2
5246	5246	5247	+	14111 -	-		-	1	1	2
5252	5251	5252	+	12854 -	-		+	1	1	2

5295	5295	5298	+	11442	11440	11442	+	1	1	2
5354	5354	5358	+	13404	-	-	+	1	1	2
5363	-	-	-	14414	-	-	+	1	2	3
5393	5390	5394	+	10572	-	-	+	1	1	2
5408	5408	5412	-	15039	15035	15039	+	1	42	43
5431	5431	5435	-	15202	15199	15202	+	1	1	2
5445	5443	5449	-	15191	15187	15191	+	1	3	4
5581	5581	5582	-	14795	14795	14796	+	1	1	2
5589	-	-	-	15292	-	-	+	1	3	4
5737	-	-	-	13560	-	-	+	1	1	2
5750	-	-	-	10799	-	-	+	1	1	2
5764	-	-	-	5799	-	-	+	1	1	2
5785	5783	5789	+	15190	15190	15191	-	1	1	2
5797	-	-	+	12027	-	-	+	1	1	2
5810	-	-	+	13587	-	-	+	1	1	2
5827	5827	5828	+	12902	-	-	+	1	1	2
5866	5866	5868	-	14871	14869	14871	+	1	3	4
5878	5874	5881	+	14292	-	-	+	1	1	2
6095	6092	6095	+	14439	-	-	-	1	1	2
6191	-	-	+	14033	-	-	+	1	1	2
6228	6226	6233	-	6308	6308	6309	-	1	1	2
6236	6236	6241	-	7067	7067	7068	-	1	1	2
6274	6274	6278	+	11341	-	-	+	1	1	2
6290	6290	6293	+	14141	14138	14141	+	1	1	2
6303	6303	6308	-	13911	13907	13911	+	1	1	2
6352	6349	6352	-	6860	-	-	-	1	1	2
6390	-	-	-	11804	-	-	+	1	2	3
6733	6729	6736	+	10651	-	-	+	1	1	2
6753	-	-	+	15141	-	-	+	1	6	7
6768	-	-	+	13789	-	-	+	1	1	2
6774	-	-	-	15043	-	-	+	1	1	2
6826	-	-	-	13832	-	-	-	1	1	2
6926	-	-	+	14096	-	-	+	1	1	2
6969	-	-	+	7054	-	-	+	1	1	2
7046	-	-	-	13264	-	-	+	1	1	2
7073	-	-	-	14168	-	-	+	1	1	2
7118	7114	7120	-	11332	-	-	+	1	1	2
7133	7133	7134	-	15043	15043	15044	-	1	1	2
7146	7143	7146	+	15105	15105	15106	-	1	5	6
7250	7248	7250	-	14580	-	-	+	1	1	2
7250	7248	7250	-	15251	-	-	+	1	1	2
7345	7342	7345	-	9690	-	-	+	1	1	2
7352	7349	7355	-	14167	14167	14168	+	1	1	2
7365	-	-	-	14288	-	-	+	1	1	2
7467	7464	7467	-	14866	-	-	+	1	1	2
7517	7515	7519	+	13649	-	-	+	1	1	2
7520	-	-	-	15142	-	-	+	1	1	2
7527	-	-	-	14332	-	-	+	1	1	2
7841	7841	7842	+	8048	-	-	+	1	1	2
7852	7852	7855	-	14339	14339	14340	+	1	1	2
7910	-	-	+	15245	-	-	+	1	1	2
7917	7917	7918	-	14848	-	-	+	1	1	2
7922	7922	7923	-	8164	8164	8165	-	1	1	2
8147	8147	8150	-	13128	-	-	+	1	1	2
8149	8146	8149	+	12601	-	-	+	1	2	3
8258	8258	8261	-	11540	-	-	+	1	1	2
8293	-	-	+	14883	-	-	+	1	1	2
8295	8295	8299	-	13486	-	-	+	1	3	4
8308	-	-	-	13587	-	-	+	1	1	2
8332	8332	8333	+	15094	-	-	-	1	1	2
8342	8338	8344	-	15089	15085	15089	+	1	1	2
8349	8348	8351	-	15082	15079	15082	+	1	1	2
8473	-	-	+	8624	-	-	+	1	1	2
8501	-	-	-	10975	-	-	+	1	1	2
8507	-	-	-	14456	-	-	+	1	1	2
8603	-	-	-	11564	-	-	+	1	1	2
8744	8744	8746	+	13514	13514	13516	+	1	3	4
8746	8742	8746	-	13479	-	-	+	1	1	2
8778	-	-	-	12026	-	-	+	1	1	2
8915	8911	8918	-	10742	-	-	+	1	1	2
8974	8971	8977	-	14138	14138	14141	+	1	2	3
9016	9012	9018	-	14774	-	-	+	1	1	2
9029	-	-	-	13167	-	-	+	1	1	2
9061	9060	9065	-	15012	15008	15012	+	1	1	2
9086	9086	9090	+	14144	14144	14145	+	1	1	2
9086	9086	9090	+	14149	-	-	+	1	1	2
9100	9097	9100	-	11847	-	-	+	1	2	3
9147	-	-	-	14783	-	-	+	1	1	2
9209	9206	9213	-	13426	-	-	+	1	1	2
9258	9257	9258	-	10879	10879	10880	+	1	1	2
9273	9270	9277	-	12377	-	-	+	1	3	4
9350	9346	9351	-	13427	-	-	+	1	2	3
9350	9346	9351	-	14334	14331	14334	+	1	1	2
9444	9442	9445	-	11829	11826	11829	+	1	1	2
9462	9462	9464	-	14959	-	-	+	1	2	3
9492	9489	9492	-	10213	-	-	+	1	1	2
9525	-	-	-	14738	-	-	+	1	1	2
9536	-	-	-	14688	-	-	+	1	1	2
9540	9536	9544	+	9752	9749	9752	+	1	2	3
9618	9614	9619	-	14025	-	-	+	1	1	2
9618	9614	9619	-	14376	14376	14377	+	1	1	2

9618	9614	9619	-	14590	14590	14592	+	1	1	2
9816	9815	9817	-	13080	-	-	+	1	2	3
9831	-	-	-	14986	-	-	+	1	2	3
9860	9857	9862	-	13876	-	-	+	1	1	2
9878	9877	9879	-	14776	14776	14777	+	1	2	3
9886	9886	9887	+	14253	14253	14254	+	1	1	2
9889	9885	9890	-	13662	-	-	+	1	2	3
9905	9903	9910	-	13275	13275	13276	+	1	1	2
9905	9903	9910	-	14079	-	-	+	1	1	2
9926	9926	9927	-	13276	13276	13277	+	1	1	2
9991	9989	9992	-	13643	13643	13644	+	1	1	2
10013	10011	10013	-	13299	13297	13299	+	1	1	2
10019	10017	10019	-	12364	-	-	+	1	1	2
10019	10017	10019	-	13928	-	-	+	1	1	2
10023	-	-	+	13662	-	-	+	1	2	3
10024	10023	10026	-	14010	14010	14011	+	1	1	2
10038	10035	10041	-	13350	-	-	-	1	1	2
10058	10058	10060	-	12258	-	-	+	1	3	4
10073	10073	10075	-	11678	-	-	+	1	3	4
10097	10094	10098	-	14708	-	-	+	1	14	15
10097	10094	10098	-	15265	15265	15268	+	1	3	4
10131	10129	10131	-	13416	-	-	+	1	1	2
10151	10147	10151	-	14384	-	-	+	1	1	2
10151	10147	10151	-	15062	15062	15063	+	1	1	2
10182	-	-	+	13218	-	-	+	1	1	2
10278	10276	10282	-	10473	-	-	-	1	1	2
10326	10324	10326	+	10493	10493	10494	+	1	1	2
10332	-	-	-	14594	-	-	+	1	2	3
10370	10367	10374	-	14052	-	-	+	1	1	2
10383	10379	10387	-	13697	-	-	+	1	1	2
10383	10379	10387	-	14524	-	-	+	1	4	5
10400	10397	10400	-	15190	15188	15190	-	1	1	2
10406	10403	10410	-	13445	13441	13445	+	1	1	2
10420	10416	10421	-	15177	-	-	+	1	4	5
10436	10434	10436	-	14921	14921	14923	+	1	2	3
10453	10453	10455	-	13229	13229	13230	+	1	1	2
10485	10481	10486	-	13910	-	-	+	1	1	2
10512	10508	10514	-	15031	-	-	+	1	5	6
10532	10532	10533	-	11423	-	-	+	1	1	2
10571	10568	10573	-	13527	-	-	+	1	1	2
10581	10580	10583	-	13803	-	-	+	1	1	2
10588	10587	10588	-	13444	-	-	+	1	1	2
10630	10630	10632	-	13111	13111	13112	+	1	1	2
10668	10665	10670	-	11379	11379	11380	+	1	1	2
10671	10671	10673	+	12588	-	-	+	1	1	2
10694	10690	10697	-	14292	-	-	+	1	1	2
10699	10698	10699	-	14870	-	-	+	1	3	4
10706	10703	10710	-	14512	-	-	+	1	2	3
10715	10712	10715	-	13952	13952	13953	+	1	4	5
10720	10720	10724	-	15288	-	-	+	1	1	2
10726	10725	10733	-	11156	11156	11157	+	1	1	2
10726	10725	10733	-	14564	-	-	+	1	1	2
10776	10773	10778	-	14632	-	-	+	1	2	3
10787	10787	10791	-	15122	15122	15123	+	1	2	3
10807	10803	10808	-	13044	13044	13045	+	1	1	2
10807	10803	10808	-	14370	-	-	+	1	2	3
10823	10819	10827	-	13518	-	-	+	1	5	6
10847	10844	10850	-	13217	-	-	+	1	1	2
10847	10844	10850	-	14033	-	-	+	1	1	2
10855	10852	10856	-	11850	-	-	-	1	2	3
10855	10852	10856	-	11856	-	-	+	1	1	2
10885	10882	10887	-	13801	13801	13802	+	1	1	2
10911	10908	10911	-	14071	-	-	+	1	1	2
10983	10982	10987	-	13499	-	-	+	1	1	2
10983	10982	10987	-	14687	14683	14687	-	1	1	2
11028	11024	11032	-	13238	-	-	+	1	2	3
11028	11024	11032	-	13334	13331	13334	+	1	1	2
11054	11054	11058	-	13526	-	-	+	1	1	2
11070	11070	11074	-	12344	-	-	+	1	2	3
11070	11070	11074	-	14357	-	-	+	1	1	2
11089	11089	11092	-	12833	12833	12834	+	1	2	3
11089	11089	11092	-	14694	-	-	+	1	1	2
11117	11114	11117	-	13693	-	-	+	1	1	2
11122	11119	11125	-	14170	-	-	+	1	1	2
11202	11201	11202	-	12217	-	-	+	1	1	2
11202	11201	11202	-	12739	-	-	+	1	2	3
11218	11214	11218	-	13344	-	-	+	1	1	2
11228	11226	11234	-	14411	14408	14411	+	1	1	2
11249	11248	11252	-	15080	-	-	+	1	2	3
11287	-	-	-	13939	-	-	-	1	5	6
11294	11293	11298	-	13990	13986	13990	-	1	1	2
11294	11293	11298	-	14134	-	-	+	1	1	2
11319	11317	11320	-	12328	12326	12328	+	1	1	2
11319	11317	11320	-	12858	-	-	+	1	1	2
11331	11331	11336	-	14055	-	-	+	1	1	2
11369	11368	11371	-	13586	-	-	+	1	1	2
11385	-	-	-	13774	-	-	+	1	1	2
11391	11388	11394	-	13042	13042	13043	+	1	1	2
11418	11416	11422	-	13413	-	-	+	1	1	2
11418	11416	11422	-	13880	13880	13881	-	1	2	3
11435	11429	11438	-	11485	-	-	+	1	1	2

11435	11429	11438	-	13499	-	-	+	1	1	2
11435	11429	11438	-	14296	-	-	-	1	3	4
11435	11429	11438	-	14331	14331	14333	+	1	8	9
11446	11442	11446	-	15175	15172	15175	-	1	2	3
11446	11442	11450	+	14376	-	-	+	1	1	2
11452	11448	11456	-	12944	-	-	+	1	1	2
11465	11463	11469	-	13345	-	-	+	1	2	3
11493	11490	11494	-	12497	-	-	+	1	1	2
11511	11510	11513	-	13400	13400	13401	+	1	1	2
11523	11519	11526	-	14804	-	-	+	1	3	4
11551	11548	11554	-	14557	-	-	+	1	2	3
11602	11597	11605	-	14292	-	-	+	1	1	2
11622	11621	11622	-	14400	14400	14401	+	1	2	3
11632	11629	11633	-	14163	-	-	+	1	1	2
11693	11690	11696	-	13608	13605	13608	+	1	1	2
11721	-	-	+	11866	-	-	+	1	1	2
11764	11763	11768	-	12327	-	-	+	1	1	2
11792	11789	11793	-	13247	13247	13248	+	1	6	7
11799	11798	11802	-	13666	-	-	+	1	2	3
11823	11821	11826	-	13393	13393	13395	+	1	2	3
11823	11821	11826	-	13971	-	-	+	1	1	2
11830	11828	11834	-	13985	-	-	+	1	3	4
11836	11836	11840	-	13708	-	-	+	1	1	2
11847	11843	11851	-	13849	-	-	+	1	1	2
11864	11863	11868	-	14187	-	-	+	1	2	3
11873	11870	11877	-	13499	13496	13499	+	1	1	2
11895	11894	11898	-	14695	-	-	+	1	2	3
11909	-	-	-	12820	-	-	-	1	1	2
11920	11915	11924	-	14565	-	-	+	1	5	6
11937	11937	11940	-	14179	14179	14180	+	1	1	2
11965	11963	11965	-	14106	-	-	+	1	1	2
11975	11974	11979	-	13379	-	-	+	1	1	2
11977	11976	11978	+	14162	-	-	+	1	1	2
11984	11984	11986	+	14171	14171	14172	+	1	1	2
11987	11987	11989	-	14706	-	-	+	1	2	3
11996	11996	12000	+	12418	12416	12418	+	1	1	2
11996	11996	12000	+	13266	13263	13266	+	1	1	2
12018	12016	12023	-	12163	12163	12164	+	1	1	2
12026	12024	12030	-	14187	-	-	+	1	2	3
12032	12032	12034	-	13505	-	-	+	1	3	4
12037	12037	12041	-	13312	-	-	+	1	1	2
12048	12045	12049	+	14396	14396	14397	+	1	3	4
12062	12058	12064	-	14964	-	-	+	1	5	6
12070	12068	12072	-	14564	14564	14565	+	1	1	2
12081	12077	12085	+	14395	-	-	+	1	1	2
12087	12085	12087	-	14009	-	-	+	1	2	3
12101	12098	12104	-	13123	-	-	+	1	1	2
12107	12105	12107	-	12333	12329	12333	+	1	3	4
12113	12109	12116	-	12321	12318	12321	-	1	1	2
12113	12109	12116	-	13404	-	-	+	1	1	2
12121	12119	12122	-	14090	-	-	+	1	2	3
12146	12143	12147	-	13944	-	-	+	1	1	2
12146	12143	12147	-	14548	-	-	+	1	2	3
12151	12150	12155	-	14664	14664	14665	+	1	2	3
12161	12159	12162	-	13930	-	-	+	1	1	2
12161	12159	12162	-	14180	-	-	+	1	2	3
12167	12167	12171	-	14871	-	-	+	1	1	2
12187	12184	12190	-	14290	-	-	+	1	1	2
12199	12194	12203	-	14959	-	-	+	1	1	2
12218	12215	12220	-	15135	-	-	-	1	1	2
12225	12225	12228	-	13753	13753	13754	+	1	2	3
12233	12229	12233	-	13827	13827	13829	+	1	2	3
12233	12229	12233	-	14368	-	-	+	1	5	6
12246	12245	12246	-	14689	-	-	+	1	1	2
12257	12252	12259	-	13646	-	-	+	1	1	2
12257	12252	12259	-	13706	-	-	+	1	1	2
12269	12266	12272	-	12419	12419	12420	-	1	1	2
12275	12274	12275	-	12410	12410	12411	-	1	1	2
12282	12282	12283	-	13644	13644	13645	+	1	1	2
12299	12299	12301	-	13124	-	-	+	1	1	2
12314	12313	12317	+	13740	13740	13741	+	1	2	3
12334	12331	12338	-	14577	-	-	-	1	1	2
12341	12340	12346	-	13084	-	-	+	1	1	2
12348	12348	12349	+	15270	15270	15271	+	1	1	2
12356	-	-	+	13957	-	-	+	1	2	3
12376	12372	12376	-	14046	14046	14047	+	1	2	3
12407	12403	12410	-	13901	-	-	+	1	1	2
12407	12403	12410	-	14314	14314	14315	+	1	10	11
12418	12413	12420	-	13411	-	-	+	1	1	2
12423	12422	12426	-	12826	12824	12826	+	1	1	2
12423	12422	12426	-	14025	14025	14026	+	1	3	4
12467	12462	12470	-	13061	13057	13061	+	1	2	3
12467	12462	12470	-	13941	-	-	+	1	1	2
12476	12471	12477	-	13901	-	-	+	1	1	2
12481	12478	12484	-	14777	14777	14778	+	1	1	2
12496	12494	12496	-	13175	13173	13175	+	1	1	2
12517	12517	12518	+	14676	-	-	-	1	1	2
12518	12512	12523	-	13559	-	-	+	1	1	2
12518	12512	12523	-	14193	14189	14193	+	1	1	2
12529	12524	12531	-	13290	13290	13292	+	1	4	5
12529	12524	12531	-	14052	-	-	+	1	1	2

12529	12524	12531	-	14650	14650	14651	+	1	9	10
12536	12535	12540	-	15196 -	-		-	1	2	3
12553	12549	12558	-	13551 -	-		+	1	2	3
12553	12549	12558	-	13792 -	-		+	1	3	4
12568	12565	12568	-	13522 -	-		+	1	1	2
12577	12573	12581	-	13847 -	-		+	1	1	2
12591	12589	12594	-	13921 -	-		+	1	1	2
12591	12589	12594	-	14415 -	-		+	1	1	2
12611	12608	12614	-	14102	14102	14103	+	1	1	2
12611	12608	12614	-	14694 -	-		+	1	1	2
12619	12615	12620	-	14290 -	-		+	1	2	3
12619	12615	12620	-	14808 -	-		+	1	3	4
12641	12636	12645	-	12720	12717	12720	+	1	1	2
12641	12636	12645	-	12728 -	-		+	1	1	2
12641	12636	12645	-	12908	12908	12909	+	1	2	3
12641	12636	12645	-	14403 -	-		+	1	1	2
12658	12654	12662	+	12764 -	-		+	1	1	2
12661	12657	12666	-	13765	13765	13768	+	1	1	2
12661	12657	12666	-	14880	14880	14881	+	1	2	3
12676	12671	12678	-	14599 -	-		-	1	1	2
12696	12692	12697	-	13739 -	-		-	1	2	3
12712	12709	12712	-	14692 -	-		-	1	1	2
12717	12714	12721	-	13163 -	-		+	1	1	2
12717	12714	12721	-	14777 -	-		+	1	1	2
12732	12727	12735	-	14892 -	-		-	1	1	2
12741	12736	12744	-	13168	13164	13168	-	1	1	2
12754	12753	12759	-	13528 -	-		+	1	1	2
12767	12764	12770	+	14638	14635	14638	+	1	1	2
12775	12772	12778	-	14178 -	-		+	1	1	2
12775	12772	12778	-	14552 -	-		+	1	2	3
12775	12772	12778	-	14622 -	-		+	1	1	2
12788	12787	12792	-	13534	13534	13535	+	1	3	4
12802	12799	12802	-	13756	13756	13758	+	1	8	9
12802	12799	12802	-	14247	14247	14249	+	1	1	2
12802	12799	12802	-	14267	14265	14267	+	1	1	2
12813	12811	12816	-	13977 -	-		+	1	2	3
12825	12822	12828	-	13261	13261	13262	+	1	1	2
12825	12822	12828	-	13810 -	-		+	1	4	5
12838	12836	12841	-	14067 -	-		+	1	3	4
12843	12842	12846	-	13960 -	-		+	1	1	2
12864	12861	12869	-	14487	14487	14488	+	1	2	3
12884	12881	12886	-	14503 -	-		+	1	2	3
12884	12881	12886	-	15044	15041	15044	+	1	1	2
12896	12896	12898	-	13082 -	-		-	1	1	2
12896	12896	12898	-	13683 -	-		+	1	1	2
12896	12896	12898	-	14107 -	-		-	1	1	2
12896	12896	12898	-	14283	14283	14284	+	1	2	3
12911	12906	12916	-	14023 -	-		-	1	2	3
12911	12906	12916	-	14748 -	-		+	1	1	2
12921	12919	12925	-	15019 -	-		+	1	6	7
12921	12919	12925	-	15062 -	-		+	1	5	6
12941	12938	12945	-	13951	13951	13952	+	1	5	6
12941	12938	12945	-	14260	14260	14262	+	1	2	3
12941	12938	12945	-	14566 -	-		+	1	6	7
12946	12942	12948	+	15179 -	-		+	1	2	3
12948	12947	12950	-	13261 -	-		-	1	1	2
12948	12947	12950	-	13327 -	-		+	1	3	4
12948	12947	12950	-	13741 -	-		+	1	3	4
12948	12947	12950	-	14634 -	-		+	1	8	9
12976	12975	12979	-	13332 -	-		+	1	1	2
12976	12975	12979	-	14412 -	-		+	1	4	5
12976	12975	12979	-	14734	14734	14738	+	1	1	2
12985	12982	12988	-	14097 -	-		+	1	1	2
12985	12982	12988	-	14572	14568	14572	-	1	8	9
12996	12993	12996	+	13272 -	-		+	1	1	2
13007	13005	13008	-	13440	13440	13443	+	1	10	11
13013	13009	13017	-	13224	13224	13225	+	1	1	2
13013	13009	13017	-	13742	13741	13742	-	1	3	4
13013	13009	13017	-	13750 -	-		+	1	5	6
13013	13009	13017	-	14265	14265	14266	+	1	3	4
13019 -	-		-	13157 -	-		-	1	10	11
13025	13023	13029	-	13137 -	-		+	1	3	4
13025	13023	13029	-	14222 -	-		+	1	1	2
13031	13030	13031	-	15152	15152	15153	+	1	6	7
13043	13039	13043	+	13069	13069	13070	-	1	2	3
13044	13040	13046	-	13795 -	-		+	1	3	4
13044	13040	13046	-	13877 -	-		+	1	1	2
13044	13040	13046	-	13940	13940	13941	+	1	2	3
13055	13051	13058	-	13198 -	-		-	1	1	2
13055	13051	13058	-	13424 -	-		-	1	1	2
13055	13051	13058	-	13913 -	-		+	1	1	2
13055	13051	13058	-	14315 -	-		+	1	4	5
13060	13059	13064	-	13835 -	-		-	1	1	2
13071	13067	13075	+	13141	13141	13142	+	1	1	2
13082	13079	13082	-	14429 -	-		+	1	2	3
13094	13088	13097	-	14345 -	-		+	1	1	2
13127	13124	13129	-	13791 -	-		+	1	1	2
13149	13146	13152	-	14061 -	-		-	1	1	2
13149	13146	13152	-	14289 -	-		+	1	1	2
13159	13157	13160	-	13903	13903	13904	+	1	1	2
13159	13157	13160	-	14524 -	-		+	1	1	2

13174	13169	13178	-	14543 -	-		+	1	1	2
13174	13169	13178	-	15029 -	-		+	1	2	3
13187	13182	13188	-	14207	14207	14208	+	1	1	2
13187	13182	13188	-	14687 -	-		+	1	1	2
13187	13182	13188	-	14711	14711	14712	+	1	1	2
13210 -	-		+	14423 -	-		+	1	1	2
13218 -	-		-	14707 -	-		+	1	2	3
13224	13220	13228	-	13347	13345	13347	+	1	1	2
13224	13220	13228	-	14676	14676	14677	+	1	3	4
13224	13220	13228	-	15064 -	-		+	1	3	4
13265	13261	13268	-	14184 -	-		+	1	1	2
13265	13261	13268	-	15050	15050	15051	-	1	7	8
13265	13261	13268	-	15159	15159	15161	+	1	1	2
13278	13277	13282	-	14033	14033	14034	-	1	1	2
13278	13277	13282	-	14435	14435	14437	+	1	2	3
13288	13287	13289	-	13694	13694	13696	+	1	3	4
13298	13296	13302	-	14354	14354	14355	-	1	2	3
13298	13296	13302	-	14503 -	-		+	1	2	3
13303	13302	13304	+	14344	14341	14344	+	1	6	7
13308	13303	13313	-	13543 -	-		+	1	1	2
13308	13303	13313	-	13921 -	-		+	1	1	2
13308	13303	13313	-	14456	14456	14458	+	1	4	5
13308	13303	13313	-	14711 -	-		-	1	8	9
13308	13303	13313	-	14846	14846	14847	-	1	3	4
13316	13315	13320	-	14036 -	-		+	1	3	4
13316	13315	13320	-	14863 -	-		+	1	1	2
13334	13333	13334	+	15088	15088	15089	+	1	1	2
13339	13335	13341	-	13710	13710	13711	-	1	1	2
13339	13335	13341	-	13899 -	-		-	1	2	3
13339	13335	13341	-	15206 -	-		-	1	1	2
13340	13340	13343	+	15001	15001	15004	+	1	16	17
13347	13344	13353	-	13404 -	-		+	1	1	2
13347	13344	13353	-	14171 -	-		+	1	1	2
13359	13355	13363	-	13486 -	-		+	1	1	2
13372	13372	13375	-	14756	14756	14757	+	1	1	2
13377	13376	13379	-	14547	14543	14548	-	1	2	3
13385	13381	13388	-	13598 -	-		-	1	1	2
13385	13381	13388	-	13908 -	-		+	1	1	2
13385	13381	13388	-	14354 -	-		+	1	2	3
13408	13404	13411	-	15173	15173	15174	+	1	2	3
13416	13413	13420	-	14533 -	-		-	1	1	2
13423	13423	13425	-	14983 -	-		+	1	1	2
13431	13428	13435	-	14746 -	-		+	1	1	2
13451	13448	13454	-	13944 -	-		+	1	1	2
13451	13448	13454	-	14369 -	-		+	1	1	2
13462	13457	13464	-	14123 -	-		+	1	1	2
13462	13457	13464	-	14199 -	-		+	1	1	2
13462	13457	13464	-	14339 -	-		+	1	1	2
13462	13457	13464	-	14631	14631	14632	+	1	1	2
13479	13475	13484	-	15045	15045	15047	-	1	1	2
13488	13485	13491	-	14612	14612	14613	+	1	1	2
13498	13495	13504	-	13986	13986	13988	+	1	2	3
13499 -	-		+	15080 -	-		+	1	4	5
13507	13507	13512	-	14749	14747	14751	-	1	3	4
13523	13522	13527	-	13709 -	-		+	1	1	2
13523	13522	13527	-	14031 -	-		-	1	1	2
13533	13529	13537	-	14525 -	-		+	1	1	2
13533	13529	13537	-	15143 -	-		+	1	3	4
13538	13538	13542	+	13685 -	-		+	1	23	24
13544	13539	13545	-	14461	14460	14461	-	1	4	5
13555	13554	13555	-	13973 -	-		+	1	1	2
13562	13556	13567	-	13956	13956	13958	+	1	2	3
13579	13575	13584	-	14586 -	-		-	1	2	3
13604	13599	13604	-	14227	14223	14227	-	1	1	2
13604	13599	13604	-	14434 -	-		+	1	1	2
13616	13612	13619	-	13862 -	-		+	1	3	4
13616	13612	13619	-	14528	14528	14529	+	1	1	2
13629	13625	13631	-	14673	14673	14676	-	1	14	15
13634	13632	13635	-	14370	14368	14370	+	1	1	2
13634	13632	13635	-	14747	14747	14748	+	1	4	5
13639	13637	13642	-	14616 -	-		-	1	1	2
13645	13644	13649	-	14227	14224	14227	+	1	5	6
13645	13644	13649	-	14530	14530	14532	+	1	2	3
13645	13644	13649	-	14843	14842	14843	+	1	4	5
13656	13654	13656	-	14755	14754	14755	+	1	3	4
13673	13669	13675	-	14193 -	-		-	1	56	57
13673	13669	13675	-	14575 -	-		+	1	7	8
13673	13669	13675	-	14647 -	-		-	1	1	2
13673	13669	13675	-	15002	15002	15003	-	1	1	2
13678	13676	13680	-	13774 -	-		-	1	1	2
13678	13676	13680	-	14200 -	-		-	1	1	2
13678	13676	13680	-	14827 -	-		+	1	1	2
13685	13682	13688	-	13937 -	-		+	1	2	3
13709	13703	13713	-	14301 -	-		+	1	1	2
13709	13703	13713	-	14664 -	-		+	1	1	2
13709	13703	13713	-	14945	14942	14945	+	1	1	2
13720	13718	13723	-	13929 -	-		+	1	2	3
13720	13718	13723	-	13994 -	-		+	1	1	2
13720	13718	13723	-	14391 -	-		+	1	1	2
13728	13728	13731	-	14467 -	-		+	1	2	3
13733	13733	13736	-	14553 -	-		+	1	2	3

13738	13738	13742	+	15104	15104	15105	+	1	1	2
13741	13738	13745	-	14377 -	-		+	1	1	2
13741	13738	13745	-	14436	14436	14438	+	1	2	3
13751	13746	13755	-	14048 -	-		+	1	1	2
13751	13746	13755	-	14350 -	-		+	1	1	2
13751	13746	13755	-	14673 -	-		+	1	1	2
13757	13756	13761	-	14544	14544	14546	+	1	11	12
13763	13762	13763	-	13975 -	-		-	1	1	2
13767	13767	13768	+	14339	14339	14340	+	1	1	2
13791	13789	13797	-	14503 -	-		-	1	1	2
13803	13802	13803	+	15267 -	-		+	1	1	2
13830	13826	13834	-	14842	14841	14842	+	1	3	4
13838 -	-		-	14391 -	-		-	1	1	2
13848	13843	13849	-	14214	14210	14214	-	1	1	2
13848	13843	13849	-	15278	15278	15279	+	1	2	3
13851	13850	13855	+	13937	13934	13937	+	1	1	2
13851	13850	13855	+	14868 -	-		+	1	1	2
13855	13850	13859	-	14104 -	-		+	1	1	2
13855	13850	13859	-	14612 -	-		-	1	1	2
13855	13850	13859	-	15045 -	-		+	1	2	3
13863	13861	13866	-	14264	14262	14264	-	1	2	3
13870	13867	13871	-	14994	14990	14994	-	1	11	12
13876	13872	13879	-	14349 -	-		-	1	2	3
13876	13872	13879	-	14508 -	-		+	1	2	3
13885	13880	13885	-	14214 -	-		-	1	1	2
13891	13887	13891	+	13918	13915	13918	-	1	1	2
13891	13887	13891	+	14890	14890	14891	+	1	1	2
13898	13898	13900	-	14896 -	-		+	1	2	3
13908	13906	13912	-	14693	14693	14695	+	1	5	6
13908	13906	13912	-	15113 -	-		-	1	1	2
13936	13932	13940	-	14133 -	-		-	1	1	2
13947	13941	13951	-	14079 -	-		+	1	1	2
13947	13941	13951	-	14500 -	-		-	1	1	2
13947	13941	13951	-	14907 -	-		+	1	1	2
13953	13952	13956	-	14842 -	-		+	1	3	4
13958 -	-		-	14800 -	-		+	1	2	3
13967	13964	13968	-	14709 -	-		+	1	1	2
13967	13964	13968	-	15095 -	-		+	1	3	4
13972	13971	13974	-	14217 -	-		+	1	1	2
13988	13984	13992	-	14477	14477	14478	+	1	1	2
13988	13984	13992	-	14699 -	-		+	1	1	2
14012	14008	14013	-	14283	14283	14284	+	1	2	3
14012	14009	14016	+	14129 -	-		+	1	1	2
14019	14015	14022	-	14411 -	-		-	1	3	4
14025	14023	14028	-	14926 -	-		+	1	1	2
14028	14025	14032	+	14402	14402	14403	-	1	1	2
14040	14040	14042	-	14150	14150	14151	-	1	1	2
14046	14044	14049	-	14069 -	-		-	1	1	2
14046	14044	14049	-	14295 -	-		+	1	1	2
14046	14044	14049	-	14421 -	-		+	1	2	3
14046	14044	14049	-	14741	14741	14742	+	1	1	2
14056	14054	14059	-	14199	14199	14200	+	1	1	2
14065	14062	14068	-	14597 -	-		+	1	2	3
14065	14062	14068	-	14672 -	-		-	1	2	3
14065	14062	14068	-	14680	14680	14681	+	1	1	2
14065	14062	14068	-	14773 -	-		+	1	1	2
14067	14067	14071	+	14868 -	-		+	1	1	2
14085	14085	14087	-	14614 -	-		-	1	2	3
14092	14089	14096	+	14128	14128	14129	+	1	1	2
14100	14100	14103	-	14628	14628	14629	-	1	3	4
14108	14106	14112	-	14228 -	-		+	1	1	2
14114 -	-		-	14801 -	-		+	1	4	5
14116	14116	14119	+	14237 -	-		-	1	1	2
14116	14116	14119	+	14287 -	-		+	1	1	2
14120	14115	14126	-	14706	14706	14707	+	1	1	2
14138	14134	14142	-	14677 -	-		+	1	3	4
14145	14145	14148	-	14607 -	-		+	1	1	2
14152	14151	14156	+	14221 -	-		+	1	1	2
14164	14161	14168	-	14391	14391	14393	-	1	21	22
14164	14161	14168	-	14574	14571	14574	+	1	1	2
14174	14174	14180	-	14532 -	-		+	1	1	2
14174	14174	14180	-	14747	14747	14748	+	1	1	2
14193	14188	14196	-	15077 -	-		+	1	3	4
14202	14198	14205	-	14894 -	-		-	1	1	2
14202	14198	14205	-	14992 -	-		+	1	2	3
14223	14219	14230	-	14599 -	-		-	1	1	2
14223	14219	14230	-	14864 -	-		+	1	2	3
14223	14219	14230	-	15164 -	-		-	1	2	3
14238	14234	14240	-	14236 -	-		+	1	1	2
14238	14234	14240	-	14844 -	-		+	1	10	11
14238	14234	14240	-	15153 -	-		+	1	2	3
14238	14234	14240	-	15271	15271	15272	+	1	6	7
14239	14238	14242	+	15171	15167	15171	+	1	2	3
14243	14241	14243	-	14800 -	-		+	1	1	2
14243	14241	14243	-	14852	14852	14853	+	1	2	3
14243	14241	14243	-	15025	15022	15026	+	1	3	4
14248	14244	14252	-	15147 -	-		+	1	2	3
14252	14249	14257	+	15031	15031	15034	+	1	4	5
14259	14258	14261	+	14413 -	-		+	1	1	2
14259	14258	14261	+	15149 -	-		+	1	1	2
14267	14263	14270	-	14505 -	-		-	1	1	2

14267	14263	14270	-	15216	15216	15217	+	1	1	2
14284	14280	14285	-	14546	14546	14547	+	1	4	5
14294	14291	14295	-	14928	-	-	-	1	3	4
14307	14302	14308	-	14441	14437	14441	+	1	1	2
14324	14319	14326	-	14808	-	-	+	1	1	2
14324	14319	14326	-	14815	-	-	+	1	3	4
14330	14330	14334	+	14357	-	-	+	1	1	2
14331	14328	14337	-	14424	14423	14424	-	1	10	11
14331	14328	14337	-	14507	-	-	-	1	4	5
14331	14328	14337	-	14581	-	-	-	1	1	2
14341	-	-	+	15159	-	-	+	1	1	2
14342	14339	14346	-	14678	-	-	+	1	1	2
14342	14339	14346	-	14813	14813	14815	+	1	2	3
14342	14339	14346	-	15115	-	-	-	1	1	2
14346	14344	14350	+	14467	-	-	+	1	2	3
14350	14348	14353	-	14913	-	-	-	1	1	2
14358	14354	14358	+	15128	-	-	+	1	3	4
14360	14355	14365	-	14576	-	-	+	1	1	2
14360	14355	14365	-	14586	-	-	+	1	1	2
14360	14355	14365	-	14815	-	-	-	1	1	2
14360	14355	14365	-	15030	-	-	+	1	1	2
14369	14367	14371	-	14560	-	-	-	1	1	2
14369	14367	14371	-	14619	-	-	+	1	2	3
14369	14367	14371	-	15031	15031	15033	+	1	1	2
14385	14383	14388	-	14689	-	-	+	1	1	2
14391	14390	14393	-	15067	-	-	-	1	1	2
14391	14388	14392	+	14844	-	-	-	1	1	2
14397	-	-	+	14414	-	-	+	1	1	2
14402	14402	14406	+	14635	-	-	+	1	2	3
14403	14399	14406	-	14864	-	-	+	1	3	4
14403	14399	14406	-	14940	-	-	-	1	1	2
14408	14407	14409	-	14877	-	-	+	1	1	2
14408	14407	14409	-	15186	15186	15187	-	1	2	3
14415	14410	14416	-	14876	14873	14876	-	1	1	2
14428	14427	14429	-	14678	14678	14680	+	1	4	5
14443	14442	14447	-	14589	-	-	-	1	1	2
14451	14449	14451	-	14633	14633	14634	-	1	1	2
14456	14452	14461	-	14690	14690	14693	+	1	6	7
14456	14452	14461	-	14780	-	-	-	1	1	2
14456	14452	14461	-	14807	-	-	+	1	1	2
14456	14452	14461	-	14867	14865	14867	-	1	11	12
14456	14452	14461	-	15016	15016	15018	+	1	1	2
14464	14463	14470	-	14617	14614	14617	-	1	1	2
14464	14463	14470	-	15033	-	-	+	1	1	2
14464	14463	14470	-	15291	15291	15292	+	1	1	2
14489	14484	14493	-	14672	-	-	-	1	4	5
14495	14494	14495	-	14697	-	-	+	1	1	2
14501	14498	14507	-	14532	-	-	+	1	1	2
14501	14498	14505	+	14697	-	-	+	1	1	2
14501	14498	14505	+	14927	-	-	+	1	1	2
14501	14498	14505	+	14948	-	-	+	1	1	2
14518	14510	14522	-	14582	-	-	-	1	1	2
14518	14510	14522	-	14916	14916	14917	-	1	1	2
14518	14510	14522	-	15010	15010	15011	+	1	2	3
14526	14524	14530	-	14890	-	-	-	1	1	2
14526	14524	14530	-	14919	14915	14919	+	1	1	2
14532	14531	14536	+	15121	-	-	+	1	2	3
14559	14555	14563	-	15037	-	-	-	1	2	3
14561	14557	14566	+	14579	-	-	+	1	1	2
14565	14564	14568	-	15017	-	-	+	1	2	3
14578	14576	14582	-	14783	-	-	+	1	1	2
14578	14576	14582	-	14828	14828	14830	+	1	3	4
14578	14576	14582	-	15251	-	-	-	1	2	3
14586	14584	14590	-	14891	-	-	+	1	2	3
14589	14584	14594	+	15054	15050	15054	-	1	1	2
14595	14592	14597	-	14796	14792	14796	+	1	1	2
14605	14600	14609	+	14861	14858	14861	+	1	1	2
14612	14611	14615	-	14965	-	-	+	1	1	2
14622	14619	14626	-	14880	14878	14880	+	1	1	2
14623	14622	14624	+	14775	14775	14776	+	1	1	2
14633	14632	14635	-	14698	-	-	+	1	1	2
14633	14632	14635	-	14914	14914	14915	-	1	1	2
14638	14638	14641	-	15021	15021	15025	+	1	2	3
14638	14634	14642	+	15052	15052	15053	+	1	1	2
14651	14648	14651	+	14634	14631	14635	-	1	6	7
14653	14649	14655	-	14676	14675	14676	+	1	14	15
14673	14671	14674	-	14770	14768	14770	+	1	10	11
14676	14672	14677	+	14732	14730	14732	+	1	2	3
14678	14675	14679	-	15188	15185	15189	-	1	10	11
14696	14692	14700	+	15189	-	-	+	1	4	5
14703	14701	14708	-	14754	14754	14755	+	1	1	2
14703	14701	14708	-	14867	-	-	-	1	1	2
14703	14701	14708	-	15079	-	-	-	1	1	2
14727	14724	14728	+	15023	-	-	+	1	5	6
14732	14727	14737	-	14866	-	-	-	1	3	4
14732	14727	14737	-	14871	14871	14873	+	1	1	2
14748	14743	14752	-	14835	-	-	+	1	1	2
14780	14780	14782	+	14936	14936	14938	+	1	2	3
14789	14785	14794	+	15068	15064	15068	+	1	1	2
14805	14800	14806	+	14870	-	-	+	1	1	2
14806	14802	14807	-	15043	-	-	+	1	23	24

14806	14802	14807	-	15068	15068	15069	+	1	2	3
14806	14802	14807	-	15104	-	-	+	1	3	4
14811	14810	14815	-	15163	-	-	-	1	1	2
14818	14814	14822	+	14801	14797	14801	-	1	3	4
14823	-	-	-	14956	-	-	+	1	1	2
14830	14830	14832	+	15207	-	-	-	1	1	2
14838	14834	14843	+	15030	-	-	+	1	2	3
14847	14843	14850	-	15117	-	-	-	1	6	7
14847	14843	14850	-	15163	15163	15164	-	1	1	2
14854	14851	14855	-	14978	-	-	-	1	6	7
14854	14851	14855	-	15040	15037	15040	+	1	2	3
14854	14851	14855	-	15205	-	-	-	1	1	2
14858	14855	14862	+	14837	14835	14838	-	1	2	3
14858	14855	14862	+	15031	15031	15032	-	1	2	3
14858	14855	14862	+	15180	-	-	+	1	1	2
14859	14856	14862	-	15015	15014	15015	-	1	5	6
14859	14856	14862	-	15039	-	-	+	1	1	2
14859	14856	14862	-	15183	-	-	-	1	2	3
14864	14863	14864	-	15027	15023	15027	-	1	1	2
14864	14864	14865	+	14894	14894	14895	-	1	1	2
14869	14865	14872	-	15034	-	-	-	1	2	3
14877	14873	14878	-	15066	15066	15068	-	1	1	2
14877	14875	14882	+	14900	14898	14900	-	1	2	3
14877	14875	14882	+	14935	-	-	+	1	1	2
14882	14879	14886	-	15057	-	-	+	1	3	4
14887	14884	14887	+	15028	-	-	+	1	1	2
14892	14889	14895	+	15027	15025	15027	-	1	1	2
14894	14890	14897	-	15019	15019	15023	-	1	2	3
14916	14912	14918	-	14929	-	-	+	1	1	2
14933	14932	14936	-	15034	-	-	+	1	1	2
14935	14935	14937	+	15027	-	-	+	1	1	2
14935	14935	14937	+	15088	15088	15089	-	1	1	2
14947	14942	14953	-	15040	-	-	-	1	1	2
14947	14942	14953	-	15099	-	-	-	1	1	2
14947	14942	14953	-	15104	15104	15107	-	1	9	10
14947	14942	14953	-	15115	15115	15116	-	1	1	2
14957	14956	14960	-	15165	15165	15166	-	1	1	2
14957	14956	14960	-	15383	-	-	-	1	1	2
14982	14978	14986	-	14996	-	-	+	1	1	2
15002	14998	15003	-	15042	15040	15042	-	1	1	2
15004	15000	15010	+	15161	-	-	+	1	1	2
15015	15011	15019	+	15068	15066	15068	+	1	1	2
15025	15023	15027	-	15026	15024	15026	+	1	1	2
15030	15029	15030	-	15121	-	-	-	1	1	2
15042	15041	15042	+	15012	-	-	-	1	1	2
15047	15043	15050	+	15198	-	-	+	1	1	2
15065	15065	15066	-	15212	-	-	-	1	1	2
15078	15077	15085	-	15107	-	-	+	1	3	4
15078	15077	15085	-	15129	15127	15129	-	1	2	3
15078	15077	15085	-	15182	15180	15182	-	1	2	3
15078	15077	15085	-	15228	-	-	-	1	1	2
15086	15082	15086	+	15163	15163	15167	+	1	4	5
15092	15087	15096	+	15070	15066	15070	-	1	1	2
15092	15087	15096	+	15103	-	-	+	1	1	2
15102	15098	15105	+	15145	-	-	+	1	1	2
15116	15114	15119	+	15044	-	-	-	1	16	17
15116	15114	15119	+	15098	-	-	-	1	1	2
15135	15132	15138	+	15160	-	-	-	1	1	2
15142	15142	15145	-	15158	-	-	+	1	1	2
15148	15147	15152	+	15124	15122	15124	-	1	3	4
15148	15147	15152	+	15164	-	-	-	1	1	2
15148	15147	15152	+	15203	15203	15206	-	1	1	2
15192	15189	15192	-	15206	-	-	-	1	1	2
15201	15199	15207	+	15185	15185	15189	-	1	9	10
15201	15199	15207	+	15221	15221	15222	+	1	1	2
15230	15226	15230	+	15294	-	-	+	1	1	2
15236	15232	15239	+	15212	-	-	-	1	35	36
15246	15242	15246	+	15281	-	-	+	1	1	2
15257	15257	15260	+	15291	-	-	+	1	1	2
15268	15268	15269	+	15242	15242	15243	-	1	1	2
15384	-	-	+	15382	15382	15384	-	1	3	4
8	-	-	+	14326	-	-	-	0	2	2
61	-	-	-	14053	-	-	-	0	3	3
100	98	100	+	956	-	-	+	0	2	2
100	98	100	+	14915	-	-	+	0	4	4
120	-	-	-	229	-	-	-	0	2	2
186	186	188	+	14999	14999	15001	+	0	13	13
228	225	229	+	15167	15163	15167	-	0	9	9
241	241	243	-	15144	-	-	+	0	6	6
242	240	246	+	13587	-	-	+	0	3	3
249	248	249	+	226	-	-	-	0	2	2
250	249	250	-	15134	-	-	+	0	2	2
254	253	257	+	15284	-	-	+	0	8	8
259	259	261	+	15290	15289	15290	+	0	3	3
266	262	270	-	14607	-	-	+	0	2	2
287	284	291	+	11877	-	-	+	0	2	2
287	284	291	+	14916	-	-	+	0	2	2
312	312	315	+	14340	-	-	+	0	3	3
316	315	320	-	15008	15008	15009	-	0	2	2
328	-	-	+	13840	-	-	+	0	2	2
396	393	396	-	13873	13869	13873	+	0	2	2

409	405	412	+	15290	15287	15290	+	0	2	2
461	461	463	-	14734	14732	14734	+	0	2	2
465	464	465	+	492	489	492	+	0	2	2
505	503	506	+	13337	13334	13337	+	0	2	2
511	509	511	+	14268 -	-		+	0	2	2
573 -	-		-	15136 -	-		+	0	3	3
581	578	581	+	14585 -	-		-	0	2	2
611 -	-		-	696 -	-		-	0	2	2
629 -	-		-	14191 -	-		+	0	2	2
654	652	658	+	13677 -	-		+	0	2	2
654	652	658	+	15221 -	-		+	0	2	2
668 -	-		-	906 -	-		-	0	2	2
754 -	-		+	14247 -	-		+	0	2	2
770	769	775	+	14065 -	-		+	0	4	4
770	769	775	+	15025 -	-		-	0	2	2
770	769	775	+	15033 -	-		+	0	3	3
791	789	794	+	14147 -	-		+	0	2	2
797	797	798	+	13146 -	-		+	0	2	2
802	802	803	+	14036 -	-		+	0	2	2
814	812	818	+	13182 -	-		+	0	2	2
871	870	871	+	15121 -	-		+	0	2	2
896	893	896	+	14619 -	-		+	0	2	2
923	920	927	+	13156	13156	13157	+	0	2	2
957	954	960	+	13116 -	-		+	0	3	3
1021	1020	1021	+	13543 -	-		+	0	2	2
1038	1035	1041	+	13017 -	-		+	0	2	2
1038	1035	1041	+	14561 -	-		+	0	2	2
1099	1097	1103	+	12997 -	-		+	0	2	2
1099	1097	1103	+	13366 -	-		+	0	2	2
1134	1131	1134	+	13587 -	-		+	0	2	2
1134	1131	1134	+	15169 -	-		+	0	7	7
1140	1138	1143	+	14547 -	-		+	0	2	2
1149	1148	1151	+	14163 -	-		+	0	2	2
1152 -	-		-	1203 -	-		-	0	2	2
1223	1222	1228	+	13050 -	-		+	0	3	3
1223	1222	1228	+	14972	14969	14972	-	0	3	3
1241	1240	1242	+	14079 -	-		-	0	2	2
1264 -	-		-	1417 -	-		-	0	3	3
1275 -	-		+	15291 -	-		+	0	3	3
1372	1370	1372	+	14590 -	-		+	0	2	2
1388 -	-		+	1458 -	-		+	0	4	4
1462	1462	1467	-	15172	15172	15173	-	0	2	2
1565 -	-		-	2312 -	-		-	0	7	7
1605 -	-		+	14118 -	-		-	0	7	7
1616	1615	1617	+	1706 -	-		+	0	7	7
1621	1619	1625	+	1703 -	-		+	0	12	12
1642	1641	1642	-	1672 -	-		-	0	3	3
1647 -	-		-	13988 -	-		+	0	2	2
1665	1665	1666	+	1707 -	-		+	0	2	2
1769	1769	1773	+	14594 -	-		+	0	2	2
1784	1784	1786	+	13760 -	-		-	0	2	2
1802	1801	1806	+	13740 -	-		-	0	2	2
1863	1860	1866	+	12228 -	-		+	0	7	7
1885	1882	1885	+	13813 -	-		+	0	2	2
1891	1887	1894	+	13113 -	-		+	0	3	3
1891	1887	1894	+	14946 -	-		-	0	2	2
1900	1896	1902	-	3313	3313	3314	+	0	2	2
1900	1896	1904	+	13386 -	-		+	0	2	2
1909	1906	1913	+	15364 -	-		+	0	2	2
1917	1917	1919	+	12999 -	-		-	0	2	2
1951	1948	1951	+	12598 -	-		+	0	2	2
1959	1953	1963	+	13773 -	-		+	0	2	2
1964	1962	1964	-	13930 -	-		+	0	2	2
2024	2024	2028	-	12507	12505	12507	-	0	2	2
2024	2024	2028	+	12507 -	-		+	0	5	5
2024	2024	2028	+	13736 -	-		+	0	4	4
2030	2030	2031	+	12509	12509	12510	+	0	2	2
2037	2035	2038	-	12516	12514	12516	-	0	2	2
2037	2034	2038	+	2038 -	-		+	0	3	3
2052	2049	2055	+	8947 -	-		+	0	2	2
2052	2049	2055	+	12529	12527	12529	+	0	3	3
2070 -	-		-	2203 -	-		-	0	4	4
2070	2069	2070	+	14464 -	-		-	0	2	2
2092 -	-		-	2184 -	-		-	0	2	2
2117	2117	2120	-	2173 -	-		-	0	12	12
2118	2115	2121	+	13950 -	-		+	0	3	3
2139	2139	2143	+	12111 -	-		+	0	4	4
2146	2146	2147	+	11159 -	-		+	0	3	3
2155	2152	2155	+	13469 -	-		+	0	2	2
2168	2166	2168	+	13503 -	-		+	0	2	2
2186 -	-		-	15225 -	-		+	0	2	2
2196 -	-		-	13883 -	-		-	0	2	2
2198	2196	2198	+	14384 -	-		+	0	3	3
2205	2205	2206	+	14379 -	-		+	0	2	2
2213	2213	2219	+	11030 -	-		+	0	2	2
2249	2248	2252	+	15190 -	-		+	0	2	2
2300	2299	2300	-	14730 -	-		+	0	2	2
2317	2315	2317	+	11916 -	-		+	0	5	5
2325	2322	2325	+	13593 -	-		+	0	2	2
2366	2363	2370	-	15205	15203	15205	-	0	2	2
2386	2385	2390	+	6157 -	-		+	0	2	2

2408	2408	2409	+	13776 -	-		-	0	3	3
2414	2412	2416	+	13315 -	-		+	0	2	2
2426	2424	2429	+	12978 -	-		+	0	2	2
2426	2424	2429	+	13738 -	-		-	0	3	3
2473	2470	2473	+	11863 -	-		+	0	4	4
2473	2470	2473	+	13395 -	-		+	0	3	3
2486 -	-		+	13766 -	-		+	0	11	11
2532 -	-		+	13524 -	-		+	0	2	2
2571	2571	2573	+	13410 -	-		+	0	2	2
2620	2620	2622	+	12220 -	-		+	0	2	2
2668 -	-		+	13779 -	-		+	0	3	3
2696	2692	2696	+	4320 -	-		+	0	2	2
2754	2754	2757	-	14420 -	-		-	0	2	2
2795	2793	2795	+	14294 -	-		+	0	2	2
2820 -	-		+	13224 -	-		+	0	2	2
2827	2826	2831	+	10885 -	-		+	0	3	3
2841	2838	2842	-	15292	15292	15295	+	0	4	4
2855	2855	2857	+	15279 -	-		-	0	2	2
2861	2857	2861	-	2882	2881	2882	+	0	4	4
2931	2926	2931	+	13779 -	-		+	0	4	4
2931	2926	2931	+	14808	14806	14808	-	0	3	3
2975	2975	2976	-	3006 -	-		-	0	2	2
2992 -	-		+	12988 -	-		+	0	4	4
3115	3114	3115	-	15198	15197	15198	-	0	9	9
3123	3123	3126	+	14757 -	-		+	0	2	2
3140	3137	3142	+	14009 -	-		+	0	3	3
3140	3137	3142	+	14270 -	-		+	0	3	3
3166	3165	3166	+	14104 -	-		+	0	3	3
3175	3171	3176	+	10773 -	-		+	0	2	2
3175	3171	3176	+	14513 -	-		-	0	8	8
3184	3180	3188	+	12620 -	-		+	0	2	2
3198	3196	3199	+	11827 -	-		+	0	2	2
3214	3213	3218	+	4464 -	-		+	0	2	2
3220	3219	3222	+	8110 -	-		-	0	2	2
3220	3219	3222	+	11307 -	-		+	0	2	2
3238	3236	3238	+	4494 -	-		+	0	3	3
3243	3239	3243	+	14128 -	-		+	0	7	7
3263	3259	3263	-	14667	14666	14667	-	0	17	17
3272	3270	3275	+	13581 -	-		+	0	2	2
3317	3313	3317	+	14731 -	-		+	0	2	2
3386	3384	3389	+	14081 -	-		+	0	2	2
3410	3408	3410	+	12464 -	-		+	0	2	2
3432 -	-		-	4774 -	-		+	0	2	2
3436 -	-		+	13642 -	-		+	0	2	2
3472	3469	3472	+	15276 -	-		+	0	19	19
3472	3469	3472	+	15289 -	-		+	0	7	7
3480	3476	3480	+	15016 -	-		+	0	4	4
3486	3482	3489	-	15067 -	-		-	0	2	2
3492	3490	3494	+	12021 -	-		-	0	2	2
3492	3490	3494	+	15080 -	-		+	0	2	2
3508	3505	3512	+	13516 -	-		+	0	2	2
3517	3515	3518	+	15105 -	-		+	0	2	2
3565	3565	3567	+	13795 -	-		+	0	2	2
3690	3686	3693	+	15284	15284	15288	+	0	3	3
3721	3717	3723	+	7321	7317	7321	-	0	2	2
3721	3717	3723	+	12104 -	-		+	0	2	2
3778	3778	3782	-	15204	15202	15204	+	0	2	2
3826 -	-		-	15122 -	-		+	0	2	2
3835	3832	3836	+	12616 -	-		+	0	2	2
3846 -	-		-	15103 -	-		+	0	3	3
3860	3860	3864	+	13012 -	-		+	0	2	2
3896 -	-		+	12056 -	-		+	0	3	3
3942	3942	3946	+	12672 -	-		+	0	2	2
3962	3961	3962	+	15274 -	-		-	0	2	2
3982	3979	3982	+	15116 -	-		+	0	10	10
3987	3986	3990	+	11001 -	-		+	0	3	3
4003	4001	4004	+	13568 -	-		+	0	3	3
4008 -	-		+	10536 -	-		+	0	2	2
4124 -	-		+	12501 -	-		+	0	2	2
4167	4164	4168	-	13685 -	-		-	0	2	2
4168 -	-		+	14525	14524	14525	+	0	3	3
4181	4180	4184	+	10750	10750	10754	+	0	4	4
4186	4185	4190	+	13733 -	-		-	0	2	2
4433	4429	4433	+	15030 -	-		+	0	4	4
4445	4442	4445	-	15043	15042	15043	-	0	3	3
4489 -	-		-	5834 -	-		-	0	2	2
4586	4585	4586	+	13657 -	-		+	0	3	3
4648	4645	4648	+	14819 -	-		+	0	4	4
4662	4658	4666	+	14358 -	-		+	0	3	3
4687	4683	4688	+	14586 -	-		+	0	6	6
4692 -	-		+	14878 -	-		-	0	2	2
4764	4764	4766	+	11137 -	-		+	0	5	5
4842 -	-		-	5074 -	-		+	0	3	3
4848	4847	4850	+	14532 -	-		+	0	6	6
4856	4856	4857	-	9726	9725	9726	+	0	3	3
4887	4884	4890	+	14656	14653	14656	-	0	5	5
4910 -	-		-	14631 -	-		+	0	6	6
4932 -	-		-	13029 -	-		+	0	3	3
4944	4939	4947	+	12865 -	-		+	0	2	2
4979 -	-		-	5013 -	-		-	0	3	3
4979 -	-		-	11330 -	-		+	0	2	2

5048	5045	5048	+	13043 -	-		+	0	3	3
5060	5059	5061	-	11378 -	-		+	0	2	2
5086	5083	5089	-	15034	15034	15037	-	0	19	19
5095 -	-		+	14179 -	-		+	0	2	2
5126 -	-		+	15283 -	-		+	0	2	2
5179	5177	5179	+	13068 -	-		+	0	2	2
5206	5206	5207	-	5465 -	-		-	0	2	2
5401 -	-		+	14654 -	-		+	0	2	2
5444	5444	5445	+	10545 -	-		+	0	2	2
5444	5444	5445	+	15190 -	-		-	0	2	2
5450	5449	5454	+	14804 -	-		+	0	6	6
5450	5449	5454	+	15187 -	-		-	0	4	4
5451	5451	5452	-	15182 -	-		+	0	2	2
5476	5473	5477	-	15146 -	-		+	0	2	2
5489	5489	5493	-	15141 -	-		+	0	2	2
5525 -	-		-	7594 -	-		+	0	2	2
5542	5542	5543	-	13841	13840	13841	+	0	3	3
5553 -	-		+	13830 -	-		-	0	2	2
5585 -	-		+	14734 -	-		+	0	2	2
5606 -	-		+	14264 -	-		+	0	3	3
5660	5660	5662	-	13501 -	-		+	0	2	2
5745	5742	5745	+	14691 -	-		+	0	2	2
5773	5773	5775	+	15182 -	-		-	0	9	9
5778	5778	5780	-	15177 -	-		+	0	30	30
5780	5778	5780	+	15169 -	-		+	0	3	3
5780	5778	5780	+	15185	15185	15187	-	0	21	21
5785	5783	5789	+	14639 -	-		+	0	2	2
5786	5786	5787	-	14881 -	-		+	0	3	3
5786	5786	5787	-	15179 -	-		+	0	6	6
5798 -	-		-	15197 -	-		-	0	2	2
5856 -	-		-	6048 -	-		-	0	2	2
5860	5858	5864	+	14104 -	-		+	0	2	2
5893 -	-		-	14688 -	-		+	0	2	2
5907 -	-		+	12765 -	-		+	0	3	3
6038 -	-		+	13041 -	-		+	0	2	2
6050	6050	6053	-	15288	15285	15288	+	0	2	2
6199	6196	6202	-	12405	12402	12405	+	0	2	2
6202	6198	6204	+	15103	15101	15103	+	0	2	2
6209	6209	6213	-	11113	11109	11113	+	0	3	3
6290 -	-		-	15163 -	-		-	0	2	2
6312	6308	6315	+	15282	15278	15282	+	0	5	5
6325	6323	6325	+	15295 -	-		+	0	2	2
6332	6329	6334	+	15304	15301	15304	+	0	2	2
6431	6431	6434	-	14082	14079	14082	-	0	2	2
6468 -	-		-	13160 -	-		+	0	2	2
6474 -	-		+	13986 -	-		+	0	2	2
6517 -	-		-	7053 -	-		-	0	2	2
6544 -	-		-	6716 -	-		-	0	2	2
6638	6638	6640	+	15293	15293	15294	+	0	2	2
6800 -	-		-	6887 -	-		-	0	4	4
6808	6808	6810	+	10914 -	-		+	0	2	2
6816	6814	6816	+	8904 -	-		-	0	2	2
6816	6814	6816	+	13822 -	-		+	0	4	4
6944	6941	6948	+	15191 -	-		-	0	4	4
6989 -	-		-	14144 -	-		+	0	2	2
7032 -	-		-	13111 -	-		+	0	2	2
7055	7055	7058	+	14707	14707	14710	+	0	3	3
7083	7082	7087	-	7165 -	-		-	0	3	3
7130	7130	7134	+	7221 -	-		+	0	3	3
7207 -	-		-	15030 -	-		+	0	2	2
7337 -	-		-	15213 -	-		+	0	2	2
7433 -	-		+	13498 -	-		+	0	2	2
7467	7464	7467	-	13582 -	-		+	0	2	2
7480	7479	7480	-	13719 -	-		+	0	2	2
7502	7498	7506	-	7594 -	-		-	0	2	2
7510	7509	7514	-	15286	15282	15286	+	0	4	4
7517	7515	7519	+	14348 -	-		-	0	2	2
7830	7827	7830	-	8026 -	-		-	0	4	4
7852	7852	7855	-	8060 -	-		-	0	2	2
7883 -	-		-	11732 -	-		-	0	2	2
7883 -	-		-	14078 -	-		+	0	2	2
8003 -	-		-	8426 -	-		+	0	2	2
8007	8007	8008	+	8819	8817	8819	+	0	2	2
8038 -	-		-	13881 -	-		+	0	2	2
8049	8046	8050	+	14609	14609	14610	+	0	2	2
8065	8061	8066	-	14594 -	-		+	0	2	2
8065	8061	8066	-	14668	14666	14668	+	0	2	2
8071	8070	8075	-	13908 -	-		+	0	2	2
8098	8095	8100	+	8113	8113	8115	+	0	3	3
8113	8112	8113	-	13740 -	-		+	0	4	4
8147	8147	8150	-	8243 -	-		-	0	4	4
8147	8147	8150	-	14915 -	-		+	0	3	3
8213	8209	8213	+	14698 -	-		+	0	2	2
8233	8231	8234	-	15205 -	-		+	0	3	3
8314	8314	8315	+	13681	13681	13682	-	0	2	2
8377 -	-		+	12847 -	-		+	0	3	3
8531 -	-		-	13094 -	-		+	0	3	3
8674 -	-		-	14049 -	-		+	0	3	3
8712	8710	8712	-	12440 -	-		+	0	2	2
8875	8875	8876	-	15023 -	-		+	0	2	2
8952	8948	8955	-	12380 -	-		+	0	2	2

8974	8971	8977	-	11305 -	-		+	0	2	2
9044	9044	9045	-	9150 -	-		-	0	2	2
9086	9086	9090	+	13374 -	-		+	0	2	2
9256	9256	9259	+	15288	15288	15291	+	0	6	6
9261	9260	9265	+	15293	15292	15293	+	0	3	3
9280	9280	9281	-	13574 -	-		+	0	2	2
9290 -	-		+	14258 -	-		+	0	2	2
9320	9319	9320	+	15243	15243	15245	-	0	3	3
9339 -	-		-	14315 -	-		+	0	2	2
9394	9394	9396	-	9618 -	-		-	0	2	2
9473	9473	9474	+	15295 -	-		+	0	3	3
9574 -	-		-	14186 -	-		+	0	2	2
9574	9570	9574	+	14677 -	-		-	0	2	2
9606	9604	9607	-	12210 -	-		+	0	4	4
9816	9815	9817	-	14065 -	-		+	0	5	5
9838 -	-		-	14175 -	-		+	0	3	3
9905	9903	9910	-	11549 -	-		-	0	9	9
9942	9938	9946	+	13296 -	-		+	0	2	2
9967	9964	9967	-	14841 -	-		+	0	2	2
9977 -	-		-	11684 -	-		+	0	2	2
9984	9981	9987	-	14622 -	-		+	0	2	2
9997	9996	9998	-	14688 -	-		+	0	3	3
10008	10006	10008	-	14677 -	-		+	0	2	2
10013	10011	10013	-	12307 -	-		+	0	2	2
10052	10048	10054	-	13166 -	-		+	0	2	2
10065	10061	10068	-	11070 -	-		+	0	3	3
10067	10063	10067	+	13162 -	-		-	0	2	2
10080	10078	10080	-	13453 -	-		+	0	2	2
10103	10103	10106	-	13550 -	-		+	0	3	3
10115	10114	10116	-	15255 -	-		+	0	4	4
10156	10156	10159	-	13911 -	-		+	0	3	3
10210	10207	10214	-	13197 -	-		+	0	2	2
10216 -	-		-	13416 -	-		+	0	2	2
10247	10245	10247	-	14163 -	-		+	0	2	2
10250	10246	10250	+	15184	15182	15184	+	0	2	2
10278	10276	10282	-	10521 -	-		-	0	2	2
10311	10309	10311	-	12319 -	-		-	0	2	2
10360	10360	10364	-	13857 -	-		+	0	2	2
10370	10367	10374	-	13911 -	-		+	0	2	2
10370	10367	10374	-	15092 -	-		+	0	2	2
10389	10386	10392	+	14843 -	-		+	0	4	4
10400	10397	10400	-	13872 -	-		+	0	2	2
10420	10416	10421	-	14654 -	-		+	0	2	2
10462	10459	10462	-	11807 -	-		-	0	2	2
10485	10481	10486	-	13837 -	-		+	0	2	2
10496	10496	10499	-	10517 -	-		+	0	2	2
10499	10496	10500	+	14974 -	-		+	0	3	3
10527 -	-		-	11098 -	-		+	0	2	2
10548	10548	10550	-	13885 -	-		+	0	2	2
10571	10568	10573	-	14358 -	-		+	0	2	2
10617 -	-		-	14740 -	-		+	0	2	2
10624	10621	10624	-	13431 -	-		+	0	4	4
10668	10665	10670	-	10966 -	-		+	0	2	2
10668	10665	10670	-	13658 -	-		+	0	2	2
10685	10684	10688	-	14756 -	-		+	0	4	4
10694	10692	10698	+	14871	14871	14872	-	0	2	2
10719	10715	10719	+	14501 -	-		+	0	2	2
10771 -	-		-	14512 -	-		+	0	2	2
10782	10782	10783	-	12654	12654	12656	+	0	3	3
10817	10815	10817	-	15062 -	-		+	0	8	8
10860	10858	10862	-	12327 -	-		+	0	2	2
10860	10858	10862	-	14729 -	-		+	0	2	2
10869	10866	10870	-	14971 -	-		+	0	2	2
10899	10895	10899	+	12732 -	-		+	0	2	2
10918	10918	10920	-	14499 -	-		+	0	3	3
10925	10922	10925	+	14668 -	-		+	0	2	2
10942 -	-		+	14802 -	-		+	0	2	2
10955	10952	10955	-	13464 -	-		+	0	4	4
11020	11019	11021	-	14343 -	-		+	0	2	2
11028	11024	11032	-	13150 -	-		+	0	2	2
11036 -	-		-	14130 -	-		-	0	3	3
11054	11054	11058	-	13385 -	-		+	0	3	3
11077 -	-		-	12352 -	-		-	0	2	2
11097	11096	11100	-	15297 -	-		+	0	2	2
11108	11108	11109	-	13275 -	-		+	0	2	2
11117	11114	11117	-	12660 -	-		-	0	4	4
11122	11119	11125	-	11545 -	-		+	0	2	2
11122	11119	11125	-	13941 -	-		+	0	18	18
11145	11141	11145	-	13792 -	-		+	0	2	2
11218	11214	11218	-	13739 -	-		+	0	3	3
11235	11234	11237	+	15292	15288	15292	+	0	3	3
11247	11244	11247	+	14786 -	-		+	0	3	3
11281	11281	11282	-	13968 -	-		+	0	2	2
11294	11293	11298	-	13946 -	-		-	0	6	6
11347 -	-		-	14586 -	-		+	0	2	2
11365	11365	11369	+	14299 -	-		+	0	2	2
11365	11365	11369	+	14722 -	-		+	0	2	2
11369	11368	11371	-	13602 -	-		+	0	2	2
11391	11388	11394	-	14163 -	-		+	0	2	2
11396	11392	11399	+	14560 -	-		-	0	2	2
11446	11442	11446	-	14433 -	-		-	0	2	2

11446	11442	11450	+	15192	-	-	+	0	3	3
11458	11457	11458	-	12765	-	-	+	0	2	2
11476	11472	11479	-	13362	-	-	+	0	2	2
11478	-	-	+	14806	-	-	+	0	2	2
11493	11490	11494	-	15025	-	-	-	0	2	2
11517	-	-	-	12658	-	-	+	0	7	7
11528	-	-	-	11789	-	-	+	0	2	2
11534	11530	11536	-	12161	-	-	+	0	4	4
11542	11542	11543	-	14301	-	-	+	0	2	2
11556	11556	11559	-	13309	-	-	+	0	12	12
11587	-	-	+	14155	-	-	+	0	2	2
11602	11597	11605	-	13003	-	-	+	0	2	2
11610	-	-	+	15296	-	-	+	0	2	2
11622	11621	11622	-	11647	-	-	-	0	2	2
11641	11638	11644	-	13780	-	-	+	0	2	2
11648	11648	11649	-	14935	-	-	-	0	2	2
11665	11664	11669	-	14049	-	-	+	0	3	3
11699	11699	11702	-	14040	-	-	+	0	2	2
11708	11706	11711	-	15200	-	-	+	0	2	2
11758	11754	11759	-	14391	-	-	+	0	3	3
11765	11765	11767	+	14843	-	-	+	0	6	6
11774	11771	11774	-	13901	-	-	+	0	6	6
11783	11780	11787	-	12820	-	-	+	0	2	2
11783	11780	11787	-	14265	-	-	+	0	3	3
11783	11780	11787	-	15031	-	-	+	0	4	4
11790	-	-	+	13923	-	-	+	0	2	2
11792	11789	11793	-	14740	-	-	+	0	4	4
11816	11811	11816	-	13499	13497	13499	+	0	7	7
11836	11836	11840	-	13054	-	-	+	0	2	2
11836	11836	11840	-	13921	-	-	-	0	2	2
11836	11836	11840	-	14186	-	-	+	0	3	3
11842	11841	11842	-	13221	-	-	+	0	2	2
11864	11863	11868	-	13794	-	-	-	0	2	2
11873	11870	11877	-	13164	-	-	+	0	3	3
11873	11870	11877	-	13456	-	-	+	0	2	2
11895	11894	11898	-	13736	-	-	+	0	3	3
11895	11894	11898	-	13791	-	-	-	0	3	3
11903	-	-	-	11971	-	-	-	0	2	2
11914	11911	11914	-	13845	-	-	-	0	2	2
11914	11911	11914	-	14760	-	-	+	0	2	2
11914	11911	11914	-	15045	-	-	+	0	4	4
11920	11915	11924	-	12974	-	-	-	0	3	3
11920	11915	11924	-	14343	-	-	+	0	2	2
11950	11948	11950	-	14524	-	-	+	0	2	2
11955	11952	11955	-	14302	-	-	+	0	12	12
11975	11974	11979	-	14779	-	-	+	0	2	2
11975	11974	11979	-	15161	-	-	+	0	2	2
11987	11987	11989	-	12722	-	-	+	0	3	3
11987	11987	11989	-	13256	-	-	+	0	2	2
11994	11992	11997	-	14416	-	-	-	0	3	3
11994	11992	11997	-	14847	-	-	+	0	2	2
12002	12000	12003	-	14475	-	-	-	0	2	2
12012	12010	12012	-	14834	14831	14834	+	0	2	2
12018	12016	12023	-	13522	-	-	+	0	3	3
12026	12024	12030	-	13545	13545	13547	+	0	3	3
12037	12037	12041	-	14379	-	-	-	0	3	3
12065	-	-	+	12139	-	-	+	0	2	2
12070	12068	12072	-	15044	-	-	+	0	3	3
12087	12085	12087	-	14879	-	-	+	0	3	3
12094	12090	12095	-	14107	-	-	+	0	2	2
12101	12098	12104	-	13331	-	-	+	0	3	3
12101	12098	12104	-	14386	-	-	-	0	2	2
12113	12109	12116	-	14368	-	-	+	0	4	4
12113	12109	12116	-	14410	-	-	+	0	2	2
12136	12133	12139	-	12912	-	-	+	0	2	2
12146	12143	12147	-	14422	-	-	+	0	2	2
12167	12167	12171	-	14376	-	-	+	0	4	4
12187	12184	12190	-	14518	-	-	+	0	3	3
12187	12187	12189	+	15042	-	-	+	0	3	3
12199	12194	12203	-	13042	-	-	+	0	4	4
12199	12194	12203	-	13079	-	-	+	0	5	5
12199	12194	12203	-	13372	-	-	+	0	2	2
12199	12194	12203	-	13619	-	-	+	0	2	2
12199	12194	12203	-	14507	-	-	+	0	3	3
12202	12202	12203	+	14612	-	-	+	0	6	6
12218	12215	12220	-	14336	-	-	+	0	2	2
12246	12245	12246	-	13886	-	-	+	0	2	2
12269	12266	12272	-	13178	13176	13178	+	0	4	4
12269	12266	12272	-	13298	-	-	+	0	2	2
12282	12282	12283	-	12446	-	-	+	0	3	3
12291	12287	12293	-	14992	-	-	-	0	2	2
12321	12318	12325	-	13942	-	-	+	0	2	2
12321	12318	12325	-	14577	-	-	+	0	5	5
12327	12326	12329	-	13787	-	-	-	0	2	2
12341	12340	12346	-	13963	-	-	-	0	2	2
12352	12352	12356	-	13013	-	-	+	0	2	2
12352	12352	12356	-	13756	-	-	-	0	2	2
12359	12358	12360	-	14282	-	-	+	0	3	3
12364	12362	12367	-	14056	-	-	+	0	2	2
12364	12362	12367	-	14294	-	-	+	0	2	2
12383	12381	12385	-	13766	-	-	+	0	10	10

12383	12381	12385	-	14524	-	-	+	0	2	2
12407	12403	12410	-	13303	-	-	+	0	4	4
12407	12403	12410	-	14591	-	-	+	0	2	2
12423	12422	12426	-	13629	-	-	+	0	2	2
12433	12429	12439	-	13287	-	-	+	0	2	2
12433	12429	12439	-	13635	-	-	+	0	2	2
12442	-	-	+	14093	-	-	+	0	12	12
12445	12441	12448	-	13264	-	-	+	0	4	4
12445	12441	12448	-	13507	-	-	+	0	2	2
12456	12456	12457	-	14586	-	-	+	0	3	3
12467	12462	12470	-	14573	-	-	-	0	2	2
12469	12469	12473	+	14521	-	-	+	0	2	2
12476	12471	12477	-	13706	-	-	-	0	2	2
12476	12471	12477	-	14174	-	-	+	0	2	2
12481	12478	12484	-	14529	-	-	+	0	2	2
12501	12501	12504	+	14320	-	-	+	0	4	4
12508	12508	12510	-	12997	-	-	+	0	2	2
12518	12512	12523	-	12640	-	-	-	0	2	2
12518	12512	12523	-	15205	-	-	+	0	3	3
12529	12524	12531	-	12638	-	-	-	0	2	2
12529	12524	12531	-	14565	-	-	+	0	4	4
12536	12535	12540	-	12684	12684	12685	-	0	20	20
12536	12535	12540	-	12897	-	-	+	0	6	6
12536	12535	12540	-	13439	-	-	+	0	3	3
12536	12535	12540	-	14154	-	-	+	0	4	4
12536	12535	12540	-	14401	-	-	+	0	2	2
12542	-	-	-	13378	-	-	+	0	3	3
12553	12549	12558	-	13545	-	-	+	0	2	2
12553	12549	12558	-	14182	-	-	-	0	3	3
12553	12549	12558	-	14236	-	-	+	0	6	6
12568	12565	12568	-	14209	-	-	-	0	3	3
12591	12589	12594	-	12625	-	-	-	0	2	2
12598	12596	12599	-	12909	-	-	+	0	2	2
12598	12596	12599	-	14841	-	-	-	0	2	2
12604	12600	12607	-	13080	-	-	+	0	3	3
12604	12600	12607	-	14295	-	-	+	0	2	2
12611	12608	12614	-	12644	-	-	-	0	3	3
12619	12615	12620	-	14896	-	-	+	0	2	2
12625	-	-	-	13760	-	-	-	0	3	3
12635	12632	12635	-	13472	-	-	+	0	2	2
12635	12632	12635	-	13595	-	-	+	0	2	2
12641	12636	12645	-	13071	-	-	+	0	2	2
12641	12636	12645	-	14033	-	-	+	0	2	2
12652	12649	12653	-	13521	-	-	+	0	2	2
12652	12649	12653	-	15276	-	-	+	0	2	2
12658	12654	12662	+	13008	-	-	+	0	5	5
12661	12657	12666	-	13158	-	-	+	0	3	3
12661	12657	12666	-	13388	-	-	+	0	2	2
12676	12671	12678	-	14193	-	-	+	0	3	3
12696	12692	12697	-	13598	-	-	-	0	2	2
12696	12692	12697	-	13644	-	-	+	0	2	2
12707	12703	12708	-	14025	-	-	+	0	2	2
12707	12703	12708	-	14653	-	-	+	0	2	2
12732	12727	12735	-	14141	-	-	+	0	3	3
12734	12733	12734	+	14715	-	-	+	0	2	2
12741	12736	12744	-	15024	-	-	+	0	16	16
12754	12753	12759	-	13972	-	-	+	0	4	4
12761	12760	12765	-	13905	13905	13907	+	0	3	3
12761	12760	12765	-	14631	-	-	+	0	2	2
12775	12772	12778	-	13404	-	-	+	0	2	2
12780	12779	12780	-	13459	-	-	+	0	17	17
12780	12779	12780	-	14055	-	-	+	0	6	6
12788	12787	12792	-	13345	-	-	+	0	3	3
12788	12787	12792	-	14076	-	-	+	0	2	2
12788	12787	12792	-	14401	-	-	+	0	7	7
12813	12811	12816	-	12931	-	-	-	0	5	5
12813	12811	12816	-	14225	-	-	+	0	2	2
12819	-	-	+	12906	-	-	+	0	2	2
12825	12822	12828	-	13124	-	-	+	0	2	2
12825	12822	12828	-	13596	-	-	+	0	2	2
12838	12836	12841	-	13558	-	-	+	0	5	5
12838	12836	12841	-	13594	-	-	+	0	3	3
12838	12836	12841	-	14225	-	-	-	0	2	2
12838	12836	12841	-	14397	-	-	+	0	4	4
12838	12836	12841	-	14967	-	-	+	0	4	4
12843	12842	12846	-	13551	-	-	+	0	3	3
12843	12842	12846	-	14463	-	-	+	0	2	2
12843	12842	12846	-	14995	14993	14995	+	0	2	2
12843	12842	12846	-	15092	-	-	+	0	3	3
12859	-	-	+	14050	-	-	+	0	2	2
12884	12881	12886	-	12948	-	-	-	0	2	2
12894	12893	12894	+	12871	-	-	-	0	5	5
12896	12896	12898	-	13984	-	-	+	0	2	2
12896	12896	12898	-	14331	-	-	+	0	3	3
12896	12896	12898	-	14717	-	-	+	0	3	3
12911	12906	12916	-	13698	-	-	+	0	2	2
12911	12906	12916	-	14117	-	-	-	0	2	2
12911	12906	12916	-	14739	14739	14740	+	0	2	2
12911	12906	12916	-	14840	14840	14843	+	0	4	4
12911	12906	12916	-	15025	-	-	-	0	2	2
12924	12921	12928	+	14083	-	-	+	0	2	2

12924	12921	12928	+	15147	15145	15147	+	0	5	5
12928	12926	12931	-	13314	13314	13315	-	0	2	2
12941	12938	12945	-	13721	-	-	+	0	14	14
12956	12952	12960	-	13079	-	-	+	0	2	2
12956	12952	12960	-	13498	-	-	+	0	3	3
12956	12952	12960	-	13583	-	-	+	0	2	2
12956	12952	12960	-	14308	-	-	+	0	4	4
12956	12952	12960	-	14588	-	-	+	0	2	2
12965	12964	12966	-	13735	-	-	+	0	5	5
12970	12967	12973	-	13579	-	-	+	0	2	2
12970	12967	12973	-	13860	-	-	+	0	3	3
12970	12967	12973	-	14340	-	-	+	0	2	2
12970	12970	12972	+	13001	-	-	-	0	2	2
12970	12970	12972	+	14063	-	-	-	0	3	3
12976	12975	12979	-	14313	-	-	+	0	2	2
12976	12975	12979	-	14559	-	-	+	0	8	8
12978	12976	12980	+	14033	-	-	+	0	2	2
12985	12982	12988	-	15234	-	-	+	0	3	3
12990	12990	12993	-	14496	-	-	-	0	2	2
13000	12995	13002	-	13574	-	-	+	0	2	2
13000	12995	13002	-	14599	-	-	+	0	3	3
13013	13009	13017	-	13062	-	-	+	0	2	2
13013	13009	13017	-	13743	-	-	+	0	2	2
13013	13009	13017	-	13767	-	-	+	0	2	2
13013	13009	13017	-	14530	-	-	+	0	4	4
13016	13016	13019	+	13442	-	-	+	0	2	2
13016	13016	13019	+	13690	-	-	+	0	2	2
13043	13039	13043	+	14883	-	-	+	0	2	2
13055	13051	13058	-	13813	-	-	-	0	2	2
13055	13051	13058	-	15030	-	-	+	0	2	2
13060	13059	13064	-	13438	13434	13438	+	0	6	6
13060	13059	13064	-	13706	-	-	-	0	2	2
13060	13059	13064	-	13828	-	-	+	0	2	2
13060	13059	13064	-	14883	-	-	+	0	22	22
13070	13069	13071	-	14148	-	-	+	0	2	2
13070	13069	13071	-	14537	-	-	+	0	4	4
13104	13100	13107	-	13697	-	-	+	0	2	2
13104	13100	13107	-	14294	-	-	+	0	2	2
13104	13100	13107	-	14777	-	-	+	0	3	3
13106	13104	13106	+	13626	-	-	-	0	2	2
13106	13104	13106	+	14370	-	-	+	0	2	2
13127	13124	13129	-	14315	-	-	+	0	2	2
13141	13139	13145	-	14870	-	-	-	0	2	2
13149	13146	13152	-	14314	14314	14316	+	0	5	5
13174	13169	13178	-	13987	-	-	+	0	3	3
13180	-	-	-	13890	-	-	-	0	2	2
13187	13182	13188	-	13700	-	-	+	0	2	2
13187	13182	13188	-	14721	-	-	+	0	3	3
13187	13182	13188	-	14873	-	-	+	0	2	2
13207	13203	13210	-	14716	-	-	+	0	3	3
13224	13220	13228	-	13734	-	-	+	0	4	4
13224	13220	13228	-	13972	-	-	+	0	2	2
13224	13220	13228	-	14307	-	-	+	0	3	3
13224	13220	13228	-	14400	-	-	+	0	2	2
13224	13220	13228	-	15104	-	-	+	0	2	2
13246	13240	13246	-	15010	-	-	+	0	2	2
13252	13247	13256	-	14370	-	-	+	0	2	2
13258	13258	13260	-	13899	-	-	+	0	10	10
13272	13269	13276	-	13407	-	-	+	0	2	2
13278	13277	13282	-	14023	-	-	-	0	2	2
13293	13291	13294	-	14095	-	-	+	0	3	3
13298	13296	13302	-	13591	13590	13591	+	0	5	5
13298	13296	13302	-	14161	-	-	-	0	4	4
13298	13296	13302	-	14980	-	-	+	0	3	3
13303	13302	13304	+	14261	-	-	+	0	4	4
13308	13303	13313	-	14111	-	-	-	0	2	2
13308	13303	13313	-	14867	-	-	-	0	4	4
13308	13303	13313	-	15029	-	-	-	0	3	3
13316	13315	13320	-	15022	-	-	-	0	3	3
13322	13321	13324	+	14842	-	-	-	0	14	14
13329	13328	13332	+	14988	-	-	+	0	3	3
13329	13328	13332	+	15093	-	-	+	0	5	5
13330	13324	13334	-	14987	14987	14988	-	0	2	2
13334	13333	13334	+	14847	-	-	-	0	2	2
13339	13335	13341	-	13500	-	-	-	0	2	2
13339	13335	13341	-	13584	-	-	+	0	2	2
13347	13344	13353	-	13703	-	-	+	0	2	2
13347	13344	13353	-	13750	-	-	+	0	3	3
13347	13344	13353	-	14705	-	-	+	0	2	2
13359	13355	13363	-	13755	-	-	+	0	2	2
13372	13372	13375	-	13497	-	-	+	0	2	2
13377	13376	13379	-	13789	-	-	-	0	2	2
13385	13381	13388	-	15072	-	-	+	0	2	2
13398	13394	13402	+	14194	-	-	-	0	2	2
13408	13404	13411	-	14305	-	-	+	0	8	8
13416	13413	13420	-	13972	-	-	+	0	2	2
13416	13413	13420	-	14025	-	-	+	0	5	5
13416	13413	13420	-	15213	-	-	+	0	4	4
13431	13428	13435	-	15153	-	-	+	0	2	2
13451	13448	13454	-	14158	-	-	-	0	2	2
13462	13457	13464	-	14178	-	-	+	0	2	2

13466	13466	13467	+	15064	-	-	+	0	2	2
13467	13466	13467	-	13684	-	-	+	0	2	2
13471	13471	13473	+	14537	-	-	+	0	2	2
13471	13471	13473	+	14950	-	-	+	0	2	2
13473	13468	13474	-	14705	-	-	+	0	2	2
13479	13475	13484	-	13892	-	-	+	0	2	2
13479	13475	13484	-	15070	15069	15070	+	0	3	3
13479	13475	13484	-	15146	-	-	-	0	3	3
13483	13483	13484	+	13564	-	-	+	0	2	2
13488	13485	13491	-	14045	-	-	+	0	2	2
13498	13495	13504	-	14678	-	-	+	0	5	5
13507	13504	13508	+	15297	15297	15298	+	0	2	2
13523	13522	13527	-	14532	-	-	+	0	5	5
13532	13532	13533	+	13679	-	-	+	0	25	25
13544	13539	13545	-	14007	-	-	+	0	6	6
13544	13539	13545	-	14498	-	-	-	0	3	3
13549	13547	13553	-	14514	14513	14514	-	0	4	4
13549	13547	13553	-	14637	-	-	-	0	5	5
13550	13550	13553	+	13686	-	-	+	0	4	4
13558	13558	13561	+	14436	-	-	+	0	2	2
13562	13556	13567	-	13684	-	-	+	0	2	2
13562	13556	13567	-	14521	-	-	-	0	2	2
13569	13568	13572	-	14007	-	-	+	0	2	2
13569	13568	13572	-	14660	-	-	-	0	2	2
13572	13572	13575	+	14570	-	-	+	0	2	2
13579	13575	13584	-	14677	-	-	-	0	13	13
13587	13586	13589	-	13886	-	-	-	0	2	2
13587	13586	13589	-	14618	-	-	-	0	2	2
13604	13599	13604	-	14575	-	-	+	0	3	3
13610	13605	13611	-	13965	-	-	+	0	2	2
13616	13612	13619	-	14036	-	-	+	0	2	2
13616	13612	13619	-	14834	-	-	+	0	4	4
13616	13612	13619	-	14840	-	-	+	0	4	4
13623	13620	13623	-	14003	-	-	-	0	3	3
13629	13625	13631	-	13783	13783	13784	-	0	3	3
13629	13625	13631	-	13798	13794	13798	-	0	6	6
13629	13625	13631	-	14867	-	-	+	0	2	2
13639	13637	13642	-	14220	-	-	-	0	5	5
13639	13637	13642	-	14635	-	-	+	0	3	3
13644	13641	13644	+	14705	-	-	+	0	2	2
13645	13644	13649	-	14466	-	-	-	0	6	6
13645	13644	13649	-	14472	-	-	-	0	2	2
13651	13649	13651	+	14029	-	-	+	0	13	13
13673	13669	13675	-	13850	-	-	+	0	3	3
13678	13676	13680	-	14020	-	-	-	0	2	2
13678	-	-	+	13725	-	-	-	0	3	3
13709	13703	13713	-	13920	-	-	-	0	2	2
13709	13703	13713	-	14332	-	-	+	0	4	4
13715	13714	13717	-	13791	-	-	-	0	2	2
13715	13714	13717	-	14005	-	-	-	0	2	2
13720	13718	13723	-	14300	-	-	+	0	2	2
13728	13728	13731	-	15288	15288	15289	+	0	4	4
13741	13738	13745	-	14805	-	-	+	0	4	4
13757	13756	13761	-	14432	-	-	+	0	23	23
13767	13767	13768	+	14333	-	-	+	0	2	2
13770	13764	13773	-	14515	-	-	-	0	3	3
13770	13764	13773	-	14580	-	-	+	0	2	2
13774	13772	13777	+	14343	14342	14343	+	0	5	5
13774	13772	13777	+	14355	-	-	+	0	2	2
13779	13776	13783	-	14608	-	-	+	0	3	3
13808	-	-	+	13832	-	-	+	0	2	2
13810	13807	13815	-	14412	-	-	-	0	2	2
13810	13807	13815	-	14781	-	-	+	0	2	2
13820	13818	13820	-	15045	-	-	-	0	2	2
13848	13843	13849	-	15078	-	-	+	0	2	2
13855	13850	13859	-	14377	-	-	+	0	4	4
13855	13850	13859	-	14697	-	-	+	0	2	2
13855	13850	13859	-	14956	-	-	+	0	2	2
13855	13850	13859	-	15007	-	-	+	0	6	6
13862	13861	13862	+	13841	-	-	-	0	2	2
13863	13861	13866	-	14000	-	-	-	0	4	4
13870	13867	13871	-	14265	-	-	+	0	3	3
13873	-	-	+	14995	-	-	+	0	2	2
13876	13872	13879	-	14385	-	-	+	0	3	3
13878	13875	13882	+	14370	-	-	+	0	2	2
13878	13875	13882	+	14616	-	-	+	0	2	2
13878	13875	13882	+	14875	-	-	+	0	4	4
13878	13875	13882	+	14987	-	-	+	0	2	2
13885	13880	13885	-	14500	-	-	+	0	2	2
13885	13880	13885	-	14836	-	-	+	0	4	4
13893	13893	13895	-	14152	-	-	-	0	5	5
13893	13893	13895	-	14169	-	-	-	0	2	2
13898	13897	13899	+	15265	-	-	+	0	2	2
13914	13913	13917	-	14391	-	-	+	0	2	2
13947	13941	13951	-	14101	-	-	-	0	2	2
13953	13952	13956	-	14038	-	-	-	0	3	3
13953	13952	13956	-	14148	-	-	-	0	6	6
13982	13978	13982	-	14415	-	-	+	0	2	2
13988	13984	13992	-	14412	-	-	-	0	2	2
13988	13984	13992	-	14927	-	-	+	0	2	2
13988	13984	13992	-	15135	-	-	-	0	9	9

13992	13988	13993	+	14896 -	-		+	0	2	2
14001	13996	14005	-	14009 -	-		+	0	2	2
14001	13996	14005	-	14016 -	-		+	0	25	25
14001	13996	14005	-	14457 -	-		+	0	3	3
14012	14009	14016	+	14291 -	-		+	0	3	3
14019	14015	14022	-	14196 -	-		-	0	5	5
14019	14015	14022	-	14202 -	-		-	0	5	5
14034	14033	14038	+	15202	15202	15204	-	0	4	4
14046	14044	14049	-	14153 -	-		-	0	2	2
14046	14044	14049	-	14650 -	-		+	0	3	3
14056	14054	14059	-	14662 -	-		+	0	2	2
14061	14058	14061	+	15010 -	-		+	0	67	67
14065	14062	14068	-	14199 -	-		-	0	6	6
14065	14062	14068	-	14531 -	-		+	0	2	2
14078	14076	14078	+	14616 -	-		+	0	2	2
14079	14079	14083	-	14358	14356	14358	+	0	2	2
14091	14089	14096	-	14982 -	-		-	0	2	2
14092	14089	14096	+	14255 -	-		+	0	2	2
14092	14089	14096	+	14888	14886	14888	+	0	6	6
14100	14098	14102	+	14479 -	-		-	0	3	3
14108	14106	14112	-	14203 -	-		-	0	2	2
14108	14106	14112	-	14425 -	-		+	0	2	2
14137	14133	14138	+	14181 -	-		+	0	2	2
14138	14134	14142	-	14200 -	-		-	0	7	7
14138	14134	14142	-	14242 -	-		-	0	3	3
14138	14134	14142	-	14370 -	-		+	0	2	2
14138	14134	14142	-	14432 -	-		+	0	2	2
14138	14134	14142	-	14608 -	-		+	0	10	10
14138	14134	14142	-	14801 -	-		+	0	4	4
14152	14151	14156	+	14739 -	-		+	0	6	6
14158 -	-		+	14248 -	-		+	0	2	2
14159	14157	14160	-	14194 -	-		-	0	17	17
14180	14177	14183	+	14215 -	-		-	0	3	3
14193	14188	14196	-	14673 -	-		+	0	2	2
14193	14188	14196	-	15037 -	-		-	0	2	2
14193	14188	14196	-	15087 -	-		+	0	3	3
14194 -	-		+	15113 -	-		+	0	2	2
14207	14207	14211	+	15174 -	-		-	0	3	3
14212	14210	14216	-	14249 -	-		+	0	3	3
14212	14210	14216	-	15169	15165	15171	+	0	20	20
14212	14210	14216	-	15176 -	-		+	0	6	6
14219	14215	14222	+	14710	14707	14710	+	0	7	7
14223	14219	14230	-	14239 -	-		+	0	3	3
14223	14219	14230	-	14247 -	-		+	0	2	2
14223	14219	14230	-	15033 -	-		+	0	3	3
14223	14219	14230	-	15107	15103	15107	-	0	7	7
14223	14219	14230	-	15151 -	-		-	0	2	2
14233	14231	14233	-	15096 -	-		-	0	2	2
14248	14244	14252	-	15023	15020	15024	+	0	5	5
14252	14249	14257	+	14786 -	-		+	0	3	3
14254	14253	14258	-	14405	14405	14406	-	0	5	5
14254	14253	14258	-	14710 -	-		+	0	8	8
14254	14253	14258	-	14847 -	-		+	0	2	2
14254	14253	14258	-	15034 -	-		-	0	2	2
14254	14253	14258	-	15145 -	-		+	0	3	3
14259	14258	14261	+	14876 -	-		+	0	2	2
14259	14258	14261	+	14967 -	-		+	0	2	2
14267	14263	14270	-	14413	14412	14413	+	0	8	8
14267	14263	14270	-	14565 -	-		+	0	2	2
14267	14263	14270	-	14795 -	-		+	0	5	5
14289	14287	14289	-	14776 -	-		+	0	2	2
14289	14287	14289	-	15319 -	-		-	0	2	2
14294	14291	14295	-	15180 -	-		+	0	2	2
14300	14298	14301	-	14837 -	-		+	0	8	8
14300	14298	14301	-	14913	14913	14917	-	0	4	4
14324	14319	14326	-	14358 -	-		-	0	2	2
14324	14319	14326	-	14937	14937	14941	-	0	8	8
14331	14328	14337	-	14443 -	-		-	0	2	2
14342	14339	14346	-	14390 -	-		-	0	7	7
14342	14339	14346	-	14845 -	-		-	0	6	6
14342	14339	14346	-	14879 -	-		-	0	2	2
14342	14339	14346	-	15053 -	-		-	0	2	2
14342	14339	14346	-	15164 -	-		-	0	2	2
14346	14344	14350	+	14523 -	-		-	0	2	2
14350	14348	14353	-	14867	14867	14869	-	0	3	3
14360	14355	14365	-	14503 -	-		-	0	11	11
14360	14355	14365	-	14764 -	-		+	0	4	4
14369	14367	14371	-	14585 -	-		-	0	2	2
14370	14366	14373	+	14821 -	-		+	0	2	2
14370	14366	14373	+	14894 -	-		+	0	3	3
14375	14374	14381	+	15060 -	-		-	0	2	2
14377	14372	14382	-	14861 -	-		+	0	25	25
14403	14399	14406	-	14598 -	-		+	0	2	2
14403	14399	14406	-	15184 -	-		+	0	8	8
14415	14410	14416	-	14780 -	-		-	0	2	2
14415	14410	14416	-	14895	14894	14895	+	0	3	3
14422	14420	14425	+	14422 -	-		+	0	4	4
14427	14427	14428	+	14470 -	-		+	0	2	2
14435	14434	14439	-	14472	14472	14476	+	0	4	4
14437	14432	14442	+	14507 -	-		+	0	2	2
14437	14432	14442	+	15066 -	-		+	0	5	5

14437	14432	14442	+	15171	-	-	+	0	3	3
14443	14442	14447	-	14513	-	-	-	0	10	10
14451	14449	14451	-	14511	14510	14512	-	0	4	4
14456	14452	14461	-	14736	-	-	-	0	2	2
14464	14463	14470	-	14848	-	-	-	0	2	2
14470	14470	14475	+	14841	-	-	-	0	2	2
14472	14472	14473	-	14582	-	-	-	0	2	2
14479	14475	14482	-	14572	-	-	+	0	2	2
14479	14475	14482	-	14611	-	-	-	0	2	2
14479	14475	14482	-	14978	-	-	+	0	9	9
14489	14484	14493	-	14638	-	-	+	0	2	2
14489	14484	14493	-	14793	-	-	-	0	3	3
14489	14484	14493	-	15244	-	-	-	0	2	2
14518	14510	14522	-	14837	-	-	-	0	3	3
14518	14510	14522	-	15179	-	-	-	0	2	2
14526	14524	14530	-	14721	-	-	+	0	2	2
14540	14536	14541	-	14890	-	-	-	0	2	2
14547	14547	14550	-	14674	-	-	-	0	3	3
14559	14555	14563	-	15125	-	-	+	0	2	2
14561	14557	14566	+	15384	-	-	-	0	3	3
14565	14564	14568	-	14750	14750	14752	+	0	4	4
14571	14570	14572	-	14669	-	-	+	0	2	2
14578	14576	14582	-	15122	-	-	+	0	4	4
14580	14578	14583	+	14635	-	-	-	0	3	3
14595	14592	14597	-	14781	-	-	-	0	9	9
14607	14602	14610	-	14785	-	-	+	0	2	2
14607	14602	14610	-	15043	-	-	+	0	5	5
14612	14611	14615	-	14840	14840	14842	+	0	29	29
14612	14611	14615	-	15029	-	-	-	0	2	2
14612	14611	14615	-	15043	-	-	+	0	9	9
14612	-	-	+	15008	-	-	+	0	3	3
14617	-	-	-	15013	-	-	-	0	3	3
14617	14617	14620	+	15291	-	-	+	0	3	3
14633	14632	14635	-	15039	-	-	-	0	3	3
14638	14634	14642	+	14986	-	-	+	0	3	3
14646	14645	14647	+	15231	-	-	+	0	2	2
14651	14648	14651	+	14671	-	-	+	0	2	2
14653	14649	14655	-	14855	-	-	-	0	2	2
14656	14652	14661	+	14632	14632	14633	-	0	2	2
14656	14652	14661	+	14770	-	-	+	0	3	3
14656	14652	14661	+	14926	-	-	+	0	6	6
14668	14666	14668	+	14661	-	-	-	0	2	2
14678	14675	14679	-	14944	-	-	-	0	7	7
14685	14681	14687	+	14791	-	-	+	0	2	2
14688	14685	14689	-	14788	-	-	-	0	2	2
14703	14701	14708	-	14804	-	-	-	0	2	2
14705	14705	14708	+	15023	-	-	+	0	2	2
14725	14725	14726	-	14816	14813	14816	+	0	2	2
14732	14727	14737	-	14846	-	-	-	0	5	5
14732	14727	14737	-	14993	-	-	+	0	2	2
14732	14727	14737	-	15043	-	-	+	0	2	2
14732	14727	14737	-	15079	-	-	-	0	2	2
14739	14738	14742	+	15029	-	-	+	0	4	4
14748	14743	14752	-	14918	-	-	-	0	6	6
14748	14743	14752	-	15066	-	-	-	0	2	2
14772	14771	14773	-	14929	-	-	-	0	2	2
14775	14775	14778	+	14927	-	-	+	0	8	8
14777	14774	14780	-	15193	-	-	+	0	2	2
14789	14785	14794	+	14866	-	-	+	0	2	2
14811	14810	14815	-	14841	-	-	-	0	2	2
14847	14843	14850	-	15152	-	-	+	0	3	3
14847	14845	14852	+	15177	-	-	+	0	5	5
14854	14851	14855	-	14999	-	-	-	0	2	2
14859	14856	14862	-	15044	-	-	+	0	4	4
14869	14865	14872	-	15071	15071	15073	-	0	3	3
14869	14865	14872	-	15133	15131	15133	-	0	2	2
14870	14866	14873	+	15032	-	-	+	0	2	2
14877	14873	14878	-	15023	15020	15026	+	0	6	6
14877	14875	14882	+	15015	15015	15018	-	0	3	3
14882	14879	14886	-	15235	-	-	+	0	5	5
14887	14884	14887	+	14867	14865	14867	-	0	3	3
14892	14889	14895	+	15051	-	-	+	0	3	3
14898	14898	14900	+	14912	-	-	+	0	2	2
14898	14898	14900	+	15030	-	-	+	0	2	2
14902	14899	14908	-	14933	-	-	+	0	2	2
14905	14904	14907	+	14987	-	-	+	0	2	2
14916	14912	14918	-	14943	14943	14945	+	0	3	3
14916	14912	14918	-	14957	14957	14958	+	0	2	2
14923	14919	14926	-	15211	-	-	-	0	2	2
14933	14932	14936	-	14950	-	-	+	0	14	14
14933	14932	14936	-	14994	-	-	-	0	2	2
14933	14932	14936	-	15157	-	-	+	0	4	4
14941	14938	14941	-	15092	-	-	-	0	2	2
14941	14938	14941	-	15100	15098	15100	-	0	6	6
14941	14938	14941	-	15142	-	-	-	0	4	4
14947	14942	14953	-	15109	-	-	-	0	4	4
14951	14948	14955	+	15129	-	-	-	0	2	2
14957	14956	14960	-	15132	-	-	+	0	111	111
14960	14956	14964	+	15172	-	-	+	0	3	3
14964	14962	14968	-	15160	-	-	-	0	2	2
14968	14966	14971	+	15042	15042	15043	-	0	2	2

14970	14969	14974	-	15061	-	-	-	0	3	3
14970	14969	14974	-	15108	-	-	-	0	4	4
14970	14969	14974	-	15120	-	-	-	0	2	2
14970	14969	14974	-	15146	-	-	-	0	2	2
14978	14974	14978	+	15169	-	-	+	0	2	2
15002	14998	15003	-	15051	-	-	+	0	2	2
15004	15000	15010	+	15051	-	-	+	0	4	4
15014	15010	15016	-	15154	-	-	-	0	2	2
15025	15023	15027	-	15048	-	-	+	0	2	2
15025	15023	15027	-	15171	-	-	-	0	2	2
15037	15033	15037	-	15072	-	-	+	0	4	4
15037	15033	15037	-	15124	-	-	-	0	2	2
15042	15042	15045	-	15074	-	-	+	0	9	9
15065	15065	15066	-	15079	-	-	+	0	2	2
15072	15068	15075	-	15179	-	-	-	0	3	3
15078	15077	15085	-	15157	15156	15157	-	0	15	15
15086	15082	15086	+	15165	-	-	-	0	2	2
15090	15087	15093	-	15143	-	-	-	0	2	2
15095	15095	15099	-	15159	-	-	-	0	2	2
15102	15102	15104	-	15139	15135	15139	-	0	3	3
15102	15098	15105	+	15073	15072	15073	-	0	4	4
15102	15098	15105	+	15085	15085	15086	-	0	10	10
15116	15114	15119	+	15085	-	-	-	0	3	3
15124	15121	15128	+	15137	-	-	+	0	2	2
15143	15140	15146	+	15122	-	-	-	0	3	3
15148	15147	15152	+	15086	-	-	-	0	3	3
15157	15154	15157	+	15130	-	-	-	0	2	2
15158	15158	15161	-	15384	-	-	-	0	2	2
15201	15199	15207	+	15172	15171	15172	-	0	3	3
15201	15199	15207	+	15177	-	-	-	0	4	4
15212	15211	15212	+	15191	15190	15191	-	0	6	6
15217	15215	15222	+	15195	-	-	-	0	3	3
15257	15257	15260	+	15258	-	-	-	0	2	2
15360	-	-	+	15355	-	-	+	0	2	2
