# Supplementary material for: Identification and quantification of defective virus genomes in high throughput sequencing data using DVG-profiler, a novel post-sequence alignment processing algorithm
Source: PLoS One. 2019 May 17;14(5):e0216944. doi: 10.1371/journal.pone.0216944 (PMC6524942; doi:10.1371/journal.pone.0216944)
Supplement: S10 Table — (DOCX) [file pone.0216944.s015.docx]

**S10 Table. Repeatability of results.**

|  | **Virus #2,**  **1^st^ HiSeq run ^a^** | **Virus #2,**  **2^nd^ HiSeq run ^c^** |
| --- | --- | --- |
| **Number of 5’ -and 3’- copyback DVGs,**  **> 50 reads** | 183 (98.9) ^b^ | 209 (99%) ^d^ |
| **Number of insertion and deletion type DVGs, > 50 reads** | 98 (95.9%) | 76 (93.4%) |

^a^ In parentheses percentage of DVGs that were also identified in the second HiSeq run.

^b^ two 3’ copyback DVGs were identified with breakpoint/reinitiation sites at 78-83/96-98 and 94-97/82, with 95 and 82, respectively, sequence reads in the 1^st^ HiSeq run and no sequence reads in the second run.

^c^ In parentheses percentage of DVGs that were also identified in the first HiSeq run

^d^ Two 5’ copyback DVGs were identified with breakpoints / reinitiation sites at 291 / 15094 and 277 / 14333, with 193 and 57 sequence reads, respectively. There were no sequence reads for these two DVGs identified in the 1^st^ HiSeq run.
