# Supplementary material for: Identification and quantification of defective virus genomes in high throughput sequencing data using DVG-profiler, a novel post-sequence alignment processing algorithm
Source: PLoS One. 2019 May 17;14(5):e0216944. doi: 10.1371/journal.pone.0216944 (PMC6524942; doi:10.1371/journal.pone.0216944)
Supplement: S11 Table — (PDF) [file pone.0216944.s016.pdf]

Position (left)	Group start (left)	Group end (left)	Strandness (left)	Position (right)	Group start (right)	Group end (right)	Strandness (right)	Forward hits	Reverse hits	Fwd and reverse	Mumps Reads	Total Reads
14130	14129	14135	-	14196	14195	14200	-	542	535	1077	3.88E+06	1.01E+07
7926	7921	7930	-	7986	7986	7990	-	268	281	549		
5078	5076	5082	-	15083	15079	15086	+	175	156	331		
8147	8146	8150	-	8243	8242	8246	-	7	254	261		
8140	8137	8145	-	8236	8236	8240	-	258	0	258		
14060	14055	14066	-	14195	14191	14200	-	119	107	226		
12536	12532	12539	-	12684	12680	12686	-	22	182	204		
6322	6318	6327	-	6414	6412	6418	-	85	86	171		
12529	12526	12531	-	12677	12675	12679	-	162	2	164		
14353	14347	14357	-	14497	14495	14502	-	152	6	158		
14359	14358	14365	-	14503	14502	14506	-	4	143	147		
8076	8074	8081	-	8229	8227	8233	-	70	73	143		
13508	13504	13509	-	13588	13586	13589	-	70	69	139		
11339	11335	11342	-	11441	11436	11444	-	97	33	130		
14933	14933	14936	-	15078	-	-	-	61	60	121		
5861	5859	5863	-	6009	6009	6010	-	59	58	117		
13948	13944	13953	-	14052	14048	14057	-	59	58	117		
6881	6880	6881	-	6980	-	-	-	57	56	113		
14442	14437	14444	-	14513	14509	14514	-	9	104	113		
7074	7073	7077	-	7131	7131	7132	-	2	101	103		
5065	5065	5069	-	15097	15096	15097	+	91	9	100		
6170	6166	6173	-	6322	6322	6323	-	47	52	99		
4808	4803	4812	-	4848	4844	4848	-	48	50	98		
14436	-	-	-	14507	-	-	-	97	1	98		
7069	7065	7069	-	7126	-	-	-	97	0	97		
5892	5890	5895	-	5960	5960	5961	-	93	3	96		
7245	7243	7248	-	7314	7314	7317	-	0	95	95		
5898	5897	5899	-	5966	-	-	-	0	92	92		
7240	7237	7242	-	7309	-	-	-	92	0	92		
14016	14012	14016	-	14196	14192	14196	-	78	11	89		
12146	12141	12150	-	14213	14213	14216	+	41	43	84		
6435	6431	6438	-	6476	6473	6477	-	41	42	83		
12837	12834	12837	-	12883	-	-	-	41	41	82		
9274	9269	9276	-	9358	9354	9361	-	40	38	78		
11344	11343	11348	-	11446	11442	11450	-	2	76	78		
9121	9120	9124	-	9207	9206	9210	-	39	37	76		
14022	14022	14025	-	14202	14202	14204	-	1	72	73		
7130	7128	7133	-	7229	7229	7231	-	35	37	72		
5101	5096	5104	-	5195	5190	5195	-	35	35	70		
14391	14388	14396	-	15010	-	-	+	33	34	67		
10255	10251	10258	-	10324	10320	10327	-	31	31	62		
12304	12299	12304	-	12362	-	-	-	60	1	61		
12309	12309	12310	-	12367	-	-	-	0	59	59		
5701	5698	5704	-	5786	5783	5789	-	28	27	55		
7221	7217	7222	-	7355	7351	7355	-	27	28	55		
12536	12532	12539	-	12662	12661	12662	-	28	26	54		
4284	4283	4287	-	4409	4408	4412	-	50	3	53		
11840	11838	11841	-	11915	11915	11916	-	26	26	52		
4289	4289	4292	-	4414	4414	4417	-	0	48	48		
7549	7545	7551	-	7705	7701	7706	-	25	23	48		
1513	1510	1517	-	1585	1583	1586	-	24	23	47		
13303	13299	13306	+	13261	13260	13261	-	23	23	46		
12584	12584	12587	-	12620	-	-	-	45	0	45		
12589	12589	12593	-	12625	-	-	-	0	45	45		
13640	13636	13640	-	13703	-	-	-	22	22	44		
11250	11249	11251	-	11332	11332	11333	-	4	39	43		
7512	7507	7515	-	7591	7591	7592	-	22	18	40		
13379	13377	13382	-	13469	13469	13472	-	38	2	40		
9176	9173	9179	-	9227	9223	9228	-	20	19	39		
15256	-	-	+	15242	-	-	-	20	19	39		
12047	12042	12051	-	12085	12085	12089	-	19	19	38		
7981	7977	7986	+	7953	7952	7956	-	18	19	37		
13384	13384	13386	-	13474	13474	13476	-	1	36	37		
13532	13531	13534	-	13679	13679	13681	-	37	0	37		
8442	8440	8443	-	8559	8559	8560	-	17	19	36		
9612	9610	9614	-	9695	9695	9697	-	18	18	36		
13538	13537	13542	-	13685	13684	13685	-	0	36	36		
14980	14977	14980	-	15114	15114	15115	-	18	18	36		
14465	14464	14465	+	14427	14427	14428	-	21	14	35		
3595	3593	3600	-	3626	-	-	+	2	32	34		
7278	7278	7280	-	7356	7356	7358	-	18	16	34		
7573	7569	7574	-	7635	7635	7636	-	17	17	34		
13808	13804	13812	-	14885	-	-	+	17	17	34		
7966	7961	7970	-	8065	8061	8065	-	17	16	33		
4517	4513	4520	-	4590	4588	4590	-	16	16	32		
11186	11185	11186	-	11281	11281	11282	-	16	16	32		
11245	11241	11247	-	11327	11327	11329	-	32	0	32		
12637	12634	12641	-	12783	12783	12788	-	16	16	32		
13384	13384	13386	-	14475	14474	14475	+	16	16	32		
13562	13561	13564	-	13665	-	-	-	16	16	32		
15275	-	-	+	15241	-	-	-	16	16	32		
10797	10795	10802	-	10906	10906	10907	-	15	16	31		
11238	11235	11238	-	11284	11281	11286	-	15	16	31		
3585	3583	3586	-	3636	-	-	+	30	0	30		
8162	8161	8166	-	8235	-	-	-	15	15	30		
9870	9866	9870	-	9920	9919	9920	-	15	15	30		
10347	10344	10347	-	10423	10420	10423	-	14	16	30		
14303	14303	14304	-	14384	14384	14385	-	14	16	30		
15192	-	-	-	15255	-	-	-	15	15	30		
5101	5096	5104	-	11815	-	-	+	11	18	29		
6745	6742	6749	-	6913	6909	6913	-	14	15	29		
6915	6914	6915	+	6874	6874	6875	-	14	15	29		
14346	14341	14346	-	14467	-	-	-	14	15	29		
2174	2169	2174	-	2314	2312	2314	-	25	3	28		
9597	9593	9601	+	9550	9549	9554	-	14	14	28		
12158	12154	12162	+	14220	14217	14224	+	14	14	28		
12853	12849	12857	-	12897	12894	12898	-	14	14	28		
1459	1457	1461	+	1398	1397	1398	-	14	13	27		
3944	3943	3948	+	3952	3952	3953	-	13	13	26		
10694	10691	10697	-	14876	-	-	+	13	13	26		

11054	11050	11057	-	11136	11132	11136	-	13	13	26
11266	11262	11270	-	11339	11336	11339	-	13	13	26
11990	11988	11994	-	12017	12017	12021	-	13	13	26
12134	12130	12136	+	12078	12078	12079	-	13	13	26
7991	7987	7995	-	8061	8061	8065	-	13	12	25
13972	13969	13975	+	13936	13936	13938	-	12	13	25
611	608	616	-	696	693	696	-	3	21	24
2181	2181	2184	-	2321	2321	2323	-	1	23	24
2518	2514	2521	-	2547	-	-	-	12	12	24
5293	5291	5293	-	5347	5345	5347	-	12	12	24
10484	10481	10487	-	10581	10581	10584	-	23	1	24
10635	10633	10639	-	10700	10700	10704	-	12	12	24
12584	12584	12587	-	12668	-	-	-	12	12	24
13880	13876	13884	-	14157	14155	14159	-	12	12	24
14973	14972	14975	-	15108	-	-	-	12	12	24
2757	2753	2758	-	2799	2799	2800	-	10	13	23
3722	3720	3724	-	7316	-	-	+	11	12	23
8059	8056	8063	+	8041	-	-	-	23	0	23
9905	9904	9909	-	11549	-	-	-	20	3	23
10672	10671	10676	+	10662	10658	10662	-	12	11	23
13114	13113	13114	+	13039	-	-	-	12	11	23
242	240	245	-	321	321	324	-	14	8	22
1490	1488	1492	-	1564	1561	1565	-	13	9	22
11321	11320	11321	-	11412	11411	11412	-	12	10	22
606	603	606	-	691	689	691	-	20	1	21
1488	1484	1490	+	1400	1398	1400	-	11	10	21
2028	2026	2034	+	12507	12506	12508	+	9	12	21
2350	2347	2354	-	14752	14751	14752	-	10	11	21
6506	6506	6507	-	6620	6620	6621	-	11	10	21
8459	8459	8463	+	8396	8392	8396	-	10	11	21
9697	9693	9700	+	9658	9655	9660	-	10	11	21
12876	12872	12876	-	12943	-	-	-	21	0	21
12881	12878	12884	-	12948	-	-	-	0	21	21
15084	15082	15088	-	15167	15167	15171	-	11	10	21
1755	1752	1759	-	1848	1848	1849	-	10	10	20
2377	2372	2378	-	2502	2500	2502	-	10	10	20
2495	2491	2499	-	2531	2527	2531	-	10	10	20
2702	2698	2706	+	2676	2673	2677	-	11	9	20
3213	3213	3217	-	3338	3338	3340	-	0	20	20
5820	5819	5823	-	5952	-	-	-	10	10	20
6265	6263	6267	-	6348	6348	6349	-	10	10	20
10242	-	-	-	10309	-	-	-	10	10	20
11339	11335	11342	-	11434	-	-	-	20	0	20
12326	12322	12330	-	12393	12389	12393	-	10	10	20
12488	12487	12494	-	12570	12569	12570	-	10	10	20
13629	13625	13632	-	13794	13793	13798	-	10	10	20
2091	2087	2092	-	2197	2194	2198	-	10	9	19
3208	3206	3208	-	3333	-	-	-	19	0	19
4061	4058	4061	-	4165	-	-	-	12	7	19
6198	6195	6201	-	11121	11119	11123	+	14	5	19
7270	7268	7271	-	7349	7349	7350	-	9	10	19
10490	10489	10494	-	10587	10587	10588	-	0	19	19
10498	10495	10501	-	10515	10515	10518	+	18	1	19
959	955	961	+	928	-	-	-	9	9	18
2174	2169	2174	-	2205	-	-	-	9	9	18
2443	2438	2446	+	2463	-	-	+	9	9	18
9327	9323	9330	-	9448	9448	9449	-	9	9	18
10036	10033	10040	-	13350	-	-	-	9	9	18
11976	11976	11981	-	14971	-	-	+	9	9	18
12637	12634	12641	-	12774	12769	12781	-	9	9	18
12847	12843	12847	-	13017	13013	13017	-	9	9	18
12908	12904	12908	-	12946	12943	12946	-	9	9	18
13261	-	-	+	13303	-	-	-	9	9	18
13879	13876	13882	+	14155	14152	14159	+	9	9	18
14391	14388	14396	-	14512	14509	14515	-	9	9	18
429	424	433	-	557	553	558	-	8	9	17
12813	12811	12818	-	12931	-	-	-	9	8	17
13460	13458	13461	-	13586	13586	13587	-	8	9	17
13651	13649	13654	+	14029	14029	14031	+	0	17	17
13961	13960	13964	-	14063	14062	14063	-	8	9	17
3863	3858	3864	-	3924	-	-	-	8	8	16
6390	6390	6392	+	6338	-	-	-	9	7	16
6474	6473	6477	+	6401	-	-	-	8	8	16
6686	6683	6686	+	11420	-	-	+	8	8	16
6935	6931	6939	-	7074	-	-	-	8	8	16
9314	9311	9320	-	9463	-	-	-	8	8	16
9911	9910	9914	-	10058	10058	10059	-	8	8	16
9987	9987	9990	-	10117	10117	10118	-	8	8	16
10797	10795	10802	-	12300	12296	12301	-	5	11	16
12082	12080	12087	+	12129	12125	12129	-	8	8	16
12618	12616	12622	-	12774	12772	12778	-	9	7	16
13391	13388	13391	-	13477	13477	13479	-	8	8	16
13591	13591	13592	-	13688	13688	13689	-	8	8	16
14744	14743	14748	-	14918	-	-	-	8	8	16
1230	1227	1235	-	5977	5974	5977	+	8	7	15
1361	1356	1364	-	1485	1481	1490	-	8	7	15
1969	1965	1973	-	2082	2082	2086	-	8	7	15
6852	6849	6856	+	6830	-	-	-	7	8	15
6976	6973	6979	-	7006	-	-	-	7	8	15
7611	7607	7611	-	7679	-	-	-	7	8	15
8431	8427	8435	+	15363	15360	15364	+	11	4	15
10286	10281	10290	-	10424	10424	10428	-	8	7	15
11426	11423	11426	-	11520	11516	11520	-	14	1	15
11431	11431	11436	-	11525	-	-	-	1	14	15
12123	12121	12127	-	13012	-	-	+	7	8	15
12407	12403	12410	-	12452	-	-	-	15	0	15
12412	12411	12415	-	12457	12456	12457	-	0	15	15
12893	12891	12897	+	12872	12871	12875	-	7	8	15
14243	14239	14243	+	14166	14164	14166	-	8	7	15
14314	14309	14318	-	14317	-	-	+	8	7	15
15062	15062	15066	-	15161	15161	15165	-	8	7	15

15251	15247	15251	+	15197	15197	15201	-	7	8	15
123	118	124	-	150	150	155	+	12	2	14
551	548	552	-	599	-	-	-	6	8	14
913	909	914	-	999	-	-	-	14	0	14
1220	1216	1224	-	1324	1324	1328	-	7	7	14
2022	2019	2023	+	2056	-	-	-	7	7	14
3441	3437	3445	-	3572	-	-	-	7	7	14
4539	4536	4544	-	4651	4651	4652	-	7	7	14
4544	4544	4546	+	9369	-	-	+	7	7	14
5276	5273	5280	-	5324	-	-	-	7	7	14
6634	6630	6637	-	6711	6709	6711	-	7	7	14
6963	6959	6966	-	7131	7130	7131	-	7	7	14
7504	7503	7505	-	7594	7592	7594	-	7	7	14
7897	7896	7900	-	8021	-	-	-	7	7	14
8611	8607	8615	-	8664	8664	8666	-	7	7	14
8650	8648	8652	-	8707	8706	8708	-	5	9	14
8935	8935	8937	+	8877	8877	8878	-	7	7	14
9126	9125	9131	-	9215	9211	9215	-	7	7	14
9327	9323	9330	-	9476	-	-	-	7	7	14
9782	9781	9786	-	12430	-	-	+	14	0	14
12351	12348	12351	-	12502	-	-	+	14	0	14
13157	13153	13157	-	13196	-	-	-	7	7	14
13664	13662	13669	-	13750	-	-	-	14	0	14
13664	13662	13669	-	13756	13756	13757	-	0	14	14
13768	13767	13771	-	13805	13805	13807	-	7	7	14
14069	14068	14071	-	14198	14196	14200	-	4	10	14
14161	14159	14165	-	14200	-	-	-	7	7	14
14806	14805	14806	-	14835	-	-	-	14	0	14
14812	-	-	-	14841	-	-	-	0	14	14
15220	15220	15221	-	15331	15331	15332	-	7	7	14
15235	-	-	+	15204	-	-	-	7	7	14
15251	15247	15251	+	15188	-	-	-	7	7	14
382	379	385	-	555	555	558	-	7	6	13
844	839	846	-	956	-	-	-	7	6	13
918	916	921	-	1004	-	-	-	0	13	13
1230	1227	1235	-	11175	-	-	-	0	13	13
7919	7915	7920	-	7979	7978	7980	-	10	3	13
8624	8621	8627	-	13809	13807	13810	-	0	13	13
8752	8748	8752	-	8862	8858	8862	-	6	7	13
9949	9945	9952	-	14118	14116	14118	-	2	11	13
10710	10706	10715	+	14861	14861	14864	-	0	13	13
10721	10719	10724	-	10793	10793	10796	-	1	12	13
12064	12059	12066	-	15303	15299	15303	-	13	0	13
13664	13662	13669	-	13798	-	-	-	6	7	13
13823	13819	13827	-	14892	14890	14896	-	6	7	13
251	247	255	-	330	330	334	-	3	9	12
325	323	331	-	14557	-	-	+	0	12	12
957	952	963	-	1015	1011	1015	-	6	6	12
2055	2050	2055	+	2023	-	-	-	6	6	12
3231	3225	3236	+	4486	4486	4489	+	9	3	12
3496	3493	3498	-	3568	-	-	-	6	6	12
5282	5281	5283	-	5331	5330	5332	-	6	6	12
5445	5443	5449	-	5501	-	-	-	6	6	12
5691	5690	5694	-	5763	-	-	-	12	0	12
5696	-	-	-	5768	-	-	-	0	12	12
5763	5760	5765	+	5741	5741	5744	-	6	6	12
6024	6022	6027	-	6059	-	-	-	6	6	12
6852	6849	6856	+	6813	-	-	-	6	6	12
7008	7005	7011	-	7122	7119	7122	-	6	6	12
7469	7469	7472	+	7452	-	-	-	6	6	12
8088	8084	8092	-	8260	8258	8263	-	6	6	12
8650	8648	8652	-	8730	-	-	-	6	6	12
8731	8726	8733	-	8859	-	-	-	6	6	12
9196	9193	9199	-	9341	9338	9341	-	6	6	12
10107	10103	10107	-	10154	-	-	-	6	6	12
10143	10139	10147	-	10209	10209	10210	-	6	6	12
10221	10221	10225	-	10247	10243	10247	+	6	6	12
10716	10714	10716	-	10788	-	-	-	12	0	12
11036	11031	11039	-	11102	11099	11102	-	6	6	12
11431	11431	11436	-	11505	-	-	-	6	6	12
12344	12343	12348	+	12312	12308	12312	-	6	6	12
12488	12487	12494	-	12557	12553	12557	-	6	6	12
12542	12542	12547	+	12603	12603	12605	-	6	6	12
13058	-	-	-	13126	-	-	-	6	6	12
13106	13104	13107	+	13055	13055	13058	-	6	6	12
13396	13396	13399	-	13482	-	-	-	6	6	12
14161	14159	14165	-	14238	-	-	-	6	6	12
14314	14309	14318	-	14373	14373	14375	-	6	6	12
14734	14732	14736	-	14789	-	-	-	6	6	12
15051	15049	15054	-	15115	15112	15115	-	6	6	12
15078	15078	15079	-	15157	15157	15161	-	2	10	12
15241	15239	15244	+	15275	15275	15276	-	6	6	12
300	298	303	-	436	434	436	-	6	5	11
1552	1550	1555	-	1579	-	-	-	5	6	11
4060	4058	4064	+	12334	12334	12335	+	5	6	11
4418	4415	4420	+	9671	-	-	+	1	10	11
5020	5017	5022	-	5156	5156	5158	-	10	1	11
5815	5814	5816	-	12720	12720	12722	+	6	5	11
6042	6041	6045	-	6148	-	-	-	6	5	11
6159	6157	6163	-	6310	6310	6314	-	6	5	11
6214	6208	6220	+	11131	11130	11132	+	11	0	11
6338	6338	6341	+	6390	-	-	-	6	5	11
7772	7770	7776	-	7877	7876	7877	-	0	11	11
7910	7910	7912	+	7874	7873	7875	-	5	6	11
8088	8084	8092	-	8243	-	-	-	6	5	11
9280	9279	9283	-	9364	9363	9366	-	5	6	11
10721	10717	10725	+	14850	14848	14851	-	10	1	11
12426	12423	12429	-	12498	-	-	-	11	0	11
12431	12431	12435	-	12503	-	-	-	0	11	11
14118	14114	14122	-	14194	14191	14194	-	5	6	11
14640	14640	14643	+	14687	-	-	-	5	6	11

14676	14672	14679	+	14653	14653	14657	-	5	6	11
14864	14860	14867	+	14818	14817	14818	-	5	6	11
15072	15067	15073	-	15151	-	-	-	10	1	11
633	632	637	+	666	665	666	-	5	5	10
1513	1510	1517	-	1558	1558	1560	-	2	8	10
2055	2050	2055	+	12531	12531	12534	+	8	2	10
2675	2672	2679	+	2703	2700	2704	-	5	5	10
3585	3583	3586	-	3588	-	-	+	5	5	10
5039	5037	5042	-	5207	5207	5210	-	10	0	10
5046	5045	5051	-	5125	-	-	-	5	5	10
5046	5045	5051	-	5205	-	-	-	10	0	10
5094	5093	5094	+	5055	5055	5056	-	1	9	10
5552	5551	5552	-	5641	-	-	-	10	0	10
5558	5554	5562	-	5647	-	-	-	0	10	10
6271	6270	6274	-	6373	6373	6374	-	5	5	10
6862	6861	6866	-	6969	6964	6969	-	5	5	10
7017	7014	7019	-	7119	7119	7120	-	5	5	10
7767	7763	7768	-	7872	-	-	-	10	0	10
8130	8126	8132	-	8175	-	-	-	5	5	10
8395	8391	8399	-	8563	8559	8563	-	5	5	10
8404	8400	8406	+	8429	8428	8429	-	5	5	10
9251	9250	9254	-	9406	9406	9407	-	5	5	10
9976	9974	9979	+	9987	9987	9988	-	5	5	10
10301	10300	10305	-	10430	10427	10430	-	5	5	10
10512	10509	10516	-	10501	10499	10504	+	0	10	10
10764	10761	10765	+	10699	-	-	+	5	5	10
11061	11058	11061	+	11005	-	-	-	5	5	10
11127	11123	11127	-	11283	11279	11283	-	5	5	10
11539	11539	11541	-	11620	-	-	-	6	4	10
11757	11754	11757	-	14500	14500	14503	+	5	5	10
12146	12141	12150	-	12208	12207	12208	-	5	5	10
12281	12278	12282	-	12507	12507	12508	-	5	5	10
12689	12687	12694	-	12795	12795	12798	-	5	5	10
13070	13067	13073	+	13001	13001	13002	-	5	5	10
13145	13141	13145	-	13307	13303	13307	-	5	5	10
13430	-	-	-	13556	-	-	-	5	5	10
13440	13440	13442	-	14553	-	-	+	5	5	10
13662	-	-	+	13598	-	-	-	5	5	10
13938	13936	13942	-	14042	-	-	-	6	4	10
13948	13944	13953	-	14033	14033	14034	-	10	0	10
13956	13954	13959	-	14038	-	-	-	0	10	10
14008	14004	14009	-	14187	-	-	+	6	4	10
14328	14323	14332	-	14454	14449	14455	-	5	5	10
14947	14943	14950	-	15107	-	-	-	5	5	10
15175	15172	15176	-	15251	15247	15251	-	5	5	10
15284	-	-	-	15336	-	-	-	5	5	10
15369	15365	15369	+	15340	15340	15344	-	5	5	10
748	744	752	+	708	707	708	-	5	4	9
1642	1640	1647	-	1672	-	-	-	0	9	9
2014	2010	2017	-	12493	12489	12493	-	0	9	9
2014	2011	2017	+	12493	12490	12493	+	2	7	9
2035	2035	2039	-	12514	12514	12518	-	7	2	9
2066	2066	2069	-	2171	2170	2173	-	5	4	9
2514	2512	2517	+	2458	2455	2458	+	5	4	9
2554	2550	2557	-	2662	-	-	-	4	5	9
4067	4065	4071	-	4171	4171	4174	-	4	5	9
4940	4936	4942	-	12865	-	-	-	0	9	9
5053	5053	5056	-	5212	-	-	-	0	9	9
5084	5083	5088	-	15077	-	-	+	9	0	9
5100	5096	5104	+	5049	5048	5049	-	8	1	9
5976	5974	5977	-	12329	-	-	+	1	8	9
6800	6798	6804	-	6887	-	-	-	4	5	9
7263	7258	7263	+	7221	-	-	-	5	4	9
8059	8056	8063	+	8148	8148	8151	-	0	9	9
8065	-	-	+	8145	-	-	-	9	0	9
8148	8145	8152	+	7992	-	-	-	5	4	9
8396	8392	8397	+	8461	8459	8462	-	4	5	9
8791	8788	8794	-	8920	8918	8920	-	4	5	9
9152	9148	9155	-	9326	9323	9326	-	5	4	9
9196	9193	9199	-	9361	9358	9366	-	5	4	9
9373	9370	9373	-	9457	-	-	-	5	4	9
10657	10655	10659	+	10677	10676	10677	-	4	5	9
11501	11497	11501	-	11620	-	-	-	4	5	9
12146	12141	12150	-	12272	-	-	-	8	1	9
12322	12319	12324	+	12282	-	-	-	4	5	9
12658	12654	12662	+	13008	13004	13008	+	7	2	9
13160	13157	13164	+	13095	13094	13095	-	4	5	9
13179	13179	13182	+	13129	13127	13130	-	4	5	9
13596	13595	13596	-	13703	13702	13703	-	1	8	9
14457	14455	14457	+	14435	14435	14437	-	1	8	9
5	3	10	-	89	85	91	-	4	4	8
30	29	34	+	7309	-	-	-	4	4	8
93	91	95	-	173	171	173	-	5	3	8
184	181	190	-	291	289	291	-	4	4	8
280	274	282	-	426	-	-	-	4	4	8
756	756	759	+	700	-	-	-	4	4	8
881	877	883	-	12715	-	-	-	0	8	8
913	912	921	+	904	-	-	-	4	4	8
1031	1029	1031	+	1067	-	-	-	3	5	8
1338	1335	1340	-	1771	-	-	+	4	4	8
1435	1431	1442	-	1603	1600	1603	-	4	4	8
1513	1510	1517	-	1636	1636	1637	-	4	4	8
2091	2087	2092	-	2206	2206	2207	-	3	5	8
2157	2153	2161	+	2117	2117	2118	-	4	4	8
2177	2175	2182	+	2099	-	-	-	4	4	8
2416	2416	2419	-	2507	-	-	-	4	4	8
2962	2958	2962	+	2941	2940	2942	-	4	4	8
3290	3285	3291	-	3331	3331	3332	-	4	4	8
3678	3675	3679	-	3712	-	-	-	4	4	8
3957	3953	3960	+	3939	3939	3940	-	4	4	8
4081	4080	4085	+	4102	4102	4105	+	4	4	8

4639	4635	4643	-	4682	4678	4682	-	4	4	8
5046	5045	5051	-	5213	-	-	-	0	8	8
5162	5159	5168	+	15100	-	-	-	5	3	8
5467	5466	5468	-	5531	-	-	-	4	4	8
5826	5825	5829	-	5958	5958	5959	-	4	4	8
6077	6076	6082	+	6047	6047	6048	-	4	4	8
6390	6390	6392	+	6414	-	-	-	4	4	8
6561	6558	6561	+	8284	8283	8284	-	7	1	8
6726	6722	6729	+	6964	6960	6965	+	6	2	8
6745	6742	6749	-	6818	-	-	-	4	4	8
6935	6931	6939	-	7042	-	-	-	4	4	8
7140	7137	7141	-	7239	7239	7241	-	5	3	8
7450	7446	7453	-	7577	7574	7577	-	4	4	8
7504	7503	7505	-	7572	-	-	-	4	4	8
7611	7607	7611	+	9512	9512	9513	-	4	4	8
7633	7630	7637	-	7708	7707	7708	-	5	3	8
8022	8022	8023	+	8078	8078	8079	-	4	4	8
8148	8145	8152	+	8118	-	-	-	4	4	8
8278	8278	8283	+	8240	-	-	-	4	4	8
8404	8400	8406	+	8360	8360	8363	-	4	4	8
8834	8831	8838	-	11350	11347	11351	+	8	0	8
9152	9148	9155	-	9338	9336	9338	-	4	4	8
9201	9200	9206	-	9318	9318	9322	-	3	5	8
9201	9200	9206	-	9336	9336	9337	-	4	4	8
9241	9238	9245	-	11500	-	-	-	0	8	8
9379	9376	9384	-	9401	9397	9401	+	4	4	8
9604	9603	9606	+	9627	9627	9629	+	4	4	8
9611	9609	9612	+	9579	-	-	-	0	8	8
9616	9616	9618	+	9574	-	-	-	8	0	8
9658	9657	9662	+	9697	-	-	-	4	4	8
10036	10033	10040	-	10158	-	-	-	4	4	8
10107	10103	10107	-	10220	-	-	-	4	4	8
10301	10300	10305	-	10402	10402	10403	-	4	4	8
11222	11220	11226	-	11399	11395	11399	-	4	4	8
11923	11920	11923	-	12039	-	-	-	4	4	8
11970	11968	11970	-	12014	-	-	-	4	4	8
11986	11984	11988	+	14980	14980	14983	+	4	4	8
12097	12094	12099	+	12083	12083	12084	-	4	4	8
12126	12124	12129	+	13023	-	-	+	4	4	8
12158	12154	12162	+	12136	12133	12136	-	4	4	8
12158	12154	12162	+	14262	-	-	+	4	4	8
12657	12654	12660	-	13007	13005	13008	-	3	5	8
12682	-	-	-	12769	-	-	-	8	0	8
12689	12687	12694	-	12776	-	-	-	0	8	8
12765	12763	12768	-	12931	12930	12932	-	4	4	8
12834	12834	12836	+	12707	-	-	-	4	4	8
12893	12891	12897	+	12806	-	-	-	4	4	8
13055	13055	13058	+	13199	13195	13199	-	4	4	8
13230	13226	13233	-	13281	-	-	-	4	4	8
13448	13445	13448	-	13540	13537	13543	-	4	4	8
13452	13452	13455	+	13331	13331	13332	-	4	4	8
14060	14055	14066	-	14241	14238	14241	+	4	4	8
14132	14129	14132	+	14107	14107	14110	-	4	4	8
14185	14183	14188	+	14107	-	-	-	4	4	8
14449	14448	14451	-	14510	-	-	-	4	4	8
14613	-	-	+	14562	-	-	-	5	3	8
14640	14640	14643	+	14512	14512	14513	-	4	4	8
15014	15012	15014	-	15066	-	-	-	4	4	8
15047	15047	15050	+	15113	15113	15114	-	4	4	8
15053	15053	15056	+	15108	-	-	-	4	4	8
15263	15263	15266	+	15255	-	-	-	4	4	8
150	148	150	-	123	123	125	+	7	0	7
635	633	637	-	711	-	-	-	4	3	7
813	813	818	-	939	935	942	-	3	4	7
1277	1273	1281	-	1343	1343	1344	-	1	6	7
1637	1634	1637	-	1667	-	-	-	7	0	7
2328	2327	2331	+	7238	-	-	+	3	4	7
2443	2438	2446	+	2486	2485	2486	+	4	3	7
2589	2585	2592	-	2669	-	-	-	7	0	7
2595	2594	2598	-	2675	2675	2676	-	0	7	7
2793	2791	2795	-	8972	-	-	-	7	0	7
2845	2841	2848	-	2910	-	-	-	7	0	7
3231	3225	3236	+	11014	-	-	-	7	0	7
3645	3642	3648	-	3820	-	-	-	7	0	7
3650	-	-	-	3825	-	-	-	0	7	7
5026	5025	5028	-	5162	-	-	-	0	7	7
5658	5655	5663	-	5796	5796	5798	-	4	3	7
5730	5727	5731	-	5877	5873	5877	-	4	3	7
6037	6034	6040	-	6143	6142	6143	-	4	3	7
6214	6208	6220	+	14766	14765	14766	+	7	0	7
6214	6208	6220	+	14957	14953	14958	+	7	0	7
6271	6270	6274	-	6399	6398	6402	-	4	3	7
6277	6275	6280	-	6379	6379	6380	-	3	4	7
6947	6947	6949	-	7116	-	-	-	4	3	7
6954	6953	6958	-	7123	7122	7126	-	3	4	7
6986	6982	6989	+	6956	6956	6960	-	3	4	7
7073	7068	7074	+	6957	6957	6961	-	4	3	7
7452	7447	7455	+	7469	-	-	-	4	3	7
7772	7769	7776	+	7753	7750	7755	-	4	3	7
7825	7821	7827	-	11157	11153	11157	-	2	5	7
7830	7829	7835	-	11163	11163	11164	-	0	7	7
8693	8689	8693	-	8761	-	-	-	7	0	7
9366	9363	9366	-	9468	-	-	-	4	3	7
9379	9376	9384	-	9493	9492	9493	-	3	4	7
10494	10491	10498	+	10517	10515	10519	-	3	4	7
10755	10752	10758	-	12680	12679	12681	+	3	4	7
11363	11363	11364	-	11434	-	-	-	5	2	7
11875	11875	11879	+	11781	-	-	-	4	3	7
12146	12141	12150	-	12277	-	-	-	0	7	7
12206	12205	12207	-	12409	12409	12410	-	1	6	7
12336	12332	12338	-	12452	12452	12453	-	2	5	7

12514	12510	12517	-	12605	12604	12605	-	6	1	7
12607	12603	12609	-	12784	12781	12786	-	4	3	7
12613	12611	12614	-	12682 -	-		-	7	0	7
12613	12611	12614	-	12711 -	-		-	7	0	7
12618	12616	12622	-	12687 -	-		-	0	7	7
12618	12616	12622	-	12716 -	-		-	0	7	7
12695	12692	12698	+	15354	15352	15355	+	1	6	7
13591	13591	13592	-	13698 -	-		-	7	0	7
13866	13864	13870	-	14000 -	-		-	0	7	7
13894	13891	13894	-	13912	13912	13915	+	6	1	7
13961	13960	13964	-	14068 -	-		-	3	4	7
14046	14044	14051	-	14199	14196	14199	-	3	4	7
14098	14096	14101	-	14262	14261	14262	-	2	5	7
15142	15141	15145	-	15299	15299	15301	-	3	4	7
15183	15183	15185	-	15246 -	-		-	3	4	7
157	156	161	-	173	173	174	+	5	1	6
308	305	312	+	343 -	-		-	3	3	6
647	643	651	-	725	724	725	-	3	3	6
647	643	651	-	773 -	-		-	3	3	6
655	654	661	-	11857	11856	11857	+	2	4	6
700	696	706	+	756	756	757	-	3	3	6
803	800	808	-	865	863	866	+	3	3	6
849	847	852	+	9147	9147	9150	+	5	1	6
851	849	853	-	9150	9147	9151	-	0	6	6
924	924	929	+	893 -	-		-	3	3	6
1020	1016	1024	+	961	957	961	-	3	3	6
1093	1093	1097	-	1183 -	-		-	3	3	6
1230	1227	1235	-	1406	1402	1406	-	3	3	6
1286	1285	1287	-	1363	1363	1364	-	3	3	6
1345	1344	1348	+	1331	1331	1332	-	3	3	6
1419	1414	1423	-	1515	1515	1516	-	3	3	6
1435	1431	1442	-	1538 -	-		+	3	3	6
1467	1467	1470	-	1545 -	-		-	3	3	6
1497	1495	1501	-	1676 -	-		-	3	3	6
1506	1506	1508	-	1551 -	-		-	6	0	6
1508	1507	1512	+	1446 -	-		-	3	3	6
1753	1753	1755	+	2328	2328	2329	+	3	3	6
1902	1901	1902	-	3314	3314	3315	+	2	4	6
2025	2023	2028	-	12506	12505	12507	-	2	4	6
2030	2029	2030	-	12509	12509	12510	-	5	1	6
2066	2066	2069	-	2429	2427	2429	-	3	3	6
2082	2082	2085	-	2187	2187	2188	-	3	3	6
2099	2096	2101	+	2177 -	-		-	3	3	6
2137	2134	2141	-	2264	2261	2264	-	3	3	6
2265	2262	2268	+	2194	2194	2195	-	3	3	6
2350	2347	2354	-	2441	2441	2443	-	3	3	6
2423	2420	2424	-	2512 -	-		-	3	3	6
2503 -	-		-	2525 -	-		+	3	3	6
2658	2655	2662	-	2802 -	-		-	3	3	6
2663	2662	2666	+	2715 -	-		-	3	3	6
2721	2718	2726	-	2805 -	-		-	3	3	6
2721	2718	2726	-	2857	2857	2860	-	3	3	6
2850	2849	2853	-	2915 -	-		-	0	6	6
3481	3477	3482	+	4755 -	-		+	3	3	6
3595	3593	3600	-	3698	3696	3699	-	4	2	6
3595	3593	3600	-	3708	3708	3710	-	3	3	6
3679 -	-		+	12175 -	-		+	3	3	6
3684	3683	3684	+	10824 -	-		+	3	3	6
3726	3723	3727	+	7365	7362	7365	-	3	3	6
3903	3899	3905	-	3998 -	-		-	3	3	6
3961	3957	3963	-	4039	4037	4041	-	3	3	6
4067	4065	4071	-	4120 -	-		-	3	3	6
4236	4233	4241	-	4348	4348	4352	-	3	3	6
4437	4433	4438	-	4503 -	-		-	3	3	6
4437	4433	4438	-	10721 -	-		-	6	0	6
4448	4447	4452	-	4632 -	-		-	3	3	6
4498	4494	4501	-	4635 -	-		-	3	3	6
4639	4635	4643	-	5191 -	-		-	3	3	6
4668	4665	4669	-	4783	4783	4784	-	3	3	6
4723	4720	4725	+	4749 -	-		-	3	3	6
4855	4851	4859	-	5093	5093	5094	-	3	3	6
4879	4877	4882	+	12104 -	-		+	3	3	6
5261	5259	5261	-	5367 -	-		-	3	3	6
5266	5265	5267	-	5411 -	-		-	4	2	6
5297	5295	5299	+	5270 -	-		-	3	3	6
5354	5351	5357	+	10855	10855	10856	+	3	3	6
5476	5471	5480	-	14194 -	-		-	0	6	6
5564	5564	5566	-	5636 -	-		-	3	3	6
5756	5755	5758	-	5882 -	-		-	3	3	6
5840	5836	5840	-	6077 -	-		-	3	3	6
5866	5865	5868	-	6014	6013	6014	-	2	4	6
6010	6006	6013	+	6183	6183	6186	-	3	3	6
6047	6047	6049	-	6077 -	-		-	3	3	6
6146	6144	6146	-	6323 -	-		-	3	3	6
6178	6174	6181	+	6224	6224	6225	-	3	3	6
6265	6263	6267	-	6365 -	-		-	3	3	6
6277	6275	6280	-	6408 -	-		-	3	3	6
6345	6343	6349	+	6383 -	-		-	3	3	6
6414	6414	6418	-	6474	6470	6474	-	3	3	6
6538	6531	6541	+	10842	10842	10843	+	6	0	6
6541	6538	6542	-	6653	6650	6653	-	2	4	6
6580	6577	6582	-	6699 -	-		-	3	3	6
6590	6586	6592	+	6731	6731	6732	+	3	3	6
6641	6637	6642	+	6703	6699	6703	-	3	3	6
7035	7035	7038	-	7119	7119	7120	-	3	3	6
7082	7081	7085	-	7145 -	-		-	3	3	6
7094	7090	7098	-	7161 -	-		-	3	3	6
7105	7100	7109	-	7232	7228	7237	-	3	3	6
7168	7165	7170	-	7263	7260	7263	-	3	3	6
7270	7268	7271	-	8595 -	-		+	6	0	6
7407	7407	7409	+	7388 -	-		-	3	3	6

7485	7485	7489	-	7518	-	-	-	3	3	6
7512	7507	7515	-	7638	7638	7639	-	3	3	6
7731	7731	7735	-	7799	-	-	-	3	3	6
7742	7742	7743	+	7664	-	-	-	3	3	6
7825	7821	7827	-	7894	-	-	-	3	3	6
7871	7870	7874	-	7979	7979	7981	-	1	5	6
7988	7987	7994	+	8148	8147	8150	-	2	4	6
8040	8037	8044	+	8088	8088	8090	+	3	3	6
8088	8084	8092	-	8237	8233	8237	-	3	3	6
8094	8092	8099	+	7991	-	-	-	3	3	6
8140	8137	8145	-	8198	-	-	-	3	3	6
8154	8152	8158	-	12921	-	-	+	5	1	6
8193	8191	8194	+	8161	-	-	-	3	3	6
8436	8430	8438	-	8530	8530	8531	-	1	5	6
8561	8558	8563	-	8675	8675	8676	-	3	3	6
8561	8558	8563	-	8692	-	-	-	3	3	6
8631	8629	8635	-	10962	-	-	+	0	6	6
8696	8696	8699	+	8662	-	-	-	3	3	6
8698	8697	8699	-	8766	-	-	-	0	6	6
8712	8709	8712	-	8860	-	-	-	3	3	6
8757	8757	8759	+	8717	8716	8717	-	4	2	6
8765	8762	8769	+	8708	-	-	+	2	4	6
8765	8762	8769	+	8711	8711	8712	-	3	3	6
8778	8776	8779	+	8695	8695	8697	-	3	3	6
8951	8947	8955	+	8863	8860	8864	-	3	3	6
8976	8974	8978	-	11491	-	-	+	3	3	6
9094	9091	9097	-	9232	9228	9232	-	3	3	6
9300	9298	9305	-	9462	-	-	-	3	3	6
9314	9311	9320	-	9439	9439	9440	-	3	3	6
9322	-	-	-	9403	-	-	-	6	0	6
9327	9323	9330	-	9407	-	-	-	0	6	6
9372	9372	9375	+	9335	9333	9336	-	3	3	6
9428	9423	9430	-	9475	-	-	-	3	3	6
9522	9519	9524	+	9635	-	-	-	3	3	6
9579	9575	9579	-	9622	9618	9622	-	3	3	6
9597	9593	9601	+	9525	-	-	-	3	3	6
9635	9631	9635	+	9522	-	-	-	3	3	6
9645	9645	9648	+	9557	9557	9559	-	3	3	6
10160	10160	10162	-	10225	-	-	-	3	3	6
10324	10320	10325	-	10415	10412	10415	-	3	3	6
10403	10402	10403	-	13047	-	-	+	0	6	6
10518	10518	10521	-	10544	10541	10544	+	3	3	6
10524	10524	10527	-	10585	-	-	-	3	3	6
10529	10529	10531	-	10609	-	-	-	3	3	6
10572	10569	10574	-	10635	10634	10637	-	3	3	6
10699	10698	10704	-	10764	-	-	-	3	3	6
10771	10771	10775	+	12664	12662	12666	-	2	4	6
10829	10828	10829	+	12332	-	-	+	6	0	6
10836	10836	10839	+	10849	-	-	+	3	3	6
10993	10989	10996	-	11121	11118	11121	-	3	3	6
11036	11031	11039	-	14427	14427	14428	+	3	3	6
11127	11123	11127	-	11257	-	-	-	3	3	6
11245	11241	11247	-	11403	-	-	-	3	3	6
11245	11241	11247	-	13219	-	-	+	3	3	6
11255	11255	11258	-	11296	-	-	-	3	3	6
11255	11255	11258	-	11340	-	-	-	3	3	6
11266	11262	11270	-	11305	-	-	-	3	3	6
11266	11262	11270	-	11415	11414	11415	-	3	3	6
11624	11620	11627	-	11796	11792	11796	-	3	3	6
11826	11822	11831	+	11793	-	-	-	3	3	6
11862	11862	11866	-	11984	-	-	-	3	3	6
11866	11865	11869	+	11790	11790	11791	-	3	3	6
11876	11876	11880	-	12012	-	-	-	3	3	6
11900	11898	11903	-	11986	11983	11986	-	3	3	6
11970	11968	11970	-	12020	-	-	-	3	3	6
12082	12080	12087	+	11981	11981	11982	-	3	3	6
12113	12110	12117	+	12060	-	-	-	3	3	6
12123	12121	12127	-	12194	-	-	-	3	3	6
12126	12124	12129	+	12088	12084	12088	-	3	3	6
12159	12155	12162	-	12182	12179	12182	+	3	3	6
12159	12155	12162	-	12203	-	-	-	6	0	6
12164	12164	12167	-	12207	-	-	-	0	6	6
12184	12179	12184	-	12302	12298	12302	-	3	3	6
12282	12282	12285	+	12322	-	-	-	3	3	6
12304	12299	12304	-	12395	12395	12396	-	3	3	6
12377	12373	12379	+	15109	15105	15110	+	3	3	6
12460	12456	12463	-	12556	-	-	-	3	3	6
12501	12501	12503	-	12581	12581	12582	-	3	3	6
12514	12510	12517	-	12640	-	-	-	3	3	6
12529	12526	12531	-	12641	12638	12642	-	3	3	6
12557	12553	12558	+	12514	12514	12515	+	3	3	6
12576	12572	12579	+	12467	-	-	-	3	3	6
12625	12623	12629	-	12783	12782	12786	-	3	3	6
12637	12634	12641	-	12753	12752	12753	-	3	3	6
12749	12749	12754	-	12933	-	-	-	3	3	6
12758	12756	12760	-	12949	12945	12949	-	3	3	6
12790	12788	12792	-	12857	-	-	-	3	3	6
12854	12854	12858	+	12834	-	-	-	3	3	6
12859	12859	12866	-	12896	-	-	+	3	3	6
12892	12888	12894	-	13054	13050	13054	-	3	3	6
12984	12984	12989	-	13026	-	-	-	3	3	6
13061	13061	13065	+	13100	13100	13101	-	3	3	6
13084	13081	13084	+	13024	-	-	-	3	3	6
13230	13226	13233	-	13327	13323	13327	+	3	3	6
13334	13331	13339	+	13445	13445	13446	-	3	3	6
13335	13335	13337	-	13394	-	-	-	6	0	6
13341	13340	13342	-	13400	13400	13401	-	0	6	6
13373	-	-	-	13533	-	-	+	3	3	6
13396	13396	13399	-	14457	14457	14458	-	3	3	6
13536	13535	13536	+	13634	-	-	-	3	3	6
13808	13804	13812	-	13936	-	-	-	6	0	6

13808	13804	13812	-	13944 -	-		-	0	6	6
13854	13853	13857	+	13821	13818	13821	-	3	3	6
13861	13857	13863	-	13879 -	-		+	3	3	6
13866	13864	13870	-	14201 -	-		-	3	3	6
13912	13908	13912	-	13894	13894	13898	+	6	0	6
13919	13915	13919	+	13968	13965	13969	-	3	3	6
13956	13954	13959	-	14064	14060	14064	-	4	2	6
13967	13963	13968	+	13919	13916	13921	-	3	3	6
13972	13969	13975	+	13958 -	-		-	3	3	6
13972	13969	13975	+	14191 -	-		-	3	3	6
13974	13969	13974	-	14082	14082	14084	-	3	3	6
14060	14055	14066	-	14236	14236	14239	-	3	3	6
14091	14087	14091	-	14255 -	-		-	5	1	6
14118	14114	14122	-	14254	14250	14254	-	3	3	6
14171	14169	14171	+	14107 -	-		-	3	3	6
14185	14183	14188	+	14146	14146	14147	-	3	3	6
14339	14334	14340	-	14383 -	-		-	3	3	6
14376	14373	14376	-	14435 -	-		-	3	3	6
14403	14399	14404	+	15034	15030	15034	+	3	3	6
14411	14408	14411	+	14386	14383	14386	-	3	3	6
14416	14414	14419	-	14595	14594	14595	-	3	3	6
14416	14414	14419	-	14604	14601	14604	+	3	3	6
14435	14433	14437	+	14455	14455	14457	-	4	2	6
14475	14472	14478	+	14462	14462	14463	-	3	3	6
14494	14494	14497	-	14543	14540	14543	-	3	3	6
14503	14499	14507	-	14531	14527	14531	+	4	2	6
14535	14534	14536	+	14482	14482	14483	-	3	3	6
14642	14639	14646	-	14768	14765	14770	-	3	3	6
14687	14687	14688	+	14640 -	-		-	3	3	6
14739	14738	14739	-	14898 -	-		-	3	3	6
14755 -	-		-	14760 -	-		+	3	3	6
14855	14852	14859	+	14771 -	-		-	3	3	6
14913	14910	14914	-	15065 -	-		-	3	3	6
15175	15172	15176	-	15384 -	-		-	3	3	6
15241	15239	15244	+	15208	15204	15209	-	3	3	6
15251	15247	15251	+	15269 -	-		+	3	3	6
15340	15339	15340	+	15369	15369	15370	-	3	3	6
596	593	601	-	685	684	685	-	3	2	5
611	608	616	-	671 -	-		-	5	0	5
620	618	623	-	676 -	-		-	0	5	5
889	885	893	-	1075	1075	1077	-	2	3	5
989	984	989	-	1112	1108	1113	-	2	3	5
1031	1027	1038	-	1067 -	-		+	3	2	5
1186	1182	1189	+	1134	1134	1138	-	3	2	5
1270	1266	1270	-	1336 -	-		-	5	0	5
1292	1289	1294	-	1379 -	-		-	2	3	5
1329	1326	1329	+	1381 -	-		-	3	2	5
1381 -	-		+	1329 -	-		-	3	2	5
1436	1432	1440	+	6691 -	-		+	0	5	5
1448	1447	1452	+	2238	2237	2238	+	2	3	5
1585	1585	1586	+	8144	8144	8146	+	4	1	5
1658	1655	1660	-	1785	1785	1787	-	4	1	5
1887	1883	1891	+	3329 -	-		-	0	5	5
1969	1965	1973	-	2090	2089	2090	-	3	2	5
2041	2040	2044	-	12522	12522	12523	-	3	2	5
2116	2112	2116	+	2081	2081	2085	-	3	2	5
2456	2454	2458	-	2555 -	-		-	5	0	5
2548	2545	2548	-	2676	2674	2676	-	1	4	5
3166	3166	3168	+	14062	14061	14062	+	3	2	5
3302	3299	3304	+	12207 -	-		-	5	0	5
3325	3324	3328	+	11127	11126	11127	+	4	1	5
3575	3572	3580	-	3661 -	-		-	3	2	5
3585	3584	3586	+	3636 -	-		-	3	2	5
3636	3632	3639	+	3585 -	-		-	3	2	5
4409	4405	4413	-	4517 -	-		-	3	2	5
4458	4455	4461	+	4404	4403	4404	-	3	2	5
4691	4690	4695	-	4832 -	-		-	5	0	5
4701	4697	4703	-	4838 -	-		-	0	5	5
4979	4977	4985	-	5013 -	-		-	4	1	5
5057	5054	5057	+	5082 -	-		+	2	3	5
5066	5065	5069	+	15096 -	-		-	0	5	5
5078	5075	5080	+	15083	15081	15083	-	4	1	5
5476	5471	5480	-	6935 -	-		+	0	5	5
6214	6208	6220	+	10226 -	-		-	5	0	5
6573	6570	6577	+	8271 -	-		-	4	1	5
6949	6946	6949	+	13057 -	-		+	3	2	5
7022	7020	7026	-	7211	7211	7213	-	1	4	5
7061	7060	7065	+	10633 -	-		-	5	0	5
7094	7090	7098	-	7233 -	-		-	2	3	5
7130	7128	7133	-	7142 -	-		+	2	3	5
7214	7210	7218	+	12064 -	-		+	0	5	5
7263	7258	7263	+	12426 -	-		+	5	0	5
7286	7282	7290	+	7257 -	-		-	2	3	5
7523	7519	7525	-	7592	7592	7594	-	3	2	5
7593	7591	7597	-	8024 -	-		-	5	0	5
7599	7598	7603	-	8030 -	-		-	0	5	5
7772	7769	7776	+	7715 -	-		+	3	2	5
7830	7829	7835	-	11168	11168	11171	-	0	5	5
8004	8000	8006	-	8011 -	-		+	5	0	5
8201	8200	8202	-	8208 -	-		+	5	0	5
8244	8241	8247	-	8385	8382	8386	-	2	3	5
8583	8579	8586	-	8640	8638	8643	-	3	2	5
9010	9004	9015	+	8980	8979	8980	-	3	2	5
9026	9022	9032	-	11820 -	-		-	0	5	5
9274	9269	9276	-	9311	9311	9314	-	1	4	5
9428	9423	9430	-	9636	9633	9636	-	1	4	5
9451	9448	9452	-	9518 -	-		+	3	2	5
9564	9559	9568	-	9712 -	-		-	3	2	5
9999	9997	10003	-	10078	10076	10079	-	2	3	5
10408	10405	10413	+	13043	13043	13046	-	3	2	5
10473	10469	10476	-	10516	10516	10520	+	2	3	5

10490	10489	10494	-	10518	10518	10519	+	3	2	5
10517	10515	10517	+	10496	10496	10498	-	4	1	5
10592	10590	10598	-	10661	10658	10661	-	2	3	5
10707	10706	10711	-	14863	14861	14864	+	1	4	5
10777	10776	10780	+	12659	12657	12661	-	2	3	5
10792	10788	10793	-	10901	10901	10902	-	3	2	5
11162	-	-	-	11187	-	-	+	3	2	5
11215	11213	11218	-	11283	11280	11283	-	2	3	5
11245	11241	11247	-	11369	-	-	-	5	0	5
11250	11249	11251	-	11374	-	-	-	0	5	5
11255	11255	11258	-	11381	-	-	-	3	2	5
11344	11343	11348	-	11439	11438	11440	-	3	2	5
11497	11493	11497	+	11454	-	-	-	3	2	5
11955	11951	11958	-	12064	-	-	+	0	5	5
12064	12059	12066	-	15240	15240	15242	-	5	0	5
12064	12059	12066	-	15291	15288	15294	-	5	0	5
12097	12094	12099	+	12059	-	-	-	0	5	5
12103	12103	12105	+	12053	-	-	-	5	0	5
12164	12164	12167	-	12317	12315	12317	-	1	4	5
12199	12199	12203	-	12402	-	-	-	5	0	5
12294	12290	12298	-	12391	12387	12391	-	3	2	5
12326	12322	12330	-	12446	12445	12446	-	4	1	5
12519	12519	12520	-	12612	-	-	-	0	5	5
12823	12820	12828	-	15365	-	-	-	0	5	5
12881	12878	12884	-	12984	-	-	-	3	2	5
13499	13496	13502	-	13580	13579	13584	-	3	2	5
13861	13857	13863	-	13995	-	-	-	5	0	5
13884	13883	13884	+	13803	-	-	-	0	5	5
13891	-	-	+	13796	-	-	-	5	0	5
13938	13936	13942	-	14058	-	-	-	3	2	5
14156	14152	14157	-	14535	-	-	-	5	0	5
14161	14159	14165	-	14538	-	-	-	0	5	5
14203	14199	14205	+	14162	14162	14163	-	1	4	5
14321	14320	14321	-	14354	-	-	-	5	0	5
14328	14323	14332	-	14358	-	-	-	0	5	5
14339	14334	14340	-	14507	14507	14508	-	2	3	5
14962	14960	14962	-	15135	-	-	-	2	3	5
14987	14987	14988	+	14880	14879	14880	-	3	2	5
15256	-	-	+	15195	-	-	+	1	4	5
14	12	16	-	5474	-	-	-	4	0	4
30	29	34	-	76	-	-	-	2	2	4
98	96	98	+	213	-	-	-	2	2	4
173	170	173	-	157	-	-	+	4	0	4
173	169	173	+	157	-	-	-	2	2	4
231	229	234	-	243	243	246	+	4	0	4
242	240	245	-	386	-	-	-	2	2	4
263	259	267	-	371	370	373	-	2	2	4
280	274	282	-	349	348	349	-	0	4	4
291	287	293	+	301	-	-	+	2	2	4
320	315	320	-	569	566	569	-	2	2	4
338	337	341	+	372	372	373	+	2	2	4
349	347	349	-	560	-	-	-	2	2	4
354	353	361	-	555	-	-	-	2	2	4
369	369	373	+	461	-	-	-	2	2	4
397	394	399	-	607	-	-	-	2	2	4
407	405	410	+	545	-	-	+	2	2	4
429	424	433	-	12303	-	-	+	0	4	4
451	448	454	-	487	-	-	-	4	0	4
451	448	454	-	619	618	619	-	4	0	4
459	456	460	-	490	-	-	-	0	4	4
459	456	460	-	627	-	-	-	0	4	4
467	467	470	-	541	-	-	-	2	2	4
495	491	495	-	851	848	851	+	2	2	4
522	518	522	-	761	-	-	-	2	2	4
560	557	562	-	699	696	699	-	2	2	4
620	618	623	-	897	-	-	-	2	2	4
626	625	628	-	883	-	-	-	2	2	4
628	627	631	+	1072	-	-	+	2	2	4
635	633	637	-	1577	-	-	-	2	2	4
656	652	659	+	583	583	587	-	2	2	4
677	676	681	+	776	-	-	-	2	2	4
684	682	684	-	833	832	833	-	2	2	4
689	685	692	+	1001	-	-	-	2	2	4
717	-	-	-	878	-	-	-	2	2	4
772	770	772	+	895	895	896	+	2	2	4
789	787	790	-	900	-	-	-	2	2	4
824	820	825	-	962	962	963	-	2	2	4
857	854	860	-	9155	9153	9155	-	1	3	4
889	885	893	-	924	-	-	+	2	2	4
901	896	905	-	980	-	-	-	2	2	4
972	972	975	-	1112	1112	1113	-	2	2	4
994	990	997	-	1173	1169	1173	-	1	3	4
1020	1016	1024	+	1009	1009	1010	-	3	1	4
1037	1036	1042	+	1062	1060	1062	-	3	1	4
1040	1039	1044	-	1298	-	-	-	2	2	4
1040	1039	1044	-	1632	-	-	+	2	2	4
1064	1059	1068	-	1093	-	-	-	2	2	4
1068	1067	1070	+	1339	-	-	-	2	2	4
1074	1073	1075	+	1084	1084	1085	-	2	2	4
1081	1077	1081	-	1249	-	-	-	2	2	4
1093	1093	1097	-	1223	-	-	-	2	2	4
1099	1099	1101	-	1249	-	-	-	2	2	4
1121	1117	1124	+	1205	-	-	+	2	2	4
1123	1121	1123	-	1158	1156	1158	+	2	2	4
1142	1141	1145	-	1338	1336	1339	-	2	2	4
1179	1176	1183	-	1240	1236	1240	+	2	2	4
1179	1176	1183	-	1281	1281	1284	-	2	2	4
1220	1216	1224	-	1437	1437	1438	-	2	2	4
1244	1241	1245	-	1436	-	-	-	2	2	4
1256	1256	1259	-	1369	-	-	-	2	2	4
1279	1276	1283	+	1357	-	-	+	2	2	4

1293	1289	1297	+	1177	-	-	-	2	2	4
1299	1298	1303	-	1346	1342	1346	+	2	2	4
1299	1298	1303	-	1380	-	-	-	2	2	4
1338	1335	1340	-	1600	-	-	-	2	2	4
1345	1344	1348	+	1307	-	-	-	2	2	4
1355	1354	1359	+	1307	1304	1307	-	2	2	4
1383	1383	1388	-	1473	-	-	+	2	2	4
1390	1389	1393	-	1447	1444	1447	-	2	2	4
1407	1404	1410	-	1438	1435	1438	+	2	2	4
1435	1431	1442	-	1506	1506	1507	-	2	2	4
1495	1492	1498	+	1421	-	-	-	2	2	4
1637	1634	1637	-	1795	-	-	-	2	2	4
1642	1640	1647	-	1696	-	-	+	0	4	4
1669	1669	1674	-	12064	-	-	+	0	4	4
1697	1697	1702	-	1834	-	-	+	2	2	4
1720	1716	1720	-	1857	-	-	-	2	2	4
1725	1724	1729	-	1782	-	-	-	2	2	4
1798	1796	1799	-	1914	-	-	-	2	2	4
1956	-	-	+	1937	-	-	-	2	2	4
1986	1984	1989	-	2099	-	-	-	2	2	4
2025	2023	2028	-	2105	-	-	+	2	2	4
2035	2035	2039	-	2108	-	-	-	4	0	4
2041	2040	2044	-	2095	-	-	-	2	2	4
2041	2040	2044	-	2113	-	-	-	0	4	4
2100	2099	2103	-	2217	-	-	-	2	2	4
2126	2126	2127	+	2468	-	-	+	2	2	4
2137	2134	2141	-	2275	-	-	-	2	2	4
2137	2137	2138	+	2480	-	-	+	2	2	4
2143	2143	2145	-	2189	2189	2190	-	2	2	4
2192	-	-	+	2268	-	-	-	2	2	4
2200	2197	2200	-	2214	-	-	+	2	2	4
2292	2288	2294	-	9684	9680	9684	+	4	0	4
2295	2291	2298	+	2435	2433	2435	+	2	2	4
2326	2322	2330	-	2418	2415	2418	-	2	2	4
2328	2327	2331	+	2357	-	-	-	2	2	4
2360	2356	2363	+	2329	-	-	-	2	2	4
2363	2358	2366	-	2438	-	-	-	2	2	4
2394	2390	2398	+	2336	2335	2336	+	2	2	4
2443	2439	2447	-	2466	-	-	+	3	1	4
2443	2438	2446	+	2481	2481	2482	+	2	2	4
2451	2448	2455	+	2468	-	-	+	2	2	4
2461	-	-	-	2560	-	-	-	0	4	4
2473	2472	2478	-	2514	-	-	-	2	2	4
2481	2477	2481	+	2653	-	-	+	2	2	4
2493	2491	2498	+	2600	-	-	+	2	2	4
2518	2514	2521	-	2652	-	-	-	2	2	4
2536	2535	2539	+	2400	-	-	-	2	2	4
2562	2559	2565	-	2670	-	-	-	3	1	4
2709	2707	2713	+	2657	2653	2657	+	4	0	4
2709	2707	2713	+	2672	-	-	-	0	4	4
2809	2807	2810	-	2832	-	-	+	3	1	4
2877	2874	2879	-	3040	-	-	-	2	2	4
2910	-	-	-	3525	-	-	-	2	2	4
2938	2934	2941	-	3019	-	-	-	2	2	4
2951	2951	2956	-	2982	-	-	-	4	0	4
2951	2951	2956	-	2987	-	-	-	0	4	4
2951	2951	2956	-	3044	-	-	+	2	2	4
2993	2990	2996	-	3089	-	-	-	2	2	4
2998	2995	3002	+	6216	-	-	-	0	4	4
3010	3007	3015	-	3129	-	-	-	2	2	4
3014	3011	3019	+	3038	-	-	-	2	2	4
3018	3018	3019	-	3115	-	-	-	4	0	4
3110	3108	3110	+	3057	3055	3057	+	3	1	4
3115	3113	3120	+	3136	3132	3136	-	2	2	4
3125	3121	3128	-	3242	3239	3242	-	2	2	4
3204	3200	3208	+	3175	-	-	+	2	2	4
3231	3225	3236	+	10873	10871	10873	-	4	0	4
3231	3225	3236	+	12143	12143	12147	-	4	0	4
3231	3225	3236	+	12182	-	-	+	4	0	4
3281	3278	3283	+	11951	11948	11951	+	2	2	4
3295	3295	3298	-	7699	-	-	+	4	0	4
3320	3318	3324	-	3355	3355	3356	-	2	2	4
3332	3329	3336	-	3637	3635	3637	-	2	2	4
3350	3347	3350	-	3848	-	-	-	2	2	4
3356	3352	3360	-	12423	-	-	+	4	0	4
3356	3352	3360	-	12620	12620	12621	+	4	0	4
3387	3386	3391	-	3712	-	-	-	2	2	4
3410	3408	3412	-	3601	-	-	+	2	2	4
3441	3437	3445	-	3532	3530	3532	-	2	2	4
3512	3509	3512	-	3580	-	-	-	2	2	4
3650	3650	3653	+	3641	3638	3641	-	2	2	4
3726	3723	3727	+	7358	-	-	-	2	2	4
3745	3742	3747	+	3673	-	-	-	0	4	4
3777	3777	3779	+	3861	3861	3862	+	2	2	4
3836	3833	3839	-	4033	-	-	-	2	2	4
3841	3841	3844	-	4039	4036	4039	-	2	2	4
3866	3862	3870	+	3845	3842	3845	-	2	2	4
3885	3881	3888	-	4036	4035	4039	-	2	2	4
3903	3899	3905	-	3965	3961	3965	-	2	2	4
3935	3932	3936	-	4266	4266	4267	-	2	2	4
3936	3934	3938	+	4312	4312	4313	+	2	2	4
3936	3934	3938	+	4320	-	-	+	2	2	4
4009	4005	4012	+	5308	5306	5308	+	2	2	4
4060	4058	4064	+	4125	-	-	-	2	2	4
4067	4065	4071	-	4165	-	-	-	2	2	4
4098	4094	4101	-	4248	-	-	-	2	2	4
4236	4233	4241	-	4390	-	-	-	2	2	4
4274	4270	4278	-	4286	-	-	+	2	2	4
4348	4348	4349	-	4499	-	-	-	2	2	4
4363	-	-	+	4349	-	-	-	2	2	4
4437	4436	4441	+	4571	4571	4572	-	2	2	4

4437	4436	4441	+	11469	-	-	+	2	2	4
4458	4455	4461	+	4528	4528	4529	+	2	2	4
4566	4564	4566	-	4644	-	-	+	2	2	4
4590	4590	4592	-	4792	4792	4793	-	2	2	4
4658	4657	4662	-	5183	5181	5183	-	2	2	4
4660	4660	4664	+	4707	4707	4708	+	2	2	4
4691	4690	4695	-	14064	-	-	-	2	2	4
4750	4747	4753	+	4666	-	-	-	2	2	4
4792	4791	4792	-	9790	9790	9791	+	3	1	4
4823	-	-	-	4866	-	-	-	2	2	4
4832	4832	4836	+	5028	-	-	+	2	2	4
4866	4862	4869	+	4955	-	-	+	2	2	4
4904	4900	4907	-	5232	-	-	-	2	2	4
4926	4923	4929	-	5236	-	-	-	2	2	4
4971	4968	4974	-	5009	5006	5009	+	2	2	4
5020	5017	5022	-	5141	-	-	-	2	2	4
5039	5037	5042	-	5102	-	-	-	2	2	4
5046	5045	5051	-	5259	-	-	-	1	3	4
5144	5144	5148	-	6689	-	-	+	2	2	4
5153	5151	5156	+	9575	-	-	+	2	2	4
5230	5228	5231	+	5185	5185	5187	-	2	2	4
5264	5264	5265	+	5517	5517	5518	+	2	2	4
5276	5273	5280	-	5423	-	-	-	2	2	4
5276	5273	5280	-	5455	-	-	-	2	2	4
5276	5273	5280	-	5524	-	-	+	2	2	4
5321	5319	5321	+	5352	-	-	+	2	2	4
5321	5319	5321	+	5433	-	-	+	2	2	4
5333	5329	5336	+	5476	5476	5477	-	2	2	4
5356	5353	5360	-	12345	-	-	-	2	2	4
5377	5373	5381	-	5585	5585	5586	-	2	2	4
5398	5398	5399	+	5344	-	-	-	2	2	4
5414	5411	5418	+	5452	-	-	+	2	2	4
5463	5460	5466	+	5399	-	-	-	2	2	4
5476	5471	5480	-	5636	5633	5636	-	2	2	4
5536	5532	5541	-	6001	-	-	-	2	2	4
5564	5564	5566	-	5647	-	-	-	2	2	4
5658	5655	5663	-	5761	-	-	-	2	2	4
5701	5698	5704	-	5811	-	-	-	2	2	4
5714	5713	5715	+	5687	-	-	-	2	2	4
5736	5735	5738	-	5897	5897	5898	-	2	2	4
5756	5755	5758	-	6018	-	-	-	2	2	4
5809	5806	5809	-	5870	-	-	+	4	0	4
5815	5814	5816	-	5864	-	-	+	0	4	4
5820	5819	5823	-	6061	-	-	-	2	2	4
5831	5828	5833	+	5845	-	-	+	2	2	4
5853	5851	5853	+	5914	-	-	+	2	2	4
5873	5873	5876	+	5853	5850	5853	-	2	2	4
6037	6034	6040	-	6178	-	-	-	2	2	4
6099	6096	6103	-	6228	-	-	-	2	2	4
6214	6208	6220	+	9066	-	-	-	4	0	4
6214	6208	6220	+	10815	10815	10819	-	4	0	4
6214	6208	6220	+	13188	13188	13190	+	4	0	4
6217	6214	6217	-	6369	-	-	-	2	2	4
6229	6226	6232	+	6555	-	-	-	2	2	4
6317	6312	6317	-	6409	6405	6409	-	3	1	4
6407	6405	6407	-	6448	-	-	-	2	2	4
6450	6447	6453	-	6623	6623	6626	-	2	2	4
6580	6577	6582	-	6605	-	-	+	2	2	4
6599	6595	6599	+	6677	-	-	+	2	2	4
6644	6644	6646	-	6686	6683	6686	-	2	2	4
6675	6671	6680	-	6818	-	-	-	2	2	4
6699	6695	6705	+	6637	-	-	-	2	2	4
6743	-	-	+	6723	-	-	-	2	2	4
6783	-	-	+	6952	-	-	-	2	2	4
6815	6811	6819	-	6834	-	-	+	4	0	4
6815	6813	6817	+	6834	-	-	-	2	2	4
6834	6834	6838	-	6815	-	-	+	4	0	4
6922	6919	6925	-	7018	-	-	+	2	2	4
6922	6919	6925	-	7163	7163	7164	-	2	2	4
6963	6959	6966	-	7109	-	-	-	2	2	4
7155	7152	7160	-	7233	-	-	-	2	2	4
7214	7210	7218	+	7184	-	-	-	2	2	4
7263	7258	7263	+	7359	-	-	-	2	2	4
7272	7272	7273	+	7336	-	-	-	2	2	4
7377	7377	7378	-	7588	-	-	-	4	0	4
7389	7386	7392	+	7332	7332	7333	-	2	2	4
7485	7485	7489	-	7515	7512	7515	+	2	2	4
7496	7494	7500	-	7572	-	-	-	2	2	4
7496	7494	7500	-	13552	13552	13556	-	0	4	4
7512	7507	7515	-	7548	7544	7548	-	2	2	4
7548	7548	7552	+	7705	7704	7705	-	2	2	4
7566	7565	7567	-	7973	7973	7974	-	2	2	4
7570	7566	7571	+	7684	-	-	-	2	2	4
7626	7623	7628	-	7699	7699	7700	-	2	2	4
7683	7679	7684	-	7843	-	-	-	2	2	4
7694	7694	7698	-	7760	-	-	-	2	2	4
7711	7710	7715	-	7776	-	-	-	2	2	4
7742	7739	7745	-	7833	7830	7833	-	2	2	4
7752	7751	7753	+	7740	-	-	-	2	2	4
7772	7769	7776	+	7698	-	-	+	2	2	4
7810	7807	7818	-	7826	7825	7826	+	2	2	4
7830	7827	7833	+	9198	-	-	+	2	2	4
7856	7852	7857	-	8060	-	-	-	2	2	4
7856	7852	7857	-	10717	-	-	-	4	0	4
7866	7861	7866	-	7974	-	-	-	4	0	4
7919	7915	7920	-	7907	-	-	+	3	1	4
7922	7919	7922	+	7864	-	-	-	2	2	4
7937	7934	7939	+	7899	-	-	-	2	2	4
7949	7948	7957	-	8034	-	-	-	2	2	4
7960	7957	7963	+	7924	-	-	-	2	2	4
7960	7957	7963	+	7949	7947	7949	-	2	2	4

7981	7977	7986	+	7964	-	-	-	2	2	4
8000	7996	8004	+	7896	-	-	-	2	2	4
8056	8055	8059	-	8176	-	-	-	2	2	4
8127	8124	8130	+	8141	-	-	-	2	2	4
8154	8152	8158	-	8264	-	-	-	2	2	4
8218	8216	8219	+	8184	8184	8185	-	2	2	4
8231	8229	8231	-	8340	-	-	-	2	2	4
8244	8241	8247	-	8375	-	-	-	2	2	4
8305	8303	8306	+	8263	8262	8263	-	2	2	4
8333	8331	8336	+	8385	-	-	-	2	2	4
8342	8339	8346	-	10965	10962	10968	+	1	3	4
8345	8341	8348	+	8401	8398	8401	-	2	2	4
8396	8392	8397	+	8332	-	-	-	2	2	4
8416	8414	8419	+	15347	15347	15350	+	2	2	4
8436	8430	8438	-	8525	8525	8526	-	4	0	4
8520	8519	8526	+	12064	-	-	+	0	4	4
8531	8531	8533	-	8665	-	-	-	2	2	4
8567	8566	8567	-	8679	8679	8680	-	2	2	4
8611	8607	8615	-	8659	8659	8660	-	2	2	4
8671	8671	8675	-	8749	-	-	-	2	2	4
8746	8742	8749	+	8726	8723	8727	-	2	2	4
8752	8752	8754	+	8722	-	-	-	0	4	4
8791	8788	8794	-	8925	-	-	-	2	2	4
8808	8804	8809	+	8874	8874	8875	+	2	2	4
8816	8812	8816	-	8985	8983	8985	-	2	2	4
8843	-	-	+	8765	8765	8766	+	2	2	4
8917	8913	8917	+	8968	-	-	-	2	2	4
8949	-	-	-	9038	-	-	-	4	0	4
8951	8947	8955	+	11347	11347	11348	-	2	2	4
8954	8953	8956	-	9001	-	-	-	4	0	4
8954	8953	8956	-	9034	-	-	-	4	0	4
8960	8957	8964	-	9006	-	-	-	0	4	4
8960	8957	8964	-	9038	-	-	-	0	4	4
8960	8957	8964	-	9145	9141	9145	-	2	2	4
8973	8972	8976	+	9014	9014	9015	-	2	2	4
9003	9002	9003	+	9034	-	-	+	2	2	4
9010	9004	9015	+	8937	-	-	-	2	2	4
9081	9078	9083	-	9289	-	-	-	2	2	4
9105	9105	9106	+	9006	9006	9007	-	2	2	4
9126	9125	9131	-	9163	-	-	+	2	2	4
9133	9132	9136	-	9222	-	-	-	2	2	4
9158	9156	9161	-	9302	-	-	-	2	2	4
9158	9156	9161	-	9308	9308	9309	-	2	2	4
9196	9193	9199	-	9386	9383	9386	-	2	2	4
9252	9250	9258	+	9240	9240	9241	-	2	2	4
9266	9266	9267	-	9362	-	-	-	2	2	4
9274	9269	9276	-	9335	9335	9336	-	2	2	4
9300	9298	9305	-	9467	9467	9468	-	2	2	4
9349	9346	9350	+	9360	9359	9363	-	2	2	4
9366	9363	9369	+	9342	9339	9343	-	2	2	4
9379	9376	9384	-	9447	-	-	-	2	2	4
9451	9448	9452	-	9543	9542	9543	-	2	2	4
9522	9519	9524	+	9599	-	-	-	2	2	4
9540	9536	9541	-	9669	-	-	-	2	2	4
9540	9536	9541	-	9730	9730	9731	+	2	2	4
9540	9539	9544	+	9520	9520	9521	-	2	2	4
9564	9559	9568	-	9669	9668	9669	-	2	2	4
9584	9582	9588	+	9697	-	-	-	2	2	4
9594	9590	9596	-	9685	-	-	-	2	2	4
9604	9603	9606	+	9707	9705	9707	+	2	2	4
9658	9657	9662	+	9677	9673	9677	-	2	2	4
9697	9693	9700	+	9680	9680	9681	-	1	3	4
9704	9704	9706	+	9672	9672	9673	-	2	2	4
9710	9710	9713	+	9906	-	-	-	2	2	4
9773	9772	9773	-	9906	-	-	-	2	2	4
9829	9829	9832	-	9943	9943	9946	-	4	0	4
9834	9834	9838	-	9948	-	-	-	0	4	4
9905	9904	9909	-	10065	10062	10065	-	2	2	4
9926	9924	9928	-	10035	-	-	-	2	2	4
9935	9935	9938	+	10018	10015	10018	+	2	2	4
9949	9945	9952	-	14112	14112	14113	-	1	3	4
9994	9994	9995	+	9970	9969	9970	-	2	2	4
10031	10029	10032	-	10096	-	-	+	2	2	4
10056	10054	10056	-	10081	-	-	-	4	0	4
10061	10061	10067	-	10086	-	-	-	0	4	4
10061	10061	10067	-	10121	10118	10121	+	2	2	4
10061	10061	10067	-	10158	-	-	-	2	2	4
10077	10074	10082	-	10158	-	-	-	2	2	4
10128	10128	10130	-	10241	10241	10242	-	2	2	4
10128	10128	10130	-	14012	-	-	+	2	2	4
10189	10185	10189	-	10337	-	-	-	2	2	4
10245	10242	10247	+	10362	-	-	-	2	2	4
10273	10269	10275	-	10304	-	-	-	2	2	4
10273	10269	10275	-	10430	10428	10430	-	2	2	4
10313	10311	10314	-	10389	10385	10389	-	4	0	4
10386	10382	10386	+	10221	-	-	-	2	2	4
10540	-	-	-	10622	-	-	-	4	0	4
10545	-	-	-	10627	-	-	-	0	4	4
10590	10585	10590	+	10533	-	-	-	4	0	4
10721	10719	10724	-	14849	14846	14849	+	3	1	4
10744	10744	10746	-	12689	-	-	+	3	1	4
10771	10771	10775	+	13531	-	-	+	2	2	4
10797	10795	10802	-	11037	11037	11038	-	2	2	4
10812	10809	10814	+	10759	10756	10759	-	2	2	4
10841	10838	10844	-	10988	-	-	-	2	2	4
10841	10838	10844	-	11011	-	-	-	2	2	4
10915	10911	10917	-	11557	-	-	+	2	2	4
10939	10935	10943	-	10985	10983	10985	-	2	2	4
11005	11005	11008	-	11061	-	-	+	3	1	4
11054	-	-	+	11030	-	-	-	2	2	4
11061	11058	11061	+	10979	10979	10983	+	3	1	4

11203	11200	11203	+	11329 -	-		-	2	2	4
11222	11220	11226	-	11283	11283	11284	-	2	2	4
11313	11311	11316	-	11503 -	-		-	2	2	4
11323	11319	11323	+	11381 -	-		-	2	2	4
11337	11337	11340	+	11440	11438	11440	-	2	2	4
11369	11369	11371	-	11440	11440	11442	-	1	3	4
11383	11379	11385	+	11372 -	-		-	2	2	4
11398	11394	11401	+	11341	11341	11342	-	2	2	4
11545	11545	11547	-	11626 -	-		-	2	2	4
11577	11573	11577	-	11747 -	-		-	2	2	4
11605 -	-		-	11711 -	-		-	0	4	4
11610	11610	11614	-	11729 -	-		-	2	2	4
11634	11632	11638	-	11662 -	-		-	2	2	4
11726	11724	11726	+	11625 -	-		-	2	2	4
11742	11742	11746	-	12619 -	-		+	2	2	4
11800	11796	11803	-	12706 -	-		-	2	2	4
11805 -	-		-	11805 -	-		+	4	0	4
11811	11811	11812	-	12705 -	-		-	2	2	4
11826	11822	11831	+	11703 -	-		-	2	2	4
11866	11865	11869	+	11931 -	-		-	2	2	4
11929	11928	11931	+	12299 -	-		-	2	2	4
11939 -	-		-	12057 -	-		-	2	2	4
11955	11951	11958	-	11971	11967	11971	+	2	2	4
12000	11996	12002	+	12167 -	-		-	2	2	4
12002	11998	12003	-	12125	12121	12125	-	2	2	4
12033	12031	12036	+	11988 -	-		-	2	2	4
12064	12059	12066	-	12803	12802	12805	-	2	2	4
12064	12059	12066	-	12887	12885	12887	-	4	0	4
12105	12105	12110	-	12338 -	-		-	2	2	4
12145	12141	12145	+	12268 -	-		+	2	2	4
12167	12164	12169	+	14219	14219	14220	+	2	2	4
12167	12164	12169	+	14267 -	-		+	2	2	4
12181	12177	12185	+	14219	14215	14219	+	2	2	4
12209	12205	12209	+	12181	12181	12182	-	2	2	4
12232	12229	12232	-	12350 -	-		-	2	2	4
12281	12278	12282	-	12390	12390	12393	-	3	1	4
12322	12319	12324	+	12350 -	-		-	2	2	4
12356	12356	12358	-	13899 -	-		-	2	2	4
12361	12360	12361	-	12461 -	-		-	2	2	4
12388	12385	12393	-	12485	12485	12487	-	2	2	4
12388	12385	12393	-	14344 -	-		+	2	2	4
12402	12402	12405	+	12679 -	-		+	2	2	4
12420	12418	12420	-	12438	12436	12438	+	2	2	4
12460	12456	12463	-	12627 -	-		-	2	2	4
12460	12456	12463	-	12668 -	-		-	2	2	4
12461	12460	12463	+	12438	12437	12440	-	2	2	4
12493	12493	12495	+	12360 -	-		-	2	2	4
12514	12510	12517	-	12670 -	-		-	2	2	4
12618	12616	12622	-	12738 -	-		-	2	2	4
12625	12623	12629	-	12753	12750	12753	-	2	2	4
12625	12623	12629	-	13760 -	-		-	2	2	4
12672	12669	12672	-	13022 -	-		-	2	2	4
12672	12668	12674	+	12705	12703	12705	+	2	2	4
12705	12701	12710	+	12640 -	-		-	2	2	4
12715	12711	12715	-	12774	12770	12774	-	2	2	4
12743	12739	12747	+	12661 -	-		-	2	2	4
12762	12760	12763	+	12804	12802	12805	+	2	2	4
12765	12763	12768	-	12802 -	-		+	2	2	4
12806	12805	12806	+	12893 -	-		-	2	2	4
12811	12809	12811	+	12795	12795	12796	-	2	2	4
12859	12859	12866	-	13000	13000	13002	-	4	0	4
12868 -	-		-	13006 -	-		-	0	4	4
13027	13026	13028	-	13679	13679	13680	+	2	2	4
13034	13031	13035	-	13173	13169	13173	-	2	2	4
13039	13037	13040	-	13114 -	-		+	2	2	4
13039	13037	13040	-	13197	13197	13199	-	2	2	4
13048	13047	13048	-	13213 -	-		-	2	2	4
13070	13067	13073	+	13014 -	-		-	2	2	4
13084	13081	13084	+	13003	13003	13004	-	2	2	4
13127	13124	13131	+	13182 -	-		-	2	2	4
13206 -	-		+	13247 -	-		-	2	2	4
13230	13226	13233	-	13254	13254	13255	+	2	2	4
13235	13231	13236	+	13200	13200	13201	-	2	2	4
13237	13236	13240	-	13361 -	-		-	2	2	4
13247	13243	13247	+	13206 -	-		-	2	2	4
13284	13284	13287	-	13668 -	-		-	2	2	4
13284	13284	13287	-	13674	13674	13675	+	2	2	4
13284	13284	13287	-	13676 -	-		-	2	2	4
13303	13299	13306	+	13320 -	-		-	2	2	4
13314	13310	13318	+	13243	13242	13243	-	2	2	4
13328	13324	13329	+	13234 -	-		-	0	4	4
13334	13331	13339	+	13228 -	-		-	4	0	4
13384	13383	13389	+	13342	13342	13343	-	2	2	4
13414	13410	13414	+	13386	13386	13387	-	2	2	4
13448	13445	13448	-	13587	13585	13587	-	2	2	4
13473	13469	13474	-	13511 -	-		-	2	2	4
13479	13477	13483	-	13517 -	-		-	2	2	4
13491 -	-		+	13536 -	-		+	2	2	4
13499	13496	13502	-	13663 -	-		-	2	2	4
13518	13518	13520	+	13640 -	-		-	2	2	4
13528	13526	13530	+	13504	13504	13505	-	2	2	4
13538	13537	13542	-	13587	13587	13588	-	2	2	4
13549	13549	13551	+	13607	13607	13608	-	2	2	4
13664	13662	13669	-	13763 -	-		-	2	2	4
13664	13662	13669	-	13778	13775	13778	-	2	2	4
13680 -	-		-	14200 -	-		-	2	2	4
13751	13748	13754	-	13803 -	-		-	2	2	4
13768	13768	13769	+	13721 -	-		-	2	2	4
13800	13796	13803	+	13849 -	-		-	2	2	4
13802	13801	13802	-	14877 -	-		-	2	2	4
13823	13823	13824	+	13781	13781	13782	-	2	2	4

13829	-	-	+	13776	-	-	-	2	2	4
13848	13846	13849	+	14178	-	-	-	2	2	4
13851	13849	13854	-	14806	14806	14807	+	2	2	4
13879	13876	13882	+	13861	-	-	-	2	2	4
13903	13903	13904	+	13874	13874	13875	-	2	2	4
13919	13919	13922	-	13994	-	-	-	4	0	4
13919	13919	13922	-	14027	-	-	-	2	2	4
13949	13949	13950	+	13959	-	-	-	2	2	4
13956	13954	13959	-	14057	14056	14058	-	3	1	4
13967	13963	13968	+	14198	14198	14199	-	2	2	4
13972	13969	13975	+	13894	-	-	+	2	2	4
13974	13969	13974	-	14114	-	-	-	2	2	4
14028	14027	14029	-	14204	14204	14205	-	2	2	4
14045	-	-	+	13920	-	-	-	2	2	4
14053	-	-	-	14155	-	-	-	4	0	4
14060	14055	14066	-	14158	-	-	-	0	4	4
14077	14073	14081	+	13992	-	-	-	2	2	4
14077	14073	14081	+	14249	-	-	+	2	2	4
14117	14115	14119	+	14035	-	-	-	2	2	4
14156	14152	14157	-	14191	-	-	-	4	0	4
14161	14159	14165	-	14194	-	-	-	0	4	4
14210	14208	14210	+	14182	-	-	-	0	4	4
14215	14215	14218	+	14177	-	-	-	4	0	4
14220	14217	14222	-	14362	-	-	+	2	2	4
14339	14334	14340	-	14413	-	-	-	2	2	4
14339	14334	14340	-	14454	-	-	-	2	2	4
14339	14334	14340	-	14478	14478	14479	-	2	2	4
14346	14341	14346	-	14480	14480	14483	-	2	2	4
14353	14347	14357	-	14468	14468	14469	-	2	2	4
14369	14369	14373	+	14324	-	-	-	2	2	4
14398	14397	14398	-	15045	-	-	+	2	2	4
14403	14399	14404	+	15041	-	-	+	1	3	4
14408	14405	14410	-	14496	14493	14496	-	2	2	4
14411	14408	14411	+	15017	15017	15018	+	2	2	4
14416	14414	14419	-	14568	14568	14571	-	1	3	4
14442	14437	14444	-	14481	-	-	-	2	2	4
14471	14471	14475	-	14558	14554	14558	-	2	2	4
14475	14472	14478	+	14419	-	-	-	2	2	4
14543	14539	14544	+	14447	-	-	-	3	1	4
14551	14551	14552	+	14504	-	-	-	2	2	4
14577	14577	14579	-	14956	14954	14956	+	2	2	4
14578	-	-	+	14563	-	-	-	2	2	4
14589	14589	14590	-	14775	-	-	-	2	2	4
14651	14651	14653	+	14634	-	-	-	2	2	4
14651	14651	14653	+	14676	14676	14679	-	2	2	4
14693	14690	14693	-	14748	-	-	+	2	2	4
14771	14767	14771	+	14855	-	-	-	2	2	4
14818	-	-	-	14864	-	-	+	2	2	4
14824	14824	14828	+	14857	14857	14858	-	2	2	4
14856	14856	14858	-	14976	14976	14978	-	4	0	4
14861	14859	14862	-	14981	14981	14982	-	0	4	4
14864	14860	14867	+	14763	-	-	-	2	2	4
14895	14891	14895	-	15023	15019	15023	-	2	2	4
14902	14900	14905	-	15065	-	-	-	2	2	4
14918	14916	14919	+	14857	-	-	-	2	2	4
14926	-	-	-	14990	-	-	-	2	2	4
14941	14941	14942	+	14995	14995	14996	-	2	2	4
14973	14972	14975	-	15155	-	-	+	2	2	4
14980	14977	14980	-	15083	-	-	-	2	2	4
15025	15025	15030	+	15138	-	-	-	2	2	4
15072	15067	15073	-	15129	15125	15129	-	2	2	4
15084	15082	15088	-	15143	-	-	-	2	2	4
15095	15091	15099	-	15159	15155	15159	-	2	2	4
15115	15112	15117	-	15242	15240	15242	-	2	2	4
15116	15113	15120	+	15146	15143	15146	-	2	2	4
15142	15141	15145	-	15280	15279	15280	-	2	2	4
15146	15143	15146	+	15113	15113	15116	-	2	2	4
15152	-	-	+	15109	-	-	-	2	2	4
15162	15158	15162	-	15238	15234	15238	-	2	2	4
15207	15203	15207	+	15112	-	-	-	2	2	4
15208	-	-	-	15257	-	-	-	2	2	4
15241	15239	15244	+	15252	-	-	-	2	2	4
15256	-	-	+	15193	-	-	-	2	2	4
98	96	103	-	119	119	122	+	1	2	3
173	169	173	+	93	-	-	+	3	0	3
206	203	210	+	105	-	-	-	0	3	3
216	212	219	+	98	-	-	-	3	0	3
242	240	245	-	417	415	417	-	2	1	3
263	259	267	-	15123	-	-	+	0	3	3
280	274	282	-	343	-	-	-	3	0	3
343	343	344	+	308	-	-	-	2	1	3
370	366	376	-	460	-	-	-	1	2	3
422	418	423	-	551	-	-	-	2	1	3
422	418	423	-	12314	12312	12314	+	0	3	3
522	518	522	-	618	-	-	-	2	1	3
761	761	762	+	725	-	-	-	0	3	3
832	827	836	-	9494	-	-	-	0	3	3
857	854	860	-	958	-	-	-	2	1	3
860	858	861	+	9157	9157	9159	+	2	1	3
901	896	905	-	1003	999	1003	+	2	1	3
948	945	951	-	11112	-	-	+	0	3	3
1003	999	1007	+	900	900	904	-	2	1	3
1009	1009	1011	+	1021	1020	1021	-	0	3	3
1031	1027	1038	-	3227	3227	3231	-	0	3	3
1040	1039	1044	-	1136	-	-	-	2	1	3
1121	1117	1124	+	1129	-	-	-	1	2	3
1232	1228	1233	+	1236	1236	1240	+	1	2	3
1270	1266	1270	-	1283	1281	1283	+	2	1	3
1270	1266	1270	-	1377	-	-	-	1	2	3
1398	1394	1402	-	1459	1459	1461	+	2	1	3
1442	1441	1444	+	1460	-	-	-	1	2	3

1452	1448	1455	-	2238	-	-	-	3	0	3
1459	1457	1461	+	1442	-	-	-	1	2	3
1495	1492	1498	+	1442	1442	1444	-	2	1	3
1513	1510	1517	-	1667	1664	1667	-	2	1	3
1586	1585	1589	-	8146	8146	8150	-	0	3	3
1625	1622	1626	-	1708	1708	1709	-	1	2	3
1632	1631	1632	-	1705	-	-	+	3	0	3
1815	1812	1820	-	10779	10776	10779	-	1	2	3
1821	1819	1824	+	2329	2329	2330	+	1	2	3
1905	1901	1906	+	1901	-	-	+	1	2	3
1962	1962	1963	-	2145	-	-	+	3	0	3
1969	1965	1973	-	2140	-	-	+	0	3	3
1999	1995	2001	+	12470	12469	12470	+	2	1	3
2038	2036	2044	+	12519	12517	12521	+	3	0	3
2049	2045	2049	+	12524	12524	12527	+	3	0	3
2050	2046	2052	-	12529	-	-	-	1	2	3
2255	2252	2257	-	14656	14654	14656	-	3	0	3
2278	2276	2281	-	2312	-	-	-	3	0	3
2284	-	-	-	2316	-	-	-	0	3	3
2384	2376	2389	+	9004	9004	9006	+	1	2	3
2391	2388	2394	-	11272	-	-	+	3	0	3
2411	2409	2414	+	2491	-	-	-	2	1	3
2466	-	-	-	2443	-	-	+	3	0	3
2503	-	-	-	2590	2589	2590	-	1	2	3
2518	2514	2521	-	2519	2519	2521	+	2	1	3
2555	2553	2557	+	12064	-	-	+	0	3	3
2640	2639	2644	-	2732	2731	2732	-	2	1	3
2675	2672	2679	+	8429	-	-	-	0	3	3
2709	2707	2713	+	2645	-	-	+	2	1	3
2875	2875	2880	+	6214	6210	6214	-	0	3	3
2951	2951	2956	-	3053	-	-	-	2	1	3
3002	2998	3005	-	3064	-	-	-	3	0	3
3010	3007	3015	-	3069	-	-	-	0	3	3
3023	3023	3025	-	3120	-	-	-	0	3	3
3136	3135	3136	+	3115	3115	3116	-	1	2	3
3228	3224	3234	-	6536	6534	6536	-	2	1	3
3231	3225	3236	+	5298	-	-	+	3	0	3
3231	3225	3236	+	11271	-	-	-	3	0	3
3231	3225	3236	+	12711	12711	12713	-	3	0	3
3231	3225	3236	+	12829	-	-	+	3	0	3
3256	3252	3258	+	14668	-	-	-	2	1	3
3306	3302	3312	-	10875	10875	10878	+	3	0	3
3370	3367	3375	-	3453	-	-	-	1	2	3
3517	3515	3521	+	3448	-	-	+	2	1	3
3664	3660	3665	+	3588	-	-	+	1	2	3
3753	3753	3755	+	3665	-	-	-	3	0	3
3758	3755	3762	-	3774	-	-	+	3	0	3
3812	3811	3812	-	4150	-	-	-	3	0	3
3818	3816	3818	-	4156	-	-	-	0	3	3
3945	3944	3950	-	4117	-	-	-	2	1	3
4052	4052	4056	-	12636	12634	12636	+	3	0	3
4076	4074	4080	-	4131	-	-	-	1	2	3
4076	4074	4080	-	12609	12609	12611	+	2	1	3
4092	4089	4092	-	4107	4104	4107	+	1	2	3
4324	4320	4330	+	12064	-	-	+	0	3	3
4609	4606	4612	-	4633	-	-	+	2	1	3
5039	5037	5042	-	5250	-	-	-	3	0	3
5066	5065	5069	+	6135	-	-	-	3	0	3
5100	5096	5104	+	5114	-	-	-	2	1	3
5107	5107	5112	+	12064	12063	12064	+	0	3	3
5144	5144	5148	-	12064	-	-	+	0	3	3
5254	5252	5257	+	5219	-	-	-	2	1	3
5254	5252	5257	+	5230	-	-	-	0	3	3
5259	5259	5260	+	5225	-	-	-	3	0	3
5377	5373	5381	-	5509	-	-	-	1	2	3
5476	5471	5480	-	12308	-	-	+	0	3	3
5503	5502	5509	+	10936	10932	10937	-	0	3	3
5667	5667	5671	-	5676	-	-	+	3	0	3
5691	5690	5694	-	5710	-	-	+	3	0	3
5710	5706	5712	-	5691	-	-	+	3	0	3
5780	-	-	-	5855	-	-	-	3	0	3
5786	5786	5790	-	5861	-	-	-	0	3	3
5856	5853	5857	-	6004	-	-	-	2	1	3
5884	5884	5886	+	5897	5896	5897	-	1	2	3
5892	5890	5895	-	6007	6007	6008	-	2	1	3
5959	5957	5960	-	6097	-	-	-	1	2	3
5959	5957	5960	-	6117	-	-	-	2	1	3
6082	6080	6086	-	10946	10944	10947	+	3	0	3
6096	6093	6100	+	10926	10926	10930	-	0	3	3
6175	6174	6180	-	11139	11139	11140	+	1	2	3
6197	6193	6198	+	11131	11131	11133	-	3	0	3
6214	6208	6220	+	8130	-	-	+	3	0	3
6214	6208	6220	+	8454	8454	8455	+	3	0	3
6214	6208	6220	+	9098	-	-	-	3	0	3
6214	6208	6220	+	10929	-	-	-	3	0	3
6214	6208	6220	+	11186	-	-	+	3	0	3
6214	6208	6220	+	12882	12880	12882	-	3	0	3
6214	6208	6220	+	13254	13254	13257	-	3	0	3
6333	6329	6336	+	6385	6384	6385	-	2	1	3
6363	6359	6367	+	12064	-	-	+	0	3	3
6538	6531	6541	+	12371	-	-	+	3	0	3
6561	6558	6561	+	11161	-	-	-	3	0	3
6570	6566	6574	-	8274	8273	8274	+	1	2	3
6634	6630	6637	-	7415	7415	7416	-	2	1	3
6715	6715	6716	+	6753	-	-	-	1	2	3
6778	6774	6778	+	6837	-	-	+	2	1	3
6838	6837	6839	+	7624	-	-	+	0	3	3
6845	6843	6848	+	7629	-	-	+	3	0	3
6857	6855	6857	-	9831	-	-	-	3	0	3
6929	6929	6935	+	6911	6911	6913	-	1	2	3
6986	6982	6989	+	10842	-	-	+	3	0	3

6993	6991	6995	+	10848	10848	10850	+	3	0	3
7017	7014	7019	-	7206 -	-		-	3	0	3
7130	7128	7133	-	7221 -	-		-	2	1	3
7285	7284	7290	-	7435	7432	7435	-	1	2	3
7386	7383	7390	-	7594 -	-		-	0	3	3
7611	7607	7611	+	9517 -	-		-	2	1	3
7633	7630	7637	-	15101 -	-		-	3	0	3
7711	7710	7715	-	12064 -	-		+	0	3	3
7726 -	-		-	9240 -	-		+	0	3	3
7742	7739	7745	-	7862 -	-		-	2	1	3
7809	7805	7809	+	11141 -	-		+	1	2	3
7817	7814	7822	+	11153	11153	11154	+	2	1	3
7866	7861	7866	-	13216	13212	13217	+	3	0	3
7966	7961	7970	-	12764 -	-		-	0	3	3
7972	7969	7975	+	8025	8022	8025	-	2	1	3
8007	8007	8011	+	8008	8007	8008	-	2	1	3
8094	8092	8099	+	8176 -	-		-	2	1	3
8193	8191	8194	+	14297	14295	14297	+	3	0	3
8244	8241	8247	-	8391	8391	8392	-	2	1	3
8267	8264	8269	-	8301	8301	8304	+	3	0	3
8318	8317	8320	-	15108	15106	15108	+	1	2	3
8466	8466	8469	-	8598 -	-		-	3	0	3
8471	8471	8473	-	8603 -	-		-	0	3	3
8573	8572	8574	-	8632	8632	8633	-	1	2	3
8624	8621	8627	-	13497 -	-		+	0	3	3
8644	8644	8645	-	8701 -	-		-	3	0	3
8647	8644	8651	+	8659	8655	8659	-	2	1	3
8731	8726	8733	-	8899	8899	8901	-	2	1	3
8765	8762	8769	+	8731	8731	8734	-	1	2	3
8796	8796	8798	-	8921	8921	8925	-	2	1	3
8954	8953	8956	-	9043 -	-		-	0	3	3
8976	8974	8978	-	12064 -	-		+	0	3	3
9070	9067	9073	-	14846	14844	14847	-	0	3	3
9146	9143	9146	-	9322	9320	9322	-	1	2	3
9152	9148	9155	-	9310	9310	9311	-	2	1	3
9158	9156	9161	-	9356	9355	9356	-	1	2	3
9164	9162	9166	-	9334	9334	9338	-	1	2	3
9195	9191	9198	+	10925 -	-		+	2	1	3
9209	9208	9211	-	9557 -	-		-	2	1	3
9244	9242	9247	+	9195	9195	9197	-	2	1	3
9266	9266	9267	-	9308 -	-		-	3	0	3
9331	9331	9335	+	10110 -	-		-	3	0	3
9428	9423	9430	-	9630 -	-		-	3	0	3
9459	9459	9462	-	9582	9580	9582	-	1	2	3
9474	9471	9478	+	12064 -	-		+	0	3	3
9550	9548	9554	+	9597	9593	9597	-	1	2	3
9566	9563	9570	+	12064 -	-		+	0	3	3
9926	9924	9928	-	12064 -	-		+	0	3	3
9946	9943	9949	+	14113 -	-		+	0	3	3
10049	10049	10051	-	10144	10143	10144	-	1	2	3
10052	10048	10055	+	10012	10012	10015	-	2	1	3
10077	10074	10082	-	10153 -	-		-	3	0	3
10081	10076	10083	+	10154	10154	10156	-	2	1	3
10085	10085	10088	-	10157 -	-		-	0	3	3
10112	10108	10115	+	10728	10728	10731	-	0	3	3
10112	10108	10115	+	15294	15294	15298	-	0	3	3
10184 -	-		+	12689 -	-		+	3	0	3
10211 -	-		+	11093 -	-		+	0	3	3
10237	10236	10237	-	12064 -	-		+	0	3	3
10319	10316	10319	-	10395 -	-		-	0	3	3
10410	10409	10414	-	13041	13040	13041	+	2	1	3
10426	10424	10426	+	10456 -	-		+	0	3	3
10688	10684	10692	+	14880	14878	14881	-	2	1	3
10699	10698	10704	-	14870 -	-		+	1	2	3
10704	10703	10704	+	12698 -	-		-	3	0	3
10710	10706	10715	+	12064 -	-		+	0	3	3
10727	10727	10729	+	14840	14840	14843	-	1	2	3
10744	10744	10746	-	10795 -	-		-	3	0	3
10755	10752	10758	-	10803 -	-		-	0	3	3
10763 -	-		-	12266 -	-		-	3	0	3
10782	10780	10782	-	12284 -	-		-	0	3	3
10786	10785	10787	+	12653	12649	12654	-	1	2	3
10841	10838	10844	-	10966 -	-		-	3	0	3
10850	10850	10852	-	10973 -	-		-	0	3	3
10926	10924	10930	+	12378	12375	12378	+	3	0	3
10945	10945	10950	-	14801	14801	14805	+	2	1	3
11079	11076	11079	-	11206 -	-		-	3	0	3
11122	11120	11126	+	13499	13499	13501	+	0	3	3
11132	11132	11136	-	12064 -	-		+	0	3	3
11152	11152	11156	-	11251	11251	11254	-	2	1	3
11238	11235	11238	-	11389	11389	11392	-	1	2	3
11270	11266	11274	+	11174 -	-		-	2	1	3
11276	11272	11279	-	11310	11310	11313	+	2	1	3
11489	11489	11491	+	11460	11460	11462	-	1	2	3
11511	11508	11511	-	11636	11633	11636	-	1	2	3
11600	11597	11600	-	11706 -	-		-	3	0	3
11624	11620	11627	-	12064 -	-		+	0	3	3
11788	11787	11790	-	11922 -	-		-	3	0	3
11793 -	-		-	11927 -	-		-	0	3	3
11800	11796	11803	-	11934 -	-		-	2	1	3
11921	11921	11922	+	11881	11880	11881	-	2	1	3
11964 -	-		-	12146 -	-		-	3	0	3
11970	11968	11970	-	12150 -	-		-	0	3	3
12028	12026	12028	+	12064 -	-		+	0	3	3
12064	12059	12066	-	12358 -	-		+	3	0	3
12064	12059	12066	-	12730	12730	12732	-	3	0	3
12064	12059	12066	-	12819	12817	12820	+	3	0	3
12064	12059	12066	-	13587	13587	13588	-	3	0	3
12064	12059	12066	-	14056	14053	14056	+	3	0	3
12064	12059	12066	-	14434	14434	14435	-	3	0	3
12064	12059	12066	-	14772 -	-		+	3	0	3

12064	12059	12066	-	15264	15264	15267	-	3	0	3
12076	12076	12079	-	12137	12134	12137	+	2	1	3
12092	12089	12092	+	12185	12182	12186	-	1	2	3
12103	12103	12105	+	12081	-	-	-	0	3	3
12113	12110	12117	+	12076	-	-	-	3	0	3
12159	12155	12162	-	12312	12311	12312	-	3	0	3
12164	12164	12167	-	12297	-	-	-	2	1	3
12232	12229	12232	-	12249	-	-	+	3	0	3
12249	12248	12249	-	12230	-	-	+	3	0	3
12282	12282	12285	+	12294	12294	12295	-	1	2	3
12294	12290	12298	-	12558	-	-	-	1	2	3
12460	12456	12463	-	12583	12583	12587	-	2	1	3
12493	12493	12495	+	12468	12467	12468	-	1	2	3
12672	12668	12674	+	13023	-	-	+	2	1	3
12806	12805	12806	+	12775	-	-	-	0	3	3
12811	12809	12811	+	12771	-	-	-	3	0	3
12854	12854	12858	+	12822	-	-	-	0	3	3
12861	12860	12861	+	12817	-	-	-	3	0	3
12994	12993	12996	-	13121	-	-	-	3	0	3
12999	12999	13003	-	13126	-	-	-	0	3	3
13022	13020	13025	-	12988	12988	12990	+	2	1	3
13039	13039	13043	+	13114	-	-	-	1	2	3
13172	13169	13176	-	13209	13207	13209	+	2	1	3
13212	-	-	-	13347	-	-	-	3	0	3
13218	13218	13220	-	13353	-	-	-	0	3	3
13303	13299	13306	+	13680	-	-	+	2	1	3
13448	13445	13448	-	14373	14373	14375	+	3	0	3
13499	13496	13502	-	13639	-	-	-	2	1	3
13823	13823	13824	+	13798	-	-	-	2	1	3
13843	13841	13843	-	14895	14893	14895	-	1	2	3
13924	13924	13929	-	13999	-	-	-	0	3	3
13924	13924	13929	-	14031	14031	14032	-	2	1	3
13948	13944	13953	-	14097	14097	14101	-	1	2	3
13997	13994	14002	-	14081	14079	14081	-	2	1	3
14098	14096	14101	-	14111	-	-	+	3	0	3
14111	-	-	-	14098	-	-	+	3	0	3
14137	14136	14137	-	14202	14202	14203	-	0	3	3
14156	14152	14157	-	14280	14279	14280	-	1	2	3
14171	14169	14171	+	14147	-	-	-	2	1	3
14210	14208	14210	+	14157	-	-	-	3	0	3
14353	14347	14357	-	14492	14488	14493	-	2	1	3
14503	14499	14507	-	14619	14616	14619	-	2	1	3
14531	14531	14536	-	14601	14600	14601	-	2	1	3
14847	14847	14850	+	15044	-	-	-	1	2	3
14855	14852	14859	+	15077	-	-	+	3	0	3
14902	14900	14905	-	14996	14992	14996	-	3	0	3
14913	14910	14914	-	15114	15110	15115	-	3	0	3
14950	-	-	+	14933	-	-	-	0	3	3
14955	14955	14957	+	14928	-	-	-	3	0	3
15019	15019	15022	-	15138	-	-	-	2	1	3
15051	15049	15054	-	15129	-	-	-	1	2	3
15053	15053	15056	+	14970	14970	14972	-	2	1	3
15072	15067	15073	-	15182	15181	15182	-	1	2	3
15074	15073	15074	+	15042	-	-	-	0	3	3
15079	15076	15079	+	15037	-	-	-	3	0	3
15095	15091	15095	+	15287	-	-	-	0	3	3
15104	15104	15108	+	15278	-	-	-	3	0	3
15197	-	-	-	15245	-	-	-	2	1	3
