# Supplementary material for: Identification and quantification of defective virus genomes in high throughput sequencing data using DVG-profiler, a novel post-sequence alignment processing algorithm
Source: PLoS One. 2019 May 17;14(5):e0216944. doi: 10.1371/journal.pone.0216944 (PMC6524942; doi:10.1371/journal.pone.0216944)
Supplement: S12 Table — (PDF) [file pone.0216944.s017.pdf]

Position (left)	Group start (left)	Group end (left)	Strandness (	Position (right)	Group start (right)	Group end (right)	Strandness (	Forward hits	Reverse hits	Fwd and reverse	Mumps Reads	Total Reads
5078	5076	5082	-	15083	15079	15086	+	126	131	257	2.66E+06	4.01E+06
3595	3591	3601	-	3626	3626	3627	+	11	183	194		
12145	12141	12149	-	14214	14213	14216	+	96	94	190		
13808	13808	13812	-	14885	14885	14887	+	96	90	186		
14391	14386	14395	-	15010	-	-	+	97	87	184		
3585	3581	3588	-	3636	3633	3637	+	173	1	174		
7992	7987	7996	-	12740	-	-	+	68	67	135		
14008	14008	14012	-	14187	-	-	+	57	55	112		
5065	5065	5070	-	15097	15093	15097	+	94	12	106		
1460	1459	1462	+	1398	1396	1398	-	43	42	85		
6322	6322	6324	-	6414	6414	6416	-	38	45	83		
6390	6385	6390	+	6338	6335	6341	-	44	39	83		
10472	10468	10476	-	10516	10516	10520	+	35	35	70		
14818	14816	14822	-	14864	14860	14865	+	35	32	67		
13861	13858	13864	-	13879	13876	13882	+	33	32	65		
1031	1030	1034	-	1067	1064	1067	+	33	28	61		
7926	7924	7929	-	7986	7986	7987	-	29	32	61		
1	2	8	-	126	122	127	-	30	30	60		
13114	13110	13115	+	13039	13038	13040	-	31	26	57		
7069	7066	7069	-	7126	7125	7126	-	54	2	56		
14687	14686	14691	-	15152	-	-	+	26	29	55		
7074	7071	7077	-	7131	-	-	-	0	53	53		
1179	1176	1183	-	1240	1236	1240	+	26	26	52		
2702	2699	2707	+	2675	2672	2679	-	25	18	43		
10498	10496	10498	-	10515	10515	10517	+	35	6	41		
13303	13301	13303	+	13261	13261	13263	-	20	19	39		
14130	14129	14134	-	14200	14196	14200	-	19	20	39		
4804	4804	4808	-	4844	4844	4848	-	19	18	37		
12641	12635	12645	-	12783	12781	12788	-	18	19	37		
15275	15272	15275	+	15241	15240	15244	-	18	19	37		
213	212	216	+	98	98	102	-	18	18	36		
1293	1292	1293	+	1177	-	-	-	17	17	34		
13039	13034	13040	-	13114	13114	13116	+	17	17	34		
13261	13260	13261	+	13303	13303	13304	-	17	17	34		
13870	13865	13873	-	14145	14143	14145	+	19	15	34		
2662	2661	2665	+	2719	2715	2720	-	16	17	33		
6170	6170	6171	-	6322	6322	6323	-	16	17	33		
14359	14355	14361	-	14503	14498	14506	-	6	27	33		
5861	5857	5862	-	6009	6009	6013	-	16	16	32		
12134	12130	12135	+	12079	12078	12080	-	18	14	32		
14220	14220	14221	-	14362	-	-	+	16	16	32		
14836	14836	14839	-	15057	15056	15057	+	18	14	32		
6338	6335	6338	+	6390	-	-	-	17	14	31		
8978	8974	8981	-	9034	9034	9035	+	26	5	31		
12168	12164	12168	-	12218	-	-	+	16	15	31		
14143	14142	14145	-	14410	14410	14412	+	15	16	31		
14864	14862	14866	+	14818	14817	14822	-	16	15	31		
3722	-	-	-	7316	-	-	+	16	14	30		
13024	13018	13028	-	13076	13073	13081	+	15	15	30		
665	661	666	+	634	633	638	-	17	12	29		
14398	14397	14402	-	15045	-	-	+	16	13	29		
206	203	207	-	283	283	287	+	14	14	28		
853	851	858	-	991	991	993	+	13	15	28		
960	956	964	-	1100	1100	1104	-	14	14	28		
6813	6809	6818	+	6850	-	-	-	14	14	28		
12304	12301	12304	-	12362	-	-	-	28	0	28		
12309	12306	12309	-	12367	-	-	-	0	28	28		
15251	15247	15251	+	15197	-	-	-	14	14	28		
5277	5273	5281	-	5330	5327	5330	-	14	13	27		
8076	8076	8077	-	8229	-	-	-	14	13	27		
10676	10672	10677	+	10662	10657	10662	-	13	14	27		
11444	11444	11445	-	11568	11567	11568	+	0	27	27		
748	748	750	+	708	706	708	-	13	13	26		
1407	1404	1412	-	1450	1447	1450	+	13	13	26		
6726	6722	6727	+	6964	6960	6965	+	17	9	26		
13894	13891	13894	-	13912	13912	13915	+	24	2	26		
14069	14063	14072	-	14088	14085	14088	+	15	11	26		
14416	14413	14419	-	14601	-	-	+	14	12	26		
395	389	399	-	344	341	347	+	14	11	25		
2265	2265	2266	+	2195	2194	2195	-	13	12	25		
3336	3333	3336	-	3353	3353	3355	+	13	12	25		
11269	11265	11274	-	11441	11439	11444	-	13	12	25		
123	119	123	-	150	150	154	+	23	1	24		
1854	1851	1856	-	2000	2000	2005	-	12	12	24		
2590	2585	2593	+	2494	2492	2498	-	12	12	24		
2715	2711	2719	-	2663	2662	2663	+	17	7	24		
11439	11436	11440	-	11573	-	-	+	24	0	24		
11976	11974	11976	-	14971	-	-	+	12	12	24		
12536	12535	12537	-	12684	12684	12685	-	5	19	24		
13285	13283	13287	-	13675	13674	13675	+	12	12	24		
14022	14018	14022	-	14202	14198	14206	-	5	19	24		
14353	14351	14353	-	14497	14497	14500	-	24	0	24		
14428	14428	14430	-	14464	14462	14464	+	23	1	24		
503	498	506	-	695	693	700	-	11	12	23		
1202	1199	1207	-	1381	1377	1385	-	12	11	23		
1494	1494	1497	+	1421	1419	1421	-	12	11	23		
4082	4081	4086	+	4102	4102	4103	+	12	11	23		
5009	5006	5013	-	5132	5132	5133	+	12	11	23		
6474	6473	6474	+	6401	-	-	-	10	13	23		
8935	-	-	+	8878	8877	8878	-	13	10	23		
9129	9126	9132	-	9163	9160	9163	+	13	10	23		
14435	14435	14440	-	14457	-	-	+	0	23	23		
924	920	928	+	893	892	897	-	10	12	22		
1240	1237	1240	+	1179	-	-	-	11	11	22		
6435	6434	6438	-	6477	6476	6479	-	11	11	22		
11269	11265	11274	-	11430	11430	11433	-	11	11	22		
12322	12322	12324	+	12282	12280	12282	-	7	15	22		

14069	14063	14072	-	14238		14238	14240	+	2	20	22
2675	2673	2678	+	2703		2700	2705	-	10	11	21
9030	9026	9034	-	8982		8978	8986	+	20	1	21
13039	13039	13041	+	13114		13112	13114	-	12	9	21
13600	13596	13605	-	13783		13781	13788	-	10	11	21
13912	13909	13914	-	13894	-	-		+	20	1	21
14062	14059	14062	-	14241	-	-		+	21	0	21
150	147	150	-	123		123	125	+	19	1	20
1359	1354	1359	-	1485		1485	1489	-	10	10	20
1383	1380	1385	-	1473		1473	1474	+	10	10	20
1817	1814	1819	-	2001		2000	2003	-	10	10	20
8264	8264	8268	-	8304		8300	8304	+	17	3	20
11244	11240	11247	-	13219	-	-		+	11	9	20
12047	12044	12051	-	12085	-	-		-	10	10	20
12082	12078	12086	+	12128		12125	12129	-	11	9	20
12280	12278	12282	-	12507		12505	12508	-	10	10	20
13629	13625	13632	-	13782		13779	13785	-	10	10	20
1488	1488	1491	+	1400		1396	1400	-	10	9	19
1513	1509	1517	-	1585		1583	1585	-	8	11	19
2012	2008	2016	-	2112		2112	2114	+	9	10	19
5763	5761	5765	+	5741		5741	5743	-	10	9	19
157	156	157	-	173		173	174	+	15	3	18
634	630	635	+	665		665	666	-	10	8	18
1230	1228	1232	-	5977	-	-		+	9	9	18
1407	1404	1412	-	1438		1435	1438	+	9	9	18
3291	3290	3296	-	3393		3393	3395	+	9	9	18
6947	6947	6951	-	7116		7116	7117	-	9	9	18
8022	8018	8025	+	7975		7972	7975	-	9	9	18
8459	8458	8461	+	8396		8394	8396	-	9	9	18
11726	11722	11730	+	11625		11622	11625	-	9	9	18
13385	13384	13387	-	14475	-	-		+	9	9	18
13948	13945	13952	-	14052		14049	14052	-	9	9	18
15365	15365	15369	+	15343		15340	15344	-	9	9	18
1364	1361	1365	-	1489		1489	1490	-	8	9	17
1381	1381	1383	+	1329		1327	1329	-	8	9	17
1755	1752	1757	-	1848		1848	1849	-	8	9	17
7528	7524	7531	+	7471	-	-		-	9	8	17
7882	7880	7882	-	7903	-	-		+	9	8	17
8162	8162	8166	-	8192		8188	8192	+	9	8	17
10004	10000	10007	-	9956	-	-		+	8	9	17
10496	10496	10498	+	10517		10515	10517	-	4	13	17
11501	11498	11504	-	11620		11617	11620	-	9	8	17
13327	13323	13328	+	13229		13229	13232	-	5	12	17
14776	14773	14781	-	14850	-	-		+	7	10	17
1	2	8	-	137		135	141	-	8	8	16
829	825	833	-	890	-	-		+	8	8	16
1031	1028	1034	+	1067		1067	1068	-	5	11	16
2028	2026	2032	+	12507		12505	12511	+	9	7	16
2055	2050	2057	+	2023	-	-		-	8	8	16
2443	2439	2445	-	2466		2466	2467	+	14	2	16
3957	3953	3960	+	3939		3938	3940	-	8	8	16
5291	5288	5293	-	5345		5345	5347	-	8	8	16
5892	5889	5895	-	5960		5960	5963	-	16	0	16
8963	8958	8969	-	9077		9077	9078	+	9	7	16
9905	9901	9908	-	11549	-	-		-	14	2	16
10120	10118	10121	-	10208	-	-		+	8	8	16
11102	11099	11105	-	11142	-	-		+	8	8	16
12209	12206	12209	+	12181		12181	12182	-	8	8	16
12291	12290	12294	-	12558	-	-		-	8	8	16
12641	12635	12645	-	12769		12768	12770	-	8	8	16
12837	12833	12841	-	12883		12882	12886	-	8	8	16
13508	13508	13509	-	13588		13588	13589	-	8	8	16
13587	13585	13589	-	13784		13782	13784	-	8	8	16
14442	14441	14445	-	14513	-	-		-	0	16	16
173	169	176	-	157	-	-		+	15	0	15
1267	1262	1270	-	1283		1281	1283	+	10	5	15
1939	1935	1943	-	2094		2089	2098	-	8	7	15
2174	2170	2176	-	2314	-	-		-	15	0	15
2181	2179	2181	-	2321	-	-		-	0	15	15
2203	2200	2206	+	2258		2257	2258	-	8	7	15
2662	2657	2664	-	2719		2715	2719	+	13	2	15
2757	2756	2758	-	2799	-	-		-	7	8	15
5094	5093	5096	+	5055		5055	5057	-	3	12	15
5898	5898	5902	-	5966		5966	5969	-	0	15	15
7263	7262	7263	+	7221	-	-		-	8	7	15
8150	8148	8152	+	8118		8115	8118	-	7	8	15
8396	8393	8401	+	8459		8458	8461	-	8	7	15
13184	13180	13187	-	13264	-	-		+	8	7	15
13996	13992	14000	-	14202		14197	14204	-	7	8	15
14016	14014	14016	-	14196	-	-		-	15	0	15
14243	14237	14246	+	14164		14164	14166	-	8	7	15
14435	14435	14440	-	14507	-	-		-	15	0	15
14770	14766	14771	-	14856		14855	14859	+	9	6	15
14904	14904	14908	-	14944		14940	14944	+	7	8	15
15117	15113	15121	+	15044		15043	15045	-	8	7	15
1	2	8	-	31		27	31	+	7	7	14
341	338	345	-	398		395	399	+	9	5	14
450	450	454	-	513	-	-		+	7	7	14
708	704	711	-	748		748	751	+	12	2	14
807	803	811	-	865		865	867	+	7	7	14
1299	1296	1303	-	1342		1342	1346	+	7	7	14
1407	1404	1412	+	1450		1447	1451	-	8	6	14
2157	2154	2157	+	2117		2117	2118	-	7	7	14
2288	2283	2292	-	2443		2439	2444	-	7	7	14
4971	4971	4974	-	5009		5006	5009	+	7	7	14
5488	5487	5490	-	5669		5665	5670	-	7	7	14
5701	5700	5705	-	5786		5786	5789	-	7	7	14
6686	6684	6687	+	11420	-	-		+	7	7	14

7549	7549	7552 -	7705 -	-	-	7	7	14
8244	8240	8245 -	8389	8386	8390 -	7	7	14
8347	8347	8351 -	8374	8370	8374 +	12	2	14
8396	8394	8399 -	8459	8459	8461 +	7	7	14
8778	8775	8781 -	8919	8917	8920 +	7	7	14
8963	8958	8969 -	9023	9021	9023 +	8	6	14
8963	8958	8969 -	9071	9071	9072 +	0	14	14
10146	10142	10149 -	12281 -	-	+	7	7	14
10374	10368	10374 -	10527	10527	10531 -	7	7	14
10694	10693	10695 -	14876 -	-	+	7	7	14
10699	10697	10701 -	10723	10723	10725 +	13	1	14
11032	11030	11036 -	14427	14427	14430 +	7	7	14
12168	12165	12169 +	12217	12217	12221 -	7	7	14
12529	12526	12530 -	12677 -	-	-	14	0	14
12558	12557	12562 -	12600	12596	12601 +	7	7	14
12707	12704	12711 -	12834	12830	12834 +	7	7	14
13157	13157	13161 -	13196 -	-	-	7	7	14
13327	13323	13328 +	13234	13233	13234 -	6	8	14
13772	13769	13776 +	13969 -	-	-	8	6	14
14897	14892	14900 -	14952	14952	14953 +	7	7	14
15002	14998	15003 -	15170	15167	15170 -	7	7	14
15144	15141	15146 -	15297	15294	15298 -	7	7	14
15191	15187	15195 -	15379	15377	15382 -	7	7	14
849	847	853 +	9147	9147	9151 +	9	4	13
913	908	916 -	999	995	999 -	10	3	13
1460	1459	1462 +	1442 -	-	-	7	6	13
1530	1525	1535 -	1684	1684	1689 -	6	7	13
1554	1550	1556 -	1579 -	-	-	7	6	13
1705	1705	1707 +	1632	1630	1632 -	11	2	13
2322	2320	2326 -	2369	2365	2371 +	7	6	13
2466	2464	2470 -	2443	2443	2445 +	12	1	13
2518	2514	2523 -	2547	2545	2547 -	5	8	13
3458	3458	3459 -	3524 -	-	+	8	5	13
3944	3943	3945 +	3953	3952	3953 -	7	6	13
3969	3968	3973 +	4094	4094	4097 -	6	7	13
4090	4089	4094 -	4119	4116	4119 +	7	6	13
4940	4936	4944 -	7750	7750	7754 +	7	6	13
7852 -	-	-	8060 -	-	-	6	7	13
8272	8270	8277 -	8296 -	-	+	0	13	13
8957 -	-	-	9077 -	-	+	13	0	13
9530	9527	9531 -	9738	9738	9742 +	6	7	13
9916	9912	9922 -	10068	10063	10069 -	6	7	13
10036	10033	10039 -	13350 -	-	-	6	7	13
12308	12308	12312 +	12345	12344	12345 -	6	7	13
13106	13102	13107 +	13056	13055	13056 -	7	6	13
13182	13178	13184 +	13127	13127	13130 -	6	7	13
14069	14063	14072 -	14199	14196	14199 -	1	12	13
369	366	374 -	461	457	461 +	6	6	12
380	379	384 -	450 -	-	+	6	6	12
913	908	917 +	904	900	904 -	5	7	12
1329	1326	1332 +	1381	1378	1382 -	6	6	12
1359	1354	1359 -	1429	1425	1429 +	6	6	12
1452	1447	1452 -	1504 -	-	-	6	6	12
1642	1640	1643 -	1672	1672	1673 -	1	11	12
2023	2022	2024 +	2055	2055	2056 -	6	6	12
2377	2373	2379 -	2733	2731	2734 -	6	6	12
2492	2487	2496 -	2531	2527	2531 -	5	7	12
2609	2605	2611 -	2752	2751	2753 -	6	6	12
2609	2605	2611 -	6644 -	-	+	6	6	12
4343	4340	4345 -	4489	4487	4490 -	6	6	12
4572	4570	4577 +	4824 -	-	-	6	6	12
4632	4629	4636 +	4788 -	-	-	6	6	12
4940	4936	4944 -	12865 -	-	-	3	9	12
5313	5309	5317 -	5486	5484	5488 -	5	7	12
5545	5543	5548 -	5556	5556	5558 +	1	11	12
5552	5550	5552 -	5641	5639	5641 -	10	2	12
6815	6815	6819 -	6834 -	-	+	12	0	12
6834 -	-	-	6815 -	-	+	12	0	12
7088	7081	7091 -	7236	7234	7241 -	6	6	12
7105	7105	7108 +	7079 -	-	-	6	6	12
7469	7465	7472 +	7452 -	-	-	6	6	12
7574	7570	7575 -	7635	7635	7636 -	6	6	12
9128	9128	9130 +	9200	9200	9201 -	6	6	12
9372	9368	9375 +	9335	9333	9340 -	6	6	12
9451	9446	9456 -	9518 -	-	+	6	6	12
9948	9943	9953 -	10016	10016	10019 +	7	5	12
10596	10594	10597 -	10664	10664	10666 +	6	6	12
11536	11536	11538 -	11578	11575	11578 +	6	6	12
12126	12121	12130 -	12239	12235	12242 -	6	6	12
12126	12121	12130 -	12298 -	-	+	6	6	12
12126	12121	12130 -	13012	13011	13012 +	7	5	12
12344	12344	12347 +	12312	12308	12312 -	6	6	12
12625	12621	12627 -	12782	12779	12784 -	6	6	12
12751	12747	12755 -	12926	12926	12929 -	7	5	12
13131	13126	13134 +	13182	13179	13183 -	6	6	12
13199	13195	13199 +	13504 -	-	-	6	6	12
13285	13283	13287 -	13478	13476	13481 -	6	6	12
13882	13878	13882 -	13924	13924	13928 +	6	6	12
14048	14046	14051 -	14204	14201	14205 -	6	6	12
14062	14059	14062 -	14195	14195	14199 -	11	1	12
14314	14311	14314 -	14317	14315	14317 +	6	6	12
14676	14675	14676 +	14653	14653	14655 -	6	6	12
14744	14741	14748 -	14918 -	-	-	6	6	12
15052	15048	15055 -	15195	15194	15198 -	6	6	12
15235	15233	15235 +	15204	15204	15206 -	6	6	12
15241	15239	15245 +	15275	15275	15276 -	6	6	12
257	257	260 +	230	228	231 -	6	5	11
791	786	794 -	851	849	854 +	6	5	11

1259	1254	1259 -	1274	1270	1276 +	8	3	11
1902	1900	1906 -	3314	3314	3315 +	4	7	11
2503	2498	2507 -	2525	2525	2527 +	10	1	11
2602	2601	2607 +	6650	6646	6651 -	4	7	11
7088	7081	7091 -	7263	7261	7269 -	6	5	11
7984	7981	7986 -	8012	8012	8014 +	5	6	11
8084	8083	8088 -	8243	8240	8243 -	6	5	11
8386	8382	8388 -	8471	8467	8471 +	7	4	11
8963	8958	8969 -	9046	9046	9048 +	9	2	11
9185	9181	9190 -	9226	9226	9229 +	4	7	11
9194	9192	9200 -	9360	9355	9363 -	5	6	11
9379	9374	9383 -	9401	9397	9401 +	4	7	11
9697	9695	9698 +	9658	9658	9660 -	6	5	11
10286	10285	10291 -	10427	10424	10429 -	5	6	11
10705	10705	10707 -	10717 -	-	+	0	11	11
12076	12071	12079 -	12137	12134	12137 +	7	4	11
13061	13059	13065 +	13101	13100	13101 -	6	5	11
14341	14338	14344 -	14411	14407	14411 +	7	4	11
14515	14512	14515 -	14540	14540	14542 +	5	6	11
242	240	245 -	386	386	388 -	5	5	10
274	269	278 -	356	356	357 +	5	5	10
515	510	519 -	671	668	671 -	5	5	10
655	652	660 -	11857	11856	11857 +	5	5	10
700	696	704 +	756 -	-	-	5	5	10
743	742	747 -	713 -	-	+	10	0	10
874	870	874 -	1018	1015	1021 -	5	5	10
893	892	897 +	924	921	924 -	5	5	10
1081	1077	1086 -	1214	1214	1215 -	5	5	10
1098	1092	1101 -	1308	1305	1308 -	5	5	10
1202	1199	1207 -	1333	1331	1334 -	5	5	10
1244	1240	1248 -	1264	1262	1264 +	6	4	10
1401	1398	1401 -	1663 -	-	+	5	5	10
1487	1484	1491 -	1687	1685	1690 -	5	5	10
1637	1633	1637 -	1667 -	-	-	10	0	10
1808	1805	1811 -	2001	1999	2004 -	5	5	10
1861	1860	1863 -	2000 -	-	-	5	5	10
2055	2050	2057 +	12531	12529	12531 +	9	1	10
2350	2348	2353 -	14752 -	-	-	5	5	10
2709	2708	2709 +	2672	2671	2672 -	2	8	10
3048	3046	3050 -	3088	3088	3092 +	4	6	10
3367	3363	3371 -	3412	3412	3415 +	5	5	10
3545	3541	3549 -	3599 -	-	+	6	4	10
3564	3560	3564 -	3653	3653	3656 +	5	5	10
3619	3617	3620 -	3743	3739	3743 -	5	5	10
3701	3698	3703 +	7337	7336	7339 -	3	7	10
3957	3952	3960 -	4002 -	-	+	5	5	10
4458	4458	4460 +	4403	4403	4404 -	5	5	10
5078	5077	5080 +	15083	15081	15084 -	4	6	10
5540	5539	5540 -	5561 -	-	+	10	0	10
5768	5767	5769 +	5805	5805	5806 +	5	5	10
6263	6262	6267 -	6310	6308	6310 +	5	5	10
6637	6637	6638 +	6699 -	-	-	5	5	10
6935	6934	6939 -	7131	7128	7131 -	5	5	10
6963	6959	6963 -	7131 -	-	-	5	5	10
7073 -	-	+	6957 -	-	-	5	5	10
7130	7129	7133 -	7229	7229	7230 -	5	5	10
7452	7448	7455 +	7469 -	-	-	5	5	10
7591	7587	7596 -	7603	7599	7603 +	6	4	10
7701	7698	7701 -	7860	7858	7863 -	5	5	10
7802	7800	7802 -	7836	7836	7838 +	5	5	10
7992	7988	7996 +	8148	8148	8150 -	4	6	10
8060	8058	8064 -	8203	8199	8203 -	5	5	10
8065	8062	8067 +	8148	8145	8148 -	5	5	10
9185	9181	9190 -	9340	9338	9341 -	5	5	10
9305	9304	9308 -	9500	9499	9503 -	5	5	10
9530	9526	9530 +	9738	9738	9742 -	6	4	10
9594	9589	9598 -	9805	9803	9805 -	5	5	10
9658	9657	9658 +	9697 -	-	-	5	5	10
9905	9901	9908 -	10069	10068	10070 -	5	5	10
10364	10361	10366 -	10467 -	-	+	5	5	10
11061	11060	11062 +	11005	11004	11005 -	5	5	10
11363	11363	11366 -	11434 -	-	-	5	5	10
11622	11618	11624 -	11792	11790	11794 -	5	5	10
11755	11753	11758 +	11721	11718	11721 -	5	5	10
12222	12219	12225 -	12371	12369	12373 -	5	5	10
12486	12485	12491 +	12466	12466	12467 -	5	5	10
12547	12545	12547 +	12603	12603	12605 -	5	5	10
12576	12575	12576 +	12467 -	-	-	5	5	10
12625	12621	12627 -	13760 -	-	-	5	5	10
12751	12747	12755 -	12932	12930	12934 -	5	5	10
12891	12889	12894 -	13057	13055	13061 -	5	5	10
13049	13045	13052 -	13114	13114	13115 +	5	5	10
13389	13389	13392 +	13410	13410	13411 -	5	5	10
13629	13625	13632 -	13658	13658	13662 +	7	3	10
13818	13814	13820 -	13976	13972	13976 -	5	5	10
13911	13910	13913 +	13867 -	-	-	7	3	10
14048	14046	14051 -	14195	14195	14198 -	5	5	10
14619	14617	14620 -	14769 -	-	+	5	5	10
14806	14802	14807 -	14881	14881	14883 +	5	5	10
14866	14863	14870 -	14959	14959	14960 +	5	5	10
14885	14879	14888 -	15085	15083	15085 -	5	5	10
14892	14892	14895 +	14775 -	-	-	5	5	10
15144	15141	15146 -	15303	15303	15305 -	5	5	10
15195	15193	15195 +	15219 -	-	-	4	6	10
356	355	360 -	555	555	559 -	5	4	9
395	389	399 -	418	416	418 +	4	5	9
530	526	530 -	698	695	698 -	4	5	9
606	602	607 -	691	691	695 -	8	1	9

611	608	615	-	696		696	698	-	1	8	9
685	685	688	+	774	-	-	-		5	4	9
1021	1019	1024	+	957		956	957	-	8	1	9
1131	1127	1132	+	1116		1114	1118	-	5	4	9
1375	1373	1379	-	1500		1500	1501	-	5	4	9
1442	1442	1446	-	1460		1459	1460	+	7	2	9
1530	1525	1535	-	1694		1691	1695	-	4	5	9
1554	1550	1556	-	1687		1685	1688	-	5	4	9
1586	1585	1590	+	8146	-	-		+	9	0	9
1670	1667	1672	-	1754		1753	1754	+	5	4	9
1910	1910	1911	-	1973		1973	1974	+	5	4	9
2023	2022	2025	-	2055		2055	2056	+	7	2	9
2101	2099	2103	-	2137		2135	2137	+	6	3	9
2177	2173	2177	+	2099	-	-	-		5	4	9
2377	2373	2379	-	2524		2523	2525	-	4	5	9
2460	2457	2462	-	2480		2478	2480	+	5	4	9
3448	3448	3450	-	3493	-	-		+	9	0	9
4592	4589	4592	-	4785		4783	4788	-	5	4	9
4632	4629	4636	+	4777	-	-	-		5	4	9
4877	4874	4881	+	4859		4858	4859	-	5	4	9
5046	5043	5047	-	5205	-	-	-		9	0	9
5053	5049	5055	-	5212	-	-	-		0	9	9
5100	5100	5102	+	5049	-	-	-		9	0	9
5313	5309	5317	-	5493		5491	5496	-	4	5	9
5476	5471	5479	-	6935	-	-		+	0	9	9
5976	5976	5979	-	12329		12327	12329	+	2	7	9
6077	6073	6078	+	6048		6047	6052	-	5	4	9
6118	6118	6123	-	6144	-	-		+	5	4	9
7088	7081	7091	-	7275		7273	7276	-	5	4	9
7221	7219	7221	-	7355	-	-	-		4	5	9
7221	7220	7223	+	7263	-	-	-		4	5	9
7279	7276	7279	-	7302		7302	7304	+	9	0	9
7286	7284	7287	+	7257		7257	7258	-	5	4	9
7774	7772	7777	+	7753		7753	7755	-	5	4	9
8147	8143	8150	-	8243	-	-	-		1	8	9
8776	8774	8778	+	8697		8695	8697	-	5	4	9
9994	9993	9996	+	9970		9969	9970	-	5	4	9
10657	10653	10660	+	10677		10676	10677	-	3	6	9
11881	11877	11886	-	12077		12074	12078	-	5	4	9
12159	12158	12160	-	12227		12226	12228	+	4	5	9
12589	12588	12594	-	12625		12624	12625	-	0	9	9
12891	12889	12894	-	13077		13075	13077	-	5	4	9
13965	13962	13965	+	13919	-	-	-		0	9	9
13972	13968	13976	+	13916	-	-	-		9	0	9
14465	14464	14465	+	14428		14427	14428	-	7	2	9
14770	14766	14771	-	14921		14920	14925	-	5	4	9
15206	15204	15209	-	15384		15379	15384	-	5	4	9
14	9	18	-	5474	-	-	-		8	0	8
67	62	70	-	110		109	110	+	4	4	8
150	148	154	+	123		119	123	-	4	4	8
260	260	262	-	369	-	-		+	4	4	8
325	322	325	-	14557	-	-		+	0	8	8
412	410	417	-	520		520	521	+	4	4	8
474	471	476	-	637		637	638	-	4	4	8
699	699	702	-	757		756	757	+	5	3	8
874	870	874	-	945	-	-		+	4	4	8
959	957	961	+	928	-	-	-		4	4	8
974	970	978	-	1172		1168	1172	-	4	4	8
987	984	992	-	1024		1021	1024	+	4	4	8
1098	1092	1101	-	1247		1247	1251	-	4	4	8
1159	1156	1162	-	1288		1285	1290	-	4	4	8
1179	1176	1183	-	1293	-	-		+	4	4	8
1306	1303	1310	+	1145	-	-	-		4	4	8
1307	1305	1307	-	1345	-	-		+	3	5	8
1364	1361	1365	-	1535	-	-	-		4	4	8
1400	1398	1403	+	1488	-	-	-		4	4	8
1407	1404	1412	-	1659		1658	1659	+	4	4	8
1487	1484	1491	-	1565		1564	1565	-	4	4	8
1568	1564	1571	-	1739		1739	1743	-	4	4	8
1632	1631	1632	+	1705	-	-	-		0	8	8
1641	1637	1642	+	1696	-	-	-		8	0	8
1696	1696	1697	+	1641	-	-	-		0	8	8
1802	1799	1803	-	2000		2000	2002	-	4	4	8
1808	1805	1811	-	1939		1937	1941	-	4	4	8
1817	1814	1819	-	1939		1936	1940	-	4	4	8
1978	1975	1981	-	2091		2091	2093	-	4	4	8
2036	2035	2039	-	2130	-	-		+	4	4	8
2091	2090	2093	-	2128		2125	2128	+	3	5	8
2091	2090	2093	-	2197	-	-	-		4	4	8
2174	2170	2176	-	2205	-	-	-		4	4	8
2261	2257	2265	-	2440		2440	2443	-	4	4	8
2444	2443	2447	+	2486		2483	2486	+	4	4	8
2502	2499	2507	+	2412		2412	2415	-	4	4	8
2559	2559	2563	+	2482		2480	2483	-	4	4	8
2809	2807	2809	-	2832	-	-		+	5	3	8
2866	2864	2866	-	2877	-	-		+	8	0	8
2930 -	-	-		3128		3125	3128	-	4	4	8
3007	3004	3010	-	3129		3129	3131	-	4	4	8
3072	3070	3072	+	3060		3060	3062	-	1	7	8
3115	3115	3119	-	3136		3132	3136	+	5	3	8
3301	3298	3305	-	3408		3404	3410	+	5	3	8
3360	3360	3365	+	3329	-	-	-		4	4	8
3403	3399	3406	-	3573		3572	3573	-	4	4	8
3403	3399	3406	-	3665	-	-		+	4	4	8
3411	3407	3414	-	3598		3598	3601	+	4	4	8
3441	3438	3444	-	3572	-	-	-		4	4	8
3453 -	-	-		3488	-	-		+	0	8	8
3661	3656	3662	-	3810		3809	3812	-	4	4	8

3810	3807	3810 +	3998 -	-	-	4	4	8
3935	3931	3939 -	4267	4266	4267 -	4	4	8
4013	4013	4016 -	4043	4041	4044 +	4	4	8
4222	4218	4225 -	4345	4345	4347 -	4	4	8
4236	4232	4238 -	4412	4412	4416 -	4	4	8
4241	4240	4244 -	4373	4373	4374 -	4	4	8
4566	4564	4571 -	4644 -	-	+	4	4	8
4619	4617	4619 -	4661	4659	4661 +	4	4	8
4877	4875	4881 -	4938	4934	4938 +	8	0	8
4927	4924	4930 +	4957	4957	4958 +	4	4	8
5253	5253	5257 +	5230	5230	5231 -	1	7	8
5277	5273	5281 -	5324 -	-	-	4	4	8
5297	5295	5300 +	5270 -	-	-	4	4	8
5313	5309	5317 -	5532	5530	5536 -	4	4	8
5443	5443	5447 -	5471	5471	5472 +	4	4	8
5467	5462	5470 -	5668	5667	5670 -	4	4	8
5558	5554	5560 -	5647 -	-	-	0	8	8
5558	5554	5560 -	5667	5666	5670 -	4	4	8
5875	5871	5875 +	5850 -	-	-	4	4	8
5953	5950	5957 -	6124	6124	6127 -	4	4	8
6048	6047	6051 +	6077	6077	6078 -	4	4	8
6390	6385	6390 +	6494 -	-	-	4	4	8
6473	6469	6473 -	6519	6519	6523 +	7	1	8
6543	6541	6543 -	6598 -	-	+	2	6	8
6580	6577	6580 -	6605 -	-	+	4	4	8
7088	7081	7091 -	7255	7252	7258 -	5	3	8
7106	7103	7110 -	7233	7232	7233 -	4	4	8
7436	7432	7440 +	7415 -	-	-	4	4	8
7538	7533	7541 -	7713	7713	7716 -	4	4	8
7550	7548	7550 +	7704	7704	7705 -	4	4	8
7896	7892	7898 -	7927	7924	7927 +	4	4	8
8176 -	-	+	8093 -	-	-	4	4	8
8318	8314	8320 -	8376	8374	8376 +	5	3	8
8690	8687	8695 -	8858	8858	8859 -	4	4	8
9008	9003	9009 -	9129	9125	9129 -	4	4	8
9273	9269	9275 -	9300 -	-	+	7	1	8
9291	9287	9292 -	9434	9433	9437 -	4	4	8
9297	9293	9300 +	9224 -	-	-	5	3	8
9379	9374	9383 -	9502	9499	9502 -	4	4	8
9379	9374	9383 -	9563	9563	9564 -	4	4	8
9594	9589	9598 -	9783	9779	9784 -	4	4	8
9597	9593	9599 +	9524	9524	9525 -	4	4	8
9698	9696	9701 -	9919 -	-	+	4	4	8
9710	9710	9712 -	9906 -	-	+	4	4	8
9747	9743	9749 -	9920	9919	9923 -	4	4	8
9759	9756	9762 -	9935 -	-	-	4	4	8
9899	9899	9902 +	9974 -	-	-	4	4	8
9964	9959	9967 +	9999	9999	10000 -	4	4	8
9976	9974	9979 +	9987	9987	9988 -	4	4	8
10004	10000	10007 -	10042	10042	10045 +	6	2	8
10183	10178	10185 -	10335	10334	10336 -	4	4	8
10255 -	-	-	10324 -	-	-	4	4	8
10302	10299	10305 -	10459	10459	10460 -	4	4	8
10425	10422	10426 -	10528 -	-	-	4	4	8
10485	10483	10487 -	10543	10539	10543 +	4	4	8
10515 -	-	+	10498 -	-	-	8	0	8
10617	10615	10620 +	10572	10569	10572 -	4	4	8
10803 -	-	+	12680 -	-	-	4	4	8
11060	11057	11061 -	11781 -	-	-	4	4	8
11099	11096	11102 +	11055	11055	11059 -	4	4	8
11200	11197	11203 -	11334	11331	11334 +	4	4	8
11233	11230	11233 -	11399	11397	11401 -	4	4	8
11238	11235	11238 -	11284	11283	11286 -	4	4	8
11269	11265	11274 -	11318	11314	11322 +	4	4	8
11381	11381	11383 +	11323 -	-	-	4	4	8
11421 -	-	+	13883 -	-	+	8	0	8
11478	11475	11484 -	11579	11576	11583 +	4	4	8
11526	11523	11529 -	11667	11666	11669 -	4	4	8
11560	11556	11563 -	11592	11588	11595 +	4	4	8
11827	11826	11827 -	11856	11856	11857 +	5	3	8
12029	12027	12031 -	12123 -	-	+	4	4	8
12029	12027	12031 -	12194	12194	12197 -	4	4	8
12029	12027	12031 -	12203 -	-	-	4	4	8
12053	12053	12057 -	12103 -	-	+	8	0	8
12059	12059	12064 -	12097 -	-	+	0	8	8
12082	12078	12086 +	11982	11982	11983 -	4	4	8
12089	12088	12092 +	11983 -	-	-	4	4	8
12203	12200	12203 +	12188 -	-	-	4	4	8
12225	12222	12228 +	12161	12158	12161 -	5	3	8
12376	12375	12377 -	12609 -	-	-	4	4	8
12584	12584	12585 -	12620 -	-	-	8	0	8
12910	12907	12913 -	13078	13075	13078 -	4	4	8
13024	13018	13028 -	13162	13162	13163 -	4	4	8
13104	13101	13106 -	14090 -	-	+	4	4	8
13184	13180	13187 -	13363	13363	13366 -	4	4	8
13199	13195	13199 +	13057	13055	13057 -	4	4	8
13334	13332	13335 +	13228 -	-	-	8	0	8
13538	13534	13542 -	13713	13712	13713 -	4	4	8
13562	13559	13565 -	13665 -	-	-	4	4	8
13651	13649	13651 +	14029	14028	14029 +	1	7	8
13839	13837	13840 -	13977	13974	13977 -	4	4	8
13972	13968	13976 +	13894 -	-	+	4	4	8
14107	14105	14110 +	14132	14129	14132 -	4	4	8
14107	14105	14110 +	14171 -	-	-	4	4	8
14132	14129	14134 +	14110	14107	14110 -	4	4	8
14251	14248	14255 +	14438 -	-	-	4	4	8
14272	14272	14277 -	14348	14344	14348 +	4	4	8
14324	14322	14326 -	14515 -	-	-	5	3	8

14435	14435	14440 -	14536 -	-	+	4	4	8
14542	14539	14547 +	14448	14447	14448 -	5	3	8
14554	14551	14558 -	14771 -	-	-	4	4	8
14603	14598	14607 -	14624	14621	14624 +	4	4	8
14603	14598	14607 -	14789	14789	14793 -	4	4	8
14638	14634	14640 +	14685	14683	14687 -	3	5	8
14818	14816	14824 +	14763	14763	14766 -	4	4	8
14866	14863	14870 -	14894	14894	14896 +	2	6	8
14912	14910	14915 -	14954	14954	14958 +	4	4	8
14933	14933	14935 -	15078	15078	15080 -	4	4	8
14979	14978	14983 -	15114	15114	15115 -	4	4	8
15052	15048	15055 -	15212	15209	15213 -	4	4	8
15060	15060	15062 -	15094	15092	15094 +	5	3	8
15114	15112	15117 -	15242	15238	15244 -	4	4	8
15114	15112	15117 -	15273	15272	15273 +	4	4	8
15144	15141	15146 -	15256	15256	15258 -	4	4	8
15144	15141	15146 -	15321	15321	15323 -	4	4	8
15219 -	-	+	15195 -	-	-	4	4	8
15256	15255	15258 +	15193 -	-	-	4	4	8
15256	15255	15258 +	15242	15242	15245 -	4	4	8
232	231	232 -	243 -	-	+	7	0	7
635	633	637 -	11876 -	-	+	3	4	7
756	755	756 +	700	700	701 -	3	4	7
838	838	842 +	9136 -	-	+	4	3	7
918	918	919 -	1004 -	-	-	0	7	7
959	957	961 +	1097 -	-	-	4	3	7
974	970	978 -	1117	1117	1121 -	3	4	7
1016 -	-	+	961 -	-	-	0	7	7
1145	1141	1149 -	5895	5895	5899 -	2	5	7
1158	1155	1158 +	1132 -	-	-	4	3	7
1167	1163	1171 -	1384	1381	1384 -	3	4	7
1187	1186	1188 -	1381	1380	1382 -	4	3	7
1244	1240	1248 -	10223 -	-	-	0	7	7
1421	1417	1425 +	1494 -	-	-	4	3	7
1506	1503	1506 -	1551 -	-	-	7	0	7
1513	1509	1517 -	1558 -	-	-	0	7	7
1513	1509	1517 -	1637	1636	1637 -	4	3	7
2052	2050	2055 -	12529	12529	12532 -	2	5	7
2099	2099	2102 +	2177 -	-	-	3	4	7
2460	2457	2462 -	2560	2557	2560 -	3	4	7
2492	2487	2496 -	12082	12080	12086 -	7	0	7
2525	2524	2529 -	2503 -	-	+	7	0	7
2525	2524	2529 -	12082	12080	12085 -	7	0	7
2548	2543	2552 -	12082	12080	12082 -	7	0	7
2564	2560	2568 -	2670	2670	2672 -	3	4	7
2581	2581	2582 -	2605	2603	2605 +	4	3	7
2609	2605	2611 -	2634	2630	2634 +	5	2	7
2715	2715	2719 +	2663	2661	2663 -	4	3	7
2942	2941	2947 -	2962 -	-	+	7	0	7
2942	2941	2947 -	3057 -	-	+	4	3	7
2951	2951	2952 +	2982 -	-	+	0	7	7
3196	3194	3200 -	3343	3339	3344 -	3	4	7
4058	4055	4063 -	4125 -	-	+	4	3	7
4169	4167	4171 -	4226	4225	4226 +	3	4	7
4274 -	-	+	4286	4285	4286 -	4	3	7
4284	4281	4286 -	4409	4409	4412 -	6	1	7
4582	4578	4586 -	4618 -	-	+	3	4	7
4940	4936	4944 -	4879 -	-	+	7	0	7
5159	5159	5162 +	6708 -	-	-	4	3	7
5170	5167	5171 -	5315	5315	5319 -	4	3	7
5212 -	-	+	15100 -	-	-	3	4	7
5253	5253	5257 +	5218 -	-	-	4	3	7
5259	5259	5263 +	5225 -	-	-	7	0	7
5277	5273	5281 -	5423 -	-	-	4	3	7
5594 -	-	-	5612 -	-	+	6	1	7
5862	5862	5863 +	5864	5863	5864 -	4	3	7
6029	6029	6033 -	6181	6181	6185 -	4	3	7
6518	6513	6520 -	6693 -	-	-	6	1	7
6930	6928	6931 +	6817	6817	6819 -	4	3	7
7285	7281	7289 -	7435	7432	7436 -	3	4	7
7884	7884	7886 +	7796	7795	7796 -	4	3	7
7896	7892	7898 -	7989 -	-	+	3	4	7
7926	7924	7929 -	8022	8022	8023 +	3	4	7
8374	8374	8375 -	8347 -	-	+	7	0	7
8404	8403	8405 +	8429	8427	8429 -	2	5	7
8559	8557	8563 -	8566	8566	8571 +	5	2	7
8562	8558	8565 +	8660 -	-	-	3	4	7
8884	8879	8885 +	9043	9043	9048 -	3	4	7
8950	8947	8951 +	8861	8860	8864 -	4	3	7
8971 -	-	-	9068 -	-	+	3	4	7
9010	9006	9011 +	8980	8979	8984 -	4	3	7
9046	9046	9049 -	8968 -	-	+	7	0	7
9185	9181	9190 -	9215	9214	9216 +	4	3	7
9194	9192	9200 -	9419	9419	9423 -	4	3	7
9300	9296	9302 -	9273 -	-	+	7	0	7
9574	9573	9577 +	9616 -	-	-	0	7	7
9579	9579	9584 +	9611 -	-	-	7	0	7
10743	10740	10746 -	10803 -	-	+	4	3	7
10797	10797	10798 -	10906 -	-	-	3	4	7
10958	10957	10961 -	11150	11150	11151 -	3	4	7
11005	11004	11009 -	11061 -	-	+	7	0	7
11060	11057	11061 -	11005 -	-	+	7	0	7
11439	11436	11440 -	11567 -	-	-	7	0	7
11526	11523	11529 -	11652	11652	11656 -	4	3	7
11840	11838	11843 -	11915	11915	11916 -	3	4	7
11921	11921	11922 +	11881	11880	11881 -	4	3	7
11942	11938	11946 -	11974	11972	11976 +	5	2	7
11988	11982	11990 -	12033 -	-	+	4	3	7

12126	12121	12130 -	12210	12210	12214 -	4	3	7
12191	12187	12195 -	12370 -	-	-	3	4	7
12327	12326	12331 +	12277	12274	12278 -	7	0	7
12988	12986	12989 -	13022	13022	13023 +	6	1	7
13095	13091	13095 -	13160 -	-	+	4	3	7
13261 -	-	-	13303 -	-	+	5	2	7
13532 -	-	-	13679 -	-	-	7	0	7
13538	13534	13542 -	13685 -	-	-	0	7	7
13635	13634	13638 -	13798	13795	13800 -	4	3	7
13856	13853	13856 -	13878	13878	13881 +	3	4	7
13870	13865	13873 -	13911 -	-	+	4	3	7
13938	13935	13940 -	14042	14042	14044 -	4	3	7
13961	13958	13964 -	14063	14063	14066 -	3	4	7
13972	13968	13976 +	13936 -	-	-	4	3	7
14074	14074	14079 -	14083 -	-	+	2	5	7
14157	14155	14160 -	14347	14346	14349 -	4	3	7
14642	14638	14643 -	14687	14684	14687 +	4	3	7
14818	14816	14824 +	14864	14862	14864 -	4	3	7
14912	14910	14915 -	14945 -	-	+	0	7	7
1	2	8 -	153	151	153 -	3	3	6
67	62	70 -	216	216	218 -	3	3	6
106	102	106 -	187 -	-	+	3	3	6
139	135	142 -	191	188	191 +	3	3	6
224	223	229 -	429 -	-	-	3	3	6
274	269	278 -	364 -	-	+	3	3	6
274	269	278 -	458	458	459 +	3	3	6
320	316	325 +	550	550	552 -	3	3	6
343	343	346 +	308 -	-	-	3	3	6
385	380	389 +	469 -	-	-	3	3	6
515	510	519 -	698	694	699 -	3	3	6
606	602	607 -	750	748	750 -	3	3	6
634	630	635 +	731	728	732 -	3	3	6
685	682	686 -	774 -	-	+	3	3	6
717	714	721 +	659	658	659 -	3	3	6
752	749	756 -	910	908	911 -	3	3	6
807	803	811 -	889	889	891 -	3	3	6
807	803	811 -	1006	1006	1008 -	3	3	6
849	847	853 +	940 -	-	-	3	3	6
853	851	858 -	9155	9152	9155 -	0	6	6
865	862	866 +	808	807	808 -	4	2	6
874	870	874 -	1009	1007	1009 -	3	3	6
881	880	886 -	986	986	987 +	3	3	6
960	956	964 -	1087	1083	1087 -	3	3	6
1012	1012	1016 -	1153	1153	1155 -	3	3	6
1021	1019	1024 +	962 -	-	-	3	3	6
1022	1018	1026 -	1071	1067	1071 +	4	2	6
1036	1035	1036 -	1062	1061	1063 +	1	5	6
1055 -	-	-	1064 -	-	+	6	0	6
1092	1087	1094 +	1327	1325	1327 +	3	3	6
1098	1092	1101 -	1234	1230	1234 -	3	3	6
1098	1092	1101 -	1254	1254	1256 -	3	3	6
1111	1108	1115 -	1172	1168	1172 +	3	3	6
1145	1141	1149 -	1327 -	-	-	3	3	6
1167	1163	1171 -	1252	1252	1253 +	3	3	6
1167	1163	1171 -	1334	1334	1338 -	3	3	6
1167	1167	1168 +	1249	1249	1250 -	3	3	6
1167	1167	1168 +	1303 -	-	-	3	3	6
1177	1177	1179 +	1293 -	-	-	3	3	6
1179	1176	1183 -	1326	1325	1326 -	3	3	6
1202	1199	1207 -	1558 -	-	-	3	3	6
1214	1211	1215 -	5989	5989	5992 +	6	0	6
1230	1228	1232 -	11175 -	-	-	0	6	6
1248	1245	1252 +	1166	1164	1167 -	3	3	6
1268	1265	1272 +	1150 -	-	-	3	3	6
1268	1265	1272 +	1170	1166	1171 -	3	3	6
1362	1362	1366 +	1253	1250	1253 -	3	3	6
1383	1380	1385 -	1683	1679	1683 +	3	3	6
1407	1404	1412 +	1503	1503	1504 -	3	3	6
1418	1414	1422 -	1457 -	-	+	3	3	6
1442	1442	1446 -	1495 -	-	-	3	3	6
1442	1439	1447 +	1460	1456	1460 -	3	3	6
1468	1467	1472 -	1626 -	-	+	3	3	6
1487	1484	1491 -	1517	1517	1520 +	3	3	6
1487	1484	1491 -	1693	1693	1696 -	3	3	6
1499	1495	1501 -	1538 -	-	-	3	3	6
1508	1504	1511 +	1446 -	-	-	3	3	6
1513	1509	1517 -	1684	1684	1688 -	3	3	6
1530	1525	1535 -	1725	1723	1725 -	3	3	6
1568	1564	1571 -	1722	1722	1726 -	3	3	6
1655	1652	1661 -	1838	1838	1840 -	3	3	6
1691	1689	1694 -	2003 -	-	-	3	3	6
1778	1776	1779 +	1848	1848	1849 -	3	3	6
1847	1846	1848 -	2004	2000	2004 -	3	3	6
1870	1867	1871 -	2000	2000	2002 -	3	3	6
1911	1909	1911 +	1973 -	-	-	3	3	6
1919	1919	1923 -	2086	2086	2089 -	3	3	6
1962	1961	1964 -	2738 -	-	-	3	3	6
2004	2000	2004 -	2124	2124	2125 +	3	3	6
2031	2026	2031 -	12507	12505	12507 -	1	5	6
2066	2064	2069 -	2171	2169	2171 -	3	3	6
2083	2082	2085 -	2187	2187	2188 -	3	3	6
2115	2112	2115 -	2150	2150	2154 -	3	3	6
2157	2154	2157 +	2292	2292	2293 -	3	3	6
2207	2205	2211 -	2244	2241	2244 +	3	3	6
2207	2205	2211 -	2409 -	-	-	3	3	6
2303	2302	2304 -	2469	2469	2470 -	3	3	6
2313	2309	2317 +	2382 -	-	-	3	3	6
2329	2328	2329 +	2357 -	-	-	3	3	6

2359	2359	2364	-	2484	2484	2486	-	3	3	6
2415	2411	2418	-	2648	2644	2648	-	3	3	6
2466	2464	2470	-	12082	12080	12082	-	6	0	6
2492	2487	2496	-	2655	2655	2656	-	3	3	6
2590	2585	2593	+	2535 -	-		-	3	3	6
2625	2622	2625	-	2758	2756	2762	-	3	3	6
2797	2795	2797	-	2944	2943	2946	-	3	3	6
2825	2822	2830	-	3008	3008	3013	-	3	3	6
2858	2854	2858	-	2885 -	-		+	3	3	6
2883	2879	2887	-	3055	3051	3055	-	3	3	6
2907	2902	2910	-	3070 -	-		-	3	3	6
2956 -	-		+	2987 -	-		+	6	0	6
2962	2960	2966	-	2941 -	-		+	6	0	6
2962	2960	2966	-	3010	3010	3011	+	5	1	6
2962	2961	2963	+	2941	2940	2941	-	3	3	6
2981	2979	2983	-	3131	3129	3135	-	3	3	6
2993	2990	2997	-	3130 -	-		-	3	3	6
3048	3046	3050	-	3070	3068	3070	+	3	3	6
3064	3060	3070	-	3234	3231	3234	-	3	3	6
3077	3077	3080	+	3055 -	-		-	6	0	6
3196	3194	3200	-	3351 -	-		+	3	3	6
3229	3228	3233	-	3409	3407	3409	-	3	3	6
3247 -	-		+	3273 -	-		-	3	3	6
3403	3399	3406	-	3515	3515	3516	+	3	3	6
3411	3407	3414	-	3547 -	-		-	3	3	6
3482	3479	3486	-	3656	3652	3656	+	3	3	6
3515	3512	3519	-	3680	3679	3680	-	3	3	6
3585	3581	3588	-	3588	3585	3588	+	4	2	6
3595	3591	3601	-	3743 -	-		-	3	3	6
3606	3605	3609	-	3665 -	-		+	3	3	6
3825	3821	3827	-	3986	3982	3986	-	3	3	6
3911	3909	3911	+	4289	4285	4289	+	4	2	6
3975 -	-		-	4005 -	-		+	3	3	6
4102	4100	4104	-	4298 -	-		-	3	3	6
4170	4166	4174	+	4260	4256	4261	-	3	3	6
4222	4218	4225	-	4371	4371	4374	-	3	3	6
4222	4218	4225	-	4411	4411	4412	-	3	3	6
4236	4232	4238	-	4374	4372	4374	-	3	3	6
4251	4250	4252	-	4354	4354	4355	-	3	3	6
4315	4311	4316	+	8414	8413	8418	-	5	1	6
4326	4326	4329	-	4487	4487	4489	-	3	3	6
4350	4348	4353	-	4489	4486	4489	-	3	3	6
4437	4435	4438	+	4571	4571	4573	-	3	3	6
4442	4442	4444	-	4644 -	-		-	3	3	6
4483 -	-		+	4535 -	-		+	3	3	6
4508	4506	4511	-	4698	4696	4698	+	3	3	6
4592	4589	4592	-	4770	4770	4774	-	3	3	6
4610	4609	4615	-	6071 -	-		-	3	3	6
4641	4638	4645	+	5107	5106	5108	-	3	3	6
4648	4647	4649	-	5163	5161	5163	-	3	3	6
4868	4866	4869	-	9714	9713	9714	+	2	4	6
4961	4959	4962	-	5080	5078	5080	-	3	3	6
4982	4980	4986	-	5028	5024	5029	+	3	3	6
5002	5001	5002	-	5001	5001	5005	+	6	0	6
5017	5014	5021	-	5163	5159	5164	-	3	3	6
5053	5049	5055	-	5100 -	-		+	3	3	6
5053	5049	5055	-	5107	5105	5107	+	3	3	6
5087	5084	5087	-	5124 -	-		-	3	3	6
5101	5101	5106	-	5195 -	-		-	1	5	6
5133	5131	5133	-	5259 -	-		-	3	3	6
5290	5288	5290	+	5207 -	-		-	3	3	6
5313	5309	5317	-	5507	5507	5508	-	3	3	6
5412	5408	5416	+	5434	5432	5434	+	3	3	6
5513	5512	5514	-	5553 -	-		+	6	0	6
5647	5643	5647	-	5788 -	-		-	3	3	6
5658	5657	5662	-	5796	5795	5796	-	3	3	6
5667	5663	5669	-	5676 -	-		+	6	0	6
5689	5689	5691	-	5712	5710	5712	+	4	2	6
5741 -	-		+	5763 -	-		-	3	3	6
5747	5747	5751	+	5736	5736	5737	-	3	3	6
5799	5799	5801	+	5745	5745	5747	-	3	3	6
5861	5857	5862	-	5971	5970	5971	-	3	3	6
5874 -	-		-	5908 -	-		+	6	0	6
5908 -	-		-	5874 -	-		+	6	0	6
6196	6194	6199	-	11122	11121	11123	+	0	6	6
6401	6401	6402	+	6474 -	-		-	3	3	6
6404	6400	6405	-	6448 -	-		+	4	2	6
6464	6463	6467	-	6528 -	-		+	3	3	6
6749	6744	6749	-	6913	6909	6913	-	2	4	6
6850	6846	6853	+	6809	6809	6813	-	3	3	6
6864	6861	6868	-	6998	6994	6999	-	3	3	6
6915	6914	6916	+	6874	6874	6875	-	3	3	6
6977 -	-		+	7007 -	-		+	3	3	6
6988	6988	6993	-	7266 -	-		-	3	3	6
7034	7034	7037	+	13436 -	-		+	3	3	6
7106	7103	7110	-	7317	7314	7317	-	3	3	6
7141	7141	7142	-	7242 -	-		+	3	3	6
7221	7220	7223	+	7164	7161	7164	-	3	3	6
7238	7235	7240	-	7381 -	-		-	3	3	6
7285	7281	7289	-	7298 -	-		+	0	6	6
7310	7310	7314	-	7485 -	-		-	3	3	6
7420	7418	7426	-	7586	7584	7586	-	3	3	6
7429	7429	7431	-	7510	7508	7510	+	3	3	6
7507	7505	7507	+	7663 -	-		-	3	3	6
7538	7533	7541	-	7724	7724	7727	-	3	3	6
7571	7570	7573	+	7684 -	-		-	3	3	6
7583	7583	7586	-	7738	7735	7740	-	3	3	6
7612	7608	7617	-	7799	7795	7800	-	3	3	6

7701		7698	7701	-	7774	-	-		+	3	3	6
7739		7739	7743	-	7864		7860	7864	-	3	3	6
7761		7761	7765	+	7716		7712	7716	-	3	3	6
7764		7763	7768	-	8136	-	-		+	3	3	6
7888		7888	7890	-	8007		8005	8007	-	3	3	6
7940		7936	7941	-	7994		7991	7995	+	2	4	6
7962		7959	7967	-	8061		8061	8065	-	3	3	6
8094		8094	8098	+	7991	-	-		-	3	3	6
8140		8140	8142	-	8236	-	-		-	6	0	6
8156		8153	8157	-	8340	-	-		-	3	3	6
8198		8195	8201	+	8157	-	-		-	3	3	6
8200		8200	8204	-	8413	-	-		-	3	3	6
8244		8240	8245	-	8395		8395	8397	-	3	3	6
8244		8240	8245	-	8419		8418	8419	-	3	3	6
8406		8406	8410	-	8449	-	-		+	3	3	6
8474		8470	8478	-	8540		8536	8540	+	3	3	6
8524		8520	8526	-	8678		8678	8682	-	3	3	6
8524		8520	8526	-	8700		8700	8703	-	3	3	6
8531		8531	8532	-	8586	-	-		+	3	3	6
8923		8918	8927	-	9123		9123	9124	-	3	3	6
8963		8958	8969	-	9145		9142	9149	-	3	3	6
8975	-	-		+	8834	-	-		-	3	3	6
8978		8974	8981	-	11491	-	-		+	3	3	6
9008		9003	9009	-	9170	-	-		+	3	3	6
9030		9026	9034	-	9211		9210	9211	-	3	3	6
9068		9064	9073	-	9217		9215	9219	-	3	3	6
9068		9064	9073	-	9232		9230	9233	-	3	3	6
9134		9134	9135	-	9158		9157	9158	+	1	5	6
9163		9163	9165	-	9323	-	-		-	3	3	6
9165		9161	9170	+	9142		9142	9143	-	3	3	6
9185		9181	9190	-	9383		9383	9388	-	3	3	6
9194		9192	9200	-	9352		9352	9354	-	3	3	6
9231		9231	9232	+	9327	-	-		-	3	3	6
9314		9309	9315	-	9463		9462	9463	-	3	3	6
9405	-	-		-	9495	-	-		+	3	3	6
9429	-	-		+	9366	-	-		-	0	6	6
9434	-	-		+	9361	-	-		-	6	0	6
9447		9445	9451	+	9604	-	-		-	3	3	6
9477		9473	9477	-	9631	-	-		+	3	3	6
9499		9497	9502	-	9606	-	-		+	3	3	6
9547		9542	9550	-	9725		9725	9727	-	3	3	6
9571		9570	9573	-	9601		9599	9601	+	3	3	6
9597		9593	9599	+	9554		9550	9554	-	3	3	6
9704		9703	9709	+	9673		9672	9673	-	3	3	6
9704		9703	9709	+	9913		9913	9917	-	3	3	6
9768		9764	9769	-	9920		9920	9924	-	3	3	6
9773		9772	9777	-	9937	-	-		-	3	3	6
9840		9838	9840	-	9839	-	-		+	6	0	6
9905		9901	9908	-	10099		10096	10099	-	3	3	6
9916		9912	9922	-	10106	-	-		-	3	3	6
9970		9966	9970	-	9994	-	-		+	6	0	6
10004		10000	10007	-	10079	-	-		-	3	3	6
10081		10079	10087	+	10107	-	-		-	3	3	6
10162		10159	10166	-	10288		10285	10288	-	3	3	6
10208		10206	10210	-	10337		10335	10338	-	3	3	6
10217		10215	10221	-	10387		10386	10387	+	3	3	6
10302		10299	10305	-	10433		10430	10433	-	3	3	6
10403		10400	10407	-	10428	-	-		-	3	3	6
10414		10408	10417	-	10598	-	-		-	3	3	6
10467		10467	10468	+	10364		10363	10364	-	3	3	6
10485		10483	10487	-	10597		10597	10599	-	3	3	6
10676		10672	10677	+	10618	-	-		-	3	3	6
10721		10721	10723	+	14850		14849	14850	-	4	2	6
10764		10762	10765	-	10830		10827	10830	+	3	3	6
10798		10794	10798	+	12300		12296	12300	+	3	3	6
11005	-	-		+	11061	-	-		-	3	3	6
11127		11123	11130	-	11283		11279	11283	-	1	5	6
11127		11123	11130	-	11299		11297	11299	-	3	3	6
11162		11160	11162	-	11187	-	-		+	3	3	6
11163	-	-		+	11136	-	-		-	3	3	6
11200		11197	11203	-	11298		11297	11298	-	3	3	6
11244		11240	11247	-	11407		11407	11409	-	3	3	6
11292		11289	11295	-	11320		11320	11321	+	3	3	6
11318		11313	11321	-	11465		11464	11465	-	2	4	6
11363		11363	11366	-	11526		11525	11527	-	3	3	6
11444		11444	11445	-	11574	-	-		-	0	6	6
11452		11451	11457	-	11499		11497	11499	+	4	2	6
11622		11618	11624	-	11781		11781	11783	-	3	3	6
11657		11657	11660	+	11604		11601	11604	-	3	3	6
11732		11728	11734	-	11905	-	-		-	3	3	6
11948		11947	11952	-	12054		12054	12055	+	3	3	6
12000		11997	12003	-	12209		12208	12210	-	3	3	6
12027		12027	12031	+	12018		12014	12018	-	3	3	6
12047		12044	12051	-	12210		12209	12210	-	3	3	6
12076		12073	12076	+	12137	-	-		-	3	3	6
12085		12081	12086	-	12125		12124	12126	+	5	1	6
12126		12121	12130	-	12227		12224	12228	-	3	3	6
12136		12132	12140	-	12288	-	-		+	3	3	6
12222		12219	12225	-	12386		12383	12386	+	3	3	6
12228		12227	12231	-	13459	-	-		+	3	3	6
12273		12273	12277	+	12183		12183	12185	-	3	3	6
12343		12339	12348	-	12489		12489	12491	-	3	3	6
12343		12339	12348	-	12520		12516	12520	-	3	3	6
12343		12339	12348	-	12557		12557	12560	-	3	3	6
12412		12412	12415	-	12585	-	-		-	3	3	6
12508		12508	12509	-	13327		13327	13328	-	3	3	6
12589		12588	12594	-	12778		12774	12778	-	3	3	6
12607		12603	12608	-	12547		12546	12547	+	3	3	6

12676	12672	12676	-	12817	-	-	-	3	3	6
12723	12722	12723	+	12758	-	-	-	3	3	6
12751	12747	12755	-	12967	12966	12970	-	3	3	6
12767	12763	12771	-	12937	12936	12937	-	3	3	6
12767	12763	12771	-	12959	12955	12959	-	2	4	6
12814	12810	12818	-	12884	-	-	+	3	3	6
12834	12834	12838	+	12707	-	-	-	3	3	6
12850	12847	12851	-	12896	12894	12896	-	3	3	6
12933	12929	12938	-	13089	-	-	-	3	3	6
12933	12929	12938	-	13995	-	-	+	3	3	6
13001	12999	13006	-	13070	-	-	+	3	3	6
13016	13012	13016	-	13163	-	-	-	3	3	6
13024	13018	13028	-	12988	12988	12990	+	6	0	6
13056	13054	13056	-	13106	13106	13108	+	5	1	6
13082	13078	13085	+	13004	13003	13006	-	4	2	6
13110	13109	13114	-	13288	-	-	-	3	3	6
13114	13110	13115	+	13051	13049	13052	-	3	3	6
13188	13188	13189	+	13065	13064	13065	-	3	3	6
13271	13268	13273	-	13483	-	-	-	3	3	6
13279	13278	13280	-	13506	13503	13506	-	3	3	6
13373	13371	13373	-	13533	-	-	+	3	3	6
13470	13465	13471	-	13566	-	-	+	3	3	6
13580	13577	13580	-	13676	13676	13677	-	3	3	6
13611	13608	13612	+	13546	13545	13546	-	4	2	6
13632	13632	13635	+	13658	-	-	-	3	3	6
13635	13634	13638	-	13784	13781	13784	-	4	2	6
13640	13639	13641	-	13703	-	-	-	3	3	6
13647	13646	13647	-	13782	13782	13783	-	3	3	6
13668	13664	13668	-	13696	13692	13696	+	3	3	6
13745	13743	13748	-	13894	13893	13894	-	3	3	6
13762	13760	13763	+	14023	-	-	-	3	3	6
13786	13785	13787	+	13957	13956	13957	-	3	3	6
13911	13910	13913	+	13893	13893	13894	-	3	3	6
13996	13992	14000	-	14160	14159	14161	-	3	3	6
14016	14014	14016	-	14129	14129	14133	-	3	3	6
14073	-	-	+	13992	-	-	-	3	3	6
14143	14142	14145	-	14188	-	-	+	3	3	6
14155	14155	14157	+	14410	-	-	+	3	3	6
14157	14155	14160	-	14191	14191	14194	-	3	3	6
14157	14155	14160	-	14303	14303	14304	-	3	3	6
14168	14165	14171	-	14343	14343	14346	-	3	3	6
14255	14254	14257	-	14425	14424	14425	-	3	3	6
14301	14297	14306	-	14444	14443	14444	-	3	3	6
14324	14322	14326	-	14369	-	-	+	5	1	6
14382	14379	14384	-	14588	14588	14591	-	3	3	6
14409	14404	14411	-	14569	14569	14570	-	3	3	6
14409	14404	14411	-	14578	-	-	+	4	2	6
14435	14435	14440	-	14530	14528	14530	+	4	2	6
14438	14434	14442	+	14251	-	-	-	3	3	6
14447	14447	14451	-	14540	14540	14542	+	3	3	6
14457	-	-	+	14435	-	-	-	0	6	6
14479	14476	14482	-	14598	14598	14600	+	3	3	6
14504	14503	14507	-	14551	14551	14552	+	3	3	6
14566	14563	14568	-	14773	-	-	-	3	3	6
14583	14579	14587	-	14817	14815	14819	-	3	3	6
14646	14645	14648	+	14681	14678	14681	-	4	2	6
14676	14672	14680	-	14718	14718	14722	+	3	3	6
14755	14755	14757	-	14823	14821	14823	+	3	3	6
14763	-	-	-	14861	-	-	+	3	3	6
14776	14773	14781	-	14928	14927	14929	-	3	3	6
14801	14799	14801	-	14882	-	-	+	3	3	6
14802	14801	14806	+	14816	14816	14818	-	4	2	6
14806	14802	14807	-	14835	-	-	-	6	0	6
14812	14811	14812	-	14841	-	-	-	0	6	6
14824	14823	14827	-	14858	14858	14859	+	1	5	6
14847	14846	14849	-	15044	15044	15045	+	3	3	6
14859	14855	14860	+	15031	15031	15032	-	3	3	6
14861	14859	14862	-	15029	15029	15030	+	3	3	6
14902	14898	14906	+	14767	-	-	-	3	3	6
14904	14904	14908	-	14950	-	-	+	6	0	6
14912	14910	14915	-	14963	-	-	+	6	0	6
14917	14916	14917	-	14958	14958	14959	+	0	6	6
14944	14943	14947	-	15080	15080	15083	-	3	3	6
14944	14943	14947	-	15085	15085	15086	-	3	3	6
14972	14968	14974	-	15147	15145	15147	-	3	3	6
14999	14994	15000	+	15050	15050	15051	-	3	3	6
15010	15010	15015	-	15092	-	-	+	3	3	6
15010	15010	15015	-	15168	15165	15171	-	3	3	6
15114	15112	15117	-	15313	-	-	-	3	3	6
15129	15126	15130	-	15234	15234	15238	-	3	3	6
15129	15126	15130	-	15342	15342	15343	-	3	3	6
15144	15141	15146	-	15338	15338	15341	-	3	3	6
15220	15219	15220	-	15331	-	-	-	3	3	6
15236	15233	15236	-	15384	15382	15384	-	3	3	6
15340	15340	15342	+	15369	-	-	-	3	3	6
1	2	8	-	92	92	94	-	3	2	5
14	9	18	-	283	-	-	+	2	3	5
98	94	99	+	213	-	-	-	2	3	5
157	156	157	+	173	173	174	-	3	2	5
254	251	256	-	331	330	332	-	2	3	5
295	290	299	+	345	-	-	-	3	2	5
320	316	325	+	421	-	-	-	2	3	5
343	343	346	+	295	-	-	-	3	2	5
380	379	384	-	555	-	-	-	2	3	5
561	556	565	-	606	605	607	+	3	2	5
561	556	565	-	702	702	704	-	3	2	5
611	608	615	-	768	766	768	-	2	3	5
635	633	637	-	665	-	-	+	5	0	5

807	803	811 -	978	978	982 -	3	2	5
895	893	898 -	977	973	977 +	3	2	5
987	984	992 -	1186	1186	1188 -	1	4	5
1036	1035	1041 +	1062 -	-	-	5	0	5
1145	1141	1149 -	1381 -	-	-	2	3	5
1195	1192	1197 -	1220	1220	1221 +	3	2	5
1230	1228	1232 -	7403	7403	7405 +	2	3	5
1334	1333	1338 +	1414 -	-	-	0	5	5
1341 -	-	+	1408 -	-	-	5	0	5
1355	1352	1359 +	1307	1306	1307 -	2	3	5
1428	1426	1428 +	1473	1473	1477 -	3	2	5
1452	1447	1452 -	1488	1488	1490 +	2	3	5
1452	1447	1452 -	4423	4421	4423 +	3	2	5
1460	1457	1463 -	1442	1442	1443 +	5	0	5
1494	1494	1497 +	1442	1442	1444 -	3	2	5
1537	1537	1540 -	1634	1634	1638 +	2	3	5
1559	1558	1562 -	1650	1649	1650 -	2	3	5
1620	1616	1621 +	1703 -	-	+	0	5	5
1625	1624	1625 +	1708 -	-	+	5	0	5
1632	1630	1632 -	1705	1705	1707 +	4	1	5
1655	1652	1661 -	1861	1861	1863 -	2	3	5
1766	1764	1766 -	1785	1785	1787 +	4	1	5
1861	1861	1863 +	1766	1764	1766 -	2	3	5
1952	1950	1956 -	2087 -	-	-	3	2	5
2023	2022	2025 -	2105	2105	2106 +	3	2	5
2041	2040	2043 +	12521	12519	12524 +	3	2	5
2052	2050	2055 -	2023 -	-	+	5	0	5
2107	2104	2109 +	2170	2167	2170 -	4	1	5
2140	2137	2143 -	2795 -	-	-	3	2	5
2542	2536	2542 -	2709 -	-	+	2	3	5
2832	2832	2835 -	2809 -	-	+	3	2	5
2927	2923	2927 +	2980 -	-	-	1	4	5
2972	2968	2977 -	3003	3003	3006 +	1	4	5
3229	3228	3233 -	3495	3495	3496 -	2	3	5
3235	3233	3235 +	4489	4488	4489 +	5	0	5
3291	3290	3296 -	7699 -	-	+	5	0	5
3482	3479	3486 -	3557	3557	3561 +	1	4	5
3590	3589	3590 -	3631	3631	3635 +	0	5	5
4058	4055	4063 -	4120	4120	4121 +	3	2	5
4090	4089	4094 -	4107	4104	4107 +	3	2	5
4236	4232	4238 -	4437 -	-	-	2	3	5
4236	4232	4238 -	4451	4451	4454 -	2	3	5
4247	4246	4251 +	12082 -	-	-	5	0	5
4258	4255	4260 +	4348	4347	4348 -	4	1	5
4940	4936	4944 -	4953	4953	4955 +	4	1	5
4976	4976	4979 -	5037	5034	5037 +	1	4	5
5096	5092	5099 -	5216	5214	5220 -	3	2	5
5101	5101	5106 -	11815	11815	11819 +	3	2	5
5213	5209	5218 -	5226	5226	5230 +	2	3	5
5277	5273	5281 -	5418	5417	5419 -	2	3	5
5332	5331	5335 +	5346	5346	5350 -	3	2	5
5518	5517	5519 -	5548	5548	5549 +	0	5	5
5612	5609	5612 -	5594 -	-	+	5	0	5
5701	5700	5705 -	5840	5836	5840 +	3	2	5
5749	5749	5750 -	5798	5796	5798 +	3	2	5
6386	6384	6390 -	6494	6490	6494 +	3	2	5
6404	6400	6405 -	6414 -	-	+	2	3	5
6518	6513	6520 -	6473 -	-	+	5	0	5
6523	6523	6527 -	6698 -	-	-	0	5	5
6650	6650	6653 -	6684	6682	6684 +	4	1	5
7106	7103	7110 -	7300	7300	7302 -	3	2	5
7143	7143	7146 +	7131 -	-	-	0	5	5
7238	7235	7240 -	7309 -	-	-	5	0	5
7245	7245	7247 -	7314 -	-	-	0	5	5
7263	7262	7263 +	12426 -	-	+	5	0	5
7485	7481	7488 -	7515	7512	7517 +	2	3	5
7512	7512	7515 -	7600 -	-	+	5	0	5
7518	7517	7522 -	7594	7594	7595 +	0	5	5
7800	7797	7804 +	7883	7882	7883 -	3	2	5
8004	7999	8007 -	8011 -	-	+	5	0	5
8150	8148	8152 +	7984	7984	7985 -	2	3	5
8155 -	-	+	7985	7981	7985 -	3	2	5
8363	8361	8366 -	8562	8562	8563 -	3	2	5
8396	8393	8401 +	8363 -	-	-	0	5	5
8404	8403	8405 +	8360 -	-	-	5	0	5
8406	8406	8410 -	8572	8568	8574 -	3	2	5
8431	8429	8432 +	15363 -	-	+	5	0	5
8484	8480	8487 -	8644	8643	8648 -	2	3	5
8603	8603	8606 -	8660 -	-	+	5	0	5
8608	8608	8610 -	8655 -	-	+	0	5	5
8907	8907	8915 -	8956 -	-	+	2	3	5
8963	8958	8969 -	9118	9118	9122 -	3	2	5
9297	9293	9300 +	9273	9272	9273 -	3	2	5
9543	9540	9545 +	9498	9498	9501 -	3	2	5
9543	9540	9545 +	9723	9721	9724 -	2	3	5
9635	9635	9638 +	9522 -	-	-	2	3	5
9956 -	-	-	10004 -	-	+	2	3	5
10183	10178	10185 -	10413	10410	10413 +	3	2	5
10599	10599	10600 +	10515 -	-	-	2	3	5
10710	10708	10713 +	14861 -	-	-	0	5	5
11102	11099	11105 -	11257 -	-	-	3	2	5
11218	11218	11221 +	11251	11251	11254 -	3	2	5
11478	11475	11484 -	11574	11572	11574 +	2	3	5
11506	11506	11509 -	11628 -	-	-	3	2	5
11905	11901	11907 -	12076	12076	12079 -	3	2	5
11988	11982	11990 -	12156	12155	12157 -	2	3	5
12040	12040	12042 +	12058	12056	12058 -	2	3	5
12136	12132	12140 -	12157	12157	12160 +	2	3	5

12195	12192	12198 +	12317	12314	12317 -	3	2	5
12203	12200	12203 +	12223	12221	12223 -	2	3	5
12360	12357	12362 -	12493	12491	12493 +	2	3	5
12467	12467	12469 +	12576 -	-	-	2	3	5
12486	12485	12491 +	12412 -	-	+	2	3	5
12557	12555	12560 +	12624	12624	12625 -	1	4	5
12792 -	-	+	12729 -	-	-	2	3	5
12850	12847	12851 -	12905 -	-	+	2	3	5
12856	12854	12856 +	12822 -	-	-	0	5	5
12861	12861	12864 +	12817 -	-	-	5	0	5
12872	12869	12875 +	12893 -	-	-	3	2	5
12893	12893	12898 +	12806 -	-	-	2	3	5
12893	12893	12898 +	12871	12871	12875 -	3	2	5
13070	13069	13071 +	13001	13001	13002 -	2	3	5
13082	13078	13085 +	13096	13092	13096 -	3	2	5
13131	13126	13134 +	13176	13175	13176 -	3	2	5
13440 -	-	-	14553 -	-	+	3	2	5
13704	13702	13707 -	13866	13866	13869 -	2	3	5
13867 -	-	+	13911 -	-	-	4	1	5
13884 -	-	+	13803 -	-	-	0	5	5
13891 -	-	+	13796 -	-	-	5	0	5
13959	13956	13959 +	13894	13891	13894 +	3	2	5
13975	13972	13975 -	14177 -	-	+	5	0	5
13982	13977	13982 -	14170 -	-	+	0	5	5
13988	13985	13991 -	14197 -	-	-	3	2	5
13992	13990	13992 +	14073	14073	14074 -	2	3	5
14042	14038	14042 -	14246 -	-	-	3	2	5
14177	14172	14178 -	14215 -	-	+	5	0	5
14182	14180	14184 -	14210 -	-	+	0	5	5
14540	14536	14543 -	14659	14658	14660 -	3	2	5
14551	14550	14551 +	14504	14504	14505 -	2	3	5
14568	14563	14572 +	14450	14449	14450 -	3	2	5
14647	14645	14651 -	14683 -	-	+	3	2	5
14653	14651	14653 +	14676 -	-	-	2	3	5
14706	14704	14710 -	14923	14922	14923 +	3	2	5
14951	14950	14955 -	15157 -	-	-	2	3	5
15045	15043	15045 -	15117	15116	15118 +	3	2	5
15169	15167	15169 -	15185 -	-	+	5	0	5
15185 -	-	-	15169 -	-	+	5	0	5
15241	15239	15245 +	15208	15204	15209 -	3	2	5
15256	15255	15258 +	15263	15263	15266 -	3	2	5
1	2	8 -	74 -	-	-	2	2	4
1	2	8 -	164 -	-	-	2	2	4
1	2	8 -	217	217	221 -	2	2	4
1	2	8 -	7068 -	-	-	2	2	4
14	9	18 -	112 -	-	+	2	2	4
14	9	18 -	123	123	124 -	2	2	4
23	19	26 -	205 -	-	-	2	2	4
59	57	60 -	234	234	235 -	2	2	4
67	62	70 -	251	251	252 -	2	2	4
81	78	81 -	626 -	-	-	2	2	4
105	102	106 +	273	270	273 +	2	2	4
130	129	131 -	143 -	-	+	0	4	4
130	129	131 -	188	188	189 +	2	2	4
187	184	192 -	355	355	357 -	2	2	4
187	187	190 +	106 -	-	-	2	2	4
211	210	214 -	277	273	277 +	3	1	4
221	219	221 +	266	266	267 +	2	2	4
224	223	229 -	260 -	-	+	3	1	4
224	223	229 -	345	345	346 -	2	2	4
227	224	229 +	270 -	-	+	2	2	4
242	240	245 -	427	425	427 -	2	2	4
254	251	256 -	465	462	465 -	2	2	4
274	269	278 -	340 -	-	+	2	2	4
274	269	278 -	429	426	429 -	2	2	4
274	269	278 -	434 -	-	-	2	2	4
274	269	278 -	626	626	628 +	2	2	4
298	297	302 -	441	438	441 -	2	2	4
298	297	302 -	475 -	-	-	2	2	4
320	319	321 -	421 -	-	+	2	2	4
320	316	325 +	320	320	321 -	2	2	4
320	316	325 +	370 -	-	-	4	0	4
331	331	334 -	346 -	-	+	2	2	4
331	331	334 -	391	391	392 +	2	2	4
341	338	345 -	505	502	505 -	2	2	4
351	347	352 -	555 -	-	-	2	2	4
356	355	360 -	505	501	505 -	2	2	4
358	358	360 +	280	280	281 -	2	2	4
385	380	389 +	408 -	-	+	2	2	4
393	391	396 +	308	308	312 -	2	2	4
393	391	396 +	502	498	502 -	2	2	4
395	389	399 -	533 -	-	+	2	2	4
395	389	399 -	558 -	-	-	2	2	4
412	410	417 -	545 -	-	-	2	2	4
412	410	417 -	593	589	595 -	2	2	4
426	422	428 -	597 -	-	-	2	2	4
442	440	442 +	522 -	-	+	2	2	4
483	480	487 -	612	608	612 +	2	2	4
483	480	487 -	794 -	-	-	2	2	4
503	498	506 -	703	703	704 -	2	2	4
503	498	506 -	6388 -	-	-	2	2	4
513	513	516 +	450	447	450 -	2	2	4
515	510	519 -	662	662	663 -	2	2	4
515	510	519 -	680 -	-	-	2	2	4
515	510	519 -	709 -	-	-	2	2	4
530	526	530 -	671	671	673 -	2	2	4
530	526	530 -	682	678	682 -	2	2	4
530	526	530 -	709	709	713 -	2	2	4

530	526	530 -	721 -	-	-	2	2	4
530	526	530 -	731	729	731 -	2	2	4
537 -	-	-	700 -	-	-	2	2	4
550	548	553 -	609 -	-	+	2	2	4
558	554	562 +	628 -	-	-	2	2	4
558	554	562 +	679	675	679 -	2	2	4
584	582	585 -	975 -	-	-	2	2	4
592	592	595 +	679 -	-	-	2	2	4
606	602	607 -	778 -	-	-	2	2	4
611	608	615 -	667 -	-	+	2	2	4
644	638	645 -	829 -	-	-	2	2	4
644	640	645 +	595 -	-	-	2	2	4
659	656	659 +	583	583	587 -	2	2	4
670	669	673 -	811 -	-	-	2	2	4
670	670	672 +	788	788	790 -	2	2	4
680	677	680 +	780 -	-	-	2	2	4
693	689	695 -	760	760	761 +	2	2	4
693	689	695 -	767	767	768 +	2	2	4
726	725	727 -	758	758	759 +	2	2	4
743	742	747 -	779 -	-	+	4	0	4
743	742	747 -	785 -	-	+	2	2	4
752	749	756 -	774 -	-	+	0	4	4
752	749	756 -	784 -	-	+	2	2	4
760	760	763 -	780 -	-	+	2	2	4
778	774	781 -	874	871	874 -	2	2	4
791	786	794 -	911	908	911 -	2	2	4
791	786	794 -	961 -	-	-	2	2	4
791	786	794 -	1418 -	-	+	2	2	4
793	793	795 +	666	666	667 -	2	2	4
807	803	811 -	944	944	948 -	2	2	4
822	821	824 -	997	993	997 -	2	2	4
822	821	824 -	1004 -	-	-	2	2	4
829	825	833 -	1018	1018	1019 -	2	2	4
853	851	858 -	1067 -	-	-	2	2	4
855	855	859 +	9157	9153	9157 +	2	2	4
865	863	868 -	979 -	-	+	2	2	4
865	863	868 -	1523	1523	1524 -	2	2	4
874	870	874 -	984	981	984 -	2	2	4
874	870	874 -	995 -	-	-	2	2	4
895	893	898 -	924	923	924 +	2	2	4
907	902	907 -	1048	1046	1048 -	2	2	4
974	970	978 -	1128	1128	1129 -	2	2	4
977	973	978 +	895	895	898 -	2	2	4
1010	1007	1010 +	1021 -	-	-	0	4	4
1021	1019	1024 +	1010	1007	1010 -	4	0	4
1021	1019	1024 +	1102 -	-	-	2	2	4
1036	1035	1041 +	1056	1056	1057 -	2	2	4
1049	1047	1051 -	1109 -	-	+	2	2	4
1063	1063	1067 +	1102	1102	1103 -	2	2	4
1064	1060	1068 -	1092 -	-	+	2	2	4
1081	1077	1086 -	1243	1243	1244 -	2	2	4
1081	1077	1086 -	1249 -	-	-	2	2	4
1081	1077	1086 -	1261 -	-	-	2	2	4
1098	1092	1101 -	1214	1214	1215 -	2	2	4
1098	1092	1101 -	1271	1270	1271 -	2	2	4
1106	1104	1110 +	1087	1087	1088 -	2	2	4
1111	1108	1115 -	1161 -	-	+	2	2	4
1137	1134	1139 +	1328	1326	1328 +	2	2	4
1137	1134	1139 +	1336 -	-	+	2	2	4
1159	1156	1162 -	1207 -	-	-	2	2	4
1159	1156	1162 -	1307	1307	1308 +	2	2	4
1159	1156	1162 -	1327	1325	1327 -	2	2	4
1159	1156	1162 -	1334 -	-	-	2	2	4
1167	1163	1171 -	1301	1301	1302 +	2	2	4
1174 -	-	-	1335	1331	1335 -	2	2	4
1179	1176	1183 -	1335	1333	1335 -	2	2	4
1187	1186	1188 -	1419 -	-	-	2	2	4
1193	1193	1194 +	1244	1243	1244 +	2	2	4
1195	1192	1197 -	1383	1381	1383 -	2	2	4
1208	1208	1210 +	1228 -	-	+	2	2	4
1208	1208	1210 +	3853	3850	3853 -	2	2	4
1220	1220	1226 -	1328	1324	1328 -	2	2	4
1220	1220	1226 -	1368 -	-	-	2	2	4
1230	1228	1232 -	1383	1383	1384 -	2	2	4
1230	1228	1232 -	1682 -	-	-	2	2	4
1238	1235	1239 -	1381	1381	1382 -	2	2	4
1238	1235	1239 -	1778	1778	1779 -	2	2	4
1244	1240	1248 -	1388 -	-	-	2	2	4
1248	1245	1252 +	7388 -	-	-	3	1	4
1267	1262	1270 -	1510 -	-	-	2	2	4
1268	1265	1272 +	1283 -	-	-	2	2	4
1281	1280	1285 -	1269 -	-	+	4	0	4
1293	1292	1293 +	1134 -	-	-	2	2	4
1299	1296	1303 -	1359 -	-	+	2	2	4
1306	1303	1310 +	1157 -	-	-	2	2	4
1324	1322	1324 +	1374 -	-	-	2	2	4
1328	1324	1331 -	1329	1326	1329 +	4	0	4
1328	1324	1331 -	1354 -	-	+	2	2	4
1329	1326	1332 +	1356 -	-	-	2	2	4
1334	1333	1338 +	1265 -	-	-	2	2	4
1334	1333	1338 +	1344	1344	1345 -	2	2	4
1346	1343	1346 -	1353 -	-	+	4	0	4
1364	1361	1365 -	1451 -	-	+	2	2	4
1374	1372	1376 +	1324 -	-	-	2	2	4
1383	1380	1385 -	1562 -	-	-	2	2	4
1383	1380	1385 -	1596	1594	1596 -	2	2	4
1442	1442	1446 -	4425	4425	4427 +	3	1	4
1442	1439	1447 +	1497	1495	1497 -	1	3	4

1452		1447	1452	-	6702	-	-		-	1	3	4
1466		1466	1468	+	1392	-	-		-	2	2	4
1487		1484	1491	-	1660		1656	1660	-	2	2	4
1488		1488	1491	+	1451		1449	1451	-	1	3	4
1494		1494	1497	+	1507	-	-		-	2	2	4
1494		1494	1497	+	1532	-	-		-	2	2	4
1499		1495	1501	-	1676	-	-		-	2	2	4
1506		1503	1506	-	1658	-	-		-	2	2	4
1508		1504	1511	+	1395		1395	1396	-	2	2	4
1513		1512	1520	+	1395		1392	1395	-	2	2	4
1530		1525	1535	-	8088	-	-		-	2	2	4
1544		1541	1548	-	1630	-	-		+	2	2	4
1544		1541	1548	-	1700	-	-		-	2	2	4
1554		1550	1556	-	1673	-	-		-	2	2	4
1554		1550	1556	-	2166	-	-		-	2	2	4
1559		1558	1562	-	1741	-	-		-	2	2	4
1568		1564	1571	-	1693		1690	1693	-	2	2	4
1569		1566	1572	+	8130		8130	8131	+	2	2	4
1606		1606	1607	+	1566	-	-		-	2	2	4
1608		1605	1612	-	1755	-	-		-	2	2	4
1641		1637	1642	+	1672	-	-		+	3	1	4
1659	-	-		+	2252	-	-		-	2	2	4
1670		1667	1672	-	1864		1864	1865	-	2	2	4
1696		1696	1697	+	1671	-	-		-	2	2	4
1721		1718	1725	-	1751	-	-		+	2	2	4
1755		1752	1757	-	1834	-	-		-	2	2	4
1755		1752	1757	-	1893	-	-		+	2	2	4
1761	-	-		+	1780	-	-		-	2	2	4
1766		1764	1766	+	1861	-	-		-	2	2	4
1771		1771	1772	+	1779		1779	1780	-	2	2	4
1778		1778	1781	-	2008		2008	2009	-	2	2	4
1786		1785	1788	+	1765		1764	1766	-	2	2	4
1790		1786	1792	-	1958	-	-		-	2	2	4
1802		1799	1803	-	2012		2008	2012	-	2	2	4
1817		1814	1819	-	1957		1957	1959	-	2	2	4
1819		1819	1823	+	1977	-	-		+	2	2	4
1827		1823	1827	-	2012		2012	2013	-	2	2	4
1832		1831	1836	-	2003		2001	2003	-	2	2	4
1832		1831	1836	-	2013	-	-		-	2	2	4
1854		1851	1856	-	2013	-	-		-	2	2	4
1861		1860	1863	-	2027	-	-		-	2	2	4
1870		1867	1871	-	2088	-	-		-	2	2	4
1879		1876	1879	-	2079		2079	2080	-	2	2	4
1889		1887	1894	+	5844	-	-		+	2	2	4
1891		1889	1896	-	2079	-	-		-	2	2	4
1891		1889	1896	-	2087		2087	2088	-	2	2	4
1919		1919	1923	-	2093		2093	2094	-	2	2	4
1926		1925	1926	-	2091		2091	2092	-	2	2	4
1939		1935	1943	-	2072	-	-		-	2	2	4
1946	-	-		+	2261	-	-		+	2	2	4
1952		1950	1956	-	2079		2077	2079	-	2	2	4
1969		1967	1973	-	2082	-	-		-	2	2	4
1969		1967	1973	-	2106	-	-		-	2	2	4
1978		1975	1981	-	2167	-	-		-	2	2	4
1983		1983	1986	-	2182		2178	2182	-	2	2	4
2001		2000	2001	+	2124	-	-		-	2	2	4
2014	-	-		+	12493	-	-		+	2	2	4
2031		2026	2031	-	2067	-	-		+	4	0	4
2036		2035	2039	-	2062	-	-		+	0	4	4
2036		2035	2039	-	2108	-	-		-	4	0	4
2041		2041	2045	-	2113	-	-		-	0	4	4
2052		2050	2055	-	2093	-	-		+	2	2	4
2052		2050	2055	-	2115	-	-		+	2	2	4
2074		2071	2078	-	2156	-	-		-	2	2	4
2121		2121	2125	-	2144	-	-		+	2	2	4
2132		2131	2135	-	2908	-	-		+	2	2	4
2140		2137	2143	-	2264	-	-		-	2	2	4
2168	-	-		-	2568	-	-		-	2	2	4
2170		2167	2170	+	2205	-	-		+	2	2	4
2174		2170	2176	-	2361	-	-		-	2	2	4
2192		2188	2192	+	2268	-	-		-	2	2	4
2200	-	-		-	2268	-	-		+	2	2	4
2219		2215	2223	-	2325		2321	2325	+	2	2	4
2226		2223	2226	+	2234		2234	2235	-	2	2	4
2227		2226	2231	-	2375	-	-		-	2	2	4
2267		2266	2267	-	2394	-	-		-	2	2	4
2281		2279	2282	-	2446		2443	2446	-	2	2	4
2291		2287	2292	+	2223	-	-		-	2	2	4
2291		2287	2292	+	2406	-	-		-	2	2	4
2313		2309	2317	+	2394	-	-		+	2	2	4
2322		2320	2326	-	2480		2477	2480	-	1	3	4
2329		2328	2329	+	7238	-	-		+	2	2	4
2333		2329	2338	-	2487		2485	2487	-	2	2	4
2350		2348	2353	-	2443		2441	2443	-	2	2	4
2359		2359	2364	-	2443	-	-		+	2	2	4
2367		2367	2372	-	2475		2475	2476	-	2	2	4
2367		2367	2372	-	2551	-	-		-	2	2	4
2377		2373	2379	-	2502		2498	2502	-	2	2	4
2399		2395	2400	-	2700	-	-		-	2	2	4
2432		2428	2437	-	2625		2622	2625	-	2	2	4
2449		2449	2453	+	2413		2413	2415	-	2	2	4
2449		2449	2453	+	2496		2496	2498	+	2	2	4
2455		2451	2456	-	2511	-	-		+	2	2	4
2466		2464	2470	-	2620		2620	2623	-	2	2	4
2503		2498	2507	-	2518		2518	2520	+	2	2	4
2503		2498	2507	-	2653		2653	2656	+	2	2	4
2510		2510	2512	+	8349		8349	8350	+	2	2	4
2518		2514	2523	-	2521		2519	2521	+	2	2	4

2532	2532	2535 -	12082 -	-	-	4	0	4
2548	2543	2552 -	2672	2672	2673 +	2	2	4
2553	2550	2553 +	2489 -	-	-	2	2	4
2554	2554	2559 -	2631	2628	2631 +	1	3	4
2554	2554	2559 -	13855 -	-	+	2	2	4
2573	2571	2576 -	2757	2757	2762 -	2	2	4
2573	2573	2577 +	2673 -	-	-	2	2	4
2588	2585	2590 -	2763	2761	2763 -	2	2	4
2588	2585	2590 -	2768	2767	2768 -	2	2	4
2590	2585	2593 +	2518 -	-	-	2	2	4
2590	2585	2593 +	2600	2600	2605 -	2	2	4
2609	2605	2611 -	2698	2696	2698 +	2	2	4
2617	2613	2621 -	2766 -	-	-	2	2	4
2617	2613	2621 -	2793 -	-	-	2	2	4
2630	2626	2632 -	2754	2751	2754 -	2	2	4
2630	2626	2632 -	2819 -	-	-	2	2	4
2643	2640	2647 -	2696	2696	2697 +	2	2	4
2643	2640	2647 -	2787 -	-	-	2	2	4
2643	2639	2646 +	2526 -	-	-	2	2	4
2643	2639	2646 +	2550 -	-	-	2	2	4
2655	2652	2655 -	2945 -	-	-	2	2	4
2715	2711	2719 -	2784 -	-	-	2	2	4
2715	2711	2719 -	2830 -	-	-	2	2	4
2715	2711	2719 -	2899	2899	2900 -	2	2	4
2715	2711	2719 -	2919	2919	2920 -	2	2	4
2721	2721	2727 -	2860	2857	2860 -	2	2	4
2744	2744	2745 -	2939 -	-	-	2	2	4
2757 -	-	+	2799 -	-	+	2	2	4
2762	2762	2765 -	2890 -	-	-	2	2	4
2776 -	-	+	2745 -	-	-	2	2	4
2782	2779	2782 -	2950 -	-	-	2	2	4
2790	2790	2792 -	2921	2919	2921 -	2	2	4
2797	2795	2797 -	2843 -	-	+	2	2	4
2817	2815	2817 +	4341 -	-	-	2	2	4
2825	2822	2830 -	2884	2880	2884 +	2	2	4
2825	2822	2830 -	2949	2946	2953 -	2	2	4
2825	2822	2830 -	2987	2987	2989 -	2	2	4
2832	2832	2835 -	3334 -	-	+	2	2	4
2895	2891	2898 -	6007 -	-	-	2	2	4
2910	2910	2913 +	2897	2897	2898 -	2	2	4
2927	2923	2927 +	2993 -	-	+	2	2	4
2942	2941	2947 -	3095	3091	3095 -	2	2	4
2942	2941	2947 -	3122 -	-	-	2	2	4
2972	2968	2977 -	3162 -	-	-	2	2	4
2974	2971	2974 +	2998 -	-	+	2	2	4
2987	2987	2988 -	3130	3130	3131 -	2	2	4
3010	3009	3010 +	2961 -	-	-	2	2	4
3042	3040	3044 -	3215	3213	3215 -	2	2	4
3048	3046	3050 -	3110 -	-	-	2	2	4
3048	3046	3050 -	3167 -	-	-	2	2	4
3053	3053	3056 -	3181 -	-	-	2	2	4
3064	3060	3070 -	3213	3213	3214 -	2	2	4
3196	3194	3200 -	3348	3348	3349 -	2	2	4
3196	3194	3200 -	3372	3369	3372 -	2	2	4
3229	3228	3233 -	3341	3339	3341 -	2	2	4
3229	3228	3233 -	3369	3369	3373 -	2	2	4
3247	3244	3247 -	4502	4499	4502 -	1	3	4
3269	3265	3269 -	3411 -	-	-	2	2	4
3269	3265	3269 -	3413 -	-	+	4	0	4
3273	3270	3274 +	3247 -	-	-	2	2	4
3274	3271	3279 -	3408 -	-	+	0	4	4
3274	3271	3279 -	3417	3415	3417 -	2	2	4
3289	3285	3290 +	3261 -	-	-	2	2	4
3289	3285	3290 +	3272	3270	3272 -	2	2	4
3301	3298	3305 -	3354	3354	3356 +	2	2	4
3301	3298	3305 -	3422 -	-	+	2	2	4
3334	3334	3337 +	4470 -	-	+	2	2	4
3342	3342	3345 -	3499 -	-	-	2	2	4
3367	3363	3371 -	3453 -	-	-	2	2	4
3387	3385	3390 -	3572 -	-	-	2	2	4
3387	3385	3390 -	3586 -	-	-	2	2	4
3403	3399	3406 -	3585	3585	3586 -	2	2	4
3403	3399	3406 -	11894 -	-	-	0	4	4
3411	3407	3414 -	3588	3586	3588 -	2	2	4
3419	3419	3422 -	3580 -	-	-	2	2	4
3435	3432	3435 +	3454 -	-	-	2	2	4
3441	3438	3444 -	3492 -	-	-	2	2	4
3453	3453	3454 +	3435	3435	3436 -	2	2	4
3515	3512	3519 -	3699	3697	3699 -	2	2	4
3515	3512	3519 -	5264 -	-	-	2	2	4
3518	3517	3522 +	3495 -	-	-	2	2	4
3518	3517	3522 +	3543	3542	3543 +	2	2	4
3530	3529	3534 -	4111	4107	4111 -	2	2	4
3545	3541	3549 -	3615	3612	3615 +	2	2	4
3564	3560	3564 -	3585 -	-	+	2	2	4
3574	3570	3578 -	5643	5639	5643 -	2	2	4
3630	3629	3630 +	5673 -	-	+	2	2	4
3631	3628	3632 -	3863 -	-	-	2	2	4
3636	3633	3640 +	3585 -	-	-	2	2	4
3654	3651	3654 -	3722	3719	3722 +	2	2	4
3673	3670	3676 -	3814 -	-	-	2	2	4
3681	3677	3684 -	8101	8099	8101 -	4	0	4
3681	3677	3684 -	10396 -	-	+	2	2	4
3690	3686	3695 -	3841	3838	3841 -	2	2	4
3690	3687	3693 +	3728 -	-	+	2	2	4
3724	3724	3727 +	3680 -	-	-	2	2	4
3727	3727	3731 -	3769 -	-	+	2	2	4
3739	3736	3741 +	3758 -	-	+	2	2	4

3746	3743	3746 -	3883 -	-	+	2	2	4
3752	3747	3752 -	3893 -	-	-	2	2	4
3761	3757	3764 -	3884 -	-	+	2	2	4
3766	3762	3769 +	3878	3878	3881 -	2	2	4
3794 -	-	-	3944 -	-	-	2	2	4
3798	3797	3800 +	3837 -	-	+	2	2	4
3803	3802	3803 +	3839 -	-	+	2	2	4
3810	3807	3810 +	3914 -	-	-	2	2	4
3851	3845	3856 +	4000 -	-	-	2	2	4
3851	3845	3856 +	11009 -	-	+	2	2	4
3868	3864	3868 -	4013	4013	4014 -	2	2	4
3868	3864	3868 -	4097 -	-	-	2	2	4
3920	3920	3928 -	4117 -	-	-	2	2	4
3984	3981	3987 -	4210	4210	4211 -	2	2	4
4009	4005	4011 +	3936 -	-	+	2	2	4
4009	4005	4011 +	4047 -	-	-	2	2	4
4023	4019	4023 -	4055 -	-	-	4	0	4
4028 -	-	-	4060 -	-	-	0	4	4
4041 -	-	-	4078 -	-	+	2	2	4
4058	4055	4063 -	4165 -	-	-	2	2	4
4075	4071	4075 +	12614 -	-	-	0	4	4
4082	4081	4086 +	4099	4099	4100 -	2	2	4
4103	4103	4106 +	4144	4144	4146 +	2	2	4
4128	4124	4128 +	4055	4055	4059 -	2	2	4
4133	4129	4133 -	4307 -	-	-	2	2	4
4222	4218	4225 -	4376	4376	4379 -	2	2	4
4224	4223	4225 +	4206	4206	4208 -	2	2	4
4258	4255	4260 +	4288 -	-	-	2	2	4
4264	4260	4266 -	4682 -	-	-	2	2	4
4289	4287	4289 -	4414 -	-	-	0	4	4
4295	4294	4295 -	4538	4538	4539 +	2	2	4
4395 -	-	+	4428 -	-	+	2	2	4
4404	4404	4406 +	4512 -	-	+	2	2	4
4437	4435	4438 +	5312 -	-	+	2	2	4
4451	4451	4452 -	4624 -	-	-	2	2	4
4501	4496	4502 -	4534 -	-	+	2	2	4
4517	4513	4521 -	4590 -	-	-	2	2	4
4535	4535	4540 -	4662 -	-	+	2	2	4
4535	4535	4540 -	4682 -	-	-	2	2	4
4544	4544	4547 -	4591	4588	4591 -	2	2	4
4559	4556	4559 -	4682	4678	4682 -	2	2	4
4566	4564	4571 -	4622 -	-	+	0	4	4
4566	4564	4571 -	4628 -	-	+	4	0	4
4572	4570	4577 +	4819	4819	4820 -	2	2	4
4592	4589	4592 -	4723 -	-	-	2	2	4
4600 -	-	-	4673 -	-	+	2	2	4
4626	4626	4629 -	4794 -	-	+	2	2	4
4637	4637	4642 -	4682	4678	4682 -	2	2	4
4641	4638	4645 +	4772	4770	4772 -	2	2	4
4648	4647	4649 -	4819 -	-	-	2	2	4
4660	4660	4661 -	4988 -	-	-	2	2	4
4692 -	-	+	4669 -	-	-	2	2	4
4723	4719	4728 +	4737	4737	4738 -	2	2	4
4723	4719	4728 +	4749 -	-	-	2	2	4
4734	4734	4739 +	4660 -	-	-	2	2	4
4734	4734	4739 +	4798 -	-	-	2	2	4
4772	4772	4777 +	4671 -	-	-	2	2	4
4822	4822	4824 +	5011 -	-	-	2	2	4
4836	4833	4838 -	4966 -	-	-	2	2	4
4907	4906	4907 +	4827 -	-	-	2	2	4
4927	4923	4929 -	5251 -	-	-	2	2	4
4940	4936	4944 -	4981	4981	4982 -	2	2	4
4971	4971	4974 -	5313 -	-	+	2	2	4
4976	4976	4979 -	5013 -	-	-	2	2	4
4983 -	-	+	4929 -	-	-	2	2	4
4996	4996	4998 +	5014	5014	5015 -	2	2	4
5017	5014	5021 -	5029	5028	5029 +	2	2	4
5029	5025	5034 -	5036 -	-	+	4	0	4
5041 -	-	+	5110 -	-	-	2	2	4
5053	5049	5055 -	5224 -	-	+	2	2	4
5055	5053	5058 +	4996 -	-	-	2	2	4
5096	5092	5099 -	5191 -	-	-	4	0	4
5096	5092	5099 -	15065	15063	15065 +	0	4	4
5101	5101	5106 -	5281 -	-	-	2	2	4
5110	5108	5110 +	5041 -	-	-	2	2	4
5140 -	-	-	6689 -	-	+	4	0	4
5147	5143	5150 +	5025 -	-	-	2	2	4
5162	5161	5165 -	5315	5315	5316 -	2	2	4
5170	5167	5171 -	5257	5257	5258 -	2	2	4
5170	5167	5171 -	5304	5302	5304 -	2	2	4
5181	5178	5184 -	5540	5538	5540 -	2	2	4
5190	5188	5190 -	5459 -	-	+	2	2	4
5233	5230	5238 +	5294 -	-	-	2	2	4
5236	5233	5238 -	5263 -	-	+	2	2	4
5253	5253	5257 +	5119 -	-	-	2	2	4
5261	5258	5267 -	5283	5283	5288 +	1	3	4
5261	5258	5267 -	5521	5521	5522 -	2	2	4
5277	5273	5281 -	5428	5428	5429 -	2	2	4
5277	5273	5281 -	5809 -	-	-	2	2	4
5307	5307	5312 +	5498	5496	5498 +	2	2	4
5313	5309	5317 -	5375 -	-	+	2	2	4
5320	5320	5321 +	5295 -	-	-	2	2	4
5320	5320	5321 +	5587 -	-	-	2	2	4
5332	5331	5335 +	5488 -	-	-	2	2	4
5339 -	-	+	5386 -	-	+	2	2	4
5344	5344	5345 -	5398 -	-	+	2	2	4
5344	5344	5345 -	5486 -	-	-	2	2	4
5344	5344	5346 +	5335	5332	5336 -	2	2	4

5344	5344	5346 +	5398 -	-	-	2	2	4
5371	5368	5373 -	5476 -	-	+	2	2	4
5371	5368	5373 -	5486 -	-	-	2	2	4
5371	5368	5373 -	5783 -	-	-	2	2	4
5379 -	-	-	5467 -	-	+	2	2	4
5398	5398	5400 +	5443	5443	5444 -	2	2	4
5443	5443	5447 -	5593	5591	5593 -	2	2	4
5450	5450	5452 -	5555	5553	5555 +	2	2	4
5459	5457	5461 -	5548 -	-	+	2	2	4
5459	5457	5461 -	5660	5660	5661 -	2	2	4
5467	5462	5470 -	5647	5647	5649 -	2	2	4
5476	5471	5479 -	5585 -	-	-	2	2	4
5476	5471	5479 -	5665	5665	5668 -	2	2	4
5476	5471	5479 -	9575 -	-	+	0	4	4
5504	5500	5507 -	5669	5669	5670 -	2	2	4
5563	5563	5566 -	5682 -	-	-	2	2	4
5652	5651	5653 -	5788 -	-	-	2	2	4
5667	5663	5669 -	5764	5761	5764 +	2	2	4
5678	5678	5683 -	5780	5776	5780 -	2	2	4
5701	5700	5705 -	5821	5821	5822 -	2	2	4
5710	5707	5710 -	5798 -	-	+	2	2	4
5715 -	-	-	5843 -	-	-	2	2	4
5771	5771	5775 -	5897 -	-	-	2	2	4
5794	5791	5798 -	5973 -	-	+	2	2	4
5815	5811	5819 -	12720 -	-	+	2	2	4
5845	5842	5845 -	5910 -	-	+	2	2	4
5867	5867	5869 +	5878 -	-	+	2	2	4
5921	5917	5922 -	6079	6077	6079 -	2	2	4
5971	5967	5974 +	5951 -	-	-	2	2	4
5971	5967	5974 +	6016	6016	6017 -	2	2	4
5976	5976	5979 -	6125	6125	6126 -	2	2	4
6048	6047	6051 +	6027	6024	6027 -	2	2	4
6077	6073	6078 +	6238	6238	6239 -	2	2	4
6105 -	-	-	6286 -	-	-	2	2	4
6172 -	-	+	6231 -	-	-	2	2	4
6229	6229	6233 +	6555 -	-	-	2	2	4
6255	6252	6257 -	6277 -	-	+	2	2	4
6255	6252	6257 -	6334 -	-	-	2	2	4
6273	6270	6275 -	6458 -	-	-	2	2	4
6273	6273	6274 +	6373	6373	6374 -	2	2	4
6292	6288	6292 -	6368 -	-	+	1	3	4
6304 -	-	+	6463 -	-	-	2	2	4
6308	6308	6312 -	6452 -	-	-	1	3	4
6342	6338	6346 -	7094 -	-	-	2	2	4
6351	6347	6352 +	6367	6367	6368 +	2	2	4
6383	6383	6384 +	6345 -	-	-	2	2	4
6390	6385	6390 +	6414	6414	6416 -	2	2	4
6435	6434	6438 -	6573	6573	6574 -	2	2	4
6435	6434	6438 -	6600	6596	6600 -	2	2	4
6488	6487	6488 -	6505	6504	6505 +	4	0	4
6506 -	-	-	6620 -	-	-	2	2	4
6550	6549	6550 +	11168 -	-	-	3	1	4
6618	6614	6619 +	6898 -	-	+	2	2	4
6691	6689	6691 -	6899 -	-	-	2	2	4
6700	6696	6700 -	6897	6897	6898 -	2	2	4
6720 -	-	+	7672 -	-	-	2	2	4
6726	6722	6727 +	6755	6755	6756 +	2	2	4
6732	6732	6734 -	7391 -	-	-	2	2	4
6767	6767	6768 -	6962	6962	6963 -	2	2	4
6850	6846	6853 +	6786 -	-	+	2	2	4
6896	6894	6896 -	7078	7074	7078 -	2	2	4
6903 -	-	+	6886	6886	6887 -	2	2	4
6935	6934	6939 -	7029	7029	7030 +	2	2	4
6956	6953	6960 +	6986 -	-	-	2	2	4
6976	6972	6979 -	7006 -	-	-	2	2	4
7001	6997	7006 -	7155 -	-	-	2	2	4
7051	7051	7055 -	7230	7230	7231 -	2	2	4
7061	7058	7064 -	7411 -	-	-	2	2	4
7088	7081	7091 -	7216	7213	7216 -	2	2	4
7088	7081	7091 -	7229	7229	7230 -	2	2	4
7088	7081	7091 -	7281 -	-	-	2	2	4
7088	7081	7091 -	7328 -	-	-	2	2	4
7106	7103	7110 -	7247	7247	7250 -	2	2	4
7123	7120	7124 -	7254	7254	7255 +	2	2	4
7126	7123	7129 +	7148 -	-	-	1	3	4
7126	7123	7129 +	7148	7145	7148 +	2	2	4
7126	7123	7129 +	7168 -	-	-	2	2	4
7135	7134	7137 -	7286	7286	7287 -	2	2	4
7143	7143	7146 +	7268 -	-	-	4	0	4
7148	7147	7150 +	7126 -	-	-	4	0	4
7192	7192	7194 +	7173	7173	7175 -	2	2	4
7221	7220	7223 +	8006 -	-	-	2	2	4
7234	7233	7237 +	7307 -	-	+	2	2	4
7238	7235	7240 -	7436 -	-	-	2	2	4
7253	7250	7258 -	7367	7367	7368 -	2	2	4
7253	7250	7258 -	7380	7376	7380 -	2	2	4
7258	7254	7258 +	7226 -	-	-	2	2	4
7285	7281	7289 -	7454 -	-	-	2	2	4
7332	7332	7336 +	7389	7389	7390 -	2	2	4
7352 -	-	+	7387 -	-	-	2	2	4
7360	7359	7361 +	7422 -	-	-	2	2	4
7388	7385	7390 +	7332	7332	7333 -	2	2	4
7415	7414	7419 +	7581 -	-	-	2	2	4
7420	7418	7426 -	7662 -	-	-	2	2	4
7459	7457	7460 -	7537 -	-	+	2	2	4
7491	7491	7494 -	7634	7634	7635 -	2	2	4
7538	7533	7541 -	7696 -	-	-	2	2	4
7544	7542	7546 -	7716 -	-	-	2	2	4

7574	7570	7575	-	7610	7610	7611	+	2	2	4
7591	7587	7596	-	7750	7749	7754	-	2	2	4
7591	7587	7596	-	8024	8020	8024	-	3	1	4
7591	7587	7596	-	8101	8101	8102	+	2	2	4
7601	7599	7606	-	7799	7795	7799	-	2	2	4
7612	7608	7617	-	7777	-	-	-	2	2	4
7612	7608	7617	-	7793	-	-	+	2	2	4
7657	7653	7658	-	7806	-	-	-	2	2	4
7665	7665	7668	-	7820	-	-	-	2	2	4
7678	7676	7678	-	7864	-	-	-	2	2	4
7683	7680	7686	-	7843	-	-	-	2	2	4
7686	7684	7689	+	7702	-	-	+	2	2	4
7714	7710	7715	-	7763	-	-	+	2	2	4
7714	7710	7715	-	7803	7800	7803	-	2	2	4
7720	-	-	-	7782	-	-	-	2	2	4
7733	7731	7733	-	7862	-	-	-	2	2	4
7748	7744	7750	-	8313	8313	8314	-	2	2	4
7780	7776	7780	-	7997	-	-	-	2	2	4
7796	7794	7796	-	7857	7855	7857	+	2	2	4
7800	7797	7804	+	8064	8064	8065	-	2	2	4
7809	7809	7812	+	11141	-	-	+	0	4	4
7828	7826	7828	+	7804	-	-	-	2	2	4
7835	7832	7836	-	7996	-	-	-	2	2	4
7835	7832	7836	-	8028	-	-	-	2	2	4
7858	7857	7860	-	7991	7989	7991	-	2	2	4
7858	7857	7860	-	8030	8030	8031	-	2	2	4
7896	7892	7898	-	7913	-	-	+	2	2	4
7896	7892	7898	-	7944	-	-	+	2	2	4
7896	7892	7898	-	8177	-	-	-	2	2	4
7896	7894	7896	+	8029	8029	8030	-	2	2	4
7961	7960	7961	+	8109	8109	8110	-	2	2	4
7962	7959	7967	-	8042	-	-	+	2	2	4
7962	7959	7967	-	8081	-	-	-	2	2	4
7962	7959	7967	-	8202	8198	8202	-	2	2	4
7979	7975	7982	+	7864	-	-	-	2	2	4
7992	7987	7996	-	8153	8150	8153	+	2	2	4
8004	7999	8007	-	8179	8176	8179	-	2	2	4
8012	8009	8012	-	8124	8122	8124	+	2	2	4
8012	8009	8012	-	8178	-	-	-	2	2	4
8012	8009	8012	-	8210	-	-	-	2	2	4
8027	8023	8027	-	8096	8094	8096	+	2	2	4
8036	8036	8039	-	8209	8206	8209	-	2	2	4
8044	8041	8048	-	8218	-	-	-	2	2	4
8053	8053	8055	-	8104	-	-	+	2	2	4
8060	8058	8064	-	8193	-	-	-	2	2	4
8066	8065	8067	-	8238	-	-	-	2	2	4
8071	8071	8073	-	8225	8225	8226	-	2	2	4
8084	8083	8088	-	8123	-	-	+	2	2	4
8084	8083	8088	-	8238	8237	8238	-	2	2	4
8084	8083	8088	-	8260	8258	8260	-	2	2	4
8084	8083	8088	-	8376	8376	8377	-	2	2	4
8093	8090	8095	-	8176	8174	8176	+	2	2	4
8127	8125	8127	+	8006	8004	8006	-	2	2	4
8130	8129	8134	-	8285	-	-	-	2	2	4
8156	8153	8157	-	8220	-	-	+	2	2	4
8171	-	-	+	8092	-	-	-	2	2	4
8184	8180	8184	-	8257	-	-	+	2	2	4
8218	8216	8219	+	8184	8184	8185	-	2	2	4
8244	8240	8245	-	8379	8379	8382	-	2	2	4
8244	8240	8245	-	8400	-	-	-	2	2	4
8305	8305	8306	+	8263	-	-	-	2	2	4
8386	8382	8388	-	8445	-	-	-	2	2	4
8431	8429	8432	+	8442	-	-	-	3	1	4
8438	8434	8439	-	8490	-	-	+	2	2	4
8445	8441	8447	-	8559	8559	8560	-	2	2	4
8445	8441	8447	-	8589	-	-	-	2	2	4
8445	8441	8447	-	8611	8611	8612	-	2	2	4
8445	8441	8447	-	8621	8621	8622	-	2	2	4
8484	8480	8487	-	8670	-	-	-	2	2	4
8497	8496	8501	-	8519	8515	8522	+	2	2	4
8526	8523	8526	+	8603	-	-	+	2	2	4
8629	-	-	+	8973	-	-	-	2	2	4
8631	8628	8636	-	8667	-	-	+	2	2	4
8631	8628	8636	-	8820	-	-	-	2	2	4
8641	8640	8642	-	8859	8857	8859	-	2	2	4
8649	8648	8652	-	8706	8706	8707	-	2	2	4
8690	8687	8695	-	8846	-	-	-	2	2	4
8696	-	-	+	8662	-	-	-	2	2	4
8755	8752	8755	-	8923	-	-	-	2	2	4
8757	-	-	+	8698	8698	8699	-	2	2	4
8765	8764	8769	+	8731	-	-	-	2	2	4
8825	8825	8828	-	8961	8961	8962	-	2	2	4
8825	8825	8828	-	9011	-	-	-	2	2	4
8828	8827	8830	+	9414	-	-	-	2	2	4
8855	8852	8861	-	9008	9008	9011	-	2	2	4
8884	8879	8885	+	8928	-	-	-	2	2	4
8923	8918	8927	-	13649	13649	13652	+	2	2	4
8929	8928	8929	+	8971	-	-	-	2	2	4
8946	8942	8950	-	9091	9091	9095	+	2	2	4
8950	8947	8951	+	9066	9063	9066	-	2	2	4
8959	8959	8960	+	8925	-	-	-	2	2	4
8963	8958	8969	-	9124	9124	9125	-	2	2	4
8963	8958	8969	-	9137	9137	9139	-	2	2	4
8963	8958	8969	-	9265	-	-	-	2	2	4
8978	8974	8981	-	9024	-	-	+	2	2	4
8982	8980	8983	+	9029	9029	9030	-	2	2	4
9008	9003	9009	-	9210	9210	9211	-	2	2	4
9014	-	-	-	9162	-	-	+	2	2	4

9019		9019	9021	+	8970		8970	8971	-	2	2	4
9068		9064	9073	-	9183		9182	9185	+	2	2	4
9129		9126	9132	-	9236	-	-		-	2	2	4
9179		9176	9179	-	9416	-	-		-	2	2	4
9185		9181	9190	-	9303	-	-		-	2	2	4
9194		9192	9200	-	9242	-	-		+	4	0	4
9194		9192	9200	-	9338	-	-		-	2	2	4
9204		9203	9205	-	9235	-	-		+	0	4	4
9231		9231	9237	-	9430		9430	9432	-	2	2	4
9241	-	-		+	9412	-	-		-	0	4	4
9247	-	-		+	9406	-	-		-	4	0	4
9256		9256	9260	+	9240		9240	9241	-	2	2	4
9259		9259	9262	-	9310	-	-		+	2	2	4
9273		9269	9275	-	9335		9335	9336	-	2	2	4
9278	-	-		-	9413	-	-		-	2	2	4
9291		9287	9292	-	9421		9421	9424	-	2	2	4
9291		9287	9292	-	9502		9502	9503	-	2	2	4
9305		9304	9308	-	9470		9468	9470	-	2	2	4
9305		9304	9308	-	9475		9475	9479	-	2	2	4
9305		9304	9308	-	9607	-	-		-	2	2	4
9305		9305	9306	+	9435		9435	9436	-	2	2	4
9310	-	-		+	9259	-	-		-	2	2	4
9314		9309	9315	-	9469		9469	9470	-	2	2	4
9323		9319	9326	-	9407		9403	9407	-	2	2	4
9323		9319	9326	-	9585	-	-		-	4	0	4
9328		9328	9330	-	9590	-	-		-	0	4	4
9340		9338	9342	+	9695		9695	9696	-	2	2	4
9341		9337	9341	-	9483		9483	9484	-	2	2	4
9350		9347	9354	+	9359		9359	9360	-	2	2	4
9370		9366	9370	-	9404	-	-		+	4	0	4
9414		9410	9417	+	9473		9470	9473	-	2	2	4
9441	-	-		+	9399	-	-		-	2	2	4
9442		9442	9444	-	9527	-	-		+	2	2	4
9451		9446	9456	-	9572		9569	9572	-	2	2	4
9451		9446	9456	-	9612		9612	9615	-	2	2	4
9466		9461	9468	-	9693	-	-		-	2	2	4
9486		9482	9491	-	9508		9508	9512	+	2	2	4
9499		9497	9502	-	9635	-	-		-	2	2	4
9499		9497	9502	-	9651		9651	9652	-	2	2	4
9499		9497	9502	+	9763	-	-		-	2	2	4
9515		9511	9515	-	9565		9562	9565	+	2	2	4
9523		9519	9523	+	9748	-	-		-	2	2	4
9530		9527	9531	-	9626	-	-		+	2	2	4
9536		9536	9541	-	9730		9730	9731	+	2	2	4
9564		9560	9564	+	9638	-	-		-	2	2	4
9569		9569	9571	+	9637	-	-		-	2	2	4
9571		9570	9573	-	9613		9612	9615	+	2	2	4
9574		9573	9577	+	9643		9641	9643	-	2	2	4
9597		9593	9599	+	9611	-	-		-	2	2	4
9597		9593	9599	+	9665	-	-		+	2	2	4
9597		9593	9599	+	9683		9683	9684	-	2	2	4
9600	-	-		-	9784	-	-		-	2	2	4
9604		9604	9605	+	9673	-	-		-	2	2	4
9611	-	-		+	9622	-	-		-	2	2	4
9616	-	-		+	9574	-	-		-	3	1	4
9635		9635	9638	+	9569	-	-		-	2	2	4
9680		9679	9681	-	9699		9699	9700	+	3	1	4
9686		9686	9689	-	9898	-	-		-	2	2	4
9698		9696	9701	-	9775	-	-		+	2	2	4
9723	-	-		+	9653	-	-		-	2	2	4
9738		9734	9738	+	9530	-	-		-	3	1	4
9738		9734	9738	+	9640	-	-		-	2	2	4
9773		9772	9777	-	10040		10040	10041	-	2	2	4
9782		9779	9786	-	12430	-	-		+	4	0	4
9788		9787	9791	-	10270	-	-		-	2	2	4
9829		9829	9833	-	9937	-	-		-	2	2	4
9881		9879	9885	-	10070		10068	10070	-	2	2	4
9929		9929	9934	-	10032		10032	10033	+	2	2	4
9939		9938	9942	-	10023		10023	10024	+	2	2	4
9947	-	-		+	14116	-	-		+	2	2	4
9948		9943	9953	-	14118		14117	14119	-	0	4	4
9953		9953	9956	+	10010		10010	10011	-	2	2	4
9961		9960	9963	-	10002		10001	10003	+	2	2	4
9988	-	-		-	9976	-	-		+	4	0	4
9994		9993	9996	+	10055	-	-		-	2	2	4
10004	-	-		+	9956	-	-		-	4	0	4
10009		10008	10012	+	9955	-	-		-	0	4	4
10012		10011	10016	-	10216	-	-		+	2	2	4
10031		10027	10031	-	10096	-	-		+	2	2	4
10031		10027	10031	-	10178		10174	10178	-	2	2	4
10036		10033	10039	-	10010	-	-		+	4	0	4
10045		10042	10049	-	10004	-	-		+	2	2	4
10045		10042	10049	-	10172		10172	10173	-	2	2	4
10045		10042	10049	-	10213		10210	10213	-	2	2	4
10057		10054	10059	+	9993	-	-		-	2	2	4
10097		10094	10097	-	10370	-	-		-	2	2	4
10146		10142	10149	-	10209		10209	10210	-	2	2	4
10154	-	-		-	10398	-	-		-	2	2	4
10162		10159	10166	-	10325	-	-		-	2	2	4
10186		10184	10186	+	10550		10550	10551	+	2	2	4
10208		10206	10210	-	10721	-	-		-	2	2	4
10217		10215	10221	-	10353		10350	10353	-	2	2	4
10245		10243	10249	-	10386		10382	10386	-	2	2	4
10260	-	-		-	10471	-	-		-	2	2	4
10271		10266	10274	-	10325		10321	10325	+	2	2	4
10271		10266	10274	-	10409		10405	10410	-	2	2	4
10271		10266	10274	-	10427		10427	10428	-	2	2	4
10281		10278	10284	-	10341		10341	10344	+	2	2	4

10281	10278	10284 -	10356 -	-	+	2	2	4
10286	10285	10291 -	10442	10442	10443 -	2	2	4
10302	10299	10305 -	10488	10484	10488 -	2	2	4
10307	10306	10307 -	10399 -	-	-	2	2	4
10316	10313	10319 +	10252	10252	10253 -	2	2	4
10344	10344	10345 +	10330	10330	10331 -	2	2	4
10364	10361	10366 -	10527 -	-	-	2	2	4
10381	10378	10384 -	10553 -	-	-	2	2	4
10395	10393	10398 -	10554	10552	10554 -	2	2	4
10395	10393	10398 -	10600	10600	10603 -	2	2	4
10397 -	-	+	10348 -	-	-	2	2	4
10403	10400	10407 -	13047 -	-	+	0	4	4
10414	10408	10417 -	10589	10585	10589 +	2	2	4
10420 -	-	-	10611 -	-	-	2	2	4
10425	10422	10426 -	10585 -	-	-	2	2	4
10425	10422	10426 -	10629	10626	10629 -	2	2	4
10431	10431	10432 -	10613	10612	10613 -	2	2	4
10436	10435	10437 -	10609	10609	10610 -	2	2	4
10441	10441	10442 -	10410 -	-	+	2	2	4
10451	10447	10451 -	10648 -	-	+	2	2	4
10461	10459	10463 -	10636	10636	10637 +	2	2	4
10472	10468	10476 -	10632	10630	10632 -	2	2	4
10485	10483	10487 -	10521	10521	10522 +	2	2	4
10583	10579	10586 -	10677 -	-	+	2	2	4
10583	10579	10586 -	10770	10770	10771 -	2	2	4
10591	10589	10592 -	10735 -	-	-	2	2	4
10591	10589	10592 -	10761 -	-	-	2	2	4
10591	10589	10592 -	10814	10814	10815 -	2	2	4
10609	10609	10610 +	10515 -	-	-	2	2	4
10616	10614	10620 -	10687	10683	10687 +	3	1	4
10657	10653	10660 +	10834 -	-	-	2	2	4
10665	10662	10666 -	10699 -	-	+	2	2	4
10694	10693	10695 -	10826 -	-	-	2	2	4
10697	10696	10697 +	10725 -	-	-	2	2	4
10710	10708	10713 +	10725 -	-	+	2	2	4
10711	10710	10711 -	10758	10758	10759 +	3	1	4
10750 -	-	-	10889 -	-	+	2	2	4
10757	10755	10758 -	10902 -	-	-	2	2	4
10823	10821	10823 -	10980 -	-	-	2	2	4
10830	10830	10831 +	10861	10861	10862 -	2	2	4
10845	10845	10847 +	10804	10804	10805 -	2	2	4
10873	10871	10876 -	11016 -	-	-	2	2	4
10884	10884	10886 -	11040 -	-	-	2	2	4
10915 -	-	-	11557 -	-	+	2	2	4
10952	10952	10954 -	11141	11137	11141 -	2	2	4
10983	10983	10986 +	11057 -	-	+	2	2	4
11005	11004	11009 -	11238 -	-	-	2	2	4
11054	11052	11054 +	11030	11030	11032 -	2	2	4
11069 -	-	-	11260	11258	11260 -	2	2	4
11082	11079	11085 -	11155	11152	11155 +	2	2	4
11091	11091	11092 +	11192	11192	11193 -	2	2	4
11102	11099	11105 -	11284 -	-	+	2	2	4
11110	11106	11110 +	11127	11123	11127 -	2	2	4
11127	11123	11130 -	11260	11257	11260 -	2	2	4
11137	11137	11141 -	11335	11334	11335 -	2	2	4
11178	11178	11180 +	11124	11122	11124 -	2	2	4
11186	11186	11192 -	11281 -	-	-	2	2	4
11200	11197	11203 -	13462 -	-	-	2	2	4
11210	11210	11211 -	11448 -	-	-	2	2	4
11222	11220	11226 -	11399	11395	11399 -	2	2	4
11252	11249	11255 -	11363 -	-	+	2	2	4
11252	11249	11255 -	11380 -	-	-	2	2	4
11252	11249	11255 -	11414 -	-	-	2	2	4
11269	11265	11274 -	11400	11400	11401 -	2	2	4
11269	11265	11274 -	11406	11406	11407 -	2	2	4
11269	11265	11274 -	11449	11449	11451 -	2	2	4
11282	11278	11286 -	14929 -	-	+	2	2	4
11286	11283	11286 +	11319	11316	11319 -	2	2	4
11306	11302	11309 -	11441	11441	11442 -	2	2	4
11318	11313	11321 -	11386 -	-	+	4	0	4
11318	11313	11321 -	11412 -	-	-	2	2	4
11318	11313	11321 -	11427	11427	11430 -	2	2	4
11318	11313	11321 -	11432	11432	11433 -	2	2	4
11318	11313	11321 -	11501	11501	11504 -	2	2	4
11323 -	-	-	11381 -	-	+	0	4	4
11329	11326	11333 -	11340 -	-	+	4	0	4
11329	11326	11333 -	11538 -	-	-	2	2	4
11342	11337	11344 -	11446	11444	11446 -	1	3	4
11342	11337	11344 -	11482	11479	11482 -	2	2	4
11343 -	-	+	11330 -	-	-	2	2	4
11381	11381	11383 +	11372 -	-	-	2	2	4
11385	11385	11387 -	11538 -	-	-	2	2	4
11385	11385	11387 -	11574	11572	11574 -	2	2	4
11398	11398	11403 +	11342 -	-	-	2	2	4
11404	11400	11407 -	11587	11585	11587 -	2	2	4
11433	11431	11433 -	11590 -	-	-	2	2	4
11439	11436	11440 -	11587	11585	11587 -	2	2	4
11462	11458	11463 -	11603 -	-	+	2	2	4
11479	11476	11483 +	11583 -	-	-	2	2	4
11494	11493	11497 -	14253 -	-	+	2	2	4
11501	11498	11504 -	11560 -	-	+	2	2	4
11519	11519	11521 -	11548 -	-	+	2	2	4
11526	11523	11529 -	11547	11543	11547 +	2	2	4
11526	11523	11529 -	11615 -	-	-	2	2	4
11526	11523	11529 -	11717 -	-	-	2	2	4
11548	11544	11551 -	11613 -	-	+	2	2	4
11560	11556	11563 -	11717 -	-	-	2	2	4
11573	11573	11579 -	11717	11717	11718 -	2	2	4

11581	11581	11584	-	11703	11703	11704	-	2	2	4
11583	11579	11586	+	11478	11478	11479	-	2	2	4
11592	11588	11593	-	11612 -	-		+	2	2	4
11608	11604	11610	-	11716 -	-		-	2	2	4
11622	11618	11624	-	11727 -	-		+	2	2	4
11622	11618	11624	-	11829 -	-		-	2	2	4
11622	11618	11624	-	11835	11835	11838	-	2	2	4
11630 -	-		-	11801 -	-		-	2	2	4
11636 -	-		-	11692 -	-		+	2	2	4
11669	11665	11669	-	11888 -	-		-	4	0	4
11675	11675	11678	-	11894 -	-		-	0	4	4
11698	11697	11703	-	11922	11922	11923	-	2	2	4
11706 -	-		+	11768 -	-		-	2	2	4
11716 -	-		+	11894 -	-		-	2	2	4
11726	11722	11730	+	11938 -	-		-	2	2	4
11755	11753	11758	+	11768 -	-		+	2	2	4
11755	11753	11758	+	12134 -	-		-	2	2	4
11759	11759	11760	-	12460	12460	12461	-	2	2	4
11767	11766	11767	-	11919	11919	11920	-	2	2	4
11792	11787	11796	-	12696 -	-		+	2	2	4
11865	11863	11866	+	11790	11790	11791	-	2	2	4
11881	11877	11886	-	12007	12007	12009	-	2	2	4
11881	11877	11886	-	12015	12015	12017	-	2	2	4
11881	11877	11886	-	12072	12068	12072	-	2	2	4
11881	11877	11886	-	12100 -	-		-	2	2	4
11894	11894	11895	+	11972	11972	11973	-	2	2	4
11898	11898	11899	-	12068	12068	12069	-	2	2	4
11905	11901	11907	-	12051 -	-		-	2	2	4
11950	11948	11952	+	11927 -	-		-	2	2	4
11967	11966	11967	+	12090 -	-		-	2	2	4
11968	11968	11972	-	12145	12143	12145	-	2	2	4
11972	11971	11972	+	12084	12084	12085	-	2	2	4
11976	11974	11976	-	12130 -	-		-	2	2	4
11981	11980	11981	-	12104 -	-		-	2	2	4
12008	12006	12014	-	12196	12196	12199	-	2	2	4
12027	12027	12031	+	12123 -	-		-	2	2	4
12029	12027	12031	-	12210	12210	12211	-	2	2	4
12047	12044	12051	-	12238 -	-		-	2	2	4
12055	12054	12055	+	12212 -	-		-	2	2	4
12059	12059	12064	-	12113 -	-		+	2	2	4
12082	12078	12086	+	11973	11973	11974	-	2	2	4
12085	12081	12086	-	12227 -	-		-	2	2	4
12090	12090	12091	-	12123 -	-		+	2	2	4
12096	12093	12099	-	12179 -	-		+	2	2	4
12096	12093	12099	-	12226	12226	12227	-	2	2	4
12096	12093	12099	-	12239	12239	12240	-	2	2	4
12096	12093	12099	-	12256	12256	12257	-	2	2	4
12096	12093	12099	-	12279 -	-		-	2	2	4
12107	12105	12107	-	12227 -	-		-	2	2	4
12107	12105	12107	-	12263 -	-		-	2	2	4
12126	12121	12130	-	12084	12084	12086	+	4	0	4
12146	12143	12150	+	12172	12172	12176	+	4	0	4
12159	12158	12162	+	14650	14650	14651	-	2	2	4
12185	12182	12185	-	12324 -	-		+	4	0	4
12191	12187	12195	-	12317 -	-		+	0	4	4
12209	12206	12209	+	13858 -	-		-	2	2	4
12220	12217	12220	+	12346 -	-		+	2	2	4
12222	12219	12225	-	12298 -	-		+	4	0	4
12228	12227	12231	-	12295 -	-		+	0	4	4
12228	12227	12231	-	12336	12333	12336	+	2	2	4
12242	12241	12243	-	12417	12415	12417	-	2	2	4
12248	12248	12252	-	12312	12310	12312	+	2	2	4
12248	12248	12252	-	12370	12370	12371	-	2	2	4
12258	12257	12260	-	12486	12486	12487	-	2	2	4
12326	12323	12330	-	12535 -	-		-	2	2	4
12366	12364	12367	-	12591 -	-		-	2	2	4
12371	12371	12372	-	12428	12428	12429	+	3	1	4
12412	12412	12415	-	12576 -	-		-	2	2	4
12462 -	-		+	12530 -	-		-	2	2	4
12502	12502	12506	-	12598 -	-		-	2	2	4
12502	12502	12506	-	12625 -	-		+	2	2	4
12513	12513	12515	-	12626	12626	12627	-	2	2	4
12522	12519	12522	-	12624 -	-		-	2	2	4
12536	12535	12537	-	12661	12661	12662	-	2	2	4
12542	12540	12542	+	12603 -	-		-	2	2	4
12549	12549	12550	-	12595	12595	12596	+	2	2	4
12557	12555	12560	+	12517	12513	12517	+	2	2	4
12557	12555	12560	+	12580	12579	12580	-	3	1	4
12557	12555	12560	+	12661 -	-		-	2	2	4
12571	12570	12575	-	12618	12614	12618	-	2	2	4
12584	12584	12585	-	12668 -	-		-	2	2	4
12607	12603	12608	-	12783	12783	12784	-	2	2	4
12608 -	-		+	12460 -	-		-	2	2	4
12668	12664	12669	-	12744	12740	12744	+	2	2	4
12671	12669	12671	+	12738	12738	12739	-	2	2	4
12682	12682	12683	-	12769 -	-		-	4	0	4
12686	12685	12686	+	12713	12713	12714	-	2	2	4
12689	12688	12692	-	12776 -	-		-	0	4	4
12689	12688	12692	-	12795 -	-		-	2	2	4
12694 -	-		+	12831 -	-		-	2	2	4
12698	12698	12703	-	13153 -	-		-	2	2	4
12707	12704	12711	-	12750 -	-		+	2	2	4
12721	12720	12724	-	12819	12816	12819	+	2	2	4
12731 -	-		+	12644 -	-		-	2	2	4
12739	12739	12743	-	12962 -	-		-	2	2	4
12751	12747	12755	-	12915 -	-		-	2	2	4
12751	12747	12755	-	12920 -	-		-	2	2	4
12751	12747	12755	-	12950	12950	12953	-	2	2	4

12753	-	-	+	13003	-	-	+	2	2	4
12767	12763	12771	-	12926	-	-	-	2	2	4
12778	12774	12779	-	12927	-	-	-	2	2	4
12782	12782	12787	+	12947	-	-	-	2	2	4
12782	12782	12787	+	13029	-	-	+	2	2	4
12786	12783	12788	-	12968	12965	12968	-	2	2	4
12791	12791	12794	-	12959	-	-	-	2	2	4
12791	12791	12794	-	12986	-	-	-	2	2	4
12797	12797	12799	-	12986	12982	12986	-	2	2	4
12804	12804	12806	-	12852	-	-	+	2	2	4
12814	12810	12818	-	12931	-	-	-	2	2	4
12814	12810	12818	-	13320	-	-	-	2	2	4
12837	12833	12841	-	13162	-	-	+	2	2	4
12884	12881	12884	-	13052	-	-	-	2	2	4
12891	12889	12894	-	13029	-	-	-	2	2	4
12903	12903	12905	-	13061	13060	13061	-	2	2	4
13016	13012	13016	-	13344	-	-	+	2	2	4
13024	13018	13028	-	13131	-	-	+	2	2	4
13024	13018	13028	-	13214	13213	13215	-	2	2	4
13024	13018	13028	-	13219	-	-	-	2	2	4
13031	13030	13032	-	13214	13214	13215	-	2	2	4
13039	13034	13040	-	13164	-	-	-	2	2	4
13070	13069	13071	+	13014	-	-	-	2	2	4
13072	13069	13077	-	13201	13201	13205	-	2	2	4
13072	13069	13077	-	13217	13215	13217	-	2	2	4
13072	13069	13077	-	13255	13255	13256	-	2	2	4
13082	13078	13085	+	13024	13024	13028	-	2	2	4
13089	13085	13089	-	13218	13215	13218	-	2	2	4
13104	13101	13106	-	13056	-	-	+	4	0	4
13106	13102	13107	+	13200	-	-	-	2	2	4
13141	13141	13145	-	13340	13340	13341	-	2	2	4
13152	-	-	+	13226	-	-	-	2	2	4
13175	13174	13176	+	13078	13078	13080	-	2	2	4
13175	13174	13176	+	13131	13131	13134	-	2	2	4
13200	13198	13203	-	13387	13386	13388	-	2	2	4
13210	13206	13211	+	13236	-	-	-	2	2	4
13210	13206	13211	+	13247	-	-	-	2	2	4
13212	13208	13216	-	13360	13357	13360	-	2	2	4
13212	13208	13216	-	13380	13378	13380	-	2	2	4
13234	13234	13237	+	13320	13319	13321	-	2	2	4
13243	-	-	-	13313	-	-	+	2	2	4
13248	13245	13250	+	13533	-	-	-	2	2	4
13285	13283	13287	-	13506	13503	13506	-	2	2	4
13290	13290	13293	-	13334	-	-	+	2	2	4
13311	13308	13313	-	13355	-	-	-	2	2	4
13319	-	-	+	13237	13237	13238	-	2	2	4
13334	13332	13335	+	13918	13918	13919	+	2	2	4
13338	13335	13341	-	13394	-	-	-	4	0	4
13338	13335	13341	-	13400	-	-	-	0	4	4
13338	13335	13341	-	13477	-	-	-	2	2	4
13372	13372	13377	+	13362	13358	13362	-	2	2	4
13373	13371	13373	-	13520	-	-	-	2	2	4
13389	13389	13392	+	13422	-	-	-	2	2	4
13445	-	-	-	13614	-	-	-	2	2	4
13450	13449	13454	-	13664	-	-	-	2	2	4
13476	-	-	-	13710	13708	13710	-	2	2	4
13498	13498	13502	-	13648	-	-	+	2	2	4
13500	13500	13502	+	13701	-	-	-	2	2	4
13518	13514	13520	+	13685	13685	13686	-	2	2	4
13538	13534	13542	-	13774	-	-	-	2	2	4
13556	13555	13556	-	13637	-	-	-	2	2	4
13562	13559	13565	-	13784	13780	13784	-	2	2	4
13580	13577	13580	-	13788	13785	13788	-	2	2	4
13600	13596	13605	-	13804	-	-	-	2	2	4
13658	13658	13662	-	13632	-	-	+	4	0	4
13658	13658	13662	-	13782	-	-	-	2	2	4
13658	13658	13662	-	13824	-	-	-	2	2	4
13658	13658	13662	-	14035	-	-	-	2	2	4
13679	13677	13683	-	13895	-	-	-	2	2	4
13704	13702	13707	-	13882	-	-	+	2	2	4
13716	13716	13719	+	13834	-	-	+	2	2	4
13726	-	-	-	13758	-	-	+	2	2	4
13733	13733	13734	-	13933	-	-	-	2	2	4
13743	-	-	+	13764	-	-	-	2	2	4
13745	13743	13748	-	13890	-	-	+	2	2	4
13745	13743	13748	-	13915	13915	13919	-	1	3	4
13745	13743	13748	-	14971	14971	14972	-	2	2	4
13752	13751	13755	-	14053	-	-	+	1	3	4
13762	13760	13763	+	13849	-	-	-	2	2	4
13763	13759	13763	-	13962	-	-	-	2	2	4
13839	13837	13840	-	13939	13939	13941	+	3	1	4
13839	13837	13840	-	13984	-	-	-	2	2	4
13849	13848	13849	+	13800	-	-	-	2	2	4
13861	13858	13864	-	13939	13939	13940	+	2	2	4
13861	13858	13864	-	13994	13994	13995	-	3	1	4
13875	13874	13880	+	13869	-	-	+	2	2	4
13876	13875	13877	-	13998	-	-	+	2	2	4
13894	13891	13894	-	14073	-	-	-	2	2	4
13925	13921	13927	-	14042	-	-	-	2	2	4
13948	13945	13952	-	14166	14163	14166	-	2	2	4
13948	13945	13952	-	14388	-	-	-	2	2	4
13967	13965	13970	-	14094	-	-	-	2	2	4
13975	13972	13975	-	14185	14185	14186	-	2	2	4
13988	13985	13991	-	14113	-	-	-	2	2	4
13988	13985	13991	-	14138	-	-	-	2	2	4
14002	14001	14004	-	14083	14081	14083	-	2	2	4
14018	-	-	+	14003	-	-	-	2	2	4
14027	14027	14029	-	14197	14195	14197	-	2	2	4

14042	14038	14042 -	14201	14201	14202 -	2	2	4
14062	14059	14062 -	14204	14204	14205 -	2	2	4
14085	14081	14086 -	14206 -	-	-	2	2	4
14085	14081	14086 -	14305	14305	14306 -	2	2	4
14091 -	-	-	14255 -	-	-	4	0	4
14132	14129	14134 +	14200	14196	14200 +	2	2	4
14143	14142	14145 -	14532 -	-	+	2	2	4
14168	14165	14171 -	14327 -	-	-	2	2	4
14168	14165	14171 -	14373	14373	14374 -	2	2	4
14177	14172	14178 -	14304 -	-	-	2	2	4
14177	14172	14178 -	14334 -	-	-	2	2	4
14177	14172	14178 -	14343	14340	14343 -	2	2	4
14177	14177	14180 +	14112	14112	14113 -	2	2	4
14182	14180	14184 -	14283 -	-	-	2	2	4
14184	14183	14188 +	14107	14107	14108 -	2	2	4
14184	14183	14188 +	14112 -	-	-	2	2	4
14204	14199	14208 +	14233 -	-	-	2	2	4
14207	14204	14208 -	14283 -	-	-	2	2	4
14215	14211	14216 -	14367 -	-	+	2	2	4
14231	14230	14233 -	14449 -	-	+	2	2	4
14236 -	-	-	14268 -	-	+	4	0	4
14241 -	-	-	14263 -	-	+	0	4	4
14243	14237	14246 +	14179	14179	14180 -	2	2	4
14251	14248	14255 +	14186 -	-	-	2	2	4
14301	14297	14306 -	14372 -	-	+	2	2	4
14301	14297	14306 -	14484	14484	14485 -	2	2	4
14309	14308	14309 -	14515 -	-	-	2	2	4
14314	14311	14314 -	14373 -	-	-	2	2	4
14324	14322	14326 -	14790 -	-	-	2	2	4
14332	14332	14337 -	14465 -	-	-	2	2	4
14341	14338	14344 -	14463	14463	14465 -	2	2	4
14341	14338	14344 -	14480 -	-	-	2	2	4
14341	14338	14344 -	14505	14503	14506 -	2	2	4
14341	14338	14344 -	14511	14510	14511 -	2	2	4
14341	14338	14344 -	15235	15235	15238 -	2	2	4
14346	14345	14347 -	14467 -	-	-	2	2	4
14369	14366	14371 -	14324 -	-	+	4	0	4
14369	14366	14372 +	14324 -	-	-	2	2	4
14391	14386	14395 -	14512	14509	14512 -	2	2	4
14409	14404	14411 -	14563	14559	14563 -	3	1	4
14421 -	-	-	14472 -	-	+	2	2	4
14421 -	-	-	14541 -	-	+	2	2	4
14435	14435	14440 -	14551 -	-	-	2	2	4
14453	14452	14454 -	14490	14487	14490 +	3	1	4
14508	14507	14508 +	14651 -	-	-	2	2	4
14515	14513	14519 +	14583	14583	14585 -	2	2	4
14540	14536	14543 -	14644 -	-	-	3	1	4
14540	14536	14543 -	14747	14747	14750 -	2	2	4
14542	14539	14547 +	14515 -	-	-	2	2	4
14554	14551	14558 -	14699 -	-	-	2	2	4
14554	14551	14558 -	14715 -	-	-	2	2	4
14554	14551	14558 -	14726 -	-	-	2	2	4
14554	14551	14558 -	14750	14750	14754 -	2	2	4
14566	14563	14568 -	14793	14791	14793 -	2	2	4
14577	14573	14577 -	14956 -	-	+	4	0	4
14583	14579	14587 -	14717 -	-	-	2	2	4
14583	14579	14587 -	14954 -	-	+	0	4	4
14592	14589	14596 -	14792 -	-	-	2	2	4
14614	14614	14615 -	14685	14684	14685 +	2	2	4
14638	14634	14640 +	14651	14647	14651 -	2	2	4
14642	14638	14643 -	14768	14768	14772 -	2	2	4
14646	14645	14648 +	14634	14634	14635 -	2	2	4
14647	14645	14651 -	14794	14791	14794 -	2	2	4
14647	14645	14651 -	14821	14818	14821 -	2	2	4
14653	14653	14655 -	14676	14675	14676 +	2	2	4
14667	14662	14670 -	14737 -	-	+	2	2	4
14687 -	-	+	14640 -	-	-	2	2	4
14706	14704	14710 -	14754	14750	14754 +	2	2	4
14728	14726	14731 -	14872 -	-	+	2	2	4
14750	14750	14752 -	14809	14807	14809 +	2	2	4
14770	14766	14771 -	14793	14790	14793 +	2	2	4
14770	14766	14771 -	15028	15028	15029 -	2	2	4
14786	14783	14789 -	14939 -	-	-	2	2	4
14791	14790	14795 -	14863	14859	14863 +	2	2	4
14802	14801	14806 +	14835 -	-	+	2	2	4
14847	14847	14850 +	15044 -	-	-	2	2	4
14859	14855	14860 +	14770	14770	14771 -	2	2	4
14861	14859	14862 -	14898 -	-	+	4	0	4
14875	14871	14877 -	15001	14998	15001 -	2	2	4
14875	14871	14877 -	15024	15024	15025 -	2	2	4
14875	14871	14877 -	15064	15064	15068 -	2	2	4
14878 -	-	+	15015 -	-	-	2	2	4
14891	14890	14891 -	15048 -	-	-	2	2	4
14897	14892	14900 -	14965 -	-	+	2	2	4
14897	14892	14900 -	15086 -	-	-	2	2	4
14897	14892	14900 -	15152	15148	15152 +	2	2	4
14920	14920	14923 +	14992	14992	14993 +	2	2	4
14938	14937	14941 -	15000	15000	15001 +	3	1	4
14951	14950	14955 -	14986 -	-	+	2	2	4
14960	14959	14964 -	15247 -	-	-	2	2	4
14972	14968	14974 -	15108	15104	15108 -	2	2	4
14972	14968	14974 -	15155 -	-	+	2	2	4
14972	14968	14974 -	15161 -	-	-	2	2	4
14979	14978	14983 -	15183	15181	15183 -	2	2	4
14979	14978	14983 -	15335	15332	15336 -	2	2	4
14996	14993	14996 -	15204 -	-	-	2	2	4
15002	14998	15003 -	15147	15147	15148 -	2	2	4
15002	14998	15003 -	15194 -	-	-	2	2	4

15017	15016	15020 -	15095	15093	15095 +	2	2	4
15017	15016	15020 -	15170	15168	15170 -	2	2	4
15025	15024	15028 -	15167	15167	15168 -	2	2	4
15025	15022	15025 +	15138 -	-	-	2	2	4
15032	15032	15035 +	15156 -	-	-	2	2	4
15037	15032	15038 -	15123	15123	15124 +	2	2	4
15067	15066	15067 -	15258 -	-	-	2	2	4
15072	15069	15076 -	15184 -	-	+	2	2	4
15072	15069	15076 -	15211 -	-	+	2	2	4
15079	15077	15082 -	15185 -	-	-	2	2	4
15095	15093	15095 -	15310	15308	15310 -	2	2	4
15104	15100	15108 -	15133 -	-	+	2	2	4
15114	15112	15117 -	15146 -	-	+	2	2	4
15114	15112	15117 -	15234	15234	15235 -	2	2	4
15124	15121	15124 -	15238	15236	15238 -	2	2	4
15129	15126	15130 -	15303	15303	15305 -	2	2	4
15144	15141	15146 -	15209 -	-	+	2	2	4
15144	15141	15146 -	15366 -	-	-	2	2	4
15150	15150	15154 -	15325	15322	15325 -	2	2	4
15169	15167	15169 -	15323	15321	15323 -	2	2	4
15211	15211	15212 -	15290 -	-	+	2	2	4
15241	15239	15245 +	15256 -	-	-	2	2	4
15241	15239	15245 +	15263 -	-	+	2	2	4
15251	15247	15251 +	15188 -	-	-	2	2	4
15251	15247	15251 +	15269 -	-	+	2	2	4
15263	15262	15266 +	15255	15252	15255 -	2	2	4
15289	15289	15290 +	15307	15307	15308 -	2	2	4
15314	15310	15314 +	15283	15283	15287 -	2	2	4
15346	15346	15347 +	15362	15362	15363 -	2	2	4
1	2	8 -	234	233	234 -	1	2	3
59	57	60 -	94 -	-	+	3	0	3
67	62	70 -	88 -	-	+	0	3	3
93	90	95 -	123	121	123 +	2	1	3
93	90	95 -	173 -	-	-	2	1	3
173 -	-	+	93 -	-	+	3	0	3
224	223	229 -	246 -	-	+	3	0	3
242	240	245 -	321	321	324 -	2	1	3
242	240	245 -	412	412	415 -	2	1	3
265 -	-	-	15122 -	-	+	2	1	3
265	262	267 +	15123	15121	15124 -	1	2	3
277	274	278 +	15110 -	-	-	2	1	3
314	312	314 +	377 -	-	-	0	3	3
320	316	325 +	499 -	-	-	2	1	3
351	347	352 -	515 -	-	-	2	1	3
369	366	374 -	410 -	-	+	2	1	3
438	438	439 -	621 -	-	-	1	2	3
483	480	487 -	613 -	-	-	2	1	3
544 -	-	-	592 -	-	-	3	0	3
550	548	553 -	599 -	-	-	0	3	3
552	550	552 +	386 -	-	-	1	2	3
561	556	565 -	665 -	-	-	2	1	3
584	582	585 -	592 -	-	+	3	0	3
663	661	666 -	634 -	-	+	3	0	3
663	661	666 -	859	856	859 -	1	2	3
700	696	704 +	799 -	-	-	0	3	3
710	707	710 +	794 -	-	-	3	0	3
797	795	799 -	9101	9100	9101 -	3	0	3
809	809	812 +	908 -	-	-	2	1	3
814	813	817 -	940	939	940 -	1	2	3
849	847	853 +	670 -	-	-	1	2	3
853	851	858 -	1024	1024	1025 -	1	2	3
865	863	868 -	1034 -	-	-	1	2	3
881	880	886 -	12715 -	-	-	0	3	3
913	908	917 +	810 -	-	-	2	1	3
960	956	964 -	1123	1122	1123 -	2	1	3
981	980	981 -	1180 -	-	-	3	0	3
991	988	995 +	853 -	-	-	1	2	3
1092	1087	1094 +	1070	1070	1072 -	1	2	3
1126	1123	1129 -	1324	1324	1326 -	1	2	3
1179	1176	1183 -	1281 -	-	-	1	2	3
1202	1199	1207 -	1399	1399	1401 -	2	1	3
1238	1235	1239 -	1183	1180	1184 +	1	2	3
1244	1240	1248 -	5961 -	-	+	0	3	3
1306	1303	1310 +	1167 -	-	-	1	2	3
1375	1373	1379 -	1559 -	-	+	1	2	3
1418	1414	1422 -	8237	8237	8238 -	1	2	3
1452	1447	1452 -	2238 -	-	-	2	1	3
1499	1499	1501 +	1456 -	-	-	3	0	3
1508	1504	1511 +	1551 -	-	+	0	3	3
1513	1509	1517 -	1577 -	-	-	3	0	3
1513	1512	1520 +	1558 -	-	+	3	0	3
1532	1529	1536 +	1473	1471	1473 -	1	2	3
1537	1537	1540 -	1552 -	-	+	1	2	3
1537	1537	1540 -	1688	1686	1688 -	3	0	3
1544	1541	1548 -	1608 -	-	+	0	3	3
1544	1541	1548 -	1613 -	-	+	3	0	3
1559	1558	1562 -	1690	1686	1690 -	1	2	3
1618	1614	1620 -	1720	1717	1720 +	1	2	3
1642	1640	1643 -	1696 -	-	+	0	3	3
1655	1652	1661 -	1785	1785	1786 -	3	0	3
1748	1745	1750 -	1946	1943	1947 -	1	2	3
1772	1772	1776 -	1779 -	-	+	0	3	3
1790	1786	1792 -	2005	2005	2007 -	1	2	3
1905	1902	1905 +	1901	1900	1901 +	2	1	3
1930	1928	1930 +	1942 -	-	+	3	0	3
1939	1935	1943 -	2104	2101	2104 -	1	2	3
2001	2000	2001 +	2226 -	-	-	2	1	3
2004	2000	2004 -	2176	2176	2177 -	1	2	3

2031	2026	2031	-	2048	-	-		+	3	0	3
2036	2035	2039	-	12514		12514	12515	-	1	2	3
2036	2036	2039	+	12516		12516	12518	+	2	1	3
2106	2106	2110	-	2132	-	-		+	0	3	3
2140	2137	2143	-	2275	-	-		-	1	2	3
2174	2170	2176	-	2324	-	-		-	3	0	3
2181	2179	2181	-	2329	-	-		-	0	3	3
2245	2244	2245	-	2438	-	-		+	2	1	3
2281	2279	2282	-	2693		2689	2693	-	2	1	3
2288	2283	2292	-	2495	-	-		-	2	1	3
2367	2367	2372	-	2523		2519	2523	-	2	1	3
2405	2402	2409	-	5476	-	-		+	3	0	3
2484	2480	2485	-	2525		2525	2526	-	1	2	3
2503	2498	2507	-	2510	-	-		+	3	0	3
2503	2498	2507	-	12082	-	-		-	3	0	3
2518	2514	2523	-	2762		2758	2762	-	1	2	3
2518	2514	2523	-	2825		2824	2825	+	1	2	3
2532	2532	2535	-	2666		2666	2668	-	1	2	3
2542	2536	2542	-	2668	-	-		-	2	1	3
2573	2573	2577	+	2506		2506	2508	-	2	1	3
2602	2601	2607	+	2588		2585	2588	-	2	1	3
2631	2631	2632	+	2558		2557	2558	-	2	1	3
2662	2657	2664	-	2674		2674	2676	+	1	2	3
2662	2657	2664	-	2679	-	-		+	2	1	3
2662	2657	2664	-	2802	-	-		-	2	1	3
2721	2721	2727	-	2830		2828	2830	+	2	1	3
2744	2744	2745	-	2776	-	-		+	3	0	3
2750	2748	2750	-	2773	-	-		+	0	3	3
2762	2762	2765	-	2936	-	-		-	3	0	3
2767	2767	2769	-	2941	-	-		-	0	3	3
2972	2968	2977	-	3294		3291	3294	-	2	1	3
3055	3051	3056	+	3077	-	-		-	1	2	3
3091	3088	3095	-	3322		3318	3326	-	2	1	3
3136	3136	3141	-	3115	-	-		+	3	0	3
3229	3228	3233	-	3385	-	-		-	1	2	3
3273	3270	3274	+	3209	-	-		+	0	3	3
3274	3271	3279	-	3420	-	-		+	1	2	3
3289	3285	3290	+	3396	-	-		-	1	2	3
3291	3290	3296	-	3569	-	-		-	0	3	3
3403	3399	3406	-	3552	-	-		-	1	2	3
3403	3399	3406	-	3580	-	-		-	1	2	3
3434	3433	3436	-	3503		3500	3503	+	2	1	3
3441	3438	3444	-	3447		3447	3448	+	3	0	3
3525	3522	3528	-	3531		3531	3534	+	3	0	3
3545	3541	3549	-	3694		3694	3695	-	2	1	3
3706	3705	3713	-	3871		3871	3872	+	1	2	3
3761	3757	3764	-	3774	-	-		+	3	0	3
3774	3774	3777	-	3758	-	-		+	3	0	3
4064	4060	4067	+	4116		4116	4117	-	1	2	3
4084	4083	4087	-	4248		4246	4249	-	1	2	3
4140	4139	4141	+	12080		12080	12082	-	3	0	3
4204	4202	4204	+	4167		4167	4169	-	1	2	3
4247	4246	4251	+	4354	-	-		-	0	3	3
4251	4250	4252	-	4354		4354	4355	+	3	0	3
4257	4256	4257	-	4348	-	-		+	0	3	3
4334	4334	4336	-	4354	-	-		+	2	1	3
4348 -	-		+	4257	-	-		-	0	3	3
4354 -	-		+	4251	-	-		-	3	0	3
4404	4400	4404	-	4555		4553	4555	-	2	1	3
4425	4424	4428	+	6931		6931	6932	+	2	1	3
4437	4434	4437	-	10721	-	-		-	3	0	3
4566	4564	4571	-	4785		4784	4785	-	2	1	3
4582	4578	4586	-	13657	-	-		-	3	0	3
4586 -	-		+	13657	-	-		+	2	1	3
4610	4609	4615	-	4633	-	-		+	1	2	3
4632	4629	4636	+	4575		4575	4576	-	1	2	3
4723	4719	4728	+	5883		5883	5884	+	2	1	3
4759	4758	4759	+	5403		5403	5404	-	1	2	3
4792	4791	4793	-	9790		9790	9791	+	2	1	3
4827	4827	4830	+	4907	-	-		-	1	2	3
4831	4830	4831	-	4991	-	-		-	3	0	3
4836	4833	4838	-	4978		4976	4979	-	2	1	3
4836	4833	4838	-	4993	-	-		-	0	3	3
4859	4859	4863	-	4959		4955	4959	+	1	2	3
4859	4857	4859	+	4877	-	-		-	1	2	3
4932	4931	4932	-	4883		4883	4884	+	2	1	3
4988	4987	4992	+	5028		5028	5030	+	1	2	3
5017	5014	5021	-	5141		5141	5145	-	2	1	3
5046	5043	5047	-	5259	-	-		-	1	2	3
5053	5049	5055	-	15107	-	-		+	2	1	3
5069	5065	5069	+	15096		15093	15097	-	1	2	3
5078	5076	5082	-	5241		5241	5243	-	2	1	3
5087	5084	5087	-	15077	-	-		+	3	0	3
5096	5092	5099	-	5228		5225	5228	-	1	2	3
5147	5143	5150	+	6685		6685	6686	-	0	3	3
5155	5150	5156	-	6588		6587	6588	-	0	3	3
5162	5161	5165	-	5286	-	-		-	1	2	3
5181	5178	5184	-	5370	-	-		-	2	1	3
5290	5288	5290	+	5328	-	-		-	2	1	3
5374	5373	5377	+	5357		5355	5358	-	2	1	3
5476	5471	5479	-	14194	-	-		-	0	3	3
5617	5613	5619	+	5592		5592	5593	-	2	1	3
5647	5643	5647	-	5684		5684	5686	+	2	1	3
5658	5657	5662	-	5775	-	-		+	2	1	3
5658	5657	5662	-	5835		5835	5839	-	1	2	3
5710	5707	5710	-	5863	-	-		-	2	1	3
5875	5871	5875	+	5908	-	-		-	1	2	3
5926	5926	5930	-	6105		6105	6109	-	3	0	3

5960	-	-	-	6097	-	-	-	2	1	3
6308	6308	6312	-	6404	-	-	-	3	0	3
6481	6481	6483	-	12280	-	-	-	3	0	3
6506	-	-	-	6487	-	-	+	3	0	3
6506	-	-	-	6573	-	-	-	2	1	3
6538	6535	6539	-	6603	-	-	+	3	0	3
6550	6549	6550	+	6554	-	-	-	1	2	3
6558	6556	6561	+	11161	-	-	-	3	0	3
6580	6577	6580	-	6756	-	-	-	2	1	3
6634	-	-	-	6711	-	-	-	2	1	3
6650	6647	6653	+	6684	6684	6685	-	1	2	3
6684	-	-	-	6650	-	-	+	3	0	3
6813	6809	6818	+	6931	6930	6931	-	2	1	3
6857	6852	6858	-	6998	-	-	-	1	2	3
6882	6878	6883	+	6820	6819	6820	-	2	1	3
6935	6934	6939	-	7114	7110	7114	-	1	2	3
6954	6953	6957	-	7125	7122	7125	-	1	2	3
6988	6988	6993	-	7127	7124	7127	-	2	1	3
7088	7081	7091	-	7161	-	-	-	1	2	3
7088	7081	7091	-	7243	7242	7243	-	2	1	3
7088	7081	7091	-	7345	7345	7347	-	3	0	3
7106	7103	7110	-	7277	7277	7279	-	1	2	3
7106	7103	7110	-	7288	7287	7288	-	2	1	3
7106	7103	7110	-	7294	7294	7295	-	1	2	3
7135	7134	7137	-	7361	7359	7362	-	2	1	3
7148	7147	7150	+	7267	7264	7267	-	1	2	3
7253	7250	7258	-	7291	7288	7291	+	2	1	3
7258	7254	7258	+	7287	7287	7289	-	1	2	3
7326	-	-	+	7470	-	-	-	2	1	3
7461	7457	7462	+	7472	7472	7476	-	1	2	3
7469	7465	7472	+	7531	-	-	-	2	1	3
7538	7533	7541	-	7670	7669	7670	-	1	2	3
7544	7542	7546	-	7660	-	-	+	2	1	3
7544	7542	7546	-	7954	-	-	-	2	1	3
7554	7554	7558	-	7715	7714	7715	-	1	2	3
7583	7583	7586	-	7802	7802	7804	-	2	1	3
7591	7587	7596	-	7799	7799	7802	-	2	1	3
7670	7669	7673	-	9369	9368	9369	+	1	2	3
7932	7932	7934	-	8000	7997	8000	+	2	1	3
7940	7936	7941	-	8202	8198	8202	-	2	1	3
7945	7944	7950	-	8030	8030	8034	-	1	2	3
7962	7959	7967	-	7981	-	-	+	2	1	3
7979	7975	7982	+	7956	7953	7956	-	1	2	3
7992	7987	7996	-	8201	8201	8202	-	2	1	3
8036	8036	8039	-	8086	8084	8087	+	2	1	3
8053	8053	8055	-	8177	8173	8177	-	1	2	3
8084	8083	8088	-	8280	8280	8282	+	2	1	3
8118	8114	8118	-	8164	-	-	+	2	1	3
8278	8278	8282	+	8230	-	-	-	2	1	3
8371	-	-	+	11563	-	-	+	1	2	3
8386	8382	8388	-	8465	-	-	+	0	3	3
8386	8382	8388	-	8561	8561	8564	-	2	1	3
8391	8390	8391	-	8563	8563	8566	-	1	2	3
8431	8429	8432	+	8404	-	-	-	1	2	3
8474	8470	8478	-	8501	8498	8501	+	2	1	3
8539	-	-	-	8577	-	-	+	3	0	3
8545	8545	8549	-	8571	-	-	+	0	3	3
8562	8558	8565	+	8607	8606	8607	+	2	1	3
8598	8595	8598	-	8664	-	-	+	2	1	3
8603	8603	8606	-	8778	-	-	+	3	0	3
8608	8608	8610	-	8774	-	-	+	0	3	3
8624	8624	8625	-	13809	-	-	-	0	3	3
8680	8675	8684	-	8692	-	-	+	3	0	3
8690	8687	8695	-	8680	-	-	+	3	0	3
8703	8703	8706	+	8770	8769	8770	-	2	1	3
8749	8745	8750	-	8900	-	-	-	2	1	3
8765	8764	8769	+	8712	8711	8712	-	2	1	3
8778	8775	8781	-	8940	-	-	-	3	0	3
8783	8783	8788	-	8943	-	-	-	0	3	3
8834	-	-	-	11350	-	-	+	3	0	3
8849	-	-	+	8906	-	-	-	3	0	3
8978	8974	8981	-	9064	9064	9068	+	1	2	3
8986	8983	8986	-	9028	9028	9029	+	2	1	3
9103	-	-	+	9130	-	-	-	2	1	3
9143	9140	9147	-	9278	9277	9278	-	1	2	3
9179	9176	9179	-	9236	-	-	+	3	0	3
9185	9181	9190	-	9542	9542	9543	+	2	1	3
9194	9192	9200	-	9390	9390	9391	-	1	2	3
9224	-	-	+	9297	-	-	-	2	1	3
9422	9419	9424	-	9630	-	-	-	3	0	3
9428	9428	9432	-	9636	-	-	-	0	3	3
9530	9527	9531	-	9511	-	-	+	2	1	3
9679	9677	9681	+	9644	-	-	-	0	3	3
9679	9677	9681	+	9698	9695	9699	-	1	2	3
9824	-	-	-	9894	-	-	+	3	0	3
9829	9829	9833	-	9888	-	-	+	0	3	3
9948	9943	9953	-	10090	10090	10092	-	2	1	3
9977	9976	9981	-	10193	10193	10194	-	1	2	3
10012	10011	10016	-	10155	-	-	+	1	2	3
10065	10063	10067	-	10212	-	-	-	1	2	3
10183	10178	10185	-	10204	-	-	+	0	3	3
10302	10299	10305	-	10411	10408	10411	-	1	2	3
10316	10313	10319	+	10482	-	-	-	2	1	3
10472	10468	10476	-	10619	10616	10619	-	1	2	3
10482	-	-	+	10316	-	-	-	2	1	3
10490	10490	10493	-	10587	10587	10588	-	0	3	3
10529	10525	10529	-	10609	10609	10611	-	1	2	3
10662	10662	10664	+	10672	-	-	-	3	0	3

10724	10721	10724	-	14849	14846	14850	+	2	1	3
10744	10743	10744	+	10795 -	-		+	0	3	3
10752	10748	10753	+	10803 -	-		+	3	0	3
10787	10787	10791	+	12653	12652	12653	-	2	1	3
10797	10797	10798	-	12301	12300	12301	-	0	3	3
11210	11210	11211	-	11322 -	-		+	0	3	3
11269	11265	11274	-	11357	11357	11361	+	1	2	3
11368	11366	11372	+	11438	11438	11439	-	2	1	3
11501	11498	11504	-	11539 -	-		+	0	3	3
11501	11498	11504	-	11544 -	-		+	3	0	3
11598	11597	11599	-	11608	11607	11608	+	2	1	3
11622	11618	11624	-	11810 -	-		-	2	1	3
11695	11692	11695	+	11680	11678	11681	-	1	2	3
11781 -	-		+	11875 -	-		-	2	1	3
11875 -	-		+	11781 -	-		-	2	1	3
11937 -	-		-	12088	12088	12091	-	1	2	3
11942	11938	11946	-	11993 -	-		+	1	2	3
12000	11997	12003	-	12158	12156	12158	-	2	1	3
12040	12036	12041	-	12214	12211	12214	-	2	1	3
12047	12044	12051	-	12217	12215	12217	-	1	2	3
12047	12044	12051	-	12266	12263	12266	-	2	1	3
12068	12068	12069	-	12111 -	-		+	3	0	3
12076	12071	12079	-	12107 -	-		+	0	3	3
12097	12094	12097	+	12059 -	-		-	0	3	3
12103	12103	12105	+	12053 -	-		-	3	0	3
12136	12132	12140	-	12079 -	-		+	3	0	3
12153	12153	12157	+	12173 -	-		+	0	3	3
12214	12212	12217	-	12417	12415	12417	-	2	1	3
12240	12240	12242	+	12039 -	-		-	1	2	3
12258	12257	12260	-	12415	12414	12415	-	2	1	3
12291	12290	12294	-	12486 -	-		-	1	2	3
12326	12323	12330	-	12488	12488	12490	-	1	2	3
12333	12332	12338	-	12523 -	-		+	1	2	3
12351 -	-		-	12502 -	-		+	3	0	3
12388	12387	12388	-	14344 -	-		+	1	2	3
12398	12394	12402	+	12487	12487	12491	-	2	1	3
12462 -	-		+	12438 -	-		-	1	2	3
12468	12465	12468	-	12493 -	-		+	3	0	3
12473	12472	12477	-	12488 -	-		+	0	3	3
12541	12541	12542	-	12604	12603	12604	+	2	1	3
12552 -	-		+	12474 -	-		-	0	3	3
12557	12555	12560	+	12469 -	-		-	3	0	3
12564 -	-		+	12620 -	-		-	3	0	3
12641	12635	12645	-	12745	12742	12745	-	2	1	3
12765	12765	12768	+	12802	12798	12802	-	2	1	3
12767	12763	12771	-	12903	12903	12904	-	2	1	3
12797	12797	12799	-	12967	12964	12967	-	1	2	3
12856	12854	12856	+	12834 -	-		-	1	2	3
12974	12970	12977	-	12999 -	-		+	3	0	3
12974	12974	12975	+	12999 -	-		-	1	2	3
13016	13012	13016	-	13201	13197	13201	-	2	1	3
13061	13059	13065	+	13188	13188	13189	-	2	1	3
13128	13126	13132	-	13184	13182	13184	+	2	1	3
13131	13126	13134	+	13020 -	-		-	2	1	3
13139	13135	13139	+	13175	13174	13175	-	1	2	3
13141	13141	13145	-	13307	13303	13307	-	1	2	3
13228	13228	13231	+	13334 -	-		-	0	3	3
13234	13234	13237	+	13328 -	-		-	3	0	3
13250	13250	13251	-	13286 -	-		+	2	1	3
13343	13342	13343	-	13383 -	-		+	3	0	3
13349	13344	13349	-	13377 -	-		+	0	3	3
13349	13344	13351	+	13377 -	-		-	3	0	3
13379 -	-		-	13469 -	-		-	3	0	3
13385	13384	13387	-	13474 -	-		-	0	3	3
13453	13453	13454	+	13331	13330	13331	-	1	2	3
13538	13534	13542	-	13620	13620	13621	+	2	1	3
13570	13569	13572	-	13785	13783	13785	-	1	2	3
13640 -	-		+	13518 -	-		-	1	2	3
13679	13677	13683	-	13720 -	-		+	3	0	3
13704	13702	13707	-	13894	13894	13897	-	2	1	3
13733	13733	13734	-	13911 -	-		-	2	1	3
13763	13759	13763	-	13849 -	-		+	3	0	3
13772	13767	13772	-	13844 -	-		+	0	3	3
13772	13769	13776	+	13829 -	-		-	2	1	3
13861	13858	13864	-	13886	13884	13886	+	2	1	3
13870	13865	13873	-	13875 -	-		+	0	3	3
13870	13865	13873	-	14000 -	-		-	0	3	3
13875	13874	13880	+	13995	13994	13995	-	1	2	3
13876	13875	13877	-	14051 -	-		-	2	1	3
13882	13878	13882	-	14055	14055	14056	-	1	2	3
13961	13958	13964	-	14008 -	-		+	2	1	3
13982	13977	13982	-	14064	14064	14065	+	2	1	3
13996	13992	14000	-	14053 -	-		+	1	2	3
14022	14018	14022	-	14284 -	-		-	3	0	3
14048	14046	14051	-	14250	14246	14250	-	1	2	3
14098	14096	14102	-	14107	14107	14111	+	3	0	3
14098	14096	14102	-	14262 -	-		-	0	3	3
14151	14151	14154	-	14394 -	-		-	2	1	3
14197	14197	14201	-	14389 -	-		-	2	1	3
14210 -	-		+	14182 -	-		-	0	3	3
14215	14215	14218	+	14177 -	-		-	3	0	3
14270	14266	14273	+	14283 -	-		-	2	1	3
14332	14332	14337	-	14508	14507	14508	-	1	2	3
14341	14338	14344	-	14405 -	-		+	0	3	3
14374	14374	14377	-	14567 -	-		+	0	3	3
14503	14503	14504	+	14552	14551	14552	-	1	2	3
14613	14613	14614	+	14562	14562	14564	-	2	1	3
14626	14626	14630	-	14674 -	-		+	0	3	3

14676	14672	14680	-	14626	-	-	+	0	3	3
14687	14686	14691	-	14756	-	-	+	1	2	3
14706	14704	14710	-	14835	14835	14836	-	1	2	3
14767	14763	14769	+	14902	-	-	-	2	1	3
14866	14863	14870	-	14818	-	-	+	1	2	3
14885	14879	14888	-	14982	-	-	+	0	3	3
14885	14879	14888	-	14987	-	-	+	3	0	3
15045	15042	15046	+	15074	15074	15075	-	2	1	3
15052	15048	15055	-	15108	-	-	+	1	2	3
15053	15053	15055	+	15108	15107	15108	-	2	1	3
15095	15093	15095	-	15287	-	-	+	3	0	3
15095	-	-	+	15287	-	-	-	1	2	3
15104	15100	15108	-	15278	-	-	+	0	3	3
15117	15113	15121	+	14969	14969	14972	-	2	1	3
1	2	8	-	85	85	87	-	1	1	2
1	2	8	-	248	248	249	-	0	2	2
1	2	8	-	414	-	-	-	1	1	2
1	2	8	-	12847	-	-	+	1	1	2
14	9	18	-	143	143	144	-	1	1	2
14	9	18	-	150	-	-	-	1	1	2
14	9	18	-	208	208	209	-	1	1	2
23	19	26	-	61	59	61	+	1	1	2
23	19	26	-	97	-	-	+	1	1	2
23	19	26	-	138	-	-	-	1	1	2
23	19	26	-	232	-	-	-	1	1	2
23	19	26	-	257	-	-	-	1	1	2
30	-	-	+	7309	-	-	-	1	1	2
32	28	33	-	135	-	-	-	1	1	2
32	28	33	-	141	-	-	-	1	1	2
32	28	33	-	163	-	-	+	1	1	2
32	28	33	-	216	-	-	-	1	1	2
41	41	44	-	232	-	-	-	1	1	2
41	41	44	-	262	-	-	+	1	1	2
59	57	60	-	219	-	-	-	1	1	2
59	57	60	-	226	-	-	-	1	1	2
59	57	60	-	273	-	-	+	1	1	2
66	63	69	+	197	193	197	-	1	1	2
66	63	69	+	227	224	227	-	1	1	2
67	62	70	-	209	-	-	-	1	1	2
67	62	70	-	271	-	-	-	1	1	2
67	62	70	-	287	-	-	+	1	1	2
67	62	70	-	291	-	-	-	1	1	2
72	71	76	-	131	127	131	-	1	1	2
72	71	76	-	207	-	-	-	1	1	2
72	71	76	-	281	281	282	+	1	1	2
72	71	76	-	1465	-	-	-	1	1	2
81	78	81	-	487	-	-	-	1	1	2
86	84	88	-	191	-	-	-	1	1	2
86	84	88	-	232	-	-	-	1	1	2
86	84	88	-	248	-	-	-	1	1	2
86	84	88	-	277	-	-	-	1	1	2
86	84	88	-	353	-	-	-	1	1	2
93	90	95	-	253	-	-	-	1	1	2
93	90	95	-	276	-	-	-	1	1	2
98	94	99	+	82	-	-	-	1	1	2
99	98	99	-	250	-	-	-	1	1	2
105	102	106	+	228	-	-	+	1	1	2
105	102	106	+	578	-	-	-	1	1	2
106	102	106	-	234	-	-	-	1	1	2
120	-	-	+	197	-	-	+	1	1	2
135	-	-	+	197	-	-	+	1	1	2
139	135	142	-	185	-	-	+	1	1	2
139	135	142	-	293	289	293	-	1	1	2
139	135	142	-	517	-	-	-	1	1	2
139	135	142	-	615	-	-	-	1	1	2
142	140	145	+	93	-	-	-	1	1	2
150	147	150	-	184	-	-	+	1	1	2
150	148	154	+	107	-	-	+	1	1	2
157	156	157	-	363	-	-	-	1	1	2
163	163	167	-	290	-	-	+	1	1	2
165	165	168	+	462	-	-	-	1	1	2
173	169	176	-	208	-	-	-	1	1	2
173	169	176	-	312	-	-	-	1	1	2
173	169	176	-	353	-	-	-	1	1	2
173	169	176	-	6473	-	-	-	0	2	2
173	-	-	+	157	-	-	-	1	1	2
180	180	181	-	229	-	-	+	1	1	2
180	180	181	-	637	637	638	-	1	1	2
187	184	192	-	294	291	294	-	1	1	2
187	184	192	-	310	-	-	-	1	1	2
199	195	201	+	221	217	221	+	1	1	2
199	195	201	+	1039	1039	1040	-	1	1	2
199	195	201	+	14061	14059	14061	+	2	0	2
200	-	-	-	309	-	-	-	1	1	2
206	203	207	-	324	-	-	-	1	1	2
206	203	207	-	357	355	357	-	1	1	2
206	203	207	-	369	-	-	-	1	1	2
211	210	214	-	351	-	-	-	1	1	2
211	210	214	-	430	430	431	-	1	1	2
211	210	214	-	674	-	-	-	1	1	2
216	-	-	-	380	-	-	-	1	1	2
221	219	221	+	11714	-	-	+	1	1	2
224	223	229	-	386	386	387	-	1	1	2
224	223	229	-	394	394	395	-	1	1	2
227	224	229	+	181	-	-	-	1	1	2
227	224	229	+	283	-	-	+	1	1	2
232	231	232	-	257	-	-	+	2	0	2
237	236	238	-	362	362	363	-	1	1	2

237		236	238	-	432	-	-		-	1	1	2
237	-	-		+	251	-	-		-	1	1	2
242		240	245	-	329	-	-		+	1	1	2
242		240	245	-	369	-	-		-	1	1	2
242		240	245	-	391	-	-		-	1	1	2
242		240	245	-	441	-	-		-	1	1	2
242		240	245	-	468		468	469	-	1	1	2
251		251	252	+	330		330	331	+	1	1	2
254		251	256	-	237	-	-		+	2	0	2
254		251	256	-	383	-	-		-	1	1	2
260		260	262	-	228	-	-		+	2	0	2
260		260	262	-	371	-	-		-	1	1	2
260		260	262	-	882	-	-		-	1	1	2
260		260	262	-	4611	-	-		+	0	2	2
277		274	278	+	214	-	-		+	1	1	2
277		274	278	+	458	-	-		-	1	1	2
280		280	282	-	359	-	-		+	1	1	2
280		280	282	-	426	-	-		-	1	1	2
280		280	282	-	461	-	-		-	1	1	2
284		281	285	+	359	-	-		-	1	1	2
285		285	286	-	435	-	-		-	1	1	2
285		285	286	-	614	-	-		+	1	1	2
291		289	295	-	339		337	339	+	1	1	2
291		289	295	-	356		352	356	+	1	1	2
291		289	295	-	427	-	-		-	1	1	2
291		289	295	-	444	-	-		+	1	1	2
291		289	295	-	584		584	585	-	1	1	2
291		289	295	-	594	-	-		-	1	1	2
295		290	299	+	93	-	-		-	0	2	2
295		290	299	+	266	-	-		+	1	1	2
298		297	302	-	433	-	-		+	1	1	2
310		308	314	-	343	-	-		+	1	1	2
310		308	314	-	379	-	-		+	1	1	2
310		308	314	-	593	-	-		+	1	1	2
314		312	314	+	366	-	-		+	1	1	2
320		319	321	-	857	-	-		-	1	1	2
320		316	325	+	293	-	-		-	1	1	2
320		316	325	+	406		406	407	-	1	1	2
320		316	325	+	485	-	-		+	1	1	2
325		322	325	-	410	-	-		+	1	1	2
328		328	330	+	371	-	-		+	1	1	2
328		328	330	+	376	-	-		-	1	1	2
328		328	330	+	511		511	512	-	1	1	2
331		331	334	-	549	-	-		-	1	1	2
336	-	-		-	359	-	-		+	1	1	2
336	-	-		+	324	-	-		-	1	1	2
341		338	345	-	518	-	-		-	1	1	2
343		343	346	+	493	-	-		-	1	1	2
351		347	352	-	386		386	387	+	1	1	2
351		347	352	-	499	-	-		-	1	1	2
352		352	354	+	293	-	-		-	1	1	2
352		352	354	+	382		382	383	-	1	1	2
356		355	360	-	494	-	-		-	1	1	2
356		355	360	-	517	-	-		-	1	1	2
356		355	360	-	586	-	-		-	1	1	2
358		358	360	+	3085	-	-		+	1	1	2
362		361	362	-	583	-	-		-	1	1	2
364	-	-		+	331	-	-		-	1	1	2
369		366	374	-	472	-	-		+	1	1	2
369		366	374	-	518	-	-		-	1	1	2
369		366	374	-	543	-	-		-	1	1	2
369		366	374	-	657	-	-		-	1	1	2
369		366	374	-	954	-	-		-	1	1	2
373		373	374	+	456		456	457	-	1	1	2
379		377	379	+	312	-	-		-	1	1	2
386		386	388	-	350	-	-		+	1	1	2
386		386	388	-	576	-	-		-	1	1	2
393		391	396	+	326	-	-		-	1	1	2
395		389	399	-	385		385	389	+	2	0	2
395		389	399	-	546	-	-		+	1	1	2
395		389	399	-	590	-	-		-	2	0	2
395		389	399	-	861		861	862	-	1	1	2
401		398	402	+	305	-	-		-	1	1	2
401		398	402	+	414	-	-		-	1	1	2
408		405	410	+	448	-	-		+	1	1	2
408		405	410	+	466	-	-		+	1	1	2
408		405	410	+	1068	-	-		+	1	1	2
412		410	417	-	640	-	-		-	1	1	2
412		410	417	-	1283	-	-		-	0	2	2
418		418	421	+	15118	-	-		-	1	1	2
433		430	436	-	518		518	519	-	1	1	2
433		430	436	-	583	-	-		-	1	1	2
433		430	436	-	593	-	-		-	1	1	2
442		440	442	+	375	-	-		-	1	1	2
444		443	446	-	564	-	-		-	1	1	2
444		443	446	-	641	-	-		-	1	1	2
444		443	446	-	704	-	-		-	0	2	2
450		450	454	-	474	-	-		+	1	1	2
450		450	454	-	619	-	-		-	2	0	2
452		451	456	+	374		374	375	-	1	1	2
452		451	456	+	429	-	-		-	1	1	2
452		451	456	+	619	-	-		+	0	2	2
459		457	461	-	518	-	-		+	1	1	2
459		457	461	-	610	-	-		-	1	1	2
459		457	461	-	627	-	-		-	0	2	2
459		459	461	+	627	-	-		+	2	0	2
466	-	-		+	412	-	-		-	1	1	2
467		463	468	-	486		483	486	+	2	0	2

467		463	468	-	541	-	-		-	1	1	2
472		468	472	+	486		482	486	-	1	1	2
472		468	472	+	518	-	-		-	1	1	2
474		471	476	-	617	-	-		+	1	1	2
474		471	476	-	647	-	-		-	1	1	2
477		477	480	+	536		533	536	-	1	1	2
483		480	487	-	583	-	-		-	1	1	2
483		480	487	-	637	-	-		-	1	1	2
483		480	487	-	698	-	-		-	1	1	2
483		480	487	-	981	-	-		+	1	1	2
486		482	488	+	390		386	390	-	1	1	2
486		482	488	+	405	-	-		-	1	1	2
486		482	488	+	472		468	472	-	1	1	2
486		482	488	+	14158	-	-		-	1	1	2
492		489	495	-	697	-	-		-	1	1	2
492		489	495	-	707	-	-		-	1	1	2
497	-	-		-	695	-	-		-	1	1	2
503		498	506	-	627	-	-		+	1	1	2
503		498	506	-	639	-	-		-	1	1	2
505	-	-		+	458	-	-		-	1	1	2
509	-	-		-	696	-	-		-	1	1	2
515		510	519	-	826	-	-		-	1	1	2
520		517	521	+	472	-	-		-	1	1	2
520		517	521	+	480	-	-		-	1	1	2
521	-	-		-	620	-	-		-	1	1	2
530		526	530	-	704	-	-		-	1	1	2
530		526	530	-	726	-	-		-	1	1	2
530		526	530	-	1081	-	-		+	1	1	2
544	-	-		+	573	-	-		+	1	1	2
550		548	553	-	614	-	-		+	1	1	2
550		548	553	-	698	-	-		-	1	1	2
550		548	553	-	706	-	-		-	2	0	2
550		548	553	-	773	-	-		-	1	1	2
552		550	552	+	599	-	-		+	1	1	2
552		550	552	+	1539	-	-		-	0	2	2
555	-	-		-	709	-	-		-	0	2	2
558		554	562	+	729		729	730	-	1	1	2
558		554	562	+	1530	-	-		-	2	0	2
561		556	565	-	1213	-	-		-	0	2	2
567		567	570	-	697	-	-		-	1	1	2
567		567	570	-	804	-	-		-	1	1	2
568		564	570	+	533	-	-		-	1	1	2
568		564	570	+	585		585	586	+	1	1	2
568		564	570	+	614	-	-		+	1	1	2
574		574	577	-	712	-	-		-	1	1	2
575		573	578	+	614	-	-		-	1	1	2
575		573	578	+	635	-	-		-	1	1	2
584		582	585	-	622	-	-		+	1	1	2
584		582	585	-	678	-	-		-	1	1	2
585		583	585	+	659	-	-		-	1	1	2
585		583	585	+	686	-	-		-	1	1	2
585		583	585	+	774	-	-		-	1	1	2
591		589	593	-	616		612	616	+	1	1	2
591		589	593	-	712	-	-		-	1	1	2
591		589	593	-	897	-	-		-	1	1	2
592		592	595	+	726		726	727	-	1	1	2
597		596	600	-	679	-	-		-	1	1	2
597		596	600	-	785	-	-		-	1	1	2
597		596	600	-	817		817	818	-	1	1	2
606		602	607	-	786	-	-		-	1	1	2
606		602	607	-	815	-	-		-	1	1	2
609		605	611	+	865	-	-		-	1	1	2
611		608	615	-	843	-	-		-	1	1	2
617		615	621	+	587		587	588	-	1	1	2
620		617	624	-	701		701	702	-	1	1	2
620		617	624	-	729	-	-		-	1	1	2
620		617	624	-	1092		1092	1093	-	1	1	2
620		617	624	-	8918	-	-		+	0	2	2
635		633	637	-	651	-	-		+	1	1	2
635		633	637	-	711	-	-		-	1	1	2
635		633	637	-	5477	-	-		+	2	0	2
635		633	637	-	12627	-	-		-	1	1	2
644		638	645	-	643		639	643	+	2	0	2
644		638	645	-	659		659	660	+	0	2	2
644		638	645	-	749	-	-		-	1	1	2
644		638	645	-	785	-	-		-	1	1	2
644		638	645	-	804	-	-		-	1	1	2
644		638	645	-	817	-	-		-	1	1	2
644		638	645	-	847	-	-		-	1	1	2
644		638	645	-	863	-	-		-	1	1	2
644		640	645	+	612	-	-		-	1	1	2
649		649	653	+	11969	-	-		-	1	1	2
650	-	-		-	812	-	-		-	1	1	2
655		652	660	-	843	-	-		-	1	1	2
655		652	660	-	11852		11852	11853	+	0	2	2
659		656	659	+	640	-	-		-	0	2	2
659		656	659	+	802	-	-		-	1	1	2
663		661	666	-	810	-	-		-	1	1	2
663		661	666	-	820	-	-		-	1	1	2
663		661	666	-	850	-	-		-	1	1	2
663		661	666	-	906	-	-		-	1	1	2
665		661	666	+	794		794	795	-	1	1	2
670		669	673	-	818	-	-		-	1	1	2
670		669	673	-	838	-	-		-	1	1	2
670		669	673	-	847	-	-		-	1	1	2
670		669	673	-	907		904	907	-	1	1	2
670		670	672	+	847	-	-		-	1	1	2
685		682	686	-	1019	-	-		-	1	1	2

693	689	695 -	724 -	-	-	1	1	2
693	689	695 -	809	809	810 +	1	1	2
694 -	-	+	829 -	-	-	1	1	2
699	699	702 -	801 -	-	+	1	1	2
700	696	704 +	590	587	590 -	1	1	2
700	696	704 +	908 -	-	-	1	1	2
708	704	711 -	781 -	-	+	1	1	2
708	704	711 -	810 -	-	-	1	1	2
710	707	710 +	662 -	-	-	1	1	2
710	707	710 +	673 -	-	-	1	1	2
710	707	710 +	724 -	-	+	1	1	2
710	707	710 +	748 -	-	-	1	1	2
710	707	710 +	781 -	-	-	1	1	2
713	712	718 -	766	766	767 +	1	1	2
713	712	718 -	910 -	-	-	1	1	2
717	714	721 +	592 -	-	-	1	1	2
717	714	721 +	621 -	-	-	2	0	2
717	714	721 +	725	722	725 -	1	1	2
717	714	721 +	767	767	768 -	1	1	2
721	721	722 -	765	763	765 +	1	1	2
721	721	722 -	869	869	870 +	1	1	2
726	725	727 -	894 -	-	-	1	1	2
726	725	727 -	911 -	-	-	1	1	2
727	727	730 +	1030	1030	1031 -	1	1	2
735	731	735 -	1119	1115	1119 -	1	1	2
736	735	736 +	747 -	-	-	1	1	2
742	739	742 +	856 -	-	+	1	1	2
743	742	747 -	765 -	-	+	1	1	2
743	742	747 -	856 -	-	-	1	1	2
743	742	747 -	892 -	-	-	1	1	2
743	742	747 -	912 -	-	-	1	1	2
752	749	756 -	876 -	-	-	1	1	2
760	760	763 -	889 -	-	-	1	1	2
765	764	769 -	827	823	827 +	1	1	2
765	764	769 -	876	874	876 -	1	1	2
765	764	769 -	912 -	-	-	1	1	2
778	774	781 -	810	807	810 +	1	1	2
778	774	781 -	983	983	984 -	1	1	2
778	774	778 +	708 -	-	-	1	1	2
791	786	794 -	863	860	863 +	1	1	2
791	786	794 -	871 -	-	-	1	1	2
791	786	794 -	920 -	-	-	1	1	2
791	786	794 -	981 -	-	-	1	1	2
791	786	794 -	1007 -	-	-	1	1	2
791	786	794 -	1079	1079	1080 -	1	1	2
793	793	795 +	876 -	-	+	1	1	2
797	795	799 -	887 -	-	+	1	1	2
797	795	799 -	1298 -	-	-	1	1	2
798 -	-	+	911 -	-	+	1	1	2
807	803	811 -	870	870	871 +	1	1	2
807	803	811 -	994 -	-	-	1	1	2
809	809	812 +	649 -	-	-	0	2	2
809	809	812 +	869	869	870 -	1	1	2
814	813	817 -	962 -	-	-	1	1	2
814	813	817 -	1316 -	-	+	1	1	2
815	815	816 +	833 -	-	+	1	1	2
815	815	816 +	843 -	-	-	1	1	2
822	821	824 -	939 -	-	-	1	1	2
822	821	824 -	979 -	-	-	1	1	2
829	825	833 -	872 -	-	+	1	1	2
829	825	833 -	909 -	-	-	1	1	2
829	825	833 -	924 -	-	-	1	1	2
829	825	833 -	1297 -	-	+	1	1	2
833	832	833 +	1223 -	-	+	1	1	2
838	836	838 -	1075 -	-	-	1	1	2
838	836	838 -	1448 -	-	-	1	1	2
838	838	842 +	1344 -	-	+	1	1	2
844	840	849 -	876 -	-	+	0	2	2
844	840	849 -	909 -	-	+	1	1	2
844	840	849 -	956 -	-	-	1	1	2
844	840	849 -	962	962	963 -	1	1	2
844	840	849 -	998 -	-	-	1	1	2
844	840	849 -	1083 -	-	-	1	1	2
844	840	849 -	9146 -	-	-	1	1	2
844	843	845 +	876	876	877 -	1	1	2
849	847	853 +	815 -	-	-	1	1	2
849	847	853 +	879 -	-	-	1	1	2
849	847	853 +	991 -	-	-	1	1	2
853	851	858 -	879 -	-	+	1	1	2
853	851	858 -	978 -	-	-	1	1	2
853	851	858 -	1200 -	-	-	1	1	2
855	855	859 +	635 -	-	-	1	1	2
855	855	859 +	818 -	-	-	1	1	2
855	855	859 +	919	916	919 -	1	1	2
865	863	868 -	893 -	-	+	1	1	2
865	862	866 +	949 -	-	-	1	1	2
874	870	874 -	905 -	-	+	1	1	2
874	870	874 -	1028 -	-	-	1	1	2
879	876	879 +	970 -	-	+	1	1	2
881	880	886 -	1007 -	-	-	1	1	2
887	887	888 +	827	827	828 -	1	1	2
893	892	897 +	974 -	-	-	1	1	2
893	892	897 +	12082 -	-	-	2	0	2
900	900	906 +	913 -	-	-	1	1	2
900	900	906 +	994	994	995 -	1	1	2
907	902	907 -	976 -	-	+	1	1	2
907	902	907 -	983 -	-	+	1	1	2
907	902	907 -	1023 -	-	-	1	1	2

907	902	907 -	1030 -	-	-	1	1	2
907	902	907 -	1077 -	-	-	1	1	2
907	902	907 -	1082 -	-	-	1	1	2
907	902	907 -	1115 -	-	-	1	1	2
907	902	907 -	1133 -	-	-	2	0	2
913	908	916 -	987	984	987 +	1	1	2
913	908	916 -	1000	996	1000 +	1	1	2
913	908	917 +	842 -	-	-	1	1	2
913	908	917 +	941 -	-	-	1	1	2
913	908	917 +	957 -	-	-	1	1	2
913	908	917 +	969 -	-	+	1	1	2
924	920	928 +	959 -	-	-	1	1	2
924	920	928 +	998 -	-	+	1	1	2
924	920	928 +	1016 -	-	-	1	1	2
924	920	928 +	1134 -	-	+	1	1	2
930 -	-	+	2183 -	-	-	1	1	2
938	938	940 +	1146 -	-	+	1	1	2
939	939	940 -	1126 -	-	-	1	1	2
939	939	940 -	1344 -	-	-	1	1	2
947	947	949 -	1005	1003	1005 +	1	1	2
952	950	955 +	864	864	865 -	1	1	2
959	957	961 +	1020	1016	1020 -	1	1	2
959	957	961 +	1242	1242	1243 -	1	1	2
960	956	964 -	1021 -	-	+	1	1	2
960	956	964 -	1067 -	-	-	1	1	2
960	956	964 -	1129 -	-	-	1	1	2
960	956	964 -	1195 -	-	-	1	1	2
960	956	964 -	8933	8933	8937 -	2	0	2
970 -	-	+	1204 -	-	-	1	1	2
974	970	978 -	1005	1005	1006 +	1	1	2
974	970	978 -	1080 -	-	-	1	1	2
974	970	978 -	1123	1123	1124 -	1	1	2
974	970	978 -	1152 -	-	-	1	1	2
977	973	978 +	1125	1125	1126 +	1	1	2
977	973	978 +	1381 -	-	-	1	1	2
981	980	981 -	1004 -	-	+	2	0	2
981	980	981 -	1031 -	-	+	1	1	2
984	984	987 +	1108 -	-	+	0	2	2
987	984	992 -	1001 -	-	+	0	2	2
987	984	992 -	1014 -	-	+	1	1	2
987	984	992 -	1101 -	-	+	1	1	2
987	984	992 -	1114 -	-	-	1	1	2
987	984	992 -	1120 -	-	-	1	1	2
987	984	992 -	1160 -	-	-	1	1	2
987	984	992 -	1168 -	-	-	2	0	2
991	988	995 +	1113 -	-	+	2	0	2
995	994	998 -	1065 -	-	+	1	1	2
995	994	998 -	1100 -	-	-	1	1	2
995	994	998 -	1176	1173	1176 -	0	2	2
995	994	998 -	1273 -	-	-	1	1	2
1005	1002	1008 -	1076	1073	1076 +	1	1	2
1005	1002	1008 -	1103 -	-	-	1	1	2
1005	1002	1008 -	1111 -	-	+	1	1	2
1005	1002	1008 -	1160	1160	1161 -	1	1	2
1005	1002	1008 -	1301 -	-	-	1	1	2
1012	1012	1016 -	1103 -	-	+	1	1	2
1012	1012	1016 -	1208 -	-	-	1	1	2
1016 -	-	+	847 -	-	-	0	2	2
1021	1019	1024 +	988	988	991 -	1	1	2
1022	1018	1026 -	1065 -	-	+	0	2	2
1022	1018	1026 -	1091 -	-	+	1	1	2
1026 -	-	+	905 -	-	-	1	1	2
1026 -	-	+	2508 -	-	+	1	1	2
1031	1030	1034 -	1101	1097	1101 -	1	1	2
1031	1030	1034 -	1209 -	-	-	1	1	2
1031	1028	1034 +	993 -	-	-	1	1	2
1031	1028	1034 +	1090 -	-	+	1	1	2
1031	1028	1034 +	1101	1097	1101 +	1	1	2
1044	1041	1045 -	1086	1086	1087 -	1	1	2
1044	1041	1045 -	1363	1363	1364 -	1	1	2
1049	1047	1051 -	1064 -	-	+	1	1	2
1049	1047	1051 -	1071 -	-	+	1	1	2
1049	1047	1051 -	1347	1347	1348 -	1	1	2
1051	1047	1053 +	1051 -	-	-	1	1	2
1051	1047	1053 +	1117 -	-	+	1	1	2
1051	1047	1053 +	8209	8209	8210 -	1	1	2
1063	1063	1067 +	1031 -	-	-	1	1	2
1063	1063	1067 +	1054	1054	1055 -	1	1	2
1064	1060	1068 -	1093 -	-	-	1	1	2
1064	1060	1068 -	1413 -	-	-	1	1	2
1074	1073	1075 -	1231 -	-	-	2	0	2
1074	1073	1075 -	1249	1247	1249 -	1	1	2
1075	1074	1075 +	1118 -	-	-	1	1	2
1081	1077	1086 -	1236 -	-	-	0	2	2
1081	1077	1086 -	1254 -	-	-	1	1	2
1081	1077	1086 -	1266	1262	1266 +	1	1	2
1081	1077	1086 -	1289 -	-	-	1	1	2
1081	1077	1086 -	1306 -	-	-	1	1	2
1088 -	-	-	1222 -	-	-	1	1	2
1092	1087	1094 +	1106	1103	1106 -	1	1	2
1092	1087	1094 +	1135	1133	1135 -	1	1	2
1098	1092	1101 -	1205 -	-	-	1	1	2
1098	1092	1101 -	1265	1265	1266 -	1	1	2
1098	1092	1101 -	1296 -	-	-	1	1	2
1098	1092	1101 -	1334 -	-	-	1	1	2
1103	1103	1105 -	1244	1242	1244 +	1	1	2
1106	1104	1110 +	1199 -	-	+	1	1	2
1106	1104	1110 +	1331 -	-	-	1	1	2

1111	1108	1115 -	1224 -	-	-	1	1	2
1118	1114	1118 +	1106	1103	1106 -	1	1	2
1121	1117	1122 -	1251 -	-	-	1	1	2
1121	1117	1122 -	1302	1302	1303 -	1	1	2
1121	1117	1122 -	1451	1447	1451 -	1	1	2
1125	1121	1126 +	1061 -	-	-	1	1	2
1125	1121	1126 +	1103 -	-	-	0	2	2
1126	1123	1129 -	1258 -	-	-	1	1	2
1126	1123	1129 -	1284	1284	1285 -	1	1	2
1126	1123	1129 -	1317 -	-	-	1	1	2
1126	1123	1129 -	1331 -	-	+	1	1	2
1131	1127	1132 +	1055 -	-	-	1	1	2
1131	1127	1132 +	1100 -	-	-	2	0	2
1131	1127	1132 +	1158 -	-	-	1	1	2
1137	1134	1139 +	1299 -	-	-	1	1	2
1142 -	-	+	5892 -	-	+	0	2	2
1145	1141	1149 -	1219	1217	1219 +	1	1	2
1145	1141	1149 -	1323 -	-	+	1	1	2
1151 -	-	-	1286 -	-	-	1	1	2
1152	1152	1153 +	1137	1137	1138 -	1	1	2
1158	1155	1158 +	1243 -	-	+	1	1	2
1158	1155	1158 +	1308 -	-	-	1	1	2
1159	1156	1162 -	1251 -	-	+	1	1	2
1159	1156	1162 -	1298	1298	1299 -	1	1	2
1159	1156	1162 -	1356 -	-	-	1	1	2
1159	1156	1162 -	1378	1378	1379 -	1	1	2
1159	1156	1162 -	1393 -	-	-	1	1	2
1159	1156	1162 -	1417 -	-	-	0	2	2
1167	1163	1171 -	1309 -	-	-	1	1	2
1179	1176	1183 -	1299 -	-	-	1	1	2
1179	1176	1183 -	1306 -	-	-	1	1	2
1186	1183	1186 +	1137	1134	1137 -	1	1	2
1186	1183	1186 +	1155	1153	1155 -	1	1	2
1195	1192	1197 -	1227 -	-	+	1	1	2
1195	1192	1197 -	1285	1283	1285 +	1	1	2
1202	1199	1207 -	1269	1266	1269 -	1	1	2
1202	1199	1207 -	1324 -	-	-	1	1	2
1202	1199	1207 -	1375 -	-	-	1	1	2
1208	1208	1210 +	1209 -	-	-	1	1	2
1209 -	-	-	1385 -	-	-	1	1	2
1214	1211	1215 -	1438 -	-	+	1	1	2
1214	1211	1215 -	1447 -	-	-	1	1	2
1220	1220	1226 -	1195 -	-	+	1	1	2
1220	1220	1226 -	1277	1277	1278 +	1	1	2
1220	1220	1226 -	1381 -	-	-	1	1	2
1222 -	-	+	1131 -	-	-	1	1	2
1222 -	-	+	1207 -	-	-	1	1	2
1227 -	-	+	1437 -	-	+	1	1	2
1230	1228	1232 -	1373 -	-	-	1	1	2
1232	1232	1234 +	1424 -	-	-	1	1	2
1238	1235	1239 -	1337 -	-	-	1	1	2
1240	1237	1240 +	1238 -	-	+	1	1	2
1240	1237	1240 +	7398 -	-	-	2	0	2
1244	1240	1248 -	1405 -	-	-	1	1	2
1244	1240	1248 -	1456 -	-	-	1	1	2
1248	1245	1252 +	1172	1172	1173 -	1	1	2
1248	1245	1252 +	1367 -	-	-	1	1	2
1248	1245	1252 +	1408 -	-	-	1	1	2
1252 -	-	-	1381 -	-	-	1	1	2
1259	1254	1259 -	1329 -	-	+	1	1	2
1259	1254	1259 -	1381 -	-	-	1	1	2
1259	1254	1259 -	1431 -	-	+	1	1	2
1259 -	-	+	1234 -	-	-	1	1	2
1267	1262	1270 -	1334 -	-	+	0	2	2
1267	1262	1270 -	1368 -	-	-	1	1	2
1267	1262	1270 -	1421	1417	1421 -	0	2	2
1267	1262	1270 -	1423 -	-	+	1	1	2
1268	1265	1272 +	1334 -	-	-	1	1	2
1272	1272	1277 -	1259 -	-	+	2	0	2
1272	1272	1277 -	1315	1313	1315 +	1	1	2
1272	1272	1277 -	1343 -	-	-	0	2	2
1272	1272	1277 -	1409 -	-	-	2	0	2
1272	1272	1277 -	1598	1598	1599 -	1	1	2
1281	1280	1285 -	1324 -	-	-	1	1	2
1281	1280	1285 -	1413 -	-	-	0	2	2
1285	1282	1286 +	1327 -	-	-	1	1	2
1290	1287	1291 -	1360 -	-	-	1	1	2
1290	1287	1291 -	1368 -	-	-	1	1	2
1293	1292	1293 +	1388 -	-	+	1	1	2
1299	1296	1303 -	1348 -	-	+	1	1	2
1299	1296	1303 -	1349 -	-	-	1	1	2
1299	1296	1303 -	1368	1365	1368 +	1	1	2
1299	1296	1303 -	1377 -	-	-	1	1	2
1299	1296	1303 -	1386	1386	1387 -	1	1	2
1299	1296	1303 -	1485 -	-	+	1	1	2
1299	1298	1299 +	7570 -	-	-	1	1	2
1306	1303	1310 +	1336 -	-	+	1	1	2
1306	1303	1310 +	1368	1365	1368 -	1	1	2
1306	1303	1310 +	1385 -	-	-	1	1	2
1314	1313	1314 +	1395 -	-	-	1	1	2
1328	1324	1331 -	1363 -	-	-	1	1	2
1328	1324	1331 -	1369	1369	1370 +	1	1	2
1328	1324	1331 -	1442 -	-	-	1	1	2
1328	1324	1331 -	1514 -	-	-	1	1	2
1329	1326	1332 +	1366	1366	1367 -	1	1	2
1329	1326	1332 +	1427 -	-	-	1	1	2
1334	1333	1338 +	1457 -	-	-	1	1	2
1335	1335	1336 -	1414 -	-	+	2	0	2

1341	1340	1341 -	1313 -	-	+	2	0	2
1341	1340	1341 -	1408 -	-	+	0	2	2
1341	1340	1341 -	1533	1533	1534 -	1	1	2
1346	1343	1346 -	1753 -	-	+	1	1	2
1346	1345	1349 +	1262 -	-	-	1	1	2
1346	1345	1349 +	1299 -	-	-	1	1	2
1355	1352	1359 +	1269 -	-	+	1	1	2
1355	1352	1359 +	1402	1400	1402 -	1	1	2
1355	1352	1359 +	1485 -	-	+	1	1	2
1359	1354	1359 -	1371	1371	1372 -	1	1	2
1359	1354	1359 -	1460 -	-	-	1	1	2
1359	1354	1359 -	1472 -	-	-	1	1	2
1359	1354	1359 -	1503 -	-	-	1	1	2
1359	1354	1359 -	1511 -	-	-	1	1	2
1359	1354	1359 -	1516 -	-	-	1	1	2
1359	1354	1359 -	1526 -	-	-	1	1	2
1359	1354	1359 -	1580 -	-	-	1	1	2
1364	1361	1365 -	1529 -	-	-	2	0	2
1364	1361	1365 -	1861 -	-	-	1	1	2
1368	1368	1370 +	1328	1328	1329 -	1	1	2
1370	1370	1371 -	1442 -	-	+	1	1	2
1370	1370	1371 -	1534 -	-	-	0	2	2
1374	1372	1376 +	1296 -	-	-	1	1	2
1374	1372	1376 +	1501 -	-	+	1	1	2
1374	1372	1376 +	2117	2113	2117 +	1	1	2
1375	1373	1379 -	1424	1420	1424 +	1	1	2
1375	1373	1379 -	1506	1504	1506 +	1	1	2
1375	1373	1379 -	1533	1533	1534 -	1	1	2
1381	1381	1383 +	1363 -	-	-	1	1	2
1381	1381	1383 +	1509	1509	1510 +	1	1	2
1383	1380	1385 -	1434 -	-	+	1	1	2
1383	1380	1385 -	1532 -	-	-	1	1	2
1383	1380	1385 -	1554	1554	1555 +	1	1	2
1389	1389	1393 -	1468 -	-	+	1	1	2
1389	1389	1393 -	1542	1542	1543 +	1	1	2
1392	1388	1393 +	1466	1464	1466 -	1	1	2
1392	1388	1393 +	1497 -	-	-	1	1	2
1392	1388	1393 +	1543	1543	1544 -	1	1	2
1400	1398	1403 +	1369	1366	1369 -	1	1	2
1400	1398	1403 +	1469	1469	1470 -	1	1	2
1400	1398	1403 +	1619 -	-	-	1	1	2
1400	1398	1403 +	12514 -	-	+	1	1	2
1401	1398	1401 -	1443 -	-	-	1	1	2
1401	1398	1401 -	1568 -	-	-	1	1	2
1401	1398	1401 -	1607 -	-	-	1	1	2
1401	1398	1401 -	1727 -	-	-	1	1	2
1407	1404	1412 -	1430	1430	1431 +	1	1	2
1407	1404	1412 -	1470	1468	1470 +	1	1	2
1407	1404	1412 -	1531 -	-	+	1	1	2
1407	1404	1412 +	1579	1579	1580 +	1	1	2
1418	1414	1422 -	1494 -	-	+	2	0	2
1418	1414	1422 -	1510 -	-	-	1	1	2
1418	1414	1422 -	1512 -	-	+	1	1	2
1418	1414	1422 -	1520 -	-	+	1	1	2
1418	1414	1422 -	1690	1686	1690 -	1	1	2
1421	1417	1425 +	1449	1445	1449 +	1	1	2
1425	1424	1425 -	1490 -	-	+	0	2	2
1425	1424	1425 -	1858	1858	1859 -	1	1	2
1428	1426	1428 +	1331 -	-	-	1	1	2
1431	1427	1431 -	1524 -	-	-	1	1	2
1431	1427	1431 -	1589 -	-	-	1	1	2
1434	1434	1436 +	1543 -	-	-	1	1	2
1434	1434	1436 +	6691 -	-	+	0	2	2
1442	1439	1447 +	1410 -	-	-	1	1	2
1450	1448	1454 +	1362 -	-	-	1	1	2
1450	1448	1454 +	1458 -	-	+	1	1	2
1450	1448	1454 +	1490 -	-	-	2	0	2
1452	1447	1452 -	1584 -	-	-	1	1	2
1452	1447	1452 -	1642 -	-	+	0	2	2
1452	1447	1452 -	1687 -	-	-	1	1	2
1460	1457	1463 -	1513 -	-	+	1	1	2
1460	1457	1463 -	1522	1522	1523 +	1	1	2
1460	1457	1463 -	1649 -	-	+	1	1	2
1460	1457	1463 -	1655 -	-	-	1	1	2
1466	1466	1468 +	1626	1626	1627 -	1	1	2
1466	1466	1468 +	4409 -	-	-	2	0	2
1468	1467	1472 -	1613 -	-	-	1	1	2
1468	1467	1472 -	1979 -	-	+	1	1	2
1468	1467	1472 -	2008	2008	2009 -	1	1	2
1473	1471	1478 +	1383 -	-	-	1	1	2
1473	1471	1478 +	1451 -	-	-	1	1	2
1473	1471	1478 +	1556 -	-	+	1	1	2
1473	1471	1478 +	7475 -	-	-	1	1	2
1481	1481	1482 +	1446 -	-	-	1	1	2
1481	1481	1482 +	1587 -	-	-	1	1	2
1481	1481	1482 +	1660 -	-	+	1	1	2
1487	1484	1491 -	1654 -	-	-	1	1	2
1487	1484	1491 -	1722	1722	1724 -	0	2	2
1488	1488	1491 +	1446 -	-	-	1	1	2
1493 -	-	-	1660 -	-	-	1	1	2
1494	1494	1497 +	1461 -	-	-	0	2	2
1494	1494	1497 +	1573 -	-	-	1	1	2
1494	1494	1497 +	1619	1619	1621 -	1	1	2
1499	1495	1501 -	1527 -	-	+	1	1	2
1499	1495	1501 -	1686	1686	1687 -	1	1	2
1499	1495	1501 -	5984 -	-	-	2	0	2
1499	1499	1501 +	1538 -	-	+	1	1	2
1499	1499	1501 +	1572	1572	1573 +	1	1	2

1506	1503	1506 -	1519	1519	1520 +	2	0	2
1506	1503	1506 -	1577	1577	1578 -	1	1	2
1506	1503	1506 -	1687 -	-	-	1	1	2
1508	1504	1511 +	1401	1401	1402 -	1	1	2
1508	1504	1511 +	1500	1497	1500 -	1	1	2
1508	1504	1511 +	1587 -	-	+	1	1	2
1513	1509	1517 -	1673 -	-	-	1	1	2
1513	1509	1517 -	1694 -	-	-	1	1	2
1513	1509	1517 -	2117 -	-	+	1	1	2
1513	1512	1520 +	1493	1491	1493 +	1	1	2
1513	1512	1520 +	2244 -	-	-	1	1	2
1522	1520	1524 -	1700 -	-	-	1	1	2
1530	1525	1535 -	1674 -	-	-	1	1	2
1530	1525	1535 -	1719 -	-	-	1	1	2
1530	1525	1535 -	1731	1731	1732 -	1	1	2
1530	1525	1535 -	3996 -	-	-	1	1	2
1532	1529	1536 +	1404 -	-	-	1	1	2
1544	1541	1548 -	1596 -	-	+	1	1	2
1544	1541	1548 -	1689 -	-	-	0	2	2
1544	1544	1546 +	1389	1389	1390 -	1	1	2
1552 -	-	+	1579 -	-	+	1	1	2
1554	1550	1556 -	1658 -	-	-	1	1	2
1554	1550	1556 -	1693 -	-	-	1	1	2
1554	1550	1556 -	1725 -	-	-	1	1	2
1554	1550	1556 -	8116 -	-	-	2	0	2
1554	1550	1556 -	10326	10326	10327 -	1	1	2
1557	1557	1558 +	1377	1377	1378 -	1	1	2
1559	1558	1562 -	1586 -	-	-	0	2	2
1559	1558	1562 -	1757 -	-	-	1	1	2
1568	1564	1571 -	1688 -	-	-	1	1	2
1568	1564	1571 -	1705 -	-	-	1	1	2
1568	1564	1571 -	1731 -	-	-	1	1	2
1568	1564	1571 -	1878 -	-	-	1	1	2
1569	1566	1572 +	1444 -	-	-	1	1	2
1586	1586	1589 -	1690 -	-	-	1	1	2
1586	1585	1590 +	1623 -	-	-	1	1	2
1586	1585	1590 +	1726 -	-	+	1	1	2
1594 -	-	+	8065 -	-	+	1	1	2
1608	1605	1612 -	1766 -	-	-	1	1	2
1618	1614	1620 -	14109	14107	14109 +	1	1	2
1625	1624	1625 +	1718	1718	1719 -	1	1	2
1632	1630	1632 -	2101 -	-	-	0	2	2
1632	1631	1632 +	1711	1711	1712 -	1	1	2
1637	1633	1637 -	1731 -	-	-	1	1	2
1637	1633	1637 -	1795 -	-	-	1	1	2
1641	1637	1642 +	1667 -	-	+	0	2	2
1655	1652	1661 -	1690 -	-	+	1	1	2
1655	1652	1661 -	1743	1740	1743 +	1	1	2
1655	1652	1661 -	1764 -	-	-	1	1	2
1655	1652	1661 -	2008 -	-	-	1	1	2
1655	1652	1661 -	3560 -	-	+	1	1	2
1663 -	-	-	1790 -	-	-	0	2	2
1664 -	-	+	1724 -	-	+	0	2	2
1670	1667	1672 -	1686 -	-	+	1	1	2
1670	1667	1672 -	1800 -	-	-	1	1	2
1672	1669	1672 +	1729 -	-	+	2	0	2
1672	1669	1672 +	15338 -	-	-	1	1	2
1697 -	-	-	1810 -	-	-	1	1	2
1721	1718	1725 -	1852 -	-	-	1	1	2
1721	1718	1725 -	1944 -	-	-	1	1	2
1721	1718	1725 -	2060 -	-	+	1	1	2
1723	1721	1723 +	2091	2089	2091 +	1	1	2
1731	1731	1732 -	1826 -	-	-	1	1	2
1731	1731	1732 -	2001 -	-	-	0	2	2
1738	1735	1741 +	1789	1789	1790 -	1	1	2
1738	1735	1741 +	2032 -	-	-	1	1	2
1741	1740	1743 -	1957 -	-	-	1	1	2
1746 -	-	+	1822 -	-	+	1	1	2
1748	1745	1750 -	2323	2323	2324 -	1	1	2
1751 -	-	+	1954 -	-	-	1	1	2
1755	1752	1757 -	2475 -	-	-	1	1	2
1766	1764	1766 +	1781	1779	1781 -	1	1	2
1771	1771	1772 +	1721 -	-	-	1	1	2
1778	1778	1781 -	1957 -	-	-	1	1	2
1790	1786	1792 -	1936 -	-	-	1	1	2
1790	1786	1792 -	2011	2011	2012 -	1	1	2
1802	1799	1803 -	2120 -	-	-	1	1	2
1802	1802	1803 +	1800 -	-	-	1	1	2
1802	1802	1803 +	1826 -	-	+	1	1	2
1808	1805	1811 -	1945 -	-	-	1	1	2
1808	1805	1811 -	1986	1982	1986 -	1	1	2
1817	1814	1819 -	2007 -	-	-	1	1	2
1817	1814	1819 -	2012 -	-	-	1	1	2
1817	1814	1819 -	2040 -	-	-	1	1	2
1817	1814	1819 -	2074 -	-	-	0	2	2
1819	1819	1823 +	2142	2138	2142 +	1	1	2
1827	1823	1827 -	2002 -	-	-	1	1	2
1832	1831	1836 -	2063 -	-	-	1	1	2
1832	1831	1836 -	15048	15048	15049 -	1	1	2
1839	1836	1839 +	1936 -	-	-	1	1	2
1842	1838	1842 -	1972 -	-	-	1	1	2
1842	1838	1842 -	2071 -	-	-	1	1	2
1847	1846	1848 -	2025 -	-	-	1	1	2
1854	1850	1857 +	1776 -	-	+	1	1	2
1854	1850	1857 +	1796 -	-	+	1	1	2
1854	1850	1857 +	2189 -	-	-	0	2	2
1861	1860	1863 -	2094 -	-	-	1	1	2
1875	1875	1879 +	2032 -	-	-	1	1	2

1875	1875	1879 +	9734 -	-	+	1	1	2
1879	1876	1879 -	2029 -	-	-	1	1	2
1879	1876	1879 -	2086 -	-	-	1	1	2
1884 -	-	-	2025 -	-	-	1	1	2
1889	1887	1894 +	1790 -	-	-	1	1	2
1889	1887	1894 +	1871 -	-	-	1	1	2
1891	1889	1896 -	1909 -	-	+	1	1	2
1891	1889	1896 -	2008	2004	2008 +	1	1	2
1902	1900	1906 -	2075 -	-	-	1	1	2
1911	1909	1911 +	15363	15363	15364 +	1	1	2
1919	1919	1923 -	2070 -	-	-	1	1	2
1926	1925	1926 -	2108 -	-	-	1	1	2
1932	1930	1933 -	2064 -	-	-	1	1	2
1932	1930	1933 -	2086 -	-	-	1	1	2
1932	1930	1933 -	2094 -	-	-	1	1	2
1932	1930	1933 -	2107 -	-	-	1	1	2
1939	1935	1943 -	2062 -	-	-	1	1	2
1939	1935	1943 -	2078 -	-	-	1	1	2
1952	1950	1956 -	2094 -	-	-	1	1	2
1952	1950	1956 -	2243 -	-	-	1	1	2
1962	1961	1964 -	2079 -	-	-	1	1	2
1969	1967	1973 -	1983	1981	1983 +	1	1	2
1969	1967	1973 -	2112 -	-	+	1	1	2
1969	1967	1973 -	2140 -	-	-	1	1	2
1969	1967	1973 -	2204 -	-	-	1	1	2
1975	1973	1975 +	2049 -	-	-	1	1	2
1975	1973	1975 +	2357 -	-	-	1	1	2
1978	1975	1981 -	2106 -	-	-	1	1	2
1978	1975	1981 -	2134 -	-	+	2	0	2
1983	1983	1986 -	2002 -	-	+	1	1	2
1983	1983	1986 -	2130 -	-	+	0	2	2
1999	1995	1999 -	2102 -	-	+	2	0	2
1999	1995	1999 -	2147	2144	2147 -	1	1	2
1999	1995	1999 -	2189 -	-	-	1	1	2
2004	2000	2004 -	2097 -	-	+	0	2	2
2004	2000	2004 -	2107 -	-	-	1	1	2
2004	2000	2004 -	13930 -	-	+	1	1	2
2008 -	-	+	7973 -	-	-	1	1	2
2012	2008	2016 -	2041 -	-	+	0	2	2
2012	2008	2016 -	2046 -	-	+	2	0	2
2012	2008	2016 -	2205 -	-	+	1	1	2
2012	2008	2016 -	12493	12489	12493 -	0	2	2
2012	2008	2016 -	12670 -	-	-	1	1	2
2023	2022	2025 -	2098 -	-	-	1	1	2
2028	2026	2032 +	2051	2049	2051 -	1	1	2
2036	2035	2039 -	2203 -	-	-	1	1	2
2036	2035	2039 -	2282 -	-	-	1	1	2
2036	2035	2039 -	2308	2306	2308 -	1	1	2
2036	2035	2039 -	2638	2638	2639 -	1	1	2
2036	2036	2039 +	2108 -	-	+	0	2	2
2041	2041	2045 -	2033 -	-	+	2	0	2
2041	2041	2045 -	2095 -	-	-	1	1	2
2049	2046	2049 +	12527	12524	12527 +	2	0	2
2057 -	-	-	2414 -	-	-	1	1	2
2066	2064	2069 -	2105 -	-	+	2	0	2
2066	2064	2069 -	2198 -	-	-	1	1	2
2066	2064	2069 -	2209	2206	2209 -	1	1	2
2074	2071	2078 -	2100 -	-	+	0	2	2
2074	2071	2078 -	2161 -	-	-	1	1	2
2074	2071	2078 -	2231 -	-	-	1	1	2
2074	2071	2078 -	2236 -	-	+	1	1	2
2081	2081	2085 +	2024 -	-	+	1	1	2
2081	2081	2085 +	2202 -	-	+	1	1	2
2081	2081	2085 +	2228 -	-	-	1	1	2
2083	2082	2085 -	2112 -	-	+	1	1	2
2083	2082	2085 -	2132	2132	2133 +	1	1	2
2083	2082	2085 -	2202 -	-	-	1	1	2
2091	2090	2093 -	2106	2104	2106 +	1	1	2
2091	2090	2093 -	2238	2238	2239 -	1	1	2
2091	2090	2093 -	2318 -	-	-	1	1	2
2099	2099	2102 +	2137 -	-	-	1	1	2
2101	2099	2103 -	2177 -	-	+	2	0	2
2106	2106	2110 -	2170 -	-	+	0	2	2
2106	2106	2110 -	2204 -	-	-	1	1	2
2106	2106	2110 -	2276 -	-	-	1	1	2
2106	2106	2110 -	2305 -	-	-	1	1	2
2107	2104	2109 +	1976 -	-	-	1	1	2
2107	2104	2109 +	2093 -	-	-	1	1	2
2107	2104	2109 +	12493 -	-	+	1	1	2
2115	2112	2115 -	2239 -	-	-	1	1	2
2115	2112	2115 -	2291	2288	2291 +	1	1	2
2121	2121	2124 +	2150	2150	2151 -	1	1	2
2132	2131	2135 -	2239	2239	2240 -	1	1	2
2132	2131	2135 -	2323 -	-	-	1	1	2
2136	2132	2136 +	2410	2406	2410 +	1	1	2
2140	2137	2143 -	2189 -	-	-	1	1	2
2140	2137	2143 -	2280	2280	2281 -	1	1	2
2140	2137	2143 -	2370 -	-	-	1	1	2
2153	2149	2153 -	2243	2240	2243 +	1	1	2
2158	2155	2161 -	2234 -	-	+	1	1	2
2158	2155	2161 -	2305 -	-	-	1	1	2
2162 -	-	+	2483 -	-	+	1	1	2
2170	2167	2170 +	2109 -	-	-	1	1	2
2174	2170	2176 -	2337 -	-	-	1	1	2
2174	2170	2176 -	2371 -	-	-	1	1	2
2174	2170	2176 -	2380 -	-	-	1	1	2
2174	2170	2176 -	8381 -	-	-	1	1	2
2177	2173	2177 +	2054 -	-	-	1	1	2

2177	2173	2177	+	2107 -	-		-	1	1	2
2185 -	-		+	2311 -	-		+	1	1	2
2192	2188	2192	+	2128 -	-		-	1	1	2
2192	2188	2192	+	2212	2209	2212	-	1	1	2
2195	2193	2196	-	2263	2263	2264	+	1	1	2
2203	2200	2206	+	2158	2155	2158	-	1	1	2
2203	2200	2206	+	2191	2189	2191	-	1	1	2
2203	2200	2206	+	2349 -	-		-	1	1	2
2207	2205	2211	-	2234	2234	2235	+	1	1	2
2207	2205	2211	-	2415	2415	2416	-	1	1	2
2207	2205	2211	-	2519 -	-		+	1	1	2
2212	2211	2216	+	2442 -	-		-	1	1	2
2213 -	-		-	2385 -	-		+	1	1	2
2219	2215	2223	-	2418 -	-		-	1	1	2
2219	2215	2223	-	2442 -	-		-	1	1	2
2219	2215	2223	-	2504 -	-		-	1	1	2
2219	2215	2223	-	2794	2794	2795	-	1	1	2
2219	2215	2223	-	2801 -	-		-	1	1	2
2236	2236	2237	-	2387 -	-		-	1	1	2
2236	2236	2237	-	2442	2442	2443	-	1	1	2
2245	2244	2245	-	2205 -	-		+	1	1	2
2245	2243	2248	+	2214	2214	2215	-	1	1	2
2245	2243	2248	+	2221 -	-		-	1	1	2
2245	2243	2248	+	2268 -	-		-	1	1	2
2252	2252	2257	+	2212	2212	2213	-	1	1	2
2261	2257	2265	-	2377 -	-		+	1	1	2
2273	2273	2275	-	2446	2446	2447	-	1	1	2
2273	2273	2275	-	2451 -	-		-	1	1	2
2276 -	-		+	2238 -	-		-	1	1	2
2283	2283	2285	+	2272	2270	2272	-	1	1	2
2288	2283	2292	-	2419 -	-		-	1	1	2
2288	2283	2292	-	2470 -	-		-	1	1	2
2288	2283	2292	-	2486 -	-		-	1	1	2
2288	2283	2292	-	2702 -	-		-	0	2	2
2298 -	-		-	9677 -	-		+	1	1	2
2300 -	-		+	12082	12079	12082	-	2	0	2
2303	2302	2304	-	9671 -	-		+	1	1	2
2316	2312	2317	-	2449 -	-		-	1	1	2
2316	2312	2317	-	2472 -	-		-	2	0	2
2316	2312	2317	-	2485 -	-		-	1	1	2
2316	2312	2317	-	3247 -	-		-	1	1	2
2322	2320	2326	-	2641	2639	2641	-	1	1	2
2322	2320	2326	-	7207	7207	7208	+	1	1	2
2322	2320	2326	-	7231	7231	7232	-	0	2	2
2333	2329	2338	-	2441 -	-		-	1	1	2
2333	2329	2338	-	2455 -	-		-	1	1	2
2333	2329	2338	-	2480	2480	2481	-	1	1	2
2337	2334	2339	+	9637 -	-		-	1	1	2
2344	2343	2344	-	2533 -	-		-	1	1	2
2350	2348	2353	-	2484 -	-		-	1	1	2
2350	2348	2353	-	2495	2495	2496	-	1	1	2
2350	2348	2353	-	2525 -	-		-	1	1	2
2350	2348	2353	-	2595 -	-		-	1	1	2
2350	2348	2353	-	2895 -	-		-	1	1	2
2350	2348	2353	-	3490 -	-		-	1	1	2
2350	2348	2353	-	4550 -	-		-	1	1	2
2354	2352	2357	+	2329 -	-		-	1	1	2
2354	2352	2357	+	2917 -	-		-	1	1	2
2359	2359	2364	-	2523 -	-		-	1	1	2
2377	2373	2379	-	2554 -	-		-	1	1	2
2382	2382	2383	-	2488 -	-		-	1	1	2
2382	2382	2383	-	2551 -	-		-	1	1	2
2382	2380	2382	+	2554 -	-		-	1	1	2
2388 -	-		+	2307 -	-		-	1	1	2
2389	2389	2393	-	2408 -	-		+	1	1	2
2396	2396	2400	+	2418	2414	2418	-	1	1	2
2399	2395	2400	-	2536 -	-		+	1	1	2
2405	2402	2409	-	2439 -	-		+	1	1	2
2405	2402	2409	-	2458 -	-		+	1	1	2
2405	2402	2409	-	2548	2548	2549	-	1	1	2
2405	2402	2409	-	2560 -	-		-	1	1	2
2407	2404	2409	+	2449	2447	2449	-	1	1	2
2407	2404	2409	+	2528	2528	2529	-	1	1	2
2407	2404	2409	+	14096	14094	14096	-	1	1	2
2415	2411	2418	-	2507 -	-		-	1	1	2
2415	2411	2418	-	2568	2568	2569	-	1	1	2
2420	2419	2424	-	2483 -	-		+	1	1	2
2420	2419	2424	-	2574	2572	2574	-	1	1	2
2420	2419	2424	-	2611 -	-		+	1	1	2
2425	2425	2426	+	2301	2301	2302	-	1	1	2
2432	2428	2437	-	2656 -	-		-	1	1	2
2443	2439	2445	-	2512 -	-		+	1	1	2
2443	2439	2445	-	2513	2511	2513	-	1	1	2
2443	2439	2445	-	2604 -	-		-	1	1	2
2444	2443	2447	+	2466 -	-		-	1	1	2
2444	2443	2447	+	2529 -	-		+	1	1	2
2449 -	-		-	2513 -	-		-	1	1	2
2449	2449	2453	+	2407	2405	2407	-	1	1	2
2455	2451	2456	-	2540	2540	2541	-	1	1	2
2455	2451	2456	-	12082 -	-		-	2	0	2
2455	2454	2458	+	2419 -	-		-	1	1	2
2460	2457	2462	-	2546 -	-		-	1	1	2
2477	2473	2477	-	2503 -	-		+	1	1	2
2477	2473	2477	-	2514 -	-		-	1	1	2
2477	2473	2477	-	2588 -	-		-	1	1	2
2479 -	-		+	2591 -	-		-	1	1	2
2484	2480	2485	-	2661 -	-		-	1	1	2
2484	2480	2485	-	2859 -	-		-	1	1	2

2492		2487	2496	-	2675		2675	2676	-	1	1	2
2492		2487	2496	-	13885		13885	13886	-	1	1	2
2495		2495	2497	+	2419		2419	2420	-	1	1	2
2495		2495	2497	+	8391	-	-		-	1	1	2
2495		2495	2497	+	13886	-	-		+	2	0	2
2502		2499	2507	+	2477	-	-		+	1	1	2
2502		2499	2507	+	2619	-	-		+	1	1	2
2502		2499	2507	+	2649		2646	2649	-	1	1	2
2503		2498	2507	-	2550	-	-		-	1	1	2
2503		2498	2507	-	2629	-	-		-	1	1	2
2503		2498	2507	-	2691	-	-		-	1	1	2
2517		2514	2517	+	2458	-	-		+	1	1	2
2518		2514	2523	-	2586	-	-		-	1	1	2
2518		2514	2523	-	2647	-	-		-	1	1	2
2518		2514	2523	-	2654	-	-		+	1	1	2
2518		2514	2523	-	2702	-	-		-	1	1	2
2525		2524	2529	-	2751	-	-		-	1	1	2
2525		2524	2529	-	2757		2757	2758	-	1	1	2
2525	-	-		+	2481	-	-		+	1	1	2
2532		2532	2535	-	7604		7600	7604	+	2	0	2
2537	-	-		+	2486	-	-		-	1	1	2
2542		2536	2542	-	2652	-	-		-	1	1	2
2542		2536	2542	-	2673	-	-		-	1	1	2
2542		2536	2542	-	2706	-	-		-	1	1	2
2542		2536	2542	-	2761	-	-		-	1	1	2
2548		2543	2552	-	2690	-	-		-	1	1	2
2548		2543	2552	-	2706		2706	2707	-	1	1	2
2548		2543	2552	-	2719	-	-		-	1	1	2
2553		2550	2553	+	2643	-	-		-	1	1	2
2554		2554	2559	-	2662	-	-		-	1	1	2
2559		2559	2563	+	2500	-	-		-	1	1	2
2559		2559	2563	+	2601		2601	2602	-	1	1	2
2564		2560	2568	-	2619	-	-		+	1	1	2
2564		2560	2568	-	2626		2626	2627	+	1	1	2
2564		2560	2568	-	12082	-	-		-	2	0	2
2568		2566	2568	+	2655	-	-		-	1	1	2
2573		2571	2576	-	2593		2590	2593	+	1	1	2
2573		2571	2576	-	2752	-	-		-	1	1	2
2573		2573	2577	+	2608	-	-		-	1	1	2
2580	-	-		+	2624	-	-		-	1	1	2
2588		2585	2590	-	2749	-	-		-	1	1	2
2588		2585	2590	-	2780	-	-		+	1	1	2
2590		2585	2593	+	2527		2527	2529	-	1	1	2
2595		2595	2596	-	2751	-	-		-	1	1	2
2595		2595	2596	-	2759	-	-		-	1	1	2
2600		2600	2603	-	2758	-	-		-	1	1	2
2600		2600	2603	-	2770	-	-		-	1	1	2
2609		2605	2611	-	2715		2711	2715	+	1	1	2
2609		2605	2611	-	2758	-	-		-	1	1	2
2609		2605	2611	-	2812	-	-		-	1	1	2
2615		2615	2616	+	2570		2570	2571	-	1	1	2
2617		2613	2621	-	2627	-	-		+	0	2	2
2617		2613	2621	-	2682	-	-		+	1	1	2
2617		2613	2621	-	3047		3043	3047	-	1	1	2
2617		2613	2621	-	3104		3104	3105	-	1	1	2
2625		2622	2625	-	2663		2661	2663	+	1	1	2
2630		2626	2632	-	2654	-	-		+	1	1	2
2630		2626	2632	-	2759	-	-		-	1	1	2
2630		2626	2632	-	2768	-	-		-	1	1	2
2630		2626	2632	-	2780	-	-		-	1	1	2
2630		2626	2632	-	2831	-	-		-	1	1	2
2637	-	-		-	2769	-	-		-	1	1	2
2643		2640	2647	-	2722	-	-		-	1	1	2
2643		2640	2647	-	2736	-	-		+	1	1	2
2643		2640	2647	-	2758	-	-		-	1	1	2
2643		2640	2647	-	2810	-	-		-	1	1	2
2643		2640	2647	-	2822	-	-		-	1	1	2
2643		2639	2646	+	2653	-	-		+	1	1	2
2650	-	-		+	2479	-	-		-	1	1	2
2655		2652	2655	-	2758	-	-		-	1	1	2
2655		2652	2655	-	2820	-	-		-	1	1	2
2655		2652	2655	-	2826	-	-		-	1	1	2
2662		2661	2665	+	2742	-	-		+	2	0	2
2667		2667	2668	+	2702	-	-		-	1	1	2
2675		2673	2678	+	2627	-	-		-	1	1	2
2678		2678	2679	-	5663		5659	5663	-	1	1	2
2687		2686	2687	+	2533		2533	2534	-	1	1	2
2690		2689	2693	-	2760	-	-		-	1	1	2
2690		2689	2693	-	2781	-	-		-	1	1	2
2690		2689	2693	-	2807	-	-		-	1	1	2
2690		2689	2693	-	2842	-	-		-	1	1	2
2693	-	-		+	2676	-	-		-	1	1	2
2702		2699	2707	+	2667	-	-		-	1	1	2
2702		2699	2707	+	2712	-	-		-	1	1	2
2715		2711	2719	-	2838	-	-		+	1	1	2
2715		2711	2719	-	2852	-	-		-	1	1	2
2715		2711	2719	-	2935	-	-		-	1	1	2
2721		2721	2727	-	2850	-	-		-	1	1	2
2721		2721	2727	-	2950	-	-		-	1	1	2
2721		2721	2727	-	2999		2997	2999	-	0	2	2
2724	-	-		+	2867	-	-		+	1	1	2
2731		2731	2732	+	2774		2774	2775	+	1	1	2
2732	-	-		-	2925	-	-		+	0	2	2
2750		2748	2750	-	2909	-	-		-	1	1	2
2750		2748	2750	-	2909	-	-		+	1	1	2
2757		2756	2758	-	2921		2921	2922	-	1	1	2
2762		2762	2765	-	2921	-	-		-	1	1	2
2771		2771	2772	+	2749		2749	2750	-	1	1	2

2775		2773	2775	-	2869	-	-		+	1	1	2
2775		2773	2775	-	2970	-	-		+	1	1	2
2782		2779	2782	-	3113	-	-		-	1	1	2
2790		2790	2792	-	3044	-	-		+	1	1	2
2809		2807	2809	-	2935	-	-		-	1	1	2
2811	-	-		+	3010	-	-		+	0	2	2
2817	-	-		-	3012	-	-		-	1	1	2
2817		2815	2817	+	3014	-	-		+	2	0	2
2825		2822	2830	-	2866	-	-		+	1	1	2
2825		2822	2830	-	2999		2999	3000	-	1	1	2
2825		2822	2830	-	3069	-	-		-	0	2	2
2825		2822	2830	-	3194		3194	3195	+	1	1	2
2832		2832	2835	-	2976	-	-		-	1	1	2
2848		2845	2852	-	2910	-	-		-	2	0	2
2848		2845	2852	-	2915	-	-		-	0	2	2
2848		2845	2852	-	3016		3013	3016	-	1	1	2
2848		2845	2852	-	3051	-	-		-	1	1	2
2858		2854	2858	-	2967	-	-		-	1	1	2
2858		2854	2858	-	2999		2999	3000	-	1	1	2
2866	-	-		+	2877	-	-		-	0	2	2
2876		2876	2877	+	2866	-	-		-	2	0	2
2876		2876	2877	+	8936	-	-		+	1	1	2
2883		2879	2887	-	3019	-	-		-	1	1	2
2887		2887	2890	+	2902		2902	2903	-	1	1	2
2887		2887	2890	+	2946	-	-		-	1	1	2
2889		2888	2889	-	2941	-	-		+	1	1	2
2895		2891	2898	-	2933		2933	2934	+	0	2	2
2895		2891	2898	-	3044	-	-		-	1	1	2
2900	-	-		-	3068	-	-		-	1	1	2
2900		2900	2901	+	9946		9946	9947	-	1	1	2
2905		2905	2906	+	3161	-	-		-	1	1	2
2907		2902	2910	-	3004	-	-		-	1	1	2
2907		2902	2910	-	3030	-	-		-	1	1	2
2910		2910	2913	+	2957	-	-		+	1	1	2
2927		2923	2927	+	6124		6122	6124	-	1	1	2
2937	-	-		-	3062	-	-		+	1	1	2
2941		2941	2945	+	2962	-	-		-	1	1	2
2941		2941	2945	+	3027	-	-		-	1	1	2
2951		2951	2952	+	3148	-	-		-	1	1	2
2954		2951	2956	-	3122	-	-		-	1	1	2
2962		2960	2966	-	3135		3135	3136	-	1	1	2
2962		2960	2966	-	3146	-	-		-	1	1	2
2962		2961	2963	+	3010	-	-		-	1	1	2
2981		2979	2983	-	3074	-	-		+	1	1	2
2981		2979	2983	-	3078	-	-		-	1	1	2
2981		2979	2983	-	3098	-	-		-	1	1	2
2981		2979	2983	-	3104	-	-		-	1	1	2
2981		2979	2983	-	3142		3142	3143	-	1	1	2
2986		2986	2987	+	6417		6417	6418	-	1	1	2
2993		2990	2997	-	3056	-	-		+	1	1	2
2995		2995	2999	+	3056	-	-		-	1	1	2
3002		2999	3002	-	3126	-	-		-	1	1	2
3002		2999	3002	-	3200	-	-		-	1	1	2
3007		3004	3010	-	3014	-	-		+	1	1	2
3007		3004	3010	-	3044	-	-		+	1	1	2
3007		3004	3010	-	3071	-	-		-	1	1	2
3007		3004	3010	-	3106		3103	3106	-	1	1	2
3007		3004	3010	-	3162	-	-		-	1	1	2
3014		3014	3015	-	3038	-	-		+	2	0	2
3019		3018	3025	-	3033	-	-		+	0	2	2
3019		3018	3025	-	3109		3109	3110	-	1	1	2
3019		3018	3025	-	3131	-	-		-	1	1	2
3019		3018	3025	-	3273	-	-		-	1	1	2
3030		3026	3034	+	3022	-	-		-	1	1	2
3030		3026	3034	+	3128	-	-		-	1	1	2
3042		3040	3044	-	3100	-	-		+	1	1	2
3042		3040	3044	-	3232		3232	3233	-	1	1	2
3048		3046	3050	-	3096	-	-		+	2	0	2
3048		3046	3050	-	3234	-	-		-	1	1	2
3053		3053	3056	-	3217	-	-		-	1	1	2
3053		3053	3056	-	3245	-	-		-	1	1	2
3053		3053	3056	-	3261	-	-		-	1	1	2
3053		3053	3056	-	6495	-	-		-	1	1	2
3055		3051	3056	+	2995	-	-		-	1	1	2
3055		3051	3056	+	3031		3029	3031	-	1	1	2
3055		3051	3056	+	3109	-	-		+	1	1	2
3060	-	-		+	3072	-	-		-	2	0	2
3064		3060	3070	-	3143	-	-		-	1	1	2
3064		3060	3070	-	3177	-	-		-	1	1	2
3064		3060	3070	-	3303	-	-		-	1	1	2
3065	-	-		+	12652	-	-		+	2	0	2
3075	-	-		-	3256	-	-		-	1	1	2
3091		3088	3095	-	3195	-	-		-	1	1	2
3091		3088	3095	-	3234	-	-		-	1	1	2
3091		3088	3095	-	3239	-	-		-	1	1	2
3091		3088	3095	-	3281		3281	3284	-	1	1	2
3091		3088	3095	-	3295	-	-		-	1	1	2
3091		3088	3095	-	3420	-	-		-	2	0	2
3092		3092	3094	+	3050		3048	3050	-	1	1	2
3098		3097	3098	-	3368		3368	3369	-	1	1	2
3103		3099	3107	-	3211	-	-		-	1	1	2
3103		3099	3107	-	3234	-	-		-	1	1	2
3103		3099	3107	-	3250	-	-		-	1	1	2
3103		3099	3107	-	3256	-	-		-	1	1	2
3103		3099	3107	-	3424	-	-		-	0	2	2
3108		3108	3111	+	3075	-	-		-	1	1	2
3108		3108	3111	+	3143	-	-		-	1	1	2
3114	-	-		+	3155	-	-		-	1	1	2

3120	3119	3120	+	3187	-	-	+	1	1	2
3126	-	-	-	3237	-	-	-	1	1	2
3126	-	-	-	3351	-	-	-	1	1	2
3136	3136	3141	-	3176	-	-	+	1	1	2
3136	3136	3141	-	3282	-	-	-	1	1	2
3136	3136	3139	+	3101	-	-	-	1	1	2
3136	3136	3139	+	3115	-	-	-	1	1	2
3143	3143	3144	+	3214	3212	3214	-	1	1	2
3149	3149	3151	-	5379	-	-	-	1	1	2
3151	3151	3155	+	8208	8208	8209	+	1	1	2
3156	3156	3157	-	3261	-	-	-	1	1	2
3156	3156	3157	-	3328	-	-	-	1	1	2
3162	-	-	-	3283	-	-	-	1	1	2
3167	3167	3168	+	3150	3150	3151	-	1	1	2
3174	3173	3175	-	3297	-	-	+	1	1	2
3174	3173	3175	-	3324	-	-	-	1	1	2
3187	3187	3189	-	3256	3254	3256	+	1	1	2
3187	3187	3189	-	3331	-	-	-	1	1	2
3196	3194	3200	-	3273	-	-	+	1	1	2
3196	3194	3200	-	3367	-	-	-	1	1	2
3204	3204	3208	+	3136	3132	3136	-	1	1	2
3208	3204	3208	-	3333	-	-	-	2	0	2
3208	3204	3208	-	3572	-	-	-	1	1	2
3213	3213	3217	-	3338	-	-	-	0	2	2
3213	3213	3217	-	3389	-	-	+	1	1	2
3229	3228	3233	-	4486	-	-	-	1	1	2
3236	-	-	-	3407	-	-	+	1	1	2
3247	3244	3247	-	3273	-	-	+	2	0	2
3247	3244	3247	-	6100	6097	6100	-	1	1	2
3263	-	-	-	3461	-	-	-	1	1	2
3269	3265	3269	-	3462	3459	3462	-	1	1	2
3269	3265	3269	-	3515	-	-	-	2	0	2
3273	3270	3274	+	3289	3287	3289	-	1	1	2
3273	3270	3274	+	3412	-	-	-	1	1	2
3286	-	-	-	3361	-	-	+	1	1	2
3291	3290	3296	-	3322	3320	3322	+	1	1	2
3291	3290	3296	-	3361	3361	3362	+	1	1	2
3291	3290	3296	-	3434	-	-	-	1	1	2
3301	3298	3305	-	3391	-	-	+	1	1	2
3301	3298	3305	-	3499	-	-	-	1	1	2
3306	3305	3307	+	3402	-	-	-	1	1	2
3306	3305	3307	+	3493	-	-	-	1	1	2
3308	3308	3311	-	3401	-	-	+	0	2	2
3308	3308	3311	-	3453	3451	3453	-	1	1	2
3319	-	-	+	3361	-	-	+	1	1	2
3324	3320	3327	-	3355	-	-	-	1	1	2
3324	3320	3327	-	3604	-	-	-	1	1	2
3324	3320	3327	-	5767	5764	5767	-	1	1	2
3329	-	-	+	3360	-	-	-	1	1	2
3336	3333	3336	-	3504	-	-	-	1	1	2
3342	3342	3345	-	3474	-	-	+	1	1	2
3348	3348	3349	-	3578	-	-	-	1	1	2
3348	3348	3349	-	3913	-	-	-	1	1	2
3351	3348	3351	+	3459	-	-	-	1	1	2
3360	3360	3365	+	3338	3336	3338	-	1	1	2
3367	3363	3371	-	3422	-	-	+	1	1	2
3367	3363	3371	-	3562	-	-	-	1	1	2
3367	3363	3371	-	3586	-	-	-	1	1	2
3369	-	-	+	3312	-	-	+	1	1	2
3381	-	-	+	3419	-	-	-	0	2	2
3382	3378	3383	-	3548	-	-	-	1	1	2
3382	3378	3383	-	3570	-	-	-	1	1	2
3382	3378	3383	-	3586	-	-	-	1	1	2
3387	3385	3390	-	3579	-	-	-	1	1	2
3387	3385	3390	-	3712	-	-	-	1	1	2
3387	3385	3390	-	4420	-	-	-	1	1	2
3387	-	-	+	3413	-	-	-	2	0	2
3395	3394	3395	-	3673	-	-	+	1	1	2
3403	3399	3406	-	4401	4401	4404	-	1	1	2
3408	3407	3408	+	3334	3334	3335	-	1	1	2
3408	3407	3408	+	3363	-	-	-	1	1	2
3411	3407	3414	-	3450	-	-	+	2	0	2
3411	3407	3414	-	3570	-	-	-	1	1	2
3411	3407	3414	-	3658	3658	3659	+	1	1	2
3415	3415	3419	+	3401	-	-	-	1	1	2
3419	3419	3422	-	3650	-	-	+	1	1	2
3419	3419	3422	-	3710	-	-	-	1	1	2
3421	3420	3421	+	3441	-	-	+	1	1	2
3425	-	-	-	3512	-	-	+	1	1	2
3425	-	-	-	3645	-	-	+	1	1	2
3434	3433	3436	-	3483	-	-	+	1	1	2
3435	3432	3435	+	3645	-	-	+	1	1	2
3441	3438	3444	-	14386	-	-	+	1	1	2
3448	3448	3450	-	3409	-	-	+	2	0	2
3448	3448	3450	-	3538	-	-	-	1	1	2
3448	3448	3450	-	3672	-	-	-	1	1	2
3453	3453	3454	+	14613	-	-	+	1	1	2
3458	3458	3459	-	3585	-	-	-	1	1	2
3467	3464	3467	-	3517	-	-	-	1	1	2
3467	3464	3467	-	3585	-	-	+	1	1	2
3472	-	-	-	3510	-	-	+	1	1	2
3479	-	-	+	6082	-	-	+	1	1	2
3482	3479	3486	-	3563	-	-	+	2	0	2
3482	3479	3486	-	3579	3579	3580	-	1	1	2
3482	3479	3486	-	3595	-	-	-	1	1	2
3488	3487	3492	-	3569	-	-	+	1	1	2
3488	3487	3492	-	3595	-	-	-	1	1	2
3488	-	-	+	3569	-	-	-	1	1	2

3495	3495	3496 -	3522 -	-	+	2	0	2
3501 -	-	-	4308 -	-	-	1	1	2
3510	3506	3510 -	3666 -	-	-	1	1	2
3512 -	-	+	3458 -	-	+	1	1	2
3515	3512	3519 -	3589	3589	3590 -	1	1	2
3515	3512	3519 -	3659 -	-	-	1	1	2
3518	3517	3522 +	7603	7600	7603 +	1	1	2
3524 -	-	+	3493 -	-	-	1	1	2
3525	3522	3528 -	3495 -	-	+	2	0	2
3525	3522	3528 -	3660 -	-	-	1	1	2
3525	3522	3528 -	3678 -	-	-	1	1	2
3530	3529	3534 -	3585 -	-	+	1	1	2
3530	3529	3534 -	3712 -	-	-	1	1	2
3538 -	-	+	3521 -	-	-	1	1	2
3545	3541	3549 -	3676 -	-	+	1	1	2
3545	3541	3549 -	3699 -	-	-	1	1	2
3545	3541	3549 -	3741	3739	3741 -	1	1	2
3546	3546	3548 +	3459	3457	3459 -	1	1	2
3546	3546	3548 +	3586 -	-	-	2	0	2
3564	3560	3564 -	3634 -	-	-	1	1	2
3569	3569	3573 +	3488 -	-	-	1	1	2
3574	3570	3578 -	3646 -	-	+	1	1	2
3574	3570	3578 -	3728 -	-	+	1	1	2
3574	3570	3578 -	3744	3742	3744 -	1	1	2
3580 -	-	+	3634 -	-	-	1	1	2
3585	3581	3588 -	3718 -	-	-	1	1	2
3585	3581	3588 -	3741 -	-	-	1	1	2
3585	3581	3588 -	3746 -	-	-	1	1	2
3585	3581	3588 -	3760 -	-	+	1	1	2
3585 -	-	+	3636 -	-	-	1	1	2
3590	3589	3590 -	3700 -	-	+	1	1	2
3590	3590	3592 +	3606 -	-	+	1	1	2
3590	3590	3592 +	12429 -	-	+	2	0	2
3595	3591	3601 -	3635 -	-	+	1	1	2
3595	3591	3601 -	3639	3636	3639 -	1	1	2
3595	3591	3601 -	3713	3713	3715 -	1	1	2
3595	3591	3601 -	3873	3871	3873 -	1	1	2
3595	3591	3601 -	5204 -	-	-	1	1	2
3606	3605	3609 -	3743 -	-	-	1	1	2
3606	3605	3609 -	11205 -	-	+	1	1	2
3611	3611	3612 -	3694 -	-	-	1	1	2
3614 -	-	+	3546	3546	3547 -	1	1	2
3619	3617	3620 -	6697 -	-	+	1	1	2
3630	3629	3630 +	3591	3591	3592 -	1	1	2
3630	3629	3630 +	3660 -	-	-	1	1	2
3631	3628	3632 -	3833 -	-	-	1	1	2
3636	3633	3640 +	3597	3594	3597 +	1	1	2
3636	3633	3640 +	3650	3648	3650 +	1	1	2
3647	3643	3649 -	3703 -	-	+	1	1	2
3647	3643	3649 -	3759 -	-	-	1	1	2
3647	3643	3649 -	3970 -	-	-	1	1	2
3647	3643	3649 -	5084 -	-	-	1	1	2
3654	3651	3654 -	3869 -	-	-	1	1	2
3661	3656	3662 -	3756	3756	3757 +	1	1	2
3661	3656	3662 -	3832	3832	3833 -	1	1	2
3661	3656	3662 -	3846 -	-	-	1	1	2
3668	3665	3669 +	3603 -	-	-	1	1	2
3679	3676	3681 +	3691 -	-	-	2	0	2
3679	3676	3681 +	3753 -	-	-	1	1	2
3679	3676	3681 +	12175 -	-	+	1	1	2
3681	3677	3684 -	3712 -	-	-	1	1	2
3685	3685	3686 +	3727	3727	3728 -	1	1	2
3690	3686	3695 -	3808	3804	3808 -	1	1	2
3690	3686	3695 -	3877 -	-	-	1	1	2
3690	3686	3695 -	4088	4088	4089 -	1	1	2
3690	3687	3693 +	3704	3700	3704 +	1	1	2
3690	3687	3693 +	3934 -	-	+	1	1	2
3690	3687	3693 +	4846	4844	4846 +	1	1	2
3706	3705	3713 -	3858 -	-	-	1	1	2
3706	3705	3713 -	3875	3871	3875 -	1	1	2
3706	3705	3713 -	3895 -	-	-	1	1	2
3707 -	-	+	7367 -	-	+	1	1	2
3715 -	-	-	3862 -	-	-	1	1	2
3724	3724	3727 +	3743 -	-	+	1	1	2
3724	3724	3727 +	3748 -	-	+	1	1	2
3727	3727	3731 -	3869 -	-	-	1	1	2
3727	3727	3731 -	7311	7307	7311 +	0	2	2
3730	3730	3732 +	3765	3765	3766 -	1	1	2
3730	3730	3732 +	3768 -	-	+	1	1	2
3730	3730	3732 +	3812 -	-	-	1	1	2
3739	3736	3741 +	3591 -	-	-	1	1	2
3739	3736	3741 +	3889 -	-	-	1	1	2
3739	3736	3741 +	4039 -	-	-	1	1	2
3741	3738	3741 -	3928 -	-	-	1	1	2
3746	3743	3746 -	3751 -	-	+	1	1	2
3746	3743	3746 -	3821 -	-	+	1	1	2
3748 -	-	+	3837 -	-	+	1	1	2
3752	3747	3752 -	3837	3837	3838 -	1	1	2
3752	3747	3752 -	3881 -	-	-	1	1	2
3752	3747	3752 -	4229 -	-	+	1	1	2
3758	3758	3759 +	3661 -	-	-	1	1	2
3761	3757	3764 -	3895 -	-	-	1	1	2
3761	3757	3764 -	3906 -	-	-	1	1	2
3761	3757	3764 -	3925 -	-	-	1	1	2
3761	3757	3764 -	4140 -	-	-	1	1	2
3772	3772	3774 +	4772 -	-	+	1	1	2
3774	3774	3777 -	3985 -	-	-	1	1	2
3779 -	-	-	3867 -	-	+	1	1	2

3787	-	-	-	3982	-	-	-	1	1	2
3798	3797	3800	+	3758	-	-	+	1	1	2
3805	3805	3807	-	3945	3943	3945	-	1	1	2
3805	3805	3807	-	4004	-	-	-	1	1	2
3805	3805	3807	-	4664	-	-	-	1	1	2
3810	3810	3813	-	3944	-	-	-	1	1	2
3810	3810	3813	-	3951	-	-	-	1	1	2
3810	3807	3810	+	3773	-	-	+	1	1	2
3819	-	-	+	3837	-	-	+	1	1	2
3825	3821	3827	-	3836	-	-	+	1	1	2
3830	-	-	-	3985	-	-	-	1	1	2
3831	-	-	+	3992	-	-	-	1	1	2
3851	3845	3856	+	3946	-	-	-	1	1	2
3852	3851	3855	-	3975	-	-	-	1	1	2
3852	3851	3855	-	4094	4091	4094	-	1	1	2
3852	3851	3855	-	6170	-	-	+	0	2	2
3860	-	-	-	4081	-	-	-	1	1	2
3865	-	-	+	7928	-	-	-	1	1	2
3868	3864	3868	-	4108	-	-	-	1	1	2
3870	-	-	+	4092	-	-	-	1	1	2
3870	-	-	+	4416	-	-	-	1	1	2
3873	3873	3876	-	3920	-	-	+	1	1	2
3873	3873	3876	-	4081	4079	4081	-	1	1	2
3883	3883	3885	-	4035	4035	4036	-	1	1	2
3885	3882	3885	+	4579	-	-	+	1	1	2
3895	-	-	-	4319	-	-	-	1	1	2
3911	3909	3911	+	4140	4140	4141	-	1	1	2
3917	3917	3918	+	6453	6453	6454	+	1	1	2
3920	3920	3928	-	4053	-	-	-	1	1	2
3920	3920	3928	-	4074	-	-	-	1	1	2
3920	3920	3928	-	4086	-	-	-	1	1	2
3920	3920	3928	-	4094	-	-	-	1	1	2
3920	3920	3928	-	4262	-	-	-	1	1	2
3920	3920	3928	-	10139	10139	10140	-	1	1	2
3930	-	-	+	3966	-	-	-	1	1	2
3935	3931	3939	-	4087	-	-	-	1	1	2
3935	3931	3939	-	4246	-	-	-	1	1	2
3935	3931	3939	-	4434	-	-	-	1	1	2
3935	3934	3936	+	4266	4266	4267	+	1	1	2
3944	3943	3945	+	4003	4001	4003	-	1	1	2
3951	3948	3951	-	4107	-	-	-	1	1	2
3951	3948	3951	-	4116	4116	4117	-	1	1	2
3957	3952	3960	-	3968	-	-	+	2	0	2
3957	3952	3960	-	4129	-	-	-	1	1	2
3957	3952	3960	-	4246	-	-	-	1	1	2
3957	3953	3960	+	3944	-	-	-	1	1	2
3957	3953	3960	+	4112	-	-	-	1	1	2
3962	-	-	+	3901	-	-	+	1	1	2
3968	-	-	-	3954	-	-	+	2	0	2
3969	3968	3973	+	3895	-	-	+	1	1	2
3969	3968	3973	+	4084	-	-	+	1	1	2
3984	3981	3987	-	3995	-	-	+	1	1	2
3984	3981	3987	-	4014	-	-	+	1	1	2
3984	3981	3987	-	15270	-	-	-	1	1	2
3984	3982	3985	+	4212	-	-	+	1	1	2
3989	3988	3989	+	3960	-	-	-	1	1	2
3998	3996	3998	-	4043	-	-	+	1	1	2
4003	4001	4003	-	4064	-	-	+	1	1	2
4003	4001	4003	-	4117	-	-	-	1	1	2
4009	4005	4011	+	3841	-	-	-	1	1	2
4009	4005	4011	+	4033	-	-	+	1	1	2
4009	4005	4011	+	4054	4052	4054	+	1	1	2
4009	4005	4011	+	4102	-	-	+	1	1	2
4019	4019	4020	+	4034	4034	4035	+	1	1	2
4023	4019	4023	-	4054	-	-	+	1	1	2
4031	4027	4032	+	3978	-	-	-	1	1	2
4031	4027	4032	+	4009	-	-	-	1	1	2
4031	4027	4032	+	4084	-	-	+	1	1	2
4049	4047	4049	-	4085	-	-	+	1	1	2
4049	4047	4049	-	4212	4212	4213	-	1	1	2
4058	-	-	+	4125	-	-	-	1	1	2
4064	4060	4067	+	4278	-	-	-	1	1	2
4064	4060	4067	+	12334	12334	12335	+	1	1	2
4072	4068	4072	-	4120	-	-	-	1	1	2
4072	4068	4072	-	4223	4223	4224	-	1	1	2
4072	4068	4072	-	12620	-	-	+	0	2	2
4075	4071	4075	+	3992	3992	3993	-	1	1	2
4084	4083	4087	-	4111	-	-	+	1	1	2
4084	4083	4087	-	4256	4252	4256	-	2	0	2
4090	4089	4094	-	4102	-	-	+	1	1	2
4090	4089	4094	-	4211	-	-	-	1	1	2
4091	4088	4091	+	3944	-	-	-	1	1	2
4102	4100	4104	-	4129	-	-	+	1	1	2
4102	4100	4104	-	4179	4179	4180	-	1	1	2
4102	4100	4104	-	4239	-	-	-	1	1	2
4102	4100	4104	-	4400	-	-	-	1	1	2
4102	4100	4104	-	4657	-	-	-	1	1	2
4109	4109	4113	+	4136	4132	4136	-	1	1	2
4109	4109	4113	+	7254	7254	7255	-	1	1	2
4116	4113	4118	-	4223	-	-	-	1	1	2
4116	4113	4118	-	4228	-	-	+	2	0	2
4116	4113	4118	-	4244	-	-	-	1	1	2
4116	4113	4118	-	4307	-	-	-	1	1	2
4122	4122	4125	-	4224	-	-	+	0	2	2
4133	4129	4133	-	4149	-	-	+	1	1	2
4133	4129	4133	-	4563	4563	4564	+	1	1	2
4140	4139	4141	+	4314	-	-	-	1	1	2
4145	-	-	-	4970	-	-	-	1	1	2

4150	-	-		+	4165	-	-		-	1	1	2
4160		4160	4161	+	4268		4268	4269	-	1	1	2
4164		4162	4165	-	4209	-	-		+	2	0	2
4164		4162	4165	-	4284		4284	4285	+	1	1	2
4164		4162	4165	-	4367	-	-		-	2	0	2
4169		4167	4171	-	4204	-	-		+	0	2	2
4169		4167	4171	-	4345	-	-		-	1	1	2
4169		4167	4171	-	4373	-	-		-	0	2	2
4177		4177	4179	+	4202	-	-		-	1	1	2
4177		4177	4179	+	4258	-	-		-	1	1	2
4179		4175	4183	-	4215		4215	4216	+	1	1	2
4179		4175	4183	-	4225	-	-		+	1	1	2
4179		4175	4183	-	4332	-	-		-	1	1	2
4185	-	-		-	4206	-	-		+	1	1	2
4191		4191	4194	+	4211		4211	4212	-	1	1	2
4201		4199	4202	-	4304	-	-		+	1	1	2
4201		4199	4202	-	4329	-	-		+	1	1	2
4207		4207	4209	-	4401	-	-		-	1	1	2
4207		4207	4209	-	4406	-	-		-	1	1	2
4207		4207	4209	-	4411	-	-		-	1	1	2
4212		4212	4214	-	4292	-	-		+	2	0	2
4212		4212	4214	-	4374	-	-		-	1	1	2
4212		4212	4214	-	4437		4435	4437	-	1	1	2
4219		4219	4220	+	4242		4242	4243	-	1	1	2
4219		4219	4220	+	4286		4286	4287	-	1	1	2
4222		4218	4225	-	4256		4253	4256	+	1	1	2
4222		4218	4225	-	4287	-	-		+	0	2	2
4222		4218	4225	-	4351	-	-		-	1	1	2
4222		4218	4225	-	4437	-	-		-	1	1	2
4222		4218	4225	-	4452	-	-		-	1	1	2
4224		4223	4225	+	4150		4147	4150	-	1	1	2
4224		4223	4225	+	4188		4188	4189	-	1	1	2
4229		4227	4230	-	4433	-	-		-	2	0	2
4236		4232	4238	-	4366	-	-		+	1	1	2
4236		4232	4238	-	4392	-	-		-	1	1	2
4236		4232	4238	-	4397	-	-		-	1	1	2
4236		4232	4238	-	4405	-	-		-	1	1	2
4236		4232	4238	-	4418	-	-		-	1	1	2
4241		4240	4244	-	4352		4349	4352	+	1	1	2
4251		4250	4252	-	4306		4306	4307	+	1	1	2
4257		4256	4257	-	4363	-	-		-	1	1	2
4258		4255	4260	+	4223	-	-		-	1	1	2
4258		4255	4260	+	12082	-	-		-	2	0	2
4263		4263	4265	+	12082	-	-		-	2	0	2
4264		4260	4266	-	4295	-	-		+	2	0	2
4270	-	-		-	4290	-	-		+	2	0	2
4282		4282	4283	+	4265		4265	4266	-	1	1	2
4284		4281	4286	-	4274	-	-		+	2	0	2
4284		4281	4286	-	4358	-	-		-	1	1	2
4284		4281	4286	-	11996	-	-		+	1	1	2
4288		4287	4292	+	4260	-	-		-	1	1	2
4288		4287	4292	+	4269		4269	4270	-	1	1	2
4289		4287	4289	-	4499	-	-		-	1	1	2
4295		4294	4295	-	4266	-	-		+	2	0	2
4312		4308	4312	-	4491	-	-		-	1	1	2
4312		4308	4312	-	5366	-	-		-	1	1	2
4326		4326	4329	-	4511	-	-		-	1	1	2
4334		4334	4336	-	4693		4691	4693	+	1	1	2
4343		4340	4345	-	4505	-	-		-	1	1	2
4363	-	-		+	4349	-	-		-	1	1	2
4371		4371	4375	-	4416	-	-		+	1	1	2
4371		4371	4375	-	4487	-	-		-	1	1	2
4371		4371	4375	-	4761	-	-		-	1	1	2
4372		4372	4373	+	4414		4414	4415	-	1	1	2
4381	-	-		+	4303	-	-		-	1	1	2
4386		4383	4386	-	4467	-	-		-	1	1	2
4386		4383	4386	-	4685	-	-		-	1	1	2
4404		4404	4406	+	4456		4456	4457	-	1	1	2
4418		4414	4422	+	4372		4372	4373	-	1	1	2
4418		4414	4422	+	4404	-	-		-	1	1	2
4418		4414	4422	+	4474		4474	4475	-	1	1	2
4418		4414	4422	+	4597	-	-		-	1	1	2
4418		4414	4422	+	6323	-	-		-	1	1	2
4418		4414	4422	+	9671	-	-		+	0	2	2
4428	-	-		-	13461	-	-		+	2	0	2
4437		4434	4437	-	5158	-	-		-	1	1	2
4437		4434	4437	-	13452	-	-		+	0	2	2
4437		4435	4438	+	4400		4400	4401	-	1	1	2
4437		4435	4438	+	4621	-	-		+	1	1	2
4437		4435	4438	+	11469	-	-		+	1	1	2
4442		4442	4444	-	4564		4564	4565	+	1	1	2
4442		4442	4444	-	4656	-	-		-	1	1	2
4458		4458	4460	+	4428	-	-		-	1	1	2
4460	-	-		-	4641	-	-		-	1	1	2
4478	-	-		+	4524	-	-		+	1	1	2
4483		4483	4485	-	4682		4680	4682	-	1	1	2
4490		4490	4493	+	4686		4683	4686	-	1	1	2
4492	-	-		-	4501	-	-		+	2	0	2
4501		4496	4502	-	4544	-	-		+	1	1	2
4501		4496	4502	-	4629		4629	4630	+	1	1	2
4501		4496	4502	-	4634		4634	4635	-	1	1	2
4501		4496	4502	-	4646	-	-		+	1	1	2
4508		4506	4511	-	4689	-	-		-	1	1	2
4508		4506	4511	-	4723	-	-		-	1	1	2
4517		4513	4521	-	4556	-	-		-	0	2	2
4517		4513	4521	-	5121	-	-		-	1	1	2
4517	-	-		+	4678	-	-		-	1	1	2
4528	-	-		+	5278	-	-		+	1	1	2

4535		4535	4540	-	4651		4651	4652	-	1	1	2
4544		4544	4547	-	4682	-	-		-	1	1	2
4552		4551	4553	-	4592	-	-		+	1	1	2
4552		4551	4553	-	4674	-	-		-	1	1	2
4552		4551	4553	-	4686	-	-		-	1	1	2
4559		4556	4559	-	4630	-	-		+	1	1	2
4559		4556	4559	-	4647	-	-		+	1	1	2
4559		4556	4559	-	4689	-	-		-	1	1	2
4559		4556	4559	-	4786	-	-		-	2	0	2
4562		4562	4564	+	4660	-	-		-	1	1	2
4566		4564	4571	-	4711	-	-		-	1	1	2
4572		4570	4577	+	4633		4633	4634	-	1	1	2
4582		4578	4586	-	4627	-	-		+	1	1	2
4582		4578	4586	-	4817		4817	4818	+	1	1	2
4582		4578	4586	-	5570		5570	5571	+	1	1	2
4592		4589	4592	-	4634	-	-		+	1	1	2
4592		4589	4592	-	4790	-	-		-	1	1	2
4605	-	-		+	4710	-	-		+	1	1	2
4610		4609	4615	-	4791		4791	4792	+	1	1	2
4610		4609	4615	-	4976		4976	4977	-	1	1	2
4617	-	-		+	4802	-	-		-	1	1	2
4617	-	-		+	5212	-	-		-	1	1	2
4619		4617	4619	-	4911	-	-		-	1	1	2
4626		4626	4629	-	4719	-	-		+	2	0	2
4631		4630	4634	-	4713	-	-		+	0	2	2
4631		4630	4634	-	4813	-	-		-	1	1	2
4631		4630	4634	-	4853	-	-		-	1	1	2
4631		4630	4634	-	4970	-	-		-	1	1	2
4632		4629	4636	+	11050	-	-		+	1	1	2
4637		4637	4642	-	5253	-	-		-	0	2	2
4641		4638	4645	+	4713	-	-		+	1	1	2
4653		4650	4654	+	4592	-	-		-	1	1	2
4653		4650	4654	+	4687	-	-		+	1	1	2
4659		4659	4660	+	4704	-	-		-	1	1	2
4659		4659	4660	+	4785	-	-		-	1	1	2
4659		4659	4660	+	6023		6023	6024	+	1	1	2
4660		4660	4661	-	4661	-	-		+	2	0	2
4666	-	-		-	4830	-	-		-	1	1	2
4678	-	-		-	4774	-	-		+	1	1	2
4682		4682	4686	+	4569	-	-		-	1	1	2
4682		4682	4686	+	4749	-	-		-	1	1	2
4684		4684	4685	-	4784	-	-		-	1	1	2
4684		4684	4685	-	4848	-	-		-	1	1	2
4690	-	-		-	6001	-	-		-	1	1	2
4715	-	-		-	5370	-	-		-	1	1	2
4720	-	-		-	4965	-	-		-	1	1	2
4723		4719	4728	+	4739		4737	4739	+	1	1	2
4734		4732	4734	-	5043	-	-		+	1	1	2
4734		4734	4739	+	5892		5892	5893	+	1	1	2
4753	-	-		+	4776	-	-		-	1	1	2
4755		4755	4758	-	4774		4771	4774	+	1	1	2
4769		4769	4770	-	4914	-	-		-	1	1	2
4769		4769	4770	-	5213		5213	5214	-	1	1	2
4772		4772	4777	+	4753	-	-		-	1	1	2
4772		4772	4777	+	4925	-	-		+	1	1	2
4781		4781	4782	-	5110	-	-		-	2	0	2
4789		4789	4790	+	4832	-	-		-	1	1	2
4812		4808	4815	+	4843		4843	4844	-	1	1	2
4812		4808	4815	+	4956	-	-		-	1	1	2
4819		4817	4819	-	4865	-	-		-	1	1	2
4819		4817	4819	-	5329	-	-		-	1	1	2
4822		4822	4824	+	4884	-	-		-	1	1	2
4827		4827	4830	+	5114	-	-		+	1	1	2
4831		4830	4831	-	5003	-	-		+	0	2	2
4835	-	-		+	4893	-	-		-	1	1	2
4836		4833	4838	-	4898	-	-		+	1	1	2
4836		4833	4838	-	13114	-	-		-	1	1	2
4841	-	-		-	13114	-	-		-	1	1	2
4849		4846	4849	+	4764	-	-		-	1	1	2
4849		4846	4849	+	5036	-	-		+	1	1	2
4854		4852	4857	-	5031	-	-		-	1	1	2
4854		4852	4857	-	5137		5137	5138	-	1	1	2
4854		4852	4857	-	5307	-	-		-	1	1	2
4854	-	-		+	5237	-	-		+	1	1	2
4859		4859	4863	-	4877	-	-		+	1	1	2
4859		4857	4859	+	4869	-	-		+	1	1	2
4868		4866	4869	-	4956	-	-		+	1	1	2
4877		4875	4881	-	4902	-	-		+	1	1	2
4877		4874	4881	+	4895		4895	4896	-	1	1	2
4877		4874	4881	+	4957	-	-		-	1	1	2
4877		4874	4881	+	4962		4962	4963	-	1	1	2
4877		4874	4881	+	5030	-	-		-	1	1	2
4888		4884	4892	+	4909	-	-		+	1	1	2
4888		4884	4892	+	4927	-	-		+	1	1	2
4891	-	-		-	6007	-	-		-	1	1	2
4901	-	-		-	5007	-	-		+	1	1	2
4907		4907	4911	-	4983	-	-		+	1	1	2
4907		4907	4911	-	4996		4996	4997	+	2	0	2
4907		4907	4911	-	5045	-	-		-	1	1	2
4907		4907	4911	-	5067	-	-		-	1	1	2
4914		4914	4918	-	4962		4958	4962	+	1	1	2
4914		4914	4918	-	4992	-	-		+	0	2	2
4927		4923	4929	-	5009	-	-		+	2	0	2
4927		4923	4929	-	5139		5136	5139	-	1	1	2
4927		4923	4929	-	5222	-	-		-	1	1	2
4927		4924	4930	+	5038	-	-		+	1	1	2
4927		4924	4930	+	5235		5235	5236	-	1	1	2
4927		4924	4930	+	5296	-	-		-	1	1	2

4932		4931	4932	-	5006	-	-		+	0	2	2
4932		4931	4932	-	5074	-	-		-	1	1	2
4935	-	-		+	5004	-	-		+	1	1	2
4940		4936	4944	-	5080		5080	5081	-	1	1	2
4940		4936	4944	-	5097	-	-		+	1	1	2
4940	-	-		+	9602	-	-		+	1	1	2
4945	-	-		+	5273	-	-		-	1	1	2
4946		4945	4950	-	5267		5264	5267	-	1	1	2
4956		4954	4956	-	4990	-	-		+	1	1	2
4956		4954	4956	-	5074	-	-		-	1	1	2
4961		4959	4962	-	4857		4855	4857	+	1	1	2
4967		4965	4967	+	4982	-	-		+	1	1	2
4971		4971	4974	-	5045	-	-		-	1	1	2
4973		4973	4975	+	5112	-	-		+	1	1	2
4976		4976	4979	-	5003		5003	5004	+	1	1	2
4978		4978	4980	+	5051	-	-		+	2	0	2
4978		4978	4980	+	5066	-	-		+	1	1	2
4982		4980	4986	-	5825		5822	5825	-	1	1	2
4988		4987	4992	+	4914	-	-		-	1	1	2
4988		4987	4992	+	5022	-	-		+	1	1	2
4988		4987	4992	+	5265	-	-		+	1	1	2
4990		4990	4993	-	5018	-	-		+	1	1	2
4990		4990	4993	-	5189	-	-		-	1	1	2
4990		4990	4993	-	5198	-	-		-	1	1	2
5002		5001	5002	-	5007	-	-		+	1	1	2
5009		5006	5013	-	5133	-	-		-	1	1	2
5009		5006	5013	-	5309	-	-		-	1	1	2
5017		5014	5021	-	5156	-	-		-	2	0	2
5024	-	-		-	5120	-	-		+	1	1	2
5029		5025	5034	-	5134	-	-		+	1	1	2
5029		5025	5034	-	5135	-	-		-	1	1	2
5029		5025	5034	-	5147	-	-		+	1	1	2
5029		5025	5034	-	5162	-	-		-	0	2	2
5029		5025	5034	-	5171	-	-		-	1	1	2
5039		5036	5041	-	5078	-	-		+	1	1	2
5039		5036	5041	-	5110	-	-		+	1	1	2
5039		5036	5041	-	5236	-	-		+	1	1	2
5039		5036	5041	-	5291	-	-		-	1	1	2
5039		5036	5041	-	5316	-	-		-	1	1	2
5039		5036	5041	-	6969	-	-		-	2	0	2
5046		5043	5047	-	5125	-	-		-	1	1	2
5046		5043	5047	-	5244	-	-		-	1	1	2
5049	-	-		+	5031	-	-		-	2	0	2
5053		5049	5055	-	5174		5171	5174	-	1	1	2
5060		5060	5061	-	15101		15101	15102	+	2	0	2
5065		5065	5070	-	5083		5081	5083	+	1	1	2
5065		5065	5070	-	5877	-	-		-	1	1	2
5078		5076	5082	-	5103	-	-		+	1	1	2
5078		5076	5082	-	5216	-	-		-	1	1	2
5078		5076	5082	-	5231	-	-		-	1	1	2
5078		5076	5082	-	5246	-	-		-	1	1	2
5078		5076	5082	-	5259	-	-		-	1	1	2
5078		5076	5082	-	5283		5283	5284	+	1	1	2
5078		5076	5082	-	5301	-	-		-	1	1	2
5094		5093	5096	+	5531	-	-		-	0	2	2
5096		5092	5099	-	5240	-	-		-	1	1	2
5096		5092	5099	-	5268	-	-		-	1	1	2
5096		5092	5099	-	5817	-	-		-	1	1	2
5100		5100	5102	+	5522	-	-		-	2	0	2
5101		5101	5106	-	5216	-	-		-	1	1	2
5101		5101	5106	-	5287	-	-		-	1	1	2
5101		5101	5106	-	15056	-	-		+	0	2	2
5108		5107	5108	-	5168		5168	5169	+	1	1	2
5108		5107	5108	-	5269	-	-		+	1	1	2
5110		5108	5110	+	5137	-	-		+	1	1	2
5113		5111	5114	-	5259	-	-		-	1	1	2
5113		5111	5114	-	5344	-	-		-	1	1	2
5113		5111	5114	-	5449	-	-		-	1	1	2
5122		5118	5124	-	5227		5227	5228	+	1	1	2
5122		5118	5124	-	5259		5257	5259	-	1	1	2
5122		5118	5124	-	5288	-	-		-	1	1	2
5122		5118	5124	-	5349	-	-		-	0	2	2
5122		5118	5124	-	5520		5520	5521	+	1	1	2
5122		5118	5124	-	6657	-	-		-	1	1	2
5123		5122	5123	+	6280	-	-		+	1	1	2
5139		5134	5140	+	5101	-	-		-	1	1	2
5139		5134	5140	+	5654		5654	5655	+	1	1	2
5147		5143	5150	+	5127	-	-		-	1	1	2
5148		5145	5148	-	5303	-	-		-	1	1	2
5148		5145	5148	-	5315	-	-		-	1	1	2
5148		5145	5148	-	6682	-	-		+	0	2	2
5153	-	-		+	9575	-	-		+	1	1	2
5155		5150	5156	-	5184		5180	5184	+	1	1	2
5155		5150	5156	-	5284	-	-		+	1	1	2
5155		5150	5156	-	6679		6679	6680	+	1	1	2
5162		5161	5165	-	14627	-	-		+	0	2	2
5170		5167	5171	-	5285	-	-		-	1	1	2
5170		5169	5170	+	7090	-	-		+	1	1	2
5176		5176	5178	+	5654		5654	5655	+	1	1	2
5176		5176	5178	+	15098	-	-		-	1	1	2
5181		5178	5184	-	5586	-	-		-	1	1	2
5181		5178	5184	-	5602	-	-		-	1	1	2
5186		5186	5189	+	5261	-	-		+	1	1	2
5186		5186	5189	+	5284	-	-		-	1	1	2
5186		5186	5189	+	13687		13687	13688	+	1	1	2
5190		5188	5190	-	5319	-	-		-	1	1	2
5197		5197	5199	-	5318	-	-		-	1	1	2
5197		5197	5199	-	5343	-	-		-	1	1	2

5197	5197	5199	-	5360	-	-	-	1	1	2
5203	-	-	+	5132	-	-	+	1	1	2
5203	-	-	+	5271	-	-	-	1	1	2
5213	5209	5218	-	5377	-	-	-	1	1	2
5213	5209	5218	-	5626	-	-	-	1	1	2
5213	5209	5218	-	5641	-	-	-	1	1	2
5225	-	-	+	5259	-	-	-	0	2	2
5229	-	-	-	5275	-	-	+	1	1	2
5233	5230	5238	+	5185	-	-	-	1	1	2
5233	5230	5238	+	5254	-	-	-	2	0	2
5233	5230	5238	+	5652	5652	5653	+	1	1	2
5236	5233	5238	-	5271	5268	5271	+	1	1	2
5236	5233	5238	-	5602	-	-	-	1	1	2
5236	5233	5238	-	5652	-	-	-	1	1	2
5243	5240	5244	+	5220	-	-	-	1	1	2
5243	5240	5244	+	5248	-	-	-	0	2	2
5243	5240	5244	+	5283	5283	5284	-	1	1	2
5248	-	-	+	5243	-	-	-	2	0	2
5248	-	-	+	15084	-	-	-	1	1	2
5253	5253	5257	+	5167	5167	5168	-	1	1	2
5253	5253	5257	+	5182	-	-	-	1	1	2
5256	-	-	-	5286	-	-	+	2	0	2
5259	5259	5263	+	5527	-	-	-	1	1	2
5261	5258	5267	-	5376	-	-	-	1	1	2
5261	5258	5267	-	5510	-	-	-	1	1	2
5261	5258	5267	-	5535	-	-	-	1	1	2
5261	5258	5267	-	5540	-	-	-	1	1	2
5261	5258	5267	-	5574	-	-	-	1	1	2
5265	5265	5268	+	5348	-	-	-	1	1	2
5265	5265	5268	+	5527	-	-	+	1	1	2
5265	5265	5268	+	14013	-	-	+	1	1	2
5277	5273	5281	-	5413	-	-	-	1	1	2
5277	5273	5281	-	5484	-	-	-	1	1	2
5284	5283	5284	-	5901	-	-	-	2	0	2
5284	-	-	+	5504	-	-	-	1	1	2
5291	5288	5293	-	5602	-	-	-	1	1	2
5291	5288	5293	-	5906	-	-	-	0	2	2
5297	5295	5300	+	5321	-	-	-	1	1	2
5297	5295	5300	+	5489	5489	5490	+	1	1	2
5297	5295	5300	+	5498	5498	5499	+	1	1	2
5298	-	-	-	5521	-	-	-	1	1	2
5307	-	-	-	5421	-	-	+	1	1	2
5307	-	-	-	5485	-	-	-	2	0	2
5307	5307	5312	+	5453	-	-	+	1	1	2
5307	5307	5312	+	5492	5492	5493	+	1	1	2
5307	5307	5312	+	5500	-	-	-	1	1	2
5313	5309	5317	-	5342	5342	5343	-	1	1	2
5313	5309	5317	-	5498	-	-	-	1	1	2
5313	5309	5317	-	5588	-	-	-	1	1	2
5313	5309	5317	-	5721	-	-	-	1	1	2
5313	5309	5317	-	5740	-	-	+	2	0	2
5323	5319	5326	-	5523	5521	5523	-	1	1	2
5323	5319	5326	-	5640	-	-	-	1	1	2
5323	5319	5326	-	5735	-	-	+	0	2	2
5326	5325	5328	+	5353	-	-	+	1	1	2
5326	5325	5328	+	5458	5458	5459	+	1	1	2
5337	5337	5340	-	5492	-	-	-	1	1	2
5337	5337	5340	-	5516	-	-	-	1	1	2
5337	5337	5340	-	5536	5536	5537	-	1	1	2
5344	5344	5346	+	12328	12328	12329	+	1	1	2
5349	-	-	+	5382	-	-	-	1	1	2
5350	-	-	-	5499	-	-	-	1	1	2
5355	5354	5359	-	5532	-	-	+	1	1	2
5355	5354	5359	-	5534	-	-	-	1	1	2
5355	5354	5359	-	5585	5585	5586	-	1	1	2
5357	-	-	+	5428	-	-	+	1	1	2
5366	5364	5366	+	5364	-	-	+	1	1	2
5366	5364	5366	+	5379	5379	5380	+	1	1	2
5371	5368	5373	-	5491	-	-	-	1	1	2
5371	5368	5373	-	5509	-	-	-	1	1	2
5380	5379	5384	+	5313	-	-	+	1	1	2
5380	5379	5384	+	5409	-	-	-	1	1	2
5380	5379	5384	+	5463	5463	5464	-	1	1	2
5384	5384	5386	-	5460	-	-	+	1	1	2
5384	5384	5386	-	5597	-	-	-	1	1	2
5384	5384	5386	-	7122	-	-	-	1	1	2
5386	-	-	+	5407	-	-	-	1	1	2
5392	5392	5393	-	5546	-	-	-	1	1	2
5398	5398	5400	+	5344	-	-	-	1	1	2
5398	5398	5400	+	5448	5448	5449	-	1	1	2
5412	5408	5416	+	5452	-	-	+	1	1	2
5443	5443	5447	-	5496	-	-	+	1	1	2
5443	5443	5447	-	5615	-	-	-	1	1	2
5443	5443	5447	-	5624	-	-	-	1	1	2
5443	5443	5447	-	5636	-	-	-	1	1	2
5443	5443	5447	-	12423	-	-	+	2	0	2
5445	5441	5445	+	5664	-	-	-	1	1	2
5445	5441	5445	+	15333	15333	15334	+	1	1	2
5459	5457	5461	-	5597	-	-	-	1	1	2
5459	5457	5461	-	5625	-	-	-	1	1	2
5467	5462	5470	-	5597	-	-	-	1	1	2
5467	5462	5470	-	5663	-	-	-	1	1	2
5467	5462	5470	-	10451	-	-	-	1	1	2
5476	5471	5479	-	5525	-	-	+	1	1	2
5476	5471	5479	-	6178	-	-	-	0	2	2
5476	5471	5479	-	10297	10294	10297	+	0	2	2
5476	5471	5479	-	12308	-	-	+	0	2	2
5476	5471	5479	-	12997	-	-	+	0	2	2

5481	5481	5483 -	5624 -	-	-	1	1	2
5481	5481	5483 -	5634 -	-	-	1	1	2
5481	5481	5483 -	5666 -	-	-	1	1	2
5482 -	-	+	5647 -	-	-	1	1	2
5497 -	-	+	5446 -	-	-	1	1	2
5504	5500	5507 -	5560	5560	5561 +	1	1	2
5504	5500	5507 -	5568	5565	5568 +	1	1	2
5504	5500	5507 -	5635	5633	5635 +	1	1	2
5504	5500	5507 -	10932 -	-	+	2	0	2
5513	5512	5514 -	5649 -	-	+	1	1	2
5518	5517	5519 -	5655 -	-	-	1	1	2
5518	5517	5519 -	10920	10920	10921 +	0	2	2
5529 -	-	-	5670 -	-	-	1	1	2
5540	5539	5540 -	6022	6022	6023 -	1	1	2
5545	5543	5548 -	5623 -	-	-	1	1	2
5558	5554	5560 -	5630 -	-	+	1	1	2
5558	5554	5560 -	5735 -	-	-	1	1	2
5562	5562	5566 +	5539 -	-	-	1	1	2
5562	5562	5566 +	5591	5588	5591 -	1	1	2
5563	5563	5566 -	5897 -	-	-	1	1	2
5575	5575	5579 -	5667 -	-	-	1	1	2
5575	5575	5579 -	5756 -	-	-	1	1	2
5577	5576	5578 +	5693 -	-	+	1	1	2
5577	5576	5578 +	6483 -	-	+	1	1	2
5584	5583	5584 -	5642 -	-	+	1	1	2
5589 -	-	-	5689 -	-	+	1	1	2
5590	5589	5590 +	5557	5555	5557 -	1	1	2
5601 -	-	+	6000 -	-	+	1	1	2
5606 -	-	+	5662 -	-	+	1	1	2
5607	5605	5607 -	5663 -	-	+	1	1	2
5607	5605	5607 -	5811 -	-	-	1	1	2
5607	5605	5607 -	6053 -	-	-	1	1	2
5612	5609	5612 -	5746 -	-	-	1	1	2
5617	5613	5619 +	5640 -	-	+	1	1	2
5617	5613	5619 +	5670	5666	5670 -	1	1	2
5630 -	-	-	5737 -	-	+	1	1	2
5630 -	-	+	5737 -	-	-	1	1	2
5644	5642	5644 +	5724	5724	5725 -	1	1	2
5647	5643	5647 -	5808 -	-	-	1	1	2
5647	5643	5647 -	5882	5882	5883 -	1	1	2
5652	5651	5653 -	5727 -	-	+	1	1	2
5652	5651	5653 -	5838 -	-	-	1	1	2
5652	5651	5653 -	5990 -	-	+	0	2	2
5658	5657	5662 -	5743 -	-	+	2	0	2
5658	5657	5662 -	5761 -	-	-	1	1	2
5658	5657	5662 -	5769 -	-	+	1	1	2
5666 -	-	+	5619 -	-	-	1	1	2
5667	5663	5669 -	5739 -	-	+	0	2	2
5667	5663	5669 -	5779 -	-	-	1	1	2
5667	5663	5669 -	5918 -	-	+	1	1	2
5695 -	-	-	5723 -	-	+	1	1	2
5710	5707	5710 -	5691 -	-	+	2	0	2
5710	5707	5710 -	5847	5845	5847 +	1	1	2
5710	5707	5710 -	5981 -	-	-	1	1	2
5720 -	-	+	5706 -	-	-	1	1	2
5722	5720	5726 -	5838 -	-	-	1	1	2
5722	5720	5726 -	5864	5864	5865 -	1	1	2
5722	5720	5726 -	5883 -	-	-	1	1	2
5722	5720	5726 -	5905 -	-	-	1	1	2
5722	5720	5726 -	5924 -	-	-	1	1	2
5722	5720	5726 -	5971 -	-	-	1	1	2
5730	5730	5736 -	5791 -	-	+	1	1	2
5730	5730	5736 -	5806	5806	5807 +	1	1	2
5730	5730	5736 -	5877 -	-	-	1	1	2
5730	5730	5736 -	5965	5962	5965 -	1	1	2
5735	5735	5737 +	5882 -	-	+	1	1	2
5740	5740	5744 -	5768 -	-	-	1	1	2
5740	5740	5744 -	5774 -	-	+	1	1	2
5740	5740	5744 -	5802 -	-	+	1	1	2
5747	5747	5751 +	5660	5660	5661 -	1	1	2
5759 -	-	-	7263 -	-	-	1	1	2
5763	5761	5765 +	5665	5665	5666 -	1	1	2
5763	5761	5765 +	5708 -	-	+	1	1	2
5768	5767	5769 +	5849 -	-	+	1	1	2
5768	5767	5769 +	5887 -	-	-	1	1	2
5768	5767	5769 +	6097 -	-	+	1	1	2
5771	5771	5775 -	6119 -	-	-	1	1	2
5777	5777	5781 -	5806 -	-	+	1	1	2
5780 -	-	+	5808 -	-	+	1	1	2
5787	5787	5788 +	5821	5821	5822 -	1	1	2
5794	5791	5798 -	5884 -	-	+	1	1	2
5802	5802	5803 -	6276	6276	6277 +	1	1	2
5808	5808	5809 -	5855 -	-	-	1	1	2
5808	5808	5809 -	5870 -	-	+	2	0	2
5815	5811	5819 -	5864 -	-	+	0	2	2
5815	5811	5819 -	5967 -	-	-	1	1	2
5815	5811	5819 -	6061 -	-	-	1	1	2
5827	5823	5831 -	5889 -	-	+	1	1	2
5827	5823	5831 -	6063 -	-	-	1	1	2
5833	5833	5834 +	5859	5859	5860 +	1	1	2
5833	5833	5834 +	5891 -	-	+	1	1	2
5837	5835	5837 -	5865 -	-	+	2	0	2
5837	5835	5837 -	6068 -	-	-	1	1	2
5843 -	-	+	5810 -	-	-	1	1	2
5845	5842	5845 -	5860 -	-	+	0	2	2
5845	5842	5845 -	6014 -	-	-	1	1	2
5850	5850	5854 +	5875 -	-	-	1	1	2
5850	5850	5854 +	6995 -	-	+	1	1	2

5851	5851	5855	-	6020	-	-	-	1	1	2
5861	5857	5862	-	5965	-	-	-	1	1	2
5861	5857	5862	-	6045	-	-	-	1	1	2
5861	5857	5862	-	6059	-	-	-	1	1	2
5867	5867	5869	+	5889	5889	5890	-	1	1	2
5883	5880	5883	+	5906	-	-	+	1	1	2
5886	-	-	-	6014	-	-	-	1	1	2
5888	5885	5888	+	5896	-	-	-	0	2	2
5888	5885	5888	+	10202	-	-	-	1	1	2
5892	5889	5895	-	5891	5891	5892	+	1	1	2
5892	5889	5895	-	6008	-	-	-	1	1	2
5897	5896	5897	+	5885	-	-	-	2	0	2
5897	5896	5897	+	5944	-	-	-	1	1	2
5898	5898	5902	-	6027	-	-	-	1	1	2
5921	5917	5922	-	6085	-	-	-	1	1	2
5921	5917	5922	-	6100	6097	6100	-	1	1	2
5926	5926	5930	-	6067	-	-	-	1	1	2
5936	-	-	-	5939	-	-	+	2	0	2
5942	5942	5943	-	6059	-	-	+	1	1	2
5942	5942	5943	-	6520	-	-	-	1	1	2
5945	-	-	+	6009	-	-	-	1	1	2
5953	5950	5957	-	6029	-	-	+	1	1	2
5968	5965	5968	-	5998	5995	5998	+	1	1	2
5971	5967	5974	+	6279	6275	6279	+	1	1	2
6022	6022	6025	+	6101	6098	6101	-	1	1	2
6024	6022	6025	-	6059	-	-	-	1	1	2
6036	-	-	+	5972	-	-	-	1	1	2
6041	6041	6042	-	6285	6285	6286	-	1	1	2
6060	6059	6064	-	6082	-	-	+	1	1	2
6060	6059	6064	-	6232	-	-	-	1	1	2
6060	6059	6064	-	6273	-	-	+	1	1	2
6077	6073	6078	+	11993	11993	11994	-	1	1	2
6082	6082	6083	-	6194	-	-	-	1	1	2
6090	6090	6093	-	6288	-	-	-	1	1	2
6091	6088	6095	+	6017	-	-	-	1	1	2
6091	6088	6095	+	6097	-	-	+	1	1	2
6091	6088	6095	+	14439	-	-	-	1	1	2
6097	6096	6100	-	6190	-	-	+	1	1	2
6097	6096	6100	-	6221	6221	6222	-	1	1	2
6097	6096	6100	-	6298	-	-	-	1	1	2
6105	-	-	-	8625	-	-	-	0	2	2
6113	6111	6113	-	6231	-	-	-	1	1	2
6113	6111	6113	-	6298	-	-	-	1	1	2
6118	6118	6123	-	6561	6561	6562	-	1	1	2
6119	6119	6122	+	6143	6143	6144	-	1	1	2
6146	6144	6146	-	6323	-	-	-	1	1	2
6146	6144	6146	-	6860	6860	6861	-	1	1	2
6146	6146	6147	+	6148	6148	6149	-	1	1	2
6153	6153	6154	+	6184	6184	6185	-	2	0	2
6154	-	-	-	6252	-	-	-	1	1	2
6160	6159	6160	-	6336	-	-	-	1	1	2
6170	6170	6171	-	6229	-	-	-	1	1	2
6178	-	-	+	6224	6224	6225	-	1	1	2
6183	6181	6186	-	11139	-	-	+	0	2	2
6185	-	-	+	6153	-	-	-	2	0	2
6196	6194	6199	-	6216	-	-	+	2	0	2
6203	6203	6204	-	6211	-	-	+	0	2	2
6211	6211	6212	-	11117	-	-	+	0	2	2
6215	6214	6216	+	6203	6203	6204	+	2	0	2
6215	6214	6216	+	6751	6751	6752	-	1	1	2
6223	6219	6223	-	6319	6319	6320	+	1	1	2
6223	6219	6223	-	6356	-	-	-	1	1	2
6228	6226	6231	-	6272	6270	6272	+	1	1	2
6228	6226	6231	-	6287	6284	6287	-	1	1	2
6228	6226	6231	-	6380	-	-	-	1	1	2
6228	6226	6231	-	6441	-	-	-	1	1	2
6229	6229	6233	+	6389	6389	6390	-	1	1	2
6238	6234	6242	-	6284	6282	6284	+	1	1	2
6238	6234	6242	-	6382	-	-	-	1	1	2
6238	6234	6242	-	6408	-	-	-	1	1	2
6238	6234	6242	-	6425	-	-	-	1	1	2
6238	6234	6242	-	6436	-	-	-	1	1	2
6238	6234	6242	-	6491	-	-	-	1	1	2
6238	6234	6242	-	6558	6558	6559	-	1	1	2
6249	6247	6249	-	6349	-	-	-	1	1	2
6249	6247	6249	-	6361	-	-	-	1	1	2
6249	6247	6249	-	6373	6373	6374	-	1	1	2
6255	6252	6257	-	6348	-	-	-	1	1	2
6260	6260	6261	+	6373	-	-	-	1	1	2
6260	6260	6261	+	6385	-	-	-	1	1	2
6263	6262	6267	-	6382	6378	6382	+	2	0	2
6263	6262	6267	-	6390	-	-	-	1	1	2
6273	6270	6275	-	6389	-	-	+	1	1	2
6273	6270	6275	-	6398	6398	6399	-	1	1	2
6273	6270	6275	-	6438	-	-	-	1	1	2
6273	6270	6275	-	6580	-	-	-	1	1	2
6273	6270	6275	-	6603	6599	6603	-	1	1	2
6280	6280	6283	-	6408	-	-	-	1	1	2
6287	-	-	+	11729	-	-	-	1	1	2
6292	6288	6292	-	6415	-	-	-	1	1	2
6308	6308	6312	-	6585	6581	6585	-	1	1	2
6331	6331	6332	+	6425	6425	6426	-	1	1	2
6338	6335	6338	+	6422	-	-	-	1	1	2
6338	6335	6338	+	6562	-	-	+	1	1	2
6342	6338	6346	-	6493	-	-	-	1	1	2
6345	-	-	+	6383	-	-	-	1	1	2
6351	6347	6352	+	6441	-	-	+	1	1	2
6364	6364	6366	+	6386	-	-	+	1	1	2

6364	6364	6366 +	6411	6411	6412 +	1	1	2
6368	6366	6368 -	6639	6636	6639 -	1	1	2
6368	6366	6368 -	10354 -	-	+	1	1	2
6374	6373	6374 -	6549 -	-	-	1	1	2
6378 -	-	+	6476	6476	6477 -	1	1	2
6383	6383	6384 +	6324	6324	6325 -	1	1	2
6383	6383	6384 +	6329	6329	6330 -	1	1	2
6383	6383	6384 +	6334 -	-	-	2	0	2
6386	6384	6390 -	6470 -	-	-	1	1	2
6390	6385	6390 +	6258	6256	6258 -	1	1	2
6390	6385	6390 +	6379 -	-	-	1	1	2
6390	6385	6390 +	6439 -	-	-	1	1	2
6394	6394	6398 -	6457 -	-	+	1	1	2
6401	6401	6402 +	6479 -	-	-	1	1	2
6412	6412	6413 +	6435	6435	6436 +	1	1	2
6414 -	-	-	6614 -	-	+	1	1	2
6427	6423	6428 +	6441	6441	6442 +	1	1	2
6435	6434	6438 -	6588	6588	6589 -	1	1	2
6445	6443	6445 -	6642 -	-	-	1	1	2
6450 -	-	-	6669 -	-	-	1	1	2
6451	6448	6454 +	6420 -	-	-	1	1	2
6451	6448	6454 +	6666 -	-	-	1	1	2
6455	6455	6457 -	6624 -	-	-	1	1	2
6455	6455	6457 -	6668 -	-	-	1	1	2
6455	6455	6457 -	7416 -	-	-	1	1	2
6464	6463	6467 -	6573 -	-	-	1	1	2
6464	6463	6467 -	6835 -	-	-	1	1	2
6481	6481	6483 -	6655 -	-	-	1	1	2
6481	6481	6483 -	6665 -	-	-	1	1	2
6489	6488	6489 +	6402 -	-	-	1	1	2
6489	6488	6489 +	6453	6453	6454 -	1	1	2
6518	6513	6520 -	6582	6582	6583 +	1	1	2
6518	6513	6520 -	6684 -	-	-	1	1	2
6518	6513	6520 -	6705 -	-	-	1	1	2
6518	6513	6520 -	6718 -	-	-	1	1	2
6523	6523	6527 -	6607 -	-	-	1	1	2
6533	6531	6533 -	6614 -	-	+	1	1	2
6533	6531	6533 -	6655 -	-	-	1	1	2
6538	6535	6539 -	6683	6683	6684 -	1	1	2
6538	6535	6539 -	6706 -	-	-	1	1	2
6538	6535	6539 -	7257 -	-	-	1	1	2
6540	6536	6540 +	8434	8430	8434 +	2	0	2
6543	6541	6543 -	6653 -	-	-	1	1	2
6551	6551	6555 -	6614 -	-	+	1	1	2
6551	6551	6555 -	6641 -	-	+	1	1	2
6551	6551	6555 -	6654 -	-	-	1	1	2
6551	6551	6555 -	6668 -	-	-	1	1	2
6558	6556	6561 +	8284 -	-	-	2	0	2
6559	6559	6560 -	6584 -	-	+	1	1	2
6559	6559	6560 -	6785 -	-	+	1	1	2
6566	6563	6566 +	6762	6759	6762 -	1	1	2
6567	6563	6570 -	6595	6593	6595 +	1	1	2
6567	6563	6570 -	11152 -	-	+	1	1	2
6573	6573	6574 +	6563 -	-	-	1	1	2
6573	6573	6574 +	6752 -	-	-	1	1	2
6573	6573	6574 +	8271 -	-	-	2	0	2
6580	6577	6580 -	6746 -	-	-	1	1	2
6586	6586	6588 -	6675 -	-	+	1	1	2
6586	6586	6588 -	6857 -	-	+	1	1	2
6614	6611	6615 -	6695	6695	6696 -	1	1	2
6614	6611	6615 -	6722 -	-	-	1	1	2
6614	6611	6615 -	7561 -	-	-	1	1	2
6618	6614	6619 +	6603 -	-	-	1	1	2
6626	6626	6627 -	6710	6710	6711 +	1	1	2
6644 -	-	-	6700 -	-	+	1	1	2
6650	6650	6653 -	6664 -	-	+	1	1	2
6677 -	-	-	6832 -	-	-	1	1	2
6686	6684	6687 +	6650 -	-	-	1	1	2
6686	6684	6687 +	6828 -	-	-	1	1	2
6686	6684	6687 +	7731 -	-	-	1	1	2
6691	6689	6691 -	6726 -	-	+	1	1	2
6691	6689	6691 -	6745 -	-	+	1	1	2
6699	6699	6703 +	6637 -	-	-	1	1	2
6710 -	-	-	6948 -	-	-	2	0	2
6726	6722	6727 +	6692 -	-	-	1	1	2
6726	6722	6727 +	6743 -	-	-	1	1	2
6732	6732	6734 -	6805	6805	6806 +	1	1	2
6732	6732	6734 -	7004 -	-	-	1	1	2
6732	6732	6734 +	6805 -	-	-	1	1	2
6732	6732	6734 +	7316 -	-	+	1	1	2
6743	6743	6746 +	6723 -	-	-	1	1	2
6743	6743	6746 +	6840 -	-	-	1	1	2
6749	6744	6749 -	6950	6950	6951 -	1	1	2
6749	6744	6749 -	7121 -	-	-	1	1	2
6755	6755	6760 -	6930 -	-	-	1	1	2
6755	6755	6760 -	8346	8346	8347 -	1	1	2
6782 -	-	-	6902 -	-	-	1	1	2
6799	6799	6802 -	6887 -	-	-	1	1	2
6799	6799	6802 -	6915 -	-	-	1	1	2
6813	6809	6818 +	6855 -	-	-	1	1	2
6815	6815	6819 -	6960 -	-	-	1	1	2
6815	6815	6819 -	7138 -	-	-	1	1	2
6840	6837	6844 +	6906	6906	6907 +	1	1	2
6850	6846	6853 +	6706 -	-	-	1	1	2
6850	6846	6853 +	6817 -	-	-	1	1	2
6850	6846	6853 +	6849	6849	6850 -	1	1	2
6850	6846	6853 +	6938 -	-	-	1	1	2
6855 -	-	+	6885 -	-	-	1	1	2

6857	6852	6858	-	6965	6965	6966	+	1	1	2
6857	6852	6858	-	6969	-	-	-	1	1	2
6857	6852	6858	-	6986	-	-	-	1	1	2
6860	6860	6862	+	6784	-	-	+	1	1	2
6864	6861	6868	-	6979	-	-	+	1	1	2
6864	6861	6868	-	7003	-	-	-	1	1	2
6864	6861	6868	-	7087	-	-	-	1	1	2
6882	6878	6883	+	6910	6910	6911	-	1	1	2
6896	6894	6896	-	7093	-	-	-	1	1	2
6896	6894	6896	-	7164	-	-	-	1	1	2
6904	-	-	-	7031	-	-	-	1	1	2
6917	6915	6917	-	7026	-	-	-	1	1	2
6922	6919	6923	-	7005	7001	7005	-	1	1	2
6922	6919	6923	-	7018	-	-	+	1	1	2
6928	6928	6929	-	7122	-	-	-	1	1	2
6928	6928	6929	-	7138	-	-	-	1	1	2
6930	6928	6931	+	6916	6913	6916	-	1	1	2
6930	6928	6931	+	15374	15374	15375	-	1	1	2
6935	6934	6939	-	7139	-	-	+	1	1	2
6947	6947	6951	-	7047	-	-	+	1	1	2
6947	6947	6951	-	7156	-	-	-	1	1	2
6949	-	-	+	13057	-	-	+	1	1	2
6956	6953	6960	+	7004	7001	7004	+	1	1	2
6956	6953	6960	+	7267	-	-	+	1	1	2
6967	-	-	+	6997	-	-	+	1	1	2
6976	6972	6979	-	7008	-	-	+	1	1	2
6976	6972	6979	-	7125	7122	7125	-	1	1	2
6986	6986	6989	+	6956	-	-	-	1	1	2
6988	6988	6993	-	7159	-	-	-	1	1	2
6988	6988	6993	-	7186	-	-	-	1	1	2
7001	6997	7006	-	7114	-	-	-	1	1	2
7001	6997	7006	-	7122	-	-	-	1	1	2
7008	7008	7012	-	7099	7099	7100	-	1	1	2
7008	7008	7012	-	7314	-	-	-	1	1	2
7011	7011	7016	+	6924	6924	6925	-	1	1	2
7011	7011	7016	+	7094	-	-	-	1	1	2
7017	7017	7021	-	7099	-	-	+	1	1	2
7017	7017	7021	-	7118	7116	7118	-	1	1	2
7017	7017	7021	-	7165	-	-	-	1	1	2
7017	7017	7021	-	7209	-	-	+	1	1	2
7017	7017	7021	-	7306	-	-	-	1	1	2
7029	-	-	-	7075	-	-	+	1	1	2
7034	7034	7037	+	7101	-	-	-	1	1	2
7043	7042	7044	-	7260	7256	7260	-	1	1	2
7043	7042	7044	-	10650	-	-	+	1	1	2
7043	7041	7043	+	7224	-	-	+	1	1	2
7051	7051	7055	-	10643	10643	10644	+	1	1	2
7054	7051	7054	+	7325	-	-	-	1	1	2
7061	7058	7064	-	7120	-	-	+	1	1	2
7061	7058	7064	-	7643	-	-	+	1	1	2
7061	7058	7064	-	10632	-	-	+	1	1	2
7067	-	-	+	7206	-	-	+	1	1	2
7069	7066	7069	-	7116	-	-	-	1	1	2
7074	7071	7077	-	7252	-	-	-	1	1	2
7079	-	-	+	7351	-	-	+	1	1	2
7088	7081	7091	-	7145	-	-	-	1	1	2
7088	7081	7091	-	7294	7291	7294	-	1	1	2
7096	7094	7096	+	7202	7200	7202	-	1	1	2
7097	7093	7102	-	7232	-	-	-	1	1	2
7097	7093	7102	-	7276	-	-	-	1	1	2
7097	7093	7102	-	7312	-	-	-	1	1	2
7097	7093	7102	-	7322	-	-	+	0	2	2
7097	7093	7102	-	9852	-	-	+	1	1	2
7105	7105	7108	+	7288	-	-	-	1	1	2
7106	7103	7110	-	7241	-	-	-	1	1	2
7106	7103	7110	-	7309	-	-	-	1	1	2
7106	7103	7110	-	7330	-	-	-	1	1	2
7118	-	-	+	7260	-	-	-	1	1	2
7123	7120	7124	-	7804	-	-	-	1	1	2
7130	7129	7133	-	7176	-	-	+	1	1	2
7130	7129	7133	-	7273	7270	7273	-	1	1	2
7130	7129	7133	-	7290	-	-	-	1	1	2
7135	7134	7137	-	7293	-	-	+	1	1	2
7135	7134	7137	-	7356	7356	7357	-	1	1	2
7151	7148	7151	-	7324	-	-	-	1	1	2
7151	7148	7151	-	7416	-	-	-	1	1	2
7157	7157	7158	+	7450	7450	7451	+	1	1	2
7167	7164	7167	+	7202	-	-	-	1	1	2
7167	7164	7167	+	7220	-	-	-	1	1	2
7167	7164	7167	+	7250	7250	7251	+	1	1	2
7168	-	-	-	7263	-	-	-	1	1	2
7173	7173	7176	+	7192	7189	7192	-	1	1	2
7176	7176	7180	-	7203	-	-	+	1	1	2
7176	7176	7180	-	7300	-	-	-	1	1	2
7181	-	-	+	7188	-	-	-	1	1	2
7191	7190	7195	-	7189	7189	7190	+	2	0	2
7191	7190	7195	-	7248	-	-	+	1	1	2
7191	7190	7195	-	7357	-	-	-	1	1	2
7200	-	-	-	7355	-	-	-	1	1	2
7200	-	-	-	7376	-	-	-	1	1	2
7209	7209	7213	-	7362	-	-	-	1	1	2
7216	-	-	-	7420	-	-	-	1	1	2
7221	7219	7221	-	7255	-	-	+	1	1	2
7221	7219	7221	-	7375	-	-	-	1	1	2
7234	7233	7237	+	7146	7146	7147	-	1	1	2
7234	7233	7237	+	7710	-	-	+	1	1	2
7238	7235	7240	-	7269	7269	7270	-	1	1	2
7238	7235	7240	-	7338	-	-	-	1	1	2

7245	7245	7247 -	7346 -	-	-	1	1	2
7245	7245	7247 -	7403 -	-	-	1	1	2
7245	7245	7247 -	7435 -	-	-	1	1	2
7253	7250	7258 -	7397 -	-	-	1	1	2
7253	7250	7258 -	7404 -	-	-	1	1	2
7253	7250	7258 -	7421 -	-	-	1	1	2
7253	7250	7258 -	7456 -	-	-	1	1	2
7258	7254	7258 +	7219 -	-	-	1	1	2
7263	7262	7263 +	7282 -	-	-	2	0	2
7263	7262	7263 +	7344 -	-	-	1	1	2
7274	7270	7274 -	7349 -	-	-	1	1	2
7274	7270	7274 -	7517 -	-	-	1	1	2
7279	7276	7279 -	7360	7358	7360 -	1	1	2
7279	7276	7279 -	7439 -	-	-	1	1	2
7281	7281	7282 +	7300 -	-	-	1	1	2
7286	7284	7287 +	7423 -	-	-	1	1	2
7291 -	-	+	7400 -	-	-	1	1	2
7292 -	-	-	7507 -	-	+	1	1	2
7300	7300	7302 +	7281 -	-	-	1	1	2
7316	7316	7317 -	7441 -	-	+	1	1	2
7319	7315	7319 +	7401 -	-	-	1	1	2
7332	7332	7336 +	7272 -	-	-	1	1	2
7332	7332	7336 +	7440 -	-	-	2	0	2
7349	7349	7352 -	7784	7784	7785 +	1	1	2
7360	7359	7361 +	7500	7500	7501 -	1	1	2
7363	7359	7363 -	11036 -	-	+	0	2	2
7368	7366	7368 +	7356 -	-	-	1	1	2
7370	7370	7372 -	7527 -	-	-	1	1	2
7376	7376	7379 -	7473 -	-	+	1	1	2
7376	7376	7379 -	7520 -	-	-	1	1	2
7376	7376	7379 -	7527	7527	7528 -	1	1	2
7388	7385	7390 +	7407 -	-	-	1	1	2
7407	7404	7409 -	7588 -	-	-	1	1	2
7407	7404	7409 -	7594 -	-	-	1	1	2
7407 -	-	+	7388 -	-	-	1	1	2
7415	7414	7419 +	12082 -	-	-	2	0	2
7420	7418	7426 -	7506 -	-	+	1	1	2
7420	7418	7426 -	7540 -	-	-	1	1	2
7420	7418	7426 -	7612 -	-	-	1	1	2
7420	7418	7426 -	7635 -	-	-	1	1	2
7420	7418	7426 -	7671	7667	7671 +	1	1	2
7425	7425	7427 +	7358 -	-	-	1	1	2
7429	7429	7431 -	7493 -	-	-	1	1	2
7429	7429	7431 -	7581 -	-	-	1	1	2
7436	7436	7438 -	7535 -	-	-	1	1	2
7436	7436	7438 -	7639 -	-	-	1	1	2
7436	7436	7438 -	7754 -	-	+	1	1	2
7436	7432	7440 +	7362 -	-	-	2	0	2
7441 -	-	-	7636 -	-	+	1	1	2
7452	7448	7455 +	7549 -	-	-	0	2	2
7452	7448	7455 +	7561 -	-	-	1	1	2
7459	7457	7460 -	7480	7477	7480 +	1	1	2
7461	7457	7462 +	7459	7459	7460 -	1	1	2
7461	7457	7462 +	7545 -	-	-	2	0	2
7467	7464	7467 -	7532 -	-	+	1	1	2
7467	7464	7467 -	7590 -	-	+	1	1	2
7469	7465	7472 +	7482 -	-	+	1	1	2
7469	7465	7472 +	7509 -	-	-	1	1	2
7469	7465	7472 +	7515	7515	7516 -	1	1	2
7477	7473	7479 -	7533	7533	7534 +	1	1	2
7477	7473	7479 -	7615 -	-	-	1	1	2
7477	7473	7479 -	7629	7627	7629 -	1	1	2
7477	7473	7479 -	7707 -	-	-	1	1	2
7485	7481	7488 -	7518 -	-	-	1	1	2
7485	7481	7488 -	7629 -	-	-	1	1	2
7496	7496	7497 -	7724	7724	7725 -	1	1	2
7507	7505	7507 +	7471 -	-	-	1	1	2
7512	7512	7515 -	7632 -	-	-	1	1	2
7512	7512	7515 -	7714 -	-	-	1	1	2
7512	7512	7515 -	7723 -	-	-	1	1	2
7517	7513	7517 +	7711 -	-	-	1	1	2
7517	7513	7517 +	8030 -	-	-	1	1	2
7518	7517	7522 -	7538	7534	7538 +	1	1	2
7518	7517	7522 -	7716 -	-	-	1	1	2
7528	7526	7530 -	7580 -	-	+	1	1	2
7528	7526	7530 -	7686 -	-	-	1	1	2
7528	7526	7530 -	7738 -	-	-	1	1	2
7528	7524	7531 +	7605 -	-	+	1	1	2
7528	7524	7531 +	7978 -	-	+	1	1	2
7538	7533	7541 -	7608 -	-	+	1	1	2
7538	7533	7541 -	7641 -	-	-	1	1	2
7538	7533	7541 -	7679 -	-	-	1	1	2
7538	7533	7541 -	7691	7691	7692 -	1	1	2
7538	7533	7541 -	9007 -	-	-	1	1	2
7541	7541	7545 +	7490 -	-	-	1	1	2
7544	7542	7546 -	7679 -	-	-	1	1	2
7544	7542	7546 -	7691 -	-	-	1	1	2
7544	7542	7546 -	7721 -	-	-	1	1	2
7544	7542	7546 -	7791 -	-	+	1	1	2
7549	7549	7552 -	7563 -	-	+	1	1	2
7550	7548	7550 +	7866 -	-	+	1	1	2
7555	7555	7556 +	7574	7574	7575 +	1	1	2
7562	7562	7566 +	7583 -	-	+	1	1	2
7562	7562	7566 +	7692	7689	7692 -	1	1	2
7567	7564	7567 -	7690	7690	7691 +	1	1	2
7571	7570	7573 +	7669 -	-	-	1	1	2
7571	7570	7573 +	7849 -	-	-	1	1	2
7583	7583	7586 -	7755	7755	7756 -	1	1	2

7583		7583	7586	-	7792	-	-		-	1	1	2
7591		7587	7596	-	7597	-	-		+	0	2	2
7591		7587	7596	-	7757		7757	7758	-	1	1	2
7591		7587	7596	-	7774	-	-		-	1	1	2
7591		7587	7596	-	7817	-	-		-	1	1	2
7591		7591	7594	+	7488	-	-		-	1	1	2
7591		7591	7594	+	8030		8028	8030	-	1	1	2
7601		7599	7606	-	7742	-	-		-	1	1	2
7601		7599	7606	-	8030	-	-		-	0	2	2
7610		7607	7610	+	9517		9513	9517	-	0	2	2
7612		7608	7617	-	7679	-	-		-	1	1	2
7612		7608	7617	-	7719		7715	7719	-	1	1	2
7619	-	-		-	7749	-	-		-	1	1	2
7624		7621	7627	-	7713	-	-		-	1	1	2
7624		7621	7627	-	7770	-	-		-	2	0	2
7624		7621	7627	-	7816		7816	7817	-	1	1	2
7624		7621	7627	-	7833	-	-		-	1	1	2
7626		7623	7628	+	7610	-	-		+	1	1	2
7626		7623	7628	+	7626	-	-		-	1	1	2
7626		7623	7628	+	7854	-	-		-	1	1	2
7629		7629	7633	-	7707		7707	7708	-	1	1	2
7629		7629	7633	-	7772	-	-		-	0	2	2
7638		7637	7639	-	7755	-	-		-	1	1	2
7638		7637	7639	-	7792	-	-		-	1	1	2
7638		7637	7639	-	7818	-	-		-	1	1	2
7639		7639	7640	+	7792		7792	7793	-	1	1	2
7643		7642	7643	-	7691		7691	7692	+	1	1	2
7643		7642	7643	-	7859	-	-		-	1	1	2
7649		7649	7650	-	7860		7860	7861	-	1	1	2
7657		7653	7658	-	7684	-	-		+	2	0	2
7657		7653	7658	-	7765	-	-		+	1	1	2
7657		7653	7658	-	7862		7862	7863	-	1	1	2
7661		7661	7665	+	7802		7798	7802	+	1	1	2
7665		7665	7668	-	7806	-	-		-	1	1	2
7665		7665	7668	-	7960	-	-		-	1	1	2
7665		7665	7668	-	8001	-	-		-	1	1	2
7670		7669	7673	-	7739	-	-		+	1	1	2
7670		7669	7673	-	7760	-	-		+	1	1	2
7670		7669	7673	-	7861		7861	7862	-	1	1	2
7683		7680	7686	-	7654	-	-		+	2	0	2
7683		7680	7686	-	7753	-	-		-	1	1	2
7683		7680	7686	-	7861	-	-		-	1	1	2
7683		7680	7686	-	7866	-	-		-	1	1	2
7690		7688	7690	-	7862	-	-		-	1	1	2
7690		7688	7690	-	7894	-	-		-	1	1	2
7706	-	-		+	7666	-	-		-	1	1	2
7714		7710	7715	-	7785	-	-		-	1	1	2
7714		7710	7715	-	7859	-	-		-	1	1	2
7725		7725	7726	+	8198	-	-		-	1	1	2
7733		7731	7733	-	7854	-	-		-	1	1	2
7739		7739	7743	-	7753	-	-		+	1	1	2
7739		7739	7743	-	7878		7874	7878	-	1	1	2
7739		7739	7743	-	7892	-	-		-	1	1	2
7748		7744	7750	-	7852	-	-		-	1	1	2
7748		7744	7750	-	7862	-	-		-	1	1	2
7754		7753	7758	-	7834	-	-		+	1	1	2
7754		7753	7758	-	7998		7995	7998	-	2	0	2
7754		7753	7758	-	8033	-	-		-	1	1	2
7754		7753	7758	-	8145		8145	8146	+	1	1	2
7764		7763	7768	-	8153	-	-		+	1	1	2
7774		7772	7777	+	7724	-	-		-	1	1	2
7779		7779	7780	+	7605		7605	7606	-	1	1	2
7780		7776	7780	-	7763	-	-		+	1	1	2
7787		7787	7791	-	7889	-	-		+	1	1	2
7787		7787	7791	-	7937	-	-		-	1	1	2
7793		7790	7793	+	7760	-	-		-	1	1	2
7800		7797	7804	+	7672	-	-		-	1	1	2
7800		7797	7804	+	7754	-	-		-	1	1	2
7800		7797	7804	+	7781	-	-		-	1	1	2
7800		7797	7804	+	7836	-	-		-	1	1	2
7800		7797	7804	+	7963		7959	7963	+	1	1	2
7802		7800	7802	-	7880	-	-		+	1	1	2
7809		7809	7812	+	7824	-	-		-	1	1	2
7810	-	-		-	7826	-	-		+	1	1	2
7825		7825	7826	-	8236	-	-		+	1	1	2
7825		7825	7826	-	11157	-	-		-	0	2	2
7828		7826	7828	+	11159	-	-		+	2	0	2
7830	-	-		-	11163	-	-		-	0	2	2
7835		7832	7836	-	7944	-	-		-	1	1	2
7835		7832	7836	-	11171		11168	11171	-	0	2	2
7858		7857	7860	-	7972	-	-		-	1	1	2
7858		7857	7860	-	8002	-	-		-	1	1	2
7864	-	-		+	7922	-	-		-	1	1	2
7866		7866	7867	-	7919		7919	7920	+	1	1	2
7882		7880	7882	-	7962	-	-		+	1	1	2
7884		7884	7886	+	8249	-	-		-	1	1	2
7888		7888	7890	-	8028	-	-		-	1	1	2
7888		7888	7890	-	8086	-	-		-	1	1	2
7889	-	-		+	7937	-	-		+	1	1	2
7896		7892	7898	-	7981	-	-		-	1	1	2
7896		7892	7898	-	7996		7993	7996	-	1	1	2
7896		7892	7898	-	8029	-	-		-	1	1	2
7896		7894	7896	+	8000	-	-		-	1	1	2
7896		7894	7896	+	8111	-	-		-	1	1	2
7896		7894	7896	+	8241	-	-		-	1	1	2
7903	-	-		-	8074	-	-		+	1	1	2
7903		7903	7907	+	8227	-	-		-	2	0	2
7910		7910	7911	+	7874		7874	7875	-	1	1	2

7914		7911	7915	-	8036	-	-		+	1	1	2
7921		7921	7922	-	7957	-	-		+	1	1	2
7935	-	-		+	13300	-	-		+	1	1	2
7945		7944	7950	-	13196	-	-		+	1	1	2
7951		7948	7954	+	7927	-	-		-	0	2	2
7951		7948	7954	+	8039		8036	8039	+	1	1	2
7951		7948	7954	+	8059	-	-		-	1	1	2
7951		7948	7954	+	8121	-	-		-	1	1	2
7956		7954	7957	-	7977	-	-		+	1	1	2
7956		7956	7958	+	7869		7869	7870	-	1	1	2
7956		7956	7958	+	7922	-	-		-	2	0	2
7961		7960	7961	+	8002	-	-		-	1	1	2
7962		7959	7967	-	8140	-	-		+	1	1	2
7973		7969	7973	+	7910	-	-		-	1	1	2
7973		7969	7973	+	8022	-	-		-	1	1	2
7979		7974	7979	-	8044	-	-		+	1	1	2
7979		7974	7979	-	8140	-	-		+	1	1	2
7979		7975	7982	+	8022	-	-		-	1	1	2
7984		7984	7986	+	7952	-	-		-	2	0	2
7984		7984	7986	+	8151	-	-		-	2	0	2
7984		7984	7986	+	8156	-	-		-	1	1	2
7992		7987	7996	-	8075	-	-		-	1	1	2
7992		7988	7996	+	7833		7833	7834	-	1	1	2
7992		7988	7996	+	7967	-	-		-	1	1	2
7992		7988	7996	+	8232		8232	8233	-	1	1	2
7992		7988	7996	+	8260		8258	8260	-	1	1	2
8002		8000	8002	+	8067		8065	8067	-	1	1	2
8002		8000	8002	+	8139	-	-		-	1	1	2
8002		8000	8002	+	8148	-	-		-	1	1	2
8004		7999	8007	-	8201		8198	8201	-	2	0	2
8004		7999	8007	-	8212	-	-		-	2	0	2
8004		7999	8007	-	8238	-	-		-	1	1	2
8009		8009	8013	+	7987	-	-		-	1	1	2
8009		8009	8013	+	8060	-	-		-	1	1	2
8012		8009	8012	-	8199	-	-		-	1	1	2
8022		8018	8025	+	8234	-	-		-	1	1	2
8027		8023	8027	-	8049		8046	8049	+	1	1	2
8030		8028	8031	+	7974	-	-		-	1	1	2
8030		8028	8031	+	8088		8088	8089	-	1	1	2
8030		8028	8031	+	8099	-	-		+	1	1	2
8043		8043	8046	+	7991	-	-		-	1	1	2
8043		8043	8046	+	8064	-	-		-	1	1	2
8044		8041	8048	-	8073		8073	8074	+	1	1	2
8044		8041	8048	-	8094		8090	8094	-	1	1	2
8044		8041	8048	-	8261	-	-		-	1	1	2
8053		8053	8055	-	8068	-	-		+	1	1	2
8053		8053	8055	-	8094	-	-		+	1	1	2
8053		8053	8055	-	8198	-	-		-	1	1	2
8053		8049	8054	+	8056	-	-		+	1	1	2
8053		8049	8054	+	8155	-	-		-	1	1	2
8060		8058	8064	-	8111	-	-		+	1	1	2
8060		8058	8064	-	8150	-	-		+	1	1	2
8060		8058	8064	-	8176	-	-		-	1	1	2
8060		8058	8064	-	8200	-	-		+	1	1	2
8060		8058	8064	-	8235	-	-		-	1	1	2
8060		8058	8064	-	8243	-	-		-	1	1	2
8060		8058	8064	-	8297	-	-		-	1	1	2
8065		8062	8067	+	8095	-	-		+	0	2	2
8066		8065	8067	-	8103	-	-		+	1	1	2
8066		8065	8067	-	8198	-	-		-	1	1	2
8071		8071	8073	-	8236	-	-		-	1	1	2
8071		8071	8073	-	8277	-	-		-	1	1	2
8073		8070	8076	+	8101	-	-		+	2	0	2
8081	-	-		+	8040	-	-		-	2	0	2
8093		8090	8095	-	8238		8238	8239	-	1	1	2
8094		8094	8098	+	8004	-	-		+	2	0	2
8094		8094	8098	+	8115	-	-		-	1	1	2
8100	-	-		-	8169	-	-		+	1	1	2
8115		8114	8118	+	8098	-	-		-	1	1	2
8118		8114	8118	-	8153		8153	8154	+	1	1	2
8118		8114	8118	-	8392	-	-		-	1	1	2
8123		8123	8125	-	8139		8139	8140	+	1	1	2
8123		8123	8125	-	8242	-	-		+	1	1	2
8130		8129	8134	-	8138	-	-		+	1	1	2
8130		8129	8134	-	8175	-	-		-	1	1	2
8140		8140	8142	-	8262	-	-		-	1	1	2
8145		8141	8146	+	8121	-	-		-	1	1	2
8145		8141	8146	+	8127	-	-		-	1	1	2
8147		8143	8150	-	8175	-	-		+	1	1	2
8147		8143	8150	-	8198	-	-		-	1	1	2
8147		8143	8150	-	8257		8257	8258	-	1	1	2
8147		8143	8150	-	8297	-	-		-	1	1	2
8147		8143	8150	-	8316	-	-		-	1	1	2
8150		8148	8152	+	7989	-	-		-	1	1	2
8155	-	-		+	8112	-	-		-	1	1	2
8156		8153	8157	-	8198	-	-		+	1	1	2
8156		8153	8157	-	8483	-	-		+	1	1	2
8156		8153	8157	-	8709	-	-		+	1	1	2
8162		8162	8166	-	8235	-	-		-	1	1	2
8176		8176	8177	-	8423	-	-		+	1	1	2
8182	-	-		+	8196	-	-		+	1	1	2
8184		8180	8184	-	11158		11156	11158	-	1	1	2
8184		8180	8184	-	14548	-	-		+	1	1	2
8189	-	-		+	8214	-	-		+	1	1	2
8195	-	-		-	8394	-	-		-	1	1	2
8198		8195	8201	+	8342		8342	8343	+	1	1	2
8200		8200	8204	-	8316	-	-		-	1	1	2
8200		8200	8204	-	8317	-	-		+	1	1	2

8200	8200	8204	-	8389	-	-	-	1	1	2
8208	8204	8209	+	8200	8200	8201	-	1	1	2
8210	8206	8210	-	8364	-	-	+	1	1	2
8210	8206	8210	-	8387	-	-	-	1	1	2
8210	8206	8210	-	8481	-	-	-	1	1	2
8218	8216	8219	+	8358	-	-	-	1	1	2
8225	8223	8225	-	8422	-	-	-	1	1	2
8225	8223	8225	-	8431	-	-	-	1	1	2
8225	8225	8227	+	8535	-	-	-	1	1	2
8230	8230	8231	-	8253	8253	8254	-	1	1	2
8236	8234	8236	-	8282	-	-	+	2	0	2
8236	8234	8236	-	8318	8318	8319	+	1	1	2
8244	8240	8245	-	8278	-	-	+	0	2	2
8244	8240	8245	-	8346	-	-	-	1	1	2
8244	8240	8245	-	8356	-	-	-	1	1	2
8244	8240	8245	-	8365	-	-	-	1	1	2
8244	8240	8245	-	8373	-	-	-	1	1	2
8244	8240	8245	-	8378	-	-	+	1	1	2
8246	8244	8246	+	8318	-	-	+	1	1	2
8252	8252	8256	+	8185	-	-	-	1	1	2
8252	8252	8256	+	8280	-	-	-	1	1	2
8258	-	-	-	8308	-	-	+	1	1	2
8272	8270	8277	-	8308	-	-	-	1	1	2
8272	8270	8277	-	8445	-	-	-	1	1	2
8272	8270	8277	-	9536	-	-	-	1	1	2
8278	8278	8282	+	8240	-	-	-	1	1	2
8278	8278	8282	+	8252	8252	8253	-	1	1	2
8285	-	-	+	8230	-	-	-	1	1	2
8290	8289	8291	+	8229	8229	8230	-	1	1	2
8290	8289	8291	+	8326	8326	8327	-	1	1	2
8291	-	-	-	8470	8470	8471	-	1	1	2
8300	-	-	-	8445	-	-	-	1	1	2
8308	-	-	-	12903	-	-	+	1	1	2
8316	8314	8316	+	8711	-	-	-	1	1	2
8318	8314	8320	-	8323	-	-	+	2	0	2
8318	8314	8320	-	8472	-	-	-	1	1	2
8318	8314	8320	-	8486	-	-	-	1	1	2
8318	8314	8320	-	8505	-	-	-	1	1	2
8323	-	-	-	8371	-	-	+	0	2	2
8332	8332	8334	+	8285	-	-	-	1	1	2
8332	8332	8334	+	8404	-	-	-	1	1	2
8336	-	-	-	8358	-	-	+	2	0	2
8342	8342	8343	-	8382	-	-	+	1	1	2
8342	8342	8343	-	10966	-	-	+	1	1	2
8346	8343	8346	+	8297	-	-	-	1	1	2
8346	8343	8346	+	8382	-	-	-	1	1	2
8356	8356	8358	-	8380	-	-	+	1	1	2
8358	8355	8360	+	8216	-	-	-	1	1	2
8358	8355	8360	+	8365	8365	8366	-	1	1	2
8363	8361	8366	-	8463	-	-	-	1	1	2
8364	8363	8366	+	14299	-	-	-	1	1	2
8374	8374	8375	-	8534	-	-	-	1	1	2
8385	-	-	+	8333	-	-	-	1	1	2
8386	8382	8388	-	8485	-	-	-	1	1	2
8386	8382	8388	-	8573	-	-	+	1	1	2
8391	8390	8391	+	8291	-	-	-	1	1	2
8396	8394	8399	-	8562	-	-	-	1	1	2
8396	8393	8401	+	8339	8339	8340	-	1	1	2
8396	8393	8401	+	8432	-	-	-	1	1	2
8406	8406	8410	-	8558	8558	8559	-	1	1	2
8412	8411	8413	-	8569	-	-	-	1	1	2
8421	8421	8423	-	8553	8551	8553	-	1	1	2
8438	8434	8439	-	8576	-	-	-	1	1	2
8438	8434	8439	-	8586	8586	8587	-	1	1	2
8438	8434	8439	-	8606	8602	8606	-	1	1	2
8445	8441	8447	-	8549	-	-	-	1	1	2
8445	8441	8447	-	8600	-	-	-	1	1	2
8445	8441	8447	-	8632	-	-	-	1	1	2
8445	8441	8447	-	8657	-	-	-	1	1	2
8454	8450	8458	-	8587	-	-	-	1	1	2
8454	8450	8458	-	8597	-	-	-	1	1	2
8454	8450	8458	-	8603	8603	8604	-	1	1	2
8454	8450	8458	-	8650	-	-	+	1	1	2
8454	8450	8458	-	8751	-	-	-	0	2	2
8461	8461	8465	-	8623	-	-	-	1	1	2
8461	8461	8465	-	8698	-	-	-	1	1	2
8461	8461	8465	-	8749	8749	8750	+	1	1	2
8468	-	-	-	8630	-	-	+	1	1	2
8474	8470	8478	-	8589	8586	8589	-	1	1	2
8484	8480	8487	-	8704	8701	8704	-	1	1	2
8501	8501	8503	+	12403	12401	12403	-	2	0	2
8506	-	-	-	8681	-	-	-	1	1	2
8518	8515	8518	-	8648	-	-	-	1	1	2
8518	8515	8518	-	8665	-	-	-	1	1	2
8520	8520	8521	+	8594	-	-	-	1	1	2
8520	8520	8521	+	8620	-	-	-	0	2	2
8524	8520	8526	-	8564	-	-	+	1	1	2
8524	8520	8526	-	8572	-	-	+	1	1	2
8524	8520	8526	-	8589	-	-	+	1	1	2
8524	8520	8526	-	8664	-	-	-	1	1	2
8524	8520	8526	-	8684	-	-	-	1	1	2
8531	8531	8532	-	8665	-	-	-	1	1	2
8545	8545	8549	-	8611	8611	8612	-	1	1	2
8545	8545	8549	-	8671	-	-	+	1	1	2
8545	8545	8549	-	8712	-	-	+	1	1	2
8546	8546	8548	+	8697	-	-	-	1	1	2
8554	8554	8555	-	8730	8730	8731	-	1	1	2
8554	-	-	+	9692	-	-	-	1	1	2

8559		8557	8563	-	8675		8675	8676	-	1	1	2
8559		8557	8563	-	8735	-	-		-	1	1	2
8559		8557	8563	-	9183	-	-		+	1	1	2
8562		8558	8565	+	8588	-	-		-	1	1	2
8562		8558	8565	+	8696	-	-		-	1	1	2
8567		8566	8567	+	8558	-	-		-	1	1	2
8578	-	-		-	8645	-	-		+	1	1	2
8583	-	-		-	8699	-	-		-	1	1	2
8597		8594	8597	+	8534		8534	8535	+	1	1	2
8597		8594	8597	+	8750	-	-		-	1	1	2
8598		8595	8598	-	8635	-	-		+	1	1	2
8598		8595	8598	-	8750	-	-		-	1	1	2
8602	-	-		+	8507	-	-		-	1	1	2
8608		8608	8610	+	8655	-	-		-	1	1	2
8619		8618	8620	-	8751		8749	8751	-	1	1	2
8619		8618	8620	-	8946	-	-		+	1	1	2
8624		8624	8625	-	13497	-	-		+	0	2	2
8631		8628	8636	-	8744	-	-		-	1	1	2
8631		8628	8636	-	8777	-	-		-	1	1	2
8631		8628	8636	-	8798	-	-		-	1	1	2
8631		8628	8636	-	8858	-	-		-	1	1	2
8647		8643	8648	+	8655		8655	8656	-	1	1	2
8649		8648	8652	-	8734	-	-		+	1	1	2
8649		8648	8652	-	8808	-	-		-	1	1	2
8649		8648	8652	-	13460	-	-		-	1	1	2
8655		8655	8659	+	8608	-	-		-	1	1	2
8664		8663	8665	-	8831	-	-		-	1	1	2
8666		8663	8666	+	8597		8597	8598	-	1	1	2
8666		8663	8666	+	8709	-	-		-	1	1	2
8671		8668	8674	-	8697	-	-		+	1	1	2
8671		8668	8674	-	8826	-	-		-	1	1	2
8671		8668	8674	-	8849	-	-		-	1	1	2
8671		8668	8674	-	9075	-	-		+	2	0	2
8675		8673	8678	+	8829	-	-		-	1	1	2
8680		8675	8684	-	8778	-	-		-	1	1	2
8680		8675	8684	-	8863	-	-		-	1	1	2
8680		8675	8684	-	8885		8882	8885	-	1	1	2
8680		8675	8684	-	9069	-	-		+	0	2	2
8690		8687	8695	-	8863	-	-		-	1	1	2
8690		8687	8695	-	15120	-	-		+	1	1	2
8710		8709	8712	-	8891		8889	8891	-	1	1	2
8710		8709	8712	-	8900	-	-		-	1	1	2
8710		8709	8712	-	12428	-	-		+	2	0	2
8711		8709	8713	+	8548	-	-		-	1	1	2
8722		8722	8723	-	8778	-	-		+	2	0	2
8722		8722	8723	-	8859	-	-		-	1	1	2
8724		8724	8728	+	8728	-	-		-	1	1	2
8724		8724	8728	+	8910	-	-		-	1	1	2
8727	-	-		-	8812	-	-		+	1	1	2
8730		8730	8731	+	8859	-	-		+	1	1	2
8732	-	-		-	8860	-	-		-	1	1	2
8749		8745	8750	-	8775	-	-		+	1	1	2
8750		8746	8752	+	8709		8709	8710	-	1	1	2
8750		8746	8752	+	8726		8722	8726	-	0	2	2
8765		8764	8769	+	8707		8707	8708	-	1	1	2
8772	-	-		-	8851	-	-		+	1	1	2
8776		8774	8778	+	8663		8659	8663	-	1	1	2
8778		8775	8781	-	8722	-	-		+	2	0	2
8778		8775	8781	-	8982		8982	8983	-	1	1	2
8778		8775	8781	-	9002	-	-		-	1	1	2
8783		8783	8788	-	8911		8911	8912	-	1	1	2
8783		8783	8788	-	8924		8924	8925	-	1	1	2
8788		8786	8788	+	8911		8911	8912	-	1	1	2
8796		8794	8796	-	8854	-	-		+	1	1	2
8796		8794	8796	-	8905	-	-		+	1	1	2
8796	-	-		+	8822	-	-		+	1	1	2
8825		8825	8828	-	8926	-	-		-	1	1	2
8825		8825	8828	-	8966	-	-		-	1	1	2
8825		8825	8828	-	8975	-	-		-	1	1	2
8825		8825	8828	-	8993	-	-		-	1	1	2
8828		8827	8830	+	8926		8924	8926	-	1	1	2
8834	-	-		+	8975	-	-		-	1	1	2
8841	-	-		-	8914	-	-		+	1	1	2
8843	-	-		+	8912	-	-		-	0	2	2
8855		8852	8861	-	8953		8950	8953	+	2	0	2
8855		8852	8861	-	9023	-	-		-	1	1	2
8855		8852	8861	-	9038		9036	9038	-	1	1	2
8855		8852	8861	-	9045	-	-		-	1	1	2
8855		8852	8861	-	9091	-	-		-	1	1	2
8857		8854	8857	+	8913	-	-		-	1	1	2
8863	-	-		-	9023	-	-		-	1	1	2
8874		8874	8879	-	9053	-	-		+	1	1	2
8884		8883	8884	-	8927	-	-		+	1	1	2
8884		8883	8884	-	8967	-	-		+	1	1	2
8884		8879	8885	+	8839	-	-		+	1	1	2
8884		8879	8885	+	8967	-	-		-	1	1	2
8893		8890	8896	-	9009		9006	9009	-	1	1	2
8896		8896	8899	+	8820		8817	8820	-	1	1	2
8901	-	-		-	9023	-	-		-	1	1	2
8907		8907	8915	-	8951		8951	8952	+	1	1	2
8907		8907	8915	-	9024	-	-		-	1	1	2
8907		8907	8915	-	9276	-	-		-	1	1	2
8909		8909	8911	+	8954	-	-		+	1	1	2
8915		8915	8918	+	8884	-	-		-	1	1	2
8923		8918	8927	-	9064	-	-		-	1	1	2
8929		8928	8929	+	8885	-	-		-	1	1	2
8933		8929	8933	-	9122	-	-		-	1	1	2
8933		8929	8933	-	9492	-	-		-	1	1	2

8946	8942	8950 -	8867	8867	8868 +	1	1	2
8946	8942	8950 -	9125 -	-	+	1	1	2
8950	8947	8951 +	9019 -	-	-	1	1	2
8952	8952	8954 -	9060 -	-	+	1	1	2
8952	8952	8954 -	9085	9085	9086 +	1	1	2
8959	8959	8960 +	9056 -	-	-	1	1	2
8963	8958	8969 -	9068 -	-	-	1	1	2
8963	8958	8969 -	9092 -	-	-	1	1	2
8963	8958	8969 -	9129 -	-	+	1	1	2
8963	8958	8969 -	9167 -	-	-	1	1	2
8969	8966	8969 +	9047	9047	9048 -	1	1	2
8971 -	-	-	9068 -	-	-	1	1	2
8978	8974	8981 -	9039	9039	9040 +	1	1	2
8978	8974	8981 -	9091 -	-	-	1	1	2
8978	8974	8981 -	9128	9125	9128 -	1	1	2
8978	8974	8981 -	9145 -	-	-	1	1	2
8982	8980	8983 +	8962	8962	8963 -	1	1	2
8999	8999	9000 -	9125 -	-	-	1	1	2
8999 -	-	+	9113 -	-	-	1	1	2
9008	9003	9009 -	9094 -	-	+	1	1	2
9008	9003	9009 -	9123 -	-	-	1	1	2
9008	9003	9009 -	9145 -	-	-	1	1	2
9008	9003	9009 -	9512 -	-	-	1	1	2
9019	9019	9020 -	9157	9157	9158 +	1	1	2
9019	9019	9021 +	14918 -	-	+	1	1	2
9036	9035	9036 -	8976 -	-	+	2	0	2
9036	9035	9036 -	9218	9218	9219 -	1	1	2
9037	9034	9038 +	8977	8977	8978 -	1	1	2
9037	9034	9038 +	9139	9139	9140 -	1	1	2
9046	9046	9049 -	9169	9169	9170 +	1	1	2
9061	9059	9061 -	9215 -	-	-	1	1	2
9068	9064	9073 -	9186	9186	9187 -	1	1	2
9068	9064	9073 -	9226 -	-	-	1	1	2
9068	9064	9073 -	9297	9294	9297 -	1	1	2
9070 -	-	+	9185 -	-	-	1	1	2
9082	9078	9082 -	9339	9335	9339 -	1	1	2
9090	9086	9091 -	9181 -	-	+	1	1	2
9090	9086	9091 -	9233 -	-	-	1	1	2
9090	9086	9091 -	9353 -	-	+	0	2	2
9090	9086	9091 -	9358 -	-	+	2	0	2
9103	9101	9103 -	9130 -	-	+	2	0	2
9109 -	-	-	9251 -	-	-	1	1	2
9114 -	-	-	9292 -	-	+	1	1	2
9121	9121	9124 -	9149 -	-	+	2	0	2
9121	9121	9124 -	9207 -	-	-	1	1	2
9121	9121	9124 -	9294 -	-	-	1	1	2
9128	9128	9130 +	9103 -	-	-	1	1	2
9129	9126	9132 -	9327 -	-	-	1	1	2
9129	9126	9132 -	9339 -	-	-	1	1	2
9142	9141	9145 +	9179 -	-	-	1	1	2
9142	9141	9145 +	9357	9353	9357 -	1	1	2
9143	9140	9147 -	9154 -	-	+	1	1	2
9143	9140	9147 -	9201 -	-	-	2	0	2
9149	9149	9154 -	9205 -	-	-	0	2	2
9149	9149	9154 -	9327 -	-	-	1	1	2
9149	9149	9154 -	9338	9336	9338 -	1	1	2
9165	9161	9170 +	9048	9048	9049 -	1	1	2
9165	9161	9170 +	9115 -	-	-	1	1	2
9173 -	-	-	9156 -	-	+	1	1	2
9185	9181	9190 -	9345 -	-	-	1	1	2
9185	9181	9190 -	9550 -	-	+	1	1	2
9194	9192	9200 -	9274 -	-	-	1	1	2
9194	9192	9200 -	9368 -	-	-	1	1	2
9194	9192	9200 -	9427 -	-	-	1	1	2
9194	9192	9200 -	9443	9443	9445 -	1	1	2
9197 -	-	+	9126 -	-	-	1	1	2
9204	9203	9205 -	9348 -	-	-	1	1	2
9204	9203	9205 -	9363 -	-	-	1	1	2
9210 -	-	-	9341 -	-	-	1	1	2
9218 -	-	-	9461 -	-	+	1	1	2
9223	9223	9224 -	9941 -	-	-	1	1	2
9231	9231	9237 -	9290 -	-	+	0	2	2
9231	9231	9232 +	9391 -	-	-	1	1	2
9241	9241	9242 -	11500 -	-	-	0	2	2
9250	9250	9251 -	9406	9406	9407 -	1	1	2
9256	9256	9260 +	9309	9309	9310 -	1	1	2
9259	9259	9262 -	9398 -	-	+	1	1	2
9265 -	-	+	9285 -	-	-	1	1	2
9273	9269	9275 -	9358	9358	9359 -	1	1	2
9291	9287	9292 -	9406 -	-	-	1	1	2
9291	9287	9292 -	9450	9450	9451 -	1	1	2
9300	9296	9302 -	9359 -	-	+	1	1	2
9300	9296	9302 -	9456	9456	9457 -	1	1	2
9300	9296	9302 -	9485 -	-	-	1	1	2
9300	9296	9302 -	9504 -	-	-	1	1	2
9305	9304	9308 -	9356	9353	9356 -	1	1	2
9305	9304	9308 -	9416 -	-	-	1	1	2
9305	9304	9308 -	9435 -	-	-	1	1	2
9305	9304	9308 -	9436 -	-	+	1	1	2
9305	9304	9308 -	9459	9459	9460 -	1	1	2
9305	9304	9308 -	9514 -	-	-	1	1	2
9314	9309	9315 -	9425 -	-	-	1	1	2
9314	9309	9315 -	9433 -	-	-	1	1	2
9314	9309	9315 -	9447 -	-	+	1	1	2
9314	9309	9315 -	9501 -	-	-	1	1	2
9323	9319	9326 -	9424 -	-	+	1	1	2
9323	9319	9326 -	9476 -	-	-	1	1	2
9323	9319	9326 -	9484 -	-	-	1	1	2

9327	-	-	+	9231	-	-	-	1	1	2
9340	9338	9342	+	9354	9352	9354	-	1	1	2
9340	9338	9342	+	9369	9369	9370	-	1	1	2
9341	9337	9341	-	9406	-	-	+	1	1	2
9341	9337	9341	-	9642	-	-	-	1	1	2
9347	-	-	-	9453	-	-	-	1	1	2
9350	9347	9354	+	9390	9386	9390	+	1	1	2
9350	9347	9354	+	9421	-	-	-	1	1	2
9355	9355	9357	-	9483	-	-	-	1	1	2
9355	9355	9357	-	9501	-	-	-	1	1	2
9359	9359	9363	+	11452	-	-	+	1	1	2
9370	9366	9370	-	9451	-	-	-	1	1	2
9370	9366	9370	-	9468	-	-	-	1	1	2
9370	9366	9370	-	9502	-	-	-	1	1	2
9379	9374	9383	-	9447	-	-	-	1	1	2
9393	9390	9393	+	9409	9406	9409	+	1	1	2
9395	9394	9399	-	15182	-	-	-	1	1	2
9405	-	-	-	9435	-	-	+	1	1	2
9407	9407	9408	+	9589	9589	9590	-	1	1	2
9413	9413	9415	-	9462	-	-	+	1	1	2
9413	9413	9415	-	9569	-	-	-	1	1	2
9414	9410	9417	+	9579	-	-	-	1	1	2
9414	9410	9417	+	9584	9584	9585	-	1	1	2
9414	9410	9417	+	10047	10047	10048	-	1	1	2
9422	9419	9424	-	9565	-	-	-	1	1	2
9422	9419	9424	-	9595	-	-	-	1	1	2
9422	9419	9424	-	9600	9600	9601	-	1	1	2
9424	-	-	+	9319	-	-	-	1	1	2
9424	-	-	+	9785	-	-	-	1	1	2
9428	9428	9432	-	9475	-	-	-	1	1	2
9428	9428	9432	-	9492	9488	9492	-	1	1	2
9442	9442	9444	-	9574	-	-	-	1	1	2
9447	9445	9451	+	9518	-	-	-	1	1	2
9447	9445	9451	+	9867	9867	9868	+	1	1	2
9451	9446	9456	-	9488	9485	9488	+	1	1	2
9451	9446	9456	-	9508	9508	9509	+	1	1	2
9451	9446	9456	-	9584	-	-	-	1	1	2
9451	9446	9456	-	9589	-	-	-	1	1	2
9451	9446	9456	-	9604	-	-	+	1	1	2
9451	9446	9456	-	9628	-	-	-	1	1	2
9451	9446	9456	-	9653	-	-	-	1	1	2
9451	9446	9456	-	9676	9674	9676	-	1	1	2
9451	9446	9456	-	9716	9716	9720	-	1	1	2
9451	9446	9456	-	9916	-	-	-	1	1	2
9459	-	-	+	9639	-	-	-	1	1	2
9460	9459	9460	-	9606	-	-	-	2	0	2
9460	9459	9460	-	9660	-	-	-	1	1	2
9466	9461	9468	-	9609	-	-	-	0	2	2
9466	9461	9468	-	9639	-	-	-	1	1	2
9472	9472	9473	+	9778	9778	9779	-	1	1	2
9477	9473	9477	-	9638	-	-	-	1	1	2
9479	9479	9481	+	9776	9776	9777	-	1	1	2
9486	9482	9491	-	9617	9617	9618	+	1	1	2
9494	9494	9495	-	9638	9638	9639	-	1	1	2
9499	9497	9502	-	9540	-	-	+	1	1	2
9499	9497	9502	-	9599	-	-	-	1	1	2
9499	9497	9502	-	9685	-	-	-	1	1	2
9499	9497	9502	-	9726	9726	9727	-	1	1	2
9499	9497	9502	-	9763	-	-	+	1	1	2
9514	9512	9514	+	9531	-	-	+	1	1	2
9515	9511	9515	-	9574	9570	9574	+	1	1	2
9515	9511	9515	-	9670	-	-	-	1	1	2
9520	9520	9522	-	9670	-	-	-	1	1	2
9520	9520	9522	-	9699	-	-	-	1	1	2
9523	9519	9523	+	9635	-	-	-	1	1	2
9523	9519	9523	+	9916	-	-	-	1	1	2
9536	9536	9541	-	9607	-	-	+	1	1	2
9536	9536	9541	-	9623	-	-	+	1	1	2
9536	9536	9541	-	9661	-	-	-	1	1	2
9536	9536	9541	-	9698	-	-	-	1	1	2
9543	9540	9545	+	9520	9520	9521	-	1	1	2
9543	9540	9545	+	9638	-	-	+	1	1	2
9543	9540	9545	+	9782	-	-	-	1	1	2
9547	9542	9550	-	9646	-	-	-	1	1	2
9547	9542	9550	-	9700	-	-	-	1	1	2
9547	9542	9550	-	9720	-	-	+	1	1	2
9547	9542	9550	-	9752	-	-	+	1	1	2
9547	9542	9550	-	9766	-	-	-	1	1	2
9547	9542	9550	-	9776	-	-	-	1	1	2
9549	9548	9550	+	9697	-	-	-	1	1	2
9549	9548	9550	+	9718	9718	9719	-	1	1	2
9549	9548	9550	+	9793	-	-	+	1	1	2
9555	9554	9555	-	9650	-	-	+	1	1	2
9564	9560	9564	+	9584	-	-	-	1	1	2
9569	9569	9571	+	9620	-	-	-	1	1	2
9569	9569	9571	+	9747	9747	9748	-	1	1	2
9574	9573	9577	+	9671	-	-	+	1	1	2
9574	9573	9577	+	10420	10420	10421	-	1	1	2
9579	9579	9584	+	9634	-	-	-	1	1	2
9579	9579	9584	+	12841	-	-	+	1	1	2
9586	9582	9587	-	9639	-	-	+	1	1	2
9586	9582	9587	-	9719	-	-	-	1	1	2
9586	9582	9587	-	9784	-	-	-	1	1	2
9588	9588	9589	+	9627	-	-	-	1	1	2
9588	9588	9589	+	9692	9692	9693	-	1	1	2
9597	9593	9599	+	9661	-	-	-	1	1	2
9604	9604	9605	+	9567	-	-	-	1	1	2
9604	9604	9605	+	9628	-	-	-	1	1	2

9606	9606	9609	-	9764	-	-	-	1	1	2
9606	9606	9609	-	9783	-	-	-	1	1	2
9611	-	-	+	9579	-	-	-	0	2	2
9613	9611	9614	-	9697	9695	9697	-	1	1	2
9613	9611	9614	-	9786	-	-	-	1	1	2
9623	-	-	+	9536	9536	9537	-	1	1	2
9635	9631	9635	-	9663	-	-	+	1	1	2
9635	9631	9635	-	9782	-	-	-	1	1	2
9635	9631	9635	-	14818	14818	14819	+	1	1	2
9635	9635	9638	+	9564	-	-	-	1	1	2
9635	9635	9638	+	9604	-	-	-	1	1	2
9641	9639	9644	+	9576	-	-	-	1	1	2
9641	9639	9644	+	9590	9590	9591	-	1	1	2
9641	9639	9644	+	9679	-	-	-	2	0	2
9641	9639	9644	+	9684	-	-	-	0	2	2
9641	9639	9644	+	9697	9697	9698	-	1	1	2
9641	9639	9644	+	9734	-	-	-	1	1	2
9651	9649	9651	-	9854	9852	9854	-	1	1	2
9658	9657	9658	+	9677	-	-	-	1	1	2
9665	9665	9666	-	9712	-	-	+	1	1	2
9665	9665	9666	-	9811	-	-	-	1	1	2
9665	9665	9666	+	9669	9669	9670	-	1	1	2
9673	-	-	+	9707	-	-	-	1	1	2
9680	9679	9681	-	9749	-	-	-	1	1	2
9684	9684	9685	+	9639	-	-	-	2	0	2
9684	9684	9685	+	9694	9694	9695	-	1	1	2
9684	9684	9685	+	9706	-	-	+	1	1	2
9697	9695	9698	+	9584	-	-	-	1	1	2
9697	9695	9698	+	9637	9637	9638	-	1	1	2
9698	9696	9701	-	9679	-	-	+	2	0	2
9698	9696	9701	-	9954	9954	9955	-	1	1	2
9704	9703	9709	+	9668	9668	9669	-	1	1	2
9711	9711	9712	+	9759	9759	9760	-	1	1	2
9724	9722	9725	-	9862	-	-	-	1	1	2
9724	9722	9725	-	9921	-	-	-	1	1	2
9724	9722	9725	-	9957	-	-	-	1	1	2
9739	9737	9741	-	9854	-	-	+	2	0	2
9739	9737	9741	-	9878	9878	9879	+	1	1	2
9739	9737	9741	-	9926	9924	9926	-	1	1	2
9739	9737	9741	-	10083	-	-	-	1	1	2
9747	9743	9749	-	9780	-	-	-	1	1	2
9747	9743	9749	-	9849	-	-	+	0	2	2
9747	9743	9749	-	9865	-	-	+	1	1	2
9747	9743	9749	-	9875	9875	9876	-	1	1	2
9747	9743	9749	-	9897	-	-	-	1	1	2
9747	9743	9749	-	9943	9943	9944	-	1	1	2
9752	-	-	-	9772	-	-	+	1	1	2
9753	9749	9755	+	9712	-	-	-	1	1	2
9753	9749	9755	+	9732	-	-	-	1	1	2
9753	9749	9755	+	9873	-	-	+	1	1	2
9759	9756	9762	-	9898	-	-	-	1	1	2
9759	9756	9762	-	10027	-	-	-	1	1	2
9768	9764	9769	-	9884	9882	9884	-	0	2	2
9773	9772	9777	-	9906	-	-	-	1	1	2
9773	9772	9777	-	9946	-	-	-	1	1	2
9773	9772	9777	-	10033	-	-	-	1	1	2
9775	9771	9775	+	9738	9734	9738	-	1	1	2
9782	9779	9786	-	9907	-	-	-	1	1	2
9782	9779	9786	-	9938	-	-	-	1	1	2
9782	9779	9786	-	12584	12582	12584	+	1	1	2
9788	9787	9791	-	9938	-	-	-	1	1	2
9797	-	-	-	9936	-	-	-	1	1	2
9800	-	-	+	9821	-	-	+	1	1	2
9812	9812	9815	-	9903	-	-	+	1	1	2
9812	9812	9815	-	9923	-	-	-	1	1	2
9812	9812	9815	-	9983	-	-	-	1	1	2
9817	-	-	-	9983	-	-	-	1	1	2
9840	9838	9840	-	9921	-	-	-	1	1	2
9840	9838	9840	-	10047	-	-	+	1	1	2
9850	9846	9854	-	9869	-	-	+	1	1	2
9850	9846	9854	-	9930	-	-	-	1	1	2
9850	9846	9854	-	9980	-	-	-	1	1	2
9850	9846	9854	-	9985	-	-	-	1	1	2
9850	9846	9854	-	10067	-	-	-	1	1	2
9850	9846	9854	-	10149	-	-	+	0	2	2
9850	9846	9854	-	10157	-	-	+	2	0	2
9850	9846	9854	-	10197	-	-	+	1	1	2
9870	9868	9870	-	9920	-	-	-	1	1	2
9876	9873	9876	-	10066	-	-	-	1	1	2
9876	9873	9876	-	10073	-	-	-	1	1	2
9881	9879	9885	-	10109	-	-	-	1	1	2
9892	9888	9894	+	9948	-	-	-	1	1	2
9894	9890	9897	-	9912	-	-	+	1	1	2
9894	9890	9897	-	10053	10053	10054	-	1	1	2
9899	9899	9902	+	10445	-	-	-	1	1	2
9905	9901	9908	-	9944	-	-	-	1	1	2
9905	9901	9908	-	9968	-	-	+	1	1	2
9905	9901	9908	-	10088	-	-	-	1	1	2
9905	9901	9908	-	10118	-	-	-	1	1	2
9905	9901	9908	-	10145	-	-	-	1	1	2
9910	9907	9913	+	9821	-	-	-	1	1	2
9910	9907	9913	+	10073	-	-	-	1	1	2
9910	9907	9913	+	10600	-	-	+	1	1	2
9916	9912	9922	-	9955	-	-	-	1	1	2
9916	9912	9922	-	10147	-	-	-	1	1	2
9929	9929	9934	-	10132	-	-	-	1	1	2
9937	9935	9937	+	9954	9952	9954	-	1	1	2
9939	9938	9942	-	9950	-	-	+	1	1	2

9939	9938	9942	-	14107	14107	14108	-	0	2	2
9948	9943	9953	-	10050	-	-	-	1	1	2
9948	9943	9953	-	10067	-	-	-	1	1	2
9958	-	-	+	9979	-	-	+	1	1	2
9961	9960	9963	-	10134	-	-	-	1	1	2
9964	9959	9967	+	9911	-	-	-	1	1	2
9964	9959	9967	+	10079	-	-	-	1	1	2
9970	9966	9970	-	10101	-	-	-	1	1	2
9976	9974	9979	+	9899	-	-	-	1	1	2
9976	9974	9979	+	10103	10103	10104	+	1	1	2
9977	9976	9981	-	10019	-	-	-	1	1	2
9977	9976	9981	-	10111	-	-	-	1	1	2
9983	-	-	-	10050	-	-	+	1	1	2
9993	9993	9994	-	10074	-	-	-	1	1	2
9998	-	-	-	10186	-	-	-	1	1	2
10009	10008	10012	+	10151	-	-	+	1	1	2
10012	10011	10016	-	9948	-	-	+	2	0	2
10012	10011	10016	-	10037	10037	10038	+	1	1	2
10019	10018	10022	-	10201	-	-	-	1	1	2
10019	10018	10022	-	10221	-	-	-	1	1	2
10019	10018	10022	-	10245	-	-	-	1	1	2
10026	10026	10027	+	10022	10022	10023	-	1	1	2
10031	10027	10031	-	10145	-	-	-	1	1	2
10036	10033	10039	-	10105	-	-	-	1	1	2
10036	10033	10039	-	10187	-	-	-	1	1	2
10036	10033	10039	-	10212	10212	10213	-	1	1	2
10036	10033	10039	-	10221	-	-	-	1	1	2
10036	10033	10039	-	10313	-	-	+	1	1	2
10043	10041	10043	+	9924	-	-	-	1	1	2
10043	10041	10043	+	10005	10005	10006	-	1	1	2
10043	10041	10043	+	10526	-	-	+	1	1	2
10045	10042	10049	-	10234	-	-	-	1	1	2
10045	10042	10049	-	10262	-	-	-	1	1	2
10045	10042	10049	-	10288	-	-	-	1	1	2
10065	10063	10067	-	10121	10118	10121	+	1	1	2
10065	10063	10067	-	10223	-	-	-	1	1	2
10071	10071	10072	+	10137	-	-	-	1	1	2
10071	10071	10072	+	10273	-	-	-	1	1	2
10076	10075	10077	-	10226	-	-	-	1	1	2
10076	10075	10077	-	10273	-	-	-	1	1	2
10076	10075	10077	-	10353	10353	10354	-	1	1	2
10081	10079	10087	+	9963	-	-	-	1	1	2
10081	10079	10087	+	10002	-	-	-	1	1	2
10081	10079	10087	+	10100	10100	10101	-	1	1	2
10087	-	-	-	10213	-	-	+	2	0	2
10089	10089	10092	+	10027	-	-	-	1	1	2
10089	10089	10092	+	10034	-	-	-	1	1	2
10097	10094	10097	-	10206	-	-	+	0	2	2
10097	10094	10097	-	10213	-	-	-	1	1	2
10113	10113	10115	-	10235	-	-	-	1	1	2
10115	-	-	+	10235	-	-	-	1	1	2
10120	10118	10121	-	10321	-	-	-	1	1	2
10120	10118	10121	-	10326	-	-	-	1	1	2
10132	10132	10133	+	10195	10195	10196	-	1	1	2
10134	10132	10134	-	10329	-	-	-	1	1	2
10143	10143	10147	+	10092	-	-	-	1	1	2
10143	10143	10147	+	10204	-	-	+	0	2	2
10146	10142	10149	-	10248	-	-	-	1	1	2
10146	10142	10149	-	10335	-	-	-	1	1	2
10146	10142	10149	-	10346	10346	10347	-	1	1	2
10153	10153	10156	+	10210	-	-	+	2	0	2
10162	10159	10166	-	10206	-	-	+	1	1	2
10162	10159	10166	-	10239	-	-	-	1	1	2
10168	10168	10170	-	10208	-	-	+	1	1	2
10168	10168	10170	-	10314	-	-	+	1	1	2
10175	-	-	-	10208	-	-	+	2	0	2
10183	10178	10185	-	10185	-	-	+	2	0	2
10195	10195	10198	-	10337	-	-	-	1	1	2
10195	10195	10198	-	10363	10363	10364	-	1	1	2
10208	10206	10210	-	10450	-	-	-	1	1	2
10217	10215	10221	-	10329	-	-	-	1	1	2
10217	10215	10221	-	10376	-	-	-	1	1	2
10217	10215	10221	-	10402	-	-	-	1	1	2
10223	-	-	-	10328	-	-	-	1	1	2
10241	10238	10242	+	10259	-	-	-	1	1	2
10241	10238	10242	+	10327	-	-	-	1	1	2
10245	10243	10249	-	10263	-	-	+	1	1	2
10245	10243	10249	-	10295	10293	10295	+	1	1	2
10245	10243	10249	-	10324	-	-	+	1	1	2
10245	10243	10249	-	10328	-	-	-	1	1	2
10245	10243	10249	-	10357	-	-	+	1	1	2
10245	10243	10249	-	10427	-	-	-	1	1	2
10245	10243	10249	-	10618	-	-	-	1	1	2
10249	-	-	+	10301	-	-	-	1	1	2
10258	-	-	+	10280	-	-	+	1	1	2
10260	-	-	-	10434	-	-	-	1	1	2
10271	10266	10274	-	10370	-	-	+	1	1	2
10271	10266	10274	-	10402	10402	10403	-	1	1	2
10271	10266	10274	-	10417	-	-	-	1	1	2
10276	-	-	-	10427	-	-	-	1	1	2
10276	-	-	-	13407	-	-	+	1	1	2
10276	-	-	+	10230	-	-	-	1	1	2
10281	10278	10284	-	10445	-	-	-	1	1	2
10281	10278	10284	-	10460	-	-	-	1	1	2
10284	-	-	+	10244	-	-	+	1	1	2
10286	10285	10291	-	10325	-	-	-	1	1	2
10286	10285	10291	-	10459	-	-	-	1	1	2
10302	10299	10305	-	10391	-	-	+	1	1	2

10302	10299	10305	-	10425	-	-	-	1	1	2
10302	10299	10305	-	10449	-	-	-	1	1	2
10302	10299	10305	-	10495	-	-	-	1	1	2
10302	10299	10305	-	10533	10533	10534	-	1	1	2
10307	10306	10307	-	10494	-	-	-	1	1	2
10322	10322	10323	-	10532	10532	10533	-	1	1	2
10330	10326	10330	-	10350	-	-	+	1	1	2
10330	10326	10330	-	10472	10472	10473	+	1	1	2
10334	-	-	+	10268	-	-	-	1	1	2
10349	10349	10351	-	10556	-	-	-	1	1	2
10364	10361	10366	-	10481	10477	10481	-	1	1	2
10374	10368	10374	-	10420	-	-	-	1	1	2
10374	10368	10374	-	10492	-	-	+	1	1	2
10374	10368	10374	-	10545	-	-	-	1	1	2
10381	10378	10384	-	10467	-	-	+	1	1	2
10381	10378	10384	-	10529	-	-	-	1	1	2
10381	10378	10384	-	10545	-	-	-	1	1	2
10389	10388	10391	-	10492	10490	10492	+	1	1	2
10389	10388	10391	-	10541	-	-	-	1	1	2
10389	10388	10391	-	10555	10552	10555	-	1	1	2
10395	10393	10398	-	10414	-	-	-	1	1	2
10395	10393	10398	-	10450	-	-	+	1	1	2
10395	10393	10398	-	10457	-	-	+	1	1	2
10395	10393	10398	-	10538	-	-	+	1	1	2
10403	10400	10407	-	10531	10528	10531	-	1	1	2
10403	10400	10407	-	10611	-	-	-	1	1	2
10414	10408	10417	-	10437	-	-	+	1	1	2
10414	10408	10417	-	10452	-	-	+	2	0	2
10414	10408	10417	-	10540	-	-	-	1	1	2
10414	10408	10417	-	10554	-	-	-	1	1	2
10414	10408	10417	-	13041	-	-	+	0	2	2
10417	10417	10420	+	10586	-	-	-	1	1	2
10417	10417	10420	+	10643	10640	10643	+	1	1	2
10425	10422	10426	-	10610	-	-	-	1	1	2
10425	10422	10426	-	11582	-	-	+	1	1	2
10426	-	-	+	10456	-	-	+	0	2	2
10431	10431	10432	-	10603	-	-	-	1	1	2
10431	-	-	+	10461	-	-	+	2	0	2
10436	10435	10437	-	10630	-	-	-	1	1	2
10441	10441	10442	-	10586	-	-	-	1	1	2
10445	-	-	+	10410	-	-	-	1	1	2
10451	10447	10451	-	10613	-	-	-	1	1	2
10461	10459	10463	-	10551	-	-	+	1	1	2
10461	10459	10463	-	11068	-	-	+	2	0	2
10472	10468	10476	-	10613	-	-	-	1	1	2
10472	10468	10476	-	11059	-	-	+	0	2	2
10473	-	-	+	10643	-	-	+	1	1	2
10478	-	-	-	10609	-	-	-	1	1	2
10485	10483	10487	-	10581	-	-	-	2	0	2
10485	10483	10487	-	10609	-	-	-	1	1	2
10485	10483	10487	-	10632	10632	10633	-	1	1	2
10485	10483	10487	-	10663	-	-	-	1	1	2
10485	10483	10487	-	10699	-	-	-	1	1	2
10490	10490	10493	-	10637	-	-	-	1	1	2
10496	10496	10498	+	10457	10457	10458	-	1	1	2
10496	10496	10498	+	10544	-	-	-	1	1	2
10498	10496	10498	-	10576	-	-	+	1	1	2
10498	10496	10498	-	10771	-	-	+	1	1	2
10515	10512	10516	-	10501	10498	10501	+	0	2	2
10521	10518	10521	-	10544	10541	10544	+	1	1	2
10529	10525	10529	-	10585	-	-	-	1	1	2
10543	-	-	+	10483	-	-	-	1	1	2
10551	10550	10551	-	10676	-	-	-	1	1	2
10551	10550	10551	-	10683	-	-	-	1	1	2
10559	10559	10563	+	10528	10526	10528	-	1	1	2
10565	10563	10569	-	10725	-	-	-	1	1	2
10565	10563	10569	-	10735	-	-	-	1	1	2
10565	10563	10569	-	10777	-	-	-	1	1	2
10570	10568	10570	+	10751	-	-	-	1	1	2
10570	10568	10570	+	13136	-	-	-	1	1	2
10571	10570	10573	-	10634	10634	10635	-	1	1	2
10571	10570	10573	-	10723	-	-	+	1	1	2
10571	10570	10573	-	10774	-	-	-	1	1	2
10582	-	-	+	10790	-	-	-	1	1	2
10583	10579	10586	-	10742	-	-	-	1	1	2
10583	10579	10586	-	10759	-	-	-	1	1	2
10583	10579	10586	-	11443	-	-	-	2	0	2
10583	10579	10586	-	11450	-	-	-	0	2	2
10591	10589	10592	-	10669	10669	10670	+	1	1	2
10591	10589	10592	-	10679	-	-	+	2	0	2
10591	10589	10592	-	10770	-	-	-	1	1	2
10591	10590	10594	+	10739	-	-	-	1	1	2
10591	10590	10594	+	10771	10767	10771	-	1	1	2
10596	10594	10597	-	10673	-	-	+	0	2	2
10596	10594	10597	-	10763	-	-	-	1	1	2
10596	10594	10597	-	10828	-	-	-	1	1	2
10604	-	-	-	10774	-	-	-	1	1	2
10604	-	-	-	10796	-	-	-	1	1	2
10616	10614	10620	-	10770	-	-	-	1	1	2
10617	10615	10620	+	10515	-	-	-	1	1	2
10623	-	-	-	10684	-	-	+	0	2	2
10628	-	-	-	10765	-	-	-	1	1	2
10633	10629	10633	+	10515	-	-	-	1	1	2
10639	-	-	+	10686	-	-	-	1	1	2
10644	10641	10644	+	10515	-	-	-	1	1	2
10649	-	-	+	10687	-	-	+	1	1	2
10649	-	-	+	10694	-	-	-	1	1	2
10650	10647	10650	-	10694	10691	10694	-	1	1	2

10655	10655	10657	-	10768	-	-	-	1	1	2
10655	10655	10657	-	10811	-	-	-	1	1	2
10655	10655	10657	-	11569	-	-	+	2	0	2
10657	10653	10660	+	10517	10517	10518	-	1	1	2
10657	10653	10660	+	10663	10663	10664	-	1	1	2
10660	10659	10660	-	10829	-	-	-	1	1	2
10662	10662	10664	+	10824	10824	10825	-	1	1	2
10665	10662	10666	-	10797	-	-	-	1	1	2
10670	10670	10674	-	10803	-	-	-	1	1	2
10681	-	-	+	10709	-	-	+	1	1	2
10686	10684	10687	+	10623	10620	10623	-	1	1	2
10686	10684	10687	+	10639	-	-	-	1	1	2
10694	10693	10695	-	10898	-	-	-	1	1	2
10699	10697	10701	-	10793	-	-	+	1	1	2
10699	10697	10701	-	14870	-	-	+	1	1	2
10705	10705	10707	-	14864	-	-	+	2	0	2
10721	10721	10723	+	10745	-	-	+	1	1	2
10730	10727	10730	-	10781	-	-	-	1	1	2
10743	10740	10746	-	10795	10791	10795	-	1	1	2
10743	10740	10746	-	10848	-	-	-	1	1	2
10743	10740	10746	-	10904	10904	10905	-	1	1	2
10744	10743	10744	+	10821	-	-	+	1	1	2
10752	10748	10753	+	10916	-	-	+	1	1	2
10752	10748	10753	+	12978	-	-	+	1	1	2
10757	10755	10758	-	10807	10807	10808	-	1	1	2
10757	10755	10758	-	10908	-	-	-	1	1	2
10757	10755	10758	-	10941	-	-	-	1	1	2
10757	10755	10758	-	10977	-	-	-	1	1	2
10764	10762	10765	-	10975	10973	10975	-	1	1	2
10764	10764	10767	+	10633	10633	10634	-	1	1	2
10764	10764	10767	+	10706	-	-	-	1	1	2
10769	10769	10773	-	10795	10791	10795	+	1	1	2
10771	-	-	+	13531	-	-	+	1	1	2
10776	10776	10777	+	10819	-	-	-	1	1	2
10776	10776	10777	+	12661	12659	12661	-	2	0	2
10778	10778	10782	-	10891	10891	10892	-	1	1	2
10778	10778	10782	-	12279	12279	12280	-	1	1	2
10798	10794	10798	+	10749	-	-	-	1	1	2
10805	-	-	-	10846	-	-	+	2	0	2
10809	10809	10812	+	10838	10835	10838	-	1	1	2
10810	10810	10813	-	10828	10825	10828	+	1	1	2
10810	10810	10813	-	11095	11092	11095	+	1	1	2
10823	10821	10823	-	10896	-	-	+	1	1	2
10831	10831	10834	-	11005	-	-	+	1	1	2
10836	-	-	+	10849	-	-	+	1	1	2
10842	10842	10846	-	10805	-	-	+	2	0	2
10842	10842	10846	-	10872	-	-	+	1	1	2
10845	10845	10847	+	11001	-	-	+	1	1	2
10853	10853	10855	+	10796	-	-	-	1	1	2
10853	10853	10855	+	12286	-	-	+	1	1	2
10868	-	-	-	11037	-	-	-	1	1	2
10873	10871	10876	-	10913	10911	10913	+	1	1	2
10873	10871	10876	-	10987	-	-	-	1	1	2
10873	10871	10876	-	11042	-	-	-	1	1	2
10873	10871	10876	-	11074	-	-	-	1	1	2
10873	10871	10873	+	10842	10842	10843	-	1	1	2
10879	-	-	-	11040	-	-	-	1	1	2
10884	10884	10886	-	13511	-	-	+	1	1	2
10893	-	-	+	12674	-	-	-	1	1	2
10895	10891	10895	-	11039	-	-	-	1	1	2
10895	10891	10895	-	11102	-	-	-	1	1	2
10895	10891	10895	-	12430	-	-	-	1	1	2
10904	10900	10904	+	10940	-	-	+	1	1	2
10904	10900	10904	+	10964	-	-	-	1	1	2
10912	-	-	+	10936	-	-	-	1	1	2
10931	-	-	+	10954	-	-	-	1	1	2
10940	10936	10940	-	10985	10983	10985	-	1	1	2
10952	10952	10954	-	11106	-	-	-	1	1	2
10952	10952	10954	-	11134	-	-	-	1	1	2
10958	10957	10961	-	11038	11035	11038	-	1	1	2
10973	-	-	-	11101	-	-	-	1	1	2
10979	10979	10981	-	11013	-	-	+	1	1	2
10983	10983	10986	+	11172	-	-	+	1	1	2
10987	10984	10987	-	11090	-	-	+	1	1	2
10993	-	-	-	11070	-	-	+	1	1	2
10993	10993	10997	+	11009	-	-	+	1	1	2
10993	10993	10997	+	11070	-	-	-	1	1	2
11005	11004	11009	-	11154	-	-	-	1	1	2
11014	-	-	+	11051	11051	11052	-	1	1	2
11015	11011	11015	-	11055	-	-	+	2	0	2
11015	11011	11015	-	11151	-	-	-	1	1	2
11015	11011	11015	-	11169	-	-	-	1	1	2
11021	11021	11022	-	11225	-	-	-	1	1	2
11021	11021	11022	-	11439	-	-	-	1	1	2
11032	11030	11036	-	11183	11180	11183	-	1	1	2
11032	11030	11036	-	11206	-	-	-	1	1	2
11032	11030	11036	-	11366	-	-	+	1	1	2
11043	11040	11043	+	11075	-	-	+	1	1	2
11043	11040	11043	+	12621	-	-	-	1	1	2
11053	-	-	-	11013	-	-	+	2	0	2
11060	11057	11061	-	11154	-	-	-	1	1	2
11060	11057	11061	-	11213	-	-	-	1	1	2
11060	11057	11061	-	11224	-	-	-	1	1	2
11061	11060	11062	+	13159	13159	13160	-	1	1	2
11069	-	-	-	11176	-	-	-	1	1	2
11069	-	-	-	11211	-	-	-	1	1	2
11082	11079	11085	-	11107	-	-	+	1	1	2
11082	11079	11085	-	11150	-	-	+	1	1	2

11085	-	-	+	11256	-	-	-	1	1	2
11090	-	-	-	11440	-	-	-	1	1	2
11099	11096	11102	+	11206	-	-	-	1	1	2
11102	11099	11105	-	11238	-	-	+	1	1	2
11102	11099	11105	-	11297	-	-	-	1	1	2
11102	11099	11105	-	11311	-	-	-	1	1	2
11110	11106	11110	+	11183	11183	11184	+	1	1	2
11114	11114	11116	-	11152	-	-	+	2	0	2
11114	11114	11116	-	11257	-	-	-	1	1	2
11114	11114	11116	-	11297	-	-	-	1	1	2
11115	11115	11116	+	11148	11148	11149	-	1	1	2
11115	11115	11116	+	11171	-	-	+	1	1	2
11120	11120	11123	+	11151	-	-	+	1	1	2
11120	11120	11123	+	11161	11161	11162	+	1	1	2
11127	11123	11130	-	11136	-	-	+	2	0	2
11127	11123	11130	-	11320	-	-	-	1	1	2
11136	11136	11140	+	11128	11128	11129	-	1	1	2
11136	11136	11140	+	11163	-	-	-	1	1	2
11137	11137	11141	-	11246	-	-	+	1	1	2
11137	11137	11141	-	11288	-	-	-	1	1	2
11146	11146	11149	-	11225	-	-	+	1	1	2
11146	11146	11149	-	11297	-	-	-	1	1	2
11146	11146	11149	-	11321	-	-	-	1	1	2
11152	11152	11155	-	11114	-	-	+	2	0	2
11152	11152	11155	-	11127	-	-	+	1	1	2
11153	11153	11154	+	11120	11120	11121	-	1	1	2
11162	11160	11162	-	11270	-	-	+	1	1	2
11162	11160	11162	-	11443	-	-	-	1	1	2
11173	-	-	+	11128	-	-	-	1	1	2
11178	11178	11180	+	11098	-	-	-	1	1	2
11181	11180	11181	-	11300	-	-	-	1	1	2
11186	11186	11192	-	11238	-	-	-	1	1	2
11186	11186	11192	-	11368	11368	11369	-	1	1	2
11195	-	-	-	11294	-	-	-	1	1	2
11200	11197	11203	-	11329	-	-	+	2	0	2
11200	11197	11203	-	11346	-	-	+	1	1	2
11210	11210	11211	-	11428	-	-	-	1	1	2
11217	11215	11217	-	11283	11281	11283	-	1	1	2
11218	11218	11221	+	11152	-	-	+	1	1	2
11222	11220	11226	-	11344	11341	11344	+	1	1	2
11222	11220	11226	-	11391	-	-	-	1	1	2
11222	11220	11226	-	11422	-	-	-	1	1	2
11233	11230	11233	-	11416	-	-	-	1	1	2
11233	11230	11233	-	11441	-	-	-	1	1	2
11238	11235	11238	-	11384	-	-	-	1	1	2
11238	11235	11238	-	11390	-	-	-	1	1	2
11244	11240	11247	-	11312	-	-	+	1	1	2
11244	11240	11247	-	11369	-	-	-	2	0	2
11244	11240	11247	-	11400	-	-	-	1	1	2
11252	11249	11255	-	11315	11312	11315	+	1	1	2
11252	11249	11255	-	11374	-	-	-	0	2	2
11252	11249	11255	-	11455	-	-	-	1	1	2
11267	11266	11269	+	11260	11260	11261	-	1	1	2
11267	11266	11269	+	11297	11295	11297	-	1	1	2
11269	11265	11274	-	11305	-	-	+	1	1	2
11269	11265	11274	-	11369	11366	11369	-	1	1	2
11269	11265	11274	-	11389	-	-	-	1	1	2
11269	11265	11274	-	11414	11414	11415	-	1	1	2
11269	11265	11274	-	11425	-	-	-	1	1	2
11269	11265	11274	-	11436	-	-	-	1	1	2
11269	11265	11274	-	11468	-	-	-	1	1	2
11269	11265	11274	-	11484	-	-	-	1	1	2
11269	11265	11274	-	11677	-	-	-	1	1	2
11279	-	-	+	11310	-	-	-	1	1	2
11282	11278	11286	-	11282	-	-	+	2	0	2
11282	11278	11286	-	11441	-	-	-	1	1	2
11292	11289	11295	-	11442	-	-	-	1	1	2
11292	11289	11295	-	11776	-	-	-	1	1	2
11292	-	-	+	11320	-	-	-	1	1	2
11306	11302	11309	-	11431	11431	11432	-	1	1	2
11306	11302	11309	-	11458	-	-	-	2	0	2
11318	11313	11321	-	11332	11329	11332	+	1	1	2
11318	11313	11321	-	11405	-	-	-	1	1	2
11318	11313	11321	-	11442	-	-	-	1	1	2
11318	11313	11321	-	11454	-	-	-	1	1	2
11318	11313	11321	-	11591	-	-	-	1	1	2
11321	11319	11327	+	11358	11355	11358	-	1	1	2
11321	11319	11327	+	11522	-	-	-	1	1	2
11321	11319	11327	+	11876	11874	11876	+	1	1	2
11329	11326	11333	-	11431	-	-	-	1	1	2
11329	11326	11333	-	11441	-	-	-	1	1	2
11329	11326	11333	-	11481	-	-	-	1	1	2
11342	11337	11344	-	11452	-	-	-	1	1	2
11363	11363	11366	-	11521	-	-	-	1	1	2
11363	11363	11366	-	11592	-	-	-	1	1	2
11368	11366	11372	+	13683	-	-	-	0	2	2
11370	-	-	-	11527	-	-	-	1	1	2
11379	-	-	-	11523	-	-	+	0	2	2
11381	11381	11383	+	11439	-	-	-	2	0	2
11385	11385	11387	-	11561	-	-	-	1	1	2
11398	11398	11403	+	11402	11402	11403	-	1	1	2
11404	11400	11407	-	11612	-	-	-	1	1	2
11404	11400	11407	-	11691	-	-	+	1	1	2
11411	11408	11411	-	11576	-	-	-	1	1	2
11411	11408	11411	-	11626	-	-	-	1	1	2
11413	11411	11413	+	11462	-	-	-	1	1	2
11413	11411	11413	+	11582	11582	11583	+	1	1	2
11419	11415	11420	-	11574	-	-	-	1	1	2

11419	11415	11420	-	11579	-	-	-	1	1	2
11419	11415	11420	-	11595	-	-	-	1	1	2
11424	11424	11427	-	11564	-	-	-	1	1	2
11424	11424	11427	-	11598	-	-	-	1	1	2
11433	11431	11433	-	11503	11503	11504	+	1	1	2
11433	11431	11433	-	11578	-	-	-	1	1	2
11439	11436	11440	-	11610	-	-	-	1	1	2
11439	11436	11440	-	11677	-	-	-	1	1	2
11439	11436	11440	-	11875	-	-	-	1	1	2
11439	11437	11443	+	11368	-	-	-	1	1	2
11439	11437	11443	+	11382	-	-	-	2	0	2
11444	11444	11445	-	11503	-	-	+	1	1	2
11451	-	-	+	11401	-	-	-	1	1	2
11452	11451	11457	-	11521	-	-	-	1	1	2
11452	11451	11457	-	11642	-	-	-	2	0	2
11452	11451	11457	-	11647	-	-	-	0	2	2
11452	11451	11457	-	11668	-	-	-	1	1	2
11462	11458	11463	-	11583	-	-	+	1	1	2
11462	11458	11463	-	11613	-	-	-	1	1	2
11462	11458	11463	-	11653	11653	11654	-	1	1	2
11462	11458	11463	-	11671	-	-	-	1	1	2
11478	11475	11484	-	11652	-	-	-	1	1	2
11478	11475	11484	-	11667	-	-	-	1	1	2
11479	11476	11483	+	11664	11664	11665	-	1	1	2
11494	11493	11497	-	11572	-	-	+	1	1	2
11494	11493	11497	-	11611	-	-	-	1	1	2
11494	11493	11497	-	11636	-	-	-	1	1	2
11494	11493	11497	-	11678	-	-	-	1	1	2
11494	11493	11497	-	11707	11707	11708	-	1	1	2
11494	11493	11497	-	11727	11727	11728	-	1	1	2
11494	11493	11497	-	11746	-	-	-	1	1	2
11499	11497	11499	+	11452	-	-	-	1	1	2
11499	11497	11499	+	11615	-	-	+	1	1	2
11501	11498	11504	-	11647	-	-	-	2	0	2
11501	11498	11504	-	11667	-	-	-	1	1	2
11506	11506	11509	-	11652	-	-	-	0	2	2
11511	11509	11513	+	11392	11388	11392	-	1	1	2
11511	11509	11513	+	11468	-	-	-	1	1	2
11516	11516	11519	+	11548	-	-	-	1	1	2
11516	11516	11519	+	11645	-	-	-	1	1	2
11519	11519	11521	-	11664	-	-	-	1	1	2
11521	11520	11521	+	11475	-	-	+	1	1	2
11526	11523	11529	-	11682	-	-	-	1	1	2
11526	11523	11529	-	11704	-	-	-	1	1	2
11531	11530	11531	-	15303	-	-	-	1	1	2
11536	11536	11538	-	11596	-	-	+	1	1	2
11541	-	-	-	11677	-	-	-	1	1	2
11543	-	-	+	15212	-	-	-	1	1	2
11548	11544	11551	-	11566	-	-	+	1	1	2
11548	11544	11551	-	11694	-	-	-	1	1	2
11548	11544	11551	-	11703	-	-	-	1	1	2
11548	11544	11551	-	11716	-	-	-	1	1	2
11548	11544	11551	-	11766	-	-	-	1	1	2
11556	-	-	+	11574	-	-	-	1	1	2
11560	11556	11563	-	11508	-	-	+	1	1	2
11560	11556	11563	-	11704	-	-	-	1	1	2
11560	11556	11563	-	11730	-	-	-	1	1	2
11567	11567	11571	-	11651	-	-	-	1	1	2
11567	11567	11571	-	11670	-	-	-	1	1	2
11567	11567	11571	-	11717	-	-	-	1	1	2
11567	11567	11571	-	11729	11727	11729	-	1	1	2
11567	11567	11571	-	11793	-	-	-	1	1	2
11573	11573	11579	-	11667	-	-	-	1	1	2
11573	11573	11579	-	11703	-	-	-	1	1	2
11573	11573	11579	-	11747	-	-	-	1	1	2
11581	11581	11584	-	11727	11727	11728	-	1	1	2
11581	11581	11584	-	11733	11731	11733	-	1	1	2
11592	11588	11593	-	11723	11723	11724	-	1	1	2
11592	11588	11593	-	11729	-	-	-	1	1	2
11597	-	-	+	11666	-	-	-	1	1	2
11608	11604	11610	-	11749	-	-	-	1	1	2
11608	11604	11610	-	11838	-	-	-	1	1	2
11613	11612	11615	-	11741	-	-	-	1	1	2
11613	11612	11615	-	11764	-	-	-	1	1	2
11613	11612	11615	-	11791	-	-	-	1	1	2
11613	-	-	+	11637	-	-	+	1	1	2
11622	11618	11624	-	11723	-	-	-	1	1	2
11622	11618	11624	-	11733	-	-	-	1	1	2
11622	11618	11624	-	11735	-	-	+	1	1	2
11622	11618	11624	-	11762	-	-	-	1	1	2
11622	11618	11624	-	11804	-	-	-	1	1	2
11625	11622	11626	+	11636	-	-	-	1	1	2
11625	11622	11626	+	11726	-	-	-	1	1	2
11630	-	-	-	11792	-	-	-	1	1	2
11636	11636	11639	+	11692	-	-	-	1	1	2
11641	11640	11645	+	11605	11601	11605	-	1	1	2
11641	11640	11645	+	11720	-	-	-	1	1	2
11641	11640	11645	+	12931	-	-	+	1	1	2
11648	11648	11653	+	11899	11899	11900	-	1	1	2
11653	11653	11654	-	11898	11898	11899	+	1	1	2
11661	11657	11661	-	11948	-	-	-	1	1	2
11669	11665	11669	-	11766	-	-	-	1	1	2
11675	11675	11678	-	11798	-	-	-	1	1	2
11675	11675	11678	-	11902	-	-	-	1	1	2
11686	11683	11686	+	12032	12029	12032	-	2	0	2
11690	11686	11693	-	11764	11764	11765	+	1	1	2
11690	11686	11693	-	11796	-	-	+	1	1	2
11690	11686	11693	-	11919	-	-	-	1	1	2

11690	11686	11693	-	11953	-	-	-	1	1	2
11698	11697	11703	-	11754	-	-	+	1	1	2
11698	11697	11703	-	11828	-	-	+	1	1	2
11698	11697	11703	-	12600	-	-	+	1	1	2
11712	11710	11715	-	11783	-	-	-	1	1	2
11712	11710	11715	-	11933	-	-	-	1	1	2
11722	-	-	-	11669	-	-	+	1	1	2
11726	11722	11730	+	11822	-	-	-	1	1	2
11732	11728	11734	-	11763	-	-	+	1	1	2
11732	11728	11734	-	11933	-	-	-	1	1	2
11732	11732	11734	+	11619	-	-	-	1	1	2
11738	11738	11739	-	11974	11974	11975	-	1	1	2
11745	11741	11745	+	11756	11752	11756	-	1	1	2
11759	11759	11760	-	11906	11906	11907	-	1	1	2
11768	-	-	+	11706	-	-	-	1	1	2
11774	-	-	-	12003	-	-	-	1	1	2
11781	11781	11785	-	11875	-	-	+	1	1	2
11781	11781	11785	-	11928	11926	11928	-	1	1	2
11781	11781	11785	-	11953	-	-	-	1	1	2
11791	11787	11791	+	11759	-	-	-	1	1	2
11791	11787	11791	+	11825	-	-	-	1	1	2
11791	11787	11791	+	11869	-	-	-	0	2	2
11792	11787	11796	-	11798	-	-	+	1	1	2
11792	11787	11796	-	11868	11868	11869	+	1	1	2
11792	11787	11796	-	11967	-	-	-	1	1	2
11796	-	-	+	11859	11859	11860	-	1	1	2
11804	-	-	+	11833	-	-	+	1	1	2
11810	11810	11815	-	11972	-	-	-	1	1	2
11810	11810	11815	-	12025	-	-	-	1	1	2
11810	11810	11815	-	12125	-	-	+	1	1	2
11810	-	-	+	11844	-	-	-	2	0	2
11821	-	-	-	11864	-	-	+	1	1	2
11828	11824	11828	+	11703	-	-	-	1	1	2
11835	11835	11837	+	11852	-	-	-	1	1	2
11835	11835	11837	+	11865	-	-	+	1	1	2
11835	11835	11837	+	11872	-	-	-	1	1	2
11840	11838	11843	-	11992	-	-	-	1	1	2
11840	11838	11843	-	12074	-	-	-	1	1	2
11850	11848	11850	-	12015	-	-	-	1	1	2
11864	11864	11866	-	11933	-	-	+	1	1	2
11864	11864	11866	-	12000	-	-	-	1	1	2
11864	11864	11866	-	12046	-	-	-	1	1	2
11864	11864	11866	-	12074	-	-	-	1	1	2
11864	11864	11866	-	12125	12123	12125	-	1	1	2
11873	-	-	-	12085	-	-	-	1	1	2
11881	11877	11886	-	11920	11920	11921	+	1	1	2
11881	11877	11886	-	11935	11931	11935	+	1	1	2
11881	11877	11886	-	12038	12038	12042	-	1	1	2
11881	11877	11886	-	12051	12051	12052	-	1	1	2
11881	11877	11886	-	12087	-	-	-	1	1	2
11883	-	-	+	11788	-	-	-	1	1	2
11889	-	-	-	12015	-	-	-	1	1	2
11901	11901	11905	+	11966	-	-	-	1	1	2
11901	11901	11905	+	12099	12096	12099	-	1	1	2
11905	11901	11907	-	11929	-	-	+	1	1	2
11913	-	-	-	11958	-	-	+	1	1	2
11916	11916	11917	+	11885	11885	11886	-	1	1	2
11918	-	-	-	11925	-	-	+	2	0	2
11930	-	-	-	12122	-	-	-	1	1	2
11930	-	-	-	12927	-	-	+	1	1	2
11942	11938	11946	-	12058	-	-	+	1	1	2
11942	11938	11946	-	12084	-	-	-	1	1	2
11942	11938	11946	-	12092	12089	12092	-	1	1	2
11943	-	-	+	12028	-	-	+	0	2	2
11948	11947	11952	-	11963	-	-	+	2	0	2
11950	11948	11952	+	11986	11986	11987	-	1	1	2
11950	11948	11952	+	12033	-	-	+	2	0	2
11963	11962	11963	-	11952	11952	11953	+	2	0	2
11967	11966	11967	+	11956	-	-	-	1	1	2
11968	11968	11972	-	12161	-	-	-	1	1	2
11976	11974	11976	-	11942	-	-	+	2	0	2
11981	11980	11981	-	12024	-	-	-	2	0	2
11981	11979	11981	+	12104	-	-	+	1	1	2
11981	11979	11981	+	12339	-	-	+	1	1	2
11987	11987	11988	+	12080	12080	12081	-	1	1	2
11988	11982	11990	-	12027	-	-	-	0	2	2
11988	11982	11990	-	12075	-	-	-	1	1	2
11988	11982	11990	-	12085	-	-	+	1	1	2
11988	11982	11990	-	12117	-	-	-	1	1	2
11988	11982	11990	-	12129	-	-	-	1	1	2
11993	11993	11994	-	12075	-	-	+	1	1	2
12000	11997	12003	-	12125	12121	12125	-	1	1	2
12000	11997	12003	-	12238	-	-	-	0	2	2
12000	11997	12003	-	12356	-	-	+	1	1	2
12001	12001	12002	+	12129	12129	12130	+	1	1	2
12008	12006	12014	-	12143	-	-	-	1	1	2
12011	12009	12014	+	12183	-	-	-	1	1	2
12011	12009	12014	+	12546	12546	12547	-	1	1	2
12018	12018	12019	-	12160	12160	12161	-	1	1	2
12018	12018	12019	-	12196	-	-	-	1	1	2
12018	12018	12019	-	12237	-	-	-	1	1	2
12019	12018	12019	+	12175	-	-	-	1	1	2
12029	12027	12031	-	12158	-	-	-	1	1	2
12033	-	-	+	11988	-	-	-	1	1	2
12033	-	-	+	12164	-	-	+	1	1	2
12040	12036	12041	-	12056	-	-	+	1	1	2
12040	12036	12041	-	12112	12112	12113	+	1	1	2
12047	12044	12051	-	12172	-	-	-	1	1	2

12047	12044	12051 -	12182 -	-	-	1	1	2
12053	12053	12057 -	12073 -	-	+	1	1	2
12053	12053	12057 -	12209 -	-	-	1	1	2
12053	12053	12057 -	12216 -	-	-	1	1	2
12053	12053	12057 -	12228	12228	12229 -	1	1	2
12053	12053	12057 -	12239 -	-	-	1	1	2
12053	12053	12057 -	12373 -	-	-	1	1	2
12055	12054	12055 +	12147 -	-	-	1	1	2
12059	12059	12064 -	12282	12278	12282 -	1	1	2
12059	12059	12064 -	12491 -	-	-	1	1	2
12068	12068	12069 -	12227	12227	12228 -	1	1	2
12069 -	-	+	12241 -	-	-	1	1	2
12076	12071	12079 -	12208 -	-	-	1	1	2
12076	12071	12079 -	12238 -	-	+	0	2	2
12076	12071	12079 -	12244 -	-	+	2	0	2
12076	12073	12076 +	12244 -	-	-	0	2	2
12082	12078	12086 +	11963	11960	11963 -	1	1	2
12082	12078	12086 +	12195 -	-	-	1	1	2
12082	12078	12086 +	12238 -	-	-	2	0	2
12082	12078	12086 +	12307 -	-	-	0	2	2
12082	12078	12086 +	14556 -	-	-	0	2	2
12085	12081	12086 -	12195 -	-	+	1	1	2
12085	12081	12086 -	12211 -	-	-	1	1	2
12085	12081	12086 -	12297 -	-	-	1	1	2
12089	12088	12092 +	12185	12185	12186 -	1	1	2
12090	12090	12091 -	12164 -	-	+	1	1	2
12097	12094	12097 +	12349 -	-	-	1	1	2
12101	12101	12103 -	12200 -	-	-	1	1	2
12101	12101	12103 -	12340	12340	12341 +	1	1	2
12107	12105	12107 -	12154 -	-	+	1	1	2
12120 -	-	-	12373 -	-	-	1	1	2
12120	12116	12122 +	12036 -	-	-	1	1	2
12120	12116	12122 +	12265 -	-	-	1	1	2
12120	12116	12122 +	12312 -	-	-	2	0	2
12120	12116	12122 +	12317 -	-	-	0	2	2
12126	12121	12130 -	12306 -	-	-	1	1	2
12126	12121	12130 -	12317 -	-	-	1	1	2
12126	12121	12130 -	14191 -	-	-	2	0	2
12134	12130	12135 +	12157 -	-	+	1	1	2
12134	12130	12135 +	12293	12293	12294 -	1	1	2
12136	12132	12140 -	12293 -	-	-	1	1	2
12136	12132	12140 -	14197 -	-	-	0	2	2
12139	12139	12140 +	12246	12246	12247 -	1	1	2
12145	12141	12149 -	12181 -	-	+	1	1	2
12145	12141	12149 -	12272 -	-	-	2	0	2
12145	12141	12149 -	12277 -	-	-	0	2	2
12145	12141	12149 -	12288	12288	12289 -	1	1	2
12145	12141	12149 -	12299 -	-	-	1	1	2
12146	12143	12150 +	12241	12241	12242 -	1	1	2
12146	12143	12150 +	12255 -	-	-	1	1	2
12154	12151	12154 -	12217 -	-	-	1	1	2
12154	12151	12154 -	12233 -	-	+	1	1	2
12159	12158	12160 -	12312 -	-	-	2	0	2
12159	12158	12162 +	12226	12226	12227 -	1	1	2
12168	12164	12168 -	12207 -	-	-	0	2	2
12168	12164	12168 -	12317 -	-	-	0	2	2
12168	12165	12169 +	12180 -	-	+	1	1	2
12179	12177	12179 -	12297 -	-	-	1	1	2
12179	12177	12179 -	12415 -	-	-	1	1	2
12185	12182	12185 -	12209 -	-	+	1	1	2
12185	12182	12185 -	12299	12296	12299 -	1	1	2
12188	12188	12189 +	12088	12088	12089 -	1	1	2
12191	12187	12195 -	12301 -	-	-	1	1	2
12191	12187	12195 -	12350 -	-	-	1	1	2
12191	12187	12195 -	12384 -	-	-	1	1	2
12191	12187	12195 -	12564 -	-	-	1	1	2
12191	12187	12195 -	14924	14924	14925 -	1	1	2
12195	12192	12198 +	12082 -	-	-	1	1	2
12195	12192	12198 +	12223 -	-	-	1	1	2
12207	12202	12208 -	12221	12217	12221 +	1	1	2
12207	12202	12208 -	12370	12370	12371 -	1	1	2
12207	12202	12208 -	12378 -	-	-	1	1	2
12207	12202	12208 -	12410 -	-	-	1	1	2
12207	12202	12208 -	12415 -	-	-	1	1	2
12214	12212	12217 -	12369 -	-	-	1	1	2
12214	12212	12217 -	12395 -	-	-	1	1	2
12214	12212	12217 -	12623 -	-	-	1	1	2
12220	12217	12220 +	12168 -	-	-	0	2	2
12225	12222	12228 +	12248 -	-	-	1	1	2
12225	12222	12228 +	12372 -	-	-	1	1	2
12225	12222	12228 +	15023 -	-	-	1	1	2
12228	12227	12231 -	12377 -	-	-	1	1	2
12228	12227	12231 -	12377 -	-	+	1	1	2
12228	12227	12231 -	12420 -	-	-	1	1	2
12228	12227	12231 -	13452	13450	13452 +	1	1	2
12230 -	-	+	12249 -	-	-	1	1	2
12240	12240	12242 +	12148 -	-	-	1	1	2
12242	12241	12243 -	12308 -	-	+	1	1	2
12242	12241	12243 -	12431 -	-	-	1	1	2
12248	12248	12252 -	12376 -	-	+	1	1	2
12251	12247	12253 +	12316 -	-	+	1	1	2
12258	12257	12260 -	12368	12368	12369 -	1	1	2
12258	12257	12260 -	14162	14162	14163 +	1	1	2
12261	12261	12262 +	12223 -	-	-	1	1	2
12261	12261	12262 +	12281 -	-	-	1	1	2
12263	12262	12263 -	12437 -	-	-	1	1	2
12272	12268	12272 -	12319 -	-	-	1	1	2
12272	12268	12272 -	12336 -	-	+	1	1	2

12272	12268	12272	-	12394 -	-		-	1	1	2
12280	12278	12282	-	12322 -	-		+	1	1	2
12280	12278	12282	-	12323 -	-		-	1	1	2
12291	12290	12294	-	12337	12333	12337	+	1	1	2
12291	12290	12294	-	12873 -	-		+	1	1	2
12298	12298	12299	+	12891 -	-		-	1	1	2
12304	12301	12304	-	12395 -	-		-	1	1	2
12309	12306	12309	-	12347 -	-		+	1	1	2
12322	12322	12324	+	12332 -	-		-	2	0	2
12326	12323	12330	-	12495 -	-		-	1	1	2
12326	12323	12330	-	14229	14229	14230	+	1	1	2
12327	12326	12331	+	12331 -	-		-	0	2	2
12333	12332	12338	-	12509 -	-		-	1	1	2
12343	12339	12348	-	12312 -	-		+	1	1	2
12343	12339	12348	-	12467 -	-		-	1	1	2
12343	12339	12348	-	12565 -	-		-	1	1	2
12343	12339	12348	-	12571	12571	12572	-	1	1	2
12344	12344	12347	+	12306 -	-		-	1	1	2
12360	12357	12362	-	12756 -	-		-	1	1	2
12366	12364	12367	-	12436 -	-		+	1	1	2
12366	12364	12367	-	12442 -	-		+	1	1	2
12366	12364	12367	-	12489 -	-		-	1	1	2
12366	12364	12367	-	12502	12502	12503	-	1	1	2
12366	12364	12367	-	12519 -	-		-	1	1	2
12366	12364	12367	-	12669 -	-		-	1	1	2
12366	12364	12367	-	15098 -	-		+	1	1	2
12376	12375	12377	-	12425 -	-		+	0	2	2
12376	12375	12377	-	12631 -	-		-	1	1	2
12378 -	-		+	12526 -	-		+	1	1	2
12381	12380	12381	-	12547 -	-		-	1	1	2
12388	12387	12388	-	12431 -	-		+	1	1	2
12388	12387	12392	+	12451 -	-		-	1	1	2
12396 -	-		-	12513 -	-		+	2	0	2
12398	12394	12402	+	12513 -	-		-	1	1	2
12398	12394	12402	+	14103 -	-		+	1	1	2
12401	12398	12404	-	12508 -	-		+	0	2	2
12401	12398	12404	-	12671 -	-		-	1	1	2
12412	12412	12415	-	12457 -	-		-	0	2	2
12419 -	-		+	12788 -	-		-	1	1	2
12428	12428	12431	+	12387 -	-		-	1	1	2
12428	12428	12431	+	12572 -	-		+	1	1	2
12430	12428	12431	-	12548 -	-		-	1	1	2
12430	12428	12431	-	12568 -	-		-	1	1	2
12434 -	-		+	12491 -	-		+	1	1	2
12438 -	-		-	12462 -	-		+	2	0	2
12444 -	-		-	12456 -	-		+	0	2	2
12449	12449	12453	-	12515 -	-		+	1	1	2
12449	12449	12453	-	12623 -	-		-	1	1	2
12453	12450	12453	+	12387 -	-		-	1	1	2
12453	12450	12453	+	15031 -	-		+	1	1	2
12456	12456	12457	-	12625 -	-		-	1	1	2
12467	12467	12469	+	13971 -	-		-	1	1	2
12468	12465	12468	-	12673 -	-		-	1	1	2
12473	12472	12477	-	14284 -	-		+	1	1	2
12476	12475	12479	+	12391 -	-		-	1	1	2
12476	12475	12479	+	12404	12400	12404	-	1	1	2
12486	12486	12489	-	12590 -	-		-	1	1	2
12486	12485	12491	+	12410 -	-		-	1	1	2
12486	12485	12491	+	12659 -	-		-	1	1	2
12500	12497	12501	+	12519 -	-		-	1	1	2
12500	12497	12501	+	12533	12533	12534	-	1	1	2
12500	12497	12501	+	12626	12626	12627	-	1	1	2
12513	12513	12515	-	12548 -	-		+	1	1	2
12522	12519	12522	-	12645	12645	12646	-	1	1	2
12522	12519	12522	-	12709 -	-		-	1	1	2
12529	12526	12530	-	12638	12638	12639	-	1	1	2
12529	12526	12530	-	12669 -	-		-	1	1	2
12529	12526	12530	-	12716 -	-		-	1	1	2
12529	12526	12530	-	12756	12752	12756	-	1	1	2
12534 -	-		+	12606 -	-		-	0	2	2
12542	12540	12542	+	12606	12604	12606	+	1	1	2
12557	12555	12560	+	12905 -	-		+	1	1	2
12558	12557	12562	-	12671 -	-		+	1	1	2
12564 -	-		+	12607 -	-		+	1	1	2
12571	12570	12575	-	12783	12783	12784	-	1	1	2
12576	12575	12576	+	12600	12600	12601	-	1	1	2
12576	12575	12576	+	13160 -	-		-	1	1	2
12578 -	-		-	12598 -	-		+	1	1	2
12589	12588	12594	-	12787 -	-		-	1	1	2
12600	12596	12603	+	12547 -	-		-	1	1	2
12600	12596	12603	+	12558 -	-		-	1	1	2
12607	12603	12608	-	12715 -	-		-	1	1	2
12607	12603	12608	-	12744 -	-		-	1	1	2
12607	12603	12608	-	12770 -	-		-	1	1	2
12614	12614	12615	-	12752	12752	12753	-	1	1	2
12625	12621	12627	-	12768 -	-		-	1	1	2
12625	12621	12627	-	12774 -	-		-	1	1	2
12625	12621	12627	-	12790 -	-		-	1	1	2
12625	12621	12627	-	12844 -	-		-	1	1	2
12625	12621	12627	-	12850 -	-		-	1	1	2
12625	12621	12627	-	13336	13336	13338	-	1	1	2
12629	12629	12634	+	12694	12692	12694	-	1	1	2
12629	12629	12634	+	12718 -	-		-	1	1	2
12641	12635	12645	-	12737	12737	12738	-	1	1	2
12641	12635	12645	-	12776 -	-		-	1	1	2
12641	12635	12645	-	12793 -	-		-	1	1	2
12641	12635	12645	-	12812 -	-		-	1	1	2
12641	12635	12645	-	12855	12851	12855	-	1	1	2

12649		12648	12649	-	13125	-	-		+	1	1	2
12659		12655	12659	-	12891	-	-		-	1	1	2
12659		12655	12659	-	13005	-	-		-	1	1	2
12668		12664	12669	-	12702	-	-		+	1	1	2
12668		12664	12669	-	12860	-	-		+	1	1	2
12671		12669	12671	+	12860	-	-		-	1	1	2
12671		12669	12671	+	13021	-	-		+	1	1	2
12676		12672	12676	-	12898	-	-		-	1	1	2
12676		12672	12676	-	13022	-	-		-	1	1	2
12689		12688	12692	-	12870	-	-		+	0	2	2
12689		12688	12692	-	13043	-	-		-	1	1	2
12689		12688	12692	-	13598	-	-		-	1	1	2
12705		12705	12706	+	12749	-	-		-	1	1	2
12705		12705	12706	+	12769	-	-		-	1	1	2
12707		12704	12711	-	13031	-	-		-	1	1	2
12721		12720	12724	-	12758	-	-		+	1	1	2
12723		12722	12723	+	12769	-	-		-	1	1	2
12731		12728	12734	-	12808	-	-		+	1	1	2
12731		12728	12734	-	12834	-	-		-	1	1	2
12731		12728	12734	-	12899	-	-		-	1	1	2
12731		12728	12734	-	12916	-	-		-	1	1	2
12731		12728	12734	-	14062	-	-		-	1	1	2
12739		12739	12743	-	12927	-	-		-	1	1	2
12739		12738	12740	+	12669		12669	12670	-	1	1	2
12739		12738	12740	+	12719		12719	12720	-	1	1	2
12745	-	-		-	12819	-	-		+	1	1	2
12751		12747	12755	-	12907	-	-		-	1	1	2
12751		12747	12755	-	13073	-	-		-	2	0	2
12758		12758	12760	-	12930	-	-		-	0	2	2
12758		12758	12760	-	12961		12961	12962	-	1	1	2
12758	-	-		+	12723	-	-		-	1	1	2
12767		12763	12771	-	12876	-	-		+	2	0	2
12776	-	-		+	12870	-	-		-	1	1	2
12778		12774	12779	-	12794		12792	12794	+	1	1	2
12778		12774	12779	-	12828	-	-		+	1	1	2
12778		12774	12779	-	12870	-	-		+	0	2	2
12778		12774	12779	-	12936	-	-		-	1	1	2
12778		12774	12779	-	12967	-	-		-	1	1	2
12778		12774	12779	-	13169	-	-		-	1	1	2
12782		12782	12787	+	12819		12819	12820	-	1	1	2
12782		12782	12787	+	13009		13007	13009	-	1	1	2
12782		12782	12787	+	14809		14809	14810	+	1	1	2
12786		12783	12788	-	12926	-	-		-	1	1	2
12786		12783	12788	-	12959	-	-		-	1	1	2
12786		12783	12788	-	12988	-	-		-	1	1	2
12797		12797	12799	-	13499		13499	13500	+	1	1	2
12799		12799	12800	+	12807		12807	12808	-	1	1	2
12804		12804	12806	-	12872	-	-		+	1	1	2
12804		12804	12806	-	12893	-	-		+	1	1	2
12804	-	-		+	12769	-	-		-	1	1	2
12804	-	-		+	12921	-	-		-	1	1	2
12814		12810	12818	-	12842		12838	12842	+	1	1	2
12814		12810	12818	-	12960	-	-		-	1	1	2
12814		12810	12818	-	12965	-	-		-	1	1	2
12817	-	-		+	12861	-	-		-	0	2	2
12822		12819	12822	+	12856	-	-		-	2	0	2
12822		12819	12822	+	13160	-	-		-	2	0	2
12826		12822	12828	-	12960	-	-		-	1	1	2
12826		12822	12828	-	12969	-	-		-	1	1	2
12834		12834	12838	+	12854	-	-		-	1	1	2
12834		12834	12838	+	12866		12866	12867	+	1	1	2
12834		12834	12838	+	12883	-	-		+	1	1	2
12837		12833	12841	-	12869		12865	12869	+	1	1	2
12837		12833	12841	-	12961	-	-		-	1	1	2
12843	-	-		-	13013	-	-		-	2	0	2
12845	-	-		+	12903		12903	12904	-	1	1	2
12850		12847	12851	-	12861		12859	12861	+	1	1	2
12850		12847	12851	-	13017	-	-		-	0	2	2
12850		12850	12851	+	12806	-	-		-	1	1	2
12861	-	-		-	13021	-	-		-	1	1	2
12861		12861	12864	+	12891		12891	12892	-	1	1	2
12871		12871	12876	-	13052	-	-		-	1	1	2
12871		12871	12876	-	13134	-	-		-	1	1	2
12878	-	-		-	13027	-	-		-	1	1	2
12884		12881	12884	-	13117	-	-		-	1	1	2
12891		12889	12894	-	13008	-	-		-	1	1	2
12891		12889	12894	-	13037	-	-		-	1	1	2
12891		12889	12894	-	13103	-	-		-	1	1	2
12910		12907	12913	-	13008	-	-		-	1	1	2
12910		12907	12913	-	13027	-	-		-	1	1	2
12910		12907	12913	-	13040	-	-		-	1	1	2
12910		12907	12913	-	13051	-	-		-	1	1	2
12915	-	-		+	12804	-	-		-	1	1	2
12923	-	-		-	13025	-	-		+	1	1	2
12933		12929	12938	-	13017	-	-		+	1	1	2
12933		12929	12938	-	13071		13069	13071	-	1	1	2
12933		12929	12938	-	13080	-	-		-	1	1	2
12933		12929	12938	-	13130	-	-		-	1	1	2
12933		12929	12938	-	13138		13136	13138	-	1	1	2
12933		12929	12938	-	13759	-	-		+	1	1	2
12938	-	-		+	12971	-	-		+	1	1	2
12958	-	-		-	13089	-	-		-	1	1	2
12958	-	-		-	13103	-	-		+	1	1	2
12966	-	-		-	13008	-	-		+	1	1	2
12974		12974	12975	+	12993	-	-		+	1	1	2
12982		12980	12982	-	12993	-	-		+	0	2	2
12982		12980	12982	-	13001	-	-		+	1	1	2
12982		12980	12982	-	13146	-	-		-	1	1	2

12984	-	-	+	12998	-	-	-	1	1	2
12988	12986	12989	-	13037	13037	13038	+	1	1	2
12988	12986	12989	-	13080	-	-	+	1	1	2
12988	12986	12989	-	13088	-	-	-	1	1	2
12988	12986	12989	-	13145	-	-	-	1	1	2
13001	12999	13006	-	13063	-	-	+	2	0	2
13001	12999	13006	-	13136	-	-	+	2	0	2
13001	12999	13006	-	13154	-	-	-	1	1	2
13009	13009	13010	-	13060	-	-	+	0	2	2
13009	13009	13010	-	13129	-	-	+	0	2	2
13016	13012	13016	-	13023	-	-	+	1	1	2
13024	13018	13028	-	13054	13054	13055	+	1	1	2
13024	13018	13028	-	13064	-	-	+	2	0	2
13024	13018	13028	-	13181	13181	13182	-	1	1	2
13024	13018	13028	-	13193	-	-	-	1	1	2
13024	13018	13028	-	13241	-	-	-	1	1	2
13024	13018	13028	-	13679	13679	13680	+	1	1	2
13028	-	-	+	13268	-	-	+	1	1	2
13031	13030	13032	-	13146	-	-	-	1	1	2
13039	13034	13040	-	13143	-	-	-	1	1	2
13039	13034	13040	-	13184	13182	13184	-	1	1	2
13039	13034	13040	-	13215	-	-	-	1	1	2
13039	13034	13040	-	13244	-	-	-	1	1	2
13049	13045	13052	-	13107	13107	13108	+	1	1	2
13049	13045	13052	-	13168	-	-	+	1	1	2
13049	13045	13052	-	13194	-	-	-	1	1	2
13049	13045	13052	-	13238	-	-	-	1	1	2
13049	13045	13052	-	13387	13387	13388	-	1	1	2
13049	13045	13052	-	13501	-	-	-	1	1	2
13049	13045	13052	-	13656	-	-	-	1	1	2
13055	-	-	+	13199	-	-	-	1	1	2
13061	13059	13065	+	13018	-	-	-	1	1	2
13061	13059	13065	+	13143	-	-	-	1	1	2
13061	13059	13065	+	13193	-	-	-	1	1	2
13064	13062	13067	-	13019	-	-	+	2	0	2
13064	13062	13067	-	13204	-	-	-	1	1	2
13064	13062	13067	-	13216	-	-	-	1	1	2
13064	13062	13067	-	13216	-	-	+	1	1	2
13070	13069	13071	+	13100	-	-	+	1	1	2
13072	13069	13077	-	13262	-	-	-	1	1	2
13072	13069	13077	-	13285	13285	13286	-	1	1	2
13072	13069	13077	-	13305	-	-	-	1	1	2
13072	13069	13077	-	14451	-	-	-	1	1	2
13075	13074	13075	+	13135	-	-	-	0	2	2
13075	13074	13075	+	13178	13178	13179	-	1	1	2
13079	13078	13081	-	13164	-	-	+	1	1	2
13079	13078	13081	-	13288	-	-	-	1	1	2
13095	13091	13095	-	13131	13129	13131	+	1	1	2
13095	13091	13095	-	13187	-	-	+	1	1	2
13095	13091	13095	-	13215	-	-	-	1	1	2
13096	13092	13097	+	13082	13078	13082	-	1	1	2
13104	13101	13106	-	13147	13143	13147	-	1	1	2
13104	13101	13106	-	13215	-	-	-	1	1	2
13104	13101	13106	-	13352	-	-	-	2	0	2
13106	13102	13107	+	12957	12957	12958	-	1	1	2
13110	13109	13114	-	13179	-	-	+	1	1	2
13110	13109	13114	-	13316	13316	13317	-	1	1	2
13110	13109	13114	-	13357	-	-	-	0	2	2
13110	13109	13114	-	13394	-	-	-	1	1	2
13114	13110	13115	+	13193	-	-	-	1	1	2
13117	13117	13120	-	13223	13220	13223	+	1	1	2
13124	13122	13124	+	13184	-	-	-	1	1	2
13124	13122	13124	+	13205	-	-	-	1	1	2
13128	13126	13132	-	13215	-	-	+	1	1	2
13128	13126	13132	-	13247	13247	13248	-	1	1	2
13128	13126	13132	-	13255	-	-	-	1	1	2
13128	13126	13132	-	13446	-	-	+	1	1	2
13131	13126	13134	+	13091	13091	13093	-	1	1	2
13131	13126	13134	+	13435	-	-	-	1	1	2
13139	13135	13139	+	13003	-	-	-	2	0	2
13141	13141	13145	-	13290	-	-	-	1	1	2
13141	13141	13145	-	13314	13314	13315	-	1	1	2
13144	13141	13144	+	13173	-	-	-	1	1	2
13144	13141	13144	+	13335	-	-	-	1	1	2
13157	13157	13161	-	13396	-	-	+	1	1	2
13160	13157	13161	+	13094	13094	13095	-	1	1	2
13160	13157	13161	+	13170	-	-	-	1	1	2
13160	13157	13161	+	13187	13187	13188	-	1	1	2
13168	-	-	-	13212	-	-	+	1	1	2
13168	-	-	-	13247	-	-	+	1	1	2
13178	13178	13179	-	13366	-	-	-	1	1	2
13182	13178	13184	+	13118	-	-	-	1	1	2
13182	13178	13184	+	13202	13202	13203	-	1	1	2
13182	13178	13184	+	13519	-	-	-	1	1	2
13184	13180	13187	-	13320	-	-	-	1	1	2
13184	13180	13187	-	13343	-	-	-	1	1	2
13190	13189	13194	-	13321	13321	13322	-	1	1	2
13190	13189	13194	-	13420	13420	13421	-	1	1	2
13199	13195	13199	+	13238	-	-	-	1	1	2
13199	13195	13199	+	13829	-	-	+	1	1	2
13200	13198	13203	-	13350	-	-	-	1	1	2
13200	13198	13203	-	13356	-	-	-	1	1	2
13200	13198	13203	-	13365	-	-	-	1	1	2
13200	13198	13203	-	13377	-	-	-	1	1	2
13200	13198	13203	-	13772	13772	13773	+	1	1	2
13212	13208	13216	-	13336	-	-	-	1	1	2
13212	13208	13216	-	13347	-	-	-	2	0	2
13228	13225	13232	-	13327	-	-	+	1	1	2

13228		13225	13232	-	13343	-	-		-	1	1	2
13228		13225	13232	-	13361	-	-		-	1	1	2
13228		13228	13231	+	13219		13216	13219	-	1	1	2
13238		13235	13238	-	13318	-	-		+	1	1	2
13238		13235	13238	-	13358		13356	13358	-	1	1	2
13238		13235	13238	-	13392	-	-		-	1	1	2
13248		13245	13250	+	13206	-	-		-	1	1	2
13248		13245	13250	+	13310		13310	13311	-	1	1	2
13248		13245	13250	+	13375	-	-		-	1	1	2
13254	-	-		+	13266	-	-		-	0	2	2
13261		13260	13261	+	13261		13261	13262	-	1	1	2
13273	-	-		+	13251	-	-		-	1	1	2
13279		13278	13280	-	13448	-	-		-	1	1	2
13283		13283	13284	+	13279		13279	13280	-	1	1	2
13285		13283	13287	-	13417	-	-		-	1	1	2
13285		13283	13287	-	13486	-	-		-	1	1	2
13285		13283	13287	-	13494		13494	13495	-	1	1	2
13295		13294	13296	-	13313		13313	13314	+	1	1	2
13295		13291	13295	+	13269	-	-		-	1	1	2
13303		13299	13306	-	13261	-	-		+	2	0	2
13303		13299	13306	-	13326	-	-		+	1	1	2
13303		13299	13306	-	13438	-	-		-	1	1	2
13311		13308	13313	-	13464		13464	13465	-	1	1	2
13311		13308	13313	-	13557	-	-		-	0	2	2
13314	-	-		+	13467	-	-		-	1	1	2
13318		13318	13320	-	13466	-	-		-	1	1	2
13318		13318	13320	-	13485	-	-		-	1	1	2
13318		13318	13320	-	13560	-	-		-	1	1	2
13326		13326	13330	-	13458	-	-		+	1	1	2
13334		13332	13335	+	13661	-	-		+	1	1	2
13338		13335	13341	-	13446	-	-		+	1	1	2
13343	-	-		+	13383	-	-		-	0	2	2
13349		13344	13349	-	13439	-	-		+	1	1	2
13349		13344	13349	-	13476	-	-		-	1	1	2
13349		13344	13351	+	13360		13357	13360	-	1	1	2
13349		13344	13351	+	13433		13433	13434	+	1	1	2
13349		13344	13351	+	13441	-	-		+	1	1	2
13360	-	-		+	13628	-	-		+	1	1	2
13364		13362	13368	-	13486	-	-		-	1	1	2
13364		13362	13368	-	13508	-	-		-	1	1	2
13364		13362	13368	-	13546		13542	13546	-	1	1	2
13365	-	-		+	13336	-	-		-	1	1	2
13372		13372	13377	+	13349	-	-		-	0	2	2
13383		13383	13385	+	13343	-	-		-	2	0	2
13383		13383	13385	+	13409		13409	13410	+	1	1	2
13391		13391	13395	-	13477	-	-		-	1	1	2
13391		13391	13395	-	13661	-	-		-	1	1	2
13395	-	-		+	13579	-	-		-	1	1	2
13401		13397	13401	-	13639	-	-		-	1	1	2
13405		13403	13405	+	13437		13435	13437	-	1	1	2
13410		13410	13411	+	13521		13521	13522	-	1	1	2
13422		13422	13425	+	13462		13459	13462	-	1	1	2
13432	-	-		+	13842	-	-		+	1	1	2
13445	-	-		-	13666	-	-		-	1	1	2
13450		13449	13454	-	13620	-	-		-	1	1	2
13460	-	-		-	13586	-	-		-	1	1	2
13467	-	-		+	14166	-	-		+	1	1	2
13470		13465	13471	-	13486	-	-		+	0	2	2
13470		13465	13471	-	13491	-	-		+	2	0	2
13487	-	-		-	13650	-	-		-	1	1	2
13492		13492	13493	-	13656		13656	13657	-	1	1	2
13498		13498	13502	-	13843	-	-		-	1	1	2
13500		13500	13502	+	13736	-	-		-	1	1	2
13505	-	-		+	13695		13695	13696	-	1	1	2
13512	-	-		+	13749	-	-		+	1	1	2
13517	-	-		-	13711	-	-		-	1	1	2
13518		13514	13520	+	13600	-	-		+	1	1	2
13518		13514	13520	+	13612	-	-		+	1	1	2
13518		13514	13520	+	13640	-	-		-	1	1	2
13540		13540	13541	+	13623		13623	13624	-	1	1	2
13540		13540	13541	+	13641	-	-		-	1	1	2
13544	-	-		-	13981	-	-		-	2	0	2
13550		13550	13552	-	13619		13619	13620	+	1	1	2
13550		13550	13552	-	13784	-	-		-	1	1	2
13550		13550	13552	-	13987	-	-		-	0	2	2
13557	-	-		+	13665	-	-		-	1	1	2
13562		13559	13565	-	13678	-	-		+	1	1	2
13562		13559	13565	-	13705	-	-		-	1	1	2
13570		13569	13572	-	13714	-	-		-	1	1	2
13580		13577	13580	-	13849	-	-		-	2	0	2
13587		13585	13589	-	13713	-	-		-	1	1	2
13587		13585	13589	-	13742	-	-		-	1	1	2
13587		13585	13589	-	13856	-	-		-	0	2	2
13592		13591	13594	-	13689	-	-		-	1	1	2
13599		13599	13601	+	13614		13614	13615	+	1	1	2
13600		13596	13605	-	13746	-	-		-	1	1	2
13608		13608	13609	-	13677	-	-		+	1	1	2
13608		13608	13609	-	13783	-	-		-	1	1	2
13611		13608	13612	+	13622	-	-		+	1	1	2
13615		13611	13618	-	13672	-	-		+	2	0	2
13615		13611	13618	-	13781	-	-		-	1	1	2
13615		13611	13618	-	13789	-	-		-	1	1	2
13622		13622	13623	-	13772	-	-		-	1	1	2
13622		13622	13623	-	13783	-	-		-	1	1	2
13629		13625	13632	-	13763	-	-		-	1	1	2
13629		13625	13632	-	13776		13776	13777	-	1	1	2
13629		13625	13632	-	13794	-	-		-	1	1	2
13632		13632	13635	+	13649	-	-		-	1	1	2

13635	13634	13638 -	13859	13859	13860 -	1	1	2
13640	13639	13641 -	13783 -	-	-	0	2	2
13658	13658	13662 -	13750 -	-	-	2	0	2
13662	13658	13662 +	13598 -	-	-	1	1	2
13662	13658	13662 +	13632 -	-	-	1	1	2
13668	13664	13668 -	13756 -	-	-	0	2	2
13668	13664	13668 -	13799 -	-	-	1	1	2
13679	13677	13683 -	13823 -	-	-	1	1	2
13681	13681	13685 +	13694	13694	13695 -	1	1	2
13686	13686	13687 -	13784 -	-	-	1	1	2
13694 -	-	-	13853 -	-	+	1	1	2
13699	13696	13699 -	13716	13716	13717 +	1	1	2
13699	13696	13699 -	13737 -	-	+	1	1	2
13699	13696	13699 -	13779 -	-	-	0	2	2
13704	13702	13707 -	13849 -	-	-	1	1	2
13704	13702	13707 -	13858 -	-	-	1	1	2
13705	13705	13708 +	13733	13730	13733 -	1	1	2
13705	13705	13708 +	13909 -	-	-	1	1	2
13711 -	-	+	14075 -	-	-	1	1	2
13713	13713	13715 -	13868 -	-	-	1	1	2
13716	13716	13719 +	13768 -	-	+	1	1	2
13720 -	-	-	13683 -	-	+	2	0	2
13726 -	-	+	13758 -	-	-	0	2	2
13732	13732	13733 +	13751	13751	13752 -	1	1	2
13733	13733	13734 -	14715 -	-	+	1	1	2
13738 -	-	+	13769 -	-	-	1	1	2
13739 -	-	-	13794 -	-	+	2	0	2
13745	13743	13748 -	13790 -	-	+	0	2	2
13745	13743	13748 -	14557 -	-	+	1	1	2
13748	13748	13749 +	13735	13735	13736 -	1	1	2
13752	13751	13755 -	13892 -	-	+	1	1	2
13752	13751	13755 -	13896	13893	13896 -	1	1	2
13752	13751	13755 -	13956 -	-	-	1	1	2
13752	13751	13755 -	14551 -	-	+	1	1	2
13757	13757	13758 -	13949 -	-	-	1	1	2
13757	13757	13758 -	13991	13991	13992 -	1	1	2
13763	13759	13763 -	13885 -	-	+	1	1	2
13763	13759	13763 -	13939 -	-	-	1	1	2
13772	13767	13772 -	13796	13792	13796 -	1	1	2
13772	13767	13772 -	13863 -	-	+	1	1	2
13772	13767	13772 -	13969 -	-	+	1	1	2
13772	13767	13772 -	13975 -	-	-	1	1	2
13772	13769	13776 +	13738	13736	13738 -	1	1	2
13786	13785	13787 +	13819	13819	13820 -	1	1	2
13786	13785	13787 +	13970 -	-	-	1	1	2
13796	13796	13797 -	13891 -	-	+	2	0	2
13796 -	-	+	13891 -	-	-	0	2	2
13796 -	-	+	13936 -	-	-	1	1	2
13803	13800	13804 -	13849 -	-	+	1	1	2
13803	13800	13804 -	13884 -	-	+	0	2	2
13803	13800	13804 -	13936 -	-	-	2	0	2
13803	13801	13803 +	13884 -	-	-	2	0	2
13803	13801	13803 +	14085 -	-	+	1	1	2
13808	13808	13812 -	13835 -	-	+	1	1	2
13808	13808	13812 -	13944 -	-	-	0	2	2
13811 -	-	+	13827 -	-	-	1	1	2
13818	13814	13820 -	13946 -	-	-	1	1	2
13818	13814	13820 -	14002 -	-	-	1	1	2
13818	13814	13820 -	14022 -	-	-	1	1	2
13824 -	-	-	13958 -	-	+	1	1	2
13829	13829	13830 +	13775	13775	13776 -	1	1	2
13839	13837	13840 -	13964 -	-	-	1	1	2
13844	13841	13846 +	13897 -	-	-	1	1	2
13844	13841	13846 +	13934 -	-	-	1	1	2
13845 -	-	-	13934 -	-	+	0	2	2
13845 -	-	-	14037 -	-	+	1	1	2
13859	13855	13860 +	13895	13895	13896 -	1	1	2
13861	13858	13864 -	14042 -	-	-	1	1	2
13867 -	-	+	14025 -	-	-	1	1	2
13875	13874	13880 +	14044 -	-	+	1	1	2
13876	13875	13877 -	13923 -	-	+	1	1	2
13876	13875	13877 -	14502	14502	14503 +	1	1	2
13882	13878	13882 -	14085 -	-	-	1	1	2
13882	13878	13882 -	14091 -	-	-	1	1	2
13882	13878	13882 -	14159	14157	14159 -	1	1	2
13889	13887	13889 -	14005 -	-	-	1	1	2
13889	13887	13889 -	14094 -	-	-	1	1	2
13891 -	-	+	13915 -	-	-	1	1	2
13894	13891	13894 -	14095 -	-	-	1	1	2
13900	13899	13900 -	13907 -	-	+	0	2	2
13900	13899	13900 -	14055 -	-	-	1	1	2
13911	13910	13913 +	13973	13971	13973 -	1	1	2
13912	13909	13914 -	13979 -	-	+	1	1	2
13912	13909	13914 -	14089 -	-	-	1	1	2
13925	13921	13927 -	14038	14038	14039 +	1	1	2
13925	13921	13927 -	14081	14081	14082 -	1	1	2
13925	13921	13927 -	14095 -	-	-	1	1	2
13925	13921	13927 -	14107 -	-	-	1	1	2
13925	13921	13927 -	14144 -	-	-	1	1	2
13929 -	-	+	13877 -	-	-	1	1	2
13938	13935	13940 -	14024 -	-	-	1	1	2
13938	13935	13940 -	14047	14047	14048 +	1	1	2
13938	13935	13940 -	14059	14055	14059 -	1	1	2
13938	13935	13940 -	14091 -	-	-	1	1	2
13948	13945	13952 -	14065 -	-	-	1	1	2
13948	13945	13952 -	14082	14082	14084 -	1	1	2
13948	13945	13952 -	14087	14087	14088 -	1	1	2
13948	13945	13952 -	14091 -	-	+	1	1	2

13948		13945	13952	-	14098		14098	14101	-	1	1	2
13948		13945	13952	-	14132	-	-		-	1	1	2
13948		13945	13952	-	14177	-	-		-	1	1	2
13948		13948	13951	+	14052	-	-		+	1	1	2
13961		13958	13964	-	14098	-	-		+	1	1	2
13961		13958	13964	-	14121	-	-		-	1	1	2
13967		13965	13970	-	13997	-	-		+	1	1	2
13967		13965	13970	-	14076		14073	14076	+	1	1	2
13967		13965	13970	-	14110	-	-		-	1	1	2
13967		13965	13970	-	14121	-	-		-	1	1	2
13967		13965	13970	-	14134	-	-		-	1	1	2
13972		13968	13976	+	13772	-	-		-	1	1	2
13972		13968	13976	+	14001		13997	14001	+	1	1	2
13972		13968	13976	+	14148	-	-		-	1	1	2
13972		13968	13976	+	14191	-	-		-	1	1	2
13972		13968	13976	+	14214	-	-		-	1	1	2
13972		13968	13976	+	14555	-	-		-	1	1	2
13975		13972	13975	-	14142	-	-		-	1	1	2
13975		13972	13975	-	14191	-	-		+	1	1	2
13975		13972	13975	-	14200	-	-		-	1	1	2
13982		13977	13982	-	13985	-	-		+	2	0	2
13982		13982	13984	+	14170	-	-		-	2	0	2
13988		13985	13991	-	14054	-	-		+	1	1	2
13988		13985	13991	-	14160		14160	14161	+	1	1	2
13988		13985	13991	-	14206	-	-		-	1	1	2
13992		13990	13992	+	13925	-	-		-	1	1	2
13992		13990	13992	+	14090	-	-		-	1	1	2
13996		13992	14000	-	14069	-	-		+	1	1	2
13996		13992	14000	-	14102	-	-		-	1	1	2
13996		13992	14000	-	14125	-	-		-	1	1	2
13996		13992	14000	-	14262	-	-		-	1	1	2
13996		13992	14000	-	14280	-	-		+	1	1	2
13998		13998	14000	+	13962	-	-		-	1	1	2
14002		14001	14004	-	14180	-	-		-	1	1	2
14002		14001	14004	-	14218	-	-		-	1	1	2
14002		14001	14004	-	14244	-	-		-	1	1	2
14004	-	-		+	14085	-	-		-	1	1	2
14008		14008	14012	-	14027		14027	14028	+	1	1	2
14008		14008	14012	-	14178	-	-		-	1	1	2
14016		14014	14016	-	14120	-	-		-	1	1	2
14022		14018	14022	-	14091		14091	14092	+	1	1	2
14024		14023	14024	+	13976	-	-		-	1	1	2
14024		14023	14024	+	14183		14183	14184	-	1	1	2
14027		14027	14029	-	14085	-	-		+	1	1	2
14027		14027	14029	-	14173	-	-		-	1	1	2
14042		14038	14042	-	14098	-	-		+	1	1	2
14042		14038	14042	-	14196	-	-		-	1	1	2
14048		14046	14051	-	14118	-	-		+	1	1	2
14048		14046	14051	-	14176	-	-		-	1	1	2
14048		14046	14051	-	14276		14274	14276	-	1	1	2
14048		14046	14051	-	15115	-	-		+	1	1	2
14057		14057	14058	-	14196	-	-		-	1	1	2
14057		14057	14058	-	14202	-	-		-	1	1	2
14062		14059	14062	-	14226	-	-		-	1	1	2
14063		14060	14063	+	14069	-	-		+	1	1	2
14063		14060	14063	+	14447		14447	14448	-	1	1	2
14069		14063	14072	-	14123	-	-		+	1	1	2
14081		14079	14084	+	14028	-	-		-	1	1	2
14081		14079	14084	+	14198	-	-		-	1	1	2
14085		14081	14086	-	14235	-	-		-	1	1	2
14085		14081	14086	-	14278	-	-		-	1	1	2
14086		14086	14088	+	14051		14049	14051	-	1	1	2
14091	-	-		-	14115	-	-		+	1	1	2
14094	-	-		+	14166	-	-		-	1	1	2
14098		14096	14102	-	14177	-	-		+	1	1	2
14100	-	-		+	14262	-	-		-	1	1	2
14100	-	-		+	14264	-	-		+	1	1	2
14107		14105	14110	+	14250	-	-		-	1	1	2
14107		14105	14110	+	14387		14385	14387	+	1	1	2
14116		14116	14121	-	14235	-	-		-	1	1	2
14116		14116	14121	-	14256	-	-		-	1	1	2
14117		14117	14121	+	14035	-	-		-	1	1	2
14117		14117	14121	+	14084	-	-		-	1	1	2
14117		14117	14121	+	14179	-	-		-	1	1	2
14125	-	-		+	14143	-	-		+	1	1	2
14130		14129	14134	-	14149		14149	14150	+	1	1	2
14130		14129	14134	-	14280	-	-		-	1	1	2
14130		14129	14134	-	14312		14312	14313	-	1	1	2
14136		14136	14137	-	14189	-	-		+	1	1	2
14139	-	-		+	14082	-	-		-	1	1	2
14147	-	-		+	14135	-	-		-	2	0	2
14151		14151	14154	-	14328	-	-		-	1	1	2
14151		14151	14154	-	14345	-	-		-	1	1	2
14157		14155	14160	-	14208	-	-		+	2	0	2
14157		14155	14160	-	14280	-	-		-	1	1	2
14157		14155	14160	-	14298		14296	14298	-	1	1	2
14157		14155	14160	-	14326	-	-		-	1	1	2
14162	-	-		-	14203	-	-		+	0	2	2
14162	-	-		-	14544	-	-		-	1	1	2
14165		14162	14166	+	14078	-	-		-	1	1	2
14165		14162	14166	+	14107		14104	14107	-	1	1	2
14168		14165	14171	-	14197	-	-		+	1	1	2
14168		14165	14171	-	14244	-	-		+	1	1	2
14168		14165	14171	-	14334	-	-		-	1	1	2
14168		14165	14171	-	14348	-	-		-	1	1	2
14168		14165	14171	-	14354	-	-		-	1	1	2
14168		14165	14171	-	14387	-	-		-	1	1	2
14171		14171	14173	+	14147	-	-		-	1	1	2

14171	14171	14173	+	14236	14236	14237	-	1	1	2
14177	14172	14178	-	14299	-	-	-	1	1	2
14177	14172	14178	-	14348	-	-	-	1	1	2
14177	14172	14178	-	14423	14423	14424	+	1	1	2
14177	14177	14180	+	14462	-	-	-	1	1	2
14182	14180	14184	-	14275	-	-	+	1	1	2
14182	14180	14184	-	14326	-	-	-	1	1	2
14184	14183	14188	+	14254	-	-	-	1	1	2
14192	-	-	+	14794	-	-	-	0	2	2
14197	14197	14201	-	14277	-	-	+	1	1	2
14197	14197	14201	-	14327	-	-	-	1	1	2
14197	14197	14201	-	14354	-	-	-	1	1	2
14198	-	-	+	14082	-	-	-	1	1	2
14204	14199	14208	+	14434	14434	14435	-	1	1	2
14204	14199	14208	+	14772	-	-	-	1	1	2
14204	14199	14208	+	14786	-	-	-	2	0	2
14207	14204	14208	-	14233	14229	14233	+	1	1	2
14207	14204	14208	-	14274	14271	14274	+	1	1	2
14215	14211	14216	-	14407	-	-	-	1	1	2
14215	14211	14216	-	14430	-	-	-	1	1	2
14215	14211	14216	-	14464	14464	14465	+	1	1	2
14215	14215	14218	+	14183	-	-	-	0	2	2
14220	14220	14221	-	15379	-	-	-	1	1	2
14221	14220	14225	+	14160	-	-	-	1	1	2
14221	14220	14225	+	14167	-	-	-	1	1	2
14221	14220	14225	+	14180	-	-	-	2	0	2
14225	14225	14226	-	14356	14356	14357	+	1	1	2
14231	14230	14233	-	14273	14271	14273	+	1	1	2
14231	14230	14233	-	14582	-	-	+	1	1	2
14243	14237	14246	+	14112	-	-	-	1	1	2
14243	14237	14246	+	14259	-	-	-	1	1	2
14243	14237	14246	+	14541	-	-	+	1	1	2
14247	14247	14248	-	14407	-	-	-	1	1	2
14247	14247	14248	-	14459	-	-	-	1	1	2
14251	14248	14255	+	14063	-	-	-	1	1	2
14270	14266	14273	+	14527	-	-	-	1	1	2
14272	14272	14277	-	14408	-	-	-	1	1	2
14272	14272	14277	-	14425	-	-	-	1	1	2
14272	14272	14277	-	14435	-	-	-	1	1	2
14279	14278	14280	-	14299	14299	14300	+	1	1	2
14279	14278	14280	-	14424	-	-	-	1	1	2
14283	-	-	+	14207	-	-	+	1	1	2
14294	-	-	+	14518	-	-	+	1	1	2
14300	-	-	+	14782	-	-	+	1	1	2
14301	14297	14306	-	14344	-	-	+	1	1	2
14301	14297	14306	-	14409	-	-	-	1	1	2
14301	14297	14306	-	14424	-	-	-	1	1	2
14301	14297	14306	-	14463	-	-	-	1	1	2
14301	14297	14306	-	14516	-	-	-	1	1	2
14305	14303	14305	+	14372	-	-	-	1	1	2
14305	14303	14305	+	14384	-	-	+	1	1	2
14309	14308	14309	-	14422	-	-	-	1	1	2
14309	14308	14309	-	14497	-	-	-	1	1	2
14311	14311	14312	+	14405	14405	14406	-	1	1	2
14324	14322	14326	-	14505	-	-	-	1	1	2
14324	14322	14326	-	14921	14921	14922	-	1	1	2
14332	14332	14337	-	14478	14478	14479	-	1	1	2
14341	14338	14344	-	14352	14350	14352	+	1	1	2
14341	14338	14344	-	14493	-	-	-	1	1	2
14341	14338	14344	-	14498	-	-	-	1	1	2
14341	14338	14344	-	14573	-	-	-	1	1	2
14341	14338	14344	-	14902	-	-	-	1	1	2
14344	-	-	+	14863	-	-	-	1	1	2
14346	14345	14347	-	14496	-	-	-	1	1	2
14346	14345	14347	-	14505	-	-	-	1	1	2
14359	14355	14361	-	14538	-	-	+	1	1	2
14369	14366	14371	-	15067	-	-	+	1	1	2
14369	14366	14372	+	14305	-	-	-	1	1	2
14369	14366	14372	+	14403	14403	14404	+	1	1	2
14369	14366	14372	+	14435	-	-	+	1	1	2
14374	14374	14377	-	14435	-	-	-	0	2	2
14374	14374	14377	-	14574	-	-	+	1	1	2
14382	14379	14384	-	14405	-	-	+	1	1	2
14382	14379	14384	-	14573	-	-	-	1	1	2
14382	14379	14384	-	15020	-	-	+	1	1	2
14388	-	-	+	14335	-	-	+	1	1	2
14391	14386	14395	-	14387	-	-	+	2	0	2
14391	14386	14395	-	14574	-	-	+	1	1	2
14391	14386	14395	-	14591	-	-	-	1	1	2
14391	14386	14395	-	14605	-	-	-	1	1	2
14394	14394	14397	+	14544	-	-	-	1	1	2
14398	14397	14402	-	14558	-	-	-	1	1	2
14398	14397	14402	-	14560	-	-	+	0	2	2
14398	14397	14402	-	14565	-	-	+	2	0	2
14398	14397	14402	-	14570	-	-	-	1	1	2
14409	14404	14411	-	14447	-	-	-	1	1	2
14409	14404	14411	-	14546	-	-	-	1	1	2
14409	14404	14411	-	14587	-	-	-	1	1	2
14416	14413	14419	-	14554	-	-	-	1	1	2
14416	14413	14419	-	14568	-	-	-	0	2	2
14416	14413	14419	-	14574	-	-	+	0	2	2
14416	14413	14419	-	14582	-	-	-	1	1	2
14416	14416	14420	+	14472	14468	14472	+	1	1	2
14428	14428	14430	-	14541	-	-	+	1	1	2
14435	14435	14440	-	14544	-	-	+	1	1	2
14435	14435	14440	-	14602	-	-	-	1	1	2
14435	14435	14440	-	14865	14862	14865	+	1	1	2
14438	14434	14442	+	14403	14400	14403	-	1	1	2

14438	14434	14442	+	14455 -	-		-	1	1	2
14438	14434	14442	+	14573	14569	14573	-	1	1	2
14442	14441	14445	-	14524 -	-		+	0	2	2
14442	14441	14445	-	14542 -	-		+	1	1	2
14442	14441	14445	-	14590 -	-		-	1	1	2
14442	14441	14445	-	14678 -	-		-	1	1	2
14445	14445	14448	+	14893 -	-		+	1	1	2
14447	14447	14451	-	14496 -	-		+	1	1	2
14447	14447	14451	-	14519 -	-		-	1	1	2
14450 -	-		+	14511	14511	14512	+	1	1	2
14453	14452	14454	-	14500 -	-		-	1	1	2
14461 -	-		-	14612 -	-		-	1	1	2
14465	14464	14465	+	14539 -	-		-	1	1	2
14465	14464	14465	+	14569 -	-		-	1	1	2
14471	14471	14473	-	14646	14644	14646	-	1	1	2
14479	14476	14482	-	14494 -	-		+	1	1	2
14479	14476	14482	-	14570 -	-		+	1	1	2
14479	14479	14482	+	14488	14488	14489	-	1	1	2
14479	14479	14482	+	14526 -	-		-	1	1	2
14479	14479	14482	+	14541	14541	14542	-	1	1	2
14484 -	-		+	14507 -	-		-	1	1	2
14495 -	-		-	15109 -	-		+	1	1	2
14503	14503	14504	+	14488 -	-		-	1	1	2
14504	14503	14507	-	14541 -	-		+	1	1	2
14508	14507	14508	+	14484 -	-		-	1	1	2
14515	14512	14515	-	14740 -	-		-	1	1	2
14521	14519	14523	-	14568 -	-		+	1	1	2
14521	14519	14523	-	14751 -	-		-	1	1	2
14521	14519	14523	-	15384	15380	15384	-	1	1	2
14531 -	-		-	14601 -	-		-	1	1	2
14533	14529	14536	+	14641 -	-		-	1	1	2
14540	14536	14543	-	14649 -	-		-	0	2	2
14540	14536	14543	-	14723	14723	14724	-	1	1	2
14540	14536	14543	-	14732 -	-		-	1	1	2
14540	14536	14543	-	14752 -	-		-	1	1	2
14540	14536	14543	-	14783 -	-		-	1	1	2
14542	14539	14547	+	14508	14508	14509	-	1	1	2
14545	14545	14549	-	14674	14670	14674	-	1	1	2
14554	14551	14558	-	14845 -	-		-	2	0	2
14556	14556	14558	+	14480 -	-		-	1	1	2
14556	14556	14558	+	14619 -	-		-	1	1	2
14560 -	-		-	14750 -	-		-	1	1	2
14566	14563	14568	-	14752 -	-		-	1	1	2
14566	14563	14568	-	14850 -	-		-	0	2	2
14566	14563	14568	-	15129 -	-		+	1	1	2
14568	14563	14572	+	14540 -	-		+	1	1	2
14568	14563	14572	+	14594	14592	14594	+	1	1	2
14568	14563	14572	+	15155 -	-		+	1	1	2
14577	14573	14577	-	14692 -	-		+	1	1	2
14582	14582	14585	+	14638	14635	14638	-	1	1	2
14583	14579	14587	-	14789 -	-		-	1	1	2
14583	14579	14587	-	14794 -	-		-	1	1	2
14591	14591	14592	+	14796	14796	14797	-	1	1	2
14592	14589	14596	-	14659 -	-		-	1	1	2
14592	14589	14596	-	14770 -	-		-	1	1	2
14592	14589	14596	-	14781 -	-		-	0	2	2
14603	14598	14607	-	14710 -	-		-	1	1	2
14603	14598	14607	-	14733	14733	14734	-	1	1	2
14603	14598	14607	-	14769 -	-		-	1	1	2
14605	14603	14605	+	14784	14784	14785	-	1	1	2
14609 -	-		-	14780 -	-		+	1	1	2
14614	14614	14615	-	14794 -	-		-	1	1	2
14619	14617	14620	-	14792 -	-		-	1	1	2
14620	14620	14621	+	14598	14598	14599	-	1	1	2
14626	14626	14630	-	14669 -	-		+	2	0	2
14626	14626	14630	-	14730 -	-		-	1	1	2
14629	14627	14629	+	14765	14765	14766	+	1	1	2
14629	14627	14629	+	15011 -	-		+	1	1	2
14632	14632	14636	-	14687 -	-		-	1	1	2
14632	14632	14636	-	14823	14823	14824	-	1	1	2
14638	14634	14640	+	14695 -	-		-	1	1	2
14642	14638	14643	-	14750 -	-		-	1	1	2
14642	14638	14643	-	14790	14790	14791	-	1	1	2
14642	14638	14643	-	14847	14844	14847	-	1	1	2
14647	14645	14651	-	14816	14814	14816	-	1	1	2
14653	14653	14655	-	14749 -	-		+	1	1	2
14653	14651	14653	+	14508 -	-		-	1	1	2
14653	14651	14653	+	14568	14568	14569	-	1	1	2
14653	14651	14653	+	14817 -	-		-	1	1	2
14658	14658	14659	-	14826	14826	14827	-	1	1	2
14660	14659	14660	+	14757 -	-		+	1	1	2
14667	14662	14670	-	14632	14628	14632	+	2	0	2
14667	14662	14670	-	14828 -	-		-	1	1	2
14676	14672	14680	-	14729	14729	14730	+	1	1	2
14676	14672	14680	-	14754 -	-		-	1	1	2
14687	14686	14691	-	14750 -	-		+	1	1	2
14687	14686	14691	-	14883 -	-		+	1	1	2
14687	14686	14691	-	15075 -	-		+	1	1	2
14693	14693	14694	-	14804 -	-		-	1	1	2
14693	14693	14694	-	15116 -	-		-	1	1	2
14708	14704	14708	+	14754	14750	14754	-	1	1	2
14717	14714	14721	-	14724 -	-		+	1	1	2
14717	14714	14721	-	14743 -	-		+	2	0	2
14717	14714	14721	-	14825 -	-		+	1	1	2
14717	14714	14721	-	14843 -	-		-	1	1	2
14717	14714	14721	-	14897 -	-		-	1	1	2
14717	14714	14721	-	14904 -	-		-	1	1	2
14717	14714	14721	-	14922 -	-		-	1	1	2

14723	-	-	-	14861	-	-	-	1	1	2
14728	14726	14731	-	14822	-	-	+	1	1	2
14728	14726	14731	-	14925	-	-	-	1	1	2
14728	14726	14731	-	15270	-	-	+	1	1	2
14730	-	-	+	14700	-	-	-	1	1	2
14734	-	-	-	14789	-	-	-	1	1	2
14741	14739	14741	+	14650	-	-	-	1	1	2
14741	14739	14741	+	14750	14750	14751	-	1	1	2
14744	14741	14748	-	14882	14882	14883	+	1	1	2
14744	14741	14748	-	14894	-	-	-	1	1	2
14750	14750	14752	-	14875	-	-	+	1	1	2
14750	14750	14752	-	14971	-	-	+	1	1	2
14755	14755	14757	-	14760	-	-	+	1	1	2
14756	-	-	+	14686	-	-	-	1	1	2
14767	14763	14769	+	14790	-	-	+	1	1	2
14767	14763	14769	+	14861	-	-	-	1	1	2
14770	14766	14771	-	14824	-	-	+	1	1	2
14770	14766	14771	-	14894	-	-	-	1	1	2
14770	14766	14771	-	14939	14935	14939	-	1	1	2
14770	14766	14771	-	14999	-	-	-	1	1	2
14775	14775	14777	+	14811	-	-	-	1	1	2
14775	14775	14777	+	14892	-	-	-	1	1	2
14776	14773	14781	-	14785	-	-	+	1	1	2
14776	14773	14781	-	14975	-	-	-	1	1	2
14786	14783	14789	-	14920	-	-	-	1	1	2
14786	14783	14789	-	14931	-	-	-	1	1	2
14786	14783	14789	-	14945	-	-	-	1	1	2
14786	14783	14789	-	14955	14955	14956	-	1	1	2
14791	14790	14795	-	14920	-	-	-	1	1	2
14791	14790	14795	-	14930	-	-	-	1	1	2
14795	14793	14795	+	14764	-	-	-	1	1	2
14795	14793	14795	+	14866	-	-	+	1	1	2
14801	14799	14801	-	14888	-	-	+	1	1	2
14801	14799	14801	-	14928	-	-	-	1	1	2
14806	14802	14807	-	14886	-	-	+	1	1	2
14806	14802	14807	-	15142	-	-	-	1	1	2
14810	14810	14811	+	14877	14877	14878	-	1	1	2
14812	14811	14812	-	14871	-	-	+	1	1	2
14818	14816	14822	-	14867	14867	14868	-	1	1	2
14818	14816	14822	-	14898	14895	14898	-	1	1	2
14818	14816	14822	-	14963	-	-	-	1	1	2
14818	14816	14822	-	15149	-	-	-	1	1	2
14818	14816	14824	+	14801	14801	14802	-	1	1	2
14824	14823	14827	-	15353	15353	15354	-	1	1	2
14826	14826	14827	+	14761	-	-	-	1	1	2
14826	14826	14827	+	14792	-	-	-	1	1	2
14831	-	-	+	14868	-	-	+	1	1	2
14836	14836	14839	-	15074	-	-	+	1	1	2
14837	-	-	+	14796	-	-	-	1	1	2
14842	14842	14844	-	14998	-	-	-	1	1	2
14842	14842	14844	-	15014	-	-	+	1	1	2
14842	14842	14844	-	15025	-	-	-	1	1	2
14842	14842	14844	-	15061	-	-	-	1	1	2
14847	14846	14849	-	15061	-	-	+	1	1	2
14847	14847	14850	+	14776	14776	14777	-	1	1	2
14854	14852	14855	-	15002	-	-	+	1	1	2
14854	14853	14854	+	15026	-	-	-	1	1	2
14859	14855	14860	+	14918	-	-	-	1	1	2
14861	14859	14862	-	14985	-	-	-	1	1	2
14861	14859	14862	-	15327	-	-	-	1	1	2
14864	14862	14866	+	14893	14893	14894	-	1	1	2
14866	14863	14870	-	15038	15038	15039	-	1	1	2
14866	14863	14870	-	15061	-	-	-	1	1	2
14866	14863	14870	-	15153	-	-	-	1	1	2
14869	-	-	+	14880	14880	14881	-	1	1	2
14875	14871	14877	-	14979	-	-	-	1	1	2
14875	14871	14877	-	15004	-	-	+	1	1	2
14875	14871	14877	-	15036	-	-	-	1	1	2
14885	14879	14888	-	14930	-	-	+	2	0	2
14885	14879	14888	-	14977	-	-	+	1	1	2
14885	14879	14888	-	15021	-	-	-	1	1	2
14885	14879	14888	-	15053	-	-	-	1	1	2
14891	14890	14891	-	14926	-	-	+	0	2	2
14891	14890	14891	-	15086	-	-	-	1	1	2
14892	14892	14895	+	14870	-	-	-	1	1	2
14897	14892	14900	-	14925	14925	14926	+	1	1	2
14897	14892	14900	-	14942	-	-	+	1	1	2
14897	14892	14900	-	14959	14959	14960	+	1	1	2
14897	14892	14900	-	14970	-	-	+	1	1	2
14897	14892	14900	-	15021	-	-	-	1	1	2
14897	14892	14900	-	15032	-	-	-	1	1	2
14897	14892	14900	-	15053	-	-	-	1	1	2
14897	14892	14900	-	15067	-	-	-	1	1	2
14897	14892	14900	-	15076	-	-	-	1	1	2
14897	14892	14900	-	15106	-	-	+	1	1	2
14897	14892	14900	-	15117	-	-	+	1	1	2
14897	14892	14900	-	15208	-	-	-	1	1	2
14902	14898	14906	+	14827	14825	14827	-	1	1	2
14902	14898	14906	+	14874	-	-	+	1	1	2
14902	14898	14906	+	14880	14877	14880	-	1	1	2
14902	14898	14906	+	15265	-	-	+	1	1	2
14912	14910	14915	-	14935	-	-	+	1	1	2
14912	14910	14915	-	15079	-	-	-	1	1	2
14912	14910	14915	-	15085	-	-	-	1	1	2
14912	14908	14916	+	14967	14963	14967	-	1	1	2
14912	14908	14916	+	15016	-	-	+	1	1	2
14912	14908	14916	+	15106	-	-	-	1	1	2
14920	14920	14923	+	14954	-	-	+	1	1	2

14923	14923	14926 -	14962 -	-	+	1	1	2
14923	14923	14926 -	15065 -	-	+	1	1	2
14923	14923	14926 -	15150 -	-	-	1	1	2
14928	14927	14929 -	15084 -	-	-	1	1	2
14929 -	-	+	14902 -	-	-	1	1	2
14938	14937	14941 -	15086 -	-	-	1	1	2
14938	14937	14941 -	15095 -	-	-	1	1	2
14938	14937	14941 -	15102 -	-	-	1	1	2
14938	14937	14941 -	15112 -	-	+	1	1	2
14944	14943	14947 -	14997 -	-	+	0	2	2
14944	14943	14947 -	15107 -	-	-	1	1	2
14944	14943	14947 -	15147 -	-	-	1	1	2
14944	14943	14947 -	15160 -	-	-	1	1	2
14944	14943	14947 -	15384 -	-	-	1	1	2
14951	14950	14955 -	15167 -	-	-	1	1	2
14951	14950	14955 -	15384 -	-	-	2	0	2
14960	14959	14964 -	14998 -	-	+	1	1	2
14960	14959	14964 -	15055 -	-	-	1	1	2
14960	14959	14964 -	15085 -	-	-	1	1	2
14960	14959	14964 -	15132 -	-	-	1	1	2
14960	14959	14964 -	15149 -	-	-	1	1	2
14960	14959	14964 -	15160	15160	15161 -	1	1	2
14960	14959	14964 -	15166 -	-	-	1	1	2
14960	14959	14964 -	15179 -	-	-	1	1	2
14960	14959	14964 -	15197 -	-	-	1	1	2
14960	14959	14964 -	15384 -	-	-	1	1	2
14963 -	-	+	14912 -	-	-	2	0	2
14969 -	-	+	15118 -	-	-	1	1	2
14972	14968	14974 -	15003 -	-	+	1	1	2
14972	14968	14974 -	15043 -	-	+	1	1	2
14972	14968	14974 -	15128 -	-	-	1	1	2
14972	14968	14974 -	15141 -	-	-	1	1	2
14979	14978	14983 -	15107 -	-	-	1	1	2
14979	14978	14983 -	15304 -	-	-	1	1	2
14996	14993	14996 -	15057 -	-	+	1	1	2
14999	14994	15000 +	14942 -	-	-	1	1	2
15002	14998	15003 -	15184 -	-	-	1	1	2
15002	14998	15003 -	15236 -	-	-	1	1	2
15002	14998	15003 -	15321 -	-	-	1	1	2
15004	15004	15006 +	15056 -	-	+	1	1	2
15010	15010	15015 -	15086	15086	15087 +	1	1	2
15010	15010	15015 -	15154 -	-	-	1	1	2
15010	15010	15012 +	15092 -	-	-	1	1	2
15010	15010	15012 +	15102	15102	15103 -	1	1	2
15017	15016	15020 -	15149 -	-	-	1	1	2
15017	15016	15020 -	15384 -	-	-	1	1	2
15020	15016	15020 +	15084	15084	15085 -	1	1	2
15020	15016	15020 +	15144 -	-	-	1	1	2
15025	15024	15028 -	15048 -	-	+	1	1	2
15025	15024	15028 -	15138 -	-	+	1	1	2
15025	15024	15028 -	15318 -	-	-	1	1	2
15025	15022	15025 +	15156 -	-	+	1	1	2
15032	15032	15035 +	15125	15125	15126 -	1	1	2
15037	15032	15038 -	15216 -	-	-	1	1	2
15037	15037	15040 +	15072 -	-	-	1	1	2
15037	15037	15040 +	15148 -	-	-	1	1	2
15045	15043	15045 -	15195 -	-	-	1	1	2
15045	15042	15046 +	15282 -	-	+	1	1	2
15045	15042	15046 +	15384 -	-	-	1	1	2
15052	15048	15055 -	15158 -	-	-	1	1	2
15052	15048	15055 -	15166 -	-	-	1	1	2
15052	15048	15055 -	15177 -	-	-	1	1	2
15053	15053	15055 +	15338	15336	15338 -	1	1	2
15060	15060	15062 -	15161 -	-	-	1	1	2
15067	15066	15067 -	15088 -	-	+	0	2	2
15067	15066	15067 -	15192 -	-	-	1	1	2
15070	15070	15074 +	15314 -	-	-	1	1	2
15072	15069	15076 -	15129	15125	15129 -	1	1	2
15072	15069	15076 -	15221	15221	15222 -	1	1	2
15072	15069	15076 -	15295 -	-	-	1	1	2
15079	15077	15082 -	15162 -	-	+	1	1	2
15079	15077	15082 -	15203 -	-	-	1	1	2
15079	15077	15082 -	15211 -	-	-	1	1	2
15079	15077	15082 -	15235 -	-	-	1	1	2
15079	15077	15082 -	15309 -	-	+	1	1	2
15084	15083	15085 -	15167 -	-	-	1	1	2
15084	15083	15085 -	15187	15187	15188 -	1	1	2
15085 -	-	+	15267 -	-	+	1	1	2
15089	15089	15090 -	15214 -	-	-	1	1	2
15089	15089	15090 -	15290 -	-	+	1	1	2
15104	15100	15108 -	15239 -	-	-	1	1	2
15104	15100	15108 -	15256 -	-	-	1	1	2
15104	15100	15108 -	15273 -	-	-	1	1	2
15111 -	-	+	15000 -	-	-	0	2	2
15114	15112	15117 -	15278 -	-	-	1	1	2
15114	15112	15117 -	15295 -	-	-	1	1	2
15114	15112	15117 -	15308	15308	15309 -	1	1	2
15114	15112	15117 -	15323	15323	15327 -	1	1	2
15117	15113	15121 +	14996 -	-	-	2	0	2
15117	15113	15121 +	15250 -	-	-	1	1	2
15117	15113	15121 +	15256	15256	15257 -	1	1	2
15117	15113	15121 +	15267	15267	15268 -	1	1	2
15124	15121	15124 -	15250 -	-	+	1	1	2
15124	15121	15124 -	15315	15312	15315 -	1	1	2
15129	15126	15130 -	15259 -	-	-	1	1	2
15129	15126	15130 -	15278 -	-	-	1	1	2
15129	15126	15130 -	15287 -	-	-	1	1	2
15130 -	-	+	15284 -	-	-	1	1	2

15134	-	-		-	15255	-	-		-	1	1	2
15138	-	-		+	15025	-	-		-	1	1	2
15139		15137	15140	-	15166	-	-		+	1	1	2
15144		15141	15146	-	15172	-	-		+	1	1	2
15144		15141	15146	-	15280	-	-		-	1	1	2
15144		15141	15146	-	15327	-	-		-	1	1	2
15144		15141	15146	-	15380		15380	15384	-	2	0	2
15146	-	-		+	15242	-	-		-	1	1	2
15150		15150	15154	-	15211	-	-		-	1	1	2
15164	-	-		-	15190	-	-		+	1	1	2
15169		15167	15169	-	15328	-	-		-	1	1	2
15172	-	-		+	15184	-	-		+	1	1	2
15188		15188	15189	+	15204	-	-		+	1	1	2
15188		15188	15189	+	15247	-	-		-	1	1	2
15191		15187	15195	-	15219	-	-		+	2	0	2
15191		15187	15195	-	15321		15321	15322	-	1	1	2
15195		15193	15195	+	15256	-	-		-	1	1	2
15195		15193	15195	+	15320		15320	15321	-	1	1	2
15195		15193	15195	+	15378	-	-		-	2	0	2
15197	-	-		-	15384	-	-		-	1	1	2
15205		15201	15208	+	15172		15172	15173	-	1	1	2
15205		15201	15208	+	15185	-	-		-	1	1	2
15205		15201	15208	+	15208		15208	15209	-	1	1	2
15206		15204	15209	-	15232	-	-		+	1	1	2
15206		15204	15209	-	15339	-	-		-	1	1	2
15206		15204	15209	-	15375	-	-		-	1	1	2
15211		15211	15212	-	15376	-	-		-	1	1	2
15212		15210	15212	+	15235		15235	15236	-	1	1	2
15220		15219	15220	-	15195	-	-		+	2	0	2
15225	-	-		-	15375	-	-		-	1	1	2
15236		15233	15236	-	15372	-	-		-	1	1	2
15242		15241	15245	-	15256	-	-		+	1	1	2
15242		15241	15245	-	15275		15271	15275	+	1	1	2
15256		15255	15258	+	15149	-	-		-	1	1	2
15256		15255	15258	+	15297	-	-		-	1	1	2
15263		15262	15266	+	15193		15190	15193	-	1	1	2
15280		15280	15283	+	15148	-	-		-	1	1	2
15280		15280	15283	+	15206	-	-		-	1	1	2
15280		15280	15283	+	15241		15241	15242	-	1	1	2
15304	-	-		+	15305	-	-		-	1	1	2
15314		15310	15314	+	15250	-	-		-	1	1	2
15314		15310	15314	+	15263	-	-		+	0	2	2
15322		15319	15323	+	15193	-	-		-	1	1	2
15322		15319	15323	+	15241		15241	15242	-	1	1	2
15322		15319	15323	+	15384	-	-		-	1	1	2
15351		15351	15352	+	15357		15357	15358	-	1	1	2
15378	-	-		+	15194	-	-		-	2	0	2
