# Supplementary material for: Identification and quantification of defective virus genomes in high throughput sequencing data using DVG-profiler, a novel post-sequence alignment processing algorithm
Source: PLoS One. 2019 May 17;14(5):e0216944. doi: 10.1371/journal.pone.0216944 (PMC6524942; doi:10.1371/journal.pone.0216944)
Supplement: S13 Table — (PDF) [file pone.0216944.s018.pdf]

Position (left)	Group start (left)	Group end (left)	Strandness	Position (right)	Group start (right)	Group end (right)	Strandness	Forward hits	Reverse hits	fwd and reverse	Mumps Reads	Total Reads
14687	14684	14690	-	15152	15149	15157	+	2332	2380	4712	2.34E+06	3.24E+06
5078	5076	5082	-	15083	15079	15086	+	126	132	258		
12145	12145	12146	-	14214	14213	14214	+	90	87	177		
3595	3592	3599	-	3626	3622	3629	+	14	156	170		
14391	14386	14392	-	15010	15010	15012	+	80	71	151		
3585	3582	3586	-	3636	3635	3636	+	138	0	138		
5065	5062	5067	-	15097	15096	15097	+	98	11	109		
13808	13804	13812	-	14885	-	-	+	54	53	107		
14008	14003	14011	-	14187	-	-	+	40	40	80		
6390	6384	6390	+	6338	6334	6338	-	31	28	59		
1031	1030	1035	-	1067	1063	1068	+	29	26	55		
13861	13861	13865	-	13879	13876	13882	+	27	27	54		
3722	3718	3726	-	7316	7316	7320	+	27	25	52		
1460	1459	1463	+	1398	1394	1398	-	26	25	51		
6322	6322	6324	-	6414	6414	6416	-	23	26	49		
13303	13303	13307	+	13261	13258	13261	-	23	23	46		
1	2	8	-	126	122	126	-	21	21	42		
14353	14353	14357	-	14497	14497	14501	-	40	2	42		
1179	1176	1179	-	1240	-	-	+	23	18	41		
10498	10495	10498	-	10515	10515	10517	+	39	2	41		
7926	7925	7927	-	7986	-	-	-	18	22	40		
7074	7070	7077	-	7131	7128	7131	-	1	38	39		
14062	14058	14066	-	14241	14238	14241	+	20	19	39		
14359	14358	14364	-	14503	14503	14504	-	1	38	39		
6171	6170	6171	-	6322	6322	6323	-	19	19	38		
665	662	668	+	634	633	636	-	19	18	37		
2702	2699	2706	+	2675	2673	2679	-	20	17	37		
748	744	750	+	708	706	710	-	17	19	36		
1293	1292	1297	+	1177	1175	1177	-	18	18	36		
1	2	8	-	31	27	31	+	18	17	35		
7069	-	-	-	7126	-	-	-	35	0	35		
14130	14127	14134	-	14200	14196	14200	-	17	18	35		
3292	3291	3295	-	3394	3392	3395	+	17	17	34		
13114	13112	13114	+	13039	13039	13041	-	20	14	34		
1016	1013	1021	+	961	956	961	-	16	17	33		
10676	10672	10678	+	10658	10657	10662	-	17	16	33		
5763	5760	5763	+	5741	5741	5744	-	16	16	32		
13039	13037	13041	-	13114	13112	13114	+	16	16	32		
14398	14398	14401	-	15045	15045	15047	+	17	15	32		
9129	9125	9134	-	9163	9161	9163	+	17	14	31		
213	209	217	+	98	97	103	-	15	15	30		
395	390	400	-	344	340	345	+	18	12	30		
1359	1355	1364	-	1485	1485	1490	-	14	15	29		
14818	14818	14820	-	14864	14863	14864	+	16	13	29		
14864	14864	14867	+	14818	14817	14818	-	15	14	29		
5277	5273	5279	-	5330	5329	5330	-	13	15	28		
7469	7465	7471	+	7452	7452	7454	-	15	13	28		
13284	13280	13287	-	13674	13674	13675	+	14	14	28		
14416	14413	14419	-	14601	-	-	+	15	13	28		
15366	15365	15369	+	15343	15340	15344	-	13	15	28		
380	378	383	-	450	449	452	+	13	14	27		
1407	1404	1410	-	1450	1447	1450	+	13	14	27		
1230	1227	1232	-	5977	5977	5979	+	14	12	26		
2662	2661	2665	+	2719	2715	2720	-	13	13	26		
6726	6722	6727	+	6964	6964	6965	+	15	11	26		
12641	12636	12645	-	12783	12781	12785	-	13	13	26		
12123	12119	12127	-	13012	13011	13012	+	14	11	25		
15251	15247	15256	+	15197	15193	15201	-	12	13	25		
633	631	637	+	666	663	666	-	13	11	24		
1240	1236	1244	+	1179	1179	1183	-	12	12	24		
3957	3953	3960	+	3940	3937	3944	-	12	12	24		
5861	5858	5865	-	6009	6009	6010	-	11	13	24		
8076	8072	8080	-	8229	8227	8229	-	12	12	24		
14776	14773	14779	-	14850	14847	14850	+	9	15	24		
3336	3333	3339	-	3353	3351	3354	+	11	11	22		
6813	6809	6817	+	6850	6848	6850	-	11	11	22		
6815	6811	6815	-	6834	6834	6838	+	19	3	22		
8961	8959	8965	-	9077	9077	9078	+	11	11	22		
10472	10469	10477	-	10520	10516	10520	+	12	10	22		
14905	14901	14908	-	14943	14943	14944	+	11	11	22		
13598	13593	13603	-	13783	13781	13787	-	10	11	21		
13872	13870	13877	-	14145	14143	14145	+	12	9	21		
341	338	345	-	398	398	400	+	13	7	20		
1488	1484	1491	+	1400	1396	1400	-	10	10	20		
1818	1813	1819	-	2002	2000	2003	-	10	10	20		
2675	2671	2678	+	2703	2700	2704	-	10	10	20		
2757	2755	2762	-	2799	2799	2802	-	10	10	20		
4804	4804	4808	-	4848	4844	4848	-	10	10	20		
7263	7260	7263	+	7221	-	-	-	10	10	20		
9197	9193	9202	-	9361	9359	9364	-	10	10	20		
9543	9539	9544	+	9723	9723	9725	-	10	10	20		
12536	12533	12537	-	12684	12684	12685	-	3	17	20		
13882	13880	13886	-	13924	13920	13924	+	10	10	20		
14069	14069	14071	-	14088	14087	14088	+	10	10	20		
15275	15271	15275	+	15241	-	-	-	10	10	20		
369	366	369	-	461	-	-	+	9	10	19		
1303	1298	1307	-	1346	1342	1346	+	10	9	19		
1383	1381	1386	-	1473	-	-	+	10	9	19		
7985	7981	7989	+	8151	8150	8155	-	10	9	19		
11827	11825	11829	-	11856	-	-	+	11	8	19		
13385	13384	13387	-	14475	14474	14475	+	10	9	19		
959	957	961	+	928	-	-	-	9	9	18		
1530	1527	1531	-	1686	1686	1688	-	9	9	18		
1552	1548	1557	-	1579	-	-	-	9	9	18		
4566	4563	4571	-	4644	4640	4644	+	10	8	18		

7528	7525	7531 +	7471 -	-	-	10	8	18
8148	8148	8150 +	7992 -	-	-	9	9	18
8978	8975	8982 -	9034	9034	9036 +	15	3	18
12082	12080	12087 +	12128	12128	12129 -	9	9	18
12134	12133	12137 +	12079	12076	12080 -	9	9	18
13024	13021	13027 -	13076	13073	13076 +	9	9	18
14692	14691	14697 -	15147	15145	15150 +	6	12	18
1	2	8 -	137	133	141 -	9	8	17
1177	1177	1181 +	1293	1289	1293 -	9	8	17
3448	3447	3451 -	3493	3490	3493 +	16	1	17
3701	3698	3703 +	7337	7337	7340 -	7	10	17
5291	5291	5293 -	5345	5345	5347 -	7	10	17
5801	5799	5804 +	5745	5745	5747 -	10	7	17
7452	7447	7452 +	7469 -	-	-	9	8	17
10699	10697	10700 -	10723 -	-	+	17	0	17
10705	10705	10708 -	10717 -	-	+	1	16	17
11269	11265	11273 -	11409	11406	11413 -	9	8	17
12837	12834	12841 -	12883 -	-	-	8	9	17
14676	14672	14680 +	14653	14650	14657 -	8	9	17
229	227	232 -	243	243	247 +	13	3	16
1230	1227	1232 -	7403	7403	7405 +	8	8	16
5313	5309	5316 -	5484	5484	5486 -	8	8	16
6435	6431	6436 -	6476	6476	6477 -	8	8	16
6834	6833	6834 -	6815 -	-	+	16	0	16
7107	7104	7111 -	7274	7273	7278 -	8	8	16
8935	8931	8935 +	8877	8877	8878 -	8	8	16
11269	11265	11273 -	11431	11427	11434 -	8	8	16
12304	12301	12304 -	12362 -	-	-	16	0	16
12309	12306	12311 -	12367 -	-	-	0	16	16
12529	12526	12530 -	12677	12674	12677 -	15	1	16
14333	14331	14337 -	14507	14506	14508 -	8	8	16
14866	14866	14872 -	14959	14955	14959 +	8	8	16
157	155	160 -	173	172	174 +	11	4	15
2028	2026	2028 +	12507	12506	12507 +	7	8	15
2662	2660	2668 -	2719	2715	2719 +	14	1	15
3453	3453	3456 -	3488 -	-	+	0	15	15
9030	9026	9034 -	8982	8978	8986 +	14	1	15
9905	9903	9906 -	11549 -	-	-	11	4	15
11881	11878	11884 -	11921	11919	11921 +	7	8	15
12747	12745	12751 -	12930	12926	12932 -	8	7	15
13056	13050	13058 -	13110	13106	13111 +	10	5	15
13184	13181	13187 -	13264 -	-	+	8	7	15
15104	15102	15108 -	15133	15131	15133 +	7	8	15
460	458	462 -	518	518	520 +	7	7	14
546	546	549 -	617	614	617 +	7	7	14
700	699	705 +	756	756	757 -	7	7	14
1267	1265	1270 -	1283	1281	1284 +	9	5	14
2157	2154	2158 +	2117	2117	2118 -	7	7	14
2719	2719	2721 -	2662 -	-	+	14	0	14
3072	3070	3072 +	3060	3060	3062 -	1	13	14
3564	3560	3567 -	3653	3650	3656 +	7	7	14
3944	3940	3945 +	3953	3952	3953 -	7	7	14
5009	5008	5015 -	5132	5131	5133 +	6	8	14
6603 -	-	+	6538 -	-	-	7	7	14
7091	7085	7094 -	7250	7247	7253 -	7	7	14
7091	7085	7094 -	7277	7273	7281 -	7	7	14
7852 -	-	-	8060 -	-	-	7	7	14
8396	8394	8400 -	8459	8459	8461 +	7	7	14
9905	9903	9906 -	10070	10067	10070 -	7	7	14
9994	9994	9997 +	9969	9965	9970 -	7	7	14
11538	11536	11539 -	11577	11575	11578 +	7	7	14
11976	11974	11976 -	14971 -	-	+	8	6	14
12082	12080	12087 +	11982	11981	11983 -	7	7	14
13753	13750	13757 -	14053	14052	14053 +	7	7	14
14220	14220	14223 -	14362 -	-	+	7	7	14
15144	15140	15147 -	15307	15304	15311 -	7	7	14
15219	15216	15219 +	15195	15194	15195 -	7	7	14
123	119	127 -	150	150	154 +	12	1	13
308	306	312 +	343 -	-	-	7	6	13
893	891	896 +	924	921	924 -	6	7	13
1381	1377	1381 +	1329	1329	1331 -	7	6	13
1513	1509	1515 -	1585 -	-	-	7	6	13
1807	1804	1811 -	2000	1999	2002 -	6	7	13
3077 -	-	+	3055 -	-	-	13	0	13
4089	4086	4094 -	4119	4115	4119 +	7	6	13
4219	4217	4224 -	4417	4414	4420 -	7	6	13
6338	6335	6341 +	6390 -	-	-	7	6	13
6474 -	-	+	6401 -	-	-	5	8	13
6543	6540	6543 -	6598 -	-	+	2	11	13
7549	7545	7549 -	7705 -	-	-	6	7	13
9451	9447	9454 -	9518 -	-	+	6	7	13
10004	10000	10008 -	10042	10042	10045 +	8	5	13
11577	11573	11577 -	11747 -	-	-	7	6	13
14016	14014	14020 -	14196	14195	14199 -	10	3	13
98	97	98 +	213 -	-	-	5	7	12
206	205	209 -	283 -	-	+	6	6	12
700	699	702 -	756	754	757 +	8	4	12
708	704	712 -	748	748	749 +	11	1	12
822	822	826 -	997	996	998 -	6	6	12
924	921	928 +	893	892	893 -	6	6	12
960	957	963 -	1103	1100	1104 -	6	6	12
1179	1176	1179 -	1293 -	-	+	6	6	12
3249	3247	3252 -	3273	3271	3275 +	8	4	12
3458	3457	3459 -	3524 -	-	+	5	7	12
4014	4010	4018 -	4043	4043	4044 +	7	5	12
4058	4058	4063 -	4072 -	-	+	7	5	12

4081	4077	4082 +	4102 -	-	+	6	6	12
4089	4086	4094 -	4107	4104	4107 +	6	6	12
4572 -	-	+	4824 -	-	-	6	6	12
4582	4578	4586 -	4618 -	-	+	5	7	12
5892 -	-	-	5960 -	-	-	12	0	12
6686	6684	6687 +	11420 -	-	+	6	6	12
7091	7085	7094 -	7231	7229	7234 -	6	6	12
7221	7220	7225 +	7263	7262	7263 -	5	7	12
7469	7465	7471 +	7528	7528	7531 -	7	5	12
8065	8062	8067 +	8145	8145	8148 -	6	6	12
8766	8764	8769 +	8708	8707	8712 -	6	6	12
10151	10151	10154 -	10252 -	-	+	6	6	12
10705	10705	10708 -	14864	14863	14865 +	8	4	12
11244	11243	11245 -	11403	11400	11405 -	6	6	12
11269	11265	11273 -	11441	11441	11443 -	5	7	12
11500	11495	11504 -	11620	11619	11623 -	6	6	12
11711	11710	11711 +	11950 -	-	-	6	6	12
12047	12047	12050 -	12085	12085	12089 -	4	8	12
12344 -	-	+	12312 -	-	-	6	6	12
12585	12584	12589 -	12668 -	-	-	6	6	12
12893	12892	12897 +	12806 -	-	-	6	6	12
13588	13586	13589 -	13783	13780	13783 -	6	6	12
13629	13627	13633 -	13782	13778	13783 -	6	6	12
13646	13645	13648 -	13782	13781	13786 -	6	6	12
13916	13915	13919 +	13968	13965	13968 -	6	6	12
14344	14339	14346 -	14515	14510	14515 -	6	6	12
14465	14463	14468 +	14427	14427	14428 -	7	5	12
14771	14767	14771 -	14858	14855	14860 +	8	4	12
14836	14836	14837 -	15057 -	-	+	7	5	12
15003	14999	15003 -	15167	15167	15170 -	6	6	12
15251	15247	15256 +	15188 -	-	-	6	6	12
15340	15336	15343 +	15369 -	-	-	6	6	12
343	343	345 +	308 -	-	-	6	5	11
853	849	856 +	9147	9147	9151 +	8	3	11
2022	2018	2024 +	2056	2055	2060 -	5	6	11
2443	2442	2446 -	2466	2466	2467 +	8	3	11
4058	4058	4063 -	4120	4120	4121 +	6	5	11
4315	4311	4315 +	8414	8414	8416 -	11	0	11
4591	4590	4593 -	4787	4783	4787 -	6	5	11
5898	5898	5900 -	5966 -	-	-	0	11	11
6078	6077	6078 +	6047	6047	6048 -	6	5	11
6514	6512	6519 -	6669	6666	6670 -	5	6	11
7221	7218	7221 -	7355 -	-	-	6	5	11
7591	7590	7595 -	7603	7599	7604 +	7	4	11
9710	9708	9714 -	9906 -	-	+	6	5	11
10004	10000	10008 -	9956	9952	9960 +	6	5	11
10043	10040	10048 +	10006	10005	10006 -	6	5	11
11102	11100	11107 -	11320	11316	11320 -	6	5	11
11127	11123	11131 -	11283	11279	11283 -	5	6	11
11840	11837	11842 -	11916	11915	11916 -	5	6	11
12322	12319	12326 +	12282	12280	12282 -	6	5	11
12562	12558	12566 -	12596	12596	12600 +	5	6	11
13039 -	-	+	13114 -	-	-	8	3	11
13139	13134	13141 +	13175	13173	13175 -	6	5	11
14428	14423	14428 -	14464	14464	14467 +	10	1	11
15024	15020	15025 +	15139	15138	15140 -	5	6	11
15144	15140	15147 -	15290	15286	15290 -	6	5	11
15144	15140	15147 -	15379	15376	15380 -	7	4	11
214	211	217 -	274	273	279 +	5	5	10
518	515	521 -	699	699	702 -	5	5	10
710	708	712 +	662	662	663 -	5	5	10
853	851	857 -	991	991	993 +	5	5	10
863	860	865 -	981	979	981 +	5	5	10
865	862	869 +	808	807	808 -	5	5	10
953	953	954 -	1272	1272	1273 -	5	5	10
1257	1257	1259 -	1276	1274	1276 +	6	4	10
1389	1388	1398 -	1460	1459	1463 +	5	5	10
1460	1459	1463 +	1442	1442	1443 -	5	5	10
1756	1755	1756 -	1848	1848	1849 -	5	5	10
1861	1858	1863 -	2001	2000	2001 -	5	5	10
1927	1927	1931 +	1947	1944	1948 +	5	5	10
2055	2053	2058 +	2023	2022	2023 -	5	5	10
2518	2515	2522 -	2547 -	-	-	5	5	10
2645	2641	2649 -	2694	2692	2694 +	6	4	10
3403	3398	3406 -	3515	3515	3516 +	5	5	10
3521	3516	3523 +	3538 -	-	-	4	6	10
3538	3534	3540 -	3600	3596	3600 +	5	5	10
3954	3951	3958 -	4112	4112	4117 -	5	5	10
4219	4217	4224 -	4400	4400	4402 -	5	5	10
4946	4946	4949 -	7745	7744	7745 +	5	5	10
5160	5159	5161 -	5320	5318	5320 -	5	5	10
5853	5850	5853 +	5873	5873	5875 -	5	5	10
6263	6262	6266 -	6310	6308	6310 +	5	5	10
6469	6469	6473 -	6523	6519	6523 +	7	3	10
7985	7981	7989 +	7953	7952	7956 -	5	5	10
7992	7991	7995 +	8148 -	-	-	5	5	10
8007	8007	8011 +	8007	8007	8008 -	5	5	10
8022	8020	8025 +	7975	7973	7975 -	5	5	10
8022	8020	8025 +	8078	8078	8081 -	5	5	10
8148	8148	8150 +	8118 -	-	-	5	5	10
8244	8238	8245 -	8411	8411	8413 -	5	5	10
8431	8430	8434 -	8563	8560	8564 -	5	5	10
8547	8543	8547 -	8712	8712	8714 +	5	5	10
9010	9009	9011 +	8979	8979	8980 -	5	5	10
10049	10045	10051 -	10213	10210	10217 -	5	5	10
10379	10376	10382 -	10528 -	-	-	5	5	10

10496	10496	10498 +	10517	10515	10517 -	3	7	10
10694	10694	10695 -	14876 -	-	+	5	5	10
10958	10957	10960 -	11151	11150	11154 -	5	5	10
11424	11420	11428 -	11596	11595	11597 -	5	5	10
11439	11436	11439 -	11573 -	-	+	10	0	10
11444	11444	11445 -	11568 -	-	+	0	10	10
11592	11588	11596 -	11612	11612	11614 +	5	5	10
11828 -	-	+	11703 -	-	-	5	5	10
11899	11897	11902 -	12074	12073	12076 -	5	5	10
12168	12164	12168 -	12218 -	-	+	4	6	10
12326	12323	12330 -	12490 -	-	-	5	5	10
12707	12703	12711 -	12834 -	-	+	4	6	10
12822	12822	12824 -	12856 -	-	+	0	10	10
12893	12892	12897 +	12871	12871	12876 -	5	5	10
13072	13069	13076 -	13218	13215	13219 -	5	5	10
13095	13095	13099 -	13160 -	-	+	5	5	10
13284	13280	13287 -	13476	13476	13478 -	5	5	10
13323	13320	13324 +	13232	13232	13233 -	5	5	10
13508	13508	13511 -	13588 -	-	-	5	5	10
13588	13586	13589 -	13601	13601	13602 +	5	5	10
13640	13634	13643 -	13703 -	-	-	5	5	10
13857	13854	13861 +	13821	13818	13821 -	5	5	10
13961	13956	13965 -	14063 -	-	-	5	5	10
13965	13962	13971 +	13919	13916	13919 -	5	5	10
14074	14073	14078 -	14083 -	-	+	5	5	10
14540	14536	14544 -	14649	14646	14652 -	5	5	10
14635	14634	14636 +	14649	14648	14651 -	5	5	10
14640	14640	14643 +	14687 -	-	-	5	5	10
14647	14645	14651 -	14784	14784	14787 -	5	5	10
14651	14651	14653 +	14634 -	-	-	5	5	10
14771	14767	14771 -	15028	15028	15029 -	5	5	10
14897	14892	14900 -	14952	14952	14953 +	5	5	10
14972	14969	14975 -	15155 -	-	+	5	5	10
15144	15140	15147 -	15280	15276	15280 -	5	5	10
15144	15140	15147 -	15324	15322	15324 -	4	6	10
15190	15189	15193 -	15380	15377	15384 -	5	5	10
15235	15233	15238 +	15204	15200	15206 -	5	5	10
229	227	232 -	260	259	260 +	6	3	9
325	325	327 -	14557 -	-	+	0	9	9
665	662	668 +	710	709	711 -	5	4	9
743	740	745 -	713 -	-	+	9	0	9
1132	1128	1137 -	1158	1154	1158 +	6	3	9
1408	1404	1412 +	1449	1445	1450 -	4	5	9
1444	1440	1444 -	1460 -	-	+	8	1	9
2265	2265	2266 +	2195	2194	2195 -	5	4	9
4535	4531	4535 -	4662 -	-	+	4	5	9
4566	4563	4571 -	4622 -	-	+	0	9	9
4566	4563	4571 -	4628 -	-	+	9	0	9
4877	4873	4878 +	4859	4858	4859 -	5	4	9
5039	5034	5039 -	5207 -	-	-	9	0	9
5046	5044	5049 -	5213	5212	5213 -	0	9	9
5101	5099	5104 -	5195 -	-	-	4	5	9
5313	5309	5316 -	5491	5491	5494 -	5	4	9
5328	5328	5331 +	5288 -	-	-	4	5	9
5476	5472	5480 -	6935 -	-	+	0	9	9
5976	5976	5977 -	12329 -	-	+	0	9	9
7285	7281	7287 +	7257	7257	7258 -	6	3	9
7791	7788	7794 -	7889 -	-	+	5	4	9
8978	8975	8982 -	11491 -	-	+	6	3	9
9372	9372	9375 +	9335	9333	9336 -	5	4	9
9956	9956	9959 -	10004	10003	10004 +	5	4	9
10036	10036	10039 -	13350 -	-	-	4	5	9
10049	10045	10051 -	10004	10000	10004 +	5	4	9
10287	10283	10292 -	10431	10427	10431 -	5	4	9
10596	10594	10599 +	10515 -	-	-	4	5	9
11235	11231	11238 -	11284	11280	11286 -	4	5	9
12033	12029	12033 +	11988	11988	11992 -	5	4	9
12159	12155	12161 -	12227	12225	12228 +	6	3	9
12225	12225	12227 +	12160	12159	12161 -	5	4	9
12343	12340	12346 -	12517	12517	12521 -	5	4	9
12723	12720	12723 -	12758 -	-	+	9	0	9
12754	12752	12759 -	12723	12723	12726 +	9	0	9
13938	13935	13942 -	14095	14094	14096 -	6	3	9
14324	14321	14326 -	14369 -	-	+	8	1	9
14435	14434	14439 -	14457	14456	14457 +	0	9	9
1	2	8 -	207	205	212 -	5	3	8
14	9	17 -	126	125	126 -	4	4	8
85	85	88 -	246	245	250 -	4	4	8
97	93	100 -	213	213	214 +	6	2	8
475	471	476 -	518	514	518 +	4	4	8
500	497	504 -	693	693	696 -	4	4	8
530	526	534 -	677	677	681 -	4	4	8
530	526	534 -	692	692	696 -	4	4	8
530	526	534 -	701	701	704 -	4	4	8
530	526	534 -	710	708	711 -	4	4	8
672	669	672 +	788	788	790 -	4	4	8
685	684	688 +	774 -	-	-	4	4	8
689	685	689 -	813 -	-	+	4	4	8
717	714	719 +	658	658	659 -	4	4	8
810	810	812 +	908 -	-	-	4	4	8
853	851	857 -	993	993	996 -	4	4	8
873	871	878 -	993	990	997 -	4	4	8
889	885	893 -	1075 -	-	-	4	4	8
960	957	963 -	1128	1128	1129 -	4	4	8
991	987	993 -	1024	1021	1024 +	4	4	8
1100	1098	1103 +	1127	1124	1128 -	4	4	8

1143		1142	1146	-	1323	-	-		+	4	4	8
1329		1327	1333	+	1381		1381	1383	-	4	4	8
1359		1355	1364	-	1533		1532	1535	-	4	4	8
1444		1440	1444	-	4427	-	-		+	5	3	8
1487		1484	1492	-	1675		1672	1675	-	3	5	8
1494		1494	1497	+	1444		1442	1444	-	7	1	8
1535		1534	1537	-	1685		1685	1689	-	4	4	8
1658		1655	1659	-	1785	-	-		-	8	0	8
1764		1759	1766	-	1787	-	-		+	4	4	8
1852		1849	1856	-	2004		2000	2004	-	4	4	8
2042		2039	2044	-	12521		12518	12521	-	1	7	8
2050		2048	2052	-	12529		12526	12531	-	3	5	8
2138	-	-		-	2264	-	-		-	4	4	8
2592		2590	2596	+	2494		2492	2494	-	4	4	8
2653		2652	2656	-	2825		2822	2825	-	4	4	8
2673		2670	2674	-	4611	-	-		-	4	4	8
2719		2715	2722	+	2662		2662	2663	-	4	4	8
2775		2771	2780	-	2869		2866	2869	+	4	4	8
2930		2928	2932	-	3104	-	-		-	4	4	8
3933		3932	3936	-	4267		4266	4267	-	4	4	8
4089		4086	4094	-	4225		4225	4227	-	4	4	8
4513	-	-		+	4483	-	-		-	4	4	8
4629		4629	4632	+	4777	-	-		-	4	4	8
4940		4935	4944	-	5077		5076	5081	-	4	4	8
4940		4935	4944	-	7753		7749	7754	+	4	4	8
4971		4971	4974	-	5009		5006	5009	+	4	4	8
5046		5044	5049	-	5100	-	-		+	4	4	8
5081		5078	5081	+	15080		15080	15083	-	1	7	8
5093		5093	5094	+	5055		5055	5057	-	2	6	8
5148		5147	5152	-	6682		6680	6683	+	0	8	8
5259		5259	5262	+	5225	-	-		-	8	0	8
5344		5342	5347	-	5398	-	-		+	4	4	8
5529		5526	5534	-	5647		5647	5649	-	4	4	8
5691		5689	5696	-	5710		5710	5712	+	6	2	8
5908		5904	5911	-	5874		5874	5878	+	7	1	8
5926		5923	5930	-	6109		6105	6109	-	4	4	8
6181		6179	6183	-	6222		6222	6224	+	4	4	8
6244		6242	6247	-	6410		6410	6412	-	4	4	8
6383	-	-		+	6345	-	-		-	4	4	8
6404		6400	6405	-	6448		6448	6450	+	4	4	8
6435		6431	6436	-	6628		6624	6628	-	4	4	8
6502		6502	6503	+	6489		6489	6490	-	4	4	8
6538		6535	6538	-	6603	-	-		+	8	0	8
6881		6878	6881	-	6980	-	-		-	4	4	8
6980		6975	6984	-	7018		7018	7022	+	4	4	8
7074		7070	7077	-	7138	-	-		-	4	4	8
7107		7104	7111	-	7249		7249	7250	-	4	4	8
7526		7522	7529	-	7692		7692	7694	-	4	4	8
7665		7664	7666	-	7858		7857	7859	-	4	4	8
7701		7699	7701	-	7774	-	-		+	4	4	8
7934		7931	7936	-	8038		8035	8038	+	4	4	8
8004		8000	8006	-	8011	-	-		+	8	0	8
8004		8000	8006	-	8127		8125	8127	-	4	4	8
8216		8216	8217	+	8358	-	-		-	4	4	8
8443		8440	8448	-	8612		8610	8612	-	4	4	8
8607		8603	8611	-	8750	-	-		-	4	4	8
8952		8951	8958	-	9060	-	-		+	4	4	8
8961		8959	8965	-	9024	-	-		+	4	4	8
8971		8966	8974	-	9021		9019	9023	+	5	3	8
9289		9287	9292	-	9484		9484	9487	-	4	4	8
9305		9300	9309	-	9499		9497	9499	-	4	4	8
9351		9345	9353	-	9421		9417	9421	+	4	4	8
9451		9447	9454	-	9569		9568	9571	-	4	4	8
9530		9526	9533	-	9738		9738	9740	+	4	4	8
9671		9668	9674	+	9706		9705	9708	-	4	4	8
9697		9697	9699	+	9658	-	-		-	4	4	8
9830		9830	9834	-	9888	-	-		+	0	8	8
9898		9898	9901	-	10068		10068	10070	-	4	4	8
10031		10028	10031	-	10096	-	-		+	4	4	8
10031		10028	10031	-	10182		10182	10186	-	5	3	8
10061		10060	10065	-	10233		10229	10235	-	4	4	8
10120		10117	10121	-	10208		10208	10210	+	4	4	8
10164		10164	10169	-	10204	-	-		+	4	4	8
10175		10171	10179	-	10208		10204	10208	+	4	4	8
10467		10465	10467	-	10611	-	-		-	4	4	8
10485		10483	10487	-	10633		10630	10633	-	4	4	8
10724		10723	10727	-	14846		14846	14850	+	4	4	8
10771		10768	10776	-	11139	-	-		-	4	4	8
11061		11058	11063	+	11005	-	-		-	4	4	8
11102		11100	11107	-	11142	-	-		+	4	4	8
11203		11202	11203	+	11329	-	-		-	4	4	8
11244		11243	11245	-	13219	-	-		+	4	4	8
11475		11475	11479	-	11581		11578	11581	+	5	3	8
11522		11518	11526	-	11703	-	-		-	4	4	8
11579		11578	11583	+	11478		11478	11479	-	4	4	8
11726		11724	11728	+	11625		11623	11625	-	4	4	8
12082		12080	12087	+	12195	-	-		-	4	4	8
12123		12119	12127	-	12236		12236	12240	-	4	4	8
12209		12206	12210	+	12181		12181	12182	-	4	4	8
12248		12244	12252	-	12383		12379	12383	-	4	4	8
12280		12278	12282	-	12507		12507	12508	-	4	4	8
12603		12600	12603	-	12547	-	-		+	4	4	8
12626		12622	12630	-	12784		12783	12787	-	4	4	8
12641		12636	12645	-	12789		12789	12790	-	4	4	8
12689		12687	12694	-	12776		12776	12777	-	1	7	8
12767		12765	12771	-	12926		12924	12928	-	4	4	8

12776	12774	12779 -	12927	12927	12930 -	5	3	8
12776	12776	12782 +	12870 -	-	-	4	4	8
12817	12814	12817 -	12861 -	-	+	8	0	8
12891	12890	12894 -	13076	13075	13078 -	4	4	8
13033	13030	13035 -	13215	13214	13215 -	4	4	8
13064	13062	13066 +	13019 -	-	-	4	4	8
13070	13070	13074 +	13001 -	-	-	4	4	8
13157	13153	13161 -	13196 -	-	-	4	4	8
13261	13260	13261 +	13303	13303	13304 -	4	4	8
13609	13608	13609 -	13784	13780	13784 -	4	4	8
13621	13620	13626 -	13782	13780	13784 -	4	4	8
13640	13634	13643 -	13783	13783	13784 -	4	4	8
13733	13733	13734 -	14715 -	-	+	5	3	8
13786	13782	13788 +	13972 -	-	-	4	4	8
13894	13893	13894 -	13912 -	-	+	8	0	8
13912	13908	13912 -	13894 -	-	+	8	0	8
14048	14044	14051 -	14197	14196	14199 -	4	4	8
14236 -	-	-	14268 -	-	+	8	0	8
14243	14238	14247 +	14166	14164	14166 -	4	4	8
14333	14331	14337 -	14454 -	-	-	4	4	8
14344	14339	14346 -	14487	14484	14487 -	4	4	8
14369	14368	14371 -	14324 -	-	+	8	0	8
14391	14386	14392 -	14585	14585	14586 -	4	4	8
14435	14434	14439 -	14536 -	-	+	4	4	8
14540	14539	14542 +	14447	14447	14448 -	6	2	8
14651	14651	14653 +	14676 -	-	-	3	5	8
14653	14652	14655 -	14676	14674	14676 +	4	4	8
14692	14691	14697 -	15154	15154	15157 +	4	4	8
14771	14767	14771 -	14929	14925	14929 -	4	4	8
14969	14969	14972 +	15118 -	-	-	4	4	8
14994	14990	14997 -	15169	15167	15169 -	4	4	8
15082	15080	15084 -	15228 -	-	-	4	4	8
15095	15093	15096 +	15287	15287	15288 -	3	5	8
15144	15140	15147 -	15181 -	-	+	4	4	8
14	9	17 -	5474	5471	5474 -	6	1	7
150	147	152 -	123 -	-	+	7	0	7
173	169	177 -	157 -	-	+	7	0	7
239	238	242 -	385	385	386 -	4	3	7
257	257	260 +	231	228	231 -	4	3	7
295	291	297 +	345 -	-	-	4	3	7
320	318	321 -	421 -	-	+	4	3	7
1375	1371	1379 -	1500	1497	1500 -	4	3	7
1499	1497	1499 -	1538	1537	1538 -	3	4	7
1535	1534	1537 -	1693 -	-	-	3	4	7
1613	1610	1616 +	1543 -	-	-	3	4	7
1632	1630	1632 +	1705 -	-	-	0	7	7
1663	1663	1665 -	1790 -	-	-	0	7	7
1766	1763	1770 +	1861 -	-	-	4	3	7
2177	2174	2181 +	2099 -	-	-	4	3	7
3033	3032	3033 -	13297	13296	13297 -	5	2	7
3064	3062	3068 -	3239	3234	3243 -	4	3	7
3360	3358	3360 +	3329 -	-	-	3	4	7
3511	3511	3514 -	3692	3692	3695 -	3	4	7
3521	3516	3523 +	3543	3542	3543 +	3	4	7
3545	3542	3549 -	3695	3692	3695 -	5	2	7
3906	3901	3906 -	3993 -	-	+	4	3	7
3975	3972	3978 -	4005	4002	4005 +	4	3	7
4020	4019	4020 +	4035	4034	4035 +	4	3	7
4170	4169	4171 -	4225	4225	4226 +	4	3	7
4329	4325	4329 -	4502	4502	4505 -	3	4	7
5046	5044	5049 -	5259 -	-	-	2	5	7
5254	5251	5254 +	5230 -	-	-	0	7	7
5443	5440	5447 -	5472	5471	5472 +	4	3	7
5741	5738	5745 +	5763 -	-	-	4	3	7
6029	6029	6033 -	6181	6181	6185 -	5	2	7
6506 -	-	-	6620	6620	6623 -	3	4	7
7091	7085	7094 -	7239	7236	7240 -	4	3	7
7132	7132	7135 -	7142 -	-	+	3	4	7
7772	7770	7775 +	7753	7750	7755 -	4	3	7
7988	7984	7990 -	8012	8009	8013 +	4	3	7
8060	8057	8063 -	8150	8150	8154 +	3	4	7
8272	8270	8276 -	8296	8294	8296 +	1	6	7
8459	8456	8463 +	8396	8394	8396 -	4	3	7
8834	8831	8834 -	11350	11350	11351 +	7	0	7
9679 -	-	+	9644 -	-	-	0	7	7
9684	9684	9687 +	9639 -	-	-	7	0	7
9824 -	-	-	9894 -	-	+	7	0	7
9914	9914	9918 -	10105	10101	10106 -	3	4	7
9948	9948	9951 -	10016 -	-	+	6	1	7
10071	10071	10072 -	10273 -	-	+	3	4	7
10195	10194	10198 -	10337	10335	10338 -	4	3	7
10485	10483	10487 -	10543	10539	10543 +	4	3	7
10777	10776	10780 +	12659	12657	12661 -	7	0	7
11005	11005	11008 -	11061 -	-	+	5	2	7
11500	11495	11504 -	11546	11544	11546 +	4	3	7
11781 -	-	+	11875 -	-	-	4	3	7
12792	12790	12795 +	12816	12812	12816 -	4	3	7
12988	12984	12991 -	13022 -	-	+	7	0	7
13106	13105	13109 +	13056	13055	13056 -	3	4	7
13176	13173	13180 +	13129	13129	13132 -	4	3	7
13328	13327	13328 +	13234 -	-	-	0	7	7
13334	13334	13336 +	13228 -	-	-	7	0	7
13867	13866	13867 -	13911 -	-	+	4	3	7
14143	14143	14148 -	14412	14410	14412 +	4	3	7
14241 -	-	-	14263 -	-	+	0	7	7
14435	14434	14439 -	14507 -	-	-	7	0	7

14442	14442	14446 -	14513 -	-	-	0	7	7
14554	14550	14558 -	14794	14791	14794 -	3	4	7
14577	14574	14583 -	14956	14954	14956 +	4	3	7
14951	14951	14952 -	14986 -	-	+	3	4	7
14972	14969	14975 -	15108 -	-	-	4	3	7
15144	15140	15147 -	15297	15297	15301 -	4	3	7
15144	15140	15147 -	15340	15337	15340 -	4	3	7
1	2	8 -	234	233	234 -	3	3	6
260	257	263 -	369 -	-	+	3	3	6
273	270	274 -	356 -	-	+	3	3	6
273	270	274 -	365	364	365 +	3	3	6
273	270	274 -	458 -	-	+	3	3	6
358	355	360 -	418 -	-	+	3	3	6
582	579	586 -	622	619	622 +	3	3	6
607	605	609 +	554	554	557 -	3	3	6
622	622	623 -	925	925	926 -	3	3	6
634	633	638 -	665 -	-	+	6	0	6
634	633	638 -	711 -	-	-	3	3	6
643	640	648 -	849	847	850 -	3	3	6
665	663	670 -	795 -	-	-	3	3	6
717	714	720 -	767	766	767 +	3	3	6
734	731	737 -	794	791	794 +	3	3	6
743	740	745 -	779	779	781 +	5	1	6
808	805	810 -	865	865	866 +	3	3	6
808	805	810 -	978	976	978 -	3	3	6
817	812	817 -	939	935	939 -	3	3	6
853	849	856 +	991 -	-	-	3	3	6
889	885	893 -	924	924	925 +	3	3	6
900	900	904 +	1019 -	-	-	3	3	6
917	912	917 +	904	900	904 -	3	3	6
960	957	963 -	1015	1011	1015 -	3	3	6
981	978	985 -	1180	1177	1180 -	5	1	6
991	989	993 +	853	851	853 -	3	3	6
1010	1008	1011 +	1021 -	-	-	0	6	6
1016	1013	1021 +	1010 -	-	-	6	0	6
1031	1026	1031 +	1067 -	-	-	2	4	6
1081	1077	1083 -	1215	1214	1215 -	3	3	6
1089	1084	1091 -	1210	1207	1211 -	3	3	6
1100	1096	1104 -	1307	1307	1308 -	3	3	6
1158	1155	1160 -	1303	1303	1306 -	3	3	6
1158	1155	1160 -	1332	1332	1334 -	3	3	6
1187	1185	1189 -	1334	1333	1334 -	3	3	6
1205	1201	1208 -	1333	1333	1334 -	3	3	6
1205	1201	1208 -	1380	1380	1383 -	3	3	6
1215	1210	1218 -	1593 -	-	-	3	3	6
1230	1227	1232 -	11175 -	-	-	0	6	6
1345	1343	1349 +	1307 -	-	-	3	3	6
1354	1351	1354 -	1535	1533	1536 -	3	3	6
1359	1355	1364 -	1429	1425	1429 +	3	3	6
1362	1359	1364 +	1451	1449	1451 -	3	3	6
1400	1398	1402 +	1488	1488	1490 -	3	3	6
1428	1425	1428 -	1524 -	-	-	3	3	6
1442	1441	1442 +	1460 -	-	-	3	3	6
1460	1456	1462 -	1442 -	-	+	6	0	6
1487	1484	1492 -	1517	1517	1518 +	3	3	6
1487	1484	1492 -	1684	1684	1688 -	3	3	6
1487	1484	1492 -	1695	1691	1695 -	3	3	6
1488	1484	1491 +	1449 -	-	-	0	6	6
1567	1564	1571 -	1697	1693	1698 -	3	3	6
1571	1571	1572 +	8130	8130	8131 +	6	0	6
1632	1632	1635 -	1705 -	-	+	6	0	6
1641	1637	1642 -	1696 -	-	+	0	6	6
1641	1637	1645 +	1696 -	-	-	6	0	6
1658	1655	1659 -	1820	1820	1821 -	3	3	6
1696	1696	1699 +	1641 -	-	-	0	6	6
1807	1804	1811 -	1986	1982	1986 -	3	3	6
1911	1908	1915 -	1973	1972	1974 +	4	2	6
1919	1918	1925 -	2092	2088	2092 -	3	3	6
2007 -	-	-	2159 -	-	-	3	3	6
2012	2010	2015 -	2112 -	-	+	3	3	6
2055	2053	2058 +	12531 -	-	+	6	0	6
2433	2432	2435 -	2583 -	-	-	3	3	6
2574	2574	2575 -	2761	2761	2762 -	3	3	6
2748	2745	2750 -	2921 -	-	-	3	3	6
2856	2853	2859 -	3014	3011	3014 -	3	3	6
2870 -	-	+	12448	12448	12449 +	3	3	6
2879	2878	2882 -	3050 -	-	-	3	3	6
2962 -	-	+	2941 -	-	-	3	3	6
3002	3002	3006 -	3129	3129	3131 -	3	3	6
3010 -	-	+	2961 -	-	-	3	3	6
3019	3018	3023 -	3033	3030	3033 +	1	5	6
3115	3115	3118 +	3136 -	-	-	3	3	6
3412	3408	3416 -	3568	3564	3568 -	3	3	6
3441	3438	3442 -	3572 -	-	-	3	3	6
3453	3453	3456 -	3485 -	-	-	3	3	6
3496	3494	3497 -	3568 -	-	-	2	4	6
3511	3511	3514 -	3647 -	-	-	3	3	6
3545	3542	3549 -	3599 -	-	+	3	3	6
3551	3550	3554 -	3700 -	-	-	1	5	6
3629	3626	3632 +	3670	3670	3672 +	3	3	6
3660	3657	3664 -	3810	3806	3810 -	3	3	6
3674	3671	3678 +	3715 -	-	-	3	3	6
3674	3671	3678 +	3759	3759	3760 -	3	3	6
3934	3931	3938 +	4268 -	-	+	3	3	6
3954	3951	3958 -	4084	4084	4085 -	3	3	6
3954	3951	3958 -	4129	4126	4129 -	3	3	6

3962 -	-	+	3936 -	-	-	3	3	6
3980	3976	3981 +	4085	4084	4087 -	4	2	6
4219	4217	4224 -	4374	4372	4374 -	3	3	6
4235	4233	4239 -	4373	4371	4376 -	3	3	6
4284	4284	4286 -	4409 -	-	-	6	0	6
4289	4288	4292 -	4414 -	-	-	0	6	6
4329	4325	4329 -	4487 -	-	-	3	3	6
4418	4414	4422 +	9671	9670	9671 +	1	5	6
4425	4425	4426 +	6931	6931	6932 +	4	2	6
4437	4436	4437 +	4571	4571	4572 -	3	3	6
4437	4436	4437 +	11469 -	-	+	3	3	6
4566	4563	4571 -	4785 -	-	-	3	3	6
4582	4578	4586 -	4817	4817	4818 +	3	3	6
4600	4597	4600 -	4673 -	-	+	3	3	6
4650	4646	4651 -	4851 -	-	-	3	3	6
4857	4855	4859 +	4869 -	-	+	3	3	6
4926	4923	4929 -	5236	5235	5236 -	4	2	6
4940	4935	4944 -	4981	4981	4982 -	3	3	6
4965	4962	4966 +	4871	4871	4872 -	3	3	6
5030	5026	5033 -	5134 -	-	+	3	3	6
5100	5099	5102 +	5049	5048	5049 -	5	1	6
5169	5166	5171 -	5316	5316	5319 -	3	3	6
5179	5176	5182 +	5652	5651	5652 +	3	3	6
5256	5255	5256 -	5286 -	-	+	6	0	6
5290	5290	5293 +	5207 -	-	-	3	3	6
5311	5307	5314 +	5496	5492	5496 +	3	3	6
5463	5460	5463 +	5399 -	-	-	3	3	6
5503	5501	5505 -	5560	5560	5561 +	3	3	6
5540	5540	5541 -	5561 -	-	+	6	0	6
5545	5544	5548 -	5556 -	-	+	0	6	6
5558	5555	5563 -	5647 -	-	-	0	6	6
5558	5555	5563 -	5666	5666	5667 -	3	3	6
5605	5605	5609 -	5660	5656	5660 +	3	3	6
5619	5615	5619 -	5666	5666	5670 +	3	3	6
5679	5676	5682 -	5664	5664	5667 +	5	1	6
5824	5824	5825 +	5915	5915	5916 -	3	3	6
5842 -	-	+	5913 -	-	-	3	3	6
5874	5870	5874 -	5908 -	-	+	6	0	6
6397	6396	6397 +	6427 -	-	+	3	3	6
6457	6456	6457 -	6669	6665	6669 -	3	3	6
6464	6460	6464 -	6528	6528	6529 +	3	3	6
6694	6692	6699 +	6657	6655	6657 -	3	3	6
6726	6722	6727 +	6755	6755	6756 +	3	3	6
6770	6769	6771 -	6899	6899	6900 -	3	3	6
6850	6848	6855 +	6830 -	-	-	3	3	6
6866	6862	6866 -	6969	6965	6969 -	3	3	6
6933	6929	6933 +	6911 -	-	-	3	3	6
6935	6934	6935 -	7131 -	-	-	3	3	6
6971	6970	6972 -	7092	7090	7092 +	3	3	6
7035 -	-	-	7119 -	-	-	3	3	6
7051	7050	7052 -	7230	7230	7231 -	3	3	6
7141	7137	7141 -	7242 -	-	+	3	3	6
7257	7254	7260 -	7310	7307	7310 +	3	3	6
7263	7260	7263 +	12426 -	-	+	6	0	6
7289	7285	7289 -	7442	7440	7442 -	3	3	6
7420	7416	7426 -	7635	7635	7639 -	3	3	6
7463	7460	7463 -	7965 -	-	+	3	3	6
7538	7537	7541 -	7672	7672	7674 -	3	3	6
7573	7569	7574 -	7635	7635	7636 -	3	3	6
7591	7590	7595 -	7804	7803	7805 -	3	3	6
7612	7610	7613 -	7773	7772	7773 +	3	3	6
7729	7727	7733 -	7878	7875	7879 -	4	2	6
7882	7880	7882 -	7903 -	-	+	3	3	6
7894	7891	7899 -	8339 -	-	-	3	3	6
7926	7925	7927 -	8022 -	-	+	3	3	6
7988	7984	7990 -	8201 -	-	-	3	3	6
8046 -	-	+	7991 -	-	-	3	3	6
8048	8044	8049 -	8073	8073	8074 +	3	3	6
8060	8057	8063 -	8202	8200	8202 -	3	3	6
8076	8072	8080 -	8239	8239	8241 -	3	3	6
8092	8089	8093 -	8176	8176	8178 +	3	3	6
8094	8092	8095 +	7991 -	-	-	3	3	6
8200	8199	8200 -	8401	8397	8401 -	4	2	6
8232	8231	8236 -	8383	8381	8383 -	3	3	6
8285 -	-	+	8230 -	-	-	3	3	6
8373	8373	8376 -	8347	8347	8348 +	5	1	6
8403	8400	8406 +	8363	8360	8363 -	3	3	6
8443	8440	8448 -	8559	8559	8560 -	3	3	6
8577	8573	8577 -	8753 -	-	-	3	3	6
8674	8673	8676 -	8766 -	-	+	3	3	6
8687	8684	8691 -	8861	8859	8862 -	3	3	6
8748	8746	8748 -	8796 -	-	+	3	3	6
8757	8757	8758 +	8698	8698	8699 -	3	3	6
8794	8791	8797 -	8905 -	-	+	3	3	6
8873	8870	8876 +	8976	8976	8977 -	3	3	6
8879	8879	8881 +	9047	9047	9048 -	3	3	6
8923	8921	8926 -	9141	9138	9145 -	3	3	6
8950	8946	8952 +	9019 -	-	-	3	3	6
8971	8966	8974 -	9047	9045	9048 +	3	3	6
8995 -	-	+	8948 -	-	-	3	3	6
9129	9125	9134 -	9154	9151	9158 +	2	4	6
9197	9193	9202 -	9310	9307	9310 -	3	3	6
9305	9300	9309 -	9436	9434	9436 +	3	3	6
9520	9520	9521 +	9744	9744	9745 -	3	3	6
9598	9597	9602 +	9525 -	-	-	3	3	6
9606	9602	9606 -	9767	9764	9767 -	3	3	6

9637	9634	9644	+	9569	-	-	-	3	3	6
9657	9657	9658	+	9677	-	-	-	3	3	6
9734	9734	9738	+	9640	-	-	-	3	3	6
9781	9779	9782	-	9868	-	-	+	3	3	6
9853	9852	9856	-	9900	9897	9901	+	3	3	6
9886	9882	9888	-	9935	9931	9935	+	3	3	6
9899	9896	9899	+	9974	-	-	-	3	3	6
9948	9948	9951	-	14118	14117	14119	-	0	6	6
10305	10301	10305	-	10332	10331	10332	+	3	3	6
10305	10301	10305	-	10429	-	-	-	3	3	6
10343	10342	10344	-	10402	-	-	+	3	3	6
10361	10358	10364	-	10467	10466	10468	+	4	2	6
10398	10394	10403	-	10529	-	-	-	3	3	6
10472	10469	10477	-	10632	10629	10632	-	3	3	6
10485	10483	10487	-	10642	-	-	-	3	3	6
10517	10515	10517	+	10496	10496	10498	-	4	2	6
10525	10523	10528	-	10585	-	-	-	3	3	6
10590	10588	10591	-	10669	10669	10670	+	3	3	6
10590	10588	10591	-	10679	-	-	+	6	0	6
10590	10588	10591	-	10730	-	-	-	3	3	6
10595	10594	10596	-	10664	10664	10666	+	3	3	6
10595	10594	10596	-	10673	-	-	+	0	6	6
10657	10655	10659	+	10677	10676	10677	-	2	4	6
10756	10754	10759	-	10977	10974	10977	-	3	3	6
10764	-	-	-	10830	-	-	+	3	3	6
10952	-	-	-	11152	11151	11152	-	3	3	6
10958	10957	10960	-	11103	-	-	-	3	3	6
11173	11170	11173	+	11128	-	-	-	3	3	6
11295	11292	11295	-	11430	-	-	-	3	3	6
11308	-	-	+	11259	11259	11260	-	3	3	6
11398	11397	11402	+	11421	11418	11421	-	3	3	6
11494	-	-	-	14253	-	-	+	3	3	6
11529	11528	11531	-	11678	11676	11678	-	3	3	6
11569	11566	11571	-	11730	11729	11730	-	3	3	6
11625	11622	11625	+	11726	-	-	-	3	3	6
11726	11723	11727	-	11904	11902	11905	-	3	3	6
11881	11878	11884	-	12014	12014	12017	-	3	3	6
11968	11967	11970	-	12103	12099	12103	-	3	3	6
12027	12027	12028	-	12123	-	-	+	3	3	6
12033	12029	12033	+	11956	11954	11956	-	4	2	6
12041	12040	12044	-	12205	12205	12206	-	3	3	6
12092	12087	12098	-	12239	12237	12240	-	3	3	6
12123	12119	12127	-	12228	12228	12229	-	3	3	6
12123	12119	12127	-	12298	12298	12302	+	3	3	6
12128	12124	12129	+	12085	12085	12086	-	3	3	6
12136	12132	12142	-	12288	12284	12288	+	3	3	6
12274	12271	12275	+	12184	12183	12187	-	3	3	6
12288	12286	12291	-	12347	-	-	+	3	3	6
12301	12298	12301	+	12247	12247	12248	-	3	3	6
12360	-	-	-	12493	-	-	+	3	3	6
12376	12373	12377	-	12609	-	-	-	3	3	6
12486	-	-	+	12466	-	-	-	3	3	6
12497	12493	12498	+	12533	12533	12534	-	3	3	6
12596	12595	12597	-	12769	12769	12772	-	3	3	6
12608	12606	12611	-	12783	12783	12787	-	3	3	6
12635	12634	12635	-	12787	-	-	-	3	3	6
12641	12636	12645	-	12750	12750	12752	-	3	3	6
12682	12679	12682	-	12769	-	-	-	6	0	6
12682	12679	12682	-	13774	-	-	+	3	3	6
12747	12745	12751	-	12938	12937	12939	-	3	3	6
12792	12790	12795	+	12729	-	-	-	3	3	6
12806	12803	12809	+	12893	-	-	-	3	3	6
12851	-	-	-	12894	-	-	-	3	3	6
12861	12859	12864	+	12817	12816	12817	-	5	1	6
12871	12871	12872	+	12901	12901	12902	+	3	3	6
12974	12974	12976	-	12999	-	-	+	6	0	6
13001	12997	13001	-	13070	-	-	+	3	3	6
13009	13006	13012	-	13063	13060	13064	+	3	3	6
13024	13021	13027	-	12988	-	-	+	6	0	6
13024	13021	13027	-	13128	-	-	+	3	3	6
13024	13021	13027	-	13147	13144	13147	-	3	3	6
13024	13021	13027	-	13215	13215	13217	-	3	3	6
13048	13045	13049	-	13115	-	-	+	3	3	6
13055	13052	13057	+	13199	13195	13199	-	3	3	6
13101	13101	13103	+	13153	13153	13154	-	3	3	6
13124	13124	13127	-	13182	13182	13184	+	3	3	6
13178	13178	13180	-	13367	13366	13368	-	3	3	6
13229	13226	13230	-	13327	-	-	+	3	3	6
13236	13236	13238	+	13326	-	-	-	3	3	6
13243	-	-	-	13313	-	-	+	3	3	6
13251	13250	13251	-	13273	-	-	+	3	3	6
13334	13334	13336	+	13365	-	-	-	3	3	6
13621	13620	13626	-	13671	13671	13672	+	3	3	6
13705	13702	13708	-	13860	13860	13861	-	3	3	6
13726	13725	13728	-	13758	-	-	+	4	2	6
13823	13819	13827	-	14005	14004	14005	-	3	3	6
13867	13865	13867	+	13911	-	-	-	4	2	6
13891	13887	13894	+	13796	13796	13797	-	6	0	6
13948	13944	13951	-	14052	14050	14052	-	3	3	6
13990	13990	13992	+	14066	-	-	-	3	3	6
13997	13995	14001	-	14157	14156	14157	+	2	4	6
14022	14022	14023	-	14202	-	-	-	0	6	6
14062	14058	14066	-	14199	14195	14199	-	3	3	6
14178	14175	14182	-	14215	14213	14215	+	6	0	6
14178	14175	14182	-	14299	-	-	-	3	3	6
14271	14270	14273	-	14441	-	-	-	3	3	6

14324		14321	14326	-	14496		14496	14497	-	3	3	6
14333		14331	14337	-	14480		14478	14480	-	3	3	6
14391		14386	14392	-	14590		14588	14590	-	3	3	6
14507		14503	14509	+	14484	-	-		-	3	3	6
14569		14566	14572	-	14867		14867	14868	+	3	3	6
14667		14663	14670	-	14737	-	-		+	3	3	6
14681		14681	14683	-	15062		15062	15063	-	3	3	6
14719		14716	14723	-	14738		14737	14738	+	1	5	6
14776		14773	14779	-	14938	-	-		-	3	3	6
14806		14802	14807	-	14835	-	-		-	6	0	6
14812		14809	14813	-	14841	-	-		-	0	6	6
14812		14809	14813	-	14978		14974	14978	+	3	3	6
14824		14824	14827	+	14857		14857	14858	-	3	3	6
14847		14843	14851	-	15044	-	-		+	3	3	6
14913		14910	14917	-	14935		14931	14935	+	3	3	6
14957		14956	14957	-	15084		15084	15087	-	3	3	6
15052		15049	15055	-	15212		15209	15212	-	3	3	6
15112		15110	15115	-	15240		15240	15244	-	3	3	6
15112		15110	15115	-	15298		15297	15298	-	3	3	6
15117		15116	15117	-	15302	-	-		-	3	3	6
15134		15133	15138	-	15277	-	-		-	3	3	6
15144		15140	15147	-	15384		15382	15384	-	4	2	6
15148		15145	15148	+	15280	-	-		-	3	3	6
15166		15163	15169	-	15323	-	-		-	3	3	6
15281		15280	15282	+	15241		15241	15242	-	3	3	6
15297		15294	15297	+	15241		15241	15244	-	3	3	6
15347		15347	15348	+	15172	-	-		-	3	3	6
29		28	32	-	76	-	-		-	2	3	5
173	-	-		+	157	-	-		-	3	2	5
246		243	249	+	229		229	232	-	4	1	5
290		288	295	-	427		424	427	-	3	2	5
357		357	358	+	280		280	282	-	3	2	5
454		450	454	-	513		510	513	+	3	2	5
557		557	560	-	606	-	-		+	3	2	5
665		663	670	-	634	-	-		+	5	0	5
665		663	670	-	847		845	849	-	2	3	5
672		672	676	-	895		895	898	-	3	2	5
717		714	719	+	741		741	742	-	1	4	5
717		714	719	+	767		765	768	-	3	2	5
750		748	752	-	774	-	-		+	0	5	5
757		755	761	+	699		699	700	-	2	3	5
838		836	838	-	9136		9134	9136	-	2	3	5
853		851	857	-	9155		9153	9155	-	0	5	5
1074		1074	1075	+	1085		1084	1085	-	3	2	5
1081		1077	1083	-	1238		1238	1242	-	3	2	5
1121		1117	1124	-	1256		1253	1256	-	2	3	5
1158		1155	1160	-	1287		1282	1287	-	2	3	5
1240		1238	1244	-	1179	-	-		+	4	1	5
1323		1319	1323	+	1143		1143	1147	-	2	3	5
1383		1381	1386	-	1678		1678	1679	+	2	3	5
1389		1388	1398	-	1468		1465	1468	+	2	3	5
1400		1399	1401	-	1488	-	-		+	5	0	5
1449		1447	1453	-	2238	-	-		-	4	1	5
1472		1472	1474	+	1640	-	-		-	3	2	5
1487		1484	1492	-	1724	-	-		-	0	5	5
1494		1494	1497	+	1461	-	-		-	0	5	5
1499	-	-		+	1456	-	-		-	5	0	5
1513		1509	1515	-	1637		1636	1637	-	3	2	5
1641		1637	1642	-	1672	-	-		-	0	5	5
1671		1668	1674	-	1696		1695	1696	+	2	3	5
1671		1668	1674	-	1834		1834	1837	-	3	2	5
1705		1705	1708	+	1632	-	-		-	5	0	5
1786		1785	1790	-	2000		1998	2000	-	3	2	5
1919		1918	1925	-	2079		2076	2079	-	2	3	5
2143		2143	2145	-	2189		2189	2190	-	2	3	5
2174		2171	2174	-	2314	-	-		-	5	0	5
2181	-	-		-	2321	-	-		-	0	5	5
2324		2322	2326	-	2651	-	-		-	3	2	5
2466		2465	2468	-	2443	-	-		+	5	0	5
2492		2489	2495	-	2531		2527	2531	-	2	3	5
2532		2528	2532	-	12082	-	-		-	5	0	5
2548		2545	2552	-	12082	-	-		-	5	0	5
2581		2581	2585	-	2605		2603	2605	+	3	2	5
2711		2708	2716	-	2663		2663	2667	+	3	2	5
2757		2755	2762	-	2921		2917	2921	-	3	2	5
2927	-	-		+	2980	-	-		-	2	3	5
3115		3114	3115	-	3136		3136	3137	+	3	2	5
3168		3167	3169	+	3150		3149	3151	-	2	3	5
3403		3398	3406	-	3586		3585	3586	-	2	3	5
3433		3430	3436	-	3500	-	-		+	5	0	5
3441		3438	3442	-	3495	-	-		+	0	5	5
3693		3688	3693	+	3674		3674	3676	-	3	2	5
3758	-	-		-	3774	-	-		+	5	0	5
3774		3773	3774	-	3758	-	-		+	5	0	5
3849		3844	3849	-	3966		3962	3966	-	3	2	5
3849		3847	3853	+	14297	-	-		-	3	2	5
4094		4092	4097	+	3972		3972	3973	-	3	2	5
4219		4217	4224	-	4601		4601	4604	-	3	2	5
4235		4233	4239	-	4437	-	-		-	1	4	5
4869	-	-		-	9713	-	-		+	2	3	5
4940		4935	4944	-	4879		4879	4880	+	4	1	5
4940		4935	4944	-	12865	-	-		-	3	2	5
4990		4985	4991	-	5024		5024	5026	+	2	3	5
5019		5019	5020	-	5156	-	-		-	5	0	5
5030		5026	5033	-	5162	-	-		-	0	5	5
5313		5309	5316	-	5500		5496	5500	-	1	4	5

5503	5501	5505	-	10937	10936	10937	+	3	2	5
5513	-	-	-	5553	-	-	+	5	0	5
5552	-	-	-	5641	-	-	-	5	0	5
5662	5658	5664	-	5679	-	-	+	5	0	5
5710	5707	5710	-	5798	-	-	+	3	2	5
5741	5738	5745	+	5804	5801	5804	-	3	2	5
6338	6335	6341	+	6422	-	-	-	3	2	5
6390	6384	6390	+	6494	-	-	-	3	2	5
6514	6512	6519	-	6473	6473	6474	+	5	0	5
6933	6929	6933	+	6818	6817	6818	-	3	2	5
6954	6954	6958	-	7100	7100	7103	-	0	5	5
7045	7041	7045	-	7091	7091	7093	+	2	3	5
7077	7073	7080	+	7484	7484	7486	+	3	2	5
7097	7095	7097	-	7267	7263	7268	-	2	3	5
7245	7245	7247	-	7314	-	-	-	0	5	5
7485	7485	7488	-	7515	7512	7515	+	3	2	5
7753	7750	7757	+	7774	7772	7774	-	2	3	5
7772	7770	7775	+	7715	-	-	+	2	3	5
7809	7805	7812	+	11141	-	-	+	0	5	5
7820	7818	7824	+	7690	7686	7690	-	2	3	5
8155	-	-	+	7985	-	-	-	3	2	5
8264	8264	8267	-	8301	8301	8304	+	5	0	5
8351	-	-	-	8370	-	-	+	5	0	5
8366	8366	8369	-	8355	-	-	+	5	0	5
8484	8482	8486	-	8681	8678	8682	-	2	3	5
8885	8884	8885	+	8928	-	-	-	2	3	5
8929	-	-	+	8971	-	-	-	3	2	5
9005	9003	9008	-	9125	9123	9125	-	2	3	5
9176	9175	9176	-	9236	9236	9237	+	4	1	5
9183	9182	9186	-	9229	9228	9229	+	1	4	5
9197	9193	9202	-	9417	9416	9417	-	3	2	5
9197	9197	9201	+	9126	-	-	-	3	2	5
9429	-	-	+	9366	-	-	-	0	5	5
9434	9434	9437	+	9361	-	-	-	5	0	5
9593	9593	9594	+	9554	-	-	-	0	5	5
9760	9758	9760	-	9931	9931	9932	-	2	3	5
9914	9914	9918	-	10070	10068	10070	-	2	3	5
9996	9993	9996	-	10054	10054	10055	+	3	2	5
10016	10012	10016	-	9948	-	-	+	5	0	5
10031	10028	10031	-	10173	10172	10173	-	2	3	5
10195	10194	10198	-	10383	10383	10384	-	2	3	5
10398	10394	10403	-	10588	10585	10588	-	3	2	5
10459	10455	10459	-	10551	-	-	+	3	2	5
10699	10697	10700	-	14869	14869	14873	+	3	2	5
10771	10768	10776	-	12662	12662	12663	+	2	3	5
11260	11260	11261	+	11340	11339	11340	-	2	3	5
11439	11436	11439	-	11578	11576	11579	-	3	2	5
11770	11769	11770	-	11785	11785	11786	+	5	0	5
11875	-	-	+	11781	-	-	-	3	2	5
11932	-	-	+	11953	-	-	-	2	3	5
11942	11940	11943	-	11974	11974	11976	+	3	2	5
12027	-	-	+	12018	-	-	-	0	5	5
12033	12029	12033	+	12014	-	-	-	5	0	5
12047	12047	12050	-	12211	12210	12211	-	2	3	5
12053	12051	12054	-	12103	12103	12104	+	4	1	5
12257	12257	12258	-	12395	-	-	-	2	3	5
12309	12306	12311	-	12347	12345	12347	+	3	2	5
12326	12323	12330	-	12549	12549	12553	-	3	2	5
12388	12387	12388	-	14344	-	-	+	3	2	5
12468	-	-	-	12493	-	-	+	5	0	5
12473	12471	12477	-	12488	-	-	+	0	5	5
12521	12518	12521	+	13340	-	-	+	4	1	5
12542	12541	12542	-	12603	12603	12604	+	4	1	5
12805	12803	12806	-	12872	12872	12876	+	2	3	5
12907	12903	12910	-	13035	-	-	-	3	2	5
13048	13046	13048	+	13107	13105	13107	-	4	1	5
13104	13101	13106	-	14090	-	-	+	2	3	5
13188	13184	13193	+	13065	-	-	-	2	3	5
13446	13442	13449	-	13665	13665	13667	-	3	2	5
13629	13627	13633	-	13658	-	-	+	3	2	5
13658	13658	13662	-	14035	14035	14038	-	2	3	5
13743	13739	13745	-	13890	-	-	+	2	3	5
13829	-	-	+	13776	-	-	-	2	3	5
13839	13836	13840	-	13980	13977	13980	-	3	2	5
13884	13882	13884	+	13803	-	-	-	0	5	5
13965	13962	13971	+	13772	-	-	-	2	3	5
13965	13962	13971	+	14198	14198	14202	-	3	2	5
13973	13972	13974	-	14176	14175	14176	-	3	2	5
13990	13990	13992	+	14073	-	-	-	2	3	5
13997	13995	14001	-	14048	14048	14050	+	3	2	5
14035	14035	14036	-	14055	14055	14056	+	3	2	5
14210	14208	14210	+	14182	-	-	-	0	5	5
14215	14215	14218	+	14177	-	-	-	5	0	5
14268	14268	14270	+	14236	-	-	-	3	2	5
14271	14270	14273	-	14348	-	-	+	5	0	5
14276	14276	14278	-	14344	-	-	+	0	5	5
14409	14406	14410	-	14578	-	-	+	5	0	5
14416	14413	14419	-	14574	-	-	+	0	5	5
14421	-	-	-	14472	-	-	+	3	2	5
14551	14547	14554	+	14504	14503	14504	-	2	3	5
14675	14674	14679	-	14626	14622	14626	+	2	3	5
14675	14674	14679	-	14687	-	-	+	2	3	5
14687	14684	14690	-	14715	14714	14715	+	2	3	5
14776	14773	14779	-	14927	-	-	-	3	2	5
14782	14780	14782	-	15001	14997	15001	-	3	2	5
14847	-	-	+	15044	-	-	-	3	2	5

14963		14959	14966	-	15132		15132	15133	-	3	2	5
15014		15010	15018	-	15211		15210	15212	-	3	2	5
15117		15116	15117	-	15146		15143	15146	+	2	3	5
30		29	33	+	139		135	139	-	2	2	4
30		29	33	+	7309	-	-		-	2	2	4
106		105	106	-	187	-	-		+	2	2	4
106		105	106	-	206	-	-		+	0	4	4
125		122	125	+	227	-	-		+	2	2	4
130		130	131	-	143	-	-		+	0	4	4
187	-	-		+	106	-	-		-	2	2	4
189		187	190	-	341	-	-		-	2	2	4
189		187	190	-	575	-	-		-	4	0	4
195	-	-		-	580	-	-		-	0	4	4
214		211	217	-	357	-	-		-	2	2	4
222		222	227	+	182	-	-		-	2	2	4
239		238	242	-	429	-	-		-	2	2	4
244		244	245	-	1887	-	-		-	2	2	4
273		270	274	-	469	-	-		+	2	2	4
290		288	295	-	344	-	-		+	2	2	4
290		288	295	-	609	-	-		+	2	2	4
347		347	350	-	559		555	559	-	2	2	4
347		347	350	-	566	-	-		-	2	2	4
352		351	353	-	516	-	-		+	2	2	4
358		355	360	-	536	-	-		-	2	2	4
358		355	360	-	561	-	-		-	2	2	4
374		372	375	-	533	-	-		-	2	2	4
393		390	396	+	546	-	-		-	2	2	4
395		390	400	-	505		505	506	-	2	2	4
395		390	400	-	6934		6934	6935	-	2	2	4
411		408	414	-	520		520	521	+	2	2	4
438		433	438	-	597	-	-		-	2	2	4
460		458	462	-	1052	-	-		-	2	2	4
464		461	466	+	415		412	415	-	2	2	4
470		468	470	-	558	-	-		-	2	2	4
470		468	470	-	639	-	-		-	2	2	4
475		471	476	-	617	-	-		+	2	2	4
500		497	504	-	649	-	-		-	2	2	4
518		515	521	-	704	-	-		-	2	2	4
526		526	530	+	552	-	-		+	2	2	4
552		548	552	+	386		386	390	-	1	3	4
576		573	576	-	851	-	-		-	4	0	4
582		579	586	-	695		695	696	-	2	2	4
582		579	586	-	857	-	-		-	0	4	4
584		583	585	+	602		602	603	-	2	2	4
607		605	609	+	579		579	581	-	2	2	4
617		614	617	-	722	-	-		-	2	2	4
643		640	648	-	854	-	-		-	2	2	4
645	-	-		+	782	-	-		-	2	2	4
652		650	655	-	860	-	-		-	2	2	4
665		663	670	-	815		815	817	-	2	2	4
665		663	670	-	1032	-	-		-	2	2	4
672		672	676	-	775		773	775	-	2	2	4
677		676	681	+	738		738	739	-	2	2	4
678		678	679	-	896	-	-		-	2	2	4
700		699	705	+	670		670	673	-	2	2	4
735		735	739	+	681		680	681	-	3	1	4
743		740	745	-	771	-	-		+	2	2	4
748		744	750	+	674		674	675	-	2	2	4
756		756	760	-	700	-	-		+	4	0	4
779		777	786	-	862	-	-		+	2	2	4
779		777	786	-	909		909	910	-	2	2	4
779		777	786	-	942		940	942	-	2	2	4
789		787	791	-	941	-	-		-	2	2	4
808		805	810	-	951	-	-		-	2	2	4
808		805	810	-	995		993	995	-	2	2	4
808		805	810	-	1009	-	-		-	2	2	4
822		822	826	-	957		957	958	-	2	2	4
832		828	835	-	890	-	-		+	2	2	4
838		836	838	-	871	-	-		-	2	2	4
844		840	845	-	872		871	873	+	2	2	4
863		860	865	-	1005		1001	1005	-	2	2	4
873		871	878	-	1022		1022	1025	-	2	2	4
873		871	878	-	1041	-	-		-	2	2	4
873		871	878	-	1656	-	-		+	2	2	4
881		880	882	-	12715	-	-		-	0	4	4
903		903	907	-	1077		1075	1077	-	2	2	4
908		905	910	+	810	-	-		-	2	2	4
913	-	-		-	999	-	-		-	4	0	4
917		912	917	+	955		953	955	+	2	2	4
918		917	922	-	1004	-	-		-	0	4	4
941		939	947	-	1126	-	-		-	2	2	4
968		968	972	-	4236	-	-		-	2	2	4
974		973	977	-	1172		1172	1175	-	2	2	4
991		987	993	-	1123		1123	1125	-	2	2	4
991		987	993	-	1186		1186	1187	-	0	4	4
1003		1001	1004	+	1029	-	-		+	2	2	4
1012		1008	1013	-	1153		1153	1154	-	2	2	4
1025		1022	1025	-	1091	-	-		+	2	2	4
1031		1026	1031	+	1050	-	-		+	2	2	4
1037		1036	1038	-	1080		1080	1081	-	2	2	4
1037		1036	1041	+	1056		1056	1059	-	2	2	4
1049		1049	1054	-	1109	-	-		+	2	2	4
1049		1049	1054	-	1328	-	-		-	2	2	4
1067		1063	1070	-	1100	-	-		-	0	4	4
1067		1063	1070	-	1128	-	-		-	2	2	4
1081		1077	1083	-	1115		1115	1116	-	2	2	4

1087	1084	1092 +	1106	1102	1106 -	2	2	4
1089	1084	1091 -	1119 -	-	-	2	2	4
1100	1096	1104 -	1112	1110	1112 +	2	2	4
1100	1096	1104 -	1207 -	-	-	2	2	4
1100	1096	1104 -	1214	1214	1215 -	2	2	4
1100	1096	1104 -	1270	1270	1271 -	2	2	4
1100	1096	1104 -	1297	1297	1300 -	1	3	4
1100	1098	1103 +	1092	1092	1093 -	2	2	4
1111	1110	1115 -	1172	1168	1172 +	2	2	4
1121	1117	1124 -	1205 -	-	-	2	2	4
1121	1117	1124 -	1284	1284	1285 -	2	2	4
1132	1128	1137 -	1254	1250	1254 -	2	2	4
1132	1128	1137 -	1283	1281	1283 +	1	3	4
1142	1137	1142 +	1275	1275	1276 -	2	2	4
1169	1167	1172 -	1300 -	-	-	2	2	4
1169	1167	1172 -	1350 -	-	-	2	2	4
1179	1176	1179 -	1329	1329	1330 -	2	2	4
1179	1176	1179 -	1342 -	-	-	2	2	4
1205	1201	1208 -	1421	1421	1425 -	2	2	4
1215	1210	1218 -	1328 -	-	-	2	2	4
1220	1220	1224 -	1328	1324	1328 -	2	2	4
1230	1227	1232 +	1240 -	-	-	2	2	4
1240	1238	1244 -	1264	1262	1264 +	2	2	4
1250	1245	1254 -	1365	1362	1365 +	2	2	4
1250	1245	1254 -	1416	1414	1416 -	2	2	4
1257	1257	1259 -	1382	1380	1382 -	2	2	4
1268	1268	1272 +	1150 -	-	-	2	2	4
1268	1268	1272 +	1166 -	-	-	2	2	4
1281	1277	1287 -	1269 -	-	+	3	1	4
1281	1277	1287 -	1363	1363	1364 -	2	2	4
1292	1292	1294 -	1362 -	-	-	2	2	4
1303	1298	1307 -	1300 -	-	+	2	2	4
1303	1298	1307 -	1351 -	-	+	2	2	4
1322	1322	1325 -	1493 -	-	-	2	2	4
1329	1327	1333 +	1349 -	-	-	2	2	4
1345	1345	1348 -	1532 -	-	-	2	2	4
1345	1343	1349 +	1262 -	-	-	2	2	4
1345	1343	1349 +	1268	1268	1269 -	2	2	4
1345	1343	1349 +	1299 -	-	-	2	2	4
1354	1351	1354 -	2188 -	-	-	2	2	4
1356	1355	1357 +	1306	1306	1307 -	2	2	4
1359	1355	1364 -	1420 -	-	+	2	2	4
1359	1355	1364 -	1451 -	-	+	2	2	4
1359	1355	1364 -	1455	1455	1456 -	2	2	4
1383	1381	1386 -	1683	1683	1684 +	2	2	4
1389	1388	1398 -	1542	1542	1543 +	2	2	4
1395	1393	1397 +	1460	1460	1461 -	2	2	4
1400	1399	1401 -	1508 -	-	+	2	2	4
1400	1399	1401 -	1591	1587	1591 -	2	2	4
1407	1404	1410 -	1438	1435	1438 +	2	2	4
1408	1404	1412 +	1341 -	-	-	0	4	4
1414 -	-	+	1335 -	-	-	4	0	4
1423	1421	1423 -	1465 -	-	-	2	2	4
1434	1431	1436 +	1467	1464	1467 +	2	2	4
1444	1440	1444 -	1495 -	-	+	4	0	4
1449	1447	1453 -	1490 -	-	+	0	4	4
1449	1447	1453 -	4423 -	-	+	1	3	4
1455 -	-	-	1675	1673	1675 -	2	2	4
1472	1472	1474 +	1427 -	-	-	2	2	4
1487	1484	1492 -	1400 -	-	+	4	0	4
1487	1484	1492 -	1564	1564	1565 -	2	2	4
1487	1484	1492 -	1664 -	-	-	2	2	4
1494	1494	1497 +	1421 -	-	-	2	2	4
1499	1497	1499 -	1676	1674	1676 -	2	2	4
1507	1507	1508 +	1401	1401	1402 -	2	2	4
1529	1529	1532 +	1448 -	-	-	2	2	4
1535	1534	1537 -	1634 -	-	+	2	2	4
1542	1542	1543 -	1630 -	-	+	2	2	4
1552	1548	1557 -	1694	1692	1694 -	2	2	4
1552	1548	1557 -	1722	1722	1726 -	2	2	4
1552	1548	1557 -	1733 -	-	-	2	2	4
1561	1560	1563 -	1650 -	-	-	2	2	4
1567	1564	1571 -	1687 -	-	-	2	2	4
1567	1564	1571 -	1878 -	-	-	2	2	4
1586	1583	1586 +	8146 -	-	+	4	0	4
1592	1588	1595 -	1653 -	-	+	2	2	4
1616	1614	1621 -	1668	1665	1668 -	2	2	4
1616	1614	1621 -	1791 -	-	-	2	2	4
1616	1614	1621 -	14107	14107	14108 +	3	1	4
1621	1620	1625 +	1722 -	-	-	2	2	4
1632	1632	1635 -	1801	1801	1802 -	2	2	4
1641	1637	1642 -	1667 -	-	-	4	0	4
1641	1637	1645 +	1672 -	-	+	3	1	4
1647	1646	1647 +	1719 -	-	-	2	2	4
1649	1649	1654 -	1814 -	-	-	2	2	4
1683	1682	1683 -	1854 -	-	-	2	2	4
1696	1696	1699 +	1671 -	-	-	2	2	4
1696	1696	1699 +	4286 -	-	+	2	2	4
1745	1745	1747 -	1839	1839	1840 -	2	2	4
1764	1759	1766 -	1937	1934	1937 -	2	2	4
1772 -	-	+	1779 -	-	-	2	2	4
1777	1777	1780 -	1835	1833	1835 +	2	2	4
1790	1790	1792 +	1737	1737	1738 -	2	2	4
1832	1830	1835 -	2004	2002	2004 -	2	2	4
1847	1844	1848 -	2000 -	-	-	2	2	4
1861	1858	1863 -	2090 -	-	-	2	2	4

1879		1879	1881	-	2083	-	-		-	2	2	4
1891		1887	1892	-	2078	-	-		-	2	2	4
1891		1887	1892	-	2088		2088	2089	-	2	2	4
1902		1900	1906	-	3314	-	-		+	2	2	4
1919		1918	1925	-	2072		2070	2072	-	2	2	4
1921		1919	1921	+	1945	-	-		+	2	2	4
1972		1968	1973	-	2039	-	-		-	2	2	4
1972		1968	1973	-	2091		2091	2092	-	2	2	4
1981		1977	1984	-	2079	-	-		-	2	2	4
1981		1977	1984	-	2122		2122	2123	-	2	2	4
2037		2035	2037	-	2130	-	-		+	2	2	4
2037		2035	2037	-	12514		12514	12515	-	1	3	4
2042		2039	2044	-	2095	-	-		-	2	2	4
2042		2039	2044	-	2100		2096	2100	+	2	2	4
2042		2040	2042	+	12521		12519	12521	+	3	1	4
2050		2045	2050	+	12529		12527	12529	+	3	1	4
2087		2087	2089	-	2206		2206	2207	-	2	2	4
2111		2111	2115	-	2285	-	-		-	2	2	4
2144	-	-		+	2121	-	-		-	2	2	4
2154		2151	2159	-	2201		2201	2204	+	2	2	4
2202		2199	2204	+	2257		2257	2258	-	2	2	4
2208		2206	2212	-	2375	-	-		-	2	2	4
2270		2270	2275	-	2420	-	-		-	2	2	4
2270		2270	2275	-	2444		2444	2447	-	2	2	4
2324		2322	2326	-	2369		2365	2369	+	2	2	4
2353		2350	2353	-	14752	-	-		-	2	2	4
2355		2355	2358	+	2317	-	-		-	2	2	4
2359		2358	2361	-	2486	-	-		-	2	2	4
2368		2365	2371	-	2542	-	-		-	2	2	4
2440		2437	2441	+	2481		2481	2482	+	2	2	4
2466		2465	2468	-	2512		2509	2512	+	2	2	4
2538		2533	2542	-	2683	-	-		-	2	2	4
2554	-	-		-	2662	-	-		-	2	2	4
2559		2556	2562	-	2628		2626	2628	+	2	2	4
2600		2600	2603	-	2813	-	-		-	2	2	4
2608		2607	2611	-	2766		2766	2767	-	2	2	4
2609		2608	2611	+	2657		2655	2657	-	2	2	4
2632		2629	2635	-	2758		2758	2759	-	2	2	4
2643		2641	2646	+	2550	-	-		-	2	2	4
2673		2670	2674	-	2793		2791	2793	+	2	2	4
2675		2671	2678	+	3021	-	-		-	2	2	4
2711		2708	2716	-	2820		2820	2823	-	2	2	4
2711		2708	2716	-	2830		2828	2830	-	2	2	4
2711		2708	2716	-	2917		2917	2918	-	2	2	4
2775		2771	2780	-	3011	-	-		-	2	2	4
2786		2784	2788	-	2947	-	-		-	2	2	4
2793		2793	2795	-	8972	-	-		-	4	0	4
2809		2806	2809	-	2832		2832	2835	+	2	2	4
2820		2819	2820	-	2985	-	-		-	2	2	4
2827		2825	2830	-	2884		2880	2884	+	2	2	4
2827		2825	2830	-	3208		3208	3209	-	2	2	4
2832		2832	2835	-	2809	-	-		+	2	2	4
2866		2864	2866	-	2877	-	-		+	4	0	4
2877		2877	2881	+	2920		2917	2920	-	2	2	4
2937		2935	2937	-	5981	-	-		-	2	2	4
2942		2941	2947	-	3057	-	-		+	2	2	4
2953		2950	2956	+	2931		2928	2931	-	2	2	4
2968		2967	2970	-	3140		3137	3140	-	2	2	4
2995		2992	2998	+	3056	-	-		-	2	2	4
3014		3010	3017	-	3038	-	-		+	4	0	4
3014		3010	3017	-	3184	-	-		-	2	2	4
3014		3010	3017	-	3214		3214	3215	-	2	2	4
3045		3042	3048	+	3094		3094	3098	-	2	2	4
3056		3053	3056	-	6495		6492	6495	-	1	3	4
3073		3070	3073	-	3216		3214	3216	-	2	2	4
3090		3086	3090	+	3568	-	-		+	2	2	4
3091		3089	3093	-	3551	-	-		+	2	2	4
3098		3097	3098	+	3065	-	-		-	2	2	4
3145	-	-		-	3339	-	-		-	2	2	4
3167		3163	3172	-	3302		3299	3302	-	2	2	4
3167		3163	3172	-	3316		3316	3319	-	2	2	4
3196		3191	3199	-	3337		3337	3340	-	2	2	4
3196		3191	3199	-	3345	-	-		-	2	2	4
3196		3191	3199	-	3351	-	-		+	2	2	4
3221		3221	3224	+	3239	-	-		+	2	2	4
3244		3241	3245	+	3281		3278	3282	+	2	2	4
3255		3255	3258	-	3388		3388	3389	-	2	2	4
3274		3270	3276	-	3420	-	-		+	2	2	4
3274		3270	3276	-	3456	-	-		-	2	2	4
3274		3270	3276	-	3636	-	-		-	2	2	4
3292		3291	3295	-	3367	-	-		+	2	2	4
3309		3308	3313	-	3453		3451	3453	-	2	2	4
3349		3348	3349	-	3913	-	-		-	2	2	4
3360		3358	3360	+	3291	-	-		+	2	2	4
3360		3358	3360	+	3309	-	-		+	2	2	4
3376		3373	3381	+	3401	-	-		+	2	2	4
3385		3383	3388	-	3568		3568	3569	-	2	2	4
3385		3383	3388	-	3579		3576	3580	-	2	2	4
3389		3387	3393	+	4404		4404	4407	+	2	2	4
3393		3389	3394	-	3585		3585	3586	-	2	2	4
3403		3398	3406	-	3577		3577	3580	-	2	2	4
3403		3398	3406	-	3611	-	-		+	2	2	4
3403		3398	3406	-	3665		3665	3666	+	1	3	4
3427	-	-		-	3510	-	-		+	2	2	4
3433		3430	3436	-	3635	-	-		+	2	2	4
3436		3433	3436	+	3500	-	-		-	2	2	4

3453	3453	3456 -	3584	3584	3585 -	2	2	4
3453	3451	3453 +	3401 -	-	+	2	2	4
3528 -	-	-	3838 -	-	-	2	2	4
3551	3550	3554 -	3737 -	-	-	2	2	4
3564	3560	3567 -	3634 -	-	+	2	2	4
3564	3560	3567 -	3700 -	-	-	2	2	4
3567	3565	3567 +	4176 -	-	+	2	2	4
3567	3565	3567 +	7670 -	-	+	2	2	4
3585	3582	3586 -	3743	3739	3743 -	2	2	4
3606	3602	3611 -	3665	3662	3665 +	2	2	4
3619 -	-	-	3742	3739	3742 -	2	2	4
3636	3636	3639 +	3597	3594	3597 +	2	2	4
3696	3696	3700 -	3870 -	-	-	2	2	4
3712	3709	3716 -	3871	3871	3872 -	2	2	4
3712	3709	3716 -	3894	3894	3895 -	2	2	4
3722	3718	3726 -	3790 -	-	+	2	2	4
3766	3766	3767 +	3780 -	-	+	2	2	4
3768 -	-	-	3791 -	-	+	2	2	4
3778	3775	3778 +	3861	3861	3862 +	2	2	4
3791	3788	3793 -	3930 -	-	-	2	2	4
3791	3788	3793 -	3944	3944	3947 -	2	2	4
3791	3788	3793 -	4242 -	-	-	2	2	4
3830	3830	3832 -	3965 -	-	-	2	2	4
3849	3847	3853 +	4000 -	-	-	2	2	4
3868	3865	3868 -	3920 -	-	+	2	2	4
3873	3873	3875 -	4090 -	-	-	2	2	4
3915	3915	3919 -	4059	4059	4060 -	2	2	4
3934	3931	3938 +	4301	4299	4301 +	2	2	4
3944	3940	3945 +	4031 -	-	-	2	2	4
3949	3948	3949 -	4116	4114	4116 -	2	2	4
3962	3960	3965 -	4093 -	-	-	2	2	4
3969	3969	3972 +	4097	4094	4097 -	2	2	4
3975	3972	3978 -	4080 -	-	+	2	2	4
4000	3998	4000 +	3847 -	-	-	2	2	4
4058	4058	4063 -	4125 -	-	+	3	1	4
4150	4146	4154 +	4165 -	-	-	3	1	4
4155	4155	4156 -	4381 -	-	-	2	2	4
4162	4158	4166 -	4239	4235	4239 -	2	2	4
4179	4175	4181 -	4225 -	-	+	2	2	4
4209	4206	4211 +	4296 -	-	-	2	2	4
4213	4209	4214 -	4292	4292	4293 +	4	0	4
4219	4217	4224 -	4352	4349	4352 -	2	2	4
4219	4217	4224 -	4357 -	-	-	2	2	4
4235	4233	4239 -	4390	4390	4394 -	2	2	4
4235	4233	4239 -	4400	4400	4402 -	2	2	4
4235	4233	4239 -	4426 -	-	-	2	2	4
4251	4251	4255 -	4354 -	-	-	2	2	4
4300	4300	4302 -	4487 -	-	-	2	2	4
4308	4308	4311 -	4491 -	-	-	2	2	4
4337	4334	4337 -	4351 -	-	+	2	2	4
4342	4342	4348 -	4487	4487	4488 -	2	2	4
4342	4342	4348 -	4496	4496	4497 +	2	2	4
4418	4414	4422 +	4372	4372	4373 -	2	2	4
4458	4458	4460 +	4403	4403	4404 -	2	2	4
4464	4464	4465 +	4375	4375	4376 -	2	2	4
4483	4481	4485 +	4513 -	-	-	2	2	4
4505	4504	4505 +	4559 -	-	-	2	2	4
4566	4563	4571 -	4854 -	-	-	2	2	4
4591 -	-	+	4804 -	-	-	2	2	4
4608	4608	4609 -	4741	4741	4742 -	2	2	4
4629	4629	4632 +	4788 -	-	-	2	2	4
4641	4636	4642 +	4770 -	-	-	2	2	4
4662	4658	4667 -	4925	4925	4926 -	2	2	4
4671	4671	4675 +	4772 -	-	-	2	2	4
4671	4671	4675 +	4926 -	-	+	2	2	4
4693	4691	4693 +	5724	5724	5725 +	2	2	4
4693	4691	4693 +	6007 -	-	+	2	2	4
4758	4755	4761 -	4774	4771	4774 +	2	2	4
4853	4849	4853 -	5024	5024	5025 +	2	2	4
4853	4849	4853 -	5885	5885	5886 -	2	2	4
4877	4875	4881 -	4938	4934	4938 +	4	0	4
4926	4923	4929 -	5179 -	-	-	2	2	4
4955	4952	4957 -	5027 -	-	-	2	2	4
4979	4976	4982 -	5013 -	-	-	2	2	4
4979	4976	4982 -	5028	5028	5029 +	2	2	4
4984	4980	4988 +	5205 -	-	-	0	4	4
4990	4985	4991 -	5123 -	-	-	2	2	4
4990	4985	4991 -	5190 -	-	-	2	2	4
4992 -	-	+	5197 -	-	-	4	0	4
5019	5019	5020 -	5071	5071	5072 +	2	2	4
5060	5059	5060 -	5090 -	-	+	2	2	4
5060	5059	5060 -	15102	15102	15104 +	4	0	4
5078	5076	5082 -	5306	5304	5306 -	3	1	4
5101	5099	5104 -	5246 -	-	-	2	2	4
5101	5099	5104 -	5290	5286	5290 -	2	2	4
5101	5099	5104 -	11815 -	-	+	2	2	4
5119	5118	5122 -	5227	5227	5228 +	2	2	4
5140	5137	5141 -	5315 -	-	-	2	2	4
5140	5137	5141 -	6689 -	-	+	4	0	4
5148	5147	5152 -	5377 -	-	-	2	2	4
5169	5166	5171 -	5259	5257	5259 -	2	2	4
5178	5176	5178 -	5653	5653	5655 -	2	2	4
5185	5185	5186 +	5230	5230	5231 -	2	2	4
5217 -	-	+	15083 -	-	-	2	2	4
5254	5251	5254 +	12854 -	-	+	2	2	4
5261	5259	5264 -	5283 -	-	+	0	4	4

5261	5259	5264	-	5535 -	-		-	2	2	4
5277	5273	5279	-	5324	5321	5324	-	2	2	4
5290	5290	5293	+	5347 -	-		+	2	2	4
5296	5296	5300	+	5353 -	-		+	2	2	4
5313	5309	5316	-	5507 -	-		-	2	2	4
5319 -	-		-	5492 -	-		-	2	2	4
5344	5342	5347	-	5499 -	-		-	2	2	4
5352 -	-		-	5379 -	-		+	2	2	4
5355	5351	5359	+	5264 -	-		-	2	2	4
5355	5351	5359	+	5330	5327	5330	-	2	2	4
5386	5385	5386	-	5407	5407	5408	+	3	1	4
5397	5397	5398	+	5344	5344	5345	-	1	3	4
5430	5429	5431	+	5450 -	-		+	2	2	4
5467	5462	5469	-	5635 -	-		-	2	2	4
5484	5482	5484	-	5635 -	-		-	2	2	4
5518 -	-		-	5548 -	-		+	0	4	4
5523	5521	5523	-	5545 -	-		+	2	2	4
5540	5540	5541	-	5682 -	-		-	2	2	4
5540	5539	5540	+	5561	5561	5562	-	1	3	4
5545	5544	5548	-	5682 -	-		-	2	2	4
5645	5644	5645	-	5714 -	-		-	2	2	4
5667	5666	5669	-	5834	5834	5835	-	2	2	4
5691	5689	5696	-	5848	5846	5848	+	2	2	4
5715 -	-		-	5843 -	-		-	2	2	4
5721	5716	5721	-	5862	5860	5862	-	2	2	4
5726	5726	5730	-	5877 -	-		-	2	2	4
5726	5726	5730	-	5971 -	-		-	2	2	4
5726	5726	5730	-	6022 -	-		-	2	2	4
5744	5740	5744	-	5802 -	-		+	2	2	4
5801	5799	5804	+	5738 -	-		-	2	2	4
5809	5805	5809	-	5933	5933	5934	+	2	2	4
5856	5853	5857	-	5972 -	-		-	2	2	4
5861	5858	5865	-	5906	5902	5906	+	2	2	4
5887	5883	5887	+	5921 -	-		-	2	2	4
5908	5904	5908	+	5874 -	-		-	1	3	4
5960 -	-		-	6097 -	-		-	2	2	4
6007 -	-		-	6294 -	-		+	2	2	4
6019	6018	6019	-	6144	6144	6147	-	2	2	4
6032	6032	6036	+	6090	6090	6091	-	2	2	4
6118 -	-		-	6144 -	-		+	3	1	4
6144	6144	6146	-	6225	6225	6226	-	2	2	4
6159	6159	6160	-	6310 -	-		-	2	2	4
6164	6164	6165	+	6166 -	-		-	2	2	4
6230	6226	6234	-	6377	6377	6381	-	2	2	4
6244	6242	6247	-	6431 -	-		-	2	2	4
6244	6242	6247	-	6441	6437	6441	-	2	2	4
6273	6273	6276	+	6373	6373	6374	-	2	2	4
6274	6270	6274	-	6389 -	-		+	2	2	4
6274	6270	6274	-	6398	6398	6399	-	2	2	4
6288	6284	6288	-	6412	6412	6413	-	2	2	4
6328	6328	6331	+	6356 -	-		-	2	2	4
6386	6385	6390	-	6494	6490	6494	+	2	2	4
6404	6400	6405	-	6414 -	-		+	2	2	4
6435	6431	6436	-	6603	6603	6607	-	2	2	4
6435	6431	6436	-	6618	6618	6621	-	2	2	4
6514	6512	6519	-	6663 -	-		-	2	2	4
6580	6577	6580	-	6746	6746	6747	-	2	2	4
6592	6592	6596	+	6671 -	-		-	2	2	4
6614	6614	6616	-	6834	6834	6835	-	2	2	4
6637 -	-		+	6699 -	-		-	2	2	4
6704	6703	6704	-	12455	12455	12456	-	2	2	4
6712	6710	6712	-	6899	6899	6900	-	2	2	4
6745	6744	6749	-	6913	6909	6913	-	2	2	4
6807	6806	6807	+	6949	6949	6950	+	2	2	4
6809	6806	6809	-	6980 -	-		-	2	2	4
6815	6811	6815	-	6851 -	-		+	2	2	4
6834	6834	6837	+	6815 -	-		-	2	2	4
6850	6848	6855	+	6786 -	-		+	2	2	4
6850	6848	6855	+	6813 -	-		-	3	1	4
6850	6848	6855	+	6938 -	-		-	2	2	4
6857	6854	6858	-	6934 -	-		+	2	2	4
6866	6865	6868	+	6921	6921	6925	-	2	2	4
6963	6962	6966	-	7131	7130	7131	-	2	2	4
6980	6975	6984	-	7006 -	-		-	2	2	4
7011	7011	7015	+	7173	7169	7173	-	2	2	4
7029	7027	7029	-	7075 -	-		+	2	2	4
7045	7041	7045	-	7283 -	-		+	2	2	4
7060	7060	7065	-	7242 -	-		-	2	2	4
7074	7070	7077	-	7195	7195	7196	+	1	3	4
7084	7080	7084	-	7263	7259	7263	-	2	2	4
7084	7080	7084	-	7273 -	-		-	2	2	4
7107	7104	7111	-	7233 -	-		-	2	2	4
7107	7104	7111	-	7317	7317	7321	-	2	2	4
7126	7123	7126	-	7254	7254	7255	+	2	2	4
7130	7126	7131	+	7221 -	-		+	2	2	4
7132	7132	7135	-	7175 -	-		+	2	2	4
7141	7137	7141	-	7364	7364	7365	-	2	2	4
7192	7192	7194	-	7364 -	-		-	2	2	4
7228 -	-		+	7380 -	-		-	2	2	4
7240	7238	7241	-	7309 -	-		-	4	0	4
7257	7254	7260	-	7543	7543	7544	-	2	2	4
7395	7391	7396	-	7790 -	-		+	2	2	4
7420	7416	7426	-	7537 -	-		-	2	2	4
7420	7416	7426	-	7585	7581	7585	-	2	2	4
7452	7447	7452	+	8190	8190	8191	+	2	2	4
7473	7473	7475	-	7627	7623	7627	-	2	2	4

7478	-	-		+	7458		7458	7459	-	2	2	4
7480		7477	7481	-	7630		7627	7630	-	2	2	4
7495		7491	7495	+	7543	-	-		-	2	2	4
7512		7507	7515	-	7591	-	-		-	2	2	4
7538		7537	7541	-	7692	-	-		-	2	2	4
7538		7537	7541	-	7716		7713	7716	-	2	2	4
7547		7545	7549	+	7708		7708	7711	-	2	2	4
7549		7545	7549	-	7628	-	-		-	2	2	4
7556		7556	7558	-	15352		15352	15355	-	0	4	4
7591		7590	7595	-	8231	-	-		+	2	2	4
7626		7626	7631	+	7804	-	-		-	2	2	4
7658		7654	7658	-	7765	-	-		+	2	2	4
7670		7670	7673	-	7862		7861	7862	-	2	2	4
7683		7679	7684	-	7793	-	-		+	2	2	4
7683		7679	7684	-	7878	-	-		-	2	2	4
7727		7727	7728	+	7884		7884	7885	-	2	2	4
7729		7727	7733	-	7796		7796	7797	-	2	2	4
7739		7736	7742	-	7865		7861	7865	-	2	2	4
7775		7775	7779	-	7893	-	-		-	2	2	4
7791		7788	7794	-	7944	-	-		-	2	2	4
7797		7796	7800	-	9224	-	-		-	2	2	4
7802		7802	7805	-	7836		7834	7836	+	2	2	4
7812		7809	7814	-	7826		7824	7826	+	3	1	4
7825		7820	7826	-	11153		11152	11157	-	1	3	4
7864		7864	7866	+	7884		7884	7885	-	2	2	4
7878		7878	7879	+	7906		7906	7907	-	2	2	4
7888		7885	7889	-	8066		8062	8066	-	2	2	4
7894		7891	7899	-	7927	-	-		+	2	2	4
7951		7951	7953	+	7927	-	-		-	1	3	4
7966		7964	7967	-	8057	-	-		-	2	2	4
7973	-	-		+	14521	-	-		+	2	2	4
7988		7984	7990	-	8047	-	-		+	2	2	4
7988		7984	7990	-	8150	-	-		+	2	2	4
7992		7991	7995	+	8103	-	-		-	2	2	4
8012		8009	8013	-	8120		8120	8121	-	2	2	4
8036	-	-		-	8081	-	-		+	2	2	4
8036	-	-		-	8206	-	-		-	2	2	4
8060		8057	8063	-	8214	-	-		-	2	2	4
8082		8082	8085	-	12082		12082	12083	-	2	2	4
8092		8089	8093	-	8238		8236	8238	-	2	2	4
8094		8092	8095	+	8149	-	-		+	2	2	4
8105		8102	8105	-	8265		8263	8265	-	2	2	4
8114		8112	8117	-	8153		8153	8154	+	2	2	4
8118		8116	8121	+	8075	-	-		-	2	2	4
8154		8150	8155	-	8286		8286	8287	-	2	2	4
8200		8199	8200	-	8371	-	-		-	2	2	4
8200		8199	8200	-	8387	-	-		-	2	2	4
8232		8231	8236	-	8402	-	-		-	2	2	4
8232		8231	8236	-	8617	-	-		-	2	2	4
8244		8238	8245	-	8402		8402	8404	-	2	2	4
8244		8238	8245	-	8420	-	-		-	2	2	4
8313		8313	8317	+	8254		8250	8254	-	2	2	4
8336	-	-		-	8358	-	-		+	4	0	4
8343		8342	8347	-	8374	-	-		+	4	0	4
8358		8358	8360	+	8216	-	-		-	3	1	4
8383		8378	8385	-	8467		8465	8467	+	2	2	4
8391		8391	8392	-	8562		8559	8562	-	3	1	4
8396		8393	8396	+	8333		8333	8334	-	2	2	4
8409		8406	8413	-	8563	-	-		-	2	2	4
8431		8428	8431	+	15363	-	-		+	4	0	4
8459		8456	8463	+	8635		8635	8636	-	2	2	4
8523		8519	8526	-	8664		8664	8665	-	2	2	4
8523		8519	8526	-	8698	-	-		-	2	2	4
8547		8543	8547	-	8715	-	-		-	2	2	4
8594	-	-		+	8578	-	-		-	2	2	4
8624		8624	8625	-	13809	-	-		-	0	4	4
8658		8655	8659	+	8726	-	-		-	2	2	4
8669		8665	8669	-	8776		8776	8780	+	2	2	4
8700		8700	8705	-	8857		8857	8861	-	2	2	4
8703		8699	8707	+	8752		8752	8753	-	2	2	4
8720	-	-		-	8779	-	-		+	2	2	4
8742		8739	8747	+	8726		8726	8727	-	2	2	4
8769		8766	8771	-	8925		8925	8926	-	2	2	4
8775		8774	8779	-	8923		8919	8923	+	2	2	4
8778		8775	8780	+	8695	-	-		-	2	2	4
8794		8791	8797	-	9021		9021	9022	-	2	2	4
8801	-	-		+	8909	-	-		-	2	2	4
8825		8823	8828	-	8985		8985	8986	-	2	2	4
8825		8823	8828	-	9011	-	-		-	2	2	4
8825		8823	8828	-	9307		9307	9308	-	2	2	4
8873		8870	8876	+	8748		8748	8749	-	2	2	4
8907		8903	8911	-	8956	-	-		+	2	2	4
8923		8921	8926	-	9124		9122	9124	-	2	2	4
8943		8943	8944	+	8867		8867	8868	-	2	2	4
8947		8943	8948	-	8864	-	-		+	2	2	4
8950		8946	8952	+	9091		9088	9091	-	2	2	4
8961		8959	8965	-	9082	-	-		-	2	2	4
8961		8959	8965	-	9125		9125	9126	-	2	2	4
8969		8968	8969	+	8920		8917	8920	-	3	1	4
8971		8966	8974	-	9068	-	-		+	2	2	4
9005		9003	9008	-	9167		9165	9167	-	2	2	4
9010		9009	9011	+	9103		9102	9103	-	3	1	4
9016	-	-		+	9096	-	-		-	2	2	4
9030		9026	9034	-	9182		9182	9184	-	2	2	4
9030		9026	9034	-	9211		9211	9212	-	2	2	4
9030		9026	9034	-	9280	-	-		-	2	2	4

9037		9036	9038	-	9211		9211	9212	-	2	2	4
9071		9069	9075	-	9216		9213	9216	-	2	2	4
9080	-	-		-	9129	-	-		+	2	2	4
9105		9103	9106	+	9007		9006	9007	-	3	1	4
9140		9137	9140	+	9154	-	-		-	2	2	4
9185	-	-		+	9070	-	-		-	2	2	4
9190		9188	9190	-	9216		9214	9216	+	2	2	4
9197		9193	9202	-	9350	-	-		-	2	2	4
9197		9193	9202	-	9384		9384	9386	-	2	2	4
9197		9193	9202	-	9392	-	-		-	2	2	4
9209		9206	9210	-	9324		9324	9325	-	2	2	4
9233		9229	9237	+	9420		9420	9421	-	2	2	4
9241	-	-		-	11500	-	-		-	0	4	4
9256	-	-		-	9482	-	-		-	2	2	4
9289		9287	9292	-	9450		9450	9451	-	2	2	4
9289		9287	9292	-	9461	-	-		-	2	2	4
9289		9287	9292	-	9503	-	-		-	2	2	4
9305		9300	9309	-	9478		9478	9481	-	2	2	4
9305		9300	9309	-	9515	-	-		-	2	2	4
9311		9310	9315	-	9506		9502	9506	-	2	2	4
9323		9319	9324	-	9504		9501	9504	-	2	2	4
9336		9335	9339	-	9372		9372	9373	+	3	1	4
9336		9335	9339	-	9501		9501	9503	-	2	2	4
9343		9342	9345	+	9361		9361	9364	+	2	2	4
9351		9345	9353	-	9501	-	-		-	2	2	4
9362		9362	9365	-	10105	-	-		-	2	2	4
9375		9372	9379	-	9574		9571	9574	-	2	2	4
9383		9383	9385	-	9566	-	-		-	2	2	4
9417		9414	9417	-	9579	-	-		+	2	2	4
9424		9424	9425	-	9630	-	-		-	4	0	4
9430	-	-		-	9636	-	-		-	0	4	4
9451		9447	9454	-	9511		9507	9512	+	2	2	4
9451		9447	9454	-	9563		9563	9564	-	2	2	4
9451		9447	9454	-	9608	-	-		-	2	2	4
9473		9473	9474	-	9700	-	-		-	2	2	4
9490		9487	9490	-	9589	-	-		+	1	3	4
9498		9498	9499	-	9713	-	-		-	2	2	4
9530	-	-		+	9738	-	-		-	2	2	4
9541		9536	9541	-	9725	-	-		-	2	2	4
9541		9536	9541	-	9730		9730	9731	+	2	2	4
9543		9539	9544	+	9886	-	-		-	2	2	4
9548		9544	9551	-	9728	-	-		-	2	2	4
9574		9572	9574	+	9502		9502	9503	-	2	2	4
9584		9584	9588	+	9695		9695	9696	-	2	2	4
9594		9591	9599	-	9687	-	-		+	2	2	4
9594		9591	9599	-	9764	-	-		-	2	2	4
9598		9597	9602	+	9550	-	-		-	4	0	4
9604		9603	9606	+	9627		9627	9629	+	2	2	4
9619		9619	9620	-	9725	-	-		+	2	2	4
9623	-	-		+	9536		9536	9537	-	2	2	4
9671		9668	9674	+	9605	-	-		-	2	2	4
9697		9695	9699	-	9775	-	-		+	2	2	4
9697		9695	9699	-	9841	-	-		-	2	2	4
9697		9697	9699	+	9680		9680	9681	-	2	2	4
9704		9703	9705	+	9672		9672	9673	-	2	2	4
9717		9716	9719	-	9740		9740	9742	+	3	1	4
9717		9716	9719	-	9947	-	-		-	2	2	4
9723		9722	9725	-	9924	-	-		-	2	2	4
9760		9758	9760	-	10027	-	-		-	2	2	4
9763		9763	9766	+	9707		9707	9708	-	2	2	4
9767		9767	9768	-	9939	-	-		-	2	2	4
9773		9773	9775	-	9936		9936	9937	-	2	2	4
9786		9785	9786	-	9949	-	-		-	2	2	4
9792		9792	9795	-	9947		9947	9948	-	2	2	4
9797		9796	9800	+	9822		9822	9823	+	2	2	4
9817		9817	9819	-	9957	-	-		-	2	2	4
9830		9830	9834	-	9847		9845	9847	+	3	1	4
9839		9839	9840	-	9922	-	-		-	2	2	4
9844		9843	9847	-	10005	-	-		+	2	2	4
9886		9886	9889	+	9917	-	-		-	2	2	4
9905		9903	9906	-	10089	-	-		-	2	2	4
9934		9934	9935	-	10118		10114	10118	-	2	2	4
9962		9962	9965	-	10067		10063	10068	-	2	2	4
9973		9973	9977	+	9987		9987	9988	-	2	2	4
9979		9975	9981	-	9983	-	-		+	2	2	4
9979		9975	9981	-	10123	-	-		+	2	2	4
10000		10000	10004	+	10210	-	-		+	2	2	4
10031		10028	10031	-	10197	-	-		+	2	2	4
10036		10036	10039	-	10278		10275	10278	-	2	2	4
10043		10040	10048	+	9924	-	-		-	2	2	4
10049		10045	10051	-	10220		10220	10221	-	2	2	4
10061		10060	10065	-	10213		10213	10218	-	2	2	4
10066		10064	10066	+	10120	-	-		-	2	2	4
10104		10102	10106	-	10214		10211	10214	-	2	2	4
10104		10102	10106	-	10330		10328	10330	-	2	2	4
10120		10117	10121	-	10215	-	-		-	2	2	4
10120		10117	10121	-	10335		10335	10336	-	2	2	4
10120		10116	10121	+	10069		10067	10070	-	2	2	4
10181		10181	10185	-	10330	-	-		-	2	2	4
10195		10194	10198	-	10361	-	-		-	2	2	4
10206		10202	10208	-	10331		10329	10331	-	2	2	4
10219		10216	10221	-	10336	-	-		-	2	2	4
10224		10220	10224	+	10300	-	-		-	2	2	4
10242		10242	10243	-	10309	-	-		-	2	2	4
10251		10251	10255	-	10324	-	-		-	2	2	4
10251		10251	10255	-	10429		10427	10429	-	2	2	4

10266	10264	10270	-	10333	10333	10334	+	2	2	4
10273	10272	10277	-	10417	10414	10417	-	2	2	4
10273	10272	10277	-	10427	10427	10428	-	2	2	4
10287	10283	10292	-	10496	10493	10496	-	2	2	4
10334	-	-	+	10268	-	-	-	2	2	4
10343	10342	10344	-	10529	10527	10529	-	2	2	4
10369	-	-	+	10269	-	-	-	2	2	4
10370	10369	10374	-	10529	10525	10529	-	2	2	4
10379	10376	10382	-	10466	-	-	+	3	1	4
10448	10448	10453	-	10504	10500	10504	+	2	2	4
10467	-	-	+	10364	-	-	-	2	2	4
10472	10469	10477	-	10666	10664	10666	-	2	2	4
10485	10483	10487	-	10615	-	-	-	2	2	4
10498	10495	10498	-	10621	10621	10622	-	2	2	4
10522	10522	10523	+	10489	10489	10490	-	2	2	4
10549	10547	10553	-	10736	-	-	-	2	2	4
10558	10556	10558	+	10814	10814	10815	-	2	2	4
10575	10571	10575	-	10740	-	-	-	2	2	4
10582	10578	10585	+	10790	-	-	-	2	2	4
10582	10578	10585	+	10898	-	-	-	2	2	4
10684	10681	10684	-	10869	10866	10869	+	2	2	4
10721	10717	10724	+	14850	14848	14850	-	3	1	4
10797	10796	10798	-	10906	-	-	-	2	2	4
10803	10800	10803	+	12680	-	-	-	2	2	4
10955	10951	10958	+	11035	11031	11035	-	2	2	4
10990	10986	10994	-	11026	-	-	+	2	2	4
10990	10986	10994	-	11150	-	-	-	2	2	4
11005	11005	11008	-	11238	-	-	-	2	2	4
11005	11005	11008	-	13619	-	-	+	2	2	4
11005	-	-	+	11061	-	-	-	2	2	4
11032	11030	11035	-	11248	-	-	-	2	2	4
11136	11134	11139	-	11297	11295	11297	-	2	2	4
11161	-	-	-	11273	-	-	-	2	2	4
11174	11172	11174	-	11315	-	-	+	2	2	4
11181	-	-	-	11383	-	-	+	2	2	4
11190	-	-	+	11208	-	-	-	2	2	4
11199	11195	11204	-	11334	-	-	+	2	2	4
11203	11202	11203	+	11155	-	-	-	2	2	4
11209	11209	11211	-	11385	-	-	-	2	2	4
11209	11209	11211	-	11447	11447	11448	-	2	2	4
11220	-	-	+	11164	-	-	-	2	2	4
11221	11221	11226	-	11402	-	-	-	2	2	4
11235	11231	11238	-	11381	-	-	-	2	2	4
11235	11231	11238	-	11386	-	-	-	2	2	4
11235	11231	11238	-	11409	-	-	-	2	2	4
11255	11253	11256	-	11387	11387	11388	-	2	2	4
11269	11265	11273	-	11320	11318	11322	+	2	2	4
11282	11278	11287	-	11310	11306	11310	+	2	2	4
11282	11278	11287	-	14929	-	-	+	2	2	4
11290	-	-	+	11374	-	-	-	2	2	4
11295	11292	11295	-	11452	-	-	-	2	2	4
11318	11314	11321	-	11386	-	-	+	4	0	4
11318	11314	11321	-	11440	11440	11441	-	2	2	4
11323	11323	11327	-	11381	-	-	+	0	4	4
11330	-	-	+	11343	-	-	-	2	2	4
11351	11351	11354	-	11360	-	-	+	2	2	4
11365	11361	11369	-	11544	11542	11544	-	2	2	4
11398	11397	11402	+	11408	-	-	-	2	2	4
11398	11397	11402	+	11483	11483	11484	-	2	2	4
11424	11420	11428	-	11564	-	-	-	2	2	4
11424	11420	11428	-	11613	11610	11613	-	2	2	4
11439	11436	11439	-	11651	-	-	-	2	2	4
11439	11439	11442	+	11370	11367	11370	-	2	2	4
11439	11439	11442	+	11389	-	-	-	2	2	4
11452	11451	11456	-	11499	-	-	+	2	2	4
11462	11462	11466	+	11603	11599	11603	-	2	2	4
11486	-	-	-	11501	-	-	+	2	2	4
11500	11495	11504	-	11652	11652	11653	-	2	2	4
11500	11495	11504	-	11666	11666	11670	-	2	2	4
11500	11495	11504	-	11705	11702	11705	-	2	2	4
11522	11518	11526	-	11547	11547	11548	+	3	1	4
11522	11518	11526	-	11716	-	-	-	2	2	4
11529	11528	11531	-	11706	11704	11706	-	2	2	4
11549	11547	11552	-	11677	11677	11678	-	2	2	4
11561	11558	11564	-	11723	-	-	+	2	2	4
11569	11566	11571	-	11718	11716	11718	-	2	2	4
11619	-	-	+	11732	-	-	-	2	2	4
11676	11676	11678	-	15309	-	-	-	2	2	4
11697	11695	11697	+	11661	-	-	-	2	2	4
11731	11731	11734	+	11723	11720	11723	-	2	2	4
11749	11749	11751	-	11903	11903	11906	-	2	2	4
11755	11752	11757	+	11721	-	-	-	2	2	4
11761	11760	11763	-	11928	-	-	-	2	2	4
11768	11767	11769	+	11706	-	-	-	2	2	4
11781	11781	11789	-	11875	-	-	+	3	1	4
11840	11837	11842	-	11955	-	-	-	2	2	4
11845	11844	11848	-	11920	-	-	-	2	2	4
11856	11852	11856	-	12045	-	-	-	2	2	4
11861	11860	11861	-	12074	12072	12074	-	2	2	4
11881	11878	11884	-	12042	12039	12042	-	2	2	4
11881	11878	11884	-	12079	-	-	-	2	2	4
11882	-	-	+	11775	-	-	-	2	2	4
11899	11897	11902	-	12061	-	-	-	2	2	4
11948	11948	11950	-	12054	12054	12055	+	2	2	4
11953	11951	11956	-	12159	-	-	-	2	2	4
11972	11971	11976	+	12084	12084	12085	-	2	2	4

11988	11987	11992	+	11945	11945	11946	-	2	2	4
11988	11987	11992	+	12081	12080	12081	-	3	1	4
11993	11990	11995	-	12075	-	-	+	2	2	4
12010	12007	12014	-	12170	-	-	-	2	2	4
12032	12031	12032	-	12154	-	-	+	2	2	4
12041	12040	12044	-	12236	-	-	-	2	2	4
12058	12056	12063	-	12262	-	-	-	2	2	4
12058	12056	12063	-	12394	-	-	+	2	2	4
12076	12075	12076	-	12137	-	-	+	2	2	4
12082	12079	12086	-	12195	-	-	+	2	2	4
12082	12080	12087	+	11968	-	-	-	2	2	4
12091	12090	12095	+	12185	12185	12186	-	2	2	4
12092	12087	12098	-	12123	12119	12124	+	4	0	4
12092	12087	12098	-	12229	12229	12232	-	2	2	4
12105	-	-	-	12154	-	-	+	2	2	4
12123	12119	12127	-	12194	-	-	-	2	2	4
12136	12132	12142	-	12160	12157	12160	+	2	2	4
12136	12132	12142	-	12300	12300	12301	-	2	2	4
12168	12165	12169	+	12217	12217	12218	-	2	2	4
12175	-	-	-	12298	-	-	-	2	2	4
12185	12182	12185	-	12339	-	-	-	2	2	4
12196	12195	12199	-	12369	-	-	-	2	2	4
12203	-	-	+	12188	-	-	-	2	2	4
12228	12224	12231	-	12298	12295	12298	+	2	2	4
12238	12234	12241	-	12318	12315	12318	+	2	2	4
12238	12234	12241	-	12369	12369	12370	-	2	2	4
12288	12286	12291	-	12371	-	-	-	2	2	4
12301	12298	12301	+	12355	-	-	-	2	2	4
12308	-	-	+	12345	-	-	-	2	2	4
12320	12316	12322	-	12567	12567	12568	-	2	2	4
12322	12319	12326	+	12526	-	-	-	2	2	4
12343	12340	12346	-	12551	12548	12551	-	2	2	4
12351	12348	12351	-	12489	12487	12489	-	2	2	4
12376	12373	12377	-	12396	12392	12396	+	2	2	4
12393	12392	12397	+	12278	-	-	-	2	2	4
12397	12397	12400	-	12566	-	-	-	2	2	4
12414	12412	12418	-	12585	-	-	-	2	2	4
12500	12500	12502	-	12627	-	-	+	2	2	4
12513	12510	12513	+	12396	-	-	-	2	2	4
12515	12512	12519	-	12695	-	-	-	2	2	4
12536	12533	12537	-	12661	12661	12662	-	2	2	4
12542	12542	12546	+	12603	12603	12605	-	2	2	4
12570	-	-	-	12755	-	-	-	2	2	4
12585	12584	12589	-	12620	-	-	-	4	0	4
12585	12584	12589	-	12625	-	-	-	0	4	4
12603	12603	12607	+	12537	-	-	-	1	3	4
12603	12603	12607	+	14517	-	-	-	2	2	4
12608	12606	12611	-	12746	-	-	-	2	2	4
12608	12606	12611	-	12752	12752	12755	-	2	2	4
12608	12606	12611	-	12772	12772	12774	-	2	2	4
12618	12614	12619	+	12774	-	-	+	2	2	4
12620	12616	12620	-	12782	12779	12782	-	2	2	4
12626	12622	12630	-	12773	-	-	-	2	2	4
12641	12636	12645	-	12771	-	-	-	2	2	4
12682	12679	12682	-	12814	12811	12814	-	2	2	4
12698	-	-	-	12804	-	-	-	2	2	4
12706	12703	12711	+	12605	12605	12606	-	2	2	4
12706	12703	12711	+	12749	-	-	-	2	2	4
12715	-	-	+	12744	-	-	-	2	2	4
12725	12723	12725	+	12732	12732	12733	-	2	2	4
12730	12730	12733	-	12935	12932	12935	-	2	2	4
12739	-	-	+	12669	12669	12670	-	2	2	4
12747	12745	12751	-	12920	12918	12920	-	2	2	4
12747	12745	12751	-	12956	12956	12959	-	2	2	4
12754	12752	12759	-	12925	12925	12927	-	2	2	4
12754	12752	12759	-	12930	12930	12932	-	1	3	4
12754	12752	12759	-	12989	-	-	-	2	2	4
12767	12765	12771	-	12868	-	-	-	2	2	4
12767	12765	12771	-	12932	12932	12936	-	2	2	4
12776	12776	12782	+	13757	13757	13759	+	2	2	4
12784	12784	12787	+	12819	12819	12820	-	2	2	4
12784	12784	12787	+	13015	-	-	+	2	2	4
12810	12810	12812	-	12842	-	-	+	3	1	4
12856	12854	12856	+	12822	-	-	-	0	4	4
12872	12868	12875	-	13054	13054	13057	-	2	2	4
12891	12890	12894	-	13053	13050	13053	-	2	2	4
12891	12890	12894	-	13121	-	-	-	2	2	4
12905	12904	12905	+	12850	12850	12851	-	2	2	4
12907	12903	12910	-	13078	-	-	-	2	2	4
12907	12903	12910	-	13128	-	-	-	2	2	4
12915	-	-	-	13051	-	-	-	2	2	4
12928	12925	12931	-	13500	-	-	-	2	2	4
12945	-	-	-	13584	-	-	-	2	2	4
12958	12958	12962	-	13103	-	-	+	2	2	4
13001	12997	13001	-	12974	-	-	+	4	0	4
13011	13010	13016	+	13032	13032	13033	-	2	2	4
13019	13018	13022	+	13064	-	-	-	2	2	4
13024	13021	13027	-	13204	-	-	-	2	2	4
13033	-	-	+	13240	-	-	-	2	2	4
13039	13037	13041	-	13196	13196	13198	-	2	2	4
13084	13082	13088	-	13215	13213	13215	-	2	2	4
13092	13092	13096	+	13082	13078	13082	-	2	2	4
13104	13101	13106	-	13056	-	-	+	4	0	4
13104	13101	13106	-	13288	-	-	-	2	2	4
13113	13109	13117	-	13172	-	-	+	2	2	4
13168	13168	13170	-	13212	13210	13212	+	2	2	4

13168	13168	13170	-	13273	13269	13273	-	2	2	4
13184	13181	13187	-	13303	13300	13303	-	2	2	4
13184	13181	13187	-	13342	13342	13343	-	2	2	4
13215	13213	13219	-	13337 -	-		-	2	2	4
13229	13226	13230	-	13281 -	-		-	2	2	4
13261	13257	13265	-	13303 -	-		+	4	0	4
13261	13260	13261	+	13360 -	-		-	2	2	4
13284	13280	13287	-	13462	13462	13463	-	2	2	4
13284	13280	13287	-	13486	13482	13486	-	2	2	4
13300	13300	13304	-	13594 -	-		-	2	2	4
13303	13303	13307	+	13320 -	-		-	2	2	4
13317	13314	13320	-	13509	13506	13509	-	2	2	4
13317	13314	13320	-	13588	13588	13589	-	2	2	4
13338	13335	13341	-	13446 -	-		+	2	2	4
13355	13353	13355	-	13478 -	-		-	2	2	4
13409	13406	13409	-	13519	13516	13519	-	2	2	4
13427	13423	13428	-	13780	13776	13780	-	2	2	4
13446	13442	13449	-	13627	13625	13627	-	2	2	4
13497	13497	13500	-	14019 -	-		+	2	2	4
13501	13501	13505	+	13531 -	-		-	2	2	4
13508	13508	13511	-	14022 -	-		-	2	2	4
13539	13538	13542	+	13393	13393	13394	-	2	2	4
13540	13535	13543	-	13778	13778	13782	-	1	3	4
13552	13552	13553	-	13655	13655	13656	-	2	2	4
13561	13561	13562	+	13600	13600	13601	-	2	2	4
13572	13568	13573	-	13783	13782	13783	-	2	2	4
13614	13614	13618	-	13665 -	-		-	2	2	4
13673	13673	13674	-	13772 -	-		-	2	2	4
13684	13683	13686	-	14365 -	-		-	2	2	4
13687 -	-		+	13730 -	-		-	2	2	4
13693 -	-		+	13963 -	-		+	2	2	4
13699 -	-		-	13737 -	-		+	2	2	4
13707	13705	13707	+	13662 -	-		+	2	2	4
13730	13730	13731	+	13686	13686	13687	-	2	2	4
13743	13739	13745	-	13866 -	-		-	2	2	4
13763	13758	13764	-	13975 -	-		-	2	2	4
13771	13771	13772	+	13969	13969	13970	-	2	2	4
13772	13768	13776	-	13969 -	-		+	2	2	4
13785	13781	13785	-	14085	14081	14085	-	2	2	4
13786	13782	13788	+	13760 -	-		+	2	2	4
13786	13782	13788	+	13957 -	-		-	2	2	4
13796	13794	13802	-	14085	14085	14086	-	2	2	4
13807	13803	13807	+	13839	13839	13840	-	2	2	4
13839	13836	13840	-	13990 -	-		-	2	2	4
13849	13848	13849	+	13800 -	-		-	2	2	4
13879	13875	13880	+	13993	13993	13995	-	2	2	4
13882	13880	13886	-	13969	13967	13969	+	2	2	4
13882	13880	13886	-	14053 -	-		-	2	2	4
13923	13920	13928	-	14000 -	-		-	2	2	4
13923	13920	13928	-	14031 -	-		-	2	2	4
13923	13920	13928	-	14084 -	-		-	2	2	4
13923	13920	13928	-	14113 -	-		-	2	2	4
13938	13935	13942	-	14042 -	-		-	2	2	4
13948	13944	13951	-	14086	14086	14089	-	2	2	4
13948	13948	13949	+	14052 -	-		+	2	2	4
13961	13956	13965	-	14085	14085	14086	+	2	2	4
13968	13967	13971	-	14083	14083	14087	-	2	2	4
13973	13972	13974	-	14191 -	-		+	2	2	4
13974	13973	13976	+	14191 -	-		-	2	2	4
13978	13975	13982	-	14206 -	-		-	2	2	4
13983	13980	13983	+	14094 -	-		-	2	2	4
13992	13987	13993	-	14055	14053	14055	+	2	2	4
14008 -	-		+	14106 -	-		+	2	2	4
14016	14014	14020	-	14168	14168	14169	-	2	2	4
14041	14038	14041	-	14200 -	-		-	2	2	4
14048	14044	14051	-	14204	14204	14205	-	2	2	4
14063	14063	14065	+	14249 -	-		-	2	2	4
14074	14073	14078	-	14326 -	-		+	2	2	4
14107	14107	14108	+	14132	14131	14132	-	1	3	4
14113	14109	14114	+	14129	14129	14132	-	3	1	4
14113	14109	14114	+	14249 -	-		+	2	2	4
14121	14121	14122	+	14065	14065	14066	-	2	2	4
14157	14155	14162	-	14200 -	-		-	2	2	4
14157	14155	14162	-	14391 -	-		-	2	2	4
14165	14165	14169	+	14078 -	-		-	2	2	4
14171	14169	14174	-	14306 -	-		-	2	2	4
14178	14175	14182	-	14210 -	-		+	0	4	4
14179	14179	14180	+	14111	14111	14112	-	2	2	4
14201	14198	14204	-	14262 -	-		+	2	2	4
14210	14208	14210	+	14087 -	-		-	2	2	4
14256	14254	14256	-	14346 -	-		+	2	2	4
14283 -	-		+	14214 -	-		+	2	2	4
14301	14300	14305	-	14483 -	-		-	2	2	4
14301	14300	14305	-	14497	14497	14498	-	2	2	4
14303 -	-		+	14384 -	-		+	2	2	4
14316	14314	14316	-	14317 -	-		+	2	2	4
14316	14314	14316	-	14378 -	-		+	2	2	4
14324	14321	14326	-	14443 -	-		-	2	2	4
14324	14321	14326	-	14509	14509	14511	-	3	1	4
14333	14331	14337	-	14884 -	-		+	2	2	4
14344	14339	14346	-	14411 -	-		+	2	2	4
14344	14339	14346	-	14475	14475	14478	-	2	2	4
14359	14358	14364	-	14397	14397	14398	+	2	2	4
14369	14368	14371	-	14573 -	-		+	4	0	4
14369	14368	14371	-	14587 -	-		-	2	2	4
14374	14373	14380	-	14567 -	-		+	0	4	4

14384	14382	14385	-	14789	-	-	+	2	2	4
14404	14404	14408	+	14478	14475	14478	-	2	2	4
14409	14406	14410	-	14422	-	-	+	2	2	4
14409	14406	14410	-	14585	-	-	-	2	2	4
14448	14447	14450	-	14542	14540	14542	+	2	2	4
14478	14473	14481	-	14552	14549	14552	+	2	2	4
14478	14473	14481	-	14655	14655	14659	+	2	2	4
14491	14491	14493	+	14499	14499	14500	-	2	2	4
14503	-	-	-	14552	-	-	+	2	2	4
14507	14503	14509	+	14540	-	-	+	2	2	4
14540	14536	14544	-	14660	14660	14665	-	2	2	4
14540	14539	14542	+	14465	-	-	-	2	2	4
14554	14550	14558	-	14749	14749	14750	-	2	2	4
14554	14550	14558	-	14772	-	-	-	2	2	4
14577	14574	14583	-	14769	14769	14770	-	2	2	4
14600	14598	14602	-	14624	14621	14624	+	3	1	4
14600	14598	14602	-	14769	14769	14773	-	2	2	4
14600	14600	14601	+	14618	14618	14619	-	2	2	4
14614	14612	14615	-	14685	-	-	+	2	2	4
14624	14624	14626	-	14674	-	-	+	1	3	4
14642	14639	14643	-	14768	-	-	-	2	2	4
14642	14639	14643	-	14827	-	-	-	2	2	4
14667	14663	14670	-	14759	14755	14759	-	2	2	4
14675	14674	14679	-	14718	-	-	+	2	2	4
14687	14684	14690	-	14777	-	-	+	2	2	4
14687	14684	14690	-	14902	-	-	-	2	2	4
14687	14686	14687	+	14640	-	-	-	2	2	4
14714	14712	14714	-	14743	-	-	+	4	0	4
14719	14716	14723	-	14902	14898	14902	-	2	2	4
14728	14726	14728	-	14872	-	-	+	2	2	4
14744	14741	14750	-	14928	14928	14929	-	2	2	4
14752	14752	14755	-	14871	14868	14872	+	2	2	4
14767	-	-	+	14902	-	-	-	2	2	4
14771	14767	14771	-	14938	14938	14939	-	2	2	4
14791	14788	14792	-	14928	-	-	-	2	2	4
14796	14795	14799	-	14837	-	-	+	2	2	4
14806	14802	14807	-	14886	14883	14886	+	2	2	4
14818	14818	14820	-	15226	-	-	-	2	2	4
14818	-	-	+	14864	-	-	-	2	2	4
14824	14824	14827	-	14858	14856	14858	+	1	3	4
14847	14843	14851	-	14994	-	-	+	2	2	4
14847	14843	14851	-	15014	-	-	+	2	2	4
14866	14866	14872	-	14998	-	-	-	2	2	4
14866	14866	14872	-	15068	15068	15069	-	2	2	4
14866	14866	14872	-	15083	-	-	-	2	2	4
14875	14874	14875	-	15069	-	-	-	2	2	4
14897	14892	14900	-	15085	15084	15086	-	2	2	4
14902	14898	14902	+	14860	14860	14861	-	2	2	4
14913	14910	14917	-	14943	14943	14947	+	2	2	4
14925	14922	14928	-	14960	-	-	+	2	2	4
14938	14938	14939	-	14963	-	-	+	2	2	4
14944	14941	14947	-	15085	15085	15086	-	2	2	4
14944	14941	14947	-	15158	-	-	-	2	2	4
14972	14969	14975	-	15054	15054	15056	+	3	1	4
14988	14984	14988	+	14879	14879	14880	-	2	2	4
14994	14990	14997	-	15057	-	-	+	2	2	4
14998	14996	15000	+	15050	15050	15051	-	2	2	4
14998	14996	15000	+	15076	-	-	-	2	2	4
15012	15008	15015	+	15041	15038	15041	-	2	2	4
15014	15010	15018	-	15066	-	-	-	2	2	4
15014	15010	15018	-	15181	15181	15183	-	2	2	4
15027	15025	15032	-	15170	15167	15170	-	2	2	4
15032	15030	15035	+	15125	15125	15126	-	2	2	4
15043	15043	15045	-	15117	15117	15118	+	2	2	4
15045	15043	15048	+	15113	15113	15114	-	2	2	4
15045	15043	15048	+	15384	-	-	-	2	2	4
15052	15049	15055	-	15108	-	-	+	2	2	4
15052	15049	15055	-	15188	-	-	-	2	2	4
15082	15080	15084	-	15144	-	-	+	2	2	4
15093	15093	15095	-	15290	15287	15290	+	3	1	4
15112	15110	15115	-	15269	15265	15269	-	2	2	4
15112	15110	15115	-	15329	-	-	-	2	2	4
15112	15110	15115	-	15341	-	-	-	2	2	4
15118	15117	15118	+	14969	-	-	-	2	2	4
15130	15129	15133	+	15257	15257	15258	-	2	2	4
15138	15138	15141	+	15025	-	-	-	2	2	4
15174	15172	15176	-	15251	15247	15251	-	2	2	4
15190	15189	15193	-	15255	15255	15256	-	2	2	4
15204	15204	15206	-	15378	15374	15378	-	2	2	4
15204	15204	15206	-	15383	15383	15384	-	2	2	4
15204	15203	15207	+	15235	-	-	-	2	2	4
15225	-	-	-	15375	-	-	-	2	2	4
15236	15233	15236	-	15380	-	-	-	2	2	4
15240	15240	15244	+	15276	-	-	-	2	2	4
15323	15322	15325	+	15241	15239	15242	-	2	2	4
14	9	17	-	114	112	114	+	2	1	3
19	18	25	-	120	120	123	-	1	2	3
29	28	32	-	250	250	251	-	2	1	3
41	40	44	-	262	-	-	+	2	1	3
58	57	62	-	217	216	217	-	2	1	3
173	-	-	+	93	-	-	+	2	1	3
224	222	225	-	387	385	387	-	2	1	3
283	279	286	+	355	352	356	-	2	1	3
295	291	297	+	352	-	-	-	2	1	3
323	321	327	+	370	368	370	-	3	0	3
369	366	369	-	486	486	489	-	1	2	3

380	378	383 -	555	555	559 -	2	1	3
438	433	438 -	446	442	446 +	2	1	3
491 -	-	-	696 -	-	-	1	2	3
509	505	509 +	940	940	941 +	1	2	3
513	509	514 -	667	667	668 -	2	1	3
513	509	514 -	722	721	722 -	2	1	3
606	602	609 -	691	691	694 -	2	1	3
607	605	609 +	15158 -	-	+	3	0	3
659	659	662 -	716 -	-	+	3	0	3
665	663	670 -	711 -	-	+	0	3	3
672	669	672 +	815 -	-	-	0	3	3
677	676	681 +	810 -	-	-	3	0	3
710	708	712 +	748	744	748 -	3	0	3
717	714	720 -	748	748	750 +	3	0	3
794	793	796 -	9101	9100	9101 -	3	0	3
838	835	838 +	9136	9133	9136 +	2	1	3
844	840	845 -	956 -	-	-	1	2	3
928	928	930 -	959 -	-	+	3	0	3
968	968	972 -	1117 -	-	-	3	0	3
991	987	993 -	1129	1129	1133 -	1	2	3
1061	1058	1061 -	1098 -	-	-	3	0	3
1081	1077	1083 -	1252	1251	1252 -	1	2	3
1123	1122	1125 +	1103 -	-	-	1	2	3
1158	1155	1160 -	1132 -	-	+	3	0	3
1192	1191	1192 -	1227	1227	1228 +	1	2	3
1240	1238	1244 -	1536 -	-	-	0	3	3
1250	1245	1254 -	1385 -	-	-	1	2	3
1250	1245	1254 -	5958	5958	5961 +	1	2	3
1257	1257	1259 -	1340 -	-	+	3	0	3
1267	1265	1270 -	1334 -	-	+	0	3	3
1313	1309	1313 -	1340	1340	1343 +	2	1	3
1375	1371	1379 -	1557 -	-	+	1	2	3
1460	1456	1462 -	1488 -	-	-	1	2	3
1472	1472	1474 +	1383 -	-	-	2	1	3
1481	1477	1482 -	1660 -	-	-	2	1	3
1552	1548	1557 -	8116 -	-	-	3	0	3
1561	1560	1563 -	1725 -	-	-	2	1	3
1575 -	-	-	1705 -	-	-	2	1	3
1671	1668	1674 -	1753 -	-	+	3	0	3
1676 -	-	-	1748 -	-	+	0	3	3
1832	1830	1835 -	2016 -	-	-	2	1	3
1852	1849	1856 -	2013	2011	2013 -	2	1	3
1861	1858	1863 -	2014	2013	2014 -	1	2	3
1861 -	-	+	1766 -	-	-	2	1	3
1887	1883	1887 +	3329 -	-	-	0	3	3
1891	1887	1892 -	3329 -	-	+	2	1	3
1911 -	-	+	1973 -	-	-	1	2	3
1949	1946	1949 -	2084	2084	2088 -	1	2	3
2012	2010	2015 -	12493	12489	12493 -	2	1	3
2023	2023	2027 -	2055 -	-	+	3	0	3
2050	2045	2050 +	12524 -	-	+	2	1	3
2055 -	-	-	2023 -	-	+	3	0	3
2077	2076	2081 +	2182	2181	2182 +	1	2	3
2406	2405	2407 -	5476 -	-	+	3	0	3
2455	2454	2458 -	2483	2480	2483 +	2	1	3
2478	2476	2482 -	2699	2697	2700 -	1	2	3
2492	2489	2495 -	2551	2551	2552 +	1	2	3
2492	2489	2495 -	12082 -	-	-	3	0	3
2503	2498	2505 -	2525	2522	2525 +	3	0	3
2518	2515	2522 -	2727 -	-	-	1	2	3
2608	2607	2611 -	6644 -	-	+	2	1	3
2786	2784	2788 -	4008 -	-	-	1	2	3
2827	2825	2830 -	2948	2948	2950 -	2	1	3
2832	2832	2835 -	3015	3011	3016 -	3	0	3
2879	2878	2882 -	3010	3006	3010 -	2	1	3
2902	2900	2902 -	3105 -	-	-	2	1	3
2942	2941	2947 -	2962 -	-	+	3	0	3
2962	2961	2964 -	2941 -	-	+	3	0	3
3060 -	-	+	3072 -	-	-	3	0	3
3229	3229	3233 +	3217 -	-	+	0	3	3
3229	3229	3233 +	4486	4486	4487 +	2	1	3
3249	3247	3252 -	3463	3461	3463 -	1	2	3
3300	3300	3302 -	3405	3405	3408 +	2	1	3
3376	3373	3381 +	3418	3418	3419 -	1	2	3
3403	3398	3406 -	13614 -	-	-	3	0	3
3436	3433	3436 +	3453 -	-	-	1	2	3
3441	3438	3442 -	3447	3447	3449 +	2	1	3
3483	3479	3486 -	3656	3653	3656 +	1	2	3
3538 -	-	+	3521 -	-	-	1	2	3
3545	3542	3549 -	3574	3570	3574 +	2	1	3
3545	3542	3549 -	3665	3664	3665 -	2	1	3
3636	3636	3639 +	3585 -	-	-	2	1	3
3660	3657	3664 -	3791 -	-	-	2	1	3
3810	3809	3811 +	3998 -	-	-	2	1	3
3911	3909	3911 +	4289 -	-	+	2	1	3
3954	3951	3958 -	4002 -	-	+	1	2	3
4066	4063	4069 +	4068	4065	4068 -	2	1	3
4072	4071	4072 -	4111	4111	4112 +	2	1	3
4075	4072	4075 +	12614 -	-	-	0	3	3
4106	4104	4108 -	4253	4250	4254 -	2	1	3
4125	4123	4125 -	4058 -	-	+	3	0	3
4166	4162	4166 +	4237 -	-	+	0	3	3
4171	4170	4173 +	4242	4241	4242 +	3	0	3
4194	4193	4198 +	4211 -	-	-	2	1	3
4219	4217	4224 -	4287 -	-	+	0	3	3
4229 -	-	-	4433 -	-	-	3	0	3

4270		4269	4270	-	4290	-	-		+	3	0	3
4437		4437	4439	-	10721	-	-		-	3	0	3
4443		4443	4444	-	4564		4564	4565	+	1	2	3
4452		4451	4452	+	4435		4435	4436	-	1	2	3
4501		4497	4505	-	4544		4542	4544	+	2	1	3
4501		4497	4505	-	4695	-	-		+	1	2	3
4559		4555	4560	-	4786		4786	4787	-	2	1	3
4660		4659	4664	+	6007		6003	6007	+	2	1	3
4746		4746	4750	-	5417		5416	5417	+	1	2	3
4827		4827	4829	+	4907	-	-		-	2	1	3
4907	-	-		+	4827	-	-		-	2	1	3
4939		4935	4940	+	7753		7753	7755	-	1	2	3
4946		4946	4949	-	5081		5081	5082	-	1	2	3
5016		5015	5017	+	4892		4888	4892	-	2	1	3
5039		5034	5039	-	5250	-	-		-	3	0	3
5046		5044	5049	-	5205	-	-		-	3	0	3
5072		5072	5073	-	15088		15085	15089	+	3	0	3
5078		5076	5082	-	5277	-	-		+	3	0	3
5084		5084	5085	-	5273	-	-		+	0	3	3
5159		5155	5159	+	6708	-	-		-	2	1	3
5212		5211	5213	+	15100	-	-		-	0	3	3
5235		5233	5236	-	5601		5600	5601	-	2	1	3
5317		5317	5321	+	5295	-	-		-	2	1	3
5355		5351	5359	+	5373		5372	5373	-	2	1	3
5381		5380	5381	+	5404	-	-		-	1	2	3
5443		5440	5447	-	5501	-	-		-	2	1	3
5476		5472	5480	-	14194	-	-		-	0	3	3
5489		5488	5490	-	5519		5517	5519	+	2	1	3
5506		5505	5510	+	10933		10933	10934	-	1	2	3
5545	-	-		+	5556	-	-		-	2	1	3
5662		5658	5664	-	5743	-	-		+	3	0	3
5662		5658	5664	-	5761		5758	5761	-	1	2	3
5664		5664	5666	+	5679	-	-		-	1	2	3
5667		5666	5669	-	5676	-	-		+	3	0	3
5691		5689	5696	-	5763	-	-		-	3	0	3
5700		5700	5704	-	5840		5836	5840	+	2	1	3
5749		5749	5751	-	5798	-	-		+	1	2	3
5809		5805	5809	-	5870	-	-		+	3	0	3
5815		5814	5820	-	5864	-	-		+	0	3	3
5848		5847	5848	+	5890		5889	5890	+	1	2	3
5856		5853	5857	-	5870		5870	5873	+	3	0	3
6181		6179	6183	-	11136		11136	11139	+	0	3	3
6256		6256	6258	+	6388		6388	6390	-	2	1	3
6263		6262	6266	-	6379		6379	6382	+	1	2	3
6309		6305	6312	-	6580	-	-		+	1	2	3
6390		6384	6390	+	6260		6256	6260	-	2	1	3
6427		6427	6428	-	6654	-	-		-	1	2	3
6435		6431	6436	-	6653		6653	6654	-	2	1	3
6464		6460	6464	-	6618		6618	6621	-	1	2	3
6540		6536	6541	+	8434		8434	8435	+	2	1	3
6573		6573	6576	+	6752		6749	6752	-	2	1	3
6573		6573	6576	+	8271	-	-		-	3	0	3
6650		6649	6652	+	6756		6756	6758	-	2	1	3
6697		6697	6699	-	6717	-	-		+	3	0	3
6717	-	-		-	6697	-	-		+	3	0	3
6857		6854	6858	-	9831		9830	9831	-	2	1	3
6915		6914	6915	+	6874		6874	6875	-	1	2	3
6949		6946	6950	-	7095	-	-		-	3	0	3
7034		7034	7038	+	13436	-	-		+	1	2	3
7091		7085	7094	-	7259		7258	7259	-	2	1	3
7091		7085	7094	-	7321		7318	7321	-	2	1	3
7097		7095	7097	-	7322	-	-		+	0	3	3
7146	-	-		+	7268	-	-		-	3	0	3
7173		7173	7177	+	7192	-	-		-	1	2	3
7285		7281	7287	+	7262	-	-		-	0	3	3
7326		7322	7330	+	7286		7284	7287	-	2	1	3
7356		7356	7360	+	7368	-	-		-	1	2	3
7395		7391	7396	-	7404	-	-		+	3	0	3
7404	-	-		-	7391	-	-		+	3	0	3
7517		7516	7520	-	7592		7592	7594	+	1	2	3
7711		7709	7711	-	7862		7862	7863	-	1	2	3
7785		7784	7786	-	7935	-	-		-	1	2	3
7857		7856	7858	-	10717	-	-		-	3	0	3
7922		7918	7922	+	7864		7864	7866	-	2	1	3
7966		7964	7967	-	7980		7977	7981	+	2	1	3
8012		8009	8013	-	8118	-	-		+	3	0	3
8018		8018	8019	-	8113	-	-		+	0	3	3
8024		8024	8025	-	8096	-	-		+	2	1	3
8048		8044	8049	-	8268		8268	8271	-	2	1	3
8060		8057	8063	-	8089		8087	8089	+	1	2	3
8060		8057	8063	-	8249	-	-		-	1	2	3
8193	-	-		+	8161	-	-		-	2	1	3
8200		8199	8200	-	8342		8342	8344	-	1	2	3
8244		8238	8245	-	8444		8444	8447	-	2	1	3
8293		8293	8295	-	8347		8347	8348	+	2	1	3
8396		8393	8396	+	8459		8459	8461	-	2	1	3
8607		8603	8611	-	8655	-	-		+	0	3	3
8607		8603	8611	-	8821		8819	8822	-	2	1	3
8644		8640	8647	-	8690		8688	8690	+	1	2	3
8687		8684	8691	-	8847	-	-		-	2	1	3
8742		8739	8747	+	8702		8699	8703	-	1	2	3
8752		8752	8753	+	8694	-	-		-	2	1	3
8811		8810	8813	-	8979	-	-		-	2	1	3
8964		8964	8965	+	8925	-	-		-	0	3	3
8971		8966	8974	-	9040		9040	9041	+	2	1	3
8975		8970	8976	+	8834	-	-		-	2	1	3

9005	9003	9008 -	9171	9170	9171 +	1	2	3
9071	9069	9075 -	9229 -	-	-	2	1	3
9103 -	-	-	9130 -	-	+	3	0	3
9121 -	-	-	9207 -	-	-	1	2	3
9129	9125	9134 -	9103 -	-	+	3	0	3
9146	9146	9147 +	9031	9030	9031 -	2	1	3
9152	9152	9155 -	9341	9338	9341 -	1	2	3
9197	9193	9202 -	9242	9240	9242 +	3	0	3
9197	9193	9202 -	9340	9340	9342 -	2	1	3
9259	9256	9259 +	9310 -	-	-	1	2	3
9273	9269	9274 -	9300 -	-	+	3	0	3
9289	9287	9292 -	9434 -	-	-	1	2	3
9295	9295	9298 -	9451	9450	9451 -	1	2	3
9305	9300	9309 -	9470	9470	9472 -	1	2	3
9370 -	-	-	9338 -	-	+	3	0	3
9388 -	-	-	9612 -	-	-	2	1	3
9451	9447	9454 -	9642 -	-	-	1	2	3
9498	9498	9499 -	9639	9636	9639 -	2	1	3
9574	9572	9574 +	9618	9616	9618 -	1	2	3
9598	9597	9602 +	9680	9679	9680 -	2	1	3
9611	9610	9611 +	9579 -	-	-	0	3	3
9616 -	-	+	9574 -	-	-	3	0	3
9684	9684	9687 +	9695 -	-	-	2	1	3
9710	9708	9714 -	9840	9840	9843 -	2	1	3
9734	9734	9738 +	9530 -	-	-	2	1	3
9749	9746	9751 -	9865 -	-	+	1	2	3
9844	9843	9847 -	9834	9832	9834 +	3	0	3
9905	9904	9905 +	11549	11548	11549 +	1	2	3
9973	9969	9973 -	9994	9994	9995 +	2	1	3
9979	9975	9981 -	10110	10110	10112 -	1	2	3
10009	10007	10011 +	9955 -	-	-	0	3	3
10081	10079	10085 +	10107 -	-	-	2	1	3
10083	10083	10087 -	10215	10213	10215 +	2	1	3
10092	10089	10092 +	10143 -	-	-	2	1	3
10126	10125	10130 -	10202 -	-	+	2	1	3
10206	10202	10206 +	10094 -	-	-	0	3	3
10213 -	-	+	10087 -	-	-	3	0	3
10266	10264	10270 -	10276 -	-	+	3	0	3
10273	10272	10277 -	10266 -	-	+	3	0	3
10362	10362	10364 +	10245 -	-	-	0	3	3
10369 -	-	+	10238 -	-	-	3	0	3
10408	10405	10410 -	13041 -	-	+	0	3	3
10510	10510	10513 -	10502	10500	10502 +	0	3	3
10582	10578	10585 +	10618 -	-	+	0	3	3
10587	10587	10590 +	10621 -	-	+	3	0	3
10662	10662	10665 +	10672 -	-	-	3	0	3
10746	10746	10748 -	12689 -	-	+	2	1	3
10797	10796	10798 -	12300 -	-	-	0	3	3
10958	10957	10960 -	11177	11173	11177 -	2	1	3
11054	11054	11056 +	11030	11026	11030 -	2	1	3
11061	11057	11061 -	11005 -	-	+	3	0	3
11198 -	-	+	11086 -	-	-	3	0	3
11199	11195	11204 -	11294	11294	11295 -	1	2	3
11255	11253	11256 -	11414	11413	11414 -	1	2	3
11269	11265	11273 -	11398	11398	11402 -	1	2	3
11269	11265	11273 -	11415	11414	11415 -	1	2	3
11342 -	-	+	11398 -	-	-	2	1	3
11351	11351	11354 -	11456 -	-	-	2	1	3
11381	11378	11384 -	11614 -	-	-	2	1	3
11398	11397	11402 +	11342 -	-	-	2	1	3
11500	11495	11504 -	11647 -	-	-	3	0	3
11508	11506	11512 -	11652 -	-	-	0	3	3
11538	11536	11539 -	11567 -	-	+	1	2	3
11781	11781	11789 -	11770 -	-	+	3	0	3
11856	11852	11856 -	11827 -	-	+	3	0	3
11931	11929	11934 -	12110 -	-	-	1	2	3
11953	11951	11956 -	12029	12029	12032 +	1	2	3
12076	12075	12076 +	12137 -	-	-	1	2	3
12082	12079	12086 -	12124	12124	12126 +	1	2	3
12082	12080	12087 +	11973	11973	11974 -	1	2	3
12123	12119	12127 -	12091	12088	12092 +	3	0	3
12123	12119	12127 -	12214 -	-	-	3	0	3
12129 -	-	-	12218 -	-	-	0	3	3
12147	12143	12147 +	12054 -	-	-	2	1	3
12159	12155	12161 -	12312 -	-	-	3	0	3
12168	12164	12168 -	12317 -	-	-	0	3	3
12261	12261	12265 +	12281 -	-	-	1	2	3
12288	12286	12291 -	12558 -	-	-	2	1	3
12309	12306	12311 -	12801 -	-	+	1	2	3
12320	12316	12322 -	12554	12552	12554 -	1	2	3
12343	12340	12346 -	12312	12308	12312 +	2	1	3
12360 -	-	+	12493 -	-	-	1	2	3
12366 -	-	-	15098 -	-	+	1	2	3
12500	12500	12502 -	12706	12706	12707 -	1	2	3
12506	12505	12506 +	12622	12621	12622 -	2	1	3
12515	12512	12519 -	12670	12669	12670 -	2	1	3
12557	12557	12560 +	12469 -	-	-	3	0	3
12557	12557	12560 +	12625	12624	12625 -	1	2	3
12562	12558	12566 -	12584	12581	12584 +	1	2	3
12603	12600	12603 -	12542 -	-	+	3	0	3
12626	12624	12628 +	12502	12500	12503 -	2	1	3
12657	12657	12661 -	13007 -	-	-	0	3	3
12672 -	-	-	13022 -	-	-	2	1	3
12754	12752	12759 -	12960 -	-	-	2	1	3
12767	12765	12771 -	12876 -	-	+	3	0	3
12776	12774	12779 -	12870 -	-	+	0	3	3

12800	-	-	+	12870	-	-	-	2	1	3
12806	12803	12809	+	12873	12873	12875	+	2	1	3
12817	12814	12817	-	12950	12950	12951	-	1	2	3
12818	12817	12822	+	12788	12787	12788	-	2	1	3
12830	12827	12830	-	12985	12985	12989	-	2	1	3
12861	12858	12862	-	12896	-	-	+	1	2	3
12879	12879	12882	-	13063	13063	13064	-	2	1	3
12891	12890	12894	-	13116	-	-	-	2	1	3
12988	12984	12991	-	13216	13213	13216	-	1	2	3
13011	13010	13016	+	13148	-	-	-	2	1	3
13055	13052	13057	+	13106	13106	13110	-	1	2	3
13072	13069	13076	-	13276	-	-	-	2	1	3
13113	13109	13117	-	13183	13182	13183	+	2	1	3
13188	13184	13193	+	13511	13510	13511	-	2	1	3
13268	-	-	-	13296	-	-	+	0	3	3
13338	13335	13341	-	13394	-	-	-	3	0	3
13338	13335	13341	-	13400	-	-	-	0	3	3
13392	13389	13392	+	13410	13410	13411	-	1	2	3
13397	13395	13397	+	13430	13430	13432	-	1	2	3
13651	-	-	+	14029	-	-	+	0	3	3
13691	13690	13695	-	13713	13713	13714	+	2	1	3
13839	13836	13840	-	13881	13878	13881	+	1	2	3
13854	13853	13856	-	13878	13878	13881	+	1	2	3
13854	13853	13856	-	13992	-	-	-	1	2	3
13861	13861	13865	-	13884	-	-	+	2	1	3
13879	13875	13880	+	13861	-	-	-	2	1	3
13889	13889	13890	-	13902	-	-	+	0	3	3
13891	13887	13894	+	13915	13915	13918	-	2	1	3
13899	13897	13903	+	13907	13906	13907	-	2	1	3
13948	13944	13951	-	14204	14202	14204	-	3	0	3
13968	13967	13971	-	14446	14446	14448	-	2	1	3
13990	13990	13992	+	14048	-	-	-	2	1	3
14098	14098	14102	-	14262	14259	14262	-	1	2	3
14132	14129	14132	+	14107	14107	14110	-	2	1	3
14192	14192	14194	+	14794	-	-	-	0	3	3
14203	14199	14205	+	14786	-	-	-	3	0	3
14322	-	-	+	14236	-	-	+	2	1	3
14324	14321	14326	-	14353	14353	14354	-	2	1	3
14344	14339	14346	-	14467	-	-	-	1	2	3
14391	14386	14392	-	14601	-	-	-	3	0	3
14404	14403	14405	-	14573	14570	14573	-	2	1	3
14535	14530	14537	+	14452	-	-	-	0	3	3
14535	14530	14537	+	14483	14482	14483	-	2	1	3
14687	14684	14690	-	14753	14753	14756	+	1	2	3
14692	14691	14697	-	14745	14745	14748	+	2	1	3
14700	-	-	-	14740	-	-	+	3	0	3
14706	14703	14710	-	14734	-	-	+	0	3	3
14719	14716	14723	-	14879	-	-	-	2	1	3
14744	14741	14750	-	14809	-	-	+	3	0	3
14752	14752	14755	-	14807	-	-	+	0	3	3
14761	14758	14763	-	14980	14980	14981	-	1	2	3
14771	14767	14771	-	15181	-	-	+	1	2	3
14806	14802	14807	-	14881	14879	14881	+	1	2	3
14812	14809	14813	-	14989	14988	14989	-	2	1	3
14841	14837	14841	+	15036	15035	15036	+	0	3	3
14847	14843	14851	-	14857	14857	14860	+	1	2	3
14855	14855	14858	+	14771	-	-	-	1	2	3
14866	14866	14872	-	14882	14882	14883	+	2	1	3
14892	14888	14895	+	14775	-	-	-	2	1	3
14925	14922	14928	-	14955	-	-	+	3	0	3
14933	14930	14935	-	14950	-	-	+	0	3	3
14963	14959	14966	-	15150	15147	15150	-	1	2	3
14963	14961	14963	+	14912	14912	14914	-	2	1	3
14978	14977	14981	-	15049	-	-	+	0	3	3
14978	14977	14981	-	15115	15114	15115	-	2	1	3
15027	15025	15032	-	15133	15132	15133	+	1	2	3
15027	15025	15032	-	15138	-	-	+	2	1	3
15052	15049	15055	-	15236	15232	15236	-	2	1	3
15052	15049	15055	-	15353	15352	15353	-	2	1	3
15082	15080	15084	-	15275	15275	15276	-	2	1	3
15104	15102	15108	-	15230	-	-	-	2	1	3
15112	15110	15115	-	15258	-	-	-	2	1	3
15130	15129	15133	+	15104	-	-	-	1	2	3
15161	15158	15161	-	15380	15380	15381	-	1	2	3
15166	15163	15169	-	15185	-	-	+	3	0	3
15166	15163	15169	-	15375	-	-	-	2	1	3
15185	15185	15187	-	15169	-	-	+	3	0	3
15195	-	-	-	15219	-	-	+	2	1	3
1	2	8	-	69	67	69	+	1	1	2
1	2	8	-	120	-	-	-	1	1	2
1	2	8	-	343	-	-	-	1	1	2
1	2	8	-	405	-	-	-	1	1	2
1	2	8	-	448	-	-	-	1	1	2
19	18	25	-	139	-	-	-	1	1	2
19	18	25	-	283	-	-	+	1	1	2
19	18	25	-	287	284	287	-	0	2	2
19	18	25	-	1569	1569	1570	+	0	2	2
21	17	21	+	298	294	298	+	1	1	2
58	57	62	-	109	-	-	+	1	1	2
58	57	62	-	184	181	184	+	1	1	2
58	57	62	-	236	-	-	-	1	1	2
58	57	62	-	339	-	-	+	1	1	2
62	-	-	+	384	-	-	-	0	2	2
68	63	68	-	198	-	-	-	1	1	2
68	63	68	-	232	-	-	-	1	1	2
68	63	68	-	248	-	-	-	1	1	2

68	-	-		+	378	-	-		-	2	0	2
75		74	77	-	273		273	274	-	1	1	2
85		85	88	-	132	-	-		-	1	1	2
85		85	88	-	229		229	230	-	1	1	2
85		85	88	-	289	-	-		-	1	1	2
97		93	100	-	186	-	-		+	1	1	2
97		93	100	-	223		223	224	-	1	1	2
97		93	100	-	373	-	-		-	1	1	2
98		97	98	+	82	-	-		-	1	1	2
106		105	106	-	157	-	-		+	1	1	2
112	-	-		-	312	-	-		-	1	1	2
112		109	112	+	203		203	204	-	1	1	2
112		109	112	+	14422	-	-		+	1	1	2
125		122	125	+	101	-	-		-	1	1	2
125		122	125	+	9427	-	-		+	1	1	2
130		130	131	-	339	-	-		-	1	1	2
133		133	136	+	157	-	-		+	1	1	2
133		133	136	+	248	-	-		-	1	1	2
141	-	-		-	386	-	-		-	1	1	2
146		146	148	+	555	-	-		-	1	1	2
146		146	148	+	5305	-	-		-	1	1	2
150		147	152	-	182	-	-		+	1	1	2
156	-	-		+	174	-	-		-	1	1	2
157		155	160	-	206	-	-		+	1	1	2
157		155	160	-	373	-	-		-	1	1	2
163		163	167	-	290	-	-		+	1	1	2
163		163	167	-	315	-	-		-	1	1	2
173		169	177	-	271	-	-		+	1	1	2
173		169	177	-	353	-	-		-	1	1	2
173		169	177	-	385	-	-		-	1	1	2
173	-	-		+	98	-	-		+	1	1	2
182	-	-		+	222	-	-		-	1	1	2
184		184	185	-	620	-	-		-	1	1	2
189		187	190	-	425		425	426	-	1	1	2
193		192	193	+	106	-	-		-	1	1	2
201		201	203	-	348	-	-		-	2	0	2
201		201	203	-	360		360	361	-	1	1	2
206		205	209	-	274		270	274	+	1	1	2
206		205	209	-	301	-	-		-	1	1	2
206		205	209	-	353	-	-		-	0	2	2
206	-	-		+	107		107	108	-	1	1	2
214		211	217	-	268		268	269	+	1	1	2
222		222	227	+	261		261	262	-	1	1	2
224		222	225	-	380	-	-		-	1	1	2
229		227	232	-	387		387	388	-	1	1	2
229		227	232	-	439	-	-		-	1	1	2
232	-	-		+	243	-	-		-	0	2	2
239		238	242	-	307		303	307	+	1	1	2
239		238	242	-	968	-	-		-	1	1	2
246		243	249	+	468	-	-		-	1	1	2
260		257	263	-	385	-	-		-	1	1	2
260		257	263	-	1573		1573	1574	-	1	1	2
263	-	-		+	15123	-	-		-	1	1	2
268		265	268	-	363		363	364	+	1	1	2
268		265	268	-	426	-	-		-	2	0	2
268		265	268	-	504	-	-		-	1	1	2
268		265	268	-	798	-	-		-	1	1	2
273		270	274	-	428	-	-		-	0	2	2
273		270	274	-	471	-	-		-	1	1	2
273		270	274	-	495	-	-		-	1	1	2
273		270	274	-	628	-	-		+	1	1	2
278		276	283	-	294	-	-		+	1	1	2
278		276	283	-	324	-	-		+	1	1	2
278		276	283	-	357	-	-		+	1	1	2
278		276	283	-	382	-	-		-	1	1	2
278		276	283	-	673		670	673	+	1	1	2
290		288	295	-	326	-	-		+	1	1	2
290		288	295	-	432	-	-		-	1	1	2
290		288	295	-	444	-	-		+	1	1	2
290		288	295	-	645	-	-		-	1	1	2
295		291	297	+	418	-	-		-	1	1	2
295		291	297	+	427	-	-		+	1	1	2
295		291	297	+	8961	-	-		+	1	1	2
300	-	-		-	433	-	-		+	1	1	2
306		306	308	-	651		651	652	-	1	1	2
308		306	312	+	319		319	320	+	1	1	2
308		306	312	+	379	-	-		-	1	1	2
314	-	-		+	377	-	-		-	0	2	2
320		318	321	-	688		688	689	-	1	1	2
323		321	327	+	406		406	407	-	1	1	2
323		321	327	+	416	-	-		+	1	1	2
323		321	327	+	499	-	-		-	1	1	2
330	-	-		-	572	-	-		-	1	1	2
335		333	335	-	391		391	392	+	1	1	2
335		333	335	-	564	-	-		-	1	1	2
335		332	335	+	558	-	-		-	1	1	2
335		332	335	+	612	-	-		+	1	1	2
341		338	345	-	505		505	506	-	1	1	2
341		338	345	-	528	-	-		+	1	1	2
341		338	345	-	554	-	-		-	1	1	2
341		338	345	-	583	-	-		-	1	1	2
343		343	345	+	295	-	-		-	1	1	2
343		343	345	+	495	-	-		-	1	1	2
347		347	350	-	516	-	-		-	1	1	2
352		351	353	-	388		388	389	+	1	1	2
352		351	353	-	529	-	-		-	1	1	2

352		352	354	+	293	-	-		-	1	1	2
352		352	354	+	382		382	383	-	1	1	2
357		357	358	+	14958	-	-		+	1	1	2
358		355	360	-	493	-	-		-	1	1	2
358		355	360	-	518	-	-		-	1	1	2
358		355	360	-	542	-	-		-	1	1	2
358		355	360	-	555	-	-		-	1	1	2
367		367	370	+	465	-	-		+	1	1	2
367		367	370	+	489	-	-		-	1	1	2
369		366	369	-	410	-	-		+	2	0	2
369		366	369	-	441	-	-		+	1	1	2
369		366	369	-	527	-	-		-	1	1	2
374		372	375	-	585	-	-		-	1	1	2
376		373	380	+	457	-	-		-	2	0	2
376		373	380	+	5927	-	-		+	1	1	2
386		386	388	+	415	-	-		+	0	2	2
386		386	388	+	713	-	-		-	0	2	2
386		386	388	+	723	-	-		-	0	2	2
388	-	-		-	475	-	-		+	1	1	2
393		390	396	+	326	-	-		-	1	1	2
393		390	396	+	419	-	-		+	2	0	2
393		390	396	+	473	-	-		-	1	1	2
393		390	396	+	707	-	-		-	2	0	2
393		390	396	+	717	-	-		-	2	0	2
395		390	400	-	418	-	-		+	1	1	2
395		390	400	-	950		946	950	+	1	1	2
399		398	399	+	340		340	341	-	1	1	2
404		404	405	-	630	-	-		-	1	1	2
408		407	411	+	464	-	-		+	1	1	2
408		407	411	+	512		510	512	+	1	1	2
411		408	414	-	366	-	-		+	2	0	2
411		408	414	-	490		487	490	-	1	1	2
411		408	414	-	579	-	-		-	1	1	2
411		408	414	-	860		860	861	-	1	1	2
414	-	-		+	648	-	-		-	1	1	2
417	-	-		-	503	-	-		+	1	1	2
422	-	-		+	516	-	-		+	1	1	2
428		427	432	-	474	-	-		+	1	1	2
428		427	432	-	557	-	-		-	1	1	2
428		427	432	-	655		655	656	-	1	1	2
445	-	-		-	583	-	-		-	1	1	2
460		458	462	-	627		627	628	-	0	2	2
460		458	462	-	645	-	-		-	1	1	2
460		458	462	-	944	-	-		-	1	1	2
464		461	466	+	486		483	486	+	1	1	2
469		469	472	+	454	-	-		+	1	1	2
469		469	472	+	518	-	-		-	1	1	2
475		471	476	-	527	-	-		+	1	1	2
475		471	476	-	656		656	657	-	1	1	2
475		471	476	-	704		704	705	-	1	1	2
480		480	482	-	520	-	-		+	1	1	2
480		480	482	-	674	-	-		-	1	1	2
482		482	485	+	472		469	472	-	1	1	2
491	-	-		-	655	-	-		-	1	1	2
491	-	-		-	704	-	-		-	1	1	2
498		498	499	+	393	-	-		-	1	1	2
498		498	499	+	448	-	-		-	1	1	2
500		497	504	-	707		704	707	-	1	1	2
509		505	509	+	458	-	-		-	1	1	2
509		505	509	+	467		465	467	-	1	1	2
513		509	514	-	701	-	-		-	1	1	2
517		517	518	+	429	-	-		-	1	1	2
517		517	518	+	532		532	533	+	1	1	2
518		515	521	-	635	-	-		+	1	1	2
530		526	534	-	654	-	-		-	1	1	2
530		526	534	-	664	-	-		-	1	1	2
530		526	534	-	672	-	-		-	1	1	2
530		526	534	-	1544		1544	1545	-	1	1	2
530		526	534	-	3485	-	-		-	1	1	2
535		533	535	+	391	-	-		-	1	1	2
535		533	535	+	697		697	698	-	1	1	2
541		541	545	+	385	-	-		-	1	1	2
552	-	-		-	616	-	-		+	1	1	2
557		557	560	-	703	-	-		-	2	0	2
558	-	-		+	803	-	-		-	1	1	2
564		564	568	-	618	-	-		+	1	1	2
564		564	568	-	708	-	-		-	0	2	2
564		564	568	-	745	-	-		+	1	1	2
564	-	-		+	599	-	-		-	1	1	2
576		573	576	-	614	-	-		+	1	1	2
576		573	576	-	815	-	-		-	0	2	2
582		579	586	-	607		603	607	+	1	1	2
584		583	585	+	686	-	-		-	1	1	2
591	-	-		-	679	-	-		-	1	1	2
592		592	595	+	679	-	-		-	1	1	2
592		592	595	+	969	-	-		+	1	1	2
606		602	609	-	778	-	-		-	1	1	2
606		602	609	-	791		791	792	-	1	1	2
606		602	609	-	969	-	-		-	1	1	2
607		605	609	+	667	-	-		-	1	1	2
614		612	614	+	573	-	-		-	1	1	2
617		614	617	-	817	-	-		-	1	1	2
617		614	617	-	970		970	971	-	1	1	2
619	-	-		+	910	-	-		+	1	1	2
627		625	627	+	674	-	-		-	1	1	2
633		631	637	+	521	-	-		-	1	1	2

633	631	637	+	695	693	695	-	1	1	2
633	631	637	+	1039	-	-	-	1	1	2
634	633	638	-	696	-	-	-	1	1	2
634	633	638	-	746	-	-	-	1	1	2
634	633	638	-	11876	-	-	+	1	1	2
643	640	648	-	810	-	-	-	1	1	2
643	640	648	-	819	-	-	-	1	1	2
645	-	-	+	565	-	-	-	1	1	2
650	650	654	+	614	-	-	-	1	1	2
650	650	654	+	679	-	-	-	1	1	2
659	659	662	-	700	-	-	+	1	1	2
659	-	-	+	831	-	-	-	1	1	2
665	663	670	-	831	-	-	-	1	1	2
665	663	670	-	892	-	-	-	1	1	2
665	663	670	-	905	905	906	-	1	1	2
665	663	670	-	934	-	-	-	1	1	2
665	662	668	+	823	823	824	-	1	1	2
665	662	668	+	906	-	-	+	1	1	2
672	672	676	-	801	-	-	-	1	1	2
672	672	676	-	876	-	-	-	1	1	2
672	669	672	+	847	-	-	-	1	1	2
685	684	688	+	729	-	-	-	1	1	2
689	685	689	-	774	-	-	+	1	1	2
693	690	693	+	722	722	723	-	1	1	2
700	699	705	+	678	-	-	-	1	1	2
700	699	705	+	710	-	-	+	1	1	2
700	699	705	+	801	-	-	-	1	1	2
708	704	712	-	721	-	-	+	2	0	2
708	704	712	-	773	-	-	+	1	1	2
708	704	712	-	778	-	-	+	1	1	2
717	714	720	-	911	-	-	-	1	1	2
717	714	719	+	810	-	-	-	1	1	2
722	722	726	-	743	-	-	+	0	2	2
722	722	726	-	758	-	-	+	1	1	2
722	722	726	-	903	-	-	-	1	1	2
725	722	726	+	714	-	-	-	1	1	2
725	722	726	+	811	811	812	-	1	1	2
725	722	726	+	880	-	-	+	1	1	2
725	722	726	+	940	-	-	-	1	1	2
734	731	737	-	747	-	-	+	1	1	2
734	731	737	-	878	-	-	-	2	0	2
734	731	737	-	883	-	-	-	0	2	2
748	744	750	+	773	-	-	-	1	1	2
750	748	752	-	708	-	-	+	1	1	2
750	748	752	-	867	863	867	-	1	1	2
750	748	752	-	1421	-	-	+	1	1	2
756	756	760	-	780	-	-	+	1	1	2
756	756	760	-	951	947	951	-	1	1	2
757	755	761	+	714	-	-	-	1	1	2
757	755	761	+	725	725	726	-	1	1	2
769	769	772	-	1817	1815	1817	-	1	1	2
773	769	775	+	712	-	-	-	1	1	2
773	769	775	+	782	-	-	-	0	2	2
779	777	786	-	975	-	-	-	1	1	2
782	779	782	+	745	-	-	-	1	1	2
782	779	782	+	775	-	-	-	2	0	2
789	787	791	-	851	-	-	+	1	1	2
789	787	791	-	907	-	-	-	1	1	2
789	787	791	-	925	-	-	-	1	1	2
789	787	791	-	1006	-	-	-	1	1	2
794	793	796	-	909	-	-	-	1	1	2
794	793	796	-	9095	9095	9096	-	1	1	2
800	798	800	+	1228	-	-	+	1	1	2
802	798	802	-	835	-	-	+	1	1	2
802	798	802	-	1229	-	-	-	1	1	2
808	805	810	-	905	-	-	+	1	1	2
808	805	810	-	941	941	942	-	1	1	2
808	805	810	-	1015	-	-	-	1	1	2
808	805	810	-	1039	1039	1040	-	1	1	2
810	810	812	+	869	869	870	-	1	1	2
817	812	817	-	1000	998	1000	-	1	1	2
817	815	817	+	6155	-	-	+	1	1	2
822	822	826	-	983	-	-	-	1	1	2
822	822	826	-	1015	-	-	-	1	1	2
827	-	-	+	660	660	661	-	1	1	2
832	828	835	-	976	-	-	-	1	1	2
832	828	835	-	1050	1047	1050	-	1	1	2
832	828	835	-	3562	-	-	+	0	2	2
832	828	835	-	4373	-	-	-	0	2	2
844	840	845	-	909	-	-	+	1	1	2
844	840	845	-	998	-	-	-	1	1	2
844	840	845	-	1000	-	-	+	1	1	2
844	840	845	-	1083	-	-	-	1	1	2
844	840	845	-	3466	3466	3468	+	0	2	2
844	840	845	-	6177	-	-	-	0	2	2
847	843	847	+	912	908	912	-	1	1	2
853	851	857	-	958	-	-	-	1	1	2
853	851	857	-	981	-	-	-	1	1	2
853	851	857	-	1014	-	-	-	1	1	2
853	849	856	+	818	-	-	-	1	1	2
853	849	856	+	9153	-	-	+	1	1	2
863	860	865	-	978	-	-	-	1	1	2
865	862	869	+	779	-	-	-	1	1	2
865	862	869	+	950	950	951	-	1	1	2
865	862	869	+	981	-	-	-	1	1	2
873	871	878	-	978	976	978	-	1	1	2

873	871	878	-	1006	-	-		-	1	1	2
873	871	878	-	1017	-	-		-	1	1	2
873	871	878	-	1129	-	-		-	1	1	2
873	871	878	-	1134		1131	1134	+	1	1	2
873	871	878	-	1270		1270	1271	-	1	1	2
881	880	882	-	894	-	-		+	2	0	2
881	880	882	-	1415	-	-		+	1	1	2
883	879	888	+	827		827	828	-	1	1	2
883	879	888	+	852	-	-		-	1	1	2
883	879	888	+	988	-	-		+	1	1	2
889	885	893	-	891	-	-		+	0	2	2
889	885	893	-	1404	-	-		+	1	1	2
895	895	898	-	1051	-	-		-	1	1	2
895	895	898	-	1116		1116	1117	-	1	1	2
900	900	904	+	969		969	970	-	1	1	2
903	903	907	-	984	-	-		+	1	1	2
903	903	907	-	1032	-	-		-	1	1	2
903	903	907	-	1127	-	-		-	1	1	2
903	903	907	-	1197	-	-		-	1	1	2
903	903	907	-	6686	-	-		-	1	1	2
908	905	910	+	959	-	-		-	1	1	2
908	905	910	+	974	-	-		-	1	1	2
908	905	910	+	1135		1131	1135	-	1	1	2
917	912	917	+	939	-	-		-	1	1	2
917	912	917	+	942	-	-		+	1	1	2
918	917	922	-	1790		1790	1791	-	1	1	2
924	921	928	+	959	-	-		-	1	1	2
924	921	928	+	975	-	-		-	1	1	2
928	928	930	-	8479	-	-		-	2	0	2
941	939	947	-	978	-	-		+	1	1	2
941	939	947	-	1153	-	-		-	1	1	2
941	939	947	-	1267	-	-		+	1	1	2
945	941	947	+	860		857	860	-	1	1	2
945	941	947	+	874	-	-		-	1	1	2
959	957	961	+	995	-	-		-	1	1	2
959	957	961	+	1020		1016	1020	-	1	1	2
959	957	961	+	3052	-	-		+	2	0	2
960	957	963	-	1087	-	-		-	1	1	2
960	957	963	-	1134		1134	1135	-	1	1	2
968	968	972	-	1087	-	-		-	1	1	2
968	968	972	-	1103	-	-		-	1	1	2
968	968	972	-	1199	-	-		-	1	1	2
972	972	975	+	1017	-	-		-	1	1	2
974	973	977	-	1098	-	-		-	1	1	2
974	973	977	-	1121	-	-		-	0	2	2
981	978	985	-	1090		1087	1090	+	1	1	2
981	978	985	-	1123	-	-		-	1	1	2
981	978	985	-	1150	-	-		-	1	1	2
981	978	985	-	1173		1171	1173	-	1	1	2
991	987	993	-	1104	-	-		-	1	1	2
991	987	993	-	1157	-	-		-	2	0	2
991	987	993	-	1162	-	-		-	0	2	2
991	987	993	-	1177	-	-		-	1	1	2
991	989	993	+	1115		1111	1115	-	1	1	2
996	996	997	-	1155		1155	1156	-	1	1	2
1003	999	1006	-	1055	-	-		+	1	1	2
1003	999	1006	-	1153	-	-		-	1	1	2
1003	999	1006	-	1172	-	-		-	1	1	2
1003	1001	1004	+	984		981	984	-	1	1	2
1010	1008	1011	+	1111	-	-		-	1	1	2
1012	1008	1013	-	1046		1044	1046	+	1	1	2
1012	1008	1013	-	1069	-	-		+	1	1	2
1012	1008	1013	-	1111	-	-		+	1	1	2
1016	1013	1021	+	979		977	979	-	1	1	2
1016	1013	1021	+	988		988	989	+	1	1	2
1016	1013	1021	+	1158	-	-		+	1	1	2
1019	1018	1019	-	1070		1070	1071	+	1	1	2
1031	1030	1035	-	1101		1097	1101	-	1	1	2
1031	1030	1035	-	1186		1186	1187	-	1	1	2
1031	1030	1035	-	1249	-	-		+	1	1	2
1031	1026	1031	+	905	-	-		-	1	1	2
1049	1049	1054	-	1127	-	-		+	1	1	2
1049	1049	1054	-	1151	-	-		-	1	1	2
1049	1049	1054	-	1295		1295	1296	-	1	1	2
1049	1049	1054	-	4639	-	-		-	1	1	2
1049	1049	1051	+	1194		1192	1194	+	1	1	2
1067	1063	1070	-	1031	-	-		+	2	0	2
1067	1063	1070	-	1252		1252	1253	-	1	1	2
1067 -	-		+	1031	-	-		-	1	1	2
1073	1073	1074	-	1209	-	-		-	1	1	2
1073	1073	1074	-	1215	-	-		-	1	1	2
1081	1077	1083	-	1270	-	-		-	1	1	2
1081	1077	1083	-	1325	-	-		-	1	1	2
1087	1084	1092	+	1268	-	-		-	1	1	2
1089	1084	1091	-	1105	-	-		+	1	1	2
1089	1084	1091	-	1247		1244	1247	-	1	1	2
1089	1084	1091	-	1270		1270	1271	-	1	1	2
1094	1093	1095	-	1210	-	-		-	1	1	2
1094	1093	1095	-	1251	-	-		-	1	1	2
1094	1093	1095	-	1270	-	-		-	1	1	2
1094	1093	1095	-	1281	-	-		-	1	1	2
1094	1093	1095	-	1297	-	-		-	2	0	2
1100	1096	1104	-	1135	-	-		-	1	1	2
1100	1096	1104	-	1199	-	-		-	1	1	2
1100	1096	1104	-	1232	-	-		-	1	1	2
1100	1096	1104	-	1238	-	-		-	1	1	2

1100		1096	1104	-	1302	-	-		-	1	1	2
1100		1096	1104	-	1335	-	-		-	1	1	2
1100		1098	1103	+	1160		1156	1160	-	1	1	2
1106	-	-		+	1087	-	-		-	1	1	2
1111		1110	1115	-	1182		1178	1182	+	1	1	2
1118		1116	1118	+	1129	-	-		-	1	1	2
1118		1116	1118	+	1148		1148	1149	+	1	1	2
1121		1117	1124	-	1155	-	-		+	1	1	2
1121		1117	1124	-	1160	-	-		+	1	1	2
1121		1117	1124	-	1168	-	-		+	1	1	2
1123		1122	1125	+	1035	-	-		-	1	1	2
1123		1122	1125	+	1044	-	-		-	1	1	2
1132		1128	1137	-	1222	-	-		-	1	1	2
1132		1128	1137	-	1326		1326	1327	-	1	1	2
1132		1128	1137	-	1814	-	-		-	1	1	2
1132		1127	1134	+	1055	-	-		+	1	1	2
1132		1127	1134	+	1118	-	-		-	1	1	2
1142		1137	1142	+	1324	-	-		-	1	1	2
1143		1142	1146	-	1327	-	-		-	1	1	2
1150		1149	1152	-	1203	-	-		-	1	1	2
1150		1149	1152	-	1268	-	-		+	1	1	2
1150		1149	1152	-	1339	-	-		-	1	1	2
1153		1153	1156	+	1257	-	-		-	2	0	2
1158		1155	1160	-	1182		1180	1182	+	1	1	2
1158		1155	1160	-	1307		1307	1308	+	1	1	2
1158		1155	1160	-	1344		1344	1345	-	1	1	2
1158		1155	1160	-	1378		1378	1379	-	1	1	2
1162		1160	1162	+	1128	-	-		-	1	1	2
1163	-	-		-	1307	-	-		+	1	1	2
1168		1166	1168	+	1272	-	-		-	1	1	2
1168		1166	1168	+	1306	-	-		-	1	1	2
1169		1167	1172	-	1306	-	-		-	1	1	2
1179		1176	1179	-	1299	-	-		-	1	1	2
1179		1176	1179	-	1308	-	-		-	1	1	2
1186	-	-		+	1153	-	-		-	1	1	2
1187		1185	1189	-	1204		1202	1204	+	1	1	2
1187		1185	1189	-	1233		1229	1233	+	1	1	2
1187		1185	1189	-	1280	-	-		-	1	1	2
1187		1185	1189	-	1325	-	-		-	1	1	2
1197		1197	1200	-	1326	-	-		-	1	1	2
1197		1197	1200	-	1381	-	-		-	1	1	2
1197		1197	1200	-	1547		1547	1548	-	1	1	2
1200		1198	1201	+	1225	-	-		+	1	1	2
1200		1198	1201	+	1248		1248	1249	+	1	1	2
1200		1198	1201	+	1283	-	-		+	1	1	2
1205		1201	1208	-	1349	-	-		-	1	1	2
1205		1201	1208	-	1388	-	-		-	1	1	2
1212	-	-		+	1240	-	-		-	1	1	2
1215		1210	1218	-	1362	-	-		-	1	1	2
1215		1210	1218	-	1384	-	-		-	1	1	2
1215		1210	1218	-	1447	-	-		-	1	1	2
1215		1210	1218	-	5992		5989	5992	+	2	0	2
1217		1217	1220	+	1137	-	-		-	1	1	2
1220		1220	1224	-	1381	-	-		-	1	1	2
1230		1227	1232	-	1373	-	-		-	1	1	2
1230		1227	1232	-	1419	-	-		-	1	1	2
1230		1227	1232	+	1424	-	-		-	1	1	2
1230		1227	1232	+	1496	-	-		+	1	1	2
1235	-	-		-	1405	-	-		-	1	1	2
1240		1238	1244	-	1380		1380	1381	-	1	1	2
1240		1238	1244	-	10223	-	-		-	0	2	2
1240		1236	1244	+	1323	-	-		-	1	1	2
1240		1236	1244	+	1401	-	-		-	1	1	2
1240		1236	1244	+	7398	-	-		-	2	0	2
1248		1248	1251	+	1166		1166	1167	-	1	1	2
1248		1248	1251	+	7388	-	-		-	2	0	2
1250		1245	1254	-	1380	-	-		-	1	1	2
1250		1245	1254	-	1493	-	-		+	1	1	2
1257		1257	1259	-	1411	-	-		-	1	1	2
1260	-	-		+	1150	-	-		-	2	0	2
1267		1265	1270	-	1336	-	-		-	2	0	2
1267		1265	1270	-	1455	-	-		-	1	1	2
1274		1272	1275	-	1259	-	-		+	2	0	2
1274		1272	1275	-	1315		1313	1315	+	1	1	2
1274		1272	1275	-	1582	-	-		-	2	0	2
1275		1275	1280	+	1143	-	-		-	1	1	2
1275		1275	1280	+	1357	-	-		+	1	1	2
1281		1277	1287	-	1299	-	-		+	1	1	2
1281		1277	1287	-	1343	-	-		-	0	2	2
1281		1277	1287	-	1365	-	-		+	1	1	2
1281		1277	1287	-	1461	-	-		-	2	0	2
1281		1277	1287	-	1553		1550	1553	-	1	1	2
1281		1277	1287	-	1586	-	-		-	0	2	2
1292		1292	1294	-	1468	-	-		-	0	2	2
1293		1292	1297	+	1134	-	-		-	1	1	2
1299		1298	1303	+	1355		1355	1356	-	1	1	2
1299		1298	1303	+	1498	-	-		-	1	1	2
1303		1298	1307	-	1365	-	-		+	1	1	2
1306		1306	1310	+	1145	-	-		-	1	1	2
1306		1306	1310	+	1365	-	-		-	1	1	2
1313		1309	1313	-	1475	-	-		-	1	1	2
1316		1316	1317	+	4564		4564	4565	-	1	1	2
1322		1322	1325	-	1329		1329	1330	+	1	1	2
1327		1327	1331	-	1424	-	-		+	1	1	2
1327		1327	1331	-	1474	-	-		-	1	1	2
1327		1327	1331	-	1519	-	-		-	1	1	2

1329	1327	1333 +	1285 -	-	-	1	1	2
1329	1327	1333 +	1364 -	-	-	1	1	2
1329	1327	1333 +	1517 -	-	+	1	1	2
1340	1340	1341 -	1313 -	-	+	2	0	2
1340	1340	1342 +	1408	1406	1408 -	2	0	2
1340	1340	1342 +	1418 -	-	-	1	1	2
1351 -	-	+	1249 -	-	-	1	1	2
1354	1351	1354 -	1588 -	-	-	1	1	2
1356	1355	1357 +	1530	1530	1531 +	1	1	2
1359	1355	1364 -	1650 -	-	-	1	1	2
1362	1359	1364 +	1403	1400	1403 -	1	1	2
1366	1365	1370 -	1545 -	-	-	1	1	2
1366	1365	1370 -	1556	1556	1557 -	1	1	2
1372	1368	1375 +	1296 -	-	-	1	1	2
1372	1368	1375 +	1302	1302	1303 -	1	1	2
1372	1368	1375 +	1338 -	-	-	1	1	2
1372	1368	1375 +	12084	12082	12084 -	2	0	2
1375	1371	1379 -	1389 -	-	+	1	1	2
1375	1371	1379 -	1424	1420	1424 +	1	1	2
1375	1371	1379 -	1531	1527	1531 -	1	1	2
1375	1371	1379 -	1560 -	-	-	1	1	2
1375	1371	1379 -	1690 -	-	+	1	1	2
1383	1381	1386 -	1481 -	-	+	1	1	2
1383	1381	1386 -	1589 -	-	-	1	1	2
1383	1381	1386 -	1723 -	-	+	1	1	2
1388	1388	1389 +	1497 -	-	-	1	1	2
1388	1388	1389 +	1546	1546	1547 -	1	1	2
1389	1388	1398 -	1443 -	-	-	1	1	2
1389	1388	1398 -	1492 -	-	+	0	2	2
1389	1388	1398 -	1497 -	-	+	2	0	2
1389	1388	1398 -	1513 -	-	+	1	1	2
1389	1388	1398 -	1675 -	-	-	1	1	2
1395	1393	1397 +	1467 -	-	+	1	1	2
1400	1399	1401 -	1583 -	-	-	1	1	2
1400	1398	1402 +	1354	1352	1354 -	1	1	2
1400	1398	1402 +	1570 -	-	+	1	1	2
1400	1398	1402 +	1662	1662	1663 -	1	1	2
1407	1404	1410 -	1470	1468	1470 +	1	1	2
1407	1404	1410 -	1659 -	-	+	1	1	2
1412	1411	1413 -	1612 -	-	-	1	1	2
1423	1420	1424 +	1378	1374	1378 -	1	1	2
1423	1420	1424 +	1494 -	-	-	1	1	2
1423	1420	1424 +	1616 -	-	-	1	1	2
1428	1425	1428 -	1461 -	-	+	1	1	2
1428 -	-	+	1650 -	-	-	1	1	2
1435	1434	1438 -	1580 -	-	-	1	1	2
1435	1434	1438 -	1603 -	-	-	1	1	2
1435	1434	1438 -	1622 -	-	+	1	1	2
1449	1447	1453 -	1455	1453	1455 +	1	1	2
1449	1447	1453 -	1504 -	-	-	1	1	2
1449	1447	1453 -	1532 -	-	-	1	1	2
1449	1447	1453 -	1642 -	-	+	1	1	2
1449	1447	1453 -	6705	6702	6705 -	0	2	2
1451	1446	1455 +	1405	1402	1405 -	0	2	2
1451	1446	1455 +	1410 -	-	-	1	1	2
1451	1446	1455 +	1642 -	-	-	1	1	2
1451	1446	1455 +	4422	4422	4423 -	1	1	2
1460	1456	1462 -	1523 -	-	+	1	1	2
1460	1456	1462 -	1666	1663	1666 -	1	1	2
1467	1466	1470 -	1526	1526	1527 -	1	1	2
1467	1466	1470 -	1600 -	-	-	1	1	2
1467	1466	1470 -	1626	1626	1627 +	1	1	2
1467	1466	1470 -	1686	1684	1686 -	1	1	2
1467	1465	1468 +	1389	1389	1390 -	1	1	2
1467	1465	1468 +	4409 -	-	-	2	0	2
1472	1471	1474 -	1519 -	-	+	1	1	2
1472	1471	1474 -	1686 -	-	-	1	1	2
1472	1471	1474 -	1696 -	-	-	1	1	2
1481	1477	1482 -	1690 -	-	-	1	1	2
1487	1484	1492 -	12082 -	-	-	2	0	2
1488	1484	1491 +	1565 -	-	+	1	1	2
1488	1484	1491 +	1618 -	-	-	1	1	2
1488	1484	1491 +	1627 -	-	-	1	1	2
1494	1494	1497 +	1573 -	-	-	1	1	2
1505	1504	1506 -	1519	1517	1519 +	1	1	2
1505	1504	1506 -	1693 -	-	-	1	1	2
1507	1507	1508 +	1446 -	-	-	1	1	2
1513	1509	1515 -	1631 -	-	-	1	1	2
1513	1509	1515 -	1652 -	-	-	1	1	2
1513	1509	1515 -	1810 -	-	-	2	0	2
1513 -	-	+	1552 -	-	+	1	1	2
1524	1521	1524 -	2713 -	-	+	1	1	2
1524 -	-	+	4427 -	-	-	1	1	2
1529	1529	1532 +	1404 -	-	-	1	1	2
1529	1529	1532 +	1658 -	-	+	0	2	2
1530	1527	1531 -	1612 -	-	+	1	1	2
1530	1527	1531 -	1657 -	-	+	1	1	2
1530	1527	1531 -	1662 -	-	-	1	1	2
1530	1527	1531 -	1700	1700	1701 -	1	1	2
1534 -	-	+	1661 -	-	+	2	0	2
1542	1542	1543 +	1392	1392	1393 -	1	1	2
1552	1548	1557 -	1656 -	-	+	1	1	2
1552	1548	1557 -	1757 -	-	-	1	1	2
1552	1548	1557 -	1908 -	-	-	1	1	2
1552	1548	1557 -	3360 -	-	-	1	1	2
1552	1552	1554 +	1399	1399	1400 -	1	1	2

1552	1552	1554 +	1579 -	-	+	1	1	2
1561	1560	1563 -	1587	1587	1588 -	1	1	2
1561	1560	1563 -	1731 -	-	-	1	1	2
1567	1564	1571 -	1742 -	-	-	1	1	2
1567	1564	1571 -	1757 -	-	-	1	1	2
1567	1564	1571 -	1770 -	-	-	1	1	2
1567	1564	1571 -	1775 -	-	-	1	1	2
1567	1564	1571 -	1796	1796	1798 -	1	1	2
1567	1564	1571 -	2312 -	-	-	2	0	2
1586	1583	1586 +	4422 -	-	-	1	1	2
1592	1588	1595 -	1724 -	-	-	1	1	2
1592	1588	1595 -	8145 -	-	-	1	1	2
1602	1602	1604 -	1657 -	-	+	1	1	2
1602	1602	1604 -	1740 -	-	-	1	1	2
1602	1602	1604 -	1803 -	-	-	1	1	2
1616	1614	1621 -	1715 -	-	+	1	1	2
1621	1620	1625 +	1703 -	-	+	0	2	2
1621	1620	1625 +	1708 -	-	+	2	0	2
1632	1630	1632 +	6129 -	-	-	1	1	2
1641	1637	1645 +	1667 -	-	+	0	2	2
1641	1637	1645 +	3317 -	-	-	1	1	2
1647	1646	1647 +	1678	1678	1679 +	1	1	2
1649	1649	1654 -	1806 -	-	-	1	1	2
1649	1649	1654 -	1845 -	-	-	1	1	2
1649	1649	1654 -	1912 -	-	-	2	0	2
1649	1649	1654 -	1976 -	-	+	1	1	2
1658	1655	1659 -	1850 -	-	-	1	1	2
1658	1655	1659 -	1937 -	-	+	1	1	2
1663	1663	1665 -	1840	1838	1840 -	1	1	2
1663	1663	1665 -	1851 -	-	-	1	1	2
1666	1662	1666 +	1648 -	-	-	1	1	2
1671	1668	1674 -	1806 -	-	-	1	1	2
1678 -	-	+	1599 -	-	-	1	1	2
1678 -	-	+	1623 -	-	-	1	1	2
1691	1689	1691 -	2003 -	-	-	1	1	2
1697	1697	1698 -	1800 -	-	+	2	0	2
1697	1697	1698 -	1854 -	-	-	1	1	2
1703	1703	1705 -	1713	1711	1713 +	1	1	2
1703	1703	1705 -	1795 -	-	+	0	2	2
1703	1703	1705 -	1836 -	-	-	1	1	2
1720	1716	1721 -	1758	1756	1758 +	1	1	2
1720	1716	1721 -	1854 -	-	-	1	1	2
1720	1716	1721 -	1875 -	-	-	1	1	2
1720	1716	1721 -	2060 -	-	+	1	1	2
1723 -	-	+	1861 -	-	-	1	1	2
1731	1729	1731 +	1690 -	-	-	1	1	2
1739	1739	1741 +	1939 -	-	-	1	1	2
1740	1739	1741 -	1896	1896	1897 -	1	1	2
1740	1739	1741 -	1944 -	-	-	1	1	2
1740	1739	1741 -	2162 -	-	+	1	1	2
1744	1744	1748 +	1837	1837	1838 +	1	1	2
1745	1745	1747 -	1884 -	-	-	1	1	2
1761 -	-	+	1780 -	-	-	1	1	2
1764	1759	1766 -	1913 -	-	-	1	1	2
1772 -	-	+	8131 -	-	+	0	2	2
1777	1777	1780 -	1936 -	-	-	1	1	2
1778	1778	1779 +	1848	1848	1849 -	1	1	2
1785	1785	1786 +	1765	1765	1766 -	1	1	2
1786	1785	1790 -	1912 -	-	-	1	1	2
1786	1785	1790 -	2014 -	-	-	1	1	2
1802 -	-	-	2000 -	-	-	1	1	2
1807	1804	1811 -	2010 -	-	-	1	1	2
1807	1804	1811 -	2070 -	-	-	0	2	2
1818	1813	1819 -	1939 -	-	-	1	1	2
1818	1813	1819 -	1964	1964	1966 -	1	1	2
1818	1813	1819 -	1986 -	-	-	1	1	2
1818	1813	1819 -	2007 -	-	-	1	1	2
1818	1813	1819 -	2013 -	-	-	1	1	2
1827 -	-	-	2003 -	-	-	1	1	2
1832	1830	1835 -	1988 -	-	-	1	1	2
1832	1830	1835 -	2074 -	-	-	0	2	2
1838 -	-	-	1864 -	-	+	1	1	2
1838 -	-	-	2011 -	-	-	1	1	2
1847	1844	1848 -	2013 -	-	-	1	1	2
1852	1849	1856 -	2107 -	-	-	1	1	2
1866 -	-	+	1836 -	-	-	1	1	2
1869	1865	1869 -	2077 -	-	-	1	1	2
1869	1865	1869 -	9588 -	-	-	1	1	2
1873 -	-	+	4057 -	-	+	1	1	2
1880	1876	1881 +	9734 -	-	+	2	0	2
1887	1883	1887 +	9735 -	-	+	0	2	2
1891	1887	1892 -	2062 -	-	-	1	1	2
1893	1893	1894 +	1790 -	-	-	1	1	2
1893	1893	1894 +	1857 -	-	+	1	1	2
1893	1893	1894 +	1904	1904	1905 -	1	1	2
1901	1901	1905 +	3315	3313	3315 -	2	0	2
1911	1908	1915 -	2242	2242	2243 -	1	1	2
1911	1908	1915 -	15364	15362	15364 -	1	1	2
1919	1918	1925 -	2035 -	-	-	1	1	2
1919	1918	1925 -	2107 -	-	-	1	1	2
1927	1927	1931 +	1960 -	-	+	1	1	2
1933	1929	1933 -	2083 -	-	-	1	1	2
1933	1929	1933 -	2092 -	-	-	1	1	2
1938	1936	1939 -	2078 -	-	-	1	1	2
1938	1936	1939 -	2094 -	-	-	1	1	2
1938 -	-	+	1952 -	-	+	1	1	2

1960	-	-	-	2591	-	-	-	1	1	2
1960	1960	1962	+	1937	-	-	-	1	1	2
1972	1968	1973	-	2010	-	-	+	1	1	2
1972	1968	1973	-	2103	-	-	-	1	1	2
1972	1968	1973	-	2135	-	-	+	1	1	2
1981	1977	1984	-	2002	-	-	+	1	1	2
1981	1977	1984	-	2134	2130	2134	+	1	1	2
1992	1992	1994	-	2243	-	-	+	1	1	2
2001	1997	2001	-	2109	-	-	-	1	1	2
2001	1997	2001	-	2124	-	-	+	1	1	2
2001	1997	2001	-	2158	-	-	-	1	1	2
2001	1997	2001	-	2211	-	-	-	1	1	2
2012	2010	2015	-	12618	12618	12619	-	1	1	2
2023	2023	2027	-	12506	12503	12506	-	0	2	2
2031	2030	2032	-	2067	-	-	+	2	0	2
2031	2030	2032	-	12510	12510	12511	-	0	2	2
2035	-	-	+	2792	-	-	-	1	1	2
2037	2035	2037	-	2066	2062	2066	+	0	2	2
2037	2035	2037	-	3023	3023	3024	-	1	1	2
2042	2039	2044	-	2137	-	-	-	1	1	2
2050	2048	2052	-	2301	-	-	-	1	1	2
2055	-	-	-	2147	-	-	-	1	1	2
2055	2053	2058	+	12540	12537	12540	+	2	0	2
2066	-	-	-	2171	-	-	-	1	1	2
2074	2074	2077	-	2111	2108	2111	+	1	1	2
2077	2076	2081	+	2190	2186	2190	-	1	1	2
2088	2086	2088	+	2114	-	-	+	1	1	2
2095	-	-	-	2200	-	-	-	1	1	2
2099	2098	2101	+	2177	-	-	-	1	1	2
2099	2098	2101	+	2204	-	-	-	1	1	2
2108	2108	2109	+	2167	-	-	-	1	1	2
2108	2108	2109	+	2519	-	-	+	1	1	2
2111	2111	2115	-	2150	-	-	-	1	1	2
2121	2121	2124	+	2144	-	-	-	1	1	2
2123	2123	2127	-	2275	-	-	-	1	1	2
2123	2123	2127	-	2356	-	-	-	1	1	2
2129	2129	2132	-	2175	-	-	-	1	1	2
2129	2129	2132	-	2298	2298	2299	-	1	1	2
2154	2151	2159	-	2400	-	-	-	1	1	2
2157	2154	2158	+	12493	-	-	+	1	1	2
2161	-	-	-	2286	-	-	-	1	1	2
2174	2171	2174	-	2337	2334	2337	-	1	1	2
2187	-	-	+	2200	-	-	+	1	1	2
2208	2206	2212	-	2404	-	-	-	1	1	2
2212	2209	2212	+	2188	-	-	-	1	1	2
2218	2218	2221	-	2394	-	-	-	1	1	2
2218	2218	2221	-	2443	-	-	-	1	1	2
2226	2224	2226	-	2390	2388	2390	-	1	1	2
2226	2224	2226	-	2402	-	-	-	1	1	2
2226	2224	2226	-	2412	-	-	-	1	1	2
2237	2237	2238	-	2211	2211	2212	+	1	1	2
2243	2243	2245	+	2284	2282	2284	+	1	1	2
2252	2248	2256	+	2213	-	-	-	1	1	2
2252	2248	2256	+	2221	-	-	-	1	1	2
2252	2248	2256	+	2875	-	-	+	1	1	2
2276	-	-	+	2238	-	-	-	1	1	2
2277	2277	2278	-	2332	-	-	-	1	1	2
2277	2277	2278	-	2685	-	-	-	1	1	2
2288	-	-	-	2443	-	-	+	1	1	2
2302	2302	2305	+	2294	2291	2294	-	1	1	2
2310	2310	2311	-	2504	-	-	-	1	1	2
2310	2310	2311	-	2834	-	-	-	1	1	2
2324	2322	2326	-	7231	-	-	-	0	2	2
2328	-	-	+	7238	-	-	+	0	2	2
2333	2333	2334	+	7243	7243	7244	+	1	1	2
2341	2341	2342	+	2351	2351	2352	-	1	1	2
2347	-	-	-	2615	-	-	-	2	0	2
2353	2350	2353	-	2621	-	-	-	0	2	2
2353	2350	2353	-	4550	-	-	-	1	1	2
2355	2355	2358	+	2329	-	-	-	1	1	2
2368	2365	2371	-	2443	-	-	-	1	1	2
2376	2374	2377	-	2502	-	-	-	1	1	2
2376	2374	2377	-	2512	-	-	-	1	1	2
2376	2374	2377	-	2517	-	-	-	1	1	2
2376	2374	2377	-	2528	-	-	-	1	1	2
2376	2374	2377	-	2552	-	-	-	1	1	2
2376	2374	2377	-	2731	2731	2732	-	1	1	2
2384	2381	2384	+	10151	-	-	+	2	0	2
2400	-	-	+	2536	-	-	-	1	1	2
2406	2406	2408	+	2529	2529	2530	-	1	1	2
2406	2406	2408	+	2976	-	-	+	1	1	2
2448	2445	2448	+	2475	-	-	-	1	1	2
2460	2460	2462	-	2478	-	-	+	0	2	2
2491	2489	2491	+	2414	-	-	-	1	1	2
2491	2489	2491	+	3044	-	-	+	1	1	2
2492	2489	2495	-	2590	-	-	+	1	1	2
2492	2489	2495	-	2602	-	-	-	1	1	2
2492	2489	2495	-	2634	2634	2635	-	1	1	2
2496	2496	2498	+	13993	13993	13994	-	1	1	2
2503	2498	2505	-	2545	2545	2546	+	1	1	2
2503	2498	2505	-	2906	2902	2906	+	1	1	2
2503	2502	2507	+	2484	-	-	-	1	1	2
2503	2502	2507	+	2517	2514	2517	-	1	1	2
2503	2502	2507	+	2522	-	-	-	1	1	2
2503	2502	2507	+	2581	2581	2582	-	1	1	2
2508	2508	2511	-	12082	-	-	-	2	0	2

2513	-	-	+	2504	-	-	-	1	1	2
2518	2515	2522	-	2506	2504	2506	+	2	0	2
2525	2525	2526	-	2503	-	-	+	2	0	2
2525	2525	2526	-	2644	-	-	+	0	2	2
2538	2533	2542	-	2567	2565	2567	+	1	1	2
2538	2533	2542	-	2596	-	-	+	1	1	2
2538	2533	2542	-	2611	-	-	+	1	1	2
2538	2533	2542	-	2617	-	-	+	1	1	2
2538	2533	2542	-	2652	-	-	-	1	1	2
2538	2533	2542	-	2744	-	-	-	1	1	2
2538	2533	2542	-	2753	-	-	-	1	1	2
2548	2545	2552	-	2601	2601	2602	+	1	1	2
2548	2545	2552	-	2650	-	-	-	1	1	2
2548	2545	2552	-	2765	-	-	-	1	1	2
2548	2545	2552	-	5473	-	-	+	2	0	2
2551	2551	2552	+	2707	2707	2708	+	1	1	2
2559	2556	2562	-	2664	-	-	+	1	1	2
2559	2556	2562	-	2669	2669	2670	-	1	1	2
2559	2556	2562	-	2726	-	-	+	1	1	2
2559	2556	2562	-	12082	-	-	-	2	0	2
2580	-	-	+	2624	-	-	-	1	1	2
2586	2586	2587	+	2497	2497	2498	-	1	1	2
2589	2587	2593	-	2716	2713	2716	+	1	1	2
2589	2587	2593	-	2783	-	-	-	1	1	2
2589	2587	2593	-	2789	-	-	-	1	1	2
2592	2590	2596	+	2485	-	-	-	1	1	2
2598	2597	2602	+	2486	2486	2487	-	1	1	2
2598	2597	2602	+	2583	2583	2584	-	1	1	2
2608	2607	2611	-	2630	-	-	+	2	0	2
2608	2607	2611	-	2751	-	-	-	1	1	2
2608	2607	2611	-	2905	-	-	-	2	0	2
2616	2613	2619	-	2627	2627	2628	+	0	2	2
2616	2613	2619	-	2730	-	-	-	1	1	2
2619	2619	2620	+	2640	-	-	+	1	1	2
2624	2623	2624	+	2580	-	-	-	1	1	2
2626	2623	2627	-	2761	-	-	-	1	1	2
2626	2623	2627	-	2784	-	-	-	1	1	2
2631	2631	2632	+	2557	2557	2558	-	1	1	2
2632	2629	2635	-	2764	-	-	-	1	1	2
2632	2629	2635	-	2797	-	-	-	1	1	2
2632	2629	2635	-	2821	-	-	-	1	1	2
2632	2629	2635	-	6161	-	-	-	1	1	2
2638	2638	2639	-	3072	3072	3073	-	1	1	2
2643	2641	2646	+	2512	-	-	-	2	0	2
2643	2641	2646	+	2517	-	-	-	0	2	2
2643	2641	2646	+	2526	-	-	-	1	1	2
2645	2641	2649	-	2689	-	-	+	0	2	2
2645	2641	2649	-	2793	-	-	-	1	1	2
2645	2641	2649	-	2923	2923	2924	-	1	1	2
2645	2641	2649	-	3185	-	-	-	1	1	2
2653	2652	2656	-	2779	-	-	-	1	1	2
2653	2652	2656	-	2805	-	-	-	1	1	2
2653	2652	2656	-	2820	-	-	-	1	1	2
2662	2660	2668	-	2708	2706	2708	+	1	1	2
2662	2660	2668	-	2826	-	-	-	1	1	2
2662	2660	2668	-	2843	-	-	-	1	1	2
2662	2660	2668	-	3001	-	-	+	1	1	2
2662	2660	2668	-	3071	-	-	+	1	1	2
2673	2670	2674	-	2708	2708	2709	+	1	1	2
2675	2671	2678	+	2627	-	-	-	1	1	2
2682	2682	2684	-	2696	-	-	+	1	1	2
2690	-	-	-	2812	-	-	-	1	1	2
2694	2693	2694	+	2645	-	-	-	1	1	2
2702	2699	2706	+	2667	-	-	-	1	1	2
2709	-	-	+	2672	-	-	-	0	2	2
2711	2708	2716	-	2849	-	-	-	1	1	2
2711	2708	2716	-	2933	-	-	-	1	1	2
2748	2745	2750	-	2776	2773	2776	+	1	1	2
2748	2745	2750	-	2916	-	-	-	1	1	2
2748	2745	2750	-	2937	-	-	-	1	1	2
2748	2745	2750	-	3014	-	-	-	1	1	2
2757	2755	2762	-	2880	-	-	+	1	1	2
2757	2755	2762	-	2997	-	-	-	1	1	2
2764	2764	2767	-	2769	-	-	+	2	0	2
2764	2764	2767	-	2984	2984	2985	-	1	1	2
2767	-	-	+	2778	-	-	-	1	1	2
2776	-	-	+	2745	-	-	-	1	1	2
2786	2784	2788	-	2955	-	-	-	1	1	2
2820	2819	2820	-	2951	-	-	+	1	1	2
2827	2825	2830	-	3042	-	-	-	1	1	2
2827	2825	2830	-	3050	-	-	-	1	1	2
2832	2832	2835	-	2931	-	-	-	1	1	2
2837	2836	2839	-	2993	-	-	-	1	1	2
2837	2836	2839	-	3021	-	-	-	0	2	2
2843	2843	2846	-	3352	3352	3353	-	1	1	2
2856	2853	2859	-	2885	-	-	+	1	1	2
2856	2853	2859	-	2994	2990	2994	-	1	1	2
2856	2853	2859	-	2999	2999	3003	-	1	1	2
2856	2853	2859	-	3150	-	-	-	1	1	2
2856	2853	2859	-	3200	-	-	-	1	1	2
2865	2862	2866	+	2918	-	-	-	1	1	2
2879	2878	2882	-	3061	-	-	-	1	1	2
2884	2883	2888	-	2958	-	-	+	1	1	2
2884	2883	2888	-	2987	-	-	-	1	1	2
2884	2883	2888	-	3106	-	-	-	1	1	2
2896	2895	2896	-	2951	-	-	-	1	1	2

2898	-	-	+	2892	-	-	-	1	1	2
2902	2900	2902	-	3057	-	-	-	1	1	2
2907	-	-	-	3129	-	-	-	1	1	2
2914	2910	2917	-	3051	-	-	-	1	1	2
2914	2910	2917	-	3082	3082	3083	-	1	1	2
2914	2910	2917	-	3129	-	-	-	1	1	2
2914	-	-	+	2895	-	-	-	1	1	2
2924	2920	2924	-	2982	-	-	+	1	1	2
2924	2920	2924	-	3070	-	-	-	1	1	2
2930	2928	2932	-	2974	-	-	+	1	1	2
2930	2928	2932	-	2987	2984	2987	+	1	1	2
2948	2944	2948	+	2921	-	-	-	0	2	2
2951	2949	2954	-	3013	-	-	+	1	1	2
2951	2949	2954	-	3131	-	-	-	1	1	2
2953	2950	2956	+	2879	2879	2880	-	1	1	2
2953	2950	2956	+	2919	-	-	-	2	0	2
2953	2950	2956	+	2982	-	-	+	0	2	2
2953	2950	2956	+	2987	-	-	+	2	0	2
2953	2950	2956	+	3175	-	-	-	1	1	2
2953	2950	2956	+	3514	3512	3514	+	1	1	2
2953	2950	2956	+	7455	-	-	-	1	1	2
2962	2961	2964	-	3010	3007	3010	+	2	0	2
2968	2967	2970	-	3003	-	-	+	0	2	2
2981	-	-	-	3132	-	-	-	1	1	2
2995	2995	2996	-	3056	-	-	+	1	1	2
2995	2995	2996	-	3128	3128	3129	-	1	1	2
3002	3002	3006	-	3135	-	-	-	1	1	2
3002	3002	3006	-	3142	-	-	-	1	1	2
3002	3002	3006	-	3162	-	-	-	1	1	2
3008	-	-	-	3070	-	-	-	1	1	2
3014	3010	3017	-	3044	-	-	+	1	1	2
3014	3010	3017	-	3198	-	-	-	1	1	2
3014	3010	3017	-	3203	-	-	-	1	1	2
3019	3018	3023	-	3115	-	-	-	2	0	2
3019	3018	3023	-	3120	-	-	-	0	2	2
3025	3025	3029	+	2931	2927	2931	-	1	1	2
3028	3028	3029	-	3202	3202	3203	-	1	1	2
3037	3037	3039	+	3097	3095	3097	-	1	1	2
3046	3044	3050	-	3065	3065	3066	+	1	1	2
3046	3044	3050	-	3090	-	-	+	1	1	2
3046	3044	3050	-	3237	-	-	-	1	1	2
3055	3051	3058	+	2990	2990	2991	+	1	1	2
3055	3051	3058	+	3007	-	-	-	1	1	2
3055	3051	3058	+	3077	-	-	-	0	2	2
3064	3062	3068	-	3098	-	-	+	1	1	2
3064	3062	3068	-	3223	-	-	-	1	1	2
3064	3062	3068	-	3256	-	-	-	1	1	2
3065	-	-	+	3098	-	-	-	1	1	2
3084	3084	3086	-	3175	3175	3176	-	1	1	2
3090	3086	3090	+	3229	-	-	+	1	1	2
3091	3089	3093	-	3213	-	-	-	1	1	2
3091	3089	3093	-	3234	-	-	-	1	1	2
3098	-	-	-	3308	-	-	-	1	1	2
3104	3104	3105	-	3242	3240	3242	-	1	1	2
3115	3115	3118	+	3687	-	-	+	1	1	2
3122	3122	3124	-	3177	-	-	+	1	1	2
3122	3122	3124	-	3299	-	-	-	1	1	2
3130	3126	3130	-	3308	-	-	-	1	1	2
3130	3126	3130	-	3336	-	-	-	1	1	2
3130	-	-	+	3182	-	-	-	1	1	2
3137	3136	3138	-	3316	-	-	-	1	1	2
3147	-	-	+	3121	-	-	-	1	1	2
3159	-	-	-	3300	-	-	-	1	1	2
3162	-	-	+	3110	-	-	-	1	1	2
3167	3163	3172	-	3368	3368	3369	-	1	1	2
3168	3167	3169	+	3075	-	-	-	1	1	2
3179	3179	3180	-	3292	3292	3293	+	1	1	2
3180	-	-	+	10418	-	-	+	1	1	2
3187	3184	3190	-	3276	3276	3277	+	1	1	2
3187	3184	3190	-	3352	-	-	-	1	1	2
3187	3184	3190	-	3402	3398	3402	-	1	1	2
3193	-	-	+	3319	-	-	-	2	0	2
3196	3191	3199	-	3308	-	-	-	1	1	2
3196	3191	3199	-	3371	-	-	-	1	1	2
3196	3191	3199	-	3378	-	-	-	1	1	2
3208	3206	3210	-	3333	-	-	-	2	0	2
3208	3206	3210	-	3372	-	-	-	1	1	2
3208	3206	3210	-	6317	-	-	-	1	1	2
3216	3212	3220	-	6523	-	-	-	1	1	2
3221	3221	3224	+	3466	-	-	+	1	1	2
3229	3229	3233	-	3364	3364	3365	-	1	1	2
3229	3229	3233	-	3385	-	-	-	1	1	2
3229	3229	3233	-	3409	-	-	-	1	1	2
3229	3229	3233	-	3468	-	-	-	1	1	2
3235	-	-	+	4489	-	-	+	2	0	2
3237	-	-	-	3496	-	-	-	1	1	2
3255	3255	3258	-	3360	-	-	-	1	1	2
3256	3256	3257	+	3263	3263	3264	-	1	1	2
3264	-	-	-	3409	-	-	-	1	1	2
3273	3270	3273	+	3247	-	-	-	1	1	2
3273	3270	3273	+	3412	-	-	-	1	1	2
3274	3270	3276	-	3247	-	-	+	2	0	2
3274	3270	3276	-	3345	-	-	+	1	1	2
3274	3270	3276	-	3406	-	-	-	1	1	2
3274	3270	3276	-	3494	-	-	-	1	1	2
3279	3275	3279	+	3601	3597	3601	+	1	1	2

3279		3275	3279	+	11951		11948	11951	+	1	1	2
3292		3291	3295	-	3407	-	-		-	1	1	2
3301		3301	3305	+	3385	-	-		-	1	1	2
3309		3308	3313	-	3367	-	-		-	1	1	2
3309		3308	3313	-	3490	-	-		-	1	1	2
3309		3308	3313	-	3563	-	-		-	1	1	2
3309		3308	3313	-	11262	-	-		+	2	0	2
3319	-	-		+	3193	-	-		-	2	0	2
3320		3318	3320	-	3391	-	-		+	1	1	2
3320		3318	3320	-	3419	-	-		-	1	1	2
3326		3326	3329	+	3360	-	-		-	1	1	2
3326		3326	3329	+	3377	-	-		+	1	1	2
3326		3326	3329	+	3398	-	-		+	1	1	2
3326		3326	3329	+	5695		5695	5696	+	1	1	2
3328		3328	3329	-	3361	-	-		+	2	0	2
3336		3333	3339	-	3361	-	-		+	1	1	2
3336		3333	3339	-	3395	-	-		+	1	1	2
3339	-	-		+	3647	-	-		-	1	1	2
3349		3348	3349	-	3578	-	-		-	1	1	2
3356		3356	3359	-	3461		3461	3462	+	1	1	2
3356		3356	3359	-	3581	-	-		-	1	1	2
3356		3356	3359	-	12423	-	-		+	2	0	2
3362		3362	3363	-	3456		3456	3457	+	1	1	2
3370		3370	3373	-	3422	-	-		-	1	1	2
3370		3370	3373	-	3453	-	-		-	1	1	2
3370		3370	3373	-	3593	-	-		-	1	1	2
3376		3373	3381	+	3621		3618	3621	+	1	1	2
3377		3375	3377	-	3586	-	-		-	1	1	2
3377		3375	3377	-	4335	-	-		-	1	1	2
3389		3387	3393	+	3370	-	-		-	1	1	2
3393		3389	3394	-	3572	-	-		-	1	1	2
3393		3389	3394	-	3616	-	-		-	1	1	2
3403		3398	3406	-	3568		3568	3569	-	1	1	2
3403		3398	3406	-	3606	-	-		+	1	1	2
3403		3398	3406	-	3636		3636	3638	-	1	1	2
3403		3398	3406	-	3662	-	-		-	1	1	2
3403		3398	3406	-	3678		3678	3679	-	1	1	2
3403		3398	3406	-	3707		3707	3711	-	1	1	2
3403		3398	3406	-	3749	-	-		-	1	1	2
3407		3407	3409	+	3333	-	-		-	1	1	2
3407		3407	3409	+	3452	-	-		-	1	1	2
3412		3408	3416	-	3450	-	-		+	1	1	2
3412		3408	3416	-	3580	-	-		-	1	1	2
3412		3408	3416	-	3598		3598	3599	+	1	1	2
3412		3408	3416	-	3603	-	-		+	1	1	2
3412		3412	3414	+	3406		3406	3407	-	1	1	2
3421		3419	3421	+	3590	-	-		-	1	1	2
3421		3419	3421	+	9071		9071	9072	+	1	1	2
3422		3419	3422	-	3710	-	-		-	1	1	2
3433		3430	3436	-	3587	-	-		-	1	1	2
3433		3430	3436	-	3857	-	-		-	1	1	2
3436		3433	3436	+	3681	-	-		+	1	1	2
3436		3433	3436	+	4755	-	-		+	1	1	2
3458		3457	3459	-	3498		3496	3498	+	1	1	2
3469		3466	3469	-	3489		3486	3489	+	1	1	2
3474		3471	3477	-	3519		3519	3520	+	1	1	2
3474		3471	3477	-	3585	-	-		+	1	1	2
3474		3471	3477	-	3987	-	-		-	1	1	2
3483		3479	3486	-	3595	-	-		-	1	1	2
3483		3479	3486	-	3636	-	-		-	1	1	2
3483		3479	3486	-	3701	-	-		-	1	1	2
3488	-	-		+	3569	-	-		-	1	1	2
3490		3488	3490	-	3622	-	-		-	1	1	2
3490		3488	3490	-	4183	-	-		-	1	1	2
3493		3493	3494	+	3524	-	-		-	1	1	2
3493		3493	3494	+	3565	-	-		+	1	1	2
3496		3494	3497	-	3522	-	-		+	1	1	2
3496		3494	3497	-	3734	-	-		-	0	2	2
3498		3496	3498	+	3625	-	-		-	2	0	2
3511		3511	3514	-	3699	-	-		-	1	1	2
3521		3516	3523	+	3459	-	-		-	1	1	2
3521		3516	3523	+	5438	-	-		+	1	1	2
3523	-	-		-	3674	-	-		-	1	1	2
3545		3542	3549	-	3612	-	-		+	1	1	2
3545		3542	3549	-	3712	-	-		-	1	1	2
3545		3542	3549	-	3856	-	-		+	1	1	2
3545	-	-		+	3620	-	-		+	1	1	2
3551		3550	3554	-	3731	-	-		-	1	1	2
3551		3550	3554	-	3742	-	-		-	1	1	2
3551		3550	3554	-	3770	-	-		-	1	1	2
3558	-	-		-	3658	-	-		+	1	1	2
3558	-	-		-	3678	-	-		-	1	1	2
3564		3560	3567	-	3713	-	-		-	1	1	2
3564		3560	3567	-	3732	-	-		-	1	1	2
3564		3560	3567	-	3852		3852	3853	-	1	1	2
3564		3560	3567	-	4304	-	-		-	1	1	2
3564		3560	3567	-	4911	-	-		+	1	1	2
3570	-	-		-	3648	-	-		+	1	1	2
3574	-	-		+	3645	-	-		-	1	1	2
3575	-	-		-	3661	-	-		-	1	1	2
3579		3576	3579	+	3585	-	-		+	1	1	2
3579		3576	3579	+	6792	-	-		+	1	1	2
3585		3582	3586	-	3697	-	-		-	1	1	2
3585		3582	3586	-	3707		3705	3707	-	1	1	2
3590	-	-		-	3737	-	-		+	1	1	2
3591		3591	3595	+	3736	-	-		-	1	1	2

3591	3591	3595 +	12429 -	-	+	2	0	2
3595	3592	3599 -	3670 -	-	+	1	1	2
3595	3592	3599 -	3716 -	-	-	1	1	2
3595	3592	3599 -	3745 -	-	-	1	1	2
3595	3592	3599 -	5112 -	-	-	1	1	2
3599	3599	3602 +	6786 -	-	-	1	1	2
3606	3602	3611 -	3814 -	-	-	1	1	2
3606	3602	3611 -	5430 -	-	-	1	1	2
3629	3626	3632 +	3494 -	-	-	2	0	2
3629	3626	3632 +	3682 -	-	+	1	1	2
3629	3626	3632 +	3838	3838	3839 -	1	1	2
3635 -	-	-	3694 -	-	+	2	0	2
3644	3640	3645 -	3689 -	-	+	0	2	2
3644	3640	3645 -	3987 -	-	-	1	1	2
3654	3650	3654 -	3706	3703	3706 +	1	1	2
3660	3657	3664 -	3711 -	-	+	1	1	2
3660	3657	3664 -	3722 -	-	+	1	1	2
3660	3657	3664 -	3827 -	-	-	1	1	2
3660	3657	3664 -	3830 -	-	+	1	1	2
3660	3657	3664 -	3835	3833	3835 -	1	1	2
3666	3666	3669 -	3707	3704	3707 +	1	1	2
3668	3668	3669 +	3603 -	-	-	1	1	2
3668	3668	3669 +	14472 -	-	-	0	2	2
3674	3671	3678 +	3694 -	-	-	1	1	2
3676	3676	3678 -	3811 -	-	-	1	1	2
3676	3676	3678 -	3833 -	-	-	1	1	2
3681 -	-	-	10396 -	-	+	1	1	2
3693	3688	3693 +	3704	3700	3704 +	1	1	2
3693	3688	3693 +	3728 -	-	+	1	1	2
3693	3688	3693 +	3744	3744	3745 -	1	1	2
3693	3688	3693 +	7345 -	-	-	0	2	2
3696	3696	3700 -	4401 -	-	-	1	1	2
3701	3698	3703 +	3660 -	-	-	1	1	2
3712	3709	3716 -	3858 -	-	-	1	1	2
3715	3712	3715 +	3665 -	-	-	1	1	2
3715	3712	3715 +	3692 -	-	-	1	1	2
3715	3712	3715 +	7323	7323	7324 -	0	2	2
3722	3718	3726 -	3867 -	-	-	1	1	2
3722	3718	3726 -	3899 -	-	-	1	1	2
3723 -	-	+	3699 -	-	-	1	1	2
3734 -	-	+	11238 -	-	-	1	1	2
3741	3741	3747 +	3673 -	-	-	0	2	2
3741	3741	3747 +	3910 -	-	+	1	1	2
3741	3741	3747 +	4039 -	-	-	1	1	2
3743 -	-	-	3821 -	-	+	1	1	2
3748	3746	3748 -	3883 -	-	+	1	1	2
3748	3746	3748 -	3987 -	-	-	1	1	2
3753	3749	3753 +	3665 -	-	-	2	0	2
3753	3749	3753 +	3863	3863	3864 +	1	1	2
3766	3766	3767 +	3878	3878	3879 -	1	1	2
3774	3773	3774 -	3808 -	-	-	1	1	2
3778	3775	3778 +	3868 -	-	-	1	1	2
3790	3786	3790 +	3720 -	-	-	1	1	2
3790	3786	3790 +	3819	3819	3820 +	1	1	2
3790	3786	3790 +	3864 -	-	+	1	1	2
3791	3788	3793 -	3858	3855	3858 +	1	1	2
3791	3788	3793 -	3956 -	-	-	1	1	2
3801	3801	3805 -	3975 -	-	-	1	1	2
3801	3801	3805 -	4023 -	-	-	1	1	2
3810	3809	3811 +	3881	3881	3882 -	1	1	2
3811	3807	3812 -	3951 -	-	-	1	1	2
3811	3807	3812 -	4667 -	-	-	1	1	2
3820	3819	3820 +	3837 -	-	+	1	1	2
3820	3819	3820 +	4104 -	-	+	1	1	2
3821	3819	3822 -	3894 -	-	+	1	1	2
3821	3819	3822 -	4264 -	-	-	1	1	2
3821	3819	3822 -	4665	4665	4666 -	1	1	2
3830	3830	3832 -	3971 -	-	-	1	1	2
3839	3839	3843 +	3915 -	-	-	1	1	2
3841 -	-	-	4006 -	-	+	1	1	2
3849	3844	3849 -	3855	3855	3856 +	1	1	2
3849	3844	3849 -	3995 -	-	-	1	1	2
3849	3847	3853 +	3699 -	-	-	1	1	2
3849	3847	3853 +	11009 -	-	+	1	1	2
3855	3855	3856 -	4005 -	-	-	1	1	2
3855	3854	3855 +	8187 -	-	-	0	2	2
3863	3861	3863 -	3924 -	-	-	1	1	2
3863	3861	3863 -	4604 -	-	-	1	1	2
3863	3863	3864 +	3924 -	-	+	1	1	2
3868	3865	3868 -	4081 -	-	-	1	1	2
3873	3873	3875 -	3955 -	-	+	1	1	2
3877	3873	3877 +	3992	3988	3992 -	1	1	2
3884	3884	3885 -	4035	4035	4036 -	1	1	2
3885	3881	3885 +	4012	4008	4012 -	1	1	2
3891	3891	3895 +	3932	3932	3933 +	1	1	2
3891	3891	3895 +	4025	4025	4026 -	1	1	2
3891	3891	3895 +	4042 -	-	+	1	1	2
3895	3895	3899 -	3935 -	-	+	1	1	2
3895	3895	3899 -	4081 -	-	-	1	1	2
3904	3904	3905 +	3935 -	-	+	1	1	2
3904	3904	3905 +	4582 -	-	+	1	1	2
3906	3901	3906 -	3928 -	-	+	1	1	2
3906	3901	3906 -	4039 -	-	-	1	1	2
3911	3909	3911 +	4140	4140	4141 -	1	1	2
3915	3915	3919 -	3949 -	-	+	1	1	2
3915	3915	3919 -	4114 -	-	-	1	1	2

3921	3921	3923 -	4107	4107	4108 -	1	1	2
3927	3926	3931 -	4085 -	-	-	1	1	2
3927	3926	3931 -	4090 -	-	-	1	1	2
3927	3926	3931 -	4206 -	-	-	1	1	2
3933	3932	3936 -	4276 -	-	-	1	1	2
3933	3932	3936 -	4295	4295	4296 -	1	1	2
3933	3932	3936 -	5321 -	-	-	1	1	2
3934	3931	3938 +	3962 -	-	-	1	1	2
3934	3931	3938 +	4120 -	-	-	1	1	2
3934	3931	3938 +	4411 -	-	+	1	1	2
3939	3939	3943 -	4113	4113	4114 +	1	1	2
3939	3939	3943 -	4116 -	-	-	1	1	2
3949	3948	3949 -	4131 -	-	-	1	1	2
3954	3951	3958 -	3981	3978	3981 +	1	1	2
3954	3951	3958 -	4154 -	-	-	1	1	2
3962	3960	3965 -	3989 -	-	+	1	1	2
3962	3960	3965 -	4240 -	-	+	1	1	2
3962 -	-	+	3883 -	-	+	1	1	2
3962 -	-	+	3901 -	-	+	1	1	2
3969	3969	3972 +	3936 -	-	+	1	1	2
3975	3972	3978 -	4094 -	-	+	1	1	2
3980	3980	3982 -	4257	4255	4257 +	1	1	2
3980	3976	3981 +	4090 -	-	-	1	1	2
3992	3992	3994 -	4258 -	-	-	1	1	2
3993 -	-	+	4144 -	-	+	1	1	2
4001 -	-	-	4142 -	-	-	1	1	2
4007	4007	4011 +	4046	4046	4047 -	1	1	2
4007	4007	4011 +	4054	4052	4054 +	1	1	2
4007	4007	4011 +	4102	4102	4103 +	1	1	2
4014	4010	4018 -	4125 -	-	-	1	1	2
4021	4021	4025 -	4149 -	-	+	2	0	2
4021	4021	4025 -	4160 -	-	-	1	1	2
4029	4029	4030 +	4102 -	-	+	1	1	2
4030	4030	4034 -	4139	4139	4140 +	1	1	2
4030	4030	4034 -	4144 -	-	+	0	2	2
4041	4038	4043 -	4081 -	-	+	1	1	2
4041	4038	4043 -	4402	4400	4402 -	1	1	2
4050	4048	4050 -	4073 -	-	+	1	1	2
4050	4048	4050 -	4134 -	-	+	1	1	2
4057 -	-	+	4126 -	-	-	1	1	2
4067	4066	4067 -	4171 -	-	-	1	1	2
4072	4071	4072 -	4062 -	-	+	2	0	2
4072	4071	4072 -	4201 -	-	-	1	1	2
4075	4072	4075 +	4108 -	-	-	2	0	2
4075	4072	4075 +	4129 -	-	+	1	1	2
4079	4078	4079 -	4262 -	-	-	1	1	2
4081	4077	4082 +	3980	3980	3981 -	1	1	2
4081	4077	4082 +	4107 -	-	-	0	2	2
4089	4086	4094 -	4238 -	-	-	1	1	2
4089	4086	4094 -	4257	4257	4258 -	1	1	2
4089	4086	4094 -	4297 -	-	-	1	1	2
4094	4092	4097 +	4163 -	-	-	1	1	2
4103	4103	4104 +	4144	4144	4145 +	1	1	2
4106	4104	4108 -	4270 -	-	-	1	1	2
4106	4104	4108 -	5047 -	-	+	1	1	2
4110 -	-	+	4240 -	-	-	1	1	2
4111	4111	4115 -	4245 -	-	-	1	1	2
4111	4111	4115 -	4258 -	-	-	1	1	2
4131 -	-	-	4308 -	-	-	1	1	2
4139	4135	4141 +	11485 -	-	+	2	0	2
4150	4146	4154 +	4212 -	-	+	1	1	2
4155	4155	4156 -	4181 -	-	+	1	1	2
4162	4158	4166 -	4367 -	-	-	2	0	2
4166	4162	4166 +	4209 -	-	-	1	1	2
4166	4162	4166 +	4266	4266	4267 -	1	1	2
4170	4169	4171 -	4202 -	-	+	1	1	2
4170	4169	4171 -	4373 -	-	-	0	2	2
4171	4170	4173 +	4063 -	-	-	1	1	2
4179	4175	4181 -	4215	4215	4216 +	1	1	2
4179	4178	4179 +	4257	4257	4258 -	1	1	2
4200 -	-	+	4173 -	-	-	1	1	2
4201	4201	4203 -	4219 -	-	+	2	0	2
4201	4201	4203 -	4304 -	-	+	1	1	2
4206	4206	4207 -	4224 -	-	+	1	1	2
4206	4206	4207 -	4401 -	-	-	1	1	2
4209	4206	4211 +	4194 -	-	-	1	1	2
4209	4206	4211 +	4224 -	-	-	1	1	2
4209	4206	4211 +	12082 -	-	-	2	0	2
4213	4209	4214 -	4401	4401	4402 -	1	1	2
4213	4209	4214 -	4410 -	-	-	1	1	2
4217	4217	4221 +	4197	4193	4197 -	1	1	2
4219	4217	4224 -	4202 -	-	+	2	0	2
4219	4217	4224 -	4345 -	-	-	1	1	2
4219	4217	4224 -	4369 -	-	-	1	1	2
4219	4217	4224 -	4386 -	-	-	1	1	2
4219	4217	4224 -	4405 -	-	-	1	1	2
4219	4217	4224 -	4449	4449	4452 -	1	1	2
4219	4217	4224 -	4735 -	-	-	1	1	2
4232	4231	4236 +	4140	4140	4141 -	1	1	2
4232	4231	4236 +	4278 -	-	-	1	1	2
4232	4231	4236 +	4365 -	-	+	1	1	2
4232	4231	4236 +	4402	4402	4403 -	1	1	2
4235	4233	4239 -	4256	4254	4256 +	1	1	2
4235	4233	4239 -	4333 -	-	-	1	1	2
4235	4233	4239 -	4381 -	-	-	1	1	2
4235	4233	4239 -	4412 -	-	-	1	1	2

4241	4241	4242 -	4373 -	-	-	1	1	2
4251	4251	4255 -	4388 -	-	-	1	1	2
4251	4249	4251 +	4354 -	-	-	0	2	2
4251	4249	4251 +	6046	6046	6047 +	1	1	2
4257	4257	4258 +	4348 -	-	-	2	0	2
4265	4262	4267 +	4336	4334	4336 +	1	1	2
4270	4269	4270 -	4416 -	-	+	1	1	2
4284	4284	4286 -	4274 -	-	+	2	0	2
4289	4288	4292 -	14540 -	-	+	1	1	2
4292	4290	4292 +	4269	4269	4270 -	1	1	2
4300	4300	4302 -	4353 -	-	+	1	1	2
4308	4308	4311 -	4437 -	-	-	1	1	2
4308	4308	4311 -	4482 -	-	-	1	1	2
4318	4318	4321 -	4476 -	-	-	1	1	2
4318	4318	4321 -	4507 -	-	+	1	1	2
4329	4325	4329 -	4351 -	-	+	1	1	2
4338 -	-	+	4388 -	-	+	1	1	2
4342	4342	4348 -	4501	4501	4502 -	1	1	2
4353	4353	4357 -	4487 -	-	-	1	1	2
4370	4370	4374 +	4391 -	-	+	1	1	2
4370	4370	4374 +	4476 -	-	-	1	1	2
4381 -	-	+	4425 -	-	+	1	1	2
4383	4379	4383 -	11309 -	-	-	2	0	2
4393	4393	4399 -	4445	4443	4445 +	1	1	2
4393	4393	4399 -	4782	4782	4783 -	1	1	2
4400 -	-	+	4362 -	-	-	1	1	2
4405	4405	4406 +	4456	4456	4457 -	1	1	2
4410 -	-	-	4617 -	-	-	1	1	2
4418	4414	4422 +	4403 -	-	-	2	0	2
4437	4437	4439 -	4648 -	-	-	1	1	2
4437	4437	4439 -	13452 -	-	+	0	2	2
4443	4443	4444 -	4682 -	-	-	1	1	2
4458	4458	4460 +	4552 -	-	+	1	1	2
4467 -	-	-	4721 -	-	-	0	2	2
4475	4474	4479 -	4484 -	-	-	2	0	2
4475	4474	4479 -	4567 -	-	+	1	1	2
4483	4481	4485 +	4473	4469	4473 -	1	1	2
4483	4481	4485 +	4475 -	-	+	2	0	2
4501	4497	4505 -	4529 -	-	+	1	1	2
4501	4497	4505 -	4534 -	-	+	1	1	2
4501	4497	4505 -	4682 -	-	-	1	1	2
4501	4497	4505 -	4689	4687	4689 -	1	1	2
4505	4504	4505 +	4489 -	-	-	1	1	2
4513	4512	4513 -	4555	4555	4556 -	1	1	2
4520	4517	4522 -	4590 -	-	-	1	1	2
4520	4517	4522 -	4675 -	-	+	1	1	2
4520	4517	4522 -	4685 -	-	-	1	1	2
4525 -	-	-	4644 -	-	-	1	1	2
4530	4526	4531 +	4405 -	-	-	1	1	2
4530	4526	4531 +	7563 -	-	+	0	2	2
4542	4542	4543 -	4686 -	-	-	1	1	2
4542	4542	4543 -	4722 -	-	-	1	1	2
4544 -	-	+	9369 -	-	+	1	1	2
4559	4555	4560 -	4619 -	-	+	1	1	2
4559	4555	4560 -	4647 -	-	+	1	1	2
4559	4555	4560 -	4723 -	-	-	1	1	2
4559	4555	4560 -	4770	4770	4771 -	1	1	2
4566	4562	4566 +	4644 -	-	-	0	2	2
4566	4562	4566 +	4660 -	-	-	1	1	2
4582	4578	4586 -	4627	4625	4627 +	0	2	2
4582	4578	4586 -	4969 -	-	+	0	2	2
4582	4578	4586 -	13657 -	-	-	2	0	2
4586	4585	4586 +	13657 -	-	+	1	1	2
4591	4590	4593 -	4638 -	-	+	1	1	2
4591	4590	4593 -	4721 -	-	-	1	1	2
4591	4590	4593 -	4770 -	-	-	1	1	2
4609	4609	4610 +	4972 -	-	+	1	1	2
4609	4609	4610 +	5059 -	-	+	1	1	2
4620 -	-	-	4786 -	-	+	1	1	2
4641	4636	4642 +	4844	4844	4845 -	1	1	2
4650	4646	4651 -	5013 -	-	-	1	1	2
4650	4650	4653 +	4770 -	-	-	1	1	2
4650	4650	4653 +	4978 -	-	+	0	2	2
4660	4659	4664 +	4681	4681	4682 -	1	1	2
4660	4659	4664 +	4744 -	-	+	1	1	2
4660	4659	4664 +	4988 -	-	+	2	0	2
4662	4658	4667 -	4749 -	-	+	1	1	2
4662	4658	4667 -	4769 -	-	-	1	1	2
4662	4658	4667 -	4815 -	-	-	1	1	2
4662	4658	4667 -	4990 -	-	-	1	1	2
4666 -	-	+	4810 -	-	-	1	1	2
4678	4678	4679 +	4697 -	-	+	1	1	2
4678	4678	4679 +	5365 -	-	-	1	1	2
4678	4678	4679 +	6004 -	-	+	1	1	2
4683	4682	4683 +	4701	4701	4702 +	1	1	2
4684 -	-	-	4784 -	-	-	1	1	2
4693	4691	4693 +	5122 -	-	+	1	1	2
4695 -	-	-	4832 -	-	-	2	0	2
4701 -	-	-	4838 -	-	-	0	2	2
4706 -	-	-	4852 -	-	-	1	1	2
4706 -	-	-	5295 -	-	-	1	1	2
4722	4719	4723 +	4738 -	-	-	1	1	2
4722	4719	4723 +	4749 -	-	-	1	1	2
4739	4735	4741 +	4660 -	-	-	1	1	2
4739	4735	4741 +	4681 -	-	+	1	1	2
4739	4735	4741 +	4761 -	-	+	1	1	2

4750	4749	4753	+	4666	-	-	-	1	1	2
4750	4749	4753	+	4782	-	-	+	1	1	2
4758	4755	4761	-	5005	5005	5006	+	1	1	2
4777	4775	4777	+	4629	-	-	-	1	1	2
4785	4784	4785	+	4659	-	-	-	1	1	2
4785	4784	4785	+	4937	-	-	-	1	1	2
4785	4784	4785	+	5015	-	-	-	1	1	2
4790	-	-	+	4894	-	-	+	1	1	2
4795	4791	4795	-	4949	-	-	-	1	1	2
4795	4791	4795	-	9790	9790	9791	+	1	1	2
4804	4804	4808	-	4982	-	-	-	1	1	2
4813	4811	4817	-	5113	5111	5113	-	1	1	2
4813	4811	4817	-	5122	5122	5123	-	1	1	2
4815	4812	4815	+	5017	-	-	-	1	1	2
4815	4812	4815	+	5654	5654	5655	+	1	1	2
4823	4822	4823	-	4866	-	-	-	1	1	2
4827	4827	4829	+	5022	-	-	-	1	1	2
4830	4830	4832	-	4981	-	-	-	1	1	2
4830	4830	4832	-	5003	-	-	+	0	2	2
4844	-	-	-	5133	-	-	-	1	1	2
4846	-	-	+	5033	-	-	+	1	1	2
4857	4855	4859	+	4867	-	-	-	1	1	2
4857	4855	4859	+	4877	-	-	-	0	2	2
4857	4855	4859	+	4961	-	-	-	1	1	2
4861	4857	4861	-	4914	4911	4914	+	1	1	2
4861	4857	4861	-	5825	-	-	-	1	1	2
4864	-	-	+	4790	-	-	-	1	1	2
4871	-	-	+	4789	-	-	-	1	1	2
4877	4875	4881	-	4897	-	-	+	0	2	2
4877	4875	4881	-	4902	-	-	+	2	0	2
4877	4873	4878	+	4943	4943	4944	-	1	1	2
4884	4884	4888	+	5016	-	-	-	1	1	2
4885	4885	4886	-	5027	-	-	-	1	1	2
4891	-	-	+	4927	-	-	+	1	1	2
4903	4903	4904	-	4945	-	-	+	1	1	2
4903	4903	4904	-	5703	-	-	-	1	1	2
4913	4909	4914	-	4992	-	-	+	0	2	2
4913	4909	4914	-	4997	-	-	+	2	0	2
4913	4909	4914	-	5072	-	-	-	1	1	2
4919	4919	4920	+	5240	5240	5241	-	1	1	2
4926	4923	4929	-	5077	-	-	-	1	1	2
4926	4923	4929	-	5251	-	-	-	1	1	2
4927	4925	4929	+	4985	4983	4985	-	1	1	2
4927	4925	4929	+	5038	-	-	+	1	1	2
4932	4932	4933	-	5074	-	-	-	1	1	2
4932	4932	4933	-	5082	-	-	-	1	1	2
4939	4935	4940	+	5046	5046	5047	+	1	1	2
4940	4935	4944	-	4953	-	-	+	2	0	2
4945	4943	4949	+	4871	4871	4872	-	1	1	2
4945	4943	4949	+	5296	5296	5297	-	1	1	2
4953	-	-	+	5070	-	-	-	1	1	2
4955	4952	4957	-	5854	-	-	-	1	1	2
4955	4952	4957	-	8625	8625	8626	-	1	1	2
4965	4962	4966	+	5279	5279	5280	-	1	1	2
4979	4976	4982	-	5034	-	-	+	1	1	2
4979	4976	4982	-	5126	-	-	-	1	1	2
4984	4980	4988	+	5008	-	-	+	1	1	2
4990	4985	4991	-	5216	-	-	-	1	1	2
5000	5000	5004	-	5011	-	-	+	1	1	2
5000	5000	5004	-	5154	-	-	-	1	1	2
5005	-	-	+	4950	-	-	+	1	1	2
5009	5008	5015	-	5213	-	-	-	1	1	2
5019	5019	5020	-	5029	-	-	+	1	1	2
5019	5019	5020	-	5171	-	-	-	1	1	2
5022	5021	5022	+	5144	-	-	+	1	1	2
5030	5026	5033	-	5150	-	-	-	1	1	2
5030	5026	5033	-	5246	-	-	-	1	1	2
5036	5036	5038	+	8641	-	-	+	1	1	2
5039	5034	5039	-	5149	-	-	-	1	1	2
5039	5034	5039	-	5173	-	-	-	1	1	2
5039	5034	5039	-	5200	-	-	-	1	1	2
5039	5034	5039	-	5229	-	-	-	1	1	2
5039	5034	5039	-	6969	-	-	-	2	0	2
5046	5044	5049	-	5228	-	-	+	1	1	2
5049	5047	5049	+	5031	-	-	-	1	1	2
5053	5053	5055	-	5212	-	-	-	0	2	2
5056	5052	5056	+	5036	-	-	+	1	1	2
5065	5062	5067	-	5440	5440	5441	-	1	1	2
5065	5065	5066	+	15096	15096	15097	-	0	2	2
5070	5069	5074	+	5239	5235	5239	+	1	1	2
5070	5069	5074	+	15092	15092	15093	-	0	2	2
5078	5076	5082	-	5217	-	-	-	1	1	2
5078	5076	5082	-	5222	-	-	-	1	1	2
5084	5084	5085	-	15077	-	-	+	2	0	2
5095	5095	5096	-	5243	-	-	-	1	1	2
5095	5095	5096	-	5280	-	-	-	1	1	2
5095	5095	5096	-	5316	-	-	-	1	1	2
5095	5095	5096	-	15065	-	-	+	0	2	2
5100	5099	5102	+	5059	-	-	+	1	1	2
5101	5099	5104	-	5224	-	-	-	1	1	2
5101	5099	5104	-	5447	-	-	+	1	1	2
5101	5099	5104	-	5473	-	-	+	2	0	2
5110	-	-	+	5041	-	-	-	1	1	2
5112	5108	5112	-	5262	-	-	-	1	1	2
5112	5108	5112	-	5300	-	-	-	1	1	2
5112	5108	5112	-	5321	5319	5321	-	1	1	2

5112		5108	5112	-	5467	-	-		+	0	2	2
5119		5118	5122	-	5247		5247	5248	+	1	1	2
5119		5118	5122	-	5316	-	-		-	1	1	2
5119		5118	5122	-	5520		5520	5521	+	1	1	2
5124	-	-		-	5303	-	-		-	1	1	2
5131		5130	5131	-	5286	-	-		-	1	1	2
5131		5130	5131	-	5506		5506	5507	+	1	1	2
5137		5134	5139	+	5104	-	-		-	1	1	2
5137		5134	5139	+	5241		5241	5242	-	1	1	2
5148		5147	5152	-	5259	-	-		-	1	1	2
5148		5147	5152	-	5636		5636	5637	-	1	1	2
5160		5159	5161	-	5238	-	-		+	1	1	2
5160		5159	5161	-	5347	-	-		-	1	1	2
5169		5166	5171	-	5284		5284	5285	-	1	1	2
5169		5166	5171	-	5305	-	-		-	1	1	2
5169		5166	5171	-	5339	-	-		-	1	1	2
5172	-	-		+	5102	-	-		-	1	1	2
5178		5176	5178	-	5603	-	-		-	1	1	2
5179		5176	5182	+	5277	-	-		+	1	1	2
5187		5187	5189	-	5247	-	-		+	1	1	2
5187		5187	5189	-	5351	-	-		-	1	1	2
5190	-	-		+	5419	-	-		+	1	1	2
5202	-	-		+	5266	-	-		+	1	1	2
5206		5202	5206	-	5338	-	-		-	1	1	2
5212		5211	5213	+	5107	-	-		-	1	1	2
5212		5211	5213	+	5284	-	-		-	1	1	2
5224	-	-		-	5261	-	-		+	1	1	2
5225		5225	5228	+	15084	-	-		-	1	1	2
5230		5229	5232	+	5254	-	-		-	2	0	2
5235		5233	5236	-	5271		5268	5271	+	1	1	2
5239		5236	5241	+	5252	-	-		-	1	1	2
5239		5236	5241	+	11272	-	-		-	1	1	2
5242	-	-		-	5568	-	-		-	1	1	2
5247	-	-		+	15083	-	-		-	1	1	2
5248		5244	5249	-	5429	-	-		-	1	1	2
5248		5244	5249	-	5449	-	-		-	1	1	2
5248		5244	5249	-	5468		5466	5468	-	1	1	2
5248		5244	5249	-	5594	-	-		-	2	0	2
5254		5251	5254	+	5167		5167	5168	-	1	1	2
5261		5259	5264	-	5329	-	-		-	1	1	2
5261		5259	5264	-	5356	-	-		+	1	1	2
5261		5259	5264	-	5651		5649	5651	-	1	1	2
5261		5259	5264	-	5808	-	-		-	1	1	2
5264		5263	5266	+	5352		5352	5353	-	1	1	2
5264		5263	5266	+	5550	-	-		+	1	1	2
5266		5265	5266	-	5416	-	-		-	1	1	2
5266		5265	5266	-	5524	-	-		-	1	1	2
5266		5265	5266	-	5540	-	-		-	1	1	2
5275	-	-		+	5291	-	-		-	1	1	2
5277		5273	5279	-	5509	-	-		-	1	1	2
5277		5273	5279	-	5603	-	-		-	1	1	2
5282		5282	5283	-	5330		5330	5331	-	1	1	2
5291		5291	5293	-	5340	-	-		-	1	1	2
5296		5296	5300	+	5270	-	-		-	1	1	2
5296		5296	5300	+	5498		5498	5499	+	1	1	2
5302		5302	5303	+	5408		5408	5409	-	1	1	2
5307	-	-		-	5492	-	-		-	2	0	2
5311		5307	5314	+	5421	-	-		-	1	1	2
5311		5307	5314	+	5743	-	-		-	1	1	2
5317		5317	5321	+	10730	-	-		-	1	1	2
5323		5322	5325	+	5433		5431	5433	+	1	1	2
5323		5322	5325	+	5486	-	-		-	1	1	2
5328		5328	5331	+	5358	-	-		-	2	0	2
5336		5336	5338	-	5487	-	-		-	1	1	2
5336		5336	5338	-	5496	-	-		-	1	1	2
5344		5342	5347	-	5491	-	-		-	1	1	2
5344		5342	5347	-	5506	-	-		-	1	1	2
5344		5344	5349	+	5268	-	-		-	1	1	2
5344		5344	5349	+	5398	-	-		-	1	1	2
5362		5362	5366	-	5432		5428	5432	+	1	1	2
5372		5371	5372	-	5530	-	-		-	1	1	2
5378		5378	5381	-	5477	-	-		+	1	1	2
5378		5378	5381	-	5626	-	-		-	1	1	2
5381		5380	5381	+	5438	-	-		+	1	1	2
5397		5397	5398	+	5333	-	-		-	1	1	2
5405		5404	5408	+	5353	-	-		+	1	1	2
5405		5404	5408	+	5381	-	-		-	1	1	2
5405		5404	5408	+	15039	-	-		-	2	0	2
5407		5404	5407	-	5386	-	-		+	2	0	2
5414		5411	5418	+	5339		5336	5339	-	1	1	2
5414		5411	5418	+	5397		5397	5398	-	1	1	2
5414		5411	5418	+	5452	-	-		+	1	1	2
5420		5418	5424	-	5578		5576	5578	-	1	1	2
5430		5429	5431	+	5656	-	-		+	1	1	2
5443		5440	5447	-	5397	-	-		+	1	1	2
5443		5440	5447	-	5557	-	-		+	1	1	2
5443		5440	5447	-	5597		5594	5597	-	1	1	2
5443		5440	5447	-	12423	-	-		+	2	0	2
5452		5449	5453	-	5678	-	-		-	1	1	2
5459		5459	5461	-	5625		5623	5625	-	1	1	2
5463		5460	5463	+	5385		5385	5386	-	1	1	2
5463		5460	5463	+	5390	-	-		-	1	1	2
5463		5460	5463	+	5544		5544	5545	-	1	1	2
5467		5462	5469	-	5531	-	-		-	1	1	2
5467		5462	5469	-	5664	-	-		-	1	1	2
5476		5472	5480	-	5624	-	-		-	1	1	2

5476		5472	5480	-	5639	-	-		-	1	1	2
5476		5472	5480	-	5666	-	-		-	1	1	2
5476		5472	5480	-	6282		6278	6282	+	1	1	2
5476		5472	5480	-	8548	-	-		-	0	2	2
5476		5472	5480	-	9575	-	-		+	0	2	2
5476		5472	5480	-	9726		9726	9730	+	0	2	2
5476		5472	5480	-	11175		11175	11179	+	0	2	2
5476		5472	5480	-	14221	-	-		-	0	2	2
5489		5488	5490	-	5670		5670	5671	-	1	1	2
5489		5488	5490	-	5679	-	-		-	1	1	2
5494		5491	5494	+	5513	-	-		-	1	1	2
5494		5491	5494	+	5517	-	-		+	1	1	2
5498		5494	5498	-	5590	-	-		-	1	1	2
5498		5494	5498	-	5662	-	-		-	1	1	2
5498		5494	5498	-	6048	-	-		-	1	1	2
5503		5501	5505	-	5659	-	-		-	1	1	2
5503		5501	5505	-	5664	-	-		-	1	1	2
5506		5505	5510	+	6071	-	-		+	2	0	2
5512	-	-		+	6072	-	-		+	0	2	2
5529		5526	5534	-	5539	-	-		+	2	0	2
5529		5526	5534	-	5670		5668	5670	-	1	1	2
5529		5526	5534	-	5687	-	-		-	1	1	2
5529		5526	5534	-	5741	-	-		-	1	1	2
5545		5544	5548	-	5638	-	-		-	1	1	2
5563		5563	5566	+	5573	-	-		-	1	1	2
5563		5563	5566	+	5591		5588	5591	-	1	1	2
5565	-	-		-	5901	-	-		-	1	1	2
5570		5570	5573	-	5823	-	-		+	1	1	2
5570		5570	5573	-	6808	-	-		-	1	1	2
5587		5585	5587	+	5642		5640	5642	+	1	1	2
5587		5585	5587	+	6051	-	-		-	1	1	2
5612	-	-		+	5630	-	-		-	1	1	2
5617	-	-		+	5719	-	-		+	1	1	2
5619		5615	5619	-	5768	-	-		-	1	1	2
5645		5644	5645	-	5757	-	-		-	1	1	2
5645		5644	5648	+	5611		5607	5611	-	1	1	2
5645		5644	5648	+	5621	-	-		-	1	1	2
5645		5644	5648	+	5883		5883	5884	-	1	1	2
5650	-	-		-	5788	-	-		-	1	1	2
5654	-	-		+	5578	-	-		-	1	1	2
5656	-	-		-	5883	-	-		-	1	1	2
5662		5658	5664	-	5717	-	-		+	2	0	2
5662		5658	5664	-	5796	-	-		-	1	1	2
5667		5666	5669	-	5712	-	-		+	0	2	2
5667		5666	5669	-	5739	-	-		+	0	2	2
5667		5666	5669	-	5768		5766	5768	-	1	1	2
5691		5689	5696	-	5724	-	-		+	1	1	2
5691		5689	5696	-	5752	-	-		-	1	1	2
5691		5689	5696	-	5861		5861	5862	+	1	1	2
5691		5689	5696	-	5862	-	-		-	1	1	2
5691		5689	5696	-	5951	-	-		-	1	1	2
5700		5700	5704	-	5786	-	-		-	1	1	2
5704		5704	5708	+	5726		5726	5727	+	1	1	2
5710		5707	5710	-	5847		5845	5847	+	1	1	2
5720	-	-		+	5664	-	-		+	1	1	2
5720	-	-		+	5878	-	-		+	1	1	2
5721		5716	5721	-	5837		5837	5838	-	1	1	2
5721		5716	5721	-	5885	-	-		-	1	1	2
5721		5716	5721	-	5903	-	-		-	1	1	2
5721		5716	5721	-	5972	-	-		-	1	1	2
5736		5733	5736	-	5806		5806	5807	+	1	1	2
5744		5740	5744	-	5768	-	-		-	1	1	2
5749		5749	5751	-	11294		11292	11294	-	1	1	2
5751		5747	5753	+	5736	-	-		+	1	1	2
5751		5747	5753	+	5795	-	-		-	2	0	2
5758		5756	5758	+	5749		5747	5749	-	1	1	2
5765		5765	5766	-	5967		5967	5968	-	1	1	2
5768		5768	5772	+	5664		5664	5665	-	1	1	2
5768		5768	5772	+	5749		5745	5749	-	1	1	2
5768		5768	5772	+	5808		5808	5809	+	1	1	2
5780	-	-		+	5855	-	-		+	1	1	2
5801		5799	5804	+	5843	-	-		-	1	1	2
5815		5814	5820	-	5952	-	-		-	1	1	2
5815		5814	5820	-	6061	-	-		-	1	1	2
5815		5814	5820	-	6076		6076	6077	-	2	0	2
5815		5814	5820	-	12720	-	-		+	1	1	2
5827		5824	5827	-	6040	-	-		-	1	1	2
5837	-	-		-	6031	-	-		-	1	1	2
5837	-	-		-	6051	-	-		-	1	1	2
5842		5842	5844	-	5913	-	-		+	1	1	2
5842		5842	5844	-	6014	-	-		-	1	1	2
5847	-	-		-	5981	-	-		-	1	1	2
5856		5853	5857	-	6004	-	-		-	1	1	2
5856		5853	5857	-	6032	-	-		-	1	1	2
5861		5858	5865	-	5996	-	-		-	1	1	2
5861		5858	5865	-	6014	-	-		-	1	1	2
5861		5858	5865	-	6020	-	-		-	1	1	2
5861		5858	5865	-	6026	-	-		-	1	1	2
5861		5858	5865	-	6151		6147	6151	-	1	1	2
5874		5870	5874	-	5856	-	-		+	2	0	2
5874	-	-		+	5884	-	-		-	1	1	2
5874	-	-		+	5908	-	-		-	1	1	2
5880		5880	5884	-	6032		6032	6033	-	1	1	2
5908		5904	5911	-	5965	-	-		+	1	1	2
5921		5919	5925	+	5887	-	-		-	1	1	2
5921		5919	5925	+	6198		6198	6199	+	1	1	2

5926	5923	5930 -	5953 -	-	+	1	1	2
5926	5923	5930 -	6097 -	-	-	1	1	2
5934	5933	5934 -	6097 -	-	-	1	1	2
5953	5950	5955 -	6088	6088	6089 -	1	1	2
5953	5950	5955 -	6097	6095	6097 -	1	1	2
5953	5950	5955 -	6206 -	-	-	1	1	2
5962	5962	5967 +	5986	5986	5987 +	1	1	2
5962	5962	5967 +	6103	6103	6104 +	1	1	2
5973	5971	5974 +	6166 -	-	-	1	1	2
5976	5976	5977 -	6124 -	-	-	1	1	2
5981	5981	5982 +	5884 -	-	-	1	1	2
5981	5981	5982 +	6031 -	-	-	1	1	2
5985	5982	5985 -	6178 -	-	-	1	1	2
6024	6024	6026 -	6049	6049	6050 +	1	1	2
6024	6024	6026 -	6059 -	-	-	1	1	2
6032	6032	6036 +	6138 -	-	+	1	1	2
6052 -	-	+	6112 -	-	-	1	1	2
6061	6059	6062 +	6066 -	-	-	1	1	2
6061	6059	6062 +	6114	6112	6114 -	1	1	2
6063	6060	6063 -	6186	6183	6186 -	1	1	2
6082 -	-	-	6194 -	-	-	1	1	2
6083 -	-	+	10944 -	-	-	1	1	2
6097 -	-	+	6118 -	-	+	1	1	2
6121	6121	6122 +	6110 -	-	+	2	0	2
6121	6121	6122 +	6178 -	-	-	1	1	2
6132	6132	6133 -	6287 -	-	-	1	1	2
6132	6132	6133 -	6300 -	-	-	1	1	2
6144	6144	6146 -	6118 -	-	+	2	0	2
6153 -	-	-	6185 -	-	+	1	1	2
6156 -	-	+	6030 -	-	-	1	1	2
6159	6159	6160 -	6336 -	-	-	1	1	2
6164	6164	6165 +	6214 -	-	-	1	1	2
6171	6170	6171 -	6232 -	-	+	1	1	2
6178	6176	6179 +	6095	6095	6096 -	1	1	2
6178	6176	6179 +	6224	6224	6225 -	1	1	2
6185	6183	6185 +	6153 -	-	-	1	1	2
6216	6216	6218 -	6451 -	-	-	1	1	2
6230	6226	6234 -	6417 -	-	-	1	1	2
6230	6226	6234 -	6474	6472	6474 -	1	1	2
6231 -	-	+	6245 -	-	-	1	1	2
6236	6235	6236 -	6362 -	-	-	1	1	2
6244	6242	6247 -	6384 -	-	-	1	1	2
6253	6250	6254 -	6334	6334	6335 -	1	1	2
6253	6250	6254 -	6359 -	-	-	1	1	2
6253	6250	6254 -	7920 -	-	-	1	1	2
6256	6256	6258 +	6233 -	-	-	1	1	2
6256	6256	6258 +	6276 -	-	-	1	1	2
6263	6262	6266 -	6275 -	-	-	1	1	2
6263	6262	6266 -	6391 -	-	-	1	1	2
6263	6262	6266 -	6435 -	-	-	1	1	2
6273	6273	6276 +	6256 -	-	-	1	1	2
6282 -	-	-	6654 -	-	-	1	1	2
6288	6284	6288 -	6438 -	-	-	1	1	2
6294	6294	6295 -	6348 -	-	+	1	1	2
6294	6294	6295 -	6423	6423	6424 -	1	1	2
6304	6301	6306 +	6463 -	-	-	1	1	2
6309	6305	6312 -	6366 -	-	+	2	0	2
6309	6305	6312 -	6404 -	-	-	2	0	2
6309	6305	6312 -	6474 -	-	-	0	2	2
6309	6305	6312 -	6585	6581	6585 -	1	1	2
6309	6305	6312 -	6717	6717	6718 -	1	1	2
6312	6311	6312 +	6296 -	-	-	1	1	2
6333	6332	6333 +	6424	6424	6425 -	0	2	2
6338	6335	6340 -	6469 -	-	-	1	1	2
6338	6335	6340 -	6479 -	-	-	1	1	2
6345	6344	6349 +	6383 -	-	-	1	1	2
6345	6344	6349 +	7831 -	-	+	1	1	2
6347	6346	6347 -	6483 -	-	-	1	1	2
6347	6346	6347 -	6541 -	-	-	1	1	2
6361 -	-	-	6573 -	-	-	1	1	2
6364	6364	6367 +	10313 -	-	+	1	1	2
6364	6364	6367 +	14878 -	-	+	1	1	2
6380	6379	6380 -	6474 -	-	-	1	1	2
6390	6384	6390 +	6327 -	-	-	1	1	2
6390	6384	6390 +	6415 -	-	-	1	1	2
6390	6384	6390 +	6622 -	-	-	1	1	2
6404	6400	6405 -	6481 -	-	+	1	1	2
6416 -	-	+	6457 -	-	+	1	1	2
6418	6418	6422 -	6450 -	-	+	1	1	2
6418	6418	6422 -	6604 -	-	-	1	1	2
6424 -	-	+	6441 -	-	+	1	1	2
6427	6427	6428 -	6617 -	-	-	1	1	2
6427	6427	6428 -	7293 -	-	+	1	1	2
6435	6431	6436 -	6543 -	-	-	1	1	2
6435	6431	6436 -	6573 -	-	-	1	1	2
6435	6431	6436 -	6584 -	-	-	1	1	2
6435	6431	6436 -	6592 -	-	-	1	1	2
6435	6431	6436 -	7290 -	-	+	1	1	2
6439 -	-	+	6390 -	-	-	1	1	2
6444 -	-	-	6655 -	-	-	1	1	2
6450	6446	6451 -	6589 -	-	-	1	1	2
6450	6446	6451 -	6596 -	-	-	1	1	2
6450	6446	6451 -	6620	6620	6621 -	1	1	2
6450	6446	6451 -	6625 -	-	-	1	1	2
6450	6446	6451 -	6656 -	-	-	1	1	2
6464	6460	6464 -	6602 -	-	-	1	1	2

6469	6466	6469	+	6523	-	-	-	1	1	2
6481	-	-	-	6511	-	-	+	2	0	2
6487	-	-	+	6547	-	-	+	1	1	2
6490	-	-	-	6669	-	-	-	1	1	2
6514	6512	6519	-	6656	-	-	-	1	1	2
6514	6512	6519	-	6677	-	-	-	1	1	2
6514	6512	6519	-	6684	6684	6685	-	1	1	2
6514	6512	6519	-	8189	-	-	-	1	1	2
6521	-	-	+	6472	-	-	-	1	1	2
6532	-	-	-	6738	-	-	-	1	1	2
6538	6535	6538	-	6614	-	-	+	1	1	2
6538	6535	6538	-	6805	-	-	+	2	0	2
6543	6540	6543	-	6800	-	-	+	0	2	2
6551	6551	6554	-	6670	-	-	-	1	1	2
6551	6551	6554	-	6723	-	-	-	1	1	2
6551	6551	6554	-	6738	-	-	-	1	1	2
6568	6567	6568	-	6750	-	-	+	1	1	2
6568	6567	6568	-	6778	-	-	-	1	1	2
6568	6567	6568	-	11152	-	-	+	1	1	2
6578	6577	6578	+	6685	-	-	-	1	1	2
6580	6577	6580	-	6725	6722	6725	-	1	1	2
6580	6577	6580	-	6737	-	-	+	2	0	2
6580	6577	6580	-	8268	-	-	+	2	0	2
6597	6597	6598	-	6725	6725	6726	-	1	1	2
6614	6614	6616	-	7608	-	-	-	1	1	2
6618	6618	6622	+	7085	-	-	-	1	1	2
6620	6620	6621	-	6723	-	-	-	1	1	2
6620	6620	6621	-	6889	6885	6889	-	2	0	2
6642	6642	6644	+	6699	-	-	-	1	1	2
6648	6644	6650	-	6834	-	-	-	1	1	2
6650	6649	6652	+	6787	-	-	-	1	1	2
6653	6652	6656	-	6720	-	-	-	1	1	2
6653	6652	6656	-	6773	6769	6773	+	1	1	2
6661	6661	6662	+	6672	6672	6673	-	1	1	2
6664	6660	6664	-	6773	6769	6773	+	1	1	2
6667	-	-	+	6723	-	-	-	1	1	2
6672	-	-	+	8113	-	-	+	1	1	2
6677	6677	6680	-	6918	-	-	-	1	1	2
6686	6684	6687	+	6770	-	-	-	1	1	2
6686	6684	6687	+	6828	-	-	-	1	1	2
6689	6689	6690	-	6726	-	-	+	1	1	2
6689	6689	6690	-	6846	-	-	-	1	1	2
6689	6689	6690	-	6898	-	-	-	1	1	2
6689	6689	6690	-	6907	-	-	-	1	1	2
6694	6692	6699	+	6710	-	-	+	1	1	2
6694	6692	6699	+	6713	-	-	-	1	1	2
6694	6692	6699	+	6719	-	-	-	1	1	2
6736	6736	6741	-	6905	-	-	-	1	1	2
6736	6736	6741	-	6955	-	-	-	1	1	2
6736	6736	6741	-	7446	-	-	-	1	1	2
6736	-	-	+	6853	-	-	-	1	1	2
6736	-	-	+	6935	-	-	+	1	1	2
6743	-	-	+	6726	6723	6726	-	1	1	2
6745	6744	6749	-	7121	-	-	-	1	1	2
6778	-	-	+	6827	-	-	-	1	1	2
6782	6782	6786	-	7304	-	-	-	1	1	2
6782	6782	6786	-	9876	-	-	+	1	1	2
6793	-	-	+	6718	-	-	-	1	1	2
6804	-	-	-	6907	-	-	-	1	1	2
6809	6806	6809	-	6955	-	-	-	1	1	2
6813	6809	6817	+	6730	-	-	-	1	1	2
6813	6809	6817	+	6855	-	-	-	2	0	2
6815	6811	6815	-	6928	-	-	+	1	1	2
6834	6834	6837	+	6861	6861	6862	-	1	1	2
6850	6848	6855	+	6706	-	-	-	1	1	2
6850	6848	6855	+	7136	-	-	-	1	1	2
6852	6852	6853	-	6974	6974	6975	+	1	1	2
6857	6854	6858	-	6965	6965	6966	+	1	1	2
6857	6854	6858	-	6971	-	-	+	1	1	2
6866	6865	6868	+	6812	-	-	-	1	1	2
6878	6878	6879	+	6910	6910	6911	-	1	1	2
6885	6885	6886	+	6922	6922	6923	+	1	1	2
6895	6893	6895	-	7056	-	-	-	1	1	2
6895	6893	6895	-	7121	-	-	-	1	1	2
6904	6904	6907	+	7188	7188	7189	-	1	1	2
6905	6903	6905	-	7010	-	-	+	1	1	2
6905	6903	6905	-	7092	7090	7092	-	1	1	2
6905	6903	6905	-	7110	-	-	-	1	1	2
6905	6903	6905	-	7280	7280	7281	-	1	1	2
6927	-	-	-	7077	-	-	-	1	1	2
6933	6929	6933	+	13662	13662	13663	-	1	1	2
6933	6929	6933	+	15374	-	-	-	2	0	2
6945	6943	6945	+	6879	6877	6879	-	1	1	2
6949	6946	6950	-	7051	7051	7052	-	1	1	2
6949	6946	6950	-	7126	-	-	-	1	1	2
6980	6975	6984	-	7129	7127	7129	-	1	1	2
6986	-	-	+	6956	-	-	-	1	1	2
6991	6988	6994	-	7339	-	-	-	1	1	2
6993	6989	6993	+	12347	-	-	+	1	1	2
7011	7008	7011	-	7122	7119	7122	-	1	1	2
7011	7011	7015	+	7051	-	-	-	1	1	2
7019	7019	7020	-	7105	7105	7106	-	1	1	2
7034	7034	7038	+	6930	-	-	-	1	1	2
7034	7034	7038	+	7078	-	-	-	1	1	2
7044	-	-	+	7062	-	-	-	1	1	2
7045	7041	7045	-	7096	-	-	+	0	2	2

7051	7047	7053 +	7013 -	-	-	1	1	2
7060	7060	7065 -	7120 -	-	+	1	1	2
7060	7060	7065 -	7201 -	-	+	2	0	2
7061	7061	7063 +	7317	7317	7318 -	1	1	2
7070	7070	7071 +	7053	7053	7054 -	1	1	2
7074	7070	7077 -	7288 -	-	-	1	1	2
7077	7073	7080 +	6957 -	-	-	1	1	2
7077	7073	7080 +	7056 -	-	-	1	1	2
7077	7073	7080 +	7105 -	-	-	1	1	2
7084	7080	7084 -	7246	7246	7247 -	1	1	2
7084	7080	7084 -	7256 -	-	-	1	1	2
7086	7086	7087 +	7157	7157	7158 +	1	1	2
7091	7085	7094 -	7303 -	-	-	1	1	2
7091	7085	7094 -	7328 -	-	+	2	0	2
7097	7095	7097 -	7216 -	-	-	1	1	2
7104 -	-	+	7013 -	-	-	1	1	2
7107	7104	7111 -	8351	8351	8352 -	1	1	2
7118 -	-	-	7312 -	-	-	1	1	2
7126	7123	7126 -	7181 -	-	-	1	1	2
7130	7126	7131 +	7249 -	-	-	2	0	2
7130	7126	7131 +	7263 -	-	-	1	1	2
7138	7135	7138 +	7207 -	-	+	1	1	2
7138	7135	7138 +	7248 -	-	-	0	2	2
7141	7137	7141 -	7300 -	-	-	1	1	2
7147	7147	7151 -	7263	7263	7264 +	1	1	2
7147	7147	7151 -	7366 -	-	-	1	1	2
7158 -	-	-	7233 -	-	-	1	1	2
7164 -	-	-	7220 -	-	+	1	1	2
7167	7164	7167 +	7220 -	-	-	1	1	2
7167	7164	7167 +	14393 -	-	+	1	1	2
7170	7166	7170 -	7338 -	-	-	1	1	2
7170	7166	7170 -	7374 -	-	-	1	1	2
7173	7173	7177 +	7162 -	-	-	1	1	2
7184	7180	7186 +	7139	7137	7139 -	1	1	2
7184	7180	7186 +	7191 -	-	+	1	1	2
7184	7180	7186 +	7663 -	-	-	1	1	2
7192	7192	7194 -	7357 -	-	-	1	1	2
7192 -	-	+	7142 -	-	+	1	1	2
7200 -	-	-	7844 -	-	-	1	1	2
7205 -	-	+	7164 -	-	-	1	1	2
7216 -	-	-	7347 -	-	-	1	1	2
7221	7218	7221 -	7348 -	-	-	1	1	2
7221	7218	7221 -	7541 -	-	-	1	1	2
7221	7220	7225 +	7164 -	-	-	1	1	2
7240	7238	7241 -	7404 -	-	-	1	1	2
7240	7238	7241 -	7437 -	-	-	1	1	2
7240	7238	7241 -	7485 -	-	-	1	1	2
7245	7245	7247 -	7368 -	-	-	1	1	2
7245	7245	7247 -	7435 -	-	-	1	1	2
7250	7250	7252 -	7377 -	-	-	1	1	2
7250	7250	7252 -	7435 -	-	-	1	1	2
7250	7250	7252 -	7454 -	-	-	1	1	2
7257 -	-	+	7286 -	-	-	1	1	2
7263	7260	7263 +	7234 -	-	-	1	1	2
7271	7268	7271 -	7364 -	-	-	1	1	2
7283	7281	7283 -	7435 -	-	-	1	1	2
7283	7281	7283 -	7441 -	-	-	1	1	2
7283	7281	7283 -	7653 -	-	-	1	1	2
7283	7281	7283 -	12146 -	-	-	1	1	2
7285	7281	7287 +	7326	7326	7327 -	1	1	2
7289	7285	7289 -	7419 -	-	-	1	1	2
7289	7285	7289 -	7460 -	-	-	1	1	2
7293 -	-	+	7308 -	-	+	1	1	2
7298	7298	7299 -	7355	7355	7356 -	1	1	2
7307	7307	7308 -	7354 -	-	+	1	1	2
7307	7307	7308 -	7656 -	-	-	1	1	2
7315 -	-	-	7586 -	-	+	1	1	2
7321	7321	7323 -	7357 -	-	+	1	1	2
7345	7345	7346 +	7516	7516	7517 -	1	1	2
7356	7356	7360 +	7422 -	-	-	1	1	2
7369 -	-	+	7492 -	-	-	1	1	2
7372	7372	7377 -	7420 -	-	+	1	1	2
7378 -	-	+	7361 -	-	-	1	1	2
7385	7385	7390 +	7332	7332	7333 -	1	1	2
7385	7385	7390 +	7454 -	-	+	1	1	2
7385	7385	7390 +	7598 -	-	-	1	1	2
7397	7395	7397 +	7434 -	-	+	1	1	2
7415 -	-	+	7436 -	-	-	1	1	2
7420	7416	7426 -	7567 -	-	-	1	1	2
7423	7419	7423 +	7364	7360	7364 -	1	1	2
7423	7419	7423 +	7381 -	-	-	1	1	2
7428	7427	7431 -	7510	7508	7510 +	1	1	2
7428	7427	7431 -	7641 -	-	-	1	1	2
7428	7427	7431 -	7665 -	-	+	1	1	2
7428	7427	7431 -	7739 -	-	+	1	1	2
7432 -	-	+	7363 -	-	-	1	1	2
7437	7437	7438 -	7609 -	-	-	1	1	2
7437	7437	7438 -	7623 -	-	-	1	1	2
7452	7447	7452 +	7385 -	-	-	1	1	2
7455	7455	7457 -	11717 -	-	-	1	1	2
7458	7458	7459 +	7462	7462	7463 -	1	1	2
7480	7477	7481 -	7519	7519	7520 +	1	1	2
7485	7485	7488 -	7518 -	-	-	1	1	2
7490	7490	7494 -	7508	7508	7509 +	1	1	2
7490	7490	7494 -	7633 -	-	-	1	1	2
7495	7491	7495 +	7507	7507	7508 -	1	1	2

7500	-	-	+	7475	-	-	-	0	2	2
7505	7501	7505	-	7717	-	-	-	1	1	2
7512	7507	7515	-	7666	-	-	-	1	1	2
7512	7507	7515	-	7737	-	-	-	0	2	2
7517	7516	7520	-	7623	7621	7623	+	1	1	2
7517	7516	7520	-	7737	7734	7737	-	2	0	2
7518	7518	7523	+	7427	-	-	-	1	1	2
7518	7518	7523	+	7565	7565	7566	+	1	1	2
7518	7518	7523	+	7594	-	-	-	2	0	2
7526	7522	7529	-	7607	-	-	-	1	1	2
7532	7532	7534	-	7679	7679	7680	-	1	1	2
7532	7532	7534	-	7712	-	-	-	1	1	2
7538	7537	7541	-	7626	-	-	-	1	1	2
7538	7537	7541	-	7680	-	-	-	1	1	2
7538	7537	7541	-	7705	-	-	-	1	1	2
7547	7545	7549	+	7566	-	-	+	1	1	2
7552	-	-	+	7651	-	-	-	1	1	2
7567	7566	7570	+	7605	-	-	+	1	1	2
7567	7566	7570	+	7849	-	-	-	1	1	2
7567	7566	7570	+	8054	8052	8054	-	1	1	2
7572	7572	7576	+	7612	7609	7612	-	1	1	2
7572	7572	7576	+	7683	-	-	-	1	1	2
7583	7579	7583	-	7766	-	-	-	1	1	2
7591	7590	7595	-	7597	-	-	+	0	2	2
7591	7590	7595	-	7630	-	-	-	2	0	2
7591	7590	7595	-	7674	-	-	-	1	1	2
7611	7611	7614	+	7587	7587	7588	+	1	1	2
7611	7611	7614	+	9512	9512	9513	-	1	1	2
7612	7610	7613	-	7793	-	-	+	1	1	2
7621	7621	7625	-	7780	-	-	-	1	1	2
7626	7626	7631	+	7586	-	-	+	1	1	2
7626	7626	7631	+	7594	-	-	+	2	0	2
7626	7626	7631	+	7681	7681	7682	+	1	1	2
7626	7626	7631	+	7792	-	-	-	1	1	2
7626	7626	7631	+	7829	-	-	+	1	1	2
7632	7630	7633	-	7707	7707	7708	-	1	1	2
7637	7637	7639	-	7799	-	-	-	1	1	2
7637	7637	7639	-	7834	-	-	+	1	1	2
7638	7635	7638	+	7545	7545	7546	-	1	1	2
7638	7635	7638	+	7704	-	-	-	1	1	2
7642	-	-	-	7750	-	-	-	1	1	2
7649	-	-	-	7805	-	-	-	1	1	2
7658	7654	7658	-	8080	8080	8081	-	1	1	2
7658	7654	7658	-	12759	-	-	+	1	1	2
7661	7661	7662	+	7744	7744	7745	+	1	1	2
7665	7664	7666	-	7808	-	-	+	1	1	2
7669	-	-	+	7573	-	-	-	1	1	2
7670	7670	7673	-	7820	-	-	-	1	1	2
7676	-	-	+	7688	-	-	+	1	1	2
7683	7679	7684	-	7843	-	-	-	1	1	2
7683	7679	7684	-	7861	-	-	-	1	1	2
7688	7688	7691	-	7757	-	-	-	1	1	2
7688	7688	7691	-	7806	-	-	-	1	1	2
7688	7688	7691	-	7859	-	-	-	1	1	2
7688	7688	7691	-	7897	-	-	-	1	1	2
7694	7693	7694	-	7760	-	-	-	1	1	2
7696	7694	7696	+	7895	-	-	-	1	1	2
7701	7699	7701	-	11701	-	-	-	1	1	2
7707	7706	7709	+	7547	7547	7548	-	1	1	2
7707	7706	7709	+	7726	7726	7727	+	1	1	2
7707	7706	7709	+	7927	7927	7928	+	1	1	2
7716	7716	7717	+	7672	7672	7673	-	1	1	2
7716	7716	7717	+	7761	-	-	-	2	0	2
7720	-	-	-	7782	-	-	-	1	1	2
7729	7727	7733	-	7858	-	-	+	1	1	2
7729	7727	7733	-	7860	7856	7860	-	1	1	2
7739	7736	7742	-	7758	7755	7758	+	1	1	2
7739	7736	7742	-	7833	7830	7833	-	1	1	2
7739	7736	7742	-	7854	-	-	-	1	1	2
7739	7736	7742	-	7878	-	-	-	0	2	2
7739	7736	7742	-	7884	-	-	-	1	1	2
7739	7736	7742	-	7897	-	-	-	1	1	2
7754	7754	7756	-	7860	-	-	-	1	1	2
7754	7754	7756	-	8033	-	-	-	1	1	2
7761	-	-	+	7716	-	-	-	2	0	2
7767	7766	7767	+	7656	7656	7657	-	1	1	2
7770	7767	7770	-	8130	-	-	+	1	1	2
7785	7784	7786	-	7866	7864	7866	+	1	1	2
7791	7788	7794	-	7857	-	-	+	1	1	2
7791	7788	7794	-	7985	-	-	-	1	1	2
7791	7788	7794	-	8000	-	-	-	1	1	2
7797	7796	7800	-	7838	-	-	+	1	1	2
7797	7796	7800	-	7855	-	-	+	1	1	2
7797	7797	7798	+	7882	7882	7883	-	1	1	2
7802	7802	7803	+	7877	7877	7878	-	1	1	2
7809	7805	7812	+	7777	-	-	-	1	1	2
7809	7805	7812	+	7824	-	-	-	1	1	2
7809	7805	7812	+	7875	-	-	-	1	1	2
7812	7809	7814	-	8080	-	-	-	1	1	2
7812	7809	7814	-	11264	-	-	-	1	1	2
7820	7818	7824	+	8030	-	-	-	1	1	2
7825	7820	7826	-	7812	-	-	+	2	0	2
7825	7820	7826	-	7994	-	-	-	1	1	2
7825	7820	7826	-	8001	-	-	-	1	1	2
7827	7826	7830	+	7809	-	-	-	1	1	2
7827	7826	7830	+	9198	9195	9198	+	1	1	2

7827	7826	7830	+	11158	11158	11159	+	2	0	2
7830	7829	7832	-	8005	-	-	-	1	1	2
7830	7829	7832	-	8026	-	-	-	1	1	2
7830	7829	7832	-	8064	-	-	-	1	1	2
7830	7829	7832	-	8084	-	-	+	1	1	2
7837	7835	7837	-	7866	-	-	+	1	1	2
7837	7835	7837	-	7989	-	-	-	1	1	2
7841	7841	7842	+	7985	-	-	-	1	1	2
7841	7841	7842	+	8048	-	-	+	1	1	2
7846	-	-	-	7981	-	-	-	1	1	2
7853	-	-	+	15309	-	-	+	1	1	2
7857	7856	7858	-	8006	-	-	-	1	1	2
7857	7856	7858	-	8067	-	-	-	1	1	2
7857	7856	7858	-	8087	-	-	-	1	1	2
7862	7862	7866	-	7918	-	-	+	1	1	2
7862	7862	7866	-	7995	-	-	-	1	1	2
7876	7874	7876	-	8065	-	-	-	1	1	2
7882	7880	7882	-	8047	8047	8048	-	1	1	2
7883	7883	7886	+	7779	-	-	-	1	1	2
7883	7883	7886	+	13227	-	-	+	1	1	2
7888	7885	7889	-	8139	-	-	+	1	1	2
7888	7885	7889	-	8201	8198	8201	-	1	1	2
7888	7885	7889	-	8340	8340	8341	-	1	1	2
7894	7891	7899	-	7922	-	-	+	2	0	2
7894	7891	7899	-	7949	-	-	+	1	1	2
7894	7891	7899	-	7989	-	-	+	1	1	2
7895	-	-	+	8174	-	-	+	1	1	2
7902	-	-	-	7919	-	-	+	0	2	2
7902	-	-	-	8050	-	-	+	1	1	2
7912	-	-	-	7921	-	-	+	1	1	2
7920	7918	7921	-	7957	-	-	+	2	0	2
7920	7918	7921	-	7966	-	-	+	1	1	2
7926	7925	7927	-	8042	-	-	+	1	1	2
7934	7931	7936	-	8091	-	-	-	1	1	2
7935	-	-	+	13300	-	-	+	1	1	2
7940	7939	7940	-	8198	-	-	-	1	1	2
7945	7945	7949	-	7985	7983	7985	-	1	1	2
7945	7945	7949	-	8034	-	-	-	1	1	2
7945	7945	7949	-	8147	-	-	-	1	1	2
7951	7951	7953	+	7981	-	-	-	1	1	2
7956	7956	7960	+	7922	-	-	-	2	0	2
7956	7956	7960	+	7949	7947	7949	-	1	1	2
7956	7956	7960	+	8096	8096	8097	-	1	1	2
7957	7954	7961	-	7921	-	-	+	2	0	2
7957	7954	7961	-	8115	-	-	-	1	1	2
7966	7964	7967	-	8042	-	-	+	1	1	2
7966	7966	7968	+	7950	7948	7950	-	1	1	2
7975	-	-	-	8024	-	-	+	1	1	2
7979	-	-	+	7864	-	-	-	1	1	2
7985	7981	7989	+	7986	-	-	-	1	1	2
7988	7984	7990	-	8075	-	-	-	1	1	2
7988	7984	7990	-	8155	-	-	+	1	1	2
7992	7991	7995	+	8094	-	-	-	1	1	2
8002	8002	8003	+	8132	-	-	+	1	1	2
8002	8002	8003	+	8138	8138	8139	-	1	1	2
8004	8000	8006	-	8036	-	-	+	1	1	2
8004	8000	8006	-	8179	-	-	-	1	1	2
8004	8000	8006	-	8203	-	-	-	1	1	2
8007	8007	8011	+	8088	8088	8089	-	1	1	2
8012	8009	8013	-	8149	-	-	-	1	1	2
8012	8009	8013	-	8177	-	-	-	1	1	2
8012	8009	8013	-	8273	-	-	-	1	1	2
8022	8020	8025	+	7924	-	-	-	1	1	2
8024	8024	8025	-	8076	-	-	+	1	1	2
8036	-	-	-	8178	-	-	-	1	1	2
8036	-	-	-	9412	-	-	-	1	1	2
8040	8040	8041	+	7996	7996	7997	-	1	1	2
8043	8042	8043	-	8238	-	-	-	1	1	2
8048	8044	8049	-	8106	-	-	+	1	1	2
8048	8044	8049	-	8178	-	-	-	1	1	2
8048	8044	8049	-	8240	-	-	-	1	1	2
8048	8044	8049	-	8247	-	-	-	1	1	2
8055	8054	8055	-	8100	8100	8101	+	1	1	2
8059	8058	8059	+	7948	-	-	-	1	1	2
8060	8057	8063	-	8173	8171	8173	-	1	1	2
8060	8057	8063	-	8243	-	-	-	1	1	2
8065	8062	8067	+	8008	-	-	-	1	1	2
8067	8065	8067	-	8198	-	-	-	1	1	2
8067	8065	8067	-	8245	-	-	-	1	1	2
8070	8069	8073	+	8108	8108	8109	+	1	1	2
8076	8072	8080	-	8118	8118	8119	-	1	1	2
8076	8072	8080	-	8244	-	-	-	1	1	2
8081	8081	8083	+	8283	8283	8284	-	1	1	2
8082	8082	8085	-	8185	-	-	+	1	1	2
8082	8082	8085	-	8258	-	-	-	2	0	2
8087	-	-	-	8260	-	-	-	0	2	2
8088	8087	8089	+	8112	8112	8113	+	1	1	2
8099	-	-	+	8133	-	-	+	1	1	2
8114	8112	8117	-	8261	-	-	-	1	1	2
8118	8116	8121	+	8086	-	-	-	1	1	2
8118	8116	8121	+	8150	-	-	-	1	1	2
8123	8120	8124	-	8242	-	-	+	1	1	2
8132	-	-	-	8193	-	-	+	1	1	2
8142	8140	8143	-	8262	-	-	-	1	1	2
8154	8150	8155	-	8175	-	-	+	1	1	2
8154	8150	8155	-	8220	-	-	+	1	1	2

8160	-	-	+	8175	-	-	+	1	1	2
8164	8162	8166	-	8192	8188	8192	+	1	1	2
8164	8162	8166	-	8316	-	-	-	1	1	2
8176	8176	8180	+	8093	-	-	-	1	1	2
8176	8176	8180	+	8223	-	-	-	1	1	2
8186	8182	8187	-	8412	8412	8413	+	0	2	2
8198	-	-	+	8157	-	-	-	1	1	2
8216	-	-	-	8404	-	-	-	1	1	2
8224	-	-	+	13512	-	-	+	1	1	2
8232	8231	8236	-	8277	8273	8277	+	1	1	2
8244	8238	8245	-	8383	-	-	-	1	1	2
8244	8238	8245	-	8388	8388	8390	-	1	1	2
8244	8238	8245	-	8467	-	-	-	1	1	2
8262	8262	8263	+	8305	-	-	-	1	1	2
8262	8262	8263	+	8318	-	-	+	1	1	2
8272	8270	8276	-	8415	-	-	-	1	1	2
8276	-	-	+	8290	-	-	+	1	1	2
8280	8280	8282	-	8396	-	-	-	1	1	2
8280	8280	8282	-	8467	8467	8468	-	1	1	2
8305	-	-	+	8263	-	-	-	1	1	2
8308	-	-	-	12903	-	-	+	1	1	2
8321	-	-	+	8384	-	-	-	1	1	2
8323	8320	8323	-	8374	8371	8374	+	1	1	2
8323	8320	8323	-	8446	-	-	-	1	1	2
8329	-	-	-	8351	-	-	+	1	1	2
8334	-	-	+	8404	-	-	-	1	1	2
8341	8341	8342	+	8547	-	-	+	1	1	2
8341	8341	8342	+	9563	9563	9564	+	1	1	2
8343	8342	8347	-	8352	-	-	+	0	2	2
8343	8342	8347	-	8382	-	-	+	1	1	2
8343	8342	8347	-	8530	8527	8530	-	1	1	2
8343	8342	8347	-	8549	-	-	-	1	1	2
8343	8342	8347	-	10962	-	-	+	0	2	2
8347	8346	8347	+	8374	-	-	-	1	1	2
8358	8358	8360	+	8403	8403	8404	-	1	1	2
8360	8358	8360	-	8336	-	-	+	2	0	2
8360	8358	8360	-	8535	-	-	-	1	1	2
8373	8373	8376	-	8391	-	-	+	1	1	2
8373	8373	8376	-	8511	-	-	-	1	1	2
8373	8373	8376	-	12892	-	-	-	0	2	2
8383	8378	8385	-	8489	-	-	-	2	0	2
8383	8378	8385	-	8505	-	-	-	1	1	2
8383	8378	8385	-	8512	-	-	-	1	1	2
8383	8378	8385	-	8550	-	-	-	1	1	2
8387	8384	8391	+	8341	-	-	-	1	1	2
8387	8384	8391	+	8464	-	-	-	1	1	2
8391	8391	8392	-	8525	-	-	+	1	1	2
8396	8394	8400	-	8548	8544	8548	-	1	1	2
8396	8394	8400	-	8563	-	-	-	0	2	2
8396	8394	8400	-	8580	-	-	-	1	1	2
8396	8394	8400	-	8613	-	-	-	1	1	2
8396	8393	8396	+	12082	-	-	-	2	0	2
8403	8400	8406	+	8298	-	-	-	1	1	2
8403	8400	8406	+	8356	8356	8357	-	1	1	2
8403	8400	8406	+	8429	-	-	-	1	1	2
8403	8400	8406	+	8449	-	-	-	1	1	2
8409	8406	8413	-	8449	-	-	+	1	1	2
8410	8410	8413	+	8450	-	-	+	1	1	2
8415	8415	8416	+	8302	-	-	-	1	1	2
8426	8426	8427	-	8532	8532	8533	+	1	1	2
8426	8426	8427	-	8590	8590	8591	-	1	1	2
8431	8430	8434	-	8448	8444	8448	+	1	1	2
8431	8428	8431	+	8347	-	-	+	1	1	2
8443	8440	8448	-	8599	-	-	-	1	1	2
8443	8440	8448	-	8622	-	-	-	1	1	2
8450	8450	8453	-	8650	-	-	+	1	1	2
8459	8456	8459	-	8641	8641	8642	+	1	1	2
8459	8456	8463	+	12082	-	-	-	2	0	2
8464	8464	8465	-	8620	8620	8621	-	1	1	2
8472	8470	8476	-	8501	-	-	+	1	1	2
8472	8470	8476	-	8601	-	-	-	1	1	2
8484	8482	8486	-	8674	-	-	-	1	1	2
8495	-	-	-	8950	-	-	+	1	1	2
8495	-	-	-	11460	-	-	-	1	1	2
8511	8511	8512	-	8675	8675	8676	-	1	1	2
8513	-	-	+	8638	-	-	-	2	0	2
8523	8519	8526	-	8623	-	-	-	1	1	2
8523	8519	8526	-	8674	-	-	-	1	1	2
8523	8519	8526	-	8681	-	-	-	1	1	2
8523	8519	8526	-	8691	-	-	-	1	1	2
8523	8519	8526	-	8712	8712	8713	-	1	1	2
8523	8519	8526	-	8734	8730	8734	+	1	1	2
8534	-	-	+	8710	-	-	-	1	1	2
8554	-	-	+	8667	-	-	-	1	1	2
8560	8556	8560	-	8678	-	-	+	1	1	2
8560	8559	8564	+	8608	8608	8609	+	1	1	2
8560	8559	8564	+	12372	-	-	-	1	1	2
8569	8569	8571	-	8962	-	-	+	1	1	2
8571	-	-	+	8709	-	-	-	1	1	2
8577	8573	8577	-	8596	-	-	+	1	1	2
8577	8573	8577	-	8632	8632	8633	-	1	1	2
8579	8578	8583	+	8638	8638	8639	+	1	1	2
8583	8583	8586	-	8759	-	-	-	1	1	2
8598	-	-	-	8673	-	-	-	1	1	2
8607	8603	8611	-	8660	-	-	+	2	0	2
8608	-	-	+	8655	-	-	-	1	1	2

8619		8617	8619	-	8818	-	-		-	1	1	2
8619		8617	8619	-	8832	-	-		-	1	1	2
8624		8624	8625	-	12112	-	-		-	1	1	2
8635		8631	8635	-	8794	-	-		-	1	1	2
8644		8640	8647	-	8818	-	-		-	1	1	2
8644		8640	8647	-	8821	-	-		+	1	1	2
8644		8640	8647	-	8861	-	-		-	1	1	2
8649		8649	8652	-	8858		8856	8858	-	1	1	2
8650	-	-		+	8730	-	-		+	1	1	2
8658		8655	8659	+	8794		8790	8794	-	1	1	2
8659		8659	8663	-	8778		8774	8778	+	1	1	2
8659		8659	8663	-	8863	-	-		-	1	1	2
8667		8662	8669	+	8799		8796	8799	+	1	1	2
8667		8662	8669	+	8861	-	-		-	1	1	2
8667		8662	8669	+	11093		11093	11094	-	1	1	2
8674		8673	8676	-	8819	-	-		-	1	1	2
8674		8673	8676	-	8891	-	-		-	1	1	2
8688	-	-		+	8777	-	-		+	1	1	2
8707		8707	8708	-	8751		8751	8752	+	1	1	2
8715		8712	8715	-	8863		8860	8863	-	1	1	2
8727	-	-		-	8948	-	-		-	1	1	2
8728		8728	8731	+	8765		8765	8766	+	1	1	2
8740		8740	8741	-	8773		8771	8773	+	1	1	2
8742		8739	8747	+	8876	-	-		+	1	1	2
8748		8746	8748	-	8768	-	-		+	1	1	2
8748		8746	8748	-	8917	-	-		-	1	1	2
8752		8752	8753	+	8705		8705	8706	-	1	1	2
8752		8752	8753	+	8762	-	-		-	1	1	2
8757		8757	8758	+	8716		8716	8717	-	1	1	2
8763		8759	8763	-	8927	-	-		-	1	1	2
8769		8766	8771	-	9008	-	-		-	0	2	2
8775		8774	8779	-	9008	-	-		-	1	1	2
8778		8775	8780	+	8680	-	-		-	1	1	2
8778		8775	8780	+	8766	-	-		-	1	1	2
8778		8775	8780	+	8870	-	-		-	1	1	2
8784		8784	8786	-	8880	-	-		-	1	1	2
8784		8784	8786	-	8927	-	-		-	1	1	2
8794		8791	8797	-	8840		8837	8840	-	1	1	2
8794		8791	8797	-	8854	-	-		+	1	1	2
8794		8791	8797	-	8903	-	-		-	1	1	2
8811		8810	8813	-	8950	-	-		-	1	1	2
8811		8810	8813	-	8989	-	-		-	1	1	2
8811		8810	8813	-	9011	-	-		-	1	1	2
8819	-	-		-	9008	-	-		-	1	1	2
8825		8823	8828	-	8965	-	-		-	1	1	2
8833		8833	8835	+	8790	-	-		-	1	1	2
8833		8833	8835	+	8820	-	-		-	1	1	2
8843	-	-		+	8812	-	-		-	1	1	2
8858	-	-		-	9006	-	-		-	1	1	2
8861		8861	8862	+	8761		8761	8762	-	1	1	2
8861		8861	8862	+	8986	-	-		-	1	1	2
8873		8870	8876	+	8951		8947	8951	+	1	1	2
8873		8870	8876	+	11620		11618	11620	+	1	1	2
8875		8875	8879	-	9064	-	-		-	1	1	2
8885		8884	8885	+	9043	-	-		-	1	1	2
8887		8883	8887	-	9032	-	-		-	1	1	2
8887		8883	8887	-	9044		9044	9045	-	1	1	2
8887		8883	8887	-	9072		9072	9073	-	1	1	2
8892	-	-		-	9010	-	-		-	1	1	2
8905	-	-		+	8794	-	-		-	1	1	2
8907		8903	8911	-	9006	-	-		+	1	1	2
8907		8903	8911	-	9024	-	-		-	1	1	2
8907		8903	8911	-	9125	-	-		-	1	1	2
8907		8903	8911	-	9276	-	-		-	1	1	2
8917		8917	8918	+	8780		8780	8781	-	1	1	2
8917		8917	8918	+	8968	-	-		-	1	1	2
8929		8928	8929	-	8884	-	-		+	2	0	2
8935		8935	8939	-	9123	-	-		-	1	1	2
8935		8931	8935	+	9065	-	-		-	1	1	2
8947		8943	8948	-	8968	-	-		+	1	1	2
8947		8943	8948	-	9085	-	-		-	1	1	2
8947		8943	8948	-	9113	-	-		-	1	1	2
8950		8946	8952	+	8863		8863	8864	-	1	1	2
8950		8946	8952	+	9041	-	-		-	1	1	2
8950		8946	8952	+	9060	-	-		-	1	1	2
8952		8951	8958	-	9077	-	-		+	2	0	2
8952		8951	8958	-	9087		9087	9088	+	1	1	2
8952		8951	8958	-	9128	-	-		+	1	1	2
8952		8951	8958	-	9146	-	-		-	1	1	2
8959	-	-		+	8925	-	-		-	1	1	2
8961		8959	8965	-	9071	-	-		+	0	2	2
8961		8959	8965	-	9092	-	-		-	1	1	2
8961		8959	8965	-	9145	-	-		-	1	1	2
8964		8964	8965	+	9024	-	-		-	1	1	2
8971		8966	8974	-	9140	-	-		-	1	1	2
8975		8970	8976	+	8873	-	-		-	1	1	2
8975		8970	8976	+	9014		9014	9015	-	1	1	2
8975		8970	8976	+	9067		9067	9068	-	1	1	2
8978		8975	8982	-	9024	-	-		+	1	1	2
8978		8975	8982	-	9125		9125	9126	-	1	1	2
8978		8975	8982	-	9164	-	-		-	1	1	2
8983		8982	8986	+	9029		9029	9030	-	1	1	2
8986		8984	8989	-	9644	-	-		-	1	1	2
9005		9003	9008	-	9147	-	-		-	1	1	2
9005		9003	9008	-	9214	-	-		-	1	1	2
9010		9010	9011	-	9155	-	-		-	1	1	2

9019	9019	9021 -	8971 -	-	+	2	0	2
9019	9019	9021 -	9156	9156	9157 +	1	1	2
9037	9036	9038 -	9140 -	-	+	1	1	2
9037	9034	9038 +	8977	8977	8978 -	1	1	2
9037	9034	9038 +	9139	9139	9140 -	1	1	2
9048	9046	9049 -	9169	9169	9170 +	1	1	2
9054 -	-	-	9213 -	-	-	1	1	2
9061 -	-	-	9233 -	-	-	1	1	2
9066	9066	9067 -	9155 -	-	+	1	1	2
9068	9068	9071 +	9048 -	-	-	1	1	2
9071	9069	9075 -	9181 -	-	+	1	1	2
9071	9069	9075 -	9199 -	-	-	1	1	2
9071	9069	9075 -	9210 -	-	-	1	1	2
9071	9069	9075 -	9279	9279	9280 -	1	1	2
9090	9086	9091 -	9212 -	-	-	1	1	2
9090	9086	9091 -	9353 -	-	+	0	2	2
9090	9086	9091 -	9358 -	-	+	2	0	2
9105	9103	9106 +	9130 -	-	-	1	1	2
9116	9116	9117 -	9153 -	-	+	1	1	2
9116	9116	9117 -	9288 -	-	+	1	1	2
9118 -	-	+	9211 -	-	-	1	1	2
9121 -	-	-	9208 -	-	+	2	0	2
9129	9125	9134 -	9370 -	-	-	1	1	2
9130	9126	9130 +	9153 -	-	-	1	1	2
9140	9138	9144 -	9156	9154	9156 +	1	1	2
9140	9138	9144 -	9184	9184	9185 +	1	1	2
9140	9138	9144 -	9320	9317	9320 -	1	1	2
9140	9137	9140 +	9183	9183	9184 -	1	1	2
9152	9152	9155 -	9288 -	-	+	1	1	2
9162	9162	9163 -	9285 -	-	-	1	1	2
9162	9162	9163 -	9338 -	-	-	1	1	2
9167 -	-	-	9265 -	-	-	1	1	2
9179 -	-	+	9142 -	-	-	1	1	2
9183	9182	9186 -	9378 -	-	-	1	1	2
9191 -	-	+	9421 -	-	-	1	1	2
9197	9193	9202 -	9321 -	-	-	1	1	2
9197	9193	9202 -	9400 -	-	-	1	1	2
9197	9197	9201 +	9318 -	-	+	1	1	2
9204	9203	9204 -	9211 -	-	+	2	0	2
9204	9203	9204 -	9235 -	-	+	0	2	2
9206 -	-	+	9294 -	-	-	2	0	2
9209	9206	9210 -	9121 -	-	+	2	0	2
9209	9206	9210 -	9400 -	-	-	1	1	2
9209	9206	9210 -	9429 -	-	-	1	1	2
9211	9208	9211 +	9120	9120	9121 -	1	1	2
9211	9208	9211 +	9364 -	-	-	1	1	2
9219	9219	9220 -	9567 -	-	-	1	1	2
9228	9224	9228 -	9297 -	-	+	1	1	2
9228	9224	9228 -	9451	9451	9452 +	1	1	2
9233	9229	9237 +	9264 -	-	+	1	1	2
9233	9229	9237 +	9413 -	-	-	1	1	2
9244	9241	9247 +	9195 -	-	-	1	1	2
9244	9241	9247 +	9251	9251	9252 -	1	1	2
9247 -	-	-	9416 -	-	-	1	1	2
9259	9256	9259 +	9240	9240	9241 -	1	1	2
9273	9269	9274 -	9335	9335	9336 -	1	1	2
9278	9275	9278 -	9295 -	-	+	0	2	2
9278	9275	9278 -	9436	9436	9437 -	1	1	2
9278	9275	9278 -	9453 -	-	-	1	1	2
9278	9275	9278 -	9474 -	-	-	1	1	2
9278	9275	9278 -	9917 -	-	+	1	1	2
9284	9284	9285 -	9473 -	-	-	1	1	2
9284	9281	9284 +	9290	9290	9291 -	1	1	2
9289	9287	9292 -	9416 -	-	-	1	1	2
9305	9300	9309 -	9465	9465	9466 -	1	1	2
9305	9300	9309 -	9483 -	-	-	1	1	2
9305	9300	9309 -	9504 -	-	-	1	1	2
9311	9310	9315 -	9408 -	-	-	1	1	2
9311	9310	9315 -	9446 -	-	-	1	1	2
9311	9310	9315 -	9447 -	-	+	1	1	2
9311	9310	9315 -	9499 -	-	-	1	1	2
9323	9319	9324 -	9424 -	-	+	1	1	2
9333	9333	9336 +	9375 -	-	-	1	1	2
9333	9333	9336 +	9700 -	-	-	1	1	2
9338	9338	9339 +	9369	9369	9370 -	1	1	2
9343 -	-	-	9403 -	-	+	2	0	2
9343	9342	9345 +	9403 -	-	-	1	1	2
9351	9345	9353 -	9353 -	-	+	2	0	2
9351	9345	9353 -	9398 -	-	+	0	2	2
9351	9345	9353 -	9450 -	-	+	1	1	2
9351	9345	9353 -	9506 -	-	-	1	1	2
9351	9345	9353 -	9569 -	-	-	1	1	2
9354	9352	9354 +	9364 -	-	+	1	1	2
9354	9352	9354 +	9681	9681	9682 -	1	1	2
9375	9372	9379 -	9401 -	-	+	2	0	2
9375	9372	9379 -	9502 -	-	-	1	1	2
9375	9372	9379 -	9553 -	-	-	1	1	2
9375	9372	9379 -	9588	9588	9589 -	1	1	2
9375	9372	9379 -	9603 -	-	-	1	1	2
9383	9383	9385 -	9397 -	-	+	0	2	2
9383	9383	9385 -	9501 -	-	-	1	1	2
9383	9383	9385 -	9519 -	-	-	1	1	2
9394	9394	9396 -	9618 -	-	-	0	2	2
9394	9394	9396 -	9776 -	-	-	2	0	2
9399	9399	9400 -	9448 -	-	+	1	1	2
9399	9399	9400 -	9780 -	-	-	0	2	2

9404	9404	9405 -	9495 -	-	+	1	1	2
9404	9404	9405 -	9562 -	-	-	1	1	2
9407	9407	9408 +	9589	9589	9590 -	1	1	2
9417	9414	9417 -	9536 -	-	-	1	1	2
9417	9417	9420 +	9480 -	-	-	1	1	2
9417	9417	9420 +	9579 -	-	-	1	1	2
9424	9424	9425 -	9641 -	-	-	1	1	2
9424 -	-	+	9319 -	-	-	1	1	2
9436	9435	9439 -	9533 -	-	+	1	1	2
9436	9435	9439 -	9542 -	-	+	1	1	2
9436	9435	9439 -	9576 -	-	-	1	1	2
9442	9442	9443 -	9527 -	-	+	1	1	2
9442	9442	9443 -	9583 -	-	+	1	1	2
9447 -	-	+	9604 -	-	-	1	1	2
9451	9447	9454 -	9598 -	-	-	1	1	2
9451	9447	9454 -	9628 -	-	-	1	1	2
9466	9462	9468 -	9593 -	-	+	2	0	2
9466	9462	9468 -	9639 -	-	-	1	1	2
9466	9462	9468 -	9693 -	-	-	1	1	2
9473	9473	9474 -	9588 -	-	+	0	2	2
9473	9473	9474 -	9644 -	-	-	1	1	2
9490	9487	9490 -	9637 -	-	-	1	1	2
9490	9487	9490 -	9715 -	-	-	1	1	2
9498	9498	9499 -	9603 -	-	-	1	1	2
9498	9498	9499 -	9656 -	-	-	1	1	2
9498	9498	9499 -	9763 -	-	+	1	1	2
9498	9498	9501 +	9543	9540	9543 -	1	1	2
9504	9504	9505 -	14047	14047	14048 +	1	1	2
9510	9508	9510 -	9681 -	-	-	1	1	2
9517	9516	9519 -	9760	9760	9761 -	1	1	2
9517	9516	9519 -	9922 -	-	+	2	0	2
9523	9522	9524 -	9916 -	-	+	0	2	2
9530	9526	9533 -	9626 -	-	+	1	1	2
9535	9534	9535 -	9698 -	-	-	1	1	2
9535 -	-	+	9500 -	-	-	1	1	2
9541	9536	9541 -	9622	9622	9623 +	1	1	2
9543	9539	9544 +	9595 -	-	+	1	1	2
9543	9539	9544 +	9917 -	-	+	1	1	2
9548	9544	9551 -	9695 -	-	+	1	1	2
9548	9544	9551 -	9715 -	-	+	1	1	2
9548	9544	9551 -	9735 -	-	-	1	1	2
9548	9544	9551 -	9900 -	-	+	0	2	2
9548	9544	9551 -	9905 -	-	+	2	0	2
9564	9560	9565 +	9638 -	-	-	1	1	2
9564	9560	9565 +	9645	9645	9646 -	1	1	2
9564	9560	9565 +	9718 -	-	-	1	1	2
9569	9569	9570 -	9613 -	-	+	1	1	2
9569	9569	9570 -	9701 -	-	+	1	1	2
9569 -	-	+	9637 -	-	-	1	1	2
9574	9572	9574 +	9522 -	-	-	1	1	2
9574	9572	9574 +	9834 -	-	-	1	1	2
9586	9583	9586 -	9695 -	-	+	1	1	2
9586	9583	9586 -	9796 -	-	-	1	1	2
9593	9593	9594 +	9687 -	-	-	1	1	2
9594	9591	9599 -	9727 -	-	-	1	1	2
9594	9591	9599 -	9783 -	-	-	1	1	2
9598	9597	9602 +	9534 -	-	-	1	1	2
9598	9597	9602 +	9608 -	-	-	1	1	2
9604	9603	9606 +	9630	9630	9631 -	1	1	2
9606	9602	9606 -	9735	9733	9735 -	1	1	2
9606	9602	9606 -	9776	9776	9777 -	1	1	2
9619	9619	9620 -	9743 -	-	-	1	1	2
9619	9619	9620 -	9775 -	-	-	1	1	2
9629 -	-	+	9863 -	-	+	0	2	2
9635	9634	9637 -	9785 -	-	-	1	1	2
9635	9634	9637 -	9819	9819	9820 -	1	1	2
9635	9634	9637 -	10641 -	-	+	1	1	2
9637	9634	9644 +	9522 -	-	-	1	1	2
9637	9634	9644 +	9576 -	-	-	1	1	2
9637	9634	9644 +	9698	9698	9699 +	1	1	2
9637	9634	9644 +	9868 -	-	+	2	0	2
9640 -	-	-	9865 -	-	-	1	1	2
9650 -	-	-	9939	9937	9939 -	1	1	2
9657	9657	9658 -	9677 -	-	+	1	1	2
9657	9657	9658 +	9697 -	-	-	1	1	2
9662 -	-	-	9715 -	-	+	1	1	2
9671	9668	9674 +	9666	9666	9667 -	1	1	2
9671	9668	9674 +	9706	9706	9707 +	1	1	2
9679	9679	9685 -	9919	9919	9920 -	1	1	2
9679	9679	9685 -	9942	9942	9943 -	1	1	2
9684	9684	9687 +	9594 -	-	-	1	1	2
9697	9695	9699 -	9749	9747	9749 +	1	1	2
9697	9695	9699 -	9893 -	-	-	1	1	2
9697	9695	9699 -	9915	9915	9916 -	1	1	2
9697	9695	9699 -	9954	9954	9955 -	1	1	2
9702	9702	9703 -	9959	9959	9960 -	1	1	2
9717	9716	9719 -	9899 -	-	+	1	1	2
9723	9722	9725 -	9736 -	-	+	0	2	2
9723	9722	9725 -	9780	9780	9781 -	1	1	2
9723	9722	9725 -	9842 -	-	-	1	1	2
9723	9722	9725 -	9877 -	-	-	1	1	2
9723	9722	9725 -	9885	9885	9886 -	1	1	2
9723	9722	9725 -	9934	9934	9935 -	1	1	2
9723	9722	9725 -	9939 -	-	-	1	1	2
9734	9731	9734 -	9839 -	-	-	1	1	2
9734	9731	9734 -	9878 -	-	-	1	1	2

9734	9731	9734	-	9907	-	-	-	1	1	2
9734	9731	9734	-	9920	-	-	-	1	1	2
9739	9737	9742	-	9854	-	-	+	2	0	2
9739	9737	9742	-	9898	-	-	-	1	1	2
9739	9737	9742	-	9928	-	-	-	1	1	2
9739	9737	9742	-	10149	-	-	-	1	1	2
9744	-	-	-	9849	-	-	+	0	2	2
9749	9746	9751	-	9938	-	-	-	1	1	2
9749	9746	9751	-	9944	-	-	-	1	1	2
9749	9746	9751	-	9950	-	-	-	1	1	2
9760	9758	9760	-	9946	-	-	-	1	1	2
9767	9767	9768	-	10029	-	-	-	1	1	2
9773	9773	9775	-	9982	-	-	-	1	1	2
9773	9773	9775	-	10040	-	-	-	1	1	2
9781	9779	9782	-	9814	-	-	+	1	1	2
9792	9792	9795	-	9829	-	-	+	1	1	2
9792	9792	9795	-	10025	-	-	-	1	1	2
9792	9792	9795	-	14346	-	-	-	1	1	2
9797	9797	9798	-	9820	9820	9821	+	1	1	2
9802	9800	9802	-	9936	-	-	-	1	1	2
9817	9817	9819	-	9947	-	-	-	1	1	2
9822	9822	9823	+	10031	10031	10032	-	1	1	2
9824	-	-	-	9974	-	-	-	1	1	2
9830	9830	9834	-	9983	-	-	-	1	1	2
9839	9839	9840	-	10047	-	-	+	1	1	2
9839	-	-	+	9922	-	-	+	1	1	2
9853	9852	9856	-	10032	10032	10033	+	1	1	2
9858	-	-	+	9753	-	-	-	1	1	2
9877	9873	9877	-	10069	-	-	-	1	1	2
9877	9873	9877	-	10088	-	-	+	1	1	2
9886	9882	9888	-	10067	10067	10068	-	1	1	2
9886	9886	9889	+	9830	-	-	-	0	2	2
9886	9886	9889	+	9902	9899	9902	-	1	1	2
9894	9893	9894	+	9824	-	-	-	2	0	2
9898	9898	9901	-	10088	-	-	-	1	1	2
9905	9903	9906	-	9982	-	-	-	1	1	2
9905	9903	9906	-	10083	-	-	-	1	1	2
9905	9903	9906	-	10101	-	-	-	1	1	2
9905	9903	9906	-	10158	-	-	+	1	1	2
9914	9914	9918	-	9976	-	-	+	1	1	2
9914	9914	9918	-	10053	-	-	-	1	1	2
9914	9914	9918	-	10064	-	-	-	1	1	2
9919	9917	9919	+	10001	-	-	+	1	1	2
9919	9917	9919	+	10101	-	-	+	1	1	2
9920	9920	9923	-	10047	-	-	+	1	1	2
9920	9920	9923	-	10064	-	-	-	1	1	2
9920	9920	9923	-	10088	-	-	-	1	1	2
9920	9920	9923	-	10113	-	-	-	1	1	2
9920	9920	9923	-	10120	-	-	-	1	1	2
9929	9926	9929	-	10033	-	-	+	1	1	2
9934	9934	9935	-	9982	9982	9983	-	1	1	2
9935	-	-	+	9954	-	-	-	1	1	2
9942	-	-	+	10021	-	-	-	1	1	2
9948	9948	9951	-	9961	9961	9962	+	1	1	2
9953	-	-	+	10010	10010	10011	-	1	1	2
9956	9956	9959	-	10043	-	-	+	1	1	2
9963	9960	9964	+	9999	9999	10000	-	1	1	2
9963	9960	9964	+	10079	-	-	-	1	1	2
9973	9973	9977	+	9896	-	-	-	1	1	2
9979	9975	9981	-	10115	10115	10116	-	1	1	2
9979	9975	9981	-	10122	-	-	-	1	1	2
9979	9975	9981	-	10144	10144	10145	-	1	1	2
9994	9994	9997	+	15343	-	-	+	0	2	2
9996	9993	9996	-	10182	10180	10182	-	1	1	2
10000	10000	10004	+	9956	-	-	-	2	0	2
10004	10000	10008	-	10136	-	-	-	1	1	2
10004	10000	10008	-	10213	-	-	-	1	1	2
10009	10007	10011	+	10038	10038	10039	-	1	1	2
10016	-	-	+	9947	9947	9948	-	1	1	2
10026	-	-	-	10221	-	-	-	1	1	2
10026	10026	10027	+	10022	10022	10023	-	1	1	2
10031	10028	10031	-	10160	-	-	-	1	1	2
10031	10028	10031	-	10257	-	-	-	1	1	2
10036	10036	10039	-	10012	10010	10012	+	2	0	2
10042	-	-	-	10256	-	-	-	1	1	2
10043	10040	10048	+	10066	10064	10066	-	1	1	2
10043	10040	10048	+	10133	-	-	-	1	1	2
10043	10040	10048	+	11547	-	-	+	1	1	2
10049	10045	10051	-	10197	-	-	-	1	1	2
10049	10045	10051	-	10269	-	-	-	1	1	2
10049	10045	10051	-	10293	-	-	-	1	1	2
10049	10045	10051	-	10301	-	-	+	1	1	2
10059	10059	10060	+	10070	10070	10071	-	1	1	2
10061	10060	10065	-	10220	-	-	-	1	1	2
10061	10060	10065	-	10225	-	-	-	1	1	2
10066	10064	10066	+	10065	10065	10066	-	1	1	2
10072	-	-	+	10137	-	-	-	1	1	2
10081	10079	10085	+	9963	-	-	-	1	1	2
10081	10079	10085	+	10102	-	-	-	1	1	2
10081	10079	10085	+	10154	-	-	-	1	1	2
10081	10079	10085	+	10215	-	-	-	1	1	2
10083	10083	10087	-	10261	-	-	+	1	1	2
10098	10094	10099	-	10233	-	-	-	1	1	2
10098	10094	10099	-	10371	-	-	-	1	1	2
10104	10102	10106	-	10209	-	-	-	1	1	2
10104	10102	10106	-	10339	-	-	-	1	1	2

10111	-	-		-	10331	-	-		-	1	1	2
10120		10117	10121	-	10287	-	-		+	1	1	2
10120		10117	10121	-	10328	-	-		-	1	1	2
10120		10116	10121	+	10208	-	-		-	1	1	2
10125		10125	10129	+	10182	-	-		+	1	1	2
10125		10125	10129	+	10327	-	-		-	1	1	2
10126		10125	10130	-	10165	-	-		+	1	1	2
10126		10125	10130	-	10234	-	-		-	1	1	2
10126		10125	10130	-	10398	-	-		-	1	1	2
10138	-	-		+	10225	-	-		-	1	1	2
10141		10139	10143	-	10175	-	-		+	1	1	2
10141		10139	10143	-	10209		10209	10210	-	1	1	2
10141		10139	10143	-	10329	-	-		-	1	1	2
10141		10139	10143	-	10390	-	-		-	1	1	2
10143		10143	10147	+	10179	-	-		-	0	2	2
10143		10143	10147	+	10210	-	-		+	1	1	2
10151		10151	10154	-	10316	-	-		-	1	1	2
10151		10151	10154	-	10398	-	-		-	1	1	2
10151	-	-		+	10175	-	-		-	2	0	2
10156		10156	10157	-	10188	-	-		+	1	1	2
10164		10164	10169	-	10209	-	-		+	1	1	2
10164		10164	10169	-	10329	-	-		-	1	1	2
10175		10171	10179	-	10233		10231	10233	-	1	1	2
10175		10171	10179	-	10349		10349	10350	-	1	1	2
10195		10194	10198	-	10459	-	-		-	1	1	2
10206		10202	10208	-	10294	-	-		-	1	1	2
10206		10202	10208	-	10414	-	-		-	1	1	2
10206		10202	10208	-	10515	-	-		-	1	1	2
10206		10202	10208	-	10586		10586	10587	-	1	1	2
10206		10202	10206	+	10125		10125	10126	-	1	1	2
10211		10210	10214	-	10338	-	-		-	1	1	2
10219		10216	10221	-	10347	-	-		-	1	1	2
10219		10216	10221	-	10386	-	-		+	1	1	2
10219		10216	10221	-	10459	-	-		-	1	1	2
10230	-	-		-	10402	-	-		-	1	1	2
10241	-	-		+	10259	-	-		-	1	1	2
10242		10242	10243	-	10426	-	-		-	1	1	2
10249		10245	10250	+	10301	-	-		-	1	1	2
10249		10245	10250	+	10320		10320	10321	-	1	1	2
10249		10245	10250	+	10362	-	-		-	1	1	2
10249		10245	10250	+	10383	-	-		-	1	1	2
10251		10251	10255	-	10347		10347	10348	+	1	1	2
10251		10251	10255	-	10471	-	-		-	1	1	2
10256		10256	10260	+	10310		10310	10311	-	1	1	2
10259		10257	10261	-	10432	-	-		-	1	1	2
10259		10257	10261	-	14578	-	-		+	1	1	2
10273		10272	10277	-	10403	-	-		-	1	1	2
10273		10272	10277	-	10411	-	-		-	1	1	2
10287		10283	10292	-	10402	-	-		-	1	1	2
10287		10283	10292	-	10438	-	-		-	1	1	2
10297	-	-		-	10489	-	-		-	1	1	2
10305		10301	10305	-	10448	-	-		-	1	1	2
10305		10301	10305	-	10458	-	-		-	1	1	2
10316	-	-		-	10482	-	-		+	1	1	2
10324		10324	10326	-	10460	-	-		-	1	1	2
10324		10324	10326	-	10495		10495	10496	+	1	1	2
10327	-	-		+	10443	-	-		+	1	1	2
10329		10329	10330	-	10469		10469	10470	+	1	1	2
10334	-	-		-	10466	-	-		+	0	2	2
10343		10342	10344	-	10498	-	-		-	1	1	2
10361		10358	10364	-	10527	-	-		-	1	1	2
10361		10358	10364	-	12522	-	-		-	1	1	2
10370		10369	10374	-	10534	-	-		-	1	1	2
10386	-	-		+	10221	-	-		-	1	1	2
10390		10388	10390	-	10492		10490	10492	+	1	1	2
10398		10394	10403	-	10498	-	-		+	1	1	2
10398		10394	10403	-	10574	-	-		-	1	1	2
10398		10394	10403	-	10583	-	-		-	1	1	2
10398		10394	10403	-	13047		13047	13048	+	0	2	2
10402		10399	10402	+	10448	-	-		-	1	1	2
10402		10399	10402	+	10636	-	-		+	0	2	2
10408		10405	10410	-	10554	-	-		-	1	1	2
10408		10405	10410	-	10562	-	-		-	1	1	2
10408		10405	10410	-	10571	-	-		-	1	1	2
10408		10405	10410	-	10644		10642	10644	-	0	2	2
10408	-	-		+	10642	-	-		+	2	0	2
10416		10413	10416	-	10496	-	-		+	1	1	2
10416		10413	10416	-	10589	-	-		+	1	1	2
10416		10413	10416	-	10622	-	-		-	1	1	2
10425		10422	10429	-	10526	-	-		-	1	1	2
10425		10422	10429	-	10585	-	-		-	1	1	2
10425		10422	10429	-	10613	-	-		-	1	1	2
10425		10422	10429	-	10630	-	-		-	1	1	2
10425		10422	10429	-	10636		10636	10637	-	1	1	2
10425		10422	10429	-	10728		10728	10731	-	1	1	2
10436		10433	10436	-	10627	-	-		-	1	1	2
10438	-	-		+	10624	-	-		+	1	1	2
10448		10448	10453	-	10629	-	-		-	1	1	2
10459		10455	10459	-	10659	-	-		-	1	1	2
10472		10469	10477	-	10551		10551	10552	+	1	1	2
10472		10469	10477	-	10579		10575	10579	+	1	1	2
10472		10469	10477	-	10613	-	-		-	1	1	2
10475	-	-		+	10323		10323	10324	-	1	1	2
10480	-	-		-	10539	-	-		+	1	1	2
10483		10483	10484	+	10543	-	-		-	1	1	2
10483		10483	10484	+	10754	-	-		+	1	1	2

10485		10483	10487	-	10581	-	-		-	2	0	2
10485		10483	10487	-	10595	-	-		-	1	1	2
10485		10483	10487	-	10610	-	-		-	1	1	2
10485		10483	10487	-	10673	-	-		-	1	1	2
10490		10490	10493	-	10596	-	-		-	1	1	2
10490		10490	10493	-	10637	-	-		-	1	1	2
10490		10490	10493	-	10673	-	-		-	1	1	2
10490		10490	10493	-	10699	-	-		-	1	1	2
10508		10508	10509	+	10462		10462	10463	-	1	1	2
10510		10510	10513	-	10751	-	-		-	1	1	2
10522		10522	10523	+	10645	-	-		+	1	1	2
10525		10523	10528	-	10539		10539	10540	+	1	1	2
10525		10523	10528	-	10631	-	-		-	1	1	2
10525		10523	10528	-	10660		10660	10661	-	1	1	2
10539	-	-		-	10676	-	-		-	1	1	2
10543	-	-		+	10483	-	-		-	1	1	2
10544	-	-		-	10637	-	-		+	2	0	2
10549		10547	10553	-	10634	-	-		+	0	2	2
10549		10547	10553	-	10697	-	-		-	1	1	2
10549		10547	10553	-	10787	-	-		-	1	1	2
10568		10568	10569	+	10617	-	-		-	1	1	2
10568		10568	10569	+	10751	-	-		-	1	1	2
10574	-	-		+	10613	-	-		-	0	2	2
10575		10571	10575	-	10723	-	-		+	1	1	2
10580		10580	10583	-	10604		10601	10604	+	1	1	2
10580		10580	10583	-	10706	-	-		-	1	1	2
10580		10580	10583	-	10736	-	-		-	1	1	2
10580		10580	10583	-	10765		10765	10766	-	1	1	2
10582		10578	10585	+	10579		10579	10580	-	1	1	2
10582		10578	10585	+	10609	-	-		-	2	0	2
10582		10578	10585	+	10736	-	-		-	1	1	2
10587		10587	10590	+	10616	-	-		+	1	1	2
10590		10588	10591	-	10740	-	-		-	1	1	2
10590		10588	10591	-	10777		10774	10777	-	1	1	2
10596		10594	10599	+	10664	-	-		-	1	1	2
10606		10604	10606	-	10666	-	-		+	1	1	2
10606		10604	10606	-	10729	-	-		-	1	1	2
10606		10604	10606	-	10765	-	-		-	1	1	2
10615	-	-		+	10773	-	-		-	1	1	2
10617	-	-		-	10812	-	-		-	1	1	2
10628		10626	10631	-	10738	-	-		+	1	1	2
10628		10626	10631	-	10787	-	-		-	1	1	2
10628		10626	10631	-	10813	-	-		-	1	1	2
10628		10626	10631	-	10820	-	-		-	1	1	2
10631	-	-		+	10515	-	-		-	1	1	2
10643		10643	10645	+	10691		10691	10692	-	1	1	2
10650		10646	10650	-	10682		10678	10682	-	1	1	2
10657		10655	10659	+	10515	-	-		-	1	1	2
10673	-	-		-	10692	-	-		+	1	1	2
10673	-	-		-	10816	-	-		-	1	1	2
10676		10672	10678	+	10617	-	-		-	1	1	2
10688	-	-		+	10706	-	-		-	1	1	2
10694		10694	10695	-	11177	-	-		-	1	1	2
10706		10706	10710	+	14864		14861	14864	-	0	2	2
10721		10717	10724	+	10699	-	-		-	2	0	2
10721		10717	10724	+	10705	-	-		-	0	2	2
10724		10723	10727	-	10934		10932	10934	-	1	1	2
10729	-	-		-	12687	-	-		+	1	1	2
10743		10743	10746	+	10797		10795	10797	+	0	2	2
10743		10743	10746	+	10803	-	-		-	1	1	2
10746		10746	10748	-	10902	-	-		-	1	1	2
10752	-	-		+	10803	-	-		+	2	0	2
10756		10754	10759	-	10782	-	-		+	1	1	2
10756		10754	10759	-	10907	-	-		-	1	1	2
10768		10768	10771	+	10728	-	-		-	1	1	2
10768		10768	10771	+	12666	-	-		-	1	1	2
10771		10768	10776	-	10819	-	-		+	1	1	2
10771		10768	10776	-	10831	-	-		+	1	1	2
10771		10768	10776	-	10886	-	-		-	1	1	2
10771		10768	10776	-	10904	-	-		-	1	1	2
10782	-	-		-	12284	-	-		-	2	0	2
10789		10789	10790	+	10729	-	-		-	1	1	2
10797		10796	10798	-	10855	-	-		+	1	1	2
10797		10796	10798	-	10876	-	-		-	1	1	2
10798	-	-		+	12301	-	-		+	1	1	2
10812		10809	10812	+	10759		10756	10759	-	1	1	2
10821	-	-		-	10896	-	-		+	1	1	2
10821		10818	10821	+	10873		10873	10874	-	1	1	2
10821		10818	10821	+	10896	-	-		-	1	1	2
10830		10829	10832	+	10861		10861	10862	-	1	1	2
10830		10829	10832	+	12331		12331	12332	+	2	0	2
10835		10835	10839	-	11278	-	-		+	1	1	2
10835		10835	10839	-	13123	-	-		-	1	1	2
10836		10836	10839	+	10849	-	-		+	1	1	2
10842	-	-		-	10872	-	-		+	1	1	2
10850		10848	10852	-	11035		11035	11036	-	1	1	2
10850		10848	10852	-	11053		11049	11053	-	1	1	2
10850		10848	10852	-	11612	-	-		-	1	1	2
10871		10867	10871	-	11024	-	-		-	1	1	2
10871		10867	10871	-	11075	-	-		-	1	1	2
10876		10873	10878	-	11019	-	-		-	1	1	2
10876		10873	10878	-	11037	-	-		-	1	1	2
10888	-	-		-	11106	-	-		-	1	1	2
10902		10898	10903	-	11040		11040	11041	-	1	1	2
10915	-	-		-	11557	-	-		+	2	0	2
10944		10940	10944	-	11158	-	-		-	1	1	2

10987	10987	10989	+	11163	11161	11163	-	1	1	2
10990	10986	10994	-	11118	-	-	-	2	0	2
10990	10986	10994	-	11177	-	-	-	1	1	2
10990	10986	10994	-	11214	-	-	-	1	1	2
10996	-	-	-	11121	-	-	-	0	2	2
11005	11005	11008	-	11151	-	-	-	1	1	2
11010	11009	11010	-	11135	-	-	-	1	1	2
11024	11021	11024	-	11041	-	-	+	1	1	2
11024	11021	11024	-	11079	11076	11079	+	1	1	2
11024	11021	11024	-	11439	-	-	-	1	1	2
11032	11030	11035	-	11086	-	-	+	1	1	2
11032	11030	11035	-	11151	-	-	-	1	1	2
11039	11039	11040	-	11154	-	-	-	1	1	2
11039	11039	11040	-	11177	-	-	-	1	1	2
11044	11044	11046	-	11097	11095	11097	+	1	1	2
11044	11044	11046	-	13759	-	-	-	1	1	2
11061	11057	11061	-	11190	-	-	-	1	1	2
11071	-	-	-	11232	-	-	-	1	1	2
11082	11079	11086	-	11155	11152	11155	+	1	1	2
11082	11079	11086	-	11198	11198	11199	+	1	1	2
11082	11079	11086	-	11247	-	-	-	1	1	2
11085	-	-	+	11332	-	-	-	1	1	2
11094	-	-	-	11296	-	-	-	1	1	2
11096	-	-	+	11187	11187	11188	-	1	1	2
11102	11100	11107	-	11271	-	-	-	1	1	2
11102	11100	11107	-	11305	-	-	-	1	1	2
11109	11108	11109	-	11239	-	-	-	1	1	2
11114	11113	11116	-	11180	11177	11180	+	1	1	2
11114	11113	11116	-	11275	-	-	+	1	1	2
11127	11123	11131	-	11136	-	-	+	2	0	2
11127	11123	11131	-	11254	-	-	-	1	1	2
11127	11123	11131	-	11274	-	-	-	1	1	2
11127	11123	11131	-	11299	-	-	-	1	1	2
11136	11134	11139	-	11196	-	-	+	1	1	2
11136	11134	11139	-	11262	-	-	-	1	1	2
11144	-	-	-	11229	-	-	+	1	1	2
11152	-	-	-	11221	-	-	+	1	1	2
11163	-	-	+	11136	-	-	-	1	1	2
11166	11166	11167	-	11206	11206	11207	+	1	1	2
11173	11170	11173	+	11270	-	-	-	1	1	2
11174	11172	11174	-	11227	-	-	+	1	1	2
11174	11172	11174	-	11283	-	-	-	1	1	2
11174	11172	11174	-	11364	-	-	-	1	1	2
11178	-	-	+	11199	-	-	+	1	1	2
11186	-	-	-	11281	-	-	-	1	1	2
11193	11191	11193	-	11389	-	-	-	1	1	2
11193	11191	11193	-	11411	-	-	-	1	1	2
11199	11195	11204	-	11272	-	-	-	1	1	2
11199	11195	11204	-	11299	-	-	-	1	1	2
11199	11195	11204	-	11346	-	-	+	0	2	2
11199	11195	11204	-	11371	-	-	-	1	1	2
11199	11195	11204	-	11398	11394	11398	-	1	1	2
11199	11195	11204	-	11412	-	-	-	1	1	2
11199	11195	11204	-	11515	-	-	-	1	1	2
11209	11209	11211	-	11336	-	-	+	1	1	2
11221	11221	11226	-	11412	-	-	-	1	1	2
11221	11221	11226	-	11442	-	-	-	1	1	2
11227	-	-	+	11172	-	-	-	1	1	2
11235	11231	11238	-	11297	-	-	+	1	1	2
11235	11231	11238	-	11367	11367	11369	-	1	1	2
11235	11231	11238	-	11401	11399	11401	-	1	1	2
11244	11243	11245	-	11312	-	-	+	1	1	2
11244	11243	11245	-	11327	-	-	-	2	0	2
11244	11243	11245	-	11385	-	-	-	1	1	2
11250	-	-	-	11332	-	-	-	0	2	2
11252	11248	11255	+	11155	11152	11155	+	1	1	2
11252	11248	11255	+	11424	-	-	-	1	1	2
11255	11253	11256	-	11303	-	-	+	1	1	2
11255	11253	11256	-	11403	-	-	-	1	1	2
11255	11253	11256	-	11449	-	-	-	1	1	2
11261	11261	11264	-	11390	-	-	-	1	1	2
11261	11261	11264	-	11415	-	-	+	1	1	2
11266	11266	11271	+	11176	11176	11177	-	1	1	2
11269	11265	11273	-	11297	-	-	+	1	1	2
11269	11265	11273	-	11304	-	-	+	1	1	2
11269	11265	11273	-	11420	11420	11421	-	1	1	2
11269	11265	11273	-	11448	-	-	-	2	0	2
11269	11265	11273	-	11465	-	-	-	1	1	2
11282	11278	11287	-	11431	-	-	-	1	1	2
11282	11278	11287	-	11442	-	-	-	1	1	2
11285	-	-	+	11306	-	-	-	1	1	2
11295	11292	11295	-	11347	11347	11348	+	1	1	2
11295	11292	11295	-	11446	-	-	-	1	1	2
11295	11292	11295	-	11465	-	-	-	1	1	2
11302	11300	11303	-	11451	-	-	-	1	1	2
11302	11300	11303	-	13842	-	-	-	1	1	2
11308	11308	11311	-	11458	-	-	-	2	0	2
11318	11314	11321	-	11409	-	-	+	1	1	2
11318	11314	11321	-	11421	-	-	-	1	1	2
11318	11314	11321	-	11453	-	-	-	1	1	2
11318	11314	11321	-	11465	-	-	-	0	2	2
11318	11314	11321	-	11731	-	-	-	1	1	2
11320	11316	11323	+	11284	11281	11284	-	1	1	2
11320	11316	11323	+	11381	-	-	-	1	1	2
11320	11316	11323	+	11523	-	-	-	1	1	2
11323	11323	11327	-	11474	-	-	-	1	1	2

11323	11323	11327 -	11480 -	-	-	1	1	2
11339	11338	11342 -	11441	11437	11441 -	1	1	2
11339	11338	11342 -	11498 -	-	-	1	1	2
11339	11338	11342 -	11542 -	-	-	1	1	2
11344 -	-	-	11524 -	-	-	1	1	2
11355 -	-	+	11335 -	-	-	1	1	2
11362	11362	11363 +	11434 -	-	+	1	1	2
11362	11362	11363 +	11443 -	-	-	1	1	2
11365	11361	11369 -	11434 -	-	-	1	1	2
11365	11361	11369 -	11529 -	-	-	1	1	2
11365	11361	11369 -	11597	11594	11597 -	1	1	2
11369 -	-	+	11800 -	-	-	1	1	2
11371	11371	11374 -	11383 -	-	+	1	1	2
11371	11371	11374 -	11532 -	-	-	1	1	2
11371	11371	11374 -	11577 -	-	-	1	1	2
11374	11371	11374 +	13683 -	-	-	0	2	2
11374	11371	11374 +	13848 -	-	+	0	2	2
11381	11378	11384 -	11514 -	-	-	1	1	2
11381	11378	11384 -	11543	11543	11544 -	1	1	2
11385	11381	11390 +	11323 -	-	-	1	1	2
11385	11381	11390 +	11353 -	-	-	1	1	2
11385	11381	11390 +	11401	11401	11402 +	1	1	2
11385	11381	11390 +	11779 -	-	-	1	1	2
11386 -	-	-	11538 -	-	-	1	1	2
11410	11408	11410 -	11579 -	-	-	1	1	2
11410	11408	11410 -	11594 -	-	-	1	1	2
11424	11420	11428 -	11581 -	-	-	1	1	2
11429	11429	11430 +	11445	11445	11446 -	1	1	2
11434 -	-	-	11573 -	-	-	1	1	2
11444	11444	11445 -	11586 -	-	-	1	1	2
11444	11444	11445 -	11643 -	-	-	1	1	2
11449	11447	11452 +	11372	11372	11373 -	1	1	2
11449	11447	11452 +	12082 -	-	-	2	0	2
11452	11451	11456 -	11642 -	-	-	2	0	2
11452	11451	11456 -	11647 -	-	-	0	2	2
11457	11454	11457 +	11417 -	-	-	1	1	2
11457	11454	11457 +	11497 -	-	-	1	1	2
11462	11461	11462 -	11603 -	-	+	1	1	2
11462	11461	11462 -	11651	11651	11652 -	1	1	2
11467 -	-	-	11686 -	-	-	1	1	2
11476 -	-	+	11408 -	-	-	1	1	2
11486 -	-	-	11652 -	-	-	1	1	2
11496	11493	11499 +	11488 -	-	+	1	1	2
11496	11493	11499 +	11617 -	-	+	1	1	2
11500	11495	11504 -	11560 -	-	+	1	1	2
11500	11495	11504 -	11580 -	-	-	1	1	2
11508	11506	11512 -	11556	11552	11556 +	1	1	2
11522	11518	11526 -	11667 -	-	-	1	1	2
11522	11518	11526 -	11678 -	-	-	1	1	2
11522	11518	11526 -	11687 -	-	-	1	1	2
11529	11528	11531 -	11543 -	-	+	0	2	2
11529	11528	11531 -	11575 -	-	-	1	1	2
11538	11536	11539 -	11620 -	-	-	1	1	2
11538	11536	11539 -	11694 -	-	-	1	1	2
11538	11536	11539 -	11703 -	-	-	1	1	2
11549	11547	11552 -	11610 -	-	-	1	1	2
11549	11547	11552 -	11666	11666	11667 -	1	1	2
11549	11547	11552 -	11709 -	-	-	1	1	2
11549	11547	11552 -	11735 -	-	-	1	1	2
11561	11558	11564 -	11601	11599	11601 +	1	1	2
11561	11558	11564 -	11651 -	-	-	1	1	2
11561	11558	11564 -	11716 -	-	-	1	1	2
11561	11558	11564 -	11731 -	-	-	1	1	2
11561	11558	11564 -	11839 -	-	-	2	0	2
11569	11566	11571 -	11635 -	-	+	1	1	2
11569	11566	11571 -	11845 -	-	-	0	2	2
11577	11573	11577 -	11667 -	-	-	1	1	2
11577	11573	11577 -	11717 -	-	-	1	1	2
11577	11573	11577 -	11733	11733	11734 -	1	1	2
11577	11573	11577 -	11761 -	-	-	1	1	2
11592	11588	11596 -	11719 -	-	-	1	1	2
11592	11588	11596 -	11758	11755	11758 -	1	1	2
11592	11588	11596 -	11777 -	-	-	1	1	2
11604 -	-	+	11657 -	-	-	1	1	2
11610	11606	11610 -	11613 -	-	+	1	1	2
11610	11606	11610 -	11773 -	-	-	1	1	2
11610	11606	11610 -	11792 -	-	-	1	1	2
11610	11606	11610 -	11803 -	-	-	1	1	2
11610	11606	11610 -	12131 -	-	-	1	1	2
11622	11622	11625 -	11726 -	-	+	2	0	2
11622	11622	11625 -	11754	11754	11755 -	1	1	2
11622	11622	11625 -	11764 -	-	-	1	1	2
11622	11622	11625 -	11817 -	-	-	1	1	2
11622	11622	11625 -	11833 -	-	-	1	1	2
11625	11622	11625 +	11647 -	-	+	1	1	2
11630	11629	11631 -	11721 -	-	+	0	2	2
11630	11629	11631 -	11763	11761	11763 -	1	1	2
11636	11633	11639 +	11641	11641	11642 -	1	1	2
11636	11633	11639 +	11648 -	-	-	1	1	2
11637	11637	11641 -	13750 -	-	+	1	1	2
11637	11637	11641 -	14238 -	-	+	1	1	2
11654 -	-	-	11704 -	-	+	2	0	2
11654	11653	11655 +	11704 -	-	-	1	1	2
11654	11653	11655 +	12930	12930	12931 -	1	1	2
11659	11656	11659 -	11699 -	-	+	0	2	2
11659	11656	11659 -	11708 -	-	+	1	1	2

11664	-	-	+	11703	-	-	-	1	1	2
11678	-	-	+	11695	-	-	-	1	1	2
11684	11684	11685	+	11688	11688	11689	-	1	1	2
11687	11684	11687	-	11857	-	-	-	1	1	2
11687	11684	11687	-	11882	-	-	-	1	1	2
11692	11692	11693	+	11636	-	-	-	1	1	2
11692	11692	11693	+	11680	11680	11681	-	1	1	2
11697	11695	11697	+	11678	-	-	-	1	1	2
11706	-	-	+	11768	-	-	-	1	1	2
11711	11710	11711	+	11738	-	-	+	1	1	2
11720	-	-	+	11641	-	-	-	1	1	2
11726	11723	11727	-	11922	-	-	-	1	1	2
11726	11724	11728	+	11696	-	-	-	1	1	2
11726	11724	11728	+	11938	-	-	-	1	1	2
11731	11731	11734	+	11619	-	-	-	1	1	2
11732	-	-	-	11905	-	-	-	1	1	2
11737	-	-	-	11797	-	-	+	1	1	2
11741	11741	11745	+	11756	11752	11756	-	1	1	2
11742	-	-	-	12619	-	-	+	1	1	2
11755	11752	11757	+	11745	11741	11745	-	1	1	2
11761	11760	11763	-	11904	11904	11905	-	1	1	2
11761	11760	11763	-	11936	-	-	-	1	1	2
11768	11767	11769	+	11722	11720	11722	-	1	1	2
11781	11781	11789	-	11944	-	-	-	1	1	2
11781	11781	11789	-	11953	-	-	-	1	1	2
11781	11781	11789	-	11973	-	-	-	1	1	2
11781	11781	11789	-	11987	-	-	-	1	1	2
11792	11792	11796	-	12696	-	-	+	1	1	2
11796	-	-	+	11859	11859	11860	-	1	1	2
11803	-	-	-	13453	-	-	-	2	0	2
11827	11825	11829	-	11973	-	-	-	1	1	2
11827	11825	11829	-	12002	12002	12003	-	1	1	2
11832	-	-	-	11973	-	-	-	1	1	2
11840	11837	11842	-	12002	-	-	-	1	1	2
11840	11837	11842	-	12016	-	-	-	1	1	2
11840	11837	11842	-	12133	-	-	-	1	1	2
11856	11852	11856	-	11942	-	-	+	1	1	2
11861	11860	11861	-	12013	-	-	-	1	1	2
11874	11873	11875	-	11781	-	-	+	2	0	2
11874	11873	11875	-	12016	-	-	-	1	1	2
11881	11878	11884	-	12087	-	-	-	1	1	2
11893	-	-	-	11908	-	-	+	1	1	2
11899	11897	11902	-	11966	-	-	+	1	1	2
11899	11897	11902	-	11985	-	-	-	1	1	2
11904	11904	11905	-	12061	-	-	-	1	1	2
11904	11904	11905	-	12210	-	-	-	1	1	2
11915	11915	11916	-	11928	-	-	+	1	1	2
11920	-	-	-	11943	-	-	+	1	1	2
11920	-	-	-	12044	-	-	+	1	1	2
11921	11921	11922	+	11880	11880	11881	-	1	1	2
11931	11929	11934	-	12131	12127	12131	-	1	1	2
11931	11929	11934	-	12181	-	-	-	1	1	2
11942	11940	11943	-	11993	-	-	+	1	1	2
11942	11940	11943	-	12110	-	-	-	1	1	2
11942	11940	11943	-	12218	-	-	-	1	1	2
11943	11943	11944	+	11922	-	-	-	1	1	2
11943	11943	11944	+	11971	11971	11972	-	1	1	2
11949	11949	11950	+	11986	11986	11987	-	1	1	2
11953	11951	11956	-	11971	11967	11971	+	1	1	2
11956	11953	11958	+	11932	-	-	-	1	1	2
11956	11953	11958	+	12029	-	-	-	2	0	2
11958	11957	11958	-	12496	-	-	-	1	1	2
11963	11963	11964	-	12547	12547	12548	+	1	1	2
11968	11967	11970	-	12239	12236	12239	-	0	2	2
11972	11971	11976	+	12079	12079	12080	-	1	1	2
11976	11974	11976	-	11942	-	-	+	2	0	2
11976	11974	11976	-	12092	-	-	+	1	1	2
11978	11978	11980	+	12068	12068	12069	-	1	1	2
11983	11981	11983	-	12085	-	-	+	1	1	2
11983	11981	11983	-	12104	-	-	-	1	1	2
11983	11981	11983	-	12145	-	-	-	1	1	2
11988	-	-	-	12033	-	-	+	1	1	2
11988	11987	11992	+	11881	-	-	-	1	1	2
11988	11987	11992	+	12207	12203	12207	-	1	1	2
11993	11990	11995	-	12021	12017	12021	-	1	1	2
12005	-	-	-	12171	-	-	-	1	1	2
12010	12007	12014	-	12346	-	-	+	1	1	2
12010	12007	12014	-	13081	-	-	-	1	1	2
12014	12014	12018	+	12031	12027	12031	-	1	1	2
12014	12014	12018	+	12276	-	-	-	1	1	2
12018	-	-	-	12182	-	-	-	1	1	2
12027	12027	12028	-	12051	12051	12052	+	1	1	2
12027	12027	12028	-	12093	12093	12094	+	1	1	2
12033	12029	12033	+	12154	-	-	-	1	1	2
12038	12037	12038	+	12451	12451	12452	+	1	1	2
12041	12040	12044	-	12085	-	-	-	2	0	2
12041	12040	12044	-	12210	-	-	-	1	1	2
12041	12040	12044	-	12265	-	-	-	1	1	2
12047	12047	12050	-	12183	-	-	-	1	1	2
12047	12047	12050	-	12197	-	-	-	1	1	2
12047	12047	12050	-	12508	-	-	-	1	1	2
12053	12051	12054	-	12157	12157	12158	-	1	1	2
12053	12051	12054	-	12197	-	-	-	1	1	2
12053	12051	12054	-	12211	-	-	-	1	1	2
12053	12051	12054	-	12333	-	-	+	1	1	2
12058	12056	12063	-	12097	-	-	+	0	2	2

12058		12056	12063	-	12215	-	-		-	1	1	2
12058		12056	12063	-	12227	-	-		-	1	1	2
12058		12056	12063	-	12236	-	-		+	1	1	2
12058		12056	12063	-	12256	-	-		-	1	1	2
12058		12056	12063	-	12282		12278	12282	-	1	1	2
12059		12059	12060	+	12207		12207	12208	-	1	1	2
12068		12068	12073	-	12121		12119	12121	+	1	1	2
12068		12068	12073	-	12384	-	-		+	1	1	2
12070	-	-		+	12128	-	-		-	1	1	2
12076		12075	12076	-	12110	-	-		+	2	0	2
12076		12075	12076	-	12130	-	-		+	1	1	2
12076		12075	12076	-	12341	-	-		+	2	0	2
12082		12079	12086	-	12105	-	-		+	0	2	2
12082		12079	12086	-	12134	-	-		+	1	1	2
12082		12079	12086	-	12209	-	-		-	1	1	2
12082		12079	12086	-	12222	-	-		+	1	1	2
12082		12079	12086	-	12228	-	-		-	1	1	2
12082		12079	12086	-	12273	-	-		-	1	1	2
12082		12079	12086	-	12335	-	-		+	0	2	2
12082		12080	12087	+	12234	-	-		-	1	1	2
12082		12080	12087	+	14574		14572	14574	-	0	2	2
12082		12080	12087	+	14622		14620	14622	-	0	2	2
12082		12080	12087	+	15130	-	-		+	0	2	2
12091		12090	12095	+	11966		11966	11967	-	1	1	2
12091		12090	12095	+	12119	-	-		-	1	1	2
12092		12087	12098	-	12179	-	-		+	1	1	2
12092		12087	12098	-	12188	-	-		-	1	1	2
12092		12087	12098	-	12256	-	-		-	1	1	2
12092		12087	12098	-	12280	-	-		-	1	1	2
12092		12087	12098	-	12289	-	-		+	1	1	2
12092		12087	12098	-	12297	-	-		-	1	1	2
12110		12107	12115	+	12104		12104	12105	-	1	1	2
12110		12107	12115	+	12133	-	-		-	1	1	2
12123		12119	12127	-	12219	-	-		-	1	1	2
12128		12124	12129	+	12256		12256	12257	-	1	1	2
12128		12124	12129	+	12299		12299	12300	-	1	1	2
12129	-	-		-	12209	-	-		-	1	1	2
12129	-	-		-	12240	-	-		-	1	1	2
12134		12133	12137	+	12555	-	-		-	1	1	2
12136		12132	12142	-	12220	-	-		-	1	1	2
12136		12132	12142	-	12335	-	-		+	1	1	2
12136		12132	12142	-	12686		12683	12686	-	1	1	2
12141	-	-		+	12814		12814	12815	+	1	1	2
12147		12143	12147	+	12071	-	-		-	1	1	2
12150		12150	12152	-	12195		12195	12196	+	1	1	2
12159		12155	12161	-	14889	-	-		-	1	1	2
12159		12158	12163	+	12122	-	-		-	0	2	2
12159		12158	12163	+	12130	-	-		-	1	1	2
12168		12165	12169	+	12116	-	-		-	2	0	2
12173		12171	12174	+	12185		12185	12186	+	1	1	2
12173		12171	12174	+	12212		12212	12213	+	1	1	2
12173		12171	12174	+	13090		13088	13090	-	1	1	2
12181	-	-		+	12327	-	-		-	1	1	2
12185		12182	12185	-	12208		12208	12209	+	1	1	2
12191		12188	12192	-	12235		12235	12236	+	1	1	2
12191		12188	12192	-	12246		12244	12246	+	1	1	2
12191		12188	12192	-	12297	-	-		-	1	1	2
12191		12188	12192	-	12367	-	-		-	1	1	2
12192		12192	12193	+	12208	-	-		-	1	1	2
12192		12192	12193	+	12316		12316	12317	-	1	1	2
12196		12195	12199	-	12388	-	-		+	1	1	2
12206		12202	12206	-	12221		12217	12221	+	1	1	2
12206		12202	12206	-	12330	-	-		+	1	1	2
12206		12202	12206	-	12369	-	-		-	1	1	2
12209		12206	12210	+	12060		12060	12061	-	1	1	2
12209		12206	12210	+	12210	-	-		-	1	1	2
12209		12206	12210	+	12283	-	-		-	1	1	2
12215		12215	12218	-	12371	-	-		-	1	1	2
12215		12215	12218	-	12431	-	-		-	1	1	2
12221	-	-		-	12371	-	-		-	1	1	2
12228		12224	12231	-	12377	-	-		+	1	1	2
12228		12224	12231	-	12394	-	-		-	1	1	2
12228		12224	12231	-	12627	-	-		-	1	1	2
12228		12224	12231	-	13459	-	-		+	1	1	2
12238		12234	12241	-	12295		12292	12295	+	1	1	2
12241		12241	12242	+	12228		12228	12229	-	1	1	2
12246		12246	12248	+	12205		12205	12206	-	1	1	2
12248		12244	12252	-	12302	-	-		+	1	1	2
12248		12244	12252	-	12312		12310	12312	+	1	1	2
12248		12244	12252	-	12354	-	-		+	1	1	2
12248		12244	12252	-	12404	-	-		-	1	1	2
12248		12244	12252	-	12424		12421	12424	-	1	1	2
12257		12257	12258	-	12369	-	-		-	1	1	2
12257		12257	12258	-	14162		14162	14163	+	1	1	2
12261		12261	12265	+	12120	-	-		-	1	1	2
12266	-	-		-	12366	-	-		+	1	1	2
12272	-	-		-	12553	-	-		-	1	1	2
12280		12278	12282	-	12690		12686	12690	-	1	1	2
12286		12283	12286	+	12333		12333	12334	+	1	1	2
12288		12286	12291	-	12617		12614	12617	-	1	1	2
12288		12286	12291	-	14732	-	-		+	1	1	2
12294		12293	12294	-	12418		12418	12419	-	1	1	2
12294		12293	12294	-	12489	-	-		-	1	1	2
12294		12293	12294	-	12511	-	-		-	1	1	2
12301		12298	12301	+	12976	-	-		+	1	1	2
12309		12306	12311	-	12338		12335	12338	+	1	1	2

12320	12316	12322	-	12489	-	-		-	1	1	2
12322	12319	12326	+	12681		12681	12682	-	1	1	2
12326	12323	12330	-	12458	-	-		-	1	1	2
12326	12323	12330	-	12485	-	-		-	1	1	2
12326	12323	12330	-	12512	-	-		-	1	1	2
12326	12323	12330	-	12517	-	-		-	1	1	2
12326	12323	12330	-	12565	-	-		-	1	1	2
12326	12323	12330	-	12573	-	-		-	1	1	2
12333	12333	12336	-	12452	-	-		-	0	2	2
12333	12333	12336	-	12504	-	-		-	1	1	2
12333	12333	12336	-	12557	-	-		-	1	1	2
12333	12333	12334	+	12523	-	-		-	1	1	2
12338	12338	12339	+	12317		12317	12318	-	1	1	2
12343	12340	12346	-	12386	-	-		-	1	1	2
12343	12340	12346	-	12459		12459	12462	-	1	1	2
12343	12340	12346	-	12484		12484	12485	-	1	1	2
12343	12340	12346	-	12489	-	-		-	1	1	2
12343	12340	12346	-	12530	-	-		-	1	1	2
12351	12348	12351	-	12546	-	-		-	1	1	2
12374	12370	12375	+	12492		12488	12492	-	1	1	2
12374	12370	12375	+	12566	-	-		-	1	1	2
12374	12370	12375	+	12715	-	-		-	1	1	2
12376	12373	12377	-	12515		12515	12516	-	1	1	2
12376	12373	12377	-	12521	-	-		-	1	1	2
12381	12381	12382	+	12480		12480	12481	-	1	1	2
12388	12387	12388	-	12431	-	-		+	1	1	2
12393	12392	12397	+	12495	-	-		+	1	1	2
12393	12392	12397	+	12645		12645	12646	-	1	1	2
12406 -	-		-	12552	-	-		-	1	1	2
12414	12412	12418	-	12534	-	-		+	1	1	2
12426	12426	12430	+	12417		12417	12418	-	1	1	2
12426	12426	12430	+	12463		12463	12464	+	1	1	2
12428	12424	12431	-	12524	-	-		+	1	1	2
12441 -	-		+	12397	-	-		-	1	1	2
12454	12454	12457	-	12476	-	-		+	1	1	2
12454	12454	12457	-	12668	-	-		-	1	1	2
12466	12462	12466	+	12486	-	-		-	1	1	2
12473	12471	12477	-	12528		12524	12528	+	1	1	2
12473	12471	12477	-	12709	-	-		-	1	1	2
12473	12471	12477	-	12911	-	-		+	1	1	2
12473	12471	12477	-	13953	-	-		-	1	1	2
12481 -	-		-	12520	-	-		+	1	1	2
12488	12488	12489	-	12570	-	-		-	1	1	2
12488	12488	12489	-	12711	-	-		-	2	0	2
12494	12494	12496	-	12635	-	-		-	1	1	2
12494	12494	12496	-	12716	-	-		-	0	2	2
12506	12506	12508	-	12627	-	-		+	1	1	2
12506	12506	12508	-	12945	-	-		-	1	1	2
12506	12505	12506	+	12945	-	-		+	1	1	2
12513	12510	12513	+	12451	-	-		-	1	1	2
12515	12512	12519	-	12605	-	-		-	2	0	2
12515	12512	12519	-	12612	-	-		-	0	2	2
12515	12512	12519	-	12640	-	-		-	1	1	2
12515	12512	12519	-	12741	-	-		-	2	0	2
12529	12526	12530	-	12630	-	-		-	1	1	2
12529	12526	12530	-	12661	-	-		-	1	1	2
12529	12526	12530	-	12708	-	-		-	1	1	2
12536	12533	12537	-	12924		12920	12924	-	1	1	2
12542	12542	12546	+	12489	-	-		-	1	1	2
12552	12552	12553	+	12473		12473	12474	-	0	2	2
12557	12557	12560	+	12599	-	-		-	1	1	2
12557	12557	12560	+	12683		12683	12684	-	1	1	2
12578	12578	12579	+	12597		12597	12598	-	1	1	2
12585	12584	12589	-	12757		12755	12757	-	1	1	2
12585	12584	12589	-	12769	-	-		-	1	1	2
12596	12595	12597	-	12720	-	-		-	1	1	2
12596	12595	12597	-	12783	-	-		-	1	1	2
12603	12600	12603	-	12688	-	-		+	1	1	2
12608	12606	12611	-	12709	-	-		-	1	1	2
12608	12606	12611	-	12736	-	-		-	1	1	2
12608	12606	12611	-	12761	-	-		-	1	1	2
12608	12606	12611	-	12778	-	-		-	1	1	2
12620	12616	12620	-	12755		12751	12755	-	1	1	2
12620	12616	12620	-	12774	-	-		-	1	1	2
12626	12622	12630	-	12651	-	-		+	2	0	2
12626	12622	12630	-	12750	-	-		-	1	1	2
12626	12622	12630	-	12756	-	-		-	1	1	2
12626	12622	12630	-	12779		12779	12780	-	1	1	2
12626	12622	12630	-	12817	-	-		-	1	1	2
12626	12624	12628	+	12635	-	-		-	1	1	2
12633	12633	12637	+	13233	-	-		+	1	1	2
12641	12636	12645	-	12798		12798	12801	-	1	1	2
12657	12657	12661	-	12802	-	-		-	1	1	2
12661 -	-		+	12615	-	-		-	1	1	2
12667	12667	12670	-	12817	-	-		-	1	1	2
12667	12667	12670	-	12858	-	-		+	1	1	2
12667	12667	12670	-	12896	-	-		-	2	0	2
12671	12668	12671	+	12561	-	-		-	1	1	2
12671	12668	12671	+	12859		12859	12860	-	1	1	2
12677	12674	12677	-	12706	-	-		+	1	1	2
12677	12674	12677	-	12732	-	-		+	1	1	2
12677	12674	12677	-	12749	-	-		-	1	1	2
12677	12677	12678	+	12706	-	-		-	0	2	2
12677	12677	12678	+	12731		12731	12732	-	1	1	2
12685	12685	12689	+	12718		12718	12719	-	1	1	2
12685	12685	12689	+	13401		13401	13402	-	1	1	2

12701	-	-		+	12921	-	-		+	1	1	2
12706		12703	12711	+	12677	-	-		-	1	1	2
12706		12703	12711	+	12785	-	-		-	1	1	2
12707		12703	12711	-	12719		12716	12719	+	1	1	2
12707		12703	12711	-	12770	-	-		-	2	0	2
12707		12703	12711	-	15223	-	-		-	1	1	2
12715	-	-		-	12774	-	-		-	0	2	2
12725		12723	12725	+	12758	-	-		-	1	1	2
12730		12730	12733	-	12898	-	-		-	1	1	2
12730		12730	12733	-	13061		13061	13062	+	1	1	2
12739		12737	12740	-	13197		13197	13198	-	1	1	2
12747		12745	12751	-	12819	-	-		+	1	1	2
12753		12751	12753	+	12729		12729	12730	-	1	1	2
12753		12751	12753	+	12862	-	-		-	1	1	2
12754		12752	12759	-	12897	-	-		-	1	1	2
12754		12752	12759	-	12936	-	-		-	1	1	2
12758	-	-		+	12723	-	-		-	1	1	2
12767		12765	12771	-	12839		12839	12840	+	1	1	2
12767		12765	12771	-	12915	-	-		-	1	1	2
12767		12765	12771	-	12920	-	-		-	1	1	2
12767		12765	12771	-	12971	-	-		-	1	1	2
12776		12774	12779	-	12932	-	-		-	0	2	2
12776		12774	12779	-	13066	-	-		-	1	1	2
12776		12774	12779	-	13262		13262	13263	+	1	1	2
12785		12781	12787	-	12822	-	-		+	1	1	2
12785		12781	12787	-	12921	-	-		-	1	1	2
12785		12781	12787	-	12926	-	-		-	1	1	2
12785		12781	12787	-	12932	-	-		-	1	1	2
12790		12790	12793	-	12857	-	-		-	1	1	2
12790		12790	12793	-	12958	-	-		-	1	1	2
12799		12797	12799	-	12960	-	-		-	1	1	2
12799		12797	12799	-	12966	-	-		-	1	1	2
12805		12803	12806	-	12913	-	-		+	1	1	2
12805		12803	12806	-	12992	-	-		-	1	1	2
12817		12814	12817	-	12986	-	-		-	1	1	2
12817		12814	12817	-	13145	-	-		-	1	1	2
12818		12817	12822	+	12856	-	-		-	2	0	2
12818		12817	12822	+	12861	-	-		-	0	2	2
12822		12822	12824	-	12960	-	-		-	1	1	2
12830		12827	12830	-	12960	-	-		-	1	1	2
12830		12827	12830	-	12970	-	-		-	1	1	2
12834		12834	12835	+	12707	-	-		-	1	1	2
12837		12834	12841	-	12839	-	-		+	2	0	2
12837		12834	12841	-	13074	-	-		+	1	1	2
12849		12849	12850	+	12839	-	-		-	1	1	2
12849		12849	12850	+	12905	-	-		-	1	1	2
12856		12854	12856	+	12834	-	-		-	1	1	2
12856		12854	12856	+	12898		12898	12899	-	1	1	2
12861		12858	12862	-	12929	-	-		+	1	1	2
12861		12858	12862	-	14499		14496	14499	+	1	1	2
12861		12859	12864	+	12891		12891	12892	-	1	1	2
12878	-	-		+	13061	-	-		-	1	1	2
12879		12879	12882	-	13079	-	-		-	1	1	2
12891		12890	12894	-	13060	-	-		-	1	1	2
12891		12890	12894	-	13131		13128	13131	-	2	0	2
12891		12890	12894	-	13859		13859	13860	-	1	1	2
12893		12892	12897	+	13135	-	-		-	1	1	2
12907		12903	12910	-	13010	-	-		-	1	1	2
12907		12903	12910	-	13042	-	-		-	1	1	2
12907		12903	12910	-	13053	-	-		-	1	1	2
12910		12910	12913	+	12832	-	-		-	1	1	2
12910		12910	12913	+	12974	-	-		-	2	0	2
12918		12918	12919	+	12828		12828	12829	-	1	1	2
12927	-	-		+	13024	-	-		-	1	1	2
12928		12925	12931	-	13021	-	-		+	1	1	2
12928		12925	12931	-	13030	-	-		+	1	1	2
12928		12925	12931	-	13104	-	-		-	1	1	2
12928		12925	12931	-	13114	-	-		-	1	1	2
12935		12932	12935	-	13089	-	-		-	1	1	2
12935		12932	12935	-	13132	-	-		-	1	1	2
12958		12958	12962	-	13014	-	-		+	1	1	2
12962	-	-		+	13090	-	-		+	1	1	2
12974	-	-		+	12913	-	-		-	2	0	2
12980		12980	12982	-	12993	-	-		+	0	2	2
12980		12980	12982	-	13146	-	-		-	1	1	2
12988		12984	12991	-	13117	-	-		-	1	1	2
12988		12984	12991	-	13150	-	-		+	1	1	2
12988		12984	12991	-	13160	-	-		-	1	1	2
12993		12992	12995	-	13017	-	-		+	0	2	2
12993		12992	12995	-	13144	-	-		-	1	1	2
12993		12992	12995	-	13269	-	-		-	1	1	2
12993		12992	12995	-	13543	-	-		-	1	1	2
13001		12997	13001	-	13114	-	-		-	1	1	2
13006		13003	13006	+	13085	-	-		-	1	1	2
13006		13003	13006	+	13154		13154	13155	-	1	1	2
13009		13006	13012	-	13114		13111	13114	+	2	0	2
13011		13010	13016	+	12953	-	-		-	1	1	2
13011		13010	13016	+	13129	-	-		-	2	0	2
13014	-	-		-	13071	-	-		+	1	1	2
13019		13017	13019	-	13061	-	-		+	1	1	2
13019		13018	13022	+	13115		13115	13116	-	1	1	2
13019		13018	13022	+	13340	-	-		-	2	0	2
13024		13021	13027	-	13084	-	-		+	1	1	2
13024		13021	13027	-	13156	-	-		-	1	1	2
13024		13021	13027	-	13166		13166	13167	-	1	1	2
13024		13021	13027	-	13183		13183	13184	-	1	1	2

13024		13021	13027	-	13240	-	-		-	1	1	2
13026		13026	13027	+	13124		13124	13125	-	1	1	2
13033		13030	13035	-	13039	-	-		+	1	1	2
13033		13030	13035	-	13125	-	-		+	1	1	2
13033		13030	13035	-	13162		13162	13163	-	1	1	2
13033		13030	13035	-	13202		13199	13202	-	1	1	2
13039		13037	13041	-	13177	-	-		-	1	1	2
13039		13037	13041	-	13182	-	-		-	1	1	2
13039		13037	13041	-	13210	-	-		-	1	1	2
13039		13037	13041	-	13254	-	-		-	1	1	2
13039		13037	13041	-	13319	-	-		+	1	1	2
13048		13045	13049	-	13105	-	-		+	1	1	2
13048		13045	13049	-	13174	-	-		-	1	1	2
13048		13045	13049	-	13220	-	-		-	1	1	2
13048		13045	13049	-	13258	-	-		-	1	1	2
13056		13050	13058	-	13126	-	-		-	1	1	2
13056		13050	13058	-	13170	-	-		+	1	1	2
13056		13050	13058	-	13173	-	-		-	1	1	2
13056		13050	13058	-	13184	-	-		-	1	1	2
13056		13050	13058	-	13201	-	-		-	1	1	2
13056		13050	13058	-	13211		13211	13212	-	1	1	2
13056		13050	13058	-	15335	-	-		-	1	1	2
13064		13062	13066	+	13100		13096	13100	+	1	1	2
13064		13062	13066	+	13188	-	-		-	1	1	2
13067	-	-		-	13215	-	-		-	1	1	2
13070		13070	13074	+	13116		13114	13116	+	1	1	2
13078	-	-		-	13096	-	-		+	2	0	2
13078	-	-		+	13096	-	-		-	0	2	2
13084		13082	13088	-	13092	-	-		+	0	2	2
13084		13082	13088	-	13443	-	-		-	1	1	2
13085		13082	13085	+	13006	-	-		-	1	1	2
13085		13082	13085	+	13092	-	-		-	2	0	2
13095		13095	13099	-	13195	-	-		-	2	0	2
13095		13095	13099	-	13260	-	-		-	1	1	2
13101		13101	13103	+	12958	-	-		-	2	0	2
13104		13101	13106	-	13198	-	-		-	0	2	2
13104		13101	13106	-	13271	-	-		-	1	1	2
13106		13105	13109	+	12957	-	-		-	0	2	2
13106		13105	13109	+	13200	-	-		-	1	1	2
13113		13109	13117	-	13253	-	-		-	1	1	2
13114		13112	13114	+	13119	-	-		-	1	1	2
13124		13124	13127	-	13289	-	-		-	1	1	2
13127		13127	13129	+	13010	-	-		-	1	1	2
13127		13127	13129	+	13182	-	-		-	1	1	2
13139		13134	13141	+	13170		13170	13171	-	1	1	2
13143		13143	13145	-	13307	-	-		-	1	1	2
13143		13143	13145	-	13336	-	-		-	1	1	2
13148		13146	13150	+	13176		13172	13176	-	1	1	2
13150	-	-		-	13179	-	-		+	2	0	2
13157		13153	13161	-	13176	-	-		+	0	2	2
13157		13153	13161	-	13364	-	-		-	1	1	2
13160		13160	13162	+	13095	-	-		-	1	1	2
13160		13160	13162	+	13663		13663	13664	-	1	1	2
13168		13168	13170	-	13406	-	-		-	1	1	2
13178		13178	13180	-	13213	-	-		-	1	1	2
13178		13178	13180	-	13321	-	-		-	1	1	2
13184		13181	13187	-	13363	-	-		-	1	1	2
13188		13184	13193	+	13519	-	-		-	1	1	2
13189	-	-		-	13351	-	-		-	1	1	2
13189	-	-		-	13371	-	-		-	1	1	2
13199		13198	13201	+	13415	-	-		-	1	1	2
13199		13198	13201	+	13829	-	-		+	1	1	2
13205		13205	13209	+	13188	-	-		+	1	1	2
13205		13205	13209	+	13247		13244	13247	-	1	1	2
13210		13208	13210	-	13616	-	-		-	1	1	2
13215		13213	13219	-	13367	-	-		-	1	1	2
13215		13213	13219	-	13376	-	-		-	1	1	2
13224		13223	13224	-	13376	-	-		-	1	1	2
13224	-	-		+	13058	-	-		-	1	1	2
13236		13236	13238	+	13289	-	-		+	1	1	2
13238		13237	13238	-	13338	-	-		-	1	1	2
13245		13245	13246	+	13310		13310	13311	-	1	1	2
13250	-	-		+	13375	-	-		-	1	1	2
13251		13250	13251	-	13286	-	-		+	1	1	2
13261		13260	13261	+	13261		13261	13262	-	1	1	2
13273	-	-		-	13476	-	-		-	1	1	2
13273	-	-		+	13206	-	-		+	1	1	2
13278	-	-		-	13484	-	-		-	1	1	2
13284		13280	13287	-	13426	-	-		-	1	1	2
13284		13280	13287	-	13448		13448	13449	+	1	1	2
13284		13280	13287	-	13457	-	-		-	1	1	2
13300		13300	13304	-	13483	-	-		-	1	1	2
13303		13303	13307	+	13558	-	-		-	1	1	2
13306	-	-		-	13477	-	-		+	1	1	2
13309	-	-		+	13257	-	-		-	1	1	2
13317		13314	13320	-	13467	-	-		+	1	1	2
13317		13314	13320	-	13476	-	-		-	1	1	2
13317		13314	13320	-	13485	-	-		-	1	1	2
13326	-	-		-	13674	-	-		-	1	1	2
13328		13327	13328	+	13229	-	-		-	1	1	2
13345		13342	13345	+	13356		13356	13357	-	1	1	2
13345		13342	13345	+	13384	-	-		+	1	1	2
13355		13353	13355	-	13535	-	-		-	1	1	2
13359		13356	13359	+	15258		15255	15258	+	1	1	2
13365	-	-		+	13336	-	-		-	1	1	2
13367		13363	13367	-	13392	-	-		+	1	1	2

13367	13363	13367	-	13485 -	-		-	1	1	2
13373 -	-		-	13511 -	-		-	1	1	2
13379 -	-		-	13469 -	-		-	2	0	2
13385	13384	13387	-	13474 -	-		-	0	2	2
13397 -	-		-	13446 -	-		+	1	1	2
13397	13395	13397	+	13446 -	-		-	1	1	2
13397	13395	13397	+	13579 -	-		-	1	1	2
13406	13402	13406	+	13529	13529	13530	-	1	1	2
13406	13402	13406	+	13569 -	-		-	1	1	2
13406	13402	13406	+	13775 -	-		-	1	1	2
13427	13423	13428	-	13852 -	-		+	1	1	2
13434 -	-		+	13611 -	-		-	1	1	2
13437	13437	13441	-	13455 -	-		+	1	1	2
13437	13437	13441	-	13578 -	-		-	1	1	2
13446	13442	13449	-	13693 -	-		-	2	0	2
13465	13465	13466	+	13315	13315	13316	-	1	1	2
13471	13469	13471	-	13473 -	-		+	1	1	2
13471	13469	13471	-	13566 -	-		+	1	1	2
13487 -	-		-	13650 -	-		-	1	1	2
13497	13497	13500	-	13581	13581	13582	+	1	1	2
13501	13501	13505	+	13695	13695	13696	-	1	1	2
13513	13513	13514	+	13569	13569	13570	-	1	1	2
13518 -	-		+	13640 -	-		-	1	1	2
13539	13538	13542	+	13680 -	-		-	1	1	2
13540	13535	13543	-	13646 -	-		-	1	1	2
13540	13535	13543	-	13680 -	-		+	1	1	2
13540	13535	13543	-	13691 -	-		-	1	1	2
13540	13535	13543	-	13713 -	-		-	1	1	2
13540	13535	13543	-	13746 -	-		-	1	1	2
13540	13535	13543	-	13762 -	-		+	1	1	2
13540	13535	13543	-	13786	13786	13788	-	1	1	2
13540	13535	13543	-	13811 -	-		-	1	1	2
13560	13557	13560	-	13763 -	-		-	1	1	2
13560	13557	13560	-	13782	13782	13783	-	1	1	2
13572	13568	13573	-	13610 -	-		+	1	1	2
13572	13568	13573	-	13823 -	-		-	1	1	2
13574	13574	13575	+	13586	13586	13587	-	1	1	2
13583	13581	13584	-	13763 -	-		-	1	1	2
13583	13581	13584	-	13782 -	-		-	1	1	2
13583	13581	13584	-	13790 -	-		-	1	1	2
13588	13586	13589	-	13857 -	-		-	1	1	2
13598	13593	13603	-	13591	13591	13592	+	1	1	2
13598	13593	13603	-	13746 -	-		-	1	1	2
13598	13593	13603	-	13809 -	-		-	1	1	2
13611	13611	13612	+	13545	13545	13546	-	1	1	2
13614	13614	13618	-	13672	13672	13673	+	1	1	2
13614	13614	13618	-	13774 -	-		-	1	1	2
13614	13614	13618	-	13782 -	-		-	1	1	2
13614	13614	13618	-	13801 -	-		-	1	1	2
13617 -	-		+	13575 -	-		-	1	1	2
13621	13620	13626	-	13738 -	-		+	1	1	2
13629	13627	13633	-	13727	13727	13728	+	1	1	2
13629	13627	13633	-	13763 -	-		-	1	1	2
13629	13627	13633	-	13794 -	-		-	1	1	2
13640	13634	13643	-	13766 -	-		-	1	1	2
13640	13634	13643	-	13788 -	-		-	1	1	2
13640	13634	13643	-	14021	14021	14022	-	1	1	2
13640 -	-		+	13518 -	-		-	1	1	2
13646	13645	13648	-	13776 -	-		+	1	1	2
13646	13645	13648	-	13788 -	-		-	1	1	2
13658	13658	13662	-	13696 -	-		+	1	1	2
13658	13658	13662	-	13741 -	-		+	1	1	2
13658	13658	13662	-	13784 -	-		-	1	1	2
13658	13658	13662	-	13797 -	-		-	1	1	2
13658	13658	13662	-	13839 -	-		-	1	1	2
13662	13662	13666	+	13698 -	-		-	1	1	2
13662	13662	13666	+	14122	14122	14123	-	1	1	2
13666	13666	13668	-	13715 -	-		+	1	1	2
13666	13666	13668	-	13807 -	-		-	1	1	2
13684	13683	13686	-	13731 -	-		+	1	1	2
13691	13690	13695	-	13719	13719	13720	+	1	1	2
13702	13699	13702	+	13835	13833	13835	+	1	1	2
13702	13699	13702	+	13884 -	-		-	1	1	2
13705	13702	13708	-	13842 -	-		-	1	1	2
13705	13702	13708	-	13865 -	-		-	1	1	2
13705	13702	13708	-	13906 -	-		-	1	1	2
13707	13705	13707	+	13800 -	-		-	1	1	2
13719	13717	13719	+	13893 -	-		-	1	1	2
13725	13725	13728	+	13666 -	-		+	1	1	2
13725	13725	13728	+	13779 -	-		-	1	1	2
13726	13725	13728	-	13863 -	-		-	1	1	2
13733	13733	13734	-	13845 -	-		+	1	1	2
13743	13739	13745	-	13794	13790	13794	+	1	1	2
13753	13750	13757	-	13883	13880	13883	+	1	1	2
13753	13750	13757	-	13892 -	-		+	1	1	2
13753	13750	13757	-	13894 -	-		-	1	1	2
13753	13750	13757	-	13927 -	-		-	1	1	2
13753	13750	13757	-	13967 -	-		-	1	1	2
13753 -	-		+	14053 -	-		-	1	1	2
13761 -	-		+	13723 -	-		-	1	1	2
13763	13758	13764	-	13884	13882	13884	+	1	1	2
13763	13758	13764	-	13897 -	-		-	1	1	2
13763	13758	13764	-	14041 -	-		+	1	1	2
13772	13768	13776	-	13829 -	-		+	1	1	2
13785	13781	13785	-	13823	13820	13823	+	1	1	2
13786	13782	13788	+	13709 -	-		-	1	1	2

13796	13794	13802 -	13993 -	-	-	1	1	2
13796	13794	13802 -	13999 -	-	-	1	1	2
13796	13794	13802 -	14005 -	-	-	1	1	2
13796 -	-	+	13891 -	-	-	0	2	2
13807	13803	13807 +	13884 -	-	-	2	0	2
13808	13804	13812 -	13835 -	-	+	1	1	2
13821	13821	13823 +	13798 -	-	-	1	1	2
13821	13821	13823 +	14019 -	-	+	1	1	2
13823	13819	13827 -	13956 -	-	+	2	0	2
13823	13819	13827 -	13974 -	-	-	1	1	2
13831	13831	13834 -	13951 -	-	+	0	2	2
13831	13831	13834 -	13952	13950	13952 -	1	1	2
13838	13838	13839 +	13942	13940	13942 -	1	1	2
13839	13836	13840 -	13940	13940	13941 +	1	1	2
13839	13836	13840 -	13965 -	-	-	1	1	2
13839	13836	13840 -	13972 -	-	-	1	1	2
13839	13836	13840 -	14025	14023	14025 -	1	1	2
13839	13836	13840 -	14104	14104	14105 -	1	1	2
13849	13847	13849 -	13875 -	-	+	1	1	2
13857	13854	13861 +	13879 -	-	-	1	1	2
13861	13861	13865 -	14043 -	-	-	1	1	2
13867	13865	13867 +	13969 -	-	-	1	1	2
13872	13870	13877 -	13927 -	-	+	1	1	2
13872	13870	13877 -	14048 -	-	-	1	1	2
13872	13870	13877 -	14055 -	-	-	1	1	2
13872	13870	13877 -	14068 -	-	-	1	1	2
13879	13875	13880 +	14046 -	-	+	1	1	2
13882	13880	13886 -	13906 -	-	+	2	0	2
13882	13880	13886 -	13959 -	-	-	1	1	2
13882	13880	13886 -	14043 -	-	-	1	1	2
13882	13880	13886 -	14048 -	-	-	1	1	2
13882	13880	13886 -	14099 -	-	-	1	1	2
13884	13882	13884 +	13990 -	-	-	1	1	2
13891	13887	13894 +	13971 -	-	-	1	1	2
13891	13887	13894 +	14695 -	-	-	1	1	2
13894	13893	13894 -	14056 -	-	-	1	1	2
13899	13897	13903 +	13844 -	-	-	1	1	2
13901	13901	13904 -	14106 -	-	-	1	1	2
13911	13911	13913 +	13893	13893	13894 -	1	1	2
13912	13908	13912 -	14044 -	-	-	1	1	2
13912	13908	13912 -	14101 -	-	-	1	1	2
13923	13920	13928 -	14069 -	-	-	1	1	2
13923	13920	13928 -	14092 -	-	-	1	1	2
13923	13920	13928 -	14104 -	-	-	1	1	2
13923	13920	13928 -	14131	14131	14132 -	1	1	2
13924 -	-	+	14084 -	-	-	1	1	2
13930 -	-	+	13952 -	-	+	1	1	2
13932	13931	13932 -	14035	14035	14036 -	1	1	2
13938	13935	13942 -	14082 -	-	-	1	1	2
13938	13935	13942 -	14087 -	-	-	1	1	2
13938	13935	13942 -	14102 -	-	-	1	1	2
13938	13935	13942 -	14150 -	-	-	1	1	2
13938	13935	13942 -	14162 -	-	-	1	1	2
13943 -	-	+	14501 -	-	-	1	1	2
13948	13944	13951 -	14101 -	-	-	0	2	2
13948	13944	13951 -	14121 -	-	-	1	1	2
13948	13948	13949 +	13959 -	-	-	1	1	2
13957	13956	13958 +	13786 -	-	-	1	1	2
13957	13956	13958 +	14008 -	-	-	1	1	2
13961	13956	13965 -	14303 -	-	-	1	1	2
13965	13962	13971 +	13937	13937	13938 -	1	1	2
13965	13962	13971 +	13999 -	-	-	1	1	2
13968	13967	13971 -	14095	14093	14095 +	1	1	2
13968	13967	13971 -	14121	14121	14122 -	1	1	2
13968	13967	13971 -	14132 -	-	-	1	1	2
13968	13967	13971 -	14163 -	-	-	1	1	2
13968	13967	13971 -	14548	14544	14548 +	1	1	2
13973	13972	13974 -	14107 -	-	-	1	1	2
13973	13972	13974 -	14133 -	-	-	1	1	2
13973	13972	13974 -	14442 -	-	-	1	1	2
13974	13973	13976 +	13936 -	-	+	1	1	2
13974	13973	13976 +	14177 -	-	-	0	2	2
13974	13973	13976 +	14523	14523	14524 -	1	1	2
13978	13975	13982 -	14048 -	-	+	1	1	2
13978	13975	13982 -	14101 -	-	-	1	1	2
13983	13980	13983 +	13954 -	-	-	1	1	2
13990	13990	13992 +	14022 -	-	-	1	1	2
13992	13987	13993 -	14047 -	-	+	1	1	2
13992	13987	13993 -	14073 -	-	+	1	1	2
13992	13987	13993 -	14111 -	-	-	1	1	2
13992	13987	13993 -	14169 -	-	-	1	1	2
13992	13987	13993 -	14198 -	-	-	1	1	2
13997	13995	14001 -	14150 -	-	-	1	1	2
13997	13995	14001 -	14161 -	-	-	1	1	2
13997	13995	14001 -	14178 -	-	-	1	1	2
13997	13995	14001 -	14198 -	-	+	2	0	2
13997	13995	14001 -	14254 -	-	-	1	1	2
13999	13996	13999 +	14052 -	-	-	1	1	2
14008	14003	14011 -	14123 -	-	-	1	1	2
14008	14003	14011 -	14187 -	-	-	1	1	2
14008	14003	14011 -	14194 -	-	+	0	2	2
14008	14003	14011 -	14223 -	-	-	1	1	2
14016	14014	14020 -	14154 -	-	-	1	1	2
14035	14035	14036 -	14117 -	-	+	1	1	2
14039 -	-	+	14557 -	-	+	1	1	2
14041	14038	14041 -	14169 -	-	+	1	1	2

14041	14038	14041 -	14188 -	-	-	1	1	2
14041	14038	14041 -	14207 -	-	-	1	1	2
14048	14044	14051 -	14247 -	-	-	1	1	2
14048	14044	14051 -	14258 -	-	-	1	1	2
14048	14044	14051 -	14818 -	-	-	1	1	2
14053	14053	14057 -	14192 -	-	-	1	1	2
14053	14053	14057 -	14207 -	-	-	1	1	2
14062	14058	14066 -	14125 -	-	+	1	1	2
14062	14058	14066 -	14206 -	-	-	1	1	2
14062	14058	14066 -	14293 -	-	-	1	1	2
14069	14069	14071 -	14129 -	-	+	1	1	2
14069	14069	14071 -	14196 -	-	-	1	1	2
14069	14069	14071 -	14210	14208	14210 -	1	1	2
14074	14073	14078 -	14015	14012	14015 +	1	1	2
14074	14073	14078 -	14231 -	-	-	1	1	2
14074 -	-	+	13991 -	-	-	1	1	2
14081	14081	14086 +	14004	14004	14005 -	1	1	2
14081	14081	14086 +	14028 -	-	-	1	1	2
14082	14082	14085 -	14132	14129	14132 +	1	1	2
14098	14098	14102 -	14111	14107	14111 +	2	0	2
14098	14098	14102 -	14206 -	-	-	1	1	2
14098	14094	14098 +	14111 -	-	-	1	1	2
14113	14109	14114 +	14029 -	-	-	1	1	2
14113	14109	14114 +	14260 -	-	-	1	1	2
14120 -	-	-	14718 -	-	+	1	1	2
14130	14127	14134 -	14160 -	-	+	1	1	2
14130	14127	14134 -	14349 -	-	-	1	1	2
14143	14143	14148 -	14394 -	-	-	1	1	2
14143	14143	14148 -	14532 -	-	+	1	1	2
14147 -	-	+	14171 -	-	-	1	1	2
14153	14153	14155 +	14231 -	-	+	1	1	2
14157	14155	14162 -	14177 -	-	+	2	0	2
14157	14155	14162 -	14225 -	-	-	1	1	2
14157	14155	14162 -	14232	14232	14233 -	1	1	2
14157	14155	14162 -	14348 -	-	-	1	1	2
14165	14165	14169 +	14242	14242	14243 -	1	1	2
14171	14169	14174 -	14318 -	-	-	1	1	2
14171	14169	14174 -	14394 -	-	-	2	0	2
14171	14170	14171 +	14082 -	-	-	1	1	2
14171	14170	14171 +	14112 -	-	-	1	1	2
14178	14175	14182 -	14307 -	-	-	1	1	2
14178	14175	14182 -	14326 -	-	-	1	1	2
14178	14175	14182 -	14349 -	-	-	1	1	2
14179	14179	14180 +	14117 -	-	-	1	1	2
14179	14179	14180 +	14243 -	-	-	1	1	2
14184	14184	14188 -	14206 -	-	+	1	1	2
14184	14184	14188 -	14241 -	-	+	1	1	2
14184	14184	14188 -	14275 -	-	+	1	1	2
14184	14184	14188 -	14328	14328	14329 -	1	1	2
14192	14190	14192 -	14227 -	-	+	1	1	2
14192	14190	14192 -	14348 -	-	-	1	1	2
14201	14198	14204 -	14277 -	-	+	1	1	2
14201	14198	14204 -	14339 -	-	-	1	1	2
14201	14198	14204 -	14375 -	-	-	1	1	2
14203	14199	14205 +	14119 -	-	-	0	2	2
14203	14199	14205 +	14162 -	-	-	0	2	2
14203	14199	14205 +	14183 -	-	-	1	1	2
14203	14199	14205 +	14233 -	-	-	1	1	2
14203	14199	14205 +	14444 -	-	-	1	1	2
14210	14208	14210 +	14114 -	-	-	2	0	2
14210	14208	14210 +	14157 -	-	-	2	0	2
14215	14211	14215 -	14219 -	-	+	2	0	2
14220	14220	14221 +	14362 -	-	-	2	0	2
14225 -	-	-	14233 -	-	+	1	1	2
14230 -	-	+	14352 -	-	-	1	1	2
14231	14229	14231 -	14274 -	-	+	1	1	2
14231	14229	14231 -	14348	14348	14349 +	1	1	2
14231	14229	14231 -	14384 -	-	-	1	1	2
14236 -	-	+	14268 -	-	-	1	1	2
14243	14238	14247 +	14112 -	-	-	1	1	2
14243	14238	14247 +	14195 -	-	-	1	1	2
14243	14238	14247 +	14217 -	-	-	2	0	2
14243	14238	14247 +	14222 -	-	-	0	2	2
14243	14238	14247 +	14271 -	-	-	1	1	2
14248	14247	14248 -	14425 -	-	-	1	1	2
14256	14254	14256 -	14402 -	-	+	1	1	2
14256	14254	14256 -	14904 -	-	-	1	1	2
14259	14259	14263 +	14181 -	-	-	1	1	2
14259	14259	14263 +	14426	14426	14427 -	1	1	2
14265 -	-	-	14287 -	-	+	1	1	2
14268	14268	14270 +	14295 -	-	+	0	2	2
14271	14270	14273 -	14283 -	-	+	2	0	2
14273 -	-	+	14298 -	-	+	2	0	2
14276	14276	14278 -	14453 -	-	-	1	1	2
14283	14283	14285 -	14270 -	-	+	2	0	2
14283	14283	14285 -	14445	14443	14445 -	1	1	2
14283	14283	14285 -	14506 -	-	+	1	1	2
14301	14300	14305 -	14465 -	-	-	1	1	2
14301	14300	14305 -	14517 -	-	-	1	1	2
14310	14308	14310 -	14353 -	-	-	1	1	2
14316	14314	14316 -	14499 -	-	-	1	1	2
14324	14321	14326 -	14358 -	-	-	0	2	2
14324	14321	14326 -	14482 -	-	-	1	1	2
14333	14331	14337 -	14407	14405	14407 +	1	1	2
14333	14331	14337 -	14489 -	-	-	1	1	2
14333	14331	14337 -	14516 -	-	-	1	1	2

14344	14339	14346 -	14453 -	-	-	1	1	2
14344	14339	14346 -	14858 -	-	+	1	1	2
14346	14346	14347 +	14557	14557	14558 -	1	1	2
14353	14353	14357 -	14554	14554	14555 +	1	1	2
14357	14357	14358 +	14314	14314	14315 -	1	1	2
14359	14358	14364 -	14557 -	-	-	1	1	2
14362 -	-	+	14220 -	-	-	2	0	2
14369	14368	14371 -	14528 -	-	-	1	1	2
14369	14368	14371 -	14894 -	-	-	1	1	2
14374	14373	14380 -	14417	14417	14418 +	1	1	2
14374	14373	14380 -	14548 -	-	-	1	1	2
14374	14373	14380 -	14572 -	-	-	1	1	2
14374	14373	14380 -	14574 -	-	+	1	1	2
14374	14373	14380 -	14589 -	-	-	1	1	2
14375 -	-	+	14353 -	-	+	1	1	2
14384	14382	14385 -	14532	14532	14533 +	1	1	2
14384	14382	14385 -	14557 -	-	+	1	1	2
14384	14382	14385 -	14791 -	-	-	1	1	2
14391	14386	14392 -	14481 -	-	+	1	1	2
14391	14386	14392 -	14534 -	-	-	1	1	2
14391	14386	14392 -	14541 -	-	+	1	1	2
14391	14386	14392 -	14574	14574	14575 +	1	1	2
14398	14398	14401 -	14519 -	-	-	1	1	2
14398	14398	14401 -	14588 -	-	-	1	1	2
14404	14403	14405 -	14511 -	-	-	1	1	2
14404	14403	14405 -	14587 -	-	-	1	1	2
14404	14403	14405 -	14596 -	-	-	1	1	2
14409	14406	14410 -	14573 -	-	-	1	1	2
14416	14413	14419 -	14588 -	-	-	1	1	2
14416	14413	14419 -	15191 -	-	+	1	1	2
14428	14423	14428 -	14557 -	-	-	1	1	2
14435	14434	14439 -	14587 -	-	-	1	1	2
14435	14434	14439 -	14738 -	-	-	1	1	2
14442	14442	14446 -	14865 -	-	+	1	1	2
14448	14447	14450 -	14510 -	-	-	1	1	2
14448	14447	14450 -	14556	14554	14556 +	1	1	2
14448	14447	14450 -	14618 -	-	-	1	1	2
14452 -	-	+	14535 -	-	-	2	0	2
14457 -	-	+	14435 -	-	-	0	2	2
14462	14460	14462 -	14684	14682	14684 -	1	1	2
14474	14474	14476 +	14419 -	-	-	1	1	2
14474	14474	14476 +	14552 -	-	-	1	1	2
14478	14473	14481 -	14496 -	-	+	1	1	2
14478	14473	14481 -	14598 -	-	+	1	1	2
14479	14479	14484 +	14557	14554	14557 -	1	1	2
14479	14479	14484 +	14692 -	-	+	1	1	2
14484	14484	14487 -	14565	14565	14566 +	1	1	2
14496 -	-	-	14558 -	-	+	1	1	2
14507	14503	14509 +	14531 -	-	-	1	1	2
14507	14503	14509 +	14551	14547	14551 -	1	1	2
14511	14509	14515 -	14540 -	-	+	1	1	2
14511	14509	14515 -	14557	14555	14557 +	1	1	2
14511	14509	14515 -	14612 -	-	-	1	1	2
14511	14509	14515 -	14690	14690	14691 -	1	1	2
14531 -	-	-	14725 -	-	+	1	1	2
14535	14530	14537 +	14567 -	-	-	1	1	2
14540	14536	14544 -	14588 -	-	-	1	1	2
14540	14536	14544 -	14694 -	-	-	1	1	2
14540	14536	14544 -	14712 -	-	-	1	1	2
14540	14536	14544 -	14750 -	-	-	1	1	2
14546 -	-	-	14750 -	-	-	1	1	2
14546 -	-	-	14899 -	-	-	1	1	2
14551	14547	14554 +	14465	14461	14465 -	1	1	2
14551	14547	14554 +	14478	14478	14479 -	1	1	2
14551	14547	14554 +	14717 -	-	-	1	1	2
14554	14550	14558 -	14642 -	-	-	1	1	2
14554	14550	14558 -	14676 -	-	-	1	1	2
14554	14550	14558 -	14695 -	-	-	1	1	2
14554	14550	14558 -	14719	14715	14719 -	1	1	2
14554	14550	14558 -	14744 -	-	-	1	1	2
14554	14550	14558 -	14784 -	-	-	1	1	2
14556	14555	14557 +	14451 -	-	-	1	1	2
14556	14555	14557 +	14619	14619	14620 -	1	1	2
14562	14562	14564 -	15176	15176	15177 -	1	1	2
14562 -	-	+	14446 -	-	-	1	1	2
14569	14566	14572 -	14700	14700	14701 -	1	1	2
14577	14574	14583 -	14750 -	-	-	1	1	2
14577	14574	14583 -	14817 -	-	-	1	1	2
14577	14574	14583 -	15044	15042	15044 +	1	1	2
14586	14585	14589 -	14742 -	-	-	1	1	2
14586	14585	14589 -	14763 -	-	-	1	1	2
14586	14585	14589 -	14775 -	-	-	2	0	2
14587 -	-	+	14567 -	-	+	1	1	2
14593	14592	14596 -	14750 -	-	-	1	1	2
14593	14592	14596 -	14780	14780	14781 -	0	2	2
14593	14592	14596 -	14795 -	-	+	1	1	2
14593	14592	14596 -	14820 -	-	-	1	1	2
14600	14598	14602 -	14686 -	-	+	1	1	2
14600	14598	14602 -	14777 -	-	-	2	0	2
14600	14598	14602 -	14817	14817	14818 -	1	1	2
14605	14605	14609 -	14612 -	-	+	2	0	2
14605	14605	14609 -	14679	14675	14679 +	1	1	2
14605	14605	14609 -	14780 -	-	+	1	1	2
14605	14605	14609 -	14781 -	-	-	0	2	2
14614	14612	14615 -	14607 -	-	+	2	0	2
14614	14612	14615 -	14762 -	-	-	1	1	2

14619	14619	14620 -	14769 -	-	+	1	1	2
14619	14619	14621 +	14598	14598	14599 -	1	1	2
14619	14619	14621 +	14769 -	-	-	1	1	2
14624	14624	14626 -	14599 -	-	+	2	0	2
14630 -	-	+	14948 -	-	+	1	1	2
14635	14634	14636 +	14579 -	-	-	1	1	2
14636	14632	14637 -	14662 -	-	+	1	1	2
14636	14632	14637 -	14687 -	-	-	1	1	2
14636	14632	14637 -	14805 -	-	-	1	1	2
14640	14640	14643 +	14533 -	-	-	1	1	2
14642	14639	14643 -	14684	14684	14685 +	1	1	2
14642	14639	14643 -	14817	14815	14817 -	1	1	2
14642	14639	14643 -	15213 -	-	-	1	1	2
14647	14645	14651 -	14712 -	-	+	1	1	2
14647	14645	14651 -	14794 -	-	-	1	1	2
14647	14645	14651 -	14794	14790	14794 +	1	1	2
14647	14645	14651 -	14801 -	-	-	1	1	2
14647	14645	14651 -	14818 -	-	-	1	1	2
14647	14645	14651 -	14846 -	-	-	1	1	2
14651	14651	14653 +	14599 -	-	+	1	1	2
14667	14663	14670 -	14712 -	-	+	1	1	2
14667	14663	14670 -	14829 -	-	-	1	1	2
14675	14674	14679 -	14892	14892	14893 +	1	1	2
14676	14672	14680 +	14623 -	-	-	1	1	2
14687	14684	14690 -	14838 -	-	-	1	1	2
14687	14684	14690 -	15165 -	-	+	1	1	2
14687	14686	14687 +	14718 -	-	+	1	1	2
14692	14691	14697 -	14704 -	-	+	1	1	2
14692	14691	14697 -	14827 -	-	-	1	1	2
14692	14691	14697 -	14872 -	-	-	1	1	2
14692	14691	14697 -	14904 -	-	-	1	1	2
14692	14691	14697 -	15119 -	-	-	1	1	2
14704	14704	14708 +	14734 -	-	-	1	1	2
14704	14704	14708 +	15324 -	-	-	1	1	2
14706	14703	14710 -	14754	14750	14754 +	1	1	2
14706	14703	14710 -	14782 -	-	+	1	1	2
14706	14703	14710 -	14833 -	-	-	1	1	2
14706	14703	14710 -	14922	14922	14923 +	1	1	2
14706	14703	14710 -	15118 -	-	+	1	1	2
14714	14712	14714 -	14900 -	-	-	1	1	2
14714	14712	14714 -	14906 -	-	+	1	1	2
14714	14712	14714 -	14928 -	-	-	1	1	2
14714	14712	14714 -	14942 -	-	-	1	1	2
14719	14716	14723 -	14892 -	-	-	1	1	2
14719	14716	14723 -	14929 -	-	-	1	1	2
14728	14726	14728 -	14862 -	-	+	1	1	2
14744	14741	14750 -	14743	14743	14744 +	2	0	2
14744	14741	14750 -	14882	14882	14883 +	1	1	2
14744	14741	14750 -	14918 -	-	-	1	1	2
14744	14741	14750 -	14947	14945	14947 -	1	1	2
14744	14741	14750 -	15231 -	-	-	1	1	2
14761	14758	14763 -	14826 -	-	+	1	1	2
14761	14758	14763 -	14861 -	-	+	1	1	2
14771	14767	14771 -	14852	14852	14853 +	1	1	2
14771	14767	14771 -	14902 -	-	+	1	1	2
14771	14767	14771 -	14943 -	-	-	1	1	2
14771	14767	14771 -	14960 -	-	-	1	1	2
14771	14767	14771 -	15083 -	-	-	1	1	2
14776	14773	14779 -	14915 -	-	-	1	1	2
14776	14773	14779 -	14920 -	-	-	1	1	2
14776	14773	14779 -	14998 -	-	-	1	1	2
14776	14773	14779 -	15023 -	-	-	0	2	2
14777	14775	14777 +	14668 -	-	-	1	1	2
14791	14788	14792 -	15029 -	-	-	1	1	2
14792 -	-	+	15113 -	-	-	1	1	2
14796	14795	14799 -	14955 -	-	+	0	2	2
14802 -	-	+	14816 -	-	-	1	1	2
14806	14802	14807 -	14816 -	-	+	1	1	2
14807	14805	14807 +	14874 -	-	-	1	1	2
14807	14805	14807 +	14882	14882	14883 -	1	1	2
14824	14824	14827 -	14966 -	-	-	1	1	2
14824	14824	14827 -	14974 -	-	-	1	1	2
14824	14824	14827 -	15003 -	-	-	1	1	2
14824	14824	14827 +	14761 -	-	-	1	1	2
14836	14836	14837 -	14856 -	-	+	2	0	2
14841	14837	14841 +	15043 -	-	+	1	1	2
14841	14837	14841 +	15074 -	-	-	1	1	2
14847	14843	14851 -	14982	14979	14982 -	1	1	2
14847	14843	14851 -	14998 -	-	-	1	1	2
14854 -	-	-	15002 -	-	+	1	1	2
14860	14856	14862 -	14912	14910	14912 +	1	1	2
14860	14856	14862 -	15029 -	-	-	1	1	2
14860	14856	14862 -	15078 -	-	-	1	1	2
14864	14864	14867 +	14756 -	-	-	2	0	2
14866	14866	14872 -	15004 -	-	-	1	1	2
14866	14866	14872 -	15022 -	-	-	1	1	2
14866	14866	14872 -	15031 -	-	-	1	1	2
14869 -	-	+	14755 -	-	-	0	2	2
14875	14874	14875 -	15080 -	-	-	1	1	2
14878	14878	14882 +	14806	14806	14807 -	1	1	2
14878	14878	14882 +	14902	14902	14903 -	1	1	2
14878	14878	14882 +	15015 -	-	-	1	1	2
14880	14879	14882 -	14900 -	-	+	1	1	2
14880	14879	14882 -	14924 -	-	+	2	0	2
14880	14879	14882 -	14987 -	-	+	2	0	2
14880	14879	14882 -	15002 -	-	-	1	1	2

14887	14884	14891 -	14912 -	-	+	1	1	2
14887	14884	14891 -	14919 -	-	+	0	2	2
14887	14884	14891 -	14926	14926	14930 +	1	1	2
14887	14884	14891 -	14982 -	-	+	0	2	2
14887	14884	14891 -	15086 -	-	-	1	1	2
14887	14884	14891 -	15103 -	-	-	1	1	2
14887	14884	14891 -	15141 -	-	-	1	1	2
14892	14888	14895 +	14870 -	-	-	1	1	2
14892	14888	14895 +	14903 -	-	-	1	1	2
14897	14892	14900 -	14944	14942	14944 +	1	1	2
14897	14892	14900 -	14958 -	-	+	1	1	2
14897	14892	14900 -	14965 -	-	+	1	1	2
14897	14892	14900 -	15024 -	-	-	1	1	2
14897	14892	14900 -	15029 -	-	-	1	1	2
14897	14892	14900 -	15069	15067	15069 -	1	1	2
14897	14892	14900 -	15152	15148	15152 +	1	1	2
14902	14898	14902 +	14767 -	-	-	1	1	2
14902	14898	14902 +	14938 -	-	+	1	1	2
14902	14898	14902 +	15091 -	-	-	1	1	2
14902	14898	14902 +	15243 -	-	-	1	1	2
14905	14901	14908 -	14952 -	-	+	1	1	2
14905	14901	14908 -	14960 -	-	+	1	1	2
14905	14901	14908 -	15067 -	-	-	1	1	2
14905	14901	14908 -	15139 -	-	+	2	0	2
14913	14910	14917 -	14958 -	-	+	0	2	2
14913	14910	14917 -	14963 -	-	+	2	0	2
14913	14910	14917 -	15135 -	-	+	0	2	2
14925	14922	14928 -	15086 -	-	-	1	1	2
14925	14922	14928 -	15136 -	-	-	1	1	2
14928	14925	14928 +	14955 -	-	-	0	2	2
14933	14930	14935 -	15078 -	-	-	2	0	2
14933	14930	14935 -	15085 -	-	-	1	1	2
14933	14930	14935 -	15305 -	-	-	1	1	2
14933 -	-	+	14950 -	-	-	2	0	2
14938	14938	14939 -	15083	15083	15084 -	0	2	2
14938	14938	14939 -	15103 -	-	-	1	1	2
14938	14938	14939 -	15115 -	-	-	1	1	2
14938 -	-	+	14877 -	-	-	1	1	2
14944	14941	14947 -	14975	14973	14975 +	1	1	2
14944	14941	14947 -	15080	15080	15081 -	1	1	2
14944	14941	14947 -	15107 -	-	-	1	1	2
14944	14941	14947 -	15117 -	-	-	1	1	2
14944	14941	14947 -	15150 -	-	-	1	1	2
14944	14941	14947 -	15172 -	-	-	1	1	2
14951	14951	14952 -	15129 -	-	-	1	1	2
14951	14951	14952 -	15148 -	-	-	1	1	2
14952 -	-	+	14897 -	-	-	1	1	2
14957	14956	14957 -	15107 -	-	-	1	1	2
14957	14956	14957 -	15150 -	-	-	1	1	2
14963	14959	14966 -	15118 -	-	-	1	1	2
14963	14959	14966 -	15137 -	-	-	1	1	2
14963	14959	14966 -	15157	15157	15160 -	1	1	2
14963	14959	14966 -	15182 -	-	-	1	1	2
14963	14959	14966 -	15194 -	-	-	1	1	2
14963	14959	14966 -	15316 -	-	-	1	1	2
14972	14969	14975 -	15003 -	-	+	1	1	2
14972	14969	14975 -	15098 -	-	-	1	1	2
14977	14977	14979 +	14982 -	-	+	1	1	2
14977	14977	14979 +	15163 -	-	-	1	1	2
14978	14977	14981 -	14996 -	-	+	1	1	2
14978	14977	14981 -	15085 -	-	-	1	1	2
14978	14977	14981 -	15109 -	-	+	1	1	2
14978	14977	14981 -	15135 -	-	-	1	1	2
14978	14977	14981 -	15150 -	-	-	1	1	2
14982 -	-	+	15356 -	-	-	1	1	2
14984	14984	14986 -	15016	15014	15016 +	1	1	2
14984	14984	14986 -	15158 -	-	+	1	1	2
14988	14984	14988 +	15005 -	-	+	1	1	2
14988	14984	14988 +	15158	15156	15158 -	1	1	2
14994	14990	14997 -	15182 -	-	-	1	1	2
14994	14990	14997 -	15209	15209	15210 -	1	1	2
14994	14990	14997 -	15280	15277	15280 -	1	1	2
14998	14996	15000 +	15115	15111	15115 -	1	1	2
15003	14999	15003 -	15051 -	-	+	1	1	2
15003	14999	15003 -	15144 -	-	+	1	1	2
15003	14999	15003 -	15158 -	-	-	1	1	2
15003	14999	15003 -	15194 -	-	-	1	1	2
15012	15008	15015 +	15086 -	-	+	1	1	2
15014	15010	15018 -	15161	15161	15165 -	1	1	2
15014	15010	15018 -	15171 -	-	-	1	1	2
15024	15020	15025 +	14998 -	-	-	1	1	2
15027	15025	15032 -	15054 -	-	+	1	1	2
15027	15025	15032 -	15077 -	-	+	2	0	2
15027	15025	15032 -	15160 -	-	-	1	1	2
15027	15025	15032 -	15182 -	-	-	1	1	2
15027	15025	15032 -	15191	15191	15192 -	1	1	2
15027	15025	15032 -	15196 -	-	-	1	1	2
15027	15025	15032 -	15318 -	-	-	1	1	2
15027	15025	15032 -	15378	15378	15379 -	1	1	2
15032	15030	15035 +	15079 -	-	-	1	1	2
15032	15030	15035 +	15156 -	-	-	1	1	2
15037	15034	15039 -	15072 -	-	+	0	2	2
15037	15034	15039 -	15201 -	-	+	1	1	2
15039 -	-	+	15127 -	-	+	1	1	2
15052	15049	15055 -	15198 -	-	-	1	1	2
15054	15052	15054 +	14997	14995	14997 -	1	1	2

15061		15058	15064	-	15072	-	-		+	2	0	2
15061		15058	15064	-	15092	-	-		+	2	0	2
15061		15058	15064	-	15248	-	-		-	1	1	2
15061		15058	15064	-	15324	-	-		-	1	1	2
15064	-	-		+	15079	-	-		+	1	1	2
15066	-	-		-	15088	-	-		+	0	2	2
15070		15066	15073	+	15009		15009	15010	-	1	1	2
15070		15066	15073	+	15314	-	-		-	1	1	2
15072		15068	15073	-	15058	-	-		+	2	0	2
15082		15080	15084	-	15209	-	-		+	1	1	2
15082		15080	15084	-	15211	-	-		-	1	1	2
15082		15080	15084	-	15240	-	-		-	1	1	2
15085		15085	15087	+	15291		15289	15291	+	1	1	2
15088	-	-		-	15232	-	-		-	1	1	2
15093		15093	15095	-	15319	-	-		-	1	1	2
15095		15093	15096	+	15163		15160	15163	-	1	1	2
15104		15102	15108	-	15145	-	-		+	1	1	2
15104		15102	15108	-	15278	-	-		+	0	2	2
15104		15102	15108	-	15323	-	-		-	1	1	2
15104	-	-		+	15278	-	-		-	2	0	2
15112		15110	15115	-	15207	-	-		+	1	1	2
15112		15110	15115	-	15272		15272	15273	+	1	1	2
15112		15110	15115	-	15278	-	-		-	1	1	2
15112		15110	15115	-	15293	-	-		-	1	1	2
15112	-	-		+	15207	-	-		-	1	1	2
15117		15116	15117	-	15309	-	-		-	1	1	2
15118		15117	15118	+	15043		15043	15044	-	1	1	2
15122	-	-		-	15175	-	-		+	1	1	2
15125	-	-		+	15037		15034	15037	-	1	1	2
15128		15124	15129	-	15240	-	-		-	1	1	2
15128		15124	15129	-	15297	-	-		-	1	1	2
15128		15124	15129	-	15302	-	-		-	1	1	2
15128		15124	15129	-	15307	-	-		-	1	1	2
15128		15124	15129	-	15336	-	-		-	1	1	2
15130		15129	15133	+	15239	-	-		+	1	1	2
15130		15129	15133	+	15325		15325	15326	-	1	1	2
15134		15133	15138	-	15290	-	-		-	1	1	2
15134		15133	15138	-	15302	-	-		-	1	1	2
15134		15133	15138	-	15339	-	-		-	1	1	2
15138		15138	15141	+	15122	-	-		-	1	1	2
15143	-	-		+	15272	-	-		-	1	1	2
15144		15140	15147	-	15238		15238	15239	-	1	1	2
15144		15140	15147	-	15255	-	-		-	1	1	2
15144		15140	15147	-	15271	-	-		-	1	1	2
15144		15140	15147	-	15331	-	-		-	1	1	2
15148		15145	15148	+	15243	-	-		-	1	1	2
15148		15145	15148	+	15328	-	-		-	1	1	2
15161		15158	15161	-	15304		15302	15304	-	1	1	2
15161		15158	15161	-	15324	-	-		-	1	1	2
15163	-	-		+	15186	-	-		+	1	1	2
15166		15163	15169	-	15340	-	-		-	1	1	2
15166		15163	15169	-	15382	-	-		-	1	1	2
15172		15172	15177	+	15190		15190	15191	+	0	2	2
15172		15172	15177	+	15347	-	-		-	1	1	2
15174		15172	15176	-	15298	-	-		-	1	1	2
15185		15185	15187	-	15374		15374	15375	-	1	1	2
15188		15188	15189	+	15204	-	-		+	1	1	2
15188		15188	15189	+	15247	-	-		-	1	1	2
15195		15191	15195	+	15219	-	-		-	1	1	2
15195		15191	15195	+	15320		15320	15321	-	1	1	2
15195		15191	15195	+	15327		15327	15328	-	1	1	2
15204		15204	15206	-	15351	-	-		-	1	1	2
15204		15203	15207	+	15151		15148	15151	-	1	1	2
15204		15203	15207	+	15185	-	-		-	1	1	2
15204		15203	15207	+	15208		15208	15209	-	1	1	2
15210		15210	15213	+	15147	-	-		-	1	1	2
15219		15216	15219	+	15200		15200	15201	-	1	1	2
15219		15216	15219	+	15225	-	-		-	0	2	2
15219		15216	15219	+	15287	-	-		+	1	1	2
15220		15219	15220	-	15331	-	-		-	1	1	2
15225		15225	15227	+	15218	-	-		-	2	0	2
15236		15233	15236	-	15330		15330	15331	+	1	1	2
15236		15233	15236	-	15375	-	-		-	1	1	2
15251		15247	15256	+	15195	-	-		+	1	1	2
15251		15247	15256	+	15266	-	-		-	0	2	2
15251		15247	15256	+	15269	-	-		+	1	1	2
15258	-	-		+	15263	-	-		-	2	0	2
15268	-	-		+	15324	-	-		-	1	1	2
15271	-	-		-	15380	-	-		-	1	1	2
15275		15271	15275	+	15296	-	-		-	1	1	2
15281		15280	15282	+	15148	-	-		-	1	1	2
15289		15289	15290	+	15307		15307	15308	-	1	1	2
15297		15294	15297	+	15227	-	-		-	1	1	2
15303		15303	15305	+	15245		15243	15245	-	1	1	2
15303		15303	15305	+	15303	-	-		+	0	2	2
15313		15310	15313	+	15202	-	-		-	1	1	2
15313		15310	15313	+	15286		15286	15287	-	1	1	2
15323		15322	15325	+	15193	-	-		-	1	1	2
15323		15322	15325	+	15323	-	-		-	1	1	2
15347		15347	15348	+	15361		15361	15362	-	1	1	2
15376	-	-		+	15376	-	-		+	2	0	2
