# Supplementary material for: Identification and quantification of defective virus genomes in high throughput sequencing data using DVG-profiler, a novel post-sequence alignment processing algorithm
Source: PLoS One. 2019 May 17;14(5):e0216944. doi: 10.1371/journal.pone.0216944 (PMC6524942; doi:10.1371/journal.pone.0216944)
Supplement: S14 Table — (PDF) [file pone.0216944.s019.pdf]

Position (left)	Group start (left)	Group end (left)	Strandness	Position (right)	Group start (right)	Group end (right)	Strandness (right)	Forward hits	Reverse hits	fwd and reverse
14496	14493	14501	-	15062	15058	15066	+	33141	47138	80279
14380	14377	14384	-	15147	15143	15149	+	696	1284	1980
14496	14493	14501	-	15053	15053	15054	-	736	262	998
14510	14502	14513	-	14834	14831	14837	+	352	266	618
14963	14959	14966	+	15153	15151	15155	+	93	359	452
14496	14493	14501	-	15070	15069	15074	-	261	137	398
14510	14502	14513	-	15079	15074	15081	-	144	75	219
14970	14967	14972	-	15147	15147	15148	+	13	175	188
14510	14502	14513	-	14813	14811	14816	+	98	88	186
14510	14502	14513	-	15071	15067	15073	-	88	89	177
14838	14833	14841	-	14907	-	-	-	21	142	163
14874	14869	14877	-	14932	14931	14936	-	146	2	148
14670	14667	14673	-	14710	14707	14711	-	65	3	68
14861	14855	14864	-	14922	14919	14926	-	17	45	62
14699	14697	14706	-	14761	14760	14762	-	1	60	61
14522	14520	14526	-	15077	15077	15081	-	40	15	55
14879	14878	14882	-	14937	-	-	-	2	45	47
14507	14506	14511	+	15063	15063	15067	+	21	25	46
14515	14514	14519	-	14844	14844	14848	-	27	18	45
15044	15040	15047	+	15069	15069	15072	-	31	14	45
2709	2705	2709	-	2787	2786	2787	-	42	0	42
14941	14938	14943	+	15072	15072	15073	-	40	1	41
14838	14833	14841	-	14885	14882	14885	-	3	37	40
15144	15142	15147	-	15220	15216	15223	-	18	20	38
14977	14973	14978	-	15023	15021	15023	-	0	35	35
14510	14502	14513	-	15087	15087	15091	-	26	8	34
14960	14959	14962	-	15147	-	-	+	5	29	34
14742	14738	14744	-	14915	14915	14917	-	0	31	31
15144	15142	15147	-	15228	15225	15228	-	2	29	31
14838	14833	14841	-	14917	14917	14921	-	0	30	30
15044	15040	15047	+	15078	15075	15080	-	24	6	30
15066	15062	15069	-	15100	15097	15100	+	11	19	30
14884	14883	14887	-	14987	14984	14987	-	23	5	28
13729	13729	13730	-	15096	-	-	+	18	9	27
14532	14527	14535	-	14669	14666	14669	-	26	1	27
14496	14493	14501	-	15077	15075	15080	-	19	6	25
15144	15142	15147	-	15246	15243	15246	-	23	1	24
14510	14502	14513	-	14856	14852	14860	-	14	9	23
14522	14520	14526	-	14680	14680	14682	+	0	23	23
14861	14855	14864	-	14889	14886	14889	+	22	1	23
15144	15142	15147	-	15207	-	-	-	23	0	23
14784	14782	14787	-	14894	-	-	-	0	22	22
14851	14850	14853	+	14878	14878	14879	-	9	13	22
14853	14850	14854	-	14973	14973	14976	-	20	2	22
14929	14925	14933	+	15008	-	-	-	22	0	22
14960	14959	14962	-	14998	-	-	-	2	20	22
978	978	982	+	14995	-	-	+	20	1	21
14515	14514	14519	-	14542	14540	14544	+	13	7	20
14552	14550	14558	-	15058	-	-	+	8	12	20
14884	14883	14887	-	14948	-	-	-	12	8	20
14895	14891	14896	-	14953	14953	14957	+	3	17	20
14659	14657	14659	+	14741	-	-	-	19	0	19
14832	14827	14833	+	14850	14849	14853	-	14	5	19
15026	15024	15030	+	15083	15081	15085	-	16	3	19
14566	14562	14568	+	14593	14591	14593	-	4	14	18
14594	14591	14597	+	14623	14623	14626	-	12	6	18
14926	14925	14931	-	15053	-	-	-	18	0	18
14970	14967	14972	-	15012	-	-	-	0	18	18
15001	14999	15006	-	15072	15072	15073	-	0	18	18
15092	15090	15095	-	15104	-	-	+	18	0	18
15108	15104	15110	-	15246	15243	15246	-	18	0	18
8401	8397	8405	-	15223	15220	15225	-	7	9	16
14670	14667	14673	-	14719	14719	14720	-	16	0	16
14848	14842	14848	-	14894	14894	14898	-	6	10	16
14943	14939	14948	-	14960	14960	14964	+	1	15	16
15081	15079	15084	-	15120	15117	15120	+	13	3	16
15127	15124	15130	-	15246	-	-	-	14	2	16
14669	14667	14671	+	14714	14714	14716	-	5	10	15
15036	15032	15038	-	15061	15061	15065	+	15	0	15
15038	15033	15038	+	15083	15081	15087	-	10	5	15
14532	14527	14535	-	14843	14839	14843	-	12	2	14
14752	14747	14755	-	14912	14911	14912	-	0	14	14
14862	14858	14866	+	14884	14881	14888	-	1	13	14
15038	15033	15038	+	15078	15074	15078	-	12	2	14
15170	-	-	-	15219	-	-	-	14	0	14
14496	14493	14501	-	15087	15084	15089	-	13	0	13
14510	14502	14513	-	14552	14549	14552	+	10	3	13
14510	14502	14513	-	14839	-	-	-	10	3	13
14541	14537	14544	-	15070	15070	15071	-	4	9	13
14547	14545	14548	-	15076	15074	15076	-	5	8	13

14577	14573	14580 -	14641 -	-	-	0	13	13
14694	14690	14696 -	14857	14856	14862 -	12	1	13
14853	14850	14854 -	14878	14876	14878 -	0	13	13
14510	14502	14513 -	15050 -	-	-	12	0	12
14532	14527	14535 -	15071 -	-	-	4	8	12
14541	14537	14544 -	14676	14676	14677 -	1	11	12
14874	14869	14877 -	14940	14937	14940 -	10	2	12
14906	14903	14907 +	14946 -	-	-	1	11	12
14943	14939	14948 -	15146	15142	15146 -	3	9	12
15019	15014	15022 -	15162	15160	15164 -	11	1	12
15071	15071	15077 -	15246	15244	15246 -	12	0	12
15144	15142	15147 -	15189 -	-	-	12	0	12
12418	12416	12418 -	12498 -	-	-	0	11	11
14496	14493	14501 -	14567	14563	14567 -	3	8	11
14532	14527	14535 -	14705	14705	14708 -	0	11	11
14618	14616	14620 +	14642 -	-	-	0	11	11
14761	14757	14765 -	14800 -	-	+	5	6	11
14922	14918	14924 +	15018	15014	15018 -	11	0	11
15049	15048	15053 +	15073	15072	15073 -	6	5	11
8394	8394	8395 -	15216	15215	15217 -	9	1	10
11757	11757	11758 -	14772 -	-	-	10	0	10
12245	12245	12247 -	12327	12327	12329 -	10	0	10
14867	14865	14868 -	14952	14952	14954 -	8	2	10
14912	14912	14916 +	14940 -	-	-	10	0	10
14977	14973	14978 -	15011 -	-	-	0	10	10
15042	15039	15045 -	15065 -	-	+	10	0	10
15100	15099	15102 -	15186	15185	15186 -	10	0	10
15135	15132	15140 -	15246 -	-	-	9	1	10
15149	15149	15152 -	15246	15245	15246 -	9	1	10
3522	3522	3523 -	3597	3597	3598 -	1	8	9
3577	3576	3578 -	3638	3638	3639 -	3	6	9
6151	6151	6153 -	6211	6211	6214 -	9	0	9
14510	14502	14513 -	14878	14878	14881 -	6	3	9
14532	14527	14535 -	14849 -	-	-	9	0	9
14532	14527	14535 -	15125 -	-	-	9	0	9
14552	14550	14558 -	14614	14613	14614 -	8	1	9
14630	14629	14637 -	14711 -	-	-	9	0	9
14699	14697	14706 -	14960 -	-	-	9	0	9
14752	14747	14755 -	14814	14814	14815 +	1	8	9
14769	14768	14772 +	14830	14830	14831 -	2	7	9
14797	14795	14801 -	14839	14839	14841 +	9	0	9
14823	14818	14825 +	14852	14852	14856 -	6	3	9
14837	14835	14841 +	14874	14872	14875 -	8	1	9
14848	14842	14848 -	14912	14912	14916 -	0	9	9
14952	14950	14956 +	15061	15059	15061 -	9	0	9
15044	15040	15047 +	15087	15083	15087 -	7	2	9
15066	15062	15069 -	15176	15176	15180 -	0	9	9
15127	15127	15128 +	15140	15139	15140 -	5	4	9
15217	15214	15217 +	15246	15243	15246 -	5	4	9
3860	3857	3862 -	3933 -	-	-	0	8	8
5786	5782	5787 -	14588	14588	14591 +	1	7	8
7172	7169	7172 -	7313	7311	7313 -	0	8	8
9908	9906	9908 -	10014 -	-	-	8	0	8
10711	10711	10712 -	10762	10762	10763 -	0	8	8
13513 -	-	-	13576 -	-	-	0	8	8
14138 -	-	-	14286 -	-	-	0	8	8
14213	14212	14217 -	14329	14327	14329 -	0	8	8
14622	14620	14624 -	14704	14703	14704 -	6	2	8
14699	14697	14706 -	14818	14816	14821 -	7	1	8
14861	14855	14864 -	14884	14881	14884 +	8	0	8
15021	15016	15021 +	15088 -	-	-	4	4	8
15036	15032	15038 -	15074	15071	15074 +	8	0	8
15042	15039	15045 -	15078 -	-	+	8	0	8
15100	15099	15102 -	15159 -	-	-	8	0	8
15117	15113	15121 -	15177	15175	15177 -	0	8	8
410	410	412 -	496 -	-	-	0	7	7
816 -	-	+	12354 -	-	+	2	5	7
5793 -	-	+	14581 -	-	-	6	1	7
7936	7936	7938 -	7997	7995	7997 -	0	7	7
8642 -	-	-	8707 -	-	-	1	6	7
12408 -	-	-	12490 -	-	-	6	1	7
13551 -	-	-	13602 -	-	-	0	7	7
14510	14502	14513 -	15050	15050	15053 +	0	7	7
14522	14520	14526 -	15087	15087	15088 -	6	1	7
14541	14537	14544 -	14669	14667	14669 +	0	7	7
14541	14537	14544 -	14717 -	-	-	0	7	7
14571	14568	14571 -	14600 -	-	+	6	1	7
14574	14569	14577 +	14626	14623	14630 -	4	3	7
14631	14628	14634 +	14640 -	-	-	0	7	7
14761	14757	14765 -	14864	14862	14868 -	5	2	7
14761	14758	14764 +	14799	14796	14800 -	2	5	7

14779	14776	14780	-	14892	-	-	-	0	7	7
14867	14865	14868	-	14988	14986	14991	-	7	0	7
14868	14867	14872	+	14901	14901	14902	-	0	7	7
14895	14891	14896	-	15000	14996	15000	-	5	2	7
14905	14903	14908	-	14946	-	-	+	7	0	7
14912	14912	14916	+	15025	-	-	-	7	0	7
14926	14925	14931	-	15008	-	-	+	0	7	7
14929	14925	14933	+	15032	15030	15032	-	7	0	7
14953	14951	14957	-	14995	14995	14996	-	7	0	7
14953	14951	14957	-	15045	-	-	-	4	3	7
14965	-	-	-	15064	-	-	-	3	4	7
15019	15014	15022	-	15108	15108	15112	-	6	1	7
15066	15066	15068	+	15100	-	-	-	1	6	7
15087	15086	15087	-	15128	-	-	-	1	6	7
15179	15179	15180	+	15207	-	-	-	0	7	7
455	455	457	-	524	524	526	-	6	0	6
709	709	710	-	765	765	766	-	0	6	6
3860	3857	3862	-	3928	3927	3928	-	1	5	6
5799	-	-	+	14574	14574	14575	-	3	3	6
6518	6518	6519	+	6499	6498	6499	-	6	0	6
7068	7067	7068	-	7138	7137	7138	-	6	0	6
14106	14105	14106	+	14577	14577	14578	-	2	4	6
14496	14493	14501	-	14696	-	-	-	4	2	6
14510	14502	14513	-	14643	14643	14647	-	0	6	6
14522	14520	14526	-	14537	-	-	+	0	6	6
14600	14598	14605	+	14662	-	-	-	4	2	6
14659	14655	14659	-	14741	-	-	+	0	6	6
14838	14833	14841	-	14893	-	-	-	6	0	6
14848	14842	14848	-	14939	-	-	+	0	6	6
14853	14850	14854	-	14889	-	-	+	3	3	6
14853	14850	14854	-	14936	14932	14940	-	2	4	6
14879	14878	14882	-	15015	15015	15018	-	2	4	6
14884	14883	14887	-	15015	15011	15017	-	1	5	6
14926	14925	14931	-	15170	-	-	+	0	6	6
14952	14950	14956	+	15000	14999	15000	-	5	1	6
14977	14973	14978	-	15169	-	-	-	5	1	6
14978	14974	14982	+	15007	15003	15007	-	1	5	6
14978	14974	14982	+	15020	15018	15020	-	6	0	6
15019	15014	15022	-	15060	-	-	-	6	0	6
15100	15099	15102	-	15066	-	-	+	6	0	6
15122	15122	15123	+	15145	-	-	-	0	6	6
15144	15142	15147	-	15196	15194	15196	-	6	0	6
2018	2015	2018	-	2069	2067	2069	-	5	0	5
6384	6382	6384	-	6468	-	-	-	0	5	5
8726	8726	8727	-	8789	-	-	-	0	5	5
9375	-	-	-	9467	-	-	-	0	5	5
10218	-	-	-	10328	-	-	-	0	5	5
10797	10797	10798	-	10884	10884	10885	-	1	4	5
13912	13908	13912	-	15147	15147	15151	+	1	4	5
14315	14315	14318	-	14399	-	-	-	0	5	5
14375	14373	14376	-	15150	15147	15151	+	4	1	5
14496	14493	14501	-	14619	14619	14623	-	1	4	5
14510	14502	14513	-	14624	14621	14627	-	0	5	5
14510	14502	14513	-	14707	-	-	-	0	5	5
14510	14502	14513	-	14863	14863	14866	-	4	1	5
14515	14514	14519	-	14852	-	-	-	5	0	5
14547	14545	14548	-	14663	14662	14663	+	0	5	5
14552	14550	14558	-	14705	14705	14708	-	1	4	5
14597	14595	14600	-	14630	-	-	-	0	5	5
14617	14612	14619	-	14761	14761	14763	-	4	1	5
14643	14638	14647	-	14679	14677	14682	+	2	3	5
14643	14638	14647	-	14776	14776	14780	-	4	1	5
14664	14661	14665	-	14742	-	-	-	0	5	5
14677	14674	14679	-	14773	14773	14778	-	3	2	5
14697	14695	14699	+	14736	-	-	+	4	1	5
14736	14731	14736	-	14819	-	-	-	1	4	5
14736	14731	14736	-	14907	14905	14908	-	5	0	5
14780	14778	14784	+	14817	14817	14818	-	0	5	5
14814	14809	14816	-	15017	15013	15018	-	0	5	5
14822	14818	14829	-	14928	14924	14932	-	4	1	5
14838	14833	14841	-	14998	14995	14998	-	1	4	5
14838	14833	14841	-	15010	-	-	-	0	5	5
14867	14865	14868	-	14885	14881	14886	+	1	4	5
14867	14865	14868	-	14924	14924	14930	-	4	1	5
14911	14910	14914	-	14966	-	-	-	1	4	5
14911	14910	14914	-	15014	-	-	-	5	0	5
14912	14912	14916	+	14921	-	-	-	4	1	5
14933	14932	14936	-	15028	15028	15032	-	0	5	5
14941	14938	14943	+	14964	-	-	-	0	5	5
14941	14938	14943	+	15014	15014	15015	-	5	0	5
14943	14939	14948	-	14991	-	-	+	4	1	5

14969	14969	14972 +	15030	15028	15030 -	3	2	5
15108	15104	15110 -	15234	15233	15237 -	5	0	5
15117	15113	15121 -	15202 -	-	-	5	0	5
15142	15139	15145 +	15125	15122	15128 -	3	2	5
297	297	298 +	14433	14433	14435 +	2	2	4
399 -	-	-	499 -	-	-	0	4	4
447 -	-	+	12490 -	-	+	2	2	4
790	786	790 -	950	946	950 -	4	0	4
1106 -	-	+	14604 -	-	-	4	0	4
2070	2067	2070 -	2119 -	-	-	4	0	4
2079 -	-	-	2148 -	-	-	0	4	4
2338	2338	2341 -	2385 -	-	-	4	0	4
3052	3052	3054 -	3127	3124	3127 -	0	4	4
3854 -	-	-	3927 -	-	-	4	0	4
6221	6221	6224 -	15220	15220	15223 -	2	2	4
6371 -	-	-	6480 -	-	-	0	4	4
6722	6718	6726 +	15061	15058	15063 +	1	3	4
6881	6881	6882 -	14777	14775	14778 -	1	3	4
7263 -	-	-	7346 -	-	-	0	4	4
8268	8264	8268 -	8356 -	-	-	4	0	4
8401	8397	8405 -	15228 -	-	-	0	4	4
9305 -	-	-	14813 -	-	+	1	3	4
10674 -	-	-	10749 -	-	-	0	4	4
13523	13521	13523 -	13584 -	-	-	0	4	4
13842 -	-	-	13914 -	-	-	4	0	4
14335	14333	14339 -	14976 -	-	+	2	2	4
14496	14493	14501 -	15053	15053	15056 +	0	4	4
14496	14493	14501 -	15059	15059	15061 -	3	1	4
14522	14520	14526 -	14840 -	-	-	2	2	4
14532	14527	14535 -	14710	14709	14710 -	4	0	4
14541	14537	14544 -	14726	14725	14728 -	0	4	4
14562	14560	14566 -	14606	14605	14606 +	3	1	4
14566	14562	14568 +	15111	15111	15115 -	1	3	4
14582	14578	14585 +	15062 -	-	+	3	1	4
14585	14582	14586 -	14716	14714	14717 +	0	4	4
14604	14601	14610 -	14763	14763	14764 -	0	4	4
14643	14638	14647 -	14762	14758	14766 -	2	2	4
14646	14643	14650 +	14755 -	-	-	4	0	4
14694	14690	14696 -	14755	14752	14755 -	3	1	4
14716	14712	14717 -	14853	14853	14854 -	0	4	4
14728	14727	14730 -	14892 -	-	-	0	4	4
14736	14731	14736 -	14885	14885	14889 -	0	4	4
14736	14731	14736 -	14900	14897	14900 -	0	4	4
14797	14795	14801 -	14913	14911	14913 -	3	1	4
14797	14795	14801 -	14944	14943	14944 -	4	0	4
14843	14842	14847 +	14853 -	-	-	4	0	4
14889	14888	14889 -	14959 -	-	+	0	4	4
14895	14891	14896 -	15018	15015	15018 -	1	3	4
14901 -	-	+	14869 -	-	-	1	3	4
14929	14925	14933 +	15003 -	-	-	4	0	4
14941	14938	14943 +	14999	14995	14999 -	2	2	4
14989	14986	14991 +	15020	15017	15020 -	2	2	4
14995	14994	14996 -	15066 -	-	-	4	0	4
15019	15014	15022 -	15087	15087	15090 +	2	2	4
15117	15113	15121 -	15246 -	-	-	4	0	4
15127	15124	15130 -	15241	15239	15242 -	4	0	4
15135	15132	15140 -	15179 -	-	-	4	0	4
15135	15132	15140 -	15223 -	-	-	4	0	4
15135	15132	15140 -	15241	15241	15242 -	4	0	4
15186	15186	15187 +	15246 -	-	-	2	2	4
459 -	-	+	12423 -	-	+	1	2	3
480 -	-	-	529 -	-	-	3	0	3
619 -	-	+	12655 -	-	+	3	0	3
671	667	671 +	12239 -	-	+	2	1	3
748	748	751 +	14657 -	-	+	0	3	3
854	854	858 -	894	890	894 +	2	1	3
1774 -	-	-	6503 -	-	-	0	3	3
2405	2405	2406 -	2472 -	-	-	1	2	3
3073 -	-	-	3129 -	-	-	3	0	3
3079 -	-	-	3135 -	-	-	0	3	3
3984 -	-	-	4042 -	-	-	0	3	3
4158 -	-	-	4274 -	-	-	0	3	3
4243	4240	4246 -	4388 -	-	-	3	0	3
4610	4610	4612 -	14662	14662	14664 -	1	2	3
4714 -	-	-	4801 -	-	-	0	3	3
4779 -	-	-	4847	4847	4850 -	1	2	3
4906 -	-	-	5008 -	-	-	3	0	3
5918 -	-	-	5973 -	-	-	3	0	3
6392	6392	6393 -	6474 -	-	-	1	2	3
6436	6432	6440 -	6486	6482	6486 -	1	2	3
6499 -	-	+	6518 -	-	-	0	3	3

6505	-	-	+	6512	-	-	-	3	0	3
6932	-	-	-	7068	-	-	-	0	3	3
7063	7061	7063	-	7194	-	-	-	3	0	3
7664	7664	7668	-	7717	7714	7717	-	0	3	3
7707	7705	7707	-	7748	-	-	-	0	3	3
8884	8880	8884	-	8952	-	-	-	2	1	3
9205	9201	9207	-	9302	9302	9304	-	0	3	3
9824	-	-	-	9875	-	-	-	3	0	3
10117	10115	10120	-	10231	-	-	-	3	0	3
10248	-	-	+	12673	-	-	-	3	0	3
10268	10266	10268	-	10341	-	-	-	3	0	3
10702	-	-	-	10753	-	-	-	3	0	3
10728	10727	10728	-	10784	-	-	-	3	0	3
11100	11097	11100	+	15128	15128	15129	-	1	2	3
11557	-	-	-	15053	-	-	+	0	3	3
12094	-	-	-	12123	-	-	+	3	0	3
12200	12196	12200	+	14562	-	-	-	0	3	3
13597	13597	13598	+	14918	14917	14918	-	0	3	3
13623	-	-	-	14824	-	-	-	1	2	3
14283	14280	14285	-	14955	-	-	+	0	3	3
14365	14364	14368	-	14454	-	-	-	0	3	3
14496	14493	14501	-	14529	-	-	+	0	3	3
14496	14493	14501	-	14648	14648	14650	-	2	1	3
14510	14502	14513	-	15055	-	-	-	0	3	3
14510	14502	14513	-	15063	-	-	-	3	0	3
14515	14514	14519	-	14623	14623	14624	-	0	3	3
14515	14514	14519	-	14688	14685	14688	+	0	3	3
14521	14518	14524	+	14821	-	-	-	1	2	3
14522	14520	14526	-	14640	14640	14642	-	0	3	3
14522	14520	14526	-	15071	15069	15071	-	1	2	3
14528	14525	14530	+	15156	15153	15157	-	2	1	3
14532	14527	14535	-	14563	-	-	+	1	2	3
14532	14527	14535	-	15003	-	-	-	2	1	3
14541	14537	14544	-	14701	14701	14705	-	1	2	3
14546	14543	14550	+	15112	15109	15113	+	0	3	3
14552	14550	14558	-	15080	-	-	-	2	1	3
14594	14591	14597	+	15157	15157	15161	-	0	3	3
14594	14591	14597	+	15244	15244	15246	-	0	3	3
14604	14601	14610	-	14689	14688	14689	-	2	1	3
14604	14601	14610	-	14858	14858	14861	+	2	1	3
14617	14612	14619	-	14776	14776	14779	-	3	0	3
14630	14629	14637	-	14684	14684	14686	+	1	2	3
14631	14628	14634	+	15168	15168	15172	-	0	3	3
14639	14636	14641	+	14631	-	-	-	3	0	3
14639	14636	14641	+	14762	14758	14762	-	3	0	3
14639	14636	14641	+	15112	-	-	-	0	3	3
14646	14643	14650	+	14676	-	-	-	0	3	3
14650	14649	14652	-	14750	-	-	+	3	0	3
14659	14655	14659	-	14751	-	-	-	0	3	3
14659	14655	14659	-	14922	-	-	-	0	3	3
14670	14667	14673	-	14766	-	-	-	2	1	3
14677	14674	14679	-	14856	14856	14859	-	3	0	3
14685	14681	14688	-	14745	-	-	-	3	0	3
14685	14681	14688	-	14848	-	-	-	3	0	3
14697	14695	14699	+	15071	15070	15071	-	2	1	3
14699	14697	14706	-	14840	-	-	-	3	0	3
14703	14703	14707	+	15171	15170	15171	+	0	3	3
14710	14707	14710	-	14843	-	-	-	3	0	3
14716	14711	14717	+	14973	-	-	+	3	0	3
14722	14721	14726	-	14815	-	-	-	0	3	3
14728	14727	14730	-	14778	-	-	-	0	3	3
14736	14731	14736	-	14764	-	-	+	3	0	3
14736	14731	14736	-	14838	-	-	-	2	1	3
14736	14731	14736	-	14873	14869	14873	-	1	2	3
14779	14776	14780	-	14819	14819	14821	+	2	1	3
14792	14788	14792	+	14850	-	-	-	2	1	3
14822	14818	14829	-	14909	-	-	-	3	0	3
14822	14818	14829	-	14914	-	-	-	0	3	3
14837	14835	14841	+	14915	14913	14915	-	3	0	3
14838	14833	14841	-	14937	14933	14937	+	0	3	3
14848	14842	14848	-	14899	-	-	+	3	0	3
14853	14850	14854	-	14878	-	-	+	3	0	3
14853	14850	14854	-	15009	15009	15012	-	2	1	3
14853	14850	14854	-	15029	15029	15032	-	2	1	3
14861	14855	14864	-	14916	-	-	-	1	2	3
14861	14855	14864	-	15027	15027	15031	-	3	0	3
14862	14858	14866	+	14842	-	-	-	0	3	3
14874	14869	14877	-	14913	-	-	+	3	0	3
14874	14869	14877	-	14920	14920	14924	+	1	2	3
14874	14869	14877	-	14936	-	-	+	0	3	3
14874	14869	14877	-	15014	15014	15017	-	2	1	3

14878	14878	14880	+	14866	14866	14867	-	1	2	3
14879	14878	14882	-	14933 -	-		+	0	3	3
14879	14878	14882	-	14943 -	-		-	3	0	3
14900	14897	14901	-	15017	15017	15019	-	1	2	3
14905	14903	14908	-	14929 -	-		+	2	1	3
14905	14903	14908	-	14934	14934	14935	+	1	2	3
14905	14903	14908	-	14957 -	-		+	0	3	3
14905	14903	14908	-	15008 -	-		-	3	0	3
14911	14910	14914	-	14971 -	-		-	3	0	3
14926	14925	14931	-	15003 -	-		+	0	3	3
14943	14939	14948	-	14970	14968	14970	+	1	2	3
14943	14939	14948	-	15003	14999	15003	+	2	1	3
14943	14939	14948	-	15040	15040	15042	-	0	3	3
14943	14939	14948	-	15171 -	-		+	0	3	3
14953	14951	14957	-	15053	15053	15057	-	3	0	3
14969	14969	14972	+	14984 -	-		-	0	3	3
14969	14969	14972	+	15005 -	-		-	0	3	3
14969	14969	14972	+	15025 -	-		-	0	3	3
14978	14974	14982	+	15073 -	-		-	3	0	3
15001	14999	15006	-	15115	15111	15115	-	1	2	3
15001	14999	15006	-	15125 -	-		-	3	0	3
15013	15010	15013	-	15092	15092	15096	-	3	0	3
15013	15010	15013	-	15112	15110	15112	-	0	3	3
15013	15010	15013	-	15155	15155	15157	-	1	2	3
15019	15014	15022	-	15096	15096	15097	+	0	3	3
15032 -	-		+	15160 -	-		-	3	0	3
15049	15048	15053	+	15058 -	-		-	0	3	3
15066	15062	15069	-	15246 -	-		-	2	1	3
15108	15104	15110	-	15166 -	-		-	0	3	3
15108	15104	15110	-	15191 -	-		-	3	0	3
15117	15113	15121	-	15194	15194	15195	-	3	0	3
15144	15142	15147	-	15214 -	-		-	3	0	3
15154	15153	15155	+	15246 -	-		-	2	1	3
15159	15156	15159	-	15221 -	-		-	3	0	3
15211	15207	15211	+	15237 -	-		-	0	3	3
