# Supplementary material for: Identification and quantification of defective virus genomes in high throughput sequencing data using DVG-profiler, a novel post-sequence alignment processing algorithm
Source: PLoS One. 2019 May 17;14(5):e0216944. doi: 10.1371/journal.pone.0216944 (PMC6524942; doi:10.1371/journal.pone.0216944)
Supplement: S15 Table — (PDF) [file pone.0216944.s020.pdf]

Position (left)	Group start (left)	Group end (left)	Strandness (left)	Position (right)	Group start (right)	Group end (right)	Strandness (right)	Forward hits	Reverse hits	fwd and reverse
14932	14932	14935	-	15291	15291	15292	+	264	258	522
5843	5840	5843	-	10004	10004	10007	+	28	22	50
1750	1750	1753	-	6646	6646	6649	-	4	26	30
4799	-	-	+	6623	-	-	+	4	15	19
4815	4815	4818	-	15333	-	-	-	0	14	14
1719	-	-	-	6616	-	-	-	10	0	10
6372	-	-	-	6529	-	-	-	5	5	10
3646	3646	3648	-	6646	6646	6650	-	0	8	8
10942	10942	10943	+	10989	10989	10990	+	4	4	8
11906	-	-	-	12036	-	-	-	4	4	8
1752	1750	1752	+	6648	6646	6648	+	3	3	6
3629	-	-	-	6629	-	-	-	6	0	6
3820	-	-	+	4334	-	-	+	3	3	6
4211	-	-	+	4246	-	-	+	3	3	6
4251	4249	4251	-	4421	4419	4421	-	3	3	6
9813	-	-	-	10097	-	-	-	3	3	6
10636	10636	10639	+	10651	-	-	-	6	0	6
240	-	-	+	1266	-	-	+	3	2	5
714	-	-	-	2700	-	-	-	2	3	5
996	-	-	-	1678	-	-	-	2	3	5
5859	-	-	-	9985	-	-	+	0	5	5
15054	15054	15056	+	15127	15125	15127	+	3	2	5
15122	-	-	+	15316	-	-	-	2	3	5
49	-	-	-	597	-	-	-	2	2	4
390	390	392	+	1116	-	-	+	2	2	4
476	476	478	+	2782	2782	2783	+	2	2	4
1572	-	-	+	3816	-	-	-	2	2	4
1743	-	-	+	15336	-	-	+	4	0	4
1923	-	-	+	3023	-	-	+	2	2	4
2099	2099	2101	+	2338	2338	2339	+	2	2	4
3667	-	-	-	3819	-	-	-	2	2	4
3783	3779	3783	+	4328	-	-	+	4	0	4
3783	3779	3783	+	4334	-	-	+	2	2	4
3974	-	-	+	4135	-	-	+	2	2	4
4436	-	-	+	5379	-	-	+	2	2	4
4801	-	-	-	6625	-	-	-	0	4	4
5717	-	-	-	6243	-	-	-	2	2	4
6691	-	-	-	7168	-	-	-	2	2	4
7524	-	-	+	7693	-	-	+	2	2	4
7540	-	-	+	7583	-	-	+	2	2	4
9473	-	-	-	10618	-	-	-	0	4	4
10797	10797	10798	-	12019	12019	12020	-	2	2	4
11304	-	-	+	13085	-	-	+	2	2	4
11452	11452	11453	+	13134	-	-	-	2	2	4
11500	-	-	+	11513	11513	11514	+	2	2	4
12560	-	-	-	12826	-	-	-	2	2	4
13275	13273	13275	-	14954	-	-	-	2	2	4
13547	-	-	+	13713	-	-	+	2	2	4
13557	-	-	+	13662	-	-	+	2	2	4
14341	-	-	+	14364	-	-	+	2	2	4
14937	14937	14939	-	15287	15287	15292	+	0	4	4
855	851	855	-	882	-	-	+	0	3	3
4815	4815	4818	-	6641	6640	6641	-	0	3	3
5369	-	-	-	5720	-	-	-	0	3	3
5859	5859	5861	+	9986	9986	9988	-	2	1	3
6810	-	-	+	6949	-	-	+	3	0	3
8739	-	-	-	9938	-	-	-	1	2	3
8829	-	-	+	8952	-	-	+	0	3	3
10636	10636	10639	+	10911	-	-	+	1	2	3
12281	-	-	+	12315	-	-	+	0	3	3
