# Supplementary material for: Identification and quantification of defective virus genomes in high throughput sequencing data using DVG-profiler, a novel post-sequence alignment processing algorithm
Source: PLoS One. 2019 May 17;14(5):e0216944. doi: 10.1371/journal.pone.0216944 (PMC6524942; doi:10.1371/journal.pone.0216944)
Supplement: S16 Table — (PDF) [file pone.0216944.s021.pdf]

Position (left)	Group start (left)	Group end (left)	Strandness (left)	Position (right)	Group start (right)	Group end (right)	Strandness (right)	Forward hits	Reverse hits	fwd and reverse
14932	14929	14935	-	15291	15291	15294	+	6903	8922	15825
5843	5840	5844	-	10004	10003	10008	+	330	212	542
4799	4798	4799	+	6623	6622	6623	+	112	46	158
14937	14937	14939	-	15291	15287	15295	+	54	89	143
1752	1748	1752	-	6648	6645	6649	-	33	33	66
4801	4798	4805	-	6625	6625	6626	-	34	28	62
5860	5857	5861	+	9987	9986	9988	-	14	22	36
10943	10941	10944	+	10990	10989	10991	+	16	17	33
5859	5856	5863	-	9985	9984	9985	+	5	22	27
13024	13022	13025	+	13063	13061	13063	+	13	14	27
4098	4095	4102	+	7399	7399	7402	+	16	10	26
4119	4116	4124	+	7399	7399	7403	+	18	6	24
904	900	908	+	7398	7396	7402	+	13	9	22
5834	5834	5836	+	10015	10013	10015	-	18	4	22
3646	3643	3648	-	6646	6643	6649	-	3	15	18
3820	3818	3822	+	4334	4331	4334	+	7	10	17
1530	1530	1533	-	15127	15123	15132	+	16	0	16
875	874	877	-	8472	-	-	-	1	14	15
15129	15126	15130	-	15134	15132	15135	+	15	0	15
13189	13188	13193	+	15127	15124	15128	+	14	0	14
814	809	815	-	869	869	873	+	6	7	13
4816	4815	4819	-	15333	15332	15335	-	2	11	13
15132	15130	15136	+	15196	15196	15197	+	6	7	13
1527	1523	1530	+	7400	-	-	+	12	0	12
3452	3449	3452	-	15006	-	-	+	6	6	12
513	509	515	+	15065	15061	15067	-	8	3	11
702	700	705	-	15130	15126	15133	+	10	1	11
1756	1756	1759	+	3655	3655	3659	+	4	7	11
4334	4332	4337	-	15134	15133	15138	+	7	4	11
4816	4815	4819	-	6640	6640	6641	-	3	8	11
6682	6681	6688	-	15127	15127	15130	+	9	2	11
904	900	908	+	7253	7252	7253	+	5	5	10
1729	1728	1730	-	6625	6624	6628	-	9	1	10
4011	4008	4014	+	7398	7398	7399	+	3	7	10
5608	5608	5611	+	5693	5693	5696	+	4	6	10
10636	-	-	+	10651	-	-	-	9	1	10
3629	3624	3629	-	6629	6624	6629	-	7	2	9
3777	3774	3777	-	4333	4332	4333	-	5	4	9
8475	-	-	+	12332	-	-	-	7	2	9
875	874	877	-	7268	-	-	+	1	7	8
2478	2475	2480	+	7400	-	-	+	8	0	8
3674	-	-	-	4116	-	-	-	4	4	8
4439	4435	4440	+	11149	11148	11149	+	4	4	8
4762	4757	4765	+	6672	6672	6674	+	4	4	8
6124	6122	6128	+	6160	-	-	+	6	2	8
13959	13959	13961	-	15127	15127	15129	+	8	0	8
917	913	920	+	7401	7400	7401	+	4	3	7
2138	2138	2140	+	11147	11147	11148	+	4	3	7
5152	-	-	-	11844	-	-	+	3	4	7
5459	-	-	+	5577	-	-	+	3	4	7
5650	5647	5650	+	14512	-	-	-	7	0	7
7186	7184	7186	-	11613	11611	11613	+	3	4	7
7892	7892	7895	+	13151	-	-	+	4	3	7
12718	-	-	-	14582	-	-	+	0	7	7
15117	15115	15118	-	15127	15123	15127	+	6	1	7
1	-	-	-	387	-	-	+	3	3	6
77	74	78	+	213	210	214	+	4	2	6
351	348	355	+	1532	-	-	+	3	3	6
418	416	421	+	5428	-	-	-	3	3	6
540	536	543	+	15052	-	-	-	3	3	6
555	551	559	+	15054	-	-	-	3	3	6
697	696	699	-	15135	15132	15139	+	6	0	6
819	816	822	-	1177	1176	1178	-	3	3	6
825	-	-	-	857	-	-	+	1	5	6
1177	1175	1181	+	7400	7398	7400	+	3	3	6
1451	1448	1455	-	15130	15127	15130	+	6	0	6
1719	-	-	-	6616	-	-	-	6	0	6
1838	1838	1842	+	13658	-	-	+	3	3	6
2010	2010	2012	+	4661	-	-	+	3	3	6
2815	2815	2818	+	7403	7400	7403	+	4	2	6
2868	2864	2870	+	13479	-	-	+	3	3	6
3900	3900	3904	-	4384	-	-	-	3	3	6
3990	3990	3994	-	4003	-	-	+	2	4	6
4038	4033	4041	+	15297	-	-	-	6	0	6
4334	4332	4337	-	15129	15125	15130	+	4	2	6
4430	4426	4432	+	11538	-	-	+	3	3	6
4762	4757	4765	+	9465	-	-	+	3	3	6
5451	-	-	-	5620	-	-	-	3	3	6
6677	-	-	-	15196	-	-	+	3	3	6
7379	7379	7383	+	7409	7409	7413	+	3	3	6
8770	-	-	+	10739	-	-	+	3	3	6
12707	12705	12707	-	14593	14593	14595	+	5	1	6
13184	13184	13186	+	15127	15127	15128	+	5	1	6
13189	13188	13193	+	15134	15132	15134	+	5	1	6
15124	15124	15125	-	15126	15126	15127	+	6	0	6
423	419	426	-	4372	-	-	-	2	3	5
449	445	453	-	8524	-	-	-	2	3	5
791	787	795	+	809	809	812	+	2	3	5
814	809	815	-	15127	15127	15129	+	3	2	5
1520	1519	1522	+	7397	-	-	+	5	0	5

1762		1759	1765	-	15356		15352	15356	-	0	5	5
2093		2093	2097	+	2218	-	-		+	0	5	5
2296	-	-		-	15126	-	-		+	3	2	5
2673	-	-		-	3248		3248	3250	-	0	5	5
3378		3375	3382	+	4334	-	-		+	4	1	5
3447		3447	3451	+	7397	-	-		+	3	2	5
4047		4044	4051	+	15168	-	-		-	1	4	5
4116		4112	4116	-	10090	-	-		-	3	2	5
4174		4174	4177	+	13801		13801	13805	-	2	3	5
4247		4244	4250	-	4425		4422	4425	-	2	3	5
4482		4482	4485	-	15199	-	-		+	3	2	5
4801		4798	4805	-	4809	-	-		-	5	0	5
4860		4859	4864	+	4958		4957	4958	+	4	1	5
5184		5181	5184	+	5340		5337	5340	+	3	2	5
5399		5395	5401	+	7400	-	-		+	4	1	5
6333		6329	6336	-	15128		15126	15129	+	5	0	5
6682		6681	6688	-	15133		15132	15133	+	5	0	5
7387		7387	7390	+	15062	-	-		-	3	2	5
7400		7398	7404	-	9382		9382	9385	-	0	5	5
8117		8113	8119	+	8301	-	-		+	3	2	5
8521		8521	8523	+	8578	-	-		+	0	5	5
9265		9263	9265	-	15127		15124	15127	+	4	1	5
9460	-	-		+	9492	-	-		+	2	3	5
10172		10171	10172	-	14550	-	-		+	2	3	5
15066		15064	15067	-	15126		15124	15126	+	5	0	5
15198		15198	15202	+	15177		15173	15181	+	5	0	5
30		27	34	-	626	-	-		-	1	3	4
60	-	-		-	263	-	-		-	2	2	4
251		249	251	+	4452	-	-		+	2	2	4
262		257	266	-	4204	-	-		-	2	2	4
262		257	266	-	8106	-	-		-	2	2	4
262		257	266	-	9310		9308	9310	-	2	2	4
276		274	277	-	4427	-	-		-	2	2	4
281		278	282	-	5372		5368	5372	-	2	2	4
283		279	283	+	9255	-	-		-	2	2	4
296		292	300	-	924	-	-		+	2	2	4
327		327	330	+	351	-	-		+	2	2	4
327		327	330	+	5362	-	-		+	2	2	4
345		345	348	-	7816	-	-		-	2	2	4
360		359	362	-	15179	-	-		-	2	2	4
402		401	403	-	10296	-	-		+	2	2	4
423		419	426	-	8524	-	-		-	1	3	4
423		419	426	-	9493		9493	9495	-	2	2	4
449		445	453	-	15298	-	-		+	2	2	4
466		463	466	+	15350	-	-		-	1	3	4
474		472	480	-	15126		15126	15127	+	4	0	4
491		491	493	-	1310		1308	1310	-	2	2	4
527		522	532	+	9305		9305	9306	-	2	2	4
540		536	543	+	4056	-	-		-	2	2	4
540		536	543	+	14225	-	-		-	2	2	4
555		551	559	+	9305	-	-		-	2	2	4
555		551	559	+	15357	-	-		+	2	2	4
697		696	699	-	15129		15125	15129	+	4	0	4
700		698	700	+	814		814	815	+	2	2	4
700		698	700	+	14626	-	-		-	2	2	4
702		700	705	-	3230		3228	3230	-	2	2	4
720		716	721	-	904	-	-		-	2	2	4
750		747	753	-	905	-	-		-	2	2	4
769		764	770	-	944		942	944	-	2	2	4
769		764	770	-	4037		4037	4038	-	2	2	4
769		764	770	-	4662	-	-		-	2	2	4
774		774	775	-	996		996	997	-	2	2	4
796		793	799	-	4011	-	-		-	2	2	4
814		809	815	-	5412	-	-		-	2	2	4
843	-	-		-	865	-	-		+	1	3	4
904		900	908	+	6024	-	-		+	2	2	4
917		913	920	+	7252	-	-		+	3	1	4
1014		1013	1017	-	10311	-	-		+	2	2	4
1058		1057	1062	+	1542	-	-		+	2	2	4
1158		1158	1162	-	4428		4424	4429	-	3	1	4
1158		1158	1162	-	6921	-	-		+	2	2	4
1310		1306	1313	+	11598	-	-		+	2	2	4
1310		1306	1313	+	13465	-	-		-	2	2	4
1530		1530	1533	-	14658	-	-		+	2	2	4
1533		1532	1534	+	7400		7399	7400	+	4	0	4
1540		1536	1544	+	7400	-	-		+	2	2	4
1588		1586	1593	+	10957	-	-		-	2	2	4
1588		1586	1593	+	12945		12945	12946	-	2	2	4
1611		1611	1612	+	14296		14296	14297	-	2	2	4
1676		1674	1678	+	2311	-	-		-	2	2	4
1687		1684	1692	+	15151	-	-		-	2	2	4
1752		1748	1752	-	15339		15338	15340	-	3	1	4
1923		1920	1926	+	2768		2766	2768	+	3	1	4
1923		1920	1926	+	7252		7252	7253	+	2	2	4
1991		1991	1995	-	2800		2800	2801	+	2	2	4
2010		2010	2012	+	6670	-	-		+	2	2	4
2093		2093	2097	+	4457	-	-		+	2	2	4
2133	-	-		+	12759	-	-		-	2	2	4
2144		2144	2145	-	4230		4230	4231	-	2	2	4
2208		2208	2210	-	2800	-	-		+	2	2	4
2257		2257	2260	+	2247	-	-		-	2	2	4

2257		2257	2260	+	2400	-	-		-	2	2	4
2315		2313	2319	+	12676	-	-		-	2	2	4
2417		2417	2421	+	9394	-	-		-	2	2	4
2437		2435	2438	+	9306		9306	9307	-	2	2	4
2834		2834	2839	+	12804	-	-		-	2	2	4
2858		2858	2860	+	8017		8015	8017	+	2	2	4
3083		3083	3084	+	3961	-	-		+	2	2	4
3148		3145	3149	-	7396		7396	7397	+	2	2	4
3268	-	-		-	13406	-	-		+	2	2	4
3297	-	-		-	7331	-	-		-	2	2	4
3349		3347	3349	+	15205	-	-		-	2	2	4
3641	-	-		+	3681	-	-		+	2	2	4
3782		3782	3783	-	4385		4383	4385	-	2	2	4
3782		3782	3785	+	4583	-	-		+	2	2	4
3825		3824	3825	+	4056	-	-		+	2	2	4
3876		3874	3876	-	9566	-	-		+	2	2	4
3882		3881	3882	-	5399	-	-		-	2	2	4
3893		3889	3895	-	4176	-	-		-	2	2	4
3941		3941	3944	-	7448	-	-		-	2	2	4
3989		3988	3989	+	4054	-	-		+	3	1	4
4011		4008	4014	+	4062		4062	4066	+	1	3	4
4028		4025	4028	+	4062	-	-		+	2	2	4
4038		4033	4041	+	4613	-	-		+	2	2	4
4047		4044	4051	+	4396	-	-		-	2	2	4
4056		4054	4062	+	6492		6492	6493	-	1	3	4
4066		4064	4067	-	15127	-	-		+	2	2	4
4072		4071	4072	+	14767	-	-		-	2	2	4
4098		4095	4102	+	12471	-	-		-	2	2	4
4105		4105	4106	+	7399		7399	7400	+	2	2	4
4119		4116	4124	+	12161	-	-		+	2	2	4
4164		4161	4164	-	4762	-	-		-	2	2	4
4179		4179	4180	+	15156		15156	15157	-	2	2	4
4203		4203	4207	+	15236	-	-		-	2	2	4
4238		4235	4238	-	9141	-	-		+	2	2	4
4246		4244	4246	+	4660		4660	4661	+	2	2	4
4266	-	-		-	4762	-	-		-	2	2	4
4287		4287	4289	+	4352		4350	4352	+	2	2	4
4329		4329	4333	+	4355	-	-		+	2	2	4
4334		4332	4337	-	4764		4764	4765	-	2	2	4
4334		4332	4337	-	14265	-	-		+	2	2	4
4334		4332	4337	-	15184	-	-		+	2	2	4
4430		4426	4432	+	6426	-	-		+	2	2	4
4430		4426	4432	+	8492	-	-		+	2	2	4
4430		4426	4432	+	13805	-	-		-	3	1	4
4471		4471	4474	+	4661	-	-		+	3	1	4
4471		4471	4474	+	8947	-	-		-	2	2	4
4642	-	-		-	5398	-	-		-	2	2	4
4747	-	-		+	5999	-	-		+	2	2	4
4756		4755	4756	+	6661	-	-		+	2	2	4
4762		4757	4765	+	5396		5396	5397	-	2	2	4
4762		4757	4765	+	6024	-	-		+	2	2	4
4762		4757	4765	+	7304	-	-		+	2	2	4
4770		4769	4773	+	7670	-	-		+	2	2	4
4809		4805	4809	+	4804		4804	4805	+	4	0	4
4817		4815	4817	+	6641	-	-		+	4	0	4
4849	-	-		+	4959	-	-		+	3	1	4
6165	-	-		+	14824	-	-		+	0	4	4
6185		6182	6185	-	15133	-	-		+	2	2	4
6236		6234	6236	-	11774	-	-		+	4	0	4
6244		6244	6246	-	6518		6518	6519	-	2	2	4
6256	-	-		+	6402	-	-		+	2	2	4
6333		6329	6336	-	15064	-	-		+	2	2	4
6536	-	-		+	10028	-	-		+	2	2	4
6642		6640	6646	+	15065	-	-		-	3	1	4
6682		6681	6688	-	7362	-	-		-	2	2	4
6682		6681	6688	-	15064		15064	15065	+	3	1	4
6734		6730	6735	+	11580	-	-		+	2	2	4
6768		6768	6771	+	13881	-	-		+	2	2	4
6945		6944	6947	-	8075		8075	8076	-	2	2	4
6971	-	-		-	11832	-	-		+	2	2	4
7151	-	-		+	14157	-	-		+	1	3	4
7170		7170	7171	-	9319		9318	9319	+	1	3	4
7223	-	-		-	7385	-	-		-	2	2	4
7240	-	-		+	11657	-	-		-	2	2	4
7252		7252	7256	-	15305	-	-		-	2	2	4
7346		7342	7349	-	7629		7629	7630	-	4	0	4
7346		7342	7349	-	15203	-	-		+	2	2	4
7451		7451	7453	+	7516	-	-		+	2	2	4
7670		7666	7670	-	12919	-	-		-	2	2	4
8148	-	-		-	12341	-	-		-	2	2	4
8388	-	-		-	10451	-	-		-	2	2	4
8527	-	-		+	8584	-	-		+	4	0	4
8667	-	-		+	9473	-	-		+	2	2	4
8743		8739	8743	-	8866	-	-		-	2	2	4
8743		8739	8743	-	9307	-	-		-	2	2	4
8922	-	-		-	13548	-	-		-	2	2	4
9572	-	-		-	10148	-	-		+	2	2	4
10134	-	-		-	14106	-	-		+	2	2	4
10806	-	-		-	11935	-	-		-	2	2	4
10859		10859	10861	-	12063	-	-		+	2	2	4
11650		11649	11650	+	11832	-	-		+	2	2	4

11884	11882	11884 -	12185 -	-	-	2	2	4
13013	13013	13014 +	13052 -	-	+	2	2	4
13797	13795	13797 +	15190	15190	15191 +	2	2	4
13814 -	-	+	15107 -	-	+	2	2	4
13866	13864	13866 +	13939	13937	13939 +	3	1	4
13971 -	-	+	15153 -	-	+	0	4	4
14091 -	-	-	14326 -	-	+	2	2	4
14243	14243	14244 -	14330	14330	14331 -	2	2	4
14795	14795	14797 -	15059	15057	15059 -	2	2	4
14807 -	-	-	15065 -	-	-	2	2	4
14928 -	-	+	15350 -	-	-	2	2	4
15066	15064	15067 -	15245 -	-	-	2	2	4
15132	15130	15136 +	15164 -	-	+	0	4	4
15302	15302	15303 +	15306	15306	15309 -	0	4	4
15350	15347	15350 +	15307	15307	15310 -	2	2	4
30	27	34 -	15064 -	-	+	1	2	3
30	27	34 -	15127 -	-	+	3	0	3
42 -	-	-	15133	15133	15135 +	3	0	3
98	98	100 -	13077 -	-	+	2	1	3
281	278	282 -	7827 -	-	-	1	2	3
331	329	335 -	597 -	-	-	3	0	3
345	345	348 -	4197 -	-	-	2	1	3
360	360	364 +	3452 -	-	+	3	0	3
423	419	426 -	3199 -	-	+	1	2	3
423	419	426 -	4662 -	-	-	1	2	3
423	419	426 -	4955 -	-	-	1	2	3
423	419	426 -	15296	15296	15297 +	1	2	3
439 -	-	+	5797 -	-	+	0	3	3
449	445	453 -	11659 -	-	-	1	2	3
507	502	507 +	8485 -	-	+	2	1	3
507	502	507 +	15133 -	-	-	1	2	3
513	509	515 +	12632	12631	12632 -	3	0	3
513	509	515 +	15059 -	-	-	3	0	3
555	551	559 +	7346	7346	7349 +	2	1	3
673 -	-	+	835 -	-	+	2	1	3
769	764	770 -	3439	3439	3440 -	0	3	3
769	764	770 -	3782 -	-	-	0	3	3
796	793	799 -	956	955	956 -	1	2	3
796	793	799 -	1675	1673	1675 -	0	3	3
814	809	815 -	6999	6999	7000 -	2	1	3
819	816	822 -	13763	13763	13764 -	2	1	3
904	900	908 +	15060 -	-	-	2	1	3
917	913	920 +	13799 -	-	-	0	3	3
944	941	946 +	1002 -	-	+	0	3	3
944	941	946 +	15230 -	-	-	0	3	3
1096	1096	1097 +	4065 -	-	+	2	1	3
1096	1096	1097 +	8733	8729	8733 +	2	1	3
1152 -	-	-	4422 -	-	-	1	2	3
1177	1175	1181 +	13433 -	-	-	1	2	3
1177	1175	1181 +	15061	15061	15062 -	2	1	3
1211	1208	1211 -	15127 -	-	+	3	0	3
1248	1248	1252 +	15202	15202	15203 -	1	2	3
1266	1263	1270 -	3206 -	-	-	3	0	3
1461	1457	1461 -	15134	15130	15134 +	2	1	3
1477	1474	1478 -	4764	4764	4766 -	1	2	3
1490	1486	1491 -	15134 -	-	+	3	0	3
1527	1523	1530 +	4247 -	-	+	1	2	3
1527	1523	1530 +	13800	13800	13801 -	2	1	3
1537	1536	1538 -	10026 -	-	+	3	0	3
1552	1547	1555 -	8524 -	-	-	1	2	3
1581	1581	1584 -	4019 -	-	+	2	1	3
1582	1581	1584 +	15350 -	-	-	3	0	3
1600	1598	1600 +	7071 -	-	+	1	2	3
1623	1623	1627 +	7246 -	-	+	2	1	3
1750	1746	1750 +	6646 -	-	+	2	1	3
1762	1759	1765 -	6655 -	-	-	0	3	3
1905	1902	1905 +	7400	7399	7400 +	2	1	3
2237	2233	2239 +	15197 -	-	-	0	3	3
2243 -	-	+	7400 -	-	+	3	0	3
2630 -	-	-	2834 -	-	-	0	3	3
3136 -	-	-	12709 -	-	+	0	3	3
3203	3199	3205 +	13004 -	-	+	1	2	3
3349	3347	3349 +	13795	13795	13797 -	2	1	3
3408	3408	3411 +	4334 -	-	+	3	0	3
3463	3459	3464 -	15006	15004	15006 +	1	2	3
3623	3621	3623 -	4799 -	-	-	3	0	3
3651 -	-	-	6648 -	-	-	2	1	3
3667 -	-	-	4100	4098	4100 -	2	1	3
3831	3831	3835 +	4308 -	-	+	2	1	3
3867	3865	3872 +	3936	3934	3936 +	2	1	3
3871	3869	3872 -	7388 -	-	-	0	3	3
3882	3881	3882 -	4424	4424	4426 -	3	0	3
3893	3889	3895 -	4388	4385	4388 -	1	2	3
3990	3990	3994 -	15135	15134	15135 +	3	0	3
4011	4008	4014 +	4055	4055	4056 +	1	2	3
4047	4044	4051 +	15296	15295	15296 -	3	0	3
4059	4055	4059 -	4778	4776	4778 -	1	2	3
4066	4064	4069 +	6993 -	-	+	3	0	3
4098	4095	4102 +	14385	14385	14389 -	2	1	3
4098	4095	4102 +	15209 -	-	-	1	2	3
4114	4110	4114 +	7400 -	-	+	3	0	3

4179		4175	4180	-	15134		15134	15135	+	1	2	3
4226		4222	4226	-	6990		6988	6990	-	1	2	3
4226		4222	4226	-	13040	-	-		+	2	1	3
4324		4323	4328	-	15134	-	-		+	3	0	3
4334		4332	4337	-	7451		7451	7453	-	2	1	3
4334		4332	4337	-	10119		10119	10122	+	1	2	3
4334		4332	4337	-	13735		13734	13735	+	1	2	3
4334		4332	4337	-	14596		14592	14596	+	2	1	3
4355		4354	4356	-	15132	-	-		+	3	0	3
4398		4396	4402	+	7399		7397	7399	+	3	0	3
4398		4396	4402	+	13978	-	-		-	2	1	3
4430		4426	4432	+	15191		15191	15194	-	0	3	3
4447		4445	4449	+	10210		10207	10210	-	2	1	3
4471		4471	4474	+	4548	-	-		+	1	2	3
4539	-	-		-	15127		15126	15127	+	3	0	3
4545	-	-		+	7399	-	-		+	2	1	3
4580		4576	4580	-	15126		15126	15127	+	3	0	3
4672		4671	4672	-	9260		9260	9261	+	1	2	3
4733		4731	4735	+	15222		15221	15222	-	1	2	3
4778		4778	4782	+	10648	-	-		+	1	2	3
4833		4833	4834	-	7172		7172	7173	-	1	2	3
4946		4946	4950	-	5689	-	-		-	0	3	3
4997		4993	5000	+	15297		15295	15297	-	3	0	3
5013	-	-		+	15284	-	-		-	3	0	3
5107		5107	5111	+	12096	-	-		-	3	0	3
5221		5217	5221	+	15296		15296	15297	-	3	0	3
5269	-	-		+	5334	-	-		+	3	0	3
5412		5412	5413	+	5690	-	-		+	2	1	3
5424		5424	5425	-	15127		15127	15129	+	2	1	3
5619		5619	5623	+	9256		9255	9256	+	0	3	3
5794	-	-		-	13961	-	-		+	3	0	3
5859		5856	5863	-	9980	-	-		+	2	1	3
5860		5857	5861	+	15131		15127	15131	+	3	0	3
5866	-	-		-	9985	-	-		+	0	3	3
6012		6010	6014	-	6676	-	-		-	0	3	3
6019		6019	6024	-	15133		15130	15134	+	3	0	3
6128	-	-		-	10761	-	-		+	0	3	3
6421		6418	6421	+	15296	-	-		-	2	1	3
6642		6640	6646	+	15053	-	-		-	2	1	3
6725	-	-		-	7540	-	-		-	3	0	3
7086		7085	7088	+	8249	-	-		+	3	0	3
7187		7187	7189	+	15060	-	-		-	3	0	3
7247	-	-		-	14743		14741	14743	-	0	3	3
7292		7288	7292	+	11580	-	-		-	1	2	3
7346		7345	7350	+	7901	-	-		+	0	3	3
7379		7379	7383	+	15066	-	-		+	3	0	3
7400		7398	7404	-	15183		15183	15187	-	0	3	3
7458		7456	7458	+	14951	-	-		-	2	1	3
8214	-	-		-	15202	-	-		-	0	3	3
8424		8420	8424	-	15132	-	-		+	3	0	3
8600	-	-		+	15132	-	-		+	3	0	3
8870		8869	8870	-	15127		15124	15127	+	3	0	3
9298		9294	9298	+	11881	-	-		-	3	0	3
9308		9307	9311	-	9550	-	-		-	3	0	3
9763	-	-		-	15138	-	-		+	1	2	3
9908	-	-		-	15128	-	-		+	3	0	3
11021	-	-		+	11070	-	-		+	2	1	3
11884		11882	11884	-	12156		12155	12156	-	2	1	3
11899		11897	11899	-	12194		12194	12195	-	1	2	3
12330		12329	12330	+	14208	-	-		-	0	3	3
15124		15124	15125	-	15131		15131	15133	+	3	0	3
15148		15148	15149	+	15166		15166	15167	+	2	1	3
