# Supplementary material for: Identification and quantification of defective virus genomes in high throughput sequencing data using DVG-profiler, a novel post-sequence alignment processing algorithm
Source: PLoS One. 2019 May 17;14(5):e0216944. doi: 10.1371/journal.pone.0216944 (PMC6524942; doi:10.1371/journal.pone.0216944)
Supplement: S17 Table — (PDF) [file pone.0216944.s022.pdf]

Position (left)	Group start (left)	Group end (left)	Strandness (left)	Position (right)	Group start (right)	Group end (right)	Strandness (right)	Forward hits	Reverse hits	fwd and reverse
14932	14927	14937	-	15291	15290	15296	+	7623	10421	18044
15174	15172	15178	-	15204	15200	15204	+	31	29	60
10943	10942	10944	+	10990	10989	10991	+	21	21	42
13024	13022	13027	+	13063	13061	13063	+	14	16	30
1448	1444	1454	-	15133	15129	15133	+	27	2	29
11154	-	-	+	12586	-	-	+	0	28	28
4098	4094	4102	+	7399	7399	7402	+	16	11	27
14939	14938	14941	-	15292	15291	15292	+	6	18	24
904	900	907	+	7400	7398	7402	+	13	9	22
1527	1523	1530	+	7400	-	-	+	15	7	22
1729	1726	1731	-	6625	6625	6628	-	6	15	21
13189	13185	13193	+	15127	15124	15128	+	20	1	21
15128	15122	15130	-	15133	15132	15136	+	17	4	21
3820	3817	3825	+	4334	4331	4334	+	10	10	20
4816	4813	4819	-	6640	6637	6642	-	4	15	19
4119	4116	4124	+	7399	7399	7400	+	11	7	18
5399	5396	5403	-	5402	5400	5402	+	15	3	18
10602	10602	10604	-	10679	10678	10680	+	18	0	18
1476	1473	1482	+	15296	15296	15297	-	16	0	16
2478	2477	2481	+	7400	7399	7400	+	9	7	16
4860	4856	4861	+	4958	4957	4958	+	12	4	16
5687	5683	5690	+	7397	7397	7400	+	8	8	16
883	883	886	-	8480	-	-	-	0	15	15
1448	1444	1454	-	15127	15123	15127	+	15	0	15
4809	4805	4809	+	4798	4798	4802	+	9	6	15
699	696	702	-	15131	15129	15133	+	14	0	14
814	809	816	-	873	-	-	+	6	7	13
1528	1524	1533	-	15127	15125	15128	+	12	1	13
1582	1579	1585	+	14932	-	-	+	10	3	13
3450	3447	3454	-	15006	-	-	+	6	7	13
3646	3645	3650	-	6646	6645	6647	-	7	6	13
4334	4330	4337	-	15134	15133	15138	+	8	5	13
769	764	772	-	944	942	944	-	2	10	12
1685	1680	1688	+	15296	15295	15298	-	11	1	12
4821	4821	4823	-	6647	6644	6647	-	7	5	12
4853	4849	4853	+	4963	4959	4963	+	6	6	12
6940	6935	6943	+	7400	7399	7401	+	7	5	12
513	508	516	+	15065	15061	15065	-	8	3	11
1757	1756	1760	+	3656	3655	3658	+	4	7	11
4011	4008	4012	+	7398	7398	7399	+	4	7	11
6125	6122	6127	+	6159	6157	6160	+	7	4	11
15215	15215	15218	+	15254	15250	15254	+	5	6	11
904	900	907	+	7253	7252	7253	+	5	5	10
2293	2290	2295	-	15126	15126	15129	+	8	2	10
2311	2308	2316	+	11150	-	-	+	10	0	10
2667	2667	2669	-	3248	3248	3250	-	5	5	10
4334	4330	4337	-	15129	15126	15130	+	8	2	10
13189	13185	13193	+	15134	15132	15136	+	9	1	10
769	764	772	-	3129	-	-	-	4	5	9
1522	1520	1522	+	7399	7397	7399	+	5	4	9
1533	1532	1536	+	7400	7398	7400	+	5	4	9
1629	1629	1632	+	1667	-	-	+	6	3	9
3777	3774	3780	-	4333	4331	4333	-	5	4	9
4114	4110	4114	+	7400	-	-	+	9	0	9
4437	4435	4440	+	11149	11148	11150	+	4	5	9
4801	4798	4805	-	4809	-	-	-	9	0	9
5629	5628	5633	-	15134	15132	15134	+	9	0	9
6674	6670	6678	-	15128	15127	15132	+	6	3	9
15117	15114	15119	-	15127	15124	15131	+	8	1	9
796	793	800	-	1669	1669	1673	-	5	3	8
1528	1524	1533	-	4949	4949	4950	-	4	4	8
1752	1749	1752	-	6647	6645	6647	-	2	6	8
1757	1753	1759	-	3656	3655	3656	-	0	8	8
3672	-	-	-	4114	-	-	-	4	4	8
4066	4064	4069	-	7456	7456	7458	+	4	4	8
4762	4759	4765	+	6675	6672	6676	+	4	4	8
4762	4759	4765	+	7667	-	-	+	4	4	8
4809	4805	4809	+	4804	-	-	+	8	0	8
5270	-	-	+	5335	-	-	+	4	4	8
5609	5605	5612	+	5694	5694	5696	+	3	5	8
6668	6668	6669	+	15297	15295	15297	-	8	0	8
9262	9258	9265	-	15127	15123	15127	+	6	2	8
14754	14754	14756	+	14941	-	-	-	4	4	8
15132	15130	15133	+	15196	-	-	+	4	4	8
276	275	279	-	1282	1281	1283	-	3	4	7
877	874	877	-	7268	-	-	+	1	6	7
915	915	918	+	7400	-	-	+	4	3	7
1270	-	-	-	3206	-	-	-	4	3	7
1274	1271	1275	+	15296	-	-	-	7	0	7
1685	1680	1688	+	15229	15225	15229	-	5	2	7
2093	2093	2097	+	2218	-	-	+	2	5	7
2142	2138	2142	+	11150	11148	11150	+	4	3	7
3408	3407	3412	+	4334	4334	4336	+	3	4	7

3476	3472	3477 -	14932 -	-	+	4	3	7
5152	5152	5153 -	11844 -	-	+	3	4	7
6137	6137	6140 -	6145 -	-	+	4	3	7
7186	7183	7186 -	11611 -	-	+	3	4	7
7400	7398	7404 -	14932 -	-	+	1	6	7
7551	7547	7556 +	7580	7580	7583 -	0	7	7
7892	7889	7895 +	13150	13150	13151 +	4	3	7
15128	15122	15130 -	15126	15126	15127 +	7	0	7
351	348	355 +	1532 -	-	+	3	3	6
420	416	424 +	5428 -	-	-	3	3	6
477	473	481 +	1289 -	-	-	3	3	6
478	473	480 -	8496	8492	8496 -	3	3	6
540	536	544 +	15052 -	-	-	3	3	6
555	552	559 +	15054 -	-	-	3	3	6
687	684	691 -	15136	15132	15136 +	6	0	6
819	817	823 -	1177	1176	1177 -	3	3	6
825 -	-	-	857 -	-	+	1	5	6
915	915	918 +	7252 -	-	+	3	3	6
915	915	918 +	13800 -	-	-	3	3	6
1176	1175	1179 +	7400	7399	7400 +	3	3	6
1176	1175	1179 +	8727 -	-	-	3	3	6
1261	1257	1265 -	15127	15127	15129 +	5	1	6
1447	1442	1450 +	4243	4243	4247 +	3	3	6
1528	1524	1533 -	7388 -	-	-	3	3	6
1528	1524	1533 -	15132	15132	15133 +	6	0	6
1675	1671	1679 +	15134 -	-	-	3	3	6
1685	1680	1688 +	15211	15209	15211 -	3	3	6
1838	1838	1842 +	13658 -	-	+	3	3	6
2014	2010	2016 +	4661	4661	4662 +	3	3	6
2236	2235	2239 +	15197 -	-	-	3	3	6
2394	2389	2397 -	15128	15126	15129 +	6	0	6
2691	2688	2692 -	4660	4660	4661 -	3	3	6
2815	2815	2818 +	7403	7400	7403 +	4	2	6
3130	3129	3134 -	12711 -	-	+	3	3	6
3380	3379	3384 +	4334 -	-	+	3	3	6
3654	3652	3656 +	6649 -	-	+	6	0	6
3831	3826	3835 +	4305	4305	4309 +	3	3	6
3879	3874	3884 -	4424	4424	4427 -	3	3	6
3900	3899	3903 -	4386 -	-	-	3	3	6
4038	4038	4041 +	15297 -	-	-	6	0	6
4098	4094	4102 +	8447 -	-	+	3	3	6
4113	4109	4116 -	10089	10089	10090 -	3	3	6
4174	4172	4177 +	13801	13801	13805 -	3	3	6
4430	4426	4434 +	11538 -	-	+	3	3	6
4430	4426	4434 +	13805	13801	13805 -	4	2	6
4482	4481	4486 -	15199 -	-	+	3	3	6
4545	4545	4549 +	7399	7399	7400 +	3	3	6
4762	4759	4765 +	6022	6020	6022 +	3	3	6
4762	4759	4765 +	9468	9465	9468 +	3	3	6
4762	4759	4765 +	14563 -	-	-	3	3	6
4773	4769	4773 +	6126 -	-	+	3	3	6
4947	4944	4951 -	5689 -	-	-	3	3	6
5451	5450	5455 -	5620 -	-	-	3	3	6
5794	5794	5798 -	13960 -	-	+	3	3	6
6014	6010	6014 -	6675 -	-	-	3	3	6
6128	6124	6128 -	10761	10761	10765 +	3	3	6
6674	6670	6678 -	15194 -	-	+	3	3	6
6685	6681	6688 -	15127	15127	15128 +	6	0	6
6730	6727	6735 +	6781	6778	6781 +	4	2	6
6945	6944	6947 +	14807	14807	14808 -	3	3	6
7151 -	-	+	14152 -	-	+	3	3	6
7346	7343	7350 +	7901	7901	7902 +	3	3	6
7378	7376	7381 +	7409	7409	7413 +	3	3	6
7400	7398	7404 -	9382	9382	9385 -	1	5	6
7456	7453	7458 +	7475	7475	7477 +	4	2	6
8521	8521	8524 +	8578 -	-	+	0	6	6
8711 -	-	+	11525 -	-	+	3	3	6
9463	9460	9463 +	9495	9492	9495 +	3	3	6
9934	9932	9938 -	15134	15132	15134 +	6	0	6
10172	10172	10173 -	14550	14550	14553 +	3	3	6
11901	11897	11901 -	12196	12192	12196 -	1	5	6
11980 -	-	+	15226 -	-	+	3	3	6
12524	12521	12524 -	15350	15348	15350 +	3	3	6
13183	13179	13184 +	15127	15125	15127 +	6	0	6
13801	13796	13806 +	15198	15195	15198 +	3	3	6
15066	15061	15070 -	15126	15125	15126 +	5	1	6
15138	15138	15139 +	15199	15199	15200 +	3	3	6
29	29	32 -	625	625	626 -	2	3	5
276	275	279 -	7827	7824	7827 -	3	2	5
390	387	393 -	735	734	736 -	2	3	5
469	468	469 +	15351 -	-	-	1	4	5
507	502	507 +	8482	8482	8485 +	3	2	5
526	523	529 +	8453	8452	8453 +	4	1	5
622	619	626 +	1177	1176	1177 +	3	2	5

687	684	691 -	1081	1081	1083 -	3	2	5
795	791	799 +	809	809	813 +	2	3	5
814	809	816 -	15129	15127	15129 +	3	2	5
1060	1057	1065 +	15294	15294	15296 -	3	2	5
1211	1208	1214 -	15127	15127	15128 +	5	0	5
1447	1442	1450 +	4176 -	-	+	3	2	5
1452	1452	1457 +	15298	15296	15298 -	5	0	5
1460	1456	1462 -	15126	15126	15128 +	5	0	5
1711	1708	1716 +	10497	10495	10497 -	2	3	5
2133	2129	2135 +	15006	15006	15007 -	2	3	5
2243	2243	2246 +	7400 -	-	+	3	2	5
2420	2419	2425 +	7404	7399	7404 +	4	1	5
2430	2427	2433 +	7400	7398	7403 +	3	2	5
3130	3126	3133 +	3160 -	-	+	3	2	5
3213	3212	3217 +	3270	3270	3273 +	2	3	5
3447	3447	3449 +	7397 -	-	+	3	2	5
3450	3447	3454 -	15066	15062	15066 +	2	3	5
3986	3982	3992 -	4007	4003	4007 +	3	2	5
4047	4042	4048 +	15168	15168	15171 -	1	4	5
4081	4081	4086 -	4804	4804	4806 -	3	2	5
4204	4199	4207 +	15235	15234	15235 -	3	2	5
4246	4244	4247 +	4661	4660	4661 +	2	3	5
4247	4244	4250 -	4425	4422	4425 -	2	3	5
4334	4330	4337 -	15184 -	-	+	3	2	5
5050	5048	5051 -	15135	15133	15139 +	5	0	5
5191	5189	5191 +	5345 -	-	+	3	2	5
5221	5217	5222 +	15297	15296	15297 -	5	0	5
5399	5395	5401 +	7400 -	-	+	4	1	5
5465	5462	5466 +	5580 -	-	+	5	0	5
6333	6329	6335 -	15126	15126	15129 +	5	0	5
6455	6455	6459 -	7183 -	-	-	2	3	5
6640	6639	6643 +	15066	15065	15066 -	4	1	5
6685	6681	6688 -	15134	15132	15134 +	5	0	5
6859	6856	6863 -	7062	7062	7066 -	3	2	5
7247 -	-	-	14745	14741	14745 -	2	3	5
7346	7345	7349 -	7629	7629	7630 -	4	1	5
7387	7382	7390 +	15060 -	-	-	3	2	5
7508	7505	7508 +	7600 -	-	+	2	3	5
7539	7535	7542 +	7628	7624	7628 +	0	5	5
8113	8110	8113 +	8297 -	-	+	3	2	5
9402 -	-	+	11924 -	-	-	2	3	5
15144	15141	15145 -	15144	15142	15146 +	3	2	5
15174	15172	15178 -	15181	15177	15182 +	2	3	5
60 -	-	-	263 -	-	-	2	2	4
74	74	76 +	212	210	212 +	1	3	4
83 -	-	+	219 -	-	+	3	1	4
160	160	162 -	7086 -	-	+	2	2	4
187 -	-	+	7399 -	-	+	3	1	4
196	194	198 -	1687 -	-	-	2	2	4
250	248	253 +	4450 -	-	+	2	2	4
257	254	259 -	6939	6939	6942 -	2	2	4
257	254	259 -	8106 -	-	-	2	2	4
266	260	270 -	1292	1292	1295 -	2	2	4
266	260	270 -	4204 -	-	-	2	2	4
266	260	270 -	9310	9308	9312 -	2	2	4
276	275	279 -	4427 -	-	-	2	2	4
283	279	284 +	9255 -	-	-	2	2	4
296	293	296 -	924 -	-	+	2	2	4
327	323	330 +	351	347	351 +	2	2	4
327	323	330 +	5362 -	-	+	2	2	4
345	342	349 -	7816 -	-	-	2	2	4
360	358	362 -	15179 -	-	-	2	2	4
380	377	383 -	735 -	-	-	2	2	4
401	400	404 -	15319	15319	15320 +	2	2	4
418	415	418 -	4373 -	-	-	2	2	4
418	415	418 -	8500 -	-	-	2	2	4
424	419	427 -	9493	9493	9495 -	2	2	4
429	429	433 -	12716	12716	12718 +	2	2	4
438	435	442 -	8509 -	-	-	2	2	4
447	446	450 -	8524 -	-	-	2	2	4
447	446	450 -	11659 -	-	-	2	2	4
447	446	450 -	15298	15298	15299 +	2	2	4
456	453	459 -	11881 -	-	+	2	2	4
456	453	459 -	15230 -	-	+	2	2	4
486	481	489 -	15004 -	-	+	2	2	4
489	484	494 +	1582 -	-	-	2	2	4
494	491	496 -	1311 -	-	-	2	2	4
498	496	501 +	516 -	-	+	2	2	4
498	496	501 +	3035 -	-	-	2	2	4
498	496	501 +	8490	8487	8490 +	2	2	4
513	508	516 +	1046 -	-	+	2	2	4
513	508	516 +	12632	12631	12632 -	4	0	4
540	536	544 +	4056 -	-	-	2	2	4
540	536	544 +	14225 -	-	-	2	2	4

555	552	559	+	6493	-	-	-	2	2	4
555	552	559	+	9306	-	-	-	2	2	4
555	552	559	+	15357	-	-	+	2	2	4
556	555	558	-	1466	-	-	-	2	2	4
556	555	558	-	14757	14755	14757	+	2	2	4
565	561	566	+	7252	-	-	+	2	2	4
593	592	595	-	1215	-	-	-	2	2	4
613	612	613	+	5465	-	-	-	2	2	4
674	-	-	+	835	-	-	+	2	2	4
682	678	682	-	8065	8063	8065	-	2	2	4
699	696	702	-	3229	-	-	-	2	2	4
699	696	702	-	15126	15126	15127	+	4	0	4
699	698	700	+	814	814	815	+	2	2	4
699	698	700	+	14627	-	-	-	2	2	4
704	703	707	-	15126	15124	15130	+	2	2	4
709	709	712	-	12022	-	-	+	2	2	4
720	716	724	-	904	-	-	-	2	2	4
750	747	753	-	905	-	-	-	2	2	4
769	764	772	-	3439	3435	3440	-	0	4	4
769	764	772	-	4662	-	-	-	2	2	4
769	764	772	-	14565	14564	14566	-	2	2	4
769	764	772	-	14897	-	-	-	2	2	4
771	767	774	+	4994	-	-	-	2	2	4
771	767	774	+	7646	-	-	+	2	2	4
774	774	778	-	996	996	997	-	2	2	4
796	793	800	-	955	955	956	-	2	2	4
796	793	800	-	3587	-	-	-	2	2	4
796	793	800	-	4011	-	-	-	2	2	4
796	793	800	-	8447	-	-	+	2	2	4
814	809	816	-	4948	-	-	-	2	2	4
814	809	816	-	5412	-	-	-	2	2	4
837	837	840	-	868	-	-	+	1	3	4
837	837	840	-	12711	-	-	+	2	2	4
842	841	842	-	1210	-	-	-	2	2	4
904	900	907	+	1065	1065	1068	+	2	2	4
904	900	907	+	4894	-	-	+	2	2	4
904	900	907	+	6022	-	-	+	2	2	4
905	902	910	-	2766	-	-	+	2	2	4
941	940	944	+	999	998	1003	+	1	3	4
941	940	944	+	15211	15211	15213	-	2	2	4
941	940	944	+	15230	-	-	-	0	4	4
946	945	947	+	15225	-	-	-	4	0	4
956	952	961	+	15293	-	-	-	4	0	4
997	997	1001	-	1555	1553	1555	-	2	2	4
997	997	1001	-	6953	-	-	-	2	2	4
1014	1012	1017	-	10311	-	-	+	2	2	4
1059	1056	1059	-	14939	-	-	+	2	2	4
1060	1057	1065	+	1542	-	-	+	2	2	4
1065	1060	1066	-	1288	-	-	-	2	2	4
1065	1060	1066	-	6239	-	-	-	2	2	4
1081	1077	1083	+	2176	-	-	+	2	2	4
1081	1077	1083	+	14400	-	-	+	2	2	4
1096	1095	1097	+	8729	8729	8730	+	2	2	4
1151	-	-	-	4421	-	-	-	2	2	4
1156	1156	1160	-	6919	-	-	+	2	2	4
1165	1163	1167	+	6012	6010	6012	+	2	2	4
1176	1175	1179	+	13433	-	-	-	2	2	4
1177	1177	1179	-	3129	-	-	-	2	2	4
1211	1208	1214	-	3555	-	-	-	2	2	4
1211	1208	1214	-	7000	-	-	-	2	2	4
1211	1208	1214	-	7331	-	-	-	2	2	4
1248	1246	1250	+	15202	15202	15203	-	2	2	4
1261	1257	1265	-	3175	-	-	-	2	2	4
1261	1257	1265	-	15132	15132	15133	+	4	0	4
1289	1288	1292	+	8713	-	-	-	2	2	4
1289	1288	1292	+	14847	-	-	-	2	2	4
1289	1288	1292	+	15229	15229	15231	-	2	2	4
1294	1293	1295	+	4254	4254	4255	+	2	2	4
1311	1311	1315	+	11598	-	-	+	2	2	4
1311	1311	1315	+	13467	-	-	-	2	2	4
1406	1402	1410	-	5585	-	-	-	2	2	4
1406	1402	1410	-	11234	-	-	-	2	2	4
1448	1444	1454	-	4762	4762	4763	-	2	2	4
1448	1444	1454	-	10137	-	-	+	2	2	4
1460	1456	1462	-	4780	-	-	-	2	2	4
1460	1456	1462	-	15133	15132	15134	+	3	1	4
1466	1465	1466	+	14195	-	-	-	2	2	4
1476	1473	1482	+	7086	-	-	+	2	2	4
1476	1473	1482	+	15252	-	-	-	2	2	4
1490	1488	1491	-	3379	-	-	-	2	2	4
1495	1491	1499	+	15172	-	-	-	2	2	4
1496	1492	1500	-	14608	-	-	+	2	2	4
1522	-	-	-	10459	-	-	+	2	2	4
1527	1523	1530	+	4247	-	-	+	2	2	4

1527	1523	1530 +	13854	13854	13855 -	2	2	4
1527	1523	1530 +	14844	14844	14846 -	2	2	4
1528	1524	1533 -	14658 -	-	+	2	2	4
1533	1532	1536 +	15295	15291	15295 -	4	0	4
1543	1540	1546 -	14472 -	-	+	2	2	4
1551	1546	1553 +	8493	8490	8493 +	2	2	4
1558	1556	1558 +	15186 -	-	-	2	2	4
1563	1560	1565 +	3629 -	-	-	2	2	4
1563	1560	1565 +	7490 -	-	+	2	2	4
1563	1560	1565 +	8480 -	-	+	2	2	4
1570	1566	1574 +	2437	2434	2437 -	2	2	4
1570	1566	1574 +	11858 -	-	+	2	2	4
1587	1586	1590 +	10962 -	-	-	2	2	4
1587	1586	1590 +	12945	12945	12946 -	2	2	4
1587	1586	1590 +	13462 -	-	+	2	2	4
1606	1604	1607 +	7069	7069	7070 +	2	2	4
1610	1606	1611 -	12525 -	-	+	2	2	4
1611	1610	1612 +	14296	14296	14297 -	2	2	4
1623	1620	1625 +	7246 -	-	+	2	2	4
1629 -	-	-	10858 -	-	+	2	2	4
1664	1662	1664 +	15210 -	-	-	2	2	4
1669	1666	1670 +	15230	15230	15231 -	2	2	4
1675	1671	1679 +	2311 -	-	-	2	2	4
1685	1680	1688 +	10338 -	-	+	2	2	4
1685	1680	1688 +	11616 -	-	+	2	2	4
1685	1680	1688 +	12590 -	-	-	2	2	4
1685	1680	1688 +	13189 -	-	+	2	2	4
1685	1680	1688 +	15152 -	-	-	2	2	4
1704	1701	1704 +	6279 -	-	+	2	2	4
1711	1708	1716 +	9305	9304	9306 -	2	2	4
1711	1708	1716 +	10242 -	-	-	2	2	4
1711	1708	1716 +	15213	15210	15213 -	2	2	4
1711	1708	1716 +	15244	15244	15245 -	2	2	4
1719	1717	1719 -	6616 -	-	-	4	0	4
1745	1742	1748 -	15337	15334	15337 -	4	0	4
1890 -	-	-	11827 -	-	+	2	2	4
1905	1902	1906 +	7399	7399	7400 +	2	2	4
1991	1991	1995 -	2801	2798	2801 +	2	2	4
2014	2010	2016 +	6670 -	-	+	2	2	4
2050	2046	2052 +	2227 -	-	+	2	2	4
2093	2093	2097 +	4457 -	-	+	2	2	4
2102	2099	2105 +	6587 -	-	-	2	2	4
2133	2129	2135 +	12759 -	-	-	2	2	4
2147	2146	2147 -	4232	4232	4233 -	2	2	4
2205	2202	2206 -	2798 -	-	+	2	2	4
2260	2260	2263 +	2400 -	-	-	2	2	4
2293	2290	2295 -	6149 -	-	+	2	2	4
2300 -	-	-	2975 -	-	-	2	2	4
2311	2308	2316 +	12676 -	-	-	2	2	4
2322	2318	2325 +	4245 -	-	+	2	2	4
2322	2318	2325 +	8448 -	-	+	2	2	4
2420	2419	2425 +	9395 -	-	-	2	2	4
2438	2435	2439 +	9306	9306	9307 -	2	2	4
2458	2454	2461 +	7400	7400	7401 +	4	0	4
2631	2629	2631 -	2834 -	-	-	0	4	4
2726	2722	2726 -	4098 -	-	-	2	2	4
2835	2834	2839 +	4522	4522	4523 +	2	2	4
2835	2834	2839 +	12804 -	-	-	2	2	4
2846	2846	2850 -	4535 -	-	-	2	2	4
2860	2858	2860 +	8017 -	-	+	2	2	4
2872	2870	2876 +	15295	15295	15297 -	4	0	4
2952 -	-	+	6130 -	-	+	2	2	4
2971	2968	2975 +	6126 -	-	-	2	2	4
2976	2975	2976 -	3329	3329	3330 -	2	2	4
3084	3080	3086 -	13805	13803	13805 -	2	2	4
3087	3084	3087 +	3961 -	-	+	2	2	4
3130	3126	3133 +	15064 -	-	-	4	0	4
3130	3126	3133 +	15366 -	-	-	2	2	4
3148	3144	3149 -	7396	7396	7397 +	2	2	4
3162	3162	3165 +	15006	15003	15006 -	3	1	4
3173	3169	3177 +	3187	3183	3187 +	2	2	4
3185	3180	3187 +	10789 -	-	+	2	2	4
3199	3196	3201 +	10561 -	-	+	2	2	4
3213	3212	3217 +	3249 -	-	+	2	2	4
3244	3243	3248 +	3262	3258	3262 +	0	4	4
3266	3266	3268 -	8492 -	-	-	2	2	4
3266	3266	3268 -	13406 -	-	+	2	2	4
3297	3297	3300 -	7331 -	-	-	2	2	4
3349	3346	3349 +	13797	13796	13799 -	2	2	4
3349	3346	3349 +	15205 -	-	-	2	2	4
3375	3372	3376 +	3460 -	-	+	2	2	4
3375	3372	3376 +	4334 -	-	+	2	2	4
3445	3444	3445 -	6922 -	-	+	2	2	4
3554	3553	3554 +	9981 -	-	-	2	2	4

3572	3567	3579	+	6674	6674	6676	+	2	2	4
3572	3567	3579	+	6687	6685	6687	+	2	2	4
3599	3598	3602	+	4942	-	-	-	2	2	4
3599	3598	3602	+	6574	-	-	-	2	2	4
3623	3620	3626	-	6624	6620	6624	-	0	4	4
3633	3630	3638	+	6260	-	-	+	2	2	4
3641	3641	3645	+	3681	-	-	+	2	2	4
3657	3654	3659	-	4098	4098	4099	-	2	2	4
3691	-	-	-	11993	-	-	-	2	2	4
3777	3774	3780	-	4379	4379	4380	-	2	2	4
3782	3782	3785	+	4583	-	-	+	2	2	4
3831	3826	3835	+	4059	4056	4059	+	2	2	4
3866	3865	3868	+	3936	3934	3936	+	2	2	4
3879	3874	3884	-	5399	-	-	-	2	2	4
3879	3874	3884	-	9559	-	-	+	2	2	4
3879	3874	3884	-	12284	12284	12285	+	2	2	4
3892	3892	3893	+	8489	-	-	+	2	2	4
3893	3892	3897	-	4176	-	-	-	2	2	4
3906	3902	3910	+	3931	-	-	+	2	2	4
3941	3940	3944	-	7448	-	-	-	2	2	4
3955	3953	3955	-	4783	-	-	-	2	2	4
3993	3990	3995	+	4056	-	-	+	3	1	4
3994	3994	3999	-	15135	15133	15135	+	4	0	4
4011	4008	4012	+	4062	4062	4066	+	1	3	4
4027	4025	4027	+	4061	-	-	+	2	2	4
4039	4038	4042	-	7167	-	-	-	2	2	4
4047	4042	4048	+	4396	-	-	-	2	2	4
4056	4054	4056	+	6492	-	-	-	2	2	4
4061	4058	4062	+	7001	-	-	+	2	2	4
4061	4058	4062	+	8454	-	-	+	2	2	4
4066	4064	4069	-	15127	-	-	+	2	2	4
4072	4071	4073	+	14767	-	-	-	2	2	4
4098	4094	4102	+	15212	15212	15213	-	2	2	4
4105	4105	4106	+	7399	7399	7400	+	2	2	4
4107	-	-	-	5076	-	-	-	2	2	4
4113	4109	4116	-	4762	4762	4763	-	2	2	4
4114	4110	4114	+	14842	-	-	-	2	2	4
4119	4116	4124	+	12161	-	-	+	2	2	4
4164	4161	4167	-	4762	-	-	-	2	2	4
4179	4179	4180	+	15156	15156	15157	-	2	2	4
4204	4199	4207	+	6465	-	-	-	2	2	4
4226	4224	4227	-	13040	-	-	+	2	2	4
4238	4235	4238	-	9139	-	-	+	2	2	4
4262	-	-	-	4763	-	-	-	2	2	4
4273	4270	4273	+	6876	-	-	+	2	2	4
4289	4287	4289	+	4352	4350	4352	+	2	2	4
4305	4301	4309	-	4791	-	-	-	2	2	4
4329	4329	4333	+	4355	-	-	+	2	2	4
4334	4330	4337	-	4764	4764	4765	-	2	2	4
4334	4330	4337	-	14265	-	-	+	2	2	4
4351	4348	4354	+	4346	4344	4347	+	4	0	4
4355	4354	4356	-	11106	-	-	+	2	2	4
4355	4354	4356	-	15132	15132	15136	+	4	0	4
4398	4397	4402	+	7399	7399	7400	+	3	1	4
4421	4418	4424	+	7070	-	-	+	2	2	4
4430	4426	4434	+	6426	-	-	+	2	2	4
4430	4426	4434	+	8489	-	-	+	2	2	4
4471	4470	4472	+	4661	-	-	+	3	1	4
4471	4470	4472	+	8949	-	-	-	2	2	4
4524	4522	4528	-	4697	-	-	-	2	2	4
4580	4576	4580	-	15126	15126	15128	+	4	0	4
4613	4609	4617	+	4665	-	-	+	2	2	4
4613	4609	4617	+	15296	15295	15296	-	2	2	4
4632	-	-	+	15221	-	-	-	2	2	4
4642	4642	4646	-	5398	-	-	-	2	2	4
4735	4734	4736	+	14844	-	-	-	2	2	4
4735	4734	4736	+	15220	-	-	-	2	2	4
4751	4749	4752	+	6001	-	-	+	2	2	4
4757	4754	4758	+	6022	-	-	+	2	2	4
4757	4754	4758	+	6660	-	-	+	2	2	4
4762	4759	4765	+	5396	5396	5397	-	2	2	4
4762	4759	4765	+	7304	-	-	+	2	2	4
4762	4759	4765	+	7645	7645	7648	+	2	2	4
4762	4759	4765	+	7910	7910	7914	+	2	2	4
4791	4788	4792	+	5274	-	-	+	2	2	4
4791	4788	4792	+	15227	-	-	-	2	2	4
4817	4814	4817	+	6641	-	-	+	4	0	4
4942	4942	4946	+	7345	-	-	-	2	2	4
5004	5000	5004	+	15293	-	-	-	4	0	4
5311	5310	5314	+	14692	-	-	-	2	2	4
5334	5330	5334	+	10786	10786	10789	+	2	2	4
5339	5339	5343	+	14173	14173	14174	-	2	2	4
5364	5361	5364	+	15134	-	-	-	2	2	4
5433	5433	5437	-	5659	5659	5660	-	2	2	4

5458	5458	5459 +	5576 -	-	+	0	4	4
5472	5472	5476 +	5513 -	-	+	2	2	4
5529	5529	5530 +	14630 -	-	-	2	2	4
5530	5530	5531 -	5689 -	-	-	2	2	4
5549	5547	5552 +	13592 -	-	+	2	2	4
5549	5547	5552 +	15201 -	-	-	2	2	4
5687	5683	5690 +	7253 -	-	+	2	2	4
5687	5683	5690 +	7385	7385	7386 +	2	2	4
5717	5716	5719 -	6955 -	-	-	2	2	4
5718	5714	5721 +	12740	12740	12741 -	2	2	4
5759	5759	5764 +	5862	5862	5865 +	2	2	4
5787	5783	5787 +	5850	5846	5850 +	2	2	4
5833	5831	5837 -	15005 -	-	-	2	2	4
5895	5895	5896 -	7400 -	-	+	2	2	4
6008 -	-	-	14771 -	-	-	2	2	4
6120 -	-	+	13086 -	-	-	2	2	4
6245	6244	6245 -	6518 -	-	-	2	2	4
6256	6255	6256 +	6401 -	-	+	2	2	4
6290	6288	6290 -	6493 -	-	-	2	2	4
6333	6329	6335 -	15061 -	-	+	2	2	4
6536	6532	6538 +	10028 -	-	+	2	2	4
6674	6670	6678 -	7362 -	-	-	2	2	4
6674	6670	6678 -	14932 -	-	+	2	2	4
6685	6681	6688 -	15061 -	-	+	3	1	4
6691	6690	6693 -	15134	15133	15137 +	4	0	4
6724	6721	6725 +	6953	6953	6954 +	2	2	4
6730	6727	6735 +	11580 -	-	+	2	2	4
6739	6739	6741 +	6790 -	-	+	2	2	4
6739	6739	6741 +	7088	7086	7088 +	3	1	4
6775	6773	6775 +	13881 -	-	+	2	2	4
6859	6856	6863 -	8069 -	-	-	2	2	4
6900	6897	6904 -	8058	8058	8060 -	2	2	4
6915	6912	6915 +	6939	6936	6939 +	2	2	4
6942	6938	6943 -	8073 -	-	-	2	2	4
6950	6948	6950 +	7074 -	-	-	2	2	4
6960	6960	6962 -	8053 -	-	-	2	2	4
6971 -	-	-	11829 -	-	+	2	2	4
7083	7083	7087 +	9073 -	-	-	2	2	4
7132	7128	7133 +	7252 -	-	+	2	2	4
7132	7128	7133 +	7721 -	-	+	2	2	4
7133	7132	7135 -	8601 -	-	+	2	2	4
7170	7166	7171 -	7231 -	-	-	2	2	4
7224	7221	7224 -	7385 -	-	-	2	2	4
7241 -	-	+	11656 -	-	-	2	2	4
7252	7252	7254 -	15305 -	-	-	2	2	4
7292	7288	7292 +	11580 -	-	-	2	2	4
7328	7326	7333 +	8001	7999	8001 -	2	2	4
7346	7345	7349 -	14642 -	-	+	2	2	4
7346	7345	7349 -	15203 -	-	+	2	2	4
7346	7343	7350 +	14971 -	-	-	2	2	4
7363	7362	7363 +	14971 -	-	-	2	2	4
7400	7398	7404 -	8603 -	-	+	2	2	4
7400	7398	7404 -	14575 -	-	+	2	2	4
7400	7398	7404 -	15183	15183	15187 -	1	3	4
7408	7407	7412 -	10087 -	-	-	2	2	4
7448	7448	7451 +	7516	7513	7516 +	2	2	4
7451	7449	7451 -	14994 -	-	+	2	2	4
7571	7566	7575 +	15204 -	-	-	2	2	4
7667	7665	7669 -	12919 -	-	-	2	2	4
7898	7897	7902 -	15127	15127	15130 +	2	2	4
7898	7897	7902 -	15154 -	-	+	2	2	4
8016	8014	8019 -	14932 -	-	+	3	1	4
8527 -	-	+	8584 -	-	+	4	0	4
8667	8665	8669 +	9473 -	-	+	2	2	4
8727	8727	8732 +	15234	15231	15234 +	2	2	4
8735 -	-	-	9307 -	-	-	2	2	4
8743 -	-	-	8866 -	-	-	2	2	4
8780 -	-	+	15244 -	-	-	2	2	4
8922	8922	8924 -	13548 -	-	-	2	2	4
8973	8973	8975 -	9989	9989	9990 +	2	2	4
9176 -	-	+	13455 -	-	+	2	2	4
10134	10132	10134 -	14106 -	-	+	2	2	4
10283	10283	10285 +	10373 -	-	-	2	2	4
10786	10783	10786 +	14806 -	-	-	2	2	4
10806 -	-	-	11935 -	-	-	2	2	4
10807 -	-	+	14398 -	-	-	2	2	4
10859	10858	10861 -	12063 -	-	+	2	2	4
11021 -	-	+	11070 -	-	+	2	2	4
11470 -	-	-	15369 -	-	-	2	2	4
11601	11598	11601 -	11847 -	-	-	2	2	4
11649	11647	11652 +	11827	11827	11828 +	2	2	4
11782	11782	11786 -	14354 -	-	+	2	2	4
11884	11882	11884 -	12189 -	-	-	2	2	4
12279	12279	12280 +	12682 -	-	+	2	2	4

12804	-	-	+	13760	-	-	+	2	2	4
13013	-	-	+	13052	-	-	+	2	2	4
13163	13163	13169	+	13187	-	-	+	2	2	4
13183	13179	13184	+	15133	15133	15134	+	4	0	4
13866	-	-	+	13939	-	-	+	3	1	4
14091	-	-	-	14326	-	-	+	2	2	4
14190	-	-	-	15132	15128	15132	+	4	0	4
14244	-	-	-	14331	-	-	-	2	2	4
14743	14743	14745	+	15006	15006	15007	-	2	2	4
14776	-	-	-	15064	-	-	-	2	2	4
14807	-	-	-	15065	-	-	-	2	2	4
14928	14928	14932	+	15351	-	-	-	2	2	4
14932	14927	14937	-	15035	-	-	-	2	2	4
14932	14927	14937	-	15296	-	-	-	2	2	4
15066	15061	15070	-	15247	-	-	-	2	2	4
15138	15135	15139	-	15145	15142	15145	+	4	0	4
15144	15141	15145	-	15155	-	-	+	3	1	4
15190	15190	15193	-	15213	-	-	+	2	2	4
15195	15195	15196	-	15197	15197	15200	+	2	2	4
15198	15198	15199	+	15175	15173	15175	+	2	2	4
15296	15293	15297	+	15237	-	-	+	2	2	4
1	2	5	-	387	-	-	+	2	1	3
29	29	32	-	15061	15061	15062	+	1	2	3
42	-	-	-	15133	15133	15135	+	3	0	3
199	197	204	+	7409	-	-	+	2	1	3
257	254	259	-	6956	6953	6956	-	1	2	3
266	260	270	-	1273	1273	1275	-	1	2	3
266	260	270	-	1565	1562	1565	-	1	2	3
276	275	279	-	1288	1287	1288	-	1	2	3
276	275	279	-	1733	1733	1735	-	3	0	3
281	280	282	-	4800	-	-	-	2	1	3
288	288	291	-	15054	15054	15058	-	2	1	3
331	329	335	-	600	599	600	-	3	0	3
345	342	349	-	4195	4195	4197	-	2	1	3
345	342	349	-	6940	6940	6942	-	1	2	3
360	360	364	+	3450	-	-	+	3	0	3
418	415	418	-	2629	-	-	-	0	3	3
418	415	418	-	8524	-	-	-	1	2	3
420	416	424	+	1097	1096	1097	+	2	1	3
424	419	427	-	3202	3202	3203	+	1	2	3
424	419	427	-	4662	-	-	-	1	2	3
424	419	427	-	15296	15296	15297	+	1	2	3
429	427	429	+	5787	-	-	+	0	3	3
438	435	442	-	622	621	622	-	2	1	3
438	435	442	-	14984	-	-	+	3	0	3
469	466	472	-	9272	9272	9274	+	1	2	3
478	473	480	-	10987	10986	10987	+	1	2	3
478	473	480	-	15126	15126	15127	+	3	0	3
489	484	494	+	4877	-	-	+	2	1	3
507	502	507	+	15042	15042	15045	-	1	2	3
507	502	507	+	15133	-	-	-	1	2	3
513	508	516	+	8453	-	-	+	2	1	3
513	508	516	+	15059	-	-	-	3	0	3
513	508	516	+	15089	-	-	-	2	1	3
555	552	559	+	7346	7346	7349	+	2	1	3
555	552	559	+	8448	8448	8452	+	1	2	3
593	592	595	-	1177	1177	1178	-	0	3	3
649	646	650	+	814	-	-	+	1	2	3
699	696	702	-	3379	-	-	-	1	2	3
720	716	724	-	15133	15133	15134	+	3	0	3
735	734	737	-	10128	-	-	+	1	2	3
769	764	772	-	3782	-	-	-	0	3	3
774	774	778	-	1589	-	-	-	0	3	3
796	793	800	-	915	-	-	-	1	2	3
814	809	816	-	6999	6999	7000	-	2	1	3
819	817	823	-	13763	13763	13764	-	2	1	3
904	900	907	+	15060	-	-	-	2	1	3
905	902	910	-	3783	-	-	-	1	2	3
943	940	947	-	15136	15134	15137	+	2	1	3
1096	1095	1097	+	4065	-	-	+	2	1	3
1176	1175	1179	+	15061	-	-	-	2	1	3
1176	1175	1179	+	15295	15295	15297	-	2	1	3
1196	1191	1198	-	1476	-	-	-	1	2	3
1211	1208	1214	-	7388	-	-	-	2	1	3
1237	1235	1241	-	7345	-	-	-	0	3	3
1281	1278	1282	+	9560	-	-	+	2	1	3
1281	1278	1282	+	15295	15295	15297	-	3	0	3
1294	1291	1294	-	11085	11084	11085	-	2	1	3
1421	1420	1425	+	15340	15339	15340	-	2	1	3
1428	1425	1429	-	15132	15132	15134	+	3	0	3
1432	1428	1433	+	15296	15296	15297	-	3	0	3
1448	1444	1454	-	15061	15060	15061	+	3	0	3
1452	1452	1457	+	7400	-	-	+	2	1	3
1476	1473	1482	+	6949	6949	6952	-	2	1	3

1477	1475	1477	-	4764	4764	4765	-	1	2	3
1490	1488	1491	-	15134	-	-	+	3	0	3
1496	1492	1500	-	15133	15133	15134	+	3	0	3
1527	1523	1530	+	13800	13800	13801	-	2	1	3
1528	1524	1533	-	8496	8493	8496	-	1	2	3
1528	1524	1533	-	15066	15062	15066	+	2	1	3
1537	1536	1538	-	10022	-	-	+	3	0	3
1542	1538	1543	+	7400	-	-	+	1	2	3
1552	1549	1555	-	8524	-	-	-	1	2	3
1563	1560	1565	+	10120	-	-	-	2	1	3
1582	1579	1585	+	7284	-	-	+	1	2	3
1582	1579	1585	+	8453	-	-	+	3	0	3
1582	1579	1585	+	15351	-	-	-	3	0	3
1584	1580	1584	-	4019	-	-	+	2	1	3
1657	1655	1660	+	15352	15351	15352	-	2	1	3
1657	1655	1660	+	15366	15363	15366	-	2	1	3
1675	1671	1679	+	15296	-	-	-	3	0	3
1685	1680	1688	+	2849	2848	2849	+	2	1	3
1685	1680	1688	+	15223	15220	15223	-	1	2	3
1743	1743	1746	+	15333	15333	15335	+	3	0	3
1766	-	-	+	3664	-	-	+	3	0	3
1973	1972	1975	-	10272	-	-	-	2	1	3
2058	2056	2062	+	2840	-	-	+	2	1	3
2058	2056	2062	+	10125	-	-	+	1	2	3
2064	2064	2067	+	13854	-	-	+	1	2	3
2154	2152	2154	-	3266	-	-	-	1	2	3
2336	2332	2340	+	15298	15296	15298	-	2	1	3
2411	2410	2411	-	12521	12521	12522	+	1	2	3
2438	2435	2439	+	7399	7399	7401	+	1	2	3
2443	2443	2447	+	7400	-	-	+	2	1	3
2730	2730	2731	+	15346	-	-	-	2	1	3
2767	2766	2770	-	3554	-	-	-	1	2	3
2908	-	-	-	15245	-	-	-	1	2	3
2921	2917	2921	-	3105	-	-	-	0	3	3
2938	2934	2943	-	4060	4059	4060	-	1	2	3
3173	3169	3177	+	15005	-	-	-	1	2	3
3185	3180	3187	+	9262	-	-	+	2	1	3
3204	3204	3210	+	13006	-	-	+	1	2	3
3220	3219	3225	+	15006	-	-	-	2	1	3
3450	3447	3454	-	15130	15127	15130	+	2	1	3
3492	3490	3492	-	15127	-	-	+	2	1	3
3623	3620	3626	-	4799	-	-	-	3	0	3
3657	3654	3659	-	4610	4610	4613	-	1	2	3
3866	3863	3870	-	7387	-	-	-	0	3	3
3879	3874	3884	-	4388	4387	4388	-	1	2	3
3936	3932	3939	-	4776	4776	4780	-	1	2	3
4011	4008	4012	+	4055	4055	4056	+	1	2	3
4039	4038	4042	-	4809	-	-	-	3	0	3
4039	4038	4042	-	7132	-	-	-	2	1	3
4047	4042	4048	+	15296	15295	15296	-	3	0	3
4066	4064	4068	+	6991	-	-	+	3	0	3
4098	4094	4102	+	14385	14385	14389	-	2	1	3
4099	4097	4104	-	4783	-	-	-	2	1	3
4174	4172	4177	+	14932	-	-	+	2	1	3
4179	4175	4180	-	15134	15134	15135	+	1	2	3
4253	4251	4256	+	7667	7667	7670	+	1	2	3
4273	4270	4273	+	4369	-	-	+	0	3	3
4328	4323	4328	-	15134	-	-	+	3	0	3
4334	4330	4337	-	10119	-	-	+	1	2	3
4334	4330	4337	-	13735	13734	13735	+	1	2	3
4377	4373	4381	-	5797	-	-	-	0	3	3
4398	4397	4402	+	13978	-	-	-	2	1	3
4421	4418	4424	+	7400	7399	7400	+	1	2	3
4430	4426	4434	+	15191	15191	15195	-	0	3	3
4437	4435	4440	+	13799	13799	13800	-	1	2	3
4447	4445	4447	+	10210	10208	10210	-	2	1	3
4471	4470	4472	+	4544	-	-	+	1	2	3
4533	4531	4534	-	15060	15060	15061	+	3	0	3
4533	4531	4534	-	15133	-	-	+	2	1	3
4539	4539	4542	-	15127	15126	15127	+	3	0	3
4539	4537	4542	+	7400	7399	7400	+	2	1	3
4556	4553	4559	+	15297	15296	15297	-	2	1	3
4593	4590	4595	-	15134	15133	15134	+	3	0	3
4657	4653	4657	-	14238	-	-	-	1	2	3
4674	4671	4674	-	9260	9260	9261	+	1	2	3
4762	4759	4765	+	12053	12052	12053	+	2	1	3
4794	4790	4794	-	6618	6615	6618	-	3	0	3
4997	4993	4997	+	15295	15295	15297	-	3	0	3
5107	5106	5111	+	12096	-	-	-	3	0	3
5179	5177	5179	-	5944	5943	5944	-	2	1	3
5311	5310	5314	+	8562	-	-	+	0	3	3
5412	5412	5416	+	5690	-	-	+	2	1	3
5424	5424	5426	-	15127	15127	15129	+	2	1	3
5424	5424	5426	-	15195	-	-	+	1	2	3

5468	5464	5471	-	5639	-	-	-	1	2	3
5619	5619	5623	+	9256	-	-	+	0	3	3
5632	5628	5637	+	6922	6922	6923	+	1	2	3
5851	5851	5854	-	15135	15135	15137	+	3	0	3
6019	6019	6022	-	15133	-	-	+	2	1	3
6149	6149	6151	-	15133	15129	15133	+	3	0	3
6185	-	-	-	15133	-	-	+	1	2	3
6245	6244	6245	-	11848	-	-	+	1	2	3
6257	6255	6257	-	6999	6995	6999	-	2	1	3
6421	-	-	+	15296	-	-	-	2	1	3
6640	6639	6643	+	15061	15060	15061	-	1	2	3
6673	6673	6675	+	15297	15296	15297	-	3	0	3
6725	-	-	-	7540	-	-	-	3	0	3
6823	6823	6826	-	6948	6947	6948	-	1	2	3
6955	6953	6957	+	7023	-	-	+	0	3	3
6955	6953	6957	+	15296	-	-	-	3	0	3
6976	6974	6976	+	9647	-	-	-	2	1	3
7000	6996	7004	+	11543	-	-	+	2	1	3
7000	6996	7004	+	15297	15296	15297	-	2	1	3
7083	7083	7087	+	8249	-	-	+	0	3	3
7089	7089	7093	+	8251	-	-	+	3	0	3
7118	7114	7119	-	8058	-	-	-	1	2	3
7133	7132	7135	-	11508	-	-	-	1	2	3
7187	7187	7189	+	15061	-	-	-	3	0	3
7378	7376	7381	+	15065	-	-	+	3	0	3
7400	7398	7404	-	8134	8134	8136	+	1	2	3
7456	7453	7458	+	14952	-	-	-	2	1	3
7456	7453	7458	+	15133	15130	15135	+	3	0	3
7571	7566	7575	+	7692	-	-	+	0	3	3
7583	7579	7586	+	7673	-	-	+	0	3	3
7583	7579	7586	+	7687	7683	7687	+	0	3	3
7644	7642	7644	-	8058	-	-	-	0	3	3
7850	7849	7851	+	10513	-	-	-	2	1	3
8214	8214	8219	-	15202	-	-	-	0	3	3
8391	8388	8391	+	8436	-	-	+	2	1	3
8416	8416	8420	-	11158	-	-	+	2	1	3
8416	8416	8420	-	15229	-	-	-	1	2	3
8564	8560	8564	-	8622	8622	8626	+	1	2	3
8600	-	-	+	15132	-	-	+	3	0	3
8870	8869	8870	-	15127	15124	15127	+	3	0	3
9298	9294	9298	+	11881	-	-	-	3	0	3
9308	9307	9311	-	9552	-	-	-	3	0	3
9308	9307	9311	-	15133	15129	15134	+	3	0	3
9443	-	-	+	15132	15130	15132	+	3	0	3
9908	-	-	-	15128	-	-	+	3	0	3
10613	-	-	-	14932	-	-	+	2	1	3
11664	11664	11668	+	15133	-	-	+	3	0	3
11866	11863	11866	-	11868	11868	11869	+	2	1	3
11884	11882	11884	-	12156	12155	12156	-	2	1	3
12046	12045	12050	-	14971	14971	14972	+	1	2	3
12170	-	-	+	12223	-	-	+	1	2	3
12330	12329	12330	+	14208	-	-	-	0	3	3
13172	13172	13176	+	15133	-	-	+	2	1	3
13424	13424	13426	-	15154	-	-	+	2	1	3
13801	13796	13806	+	13867	-	-	+	1	2	3
13801	13796	13806	+	15346	15346	15350	-	2	1	3
13957	13953	13957	-	15132	-	-	+	3	0	3
14928	14928	14932	+	14946	14945	14946	-	2	1	3
14932	14927	14937	-	15354	15352	15354	-	2	1	3
14958	14958	14961	+	15127	-	-	+	3	0	3
15000	14995	15000	+	15289	-	-	-	3	0	3
15052	15051	15052	+	15139	15139	15141	+	1	2	3
15144	15141	15145	-	15125	15125	15126	+	1	2	3
15174	15172	15178	-	15197	-	-	-	0	3	3
15182	15178	15184	+	15221	15217	15221	+	1	2	3
15185	15181	15185	-	15216	-	-	+	1	2	3
15213	15209	15215	-	15217	15217	15221	+	1	2	3
15296	15293	15297	+	15258	15256	15258	+	1	2	3
15296	15293	15297	+	15277	15277	15279	+	1	2	3
