# Supplementary material for: Identification and quantification of defective virus genomes in high throughput sequencing data using DVG-profiler, a novel post-sequence alignment processing algorithm
Source: PLoS One. 2019 May 17;14(5):e0216944. doi: 10.1371/journal.pone.0216944 (PMC6524942; doi:10.1371/journal.pone.0216944)
Supplement: S18 Table — (PDF) [file pone.0216944.s023.pdf]

Position (left)	Group start (left)	Group end (left)	Strandness (left)	Position (right)	Group start (right)	Group end (right)	Strandness (right)	Forward hits	Reverse hits	fwd and reverse
14932	14928	14935	-	15291	15291	15294	+	325	355	680
1729	-	-	-	6625	-	-	-	0	17	17
11154	-	-	+	12586	-	-	+	0	12	12
1719	1717	1719	-	6616	-	-	-	10	0	10
6372	-	-	-	6529	-	-	-	5	5	10
3783	3779	3783	+	4328	4324	4328	+	5	4	9
2311	-	-	+	11150	-	-	+	8	0	8
4818	4816	4818	-	6641	6640	6641	-	3	5	8
10942	10942	10943	+	10989	10989	10990	+	4	4	8
11906	-	-	-	12036	-	-	-	4	4	8
4251	4249	4251	-	4421	4419	4421	-	3	4	7
9473	-	-	-	10618	-	-	-	3	4	7
11268	-	-	-	11434	-	-	-	4	3	7
712	710	713	-	2699	2699	2700	-	3	3	6
993	993	996	-	1682	1679	1682	-	3	3	6
1757	1757	1758	-	3656	-	-	-	0	6	6
3820	-	-	+	4334	-	-	+	3	3	6
4211	4211	4215	+	4246	-	-	+	3	3	6
6809	6809	6813	+	6948	-	-	+	3	3	6
7017	-	-	+	8264	-	-	+	3	3	6
7571	-	-	-	13442	-	-	+	3	3	6
8666	8663	8666	-	12923	12920	12923	-	3	3	6
9808	-	-	-	10092	-	-	-	3	3	6
10679	10677	10679	+	10602	10602	10604	-	6	0	6
13557	-	-	+	13662	-	-	+	3	3	6
239	-	-	+	1265	-	-	+	3	2	5
3199	3198	3200	+	3934	3933	3935	+	2	3	5
4794	4790	4794	-	6616	6615	6616	-	5	0	5
12279	-	-	+	12312	-	-	+	2	3	5
15054	15054	15056	+	15127	15125	15127	+	3	2	5
49	49	52	-	599	-	-	-	2	2	4
390	389	391	+	1115	-	-	+	2	2	4
766	766	767	-	1684	-	-	-	2	2	4
1177	-	-	+	1209	-	-	+	2	2	4
1570	1570	1571	+	3817	3817	3818	-	2	2	4
1743	-	-	+	15333	-	-	+	4	0	4
1923	-	-	+	3023	-	-	+	2	2	4
2101	2099	2101	+	2338	2338	2339	+	2	2	4
2133	2131	2133	+	3598	-	-	+	2	2	4
2500	-	-	+	3460	-	-	+	2	2	4
3398	-	-	+	3449	-	-	+	2	2	4
3659	-	-	-	3819	-	-	-	2	2	4
3783	3779	3783	+	4334	-	-	+	2	2	4
3889	3889	3890	-	4174	4174	4175	-	2	2	4
3974	-	-	+	4135	-	-	+	2	2	4
4436	-	-	+	5379	-	-	+	2	2	4
5717	5717	5718	-	6243	-	-	-	2	2	4
6459	-	-	-	6541	-	-	-	2	2	4
6562	-	-	-	6659	-	-	-	4	0	4
6691	-	-	-	7168	-	-	-	2	2	4
6940	-	-	+	8395	-	-	+	2	2	4
7524	-	-	+	7692	-	-	+	2	2	4
7542	-	-	+	7584	-	-	+	2	2	4
9429	9429	9431	+	9496	9494	9496	+	2	2	4
9455	-	-	-	10147	-	-	-	2	2	4
10303	-	-	-	10459	-	-	-	2	2	4
10797	-	-	-	12019	-	-	-	2	2	4
11234	-	-	+	11415	-	-	+	2	2	4
11304	11302	11304	+	13085	13084	13085	+	2	2	4
11448	11448	11449	+	13139	13139	13140	-	2	2	4
11501	-	-	+	11517	-	-	+	2	2	4
11723	11719	11723	+	11810	11809	11810	+	2	2	4
12560	-	-	-	12826	-	-	-	2	2	4
12659	12656	12659	+	12676	12673	12676	+	2	2	4
13275	13273	13275	-	14955	-	-	-	2	2	4
13547	-	-	+	13713	-	-	+	2	2	4
14341	-	-	+	14361	-	-	+	2	2	4
15137	15137	15138	-	15301	15301	15302	-	2	2	4
49	49	52	-	359	-	-	-	1	2	3
245	-	-	+	4274	-	-	+	0	3	3
847	846	847	-	891	-	-	+	0	3	3
883	-	-	-	8480	-	-	-	0	3	3
1551	-	-	+	3366	-	-	+	3	0	3
3382	-	-	+	3934	-	-	+	2	1	3
3906	-	-	+	3931	-	-	+	2	1	3
4150	-	-	+	4150	-	-	+	0	3	3
5369	-	-	-	5720	-	-	-	0	3	3
8737	8737	8738	-	9937	9937	9938	-	1	2	3
8829	-	-	+	8952	-	-	+	0	3	3
10639	-	-	+	10911	-	-	+	1	2	3
14932	14928	14935	-	15307	15306	15307	-	3	0	3
14953	-	-	-	15306	-	-	-	3	0	3
15123	-	-	+	15316	-	-	-	0	3	3
