# Supplementary material for: Identification and quantification of defective virus genomes in high throughput sequencing data using DVG-profiler, a novel post-sequence alignment processing algorithm
Source: PLoS One. 2019 May 17;14(5):e0216944. doi: 10.1371/journal.pone.0216944 (PMC6524942; doi:10.1371/journal.pone.0216944)
Supplement: S19 Table — (DOCX) [file pone.0216944.s024.docx]

================================

= 3' cb/sb DVG

=================================

DVG's type Length BP_Pos RI_Pos Delta_Positions Ref Counts %_to_Virus

3' cb/sb DVG 255 15271 15246 25 88-1961-mod|88-1961-mod 2 0.0%|0.0%

3' cb/sb DVG 679 14953 15140 187 88-1961-mod|88-1961-mod 7 0.0%|0.0%

3' cb/sb DVG 680 14945 15147 202 88-1961-mod|88-1961-mod 10 0.0%|0.0%

3' cb/sb DVG 680 14947 15145 198 88-1961-mod|88-1961-mod 224 0.0%|0.0%

3' cb/sb DVG 680 14948 15144 196 88-1961-mod|88-1961-mod 2 0.0%|0.0%

3' cb/sb DVG 680 14949 15143 194 88-1961-mod|88-1961-mod 31 0.0%|0.0%

3' cb/sb DVG 680 14950 15142 192 88-1961-mod|88-1961-mod 5 0.0%|0.0%

3' cb/sb DVG 680 15140 14952 188 88-1961-mod|88-1961-mod 2 0.0%|0.0%

3' cb/sb DVG 680 15143 14949 194 88-1961-mod|88-1961-mod 185 0.0%|0.0%

3' cb/sb DVG 680 15145 14947 198 88-1961-mod|88-1961-mod 5 0.0%|0.0%

3' cb/sb DVG 680 15147 14945 202 88-1961-mod|88-1961-mod 12 0.0%|0.0%

3' cb/sb DVG 735 14859 15178 319 88-1961-mod|88-1961-mod 9 0.0%|0.0%

3' cb/sb DVG 735 15177 14860 317 88-1961-mod|88-1961-mod 6 0.0%|0.0%

3' cb/sb DVG 818 14786 15168 382 88-1961-mod|88-1961-mod 5 0.0%|0.0%

3' cb/sb DVG 872 14865 15035 170 88-1961-mod|88-1961-mod 2 0.0%|0.0%

3' cb/sb DVG 872 14868 15032 164 88-1961-mod|88-1961-mod 421 0.0%|0.0%

3' cb/sb DVG 872 14869 15031 162 88-1961-mod|88-1961-mod 18 0.0%|0.0%

3' cb/sb DVG 872 14870 15030 160 88-1961-mod|88-1961-mod 131 0.0%|0.0%

3' cb/sb DVG 872 14871 15029 158 88-1961-mod|88-1961-mod 43 0.0%|0.0%

3' cb/sb DVG 872 15029 14871 158 88-1961-mod|88-1961-mod 32 0.0%|0.0%

3' cb/sb DVG 872 15030 14870 160 88-1961-mod|88-1961-mod 465 0.0%|0.0%

3' cb/sb DVG 872 15031 14869 162 88-1961-mod|88-1961-mod 13 0.0%|0.0%

3' cb/sb DVG 872 15032 14868 164 88-1961-mod|88-1961-mod 247 0.0%|0.0%

3' cb/sb DVG 872 15033 14867 166 88-1961-mod|88-1961-mod 2 0.0%|0.0%

3' cb/sb DVG 872 15034 14866 168 88-1961-mod|88-1961-mod 3 0.0%|0.0%

3' cb/sb DVG 872 15035 14865 170 88-1961-mod|88-1961-mod 54 0.0%|0.0%

3' cb/sb DVG 872 15036 14864 172 88-1961-mod|88-1961-mod 3 0.0%|0.0%

3' cb/sb DVG 873 14875 15024 149 88-1961-mod|88-1961-mod 7 0.0%|0.0%

3' cb/sb DVG 923 14905 14944 39 88-1961-mod|88-1961-mod 3 0.0%|0.0%

3' cb/sb DVG 923 14940 14909 31 88-1961-mod|88-1961-mod 3 0.0%|0.0%

3' cb/sb DVG 981 14725 15066 341 88-1961-mod|88-1961-mod 7 0.0%|0.0%

3' cb/sb DVG 981 15066 14725 341 88-1961-mod|88-1961-mod 6 0.0%|0.0%

3' cb/sb DVG 1016 14730 15026 296 88-1961-mod|88-1961-mod 34 0.0%|0.0%

3' cb/sb DVG 1016 14733 15023 290 88-1961-mod|88-1961-mod 12 0.0%|0.0%

3' cb/sb DVG 1016 15023 14733 290 88-1961-mod|88-1961-mod 32 0.0%|0.0%

3' cb/sb DVG 1016 15026 14730 296 88-1961-mod|88-1961-mod 7 0.0%|0.0%

3' cb/sb DVG 1034 15249 14489 760 88-1961-mod|88-1961-mod 3 0.0%|0.0%

3' cb/sb DVG 1094 14761 14917 156 88-1961-mod|88-1961-mod 7 0.0%|0.0%

3' cb/sb DVG 1094 14917 14761 156 88-1961-mod|88-1961-mod 8 0.0%|0.0%

3' cb/sb DVG 1140 15050 14582 468 88-1961-mod|88-1961-mod 3 0.0%|0.0%

3' cb/sb DVG 1184 14662 14926 264 88-1961-mod|88-1961-mod 2 0.0%|0.0%

3' cb/sb DVG 1262 14443 15067 624 88-1961-mod|88-1961-mod 7 0.0%|0.0%

3' cb/sb DVG 1262 15066 14444 622 88-1961-mod|88-1961-mod 2 0.0%|0.0%

3' cb/sb DVG 1268 14801 14703 98 88-1961-mod|88-1961-mod 2 0.0%|0.0%

3' cb/sb DVG 1286 14966 14520 446 88-1961-mod|88-1961-mod 3 0.0%|0.0%

3' cb/sb DVG 1322 14342 15108 766 88-1961-mod|88-1961-mod 116 0.0%|0.0%

3' cb/sb DVG 1322 15106 14344 762 88-1961-mod|88-1961-mod 37 0.0%|0.0%

3' cb/sb DVG 1328 14667 14777 110 88-1961-mod|88-1961-mod 4 0.0%|0.0%

3' cb/sb DVG 1328 14777 14667 110 88-1961-mod|88-1961-mod 4 0.0%|0.0%

3' cb/sb DVG 1328 14778 14666 112 88-1961-mod|88-1961-mod 5 0.0%|0.0%

3' cb/sb DVG 1328 15021 14423 598 88-1961-mod|88-1961-mod 4 0.0%|0.0%

3' cb/sb DVG 1383 14221 15168 947 88-1961-mod|88-1961-mod 9 0.0%|0.0%

3' cb/sb DVG 1383 14223 15166 943 88-1961-mod|88-1961-mod 169 0.0%|0.0%

3' cb/sb DVG 1383 14224 15165 941 88-1961-mod|88-1961-mod 5 0.0%|0.0%

3' cb/sb DVG 1383 15165 14224 941 88-1961-mod|88-1961-mod 344 0.0%|0.0%

3' cb/sb DVG 1383 15166 14223 943 88-1961-mod|88-1961-mod 4 0.0%|0.0%

3' cb/sb DVG 1383 15168 14221 947 88-1961-mod|88-1961-mod 2 0.0%|0.0%

3' cb/sb DVG 1431 14454 14887 433 88-1961-mod|88-1961-mod 2 0.0%|0.0%

3' cb/sb DVG 1431 14456 14885 429 88-1961-mod|88-1961-mod 50 0.0%|0.0%

3' cb/sb DVG 1431 14457 14884 427 88-1961-mod|88-1961-mod 2 0.0%|0.0%

3' cb/sb DVG 1431 14458 14883 425 88-1961-mod|88-1961-mod 3 0.0%|0.0%

3' cb/sb DVG 1431 14883 14458 425 88-1961-mod|88-1961-mod 3 0.0%|0.0%

3' cb/sb DVG 1431 14884 14457 427 88-1961-mod|88-1961-mod 2 0.0%|0.0%

3' cb/sb DVG 1431 14885 14456 429 88-1961-mod|88-1961-mod 88 0.0%|0.0%

3' cb/sb DVG 1460 14987 14325 662 88-1961-mod|88-1961-mod 4 0.0%|0.0%

3' cb/sb DVG 1507 14245 15020 775 88-1961-mod|88-1961-mod 3 0.0%|0.0%

3' cb/sb DVG 1507 15020 14245 775 88-1961-mod|88-1961-mod 24 0.0%|0.0%

3' cb/sb DVG 1508 14238 15026 788 88-1961-mod|88-1961-mod 54 0.0%|0.0%

3' cb/sb DVG 1532 14928 14312 616 88-1961-mod|88-1961-mod 5 0.0%|0.0%

3' cb/sb DVG 1532 14932 14308 624 88-1961-mod|88-1961-mod 7 0.0%|0.0%

3' cb/sb DVG 1537 14867 14368 499 88-1961-mod|88-1961-mod 38 0.0%|0.0%

3' cb/sb DVG 1538 14360 14874 514 88-1961-mod|88-1961-mod 2 0.0%|0.0%

3' cb/sb DVG 1586 13907 15279 1372 88-1961-mod|88-1961-mod 96 0.0%|0.0%

3' cb/sb DVG 1586 13909 15277 1368 88-1961-mod|88-1961-mod 2 0.0%|0.0%

3' cb/sb DVG 1586 15277 13909 1368 88-1961-mod|88-1961-mod 86 0.0%|0.0%

3' cb/sb DVG 1604 14677 14491 186 88-1961-mod|88-1961-mod 2 0.0%|0.0%

3' cb/sb DVG 1604 14679 14489 190 88-1961-mod|88-1961-mod 3 0.0%|0.0%

3' cb/sb DVG 1610 14837 14325 512 88-1961-mod|88-1961-mod 3 0.0%|0.0%

3' cb/sb DVG 1652 14350 14770 420 88-1961-mod|88-1961-mod 19 0.0%|0.0%

3' cb/sb DVG 1652 14770 14350 420 88-1961-mod|88-1961-mod 25 0.0%|0.0%

3' cb/sb DVG 1748 13907 15117 1210 88-1961-mod|88-1961-mod 2 0.0%|0.0%

3' cb/sb DVG 1796 13967 15009 1042 88-1961-mod|88-1961-mod 2 0.0%|0.0%

3' cb/sb DVG 1796 15008 13968 1040 88-1961-mod|88-1961-mod 7 0.0%|0.0%

3' cb/sb DVG 1827 14526 14419 107 88-1961-mod|88-1961-mod 2 0.0%|0.0%

3' cb/sb DVG 1845 14752 14175 577 88-1961-mod|88-1961-mod 2 0.0%|0.0%

3' cb/sb DVG 1868 14739 14165 574 88-1961-mod|88-1961-mod 10 0.0%|0.0%

3' cb/sb DVG 1868 14742 14162 580 88-1961-mod|88-1961-mod 2 0.0%|0.0%

3' cb/sb DVG 1879 14457 14436 21 88-1961-mod|88-1961-mod 2 0.0%|0.0%

3' cb/sb DVG 1922 13863 14987 1124 88-1961-mod|88-1961-mod 9 0.0%|0.0%

3' cb/sb DVG 1922 14987 13863 1124 88-1961-mod|88-1961-mod 2 0.0%|0.0%

3' cb/sb DVG 1946 13731 15095 1364 88-1961-mod|88-1961-mod 2 0.0%|0.0%

3' cb/sb DVG 1959 15032 13781 1251 88-1961-mod|88-1961-mod 5 0.0%|0.0%

3' cb/sb DVG 1994 14019 14759 740 88-1961-mod|88-1961-mod 2 0.0%|0.0%

3' cb/sb DVG 2012 14331 14429 98 88-1961-mod|88-1961-mod 10 0.0%|0.0%

3' cb/sb DVG 2012 14427 14333 94 88-1961-mod|88-1961-mod 6 0.0%|0.0%

3' cb/sb DVG 2012 14428 14332 96 88-1961-mod|88-1961-mod 3 0.0%|0.0%

3' cb/sb DVG 2012 14429 14331 98 88-1961-mod|88-1961-mod 7 0.0%|0.0%

3' cb/sb DVG 2012 14432 14328 104 88-1961-mod|88-1961-mod 3 0.0%|0.0%

3' cb/sb DVG 2025 14446 14301 145 88-1961-mod|88-1961-mod 2 0.0%|0.0%

3' cb/sb DVG 2078 14000 14694 694 88-1961-mod|88-1961-mod 3 0.0%|0.0%

3' cb/sb DVG 2186 13347 15239 1892 88-1961-mod|88-1961-mod 17 0.0%|0.0%

3' cb/sb DVG 2186 15239 13347 1892 88-1961-mod|88-1961-mod 9 0.0%|0.0%

3' cb/sb DVG 2228 13479 15065 1586 88-1961-mod|88-1961-mod 52 0.0%|0.0%

3' cb/sb DVG 2228 15064 13480 1584 88-1961-mod|88-1961-mod 36 0.0%|0.0%

3' cb/sb DVG 2312 13316 15144 1828 88-1961-mod|88-1961-mod 31 0.0%|0.0%

3' cb/sb DVG 2312 13317 15143 1826 88-1961-mod|88-1961-mod 2 0.0%|0.0%

3' cb/sb DVG 2312 15143 13317 1826 88-1961-mod|88-1961-mod 23 0.0%|0.0%

3' cb/sb DVG 2312 15144 13316 1828 88-1961-mod|88-1961-mod 3 0.0%|0.0%

3' cb/sb DVG 2342 13477 14953 1476 88-1961-mod|88-1961-mod 18 0.0%|0.0%

3' cb/sb DVG 2342 14950 13480 1470 88-1961-mod|88-1961-mod 12 0.0%|0.0%

3' cb/sb DVG 2360 13478 14934 1456 88-1961-mod|88-1961-mod 2 0.0%|0.0%

3' cb/sb DVG 2456 14207 14109 98 88-1961-mod|88-1961-mod 2 0.0%|0.0%

3' cb/sb DVG 2468 13749 14555 806 88-1961-mod|88-1961-mod 2 0.0%|0.0%

3' cb/sb DVG 2468 14018 14286 268 88-1961-mod|88-1961-mod 2 0.0%|0.0%

3' cb/sb DVG 2468 14532 13772 760 88-1961-mod|88-1961-mod 3 0.0%|0.0%

3' cb/sb DVG 2468 14544 13760 784 88-1961-mod|88-1961-mod 3 0.0%|0.0%

3' cb/sb DVG 2480 13252 15040 1788 88-1961-mod|88-1961-mod 5 0.0%|0.0%

3' cb/sb DVG 2480 15039 13253 1786 88-1961-mod|88-1961-mod 3 0.0%|0.0%

3' cb/sb DVG 2492 13617 14663 1046 88-1961-mod|88-1961-mod 94 0.0%|0.0%

3' cb/sb DVG 2492 14663 13617 1046 88-1961-mod|88-1961-mod 143 0.0%|0.0%

3' cb/sb DVG 2516 14379 13877 502 88-1961-mod|88-1961-mod 2 0.0%|0.0%

3' cb/sb DVG 2528 13566 14678 1112 88-1961-mod|88-1961-mod 5 0.0%|0.0%

3' cb/sb DVG 2528 14676 13568 1108 88-1961-mod|88-1961-mod 3 0.0%|0.0%

3' cb/sb DVG 2534 13497 14741 1244 88-1961-mod|88-1961-mod 5 0.0%|0.0%

3' cb/sb DVG 2534 14739 13499 1240 88-1961-mod|88-1961-mod 18 0.0%|0.0%

3' cb/sb DVG 2551 14665 13556 1109 88-1961-mod|88-1961-mod 5 0.0%|0.0%

3' cb/sb DVG 2552 13529 14691 1162 88-1961-mod|88-1961-mod 2 0.0%|0.0%

3' cb/sb DVG 2558 13562 14652 1090 88-1961-mod|88-1961-mod 28 0.0%|0.0%

3' cb/sb DVG 2558 14651 13563 1088 88-1961-mod|88-1961-mod 26 0.0%|0.0%

3' cb/sb DVG 2576 13586 14610 1024 88-1961-mod|88-1961-mod 4 0.0%|0.0%

3' cb/sb DVG 2600 13308 14864 1556 88-1961-mod|88-1961-mod 258 0.0%|0.0%

3' cb/sb DVG 2600 13309 14863 1554 88-1961-mod|88-1961-mod 4 0.0%|0.0%

3' cb/sb DVG 2600 14863 13309 1554 88-1961-mod|88-1961-mod 186 0.0%|0.0%

3' cb/sb DVG 2600 14864 13308 1556 88-1961-mod|88-1961-mod 4 0.0%|0.0%

3' cb/sb DVG 2636 13501 14635 1134 88-1961-mod|88-1961-mod 2 0.0%|0.0%

3' cb/sb DVG 2636 14633 13503 1130 88-1961-mod|88-1961-mod 8 0.0%|0.0%

3' cb/sb DVG 2660 13553 14559 1006 88-1961-mod|88-1961-mod 2 0.0%|0.0%

3' cb/sb DVG 2660 14559 13553 1006 88-1961-mod|88-1961-mod 10 0.0%|0.0%

3' cb/sb DVG 2678 13639 14455 816 88-1961-mod|88-1961-mod 2 0.0%|0.0%

3' cb/sb DVG 2697 13751 14324 573 88-1961-mod|88-1961-mod 32 0.0%|0.0%

3' cb/sb DVG 2697 14324 13751 573 88-1961-mod|88-1961-mod 14 0.0%|0.0%

3' cb/sb DVG 2708 13550 14514 964 88-1961-mod|88-1961-mod 31 0.0%|0.0%

3' cb/sb DVG 2708 14514 13550 964 88-1961-mod|88-1961-mod 23 0.0%|0.0%

3' cb/sb DVG 2726 13104 14942 1838 88-1961-mod|88-1961-mod 2 0.0%|0.0%

3' cb/sb DVG 2750 13141 14881 1740 88-1961-mod|88-1961-mod 12 0.0%|0.0%

3' cb/sb DVG 2750 14879 13143 1736 88-1961-mod|88-1961-mod 42 0.0%|0.0%

3' cb/sb DVG 2816 12912 15044 2132 88-1961-mod|88-1961-mod 8 0.0%|0.0%

3' cb/sb DVG 2816 15044 12912 2132 88-1961-mod|88-1961-mod 8 0.0%|0.0%

3' cb/sb DVG 2870 13523 14379 856 88-1961-mod|88-1961-mod 2 0.0%|0.0%

3' cb/sb DVG 2881 12919 14972 2053 88-1961-mod|88-1961-mod 6 0.0%|0.0%

3' cb/sb DVG 2882 12918 14972 2054 88-1961-mod|88-1961-mod 2 0.0%|0.0%

3' cb/sb DVG 2882 12921 14969 2048 88-1961-mod|88-1961-mod 15 0.0%|0.0%

3' cb/sb DVG 2882 14968 12922 2046 88-1961-mod|88-1961-mod 9 0.0%|0.0%

3' cb/sb DVG 2923 13700 14149 449 88-1961-mod|88-1961-mod 2 0.0%|0.0%

3' cb/sb DVG 2991 14631 13150 1481 88-1961-mod|88-1961-mod 2 0.0%|0.0%

3' cb/sb DVG 3026 12797 14949 2152 88-1961-mod|88-1961-mod 5 0.0%|0.0%

3' cb/sb DVG 3026 14948 12798 2150 88-1961-mod|88-1961-mod 4 0.0%|0.0%

3' cb/sb DVG 3128 12903 14741 1838 88-1961-mod|88-1961-mod 4 0.0%|0.0%

3' cb/sb DVG 3128 12904 14740 1836 88-1961-mod|88-1961-mod 4 0.0%|0.0%

3' cb/sb DVG 3128 14740 12904 1836 88-1961-mod|88-1961-mod 9 0.0%|0.0%

3' cb/sb DVG 3278 14247 13247 1000 88-1961-mod|88-1961-mod 3 0.0%|0.0%

3' cb/sb DVG 3285 13330 14157 827 88-1961-mod|88-1961-mod 51 0.0%|0.0%

3' cb/sb DVG 3285 14152 13335 817 88-1961-mod|88-1961-mod 2 0.0%|0.0%

3' cb/sb DVG 3285 14154 13333 821 88-1961-mod|88-1961-mod 11 0.0%|0.0%

3' cb/sb DVG 3290 14370 13112 1258 88-1961-mod|88-1961-mod 2 0.0%|0.0%

3' cb/sb DVG 3344 12591 14837 2246 88-1961-mod|88-1961-mod 8 0.0%|0.0%

3' cb/sb DVG 3344 14836 12592 2244 88-1961-mod|88-1961-mod 69 0.0%|0.0%

3' cb/sb DVG 3368 13629 13775 146 88-1961-mod|88-1961-mod 24 0.0%|0.0%

3' cb/sb DVG 3368 13775 13629 146 88-1961-mod|88-1961-mod 11 0.0%|0.0%

3' cb/sb DVG 3386 14307 13079 1228 88-1961-mod|88-1961-mod 2 0.0%|0.0%

3' cb/sb DVG 3500 12732 14540 1808 88-1961-mod|88-1961-mod 2 0.0%|0.0%

3' cb/sb DVG 3500 14981 12291 2690 88-1961-mod|88-1961-mod 4 0.0%|0.0%

3' cb/sb DVG 3608 13325 13839 514 88-1961-mod|88-1961-mod 25 0.0%|0.0%

3' cb/sb DVG 3608 13837 13327 510 88-1961-mod|88-1961-mod 21 0.0%|0.0%

3' cb/sb DVG 3608 13839 13325 514 88-1961-mod|88-1961-mod 4 0.0%|0.0%

3' cb/sb DVG 3620 13431 13721 290 88-1961-mod|88-1961-mod 24 0.0%|0.0%

3' cb/sb DVG 3620 13719 13433 286 88-1961-mod|88-1961-mod 17 0.0%|0.0%

3' cb/sb DVG 3626 13462 13684 222 88-1961-mod|88-1961-mod 27 0.0%|0.0%

3' cb/sb DVG 3626 13464 13682 218 88-1961-mod|88-1961-mod 2 0.0%|0.0%

3' cb/sb DVG 3626 13684 13462 222 88-1961-mod|88-1961-mod 16 0.0%|0.0%

3' cb/sb DVG 3644 12332 14796 2464 88-1961-mod|88-1961-mod 2 0.0%|0.0%

3' cb/sb DVG 3692 11857 15223 3366 88-1961-mod|88-1961-mod 3 0.0%|0.0%

3' cb/sb DVG 3692 15221 11859 3362 88-1961-mod|88-1961-mod 3 0.0%|0.0%

3' cb/sb DVG 3824 14036 12912 1124 88-1961-mod|88-1961-mod 3 0.0%|0.0%

3' cb/sb DVG 3860 14576 12336 2240 88-1961-mod|88-1961-mod 2 0.0%|0.0%

3' cb/sb DVG 3877 13265 13630 365 88-1961-mod|88-1961-mod 6 0.0%|0.0%

3' cb/sb DVG 3877 13627 13268 359 88-1961-mod|88-1961-mod 5 0.0%|0.0%

3' cb/sb DVG 3974 13707 13091 616 88-1961-mod|88-1961-mod 2 0.0%|0.0%

3' cb/sb DVG 4022 12550 14200 1650 88-1961-mod|88-1961-mod 2 0.0%|0.0%

3' cb/sb DVG 4022 14200 12550 1650 88-1961-mod|88-1961-mod 2 0.0%|0.0%

3' cb/sb DVG 4046 14564 12162 2402 88-1961-mod|88-1961-mod 3 0.0%|0.0%

3' cb/sb DVG 4111 12195 14466 2271 88-1961-mod|88-1961-mod 2 0.0%|0.0%

3' cb/sb DVG 4142 12481 14149 1668 88-1961-mod|88-1961-mod 7 0.0%|0.0%

3' cb/sb DVG 4172 11433 15167 3734 88-1961-mod|88-1961-mod 2 0.0%|0.0%

3' cb/sb DVG 4172 11435 15165 3730 88-1961-mod|88-1961-mod 4 0.0%|0.0%

3' cb/sb DVG 4172 11437 15163 3726 88-1961-mod|88-1961-mod 24 0.0%|0.0%

3' cb/sb DVG 4172 15162 11438 3724 88-1961-mod|88-1961-mod 6 0.0%|0.0%

3' cb/sb DVG 4244 12121 14407 2286 88-1961-mod|88-1961-mod 3 0.0%|0.0%

3' cb/sb DVG 4244 14406 12122 2284 88-1961-mod|88-1961-mod 6 0.0%|0.0%

3' cb/sb DVG 4274 13055 13443 388 88-1961-mod|88-1961-mod 27 0.0%|0.0%

3' cb/sb DVG 4274 13438 13060 378 88-1961-mod|88-1961-mod 2 0.0%|0.0%

3' cb/sb DVG 4274 13442 13056 386 88-1961-mod|88-1961-mod 16 0.0%|0.0%

3' cb/sb DVG 4274 13443 13055 388 88-1961-mod|88-1961-mod 4 0.0%|0.0%

3' cb/sb DVG 4310 11994 14468 2474 88-1961-mod|88-1961-mod 4 0.0%|0.0%

3' cb/sb DVG 4310 14468 11994 2474 88-1961-mod|88-1961-mod 2 0.0%|0.0%

3' cb/sb DVG 4484 13947 12341 1606 88-1961-mod|88-1961-mod 2 0.0%|0.0%

3' cb/sb DVG 4766 11228 14778 3550 88-1961-mod|88-1961-mod 5 0.0%|0.0%

3' cb/sb DVG 4766 14776 11230 3546 88-1961-mod|88-1961-mod 12 0.0%|0.0%

3' cb/sb DVG 4886 14758 11128 3630 88-1961-mod|88-1961-mod 2 0.0%|0.0%

3' cb/sb DVG 5030 11435 14307 2872 88-1961-mod|88-1961-mod 14 0.0%|0.0%

3' cb/sb DVG 5030 14306 11436 2870 88-1961-mod|88-1961-mod 28 0.0%|0.0%

3' cb/sb DVG 5066 15008 10698 4310 88-1961-mod|88-1961-mod 2 0.0%|0.0%

3' cb/sb DVG 5168 11823 13781 1958 88-1961-mod|88-1961-mod 8 0.0%|0.0%

3' cb/sb DVG 5168 13780 11824 1956 88-1961-mod|88-1961-mod 13 0.0%|0.0%

3' cb/sb DVG 6554 10845 13373 2528 88-1961-mod|88-1961-mod 3 0.0%|0.0%

3' cb/sb DVG 6946 13952 9874 4078 88-1961-mod|88-1961-mod 5 0.0%|0.0%

3' cb/sb DVG 7100 8928 14744 5816 88-1961-mod|88-1961-mod 2 0.0%|0.0%

3' cb/sb DVG 13994 2366 14412 12046 88-1961-mod|88-1961-mod 2 0.0%|0.0%

3' cb/sb DVG 15243 646 14883 14237 88-1961-mod|88-1961-mod 2 0.0%|0.0%

3' cb/sb DVG 15243 647 14882 14235 88-1961-mod|88-1961-mod 7 0.0%|0.0%

3' cb/sb DVG 15243 648 14881 14233 88-1961-mod|88-1961-mod 43 0.0%|0.0%

=================================

= 5' cb/sb DVG

=================================

DVG's type Length BP_Pos RI_Pos Delta_Positions Ref Counts %_to_Virus

5' cb/sb DVG 150 63 87 24 88-1961-mod|88-1961-mod 2 0.0%|0.0%

5' cb/sb DVG 178 86 92 6 88-1961-mod|88-1961-mod 4 0.0%|0.0%

5' cb/sb DVG 178 97 81 16 88-1961-mod|88-1961-mod 2 0.0%|0.0%

5' cb/sb DVG 272 130 142 12 88-1961-mod|88-1961-mod 2 0.0%|0.0%

5' cb/sb DVG 1455 756 699 57 88-1961-mod|88-1961-mod 3 0.0%|0.0%

5' cb/sb DVG 17919 4185 13734 9549 88-1961-mod|88-1961-mod 2 0.0%|0.0%

5' cb/sb DVG 20974 5785 15189 9404 88-1961-mod|88-1961-mod 2 0.0%|0.0%

5' cb/sb DVG 26754 15203 11551 3652 88-1961-mod|88-1961-mod 8 0.0%|0.0%

5' cb/sb DVG 28161 14843 13318 1525 88-1961-mod|88-1961-mod 2 0.0%|0.0%

5' cb/sb DVG 28984 14785 14199 586 88-1961-mod|88-1961-mod 2 0.0%|0.0%

5' cb/sb DVG 29386 15180 14206 974 88-1961-mod|88-1961-mod 4 0.0%|0.0%

5' cb/sb DVG 29630 14581 15049 468 88-1961-mod|88-1961-mod 4 0.0%|0.0%

5' cb/sb DVG 29630 15037 14593 444 88-1961-mod|88-1961-mod 2 0.0%|0.0%

5' cb/sb DVG 29687 14875 14812 63 88-1961-mod|88-1961-mod 2 0.0%|0.0%

5' cb/sb DVG 30088 14932 15156 224 88-1961-mod|88-1961-mod 6 0.0%|0.0%

=================================

= Deletion DVG (Fwd. strand)

=================================

DVG's type Length BP_Pos RI_Pos Delta_Positions Ref Counts %_to_Virus

Deletion DVG (Fwd. strand) 1013 661 15034 14372 88-1961-mod|88-1961-mod 2 0.0%|0.0%

Deletion DVG (Fwd. strand) 1183 588 14791 14202 88-1961-mod|88-1961-mod 3 0.0%|0.0%

Deletion DVG (Fwd. strand) 1610 764 14540 13775 88-1961-mod|88-1961-mod 2 0.0%|0.0%

Deletion DVG (Fwd. strand) 1843 637 14180 13542 88-1961-mod|88-1961-mod 2 0.0%|0.0%

Deletion DVG (Fwd. strand) 2359 1900 14927 13026 88-1961-mod|88-1961-mod 2 0.0%|0.0%

Deletion DVG (Fwd. strand) 2575 1951 14762 12810 88-1961-mod|88-1961-mod 2 0.0%|0.0%

Deletion DVG (Fwd. strand) 2635 1927 14678 12750 88-1961-mod|88-1961-mod 2 0.0%|0.0%

Deletion DVG (Fwd. strand) 2677 1944 14653 12708 88-1961-mod|88-1961-mod 7 0.0%|0.0%

Deletion DVG (Fwd. strand) 2767 813 13432 12618 88-1961-mod|88-1961-mod 6 0.0%|0.0%

Deletion DVG (Fwd. strand) 3019 2627 14994 12366 88-1961-mod|88-1961-mod 2 0.0%|0.0%

Deletion DVG (Fwd. strand) 3505 2298 14179 11880 88-1961-mod|88-1961-mod 2 0.0%|0.0%

Deletion DVG (Fwd. strand) 4051 2013 13348 11334 88-1961-mod|88-1961-mod 2 0.0%|0.0%

Deletion DVG (Fwd. strand) 4057 2426 13755 11328 88-1961-mod|88-1961-mod 2 0.0%|0.0%

Deletion DVG (Fwd. strand) 4111 3227 14502 11274 88-1961-mod|88-1961-mod 6 0.0%|0.0%

Deletion DVG (Fwd. strand) 4495 2190 13081 10890 88-1961-mod|88-1961-mod 3 0.0%|0.0%

Deletion DVG (Fwd. strand) 4741 3166 13811 10644 88-1961-mod|88-1961-mod 2 0.0%|0.0%

Deletion DVG (Fwd. strand) 4831 2120 12675 10554 88-1961-mod|88-1961-mod 2 0.0%|0.0%

Deletion DVG (Fwd. strand) 5599 2680 12467 9786 88-1961-mod|88-1961-mod 2 0.0%|0.0%

Deletion DVG (Fwd. strand) 5717 5209 14878 9668 88-1961-mod|88-1961-mod 5 0.0%|0.0%

Deletion DVG (Fwd. strand) 5821 4177 13742 9564 88-1961-mod|88-1961-mod 19 0.0%|0.0%

Deletion DVG (Fwd. strand) 5995 5784 15175 9390 88-1961-mod|88-1961-mod 3 0.0%|0.0%

Deletion DVG (Fwd. strand) 5995 5785 15176 9390 88-1961-mod|88-1961-mod 29 0.0%|0.0%

Deletion DVG (Fwd. strand) 6301 5096 14181 9084 88-1961-mod|88-1961-mod 2 0.0%|0.0%

Deletion DVG (Fwd. strand) 6397 3198 12187 8988 88-1961-mod|88-1961-mod 4 0.0%|0.0%

Deletion DVG (Fwd. strand) 6481 4061 12966 8904 88-1961-mod|88-1961-mod 2 0.0%|0.0%

Deletion DVG (Fwd. strand) 6541 2132 10977 8844 88-1961-mod|88-1961-mod 4 0.0%|0.0%

Deletion DVG (Fwd. strand) 7157 6257 14486 8228 88-1961-mod|88-1961-mod 31 0.0%|0.0%

Deletion DVG (Fwd. strand) 8281 5033 12138 7104 88-1961-mod|88-1961-mod 4 0.0%|0.0%

Deletion DVG (Fwd. strand) 10768 10103 14721 4617 88-1961-mod|88-1961-mod 2 0.0%|0.0%

Deletion DVG (Fwd. strand) 11656 11446 15176 3729 88-1961-mod|88-1961-mod 6 0.0%|0.0%

Deletion DVG (Fwd. strand) 12659 11631 14358 2726 88-1961-mod|88-1961-mod 2 0.0%|0.0%

Deletion DVG (Fwd. strand) 13598 13263 15051 1787 88-1961-mod|88-1961-mod 2 0.0%|0.0%

Deletion DVG (Fwd. strand) 13823 13317 14880 1562 88-1961-mod|88-1961-mod 3 0.0%|0.0%

Deletion DVG (Fwd. strand) 14017 13915 15284 1368 88-1961-mod|88-1961-mod 5 0.0%|0.0%

Deletion DVG (Fwd. strand) 14017 13920 15289 1368 88-1961-mod|88-1961-mod 38 0.0%|0.0%

Deletion DVG (Fwd. strand) 14595 14251 15042 790 88-1961-mod|88-1961-mod 2 0.0%|0.0%

Deletion DVG (Fwd. strand) 14869 14363 14880 516 88-1961-mod|88-1961-mod 3 0.0%|0.0%

Deletion DVG (Fwd. strand) 14869 14369 14886 516 88-1961-mod|88-1961-mod 7 0.0%|0.0%

Deletion DVG (Fwd. strand) 14927 14584 15043 458 88-1961-mod|88-1961-mod 3 0.0%|0.0%

Deletion DVG (Fwd. strand) 14928 14583 15041 457 88-1961-mod|88-1961-mod 3 0.0%|0.0%

Deletion DVG (Fwd. strand) 14928 14584 15042 457 88-1961-mod|88-1961-mod 3 0.0%|0.0%

Deletion DVG (Fwd. strand) 14928 14585 15043 457 88-1961-mod|88-1961-mod 274 0.0%|0.0%

Deletion DVG (Fwd. strand) 14928 14586 15044 457 88-1961-mod|88-1961-mod 8 0.0%|0.0%

Deletion DVG (Fwd. strand) 14928 14587 15045 457 88-1961-mod|88-1961-mod 2 0.0%|0.0%

Deletion DVG (Fwd. strand) 14928 14588 15046 457 88-1961-mod|88-1961-mod 20 0.0%|0.0%

Deletion DVG (Fwd. strand) 14928 14589 15047 457 88-1961-mod|88-1961-mod 1795 0.0%|0.0%

Deletion DVG (Fwd. strand) 14928 14593 15051 457 88-1961-mod|88-1961-mod 2 0.0%|0.0%

Deletion DVG (Fwd. strand) 14958 14462 14890 427 88-1961-mod|88-1961-mod 8 0.0%|0.0%

Deletion DVG (Fwd. strand) 14972 14367 14781 413 88-1961-mod|88-1961-mod 2 0.0%|0.0%

Deletion DVG (Fwd. strand) 15062 14371 14695 323 88-1961-mod|88-1961-mod 2 0.0%|0.0%

Deletion DVG (Fwd. strand) 15177 14958 15167 208 88-1961-mod|88-1961-mod 2 0.0%|0.0%

Deletion DVG (Fwd. strand) 15179 14950 15157 206 88-1961-mod|88-1961-mod 14 0.0%|0.0%

Deletion DVG (Fwd. strand) 15179 14955 15162 206 88-1961-mod|88-1961-mod 10 0.0%|0.0%

Deletion DVG (Fwd. strand) 15179 14957 15164 206 88-1961-mod|88-1961-mod 5 0.0%|0.0%

Deletion DVG (Fwd. strand) 15179 14960 15167 206 88-1961-mod|88-1961-mod 49 0.0%|0.0%

=================================

= Insertion DVG (Fwd. strand)

=================================

DVG's type Length BP_Pos RI_Pos Delta_Positions Ref Counts %_to_Virus

Insertion DVG (Fwd. strand) 15415 1672 1643 30 88-1961-mod|88-1961-mod 3 0.0%|0.0%

Insertion DVG (Fwd. strand) 15425 15098 15059 40 88-1961-mod|88-1961-mod 2 0.0%|0.0%

Insertion DVG (Fwd. strand) 15441 2173 2118 56 88-1961-mod|88-1961-mod 2 0.0%|0.0%

Insertion DVG (Fwd. strand) 15448 14452 14390 63 88-1961-mod|88-1961-mod 2 0.0%|0.0%

Insertion DVG (Fwd. strand) 15456 14506 14436 71 88-1961-mod|88-1961-mod 2 0.0%|0.0%

Insertion DVG (Fwd. strand) 15456 14513 14443 71 88-1961-mod|88-1961-mod 3 0.0%|0.0%

Insertion DVG (Fwd. strand) 15481 14214 14119 96 88-1961-mod|88-1961-mod 2 0.0%|0.0%

Insertion DVG (Fwd. strand) 15484 14220 14122 99 88-1961-mod|88-1961-mod 2 0.0%|0.0%

Insertion DVG (Fwd. strand) 15506 14467 14347 121 88-1961-mod|88-1961-mod 2 0.0%|0.0%

Insertion DVG (Fwd. strand) 15512 15048 14922 127 88-1961-mod|88-1961-mod 4 0.0%|0.0%

Insertion DVG (Fwd. strand) 15519 13798 13665 134 88-1961-mod|88-1961-mod 2 0.0%|0.0%

Insertion DVG (Fwd. strand) 15530 15084 14940 145 88-1961-mod|88-1961-mod 4 0.0%|0.0%

Insertion DVG (Fwd. strand) 15539 15015 14862 154 88-1961-mod|88-1961-mod 107 0.0%|0.0%

Insertion DVG (Fwd. strand) 15539 15020 14867 154 88-1961-mod|88-1961-mod 68 0.0%|0.0%

Insertion DVG (Fwd. strand) 15539 15021 14868 154 88-1961-mod|88-1961-mod 44 0.0%|0.0%

Insertion DVG (Fwd. strand) 15539 15022 14869 154 88-1961-mod|88-1961-mod 72 0.0%|0.0%

Insertion DVG (Fwd. strand) 15539 15023 14870 154 88-1961-mod|88-1961-mod 3 0.0%|0.0%

Insertion DVG (Fwd. strand) 15539 15024 14871 154 88-1961-mod|88-1961-mod 30 0.0%|0.0%

Insertion DVG (Fwd. strand) 15541 13797 13642 156 88-1961-mod|88-1961-mod 3 0.0%|0.0%

Insertion DVG (Fwd. strand) 15545 15040 14881 160 88-1961-mod|88-1961-mod 2 0.0%|0.0%

Insertion DVG (Fwd. strand) 15546 15023 14863 161 88-1961-mod|88-1961-mod 3 0.0%|0.0%

Insertion DVG (Fwd. strand) 15546 15039 14879 161 88-1961-mod|88-1961-mod 9 0.0%|0.0%

Insertion DVG (Fwd. strand) 15551 15027 14862 166 88-1961-mod|88-1961-mod 2 0.0%|0.0%

Insertion DVG (Fwd. strand) 15554 15043 14875 169 88-1961-mod|88-1961-mod 2 0.0%|0.0%

Insertion DVG (Fwd. strand) 15554 15046 14878 169 88-1961-mod|88-1961-mod 36 0.0%|0.0%

Insertion DVG (Fwd. strand) 15557 15146 14975 172 88-1961-mod|88-1961-mod 6 0.0%|0.0%

Insertion DVG (Fwd. strand) 15574 15052 14864 189 88-1961-mod|88-1961-mod 2 0.0%|0.0%

Insertion DVG (Fwd. strand) 15591 15156 14951 206 88-1961-mod|88-1961-mod 2 0.0%|0.0%

Insertion DVG (Fwd. strand) 15591 15161 14956 206 88-1961-mod|88-1961-mod 3 0.0%|0.0%

Insertion DVG (Fwd. strand) 15593 15161 14954 208 88-1961-mod|88-1961-mod 2 0.0%|0.0%

Insertion DVG (Fwd. strand) 15599 13674 13461 214 88-1961-mod|88-1961-mod 2 0.0%|0.0%

Insertion DVG (Fwd. strand) 15599 13675 13462 214 88-1961-mod|88-1961-mod 3 0.0%|0.0%

Insertion DVG (Fwd. strand) 15651 13695 13430 266 88-1961-mod|88-1961-mod 4 0.0%|0.0%

Insertion DVG (Fwd. strand) 15672 13341 13055 287 88-1961-mod|88-1961-mod 3 0.0%|0.0%

Insertion DVG (Fwd. strand) 15682 15042 14746 297 88-1961-mod|88-1961-mod 2 0.0%|0.0%

Insertion DVG (Fwd. strand) 15699 15191 14878 314 88-1961-mod|88-1961-mod 11 0.0%|0.0%

Insertion DVG (Fwd. strand) 15799 14899 14486 414 88-1961-mod|88-1961-mod 12 0.0%|0.0%

Insertion DVG (Fwd. strand) 15809 14878 14455 424 88-1961-mod|88-1961-mod 12 0.0%|0.0%

Insertion DVG (Fwd. strand) 15810 14876 14452 425 88-1961-mod|88-1961-mod 38 0.0%|0.0%

Insertion DVG (Fwd. strand) 15811 14880 14455 426 88-1961-mod|88-1961-mod 78 0.0%|0.0%

Insertion DVG (Fwd. strand) 15812 14877 14451 427 88-1961-mod|88-1961-mod 2 0.0%|0.0%

Insertion DVG (Fwd. strand) 15812 14878 14452 427 88-1961-mod|88-1961-mod 9 0.0%|0.0%

Insertion DVG (Fwd. strand) 15812 14884 14458 427 88-1961-mod|88-1961-mod 3 0.0%|0.0%

Insertion DVG (Fwd. strand) 15812 14890 14464 427 88-1961-mod|88-1961-mod 5 0.0%|0.0%

Insertion DVG (Fwd. strand) 15812 14891 14465 427 88-1961-mod|88-1961-mod 50 0.0%|0.0%

Insertion DVG (Fwd. strand) 15822 14900 14464 437 88-1961-mod|88-1961-mod 3 0.0%|0.0%

Insertion DVG (Fwd. strand) 16022 13350 12714 637 88-1961-mod|88-1961-mod 4 0.0%|0.0%

Insertion DVG (Fwd. strand) 16145 15112 14353 760 88-1961-mod|88-1961-mod 2 0.0%|0.0%

Insertion DVG (Fwd. strand) 16271 15180 14295 886 88-1961-mod|88-1961-mod 4 0.0%|0.0%

Insertion DVG (Fwd. strand) 16313 15180 14253 928 88-1961-mod|88-1961-mod 4 0.0%|0.0%

Insertion DVG (Fwd. strand) 16433 14673 13626 1048 88-1961-mod|88-1961-mod 3 0.0%|0.0%

Insertion DVG (Fwd. strand) 16435 14659 13610 1050 88-1961-mod|88-1961-mod 29 0.0%|0.0%

Insertion DVG (Fwd. strand) 16482 14677 13581 1097 88-1961-mod|88-1961-mod 3 0.0%|0.0%

Insertion DVG (Fwd. strand) 16507 14995 13874 1122 88-1961-mod|88-1961-mod 3 0.0%|0.0%

Insertion DVG (Fwd. strand) 16753 15288 13921 1368 88-1961-mod|88-1961-mod 5 0.0%|0.0%

Insertion DVG (Fwd. strand) 16938 14882 13330 1553 88-1961-mod|88-1961-mod 2 0.0%|0.0%

Insertion DVG (Fwd. strand) 18251 14300 11435 2866 88-1961-mod|88-1961-mod 2 0.0%|0.0%
