# Supplementary material for: Identification and quantification of defective virus genomes in high throughput sequencing data using DVG-profiler, a novel post-sequence alignment processing algorithm
Source: PLoS One. 2019 May 17;14(5):e0216944. doi: 10.1371/journal.pone.0216944 (PMC6524942; doi:10.1371/journal.pone.0216944)
Supplement: S20 Table — (DOCX) [file pone.0216944.s025.docx]

**S20 Table. 5’ copyback DVGs identified in virus #2 using DVG-profiler and DI-tector.**

| DVG specific reads DVG-profiler vs DI-tector ^a^ | Breakpoint / reinitiation position ^c^ | Predicted size of DVG (nt) | Detected by RT-PCR^d^ (primer pair used) |
| --- | --- | --- | --- |
| 111136 / 1374 | 14869 /15030 | 870 | + (a/b2) |
| 23962 / 533 | 14223 / 15165 | 1381 | + (a/b1) |
| 19627 / 452 | 13308 / 14863 | 2598 | + (a/b4) |
| 15867 / 483 | 14947 / 15144 | 678 | + (a/b4) |
| 10349 / 237 | 13616 /14663 | 2490 | + (a/b4) |
| 6866 / 184 | 13908 / 15277 | 1584 | + (a/b1,b2,b3) |
| 5433 / 150 | 14456 / 14885 | 1429 | + (a/b1) |
| 5282 / 153 | 14342 / 15107 | 1320 | + (a/b1) |
| 3577 / 85 | 14730 / 15025 | 1014 | + (a/b1) |
| 3398 / 81 | 14238 / 15025 | 1512 | **-** |
| 3317 / 88 | 13479 / 15064 | 1905 | + (a/b4) |
| 3065 / 35 | 13629 / 13775 | 3366 | **-** |
| 3035 / 13 | 14666 / 14777 | 1334 | **-** |
| 3004 / 77 | 12591 / 14836 | 3343 | **-** |
| 2902 / 54 | 13562 / 14651 | 2557 | **-** |
| 2248 / 64 | 13332 / 14154 | 3286 | **-** |
| 2218 / 49 | 13055 / 13442 | 4273 | **-** |
| 2081 / 59 | 13316 / 15143 | 2310 | + (a/b4) |
| 2060 / 60 | 14869 / 15036 ^e^ | 870 | + (a/b3) |
| 2013 / 44 | 14350 / 14770 | 1650 | **-** |
| 1921 / 29 | 14331 / 14428 | 2013 | **-** |
| 1880 / 46 | 13751 / 14324 | 2695 | **-** |
| 1691 / 54 | 13549 / 14514 | 2712 | **-** |
| 1690 / 50 | 13326 / 13837 | 3608 | **-** |
| 1457 / 41 | 13431 / 13720 | 3620 | **-** |
| 1451 / 54 | 13141 /14880 | 2750 | **-** |
| 1373 / 40 | 14360 / 14873 | 1537 |  |
| 1286 / 42 | 11435 / 14306 | 5029 | **-** |
| 1271 / 15 | 14761 /14917 | 1092 | + (a/b1) |
| 1175 / 45 | 13462 / 13684 | 3624 | **-** |
| 1112 / 36 | 11435 / 15162 | 4173 | **-** |
| 4 DVG ^b^ |  |  |  |
| 661 / 26 | 13347 / 15239 | 2184 | + (a/b4) |
| 9 DVG ^b^ |  |  |  |
| 415 / 8 | 13562 / 14677 | 2526 | + (a/b4) |
| 2 DVG ^b^ |  |  |  |
| 352 / 15 | 14859 / 15177 | 731 | + (a/b3) |
| 122 DVG ^b^ |  |  |  |
| 47 / 0 | 14864 / 15197 | 708 | + (a/b4) |
| 119 DVG ^b^ |  |  |  |
| 19 / 0 | 13340 / 15131 | 2300 | + (a/b4) |
| 470 DVG ^b^ |  |  |  |
| 4 / 0 | 14933 / 15271 | 564 | + (a/b3) |
| 889 DVG ^b^ |  |  |  |
| 0 / 0 | 14596 / 14896 | 1278 | + (a/b1) |

^a^ Listed are all copyback DVGs identified using DVG-profiler with 1000 or more reads. In addition, all copyback DVGs with less than 1000 reads but identified by RT-PCR were listed as well.

^b^ Indicates number of copyback DVGs that were identified by DVG- profiler, but not by RT-PCR, and not individually listed here (See S7 Table for comprehensive list of DVGs).

^c^ In addition to the proposed breakpoint and reinitiation positions for each DVG (called left and right position), DVG – profiler also provides a range (called group) of possible breakpoint and reinitiation positions for each DVG identified. For simplicity, the left and right group start -and end-positions are not given in this table, but in S7 Table.

^d^ PCR fragments were either directly sequenced or subcloned followed by sequencing

^e^ DVG-profiler identified a DVG with the following left and right group start and end positions: 14865-72/15034-15040. It is not 100% identical to the DVG 14856-14863 / 15036-15043 found by RT-PCR, but closely related.
