# Supplementary material for: Identification and quantification of defective virus genomes in high throughput sequencing data using DVG-profiler, a novel post-sequence alignment processing algorithm
Source: PLoS One. 2019 May 17;14(5):e0216944. doi: 10.1371/journal.pone.0216944 (PMC6524942; doi:10.1371/journal.pone.0216944)
Supplement: S21 Table — Highlighted in yellow: all DVGs with no reads identified using DI-tector. (PDF) [file pone.0216944.s026.pdf]

DVG-Profiler reads	DI-Tector reads	Breakpoint	Reinitiation site
923	30	13479	14950
883	21	11822	13779
832	16	12906	15041
734	23	13496	14739
661	26	13343	15239
607	17	12900	14736
582	32	12917	14968
572	12	13548	14559
550	10	13496	14633
528	0	15021	15048
511	2	14106	14207
464	12	14161	14737
463	17	11226	14774
441	11	13859	14987
435	8	13251	15037
415	8	13559	14676
373	0	12100	12329
357	4	13584	14608
352	15	14857	15177
329	9	14442	15066
325	2	14700	14799
317	0	15112	15140
316	5	14482	14677
299	9	12797	14944
290	2	14661	14923
288	13	14723	15066
265	6	14899	14943
258	5	14781	15167
250	11	13262	13625
236	7	12476	14148
236	5	13554	14665
235	9	13963	15008
230	3	14579	15050
217	12	14302	14932
215	6	11856	15221
215	0	14452	15029
211	9	12119	14406
194	2	13748	14551
192	0	4181	14575
186	0	15140	15112
173	2	13880	14370
170	4	12549	14196
166	2	14923	15157
164	3	10843	13370
162	6	13766	14532
151	0	14943	15136
151	4	12289	14981
146	0	14150	14375
142	2	13637	14454
140	4	14206	15176
137	0	12713	13372
132	3	14512	14966
130	0	14865	15043
126	0	13091	13468
121	6	11992	14467
120	5	13776	15032
120	5	9871	13952
117	2	12332	14793
111	0	13040	13609
109	0	15088	15160
107	0	14765	14917
107	2	14297	14445
101	0	13270	14359
101	0	14584	15040
99	0	1621	14102
97	0	12952	14367
95	0	78	96
94	0	14245	14289
93	0	7592	14561
93	2	2366	14409
93	0	12708	14403
93	0	12100	14401
92	2	13102	14941

91	0	2625	14993
89	3	13997	14692
89	0	5078	15083
88	0	12109	14450
87	0	13661	14179
86	0	14576	15153
86	3	12159	14563
85	0	13293	14334
85	0	13343	14273
85	3	14320	14837
85	0	2173	15240
84	3	12906	14036
84	3	14482	15249
83	0	12599	13952
82	2	94	82
82	4	14419	15021
82	0	13669	14421
80	2	12332	14577
80	0	14852	15042
79	0	11706	14207
79	0	14077	14865
79	0	11844	14698
79	0	14114	14464
77	0	8955	14807
76	3	13241	14247
74	0	15010	15038
74	0	14842	15042
74	0	14263	14407
73	0	12070	14560
71	2	13313	14843
69	2	10694	15008
69	0	14238	15149
68	0	12195	14586
68	0	15041	15074
67	0	5074	15079
66	0	14377	15012
65	2	13109	14370
65	0	13748	14327
64	0	11380	14896
64	0	14873	15030
64	0	10694	14873
64	0	14956	15143
63	2	14170	14752
61	2	14432	14457
61	0	14560	15005
60	0	10256	14172
60	0	12639	13604
59	3	14576	15049
59	2	12341	13947
59	2	13872	14379
59	0	12952	15077
58	0	13655	14465
58	0	12517	15185
57	0	12476	14136
57	0	13528	14457
57	0	14603	14967
57	0	11273	15023
57	2	13906	15116
56	0	4167	14589
56	0	12109	14591
55	0	13413	13665
55	0	14419	15094
55	0	13380	14384
55	0	14844	15042
55	0	2915	14817
54	0	13303	14360
54	0	12431	14077
54	0	14560	15057
53	0	14586	14646
53	0	13413	14529
53	0	13766	14800
52	0	13293	14255
52	0	12573	15186
51	0	13602	14674

51	0	4181	13739
51	0	14873	15093
51	0	12573	14136
51	0	10403	13044
50	0	14077	14613
49	0	12318	14581
49	0	14328	14593
48	0	8969	14796
48	0	13941	14134
48	0	13737	14729
48	0	13932	14949
48	0	7497	14360
48	0	14857	15031
47	0	15036	15049
47	0	12583	14151
47	0	14863	15197
47	0	12895	13079
46	0	14866	14892
46	0	14512	14927
45	0	13704	14437
42	0	13277	14035
41	4	14325	14987
41	0	13607	14677
40	2	14017	14758
40	0	14688	14851
40	2	13529	14691
40	0	14619	15043
39	0	10276	13627
39	0	14672	15065
38	0	14872	14890
38	2	13478	14932
38	0	13389	14177
38	0	13972	14385
38	0	12476	14303
38	0	14017	14416
38	0	15200	15185
38	0	5778	15185
37	0	15043	15113
37	0	13717	13808
37	0	12517	13948
37	0	12573	14576
37	0	13328	13834
36	3	698	756
36	0	12235	14659
36	0	13365	14533
36	0	13146	13941
36	0	14452	14836
36	0	5408	15039
36	0	14931	14950
35	0	13183	14498
35	0	14735	15037
35	0	2874	14864
35	0	1407	1450
35	0	10482	14968
34	0	8950	14813
34	0	15036	15068
34	0	12235	14580
34	0	13941	14378
34	0	13914	15103
34	0	14943	15133
33	0	14556	15005
33	0	12900	13743
33	2	14017	14284
33	0	13262	13565
33	0	12431	15092
33	0	12850	14049
32	0	119	150
32	0	14611	15005
31	0	14428	14464
31	0	2941	2962
31	0	10541	14634
31	2	12732	14540
31	0	14017	14332
31	0	13511	14715

30	0	13315	14843
30	0	14231	15157
30	0	12303	14268
30	0	12713	14704
30	0	4431	14868
29	0	15181	15201
29	2	59	88
29	0	15059	15076
29	0	13737	14238
29	0	712	741
29	0	12109	14165
29	0	12119	14436
29	0	12023	14369
29	0	13867	14864
28	0	14377	14687
28	0	14584	15025
28	0	13893	14170
28	0	14424	14464
28	0	14435	14457
27	0	14927	15161
27	0	13146	14624
26	0	13726	13779
26	0	13807	14859
26	0	14759	15025
26	0	11916	15159
26	0	12183	15120
26	0	14723	15024
26	0	12788	13973
26	0	263	14331
26	0	14649	14675
25	0	15106	15148
25	0	11163	15087
25	0	13776	15204
25	0	5065	15092
25	0	14978	15048
25	0	13669	14575
24	0	14650	14675
24	0	14201	15128
24	0	10434	14777
24	0	13475	14537
24	0	13851	14251
24	0	14873	15025
24	0	3835	15111
23	0	13117	14665
23	0	13430	14763
23	0	13343	14531
23	0	14842	14955
23	0	12495	13785
23	0	12441	14508
23	0	12830	13814
23	0	13404	14524
22	0	15210	15236
22	0	4188	14567
22	0	13952	14284
22	0	14857	14930
22	0	2022	2055
22	0	11916	14297
22	0	13241	13526
22	0	13324	14437
22	0	10196	14783
21	0	15098	15145
21	0	13389	14764
21	0	13458	14164
21	0	12350	13701
21	0	13559	13959
21	0	7365	14283
21	0	13997	14016
21	0	14211	15165
21	0	56	94
21	0	5778	15177
20	0	13448	14714
20	0	14501	14551
20	0	15072	15061
20	0	12609	13430

20	0	13475	14314
20	0	8932	14491
20	0	11599	13948
20	0	11092	12640
14 cb DVGs with 19 reads in DI-profiler and 0 reads in DI-Tector			
18	2	13091	13707
16 cb DVGs with 18 reads in DI-profiler and 0 reads in DI-Tector			
17	2	15246	15271
14 cb DVGs with 17 reads in DI-profiler and 0 reads in DI-Tector			
16	2	13700	14149
19 cb DVGs with 16 reads in DI-profiler and 0 reads in DI-Tector			
13 cb DVGs with 15 reads in DI-profiler and 0 reads in DI-Tector			
14	2	13731	15095
27 cb DVGs with 14 reads in DI-profiler and 0 reads in DI-Tector			
33 cb DVGs with 13 reads in DI-profiler and 0 reads in DI-Tector			
12	2	12195	14466
32 cb DVGs with 12 reads in DI-profiler and 0 reads in DI-Tector			
11	2	13150	14631
11	2	14875	14812
36 cb DVGs with 11 reads in DI-profiler and 0 reads in DI-Tector			
10	2	14758	11128
35 cb DVGs with 10 reads in DI-profiler and 0 reads in DI-Tector			
9	2	14419	14524
55 cbDVGs with 9 reads in DI-Profiler and 0 reads in DI-Tector			
8	2	13521	14377
66 cbDVGs with 8 reads in DI-Profiler and 0 reads in DI-Tector			
77 cbDVGs with 7 reads in DI-Profiler and 0 reads in DI-Tector			
119 cbDVGs with 6 reads in DI-Profiler and 0 reads in DI-Tector			
5	2	4185	13734
5	7	14875	15024
130 cbDVGs with 5 reads in DI-Profiler and 0 reads in DI-Tector			
4	52	646	14237
4	2	5785	15189
252 cbDVGs with 4 reads in DI-Profiler and 0 reads in DI-Tector			
3	2	130	142
3	2	14307	13079
334 cbDVGs with 3 reads in DI-Profiler and 0 reads in DI-Tector			
752 cbDVGs with 2 reads in DI-Profiler and 0 reads in DI-Tector			

9	2	14419	14524
55 cbDVGs with 9 reads in DI-Profiler and 0 reads in DI-Tector			
8	2	13521	14377
66 cbDVGs with 8 reads in DI-Profiler and 0 reads in DI-Tector			
77 cbDVGs with 7 reads in DI-Profiler and 0 reads in DI-Tector			
119 cbDVGs with 6 reads in DI-Profiler and 0 reads in DI-Tector			
5	2	4185	13734
5	7	14875	15024
130 cbDVGs with 5 reads in DI-Profiler and 0 reads in DI-Tector			
4	52	646	14237
4	2	5785	15189
252 cbDVGs with 4 reads in DI-Profiler and 0 reads in DI-Tector			
3	2	130	142
3	2	14307	13079
334 cbDVGs with 3 reads in DI-Profiler and 0 reads in DI-Tector			
752 cbDVGs with 2 reads in DI-Profiler and 0 reads in DI-Tector			
