# Supplementary material for: Identification and quantification of defective virus genomes in high throughput sequencing data using DVG-profiler, a novel post-sequence alignment processing algorithm
Source: PLoS One. 2019 May 17;14(5):e0216944. doi: 10.1371/journal.pone.0216944 (PMC6524942; doi:10.1371/journal.pone.0216944)
Supplement: S22 Table — (DOCX) [file pone.0216944.s027.docx]

**S22 Table. Insertion- type DVGs identified in virus #2 using DVG-profiler and DI-tector.**

| Reads found with DVG-profiler ^a^ | Reads found with DI-tector | Breakpoint/Reinitiation position | Size of insertion (nt) |
| --- | --- | --- | --- |
| 14252 | 220 | 14869 / 15023 | 155 |
| 2871 | 55 | 14464 / 14891 | 420 |
| 2537 | 137 | 14456 / 14877 | 434 |
| 2121 | 3 | 14456 / 14882 | 426 |
| 1521 | 29 | 13610 / 14660 | 1050 |
| **1497^b^** | **0^c^** | **14847 / 15025** | **179** |
| 1219 | 38 | 14877 / 15046 | 169 |
| 511 | 2 | 14865 / 15050 | 185 |
| **403** | **0** | **14726 / 15024** | **298** |
| 351 | 12 | 14482 / 14896 | 414 |
| 349 | 2 | 14943 / 15156 | 213 |
| **322** | **0** | **14915 / 15043** | **128** |
| 283 | 9 | 14873 / 15035 | 162 |
| 253 | 107 | 14865 / 15014 | 149 |
| 215 | 4 | 14920 / 15044 | 124 |
| **205** | **0** | **13538 / 14505** | **967** |
| 211 | 11 | 14873 / 15189 | 316 |
| **191** | **0** | **13430 / 13705** | **275** |
| 177 | 5 | 13458 / 13668 | 210 |
| **173** | **0** | **14931 / 15078** | **147** |
| 158 | 2 | 13661 / 13798 | 137 |
| 154 | 5 | 13917 / 15288 | 1371 |
| 153 | 3 | 14462 / 14895 | 433 |
| **149** | **0** | **13559 / 14638** | **1079** |
| **132** | **0** | **13356 / 15252** | **1896** |
| **114** | **0** | **14899 / 15040** | **141** |
| **108** | **0** | **13324 / 13808** | **484** |
| 108 | 4 | 14291 / 15177 | 886 |
| 102 | 2 | 14880 / 15040 | 160 |
| 100 | 2 | 14347 / 15110 | 763 |

^a^ Listed are all insertion- type DVGs identified with 100 or more reads using DVG-profiler.

^b^ DVGs that were detected only by the DVG-profiler are highlighted in bold numbers.

^c^ DVG 14847/15025 is a mosaic DVG exhibiting 17 nt of extra genomic sequence between positions 15025 and 14868 therefore mimicking copyback DVG 14865/15030. See results section for further information.
