# Supplementary material for: Identification and quantification of defective virus genomes in high throughput sequencing data using DVG-profiler, a novel post-sequence alignment processing algorithm
Source: PLoS One. 2019 May 17;14(5):e0216944. doi: 10.1371/journal.pone.0216944 (PMC6524942; doi:10.1371/journal.pone.0216944)
Supplement: S23 Table — (DOCX) [file pone.0216944.s028.docx]

**S23 Table. Deletion- type DVGs identified in virus #2 using DVG-profiler and DI-tector tools.**

| Reads found with DVG-profiler^a^ | Reads found with DI-tector | Breakpoint/Reinitiation position | Size of deletion (nt) |
| --- | --- | --- | --- |
| 62570 | 2110 | 14589 / 15046 | 457 |
| 2015 | 80 | 14960 / 15166 | 207 |
| 2009 | 43 | 13916 / 15284 | 1368 |
| 1079 | 32 | 5785 / 15175 | 9390 |
| 665 | 31^b^ | 6249 / 14475 | 8226 |
| 490 | 19 | 4175 / 13739 | 9564 |
| **304^c^** | **0** | **14949 / 15155** | **206** |
| 296 | 7 | 14365 / 14881 | 516 |
| 244 | 2 | 2422 / 13755 | 11333 |
| 219 | 2 | 14365 / 14693 | 328 |
| 200 | 6 | 812 / 13431 | 12619 |
| 181 | 8 | 14457 / 14885 | 428 |
| 169 | 6 | 11443 / 15171 | 3728 |
| 169 | 4 | 3196 / 12185 | 8989 |
| 168 | 4 | 2127 / 10975 | 8848 |
| 140 | 4 | 5029 / 12136 | 7107 |
| **111** | **0** | **14890/ 15029** | **139** |
| 110 | 3 | 13315 / 14875 | 1560 |
| 106 | 2 | 1896 / 14927 | 13031 |
| 106 | 2 | 2294 / 14177 | 11883 |

^a^ Listed are all deletion- type DVGs identified with 100 or more reads using DVG-profiler.

^b^ The DVG identified by DI-tector is not identical but very closely related to the one identified by DVG-Profiler (6251/14478 vs 6257/14486).

^c^ DVGs that were detected only by the DVG-Profiler are highlighted in bold numbers.
