# Supplementary material for: Identification and quantification of defective virus genomes in high throughput sequencing data using DVG-profiler, a novel post-sequence alignment processing algorithm
Source: PLoS One. 2019 May 17;14(5):e0216944. doi: 10.1371/journal.pone.0216944 (PMC6524942; doi:10.1371/journal.pone.0216944)
Supplement: S1 File — (DOCX) [file pone.0216944.s029.docx]

**Supplemental material and methods**

**DNA fragment used for generation of DVG RNA**

The sequence of the DNA fragment coding for DVG Urabe930 and that was used for *in vitro* transcription is depicted below. The virus specific sequence is shown in italics, the T7 RNA polymerase promoter is underlined, flanking *BamH*I and *Xho*I restriction sites are shown in bold letters.

5’ CAGC**GGATCC***ACCAAGGGGAGAAAGTAAAATCAATTTTTTCTTAAAATGATATTATGGATTTAATACTGATGTGGTTATGATTGTTCGAGGTCGTGTACGGTCTAGAAGAATTTGGATGCTGGTGTGGAACCTTTTGGTGACGTATCATGCCAGAGGAGTCTTTAGTCGATTTAAATTATGTCCCCGTGGATATCATAATCAGGTATATCATTGTCAATATCAATGCGCTCAGAATCTTCAACATCAGGGGTAAGCAAACCAAAGCAATAGATTACACACCCAATGGCTTTCCAAATTCTTTTTTGATAGGATCGATTTAACAACATGTCAGATGTTTCTCCCATTAGATGATTGAATGTTGATTGGTTTAATGCAGACTTCAGATACTTCCGGTTGGGGGATAGCTTTAGGATCTCCATGGGTGTTATCACGTCTCTTAGTCTAAACTCTCCAAGCTCGATAGACTGTCGTAAGGTGAGACGAGTTTGAGTGGGCAAGATGCACCAGTTCCTTACAGTGTACATAAGAATTCTCTCAACAATCAATCTGAGTATTGTATTTATTTTCCCGAGGGGACCTACATTGTATGAAGTAAATAATAGGGTGTCATGATCTGATGATTGGCCCTTTAGGATTTCTATTACCTCTTTAAGATGAATTGTTATGAGTCTGGCCATCTCCGATTGTAATTCATTGAACGACGGATATTGAAGATTCTGAGCGCATTGATATTGACAATGATATACCTGATTATGATATCCACGGGGACATAATTTAAATCGACTAAAGACTCCTCTGGCATGATACGTCACCAAAAGGTTCCACACCAGCATCCAAATTCTTCTAGACCGTACACGACCTCGAACAATCATAACCACATCAGTATTAAATCCATAATATCATTTTAAGAAAAAATTGATTTTACTTTCTCCCCTTGGT*CCCTATAGTGAGTCGTATTA**CTCGAG**ACTG 3’

***In vitro* transcription**

DNA fragment DVG Urabe930 (200 ng) and linearized plasmids Topo1-10 and Topo2-4 (1 μg each) were used for *in vitro* transcription employing a MEGAscript T7 Kit (Ambion, Inc) as recommended by the manufacturer. Removal of template DNA was achieved using Turbo DNase. RNA was purified by phenol/chloroform extraction and isopropanol precipitation. RNA was resolved in DEPC water supplemented with 0.5 mM EDTA. The correct size of *in* *vitro* transcribed RNA was verified by electrophoresis in a 1% agarose-formaldehyde gel containing ethidium bromide for visualization of RNA.

**Preparation of standard RNA for qRT-PCR:**

Plasmids Topo1-10 and Topo2-4 were linearized using restriction enzyme *Spe*I and gel purified. 1 μg each of linearized DNA was used in *in vitro* transcription reactions as described above. The number of RNA molecules per μl was calculated based on UV spectrophotometric quantitation. 10-fold dilutions (from 10^9^ to 10^0^ molecules/μl) of the RNA standards were carried out in dilution buffer (1μM DTT, 1 unit/μl of RnaseOUT Ribonuclease Inhibitor (Invitrogen) and tRNA (10 ng/μl)). RNA standards derived from Topo1-10 and Topo2-4 were named 43/1112 and 14750/15384, respectively.

**Quantitative RT-PCR (qRT-PCR)**

To determine the amount of full-length genome copies present in virus r88+JL(M/F/SH/HN), qRT-PCR was employed using RNA standard 43/1112. To determine the amount of *in vitro* transcribed DVG Urabe930, qRT-PCR was employed using RNA standard 14750/15384. Quantifications were based on a series of dilutions of the RNA standards, ranging from 10^0^ to 10^9^ RNA copies per reaction, analysed in duplicate. qRT-PCR was done using QuantiFast SYBR Green RT-PCR Kit (Qiagen) in a final volume of 25 μl per sample in the presence of primer pairs 612F/667R and 15315F/A (for RNA standards 43/1112 and 14750/15384, respectively, 25 pmoles each per reaction). 1 μl of viral RNA, DVG RNA (at a 1:10^8^ dilution) or RNA standard was analysed per reaction. qRT-PCR was carried out in a 96 well format on a QuantStudio6 Flex system (Applied Biosystems (Life Technologies), Foster City, CA) under the following three-stage conditions: 50 °C for 20 min followed by 95 °C for 5 min, followed by 40 cycles of 95 °C for 10 s, and 60 °C for 30 s. Obtained Ct values were used to calculate the standard curves and the number of genomes / DVG RNA molecules in tested samples.

**Infection of cells for studying the kinetics of IFN gene expression**

A549 cells were seeded into five 6-well dishes and grown to 95% confluence. Three wells per dish each were infected with rescued viruses #1, #2 and #3 at a multiplicity of infection (MOI) of 0.43 in a volume of 250 μl. The remaining dishes served as negative control. After 1 hour of incubation at 37 °C, supernatants were removed, cells washed twice with PBS, followed by addition of 1 ml of fresh medium. Cell culture supernatants were harvested after 15 minutes, 8h, 16h, 24h and 48h post infection and were kept frozen until analyzed by ELISA. 6-well dishes with cells were frozen at -70 °C until RNA was extracted.

**Total RNA extraction**

To extract RNA from A549 cells infected with viruses #1, #2 and #3, the RNeasy Plus Mini kit (Qiagen) was employed. Briefly, cells were directly lysed in 350 μl of RLT buffer supplemented with β-mercaptoethanol. Lysates were homogenized through QIAshredders (Qiagen) followed by transfer to genomic DNA eliminator spin columns and further processed according to the recommended protocol.

**Enzyme-linked immunosorbent assay (ELISA)**

The secreted levels of IFN-λ1 and IFN- λ2/3 from A549 cell culture supernatants were analyzed using ELISA kits supplied by BioLegend (San Diego, CA). ELISA to detect human IFN-β was supplied by PBL Biomedical Laboratories (Piscataway Township, NJ). ELISAs were performed as described previously [44]. The IFN levels from cell cultures infected with different viruses were analyzed in one experiment.

**Quantitative polymerase-chain reaction (qPCR) of IFN genes**

Quantification of changes in gene expression was carried out by qPCR analyses of individual IFN genes (i.e.,*IFN*- β*, IFN-*λ1, and *IFN*- λ2/3) as described previously [45]. Total cellular RNA isolated from virus-infected A549 cell cultures (1 μg) was reverse-transcribed to cDNA with Quantiscript reverse transcriptase (Qiagen). The cDNA was mixed with RT^2^ SYBR^®^ green qPCR Mastermix (Qiagen) and qPCR was performed using the ViiA^TM^7 system (Applied Biosystems). Changes in gene expression levels were analyzed using ViiA^TM^ 7 software v.1.2.2 (Applied Biosystems), and the results were expressed as the mean-fold increase relative to the untreated control gene expression levels after normalization to the housekeeping gene, *GAPDH*. Graphing and statistical analysis of qPCR results were performed using Prism 6.0 (GraphPad Software Inc.). Values represent the mean ± standard deviation (SD) of at least triplicate determinations.
